# Supplementary material for: Effects of Resveratrol-Loaded Cyclodextrin on the Quality Characteristics of Ram Spermatozoa Following Cryopreservation
Source: Animals (Basel). 2024 Sep 23;14(18):2745. doi: 10.3390/ani14182745 (PMC11428706; doi:10.3390/ani14182745)
Supplement: Supplementary file 1 [file animals-14-02745-s001.zip › animals-3177361-supplementary.pdf]

Sperm motility parameters of post-equilibration ram sperm- ANOVA

EqSonMotCasa: Total motility- post-equilibration
EqSonMotProg: Progresif motility- post-equilibration

Groups
Kontrol: Control
Res10: RES10
Res20: RES20
Res40: RES40
Sık10: CD10
Sık20: CD10
Sık40: CD40
Rlc10: RLC10
Rlc20: RLC20
Rlc40: RLC40

```
EXAMINE VARIABLES=EqSonMotCasa EqSonMotProg EqSonMotSub BY Grup
  /PLOT BOXPLOT STEMLEAF HISTOGRAM NPPLOT
  /COMPARE GROUPS
  /STATISTICS DESCRIPTIVES
  /CINTERVAL 95
  /MISSING LISTWISE
  /NOTOTAL.
```

Explore

Notes

Output Created		22-DEC-2020 10:54:14
Comments		
Input	Active Dataset	DataSet0
	Filter	<none>
	Weight	<none>
	Split File	<none>
	N of Rows in Working Data File	100
Missing Value Handling	Definition of Missing	User-defined missing values for dependent variables are treated as missing.

	Cases Used	Statistics are based on cases with no missing values for any dependent variable or factor used.
Syntax		EXAMINE VARIABLES=EqSonMotCasa EqSonMotProg BY Grup<br>/PLOT BOXPLOT STEMLEAF HISTOGRAM NPPLOT<br>/COMPARE GROUPS<br>/STATISTICS DESCRIPTIVES<br>/CINTERVAL 95<br>/MISSING LISTWISE<br>/NOTOTAL.
Resources	Processor Time	00:00:11,25
	Elapsed Time	00:00:11,15

Grup

Case Processing Summary

		Cases					
		Valid		Missing		Total	
	Grup	N	Percent	N	Percent	N	Percent
EqSonMotCasa	kontrol	10	100,0%	0	0,0%	10	100,0%
	Res10	10	100,0%	0	0,0%	10	100,0%
	Res20	10	100,0%	0	0,0%	10	100,0%
	Res40	10	100,0%	0	0,0%	10	100,0%
	sık10	10	100,0%	0	0,0%	10	100,0%
	sık20	10	100,0%	0	0,0%	10	100,0%
	sık40	10	100,0%	0	0,0%	10	100,0%
	Rlc10	10	100,0%	0	0,0%	10	100,0%
	Rlc20	10	100,0%	0	0,0%	10	100,0%
	Rlc40	10	100,0%	0	0,0%	10	100,0%
EqSonMotProg	kontrol	10	100,0%	0	0,0%	10	100,0%
	Res10	10	100,0%	0	0,0%	10	100,0%
	Res20	10	100,0%	0	0,0%	10	100,0%
	Res40	10	100,0%	0	0,0%	10	100,0%

sık10	10	100,0%	0	0,0%	10	100,0%
sık20	10	100,0%	0	0,0%	10	100,0%
sık40	10	100,0%	0	0,0%	10	100,0%
Rlc10	10	100,0%	0	0,0%	10	100,0%
Rlc20	10	100,0%	0	0,0%	10	100,0%
Rlc40	10	100,0%	0	0,0%	10	100,0%

Descriptives

	Grup			Statistic	Std. Error
EqSonMotCasa	kontrol	Mean		91,9000	1,27758
		95% Confidence Interval for Mean	Lower Bound	89,0099	
			Upper Bound	94,7901	
		5% Trimmed Mean		92,0556	
		Median		91,5000	
		Variance		16,322	
		Std. Deviation		4,04008	
		Minimum		84,00	
		Maximum		97,00	
		Range		13,00	
		Interquartile Range		6,50	
		Skewness		-,592	,687
		Kurtosis		,086	1,334
	Res10	Mean		96,2000	,64636
		95% Confidence Interval for Mean	Lower Bound	94,7378	
			Upper Bound	97,6622	
		5% Trimmed Mean		96,2778	
		Median		97,0000	
		Variance		4,178	
		Std. Deviation		2,04396	
		Minimum		93,00	
		Maximum		98,00	
		Range		5,00	
		Interquartile Range		4,25	
		Skewness		-,921	,687
		Kurtosis		-1,010	1,334
	Res20	Mean		96,0000	,51640
		95% Confidence Interval for	Lower Bound	94,8318	

	Mean	Upper Bound	97,1682	
	5% Trimmed Mean		96,0556	
	Median		96,0000	
	Variance		2,667	
	Std. Deviation		1,63299	
	Minimum		93,00	
	Maximum		98,00	
	Range		5,00	
	Interquartile Range		3,00	
	Skewness		-,191	,687
	Kurtosis		-,288	1,334
Res40	Mean		94,1000	1,40198
	95% Confidence Interval for Mean	Lower Bound	90,9285	
		Upper Bound	97,2715	
	5% Trimmed Mean		94,3889	
	Median		95,5000	
	Variance		19,656	
	Std. Deviation		4,43346	
	Minimum		85,00	
	Maximum		98,00	
	Range		13,00	
	Interquartile Range		7,00	
	Skewness		-,953	,687
	Kurtosis		,222	1,334
sık10	Mean		93,3000	,96667
	95% Confidence Interval for Mean	Lower Bound	91,1132	
		Upper Bound	95,4868	
	5% Trimmed Mean		93,2778	
	Median		93,0000	
	Variance		9,344	
	Std. Deviation		3,05687	
	Minimum		89,00	
	Maximum		98,00	
	Range		9,00	
	Interquartile Range		5,00	
	Skewness		,404	,687
	Kurtosis		-,650	1,334
sık20	Mean		93,3000	1,35851

	95% Confidence Interval for Mean	Lower Bound	90,2268	
		Upper Bound	96,3732	
	5% Trimmed Mean		93,7222	
	Median		94,0000	
	Variance		18,456	
	Std. Deviation		4,29599	
	Minimum		82,00	
	Maximum		97,00	
	Range		15,00	
	Interquartile Range		3,50	
	Skewness		-2,317	,687
	Kurtosis		6,385	1,334
sık40	Mean		93,4000	,74833
	95% Confidence Interval for Mean	Lower Bound	91,7072	
		Upper Bound	95,0928	
	5% Trimmed Mean		93,5000	
	Median		94,0000	
	Variance		5,600	
	Std. Deviation		2,36643	
	Minimum		89,00	
	Maximum		96,00	
	Range		7,00	
	Interquartile Range		4,25	
	Skewness		-,724	,687
	Kurtosis		-,540	1,334
Rlc10	Mean		95,3000	,47258
	95% Confidence Interval for Mean	Lower Bound	94,2309	
		Upper Bound	96,3691	
	5% Trimmed Mean		95,3333	
	Median		95,5000	
	Variance		2,233	
	Std. Deviation		1,49443	
	Minimum		93,00	
	Maximum		97,00	
	Range		4,00	
	Interquartile Range		3,00	
	Skewness		-,140	,687
	Kurtosis		-1,622	1,334

	Rlc20	Mean		95,4000	,68638
		95% Confidence Interval for Mean	Lower Bound	93,8473	
			Upper Bound	96,9527	
		5% Trimmed Mean		95,5000	
		Median		96,0000	
		Variance		4,711	
		Std. Deviation		2,17051	
		Minimum		91,00	
		Maximum		98,00	
		Range		7,00	
		Interquartile Range		3,25	
		Skewness		-,971	,687
		Kurtosis		,356	1,334
	Rlc40	Mean		95,2000	,69602
		95% Confidence Interval for Mean	Lower Bound	93,6255	
			Upper Bound	96,7745	
		5% Trimmed Mean		95,3333	
		Median		96,0000	
		Variance		4,844	
		Std. Deviation		2,20101	
		Minimum		90,00	
		Maximum		98,00	
		Range		8,00	
		Interquartile Range		2,25	
		Skewness		-1,488	,687
		Kurtosis		3,183	1,334
EqSonMotProg	kontrol	Mean		51,2000	2,99926
		95% Confidence Interval for Mean	Lower Bound	44,4152	
			Upper Bound	57,9848	
		5% Trimmed Mean		51,0000	
		Median		53,5000	
		Variance		89,956	
		Std. Deviation		9,48449	
		Minimum		35,00	
		Maximum		71,00	
		Range		36,00	
		Interquartile Range		10,25	
		Skewness		,465	,687
		Kurtosis		1,822	1,334

Res10	Mean		54,2000	3,00296
	95% Confidence Interval for Mean	Lower Bound	47,4068	
		Upper Bound	60,9932	
	5% Trimmed Mean		54,2222	
	Median		55,0000	
	Variance		90,178	
	Std. Deviation		9,49620	
	Minimum		40,00	
	Maximum		68,00	
	Range		28,00	
	Interquartile Range		15,75	
	Skewness		-,188	,687
	Kurtosis		-,975	1,334
Res20	Mean		52,0000	3,23866
	95% Confidence Interval for Mean	Lower Bound	44,6737	
		Upper Bound	59,3263	
	5% Trimmed Mean		52,1111	
	Median		53,5000	
	Variance		104,889	
	Std. Deviation		10,24153	
	Minimum		36,00	
	Maximum		66,00	
	Range		30,00	
	Interquartile Range		15,75	
	Skewness		-,423	,687
	Kurtosis		-,991	1,334
Res40	Mean		51,9000	2,96067
	95% Confidence Interval for Mean	Lower Bound	45,2025	
		Upper Bound	58,5975	
	5% Trimmed Mean		51,7778	
	Median		55,5000	
	Variance		87,656	
	Std. Deviation		9,36245	
	Minimum		39,00	
	Maximum		67,00	
	Range		28,00	
	Interquartile Range		15,25	
	Skewness		-,139	,687
	Kurtosis		-1,027	1,334
sık10	Mean		50,6000	2,54384
	95% Confidence Interval for	Lower Bound	44,8454	

	Mean	Upper Bound	56,3546	
	5% Trimmed Mean		50,4444	
	Median		50,0000	
	Variance		64,711	
	Std. Deviation		8,04432	
	Minimum		39,00	
	Maximum		65,00	
	Range		26,00	
	Interquartile Range		11,50	
	Skewness		,354	,687
	Kurtosis		-,252	1,334
sık20	Mean		51,8000	3,15454
	95% Confidence Interval for Mean	Lower Bound	44,6639	
		Upper Bound	58,9361	
	5% Trimmed Mean		52,0000	
	Median		53,5000	
	Variance		99,511	
	Std. Deviation		9,97553	
	Minimum		34,00	
	Maximum		66,00	
	Range		32,00	
	Interquartile Range		17,25	
	Skewness		-,448	,687
	Kurtosis		-,493	1,334
sık40	Mean		53,5000	2,67187
	95% Confidence Interval for Mean	Lower Bound	47,4558	
		Upper Bound	59,5442	
	5% Trimmed Mean		53,6111	
	Median		51,0000	
	Variance		71,389	
	Std. Deviation		8,44919	
	Minimum		39,00	
	Maximum		66,00	
	Range		27,00	
	Interquartile Range		14,50	
	Skewness		,041	,687
	Kurtosis		-,594	1,334
Rlc10	Mean		50,9000	3,50381
	95% Confidence Interval for Mean	Lower Bound	42,9738	
		Upper Bound	58,8262	
	5% Trimmed Mean		51,0556	

		Median		50,5000	
		Variance		122,767	
		Std. Deviation		11,08001	
		Minimum		31,00	
		Maximum		68,00	
		Range		37,00	
		Interquartile Range		17,25	
		Skewness		-,093	,687
		Kurtosis		-,196	1,334
	Rlc20	Mean		53,5000	3,00463
		95% Confidence Interval for Mean	Lower Bound	46,7031	
			Upper Bound	60,2969	
		5% Trimmed Mean		54,1667	
		Median		55,0000	
		Variance		90,278	
		Std. Deviation		9,50146	
		Minimum		31,00	
		Maximum		64,00	
		Range		33,00	
		Interquartile Range		11,50	
		Skewness		-1,580	,687
		Kurtosis		3,053	1,334
	Rlc40	Mean		52,1000	2,97937
		95% Confidence Interval for Mean	Lower Bound	45,3602	
			Upper Bound	58,8398	
		5% Trimmed Mean		51,9444	
		Median		50,0000	
		Variance		88,767	
		Std. Deviation		9,42161	
		Minimum		40,00	
		Maximum		67,00	
		Range		27,00	
		Interquartile Range		15,75	
		Skewness		,171	,687
		Kurtosis		-1,332	1,334

Tests of Normality

		Kolmogorov-Smirnov[a]			Shapiro-Wilk		
	Grup	Statistic	df	Sig.	Statistic	df	Sig.

EqSonMotCasa	kontrol	,145	10	,200*	,945	10	,614
	Res10	,352	10	,001	,767	10	,006
	Res20	,200	10	,200*	,890	10	,169
	Res40	,210	10	,200*	,836	10	,040
	sık10	,139	10	,200*	,941	10	,569
	sık20	,281	10	,024	,714	10	,001
	sık40	,200	10	,200*	,908	10	,266
	Rlc10	,208	10	,200*	,882	10	,138
	Rlc20	,209	10	,200*	,913	10	,299
	Rlc40	,242	10	,100	,861	10	,079
EqSonMotProg	kontrol	,244	10	,093	,913	10	,305
	Res10	,118	10	,200*	,961	10	,800
	Res20	,221	10	,182	,921	10	,363
	Res40	,207	10	,200*	,910	10	,278
	sık10	,130	10	,200*	,975	10	,935
	sık20	,148	10	,200*	,970	10	,890
	sık40	,170	10	,200*	,940	10	,550
	Rlc10	,138	10	,200*	,971	10	,897
	Rlc20	,221	10	,182	,857	10	,070
	Rlc40	,199	10	,200*	,923	10	,383

*. This is a lower bound of the true significance.

a. Lilliefors Significance Correction

EqSonMotCasa

Histograms

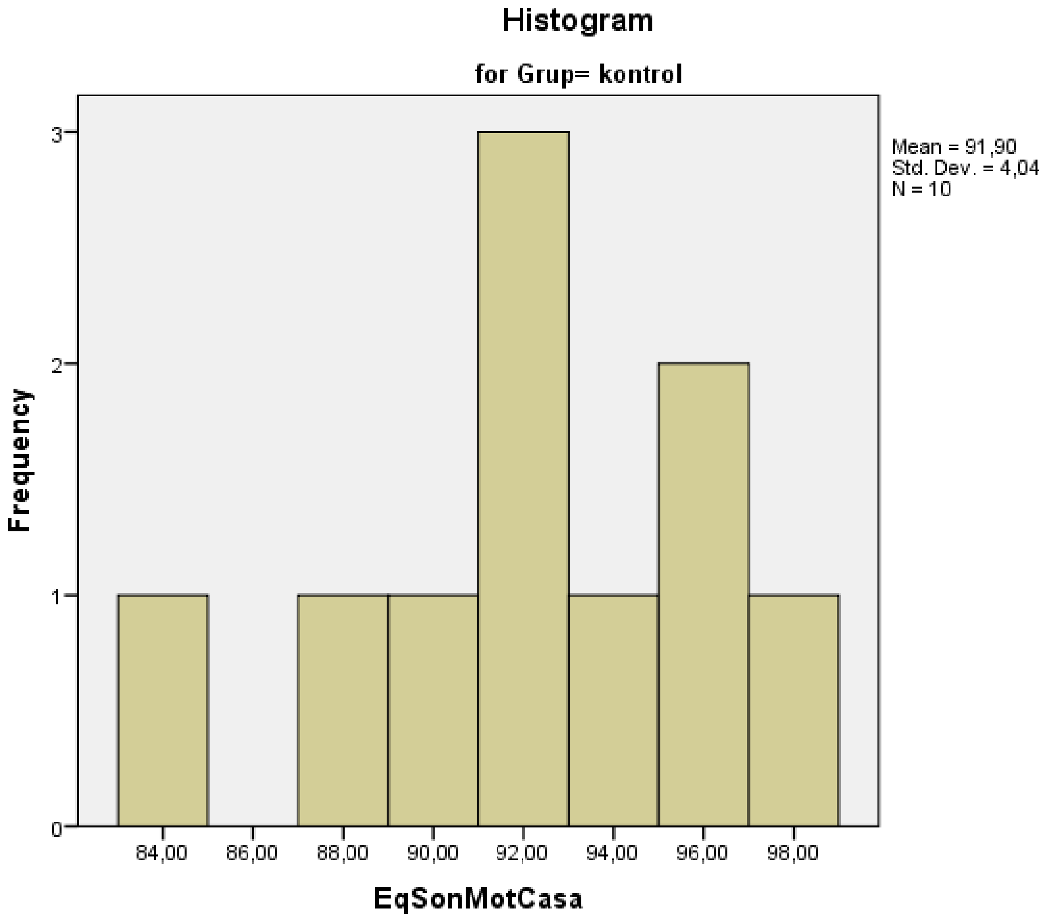

Histogram
for Grup= kontrol
Mean = 91,90
Std. Dev. = 4,04
N = 10
Frequency
0
1
2
3
84,00
86,00
88,00
90,00
92,00
94,00
96,00
98,00
EqSonMotCasa

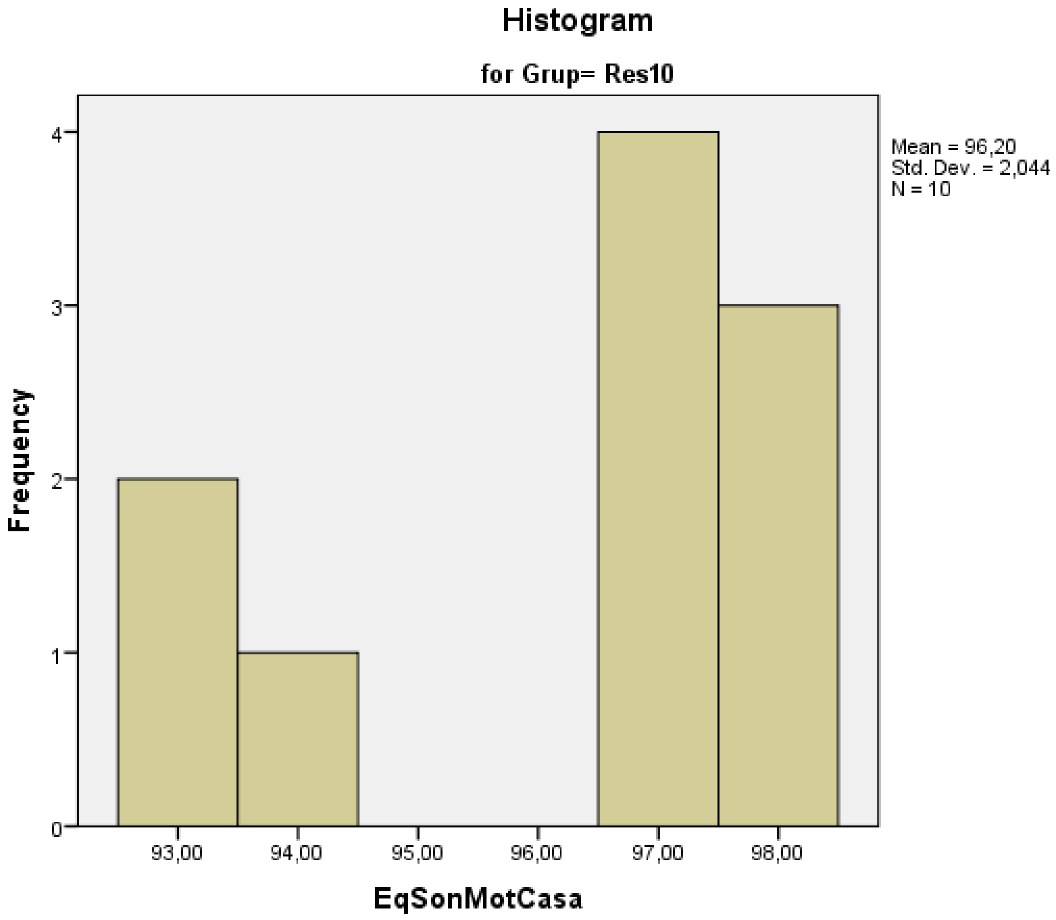
Histogram
for Grup= Res10
Mean = 96,20
Std. Dev. = 2,044
N = 10
Frequency
0
1
2
3
4
93,00
94,00
95,00
96,00
97,00
98,00
EqSonMotCasa

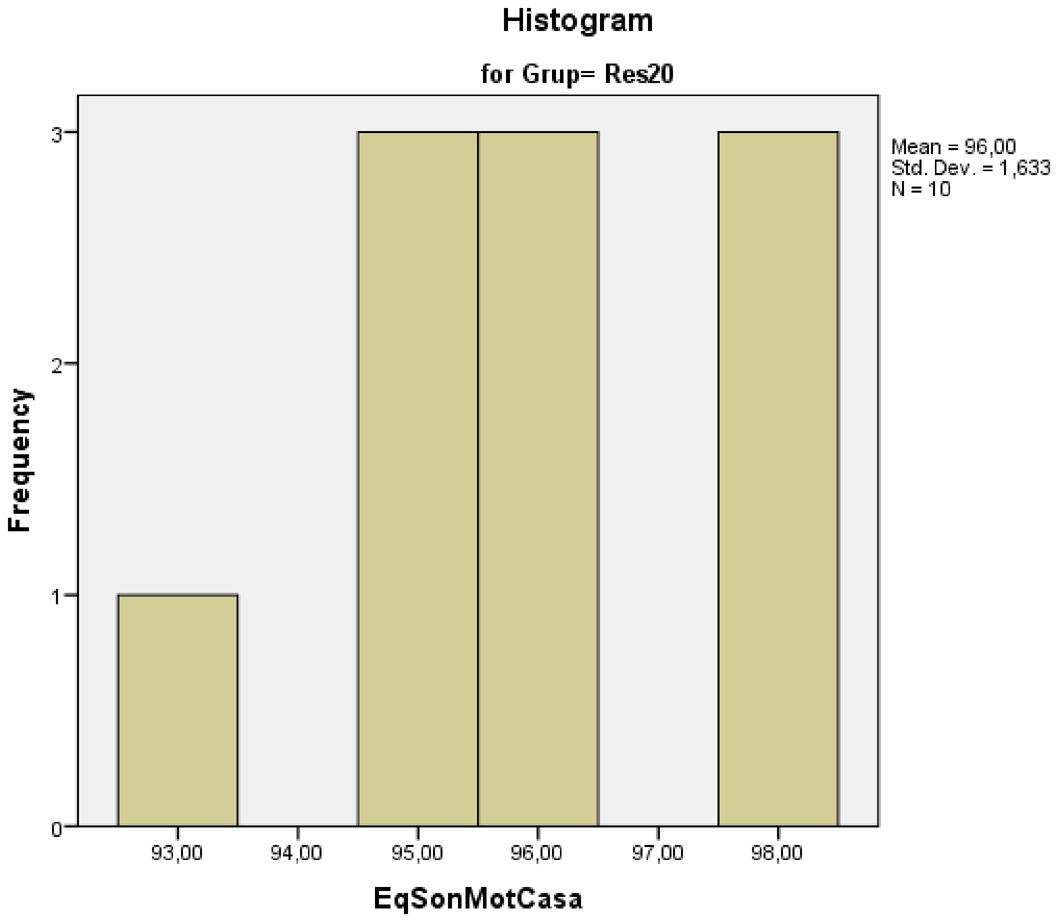
Histogram
for Grup= Res20
Mean = 96,00
Std. Dev. = 1,633
N = 10
Frequency
0
1
2
3
93,00
94,00
95,00
96,00
97,00
98,00
EqSonMotCasa

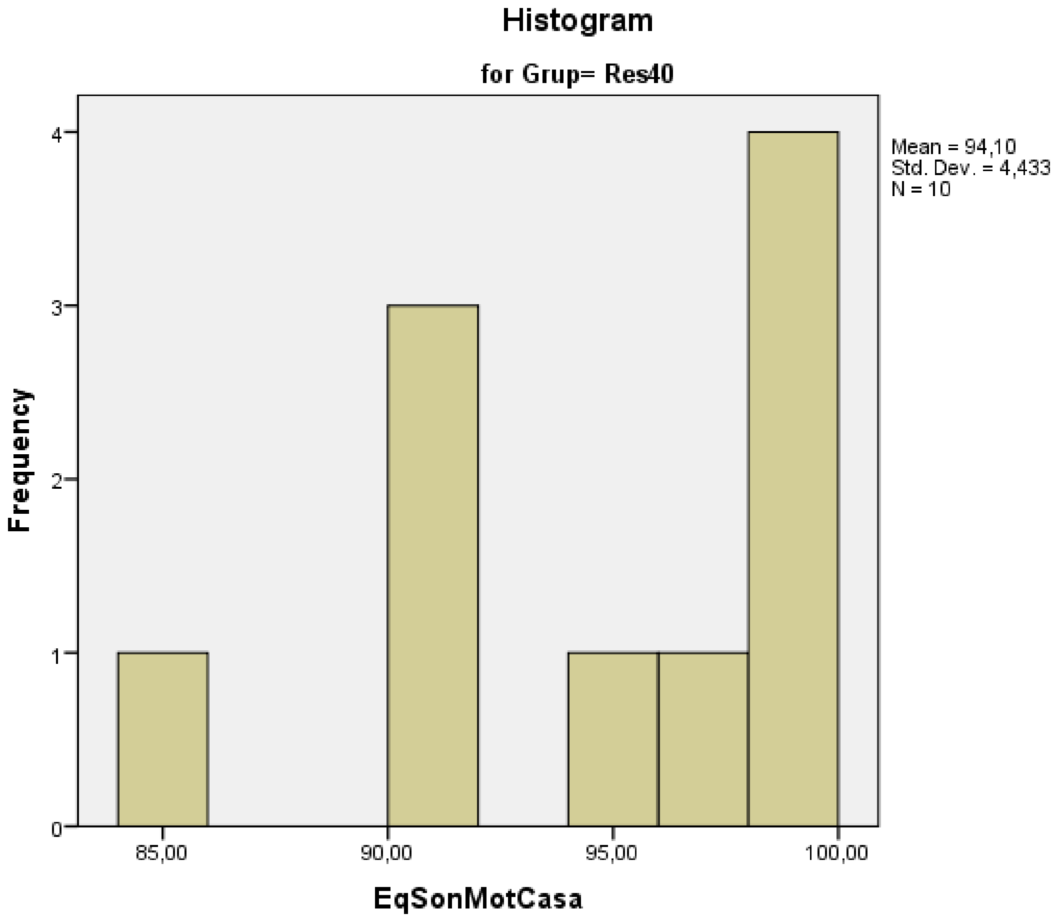

Histogram
for Grup= Res40
Mean = 94,10
Std. Dev. = 4,433
N = 10
Frequency
0
1
2
3
4
85,00
90,00
95,00
100,00
EqSonMotCasa

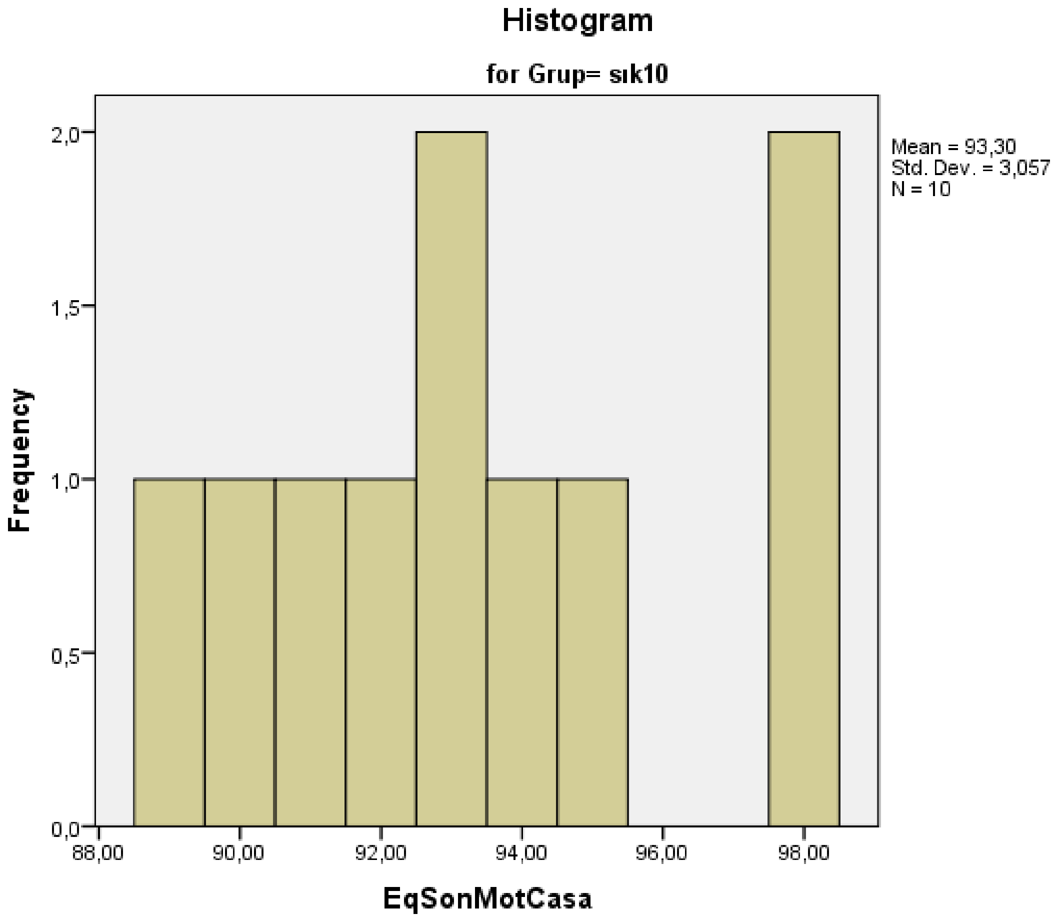
Histogram
for Grup= sık10
Mean = 93,30
Std. Dev. = 3,057
N = 10
Frequency
2,0
1,5
1,0
0,5
0,0
88,00
90,00
92,00
94,00
96,00
98,00
EqSonMotCasa

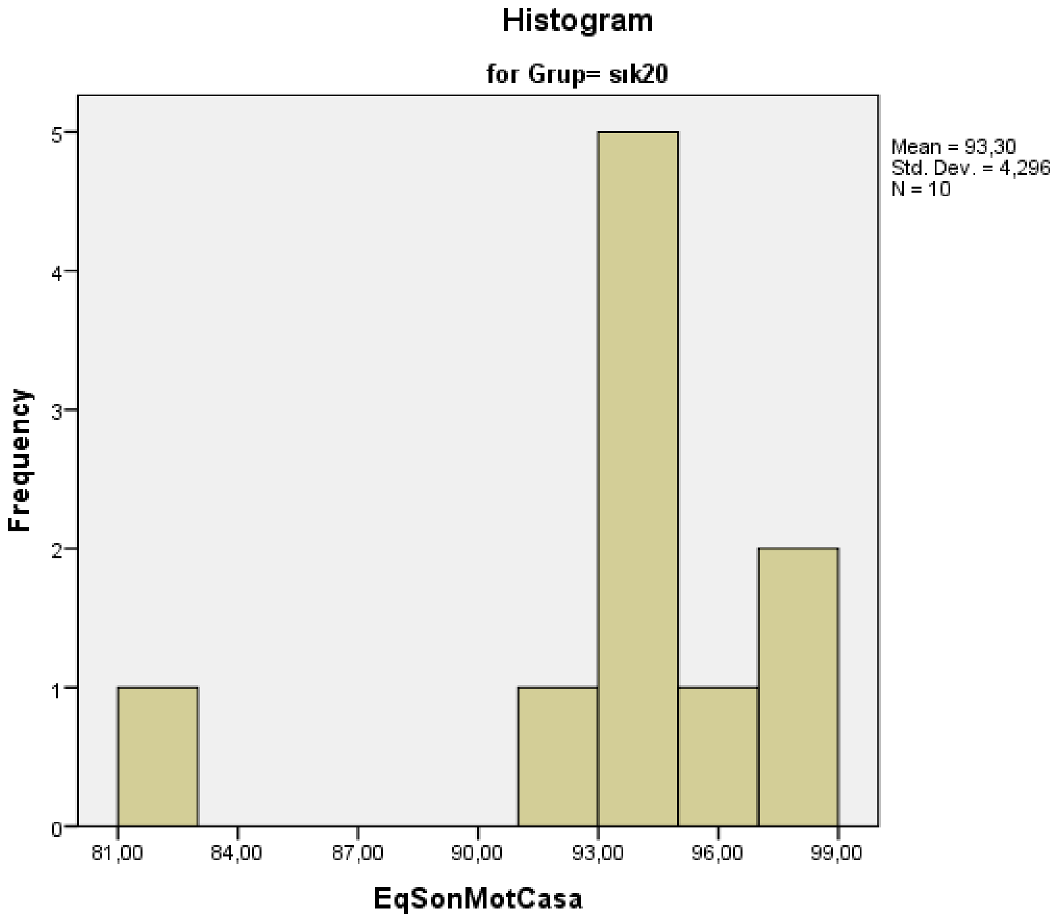
Histogram
for Grup= sık20
Mean = 93,30
Std. Dev. = 4,296
N = 10
Frequency
0
1
2
3
4
5
81,00
84,00
87,00
90,00
93,00
96,00
99,00
EqSonMotCasa

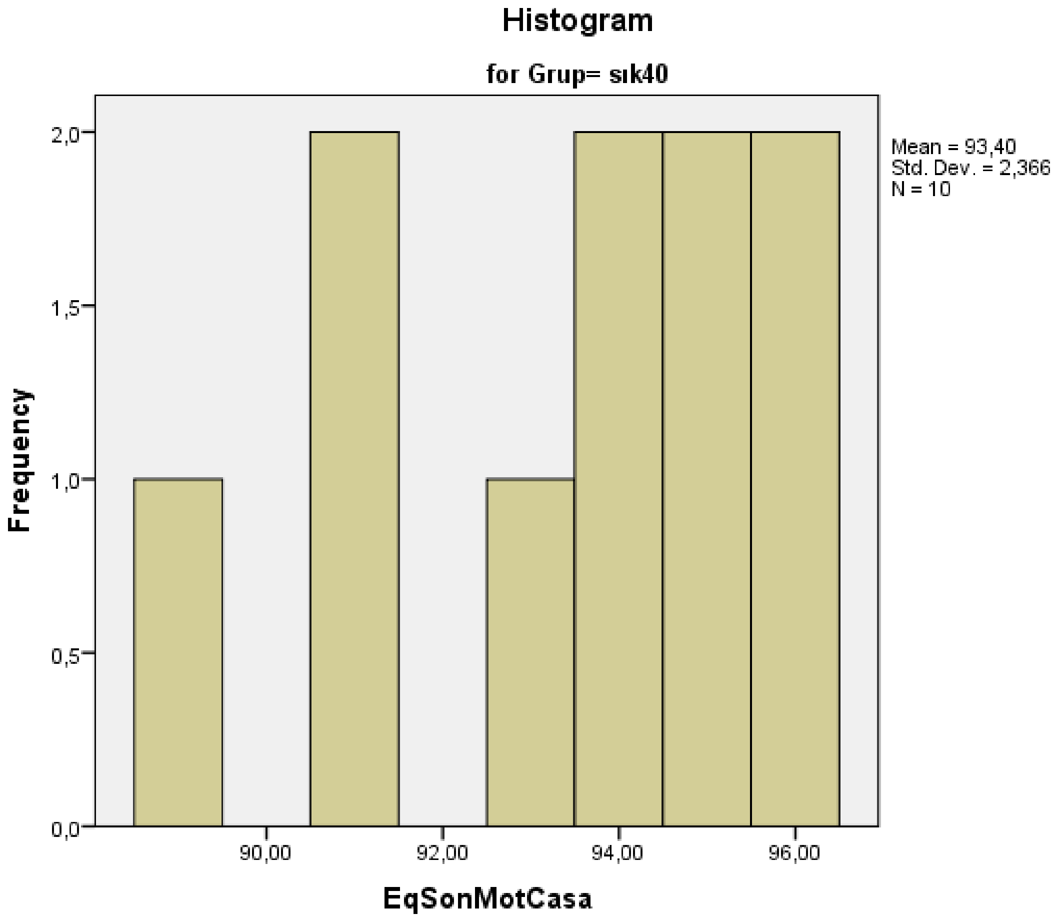
Histogram
for Grup= sık40
Frequency
2,0
1,5
1,0
0,5
0,0
90,00
92,00
94,00
96,00
EqSonMotCasa
Mean = 93,40
Std. Dev. = 2,366
N = 10

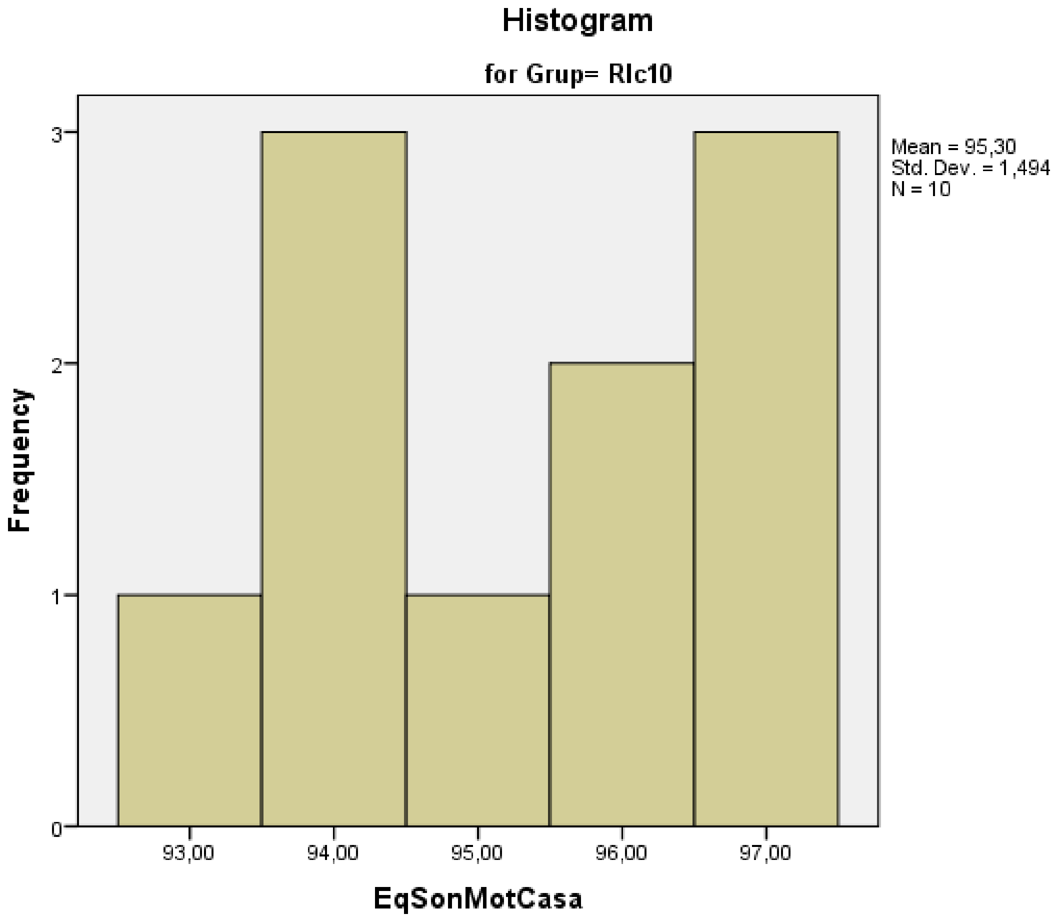

Histogram
for Grup= Rlc10
Mean = 95,30
Std. Dev. = 1,494
N = 10
Frequency
0
1
2
3
93,00
94,00
95,00
96,00
97,00
EqSonMotCasa

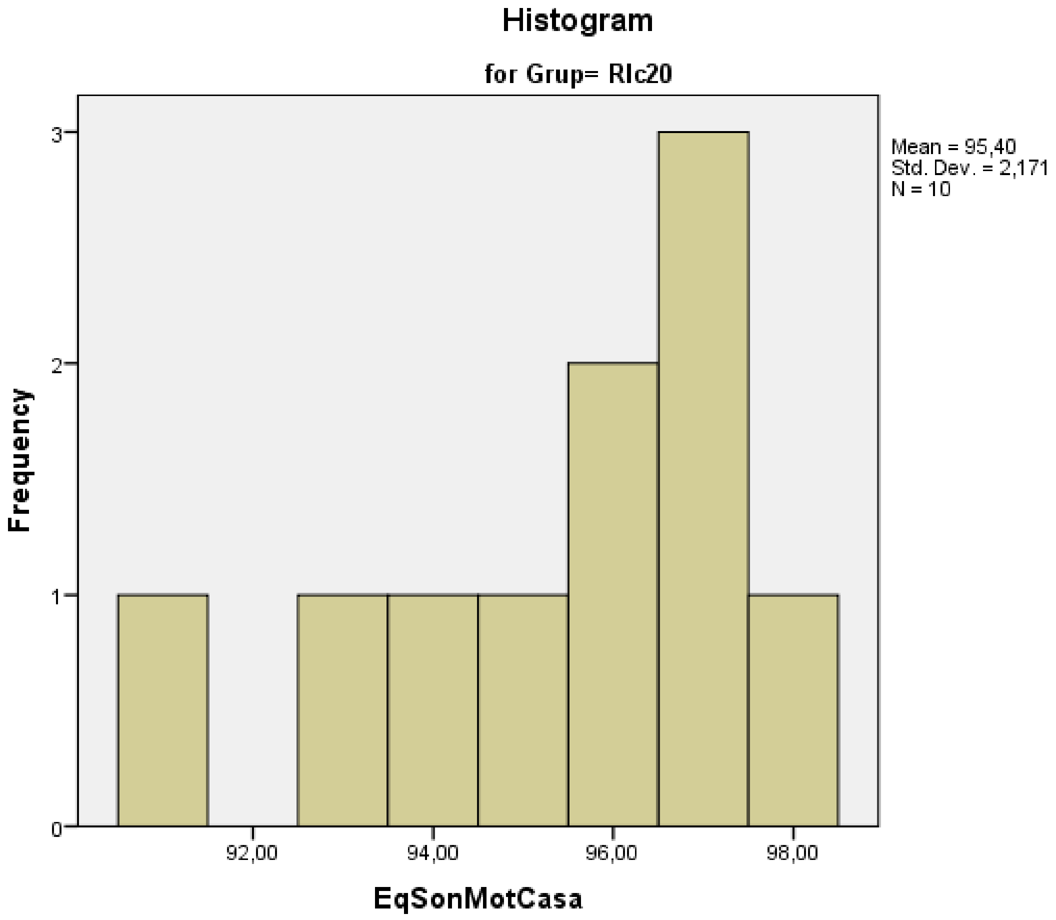
Histogram
for Grup= RIc20
Mean = 95,40
Std. Dev. = 2,171
N = 10
Frequency
0
1
2
3
92,00
94,00
96,00
98,00
EqSonMotCasa

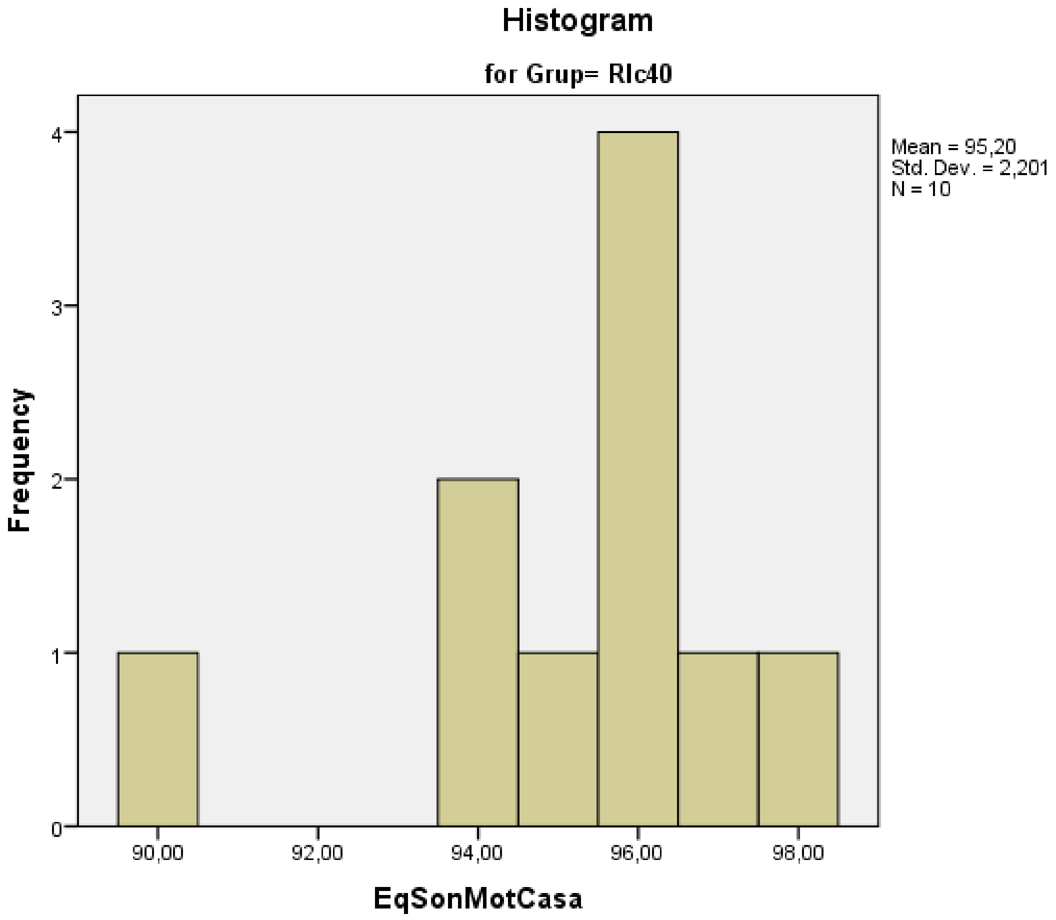

Stem-and-Leaf Plots

```
EqSonMotCasa Stem-and-Leaf Plot for
Grup= kontrol

 Frequency    Stem &  Leaf

     1,00        8 .  4
     1,00        8 .  8
     5,00        9 .  01124
     3,00        9 .  667

 Stem width:     10,00
 Each leaf:       1 case(s)
```

```
EqSonMotCasa Stem-and-Leaf Plot for
Grup= Res10

 Frequency    Stem &  Leaf

     2,00       93 .  00
     1,00       94 .  0
      ,00       95 .
      ,00       96 .
     4,00       97 .  0000
     3,00       98 .  000

 Stem width:      1,00
 Each leaf:       1 case(s)
```

```
EqSonMotCasa Stem-and-Leaf Plot for
Grup= Res20

 Frequency    Stem &  Leaf

     1,00       93 .  0
      ,00       94 .
     3,00       95 .  000
     3,00       96 .  000
      ,00       97 .
     3,00       98 .  000

 Stem width:      1,00
 Each leaf:       1 case(s)
```

```
EqSonMotCasa Stem-and-Leaf Plot for
Grup= Res40

 Frequency    Stem &  Leaf

      ,00        8 .
     1,00        8 .  5
     3,00        9 .  111
     6,00        9 .  568888

 Stem width:     10,00
 Each leaf:       1 case(s)
```

```
EqSonMotCasa Stem-and-Leaf Plot for
Grup= sık10

 Frequency    Stem &  Leaf
```

```
     1,00        8 .  9
     6,00        9 .  012334
     3,00        9 .  588

 Stem width:     10,00
 Each leaf:       1 case(s)
```

```
EqSonMotCasa Stem-and-Leaf Plot for
Grup= sık20

 Frequency    Stem &  Leaf

     1,00 Extremes    (=<82,0)
     1,00       92 .  0
     1,00       93 .  0
     4,00       94 .  0000
      ,00       95 .
     1,00       96 .  0
     2,00       97 .  00

 Stem width:      1,00
 Each leaf:       1 case(s)
```

```
EqSonMotCasa Stem-and-Leaf Plot for
Grup= sık40

 Frequency    Stem &  Leaf

     1,00        8 .  9
     5,00        9 .  11344
     4,00        9 .  5566

 Stem width:     10,00
 Each leaf:       1 case(s)
```

```
EqSonMotCasa Stem-and-Leaf Plot for
Grup= Rlc10

 Frequency    Stem &  Leaf

     1,00       93 .  0
     3,00       94 .  000
     1,00       95 .  0
     2,00       96 .  00
     3,00       97 .  000
```

```
 Stem width:      1,00
 Each leaf:       1 case(s)
```

```
EqSonMotCasa Stem-and-Leaf Plot for
Grup= Rlc20

 Frequency    Stem &  Leaf

     1,00        9 .  1
     1,00        9 .  3
     2,00        9 .  45
     5,00        9 .  66777
     1,00        9 .  8

 Stem width:     10,00
 Each leaf:       1 case(s)
```

```
EqSonMotCasa Stem-and-Leaf Plot for
Grup= Rlc40

 Frequency    Stem &  Leaf

     1,00 Extremes    (=<90,0)
     2,00       94 .  00
     1,00       95 .  0
     4,00       96 .  0000
     1,00       97 .  0
     1,00       98 .  0

 Stem width:      1,00
 Each leaf:       1 case(s)
```

Normal Q-Q Plots

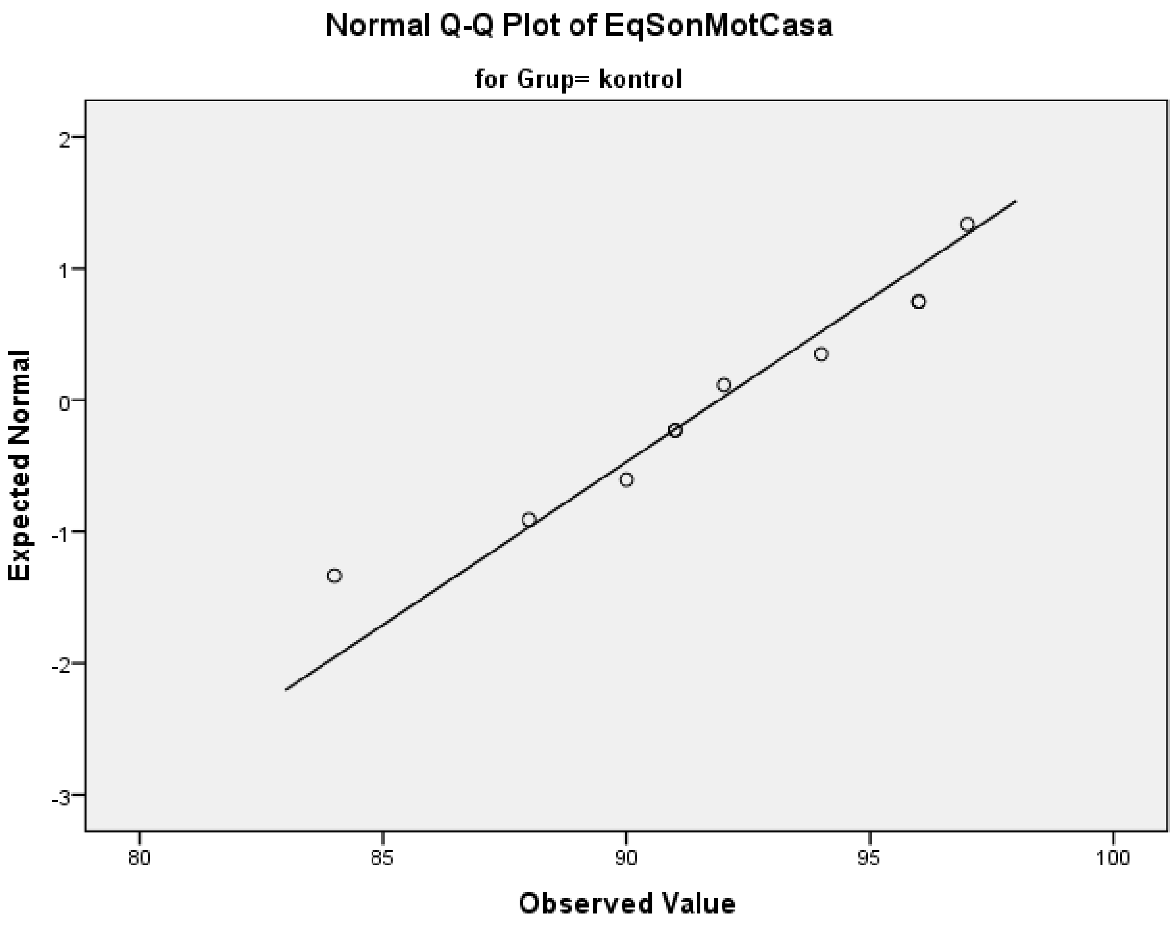

Normal Q-Q Plot of EqSonMotCasa
for Grup= kontrol
Expected Normal
Observed Value
2
1
0
-1
-2
-3
80
85
90
95
100

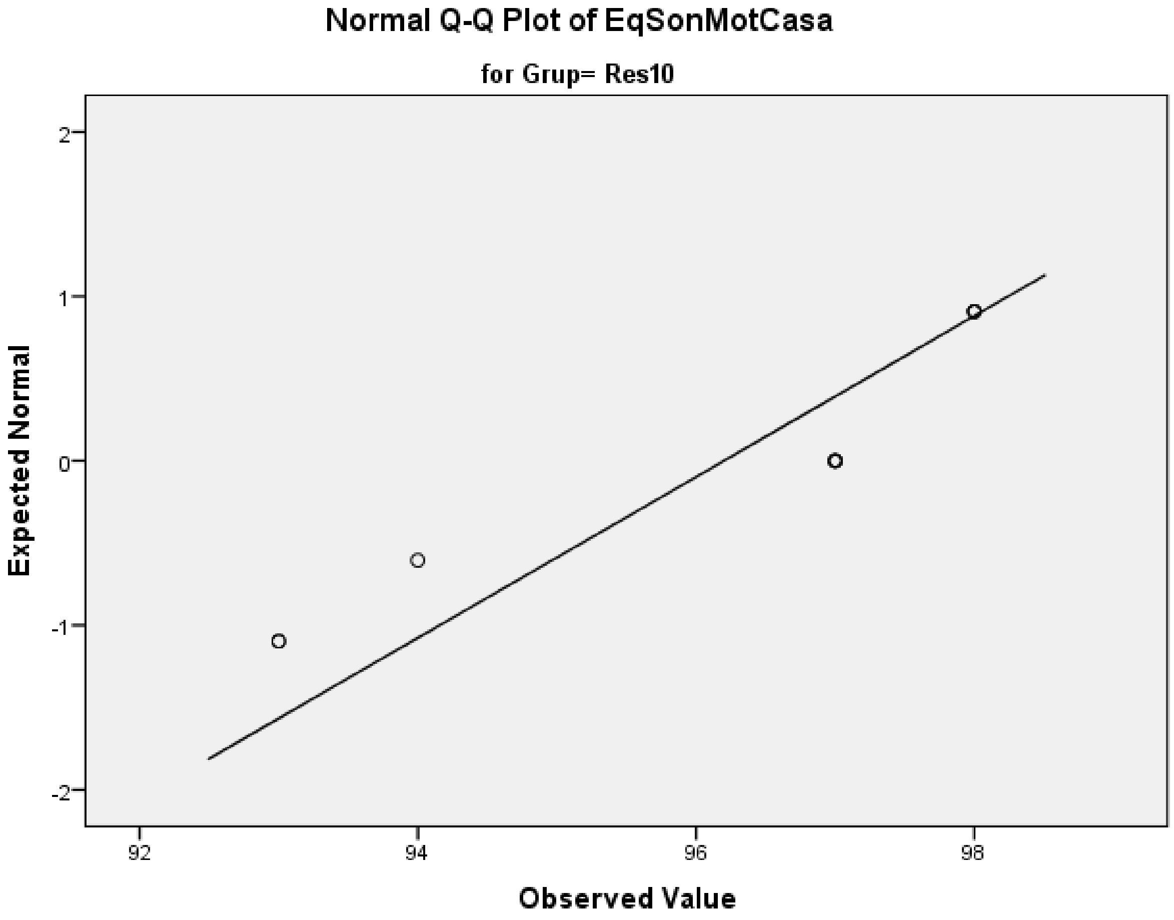
Normal Q-Q Plot of EqSonMotCasa
for Grup= Res10
Expected Normal
Observed Value
2
1
0
-1
-2
92
94
96
98

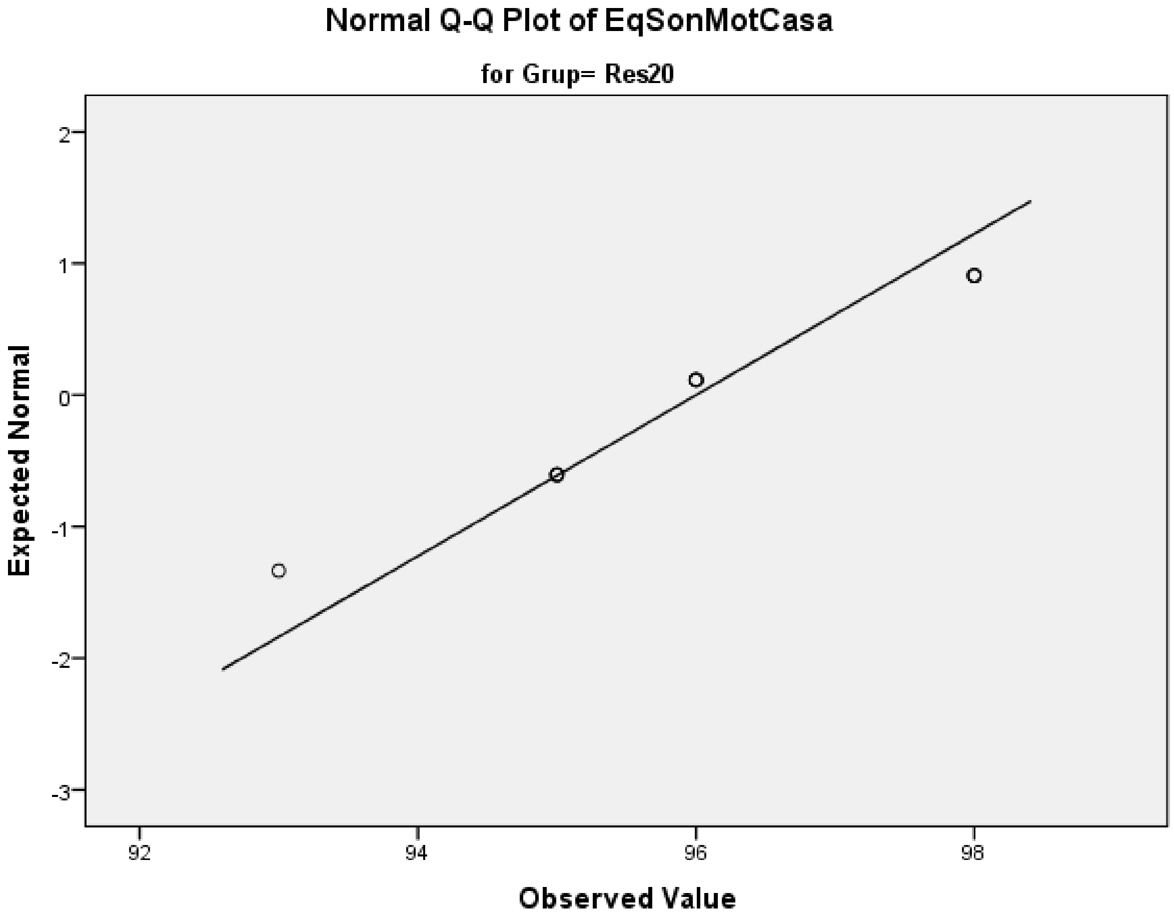

Normal Q-Q Plot of EqSonMotCasa
for Grup= Res20
Expected Normal
Observed Value
2
1
0
-1
-2
-3
92
94
96
98

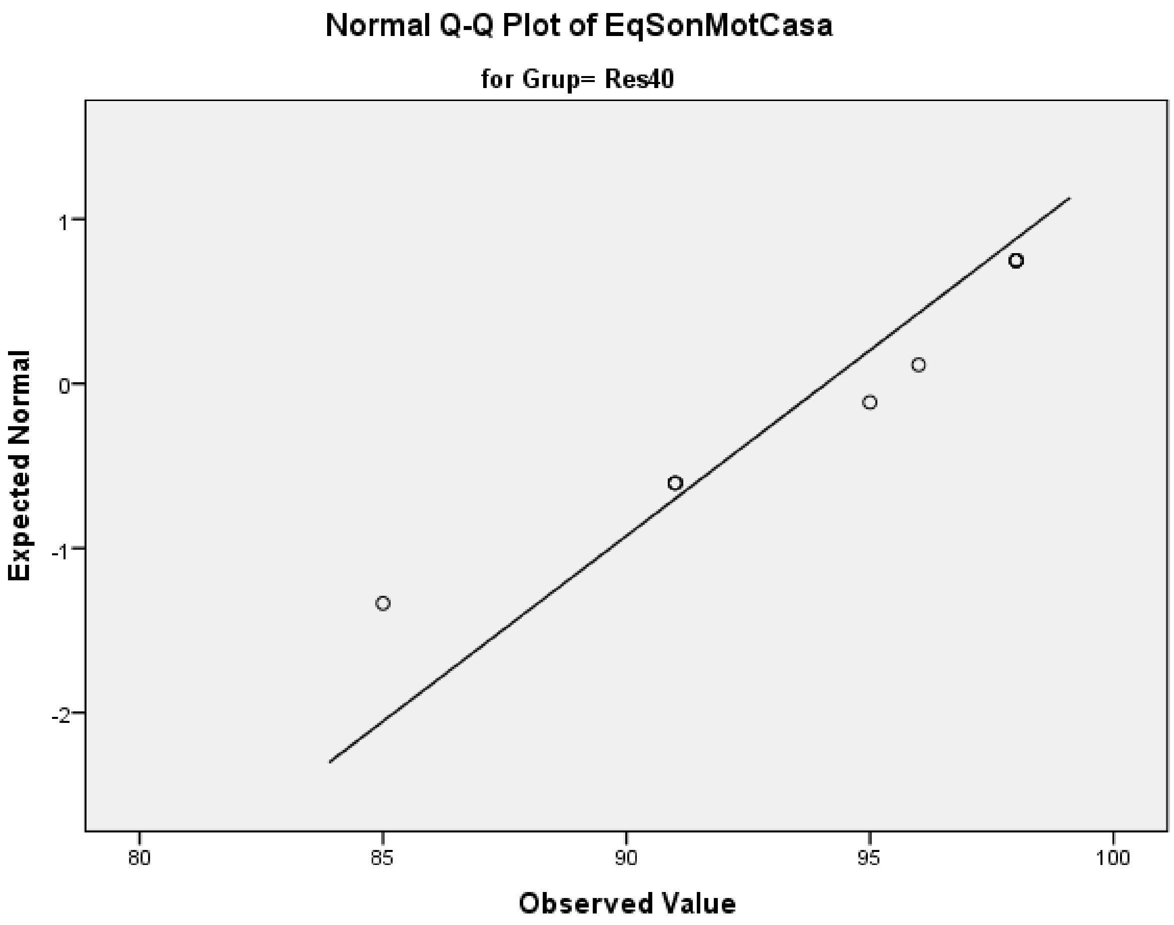

Normal Q-Q Plot of EqSonMotCasa
for Grup= Res40
Expected Normal
1
0
-1
-2
80
85
90
95
100
Observed Value

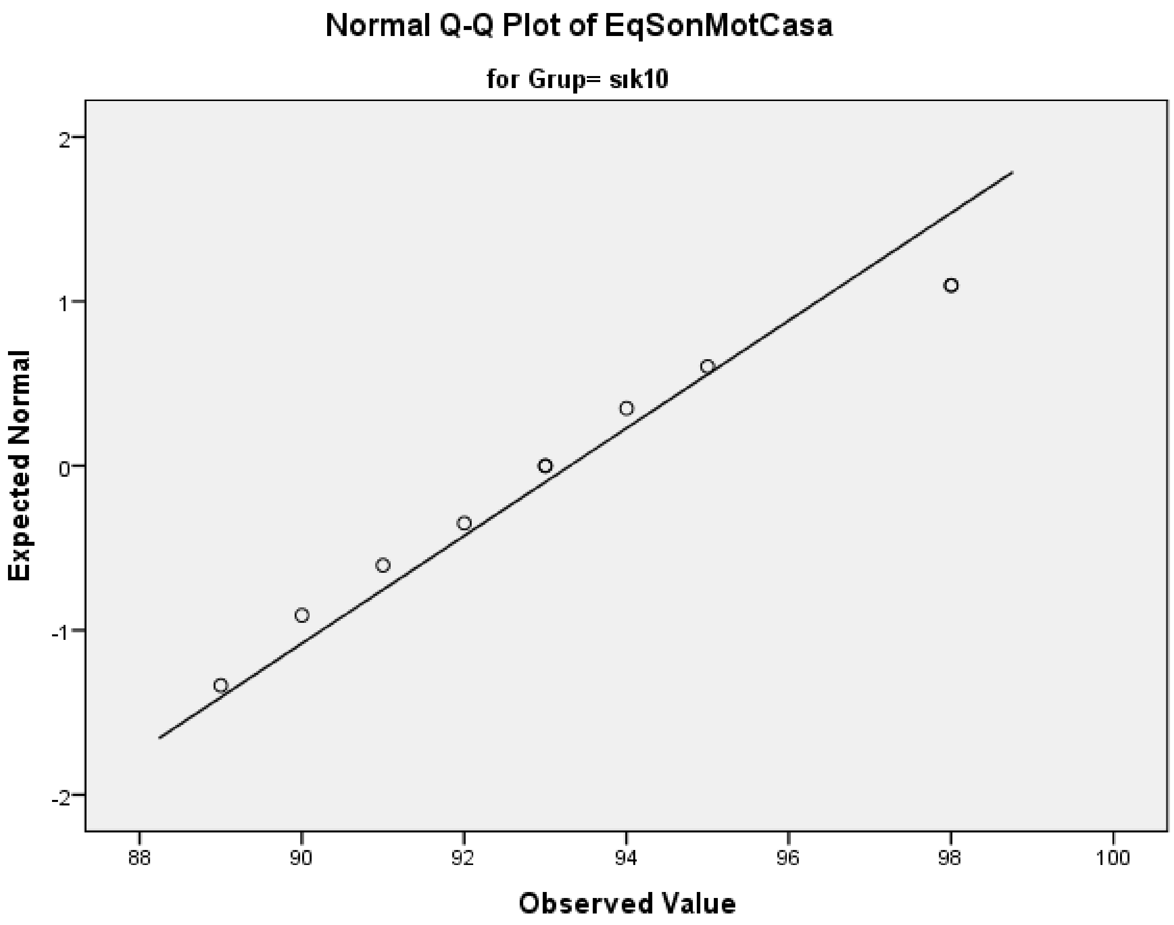
Normal Q-Q Plot of EqSonMotCasa
for Grup= sık10
Expected Normal
Observed Value
2
1
0
-1
-2
88
90
92
94
96
98
100

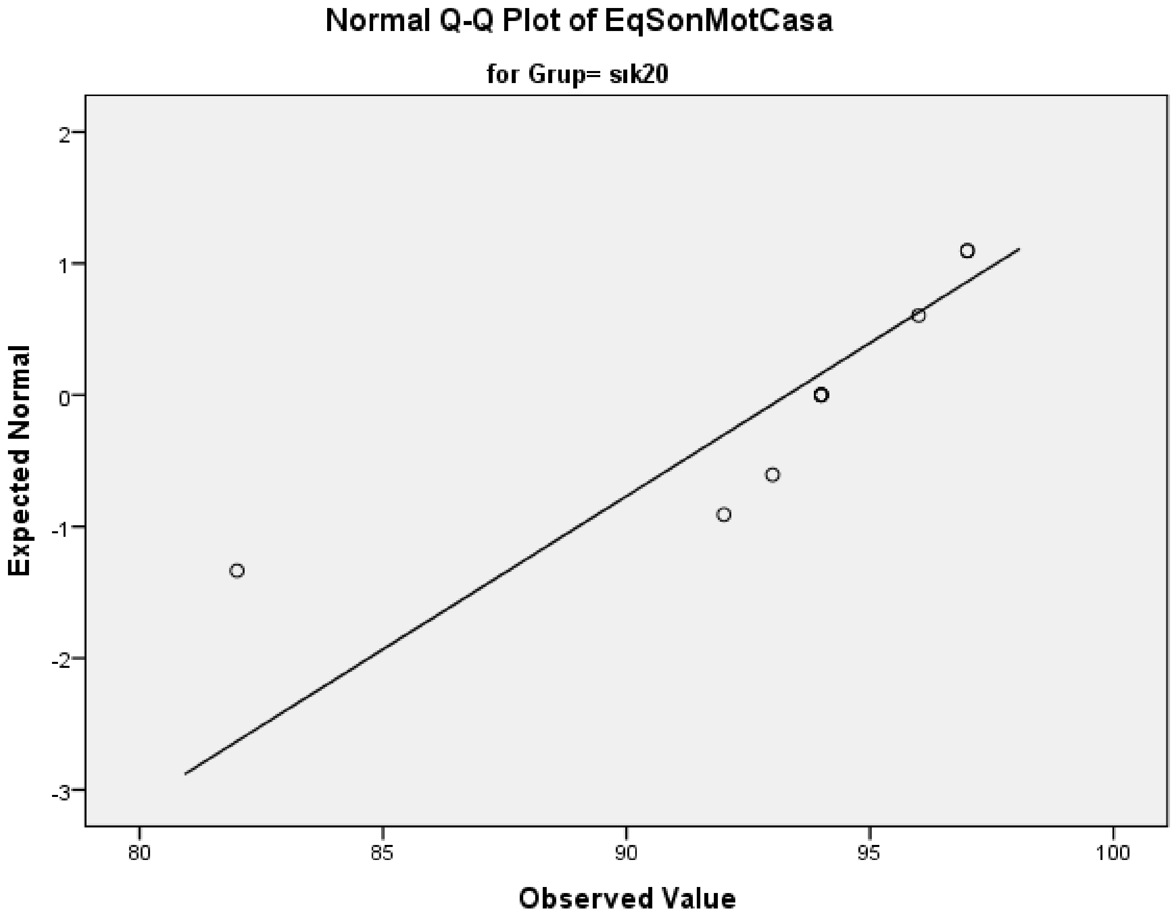
Normal Q-Q Plot of EqSonMotCasa
for Grup= sık20
Expected Normal
2
1
0
-1
-2
-3
80
85
90
95
100
Observed Value

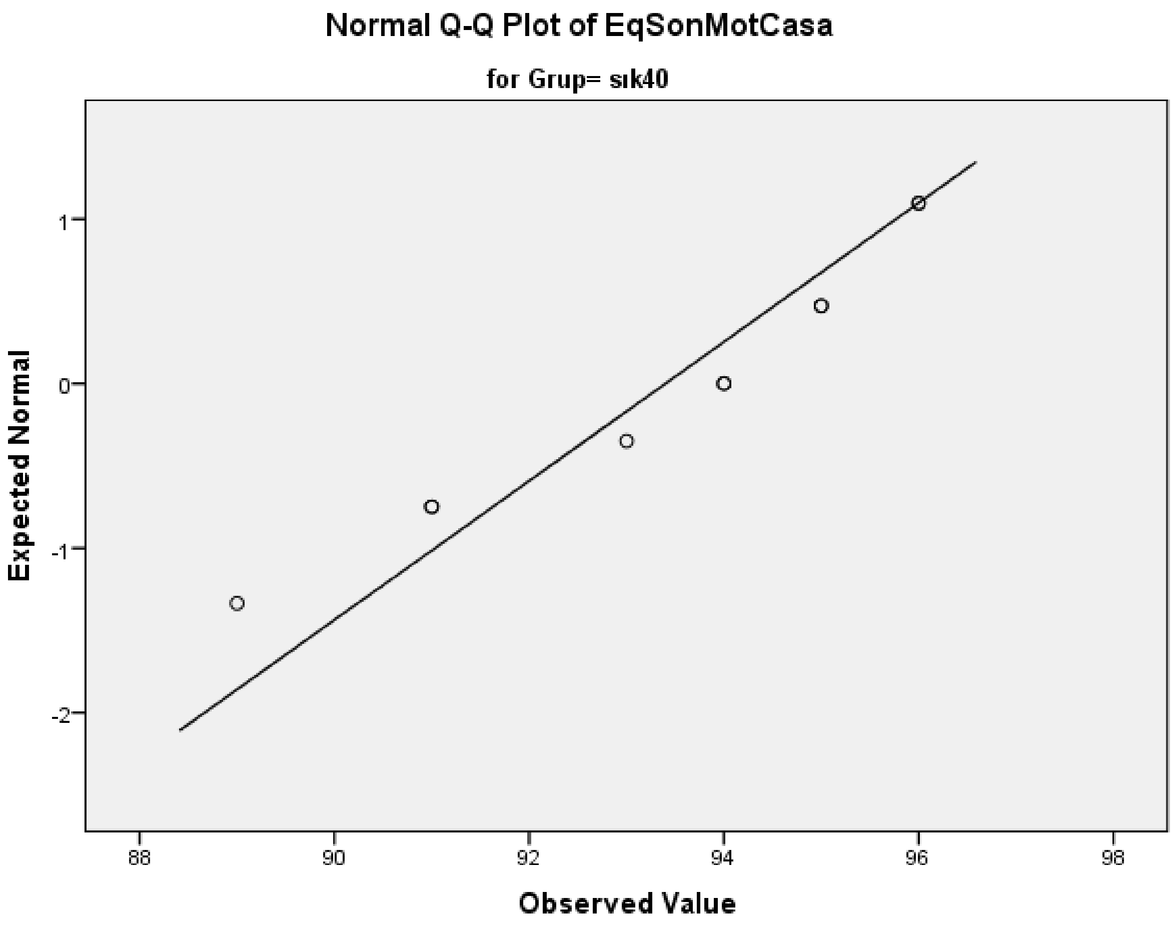
Normal Q-Q Plot of EqSonMotCasa
for Grup= sık40
Expected Normal
Observed Value
1
0
-1
-2
88
90
92
94
96
98

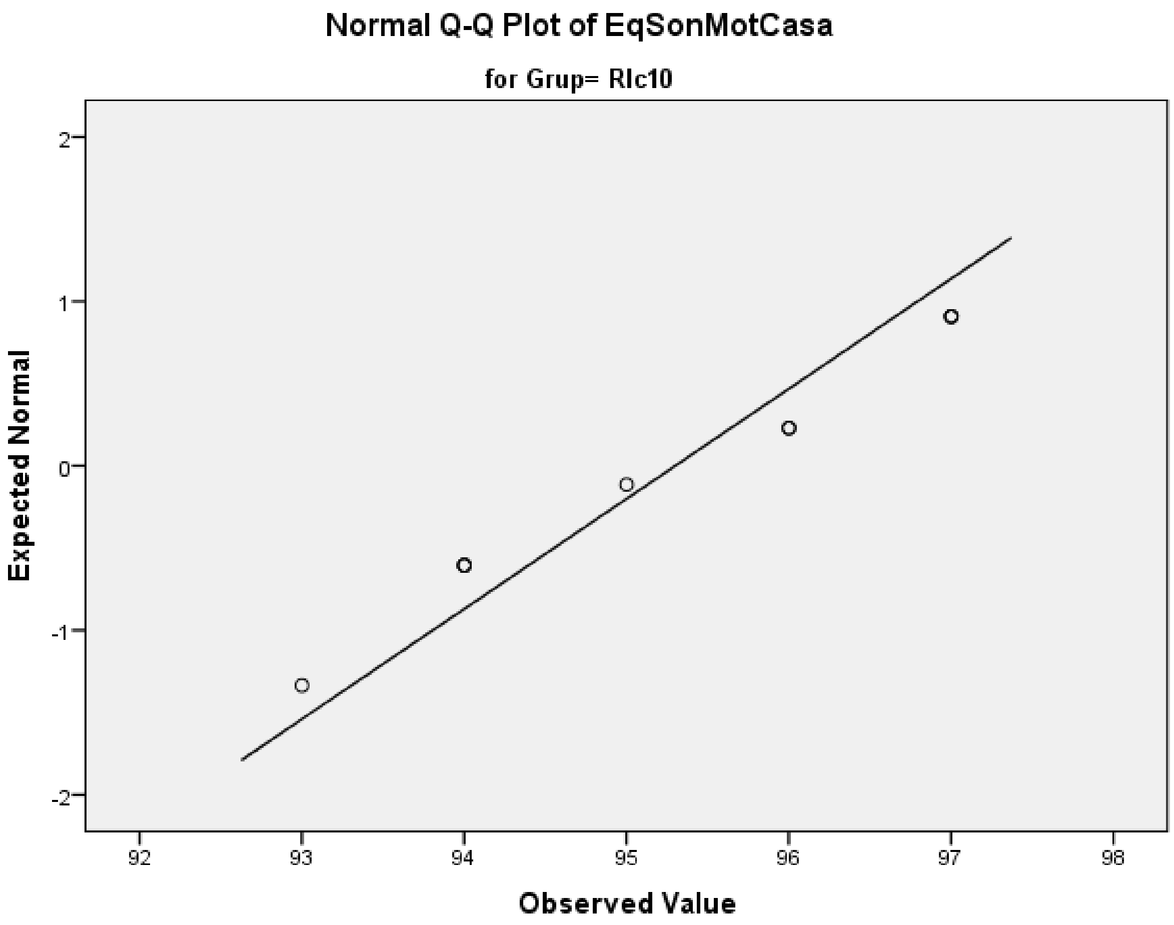
Normal Q-Q Plot of EqSonMotCasa
for Grup= Rlc10
Expected Normal
Observed Value
2
1
0
-1
-2
92
93
94
95
96
97
98

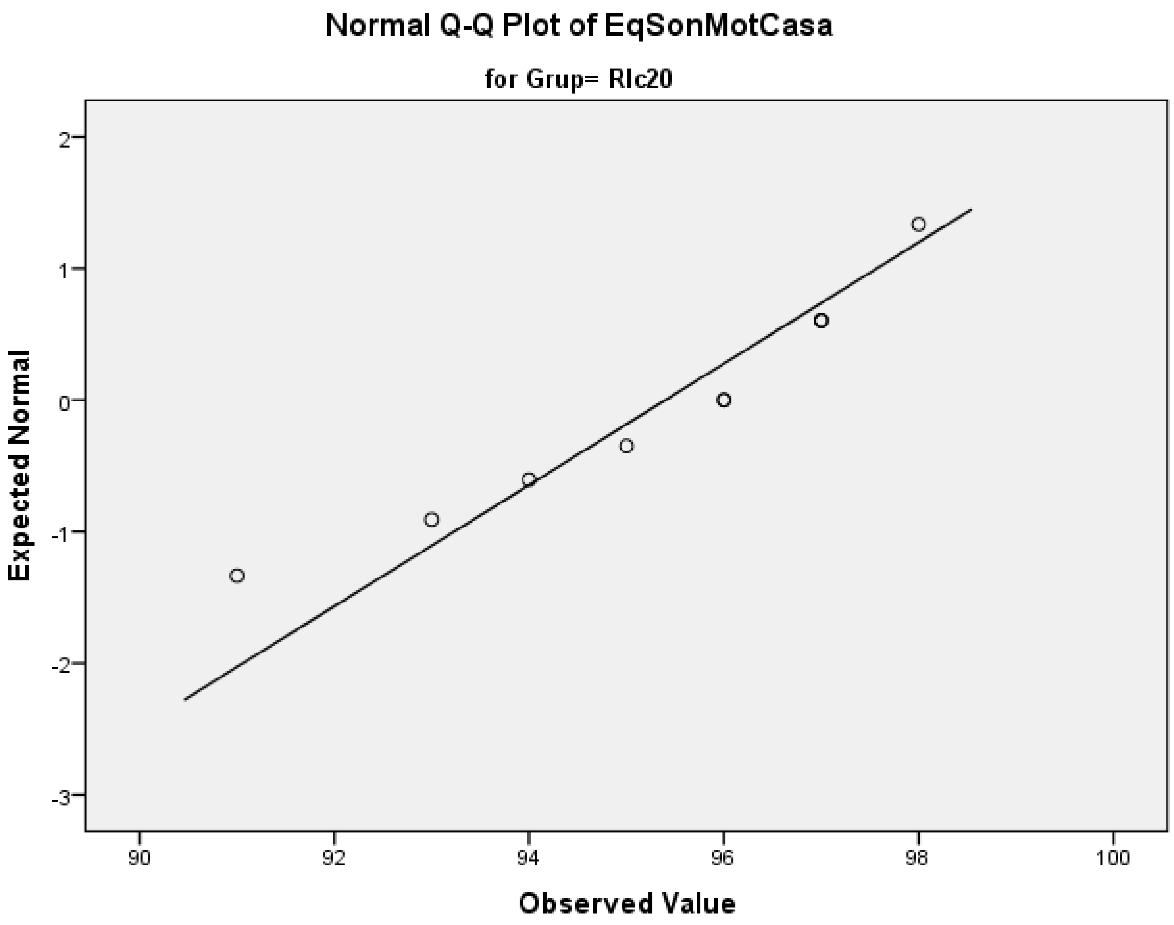
Normal Q-Q Plot of EqSonMotCasa
for Grup= RIc20
Expected Normal
Observed Value
2
1
0
-1
-2
-3
90
92
94
96
98
100

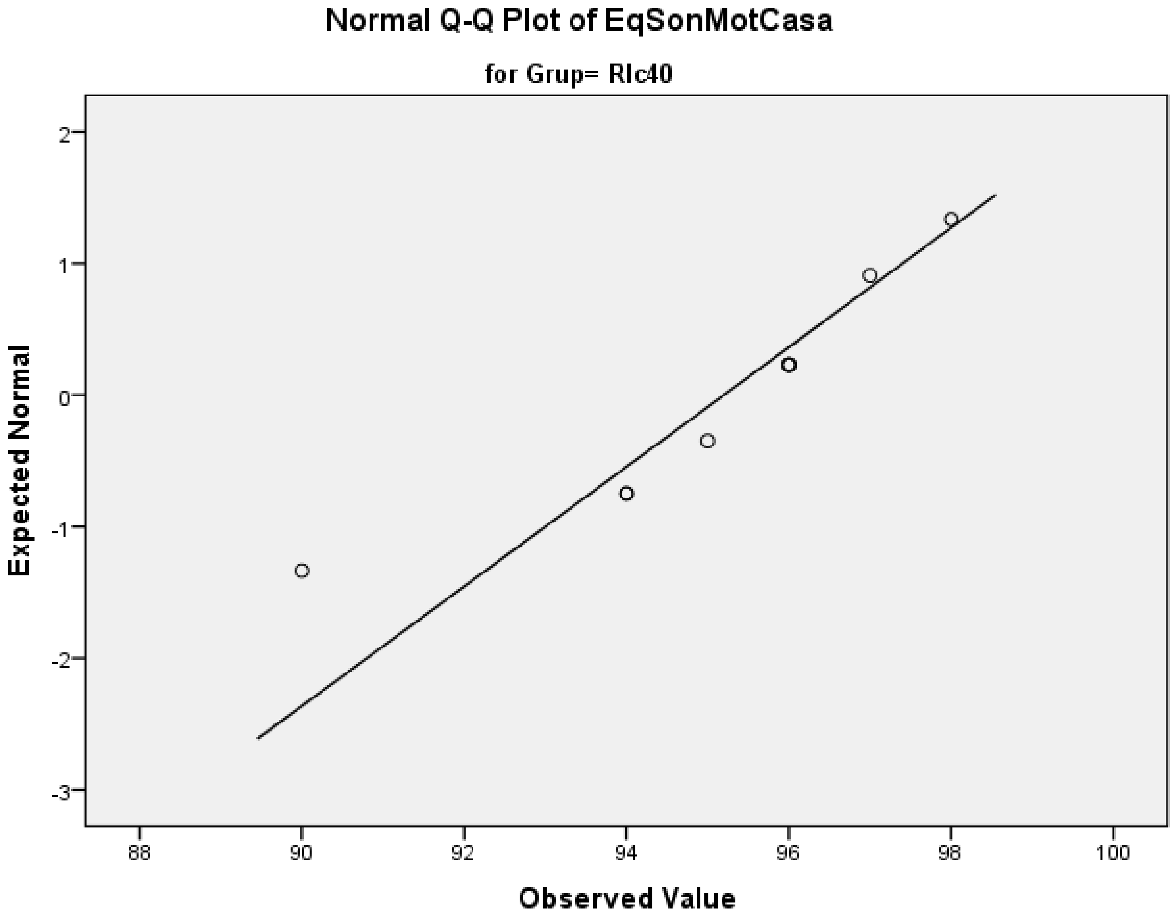

Detrended Normal Q-Q Plots

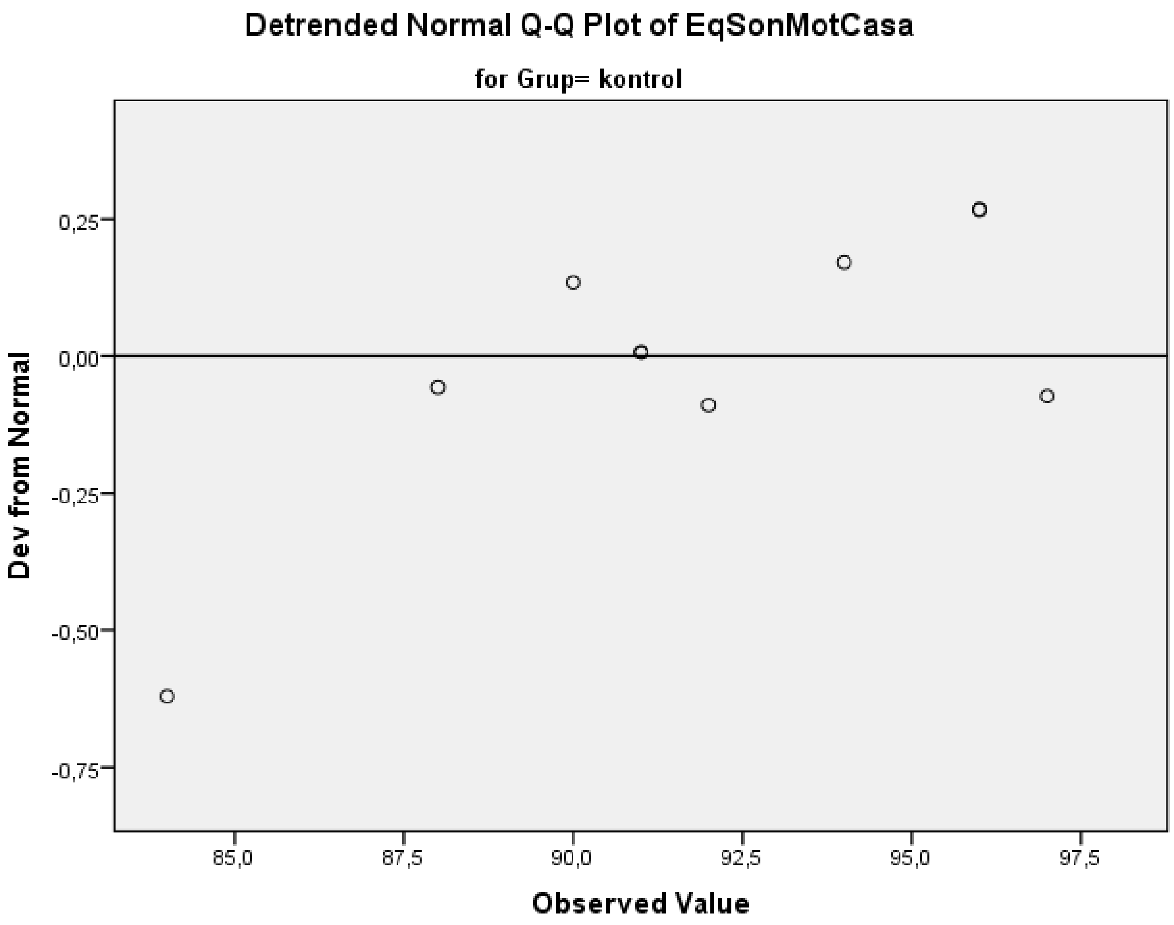
Detrended Normal Q-Q Plot of EqSonMotCasa
for Grup= kontrol
Dev from Normal
0,25
0,00
-0,25
-0,50
-0,75
85,0
87,5
90,0
92,5
95,0
97,5
Observed Value

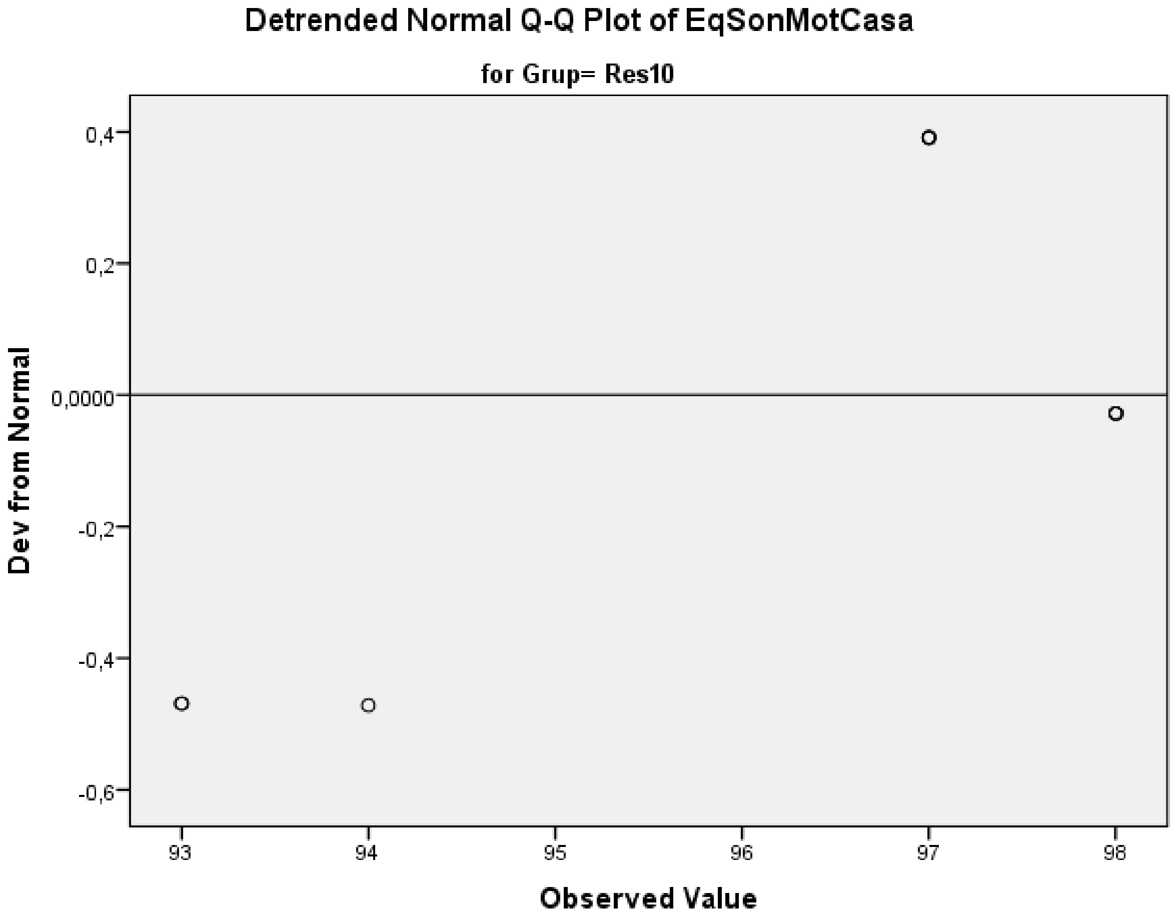

Detrended Normal Q-Q Plot of EqSonMotCasa
for Grup= Res10
Dev from Normal
0,4
0,2
0,0000
-0,2
-0,4
-0,6
93
94
95
96
97
98
Observed Value

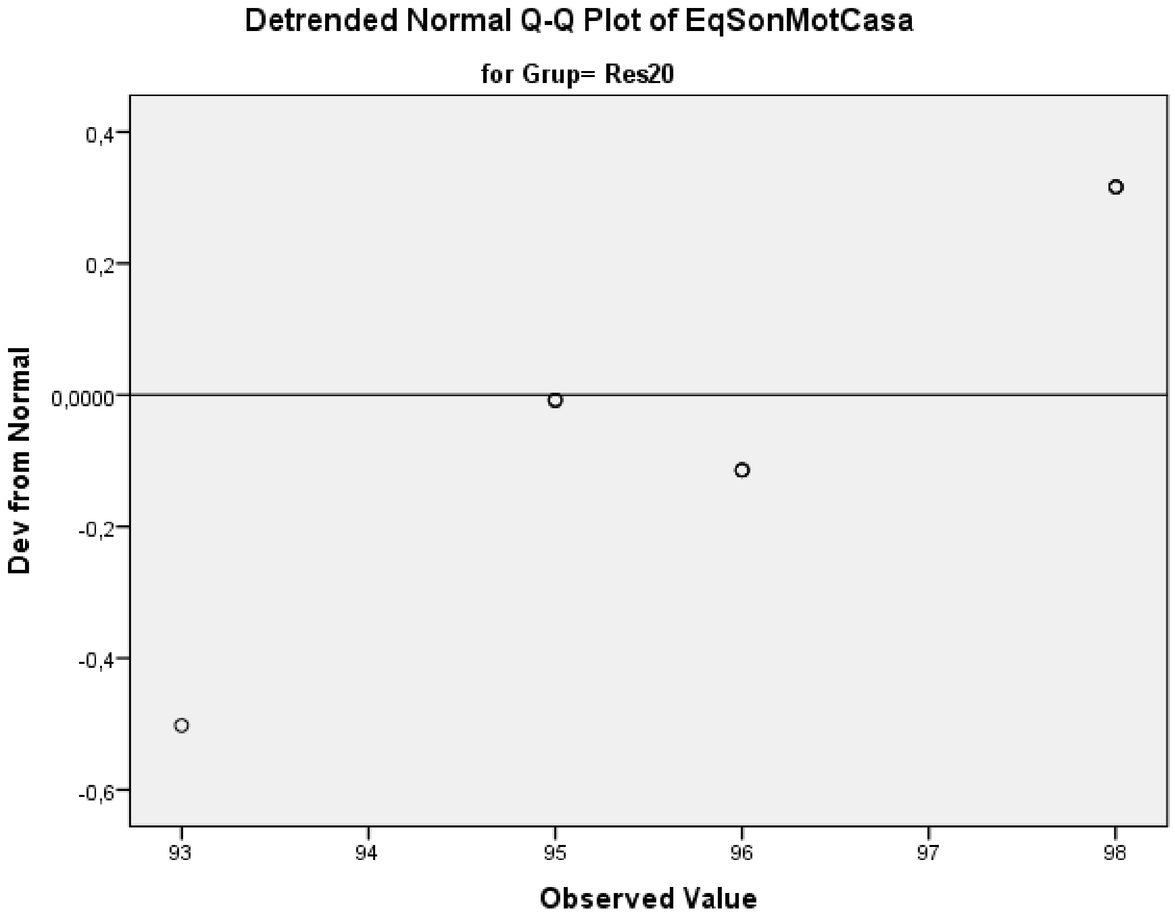
Detrended Normal Q-Q Plot of EqSonMotCasa
for Grup= Res20
0,4
0,2
0,0000
-0,2
-0,4
-0,6
Dev from Normal
93
94
95
96
97
98
Observed Value

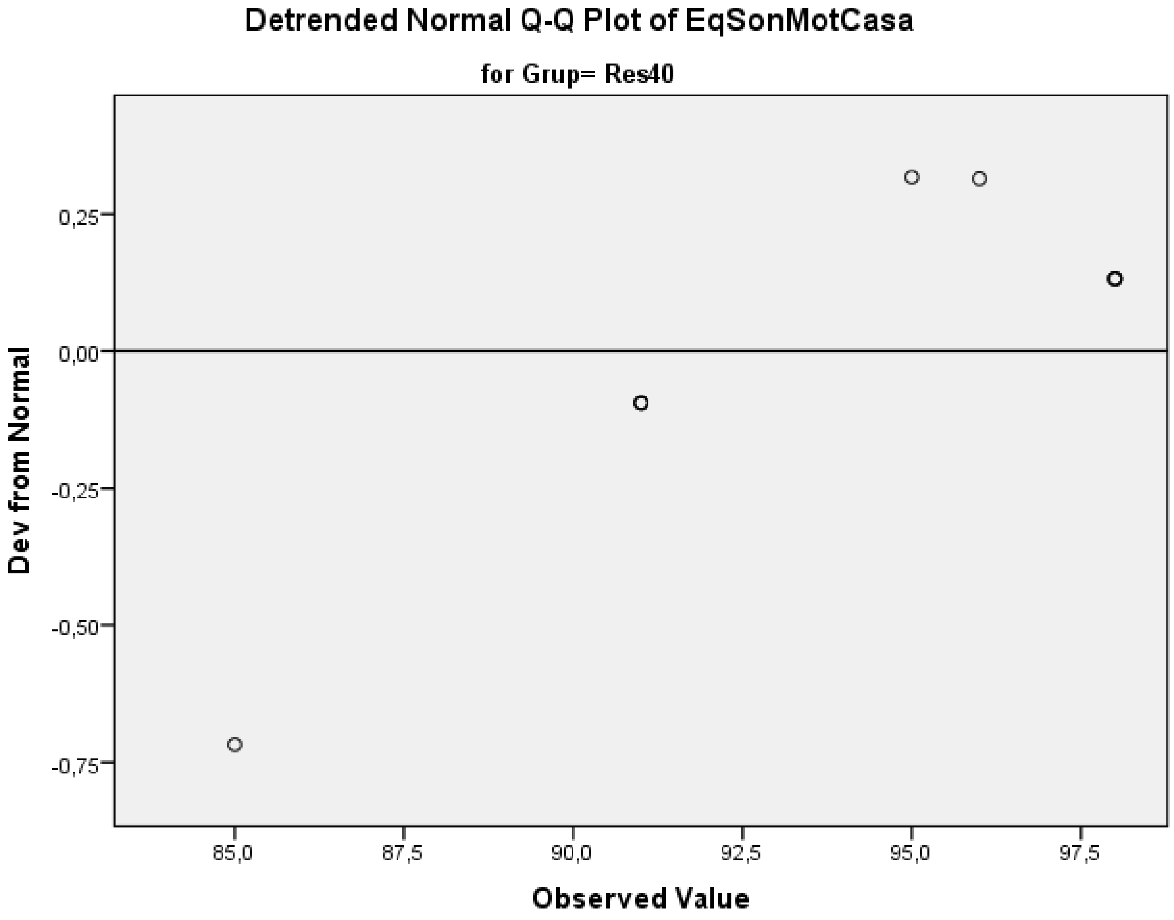
Detrended Normal Q-Q Plot of EqSonMotCasa
for Grup= Res40
Dev from Normal
0,25
0,00
-0,25
-0,50
-0,75
85,0
87,5
90,0
92,5
95,0
97,5
Observed Value

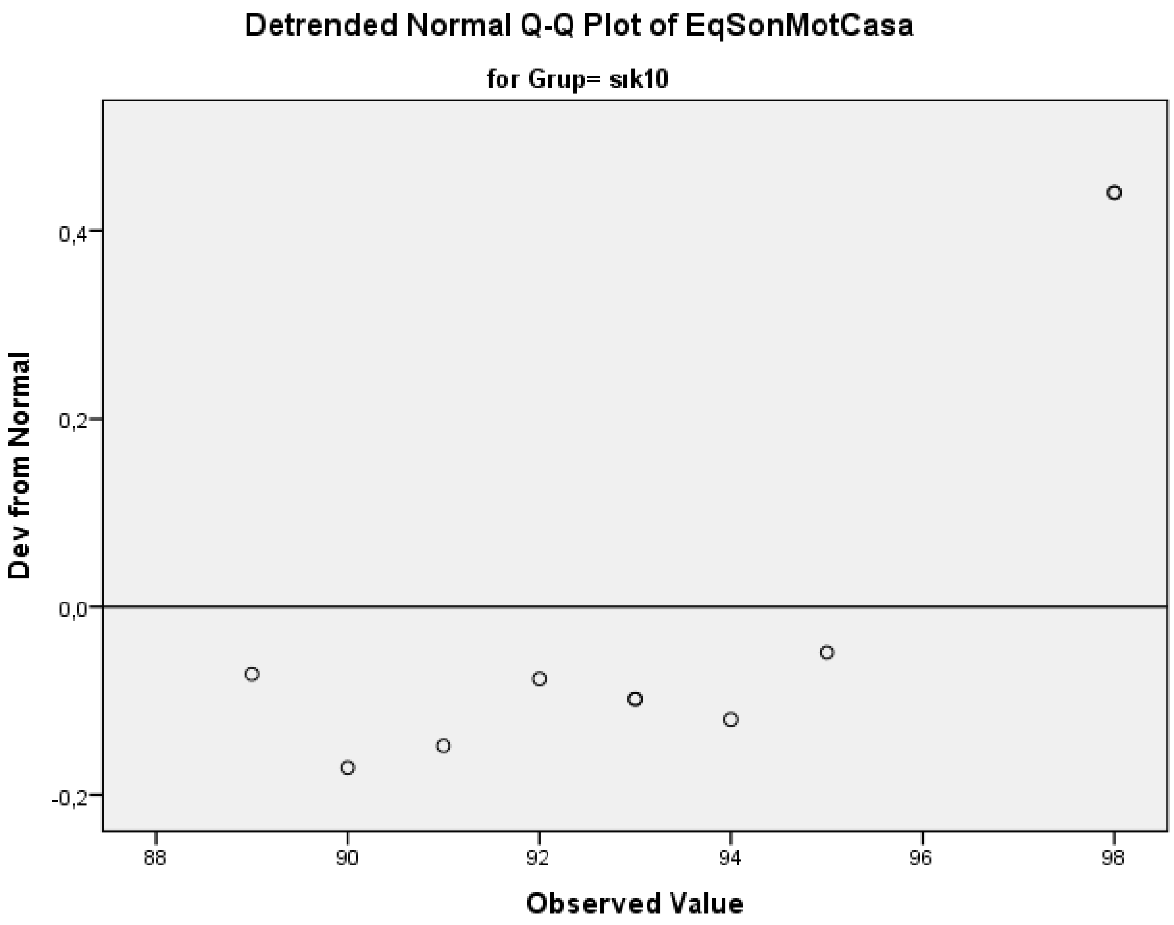
Detrended Normal Q-Q Plot of EqSonMotCasa
for Grup= sık10
Dev from Normal
0,4
0,2
0,0
-0,2
88
90
92
94
96
98
Observed Value

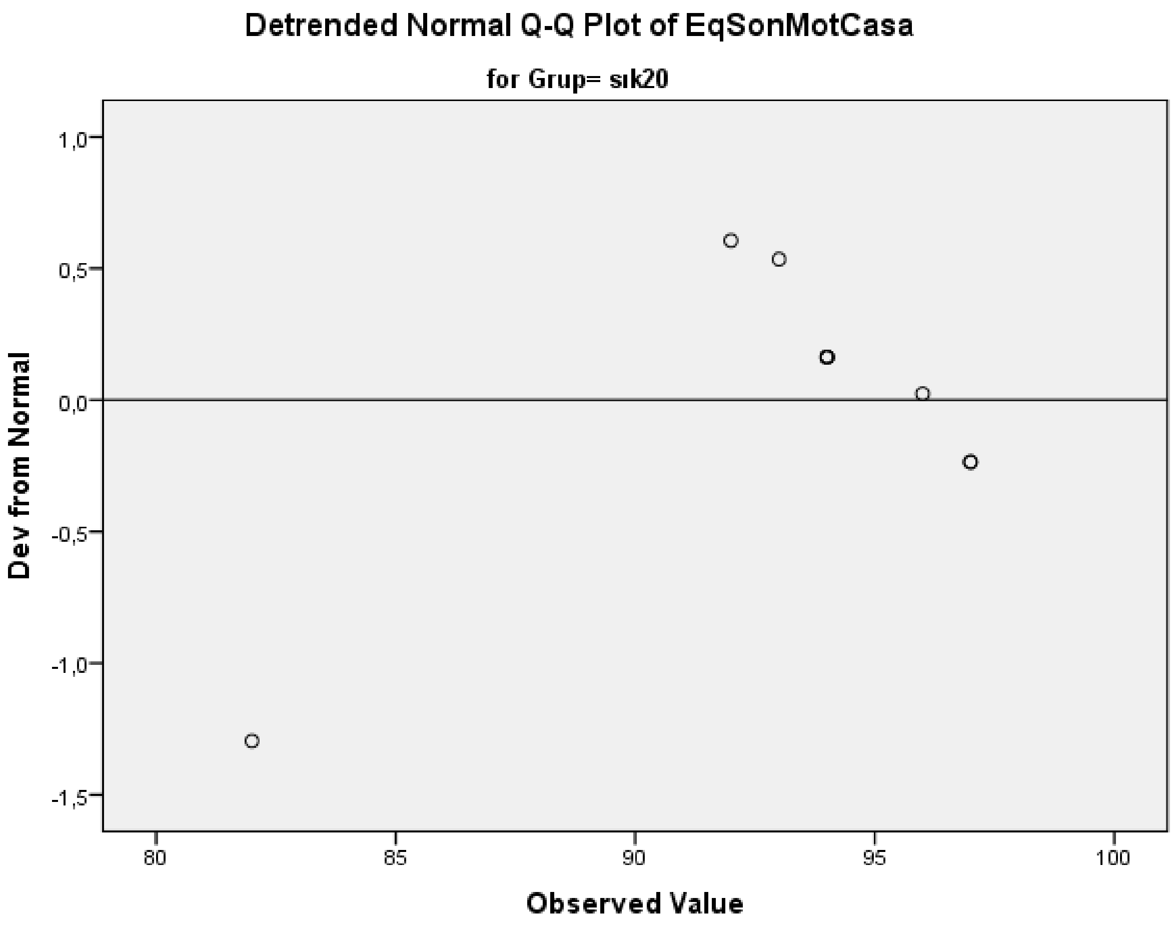
Detrended Normal Q-Q Plot of EqSonMotCasa
for Grup= sık20
Dev from Normal
1,0
0,5
0,0
-0,5
-1,0
-1,5
80
85
90
95
100
Observed Value

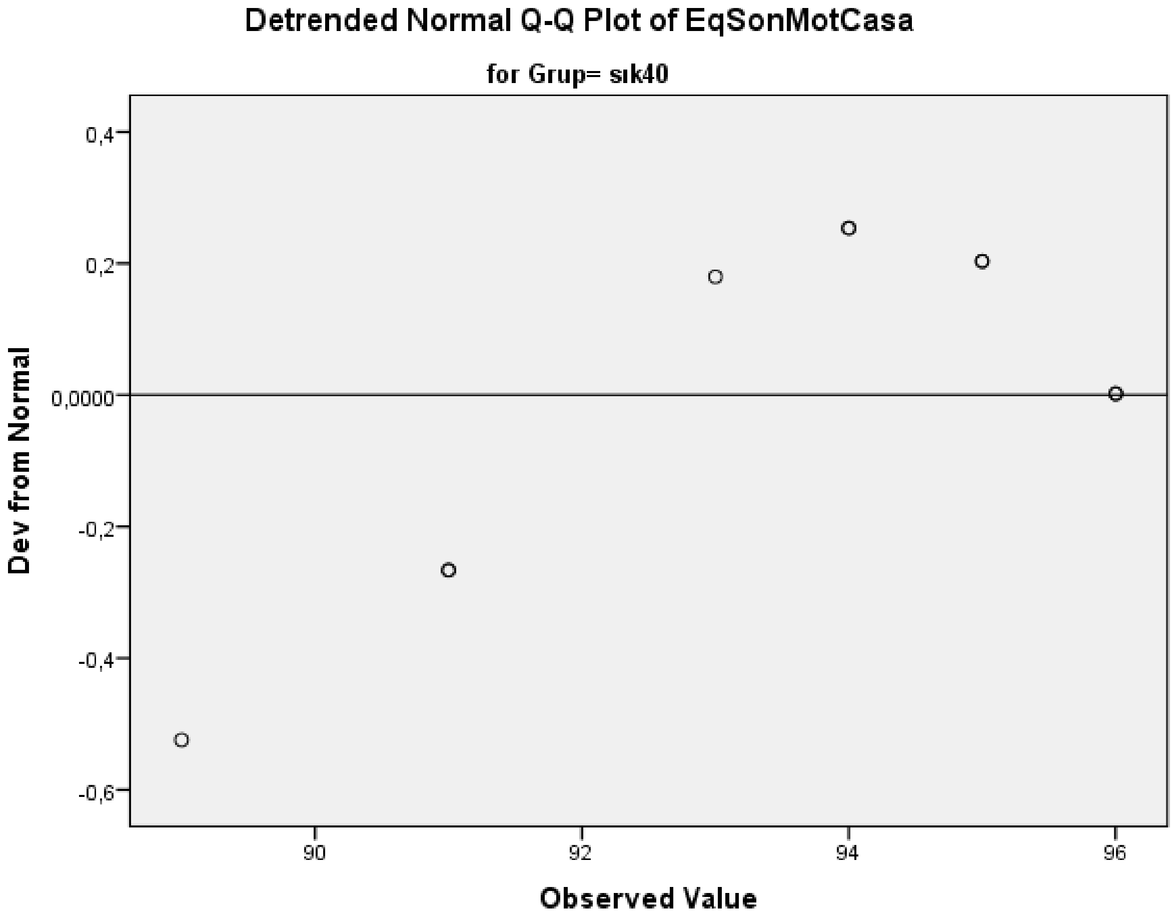

Detrended Normal Q-Q Plot of EqSonMotCasa
for Grup= sık40
Dev from Normal
0,4
0,2
0,0000
-0,2
-0,4
-0,6
90
92
94
96
Observed Value

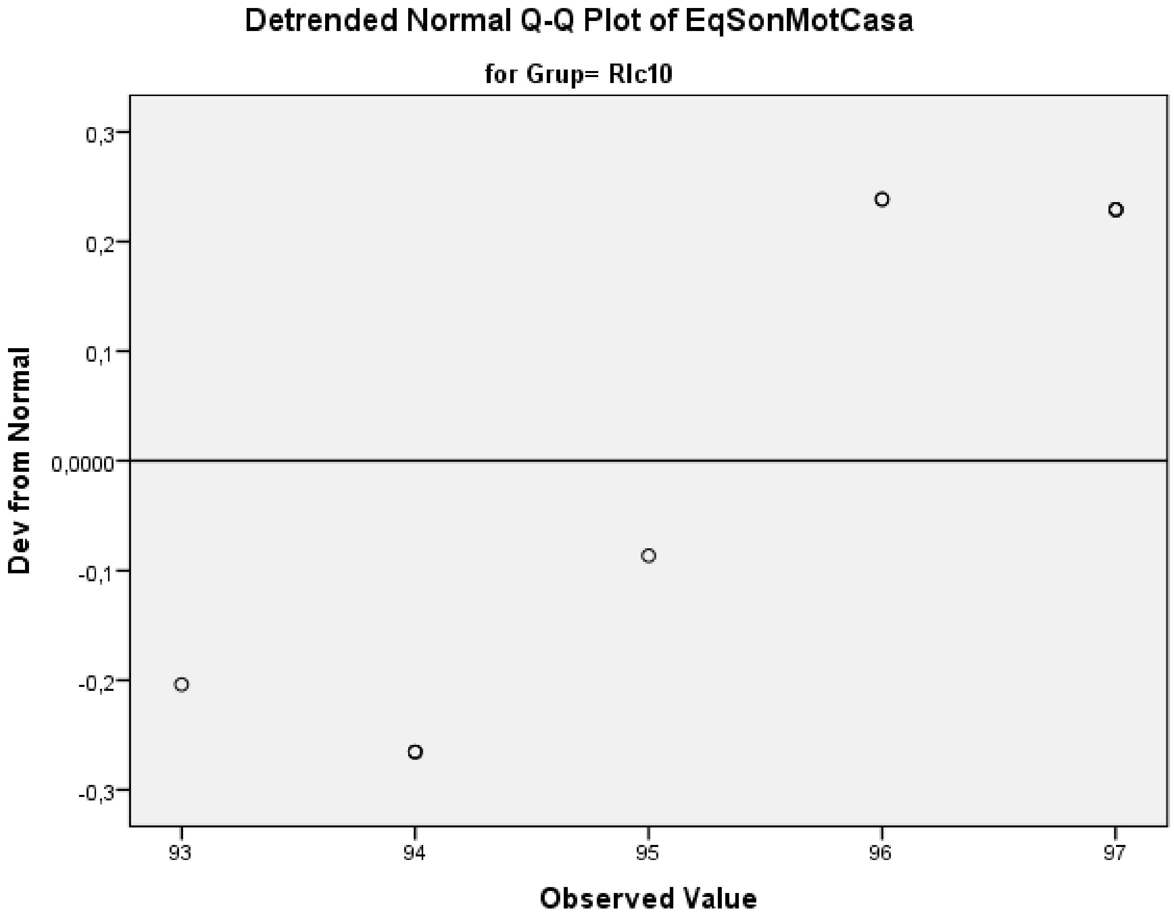
Detrended Normal Q-Q Plot of EqSonMotCasa
for Grup= Rlc10
Dev from Normal
0,3
0,2
0,1
0,0000
-0,1
-0,2
-0,3
93
94
95
96
97
Observed Value

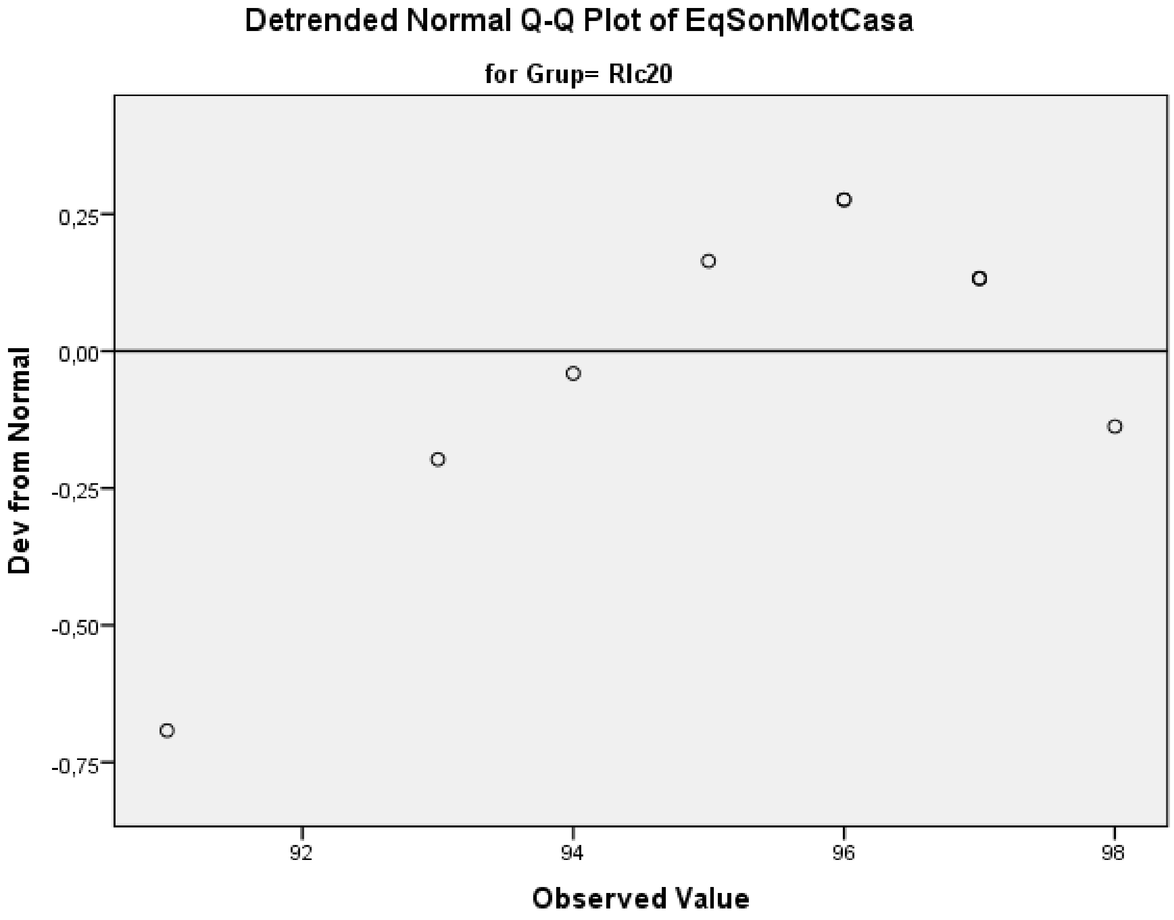

Detrended Normal Q-Q Plot of EqSonMotCasa
for Grup= RIc20
Dev from Normal
0,25
0,00
-0,25
-0,50
-0,75
92
94
96
98
Observed Value

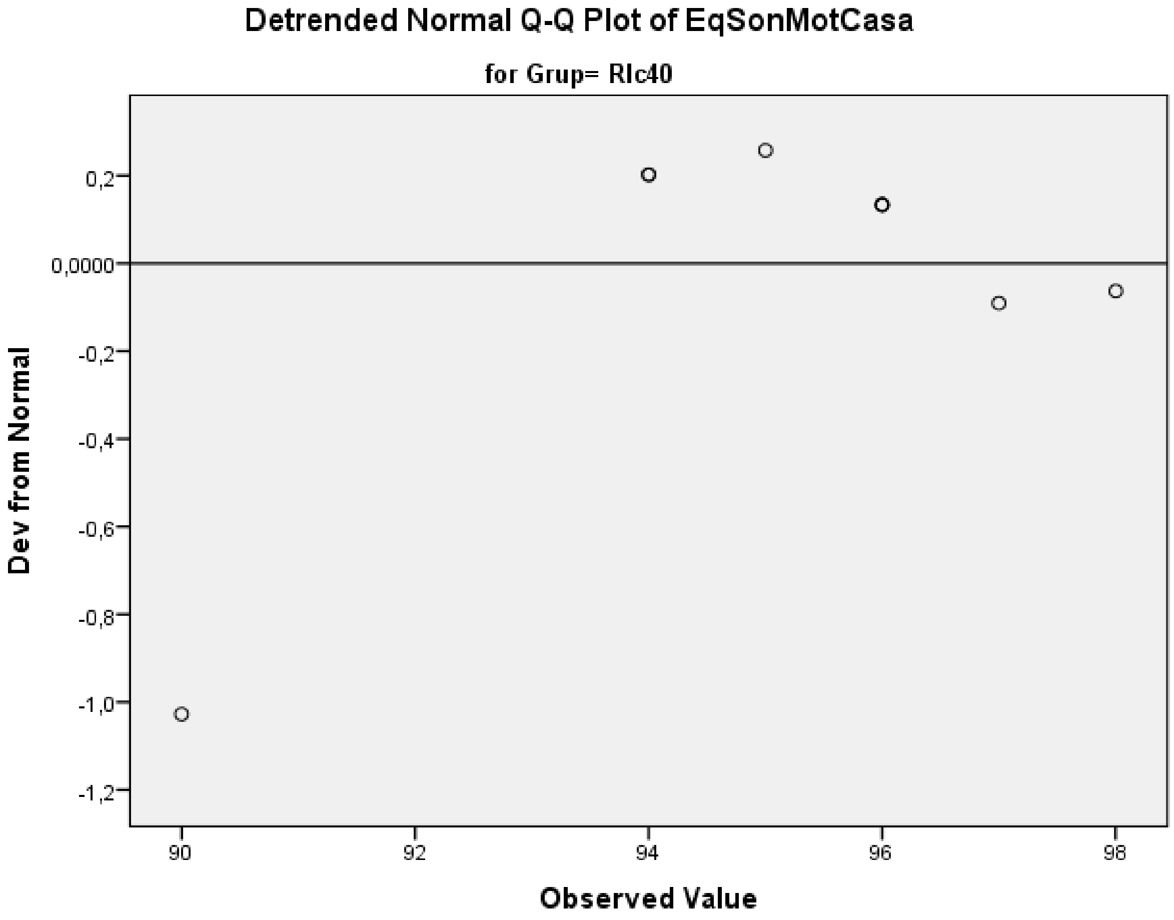

Detrended Normal Q-Q Plot of EqSonMotCasa
for Grup= Rlc40
Dev from Normal
0,2
0,0000
-0,2
-0,4
-0,6
-0,8
-1,0
-1,2
90
92
94
96
98
Observed Value

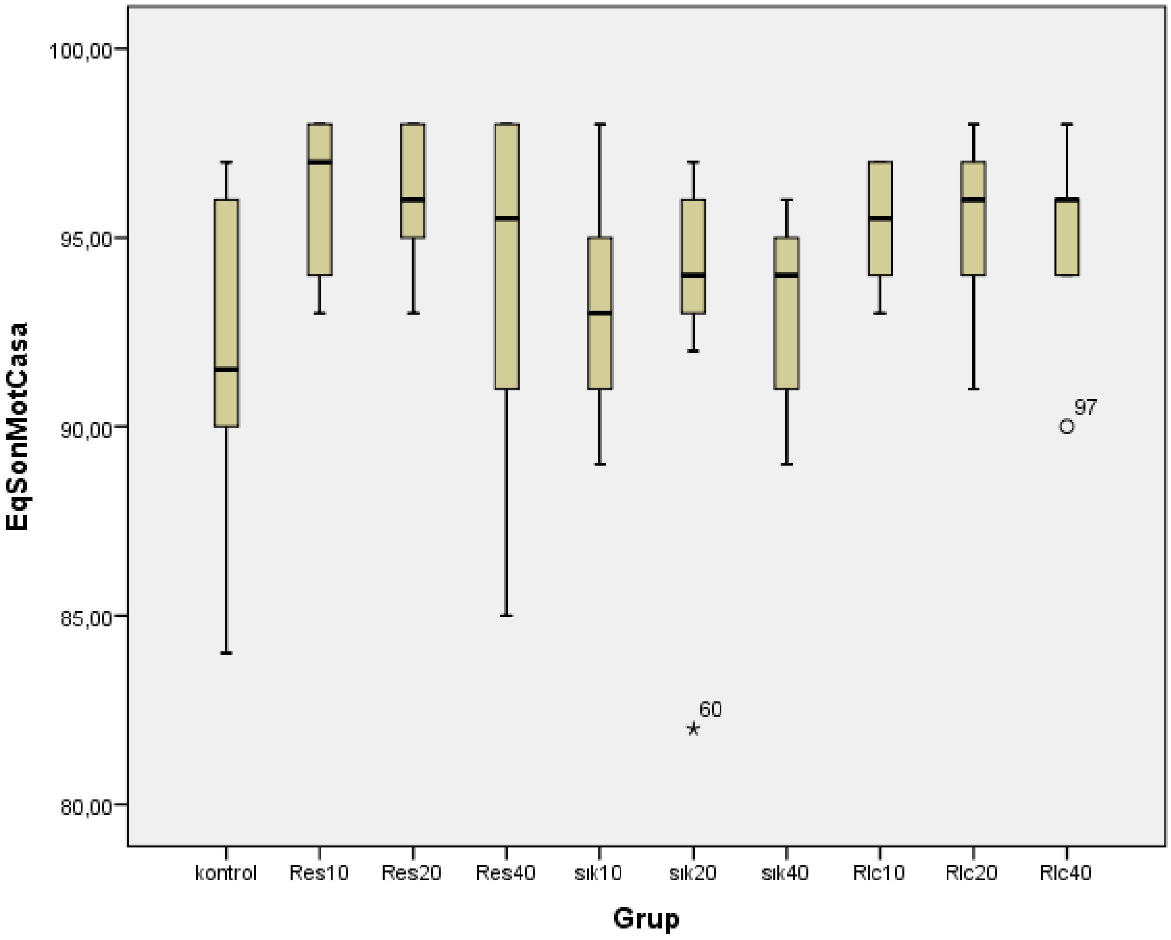

EqSonMotProg

Histograms

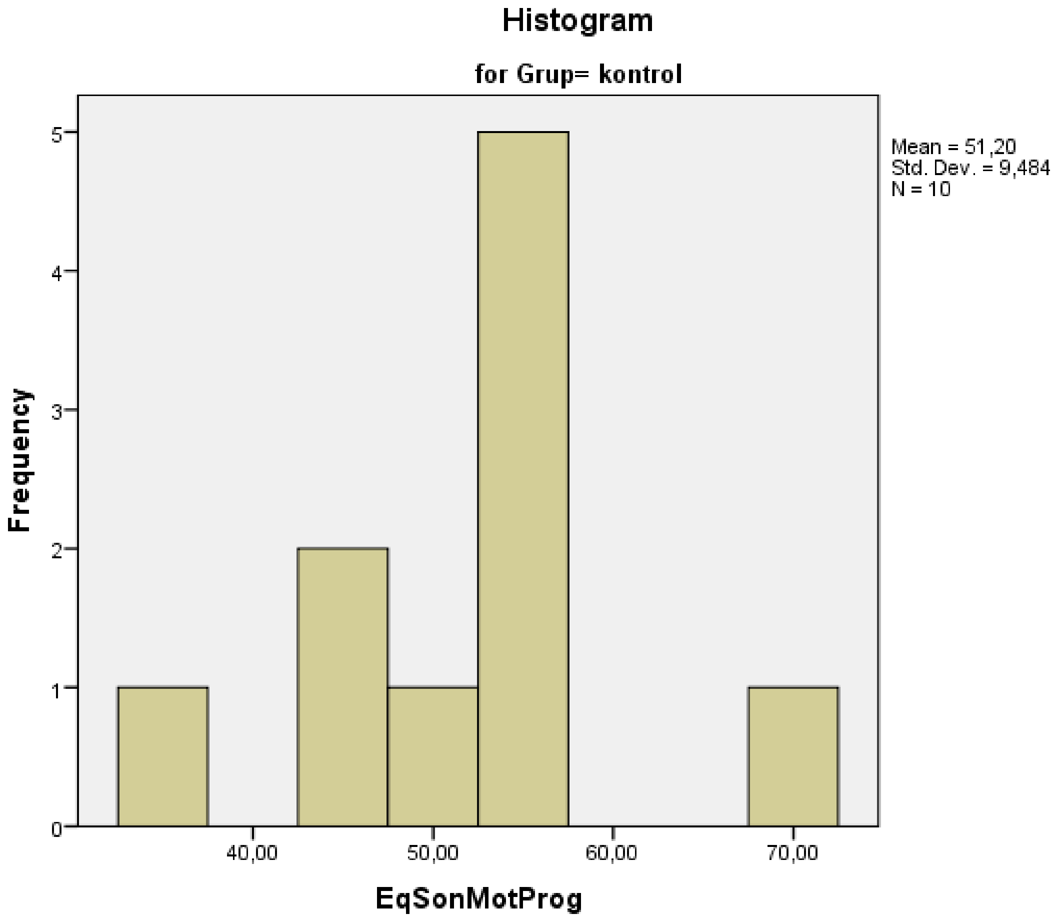

Histogram
for Grup= kontrol
Mean = 51,20
Std. Dev. = 9,484
N = 10
Frequency
0
1
2
3
4
5
40,00
50,00
60,00
70,00
EqSonMotProg

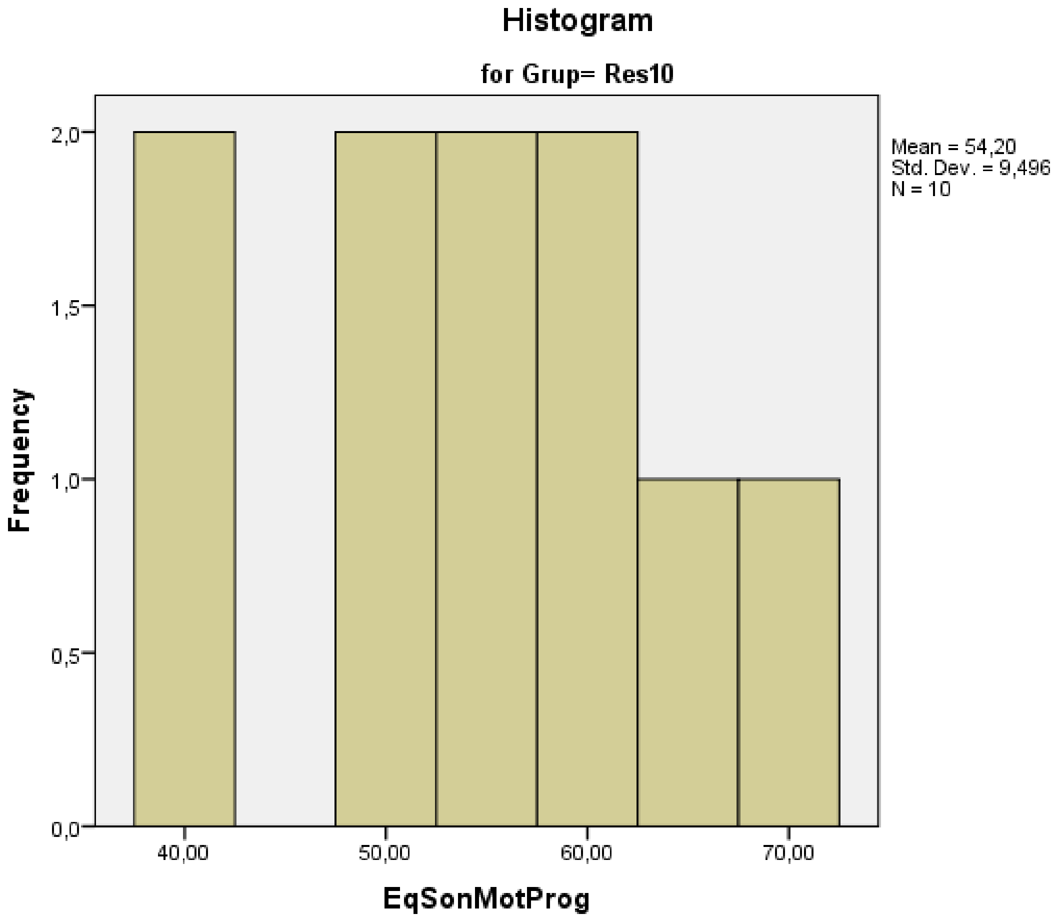

Histogram
for Grup= Res10
Mean = 54,20
Std. Dev. = 9,496
N = 10
Frequency
2,0
1,5
1,0
0,5
0,0
40,00
50,00
60,00
70,00
EqSonMotProg

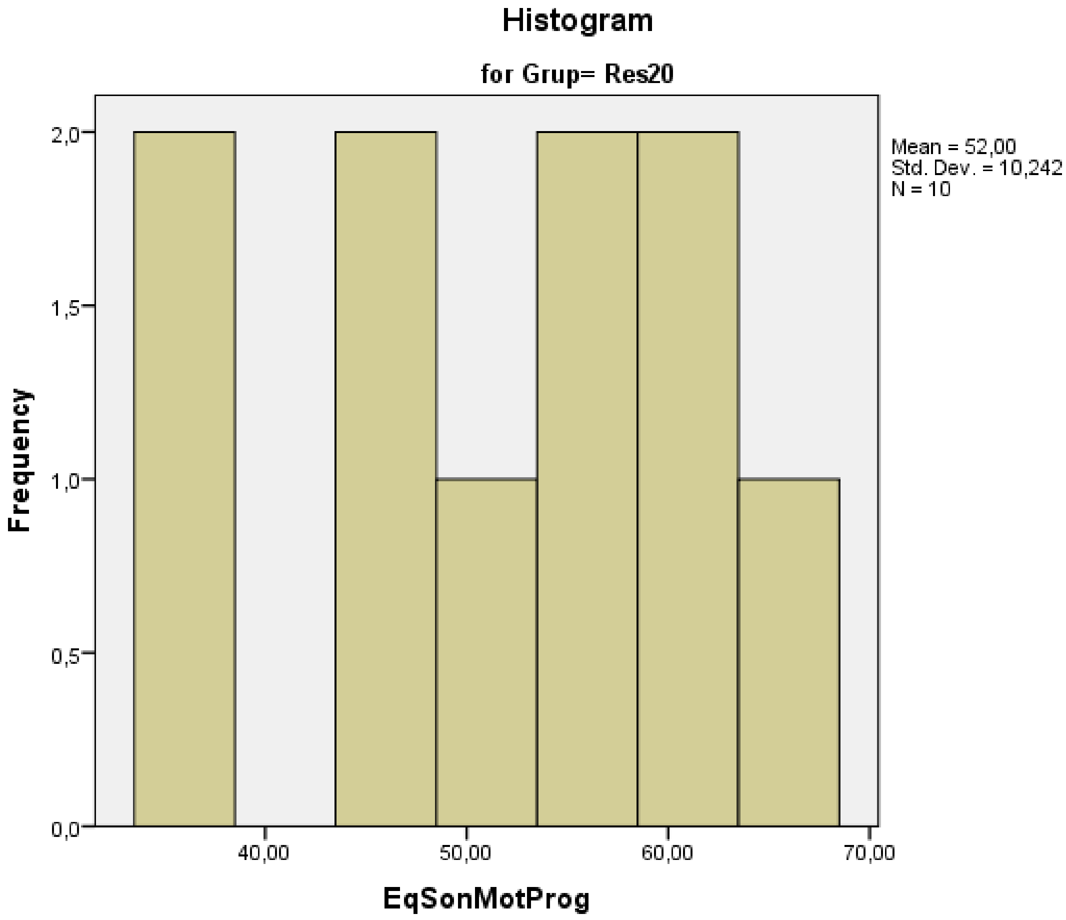
Histogram
for Grup= Res20
Mean = 52,00
Std. Dev. = 10,242
N = 10
Frequency
2,0
1,5
1,0
0,5
0,0
40,00
50,00
60,00
70,00
EqSonMotProg

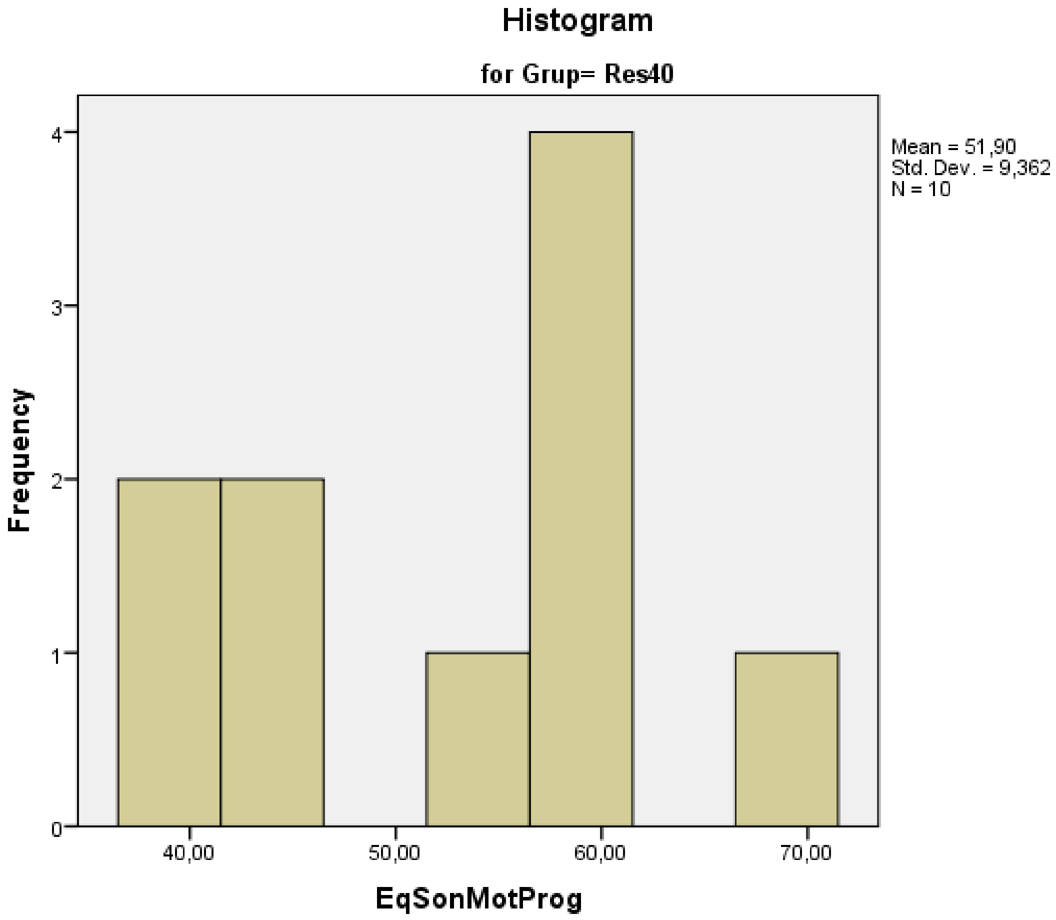
Histogram
for Grup= Res40
Mean = 51,90
Std. Dev. = 9,362
N = 10
Frequency
0
1
2
3
4
40,00
50,00
60,00
70,00
EqSonMotProg

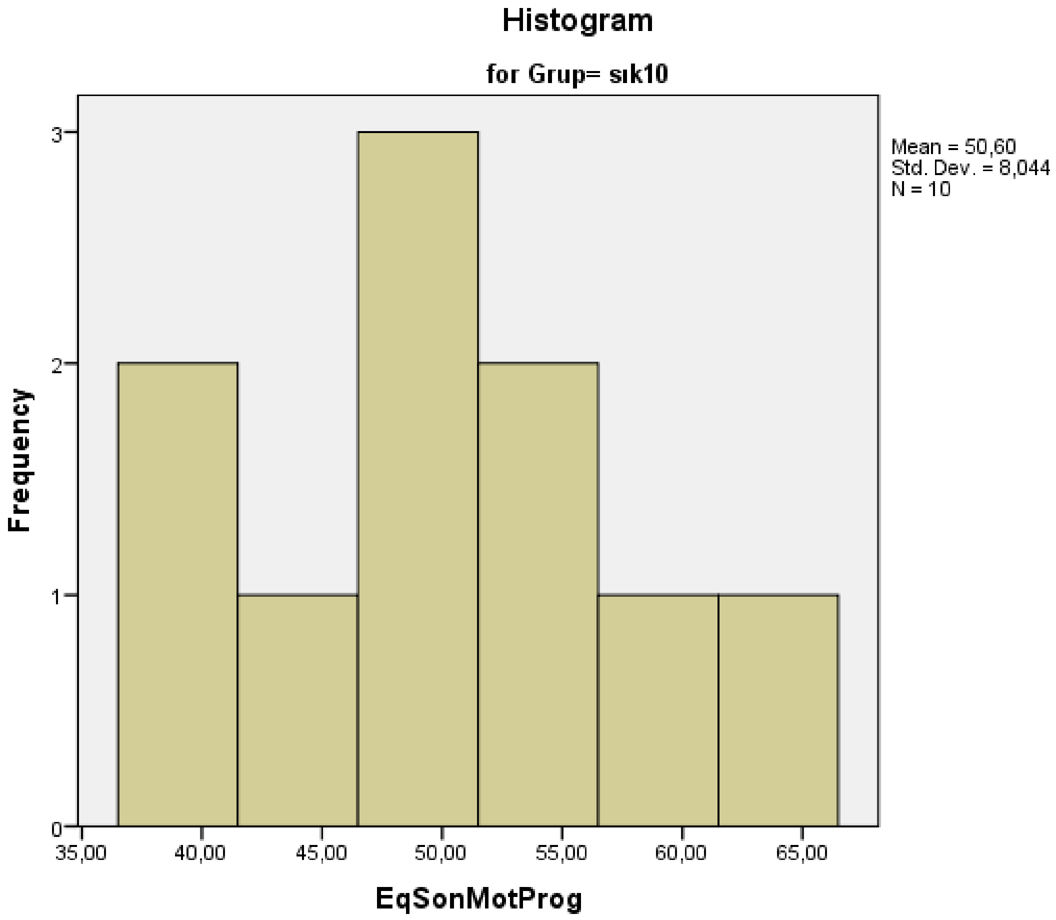
Histogram
for Grup= sık10
Mean = 50,60
Std. Dev. = 8,044
N = 10
Frequency
0
1
2
3
35,00
40,00
45,00
50,00
55,00
60,00
65,00
EqSonMotProg

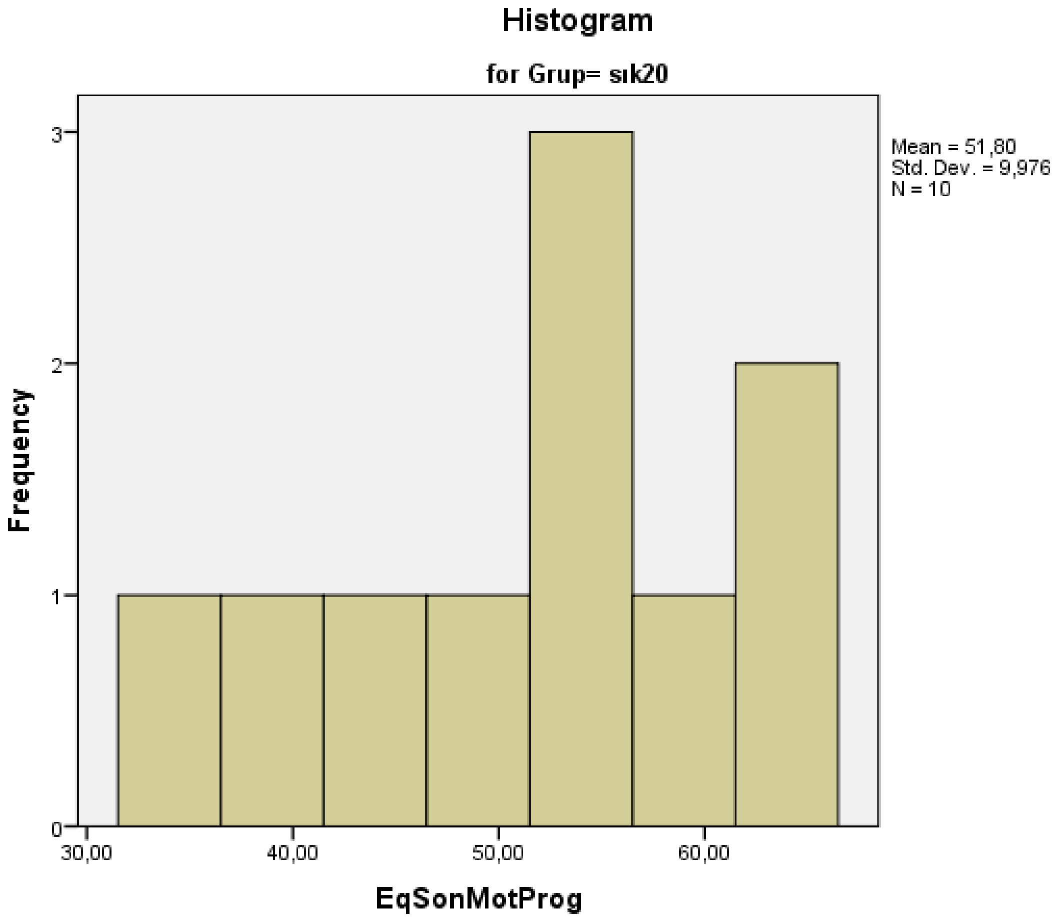
Histogram
for Grup= sık20
Mean = 51,80
Std. Dev. = 9,976
N = 10
Frequency
0
1
2
3
30,00
40,00
50,00
60,00
EqSonMotProg

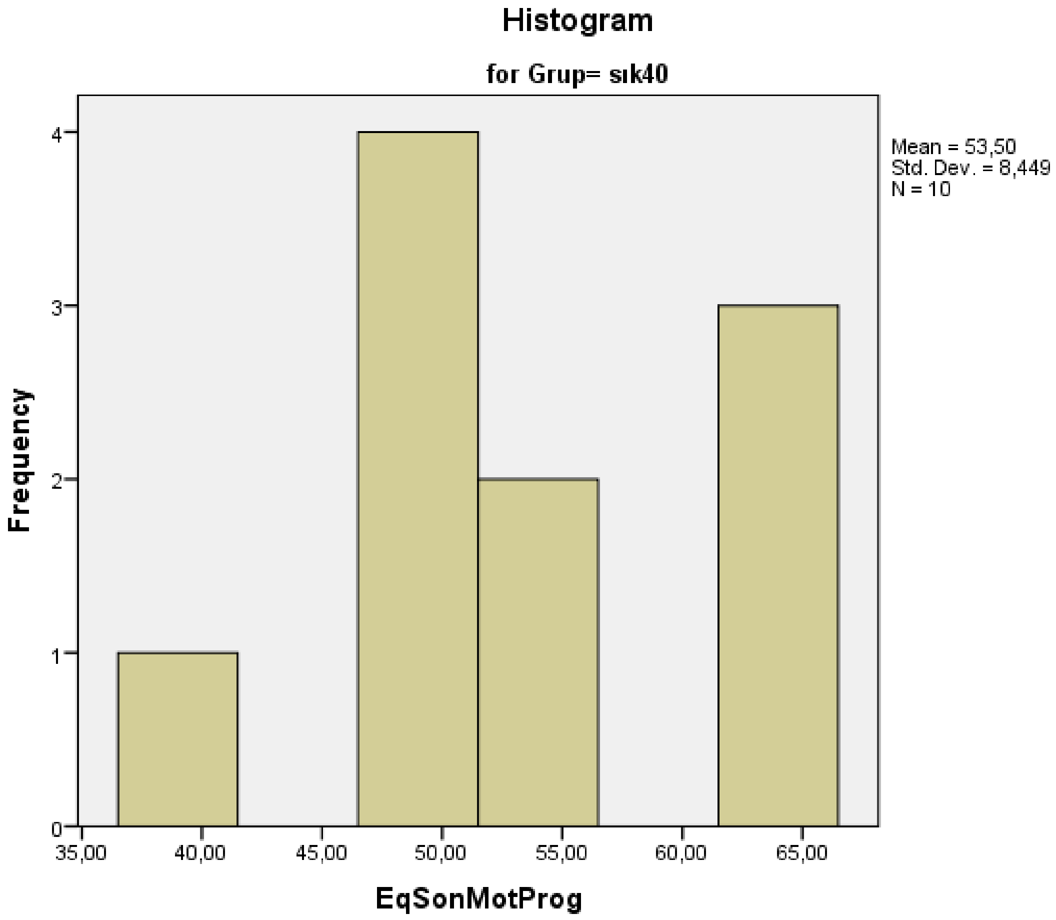
Histogram
for Grup= sık40
Mean = 53,50
Std. Dev. = 8,449
N = 10
Frequency
0
1
2
3
4
35,00
40,00
45,00
50,00
55,00
60,00
65,00
EqSonMotProg

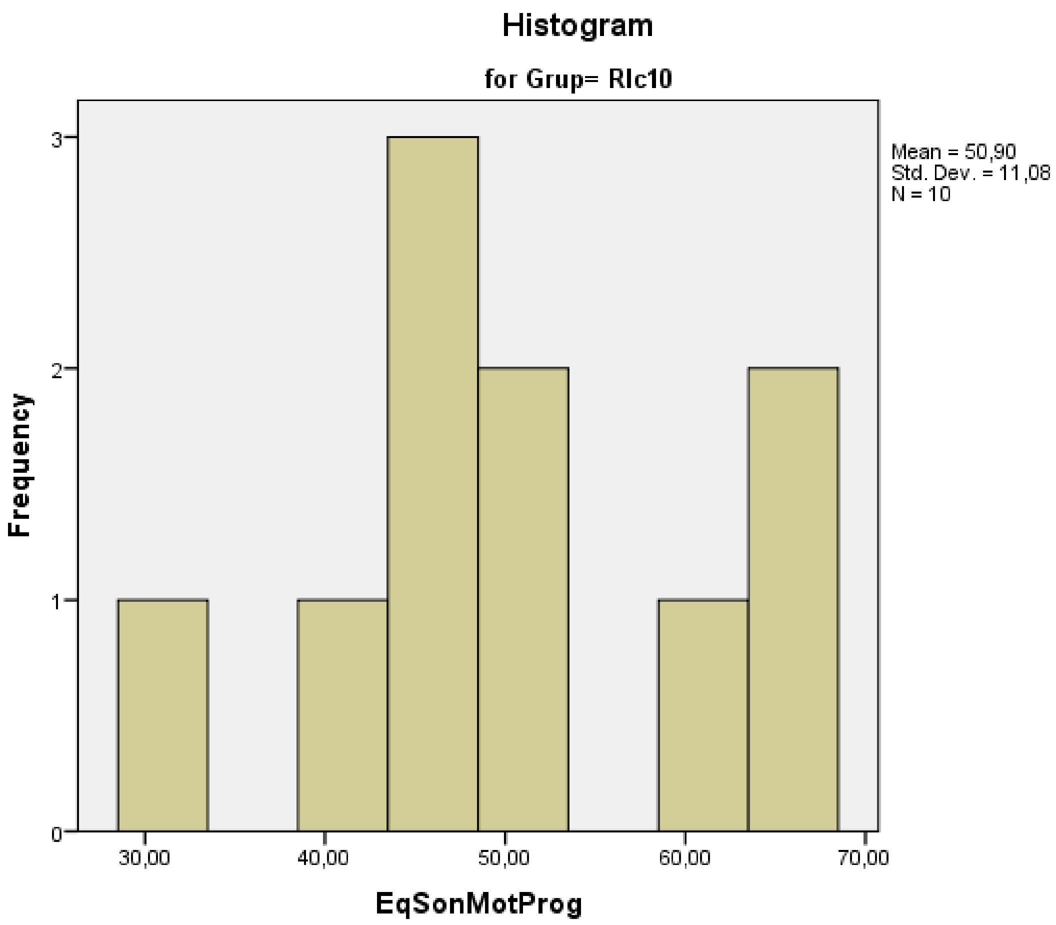
Histogram
for Grup= Rlc10
Mean = 50,90
Std. Dev. = 11,08
N = 10
Frequency
0
1
2
3
30,00
40,00
50,00
60,00
70,00
EqSonMotProg

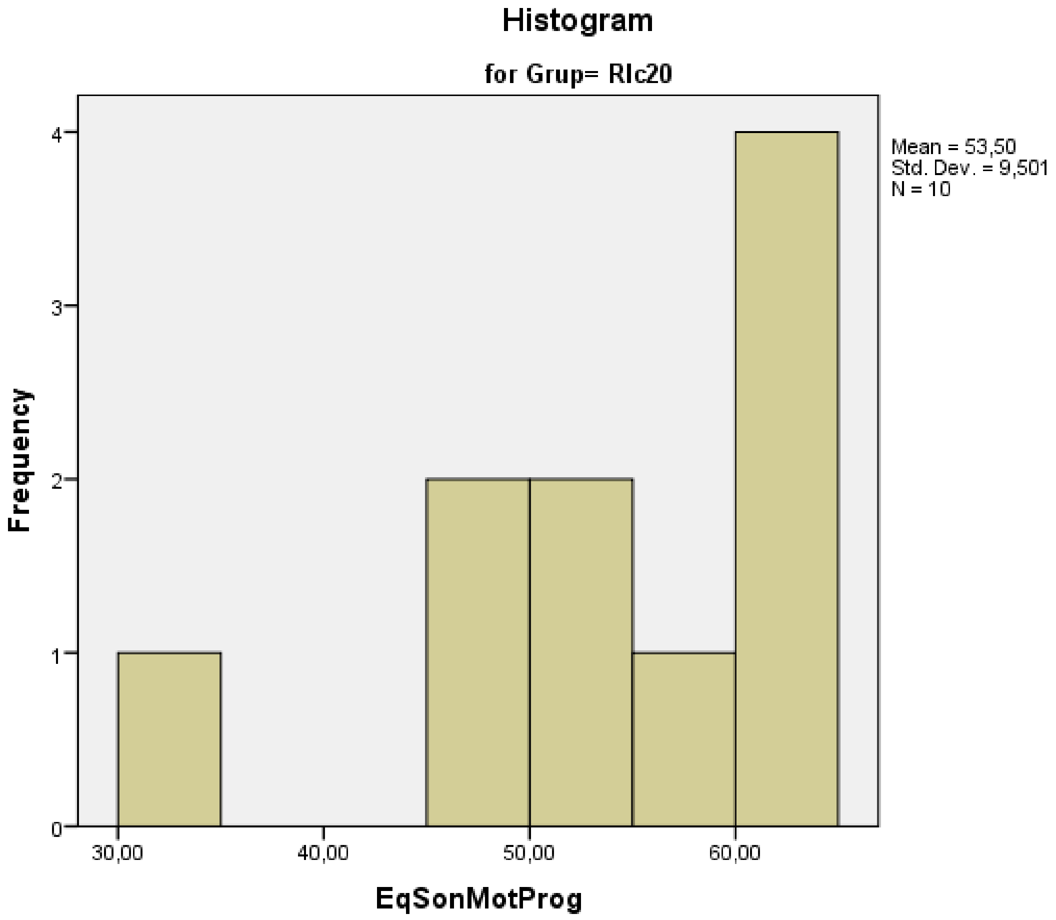
Histogram
for Grup= Rlc20
Mean = 53,50
Std. Dev. = 9,501
N = 10
Frequency
0
1
2
3
4
30,00
40,00
50,00
60,00
EqSonMotProg

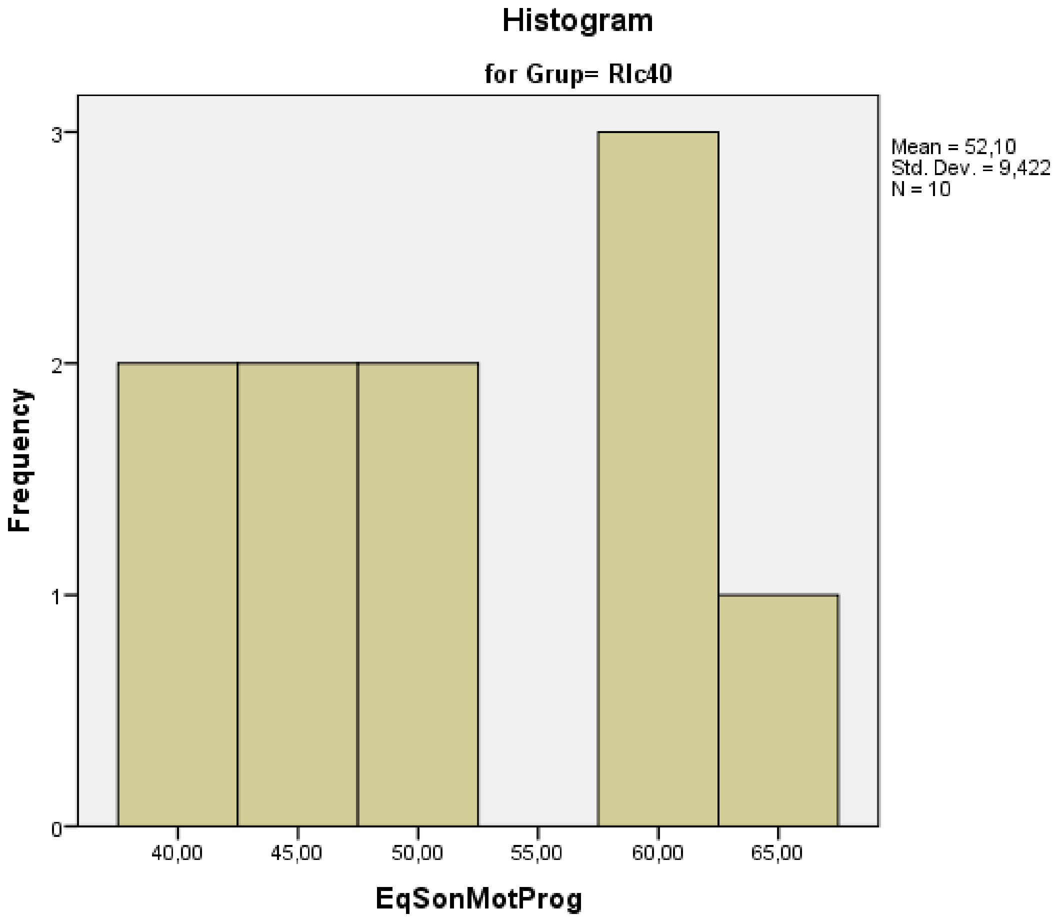

Stem-and-Leaf Plots

```
EqSonMotProg Stem-and-Leaf Plot for
Grup= kontrol

 Frequency    Stem &  Leaf

      ,00        3 .
     1,00        3 .  5
     2,00        4 .  44
     1,00        4 .  8
     4,00        5 .  3444
     1,00        5 .  5
     1,00 Extremes    (>=71)

 Stem width:     10,00
 Each leaf:       1 case(s)
```

```
EqSonMotProg Stem-and-Leaf Plot for
Grup= Res10

 Frequency    Stem &  Leaf

     2,00        4 .  01
     1,00        4 .  8
     2,00        5 .  04
     2,00        5 .  69
     1,00        6 .  1
     2,00        6 .  58

 Stem width:     10,00
 Each leaf:       1 case(s)
```

```
EqSonMotProg Stem-and-Leaf Plot for
Grup= Res20

 Frequency    Stem &  Leaf

     2,00        3 .  67
     3,00        4 .  789
     2,00        5 .  88
     3,00        6 .  016

 Stem width:     10,00
 Each leaf:       1 case(s)
```

```
EqSonMotProg Stem-and-Leaf Plot for
Grup= Res40

 Frequency    Stem &  Leaf

     2,00        3 .  99
     2,00        4 .  46
     5,00        5 .  47788
     1,00        6 .  7

 Stem width:     10,00
 Each leaf:       1 case(s)
```

```
EqSonMotProg Stem-and-Leaf Plot for
Grup= sık10
```

```
 Frequency    Stem &  Leaf

     1,00        3 .  9
     3,00        4 .  167
     4,00        5 .  0035
     2,00        6 .  05

 Stem width:     10,00
 Each leaf:       1 case(s)
```

```
EqSonMotProg Stem-and-Leaf Plot for
Grup= sık20

 Frequency    Stem &  Leaf

     1,00        3 .  4
     2,00        4 .  13
     5,00        5 .  03469
     2,00        6 .  26

 Stem width:     10,00
 Each leaf:       1 case(s)
```

```
EqSonMotProg Stem-and-Leaf Plot for
Grup= sık40

 Frequency    Stem &  Leaf

     1,00        3 .  9
     2,00        4 .  79
     4,00        5 .  0026
     3,00        6 .  336

 Stem width:     10,00
 Each leaf:       1 case(s)
```

```
EqSonMotProg Stem-and-Leaf Plot for
Grup= Rlc10

 Frequency    Stem &  Leaf

     1,00        3 .  1
     4,00        4 .  3458
     2,00        5 .  33
     3,00        6 .  048

 Stem width:     10,00
```

```
 Each leaf:       1 case(s)




EqSonMotProg Stem-and-Leaf Plot for
Grup= Rlc20

 Frequency    Stem &  Leaf

     1,00 Extremes    (=<31)
     2,00        4 .  79
     2,00        5 .  44
     1,00        5 .  6
     4,00        6 .  0004

 Stem width:     10,00
 Each leaf:       1 case(s)




EqSonMotProg Stem-and-Leaf Plot for
Grup= Rlc40

 Frequency    Stem &  Leaf

     2,00        4 .  00
     3,00        4 .  678
     1,00        5 .  2
      ,00        5 .
     3,00        6 .  001
     1,00        6 .  7

 Stem width:     10,00
 Each leaf:       1 case(s)
```

Normal Q-Q Plots

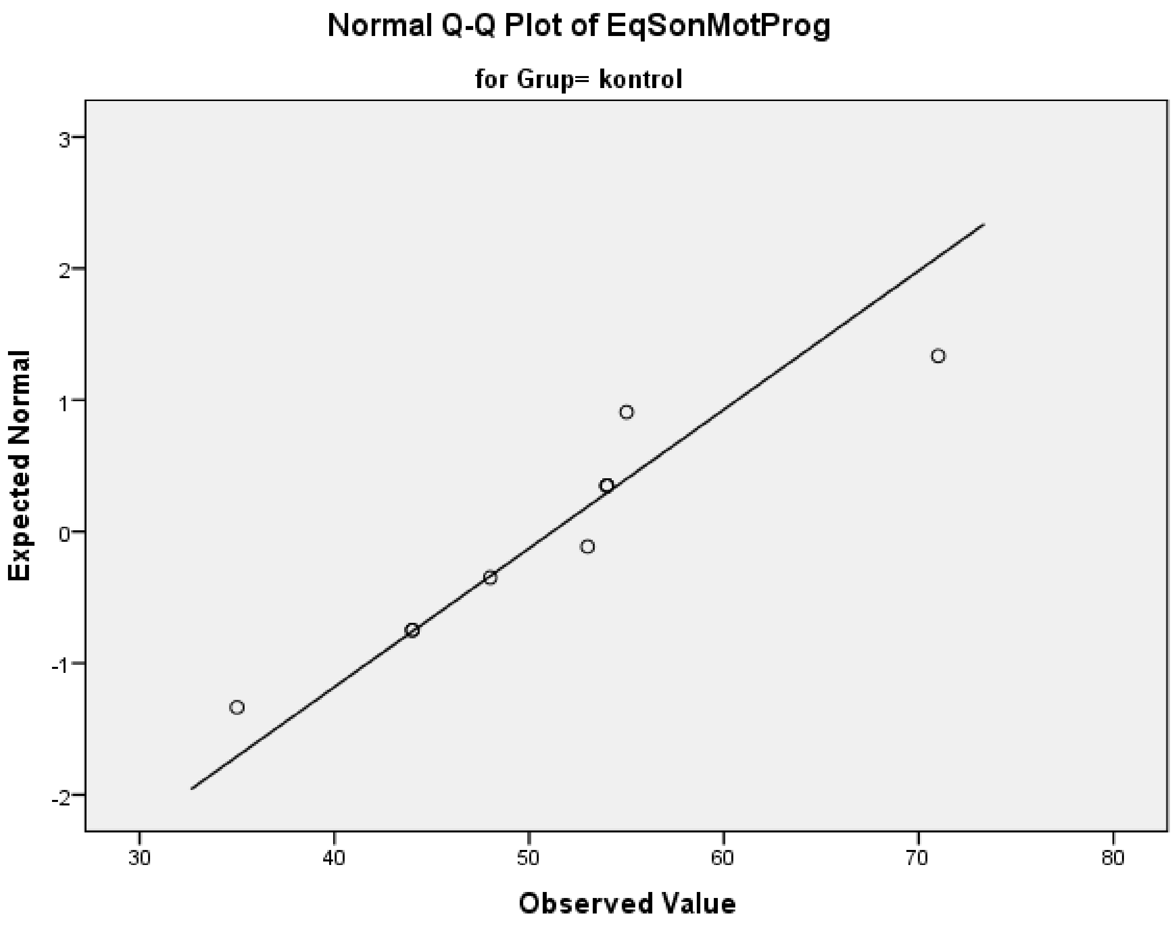
Normal Q-Q Plot of EqSonMotProg
for Grup= kontrol
Expected Normal
3
2
1
0
-1
-2
30
40
50
60
70
80
Observed Value

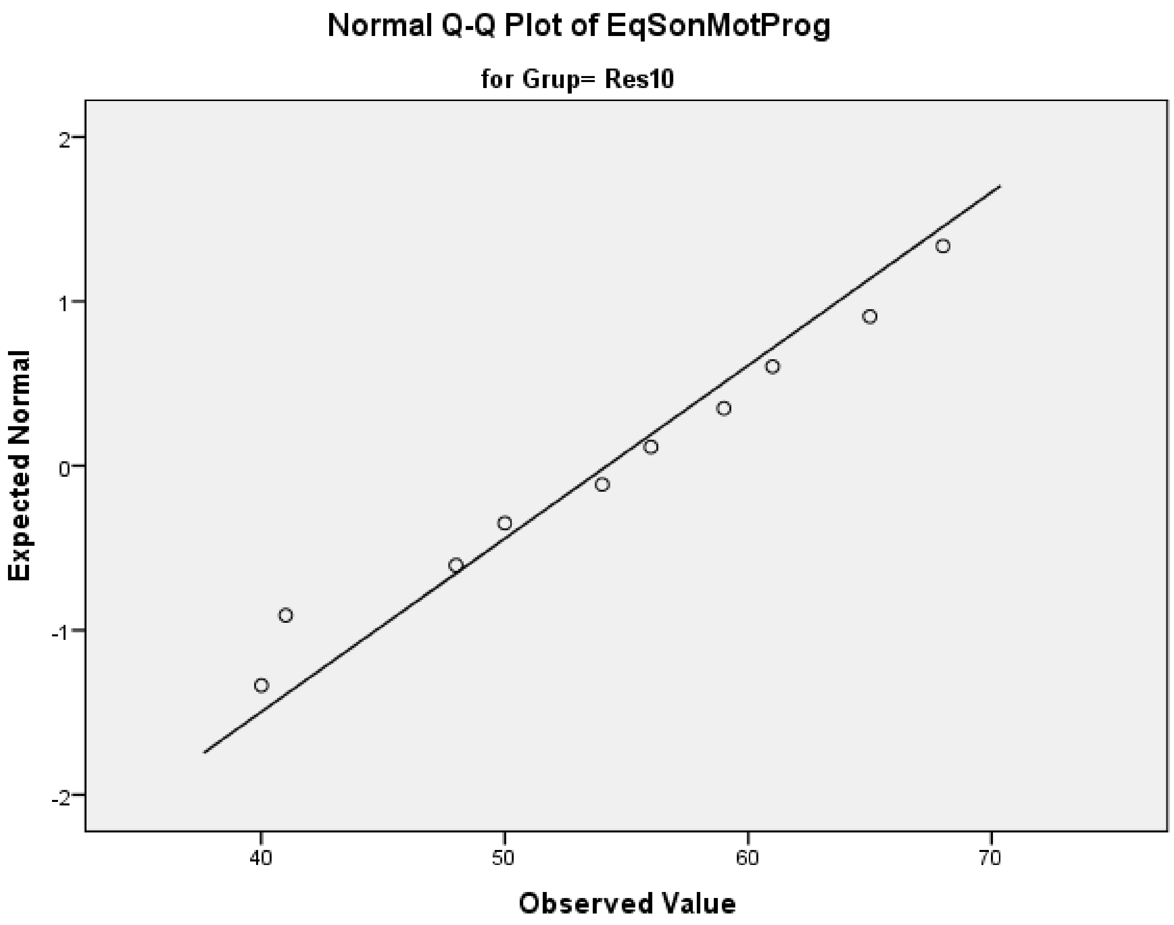

Normal Q-Q Plot of EqSonMotProg
for Grup= Res10
Expected Normal
2
1
0
-1
-2
40
50
60
70
Observed Value

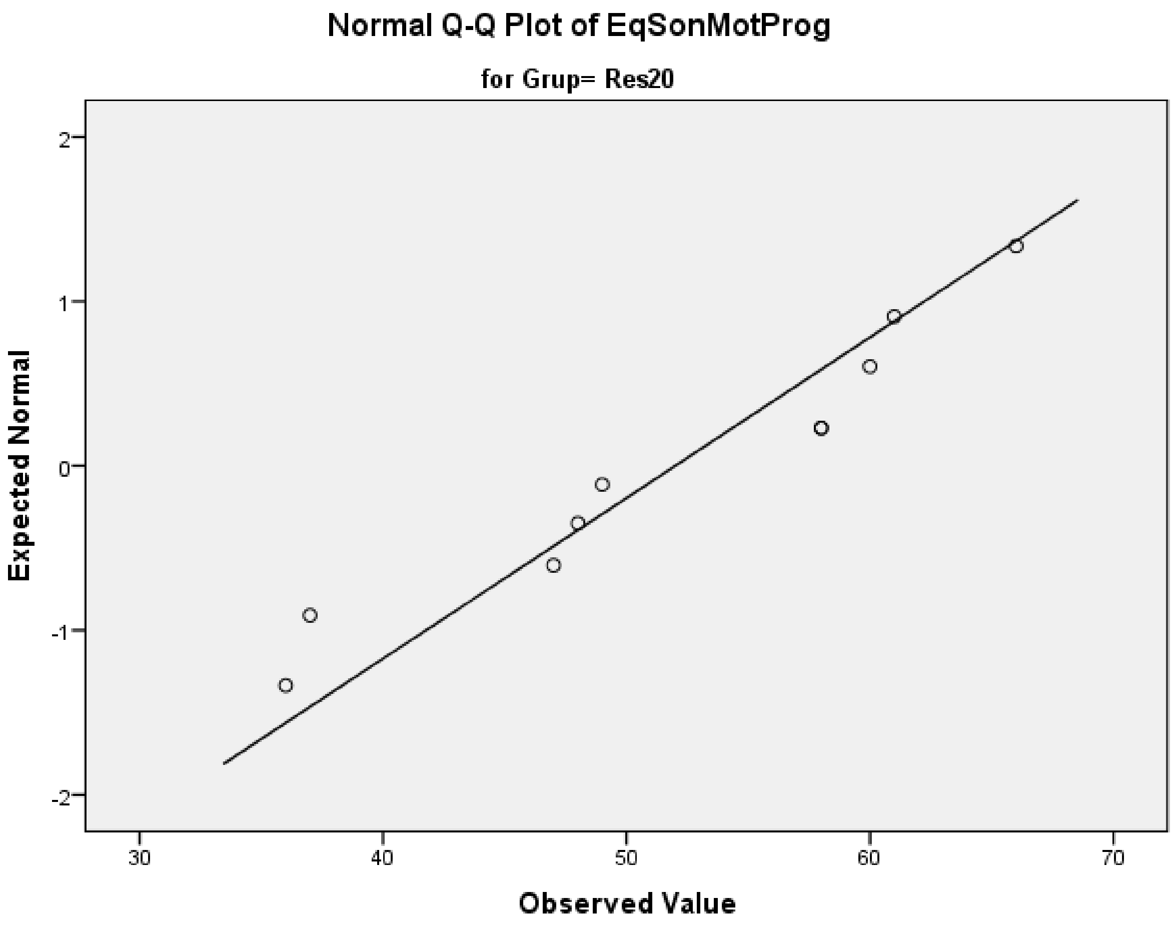
Normal Q-Q Plot of EqSonMotProg
for Grup= Res20
Expected Normal
2
1
0
-1
-2
30
40
50
60
70
Observed Value

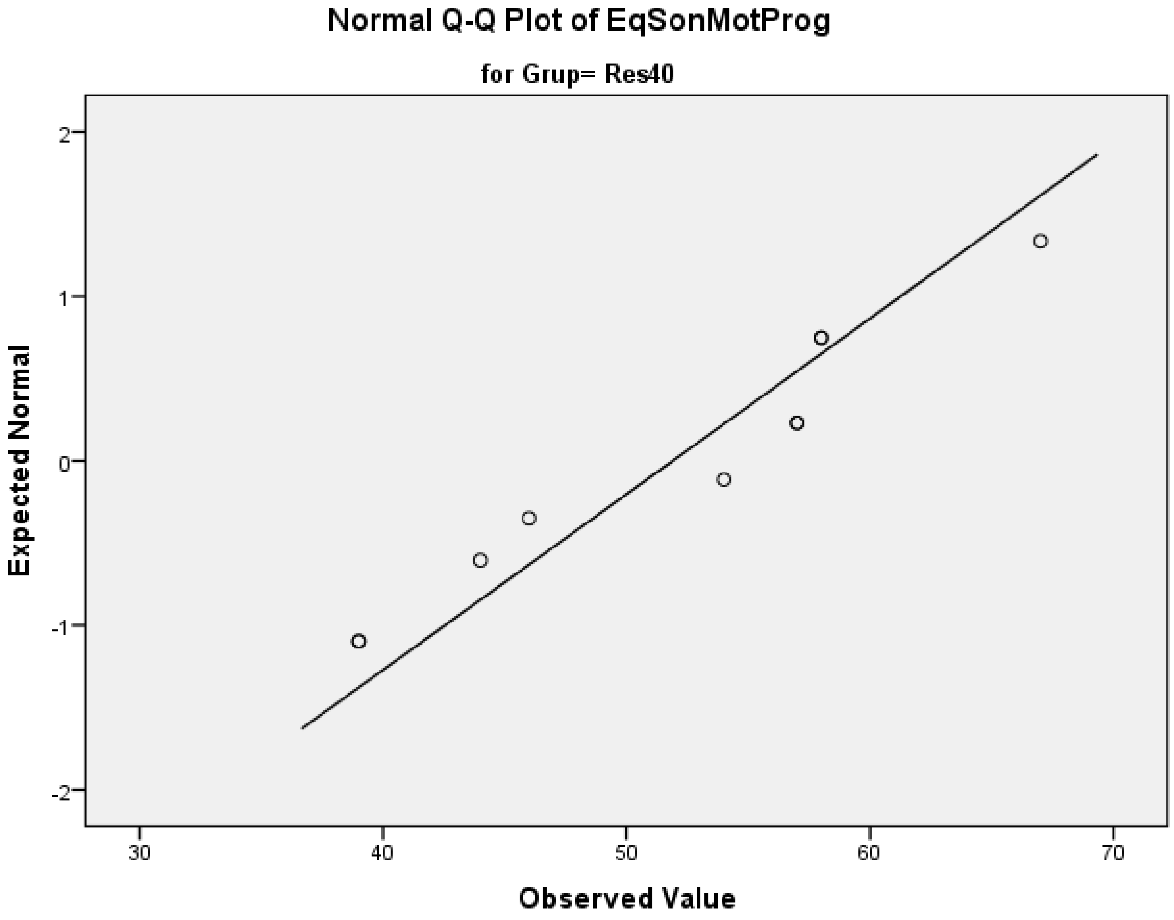
Normal Q-Q Plot of EqSonMotProg
for Grup= Res40
Expected Normal
Observed Value
2
1
0
-1
-2
30
40
50
60
70

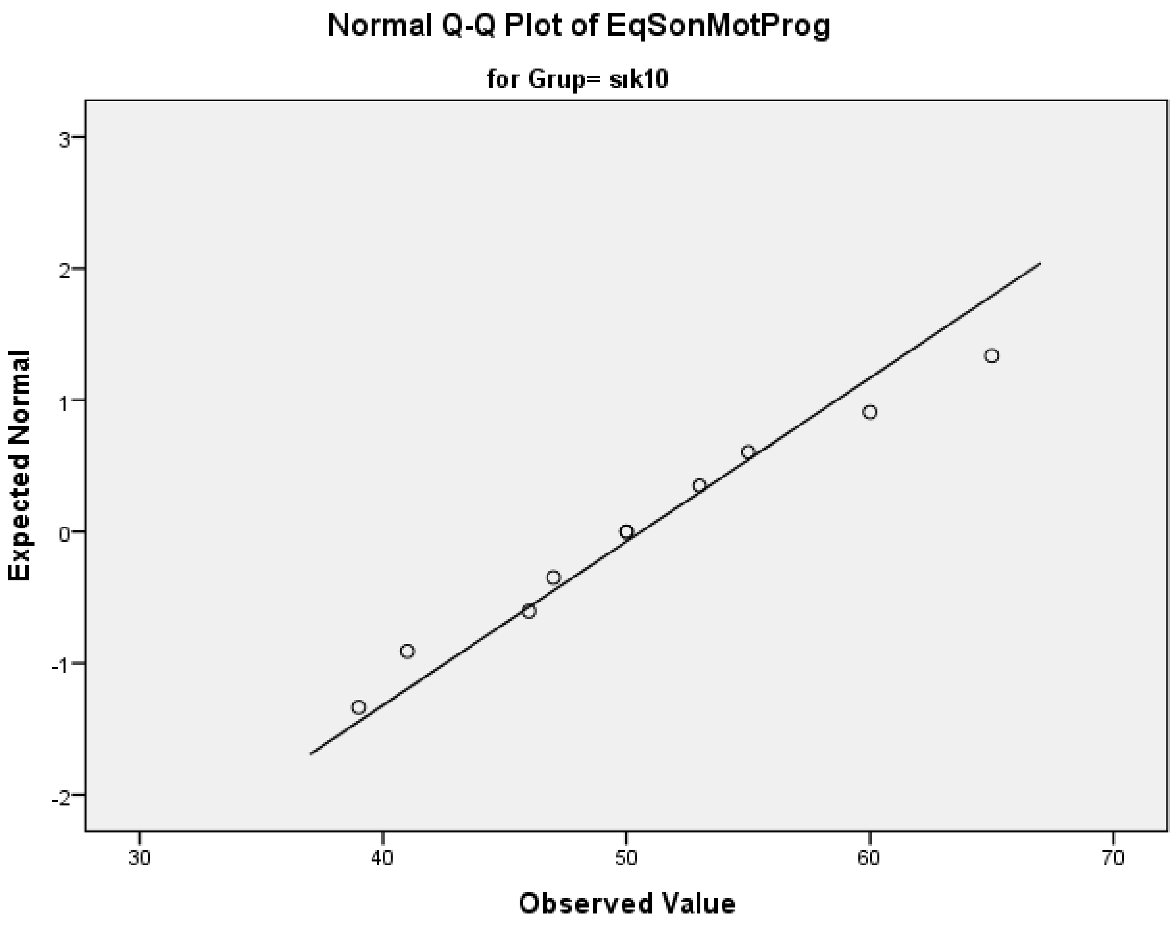

Normal Q-Q Plot of EqSonMotProg
for Grup= sık10
Expected Normal
Observed Value
3
2
1
0
-1
-2
30
40
50
60
70

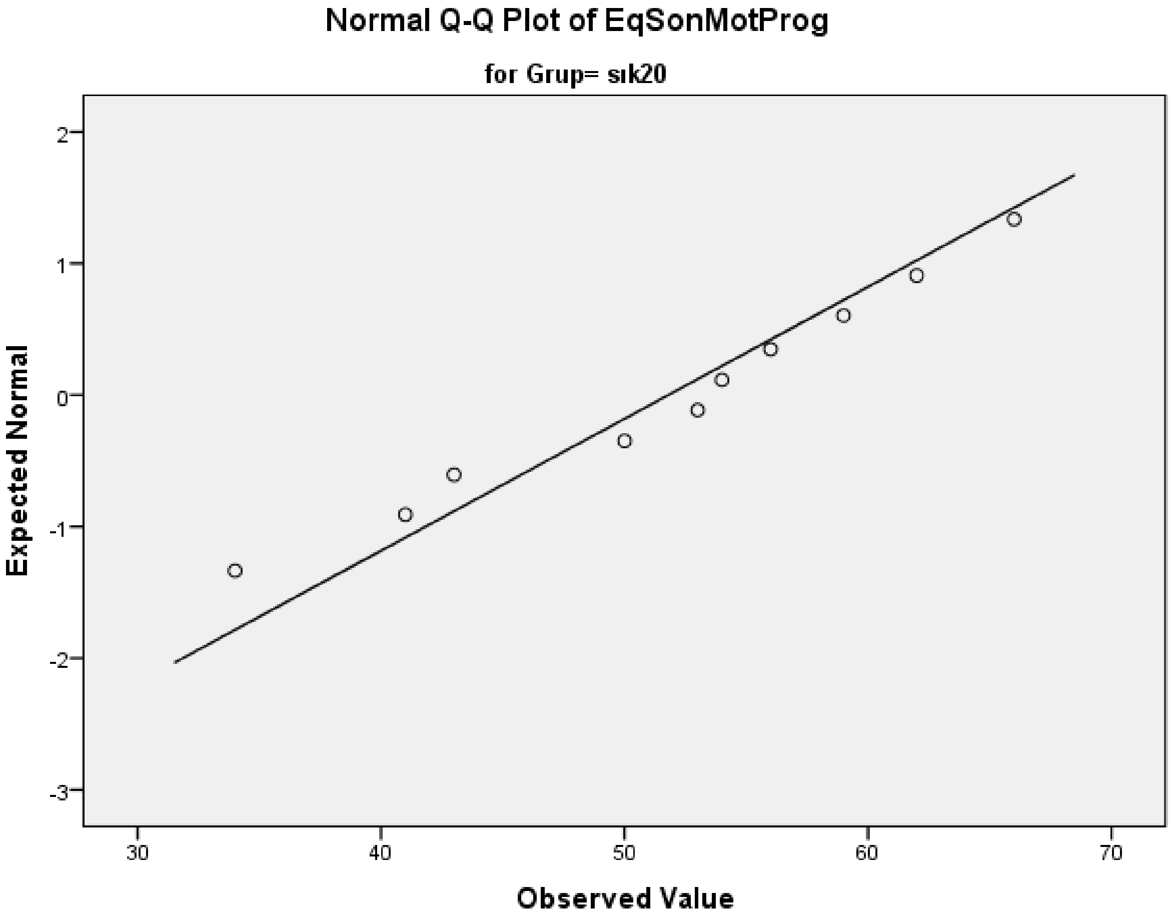

Normal Q-Q Plot of EqSonMotProg
for Grup= sık20
Expected Normal
Observed Value
2
1
0
-1
-2
-3
30
40
50
60
70

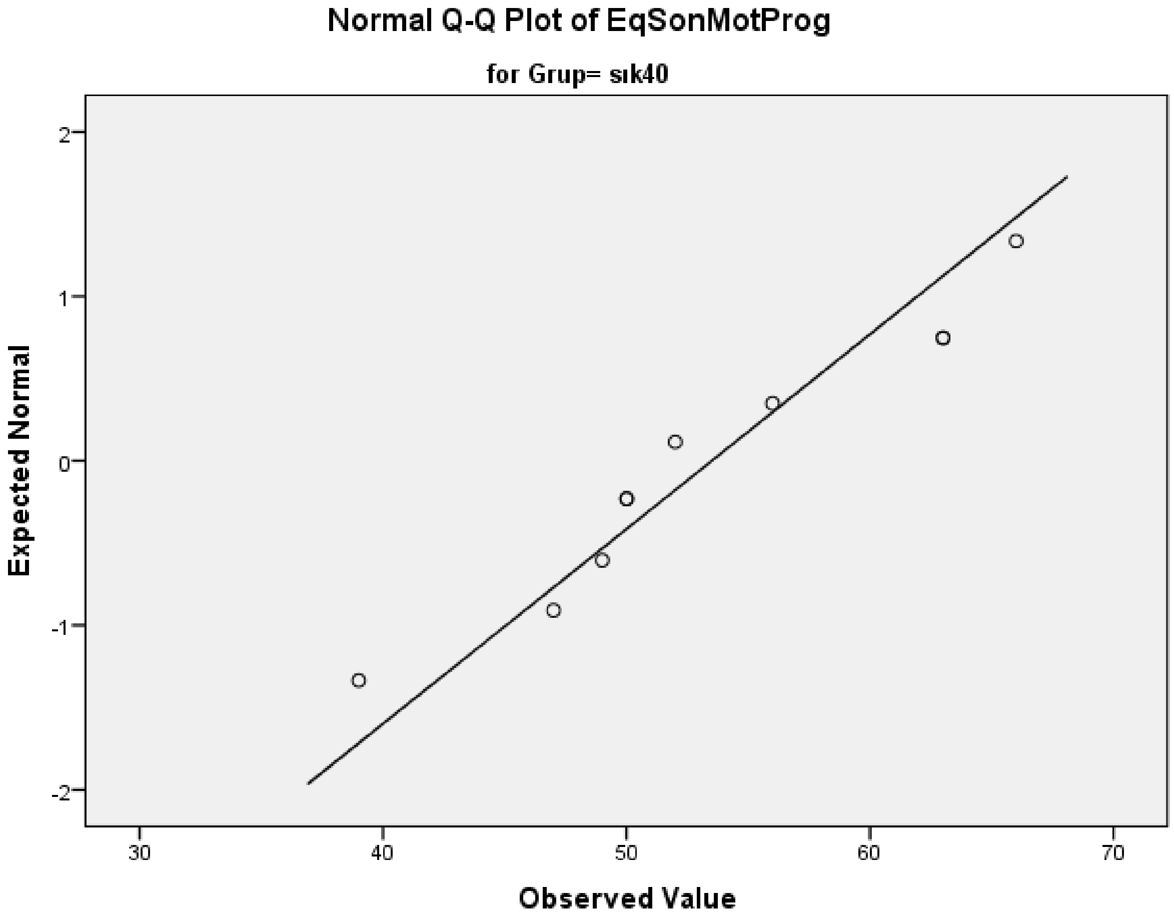
Normal Q-Q Plot of EqSonMotProg
for Grup= sık40
Expected Normal
Observed Value
2
1
0
-1
-2
30
40
50
60
70

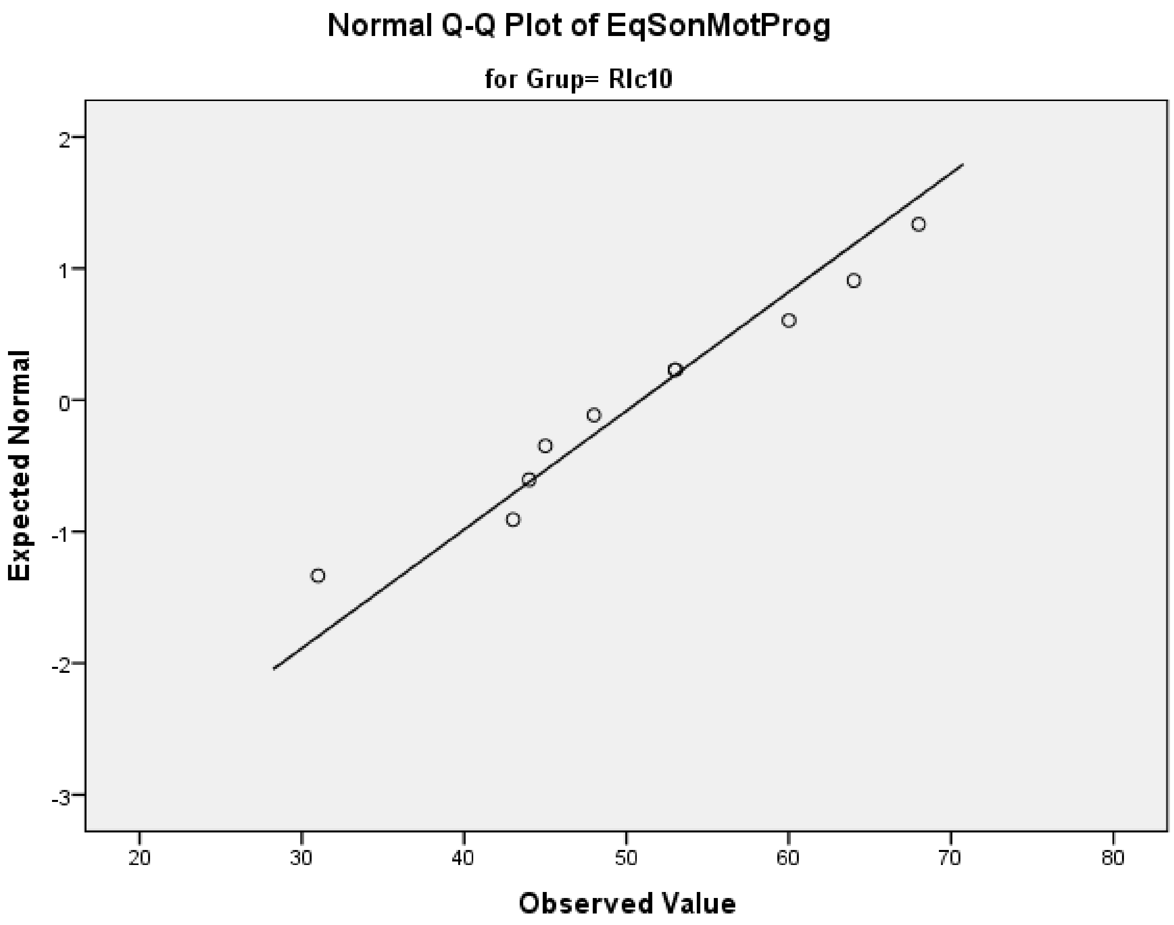
Normal Q-Q Plot of EqSonMotProg
for Grup= Rlc10
Expected Normal
Observed Value
2
1
0
-1
-2
-3
20
30
40
50
60
70
80

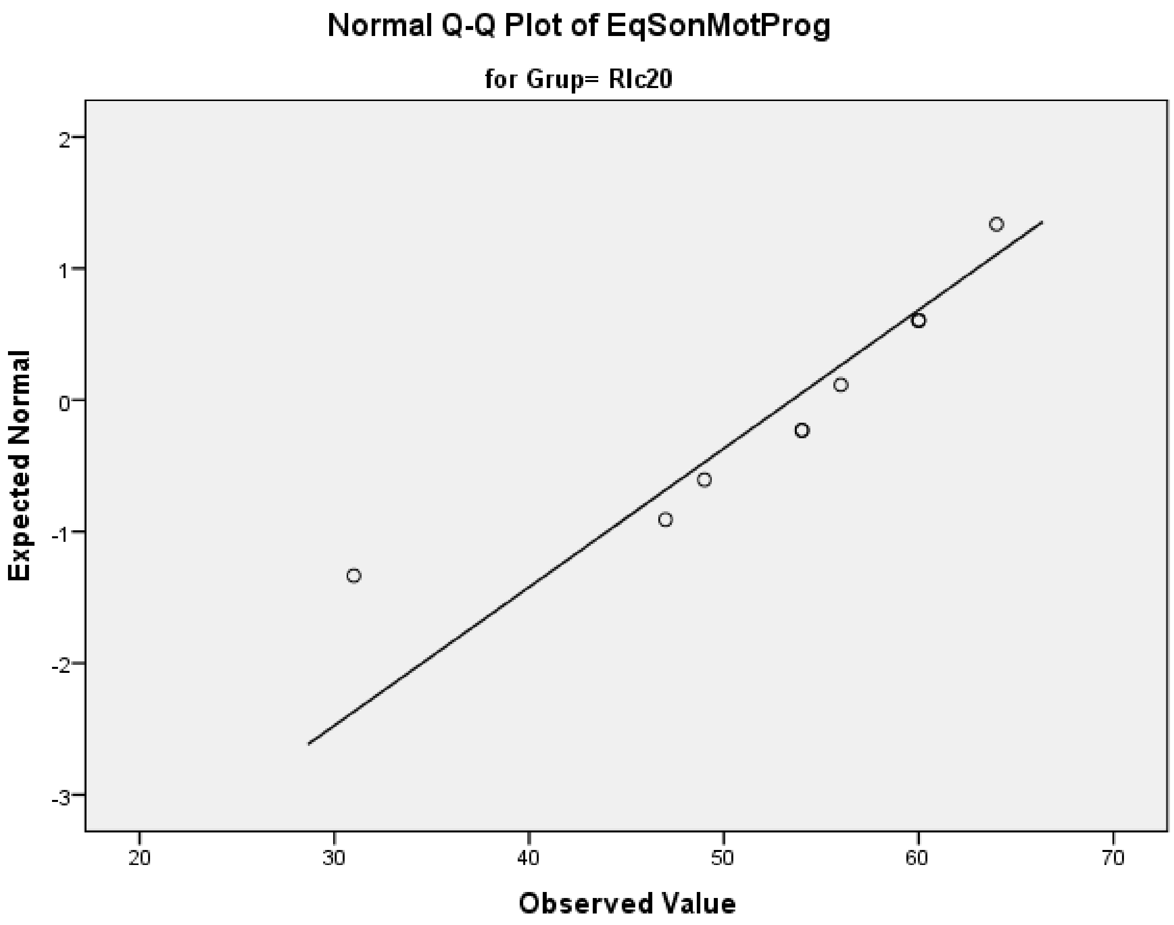
Normal Q-Q Plot of EqSonMotProg
for Grup= Rlc20
Expected Normal
2
1
0
-1
-2
-3
20
30
40
50
60
70
Observed Value

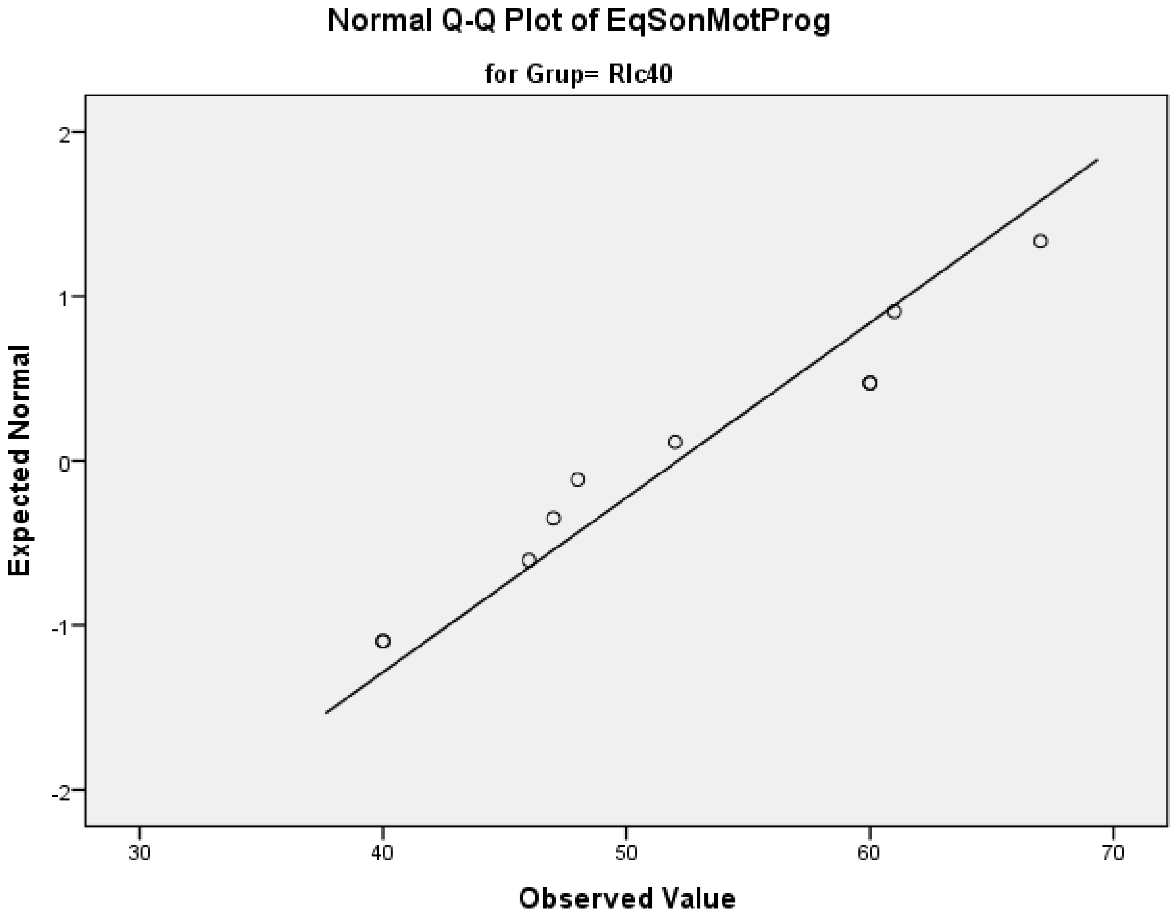

Detrended Normal Q-Q Plots

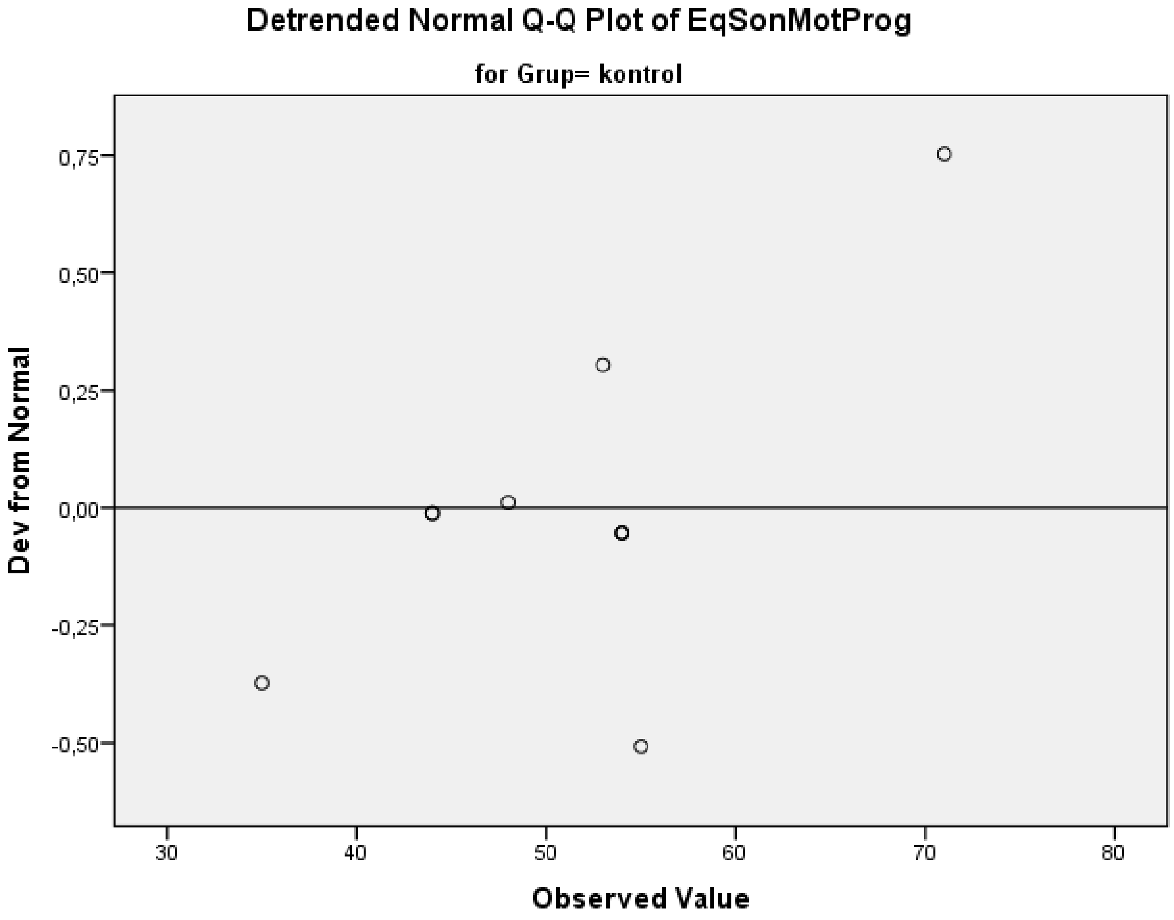
Detrended Normal Q-Q Plot of EqSonMotProg
for Grup= kontrol
Dev from Normal
0,75
0,50
0,25
0,00
-0,25
-0,50
30
40
50
60
70
80
Observed Value

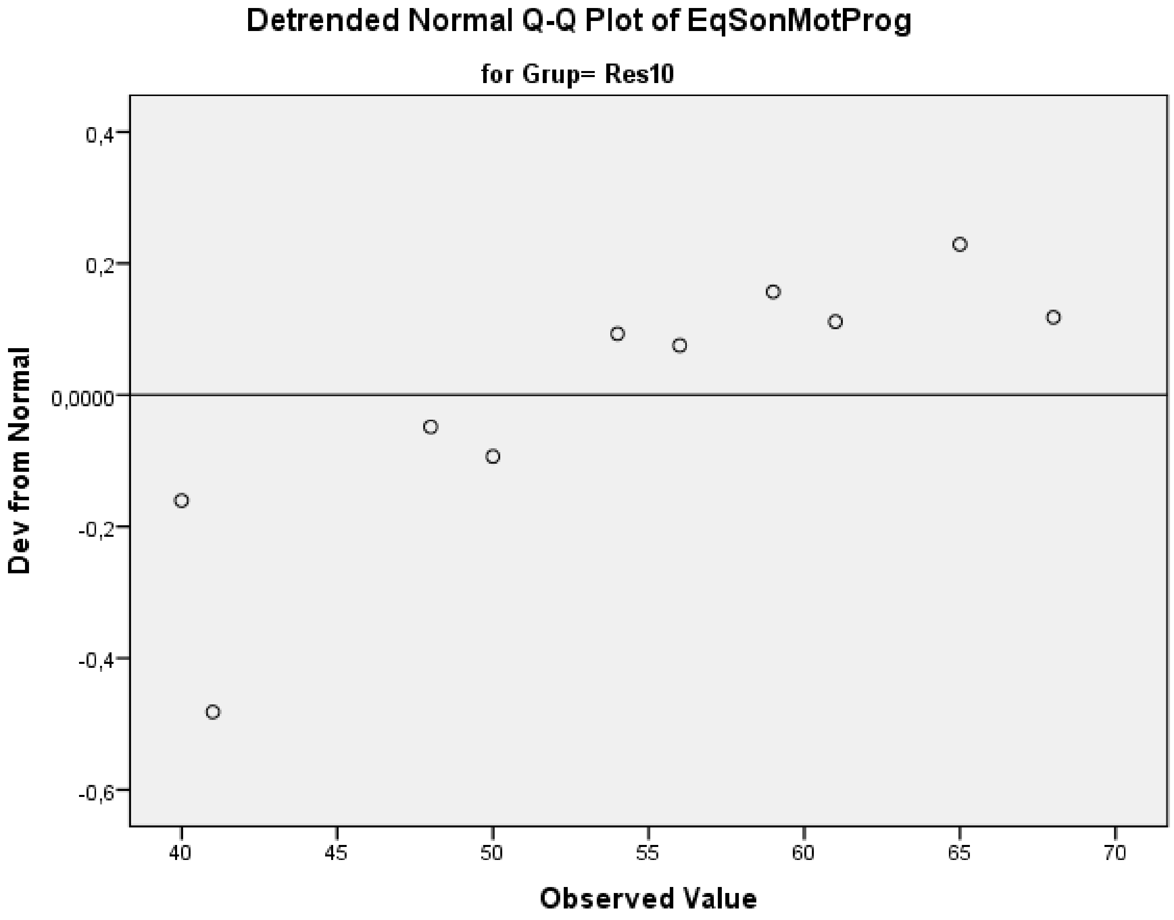
Detrended Normal Q-Q Plot of EqSonMotProg
for Grup= Res10
Dev from Normal
Observed Value
0,4
0,2
0,0000
-0,2
-0,4
-0,6
40
45
50
55
60
65
70

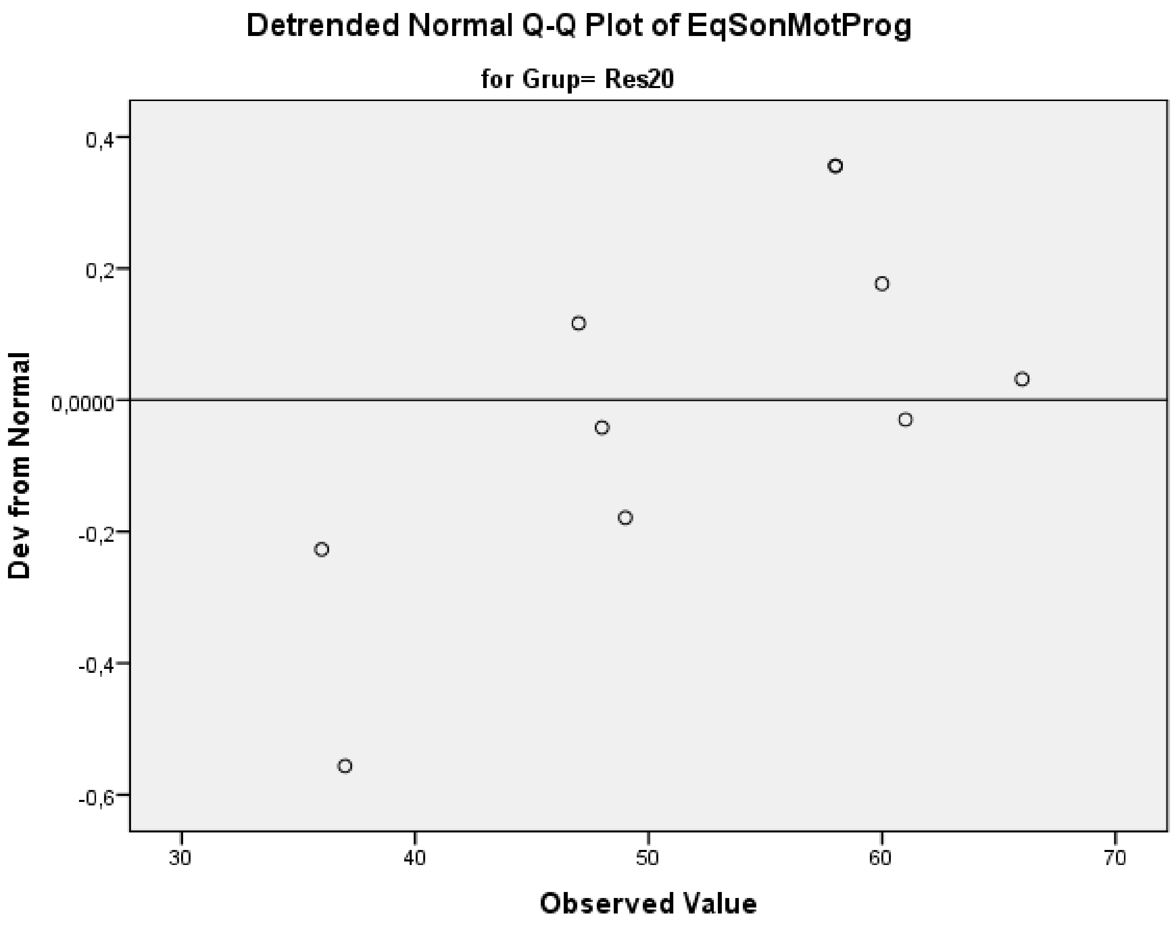
Detrended Normal Q-Q Plot of EqSonMotProg
for Grup= Res20
Dev from Normal
0,4
0,2
0,0000
-0,2
-0,4
-0,6
30
40
50
60
70
Observed Value

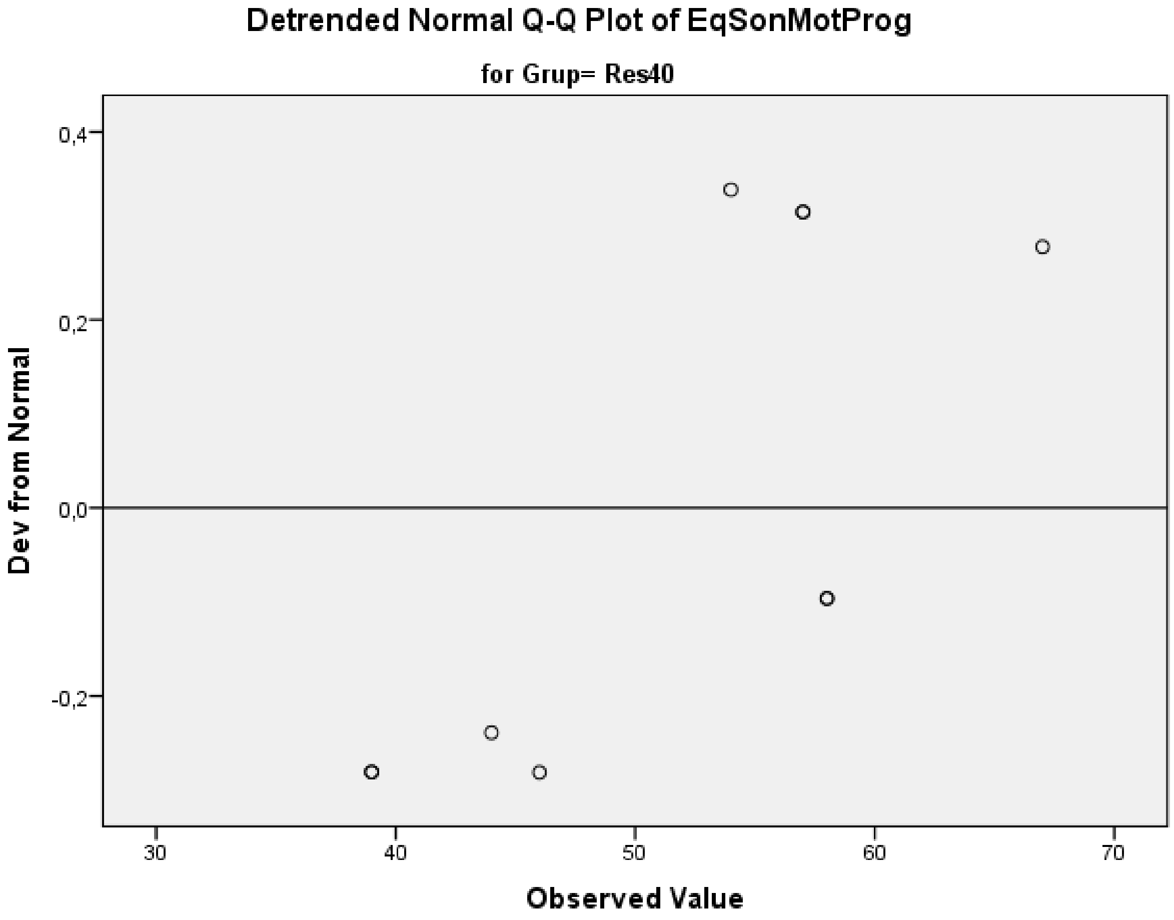

Detrended Normal Q-Q Plot of EqSonMotProg
for Grup= Res40
Dev from Normal
0,4
0,2
0,0
-0,2
30
40
50
60
70
Observed Value

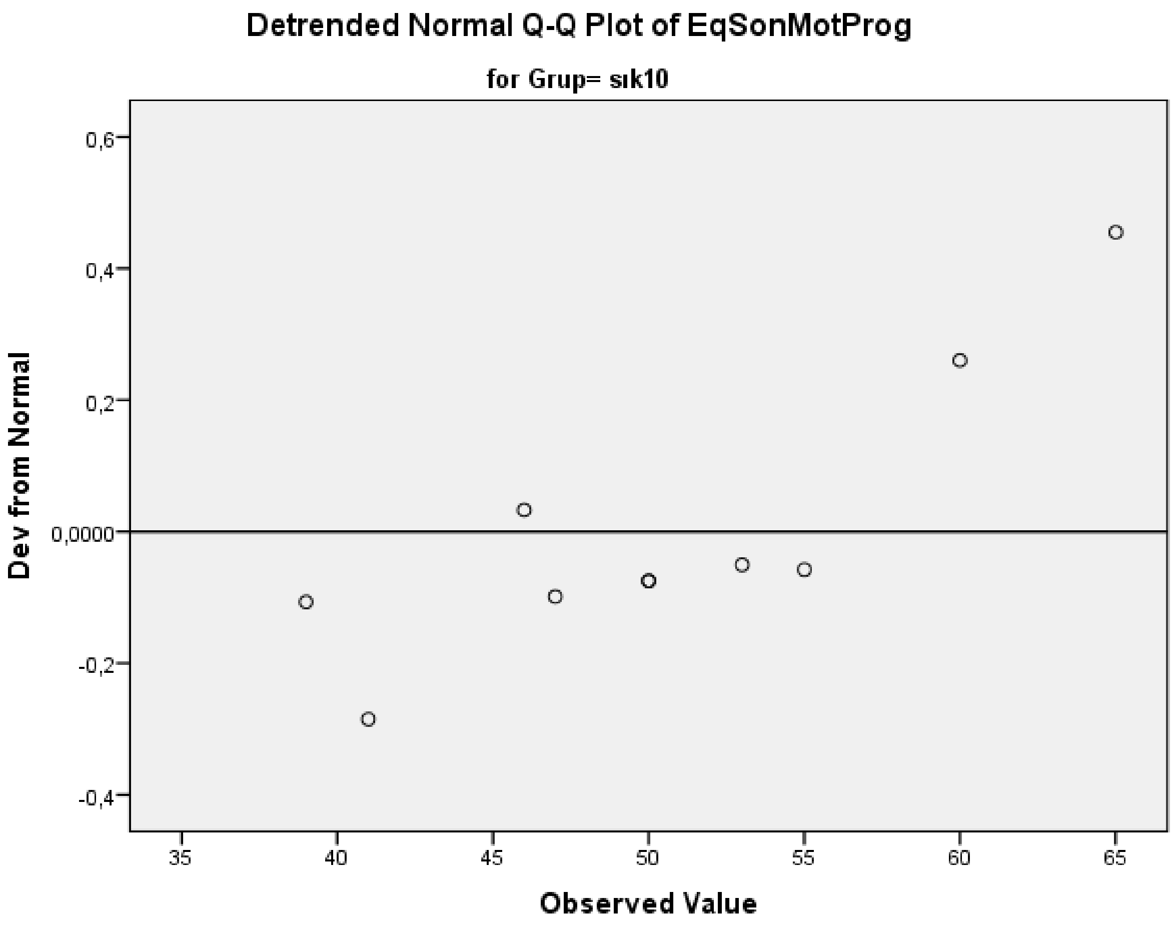

Detrended Normal Q-Q Plot of EqSonMotProg
for Grup= sık10
Dev from Normal
0,6
0,4
0,2
0,0000
-0,2
-0,4
35
40
45
50
55
60
65
Observed Value

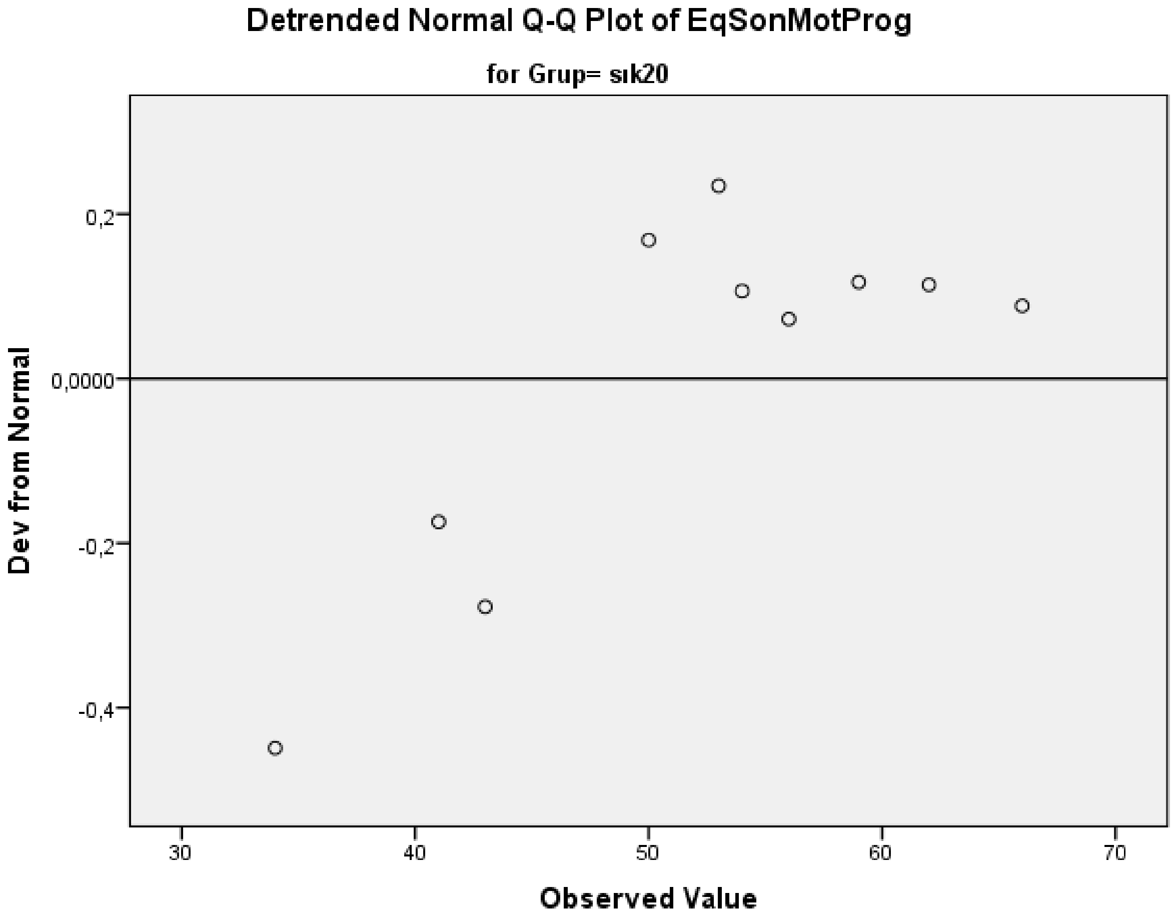
Detrended Normal Q-Q Plot of EqSonMotProg
for Grup= sık20
Dev from Normal
0,2
0,0000
-0,2
-0,4
30
40
50
60
70
Observed Value

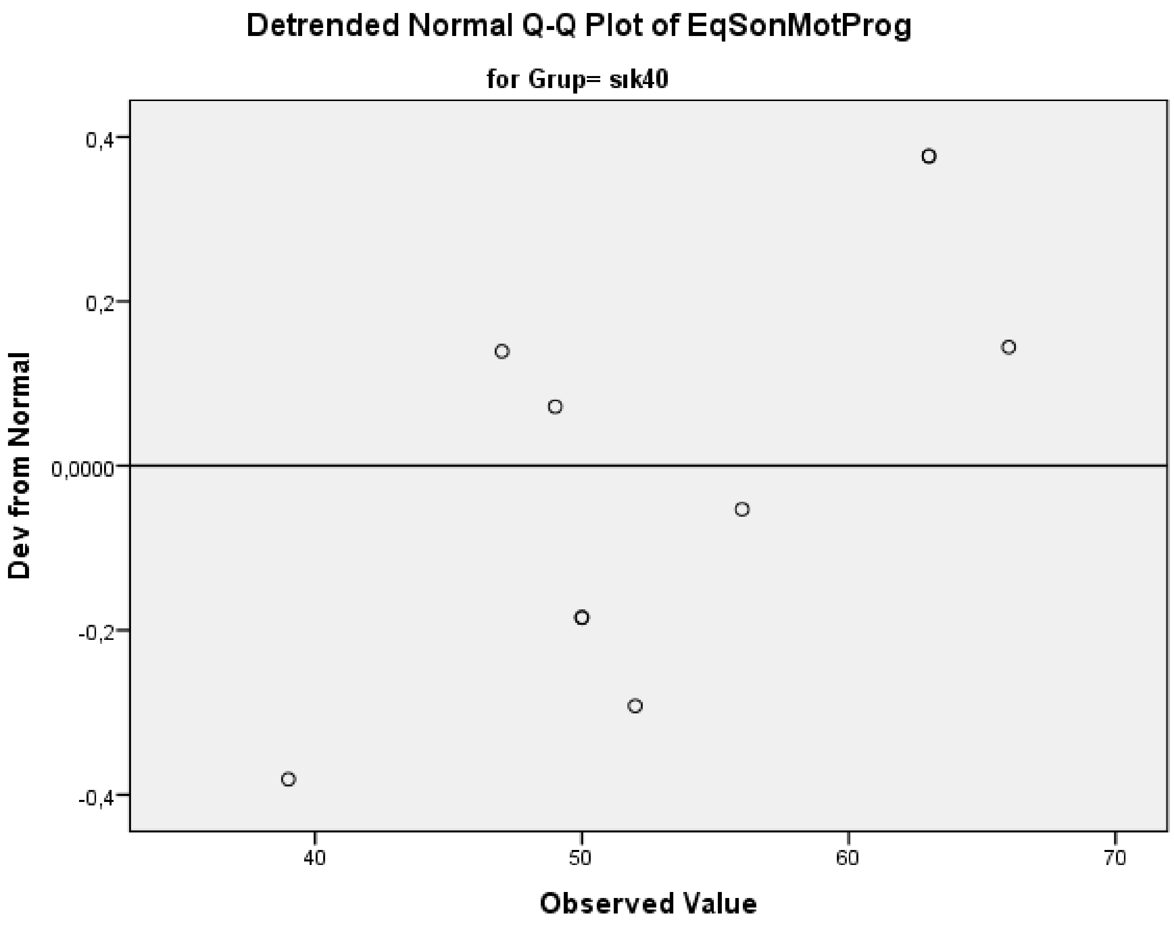
Detrended Normal Q-Q Plot of EqSonMotProg
for Grup= sık40
Dev from Normal
0,4
0,2
0,0000
-0,2
-0,4
40
50
60
70
Observed Value

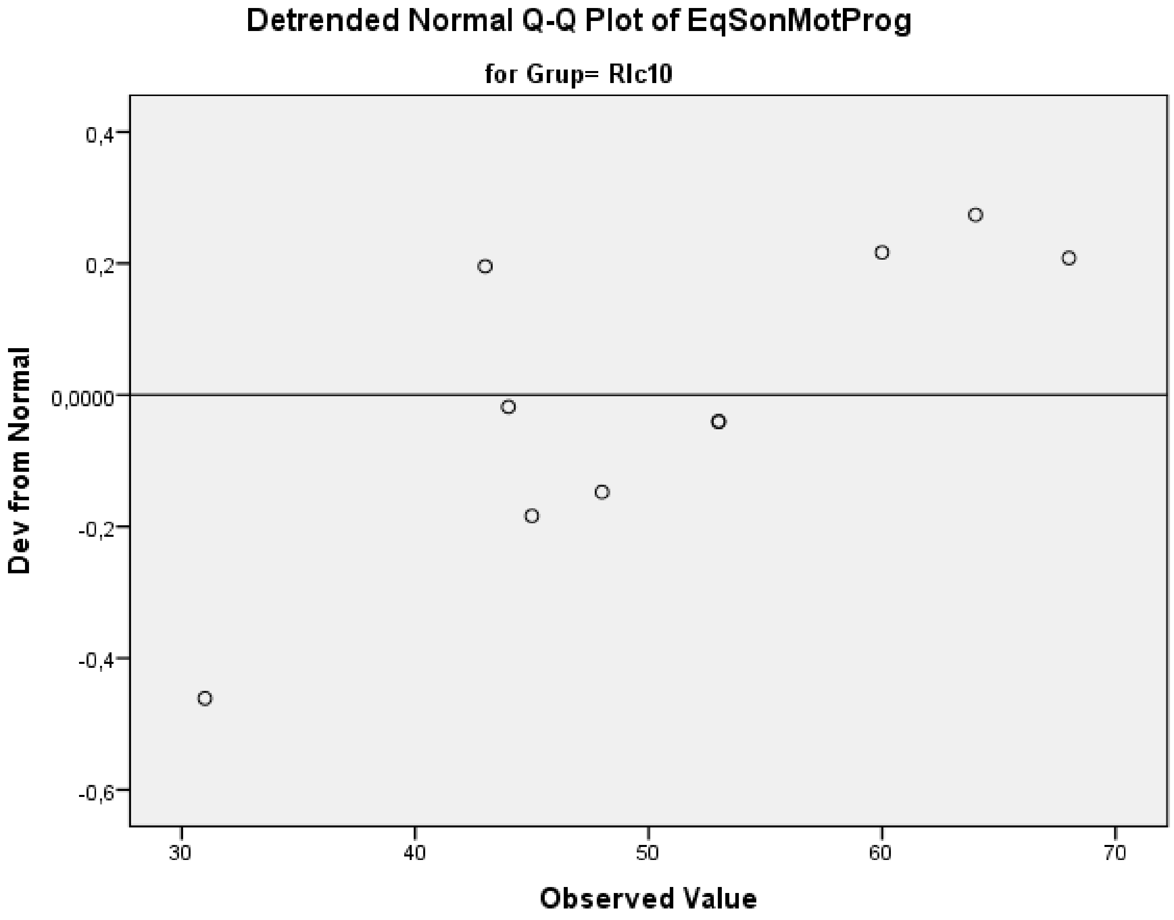

Detrended Normal Q-Q Plot of EqSonMotProg
for Grup= RIc10
Dev from Normal
0,4
0,2
0,0000
-0,2
-0,4
-0,6
30
40
50
60
70
Observed Value

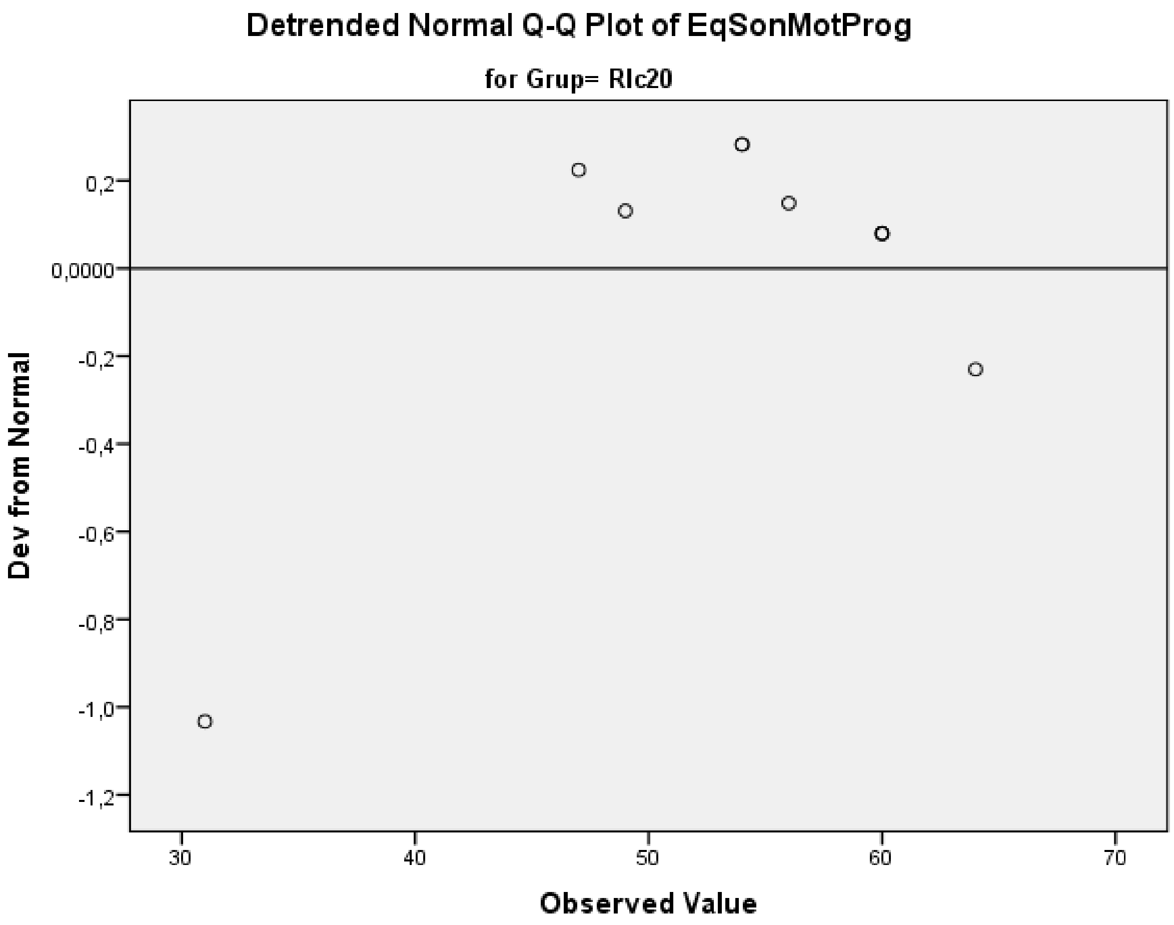

Detrended Normal Q-Q Plot of EqSonMotProg
for Grup= RIc20
Dev from Normal
0,2
0,0000
-0,2
-0,4
-0,6
-0,8
-1,0
-1,2
30
40
50
60
70
Observed Value

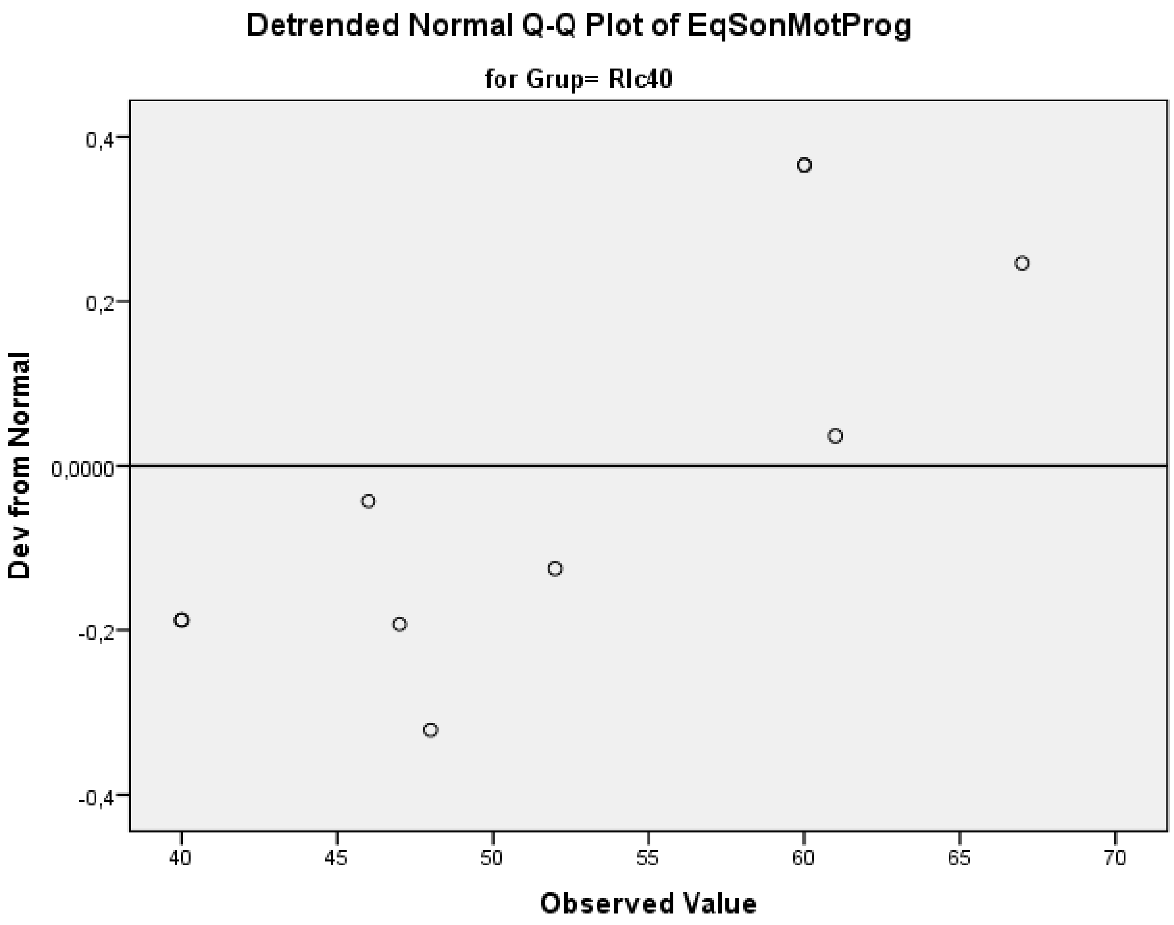
Detrended Normal Q-Q Plot of EqSonMotProg
for Grup= RIc40
Dev from Normal
0,4
0,2
0,0000
-0,2
-0,4
40
45
50
55
60
65
70
Observed Value

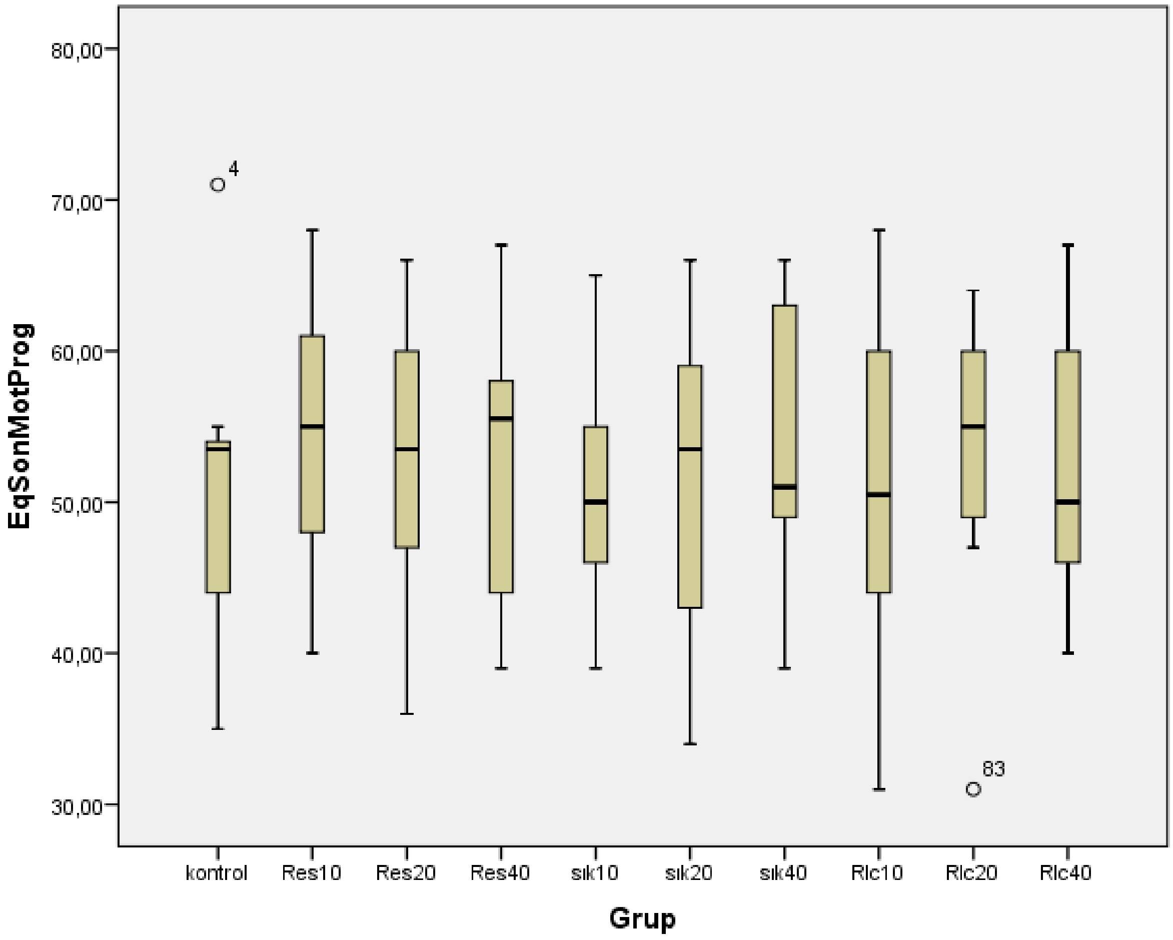
80,00
70,00
60,00
50,00
40,00
30,00
EqSonMotProg
4
83
kontrol
Res10
Res20
Res40
sık10
sık20
sık40
Rlc10
Rlc20
Rlc40
Grup

```
ONEWAY EqSonMotProg EqSonMotCasa BY Grup
  /STATISTICS DESCRIPTIVES HOMOGENEITY
  /MISSING ANALYSIS
  /POSTHOC=TUKEY DUNCAN ALPHA(0.05).
```

Oneway

Notes

Output Created		22-DEC-2020 11:05:42
Comments		
Input	Active Dataset	DataSet0
	Filter	<none>
	Weight	<none>
	Split File	<none>
	N of Rows in Working Data File	100
Missing Value Handling	Definition of Missing	User-defined missing values are treated as missing.
	Cases Used	Statistics for each analysis are based on cases with no missing data for any variable in the analysis.
Syntax		ONEWAY EqSonMotProg EqSonMotCasa BY Grup<br>/STATISTICS DESCRIPTIVES HOMOGENEITY<br>/MISSING ANALYSIS<br>/POSTHOC=TUKEY DUNCAN ALPHA(0.05).
Resources	Processor Time	00:00:00,08
	Elapsed Time	00:00:00,08

Descriptives

		N	Mean	Std. Deviation	Std. Error	95% Confidence Interval for Mean
						Lower Bound
EqSonMotProg	kontrol	10	51,2000	9,48449	2,99926	44,4152
	Res10	10	54,2000	9,49620	3,00296	47,4068
	Res20	10	52,0000	10,24153	3,23866	44,6737

	Res40	10	51,9000	9,36245	2,96067	45,2025
	sık10	10	50,6000	8,04432	2,54384	44,8454
	sık20	10	51,8000	9,97553	3,15454	44,6639
	sık40	10	53,5000	8,44919	2,67187	47,4558
	Rlc10	10	50,9000	11,08001	3,50381	42,9738
	Rlc20	10	53,5000	9,50146	3,00463	46,7031
	Rlc40	10	52,1000	9,42161	2,97937	45,3602
	Total	100	52,1700	9,16742	,91674	50,3510
EqSonMotCasa	kontrol	10	91,9000	4,04008	1,27758	89,0099
	Res10	10	96,2000	2,04396	,64636	94,7378
	Res20	10	96,0000	1,63299	,51640	94,8318
	Res40	10	94,1000	4,43346	1,40198	90,9285
	sık10	10	93,3000	3,05687	,96667	91,1132
	sık20	10	93,3000	4,29599	1,35851	90,2268
	sık40	10	93,4000	2,36643	,74833	91,7072
	Rlc10	10	95,3000	1,49443	,47258	94,2309
	Rlc20	10	95,4000	2,17051	,68638	93,8473
	Rlc40	10	95,2000	2,20101	,69602	93,6255
	Total	100	94,4100	3,13370	,31337	93,7882

Descriptives

		95% Confidence Interval for Mean		
		Upper Bound	Minimum	Maximum
EqSonMotProg	kontrol	57,9848	35,00	71,00
	Res10	60,9932	40,00	68,00
	Res20	59,3263	36,00	66,00
	Res40	58,5975	39,00	67,00
	sık10	56,3546	39,00	65,00
	sık20	58,9361	34,00	66,00
	sık40	59,5442	39,00	66,00
	Rlc10	58,8262	31,00	68,00
	Rlc20	60,2969	31,00	64,00
	Rlc40	58,8398	40,00	67,00
	Total	53,9890	31,00	71,00
EqSonMotCasa	kontrol	94,7901	84,00	97,00
	Res10	97,6622	93,00	98,00
	Res20	97,1682	93,00	98,00
	Res40	97,2715	85,00	98,00
	sık10	95,4868	89,00	98,00

	sık20	96,3732	82,00	97,00
	sık40	95,0928	89,00	96,00
	Rlc10	96,3691	93,00	97,00
	Rlc20	96,9527	91,00	98,00
	Rlc40	96,7745	90,00	98,00
	Total	95,0318	82,00	98,00

Test of Homogeneity of Variances

	Levene Statistic	df1	df2	Sig.
EqSonMotProg	,268	9	90	,982
EqSonMotCasa	2,043	9	90	,043

ANOVA

		Sum of Squares	df	Mean Square	F
EqSonMotProg	Between Groups	129,210	9	14,357	,158
	Within Groups	8190,900	90	91,010	
	Total	8320,110	99		
EqSonMotCasa	Between Groups	180,090	9	20,010	2,274
	Within Groups	792,100	90	8,801	
	Total	972,190	99		

ANOVA

		Sig.
EqSonMotProg	Between Groups	,997
	Within Groups	
	Total	
EqSonMotCasa	Between Groups	,024
	Within Groups	
	Total	

Post Hoc Tests

Homogeneous Subsets

EqSonMotProg

Duncan[a]

Grup	N	Subset for alpha = 0.05
		1
sık10	10	50,6000
Rlc10	10	50,9000
kontrol	10	51,2000
sık20	10	51,8000
Res40	10	51,9000
Res20	10	52,0000
Rlc40	10	52,1000
sık40	10	53,5000
Rlc20	10	53,5000
Res10	10	54,2000
Sig.		,489

Means for groups in homogeneous subsets are displayed.

a. Uses Harmonic Mean Sample Size = 10,000.

EqSonMotCasa

Duncan[a]

Grup	N	Subset for alpha = 0.05	
		1	2
kontrol	10	91,9000	
sık10	10	93,3000	93,3000
sık20	10	93,3000	93,3000
sık40	10	93,4000	93,4000

Res40	10	94,1000	94,1000
Rlc40	10		95,2000
Rlc10	10		95,3000
Rlc20	10		95,4000
Res20	10		96,0000
Res10	10		96,2000
Sig.		,145	,067

Means for groups in homogeneous subsets are displayed.

a. Uses Harmonic Mean Sample Size = 10,000.

Sperm Velocity parameters of of post-equilibration ram sperm- ANOVA

Groups
1: Control
2: RES10
3: RES20
4: RES40
5: CD10
6: CD20
7: CD40
8: RLC10
9: RLC20
10: RLC40

```
EXAMINE VARIABLES=VAP VSL VCL ALH BCF STR LIN BY Grup
  /PLOT BOXPLOT STEMLEAF HISTOGRAM NPPLOT
  /COMPARE VARIABLES
  /STATISTICS DESCRIPTIVES
  /CINTERVAL 95
  /MISSING LISTWISE
  /NOTOTAL.
```

Explore

Notes

Output Created		22-DEC-2020 21:41:16
Comments		
Input	Active Dataset	DataSet0
	Filter	<none>
	Weight	<none>
	Split File	<none>
	N of Rows in Working Data File	100
Missing Value Handling	Definition of Missing	User-defined missing values for dependent variables are treated as missing.
	Cases Used	Statistics are based on cases with no missing values for any dependent variable or factor used.

Syntax		EXAMINE VARIABLES=VAP VSL VCL ALH BCF STR LIN BY Grup<br>/PLOT BOXPLOT STEMLEAF HISTOGRAM NPPLOT<br>/COMPARE VARIABLES<br>/STATISTICS DESCRIPTIVES<br>/CINTERVAL 95<br>/MISSING LISTWISE<br>/NOTOTAL.
Resources	Processor Time	00:00:43,84
	Elapsed Time	00:00:48,71

Grup

Case Processing Summary

		Cases					
		Valid		Missing		Total	
	Grup	N	Percent	N	Percent	N	Percent
VAP	1,00	10	100,0%	0	0,0%	10	100,0%
	2,00	10	100,0%	0	0,0%	10	100,0%
	3,00	10	100,0%	0	0,0%	10	100,0%
	4,00	10	100,0%	0	0,0%	10	100,0%
	5,00	10	100,0%	0	0,0%	10	100,0%
	6,00	10	100,0%	0	0,0%	10	100,0%
	7,00	10	100,0%	0	0,0%	10	100,0%
	8,00	10	100,0%	0	0,0%	10	100,0%
	9,00	10	100,0%	0	0,0%	10	100,0%
	10,00	10	100,0%	0	0,0%	10	100,0%
VSL	1,00	10	100,0%	0	0,0%	10	100,0%
	2,00	10	100,0%	0	0,0%	10	100,0%
	3,00	10	100,0%	0	0,0%	10	100,0%
	4,00	10	100,0%	0	0,0%	10	100,0%
	5,00	10	100,0%	0	0,0%	10	100,0%
	6,00	10	100,0%	0	0,0%	10	100,0%
	7,00	10	100,0%	0	0,0%	10	100,0%
	8,00	10	100,0%	0	0,0%	10	100,0%

	9,00	10	100,0%	0	0,0%	10	100,0%
	10,00	10	100,0%	0	0,0%	10	100,0%
VCL	1,00	10	100,0%	0	0,0%	10	100,0%
	2,00	10	100,0%	0	0,0%	10	100,0%
	3,00	10	100,0%	0	0,0%	10	100,0%
	4,00	10	100,0%	0	0,0%	10	100,0%
	5,00	10	100,0%	0	0,0%	10	100,0%
	6,00	10	100,0%	0	0,0%	10	100,0%
	7,00	10	100,0%	0	0,0%	10	100,0%
	8,00	10	100,0%	0	0,0%	10	100,0%
	9,00	10	100,0%	0	0,0%	10	100,0%
	10,00	10	100,0%	0	0,0%	10	100,0%
ALH	1,00	10	100,0%	0	0,0%	10	100,0%
	2,00	10	100,0%	0	0,0%	10	100,0%
	3,00	10	100,0%	0	0,0%	10	100,0%
	4,00	10	100,0%	0	0,0%	10	100,0%
	5,00	10	100,0%	0	0,0%	10	100,0%
	6,00	10	100,0%	0	0,0%	10	100,0%
	7,00	10	100,0%	0	0,0%	10	100,0%
	8,00	10	100,0%	0	0,0%	10	100,0%
	9,00	10	100,0%	0	0,0%	10	100,0%
	10,00	10	100,0%	0	0,0%	10	100,0%
BCF	1,00	10	100,0%	0	0,0%	10	100,0%
	2,00	10	100,0%	0	0,0%	10	100,0%
	3,00	10	100,0%	0	0,0%	10	100,0%
	4,00	10	100,0%	0	0,0%	10	100,0%
	5,00	10	100,0%	0	0,0%	10	100,0%
	6,00	10	100,0%	0	0,0%	10	100,0%
	7,00	10	100,0%	0	0,0%	10	100,0%
	8,00	10	100,0%	0	0,0%	10	100,0%
	9,00	10	100,0%	0	0,0%	10	100,0%
	10,00	10	100,0%	0	0,0%	10	100,0%
STR	1,00	10	100,0%	0	0,0%	10	100,0%
	2,00	10	100,0%	0	0,0%	10	100,0%
	3,00	10	100,0%	0	0,0%	10	100,0%
	4,00	10	100,0%	0	0,0%	10	100,0%
	5,00	10	100,0%	0	0,0%	10	100,0%
	6,00	10	100,0%	0	0,0%	10	100,0%
	7,00	10	100,0%	0	0,0%	10	100,0%
	8,00	10	100,0%	0	0,0%	10	100,0%
	9,00	10	100,0%	0	0,0%	10	100,0%

	10,00	10	100,0%	0	0,0%	10	100,0%
LIN	1,00	10	100,0%	0	0,0%	10	100,0%
	2,00	10	100,0%	0	0,0%	10	100,0%
	3,00	10	100,0%	0	0,0%	10	100,0%
	4,00	10	100,0%	0	0,0%	10	100,0%
	5,00	10	100,0%	0	0,0%	10	100,0%
	6,00	10	100,0%	0	0,0%	10	100,0%
	7,00	10	100,0%	0	0,0%	10	100,0%
	8,00	10	100,0%	0	0,0%	10	100,0%
	9,00	10	100,0%	0	0,0%	10	100,0%
	10,00	10	100,0%	0	0,0%	10	100,0%

Descriptives

	Grup			Statistic	Std. Error
VAP	1,00	Mean		122,5800	5,27231
		95% Confidence Interval for Mean	Lower Bound	110,6532	
			Upper Bound	134,5068	
		5% Trimmed Mean		122,3056	
		Median		122,8000	
		Variance		277,973	
		Std. Deviation		16,67252	
		Minimum		94,80	
		Maximum		155,30	
		Range		60,50	
		Interquartile Range		19,95	
		Skewness		,321	,687
		Kurtosis		,957	1,334
	2,00	Mean		124,8700	4,00932
		95% Confidence Interval for Mean	Lower Bound	115,8003	
			Upper Bound	133,9397	
		5% Trimmed Mean		124,3500	
		Median		122,1000	
		Variance		160,747	
		Std. Deviation		12,67860	
		Minimum		110,70	
		Maximum		148,40	
		Range		37,70	

	Interquartile Range		21,13	
	Skewness		,660	,687
	Kurtosis		-,647	1,334
3,00	Mean		123,9100	4,17592
	95% Confidence Interval for Mean	Lower Bound	114,4634	
		Upper Bound	133,3566	
	5% Trimmed Mean		123,8278	
	Median		122,0500	
	Variance		174,383	
	Std. Deviation		13,20542	
	Minimum		99,60	
	Maximum		149,70	
	Range		50,10	
	Interquartile Range		14,43	
	Skewness		,183	,687
	Kurtosis		1,482	1,334
4,00	Mean		123,3700	3,70012
	95% Confidence Interval for Mean	Lower Bound	114,9997	
		Upper Bound	131,7403	
	5% Trimmed Mean		123,5778	
	Median		121,5000	
	Variance		136,909	
	Std. Deviation		11,70081	
	Minimum		102,10	
	Maximum		140,90	
	Range		38,80	
	Interquartile Range		18,83	
	Skewness		-,108	,687
	Kurtosis		-,130	1,334
5,00	Mean		122,3800	3,53245
	95% Confidence Interval for Mean	Lower Bound	114,3891	
		Upper Bound	130,3709	
	5% Trimmed Mean		121,9056	
	Median		118,8000	
	Variance		124,782	
	Std. Deviation		11,17058	
	Minimum		111,40	
	Maximum		141,90	

	Range		30,50	
	Interquartile Range		19,20	
	Skewness		,729	,687
	Kurtosis		-,929	1,334
6,00	Mean		123,2600	3,89276
	95% Confidence Interval for Mean	Lower Bound	114,4540	
		Upper Bound	132,0660	
	5% Trimmed Mean		123,3611	
	Median		122,4500	
	Variance		151,536	
	Std. Deviation		12,31000	
	Minimum		103,30	
	Maximum		141,40	
	Range		38,10	
	Interquartile Range		16,05	
	Skewness		-,197	,687
	Kurtosis		-,445	1,334
7,00	Mean		126,2300	3,86779
	95% Confidence Interval for Mean	Lower Bound	117,4805	
		Upper Bound	134,9795	
	5% Trimmed Mean		126,1667	
	Median		125,6000	
	Variance		149,598	
	Std. Deviation		12,23102	
	Minimum		104,80	
	Maximum		148,80	
	Range		44,00	
	Interquartile Range		16,37	
	Skewness		,236	,687
	Kurtosis		,635	1,334
8,00	Mean		124,9700	4,63489
	95% Confidence Interval for Mean	Lower Bound	114,4851	
		Upper Bound	135,4549	
	5% Trimmed Mean		124,5611	
	Median		121,3500	
	Variance		214,822	
	Std. Deviation		14,65682	
	Minimum		101,80	

		Maximum		155,50	
		Range		53,70	
		Interquartile Range		11,67	
		Skewness		,913	,687
		Kurtosis		1,625	1,334
	9,00	Mean		126,6300	4,27034
		95% Confidence Interval for Mean	Lower Bound	116,9698	
			Upper Bound	136,2902	
		5% Trimmed Mean		126,3944	
		Median		126,7000	
		Variance		182,358	
		Std. Deviation		13,50400	
		Minimum		103,00	
		Maximum		154,50	
		Range		51,50	
		Interquartile Range		13,15	
		Skewness		,384	,687
		Kurtosis		1,863	1,334
	10,00	Mean		126,9600	5,16867
		95% Confidence Interval for Mean	Lower Bound	115,2677	
			Upper Bound	138,6523	
		5% Trimmed Mean		126,7500	
		Median		125,3500	
		Variance		267,152	
		Std. Deviation		16,34477	
		Minimum		102,90	
		Maximum		154,80	
		Range		51,90	
		Interquartile Range		27,40	
		Skewness		,247	,687
		Kurtosis		-,856	1,334
VSL	1,00	Mean		102,2500	4,92958
		95% Confidence Interval for Mean	Lower Bound	91,0985	
			Upper Bound	113,4015	
		5% Trimmed Mean		101,6222	
		Median		100,0000	
		Variance		243,007	
		Std. Deviation		15,58869	
		Minimum		79,60	

	Maximum		136,20	
	Range		56,60	
	Interquartile Range		19,55	
	Skewness		,950	,687
	Kurtosis		1,795	1,334
2,00	Mean		104,0300	3,63746
	95% Confidence Interval for Mean	Lower Bound	95,8015	
		Upper Bound	112,2585	
	5% Trimmed Mean		103,4944	
	Median		99,5500	
	Variance		132,311	
	Std. Deviation		11,50266	
	Minimum		91,00	
	Maximum		126,70	
	Range		35,70	
	Interquartile Range		18,67	
	Skewness		1,026	,687
	Kurtosis		-,001	1,334
3,00	Mean		102,0200	3,84190
	95% Confidence Interval for Mean	Lower Bound	93,3290	
		Upper Bound	110,7110	
	5% Trimmed Mean		101,5944	
	Median		102,6000	
	Variance		147,602	
	Std. Deviation		12,14915	
	Minimum		83,30	
	Maximum		128,40	
	Range		45,10	
	Interquartile Range		13,47	
	Skewness		,796	,687
	Kurtosis		2,000	1,334
4,00	Mean		101,6000	3,56667
	95% Confidence Interval for Mean	Lower Bound	93,5316	
		Upper Bound	109,6684	
	5% Trimmed Mean		101,1944	
	Median		100,4500	
	Variance		127,211	
	Std. Deviation		11,27879	
	Minimum		86,30	
	Maximum		124,20	
	Range		37,90	

	Interquartile Range		13,68	
	Skewness		,710	,687
	Kurtosis		,417	1,334
5,00	Mean		102,0100	3,15047
	95% Confidence Interval for Mean	Lower Bound	94,8832	
		Upper Bound	109,1368	
	5% Trimmed Mean		101,7167	
	Median		101,2500	
	Variance		99,254	
	Std. Deviation		9,96265	
	Minimum		89,00	
	Maximum		120,30	
	Range		31,30	
	Interquartile Range		15,33	
	Skewness		,668	,687
	Kurtosis		-,100	1,334
6,00	Mean		103,2500	3,94274
	95% Confidence Interval for Mean	Lower Bound	94,3309	
		Upper Bound	112,1691	
	5% Trimmed Mean		103,3278	
	Median		105,0000	
	Variance		155,452	
	Std. Deviation		12,46803	
	Minimum		82,70	
	Maximum		122,40	
	Range		39,70	
	Interquartile Range		20,45	
	Skewness		-,168	,687
	Kurtosis		-,658	1,334
7,00	Mean		105,9800	4,12752
	95% Confidence Interval for Mean	Lower Bound	96,6429	
		Upper Bound	115,3171	
	5% Trimmed Mean		105,8833	
	Median		106,9500	
	Variance		170,364	
	Std. Deviation		13,05236	
	Minimum		85,90	
	Maximum		127,80	
	Range		41,90	
	Interquartile Range		18,88	
	Skewness		,026	,687

		Kurtosis		-,416	1,334
	8,00	Mean		102,4600	5,09301
		95% Confidence Interval for Mean	Lower Bound	90,9388	
			Upper Bound	113,9812	
		5% Trimmed Mean		101,9333	
		Median		99,7500	
		Variance		259,387	
		Std. Deviation		16,10550	
		Minimum		81,10	
		Maximum		133,30	
		Range		52,20	
		Interquartile Range		23,80	
		Skewness		,699	,687
		Kurtosis		,038	1,334
	9,00	Mean		105,0500	4,25261
		95% Confidence Interval for Mean	Lower Bound	95,4299	
			Upper Bound	114,6701	
		5% Trimmed Mean		104,7667	
		Median		107,0000	
		Variance		180,847	
		Std. Deviation		13,44794	
		Minimum		84,70	
		Maximum		130,50	
		Range		45,80	
		Interquartile Range		17,42	
		Skewness		,141	,687
		Kurtosis		,511	1,334
	10,00	Mean		104,5600	4,57414
		95% Confidence Interval for Mean	Lower Bound	94,2126	
			Upper Bound	114,9074	
		5% Trimmed Mean		104,4389	
		Median		99,6500	
		Variance		209,227	
		Std. Deviation		14,46468	
		Minimum		86,00	
		Maximum		125,30	
		Range		39,30	
		Interquartile Range		25,03	
		Skewness		,252	,687
		Kurtosis		-1,309	1,334
VCL	1,00	Mean		191,8500	9,09079

	95% Confidence Interval for Mean	Lower Bound	171,2852	
		Upper Bound	212,4148	
	5% Trimmed Mean		192,3444	
	Median		194,3500	
	Variance		826,425	
	Std. Deviation		28,74761	
	Minimum		143,70	
	Maximum		231,10	
	Range		87,40	
	Interquartile Range		51,02	
	Skewness		-,258	,687
	Kurtosis		-,867	1,334
2,00	Mean		191,7800	8,26540
	95% Confidence Interval for Mean	Lower Bound	173,0824	
		Upper Bound	210,4776	
	5% Trimmed Mean		190,7333	
	Median		193,9500	
	Variance		683,168	
	Std. Deviation		26,13749	
	Minimum		160,30	
	Maximum		242,10	
	Range		81,80	
	Interquartile Range		45,50	
	Skewness		,559	,687
	Kurtosis		-,250	1,334
3,00	Mean		192,4900	8,25209
	95% Confidence Interval for Mean	Lower Bound	173,8225	
		Upper Bound	211,1575	
	5% Trimmed Mean		192,3222	
	Median		186,5000	
	Variance		680,970	
	Std. Deviation		26,09540	
	Minimum		152,50	
	Maximum		235,50	
	Range		83,00	
	Interquartile Range		47,22	
	Skewness		,412	,687
	Kurtosis		-,667	1,334
4,00	Mean		192,0900	8,06963
	95% Confidence Interval for Mean	Lower Bound	173,8352	
		Upper Bound	210,3448	

	5% Trimmed Mean		191,4000	
	Median		188,3000	
	Variance		651,190	
	Std. Deviation		25,51842	
	Minimum		152,90	
	Maximum		243,70	
	Range		90,80	
	Interquartile Range		34,80	
	Skewness		,676	,687
	Kurtosis		,863	1,334
5,00	Mean		189,4600	6,44064
	95% Confidence Interval for Mean	Lower Bound	174,8903	
		Upper Bound	204,0297	
	5% Trimmed Mean		188,3389	
	Median		184,6000	
	Variance		414,818	
	Std. Deviation		20,36709	
	Minimum		168,30	
	Maximum		230,80	
	Range		62,50	
	Interquartile Range		25,88	
	Skewness		1,267	,687
	Kurtosis		,829	1,334
6,00	Mean		189,7400	6,77637
	95% Confidence Interval for Mean	Lower Bound	174,4108	
		Upper Bound	205,0692	
	5% Trimmed Mean		189,2778	
	Median		185,3500	
	Variance		459,192	
	Std. Deviation		21,42876	
	Minimum		157,70	
	Maximum		230,10	
	Range		72,40	
	Interquartile Range		27,97	
	Skewness		,679	,687
	Kurtosis		,294	1,334
7,00	Mean		192,9300	5,24426
	95% Confidence Interval for Mean	Lower Bound	181,0667	
		Upper Bound	204,7933	
	5% Trimmed Mean		192,5111	
	Median		191,3000	

	Variance		275,022	
	Std. Deviation		16,58380	
	Minimum		175,10	
	Maximum		218,30	
	Range		43,20	
	Interquartile Range		35,98	
	Skewness		,479	,687
	Kurtosis		-1,379	1,334
8,00	Mean		191,9900	6,23534
	95% Confidence Interval for Mean	Lower Bound	177,8847	
		Upper Bound	206,0953	
	5% Trimmed Mean		191,1056	
	Median		182,0500	
	Variance		388,794	
	Std. Deviation		19,71787	
	Minimum		173,40	
	Maximum		226,50	
	Range		53,10	
	Interquartile Range		35,03	
	Skewness		,717	,687
	Kurtosis		-1,179	1,334
9,00	Mean		190,8800	8,14495
	95% Confidence Interval for Mean	Lower Bound	172,4548	
		Upper Bound	209,3052	
	5% Trimmed Mean		190,7389	
	Median		185,9500	
	Variance		663,402	
	Std. Deviation		25,75659	
	Minimum		152,10	
	Maximum		232,20	
	Range		80,10	
	Interquartile Range		47,88	
	Skewness		,327	,687
	Kurtosis		-,914	1,334
10,00	Mean		192,4400	9,53729
	95% Confidence Interval for Mean	Lower Bound	170,8652	
		Upper Bound	214,0148	
	5% Trimmed Mean		191,9389	
	Median		187,1000	
	Variance		909,598	
	Std. Deviation		30,15955	

		Minimum		152,60	
		Maximum		241,30	
		Range		88,70	
		Interquartile Range		50,50	
		Skewness		,598	,687
		Kurtosis		-,643	1,334
ALH	1,00	Mean		6,5600	,28566
		95% Confidence Interval for Mean	Lower Bound	5,9138	
			Upper Bound	7,2062	
		5% Trimmed Mean		6,5111	
		Median		6,3000	
		Variance		,816	
		Std. Deviation		,90333	
		Minimum		5,70	
		Maximum		8,30	
		Range		2,60	
		Interquartile Range		1,55	
		Skewness		,758	,687
		Kurtosis		-,476	1,334
	2,00	Mean		6,5400	,33307
		95% Confidence Interval for Mean	Lower Bound	5,7866	
			Upper Bound	7,2934	
		5% Trimmed Mean		6,4611	
		Median		6,1500	
		Variance		1,109	
		Std. Deviation		1,05325	
		Minimum		5,50	
		Maximum		9,00	
		Range		3,50	
		Interquartile Range		1,40	
		Skewness		1,618	,687
		Kurtosis		2,628	1,334
	3,00	Mean		6,6400	,40667
		95% Confidence Interval for Mean	Lower Bound	5,7201	
			Upper Bound	7,5599	
		5% Trimmed Mean		6,5722	
		Median		6,2500	
		Variance		1,654	
		Std. Deviation		1,28599	
		Minimum		5,10	
		Maximum		9,40	

	Range		4,30	
	Interquartile Range		1,67	
	Skewness		1,109	,687
	Kurtosis		1,187	1,334
4,00	Mean		6,5400	,35845
	95% Confidence Interval for Mean	Lower Bound	5,7291	
		Upper Bound	7,3509	
	5% Trimmed Mean		6,4667	
	Median		6,0000	
	Variance		1,285	
	Std. Deviation		1,13353	
	Minimum		5,50	
	Maximum		8,90	
	Range		3,40	
	Interquartile Range		1,75	
	Skewness		1,060	,687
	Kurtosis		,385	1,334
5,00	Mean		6,4000	,28868
	95% Confidence Interval for Mean	Lower Bound	5,7470	
		Upper Bound	7,0530	
	5% Trimmed Mean		6,3500	
	Median		6,0000	
	Variance		,833	
	Std. Deviation		,91287	
	Minimum		5,40	
	Maximum		8,30	
	Range		2,90	
	Interquartile Range		1,25	
	Skewness		1,111	,687
	Kurtosis		,586	1,334
6,00	Mean		6,4200	,35239
	95% Confidence Interval for Mean	Lower Bound	5,6228	
		Upper Bound	7,2172	
	5% Trimmed Mean		6,3444	
	Median		5,7500	
	Variance		1,242	
	Std. Deviation		1,11435	
	Minimum		5,50	
	Maximum		8,70	
	Range		3,20	
	Interquartile Range		1,65	

	Skewness		1,095	,687
	Kurtosis		,170	1,334
7,00	Mean		6,4400	,29067
	95% Confidence Interval for Mean	Lower Bound	5,7825	
		Upper Bound	7,0975	
	5% Trimmed Mean		6,4000	
	Median		6,3000	
	Variance		,845	
	Std. Deviation		,91918	
	Minimum		5,30	
	Maximum		8,30	
	Range		3,00	
	Interquartile Range		1,45	
	Skewness		,793	,687
	Kurtosis		,476	1,334
8,00	Mean		6,5200	,31262
	95% Confidence Interval for Mean	Lower Bound	5,8128	
		Upper Bound	7,2272	
	5% Trimmed Mean		6,4722	
	Median		6,1500	
	Variance		,977	
	Std. Deviation		,98860	
	Minimum		5,30	
	Maximum		8,60	
	Range		3,30	
	Interquartile Range		1,30	
	Skewness		1,141	,687
	Kurtosis		,931	1,334
9,00	Mean		6,1800	,37112
	95% Confidence Interval for Mean	Lower Bound	5,3405	
		Upper Bound	7,0195	
	5% Trimmed Mean		6,1000	
	Median		5,8500	
	Variance		1,377	
	Std. Deviation		1,17360	
	Minimum		4,90	
	Maximum		8,90	
	Range		4,00	
	Interquartile Range		1,47	
	Skewness		1,463	,687
	Kurtosis		2,566	1,334

	10,00	Mean		6,3600	,34935
		95% Confidence Interval for Mean	Lower Bound	5,5697	
			Upper Bound	7,1503	
		5% Trimmed Mean		6,3222	
		Median		6,3000	
		Variance		1,220	
		Std. Deviation		1,10474	
		Minimum		5,10	
		Maximum		8,30	
		Range		3,20	
		Interquartile Range		2,00	
		Skewness		,347	,687
		Kurtosis		-,972	1,334
BCF	1,00	Mean		35,9300	1,14718
		95% Confidence Interval for Mean	Lower Bound	33,3349	
			Upper Bound	38,5251	
		5% Trimmed Mean		36,0167	
		Median		37,1000	
		Variance		13,160	
		Std. Deviation		3,62769	
		Minimum		29,50	
		Maximum		40,80	
		Range		11,30	
		Interquartile Range		6,00	
		Skewness		-,418	,687
		Kurtosis		-,800	1,334
	2,00	Mean		34,5400	1,39038
		95% Confidence Interval for Mean	Lower Bound	31,3947	
			Upper Bound	37,6853	
		5% Trimmed Mean		34,7389	
		Median		35,7500	
		Variance		19,332	
		Std. Deviation		4,39677	
		Minimum		25,70	
		Maximum		39,80	
		Range		14,10	
		Interquartile Range		7,40	
		Skewness		-1,022	,687
		Kurtosis		,239	1,334
	3,00	Mean		34,7800	1,17953
		95% Confidence Interval for	Lower Bound	32,1117	

	Mean	Upper Bound	37,4483	
	5% Trimmed Mean		35,0722	
	Median		35,3500	
	Variance		13,913	
	Std. Deviation		3,73000	
	Minimum		26,00	
	Maximum		38,30	
	Range		12,30	
	Interquartile Range		4,85	
	Skewness		-1,564	,687
	Kurtosis		2,841	1,334
4,00	Mean		33,8400	1,28575
	95% Confidence Interval for Mean	Lower Bound	30,9314	
		Upper Bound	36,7486	
	5% Trimmed Mean		34,0667	
	Median		34,0500	
	Variance		16,532	
	Std. Deviation		4,06590	
	Minimum		25,30	
	Maximum		38,30	
	Range		13,00	
	Interquartile Range		5,88	
	Skewness		-,956	,687
	Kurtosis		,821	1,334
5,00	Mean		35,3800	1,27670
	95% Confidence Interval for Mean	Lower Bound	32,4919	
		Upper Bound	38,2681	
	5% Trimmed Mean		35,6333	
	Median		35,2500	
	Variance		16,300	
	Std. Deviation		4,03727	
	Minimum		25,50	
	Maximum		40,70	
	Range		15,20	
	Interquartile Range		3,50	
	Skewness		-1,600	,687
	Kurtosis		4,294	1,334
6,00	Mean		35,5400	1,42667
	95% Confidence Interval for Mean	Lower Bound	32,3127	
		Upper Bound	38,7673	
	5% Trimmed Mean		35,5722	

	Median		36,4000	
	Variance		20,354	
	Std. Deviation		4,51152	
	Minimum		28,70	
	Maximum		41,80	
	Range		13,10	
	Interquartile Range		8,52	
	Skewness		-,168	,687
	Kurtosis		-1,477	1,334
7,00	Mean		34,8500	1,31785
	95% Confidence Interval for Mean	Lower Bound	31,8688	
		Upper Bound	37,8312	
	5% Trimmed Mean		34,8944	
	Median		35,4500	
	Variance		17,367	
	Std. Deviation		4,16740	
	Minimum		28,20	
	Maximum		40,70	
	Range		12,50	
	Interquartile Range		7,68	
	Skewness		-,295	,687
	Kurtosis		-1,077	1,334
8,00	Mean		33,0500	1,37309
	95% Confidence Interval for Mean	Lower Bound	29,9438	
		Upper Bound	36,1562	
	5% Trimmed Mean		33,2389	
	Median		33,3000	
	Variance		18,854	
	Std. Deviation		4,34211	
	Minimum		23,80	
	Maximum		38,90	
	Range		15,10	
	Interquartile Range		6,30	
	Skewness		-,875	,687
	Kurtosis		1,234	1,334
9,00	Mean		33,6400	1,05559
	95% Confidence Interval for Mean	Lower Bound	31,2521	
		Upper Bound	36,0279	
	5% Trimmed Mean		33,6944	
	Median		33,0000	
	Variance		11,143	

		Std. Deviation		3,33806	
		Minimum		28,20	
		Maximum		38,10	
		Range		9,90	
		Interquartile Range		5,77	
		Skewness		-,111	,687
		Kurtosis		-1,195	1,334
	10,00	Mean		34,2000	,81418
		95% Confidence Interval for Mean	Lower Bound	32,3582	
			Upper Bound	36,0418	
		5% Trimmed Mean		34,2056	
		Median		34,4500	
		Variance		6,629	
		Std. Deviation		2,57466	
		Minimum		29,70	
		Maximum		38,60	
		Range		8,90	
		Interquartile Range		3,70	
		Skewness		-,073	,687
		Kurtosis		-,093	1,334
STR	1,00	Mean		82,1000	1,33708
		95% Confidence Interval for Mean	Lower Bound	79,0753	
			Upper Bound	85,1247	
		5% Trimmed Mean		82,2778	
		Median		82,0000	
		Variance		17,878	
		Std. Deviation		4,22821	
		Minimum		74,00	
		Maximum		87,00	
		Range		13,00	
		Interquartile Range		5,50	
		Skewness		-,947	,687
		Kurtosis		,264	1,334
	2,00	Mean		82,4000	1,60000
		95% Confidence Interval for Mean	Lower Bound	78,7805	
			Upper Bound	86,0195	
		5% Trimmed Mean		82,7222	
		Median		85,0000	
		Variance		25,600	
		Std. Deviation		5,05964	
		Minimum		73,00	

	Maximum		86,00	
	Range		13,00	
	Interquartile Range		7,50	
	Skewness		-1,303	,687
	Kurtosis		,184	1,334
3,00	Mean		81,6000	1,51438
	95% Confidence Interval for Mean	Lower Bound	78,1742	
		Upper Bound	85,0258	
	5% Trimmed Mean		81,8333	
	Median		83,5000	
	Variance		22,933	
	Std. Deviation		4,78888	
	Minimum		72,00	
	Maximum		87,00	
	Range		15,00	
	Interquartile Range		7,00	
	Skewness		-,973	,687
	Kurtosis		,208	1,334
4,00	Mean		81,4000	1,58605
	95% Confidence Interval for Mean	Lower Bound	77,8121	
		Upper Bound	84,9879	
	5% Trimmed Mean		81,6111	
	Median		81,5000	
	Variance		25,156	
	Std. Deviation		5,01553	
	Minimum		71,00	
	Maximum		88,00	
	Range		17,00	
	Interquartile Range		8,00	
	Skewness		-,778	,687
	Kurtosis		,768	1,334
5,00	Mean		82,4000	1,38404
	95% Confidence Interval for Mean	Lower Bound	79,2691	
		Upper Bound	85,5309	
	5% Trimmed Mean		82,7222	
	Median		83,0000	
	Variance		19,156	
	Std. Deviation		4,37671	
	Minimum		72,00	
	Maximum		87,00	
	Range		15,00	

	Interquartile Range		5,50	
	Skewness		-1,490	,687
	Kurtosis		3,274	1,334
6,00	Mean		82,5000	1,68819
	95% Confidence Interval for Mean	Lower Bound	78,6810	
		Upper Bound	86,3190	
	5% Trimmed Mean		82,7778	
	Median		84,0000	
	Variance		28,500	
	Std. Deviation		5,33854	
	Minimum		71,00	
	Maximum		89,00	
	Range		18,00	
	Interquartile Range		7,00	
	Skewness		-,945	,687
	Kurtosis		1,416	1,334
7,00	Mean		82,7000	1,31698
	95% Confidence Interval for Mean	Lower Bound	79,7208	
		Upper Bound	85,6792	
	5% Trimmed Mean		82,8889	
	Median		84,0000	
	Variance		17,344	
	Std. Deviation		4,16467	
	Minimum		75,00	
	Maximum		87,00	
	Range		12,00	
	Interquartile Range		7,00	
	Skewness		-,866	,687
	Kurtosis		-,378	1,334
8,00	Mean		80,9000	1,68292
	95% Confidence Interval for Mean	Lower Bound	77,0930	
		Upper Bound	84,7070	
	5% Trimmed Mean		81,2222	
	Median		83,0000	
	Variance		28,322	
	Std. Deviation		5,32186	
	Minimum		70,00	
	Maximum		86,00	
	Range		16,00	
	Interquartile Range		8,00	
	Skewness		-1,047	,687

		Kurtosis		,350	1,334
	9,00	Mean		82,6000	1,60693
		95% Confidence Interval for Mean	Lower Bound	78,9649	
			Upper Bound	86,2351	
		5% Trimmed Mean		82,9444	
		Median		83,5000	
		Variance		25,822	
		Std. Deviation		5,08156	
		Minimum		70,00	
		Maximum		89,00	
		Range		19,00	
		Interquartile Range		3,75	
		Skewness		-1,785	,687
		Kurtosis		4,542	1,334
	10,00	Mean		82,0000	1,57762
		95% Confidence Interval for Mean	Lower Bound	78,4312	
			Upper Bound	85,5688	
		5% Trimmed Mean		82,2222	
		Median		83,5000	
		Variance		24,889	
		Std. Deviation		4,98888	
		Minimum		72,00	
		Maximum		88,00	
		Range		16,00	
		Interquartile Range		7,75	
		Skewness		-,825	,687
		Kurtosis		,217	1,334
LIN	1,00	Mean		56,1000	1,98578
		95% Confidence Interval for Mean	Lower Bound	51,6078	
			Upper Bound	60,5922	
		5% Trimmed Mean		56,3889	
		Median		58,5000	
		Variance		39,433	
		Std. Deviation		6,27960	
		Minimum		44,00	
		Maximum		63,00	
		Range		19,00	
		Interquartile Range		9,50	
		Skewness		-1,086	,687
		Kurtosis		,147	1,334

2,00	Mean		57,0000	2,48551
	95% Confidence Interval for Mean	Lower Bound	51,3774	
		Upper Bound	62,6226	
	5% Trimmed Mean		57,5000	
	Median		61,5000	
	Variance		61,778	
	Std. Deviation		7,85988	
	Minimum		42,00	
	Maximum		63,00	
	Range		21,00	
	Interquartile Range		13,00	
	Skewness		-1,213	,687
	Kurtosis		-,233	1,334
3,00	Mean		55,9000	2,28741
	95% Confidence Interval for Mean	Lower Bound	50,7255	
		Upper Bound	61,0745	
	5% Trimmed Mean		56,2778	
	Median		58,0000	
	Variance		52,322	
	Std. Deviation		7,23341	
	Minimum		41,00	
	Maximum		64,00	
	Range		23,00	
	Interquartile Range		11,75	
	Skewness		-1,122	,687
	Kurtosis		,517	1,334
4,00	Mean		55,5000	2,68845
	95% Confidence Interval for Mean	Lower Bound	49,4183	
		Upper Bound	61,5817	
	5% Trimmed Mean		55,6111	
	Median		56,0000	
	Variance		72,278	
	Std. Deviation		8,50163	
	Minimum		40,00	
	Maximum		69,00	
	Range		29,00	
	Interquartile Range		13,25	
	Skewness		-,296	,687

	Kurtosis		-,158	1,334
5,00	Mean		56,4000	2,14580
	95% Confidence Interval for Mean	Lower Bound	51,5459	
		Upper Bound	61,2541	
	5% Trimmed Mean		56,7778	
	Median		59,0000	
	Variance		46,044	
	Std. Deviation		6,78561	
	Minimum		42,00	
	Maximum		64,00	
	Range		22,00	
	Interquartile Range		10,50	
	Skewness		-1,161	,687
	Kurtosis		,865	1,334
6,00	Mean		56,6000	2,67582
	95% Confidence Interval for Mean	Lower Bound	50,5469	
		Upper Bound	62,6531	
	5% Trimmed Mean		57,1111	
	Median		59,5000	
	Variance		71,600	
	Std. Deviation		8,46168	
	Minimum		39,00	
	Maximum		65,00	
	Range		26,00	
	Interquartile Range		13,75	
	Skewness		-1,116	,687
	Kurtosis		,523	1,334
7,00	Mean		57,1000	2,30675
	95% Confidence Interval for Mean	Lower Bound	51,8818	
		Upper Bound	62,3182	
	5% Trimmed Mean		57,4444	
	Median		59,5000	
	Variance		53,211	
	Std. Deviation		7,29459	
	Minimum		43,00	
	Maximum		65,00	
	Range		22,00	
	Interquartile Range		12,75	

	Skewness		-,998	,687
	Kurtosis		-,146	1,334
8,00	Mean		55,5000	2,65100
	95% Confidence Interval for Mean	Lower Bound	49,5030	
		Upper Bound	61,4970	
	5% Trimmed Mean		55,8333	
	Median		59,0000	
	Variance		70,278	
	Std. Deviation		8,38318	
	Minimum		40,00	
	Maximum		65,00	
	Range		25,00	
	Interquartile Range		14,25	
	Skewness		-,810	,687
	Kurtosis		-,551	1,334
9,00	Mean		58,3000	2,81681
	95% Confidence Interval for Mean	Lower Bound	51,9279	
		Upper Bound	64,6721	
	5% Trimmed Mean		58,7222	
	Median		61,0000	
	Variance		79,344	
	Std. Deviation		8,90755	
	Minimum		39,00	
	Maximum		70,00	
	Range		31,00	
	Interquartile Range		12,50	
	Skewness		-1,153	,687
	Kurtosis		1,415	1,334
10,00	Mean		57,7000	2,75298
	95% Confidence Interval for Mean	Lower Bound	51,4723	
		Upper Bound	63,9277	
	5% Trimmed Mean		57,9444	
	Median		57,0000	
	Variance		75,789	
	Std. Deviation		8,70568	
	Minimum		42,00	
	Maximum		69,00	
	Range		27,00	

Interquartile Range	13,50	
Skewness	-,435	,687
Kurtosis	-,638	1,334

Tests of Normality

	Grup	Kolmogorov-Smirnov[a]			Shapiro-Wilk		
		Statistic	df	Sig.	Statistic	df	Sig.
VAP	1,00	,124	10	,200*	,981	10	,971
	2,00	,195	10	,200*	,922	10	,371
	3,00	,148	10	,200*	,960	10	,788
	4,00	,148	10	,200*	,964	10	,834
	5,00	,228	10	,150	,870	10	,101
	6,00	,177	10	,200*	,952	10	,689
	7,00	,198	10	,200*	,972	10	,912
	8,00	,267	10	,042	,878	10	,123
	9,00	,219	10	,193	,945	10	,605
	10,00	,135	10	,200*	,974	10	,927
VSL	1,00	,152	10	,200*	,946	10	,623
	2,00	,256	10	,061	,876	10	,117
	3,00	,216	10	,200*	,927	10	,420
	4,00	,184	10	,200*	,949	10	,652
	5,00	,180	10	,200*	,938	10	,535
	6,00	,111	10	,200*	,981	10	,971
	7,00	,111	10	,200*	,980	10	,965
	8,00	,127	10	,200*	,959	10	,779
	9,00	,153	10	,200*	,949	10	,659
	10,00	,192	10	,200*	,908	10	,265
VCL	1,00	,138	10	,200*	,965	10	,840
	2,00	,186	10	,200*	,927	10	,417
	3,00	,214	10	,200*	,931	10	,457
	4,00	,134	10	,200*	,967	10	,860
	5,00	,245	10	,092	,852	10	,062
	6,00	,152	10	,200*	,951	10	,678
	7,00	,174	10	,200*	,878	10	,125
	8,00	,267	10	,042	,850	10	,058
	9,00	,163	10	,200*	,944	10	,593
	10,00	,156	10	,200*	,929	10	,437
ALH	1,00	,267	10	,041	,870	10	,099

	2,00	,290	10	,017	,828	10	,031
	3,00	,204	10	,200*	,922	10	,372
	4,00	,243	10	,097	,846	10	,052
	5,00	,229	10	,147	,886	10	,152
	6,00	,311	10	,007	,812	10	,020
	7,00	,174	10	,200*	,946	10	,621
	8,00	,288	10	,018	,880	10	,132
	9,00	,194	10	,200*	,883	10	,143
	10,00	,153	10	,200*	,925	10	,402
BCF	1,00	,195	10	,200*	,943	10	,584
	2,00	,286	10	,020	,883	10	,142
	3,00	,191	10	,200*	,850	10	,058
	4,00	,136	10	,200*	,923	10	,383
	5,00	,304	10	,009	,831	10	,035
	6,00	,184	10	,200*	,934	10	,491
	7,00	,143	10	,200*	,958	10	,768
	8,00	,134	10	,200*	,950	10	,663
	9,00	,178	10	,200*	,940	10	,551
	10,00	,122	10	,200*	,983	10	,980
STR	1,00	,291	10	,017	,873	10	,108
	2,00	,296	10	,013	,740	10	,003
	3,00	,215	10	,200*	,911	10	,288
	4,00	,149	10	,200*	,946	10	,616
	5,00	,192	10	,200*	,850	10	,058
	6,00	,211	10	,200*	,909	10	,275
	7,00	,223	10	,175	,896	10	,200
	8,00	,220	10	,187	,882	10	,137
	9,00	,253	10	,069	,834	10	,038
	10,00	,179	10	,200*	,939	10	,539
LIN	1,00	,219	10	,191	,876	10	,117
	2,00	,349	10	,001	,732	10	,002
	3,00	,314	10	,006	,874	10	,110
	4,00	,160	10	,200*	,971	10	,898
	5,00	,249	10	,079	,890	10	,169
	6,00	,198	10	,200*	,881	10	,133
	7,00	,249	10	,079	,881	10	,133
	8,00	,217	10	,199	,891	10	,176
	9,00	,276	10	,030	,890	10	,169
	10,00	,165	10	,200*	,944	10	,596

*. This is a lower bound of the true significance.

a. Lilliefors Significance Correction

Grup = 1,00

Histograms

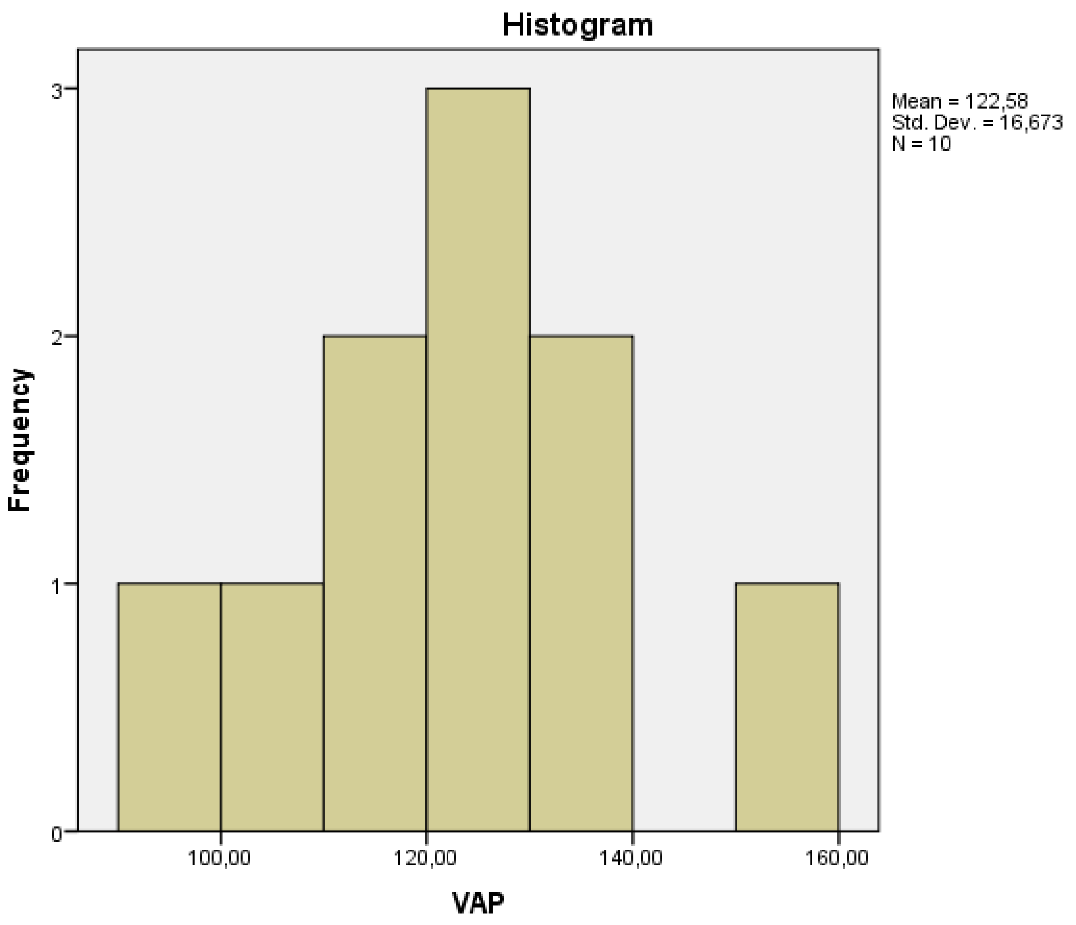

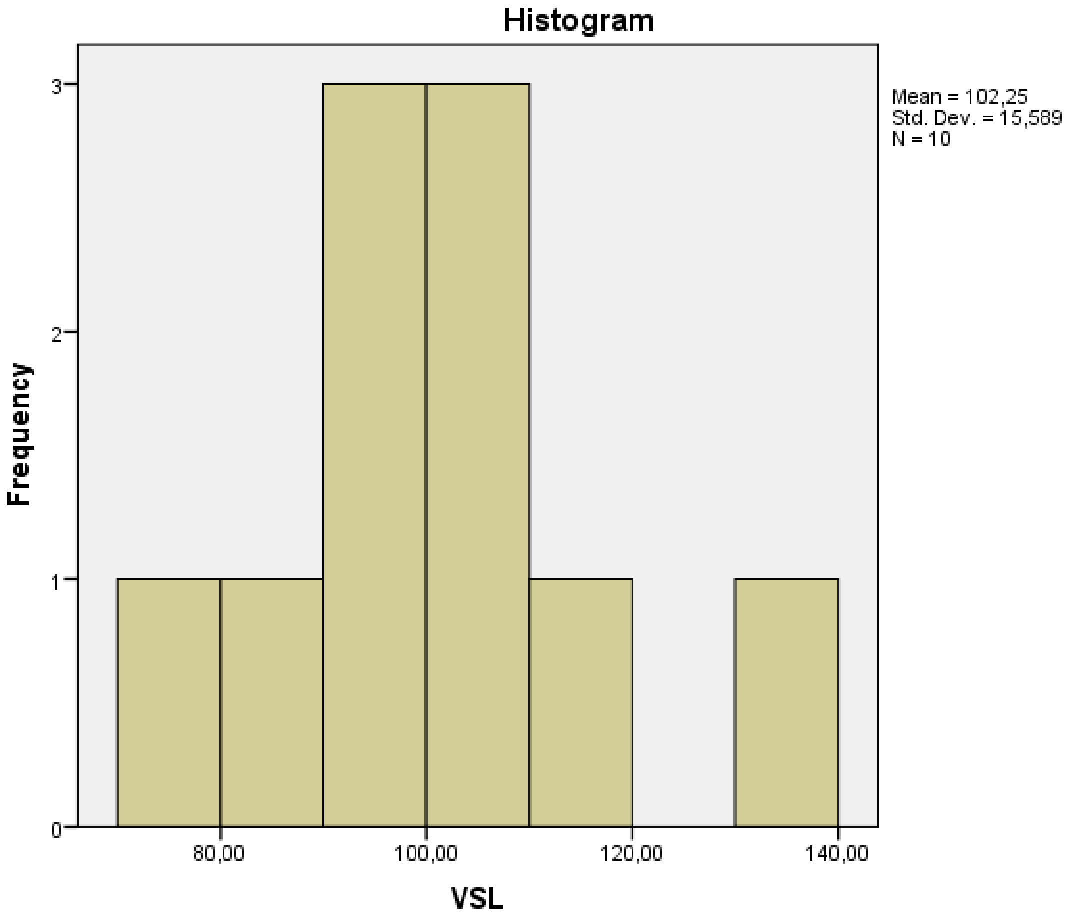
Histogram
Mean = 102,25
Std. Dev. = 15,589
N = 10
Frequency
0
1
2
3
80,00
100,00
120,00
140,00
VSL

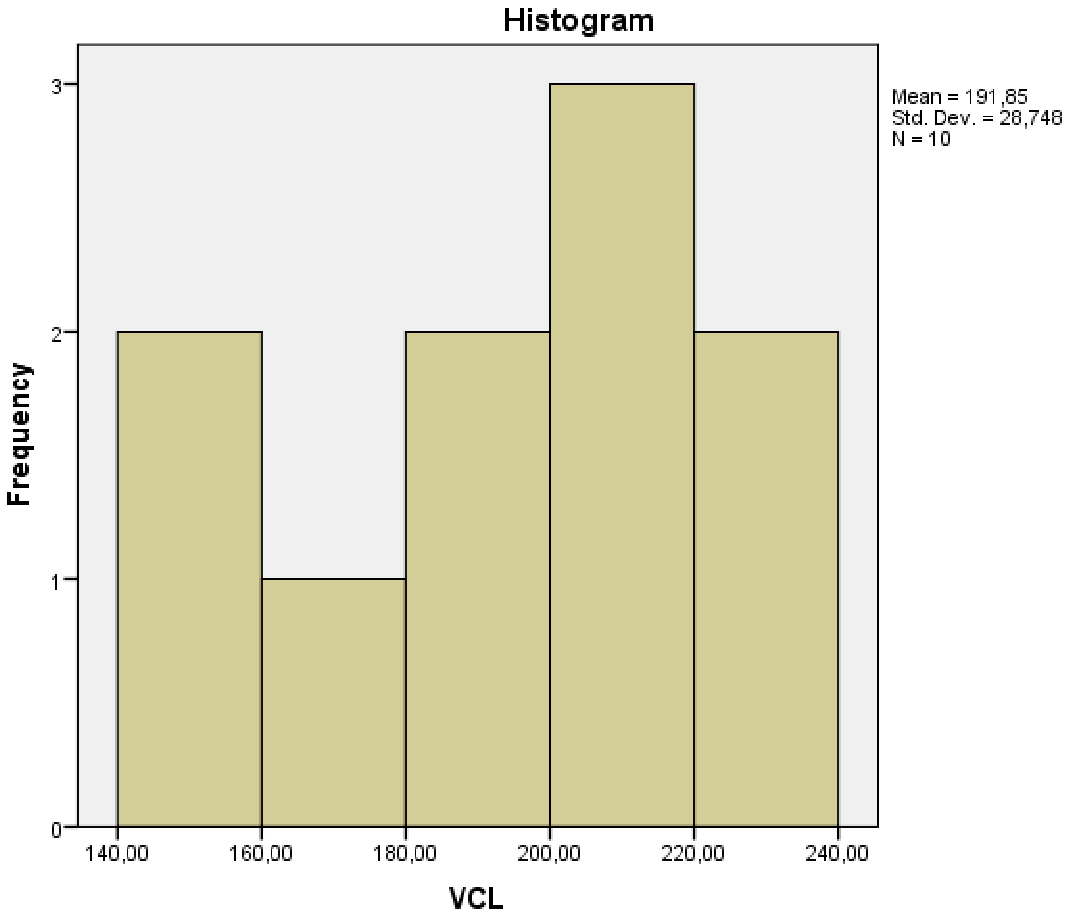

Histogram
Mean = 191,85
Std. Dev. = 28,748
N = 10
Frequency
0
1
2
3
140,00
160,00
180,00
200,00
220,00
240,00
VCL

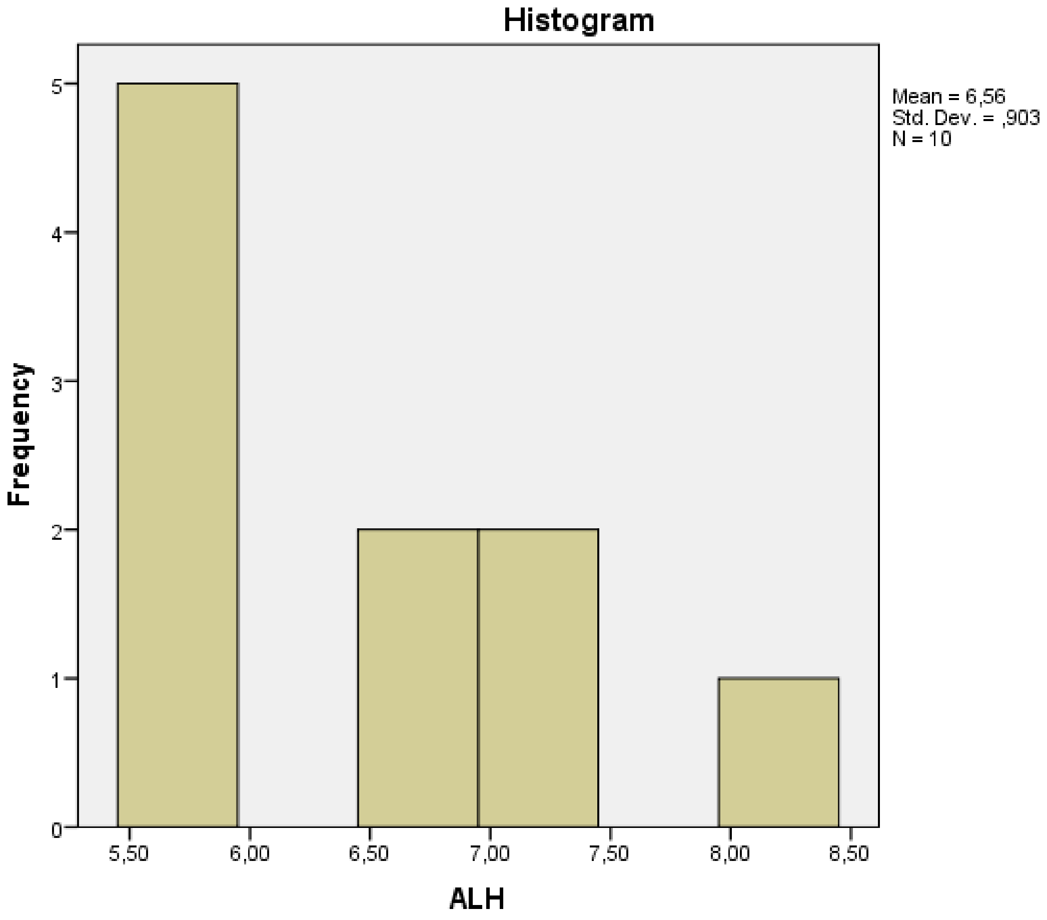
Histogram
Mean = 6,56
Std. Dev. = ,903
N = 10
Frequency
0
1
2
3
4
5
5,50
6,00
6,50
7,00
7,50
8,00
8,50
ALH

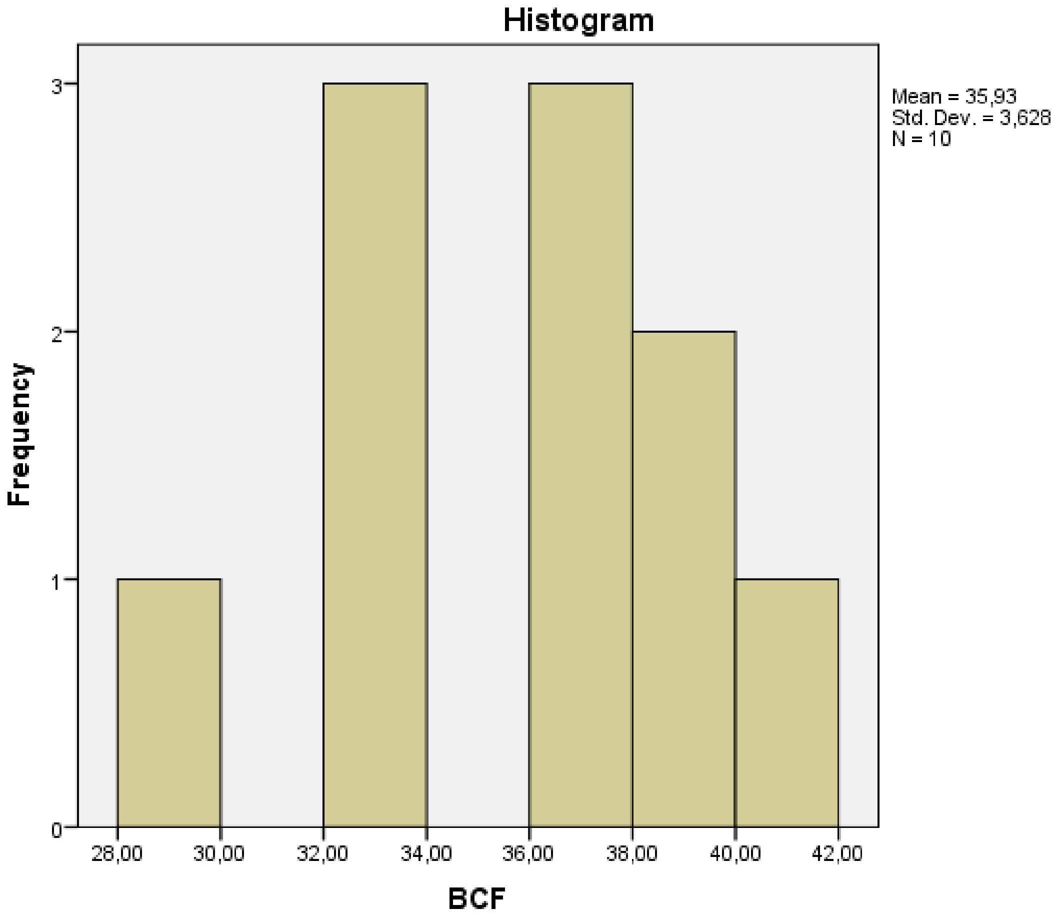
Histogram
Mean = 35,93
Std. Dev. = 3,628
N = 10
Frequency
3
2
1
0
28,00
30,00
32,00
34,00
36,00
38,00
40,00
42,00
BCF

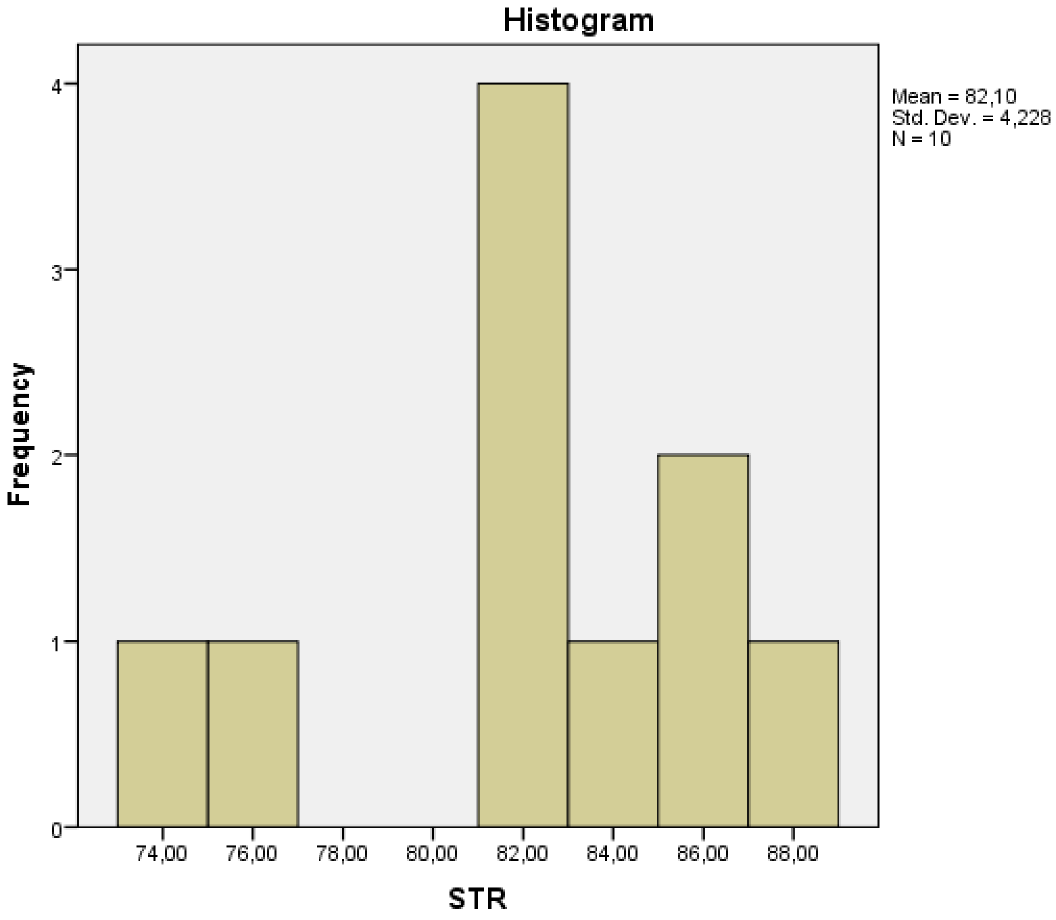
Histogram
Mean = 82,10
Std. Dev. = 4,228
N = 10
Frequency
0
1
2
3
4
74,00
76,00
78,00
80,00
82,00
84,00
86,00
88,00
STR

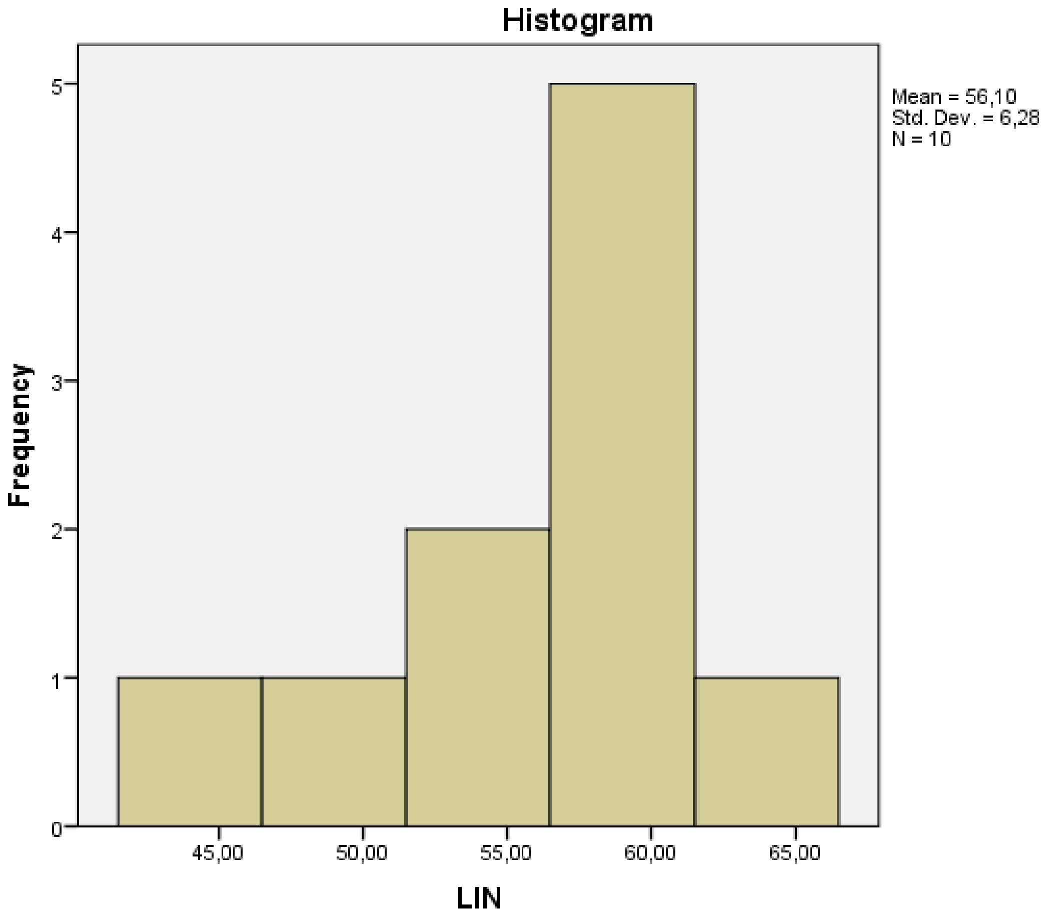

Stem-and-Leaf Plots

```
VAP Stem-and-Leaf Plot for
Grup= 1,00

 Frequency    Stem &  Leaf

     1,00        9 .  4
     1,00       10 .  5
     2,00       11 .  37
     3,00       12 .  136
     2,00       13 .  06
     1,00 Extremes    (>=155)

 Stem width:  10,00
 Each leaf:       1 case(s)
```

```
VSL Stem-and-Leaf Plot for
Grup= 1,00

 Frequency    Stem &  Leaf

     1,00        7 .  9
     1,00        8 .  8
     3,00        9 .  179
     3,00       10 .  049
     1,00       11 .  4
     1,00 Extremes    (>=136)

 Stem width:  10,00
 Each leaf:       1 case(s)
```

```
VCL Stem-and-Leaf Plot for
Grup= 1,00

 Frequency    Stem &  Leaf

     1,00        1 .  4
     4,00        1 .  5688
     5,00        2 .  00123

 Stem width:  100,0
 Each leaf:       1 case(s)
```

```
ALH Stem-and-Leaf Plot for
Grup= 1,00

 Frequency    Stem &  Leaf

     5,00        5 .  77899
     2,00        6 .  79
     2,00        7 .  34
     1,00        8 .  3

 Stem width:   1,00
 Each leaf:       1 case(s)
```

```
BCF Stem-and-Leaf Plot for
Grup= 1,00

 Frequency    Stem &  Leaf
```

```
     1,00        2 .  9
     3,00        3 .  223
     5,00        3 .  67789
     1,00        4 .  0

 Stem width:  10,00
 Each leaf:       1 case(s)
```

```
STR Stem-and-Leaf Plot for
Grup= 1,00

 Frequency    Stem &  Leaf

     1,00 Extremes    (=<74)
     1,00        7 .  6
     5,00        8 .  22224
     3,00        8 .  667

 Stem width:  10,00
 Each leaf:       1 case(s)
```

```
LIN Stem-and-Leaf Plot for
Grup= 1,00

 Frequency    Stem &  Leaf

     1,00        4 .  4
     1,00        4 .  7
     1,00        5 .  3
     4,00        5 .  6899
     3,00        6 .  113

 Stem width:  10,00
 Each leaf:       1 case(s)
```

Normal Q-Q Plots

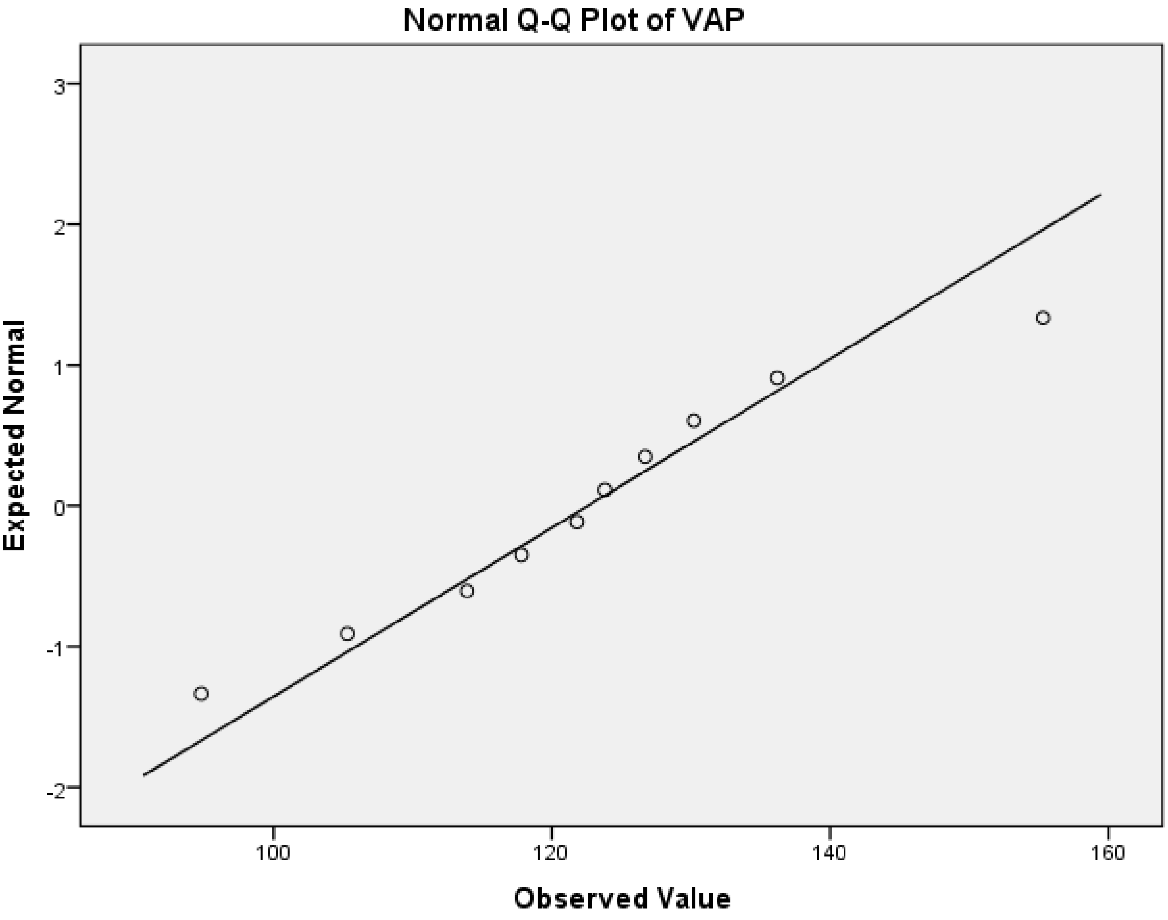
Normal Q-Q Plot of VAP
Expected Normal
Observed Value
3
2
1
0
-1
-2
100
120
140
160

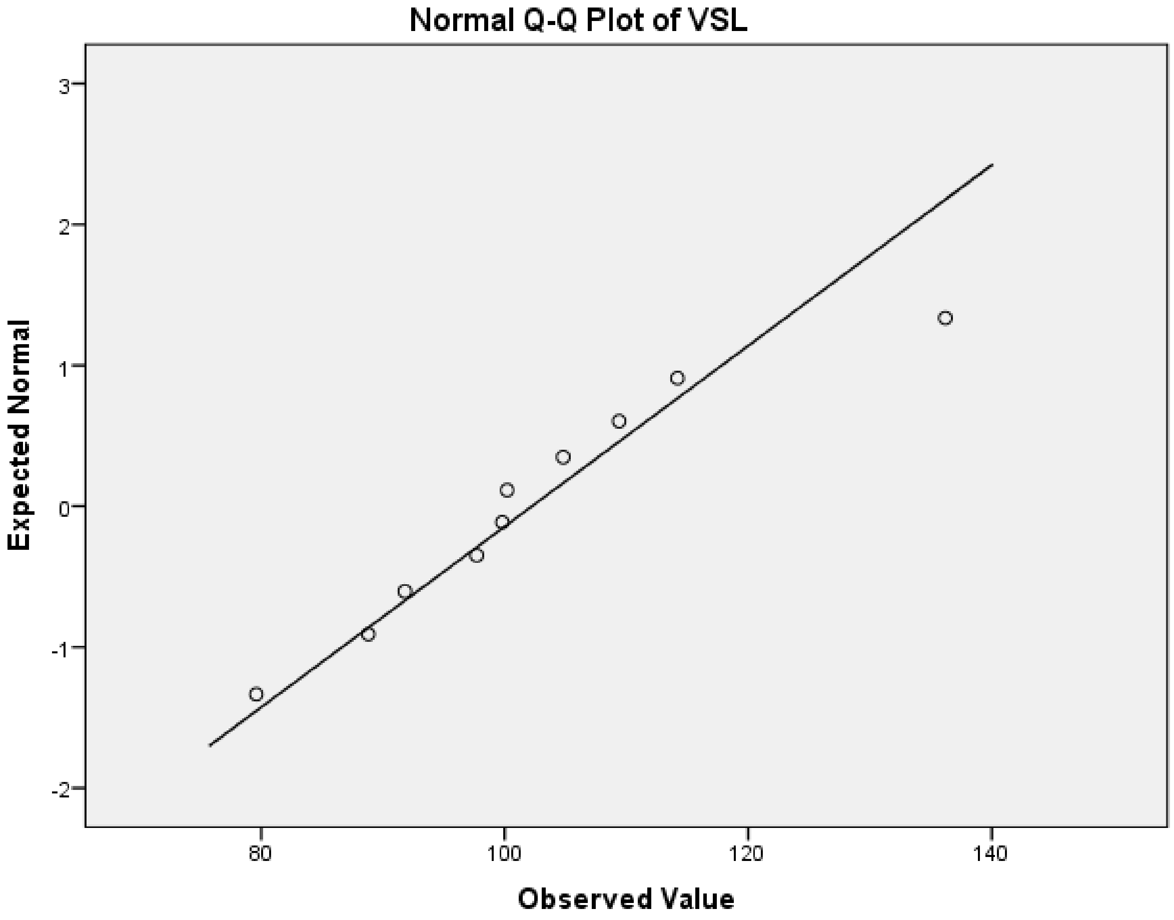
Normal Q-Q Plot of VSL
Expected Normal
Observed Value
3
2
1
0
-1
-2
80
100
120
140

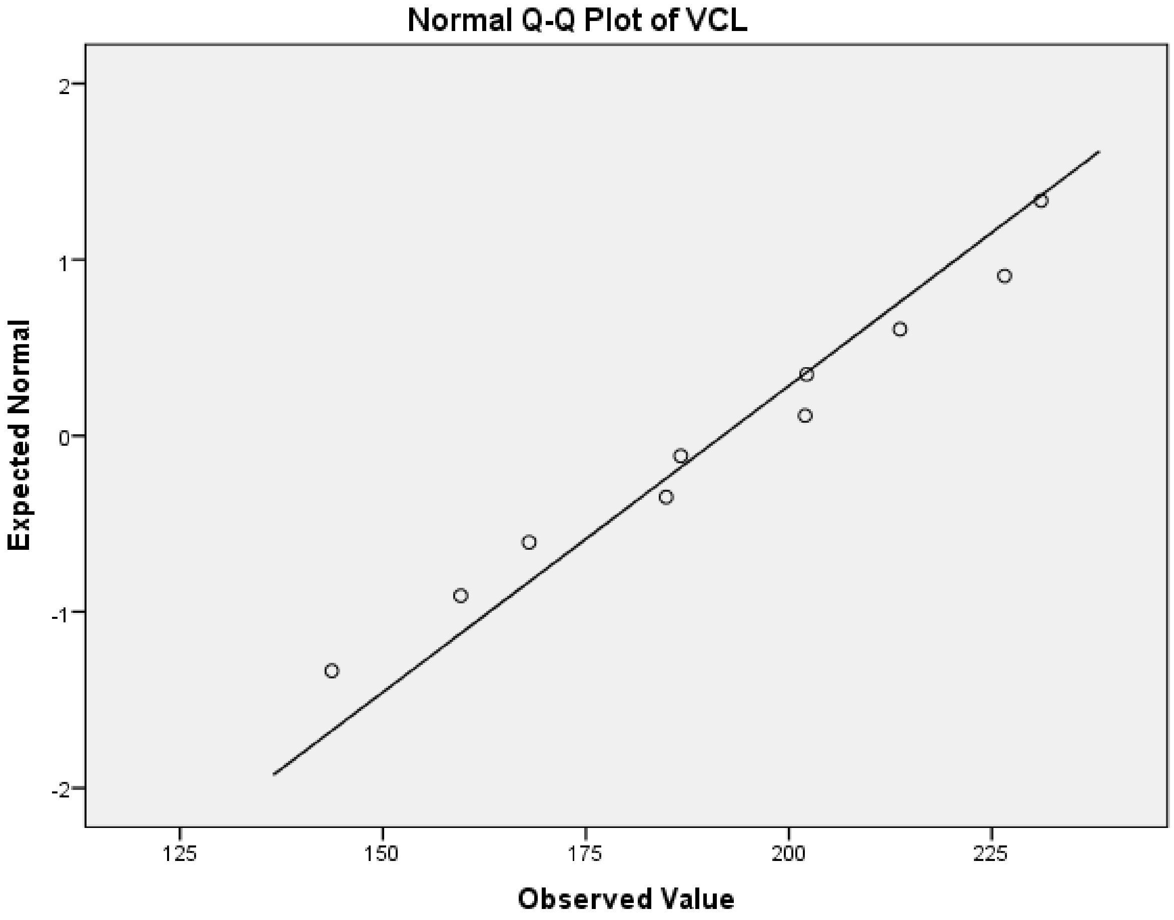
Normal Q-Q Plot of VCL
Expected Normal
2
1
0
-1
-2
125
150
175
200
225
Observed Value

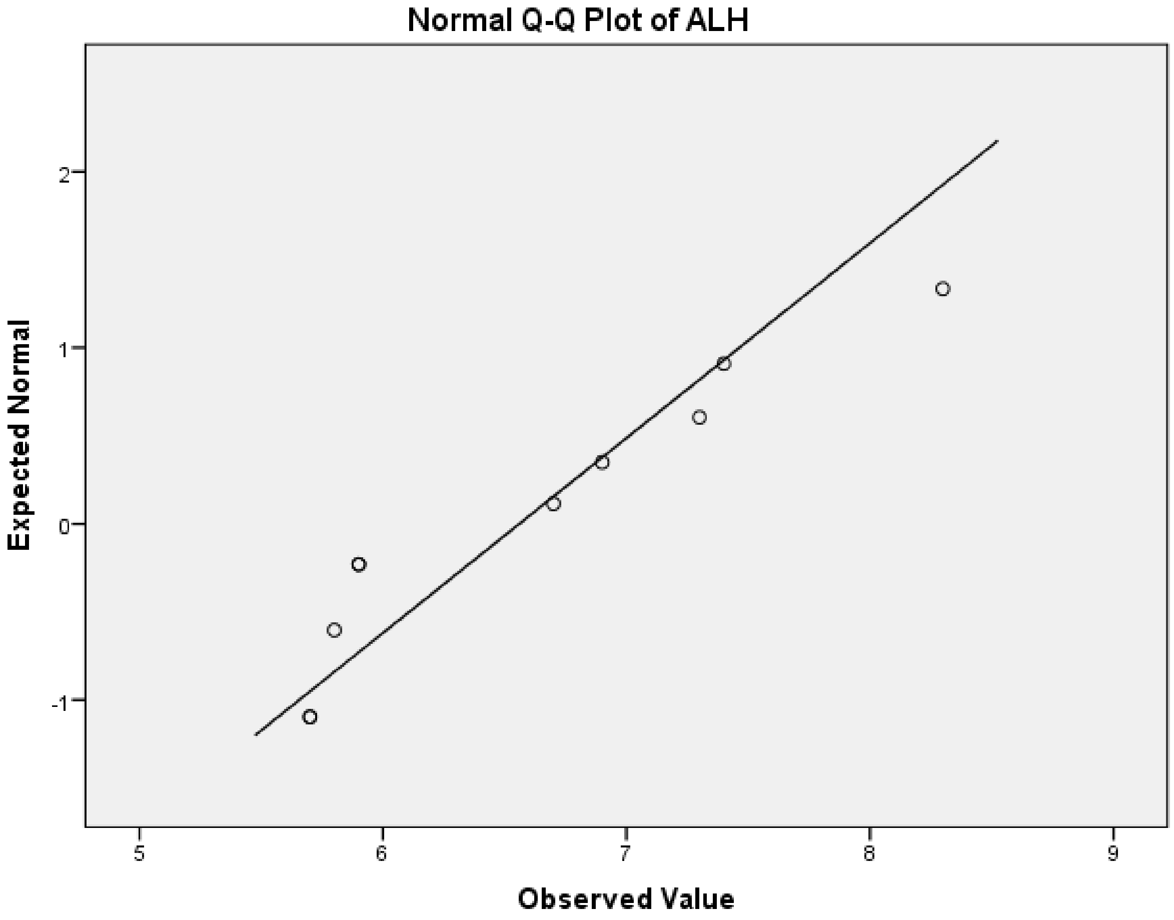
Normal Q-Q Plot of ALH
Expected Normal
2
1
0
-1
5
6
7
8
9
Observed Value

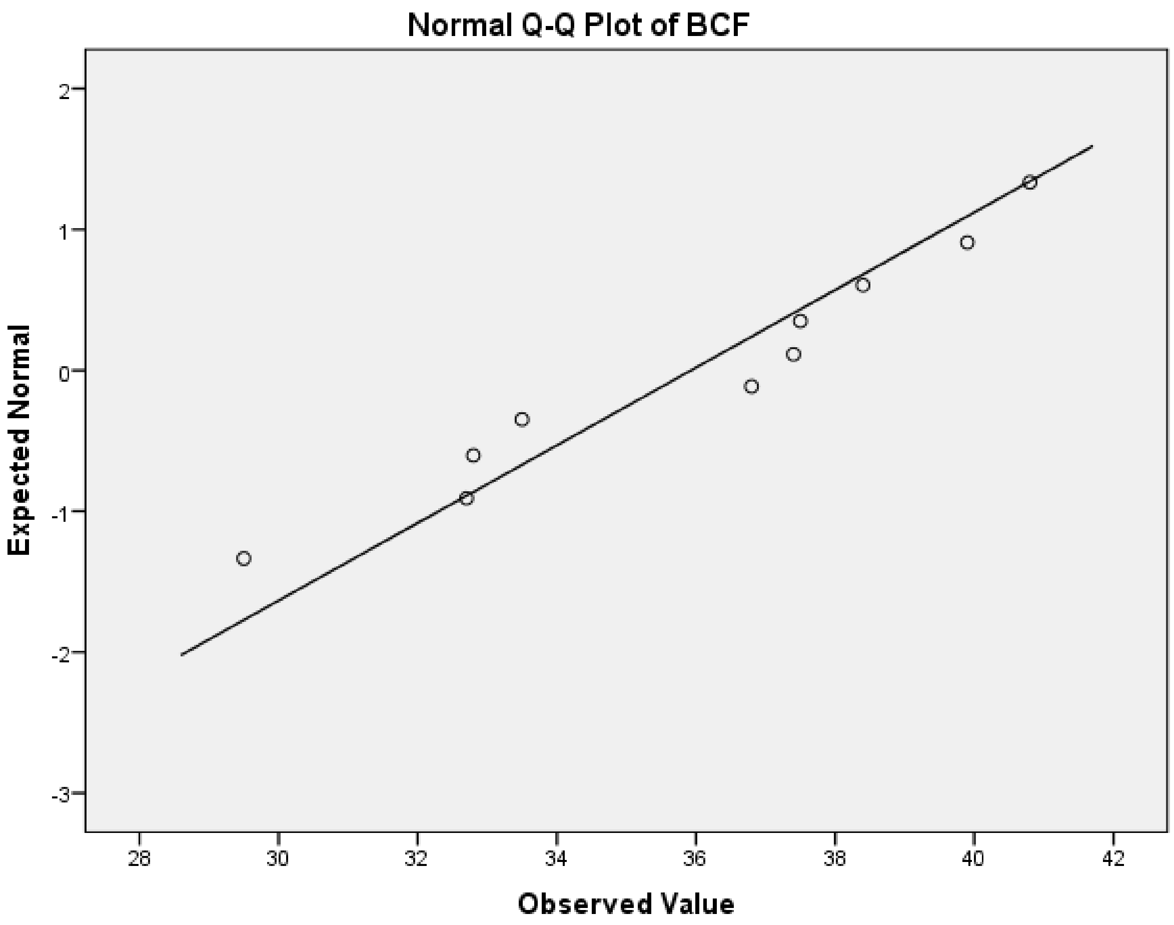
Normal Q-Q Plot of BCF
Expected Normal
Observed Value
2
1
0
-1
-2
-3
28
30
32
34
36
38
40
42

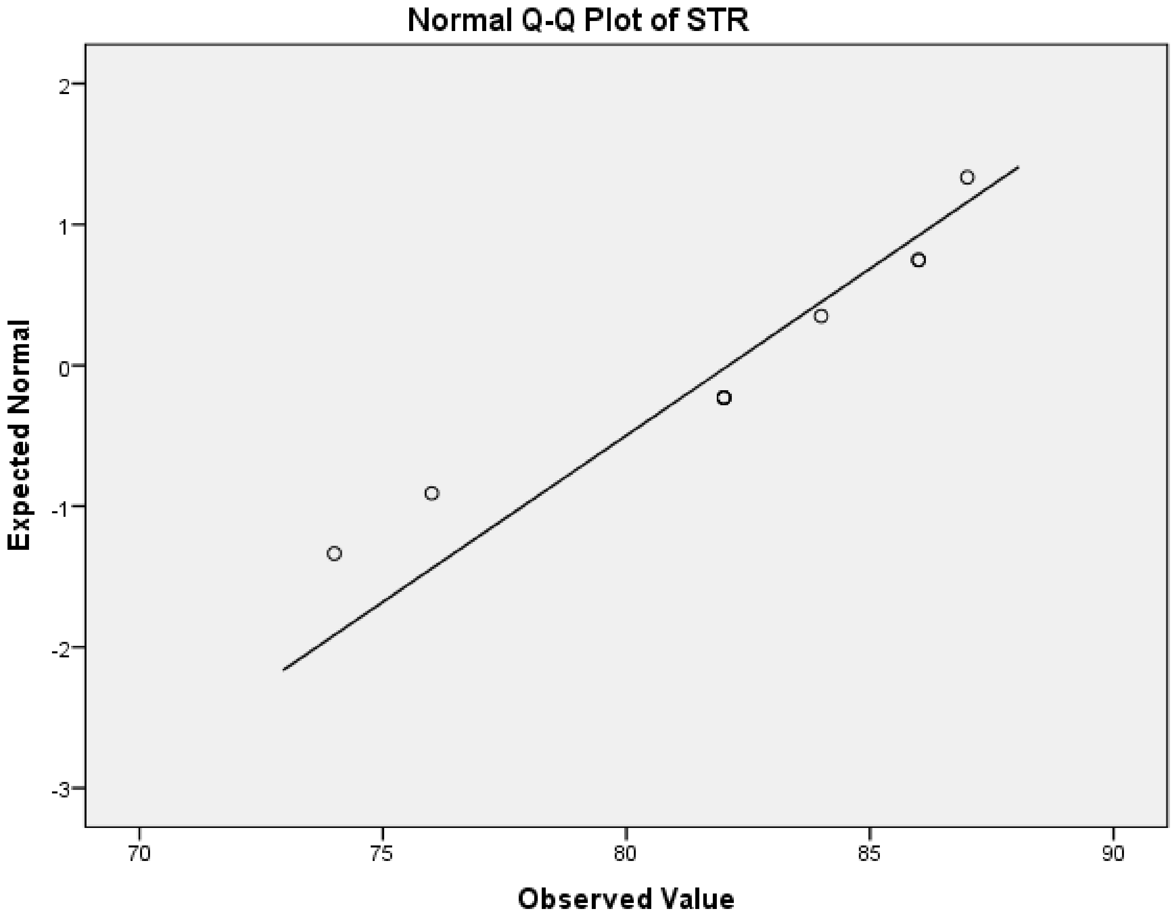

Normal Q-Q Plot of STR
Expected Normal
Observed Value
2
1
0
-1
-2
-3
70
75
80
85
90

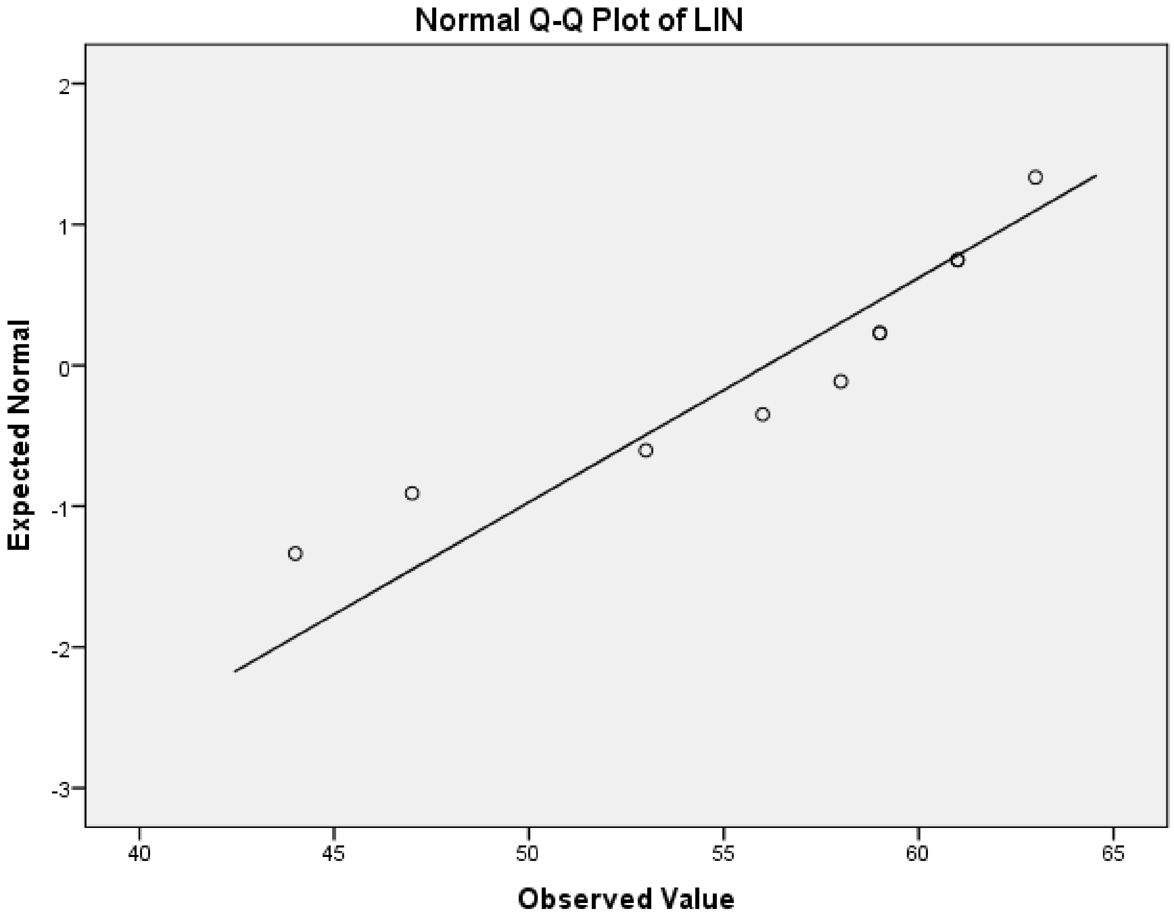

Detrended Normal Q-Q Plots

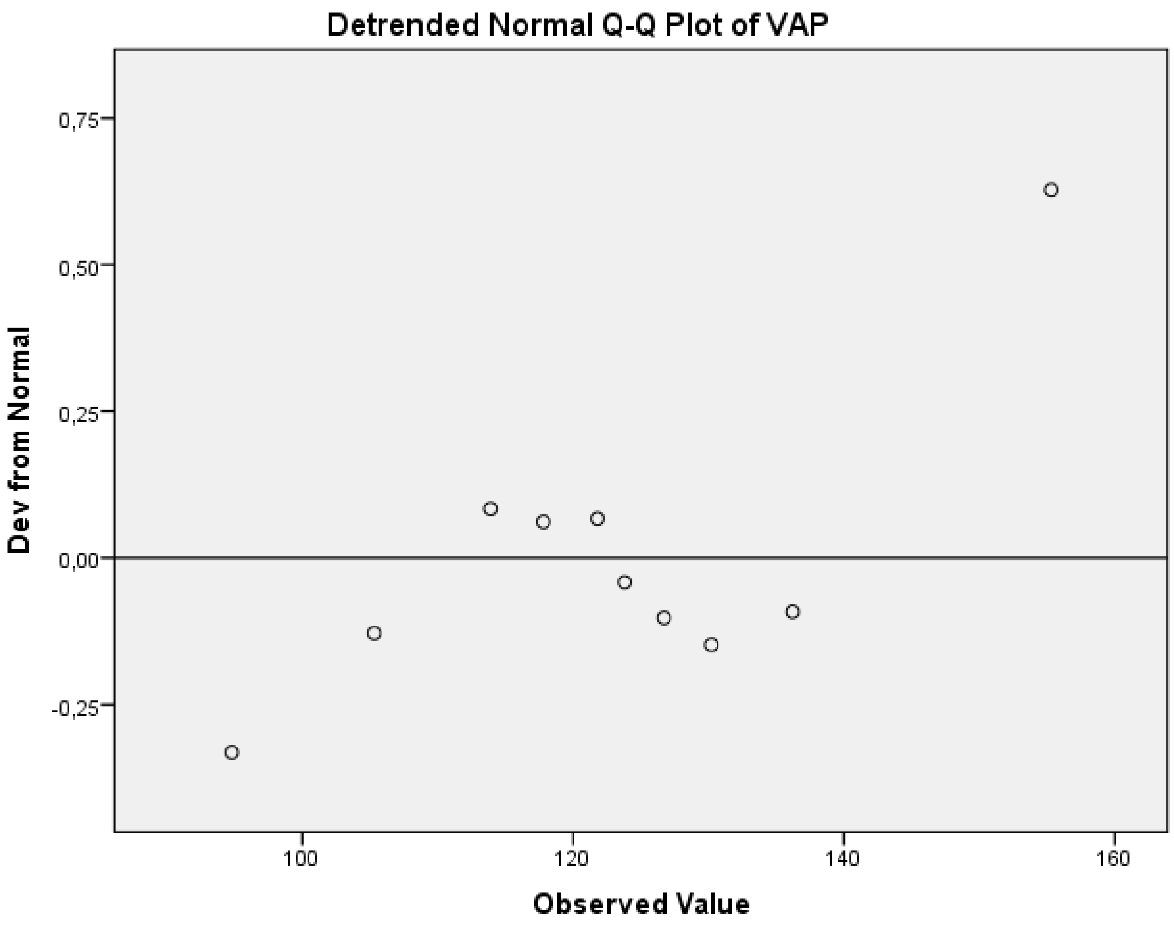
Detrended Normal Q-Q Plot of VAP
Dev from Normal
0,75
0,50
0,25
0,00
-0,25
100
120
140
160
Observed Value

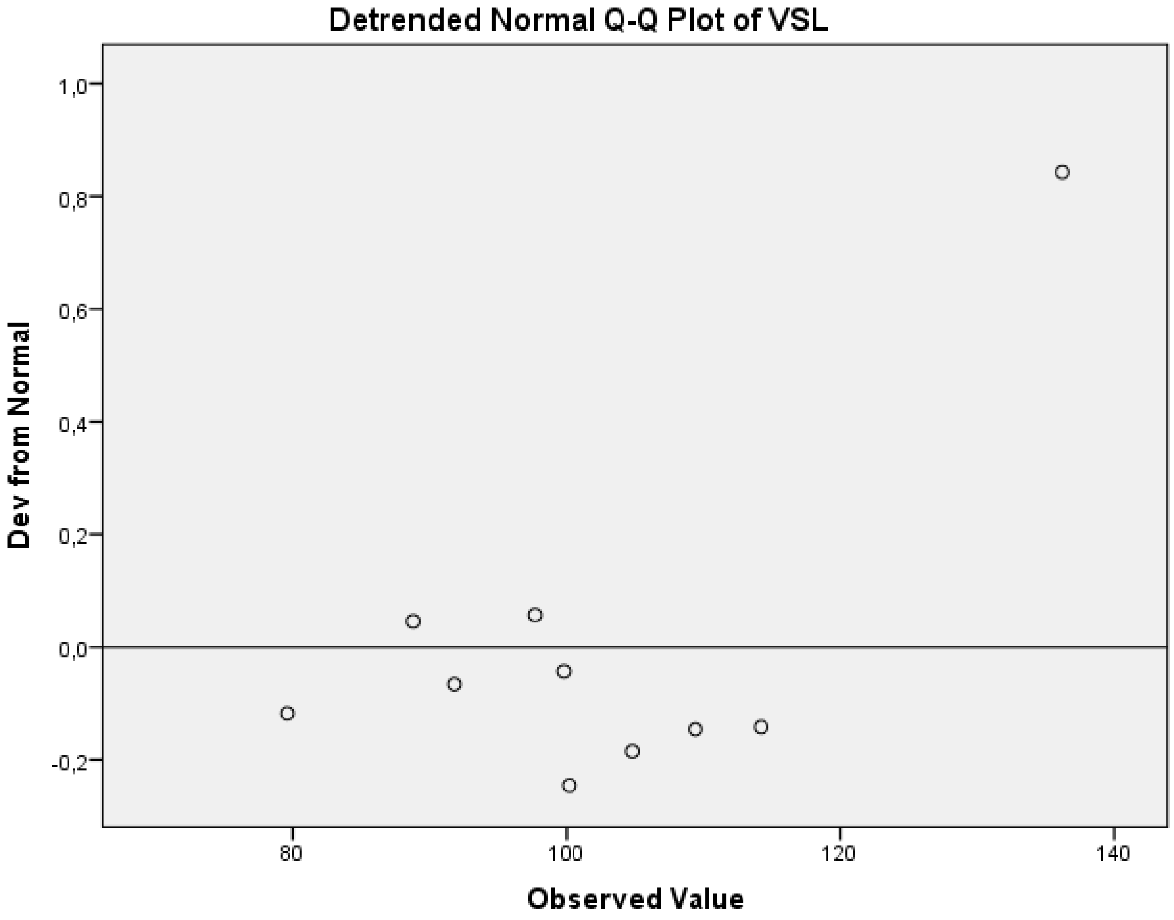
Detrended Normal Q-Q Plot of VSL
Dev from Normal
1,0
0,8
0,6
0,4
0,2
0,0
-0,2
80
100
120
140
Observed Value

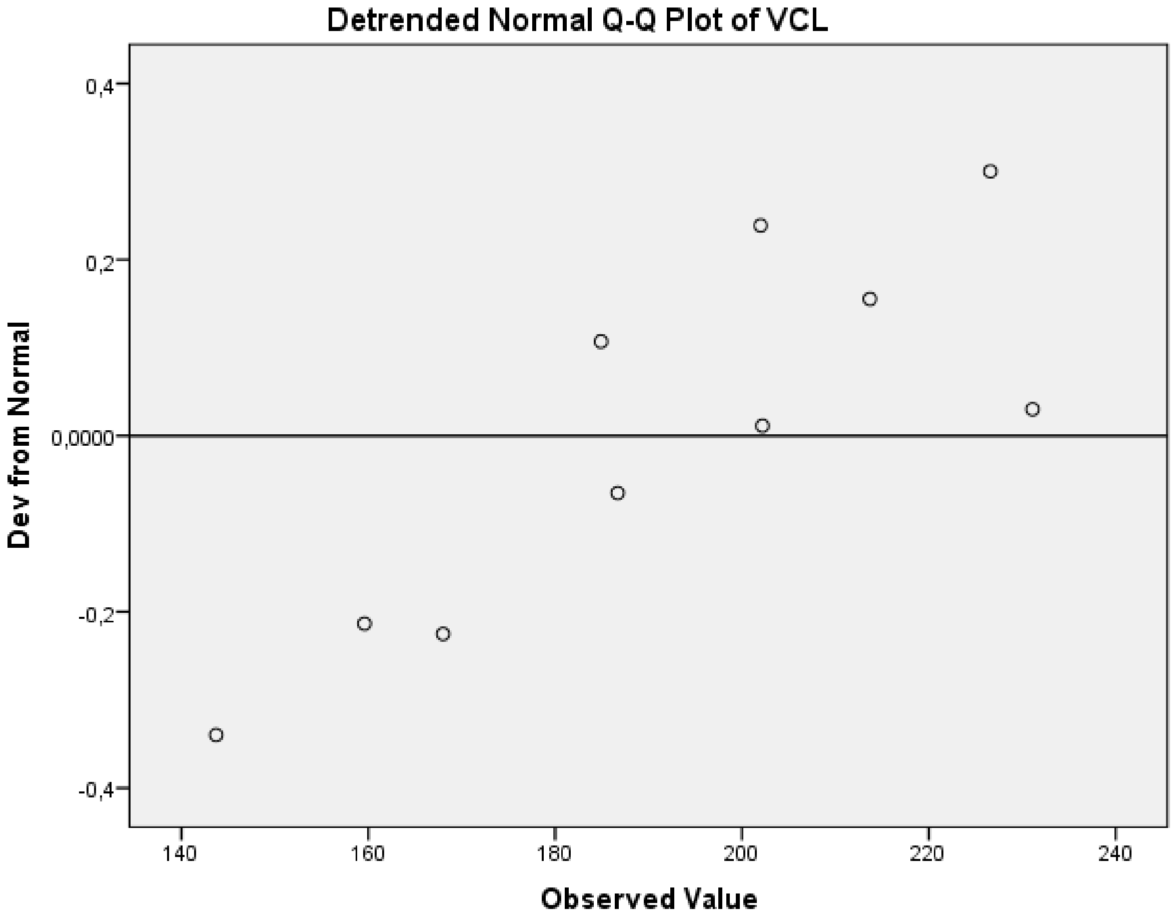

Detrended Normal Q-Q Plot of VCL
Dev from Normal
0,4
0,2
0,0000
-0,2
-0,4
140
160
180
200
220
240
Observed Value

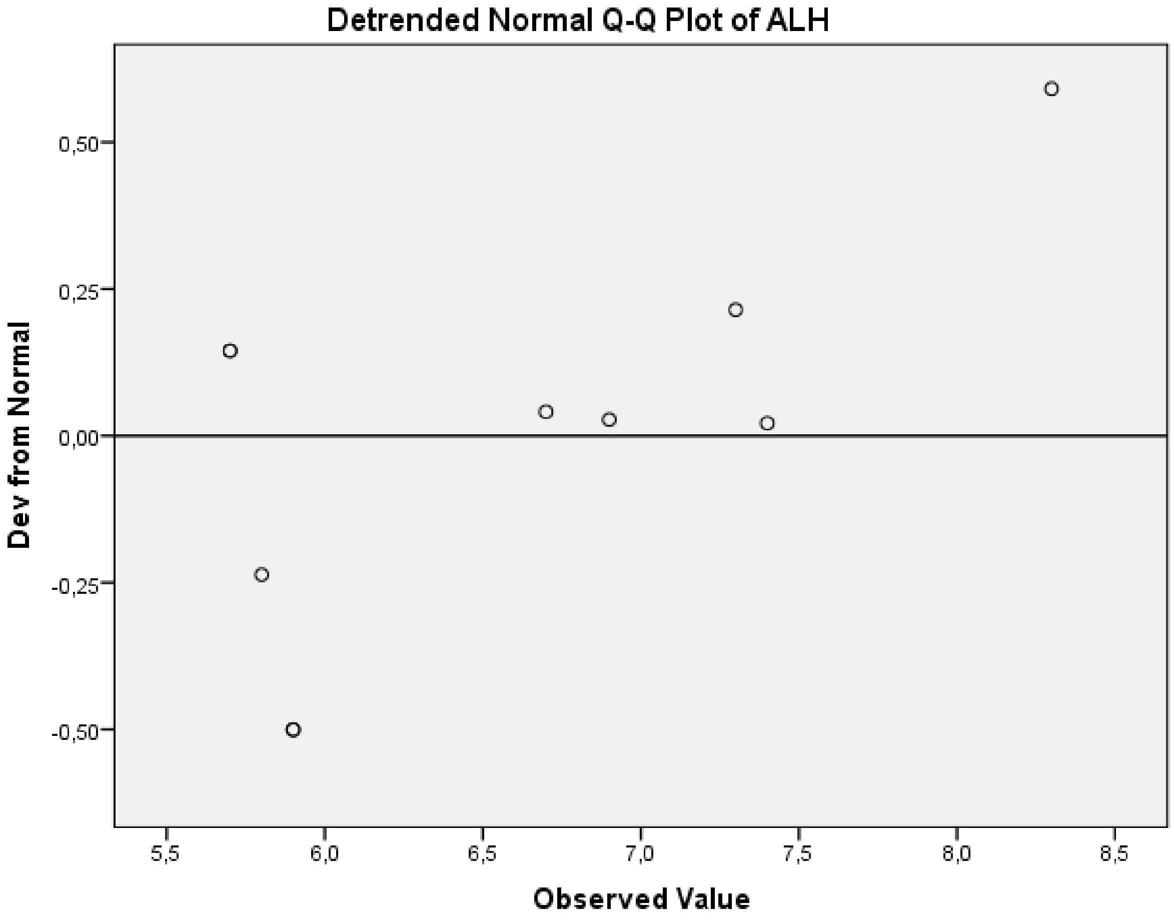
Detrended Normal Q-Q Plot of ALH
Dev from Normal
0,50
0,25
0,00
-0,25
-0,50
5,5
6,0
6,5
7,0
7,5
8,0
8,5
Observed Value

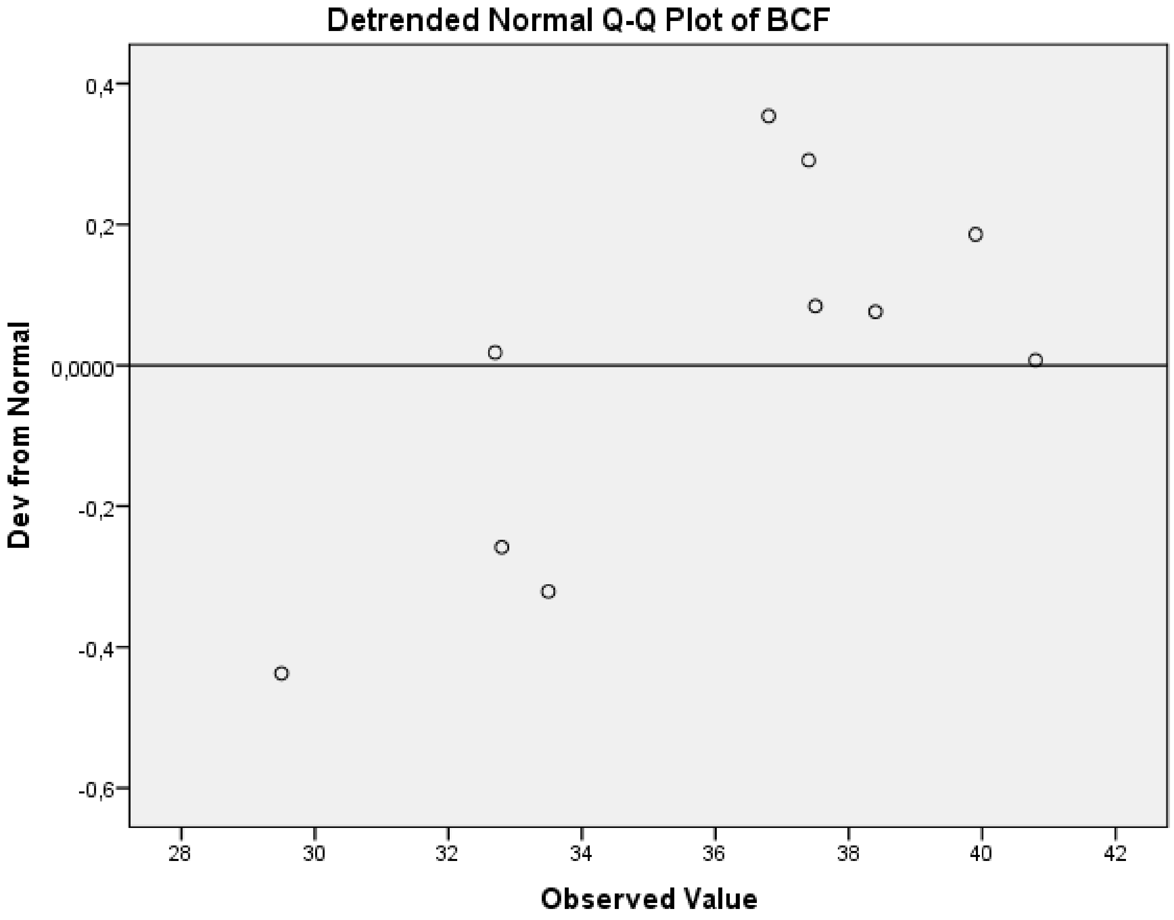

Detrended Normal Q-Q Plot of BCF
Dev from Normal
0,4
0,2
0,0000
-0,2
-0,4
-0,6
28
30
32
34
36
38
40
42
Observed Value

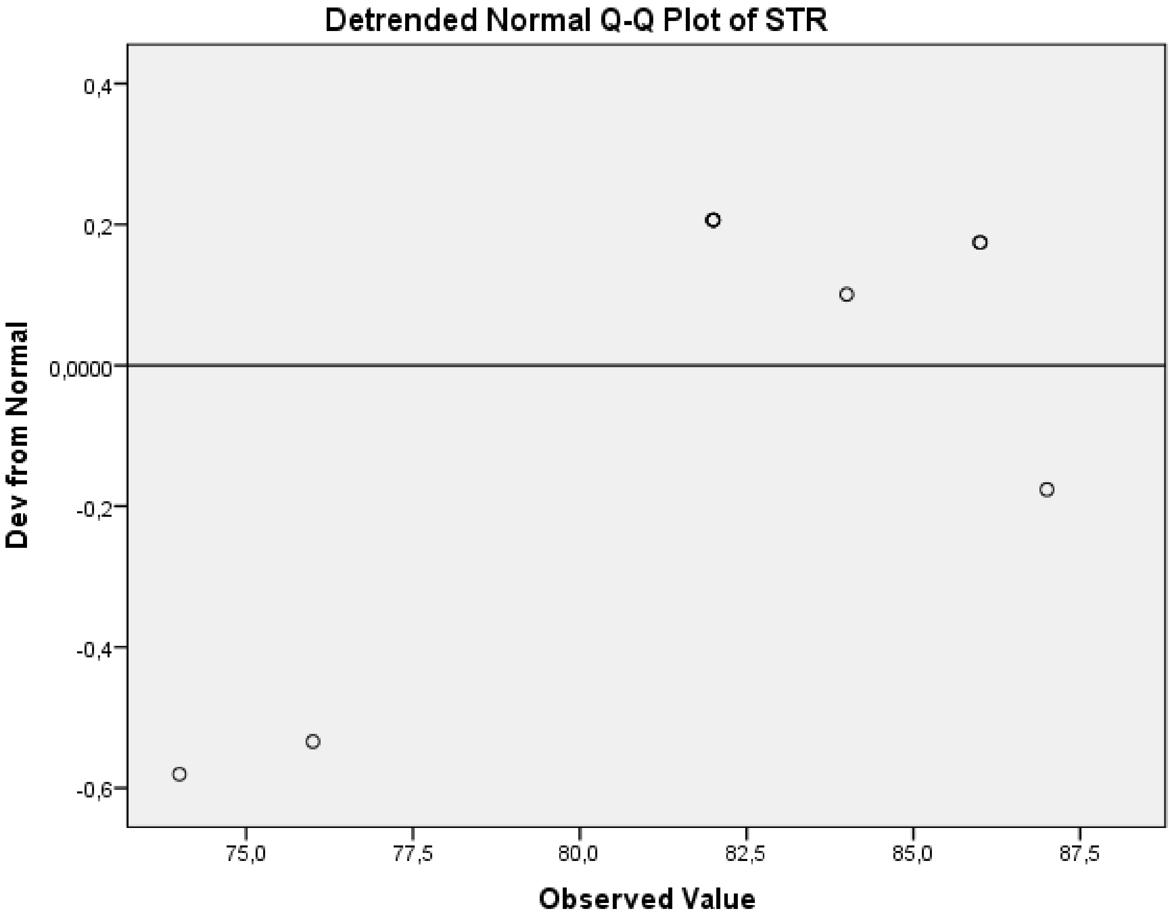
Detrended Normal Q-Q Plot of STR
Dev from Normal
Observed Value
0,4
0,2
0,0000
-0,2
-0,4
-0,6
75,0
77,5
80,0
82,5
85,0
87,5

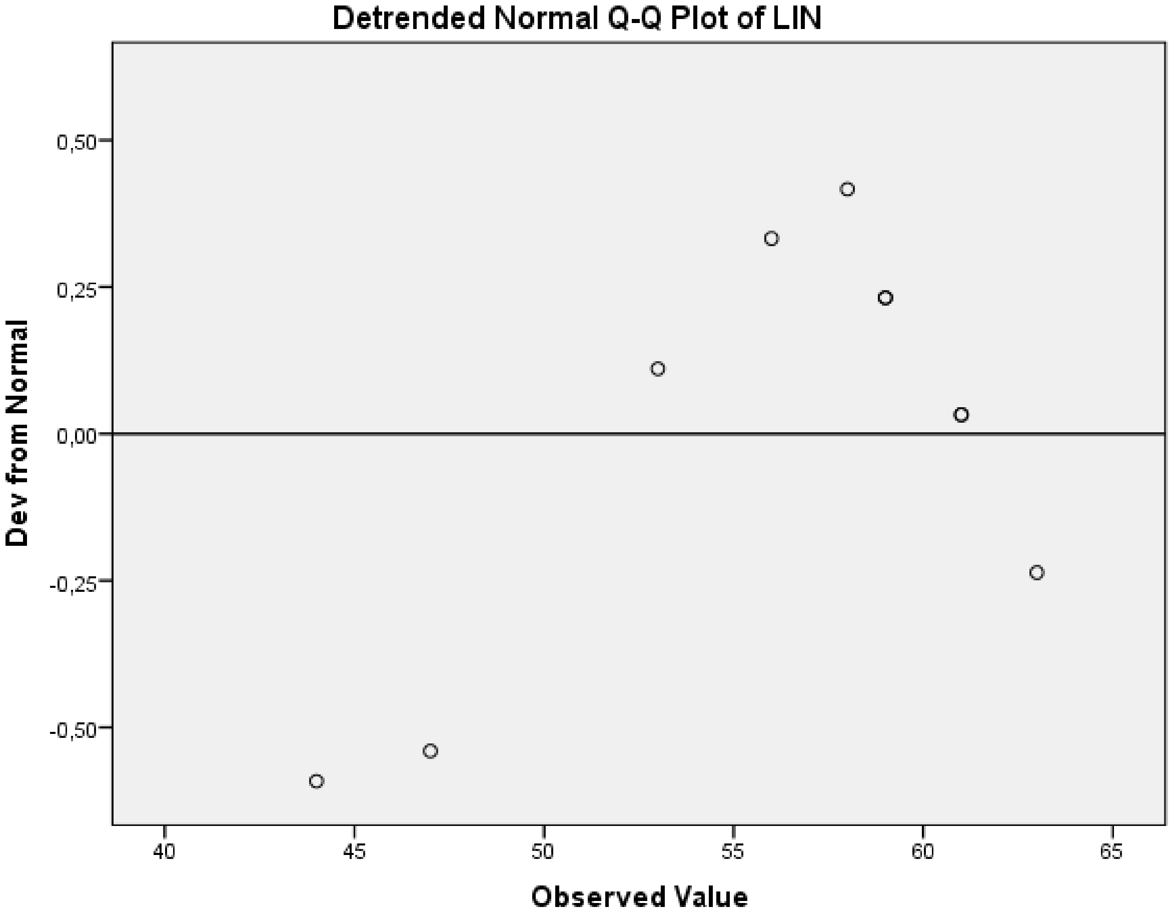

Grup = 2,00

Histograms

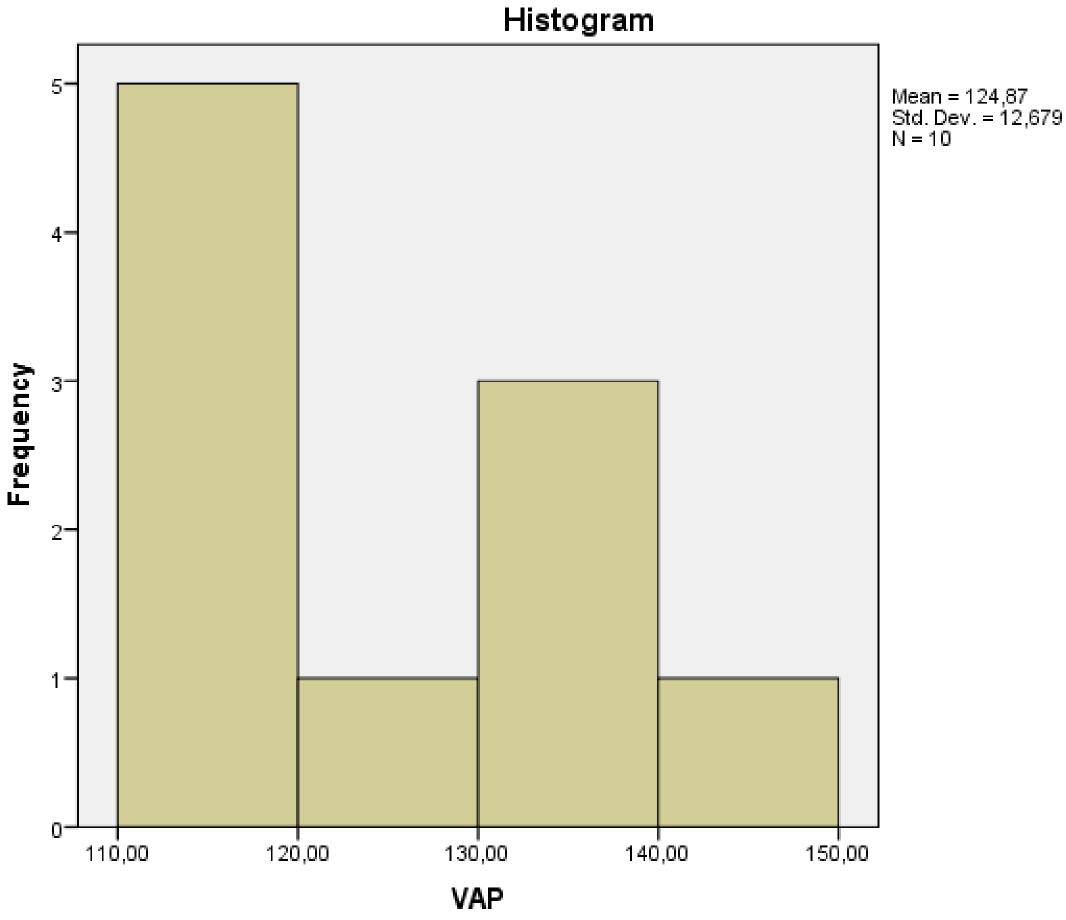
Histogram
Mean = 124,87
Std. Dev. = 12,679
N = 10
Frequency
0
1
2
3
4
5
110,00
120,00
130,00
140,00
150,00
VAP

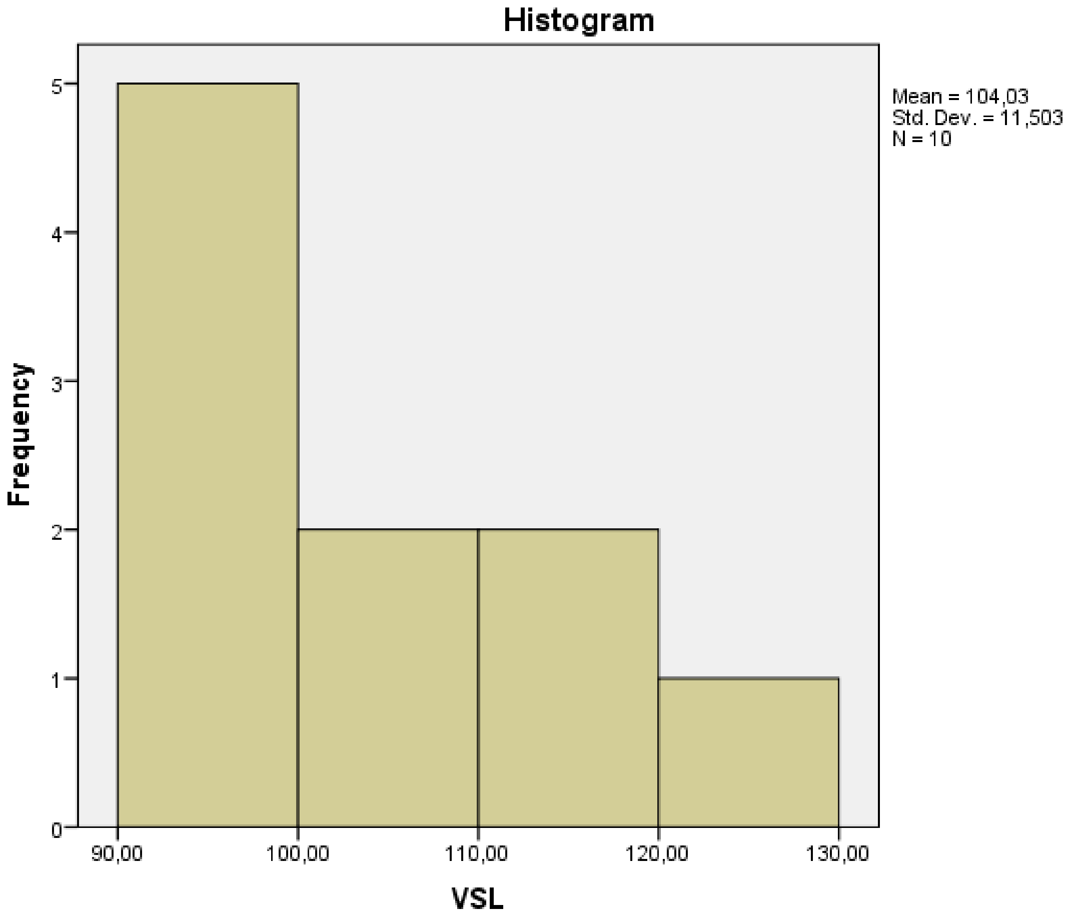

Histogram
Mean = 104,03
Std. Dev. = 11,503
N = 10
Frequency
0
1
2
3
4
5
90,00
100,00
110,00
120,00
130,00
VSL

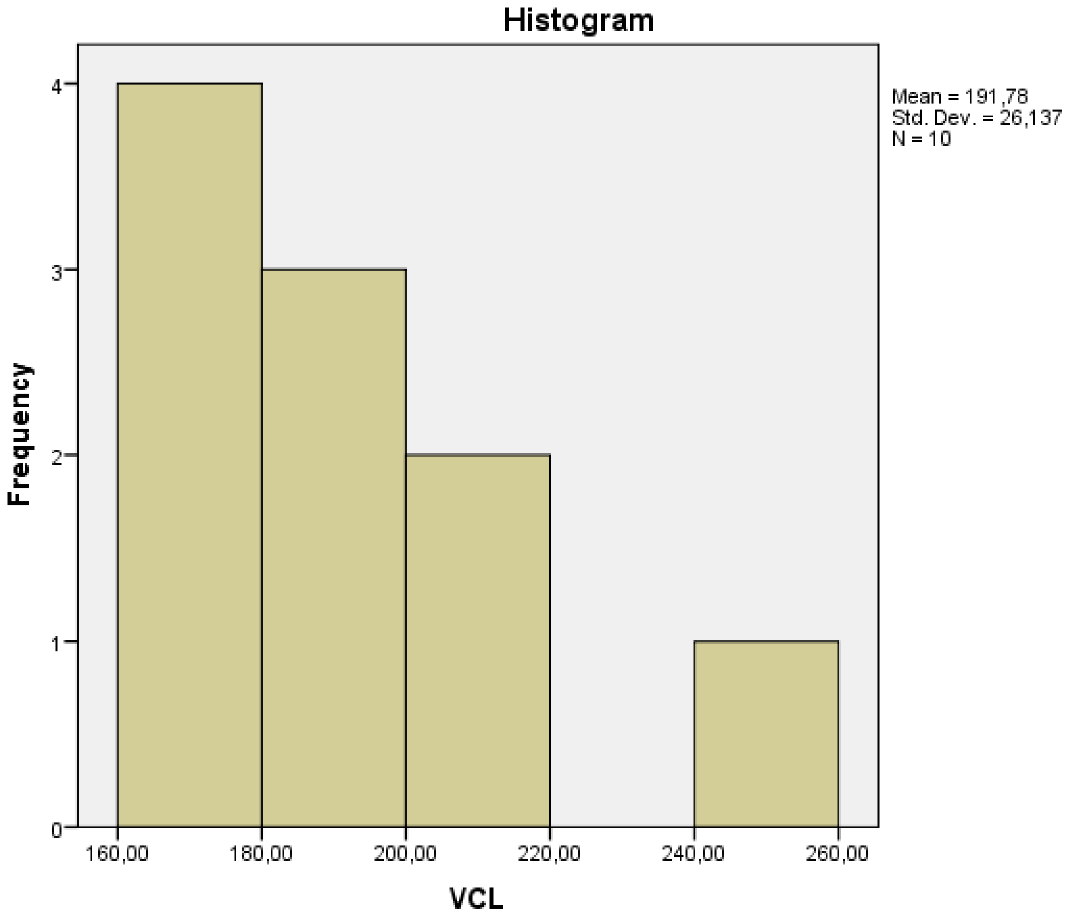

Histogram
Mean = 191,78
Std. Dev. = 26,137
N = 10
Frequency
0
1
2
3
4
160,00
180,00
200,00
220,00
240,00
260,00
VCL

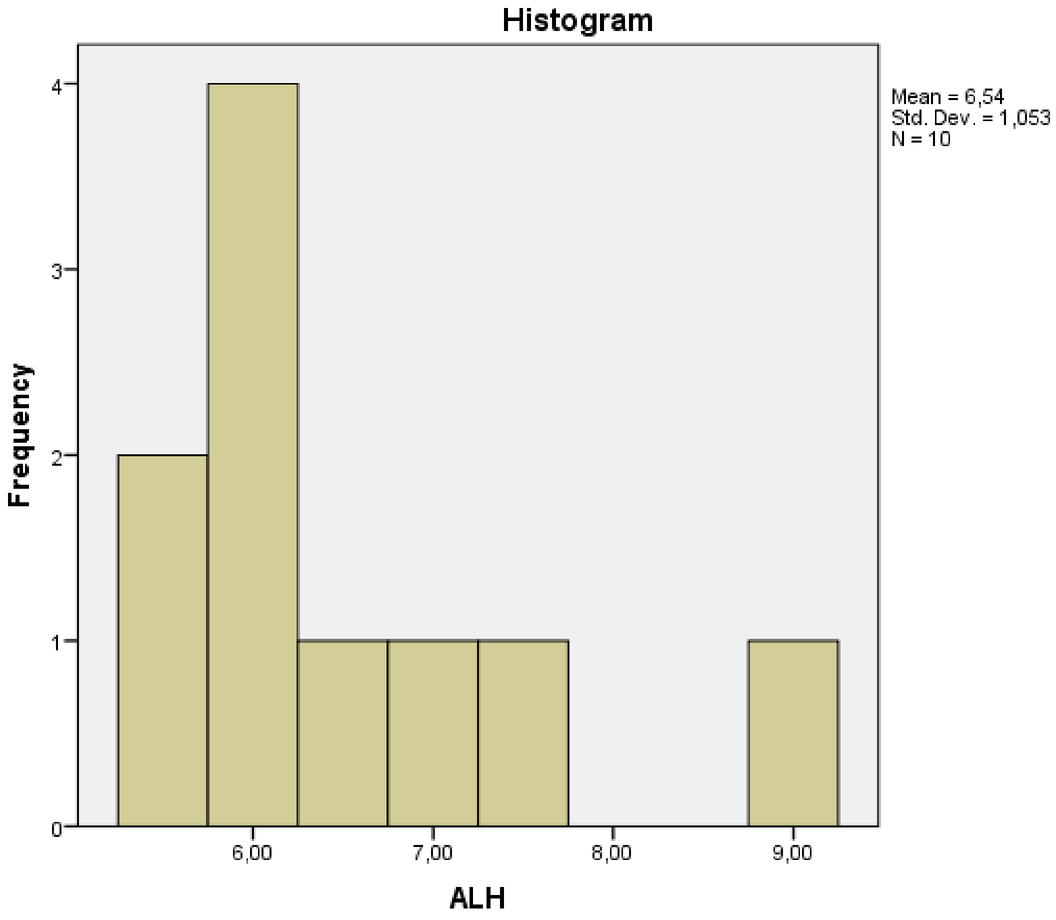
Histogram
Mean = 6,54
Std. Dev. = 1,053
N = 10
Frequency
0
1
2
3
4
6,00
7,00
8,00
9,00
ALH

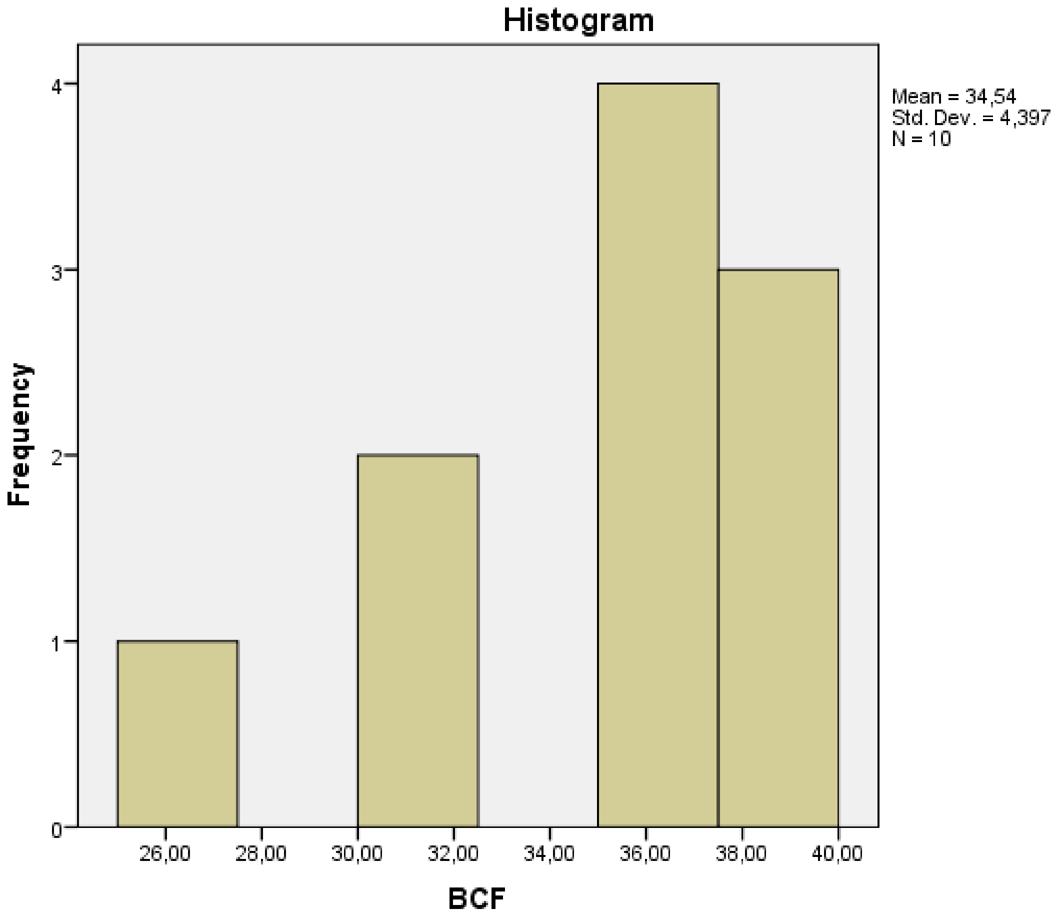
Histogram
Mean = 34,54
Std. Dev. = 4,397
N = 10
Frequency
0
1
2
3
4
26,00
28,00
30,00
32,00
34,00
36,00
38,00
40,00
BCF

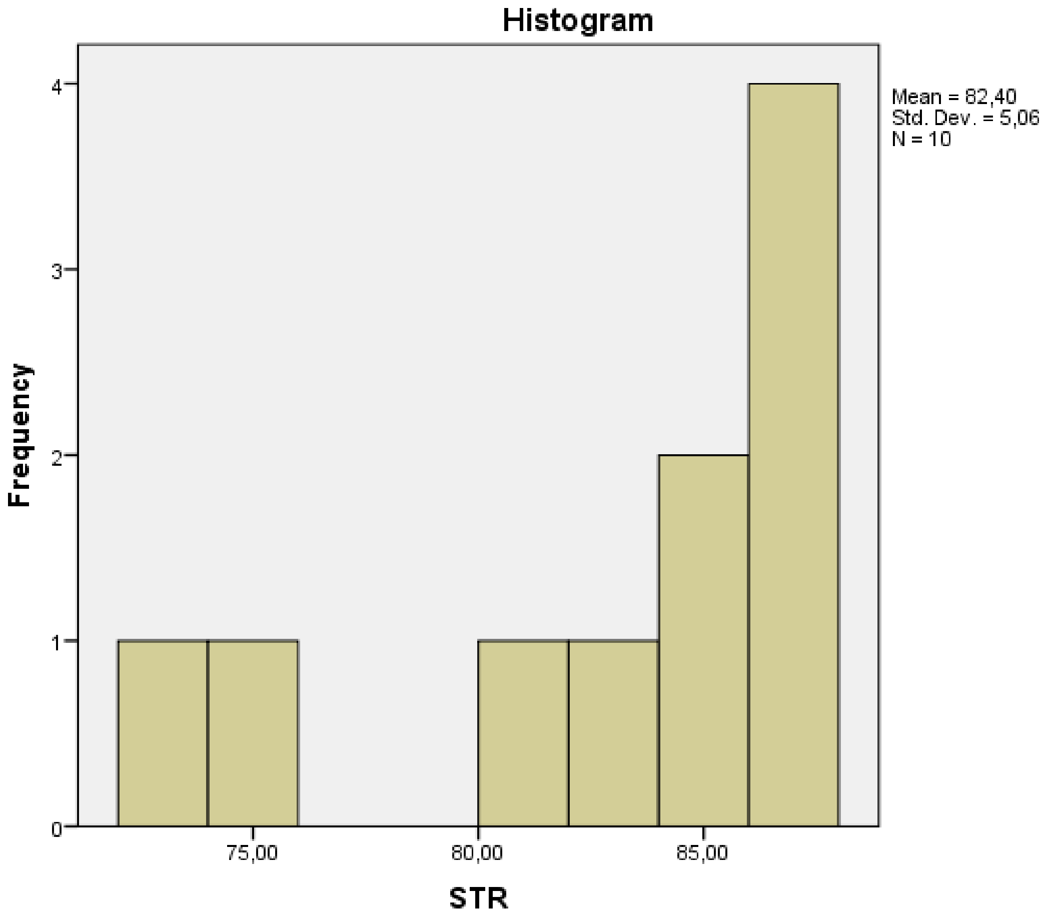
Histogram
Mean = 82,40
Std. Dev. = 5,06
N = 10
Frequency
0
1
2
3
4
75,00
80,00
85,00
STR

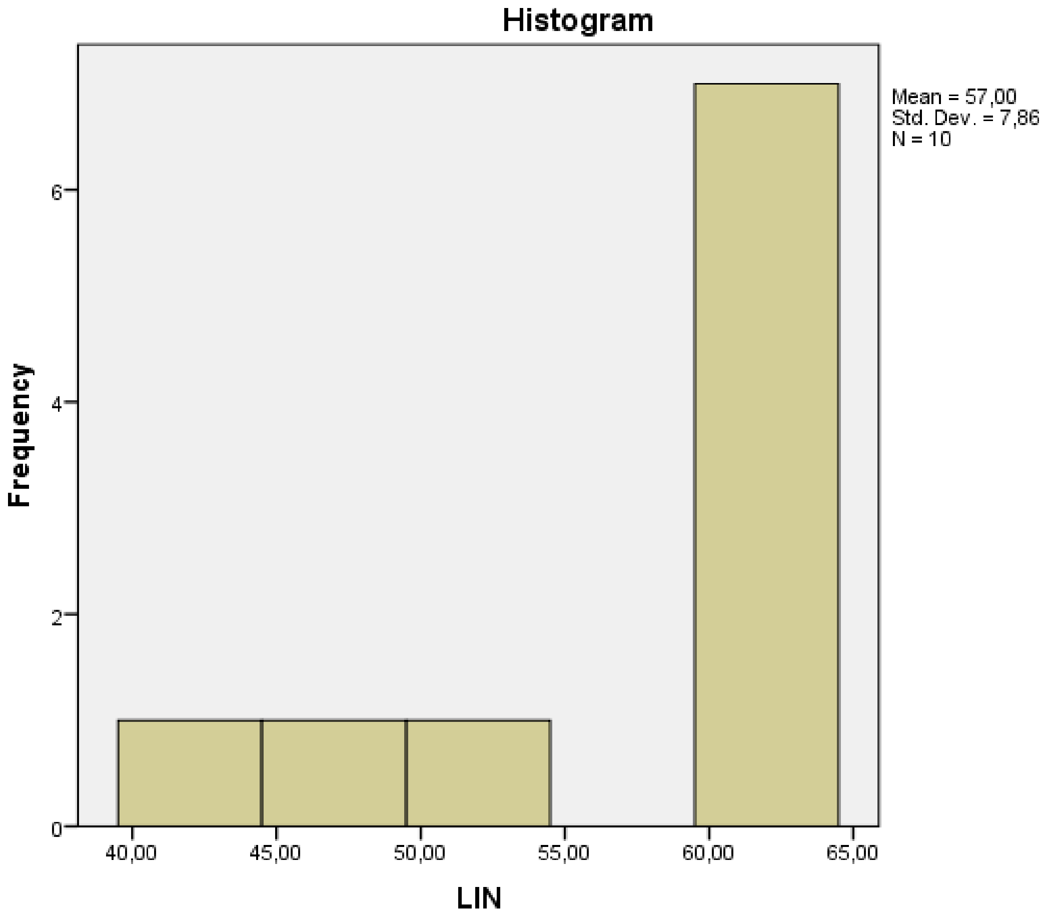

Stem-and-Leaf Plots

```
VAP Stem-and-Leaf Plot for
Grup= 2,00

 Frequency    Stem &  Leaf

     5,00       11 .  02368
     1,00       12 .  5
     3,00       13 .  039
     1,00       14 .  8

 Stem width:  10,00
 Each leaf:       1 case(s)
```

```
VSL Stem-and-Leaf Plot for
Grup= 2,00

 Frequency    Stem &  Leaf

     5,00        9 .  14778
     2,00       10 .  02
     2,00       11 .  47
     1,00       12 .  6

 Stem width:  10,00
 Each leaf:       1 case(s)
```

```
VCL Stem-and-Leaf Plot for
Grup= 2,00

 Frequency    Stem &  Leaf

     7,00        1 .  6667999
     3,00        2 .  114

 Stem width:  100,0
 Each leaf:       1 case(s)
```

```
ALH Stem-and-Leaf Plot for
Grup= 2,00

 Frequency    Stem &  Leaf

     3,00        5 .  579
     4,00        6 .  1123
     2,00        7 .  24
      ,00        8 .
     1,00        9 .  0

 Stem width:   1,00
 Each leaf:       1 case(s)
```

```
BCF Stem-and-Leaf Plot for
Grup= 2,00

 Frequency    Stem &  Leaf

     1,00        2 .  5
     2,00        3 .  00
     7,00        3 .  5557779
```

```
 Stem width:  10,00
 Each leaf:       1 case(s)
```

```
STR Stem-and-Leaf Plot for
Grup= 2,00

 Frequency    Stem &  Leaf

     2,00        7 .  34
      ,00        7 .
     2,00        8 .  03
     6,00        8 .  556666

 Stem width:  10,00
 Each leaf:       1 case(s)
```

```
LIN Stem-and-Leaf Plot for
Grup= 2,00

 Frequency    Stem &  Leaf

     1,00        4 .  2
     1,00        4 .  6
     1,00        5 .  0
      ,00        5 .
     7,00        6 .  0122223

 Stem width:  10,00
 Each leaf:       1 case(s)
```

Normal Q-Q Plots

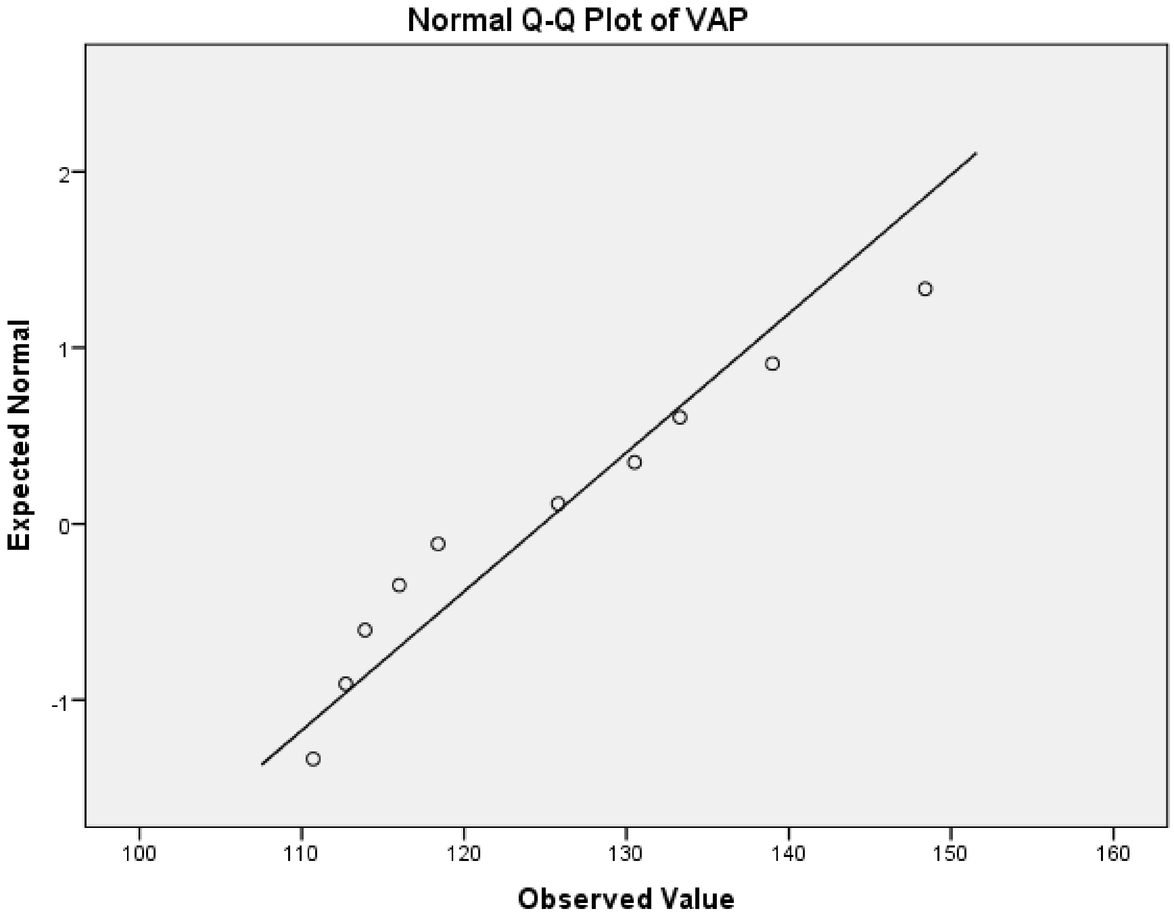
Normal Q-Q Plot of VAP
Expected Normal
Observed Value
2
1
0
-1
100
110
120
130
140
150
160

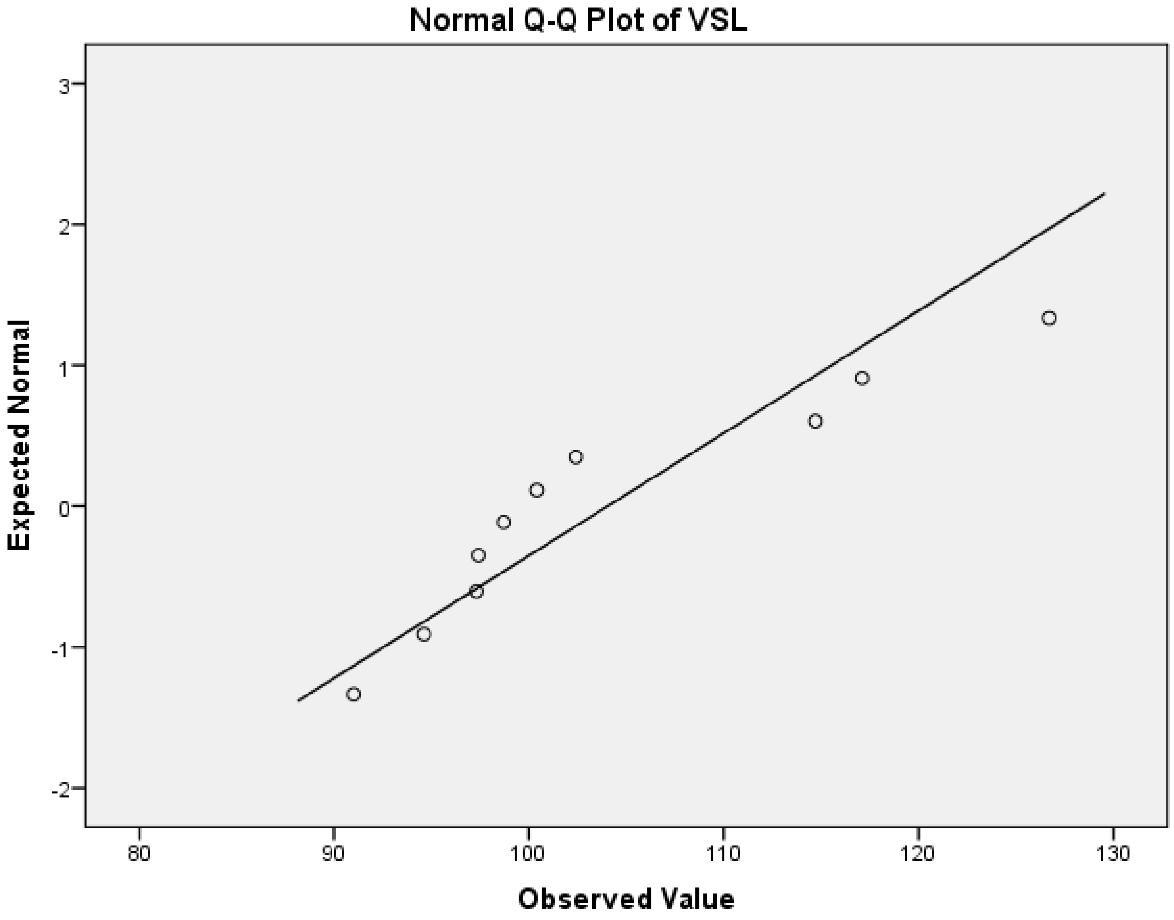
Normal Q-Q Plot of VSL
Expected Normal
Observed Value
3
2
1
0
-1
-2
80
90
100
110
120
130

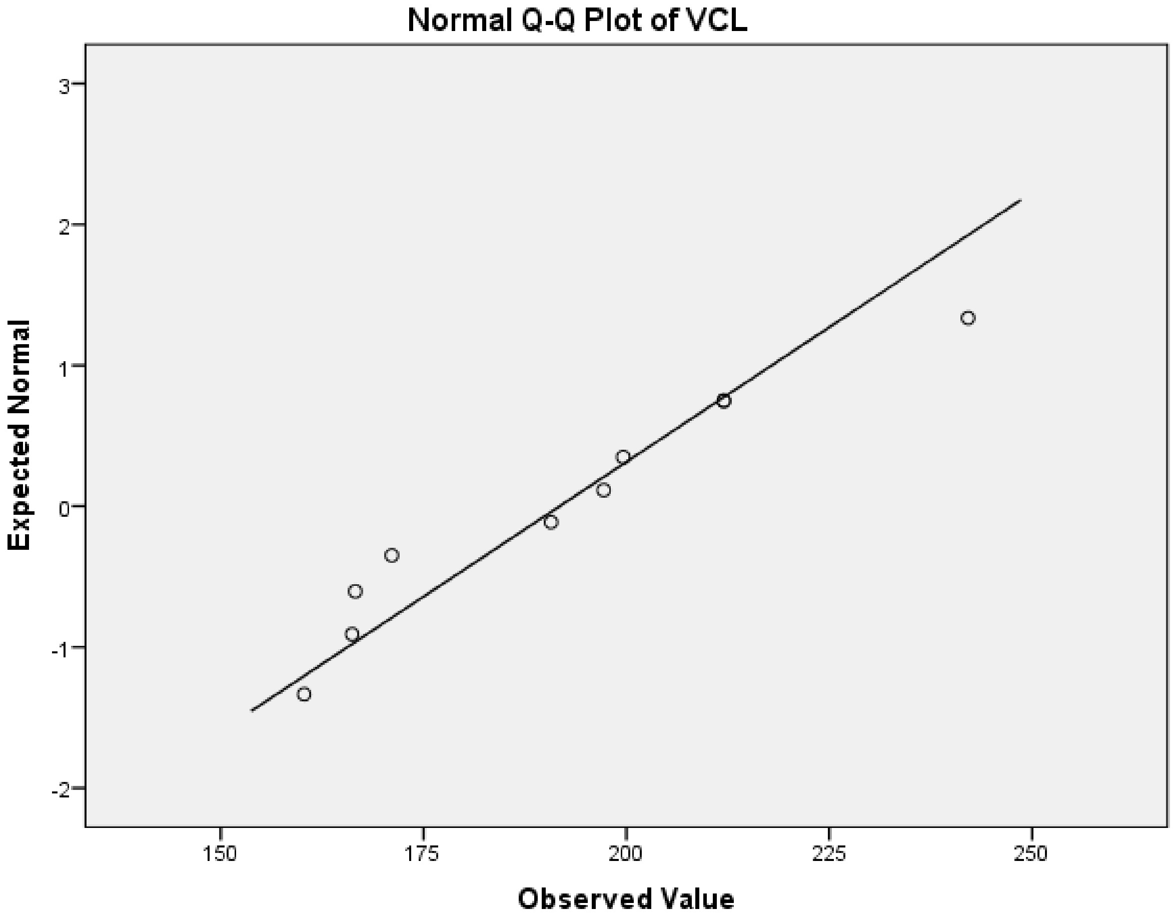

Normal Q-Q Plot of VCL
Expected Normal
Observed Value
3
2
1
0
-1
-2
150
175
200
225
250

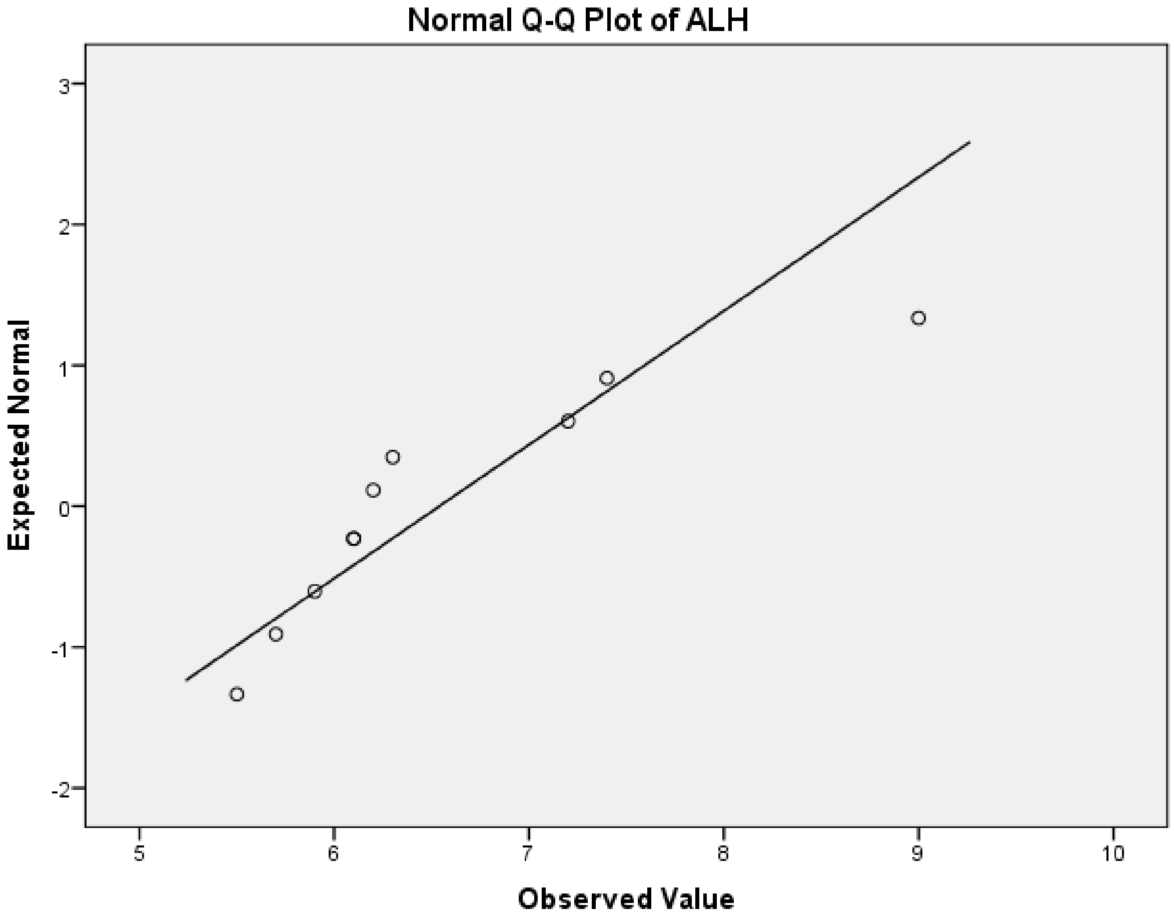

Normal Q-Q Plot of ALH
Expected Normal
Observed Value
3
2
1
0
-1
-2
5
6
7
8
9
10

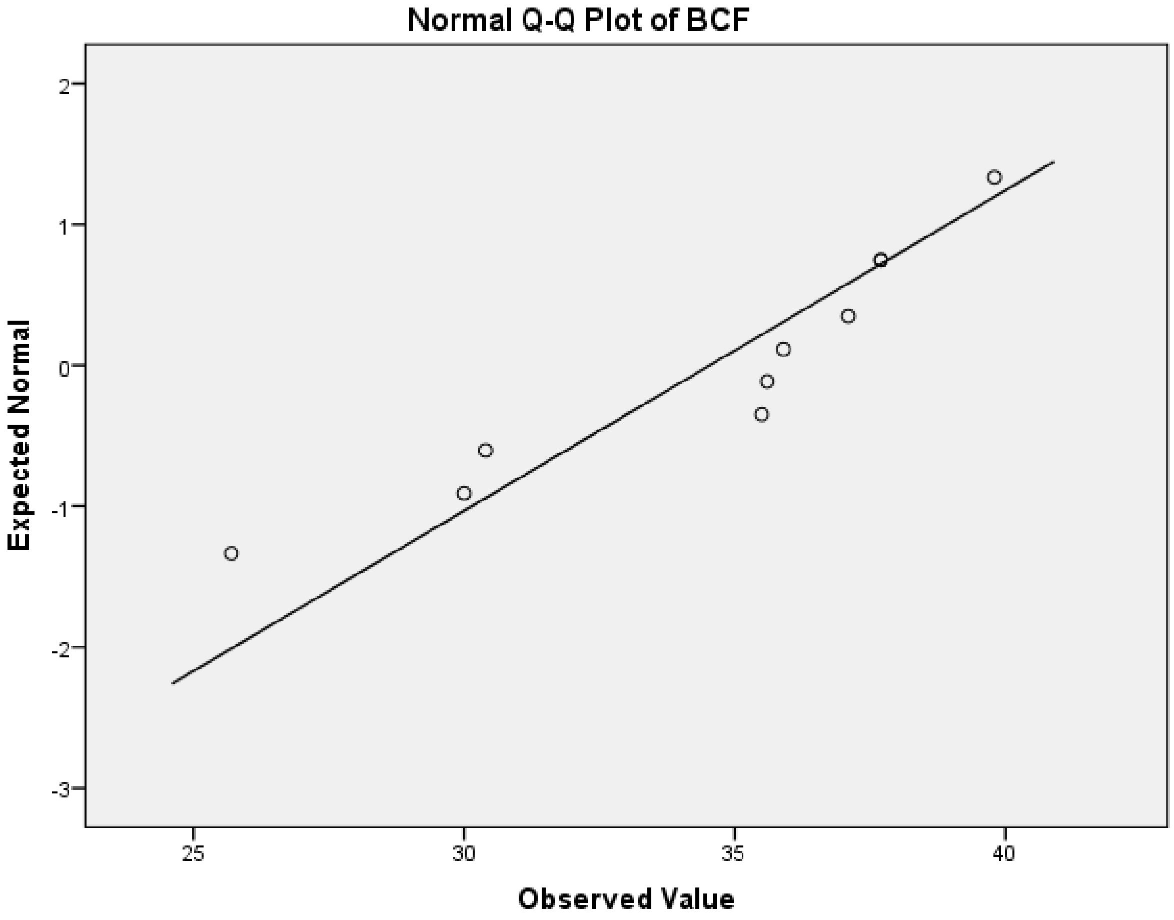

Normal Q-Q Plot of BCF
Expected Normal
Observed Value
2
1
0
-1
-2
-3
25
30
35
40

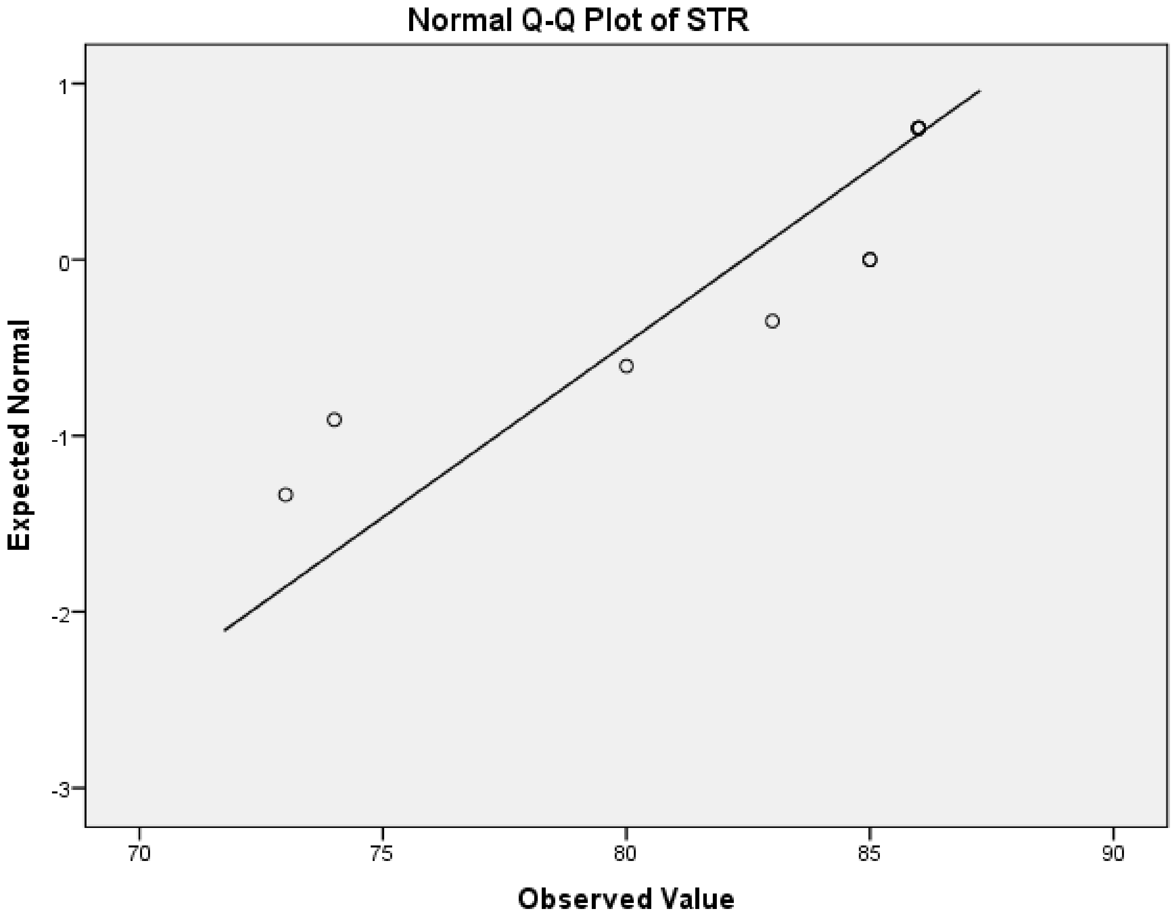

Normal Q-Q Plot of STR
Expected Normal
Observed Value
1
0
-1
-2
-3
70
75
80
85
90

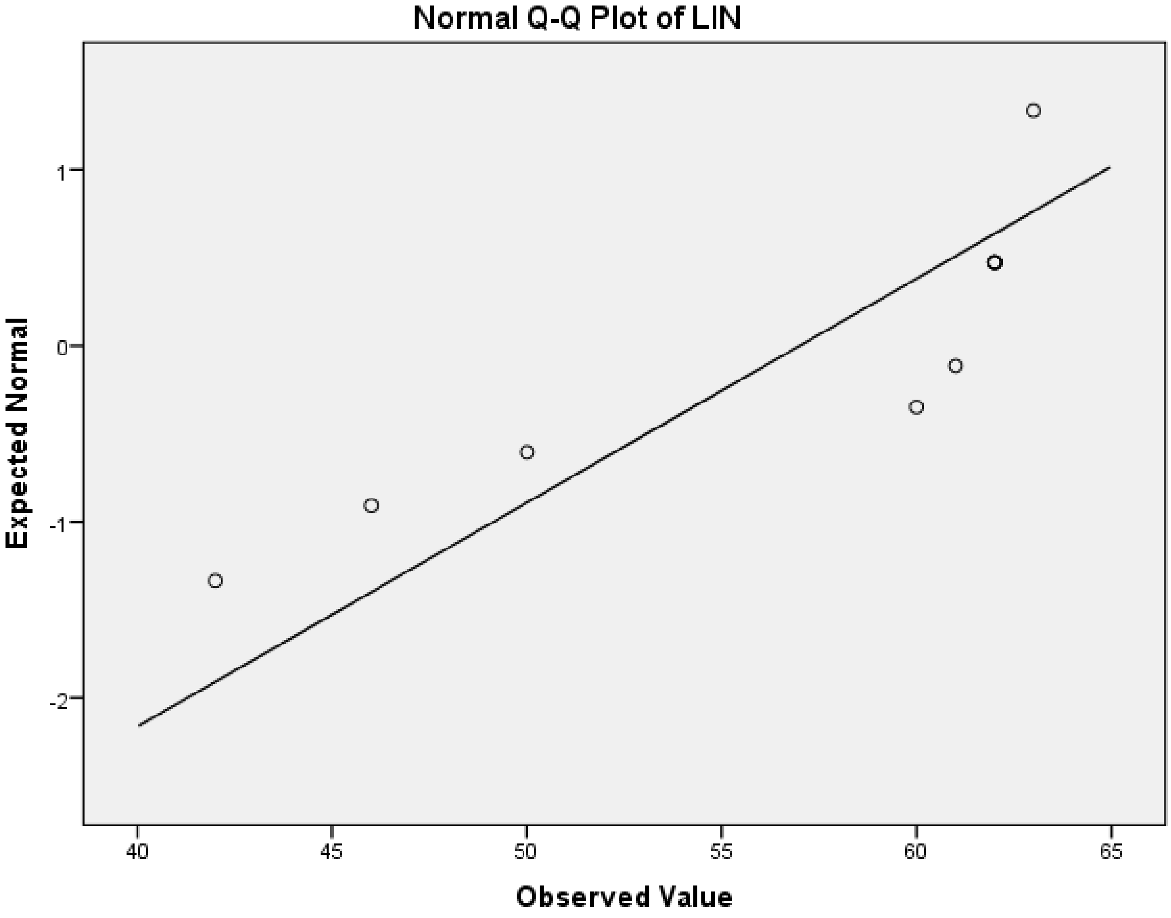

Detrended Normal Q-Q Plots

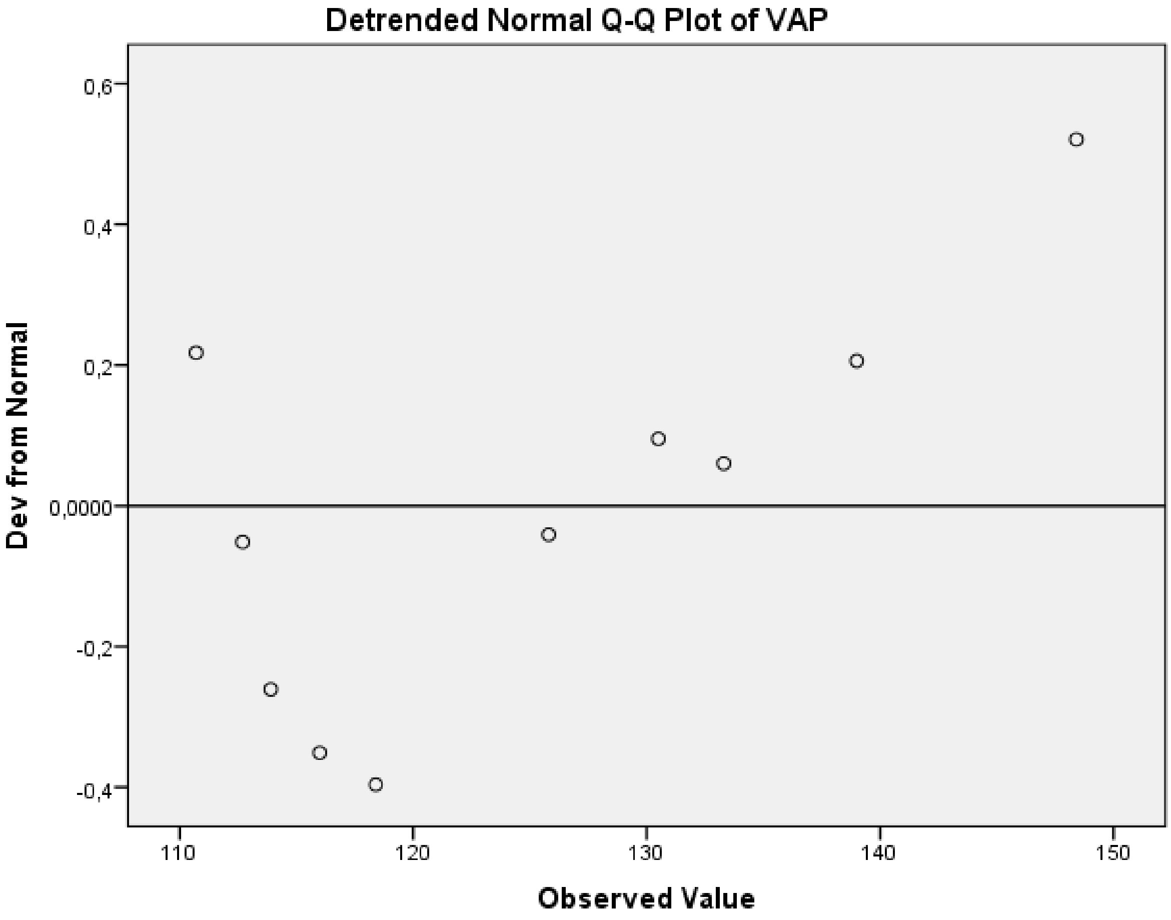

Detrended Normal Q-Q Plot of VAP
Dev from Normal
0,6
0,4
0,2
0,0000
-0,2
-0,4
110
120
130
140
150
Observed Value

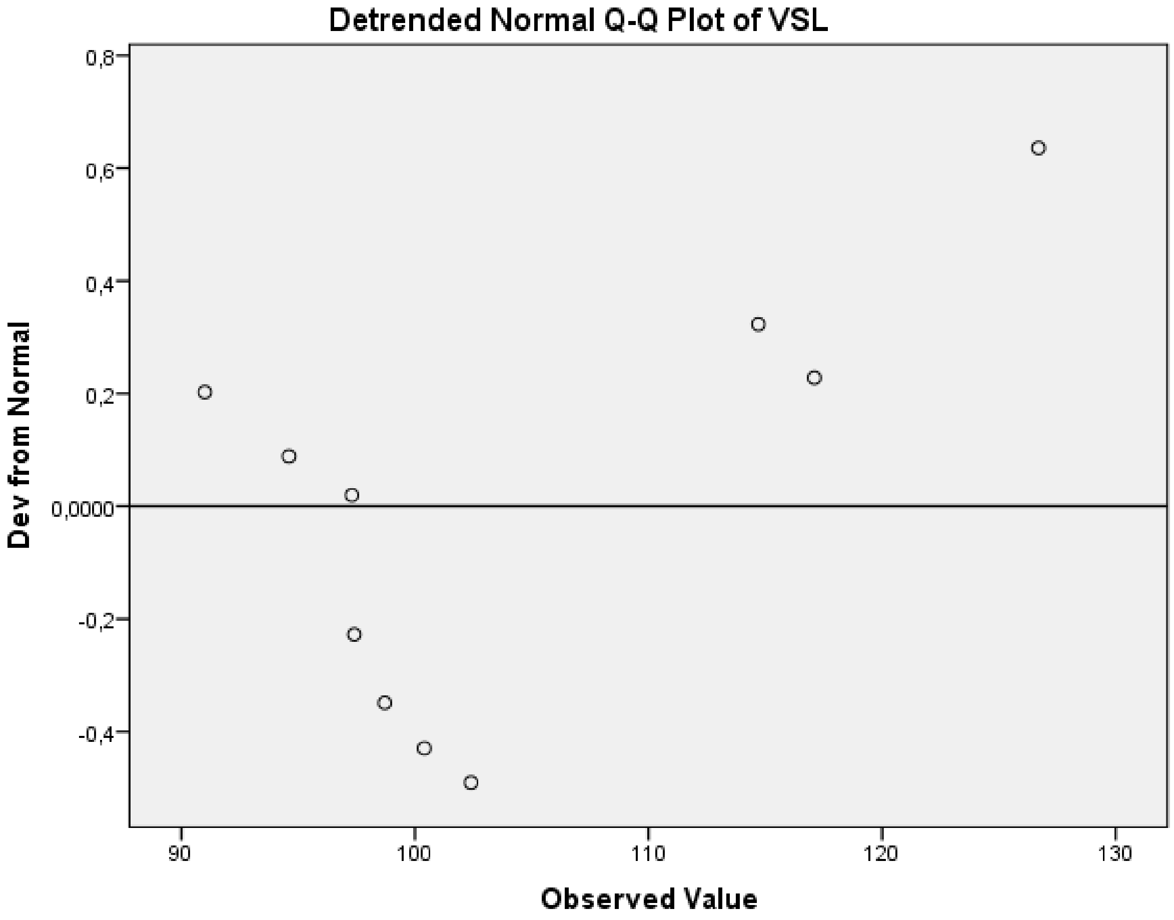
Detrended Normal Q-Q Plot of VSL
Dev from Normal
0,8
0,6
0,4
0,2
0,0000
-0,2
-0,4
90
100
110
120
130
Observed Value

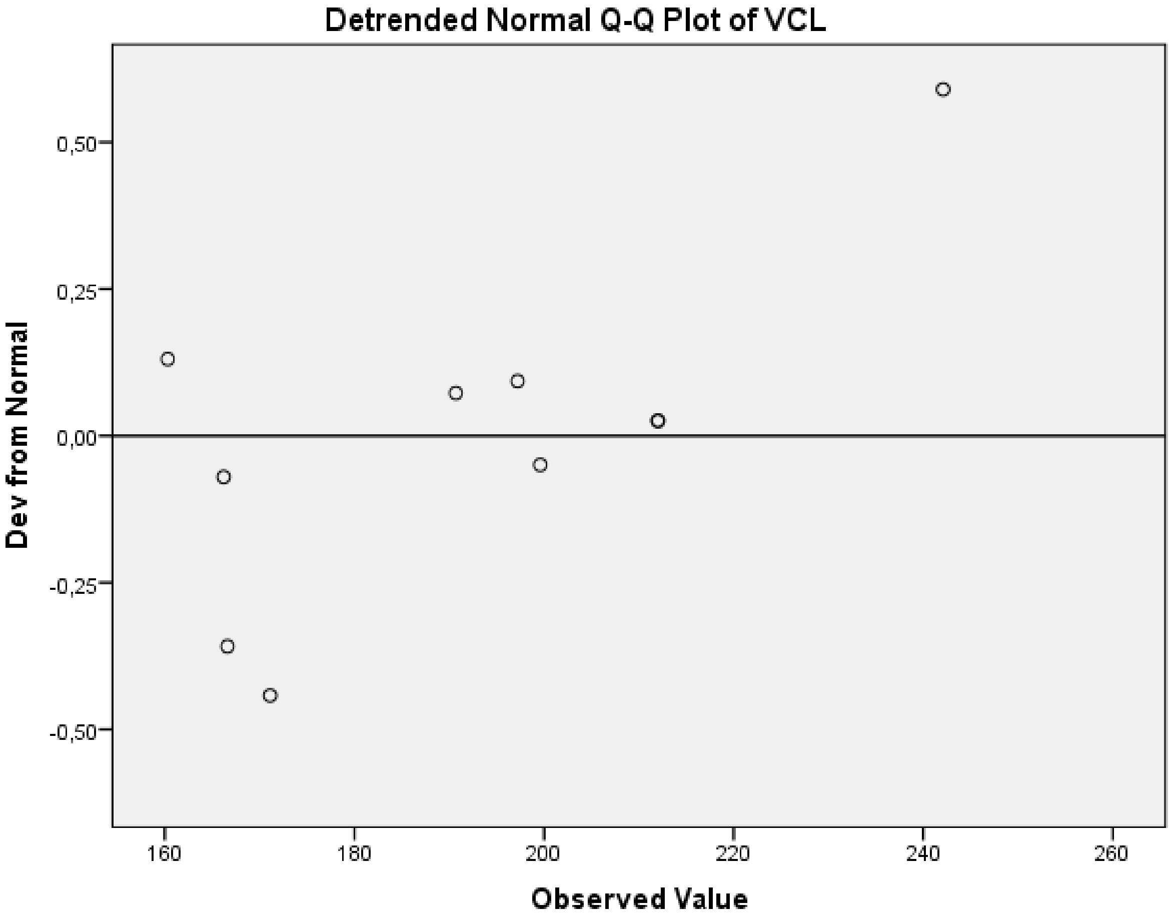
Detrended Normal Q-Q Plot of VCL
Dev from Normal
0,50
0,25
0,00
-0,25
-0,50
160
180
200
220
240
260
Observed Value

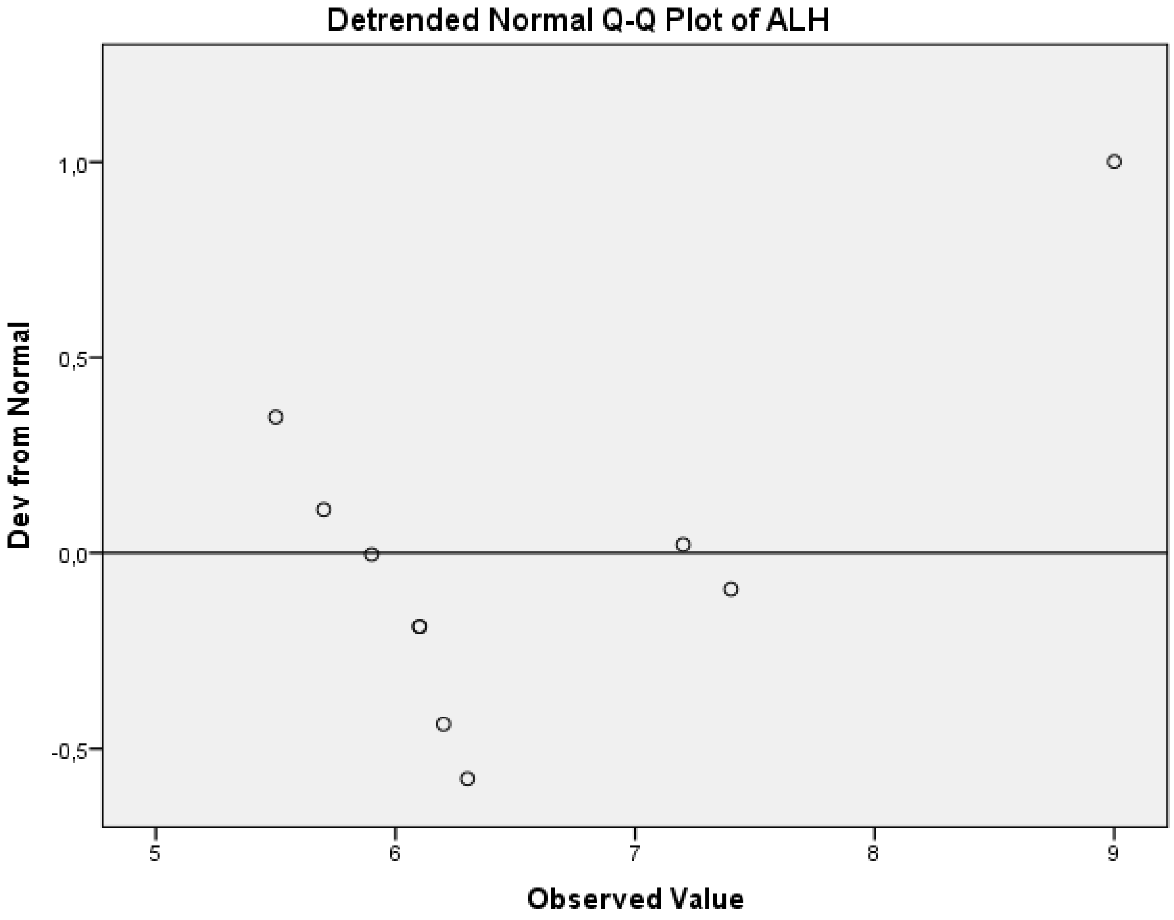

Detrended Normal Q-Q Plot of ALH
Dev from Normal
1,0
0,5
0,0
-0,5
5
6
7
8
9
Observed Value

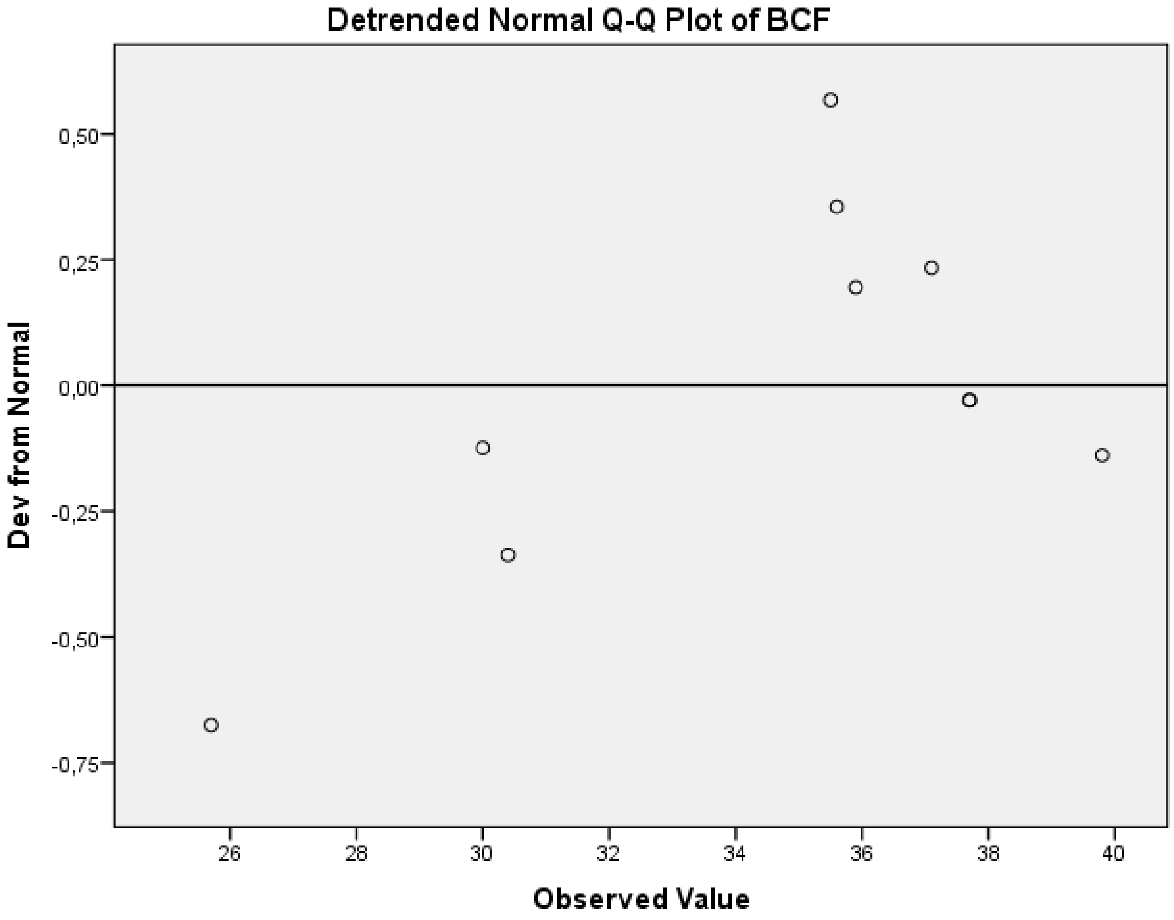
Detrended Normal Q-Q Plot of BCF
Dev from Normal
0,50
0,25
0,00
-0,25
-0,50
-0,75
26
28
30
32
34
36
38
40
Observed Value

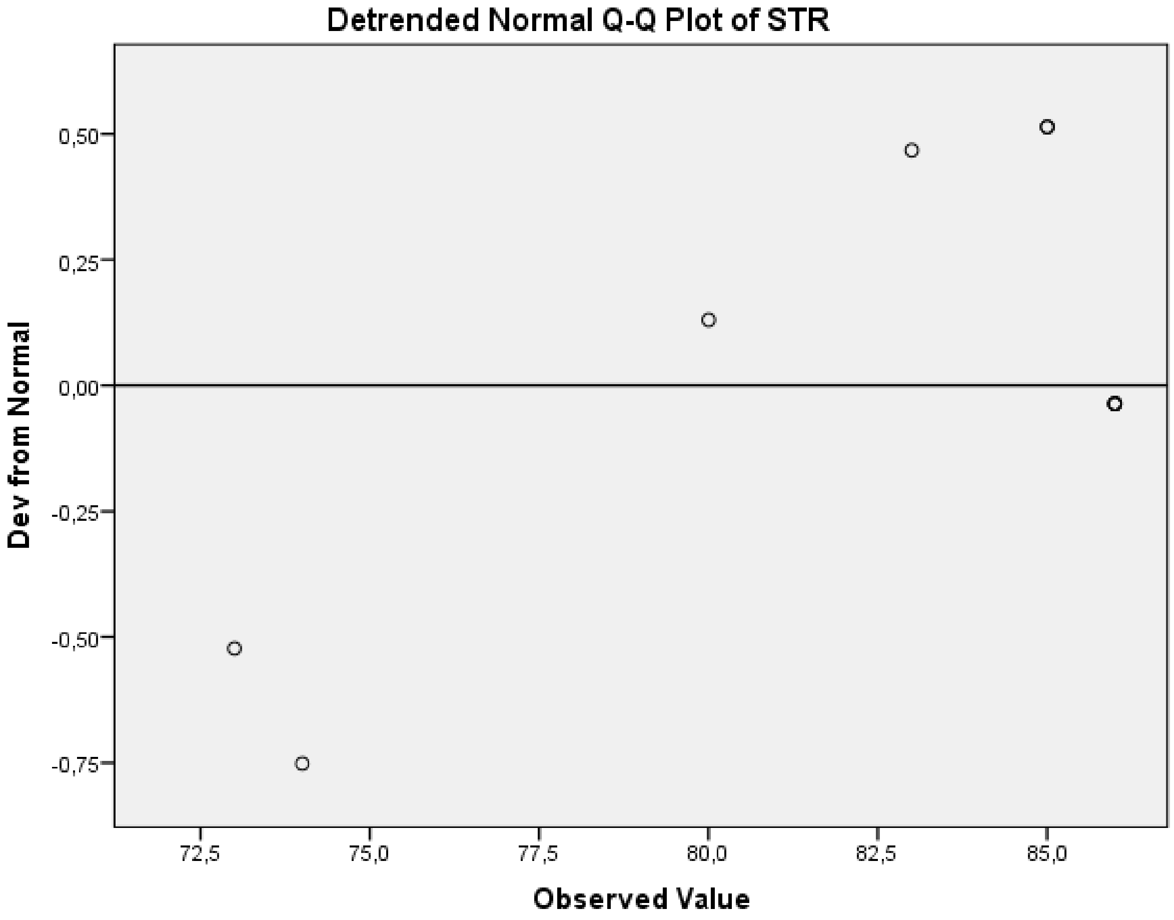
Detrended Normal Q-Q Plot of STR
Dev from Normal
0,50
0,25
0,00
-0,25
-0,50
-0,75
72,5
75,0
77,5
80,0
82,5
85,0
Observed Value

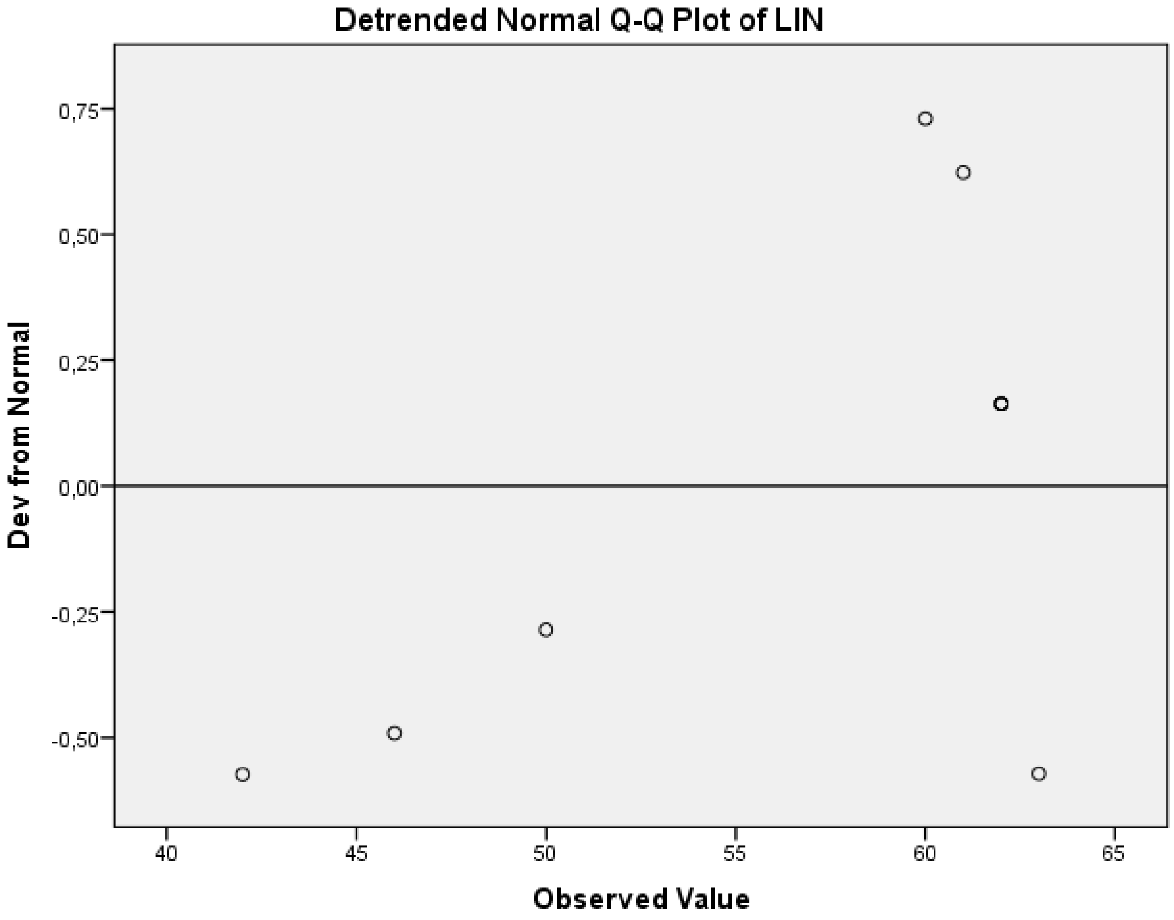

Grup = 3,00

Histograms

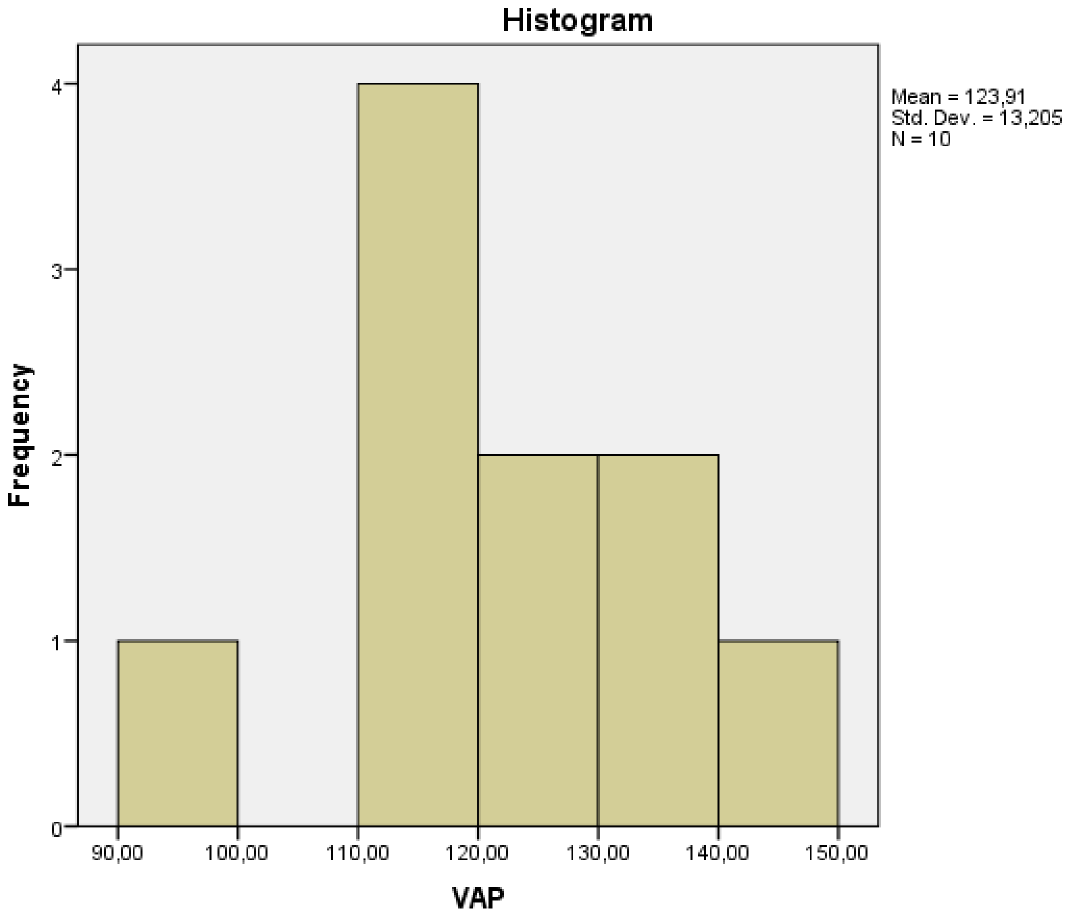

Histogram
Mean = 123,91
Std. Dev. = 13,205
N = 10
Frequency
0
1
2
3
4
90,00
100,00
110,00
120,00
130,00
140,00
150,00
VAP

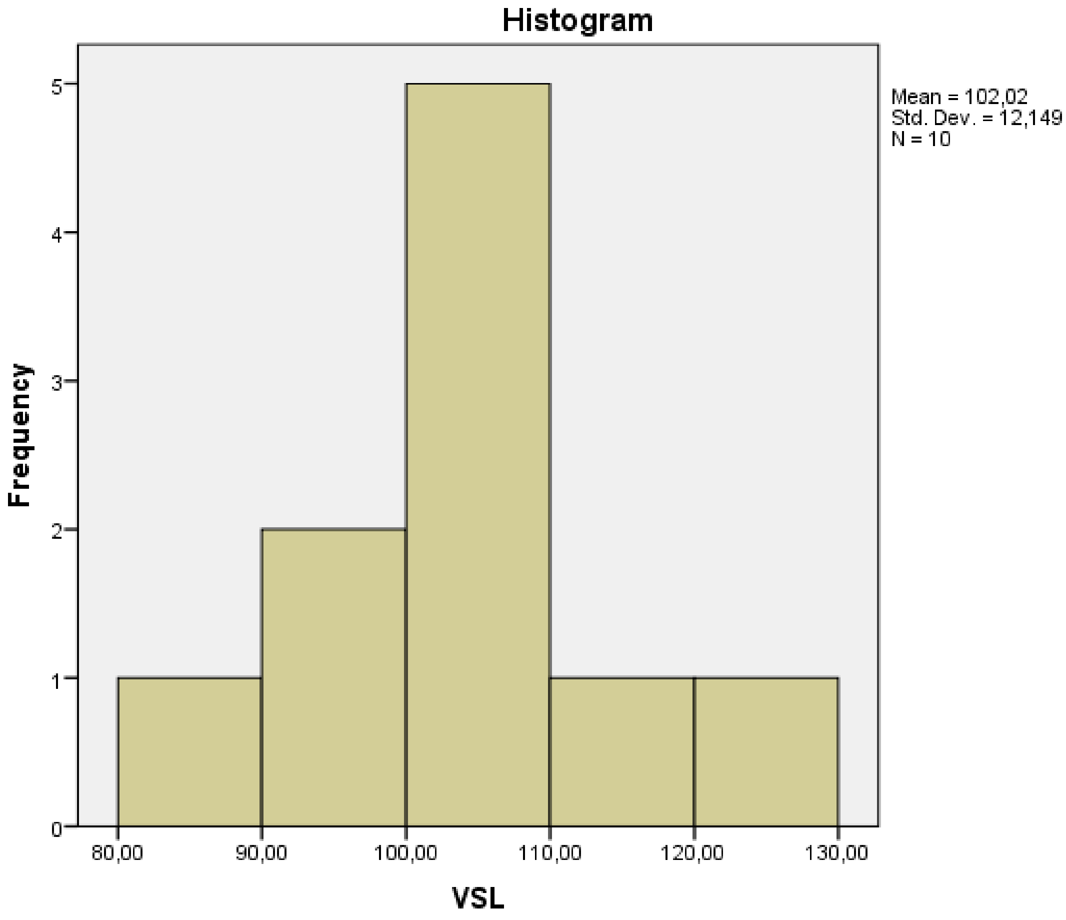
Histogram
Mean = 102,02
Std. Dev. = 12,149
N = 10
Frequency
0
1
2
3
4
5
80,00
90,00
100,00
110,00
120,00
130,00
VSL

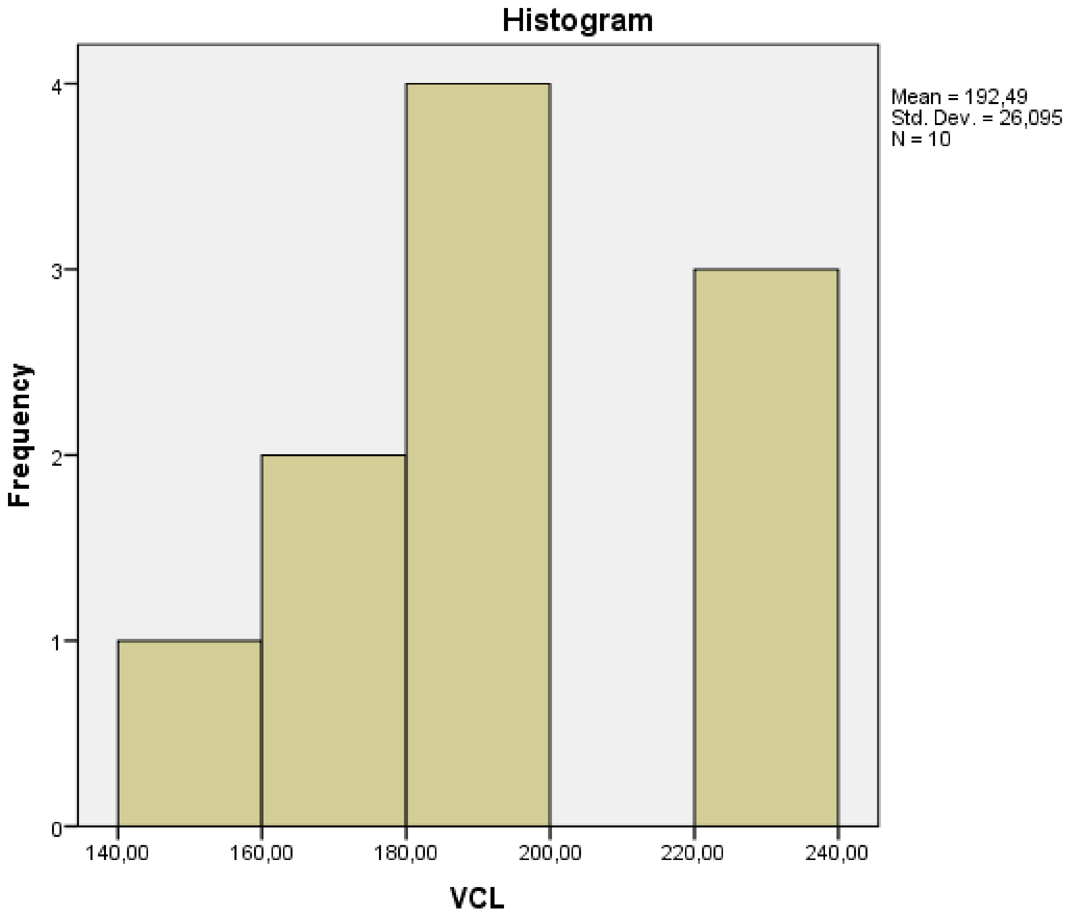
Histogram
Mean = 192,49
Std. Dev. = 26,095
N = 10
Frequency
4
3
2
1
0
140,00
160,00
180,00
200,00
220,00
240,00
VCL

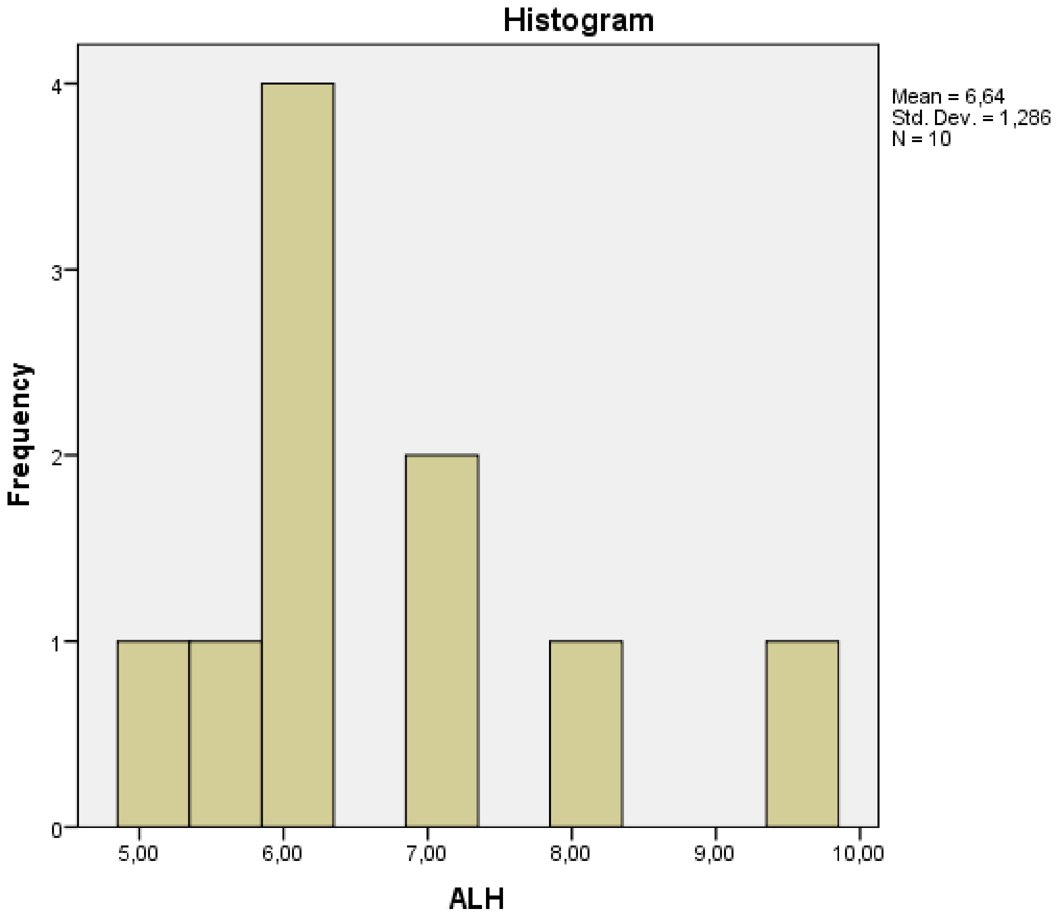
Histogram
Mean = 6,64
Std. Dev. = 1,286
N = 10
Frequency
4
3
2
1
0
5,00
6,00
7,00
8,00
9,00
10,00
ALH

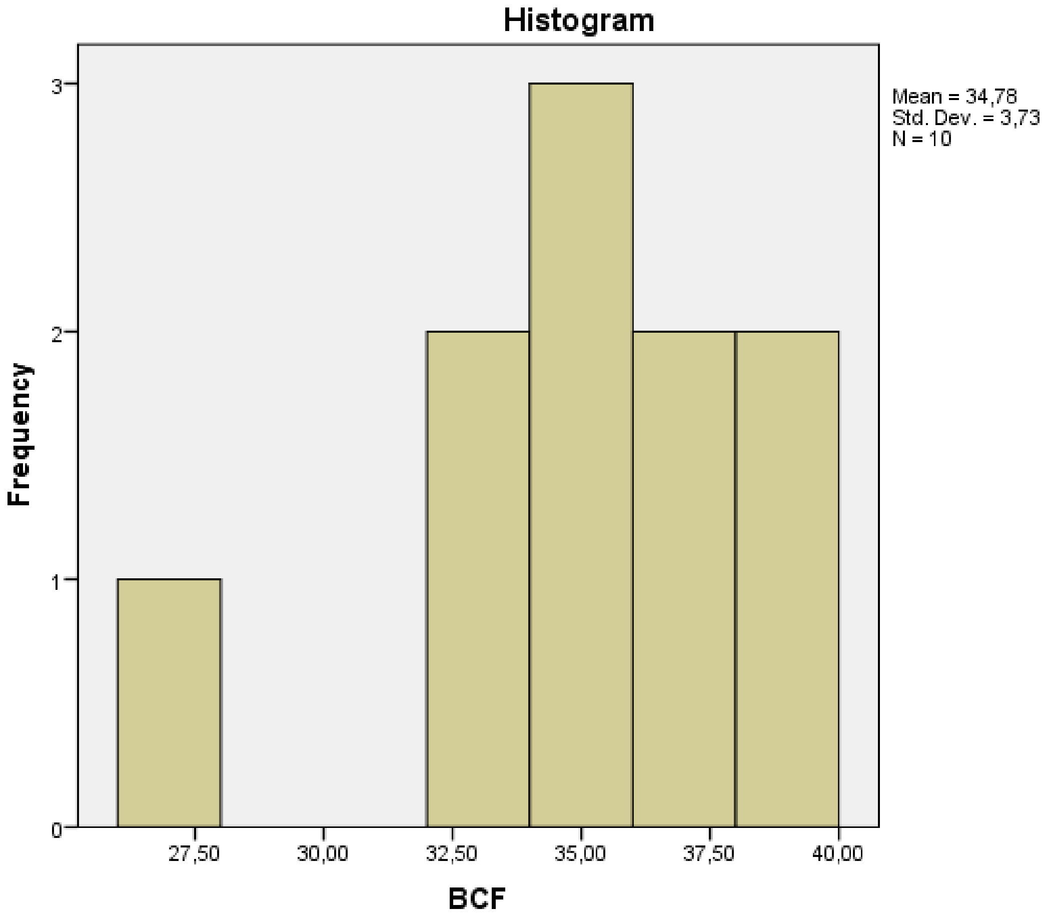

Histogram
Mean = 34,78
Std. Dev. = 3,73
N = 10
Frequency
0
1
2
3
27,50
30,00
32,50
35,00
37,50
40,00
BCF

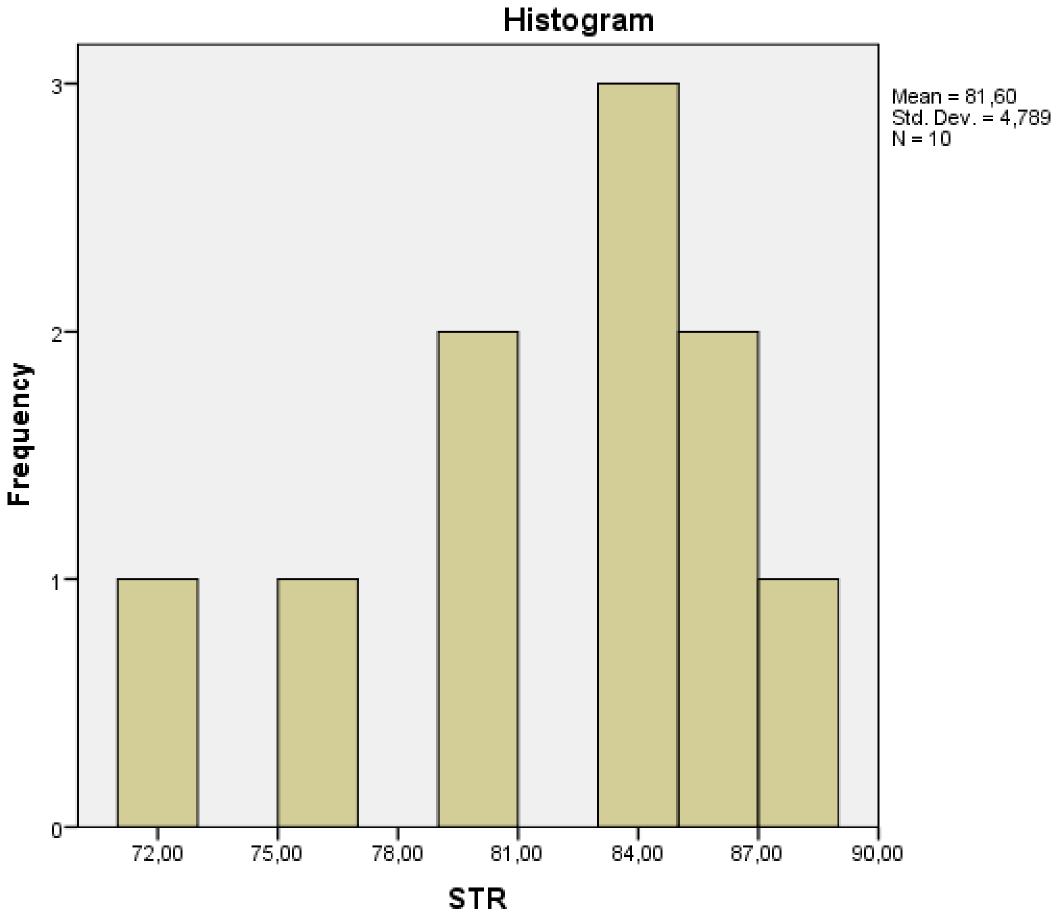
Histogram
Mean = 81,60
Std. Dev. = 4,789
N = 10
Frequency
0
1
2
3
72,00
75,00
78,00
81,00
84,00
87,00
90,00
STR

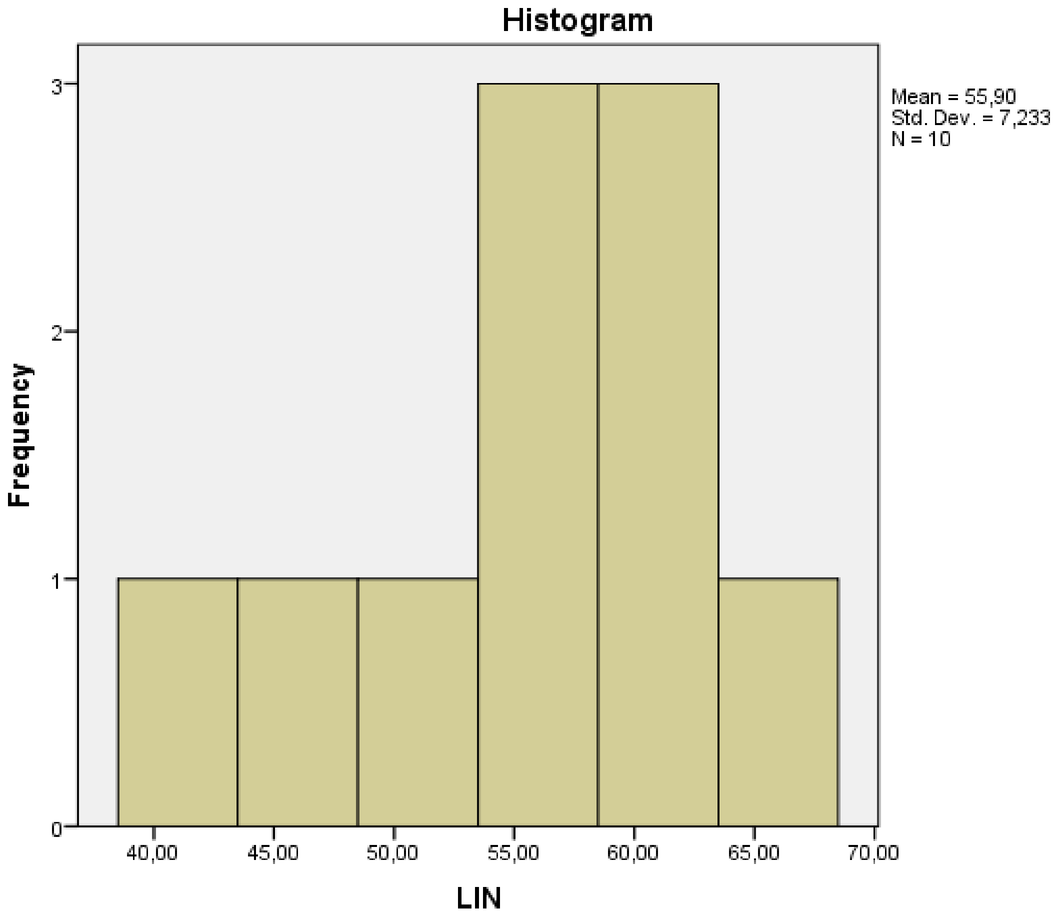

Stem-and-Leaf Plots

```
VAP Stem-and-Leaf Plot for
Grup= 3,00

 Frequency    Stem &  Leaf

     1,00        9 .  9
      ,00       10 .
     4,00       11 .  4899
     2,00       12 .  48
     2,00       13 .  12
     1,00       14 .  9

 Stem width:  10,00
 Each leaf:       1 case(s)
```

```
VSL Stem-and-Leaf Plot for
Grup= 3,00

 Frequency    Stem &  Leaf

     1,00        8 .  3
     2,00        9 .  13
     5,00       10 .  02244
     1,00       11 .  0
     1,00 Extremes    (>=128)

 Stem width:  10,00
 Each leaf:       1 case(s)
```

```
VCL Stem-and-Leaf Plot for
Grup= 3,00

 Frequency    Stem &  Leaf

     7,00        1 .  5778889
     3,00        2 .  223

 Stem width:  100,0
 Each leaf:       1 case(s)
```

```
ALH Stem-and-Leaf Plot for
Grup= 3,00

 Frequency    Stem &  Leaf

     2,00        5 .  14
     1,00        5 .  9
     3,00        6 .  023
     1,00        6 .  9
     1,00        7 .  3
     1,00        7 .  9
     1,00 Extremes    (>=9,4)

 Stem width:   1,00
 Each leaf:       1 case(s)
```

```
BCF Stem-and-Leaf Plot for
Grup= 3,00
```

```
 Frequency    Stem &  Leaf

     1,00 Extremes    (=<26)
     2,00        3 .  23
     3,00        3 .  445
     2,00        3 .  77
     2,00        3 .  88

 Stem width:  10,00
 Each leaf:       1 case(s)
```

```
STR Stem-and-Leaf Plot for
Grup= 3,00

 Frequency    Stem &  Leaf

     1,00        7 .  2
     2,00        7 .  69
     4,00        8 .  0344
     3,00        8 .  567

 Stem width:  10,00
 Each leaf:       1 case(s)
```

```
LIN Stem-and-Leaf Plot for
Grup= 3,00

 Frequency    Stem &  Leaf

     1,00        4 .  1
     1,00        4 .  8
     1,00        5 .  0
     4,00        5 .  8889
     3,00        6 .  124

 Stem width:  10,00
 Each leaf:       1 case(s)
```

Normal Q-Q Plots

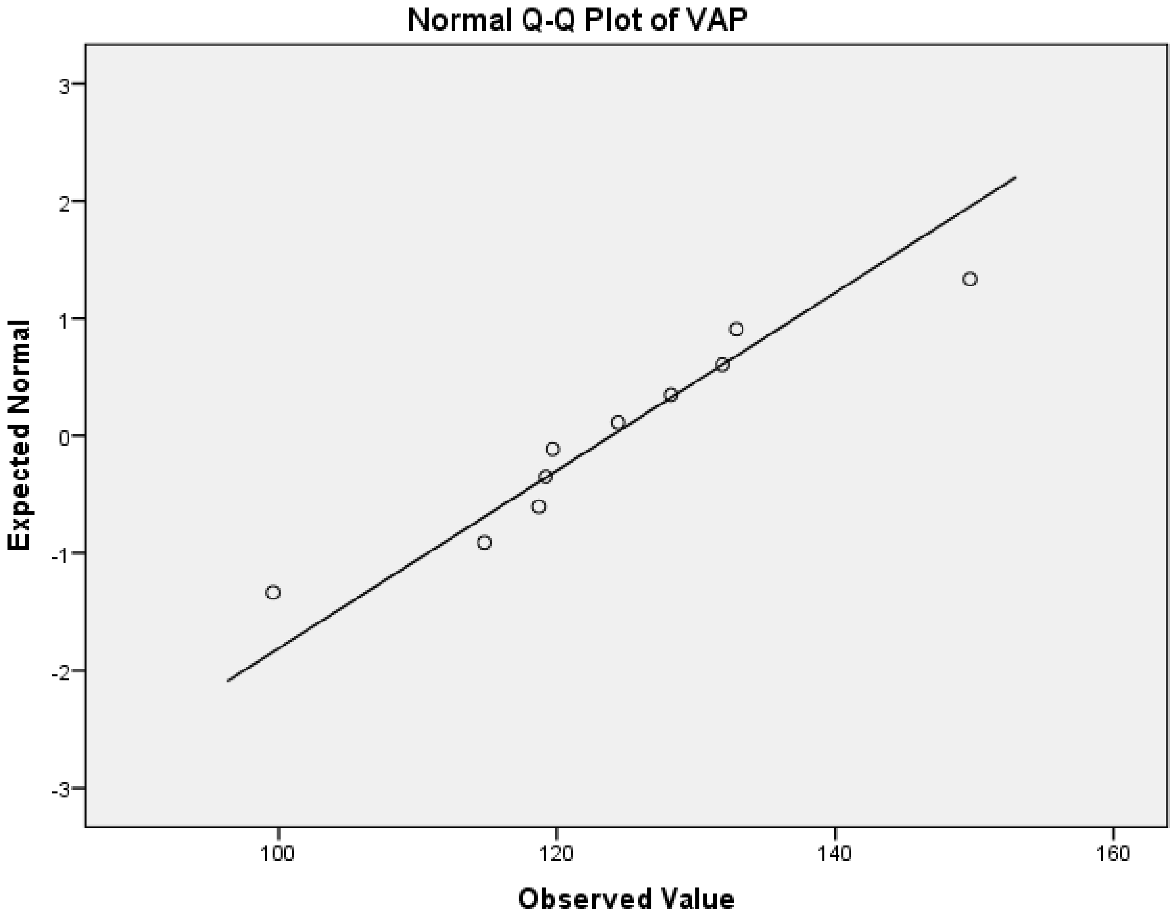
Normal Q-Q Plot of VAP
Expected Normal
3
2
1
0
-1
-2
-3
100
120
140
160
Observed Value

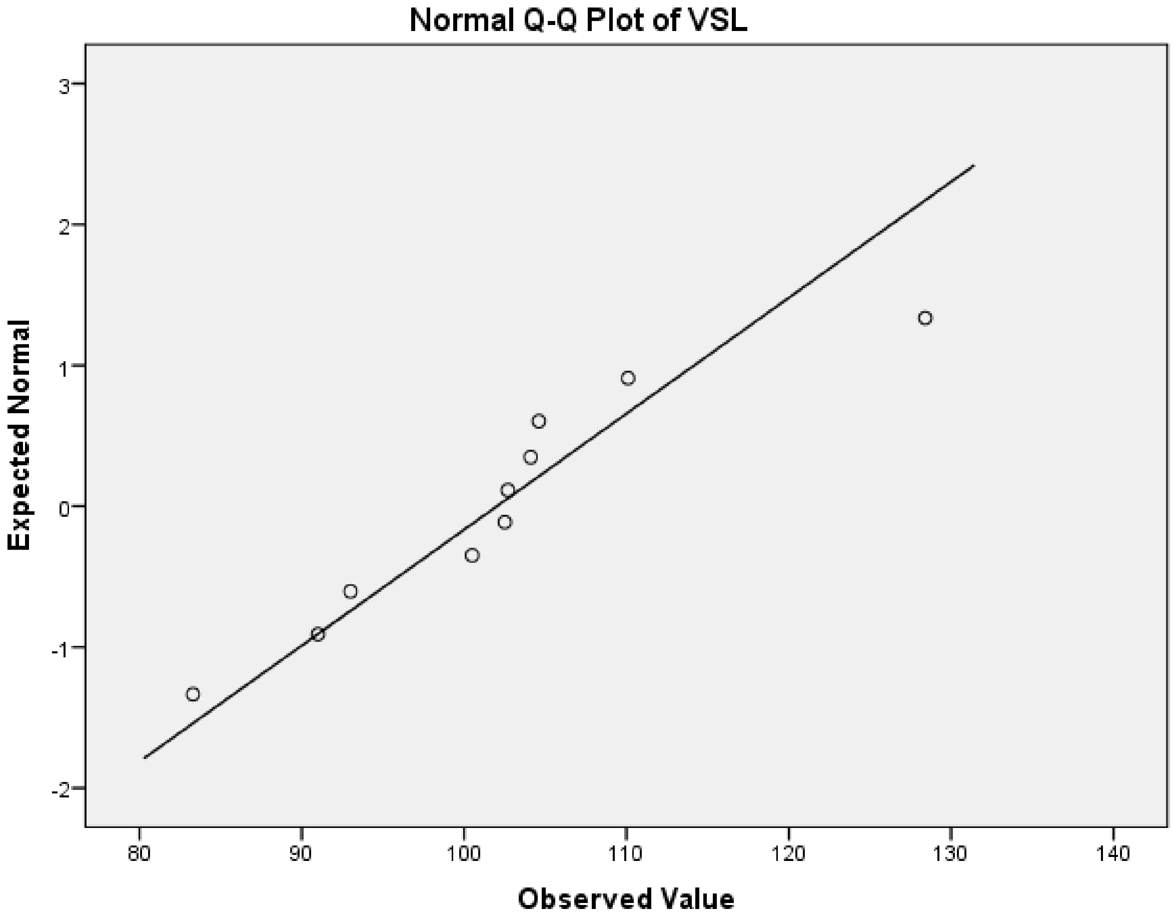
Normal Q-Q Plot of VSL
Expected Normal
Observed Value
3
2
1
0
-1
-2
80
90
100
110
120
130
140

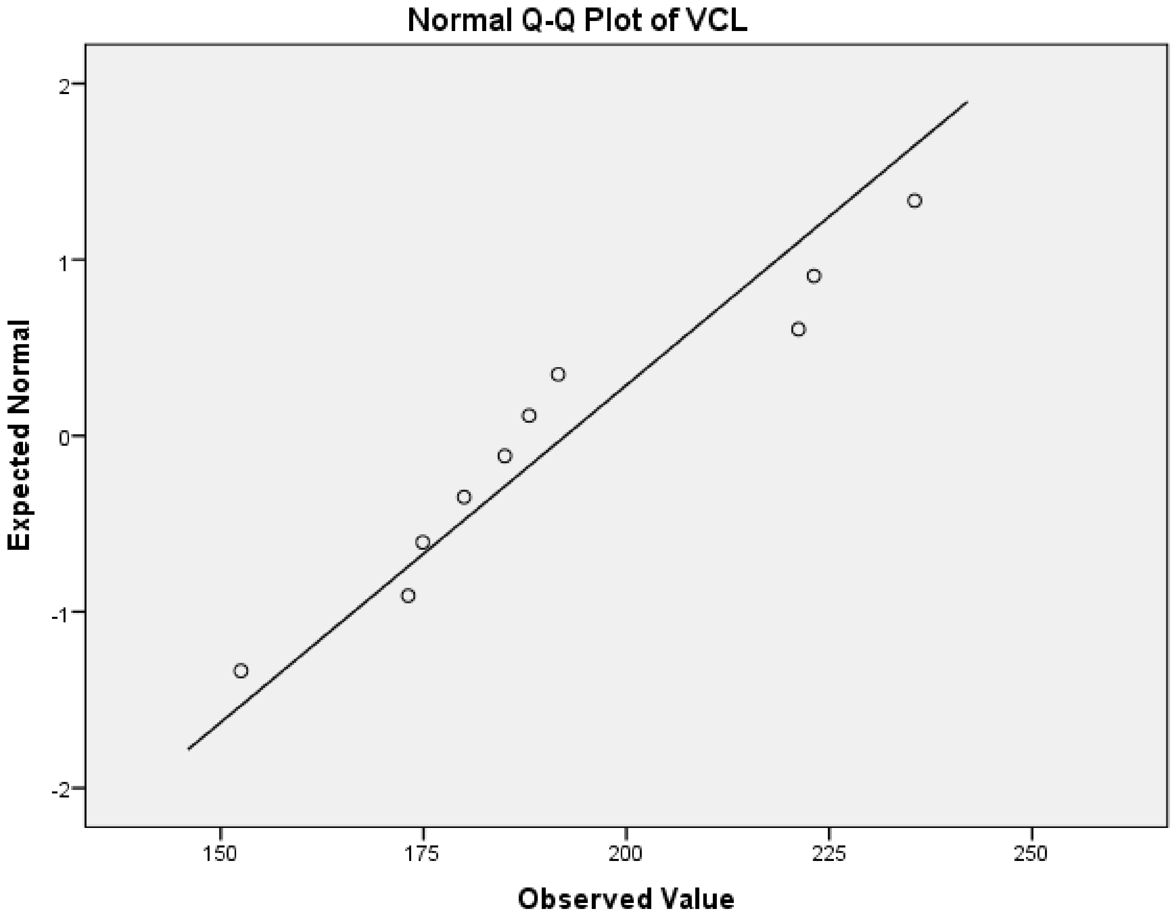

Normal Q-Q Plot of VCL
Expected Normal
2
1
0
-1
-2
150
175
200
225
250
Observed Value

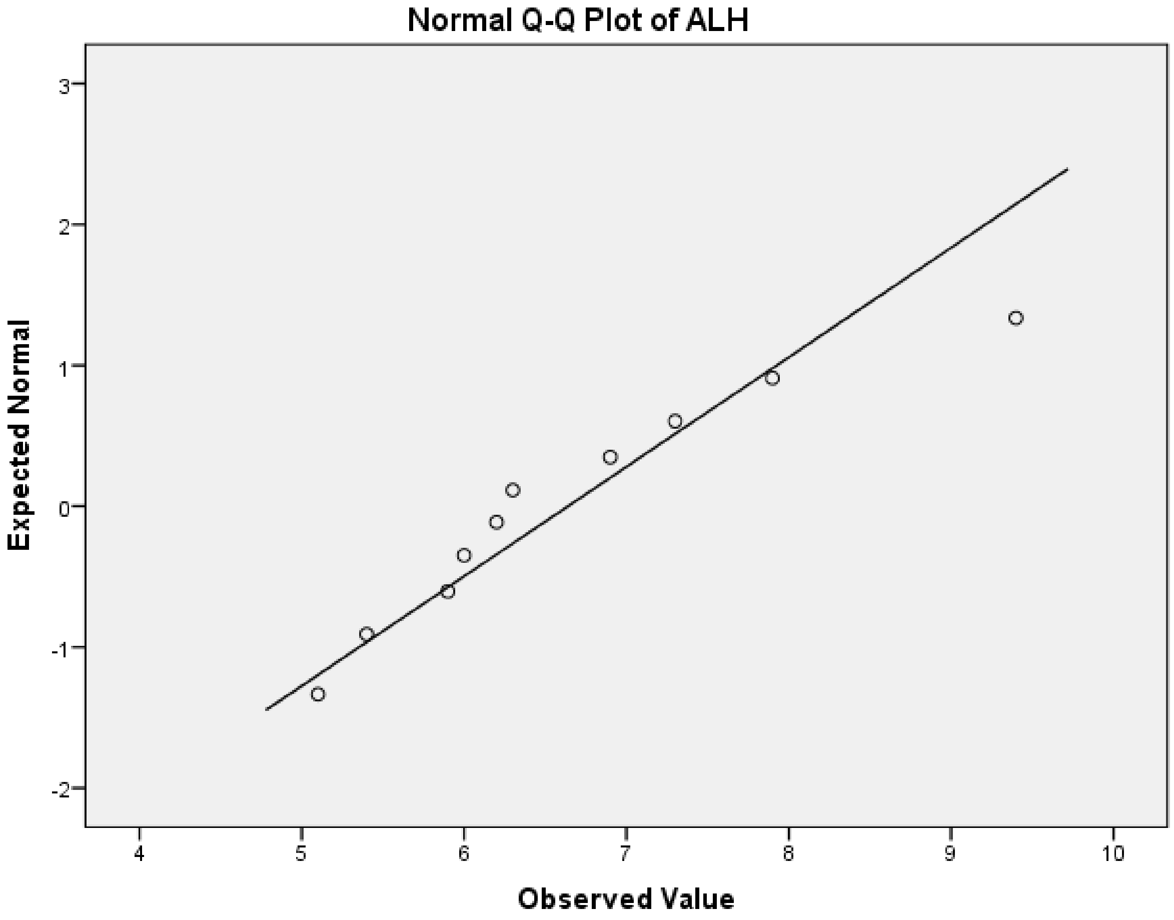
Normal Q-Q Plot of ALH
Expected Normal
3
2
1
0
-1
-2
4
5
6
7
8
9
10
Observed Value

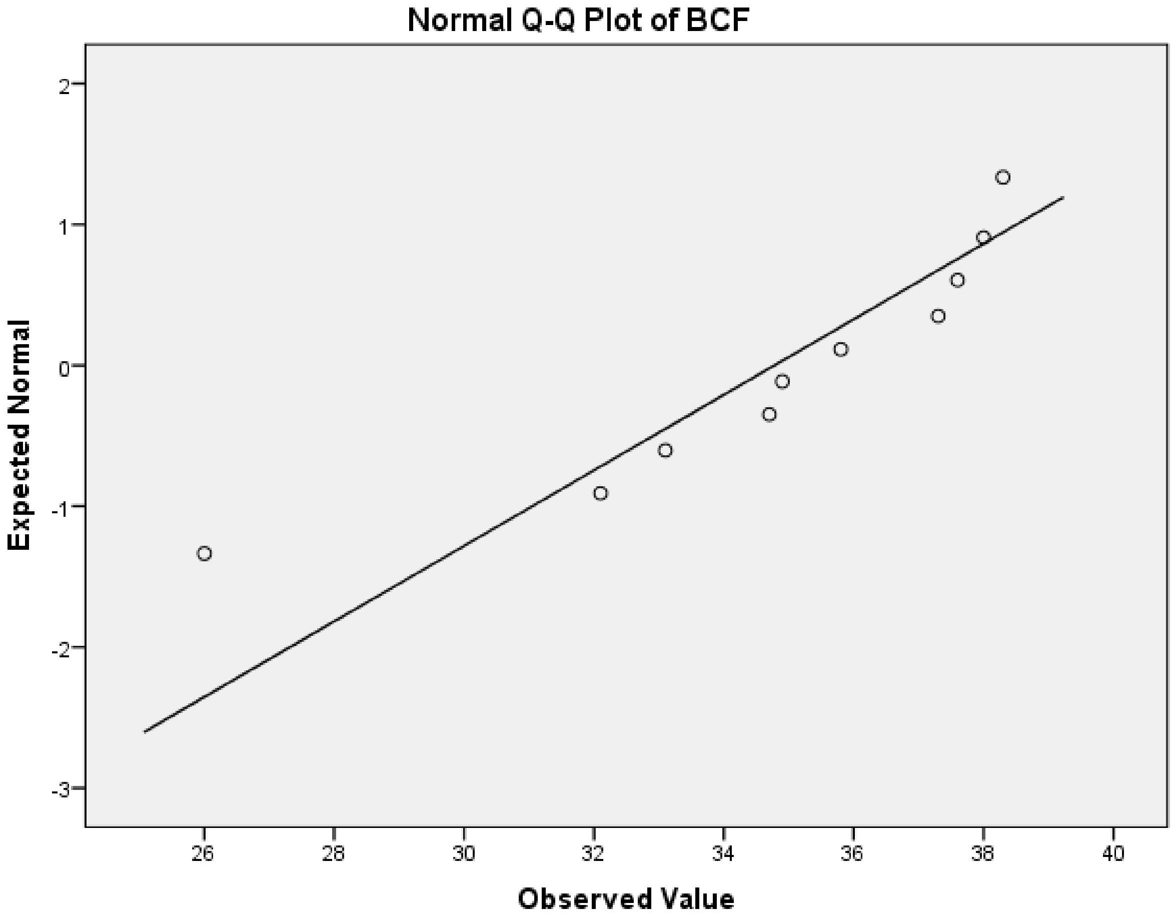
Normal Q-Q Plot of BCF
Expected Normal
Observed Value
2
1
0
-1
-2
-3
26
28
30
32
34
36
38
40

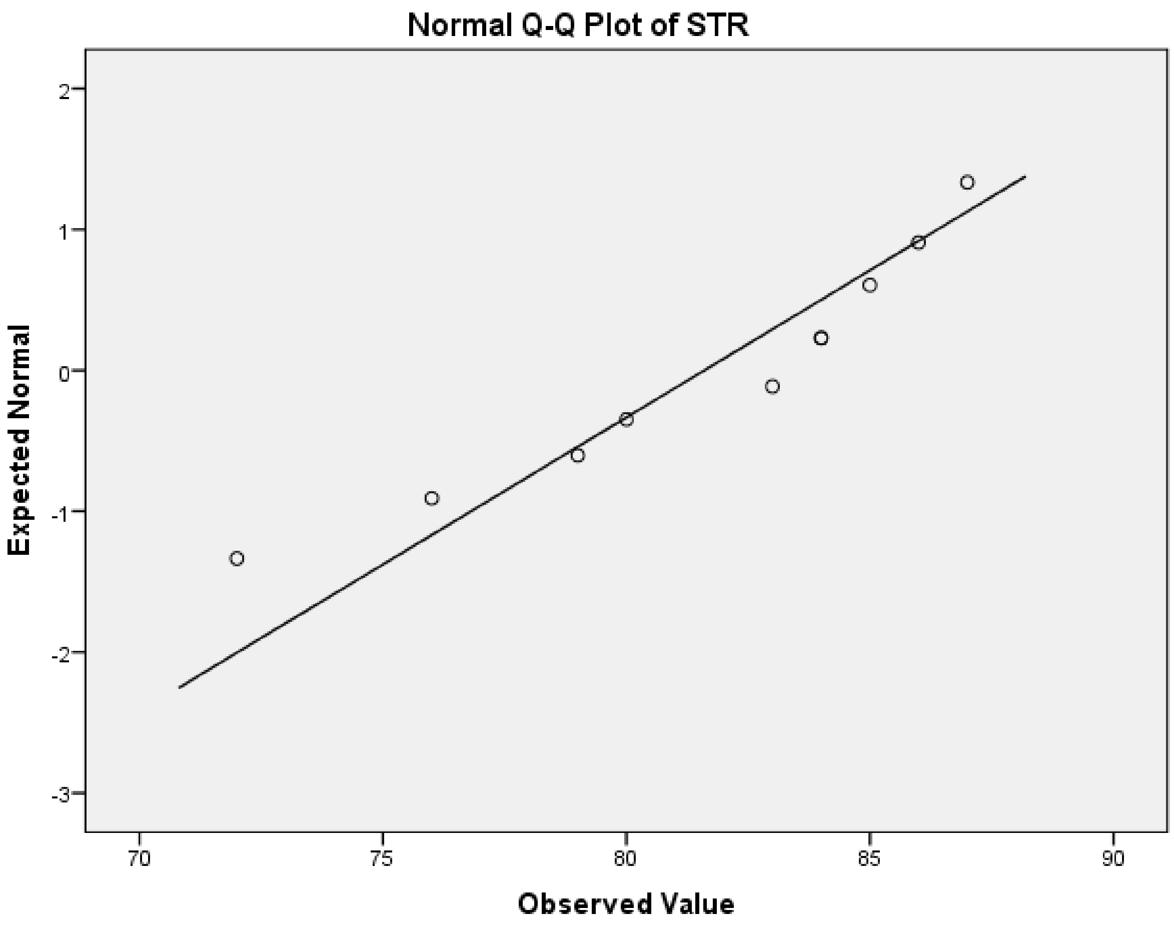

Normal Q-Q Plot of STR
Expected Normal
Observed Value
2
1
0
-1
-2
-3
70
75
80
85
90

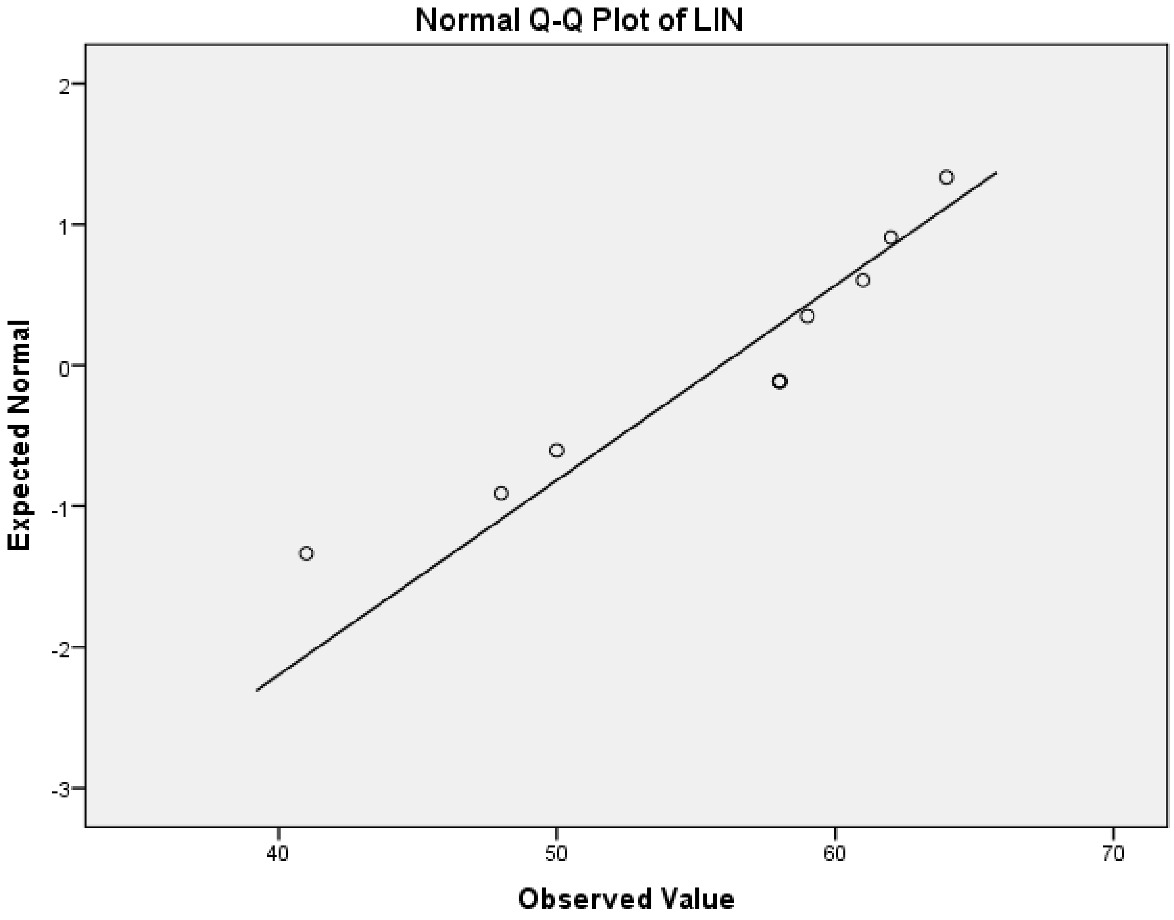

Detrended Normal Q-Q Plots

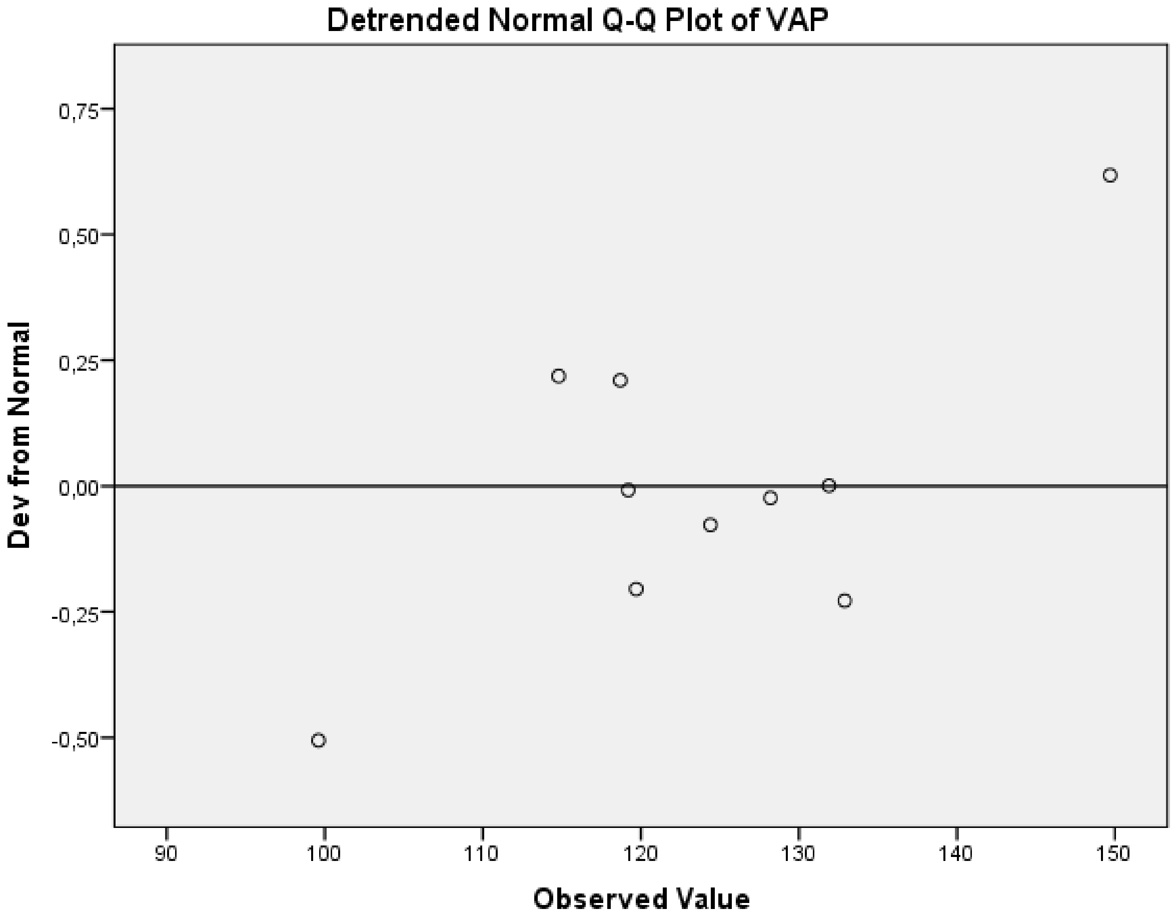
Detrended Normal Q-Q Plot of VAP
Dev from Normal
0,75
0,50
0,25
0,00
-0,25
-0,50
90
100
110
120
130
140
150
Observed Value

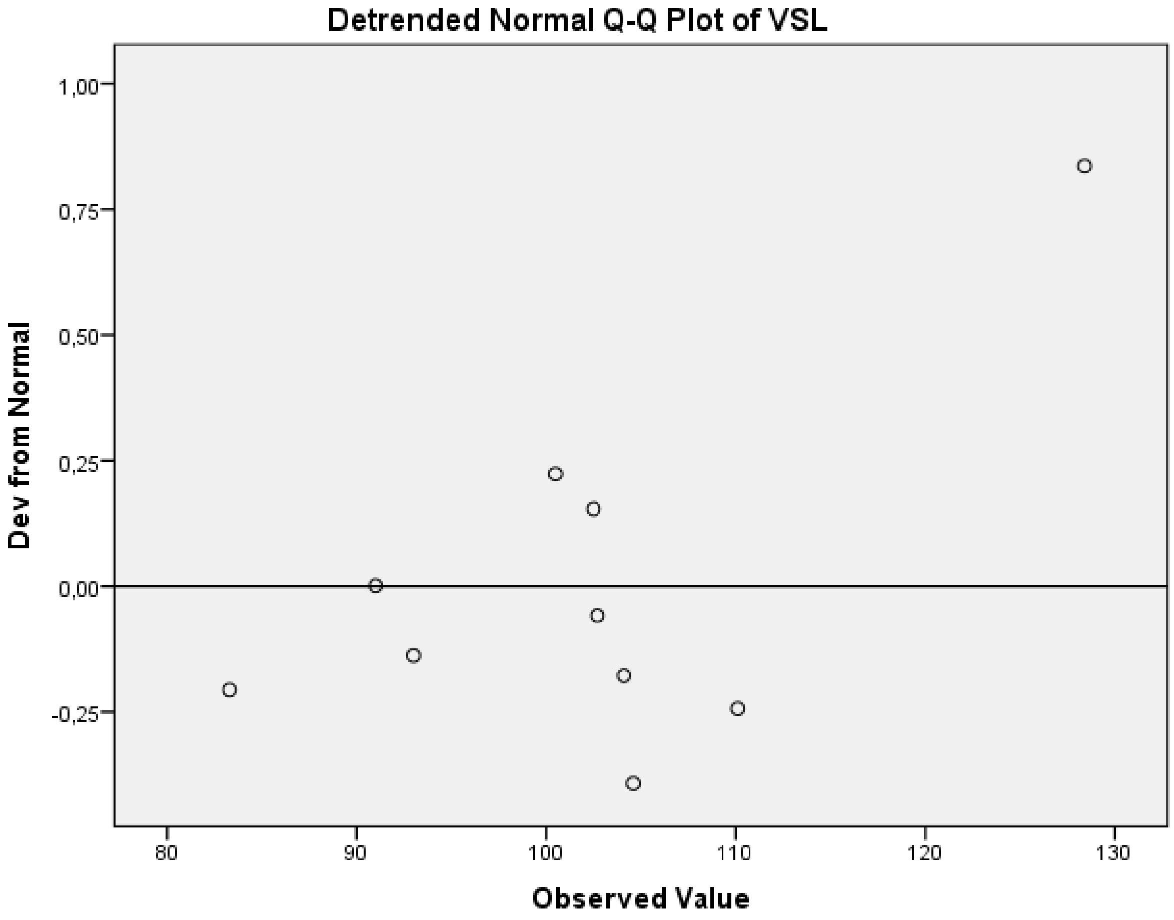
Detrended Normal Q-Q Plot of VSL
Dev from Normal
1,00
0,75
0,50
0,25
0,00
-0,25
80
90
100
110
120
130
Observed Value

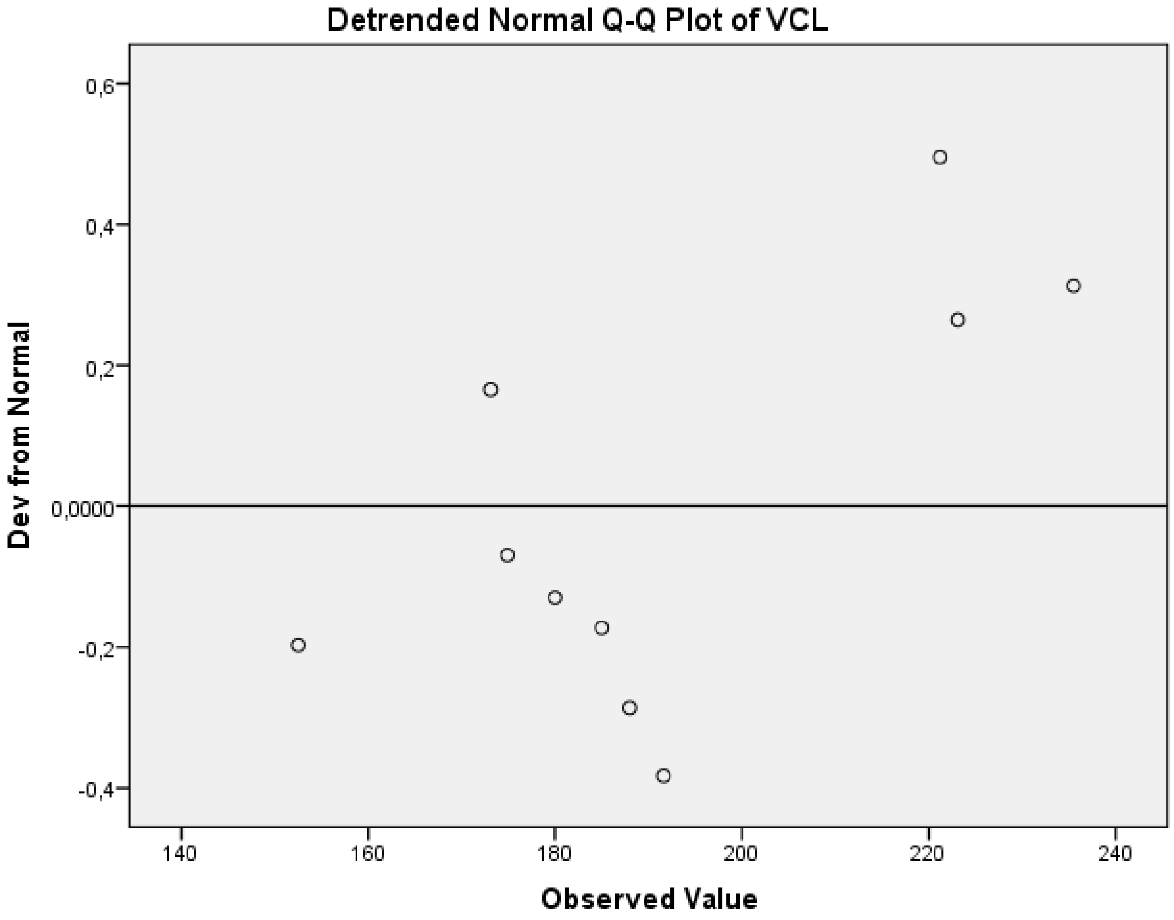
Detrended Normal Q-Q Plot of VCL
Dev from Normal
0,6
0,4
0,2
0,0000
-0,2
-0,4
140
160
180
200
220
240
Observed Value

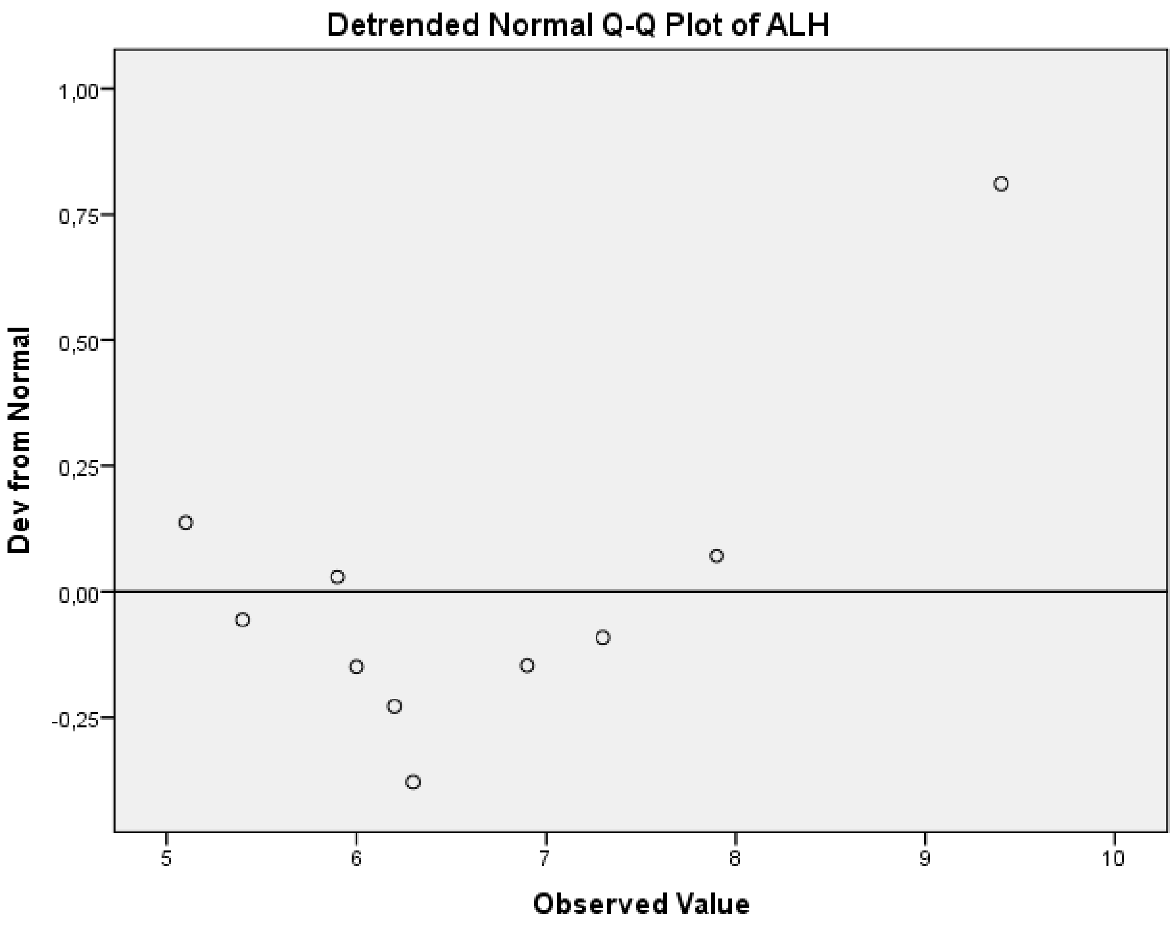

Detrended Normal Q-Q Plot of ALH
Dev from Normal
1,00
0,75
0,50
0,25
0,00
-0,25
5
6
7
8
9
10
Observed Value

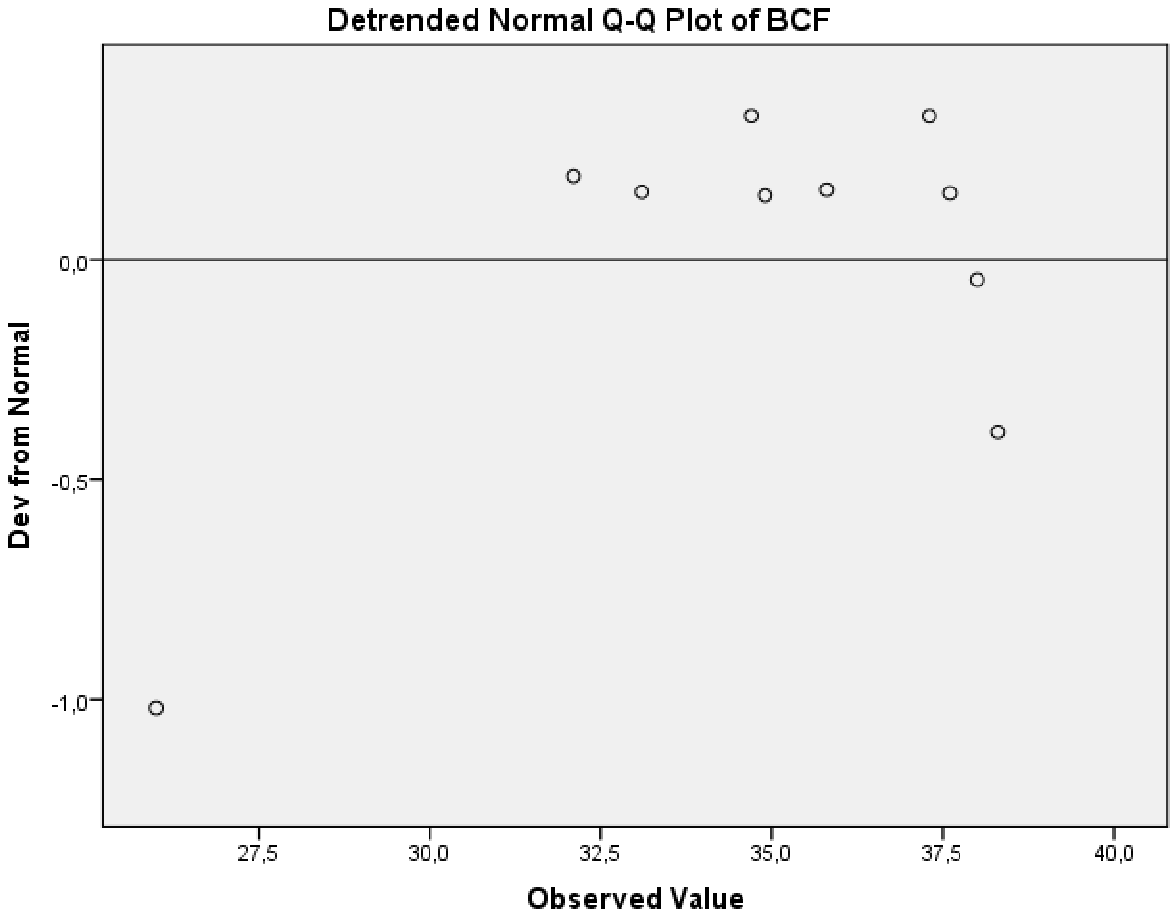
Detrended Normal Q-Q Plot of BCF
Dev from Normal
0,0
-0,5
-1,0
27,5
30,0
32,5
35,0
37,5
40,0
Observed Value

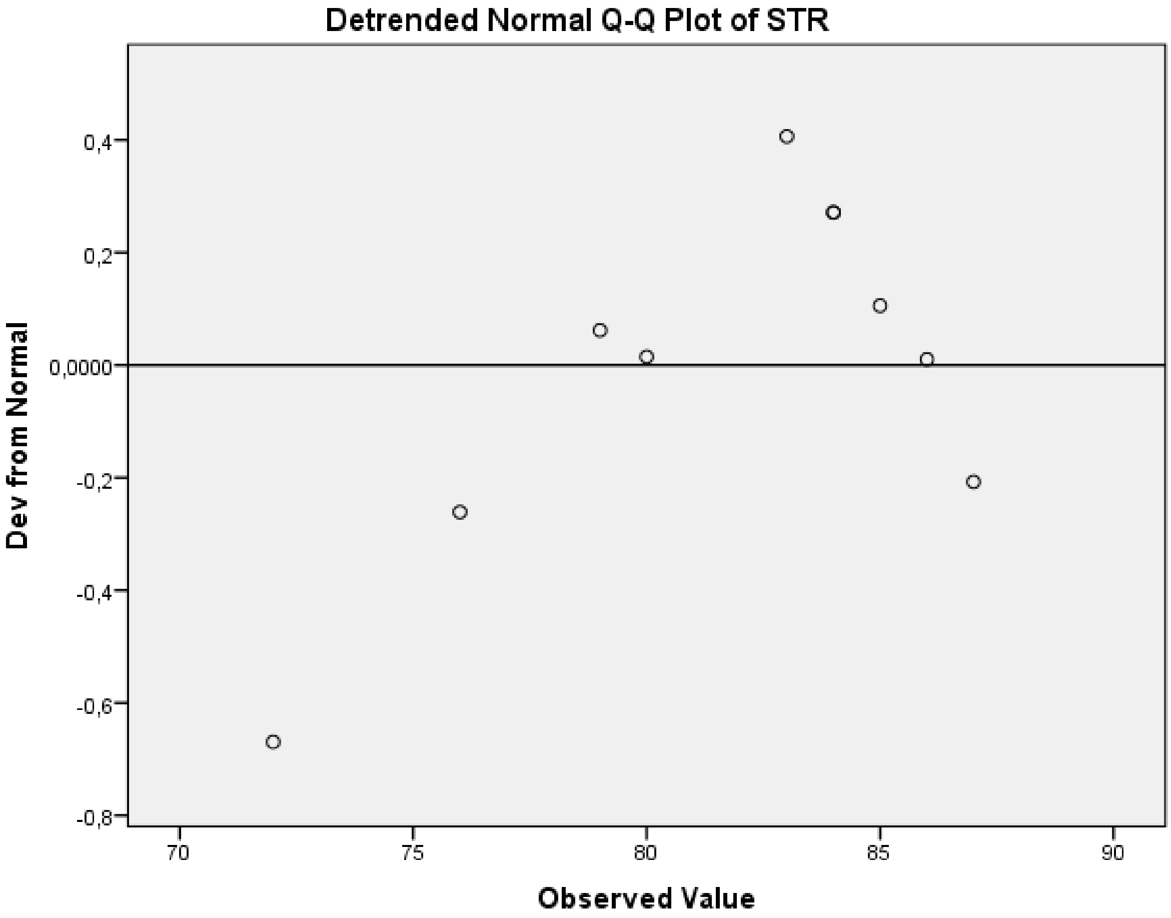
Detrended Normal Q-Q Plot of STR
Dev from Normal
Observed Value
0,4
0,2
0,0000
-0,2
-0,4
-0,6
-0,8
70
75
80
85
90

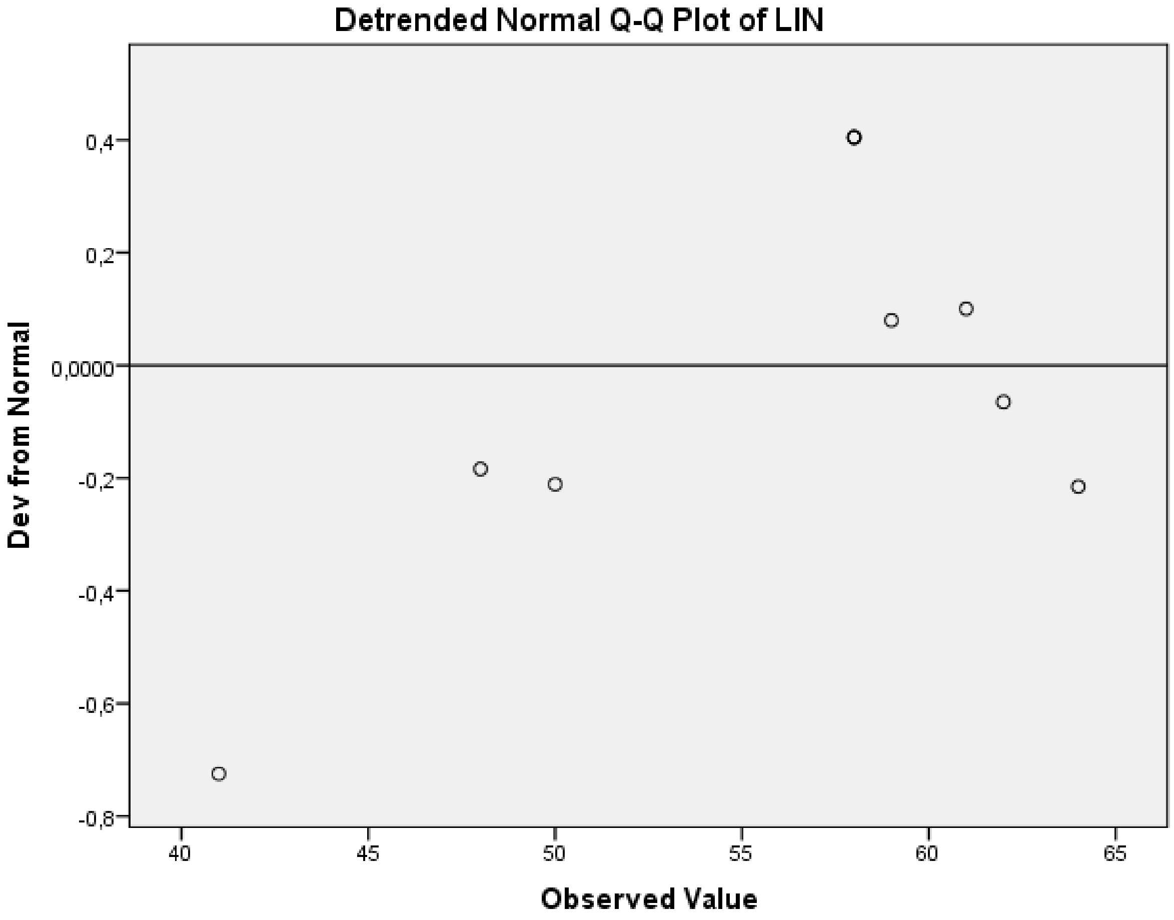

Grup = 4,00

Histograms

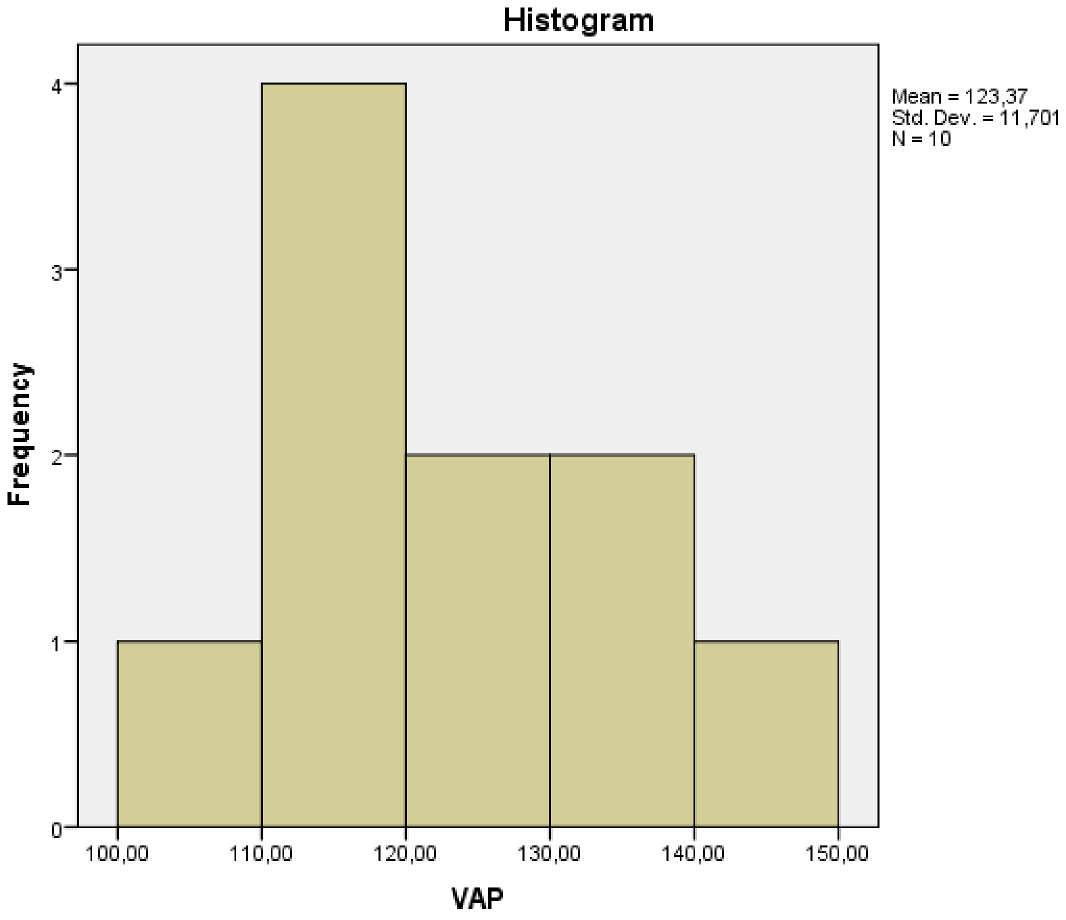
Histogram
Mean = 123,37
Std. Dev. = 11,701
N = 10
Frequency
0
1
2
3
4
100,00
110,00
120,00
130,00
140,00
150,00
VAP

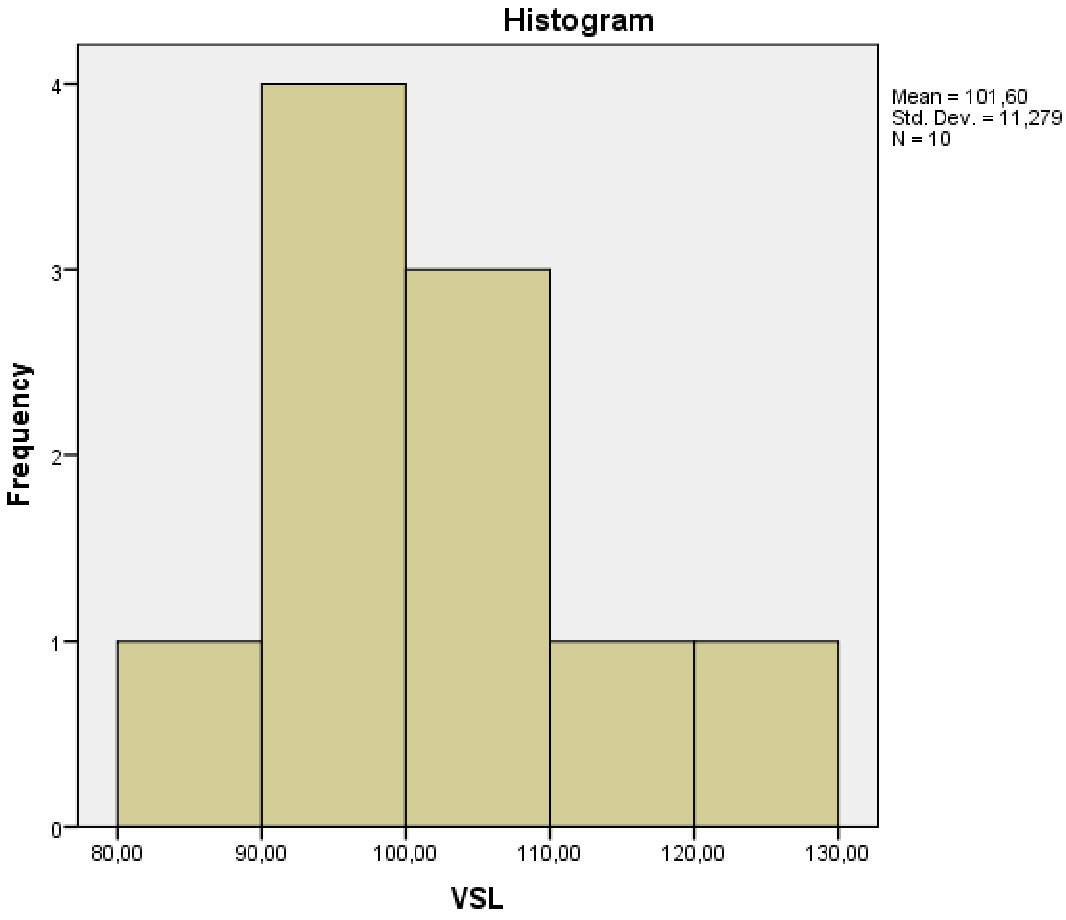
Histogram
Mean = 101,60
Std. Dev. = 11,279
N = 10
Frequency
0
1
2
3
4
80,00
90,00
100,00
110,00
120,00
130,00
VSL

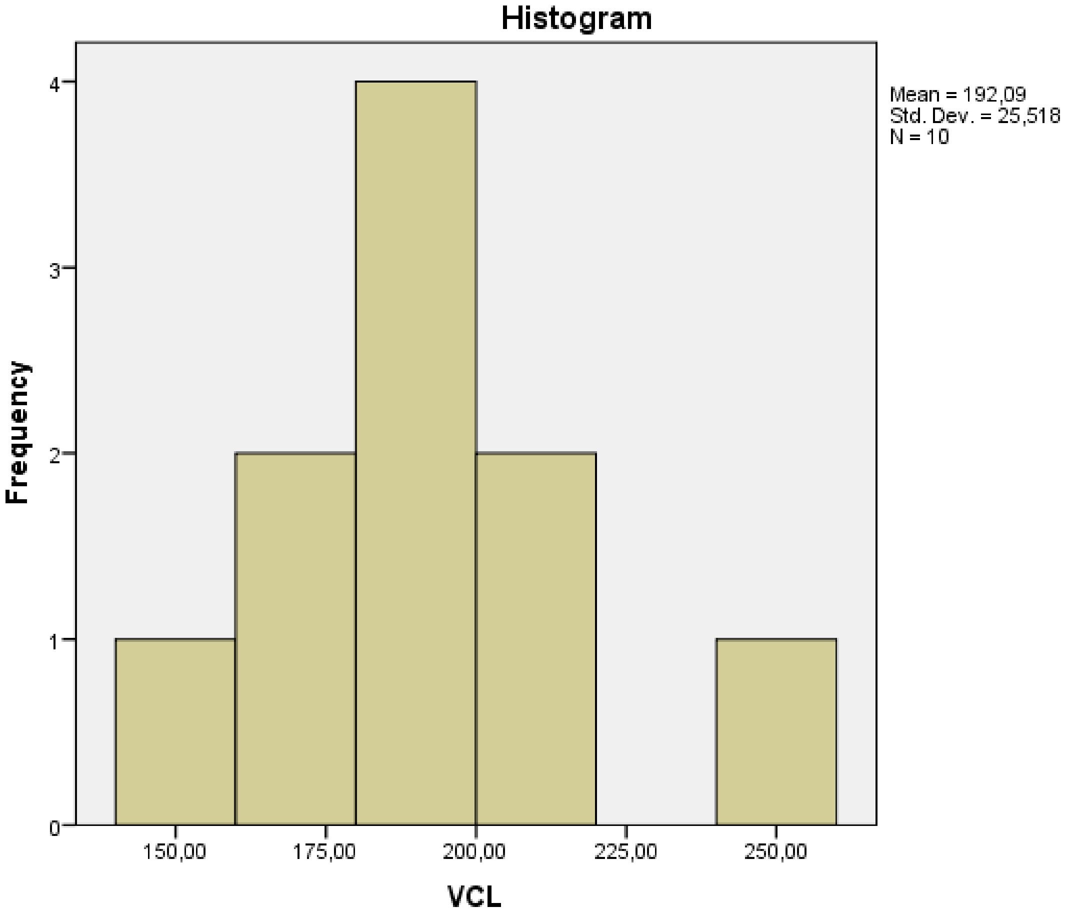

Histogram
Mean = 192,09
Std. Dev. = 25,518
N = 10
Frequency
0
1
2
3
4
150,00
175,00
200,00
225,00
250,00
VCL

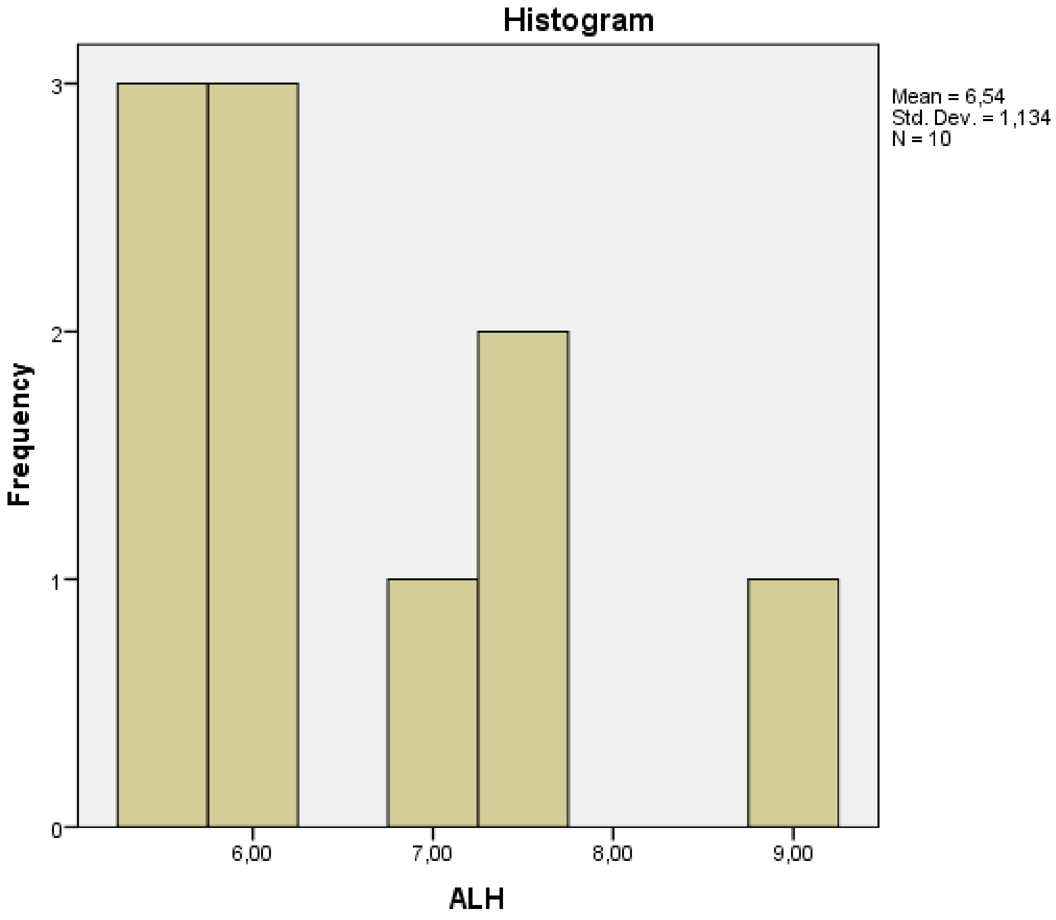
Histogram
Mean = 6,54
Std. Dev. = 1,134
N = 10
Frequency
0
1
2
3
6,00
7,00
8,00
9,00
ALH

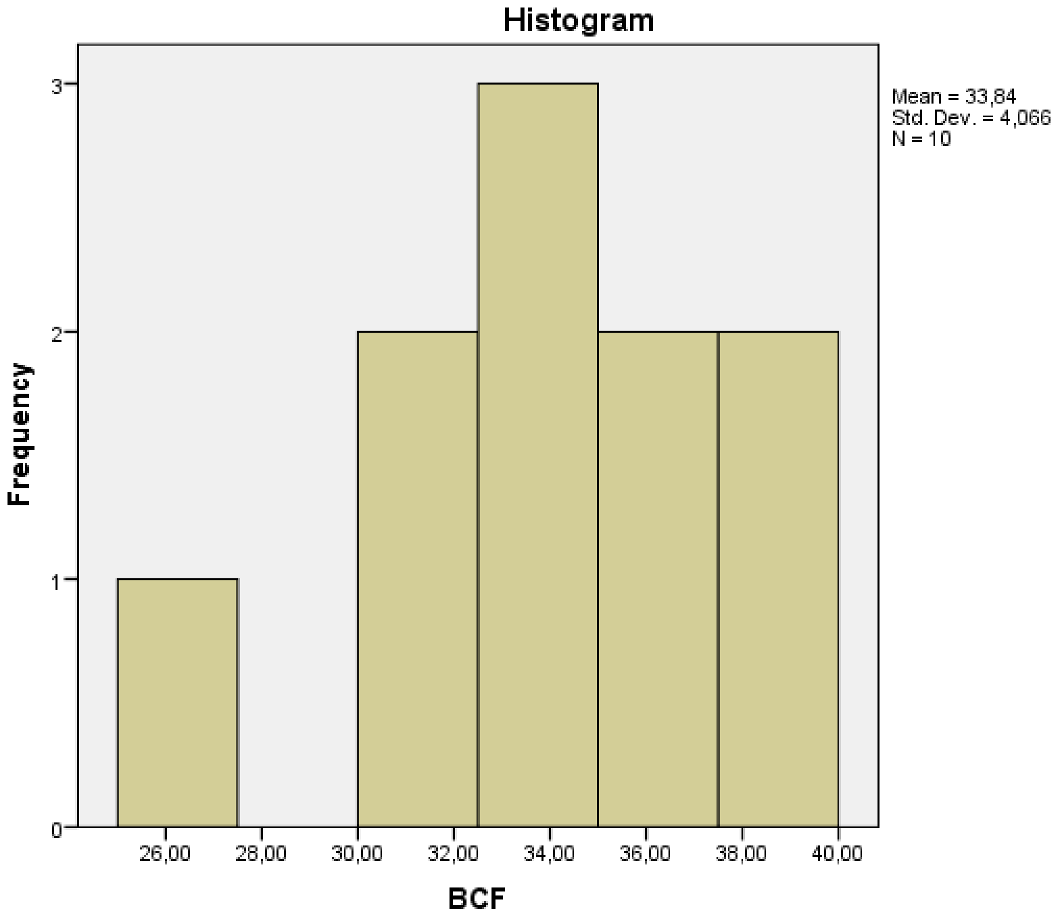
Histogram
Mean = 33,84
Std. Dev. = 4,066
N = 10
Frequency
0
1
2
3
26,00
28,00
30,00
32,00
34,00
36,00
38,00
40,00
BCF

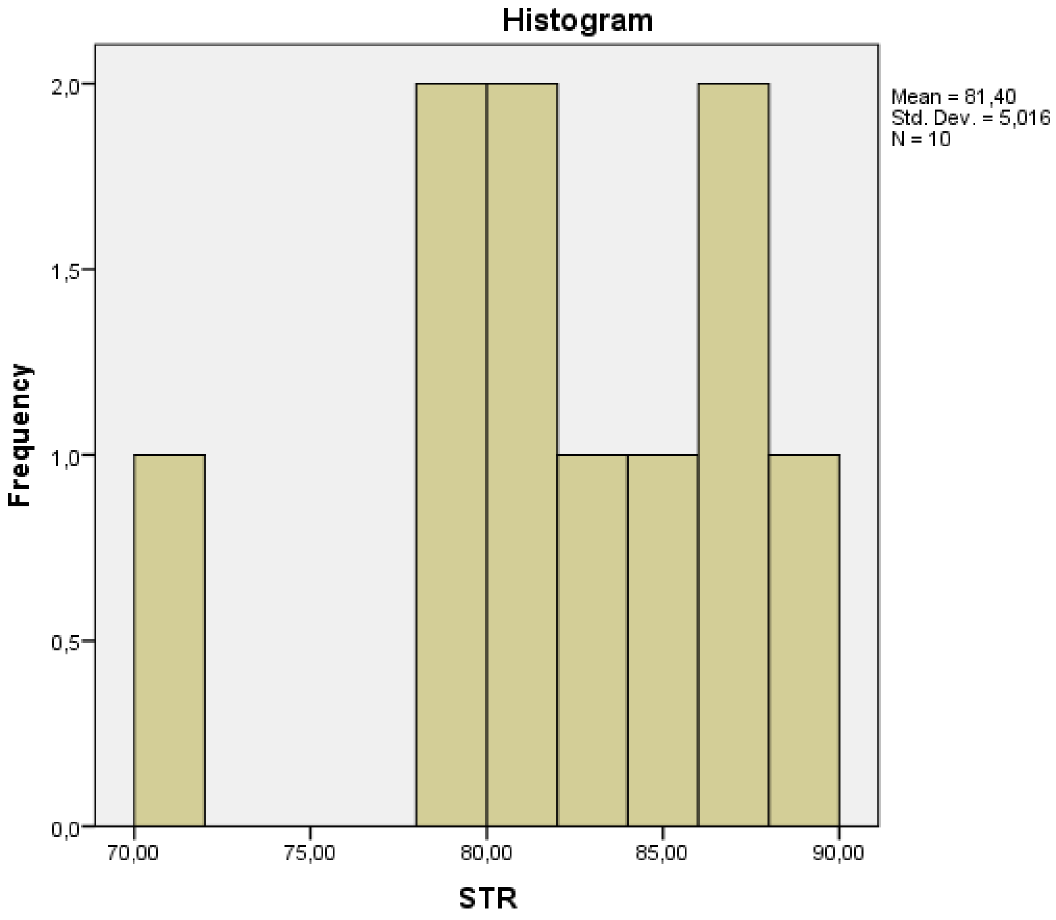
Histogram
Mean = 81,40
Std. Dev. = 5,016
N = 10
Frequency
2,0
1,5
1,0
0,5
0,0
70,00
75,00
80,00
85,00
90,00
STR

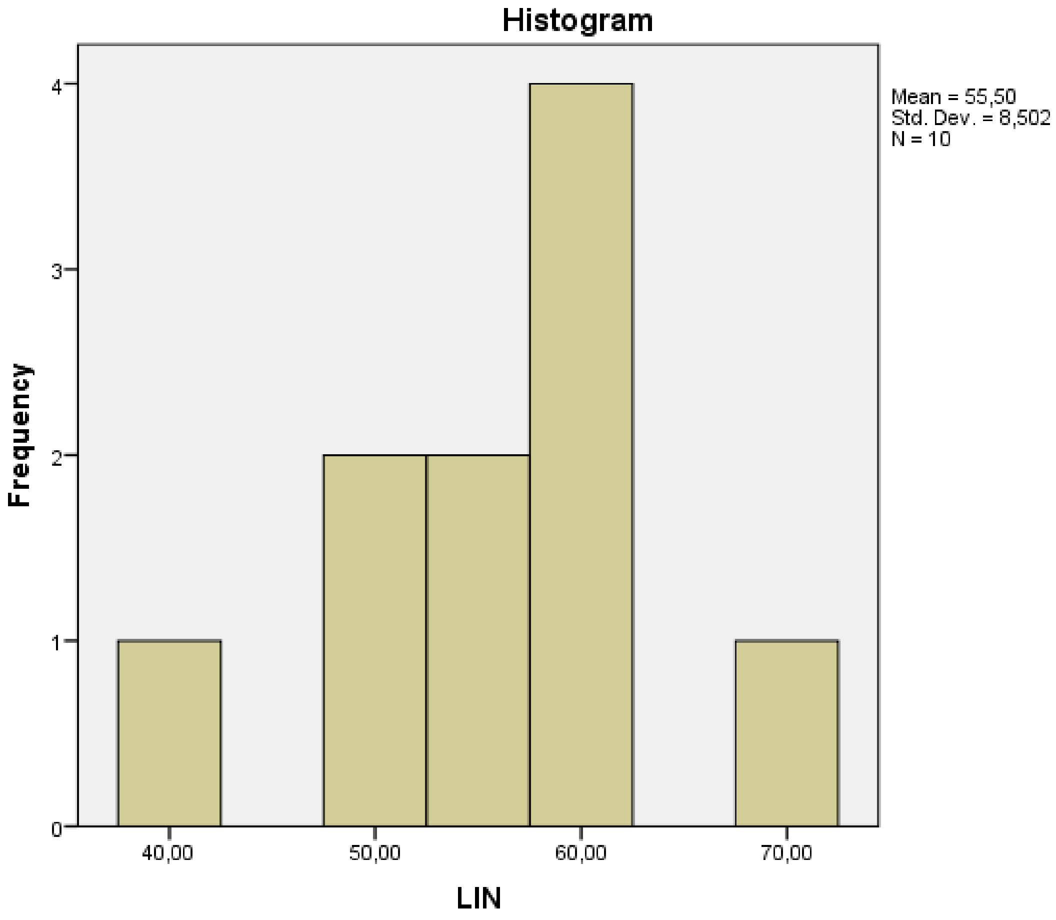

Stem-and-Leaf Plots

```
VAP Stem-and-Leaf Plot for
Grup= 4,00

 Frequency    Stem &  Leaf

     1,00       10 .  2
     4,00       11 .  5599
     2,00       12 .  36
     2,00       13 .  37
     1,00       14 .  0

 Stem width:  10,00
 Each leaf:       1 case(s)
```

```
VSL Stem-and-Leaf Plot for
Grup= 4,00

 Frequency    Stem &  Leaf

     1,00        8 .  6
     4,00        9 .  0556
     3,00       10 .  455
     1,00       11 .  2
     1,00 Extremes    (>=124)

 Stem width:  10,00
 Each leaf:       1 case(s)
```

```
VCL Stem-and-Leaf Plot for
Grup= 4,00

 Frequency    Stem &  Leaf

     7,00        1 .  5778899
     3,00        2 .  014

 Stem width:  100,0
 Each leaf:       1 case(s)
```

```
ALH Stem-and-Leaf Plot for
Grup= 4,00

 Frequency    Stem &  Leaf

     5,00        5 .  56688
     1,00        6 .  2
     3,00        7 .  235
     1,00        8 .  9

 Stem width:   1,00
 Each leaf:       1 case(s)
```

```
BCF Stem-and-Leaf Plot for
Grup= 4,00

 Frequency    Stem &  Leaf

     1,00        2 .  5
     5,00        3 .  02334
     4,00        3 .  6788
```

```
 Stem width:  10,00
 Each leaf:       1 case(s)
```

```
STR Stem-and-Leaf Plot for
Grup= 4,00

 Frequency    Stem &  Leaf

     1,00        7 .  1
     2,00        7 .  88
     4,00        8 .  0124
     3,00        8 .  668

 Stem width:  10,00
 Each leaf:       1 case(s)
```

```
LIN Stem-and-Leaf Plot for
Grup= 4,00

 Frequency    Stem &  Leaf

     1,00        4 .  0
     2,00        4 .  89
     2,00        5 .  33
     1,00        5 .  9
     3,00        6 .  022
     1,00        6 .  9

 Stem width:  10,00
 Each leaf:       1 case(s)
```

Normal Q-Q Plots

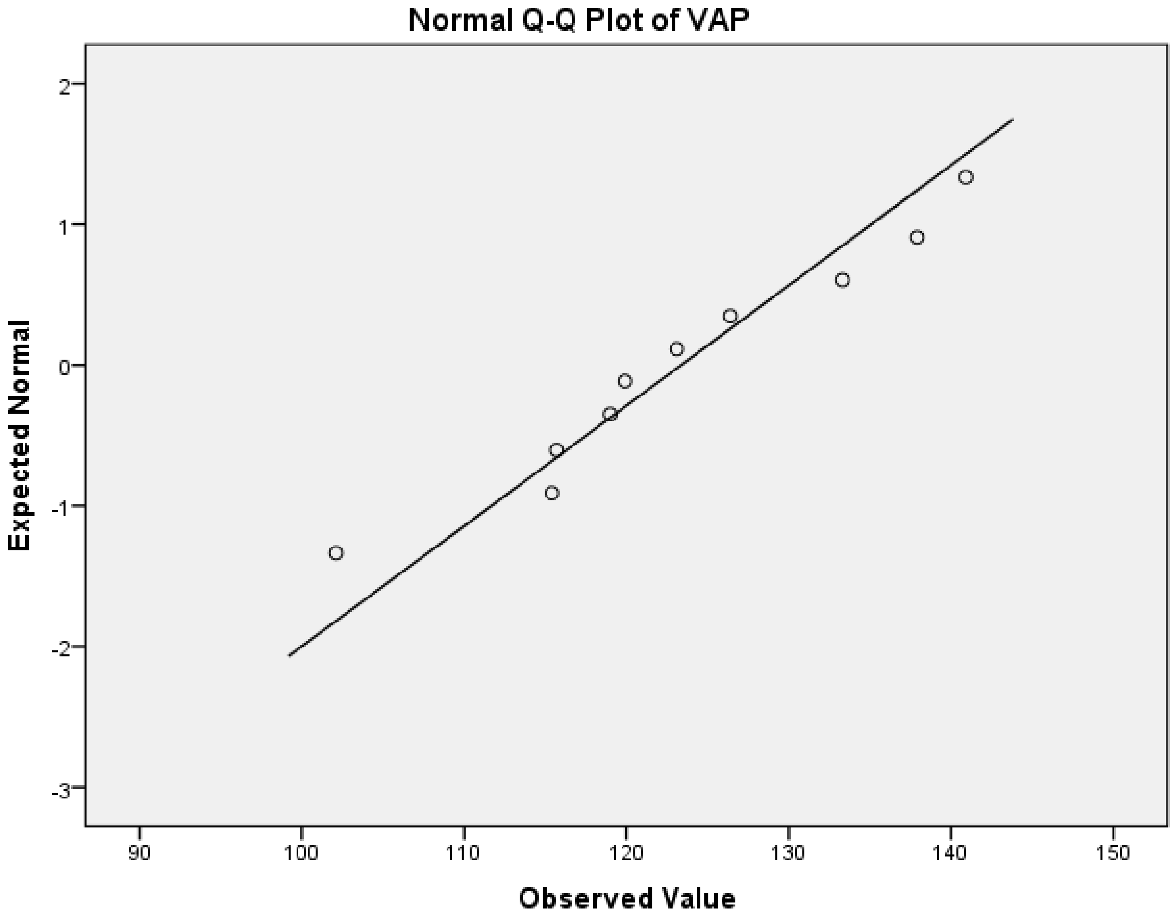
Normal Q-Q Plot of VAP
Expected Normal
2
1
0
-1
-2
-3
90
100
110
120
130
140
150
Observed Value

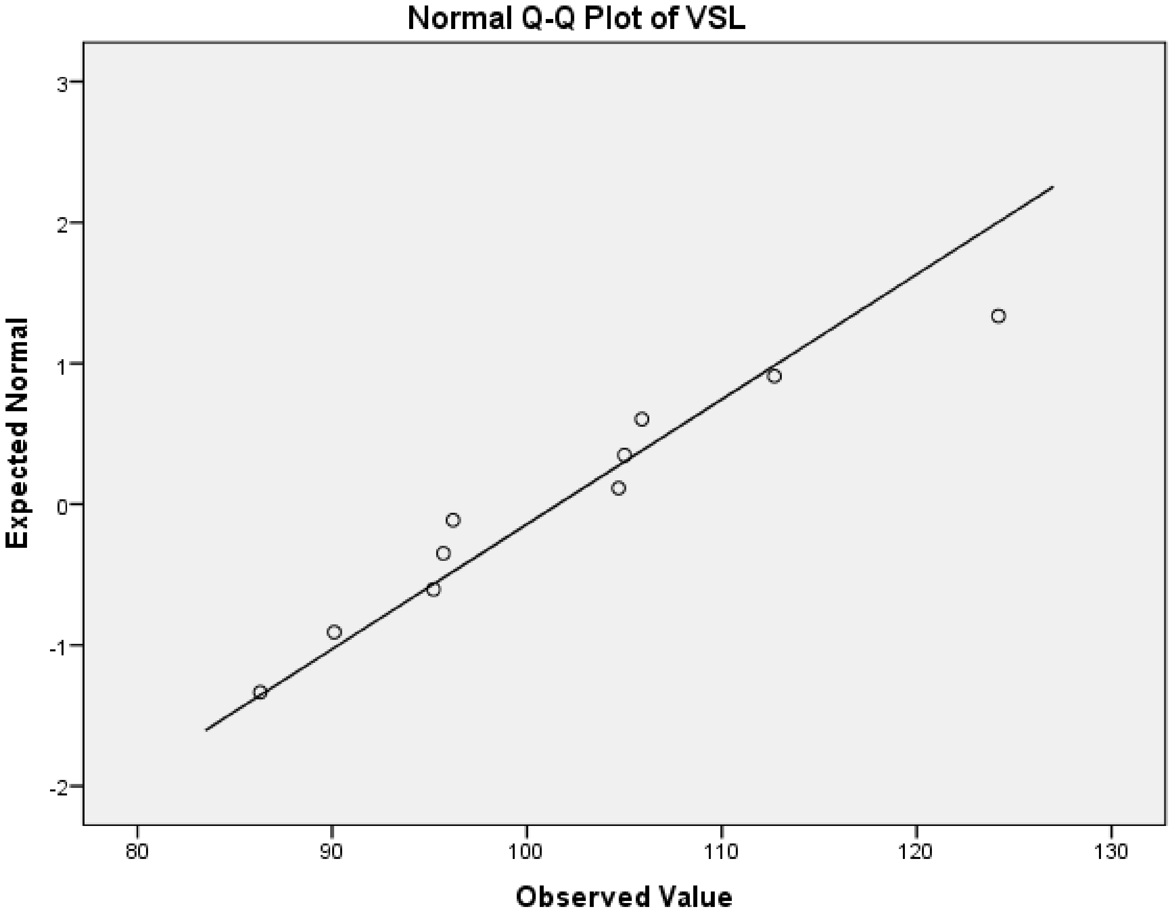

Normal Q-Q Plot of VSL
Expected Normal
Observed Value
3
2
1
0
-1
-2
80
90
100
110
120
130

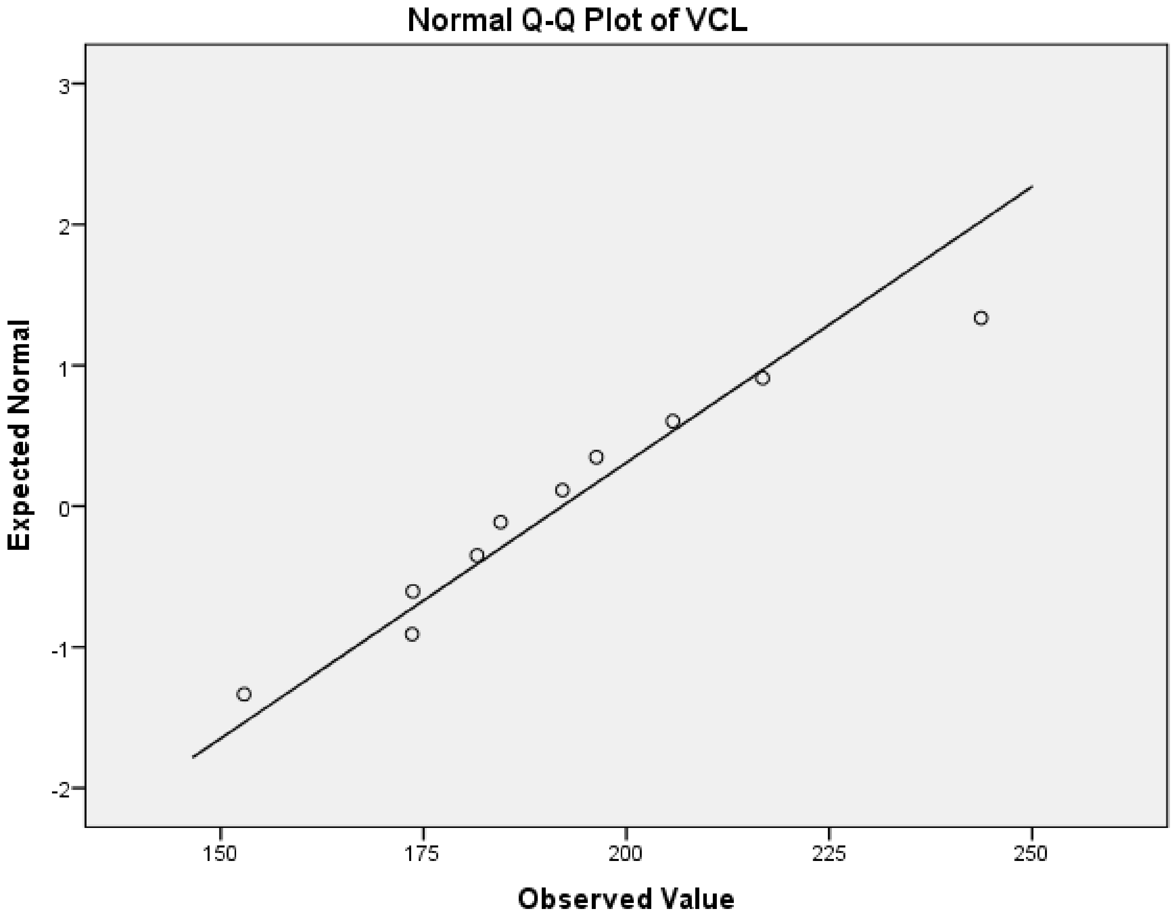
Normal Q-Q Plot of VCL
Expected Normal
3
2
1
0
-1
-2
150
175
200
225
250
Observed Value

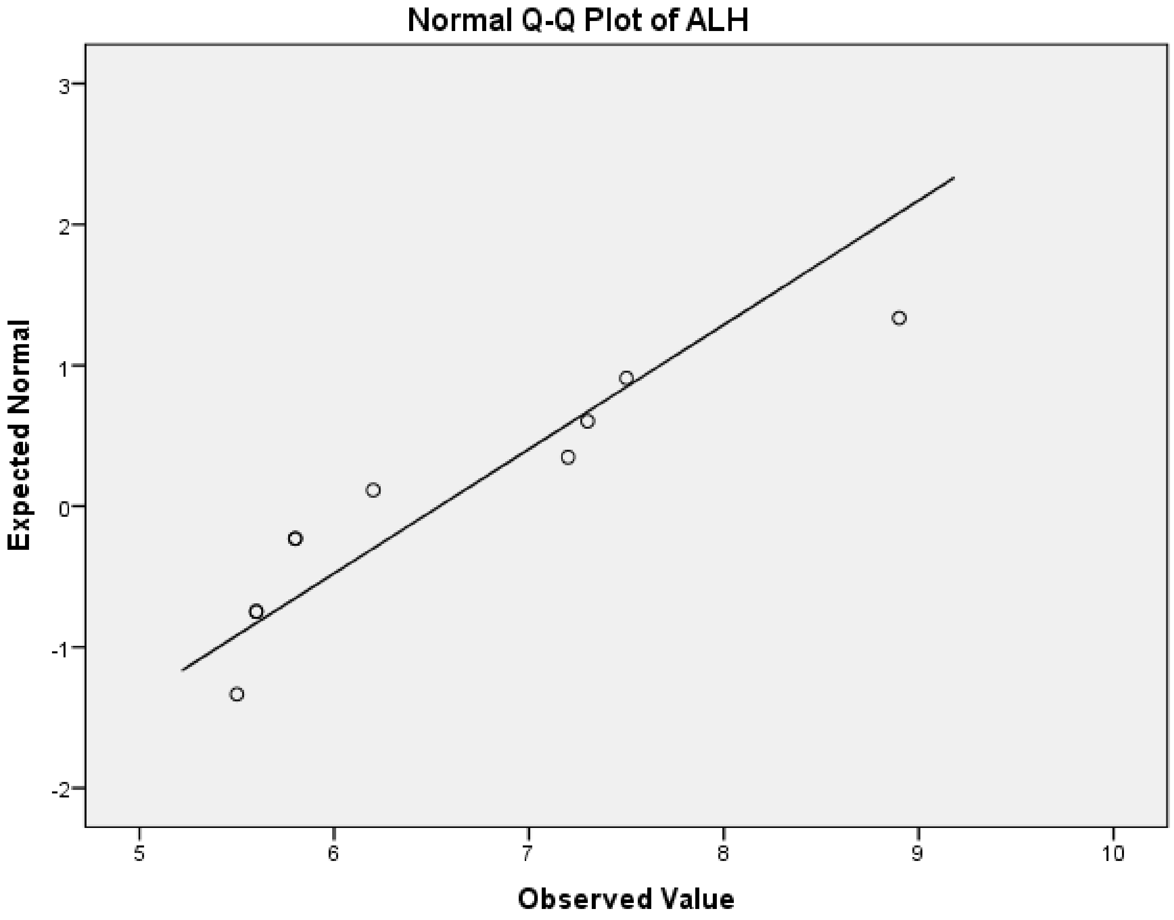
Normal Q-Q Plot of ALH
Expected Normal
Observed Value
3
2
1
0
-1
-2
5
6
7
8
9
10

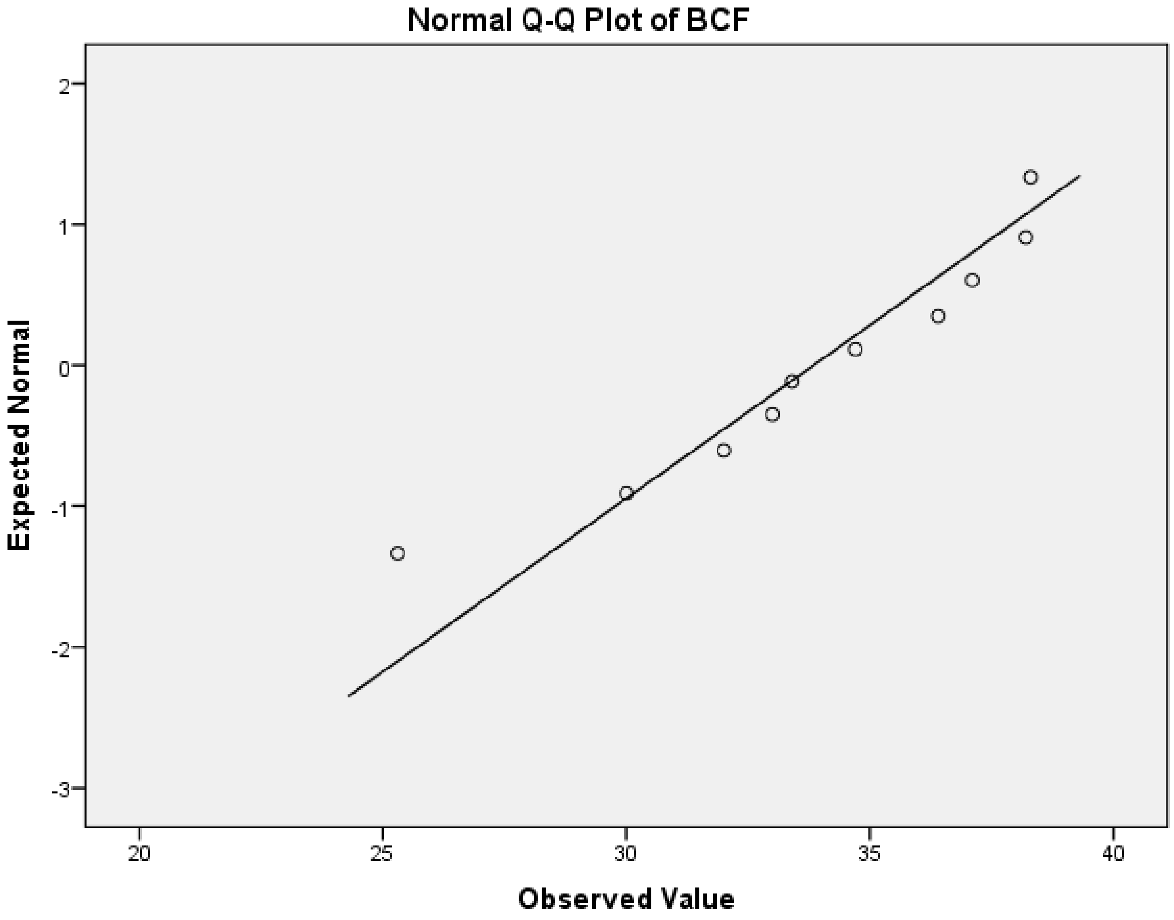
Normal Q-Q Plot of BCF
Expected Normal
Observed Value
2
1
0
-1
-2
-3
20
25
30
35
40

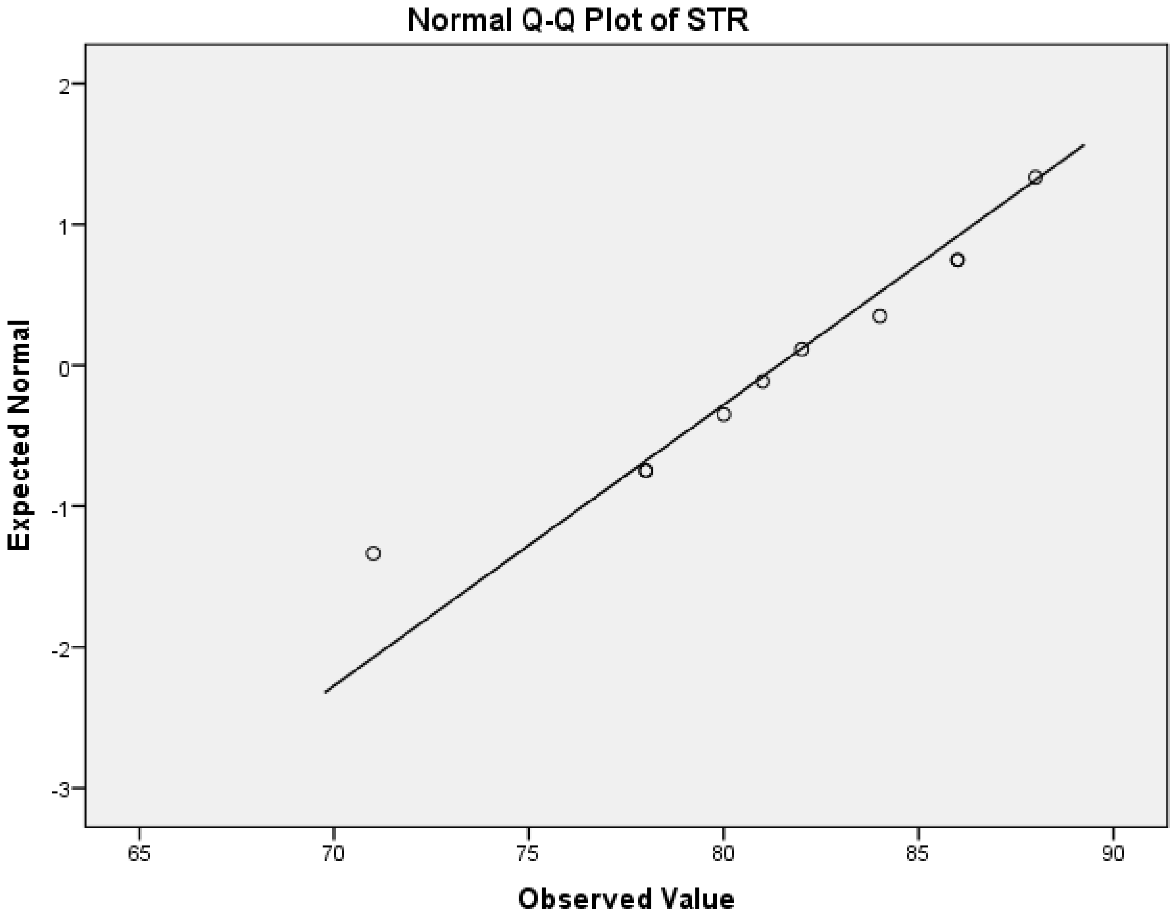

Normal Q-Q Plot of STR
Expected Normal
Observed Value
2
1
0
-1
-2
-3
65
70
75
80
85
90

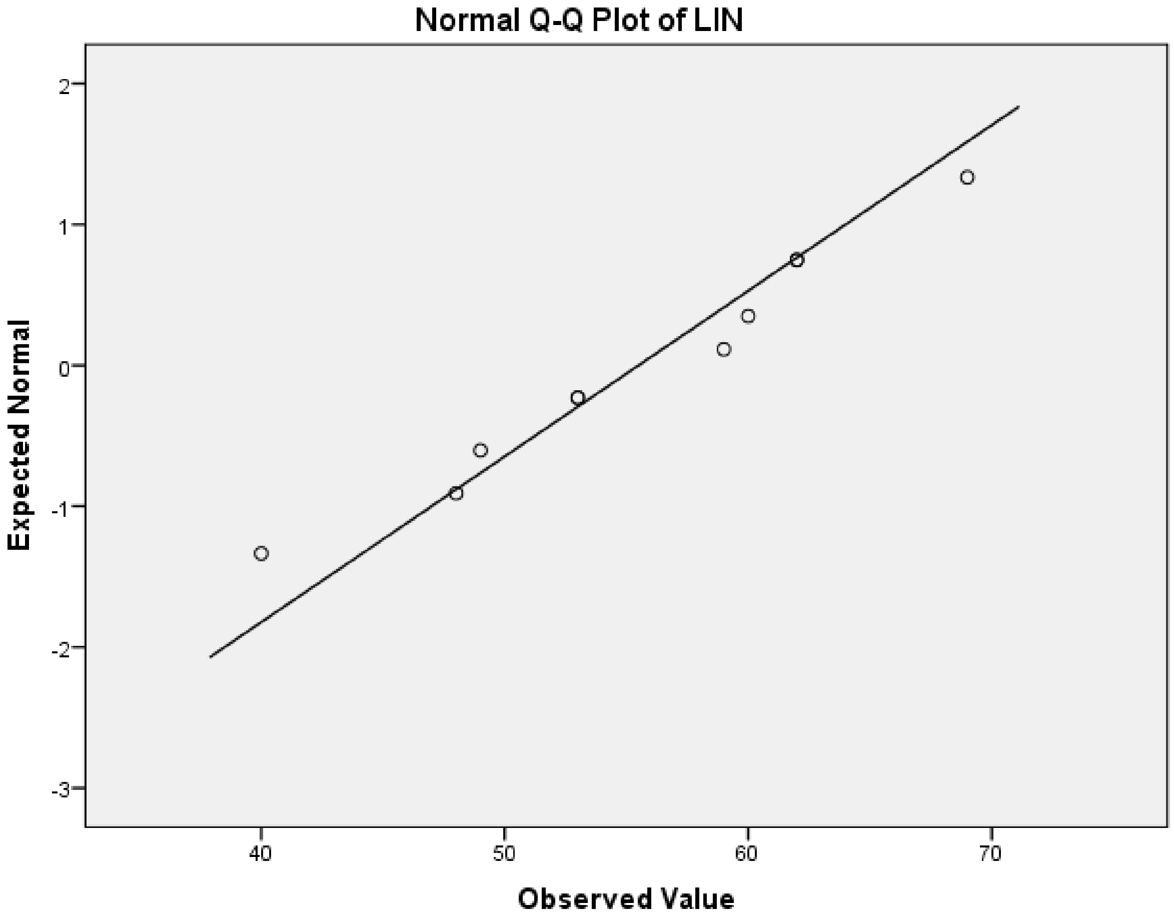

Detrended Normal Q-Q Plots

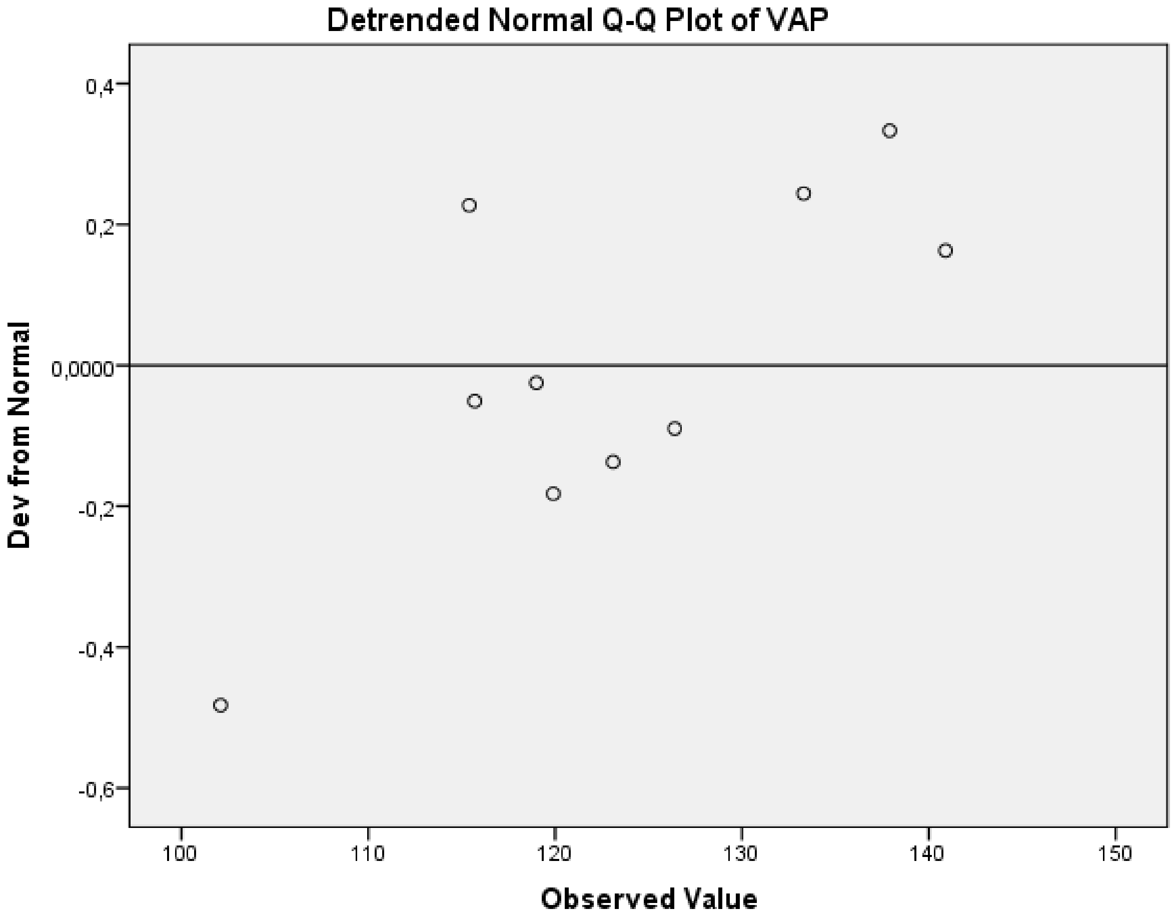
Detrended Normal Q-Q Plot of VAP
Dev from Normal
0,4
0,2
0,0000
-0,2
-0,4
-0,6
100
110
120
130
140
150
Observed Value

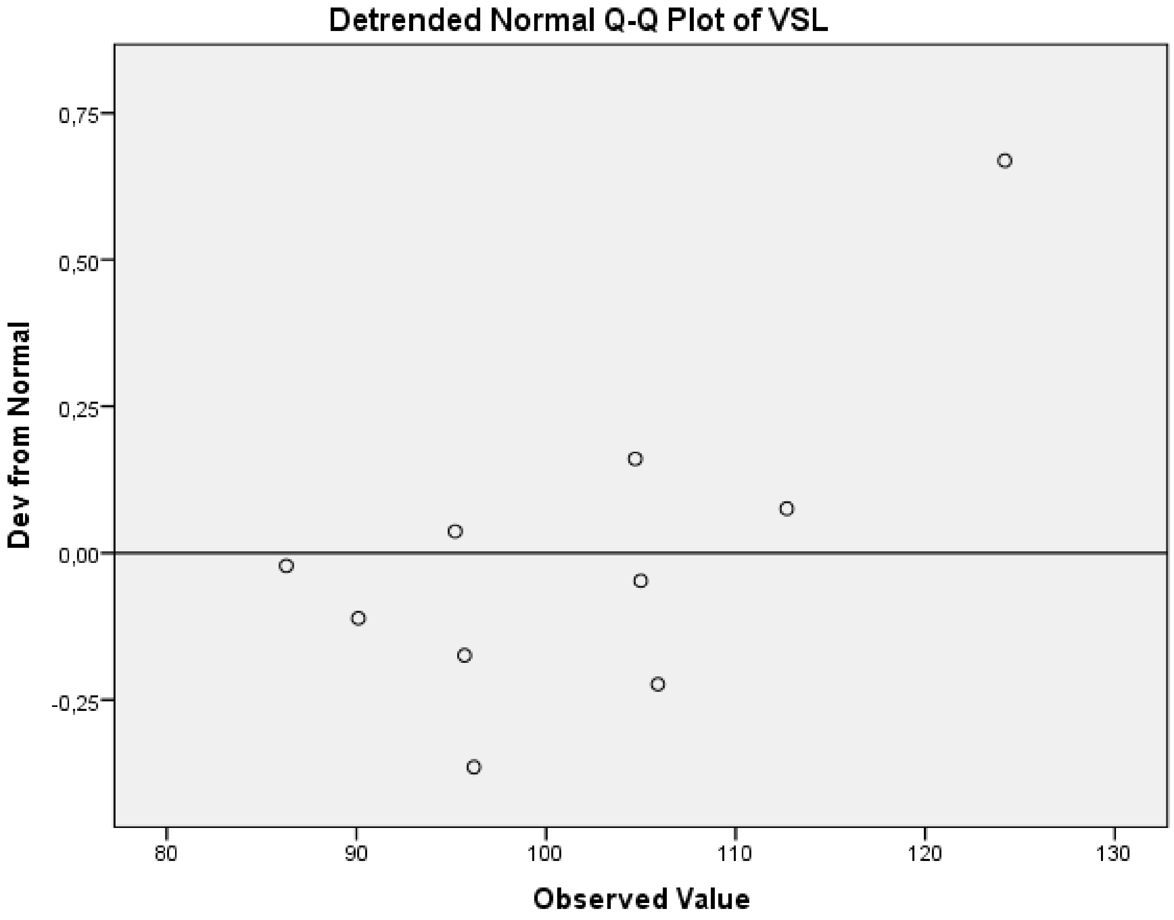
Detrended Normal Q-Q Plot of VSL
Dev from Normal
0,75
0,50
0,25
0,00
-0,25
80
90
100
110
120
130
Observed Value

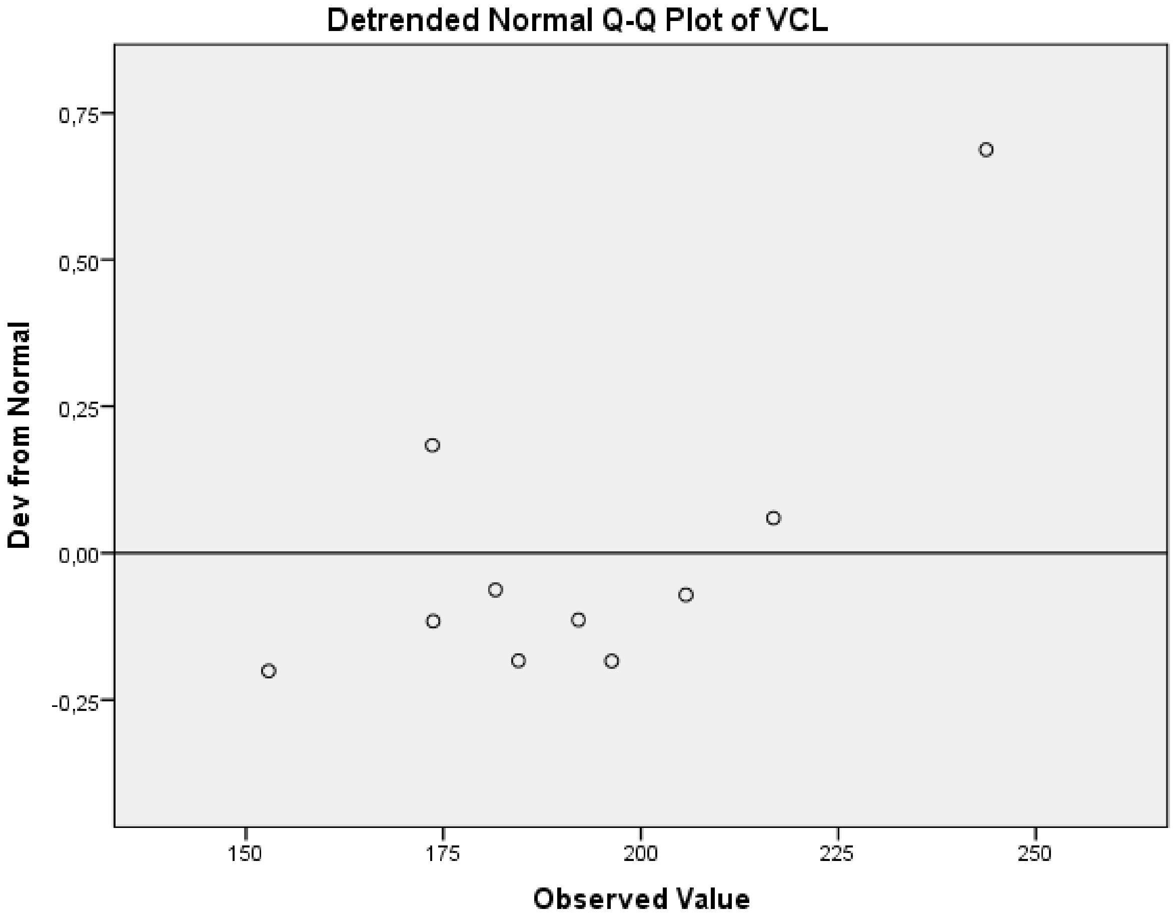

Detrended Normal Q-Q Plot of VCL
Dev from Normal
0,75
0,50
0,25
0,00
-0,25
150
175
200
225
250
Observed Value

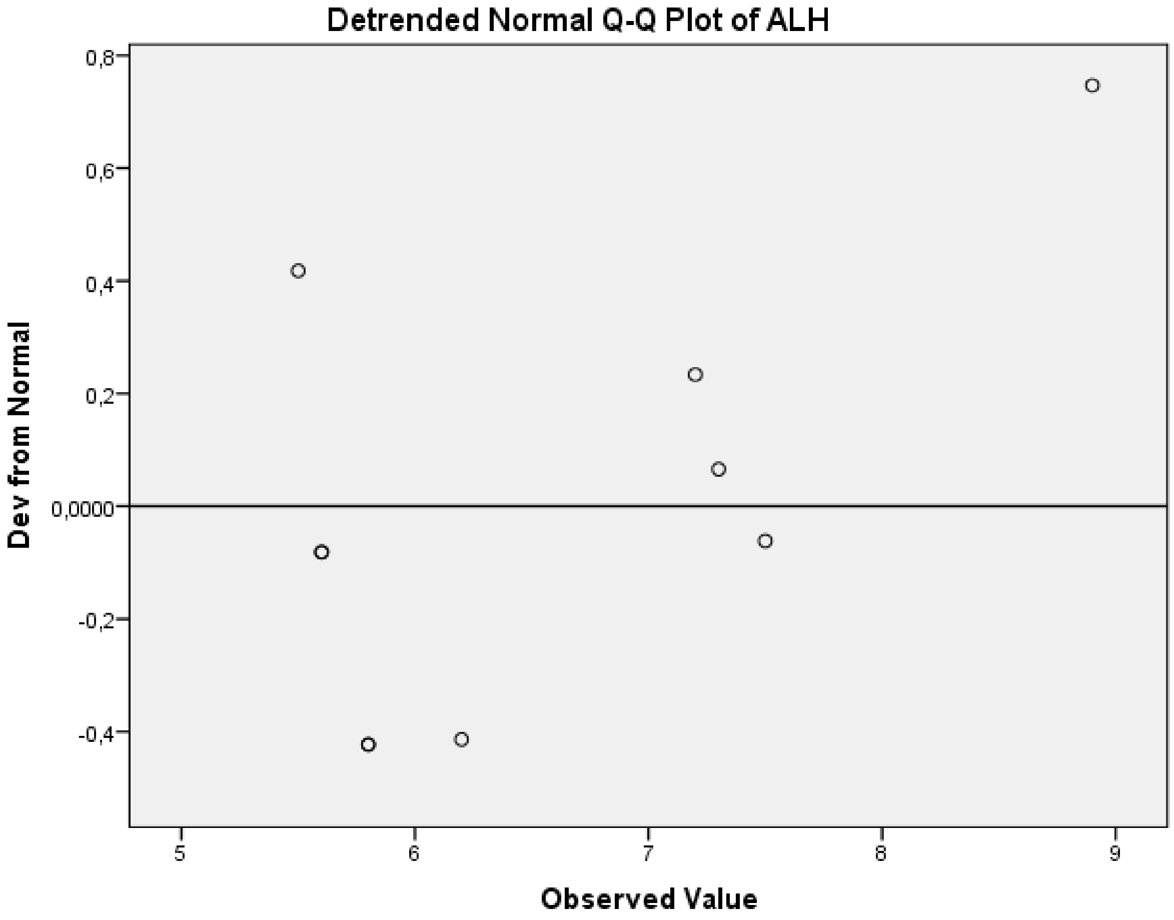
Detrended Normal Q-Q Plot of ALH
Dev from Normal
0,8
0,6
0,4
0,2
0,0000
-0,2
-0,4
5
6
7
8
9
Observed Value

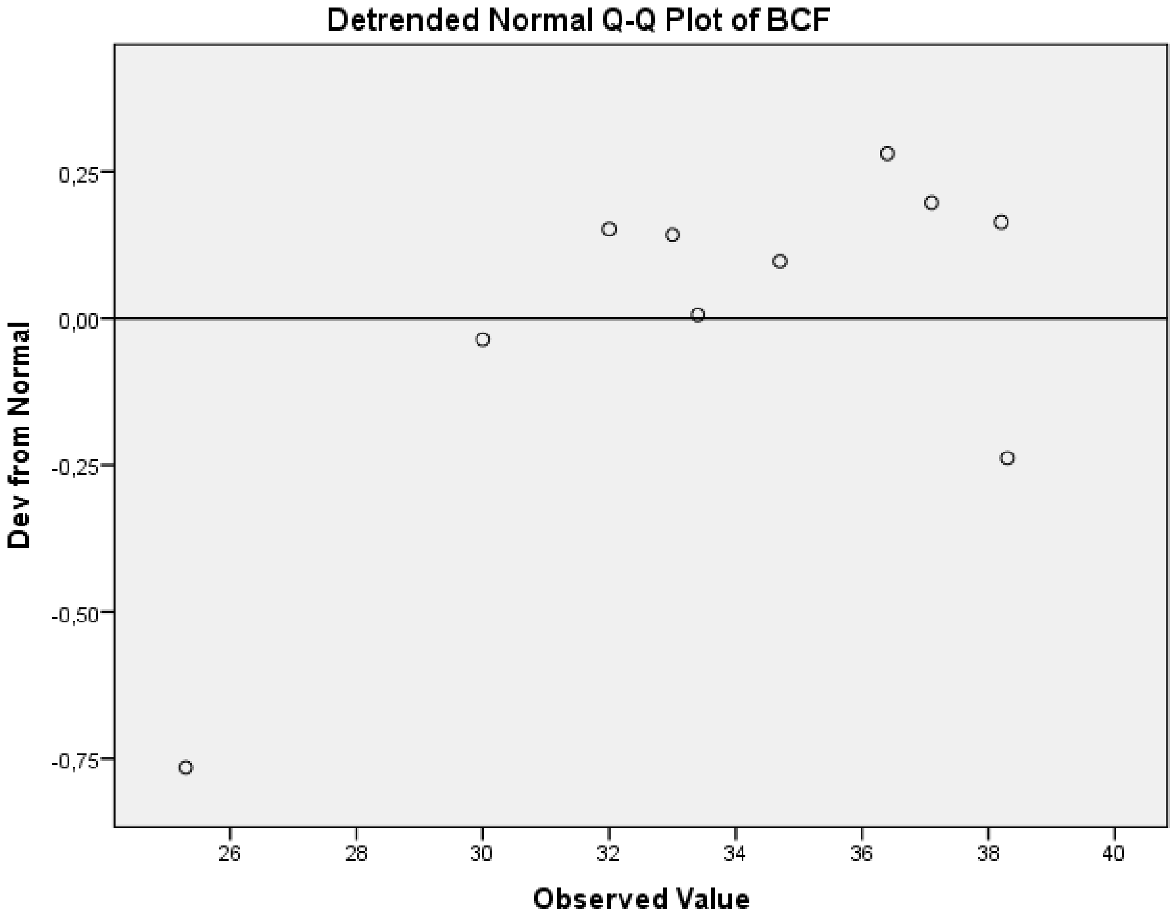
Detrended Normal Q-Q Plot of BCF
Dev from Normal
0,25
0,00
-0,25
-0,50
-0,75
26
28
30
32
34
36
38
40
Observed Value

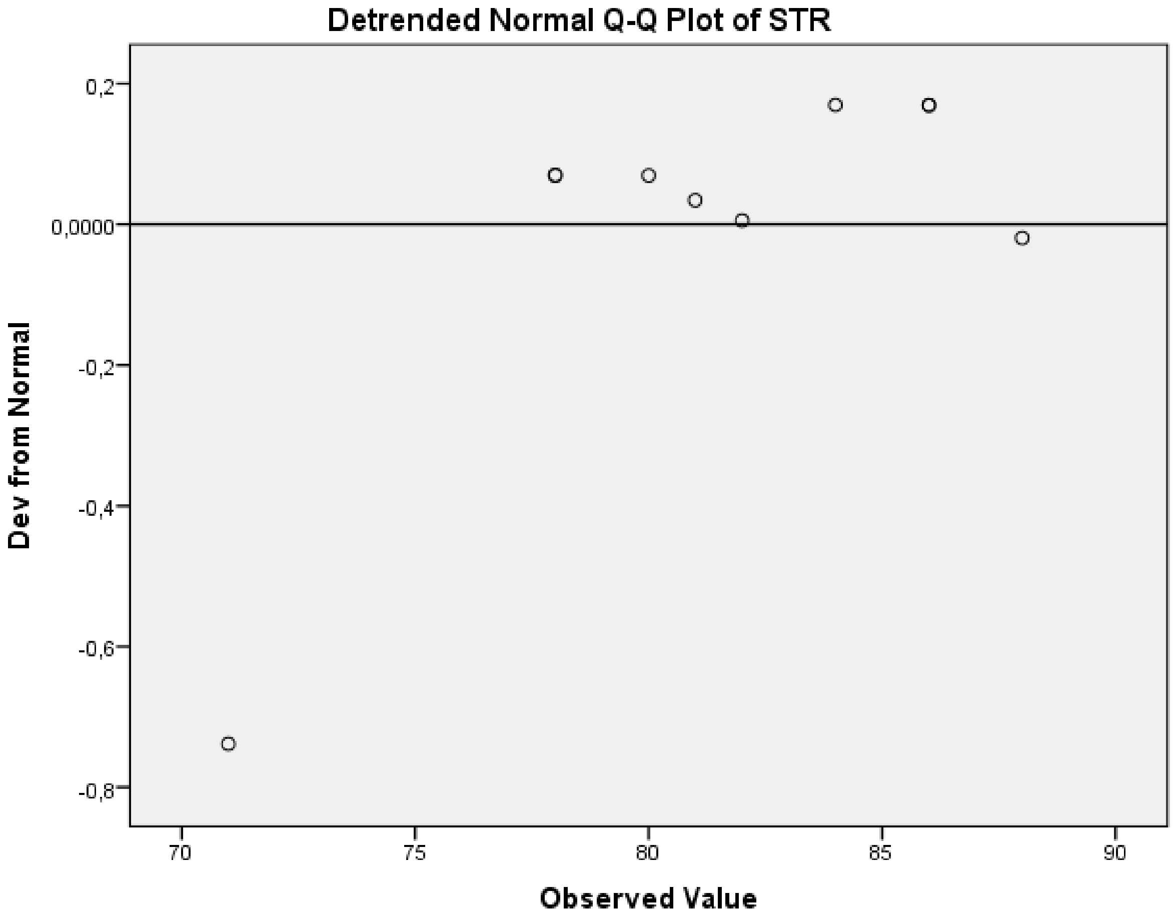
Detrended Normal Q-Q Plot of STR
0,2
0,0000
-0,2
-0,4
-0,6
-0,8
Dev from Normal
70
75
80
85
90
Observed Value

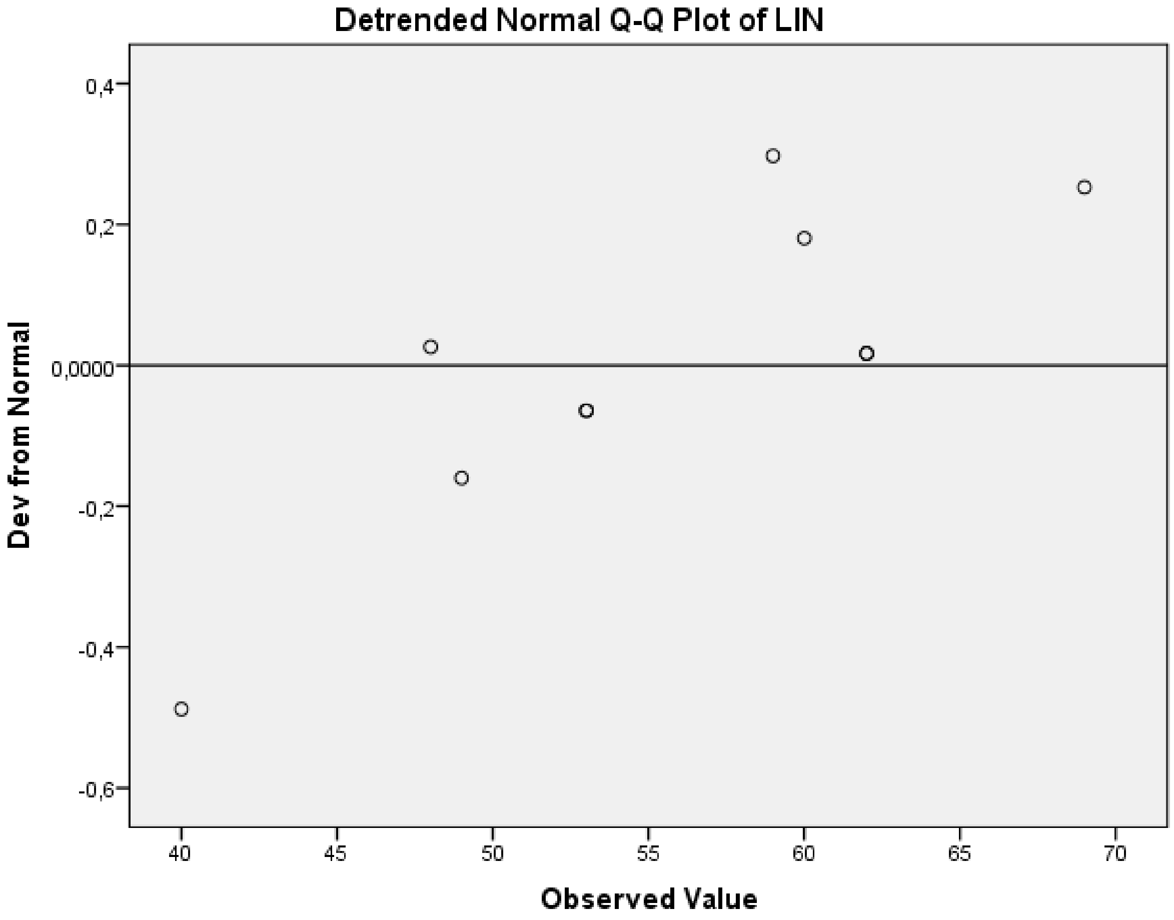

Grup = 5,00

Histograms

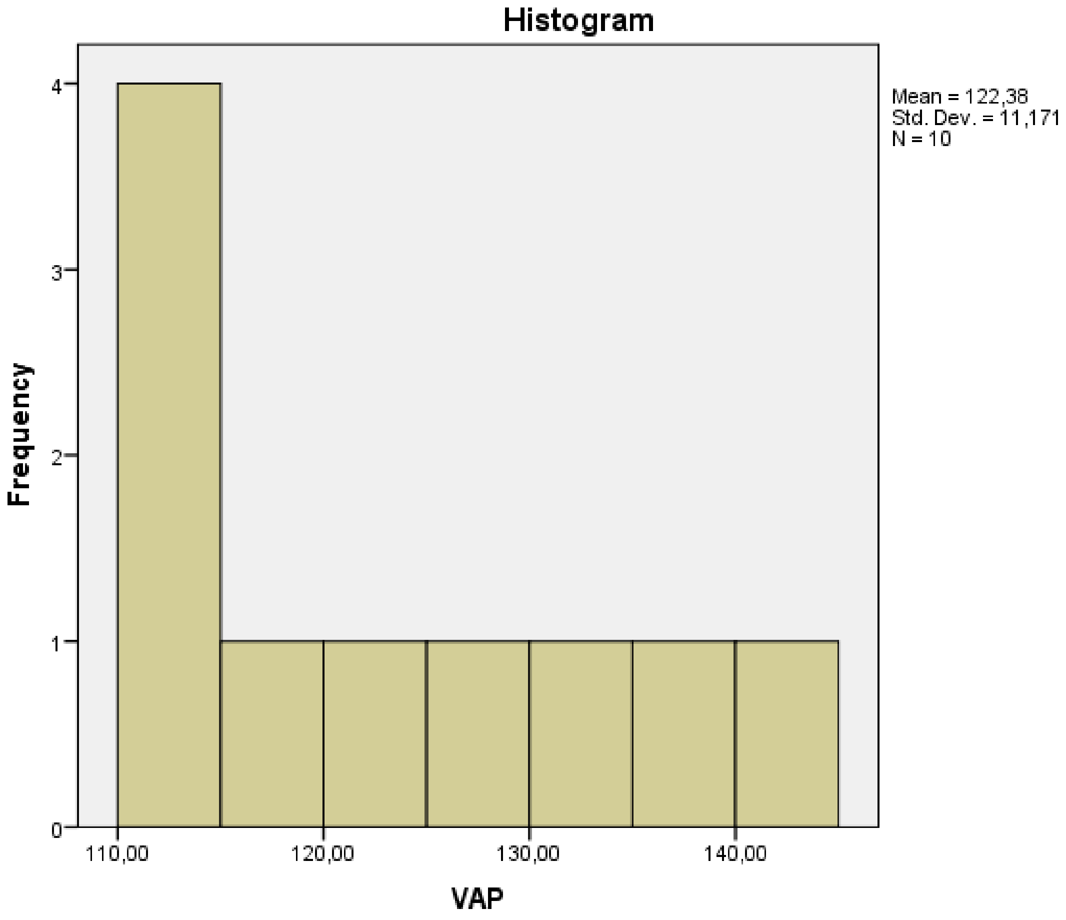

Histogram
Mean = 122,38
Std. Dev. = 11,171
N = 10
Frequency
4
3
2
1
0
110,00
120,00
130,00
140,00
VAP

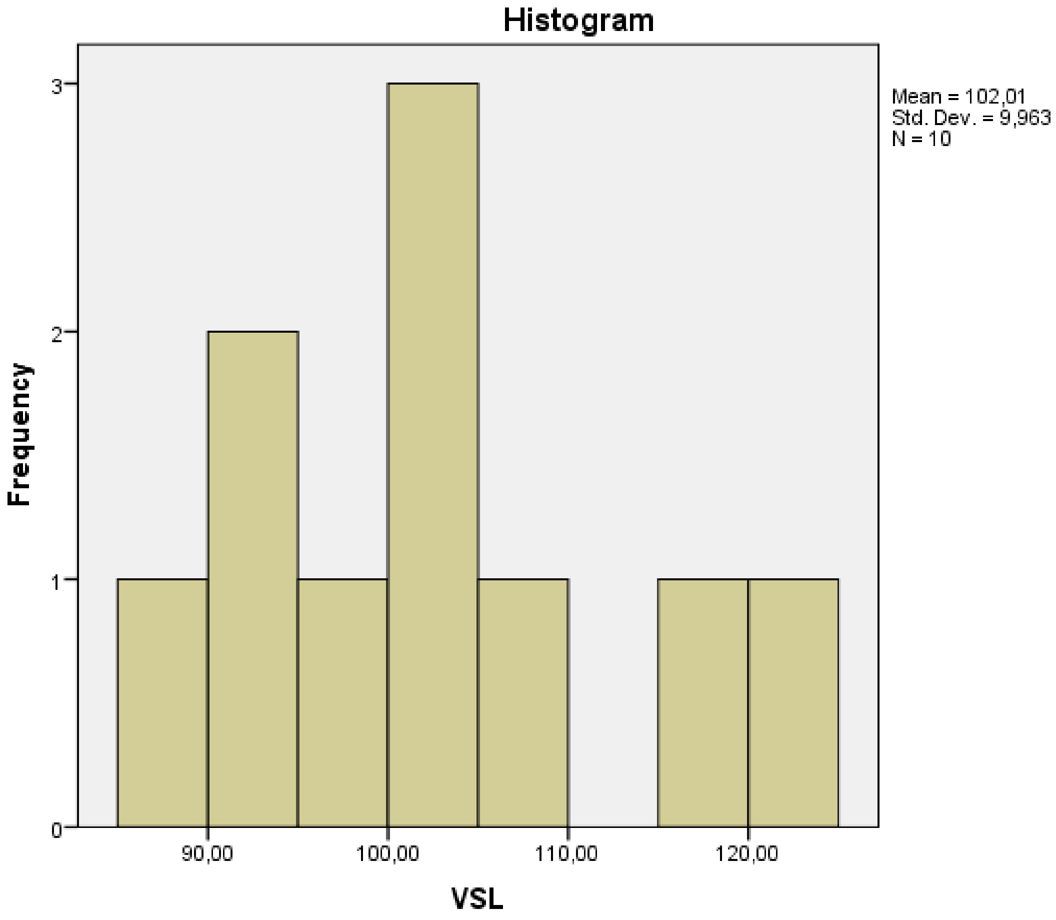
Histogram
Mean = 102,01
Std. Dev. = 9,963
N = 10
Frequency
3
2
1
0
90,00
100,00
110,00
120,00
VSL

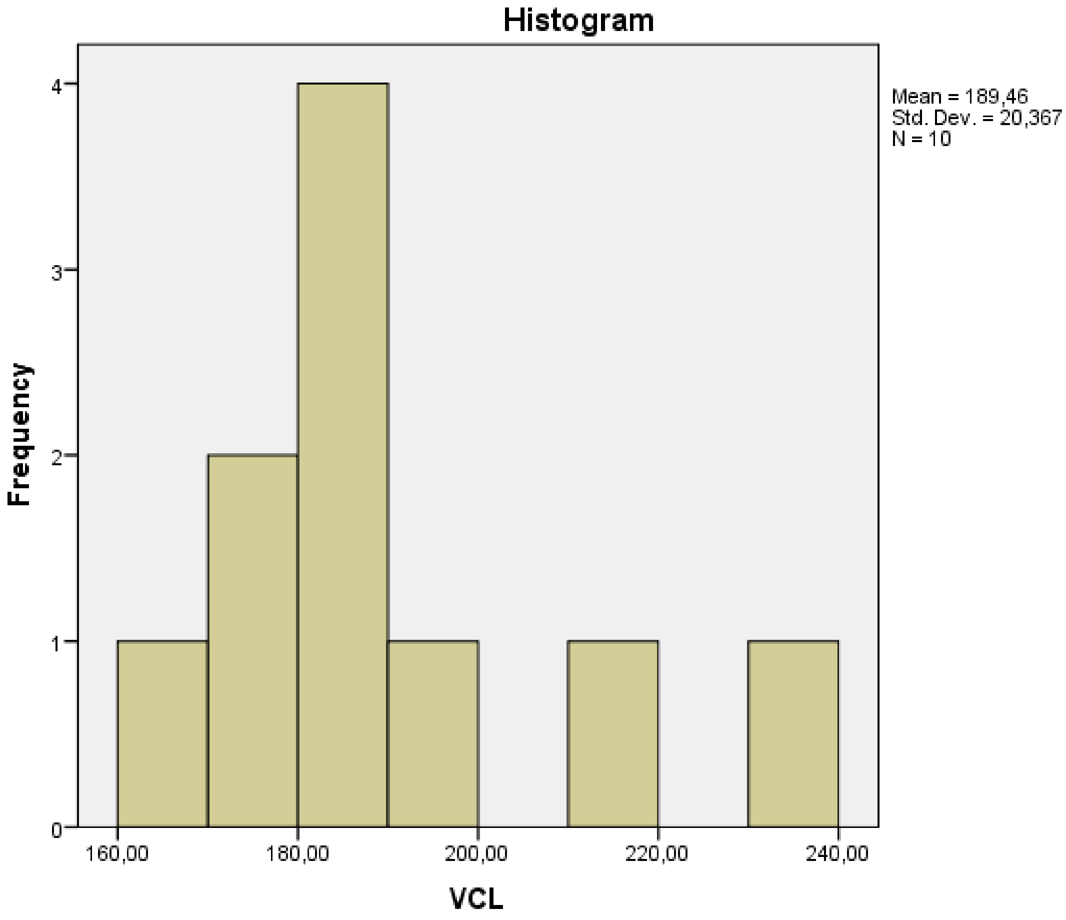
Histogram
Mean = 189,46
Std. Dev. = 20,367
N = 10
Frequency
0
1
2
3
4
160,00
180,00
200,00
220,00
240,00
VCL

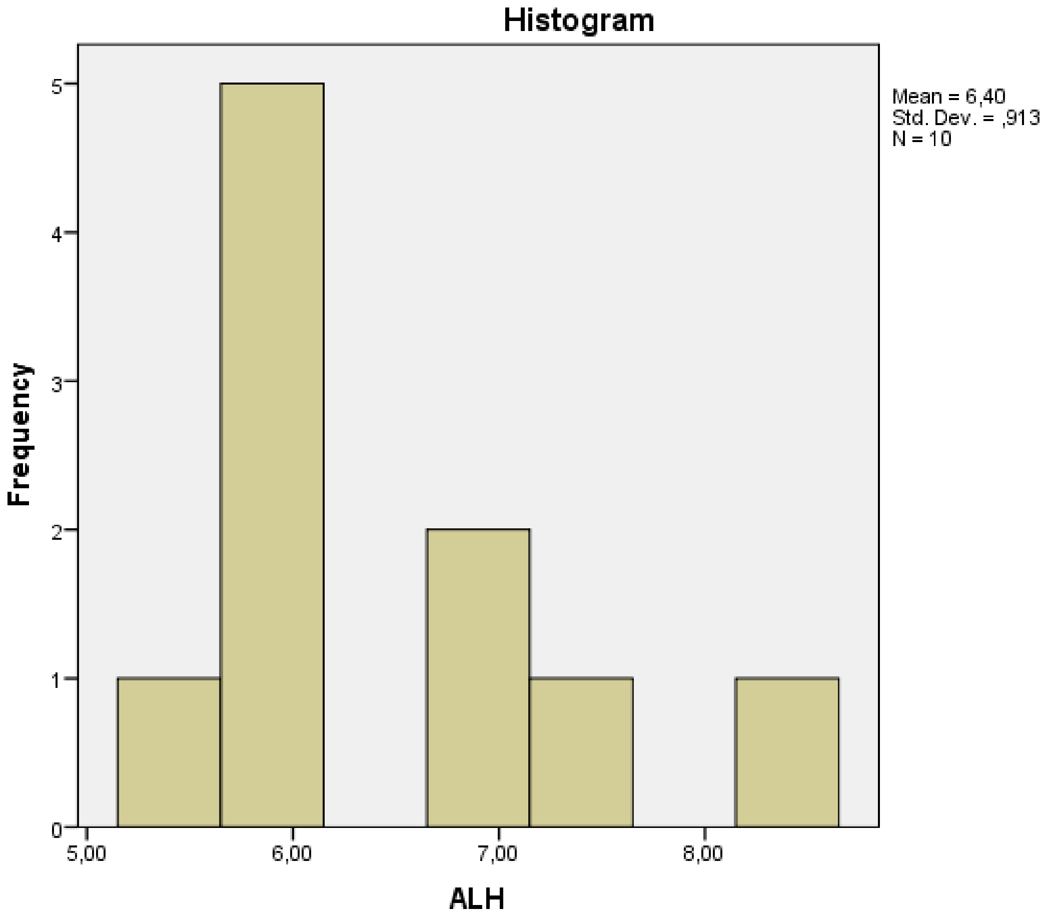

Histogram
Mean = 6,40
Std. Dev. = ,913
N = 10
Frequency
0
1
2
3
4
5
5,00
6,00
7,00
8,00
ALH

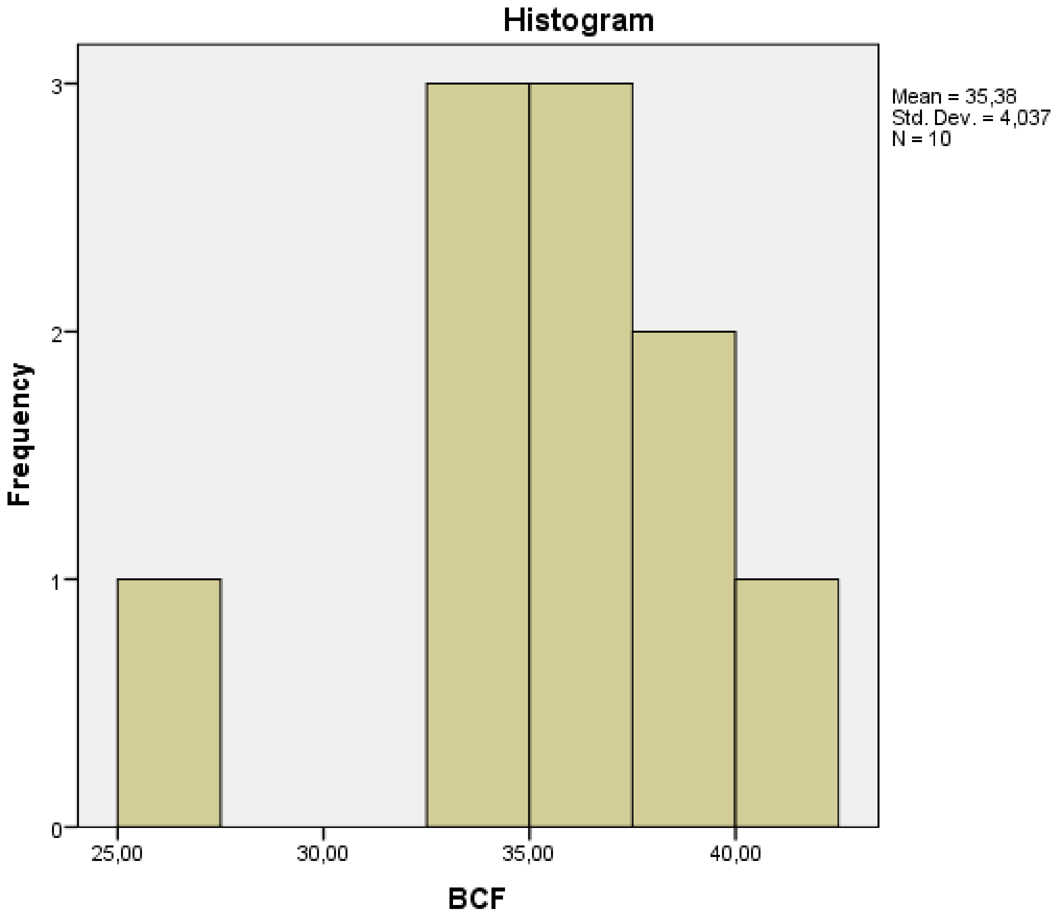
Histogram
Mean = 35,38
Std. Dev. = 4,037
N = 10
Frequency
0
1
2
3
25,00
30,00
35,00
40,00
BCF

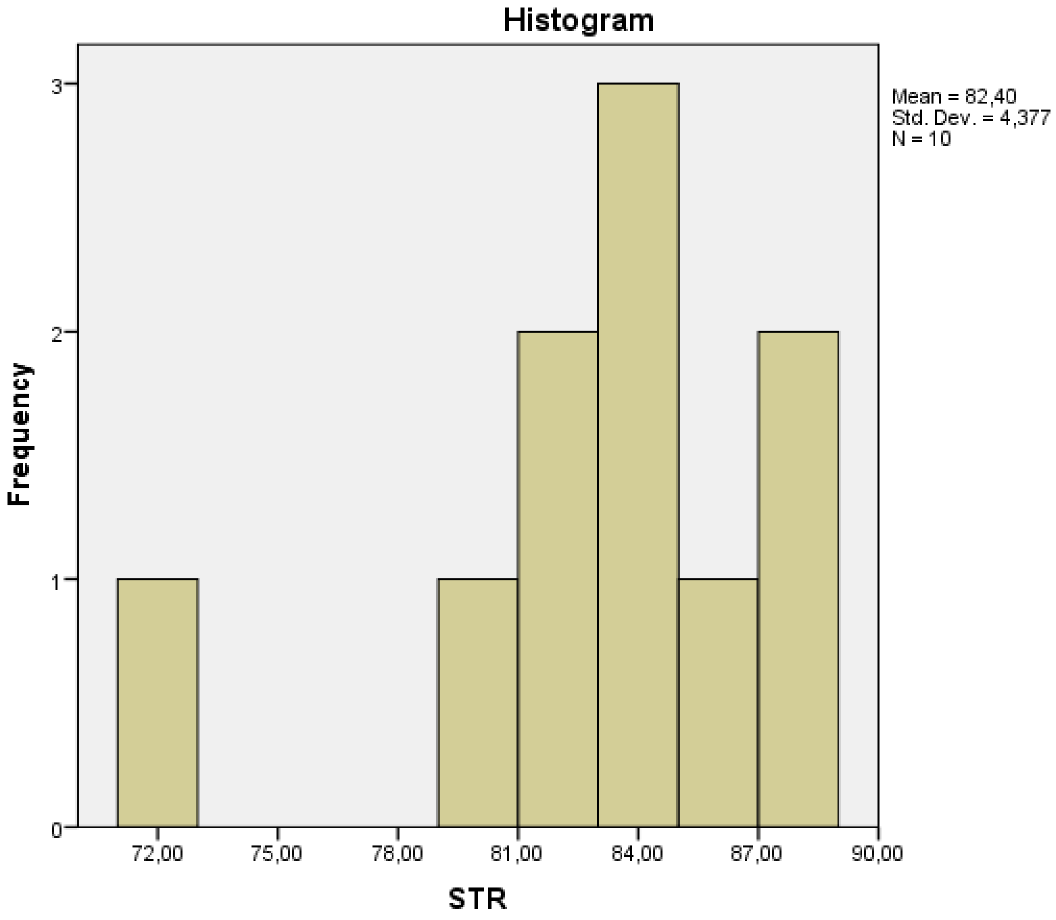
Histogram
Mean = 82,40
Std. Dev. = 4,377
N = 10
Frequency
0
1
2
3
72,00
75,00
78,00
81,00
84,00
87,00
90,00
STR

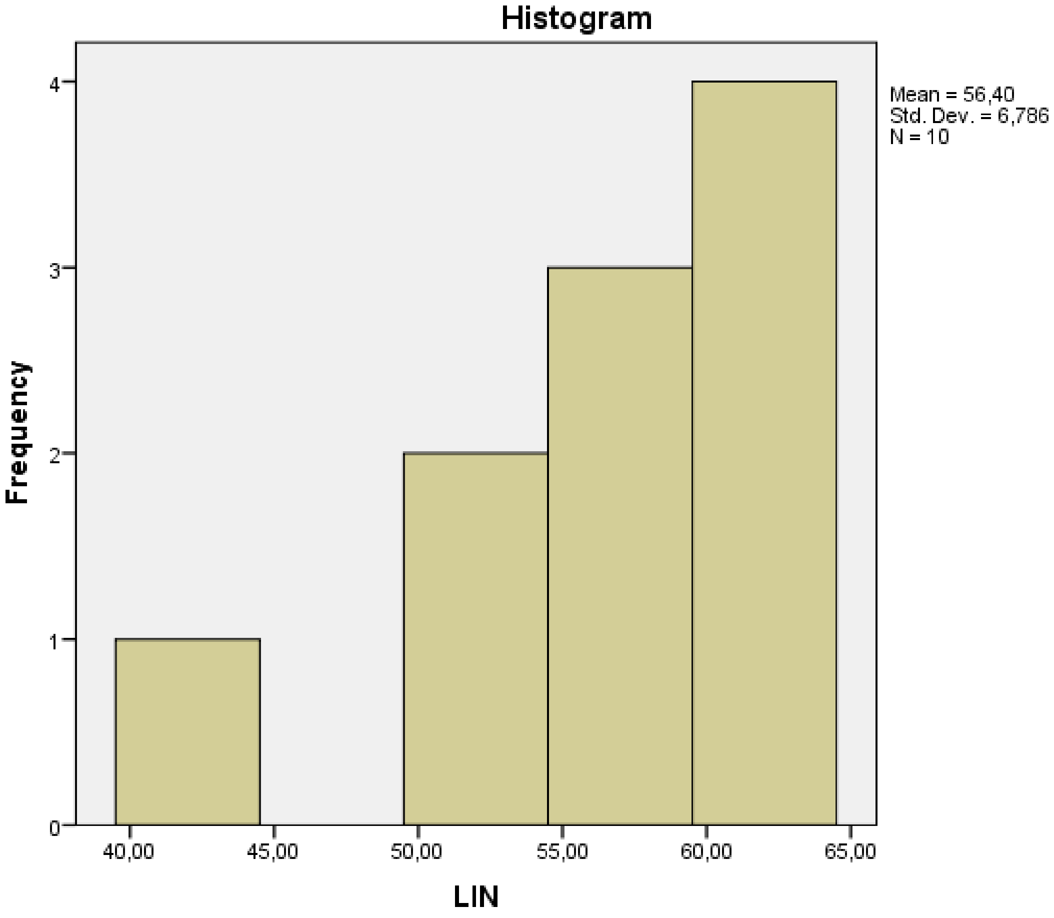

Stem-and-Leaf Plots

```
VAP Stem-and-Leaf Plot for
Grup= 5,00

 Frequency    Stem &  Leaf

     5,00       11 .  12235
     2,00       12 .  26
     2,00       13 .  07
     1,00       14 .  1

 Stem width:  10,00
 Each leaf:       1 case(s)
```

```
VSL Stem-and-Leaf Plot for
Grup= 5,00

 Frequency    Stem &  Leaf

     1,00        8 .  9
     3,00        9 .  139
     4,00       10 .  0225
     1,00       11 .  5
     1,00       12 .  0

 Stem width:  10,00
 Each leaf:       1 case(s)
```

```
VCL Stem-and-Leaf Plot for
Grup= 5,00

 Frequency    Stem &  Leaf

     1,00       16 .  8
     2,00       17 .  13
     4,00       18 .  1358
     1,00       19 .  2
      ,00       20 .
     1,00       21 .  9
     1,00 Extremes    (>=231)

 Stem width:  10,00
 Each leaf:       1 case(s)
```

```
ALH Stem-and-Leaf Plot for
Grup= 5,00

 Frequency    Stem &  Leaf

     5,00        5 .  47889
     3,00        6 .  179
     1,00        7 .  4
     1,00        8 .  3

 Stem width:   1,00
 Each leaf:       1 case(s)
```

```
BCF Stem-and-Leaf Plot for
Grup= 5,00

 Frequency    Stem &  Leaf
```

```
     1,00 Extremes    (=<26)
     3,00        3 .  444
     5,00        3 .  55678
     1,00        4 .  0

 Stem width:  10,00
 Each leaf:       1 case(s)
```

```
STR Stem-and-Leaf Plot for
Grup= 5,00

 Frequency    Stem &  Leaf

     1,00 Extremes    (=<72)
     2,00        8 .  01
     4,00        8 .  2333
      ,00        8 .
     3,00        8 .  677

 Stem width:  10,00
 Each leaf:       1 case(s)
```

```
LIN Stem-and-Leaf Plot for
Grup= 5,00

 Frequency    Stem &  Leaf

     1,00        4 .  2
      ,00        4 .
     2,00        5 .  01
     3,00        5 .  699
     4,00        6 .  0124

 Stem width:  10,00
 Each leaf:       1 case(s)
```

Normal Q-Q Plots

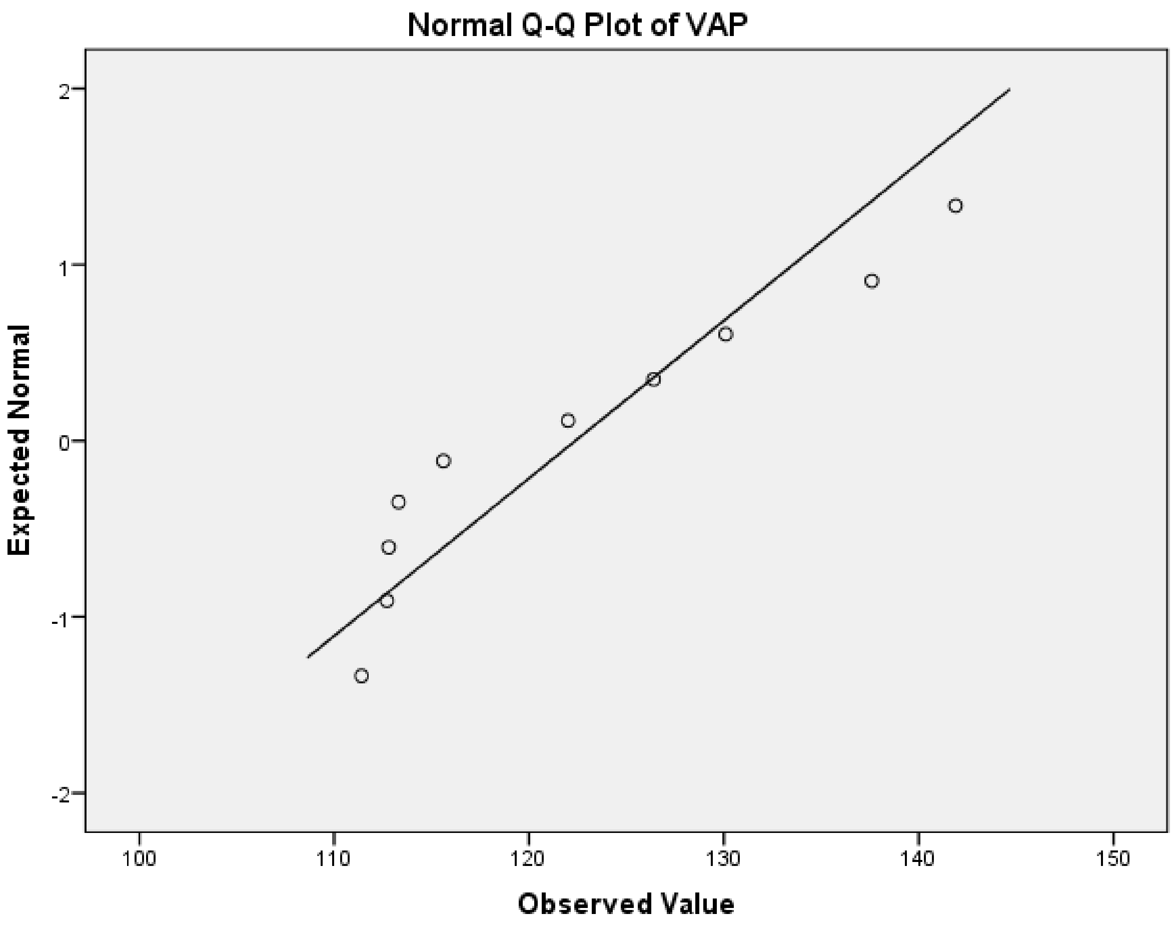

Normal Q-Q Plot of VAP
Expected Normal
Observed Value
2
1
0
-1
-2
100
110
120
130
140
150

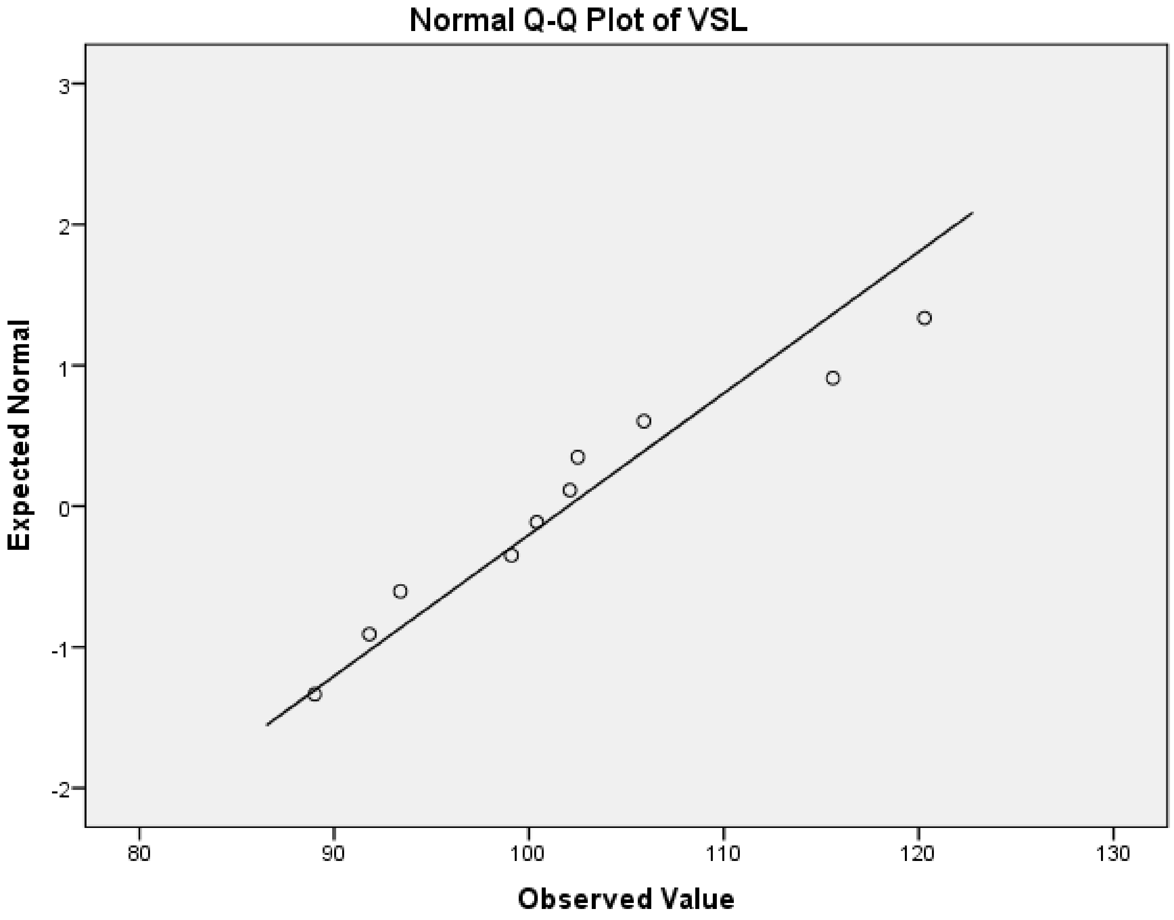

Normal Q-Q Plot of VSL
Expected Normal
3
2
1
0
-1
-2
80
90
100
110
120
130
Observed Value

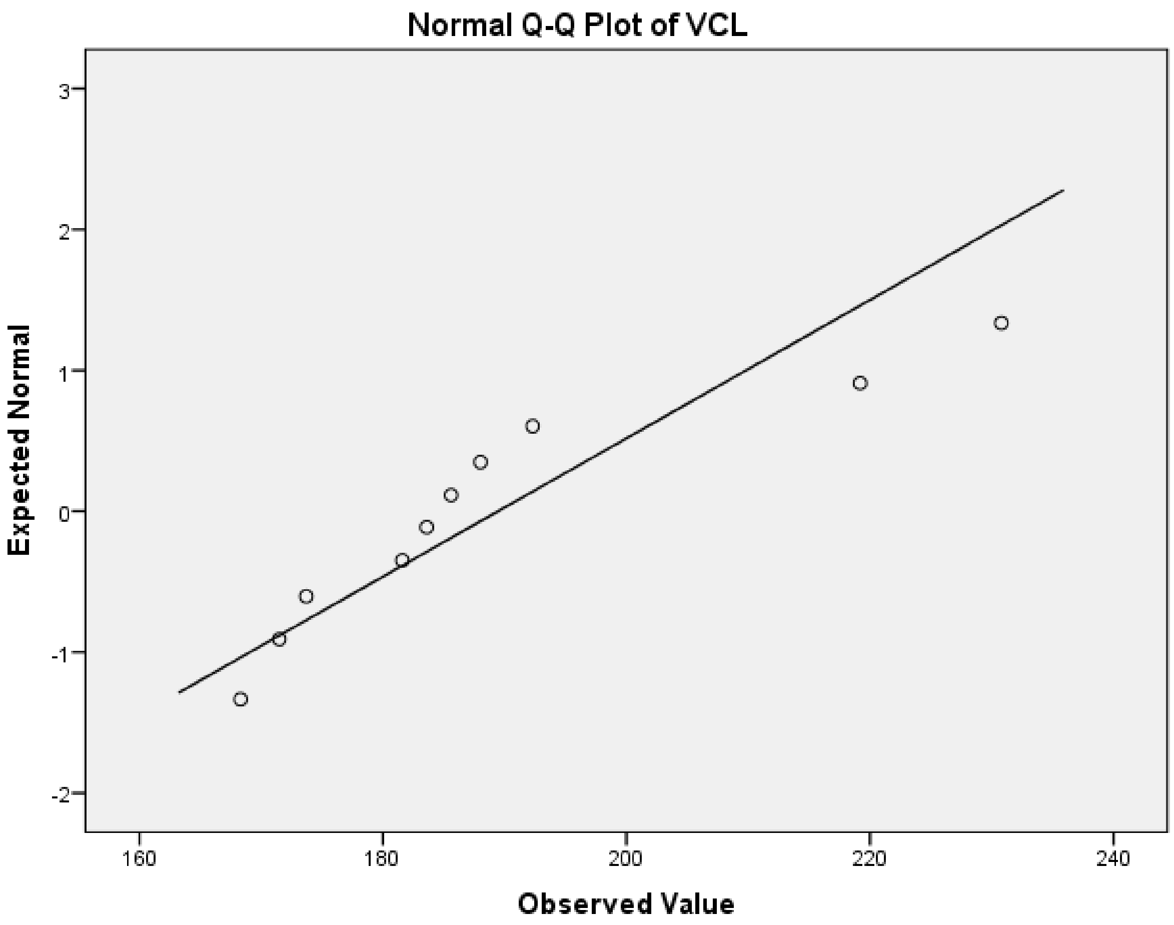

Normal Q-Q Plot of VCL
Expected Normal
Observed Value
3
2
1
0
-1
-2
160
180
200
220
240

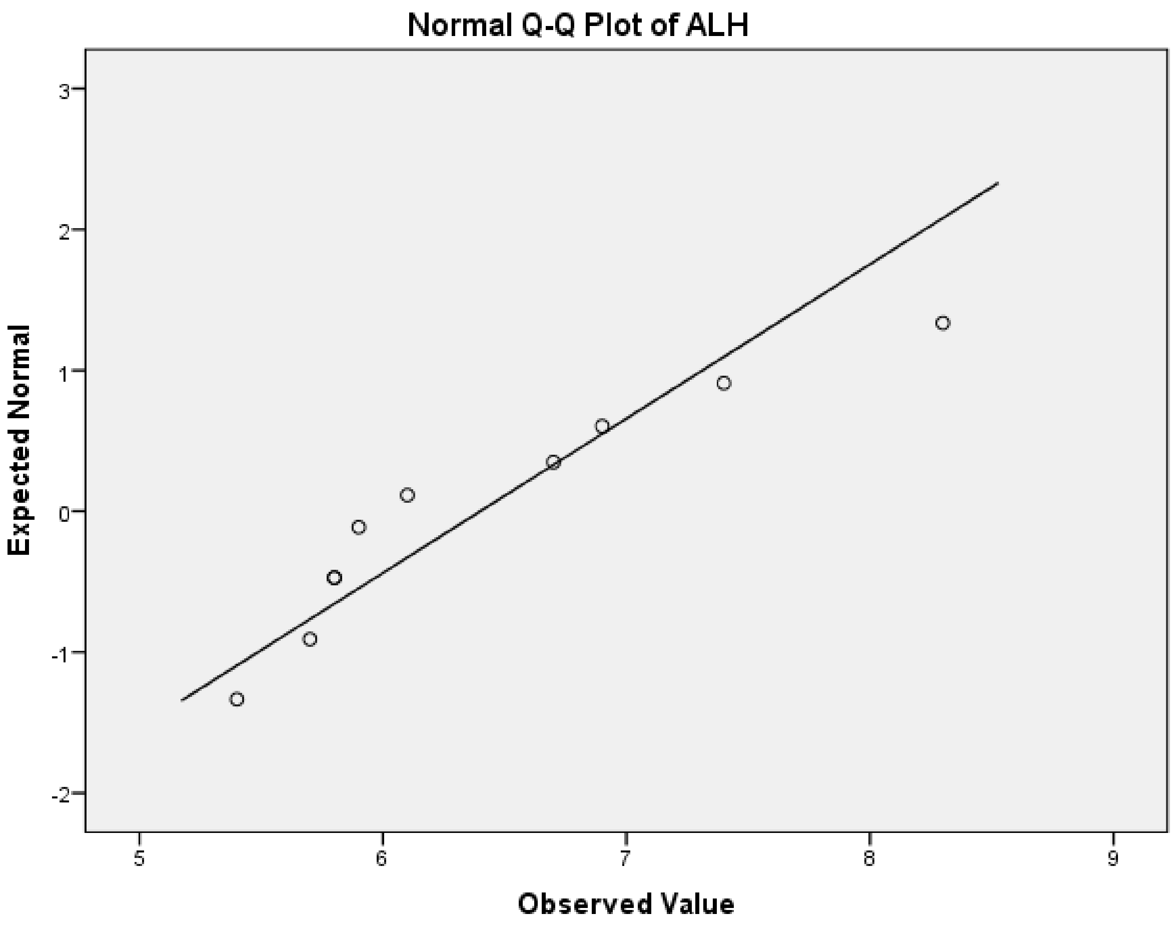

Normal Q-Q Plot of ALH
Expected Normal
Observed Value
3
2
1
0
-1
-2
5
6
7
8
9

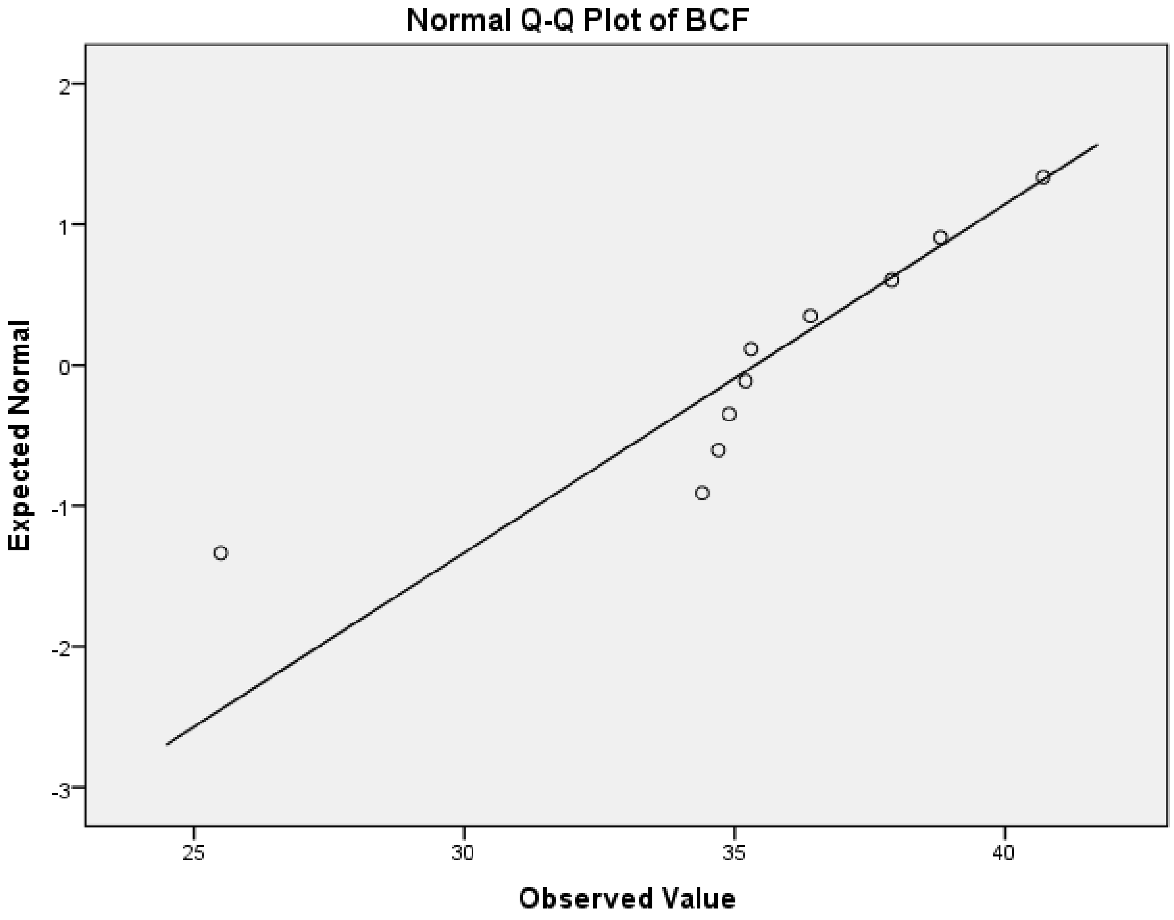

Normal Q-Q Plot of BCF
Expected Normal
2
1
0
-1
-2
-3
25
30
35
40
Observed Value

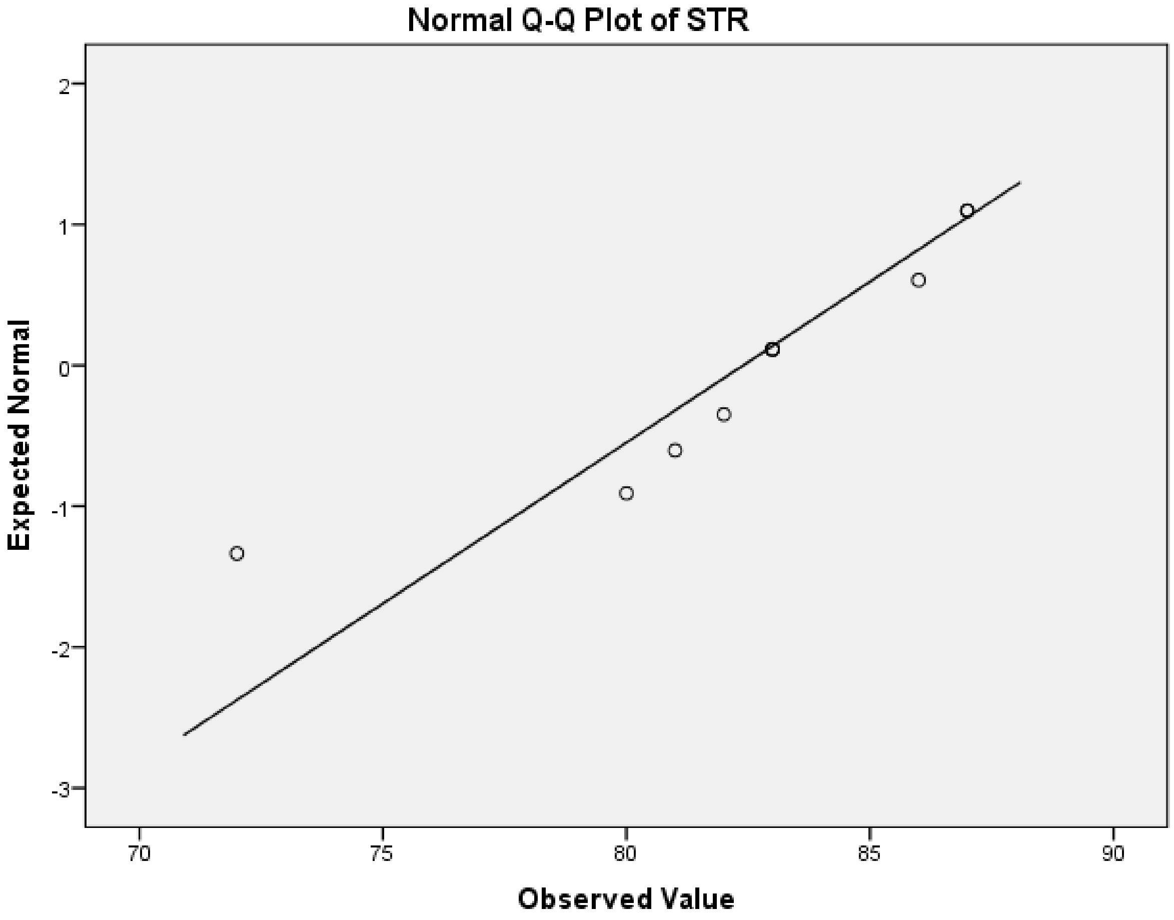
Normal Q-Q Plot of STR
Expected Normal
Observed Value
2
1
0
-1
-2
-3
70
75
80
85
90

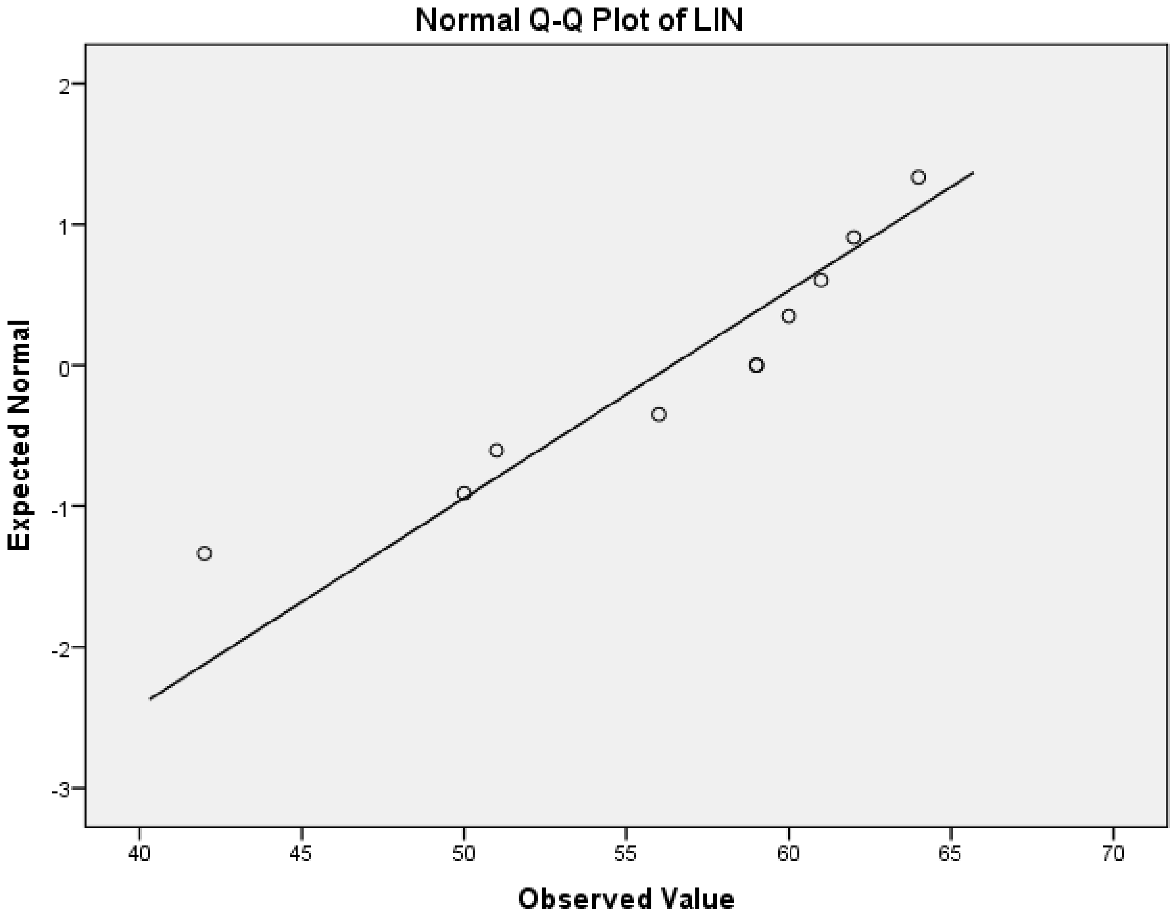

Detrended Normal Q-Q Plots

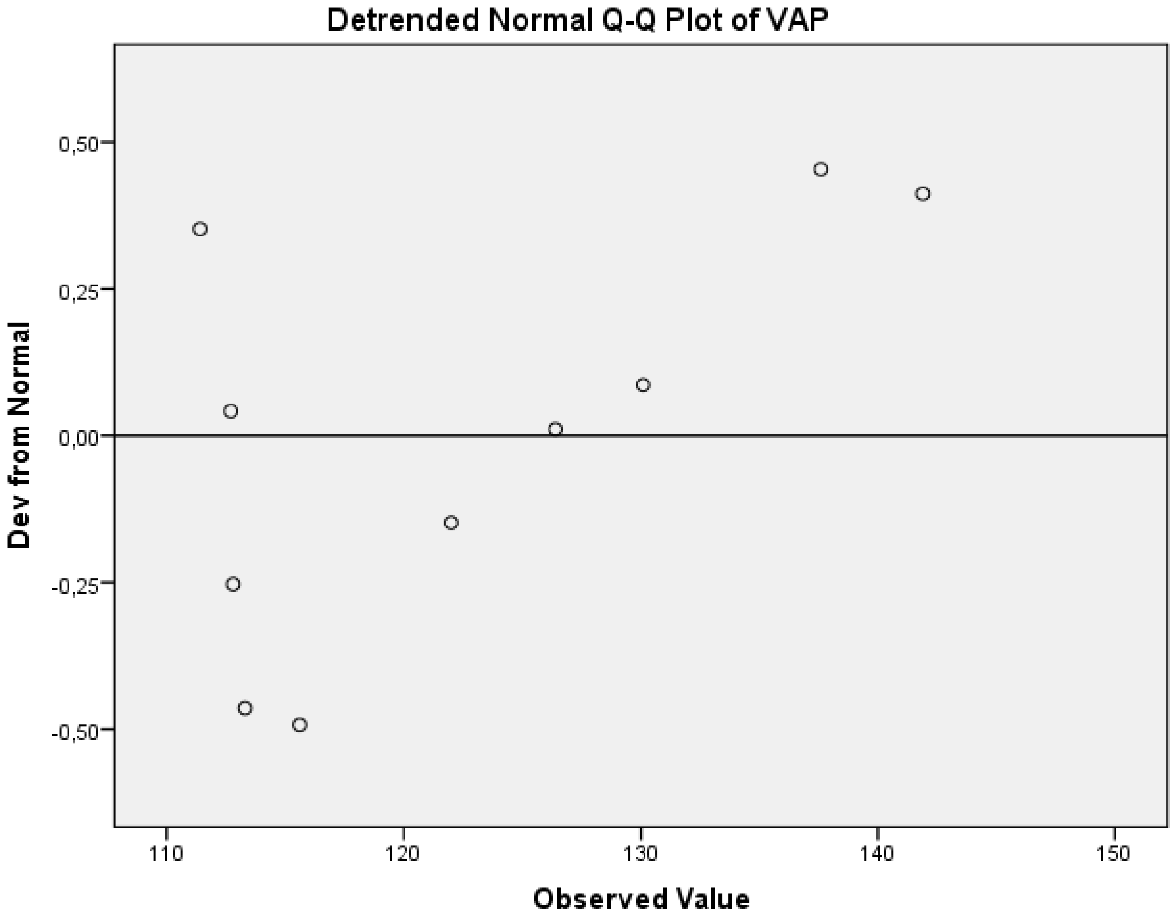
Detrended Normal Q-Q Plot of VAP
Dev from Normal
0,50
0,25
0,00
-0,25
-0,50
110
120
130
140
150
Observed Value

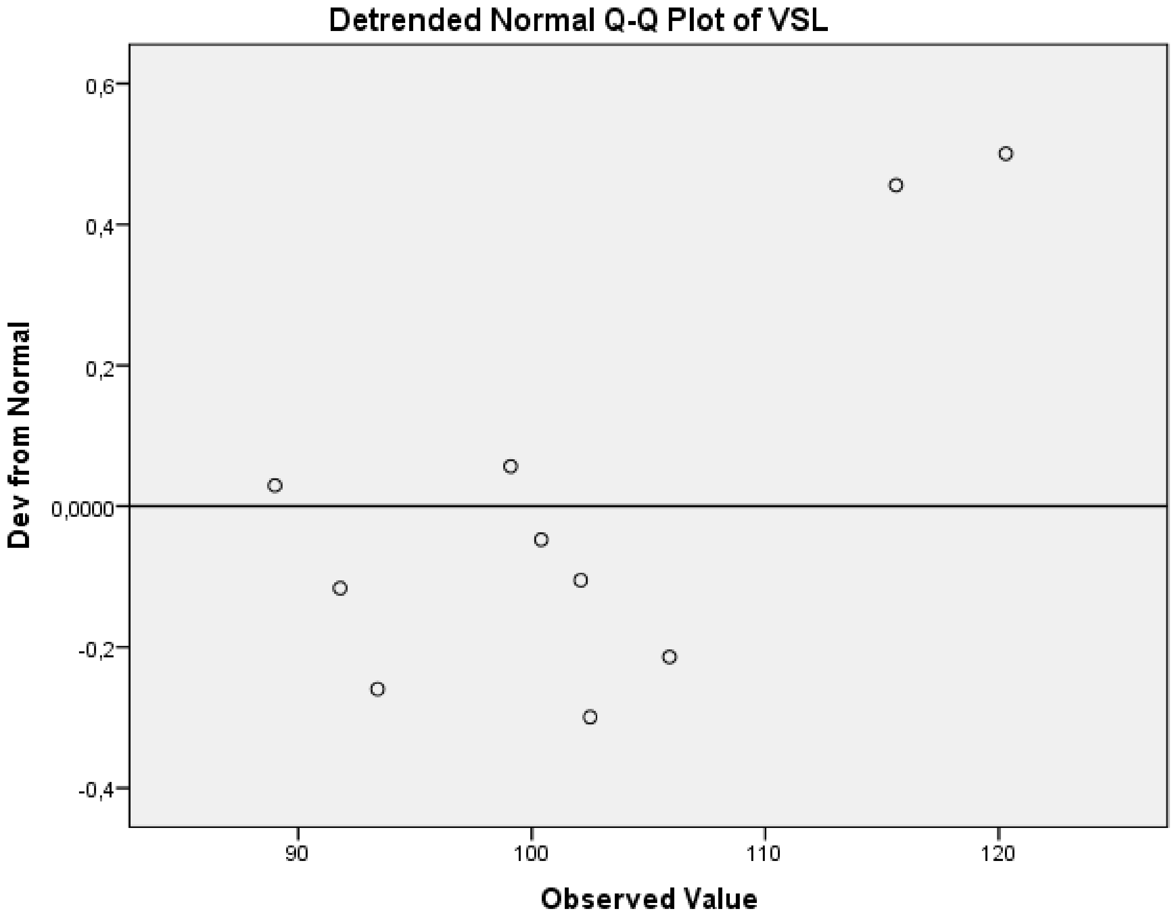
Detrended Normal Q-Q Plot of VSL
Dev from Normal
0,6
0,4
0,2
0,0000
-0,2
-0,4
90
100
110
120
Observed Value

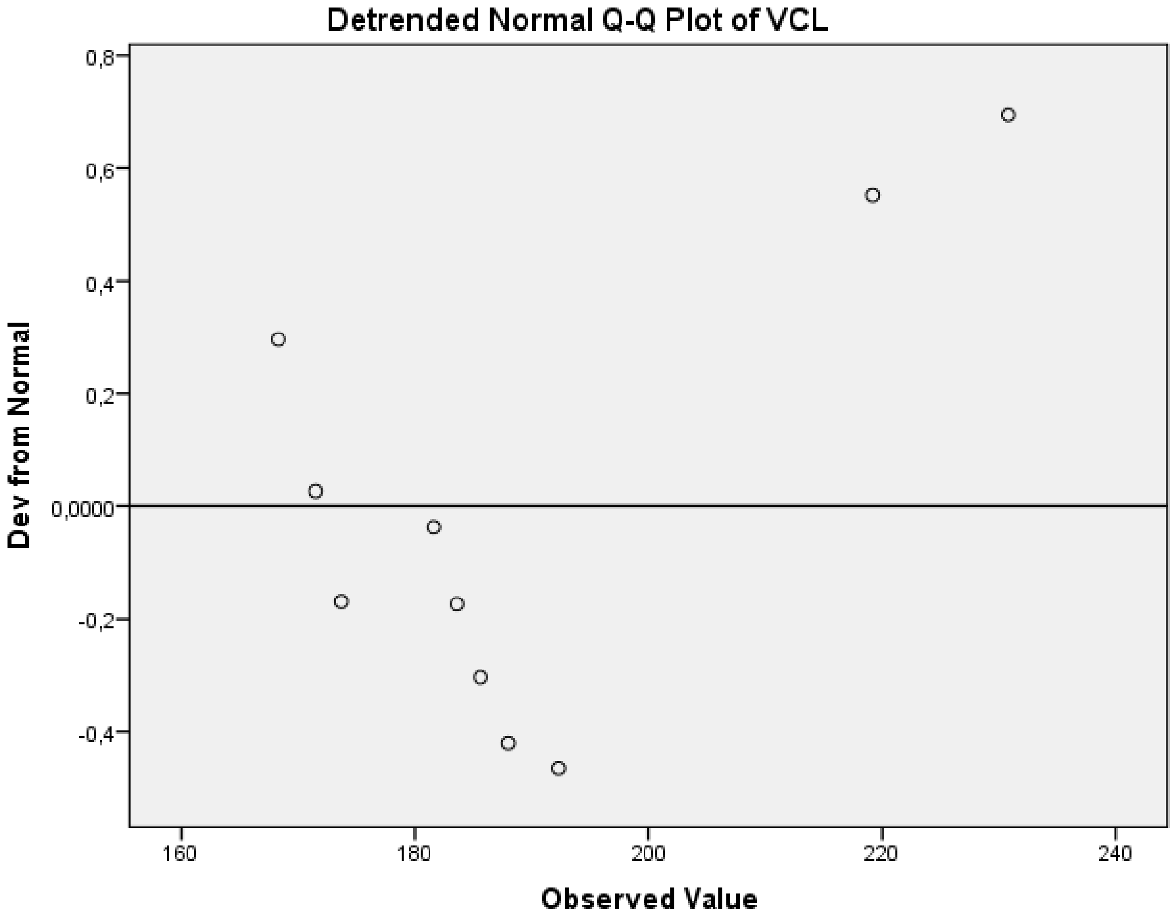
Detrended Normal Q-Q Plot of VCL
Dev from Normal
0,8
0,6
0,4
0,2
0,0000
-0,2
-0,4
160
180
200
220
240
Observed Value

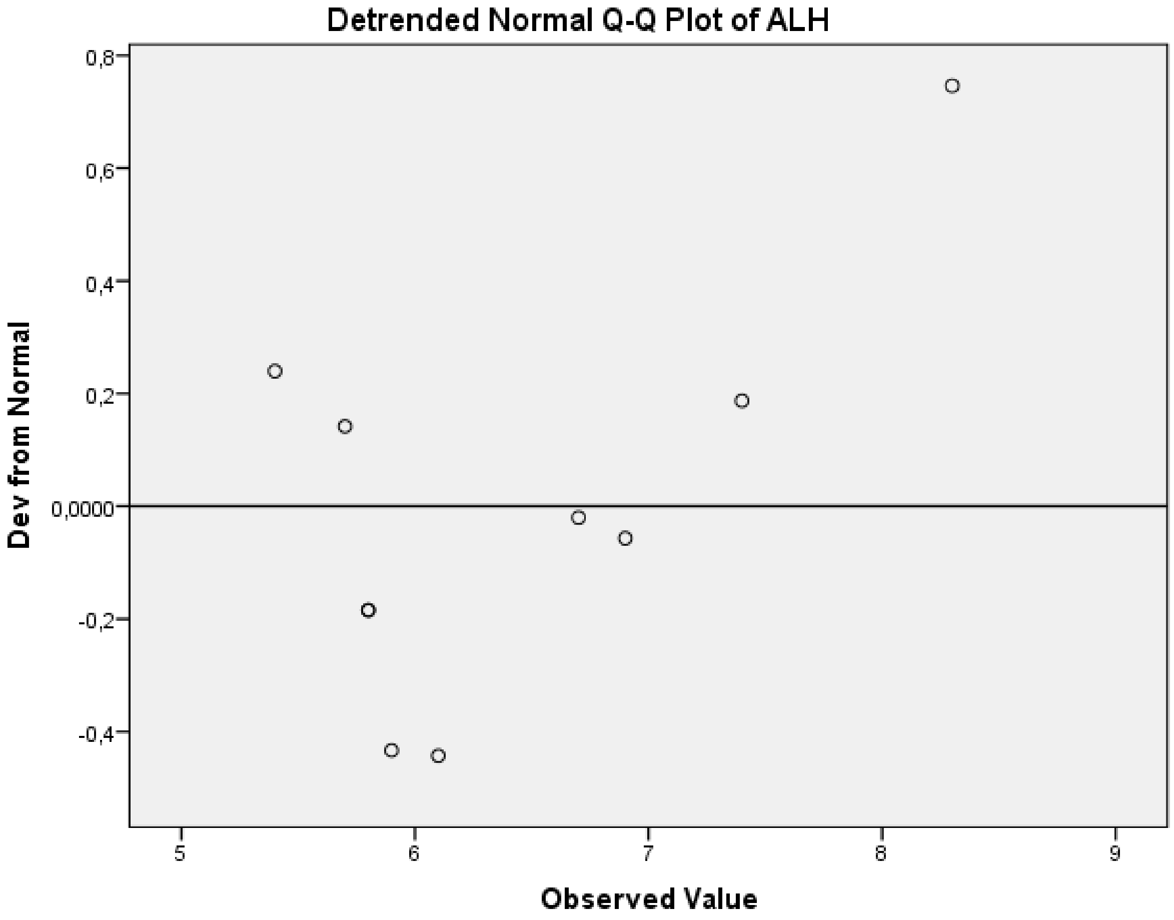

Detrended Normal Q-Q Plot of ALH
0,8
0,6
0,4
0,2
0,0000
-0,2
-0,4
Dev from Normal
5
6
7
8
9
Observed Value

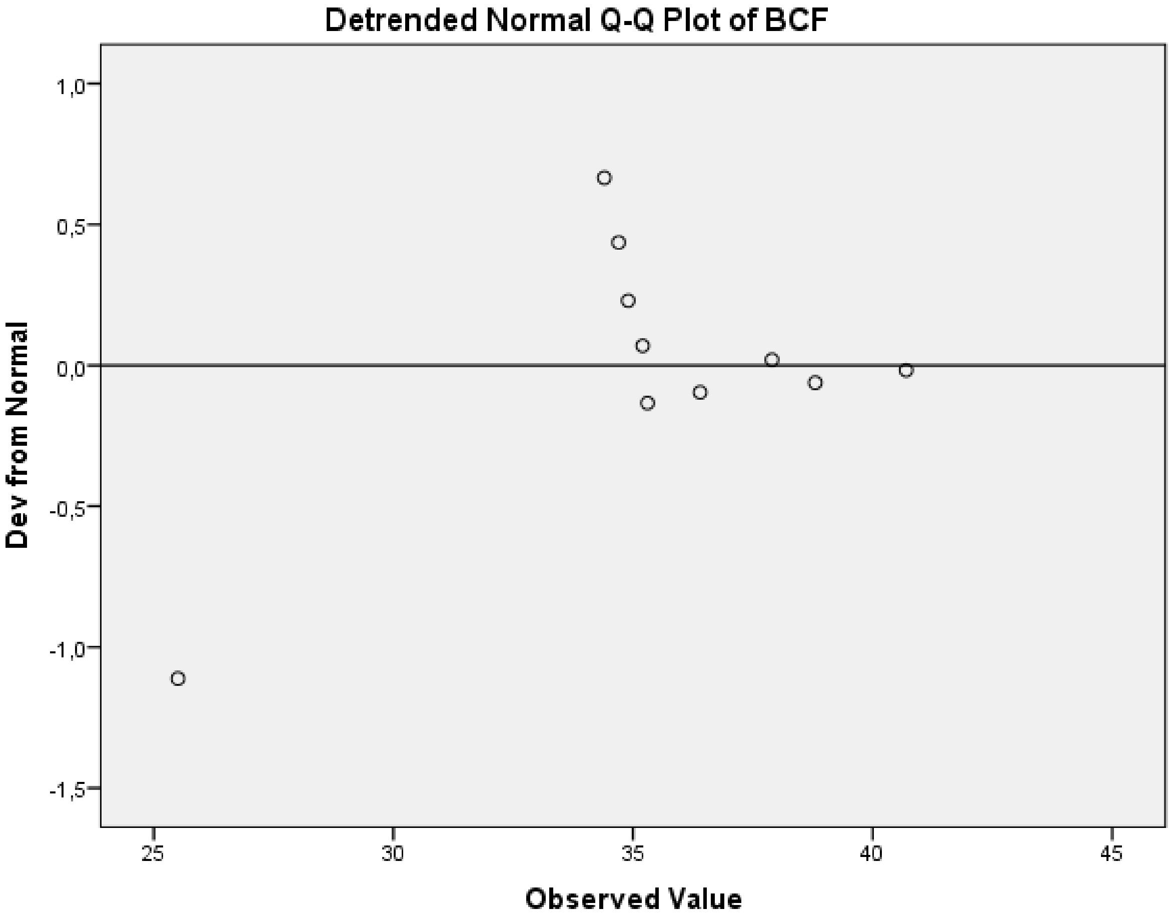
Detrended Normal Q-Q Plot of BCF
Dev from Normal
1,0
0,5
0,0
-0,5
-1,0
-1,5
25
30
35
40
45
Observed Value

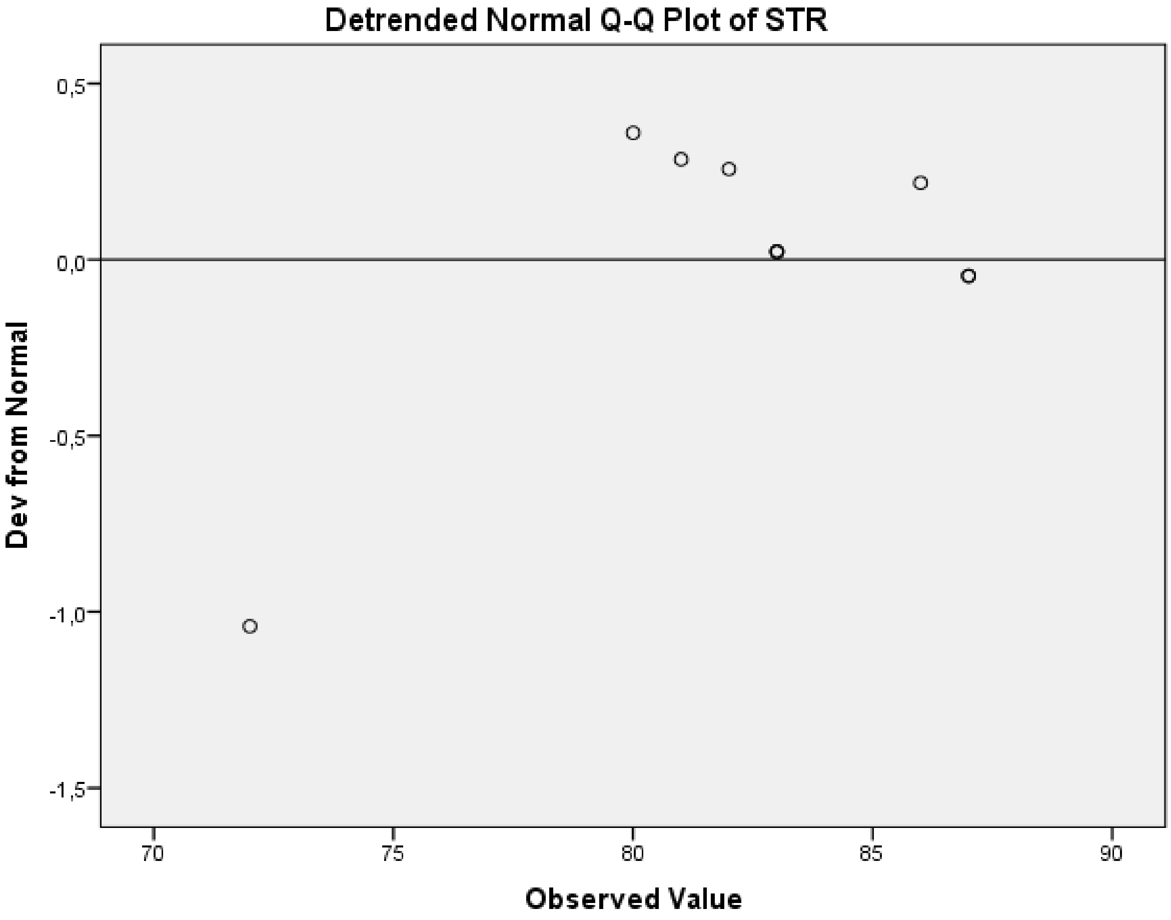

Detrended Normal Q-Q Plot of STR
Dev from Normal
0,5
0,0
-0,5
-1,0
-1,5
70
75
80
85
90
Observed Value

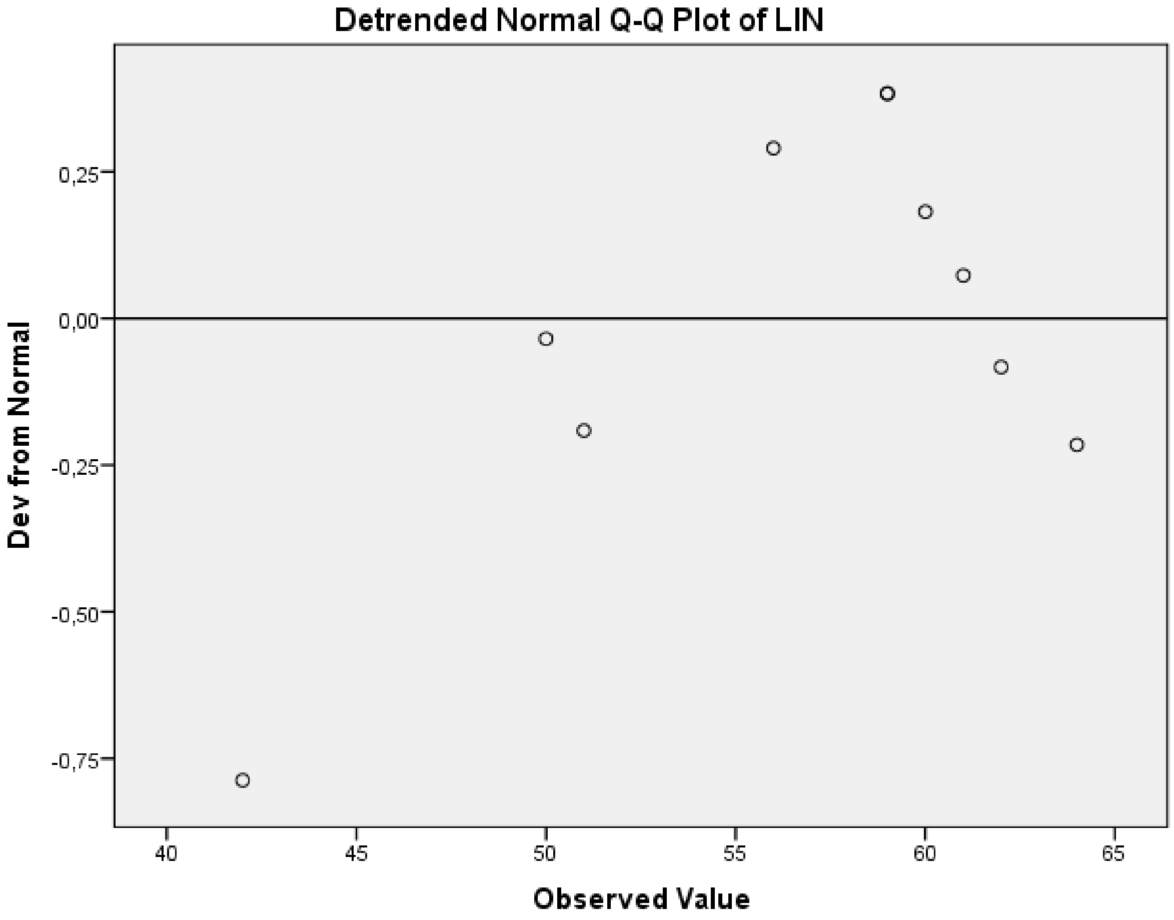

Grup = 6,00

Histograms

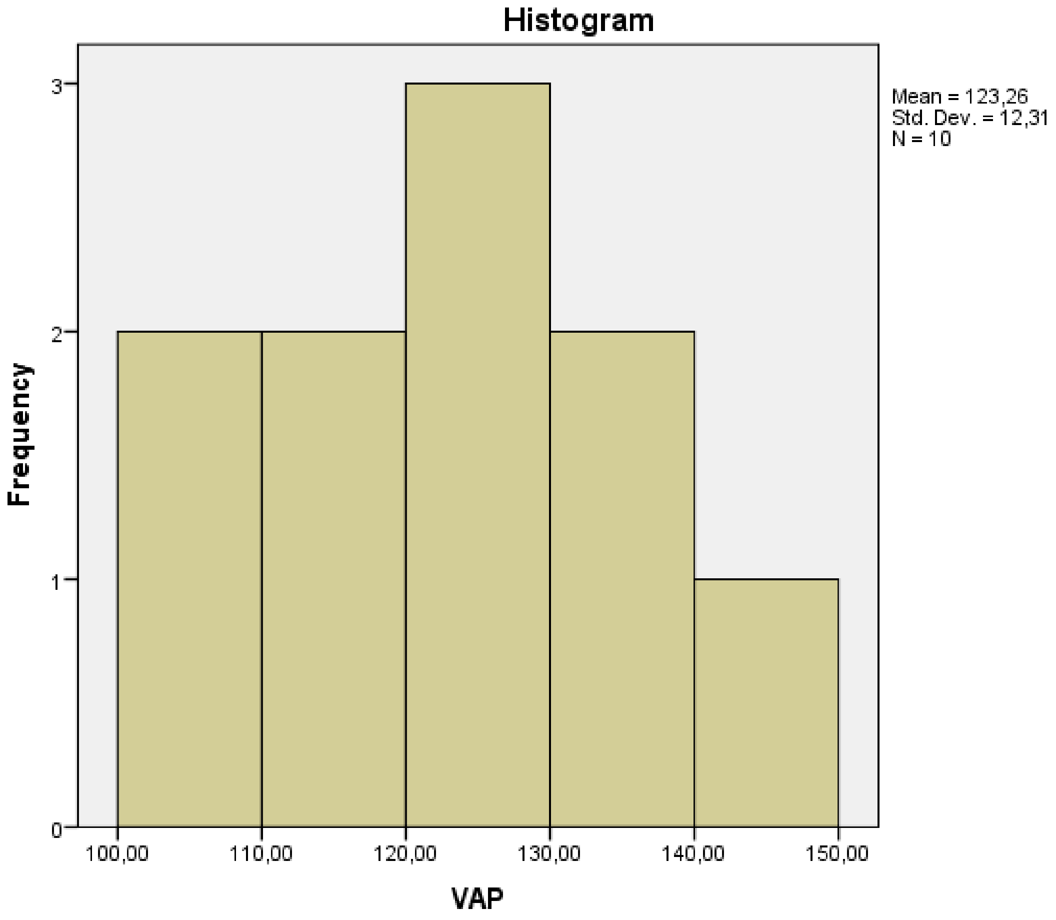

Histogram
Mean = 123,26
Std. Dev. = 12,31
N = 10
Frequency
3
2
1
0
100,00
110,00
120,00
130,00
140,00
150,00
VAP

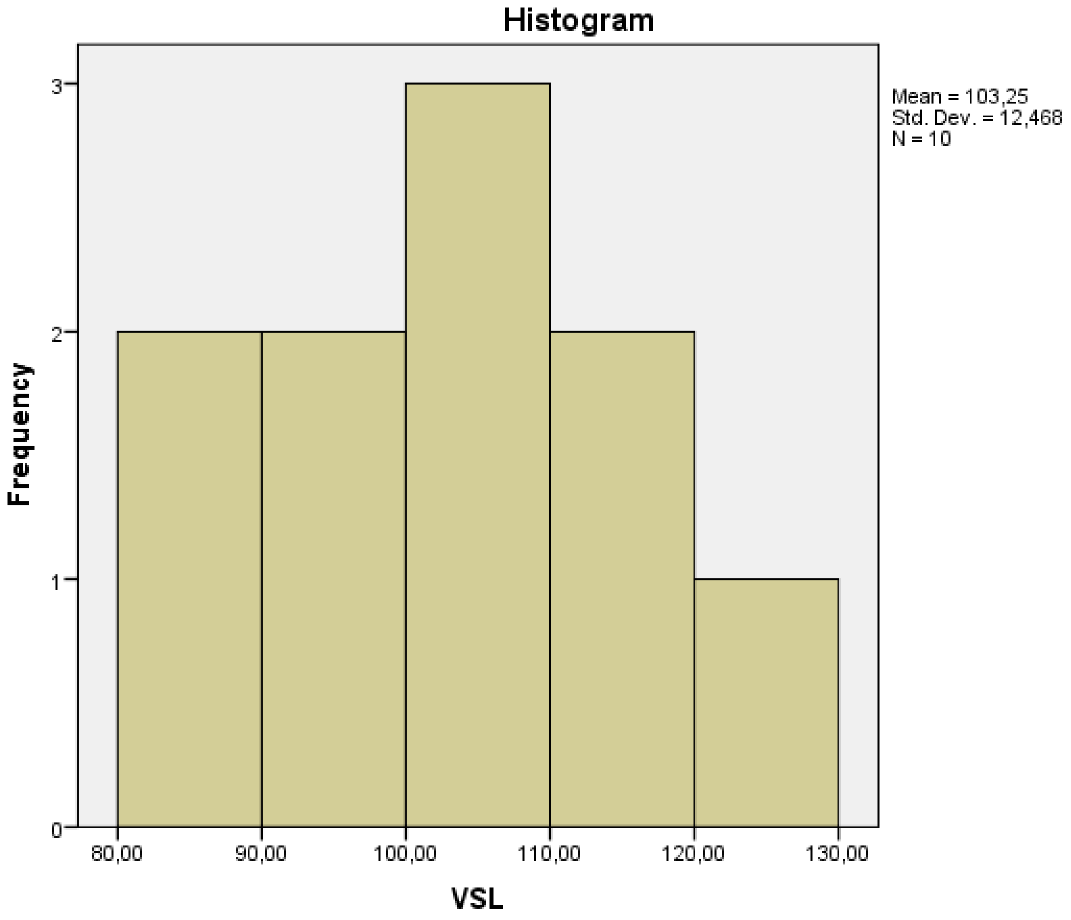
Histogram
Mean = 103,25
Std. Dev. = 12,468
N = 10
Frequency
0
1
2
3
80,00
90,00
100,00
110,00
120,00
130,00
VSL

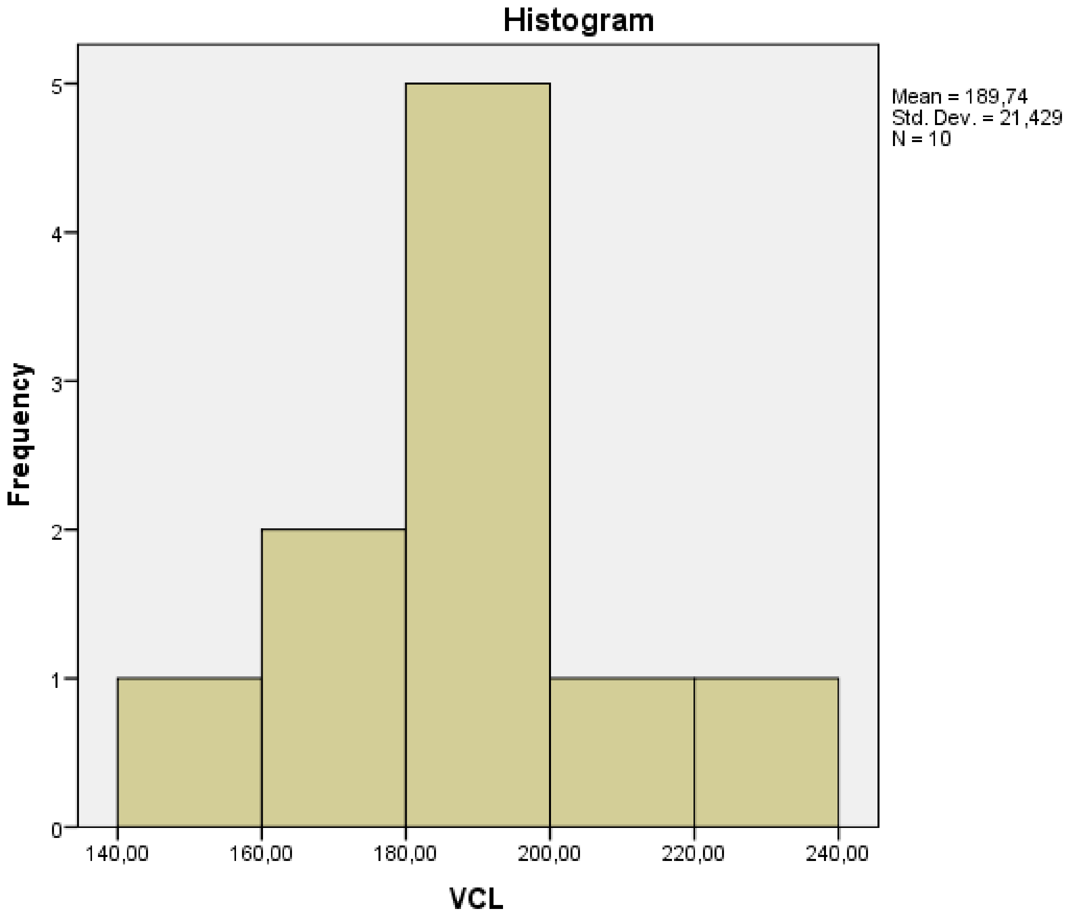

Histogram
Mean = 189,74
Std. Dev. = 21,429
N = 10
Frequency
0
1
2
3
4
5
140,00
160,00
180,00
200,00
220,00
240,00
VCL

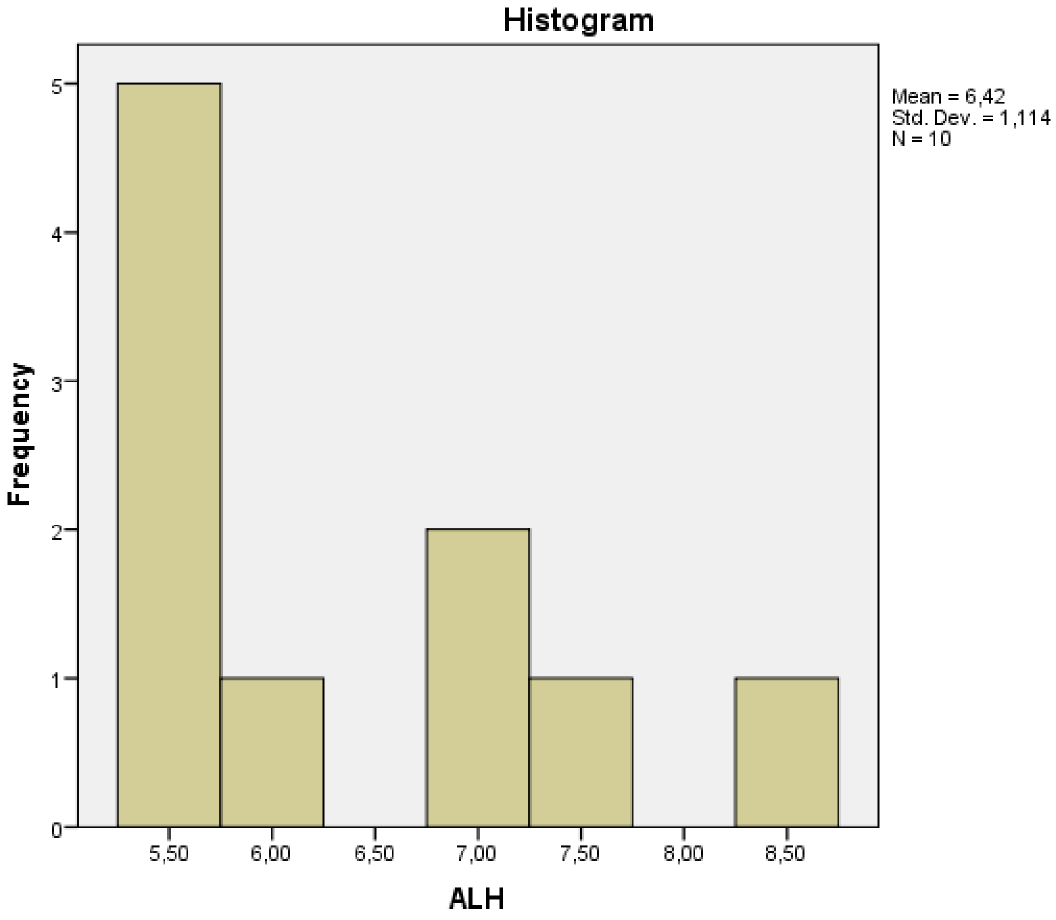
Histogram
Mean = 6,42
Std. Dev. = 1,114
N = 10
Frequency
0
1
2
3
4
5
5,50
6,00
6,50
7,00
7,50
8,00
8,50
ALH

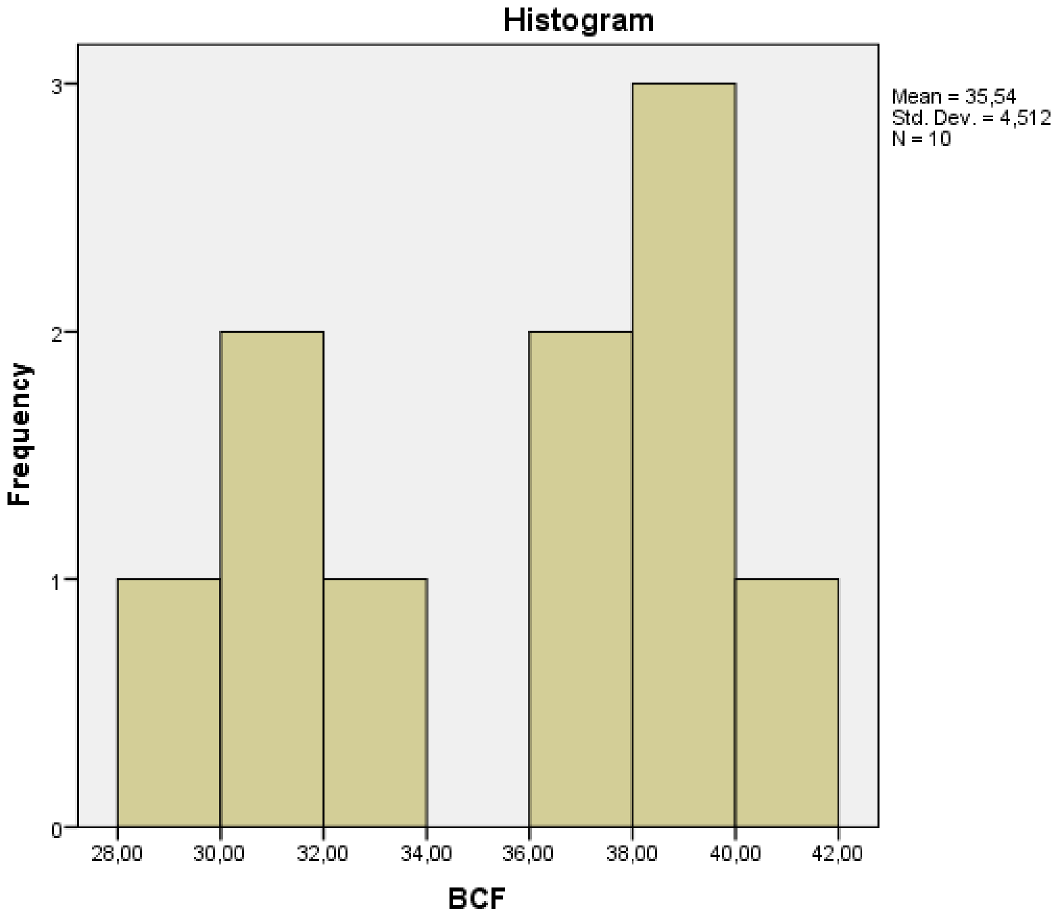

Histogram
Mean = 35,54
Std. Dev. = 4,512
N = 10
Frequency
0
1
2
3
28,00
30,00
32,00
34,00
36,00
38,00
40,00
42,00
BCF

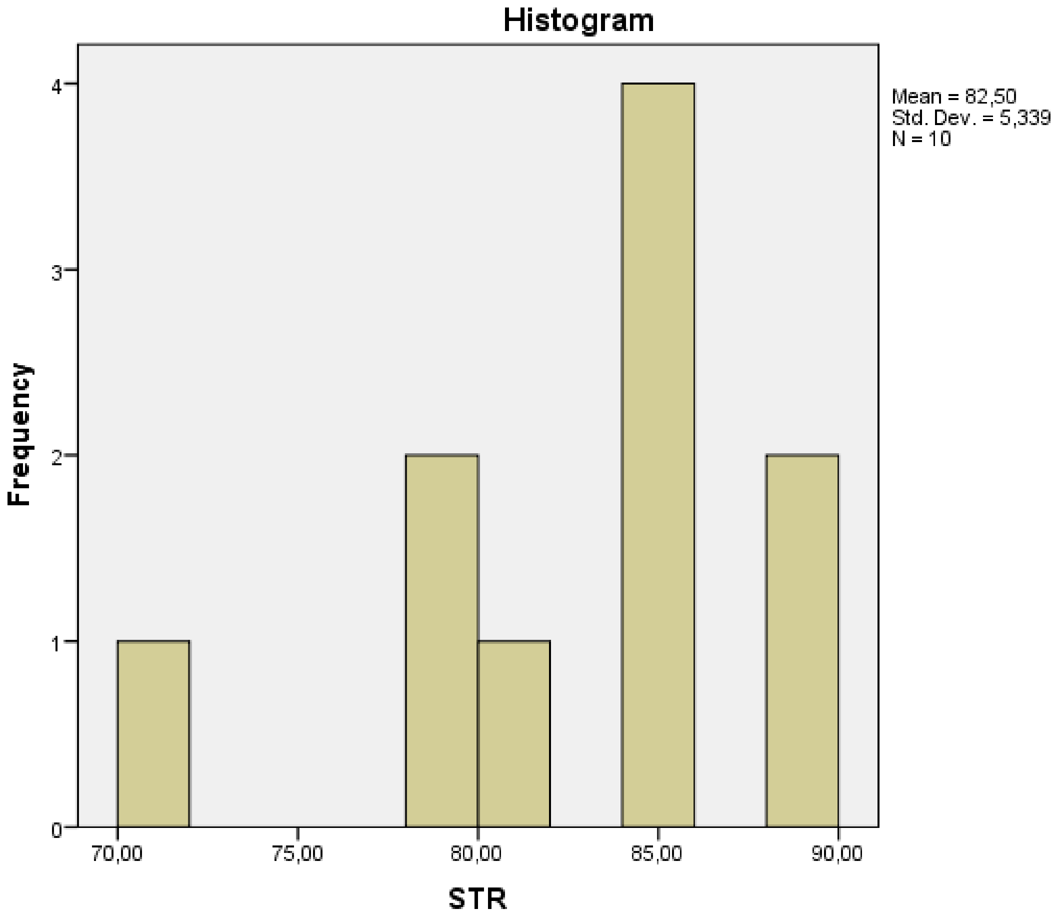

Histogram
Mean = 82,50
Std. Dev. = 5,339
N = 10
Frequency
0
1
2
3
4
70,00
75,00
80,00
85,00
90,00
STR

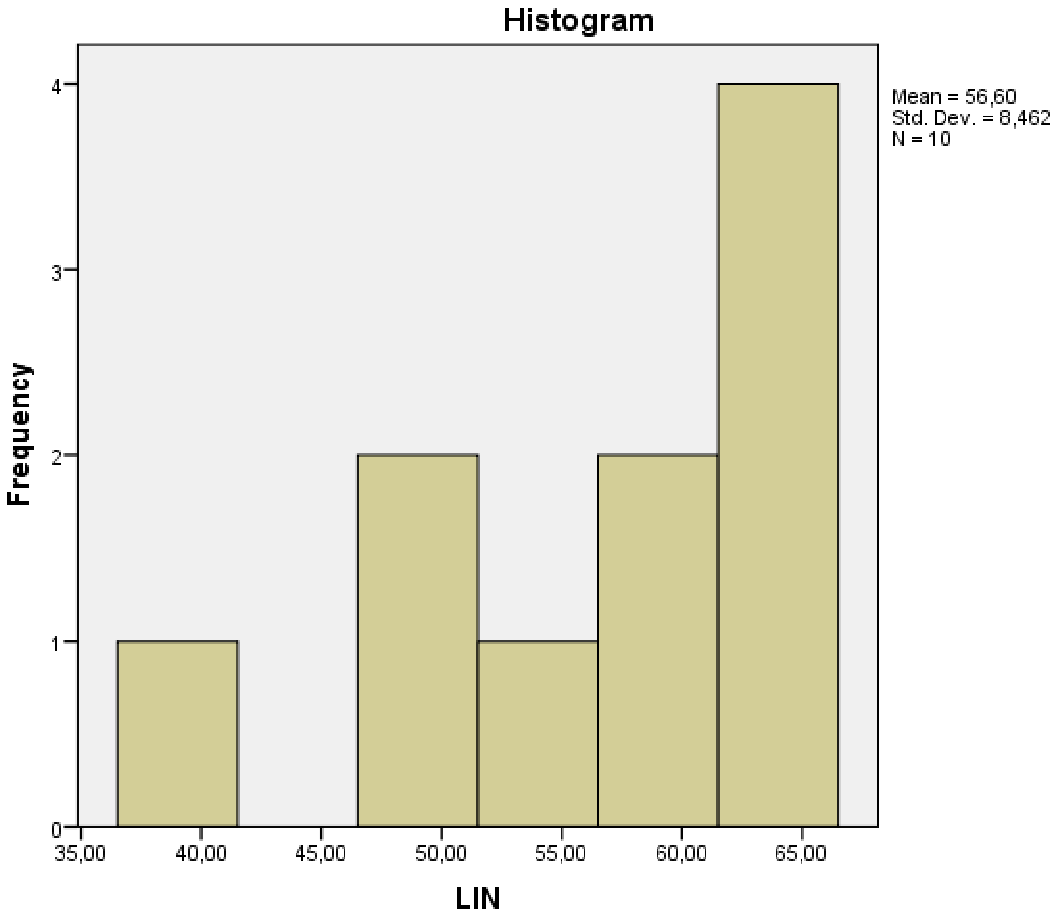

Stem-and-Leaf Plots

```
VAP Stem-and-Leaf Plot for
Grup= 6,00

 Frequency    Stem &  Leaf

     1,00 Extremes    (=<103)
     1,00       10 .  6
     2,00       11 .  99
     3,00       12 .  139
     2,00       13 .  08
     1,00       14 .  1

 Stem width:  10,00
 Each leaf:       1 case(s)
```

VSL Stem-and-Leaf Plot for
Grup= 6,00

Frequency	Stem &	Leaf
2,00	8 .	28
2,00	9 .	57
3,00	10 .	366
2,00	11 .	36
1,00	12 .	2

Stem width: 10,00
Each leaf: 1 case(s)

VCL Stem-and-Leaf Plot for
Grup= 6,00

Frequency	Stem &	Leaf
8,00	1 .	57788899
2,00	2 .	13

Stem width: 100,0
Each leaf: 1 case(s)

ALH Stem-and-Leaf Plot for
Grup= 6,00

Frequency	Stem &	Leaf
6,00	5 .	556778
,00	6 .	
3,00	7 .	016
1,00	8 .	7

Stem width: 1,00
Each leaf: 1 case(s)

BCF Stem-and-Leaf Plot for
Grup= 6,00

Frequency	Stem &	Leaf
1,00	2 .	8

```
     3,00        3 .  012
     5,00        3 .  66899
     1,00        4 .  1

 Stem width:  10,00
 Each leaf:       1 case(s)
```

```
STR Stem-and-Leaf Plot for
Grup= 6,00

 Frequency    Stem &  Leaf

     1,00        7 .  1
     2,00        7 .  99
     4,00        8 .  1444
     3,00        8 .  599

 Stem width:  10,00
 Each leaf:       1 case(s)
```

```
LIN Stem-and-Leaf Plot for
Grup= 6,00

 Frequency    Stem &  Leaf

     1,00        3 .  9
     1,00        4 .  8
     3,00        5 .  068
     5,00        6 .  12345

 Stem width:  10,00
 Each leaf:       1 case(s)
```

Normal Q-Q Plots

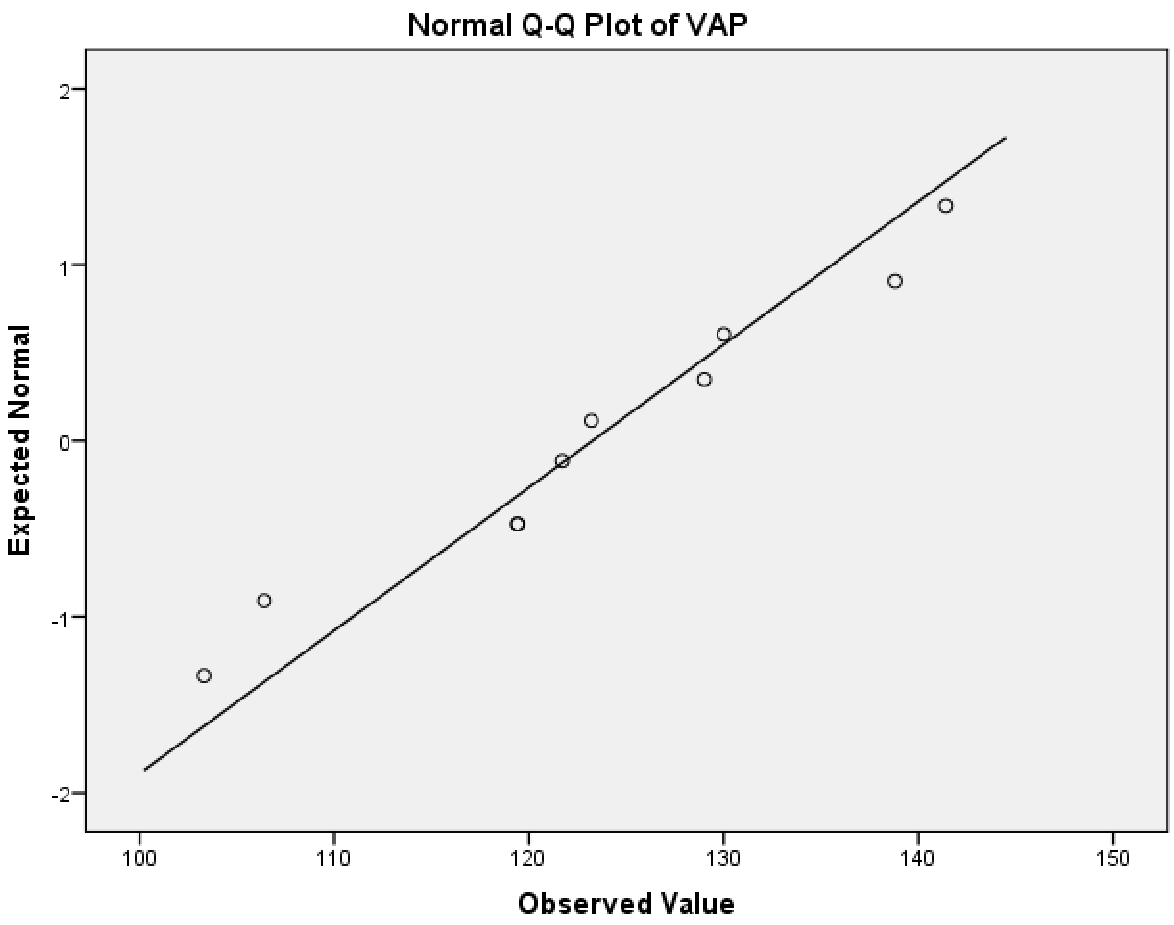

Normal Q-Q Plot of VAP
Expected Normal
Observed Value
2
1
0
-1
-2
100
110
120
130
140
150

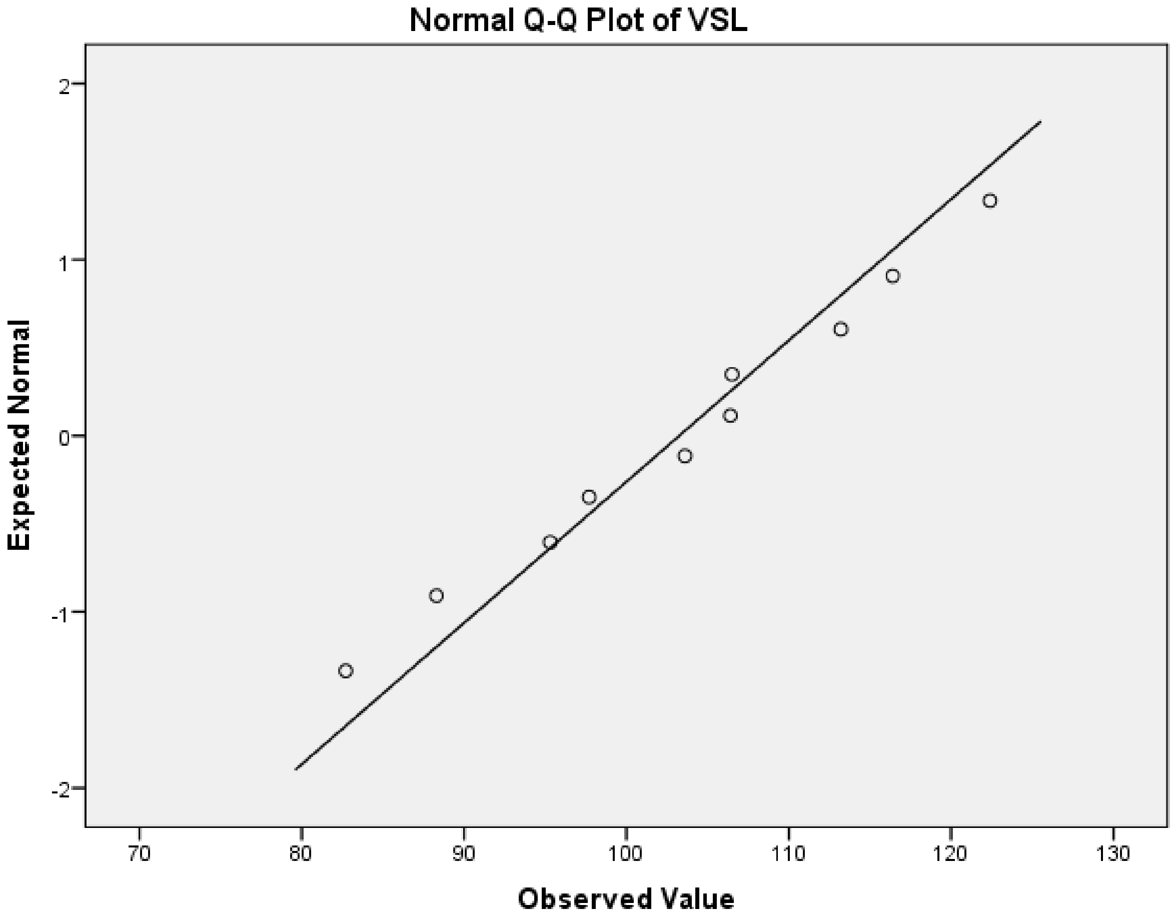

Normal Q-Q Plot of VSL
Expected Normal
Observed Value
2
1
0
-1
-2
70
80
90
100
110
120
130

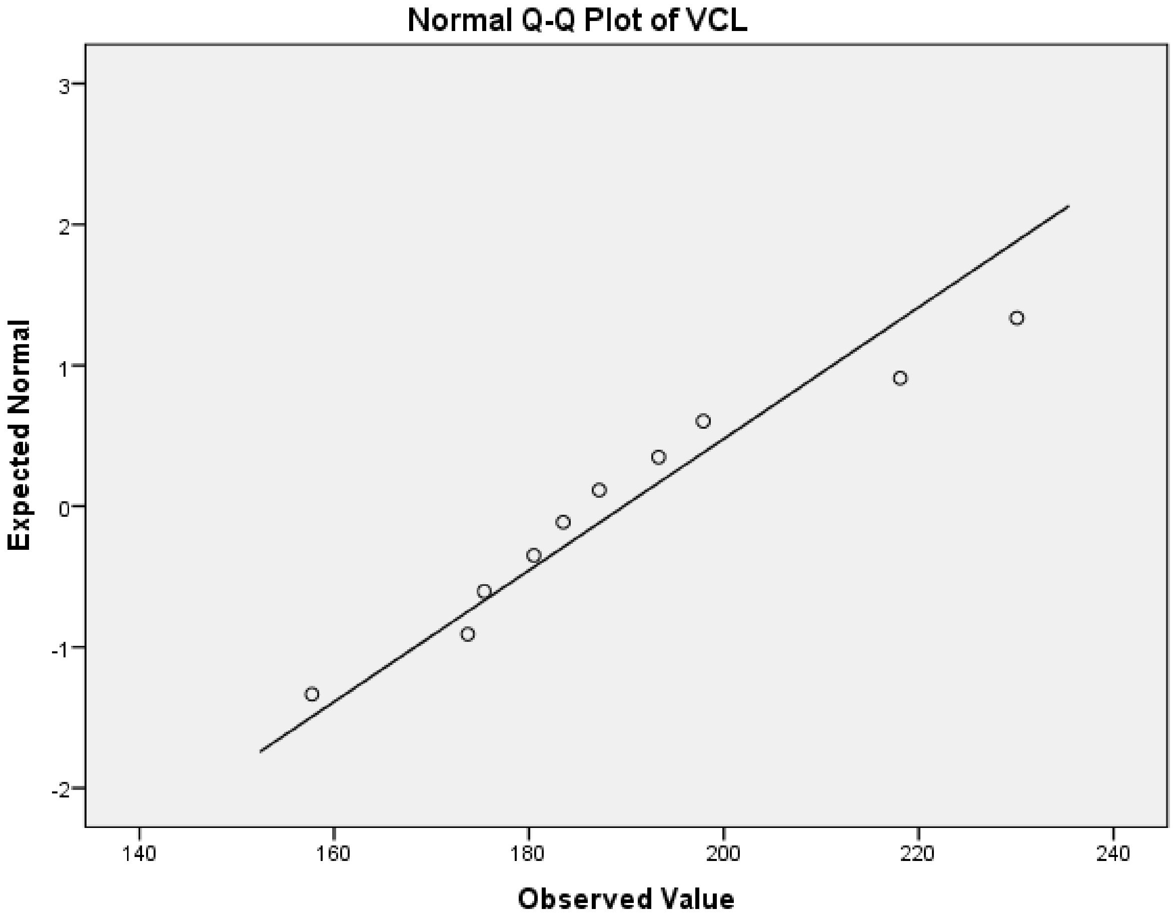
Normal Q-Q Plot of VCL
Expected Normal
Observed Value
3
2
1
0
-1
-2
140
160
180
200
220
240

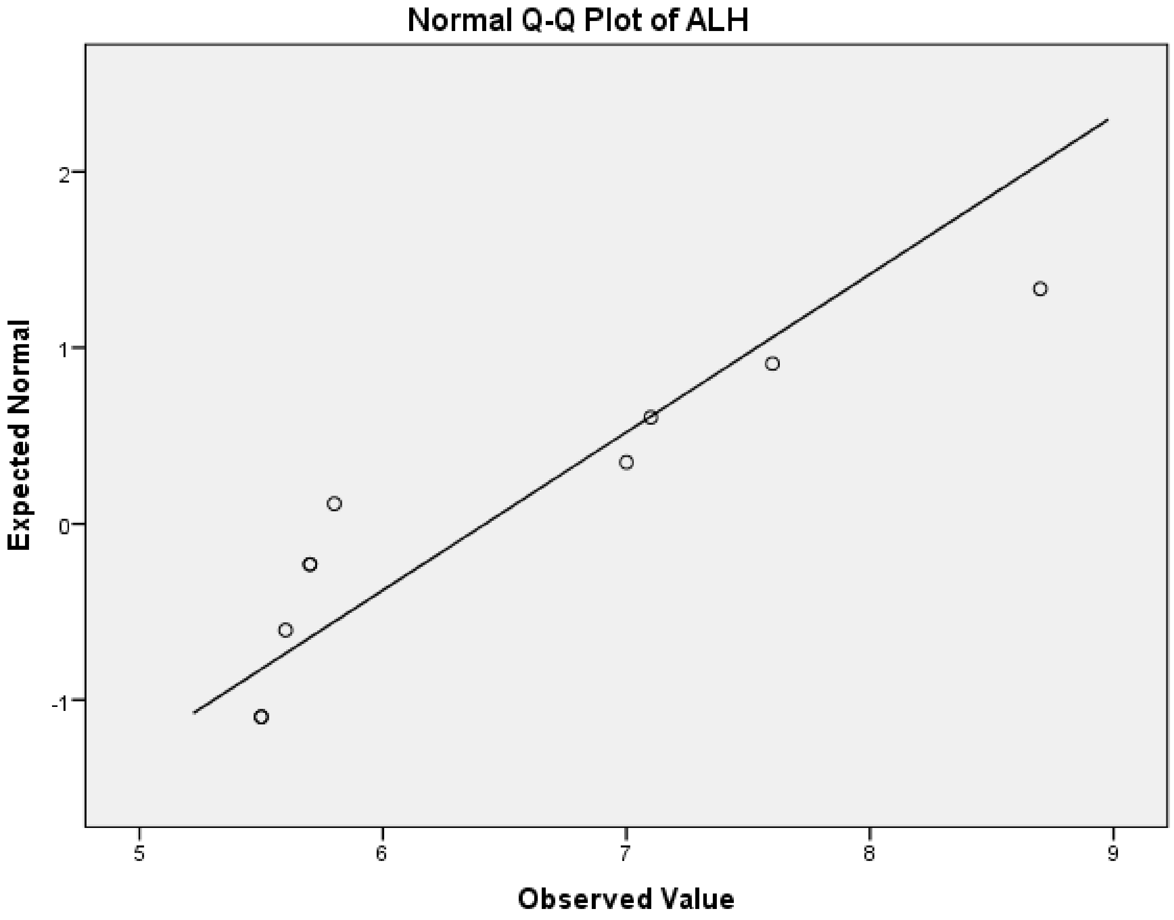

Normal Q-Q Plot of ALH
Expected Normal
Observed Value
2
1
0
-1
5
6
7
8
9

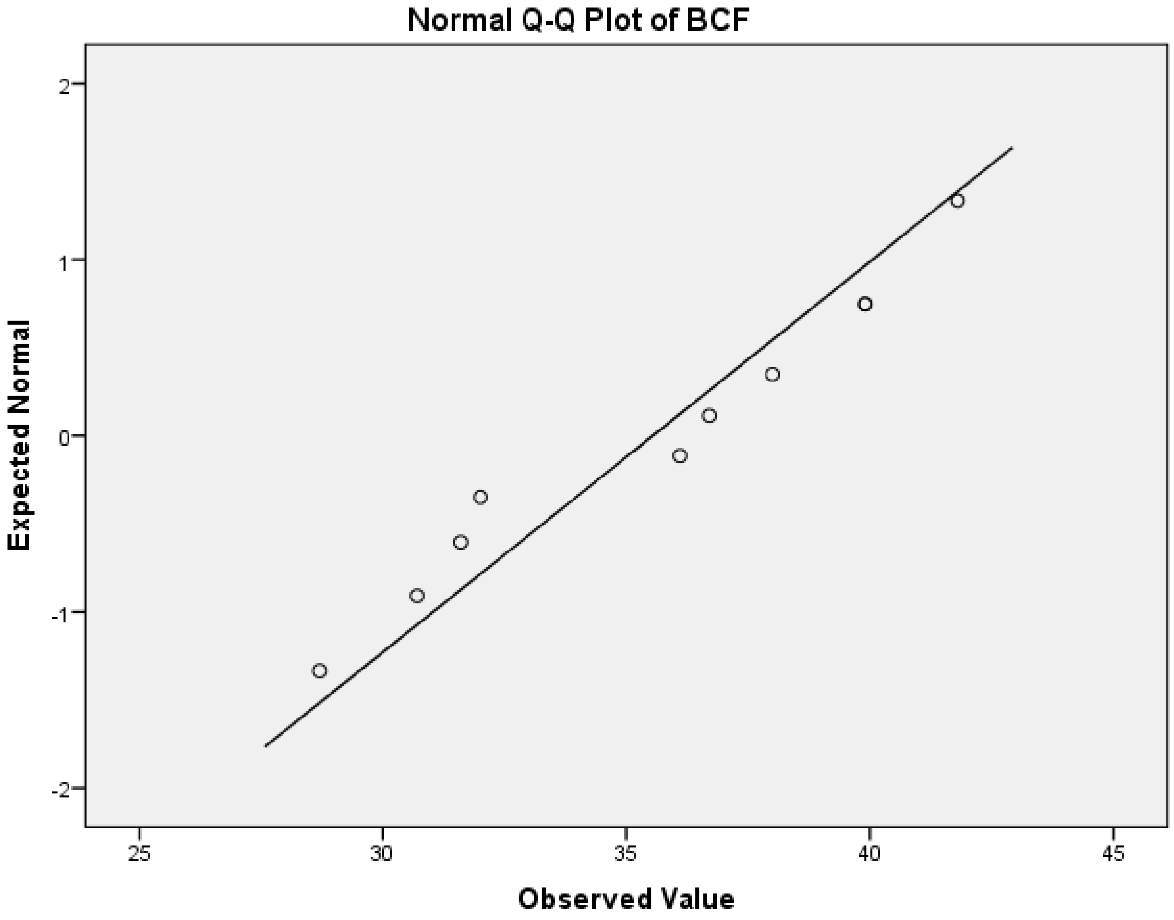
Normal Q-Q Plot of BCF
Expected Normal
Observed Value
2
1
0
-1
-2
25
30
35
40
45

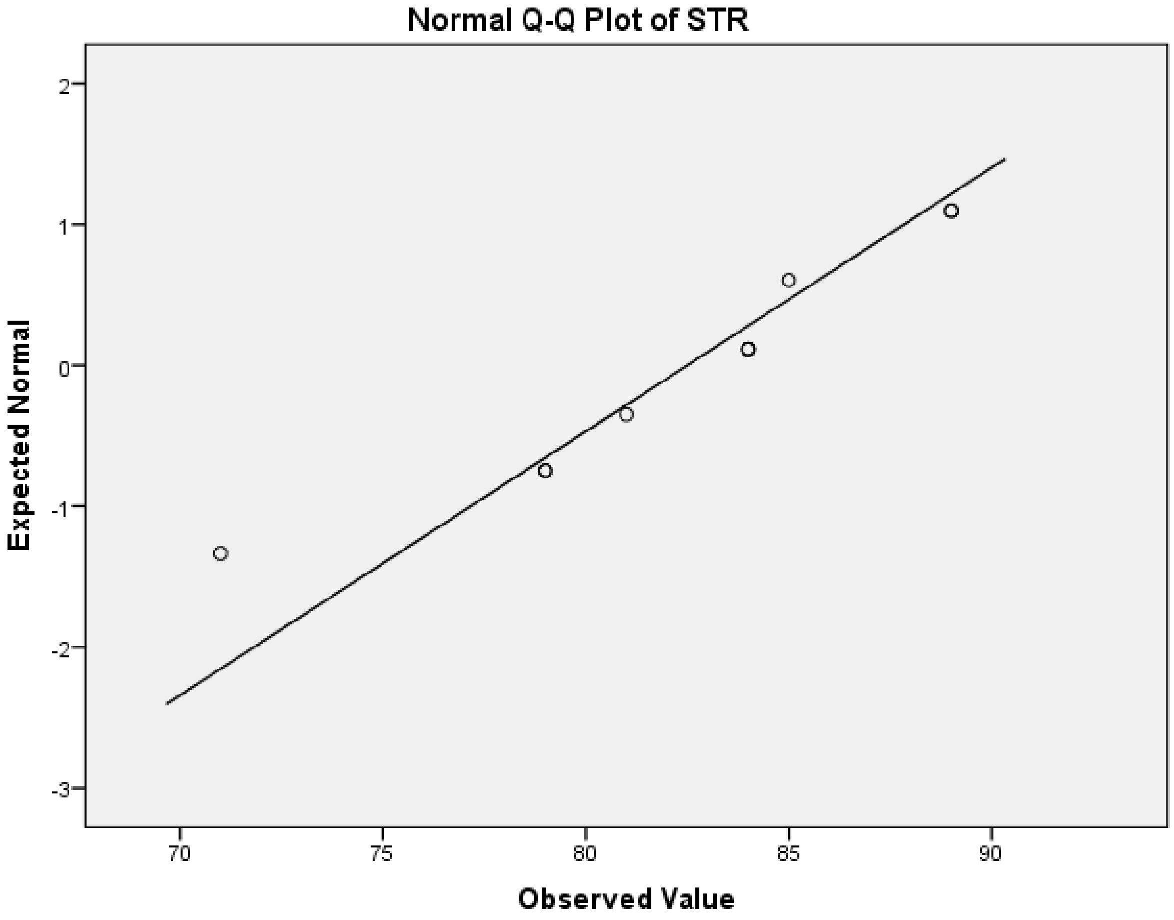

Normal Q-Q Plot of STR
Expected Normal
Observed Value
2
1
0
-1
-2
-3
70
75
80
85
90

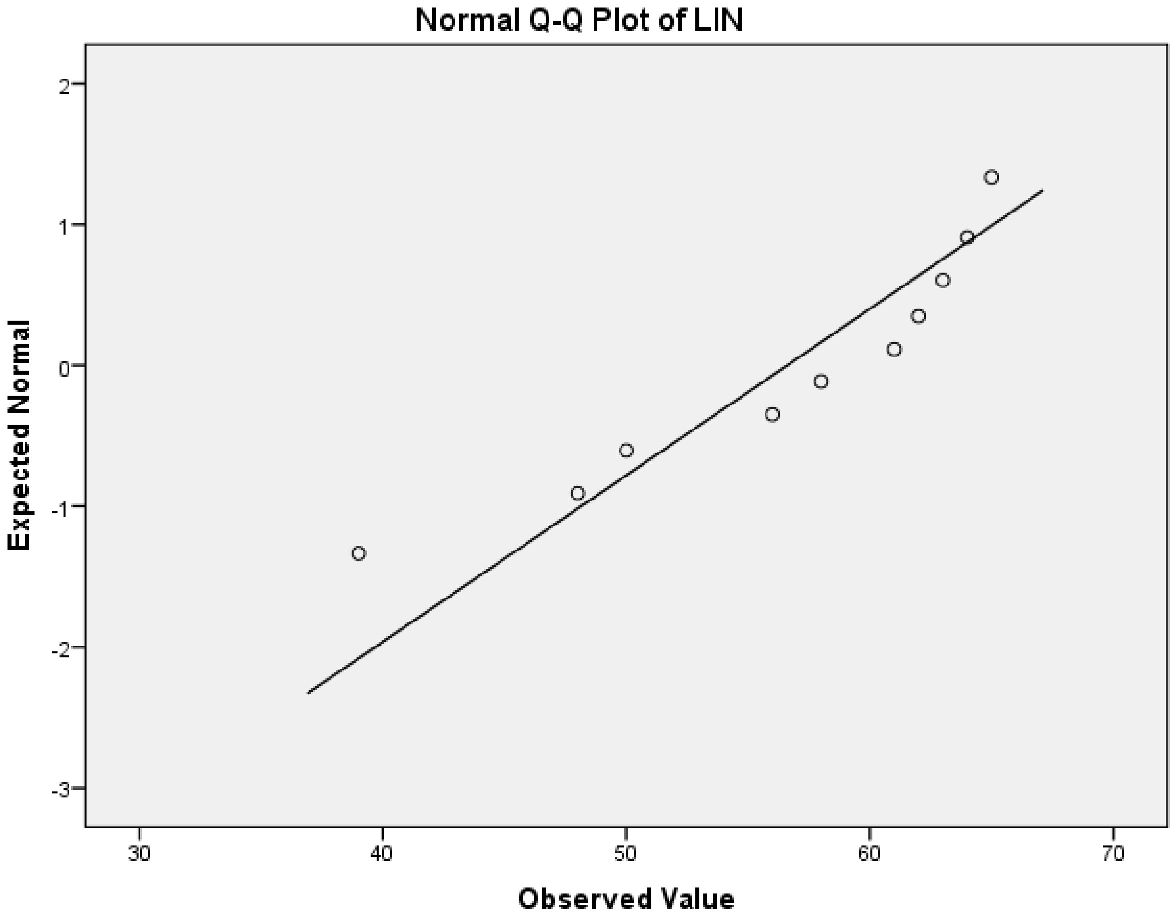

Detrended Normal Q-Q Plots

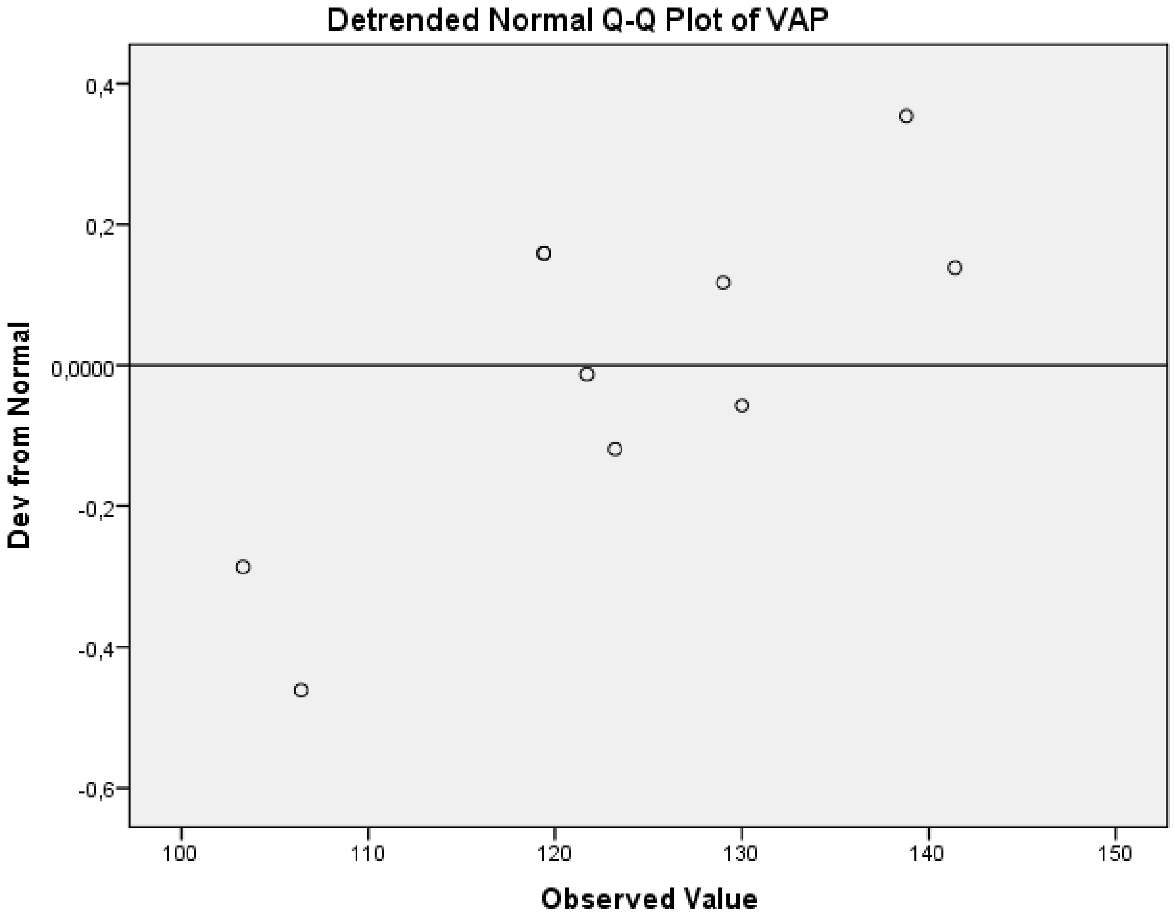

Detrended Normal Q-Q Plot of VAP
0,4
0,2
0,0000
-0,2
-0,4
-0,6
Dev from Normal
100
110
120
130
140
150
Observed Value

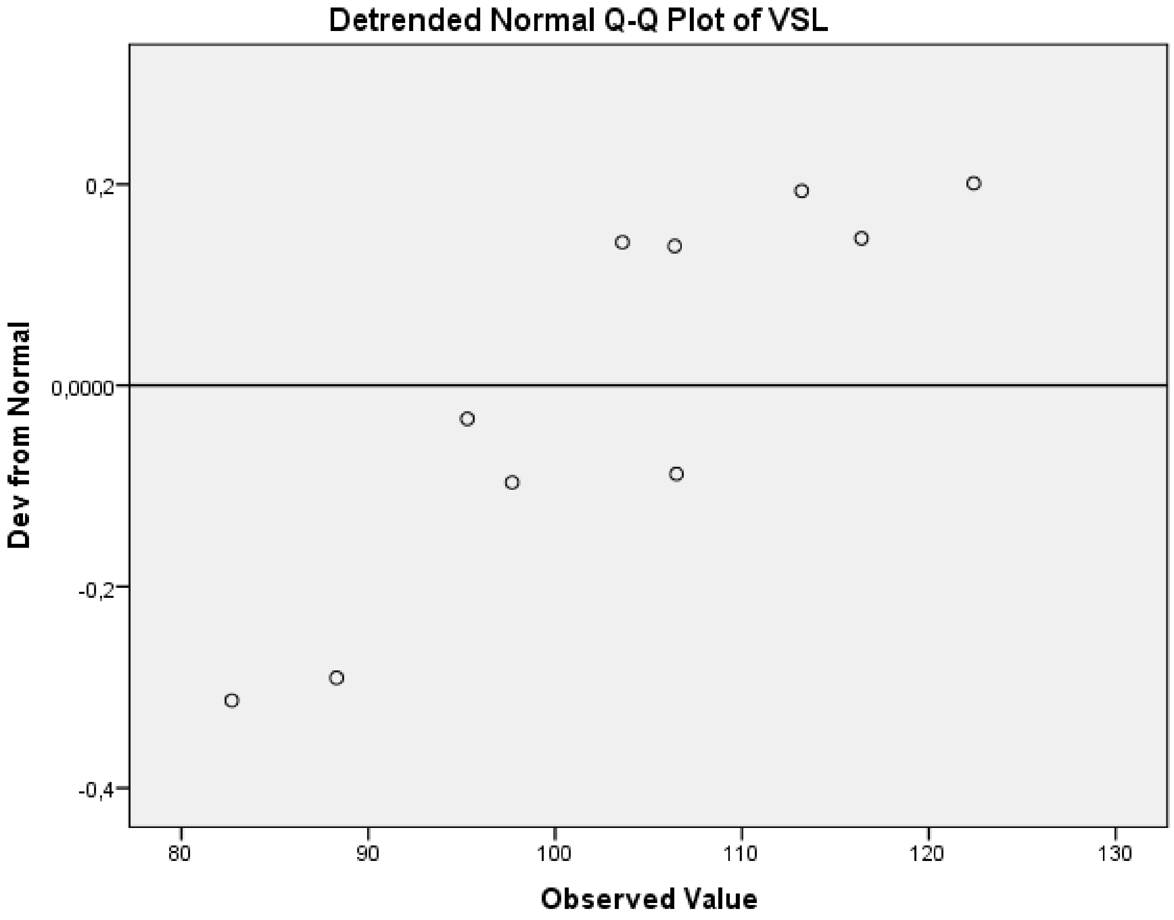
Detrended Normal Q-Q Plot of VSL
Dev from Normal
0,2
0,0000
-0,2
-0,4
80
90
100
110
120
130
Observed Value

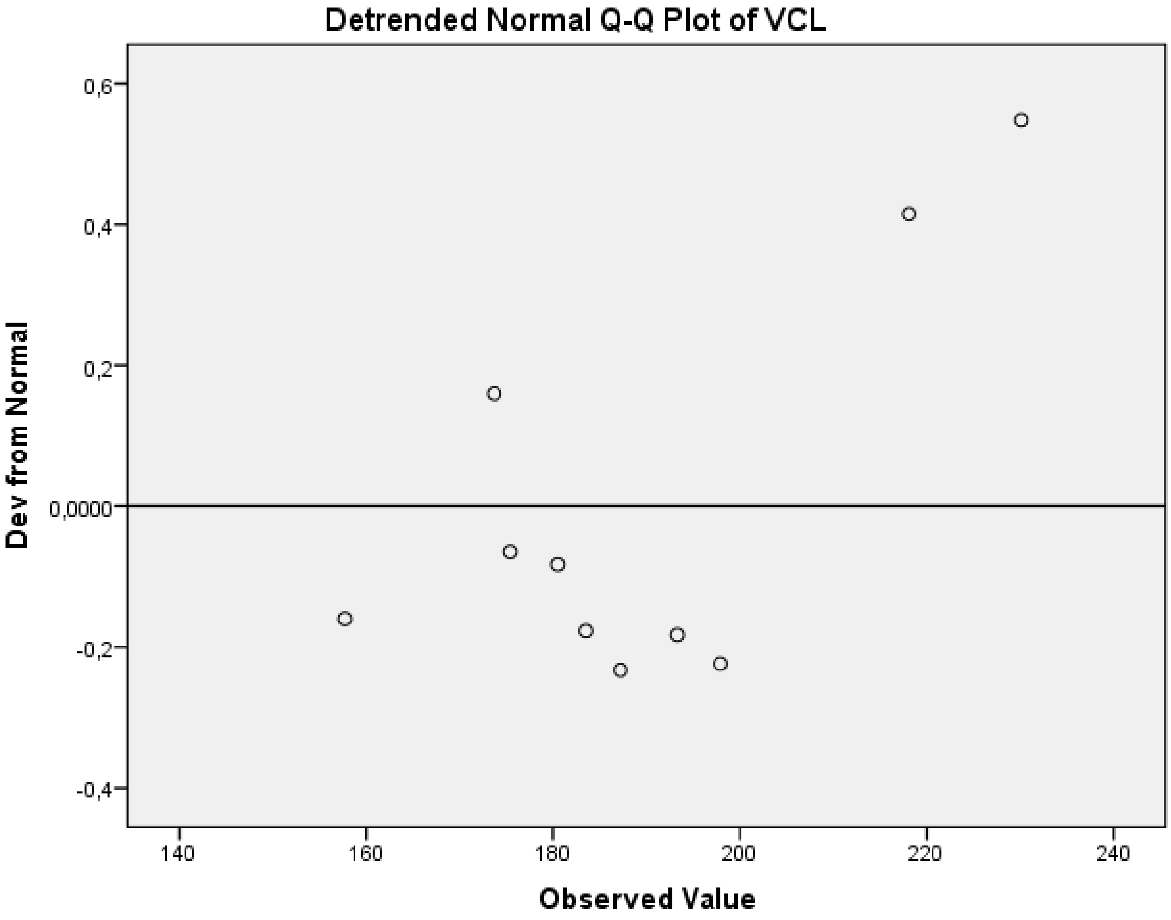
Detrended Normal Q-Q Plot of VCL
Dev from Normal
0,6
0,4
0,2
0,0000
-0,2
-0,4
140
160
180
200
220
240
Observed Value

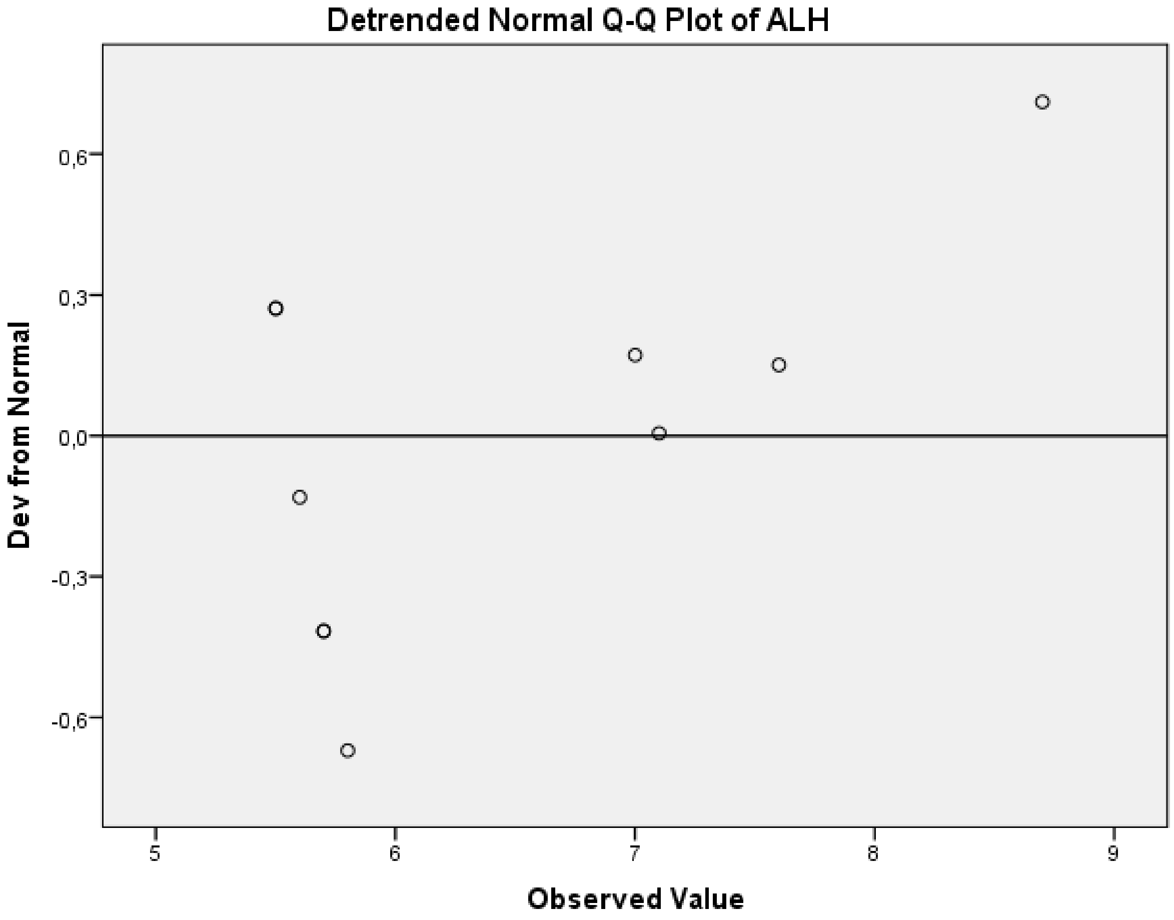
Detrended Normal Q-Q Plot of ALH
Dev from Normal
0,6
0,3
0,0
-0,3
-0,6
5
6
7
8
9
Observed Value

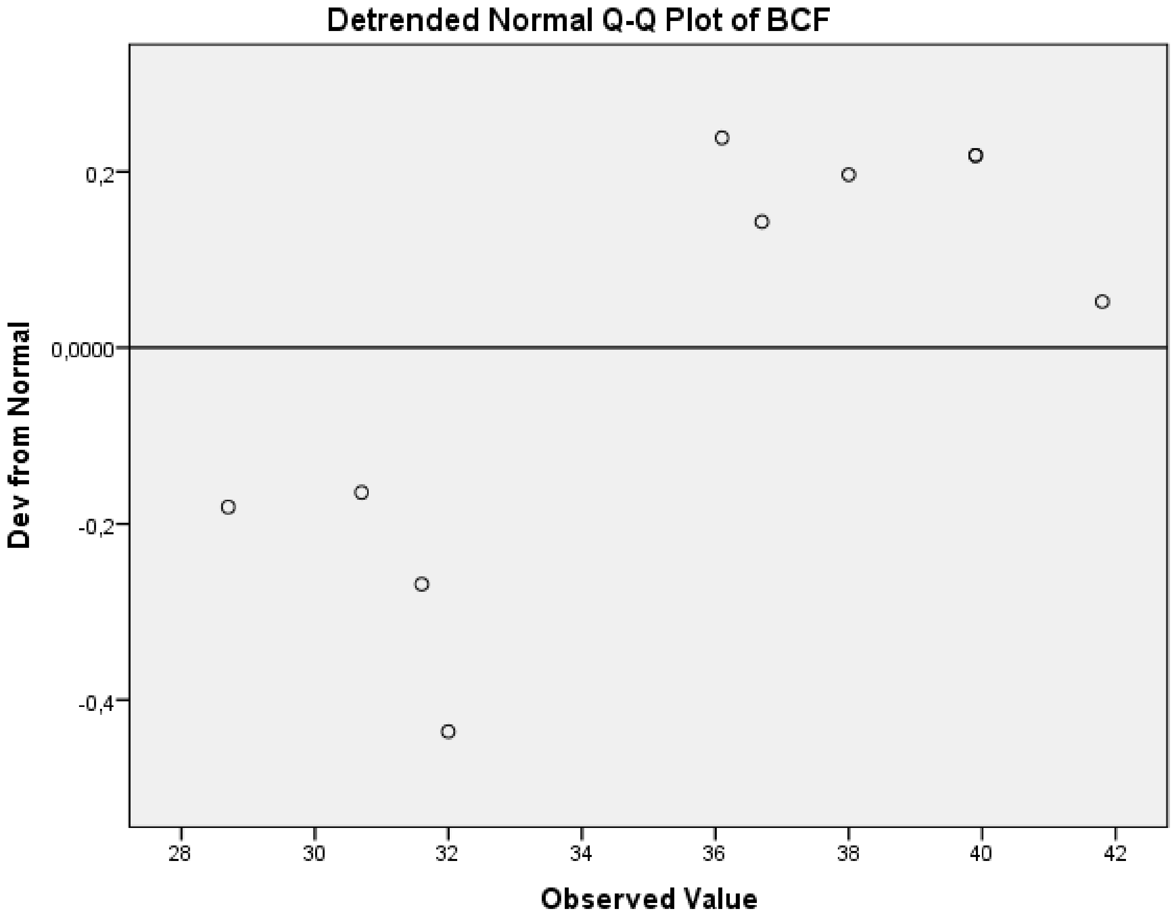

Detrended Normal Q-Q Plot of BCF
0,2
0,0000
-0,2
-0,4
Dev from Normal
28
30
32
34
36
38
40
42
Observed Value

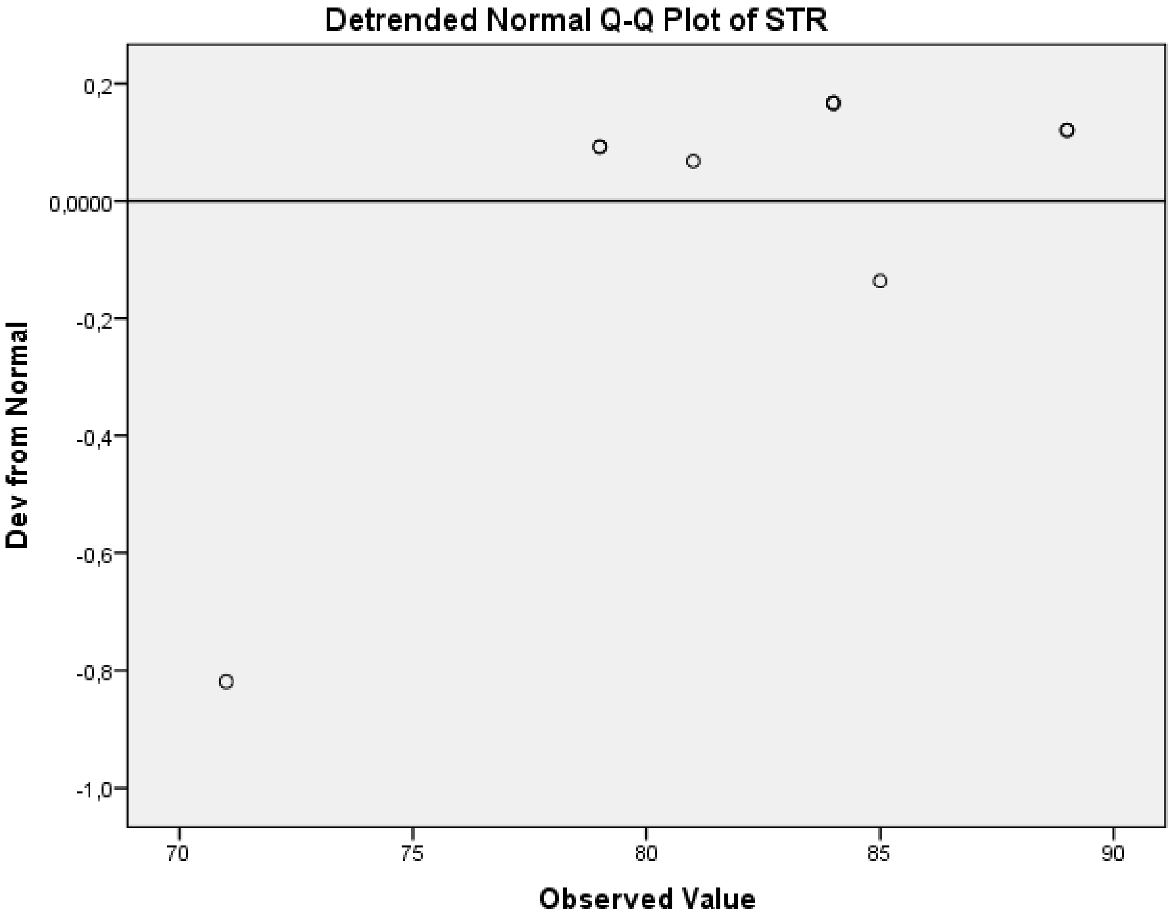
Detrended Normal Q-Q Plot of STR
Dev from Normal
Observed Value
0,2
0,0000
-0,2
-0,4
-0,6
-0,8
-1,0
70
75
80
85
90

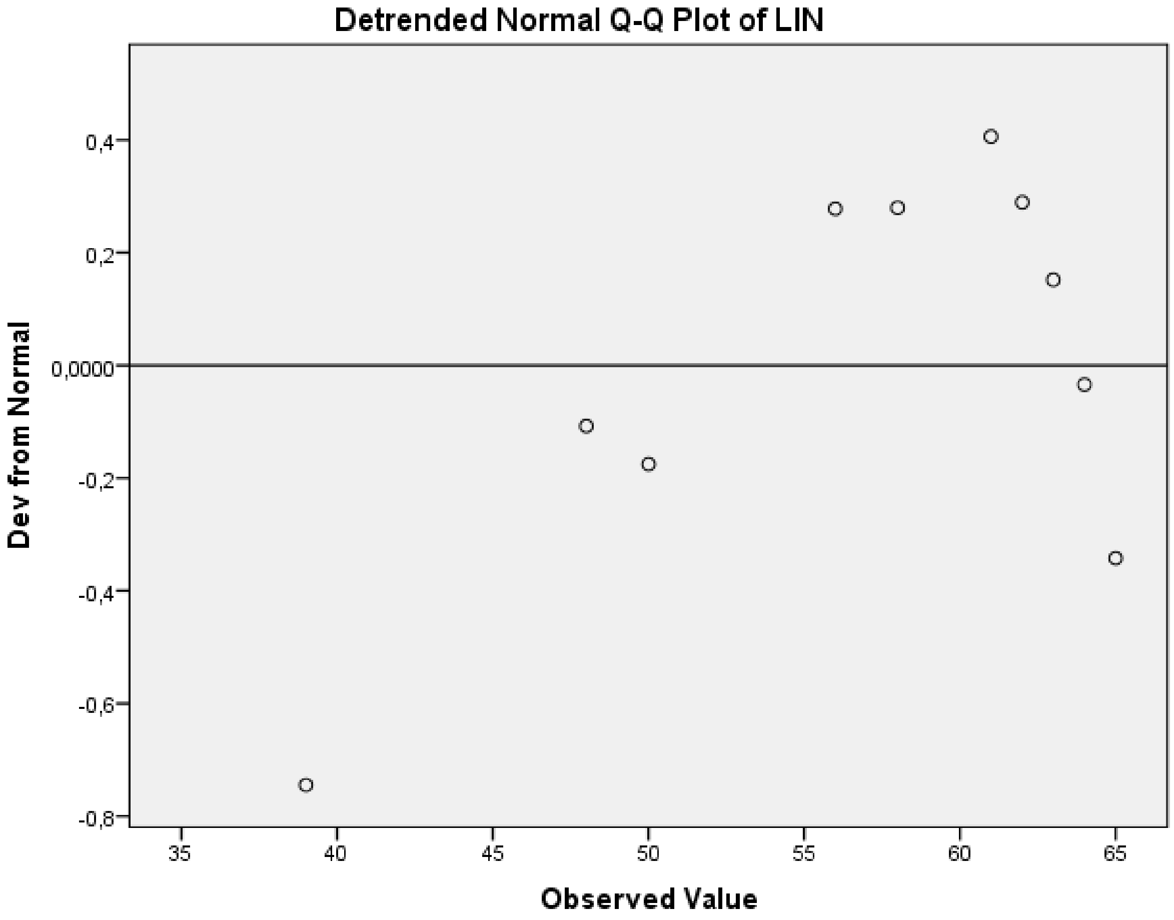

Grup = 7,00

Histograms

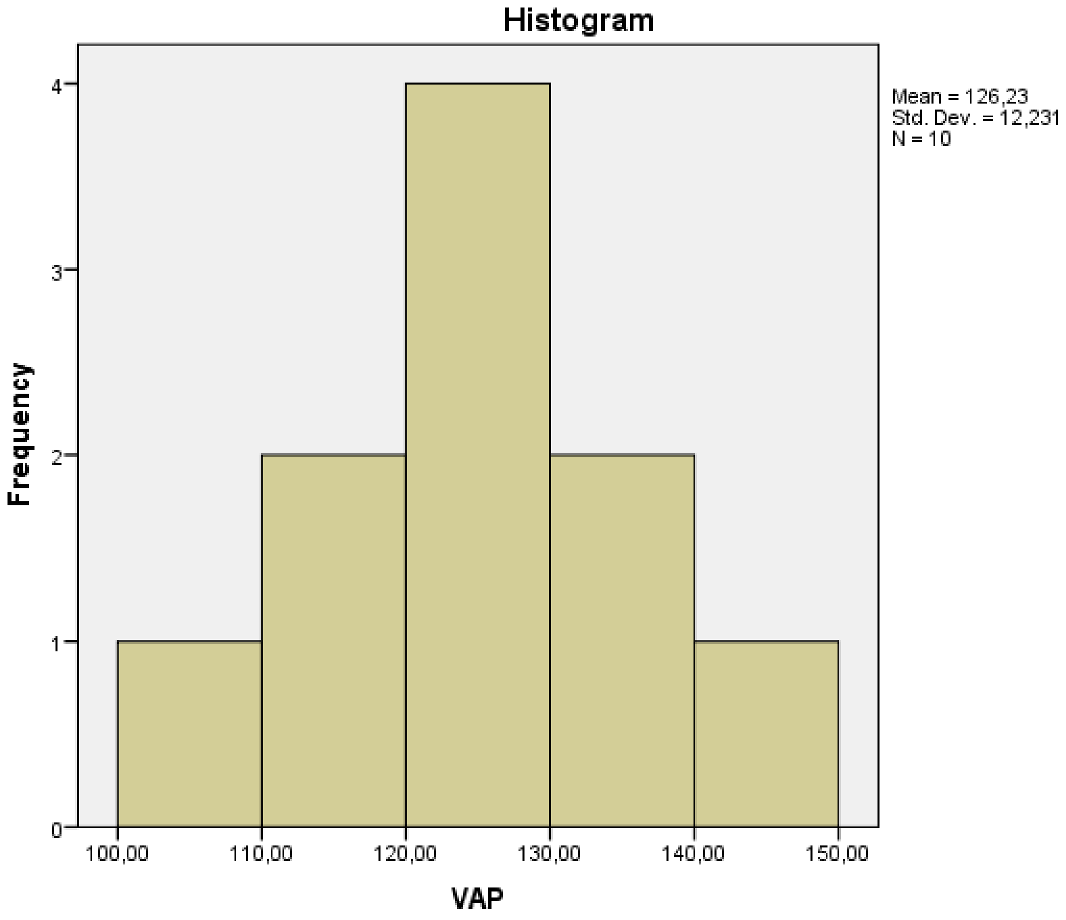
Histogram
Mean = 126,23
Std. Dev. = 12,231
N = 10
Frequency
0
1
2
3
4
100,00
110,00
120,00
130,00
140,00
150,00
VAP

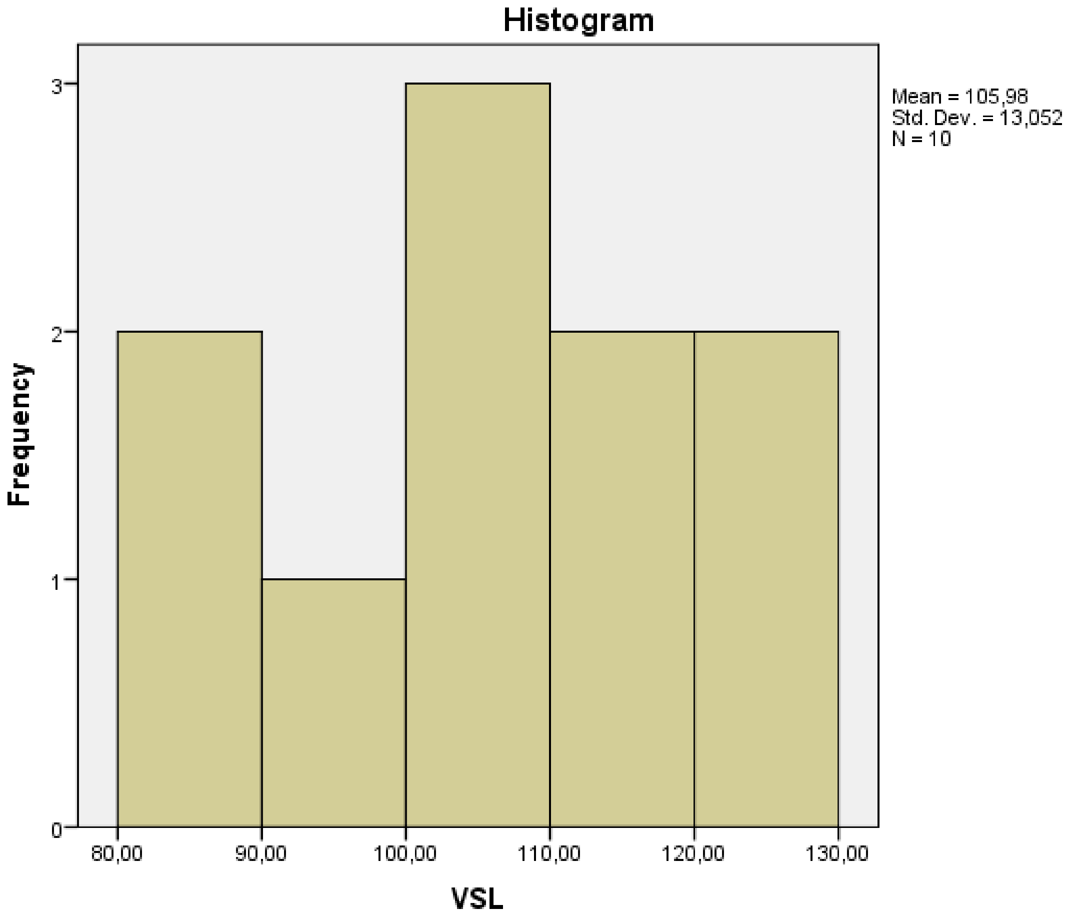
Histogram
Mean = 105,98
Std. Dev. = 13,052
N = 10
Frequency
0
1
2
3
80,00
90,00
100,00
110,00
120,00
130,00
VSL

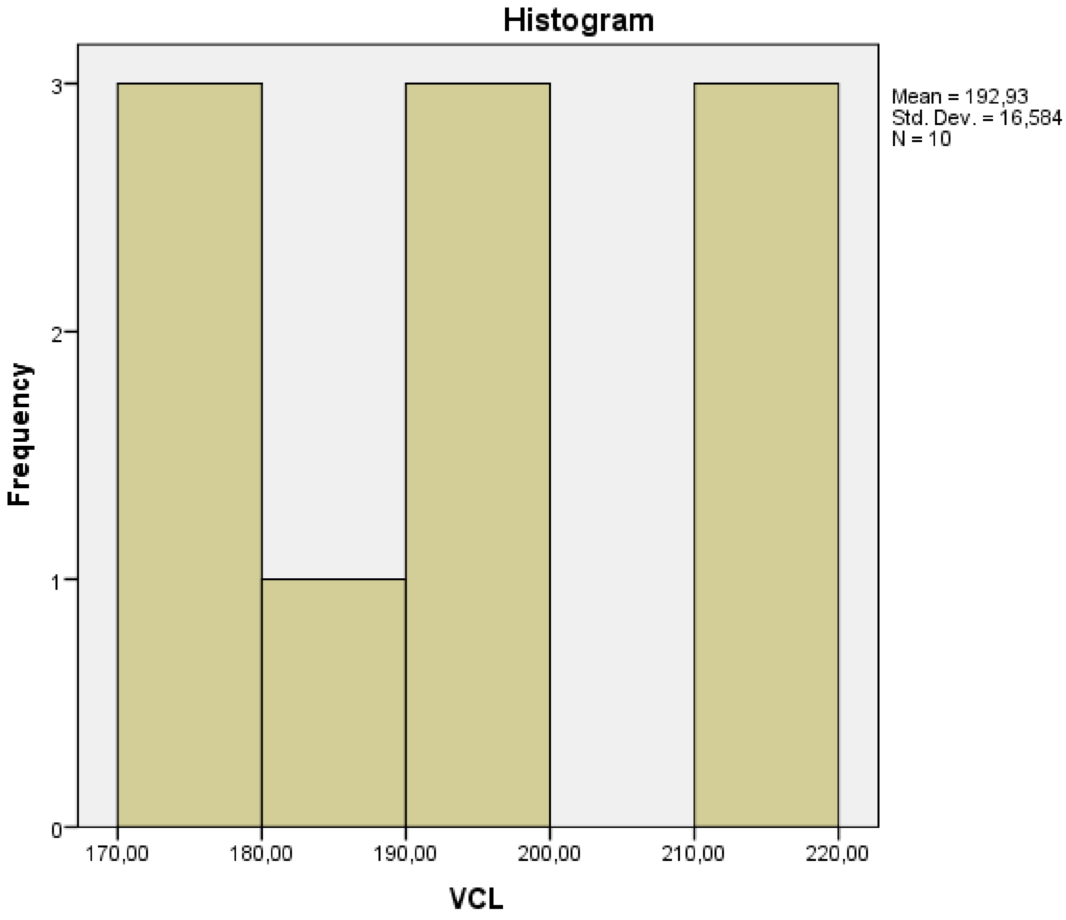

Histogram
Mean = 192,93
Std. Dev. = 16,584
N = 10
Frequency
0
1
2
3
170,00
180,00
190,00
200,00
210,00
220,00
VCL

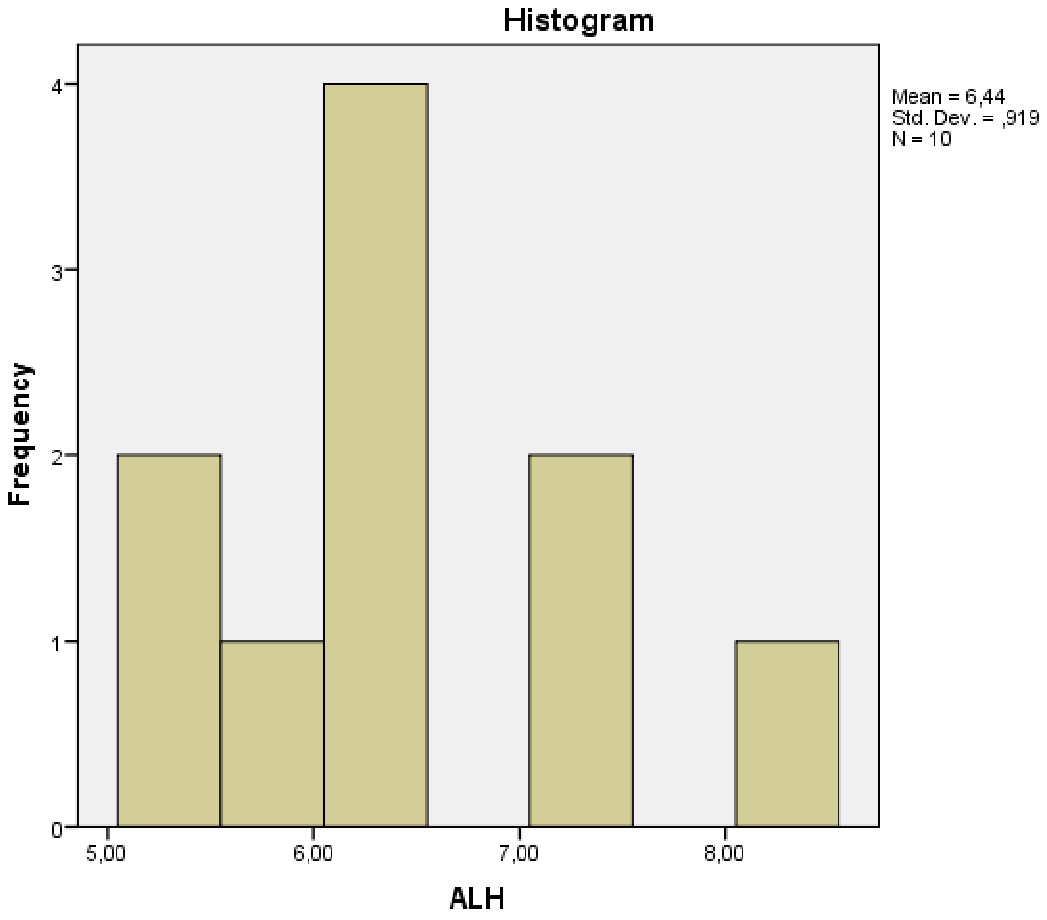
Histogram
Mean = 6,44
Std. Dev. = ,919
N = 10
Frequency
0
1
2
3
4
5,00
6,00
7,00
8,00
ALH

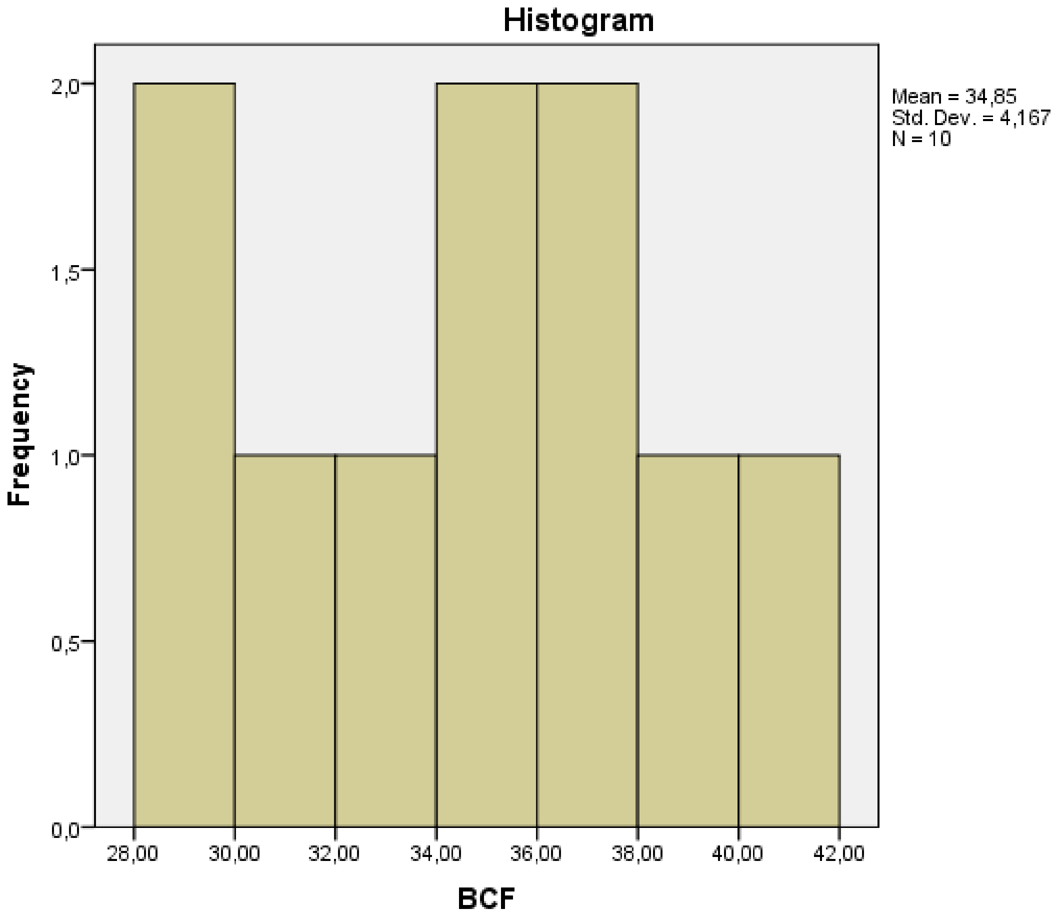

Histogram
Mean = 34,85
Std. Dev. = 4,167
N = 10
Frequency
2,0
1,5
1,0
0,5
0,0
28,00
30,00
32,00
34,00
36,00
38,00
40,00
42,00
BCF

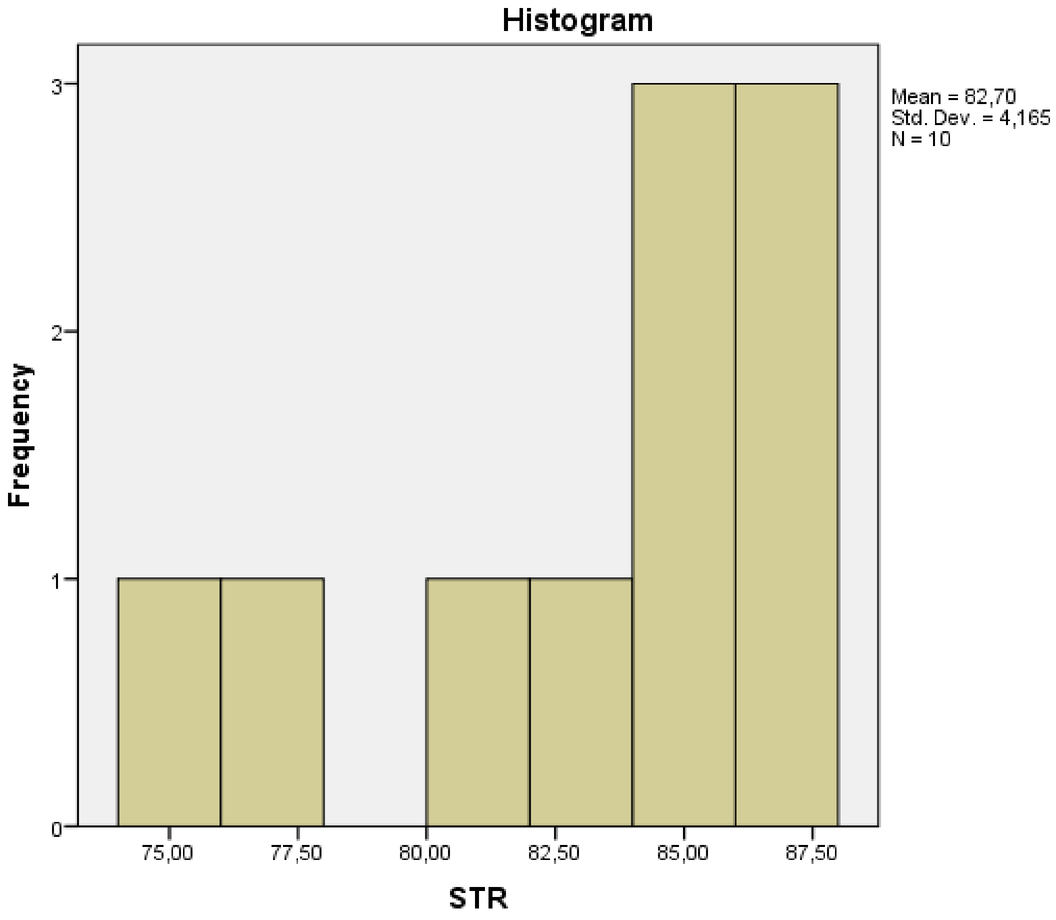
Histogram
Mean = 82,70
Std. Dev. = 4,165
N = 10
Frequency
0
1
2
3
75,00
77,50
80,00
82,50
85,00
87,50
STR

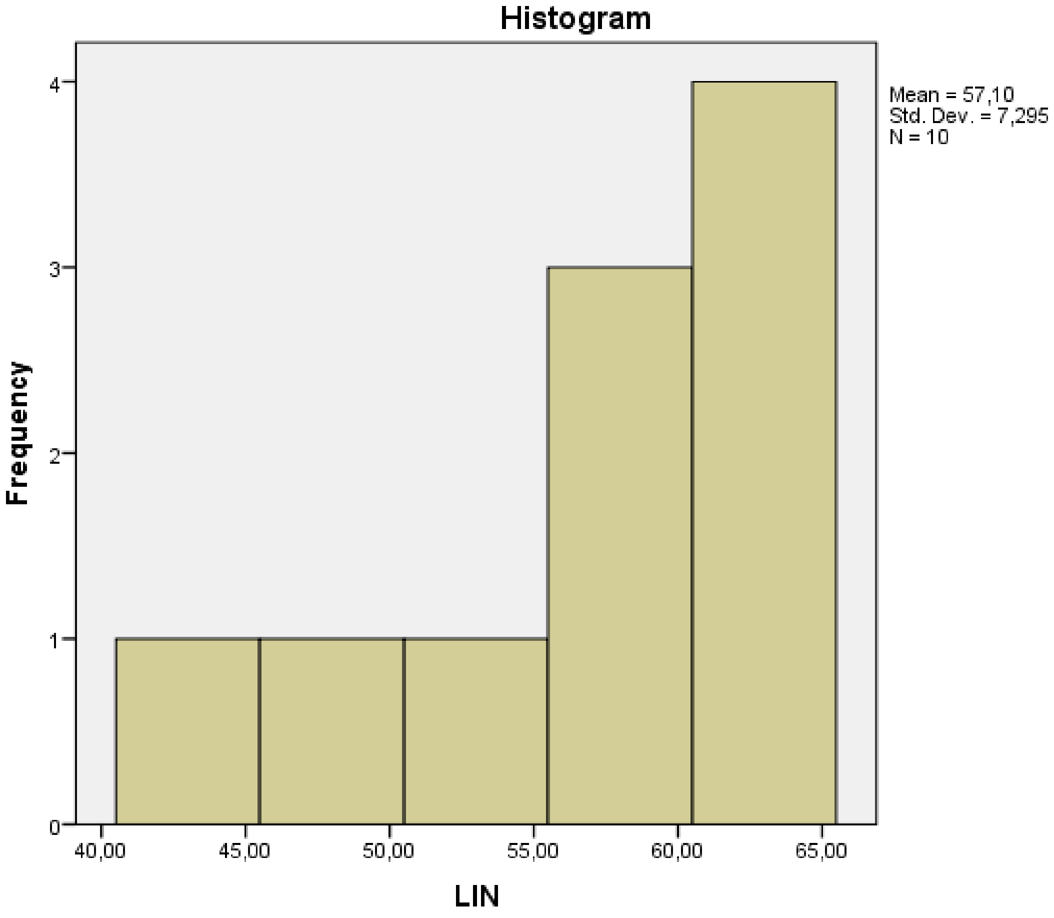

Stem-and-Leaf Plots

```
VAP Stem-and-Leaf Plot for
Grup= 7,00

 Frequency    Stem &  Leaf

     1,00       10 .  4
     2,00       11 .  79
     4,00       12 .  1566
     2,00       13 .  49
     1,00       14 .  8

 Stem width:  10,00
 Each leaf:       1 case(s)
```

VSL Stem-and-Leaf Plot for
Grup= 7,00

Frequency	Stem &	Leaf
2,00	8 .	59
1,00	9 .	7
3,00	10 .	167
2,00	11 .	02
2,00	12 .	07

Stem width: 10,00
Each leaf: 1 case(s)

VCL Stem-and-Leaf Plot for
Grup= 7,00

Frequency	Stem &	Leaf
3,00	17 .	556
1,00	18 .	1
3,00	19 .	024
,00	20 .	
3,00	21 .	148

Stem width: 10,00
Each leaf: 1 case(s)

ALH Stem-and-Leaf Plot for
Grup= 7,00

Frequency	Stem &	Leaf
3,00	5 .	348
4,00	6 .	1245
2,00	7 .	13
1,00	8 .	3

Stem width: 1,00
Each leaf: 1 case(s)

BCF Stem-and-Leaf Plot for
Grup= 7,00

Frequency   Stem & Leaf

```
     2,00        2 .  89
     2,00        3 .  03
     5,00        3 .  55779
     1,00        4 .  0

 Stem width:  10,00
 Each leaf:       1 case(s)
```

```
STR Stem-and-Leaf Plot for
Grup= 7,00

 Frequency    Stem &  Leaf

      ,00        7 .
     2,00        7 .  57
     4,00        8 .  0244
     4,00        8 .  5677

 Stem width:  10,00
 Each leaf:       1 case(s)
```

```
LIN Stem-and-Leaf Plot for
Grup= 7,00

 Frequency    Stem &  Leaf

     1,00        4 .  3
     1,00        4 .  8
     1,00        5 .  1
     2,00        5 .  89
     4,00        6 .  0133
     1,00        6 .  5

 Stem width:  10,00
 Each leaf:       1 case(s)
```

Normal Q-Q Plots

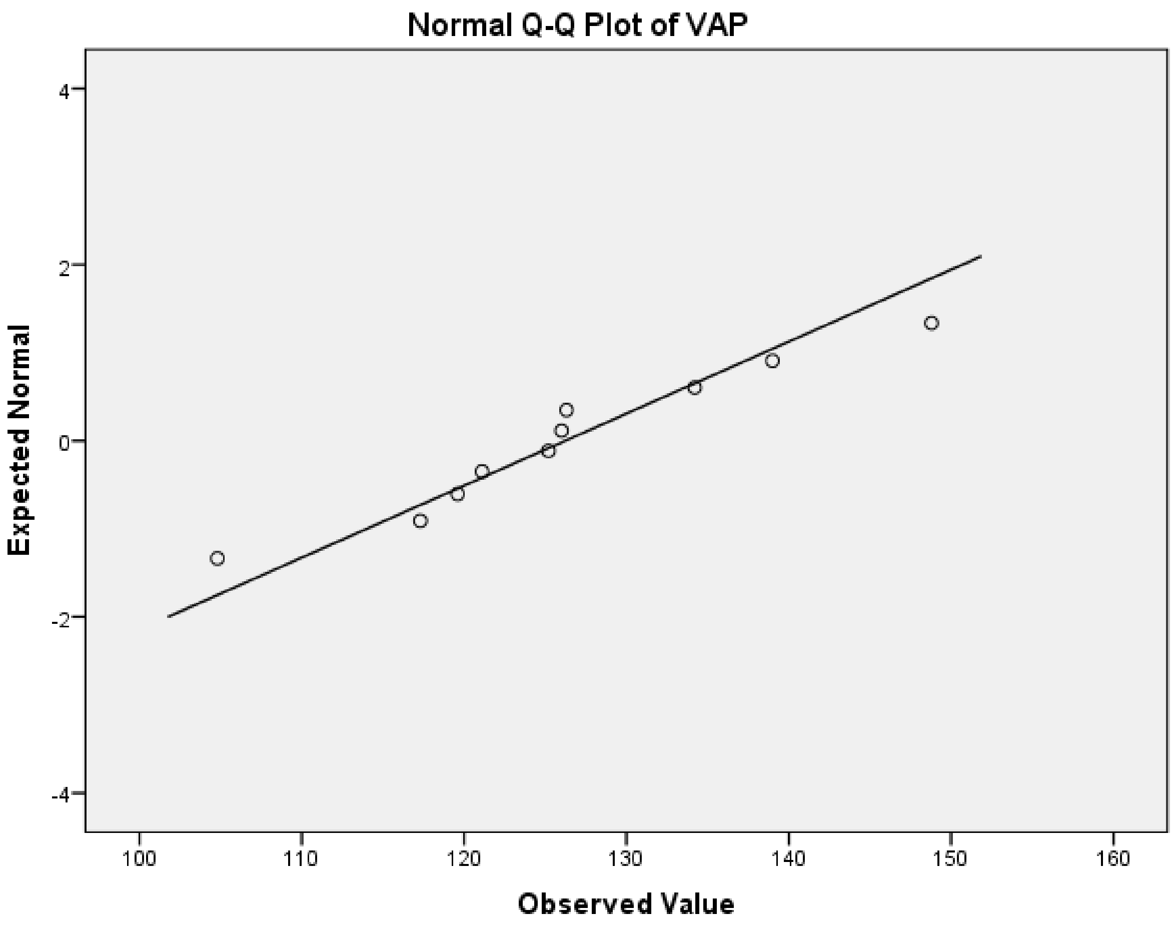
Normal Q-Q Plot of VAP
Expected Normal
4
2
0
-2
-4
100
110
120
130
140
150
160
Observed Value

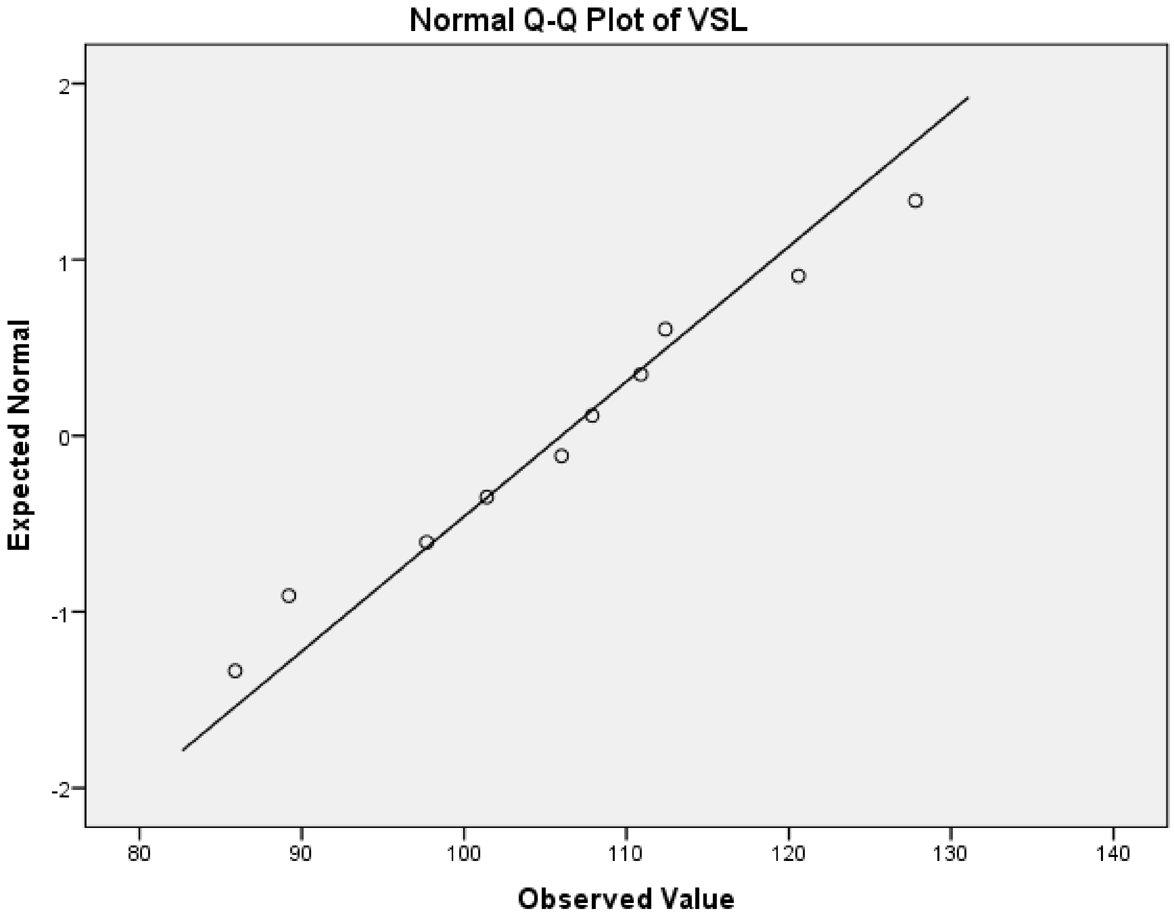
Normal Q-Q Plot of VSL
Expected Normal
2
1
0
-1
-2
80
90
100
110
120
130
140
Observed Value

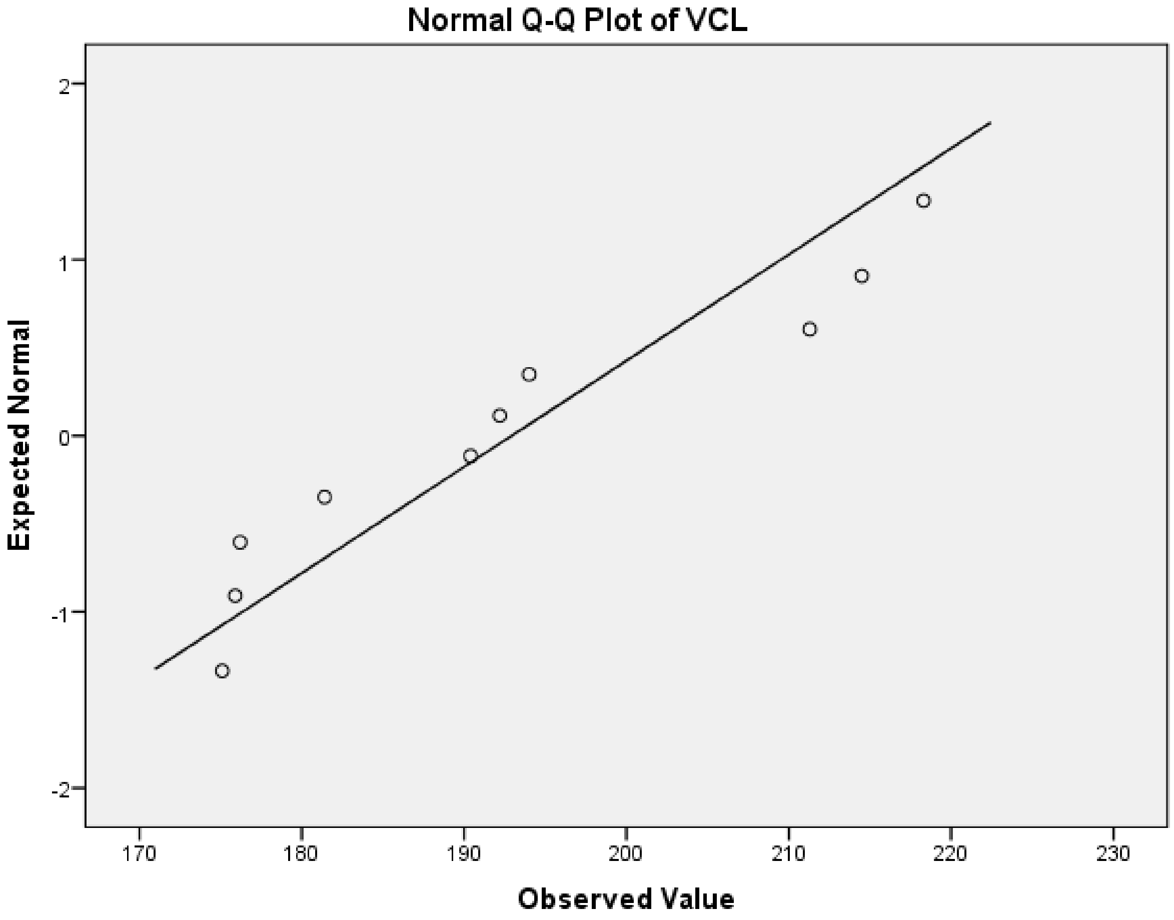

Normal Q-Q Plot of VCL
Expected Normal
Observed Value
2
1
0
-1
-2
170
180
190
200
210
220
230

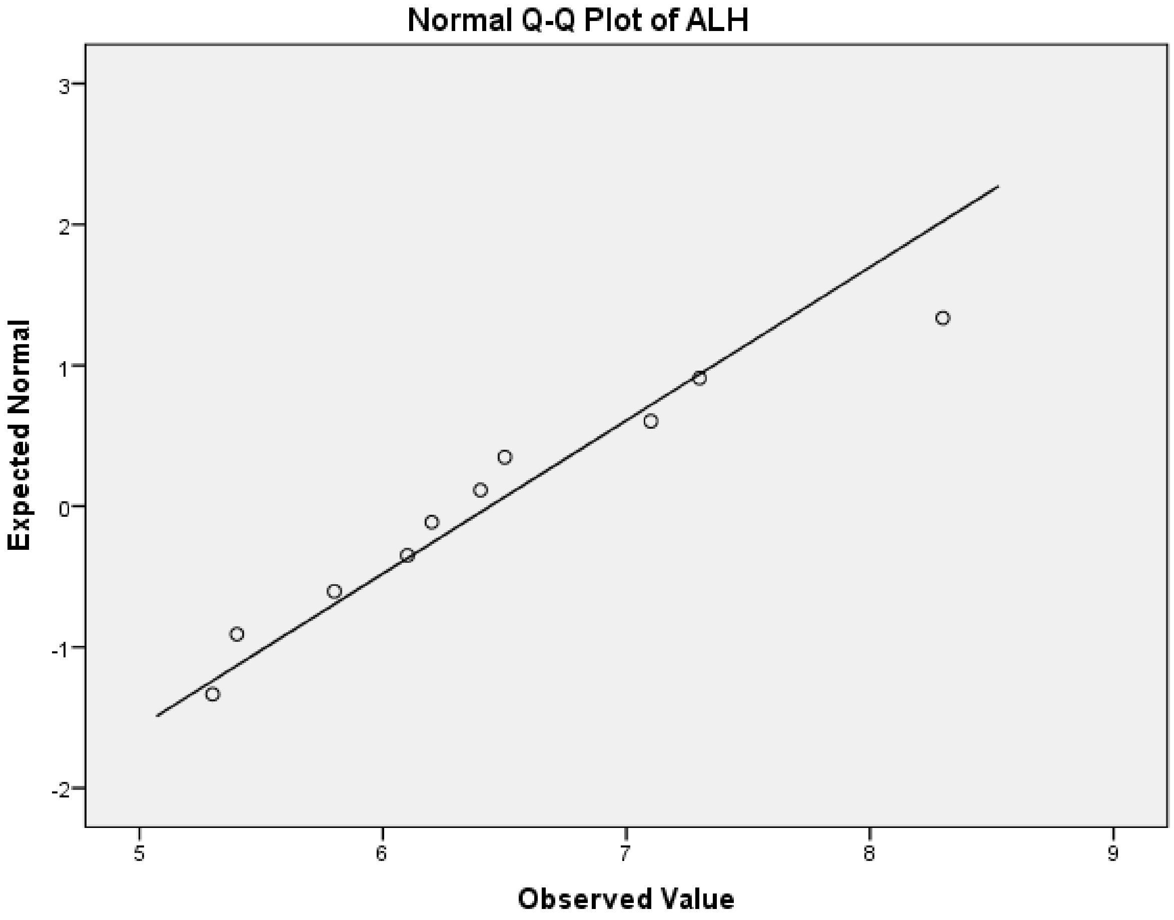
Normal Q-Q Plot of ALH
Expected Normal
Observed Value
3
2
1
0
-1
-2
5
6
7
8
9

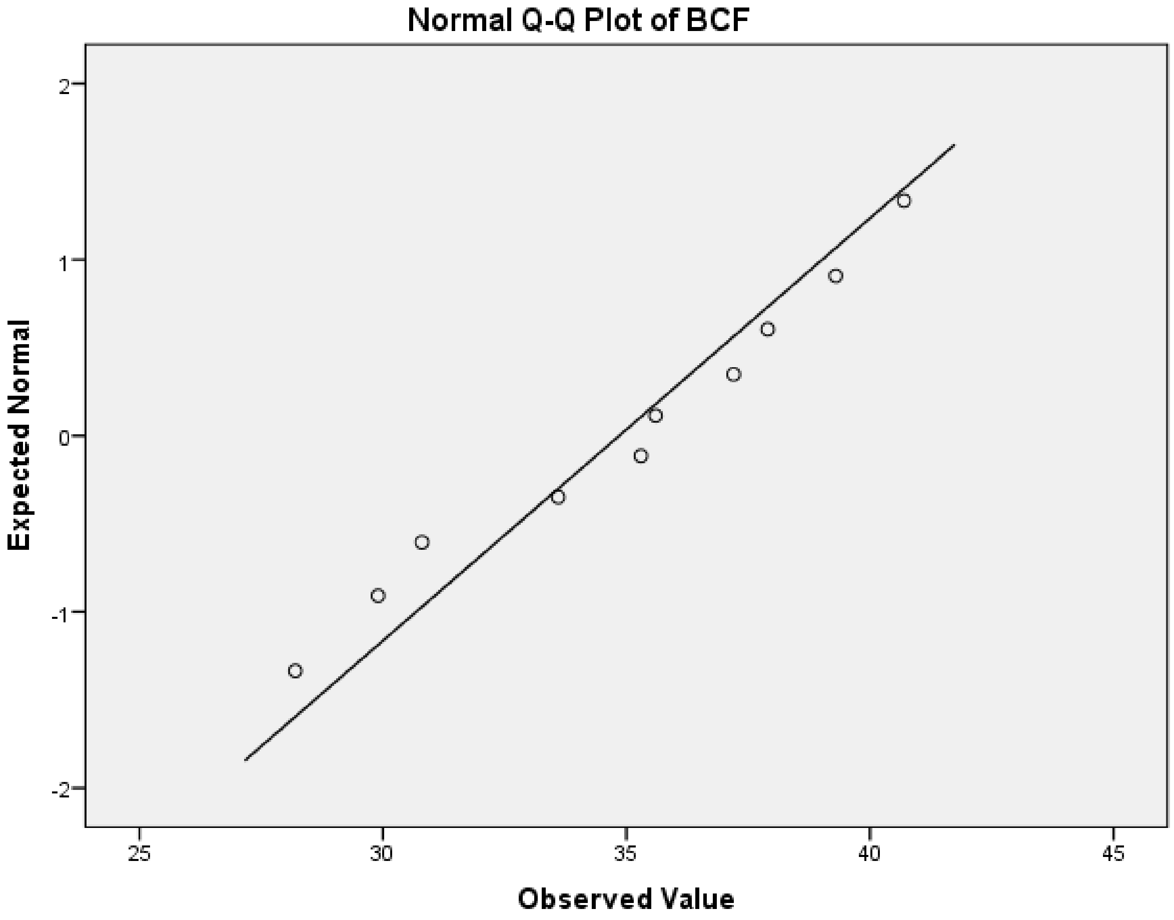
Normal Q-Q Plot of BCF
Expected Normal
2
1
0
-1
-2
25
30
35
40
45
Observed Value

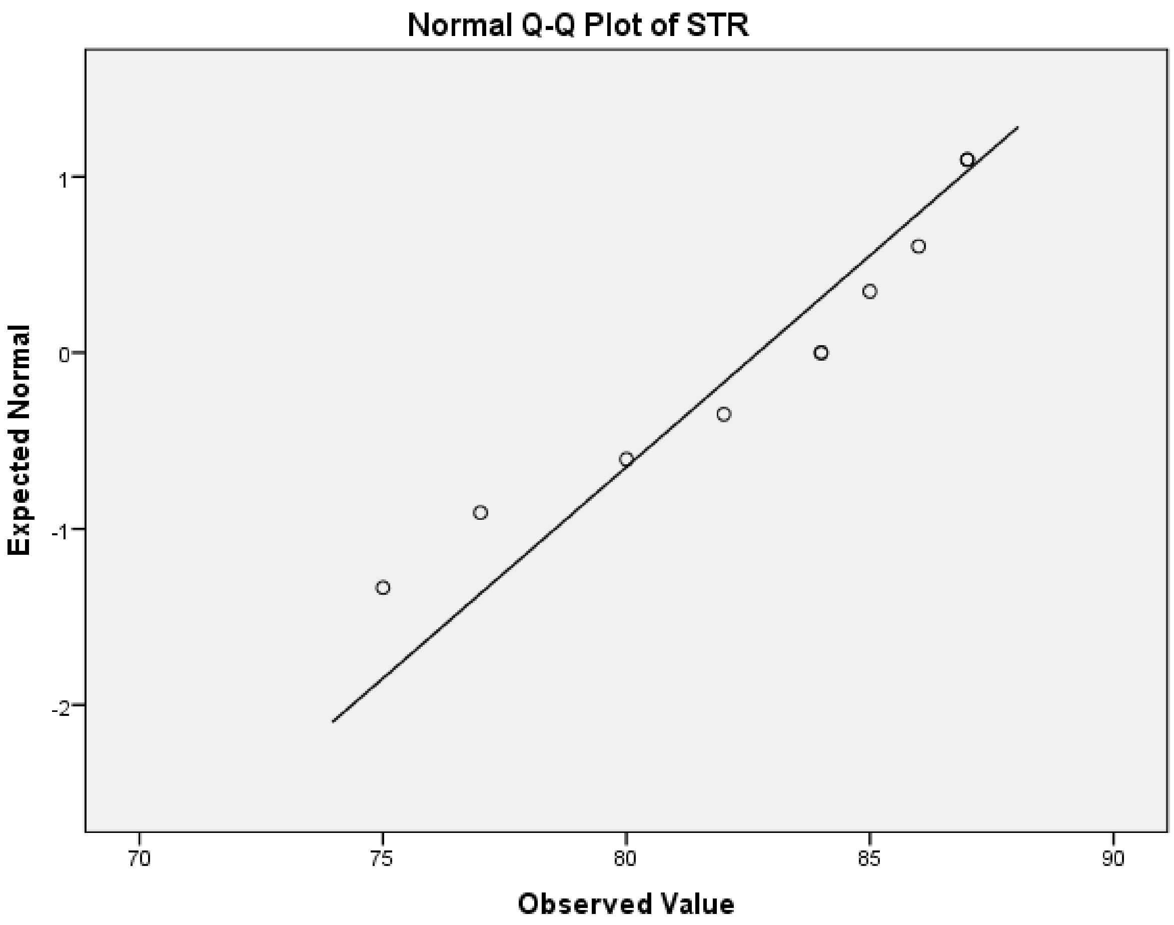

Normal Q-Q Plot of STR
Expected Normal
1
0
-1
-2
70
75
80
85
90
Observed Value

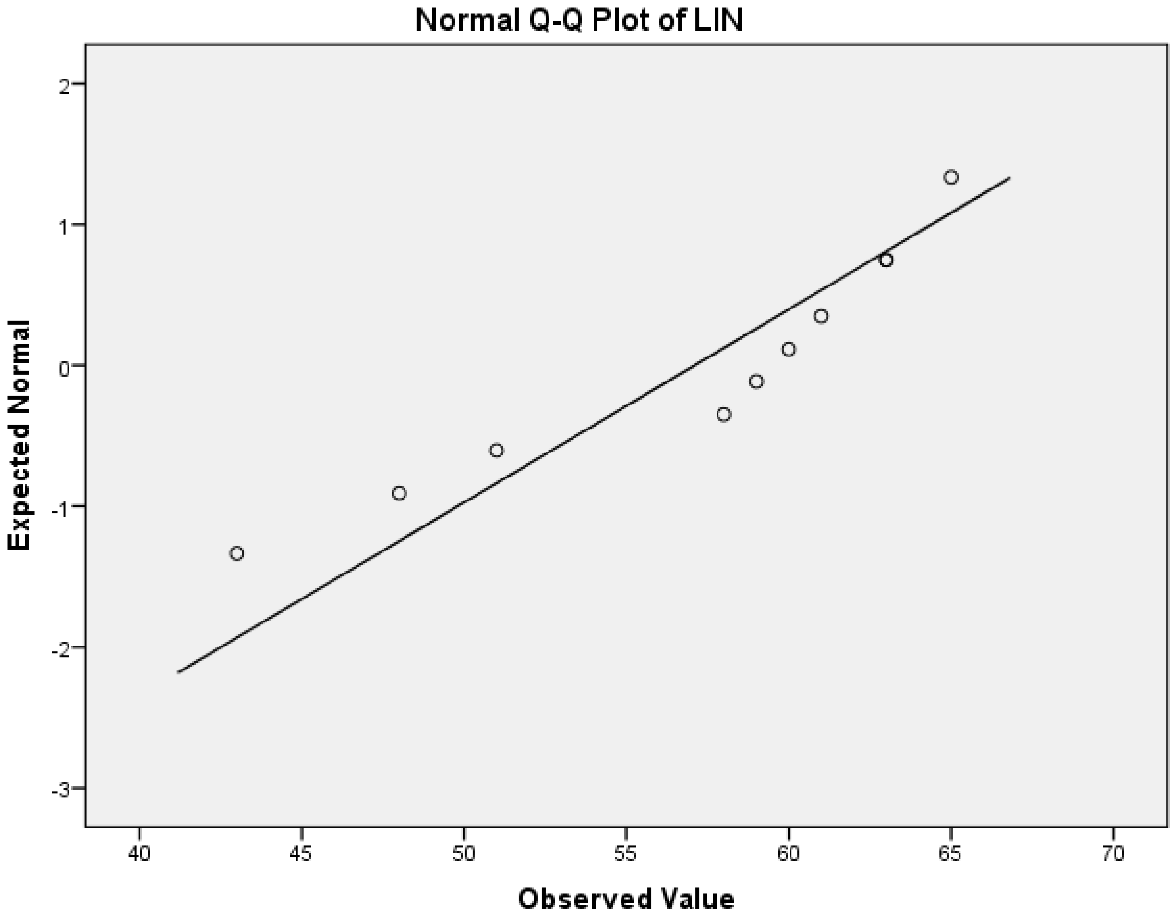

Detrended Normal Q-Q Plots

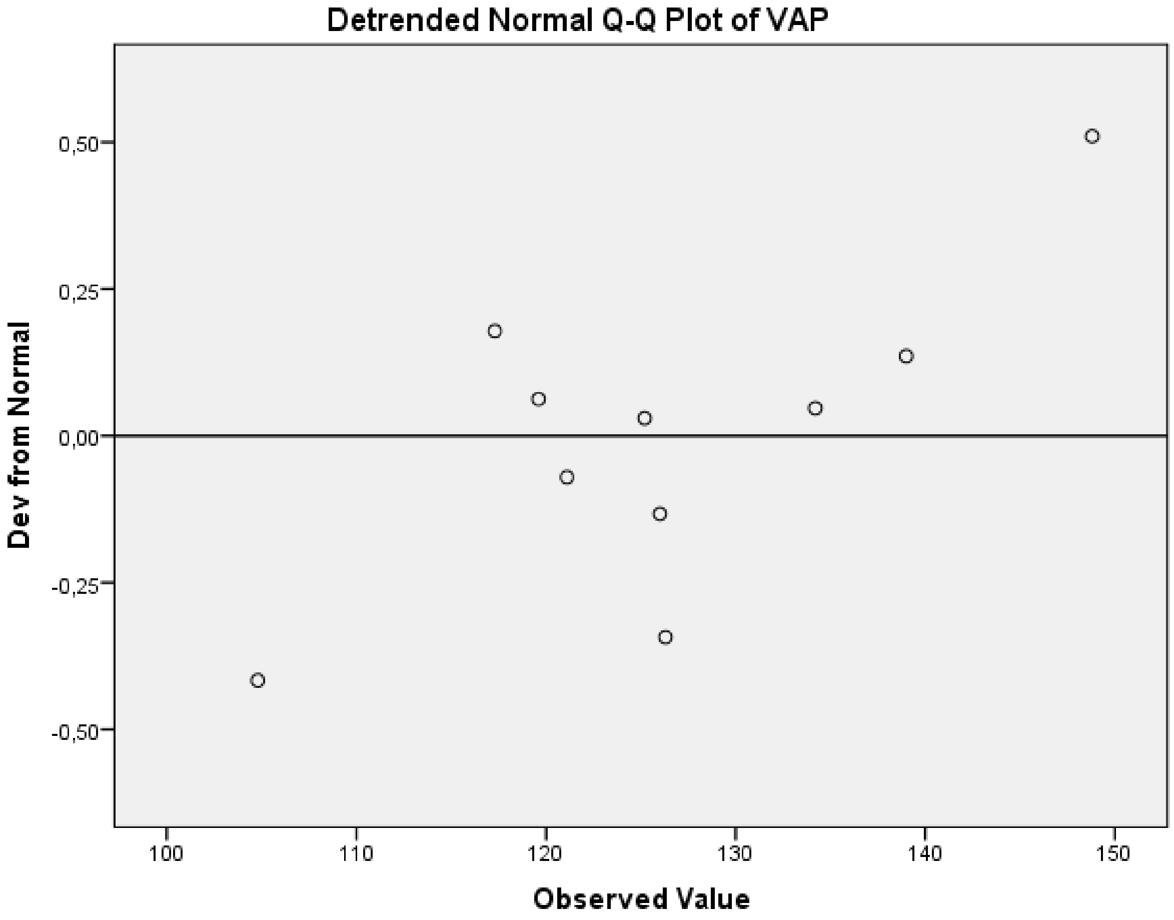
Detrended Normal Q-Q Plot of VAP
Dev from Normal
0,50
0,25
0,00
-0,25
-0,50
100
110
120
130
140
150
Observed Value

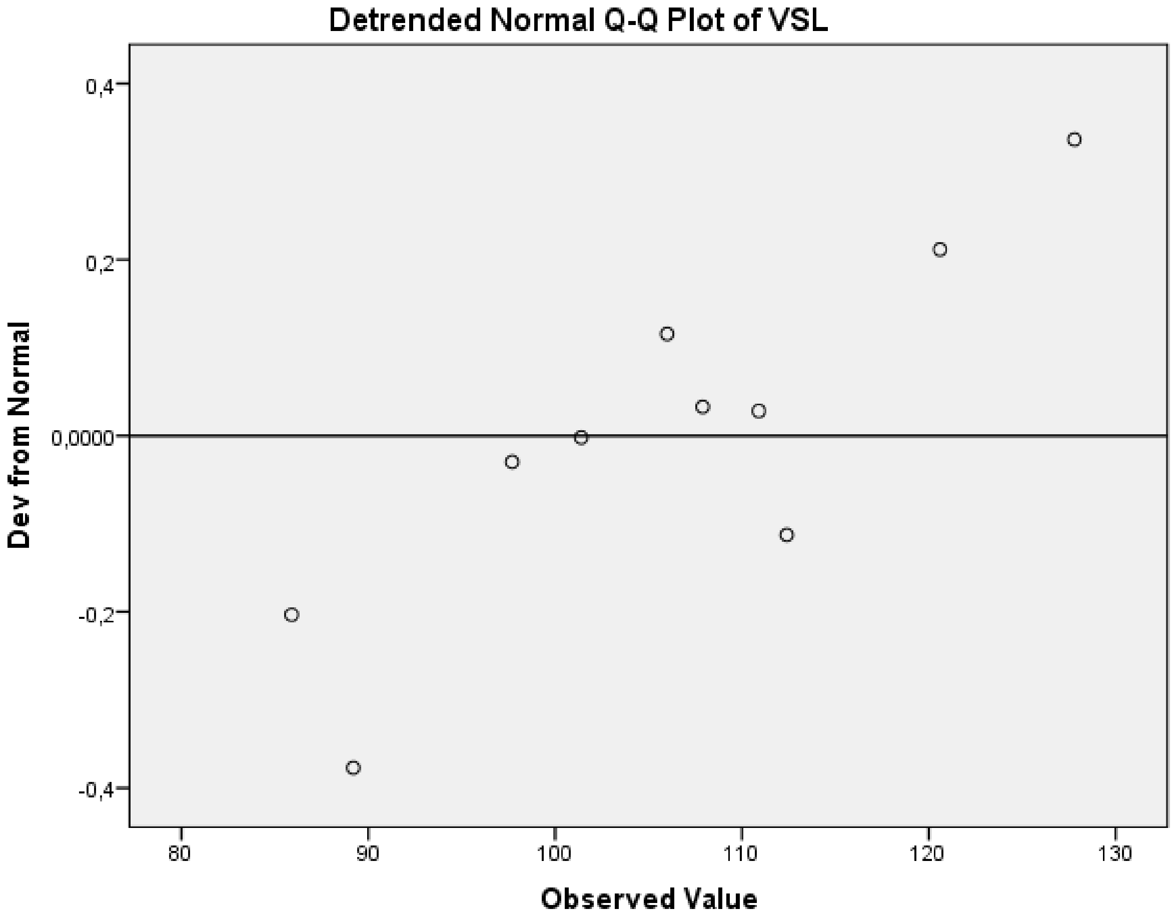
Detrended Normal Q-Q Plot of VSL
Dev from Normal
0,4
0,2
0,0000
-0,2
-0,4
80
90
100
110
120
130
Observed Value

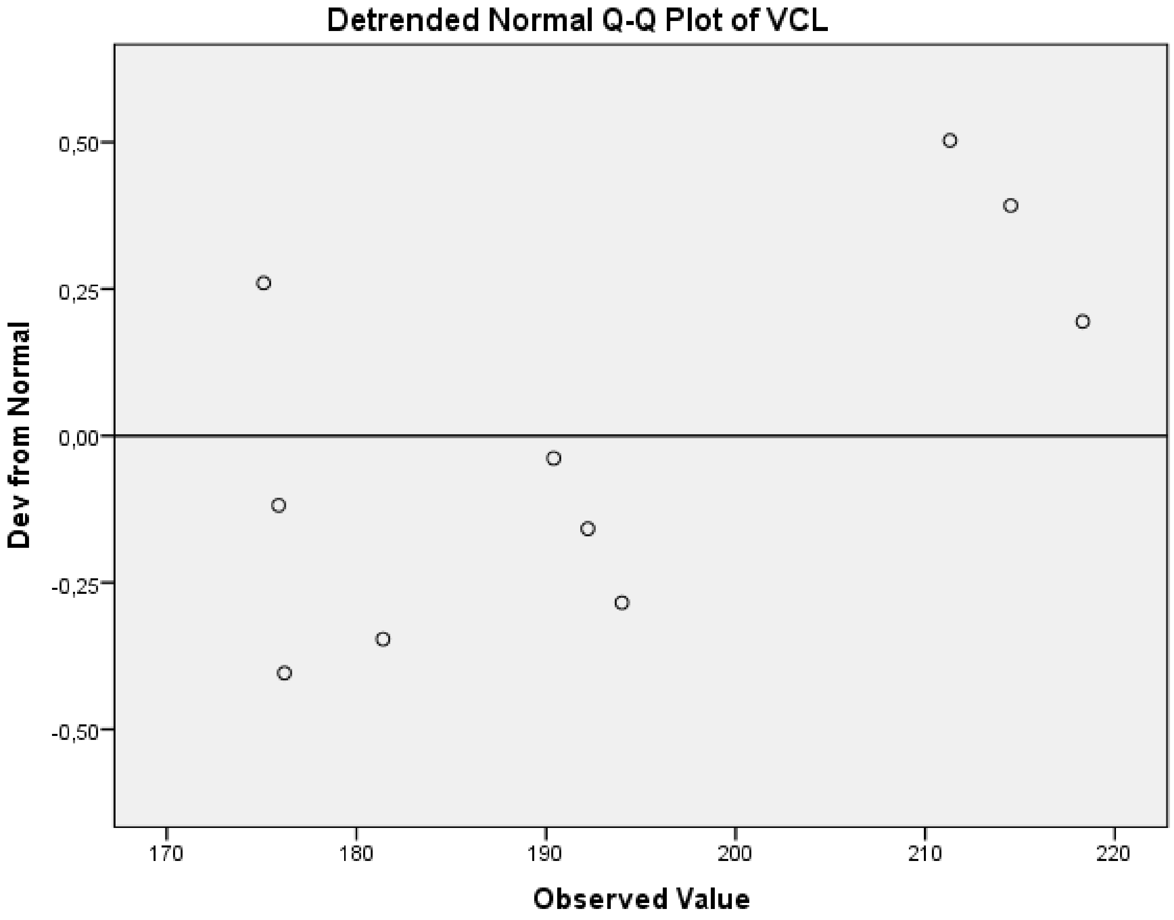

Detrended Normal Q-Q Plot of VCL
Dev from Normal
0,50
0,25
0,00
-0,25
-0,50
170
180
190
200
210
220
Observed Value

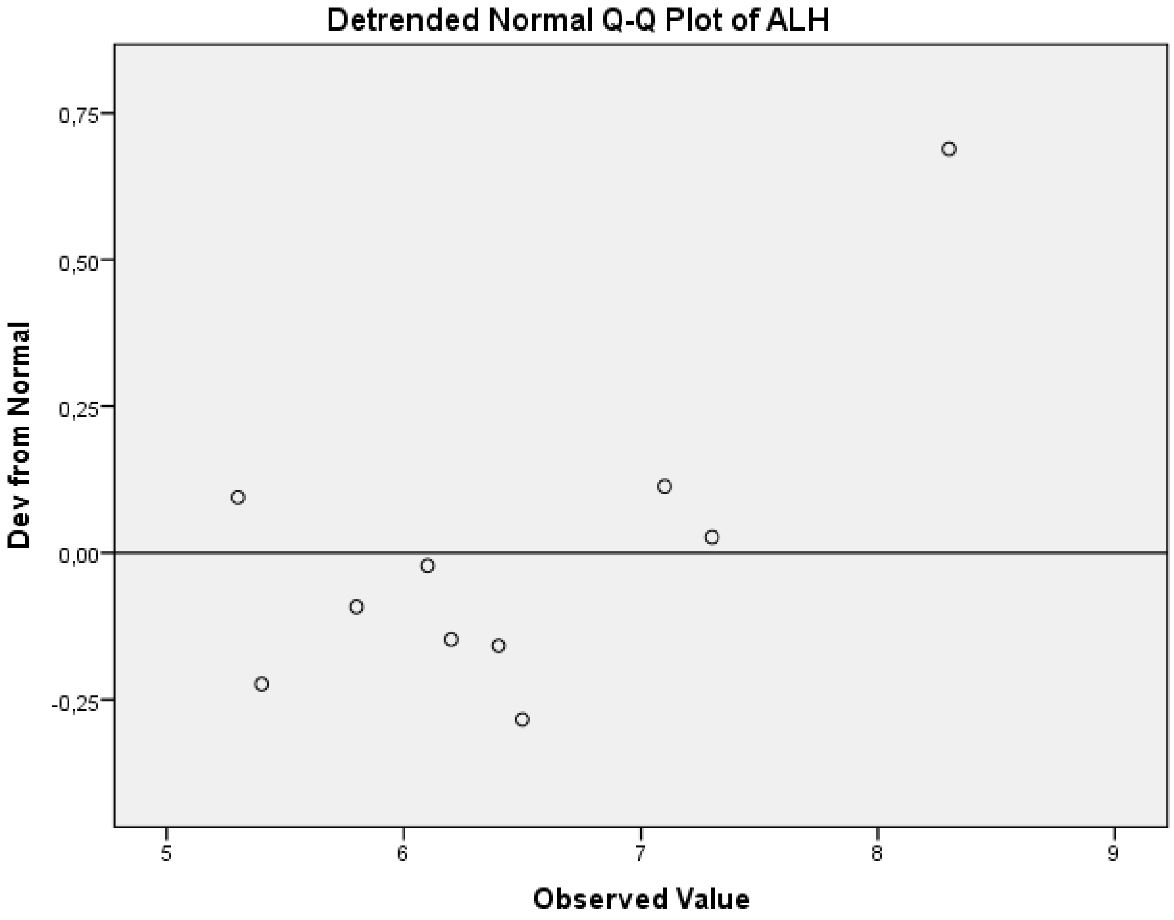

Detrended Normal Q-Q Plot of ALH
Dev from Normal
0,75
0,50
0,25
0,00
-0,25
5
6
7
8
9
Observed Value

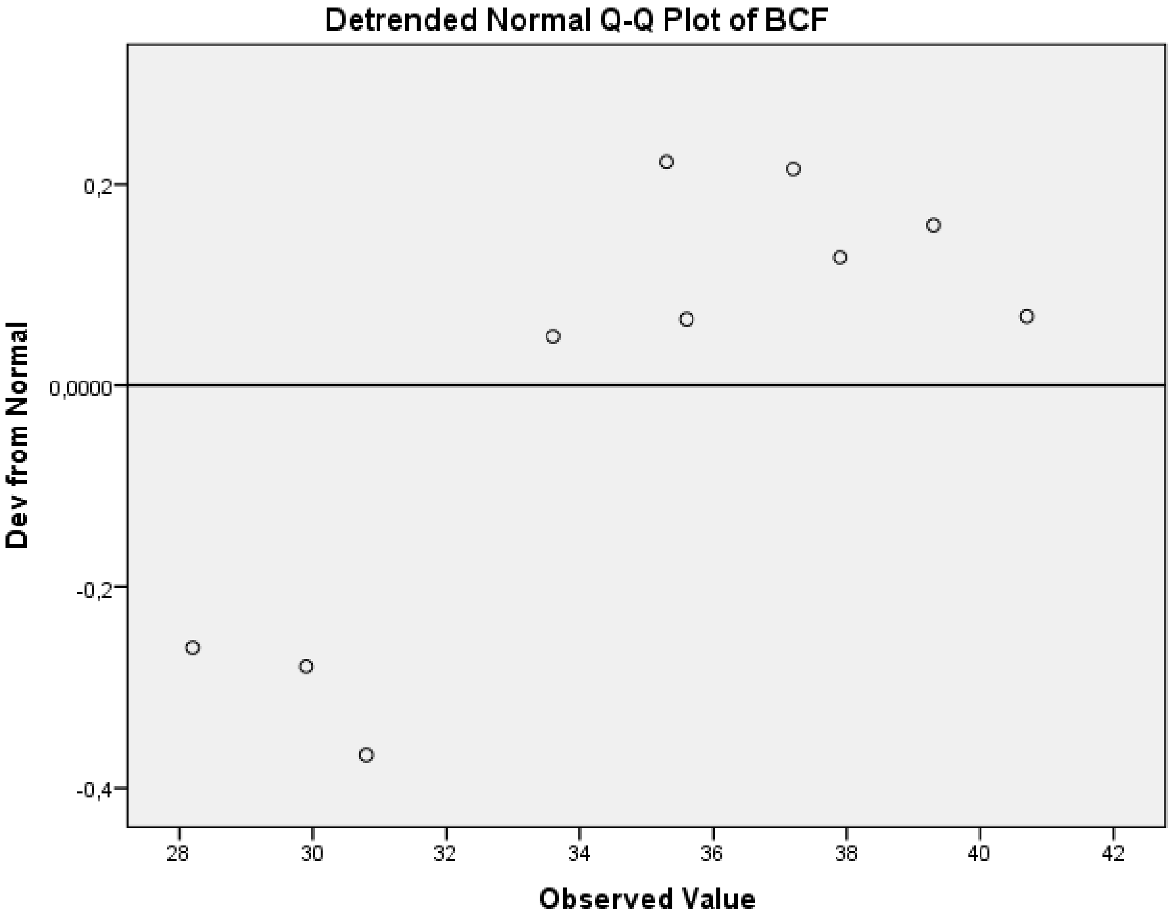

Detrended Normal Q-Q Plot of BCF
Dev from Normal
0,2
0,0000
-0,2
-0,4
28
30
32
34
36
38
40
42
Observed Value

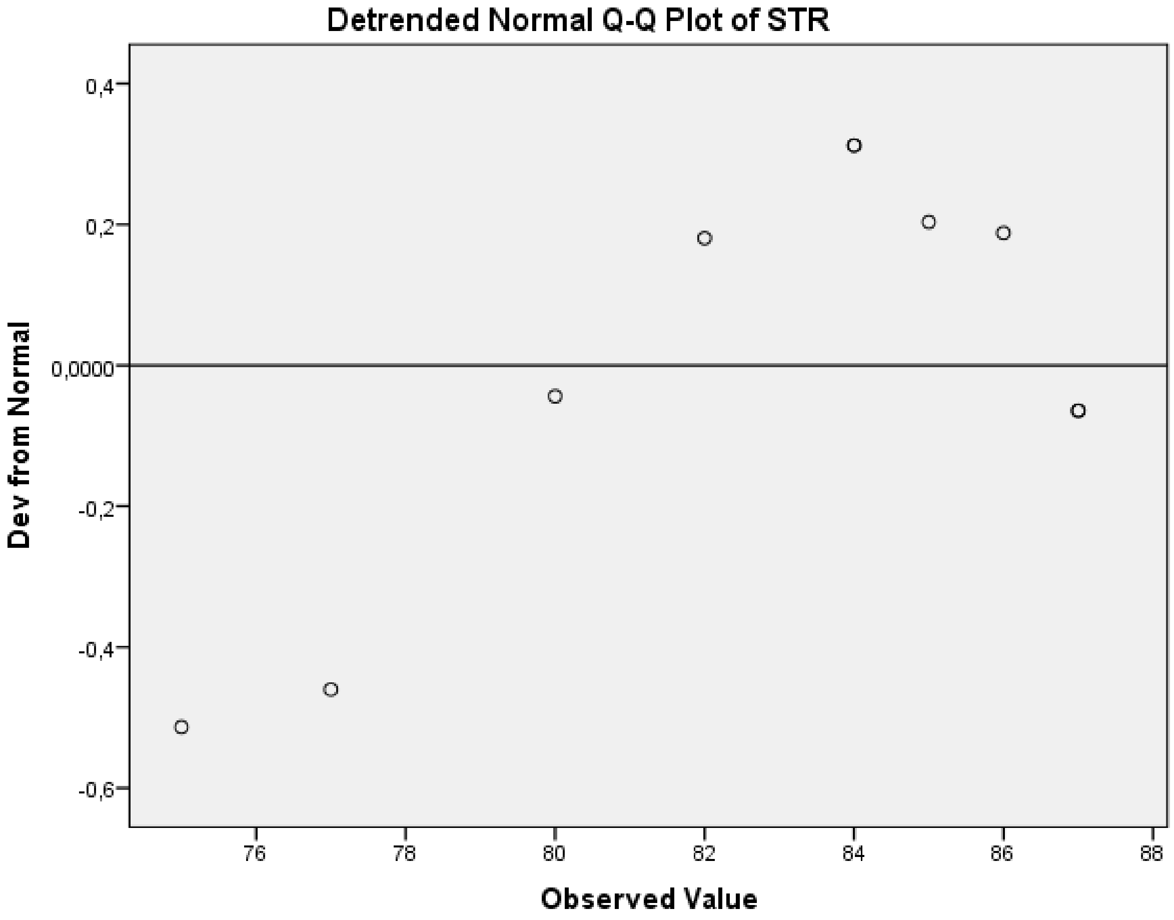

Detrended Normal Q-Q Plot of STR
Dev from Normal
Observed Value
0,4
0,2
0,0000
-0,2
-0,4
-0,6
76
78
80
82
84
86
88

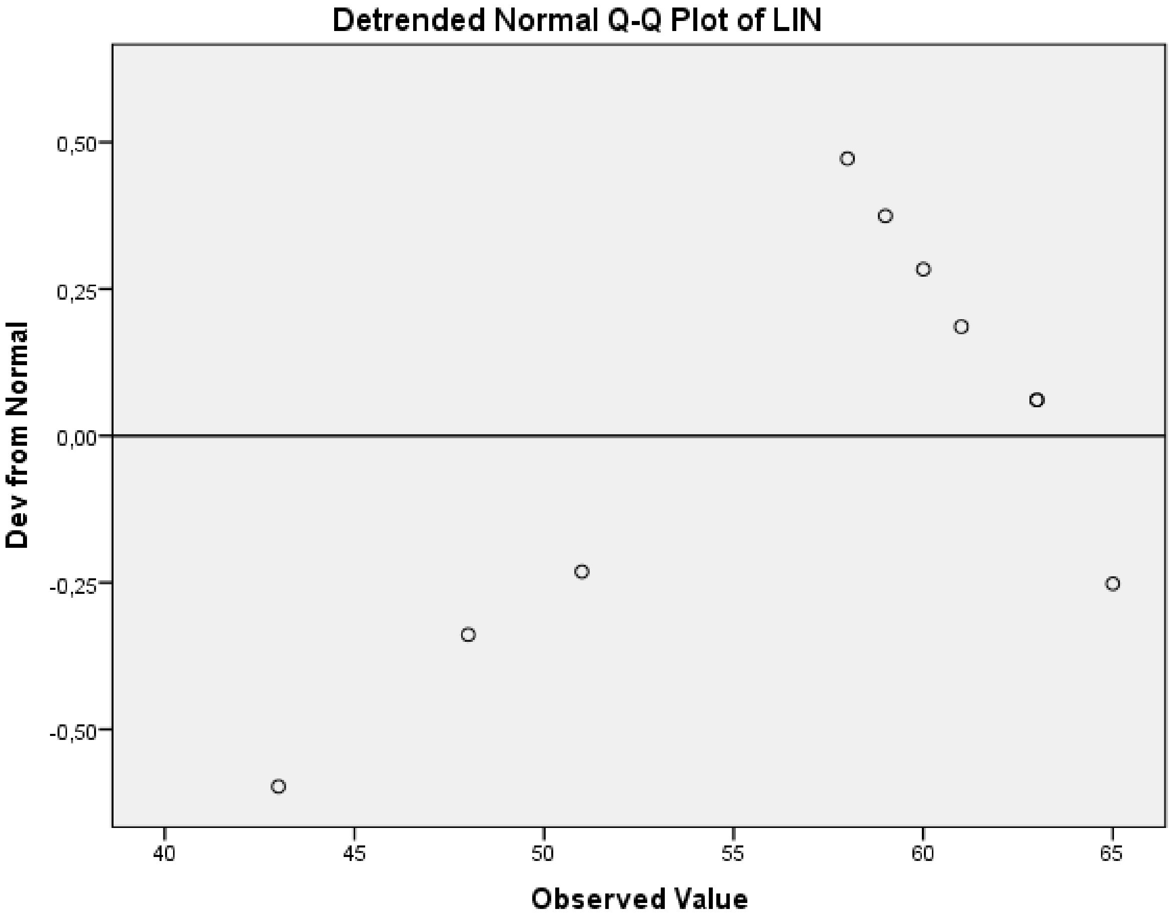

Grup = 8,00

Histograms

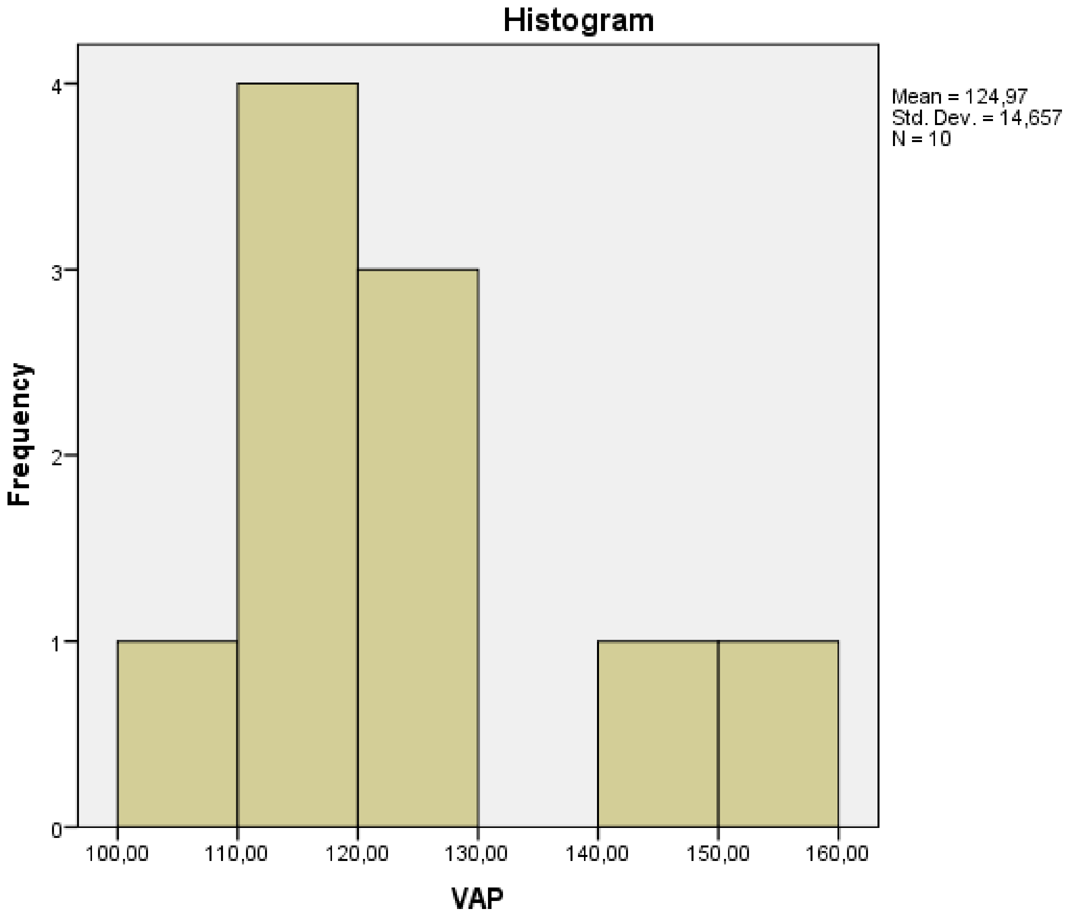
Histogram
Mean = 124,97
Std. Dev. = 14,657
N = 10
Frequency
0
1
2
3
4
100,00
110,00
120,00
130,00
140,00
150,00
160,00
VAP

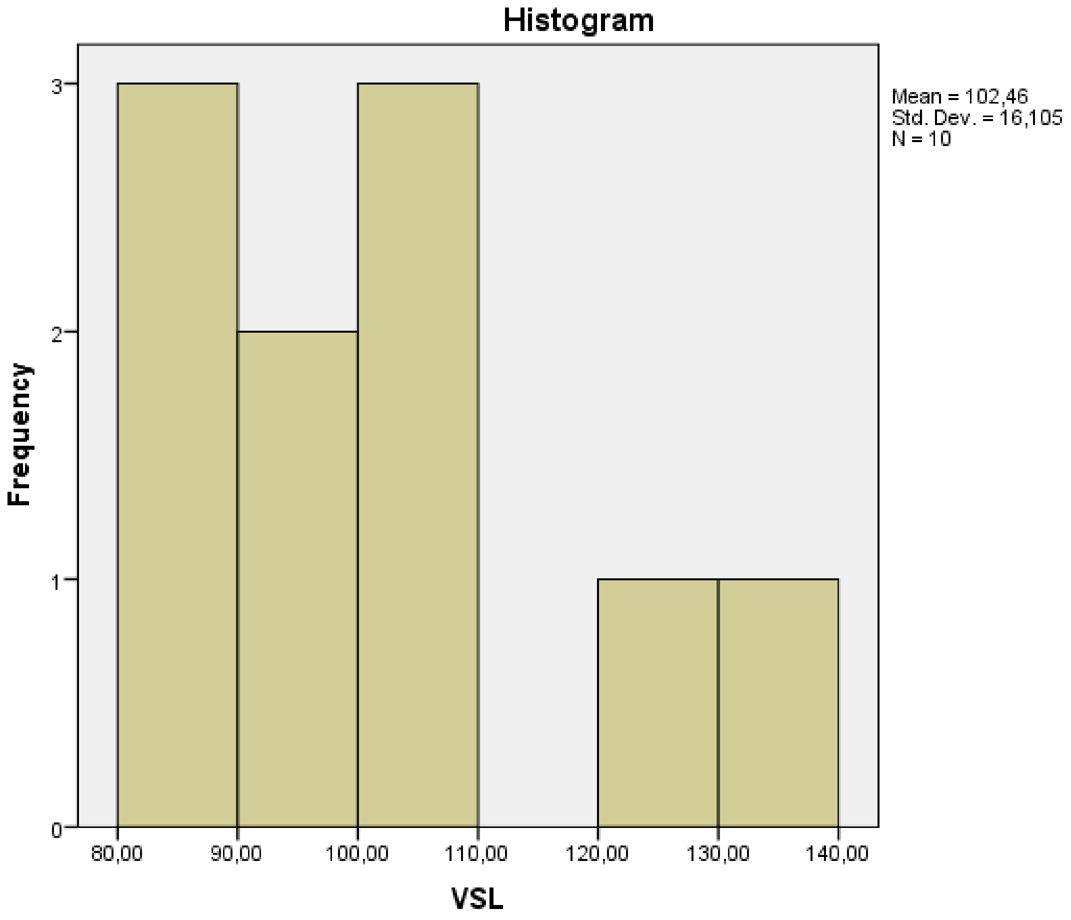
Histogram
Mean = 102,46
Std. Dev. = 16,105
N = 10
Frequency
0
1
2
3
80,00
90,00
100,00
110,00
120,00
130,00
140,00
VSL

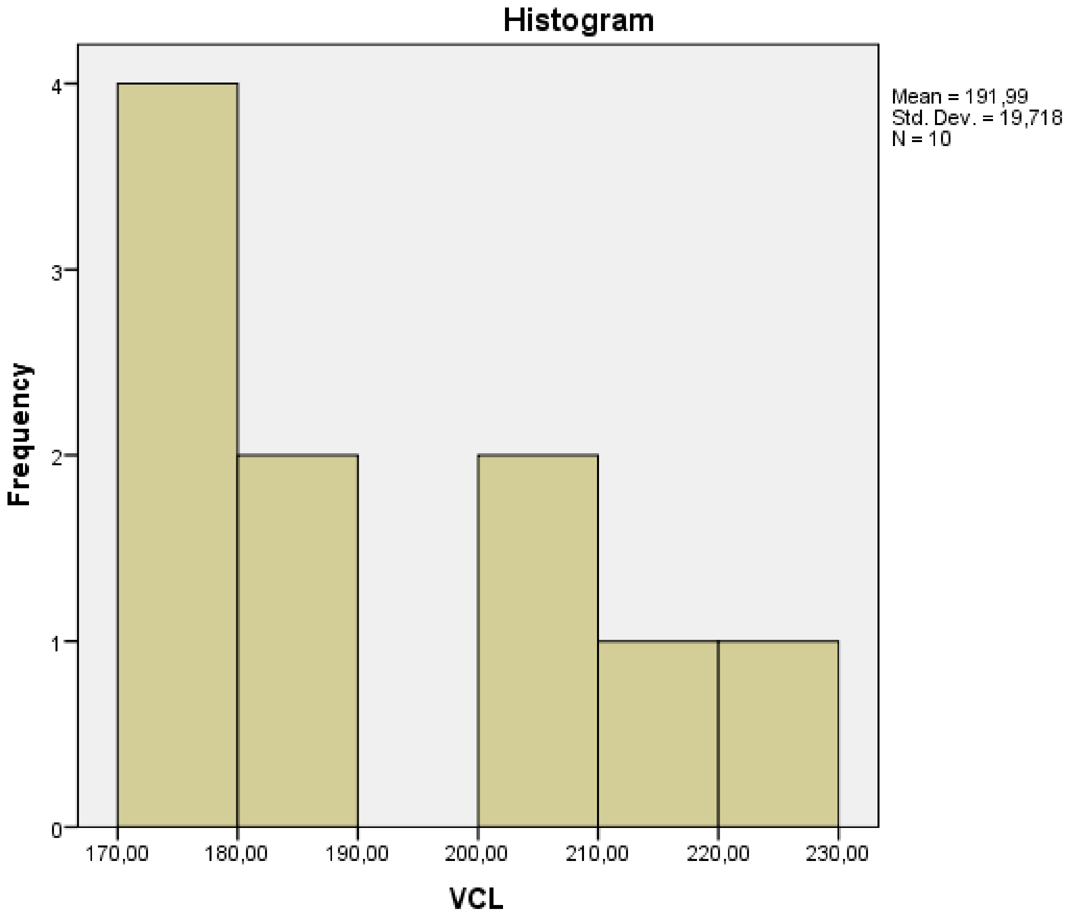
Histogram
Mean = 191,99
Std. Dev. = 19,718
N = 10
Frequency
4
3
2
1
0
170,00
180,00
190,00
200,00
210,00
220,00
230,00
VCL

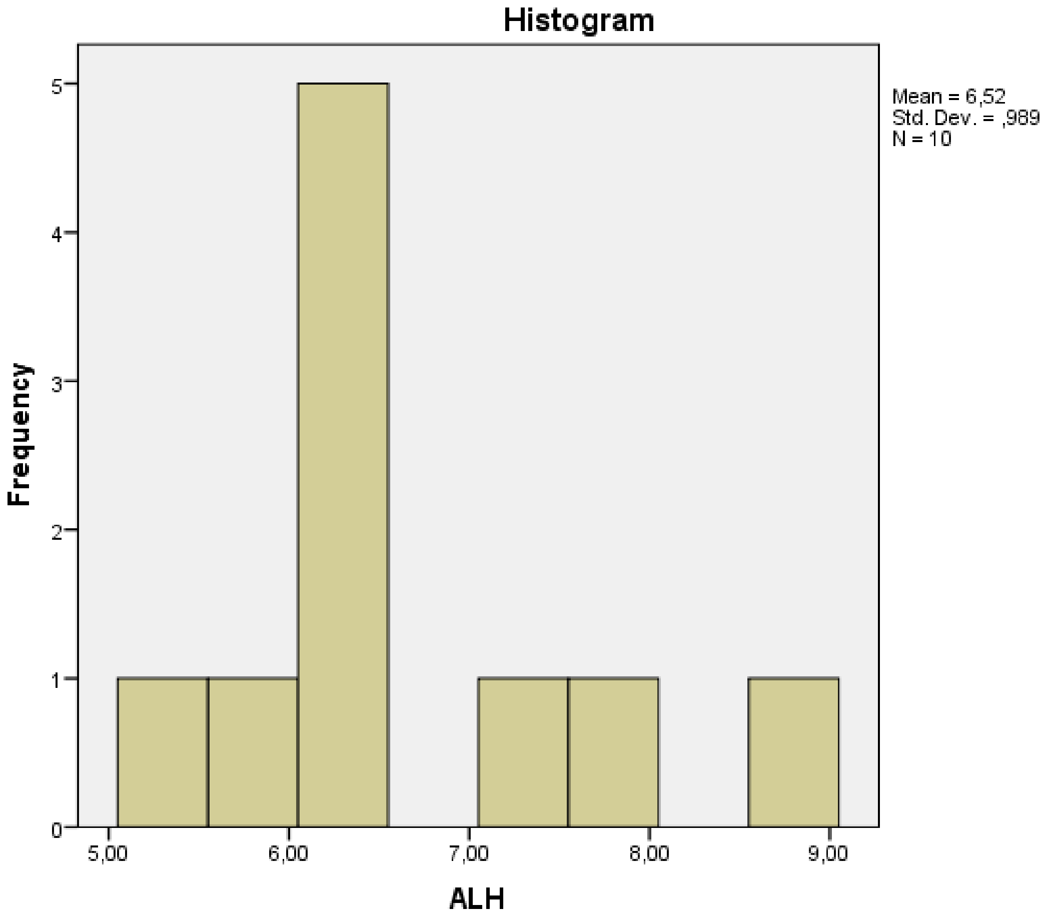

Histogram
Mean = 6,52
Std. Dev. = ,989
N = 10
Frequency
0
1
2
3
4
5
5,00
6,00
7,00
8,00
9,00
ALH

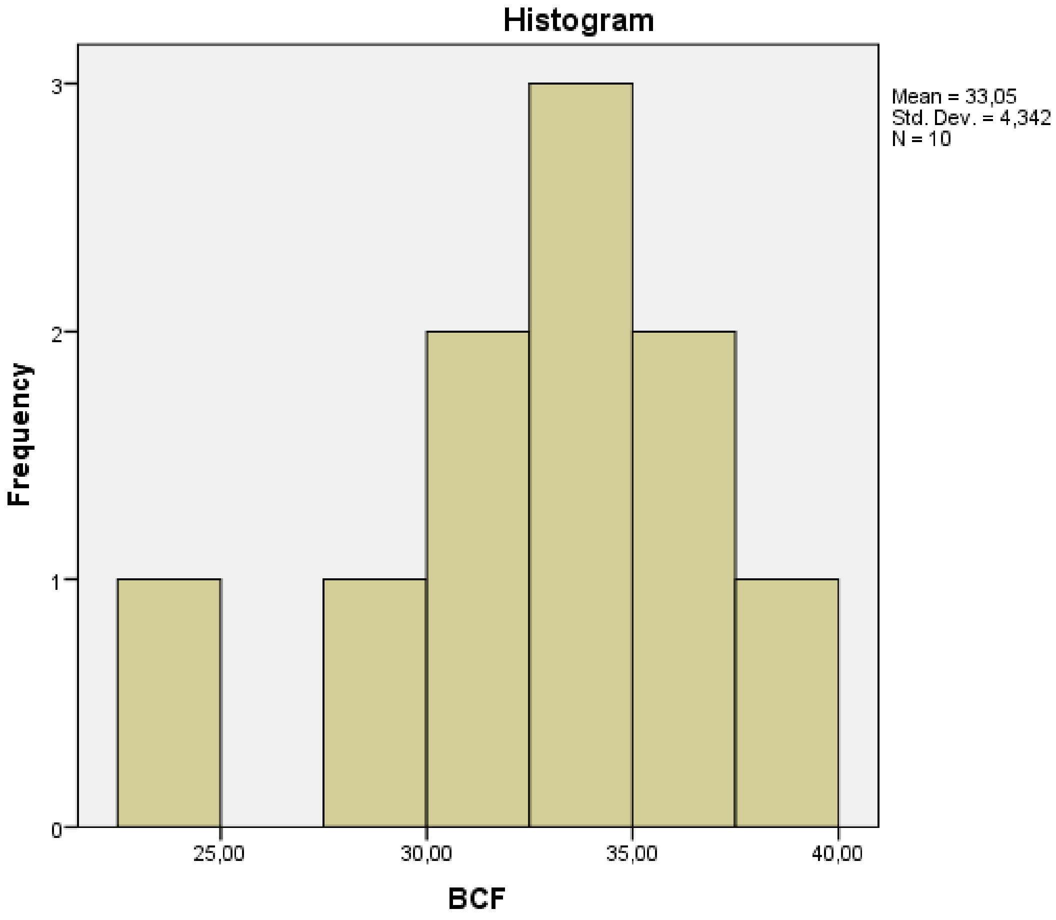
Histogram
Mean = 33,05
Std. Dev. = 4,342
N = 10
Frequency
3
2
1
0
25,00
30,00
35,00
40,00
BCF

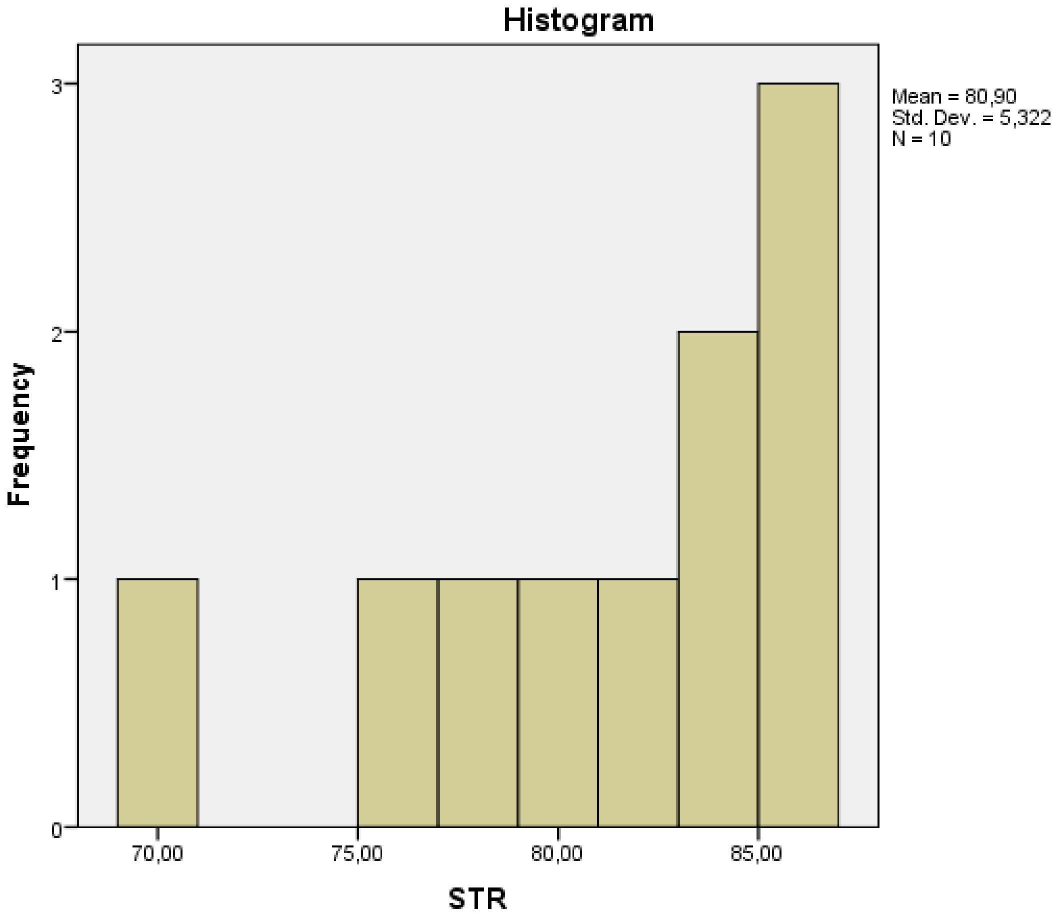
Histogram
Mean = 80,90
Std. Dev. = 5,322
N = 10
Frequency
3
2
1
0
70,00
75,00
80,00
85,00
STR

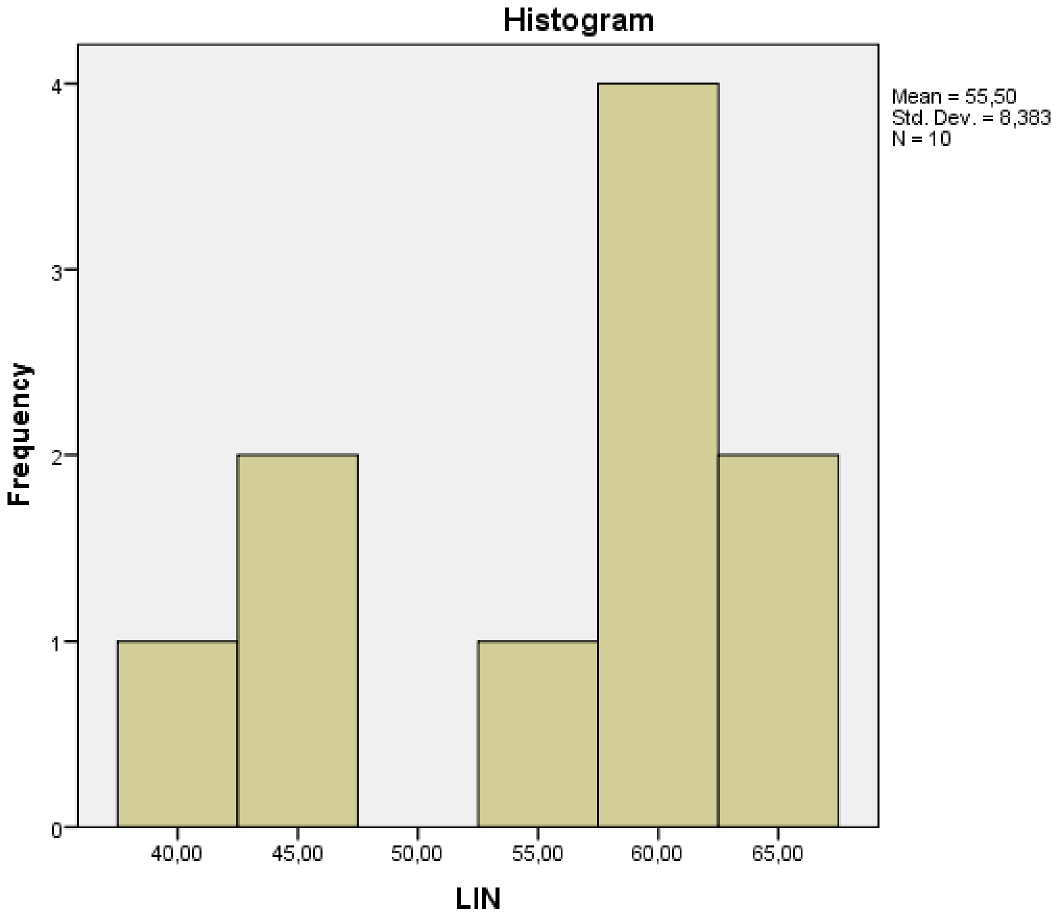

Stem-and-Leaf Plots

```
VAP Stem-and-Leaf Plot for
Grup= 8,00

 Frequency    Stem &  Leaf

     1,00 Extremes    (=<102)
     4,00       11 .  8899
     2,00       12 .  34
     1,00       12 .  6
     2,00 Extremes    (>=143)

 Stem width:  10,00
 Each leaf:       1 case(s)
```

VSL Stem-and-Leaf Plot for
Grup= 8,00

Frequency	Stem &	Leaf
3,00	8 .	169
2,00	9 .	57
3,00	10 .	169
,00	11 .	
1,00	12 .	1
1,00	13 .	3

Stem width: 10,00
Each leaf: 1 case(s)

VCL Stem-and-Leaf Plot for
Grup= 8,00

Frequency	Stem &	Leaf
4,00	17 .	3367
2,00	18 .	03
,00	19 .	
2,00	20 .	39
1,00	21 .	5
1,00	22 .	6

Stem width: 10,00
Each leaf: 1 case(s)

ALH Stem-and-Leaf Plot for
Grup= 8,00

Frequency	Stem &	Leaf
2,00	5 .	37
5,00	6 .	11123
2,00	7 .	26
1,00	8 .	6

Stem width: 1,00
Each leaf: 1 case(s)

BCF Stem-and-Leaf Plot for
Grup= 8,00

```
 Frequency    Stem &  Leaf

     1,00        2 .  3
     1,00        2 .  9
     5,00        3 .  02244
     3,00        3 .  678

 Stem width:  10,00
 Each leaf:       1 case(s)
```

```
STR Stem-and-Leaf Plot for
Grup= 8,00

 Frequency    Stem &  Leaf

     1,00        7 .  0
     3,00        7 .  589
     3,00        8 .  244
     3,00        8 .  566

 Stem width:  10,00
 Each leaf:       1 case(s)
```

```
LIN Stem-and-Leaf Plot for
Grup= 8,00

 Frequency    Stem &  Leaf

     1,00        4 .  0
     2,00        4 .  67
      ,00        5 .
     2,00        5 .  58
     4,00        6 .  0004
     1,00        6 .  5

 Stem width:  10,00
 Each leaf:       1 case(s)
```

Normal Q-Q Plots

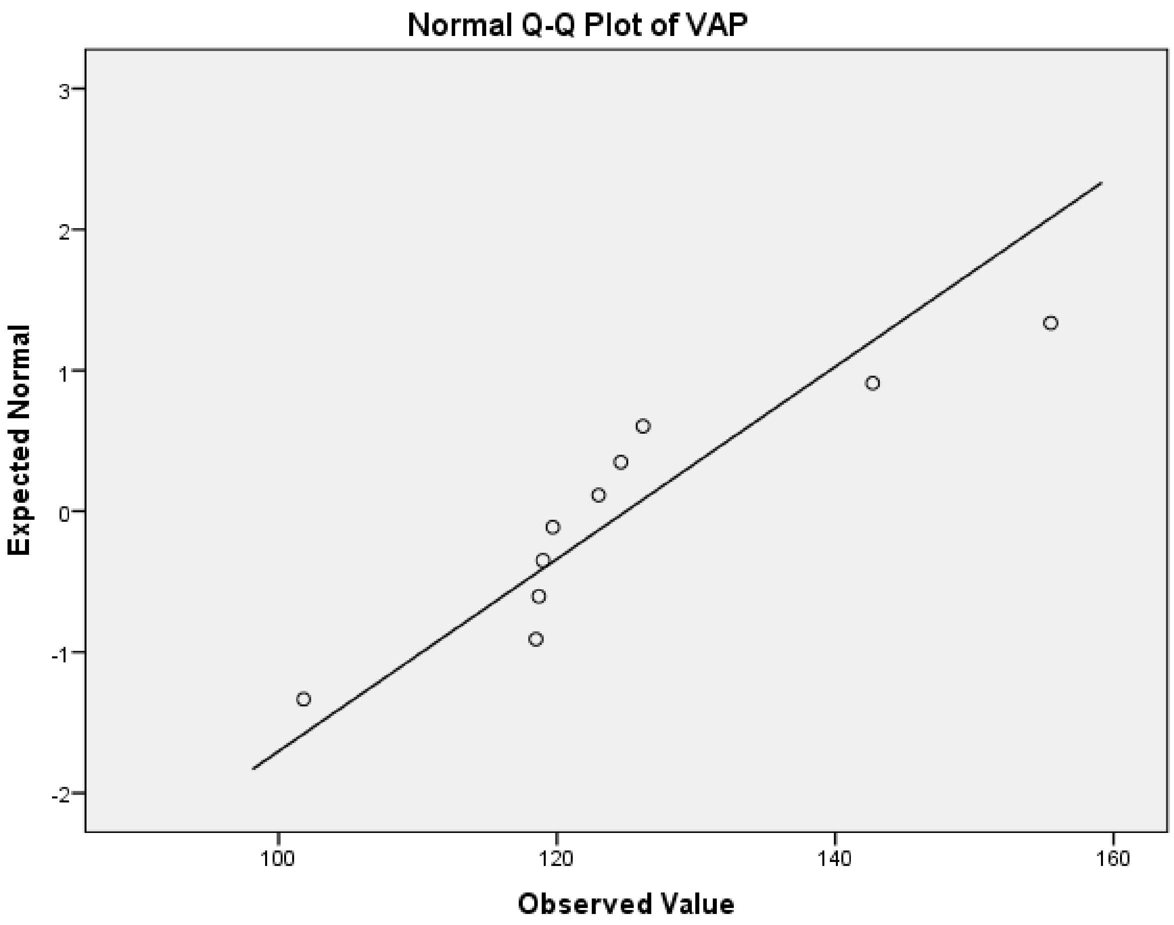
Normal Q-Q Plot of VAP
Expected Normal
Observed Value
3
2
1
0
-1
-2
100
120
140
160

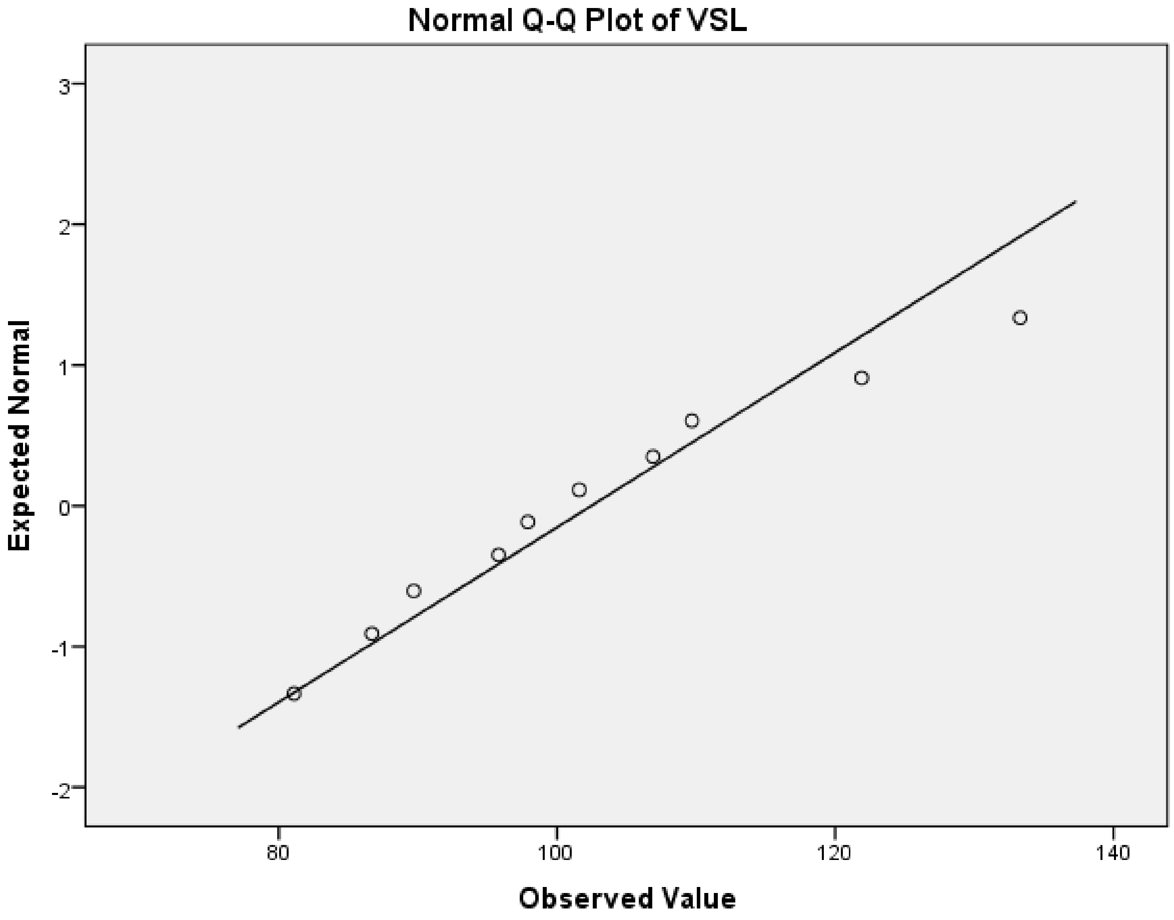
Normal Q-Q Plot of VSL
Expected Normal
Observed Value
3
2
1
0
-1
-2
80
100
120
140

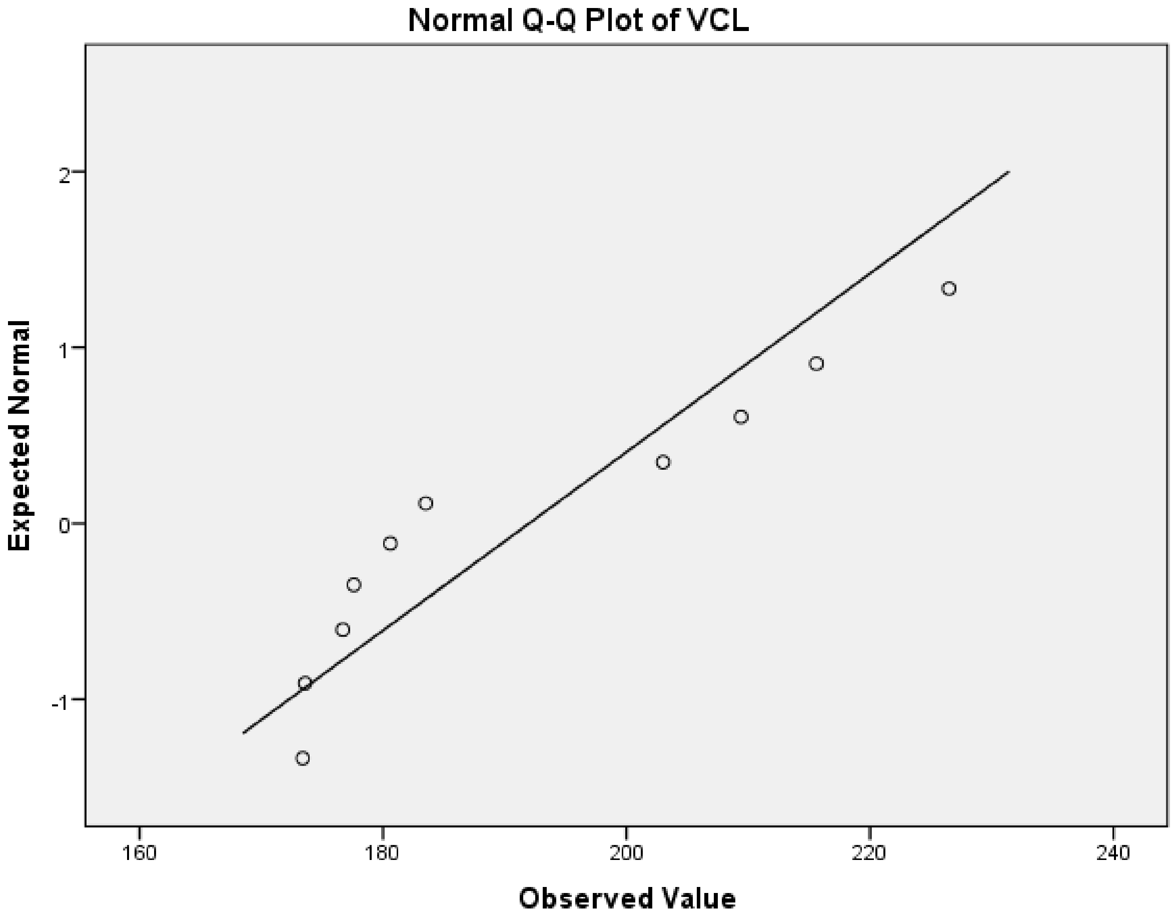
Normal Q-Q Plot of VCL
Expected Normal
2
1
0
-1
160
180
200
220
240
Observed Value

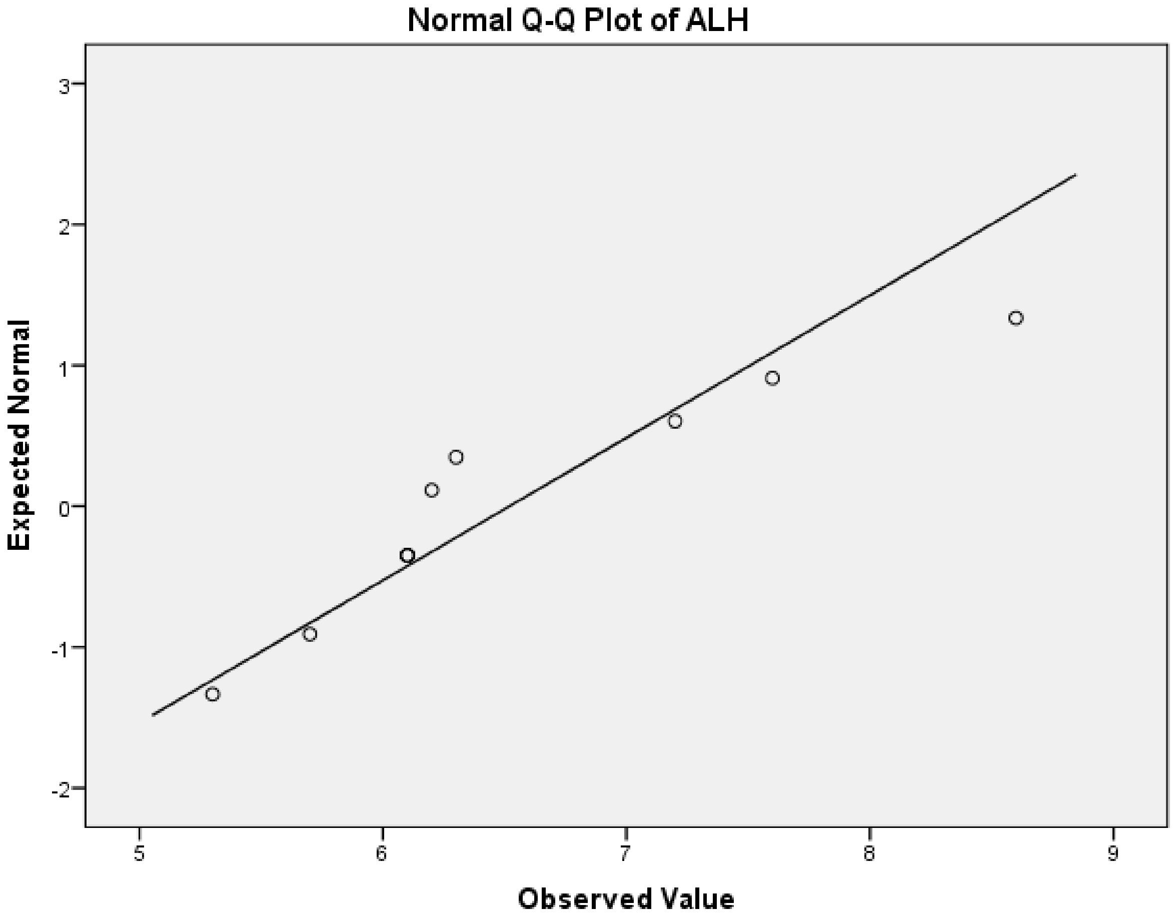

Normal Q-Q Plot of ALH
Expected Normal
Observed Value
3
2
1
0
-1
-2
5
6
7
8
9

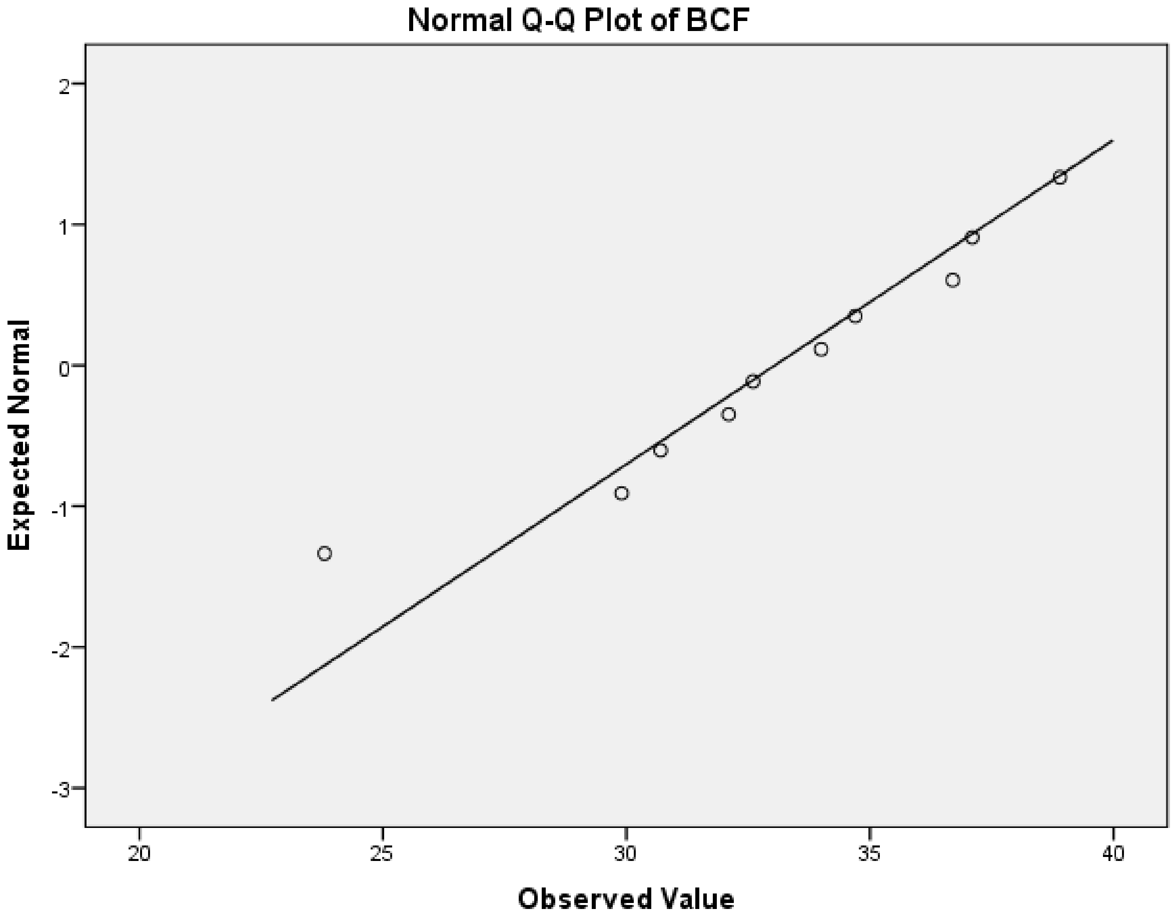

Normal Q-Q Plot of BCF
Expected Normal
Observed Value
2
1
0
-1
-2
-3
20
25
30
35
40

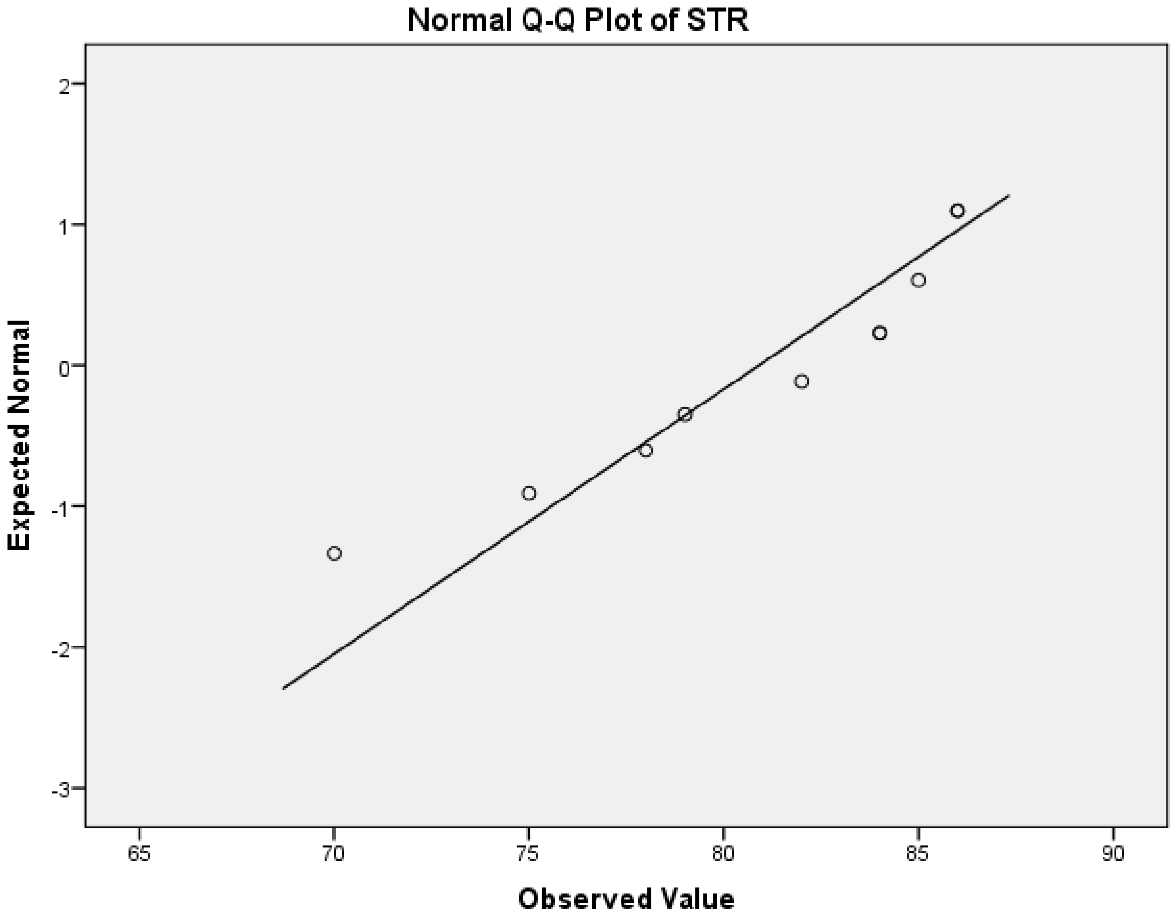
Normal Q-Q Plot of STR
Expected Normal
2
1
0
-1
-2
-3
65
70
75
80
85
90
Observed Value

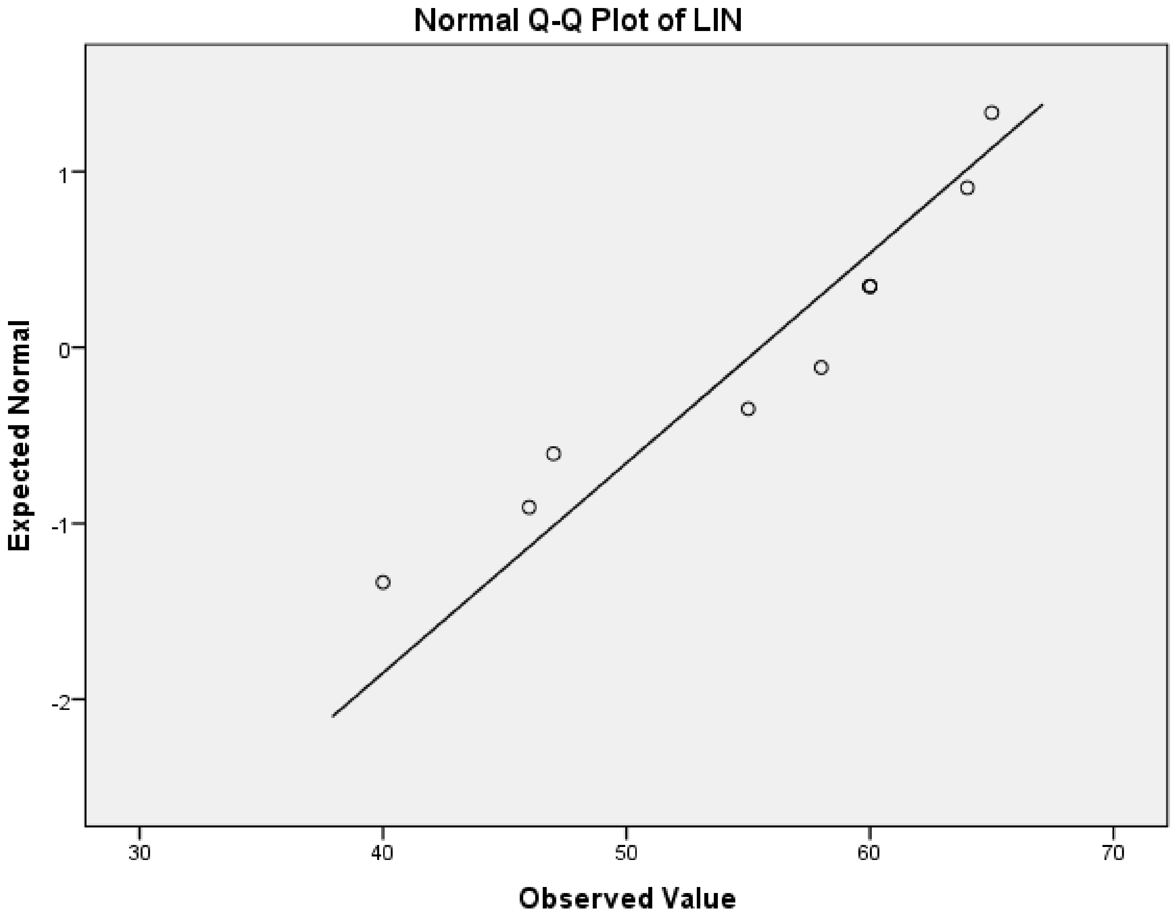

Detrended Normal Q-Q Plots

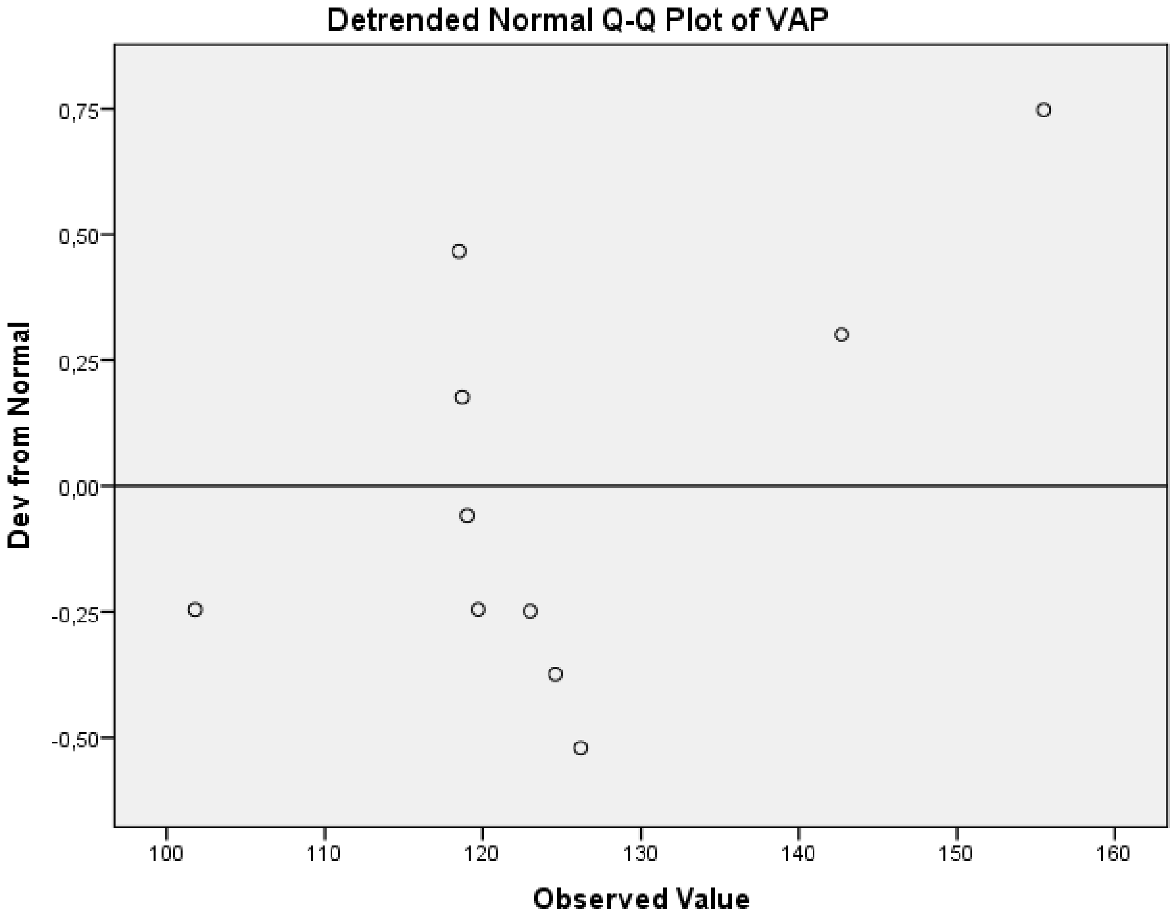

Detrended Normal Q-Q Plot of VAP
Dev from Normal
0,75
0,50
0,25
0,00
-0,25
-0,50
100
110
120
130
140
150
160
Observed Value

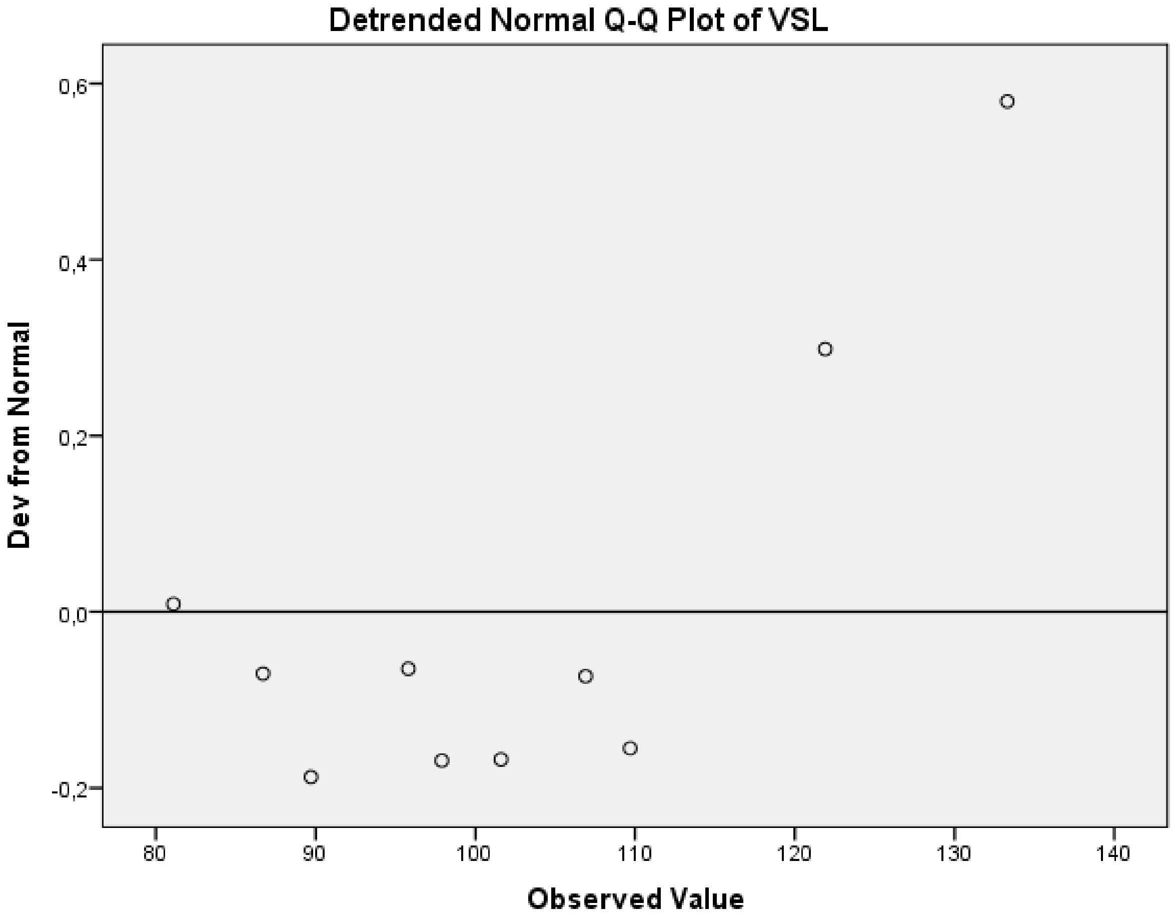

Detrended Normal Q-Q Plot of VSL
Dev from Normal
0,6
0,4
0,2
0,0
-0,2
80
90
100
110
120
130
140
Observed Value

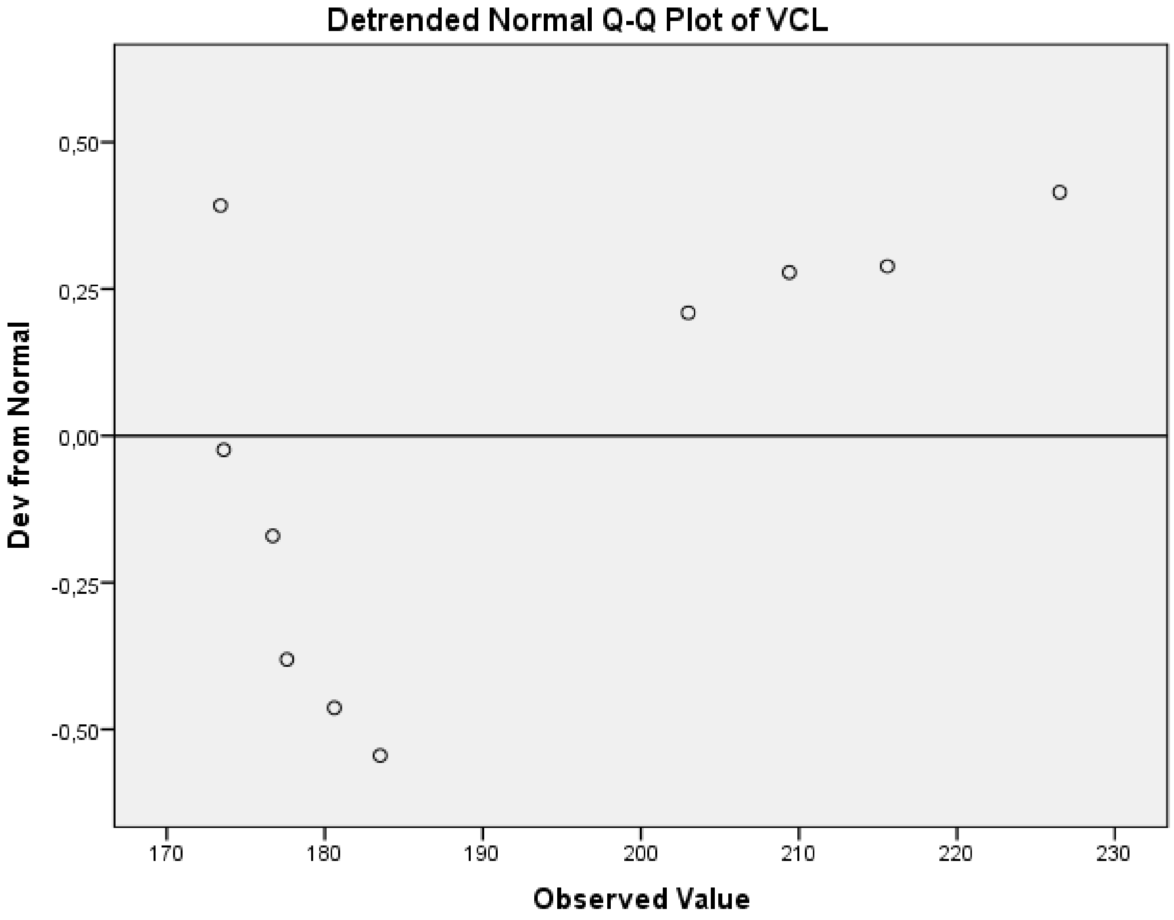
Detrended Normal Q-Q Plot of VCL
Dev from Normal
0,50
0,25
0,00
-0,25
-0,50
170
180
190
200
210
220
230
Observed Value

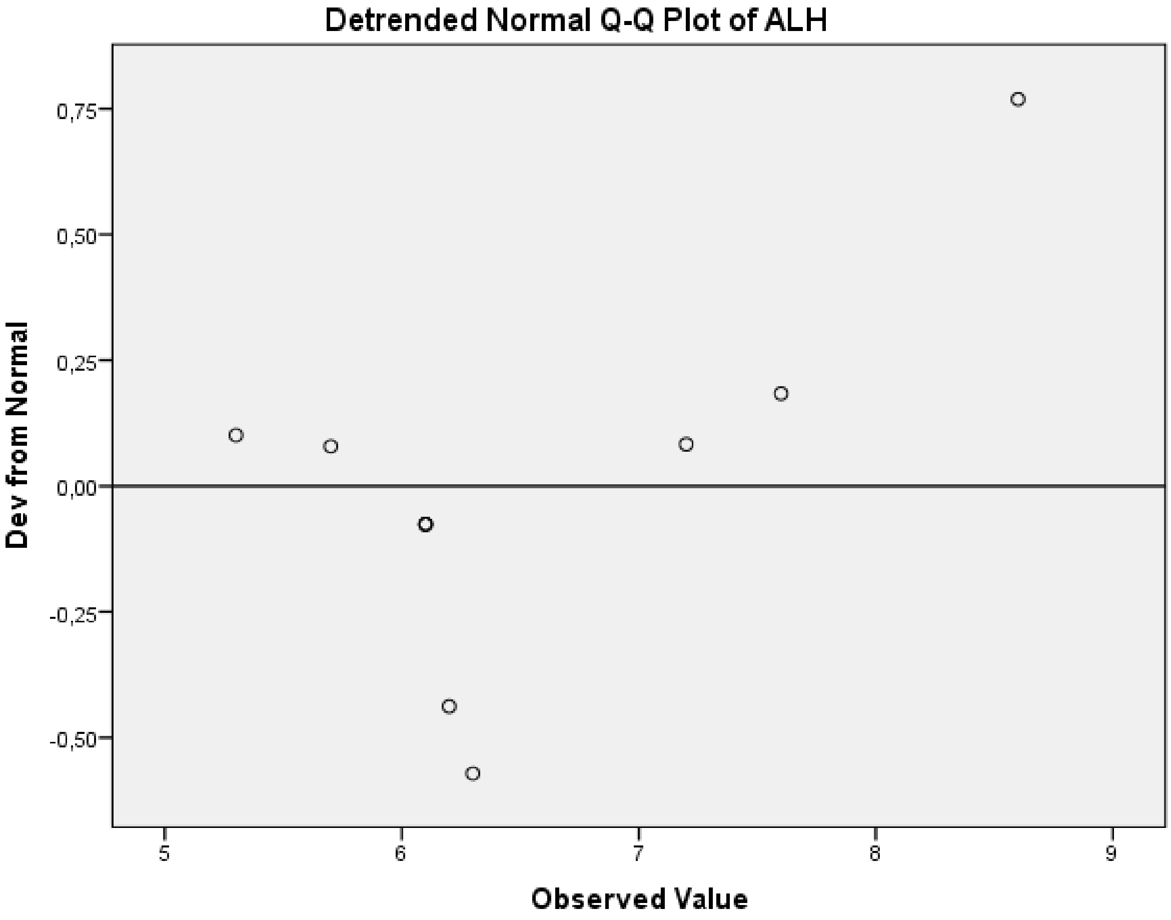
Detrended Normal Q-Q Plot of ALH
Dev from Normal
0,75
0,50
0,25
0,00
-0,25
-0,50
5
6
7
8
9
Observed Value

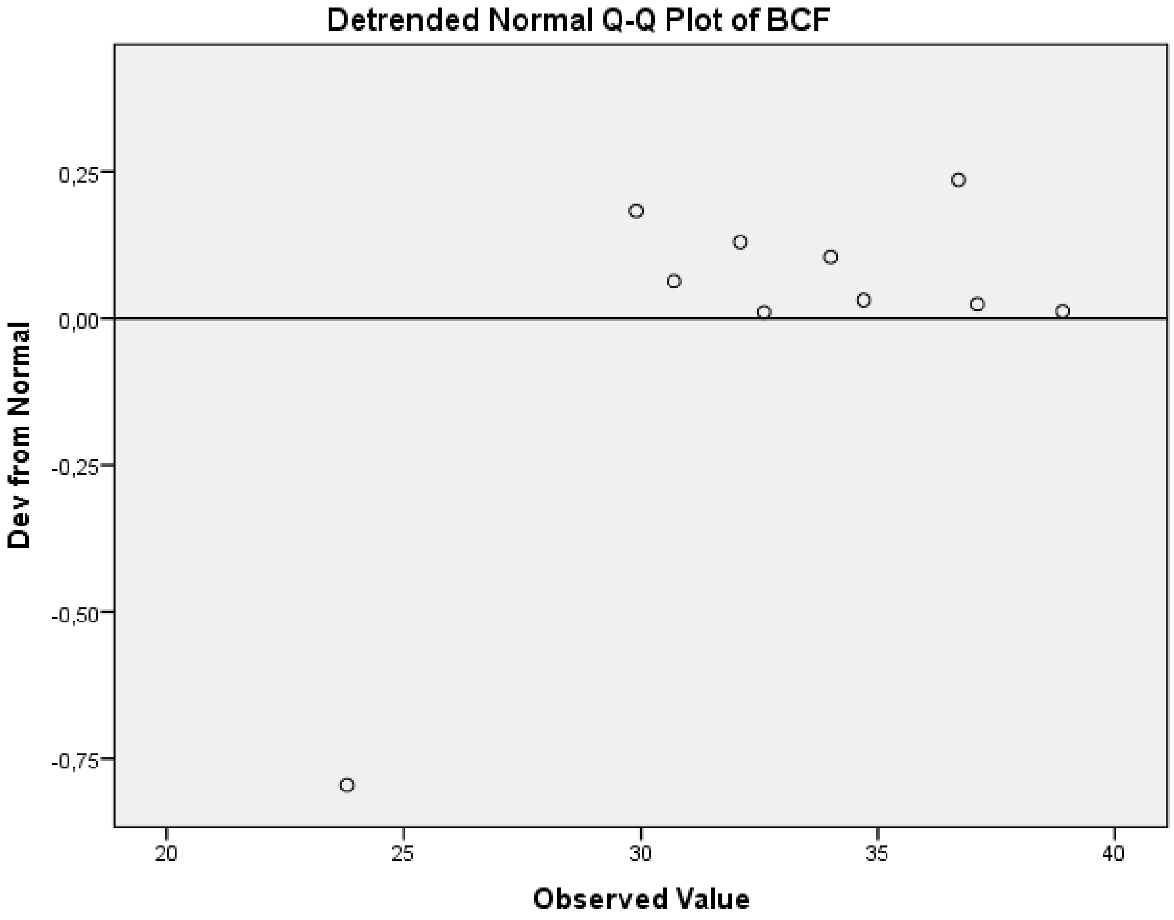

Detrended Normal Q-Q Plot of BCF
Dev from Normal
0,25
0,00
-0,25
-0,50
-0,75
20
25
30
35
40
Observed Value

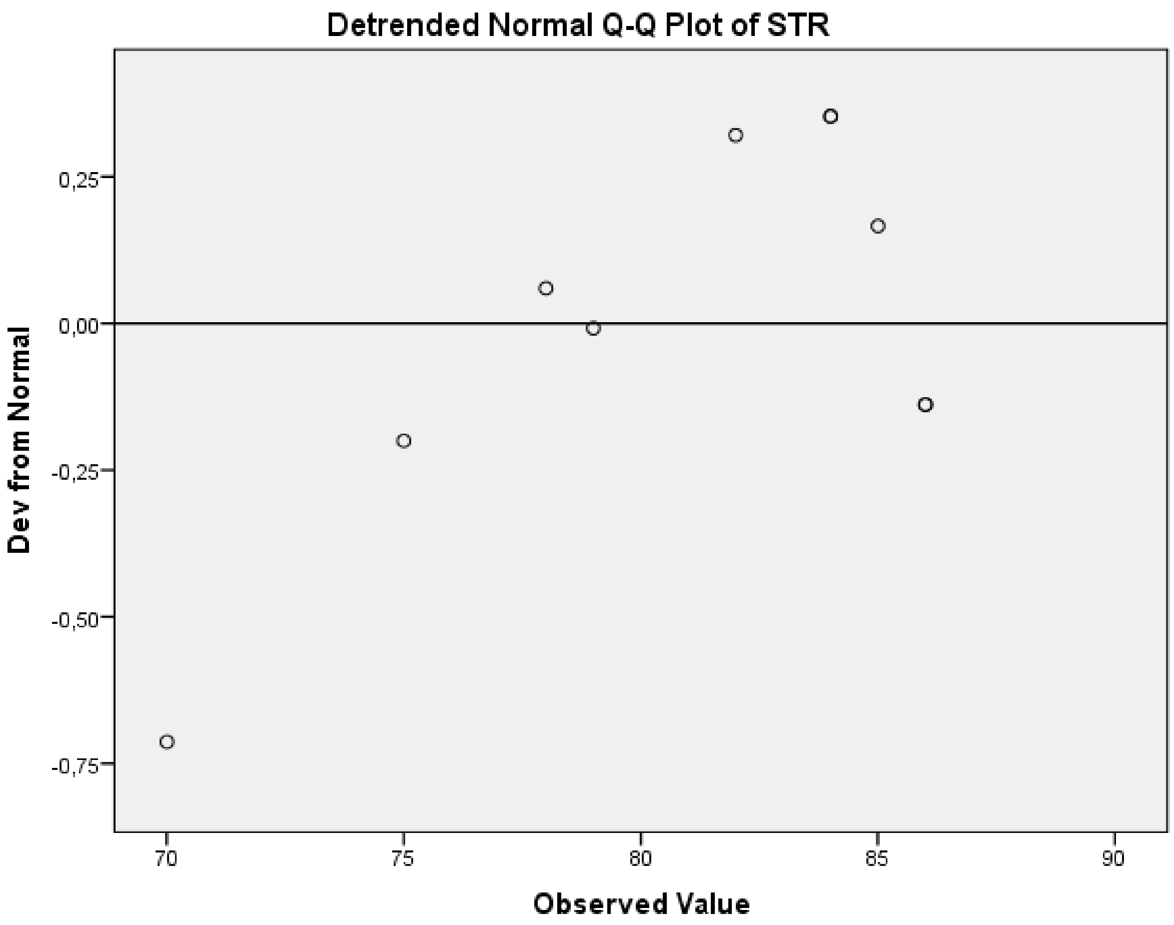

Detrended Normal Q-Q Plot of STR
Dev from Normal
0,25
0,00
-0,25
-0,50
-0,75
70
75
80
85
90
Observed Value

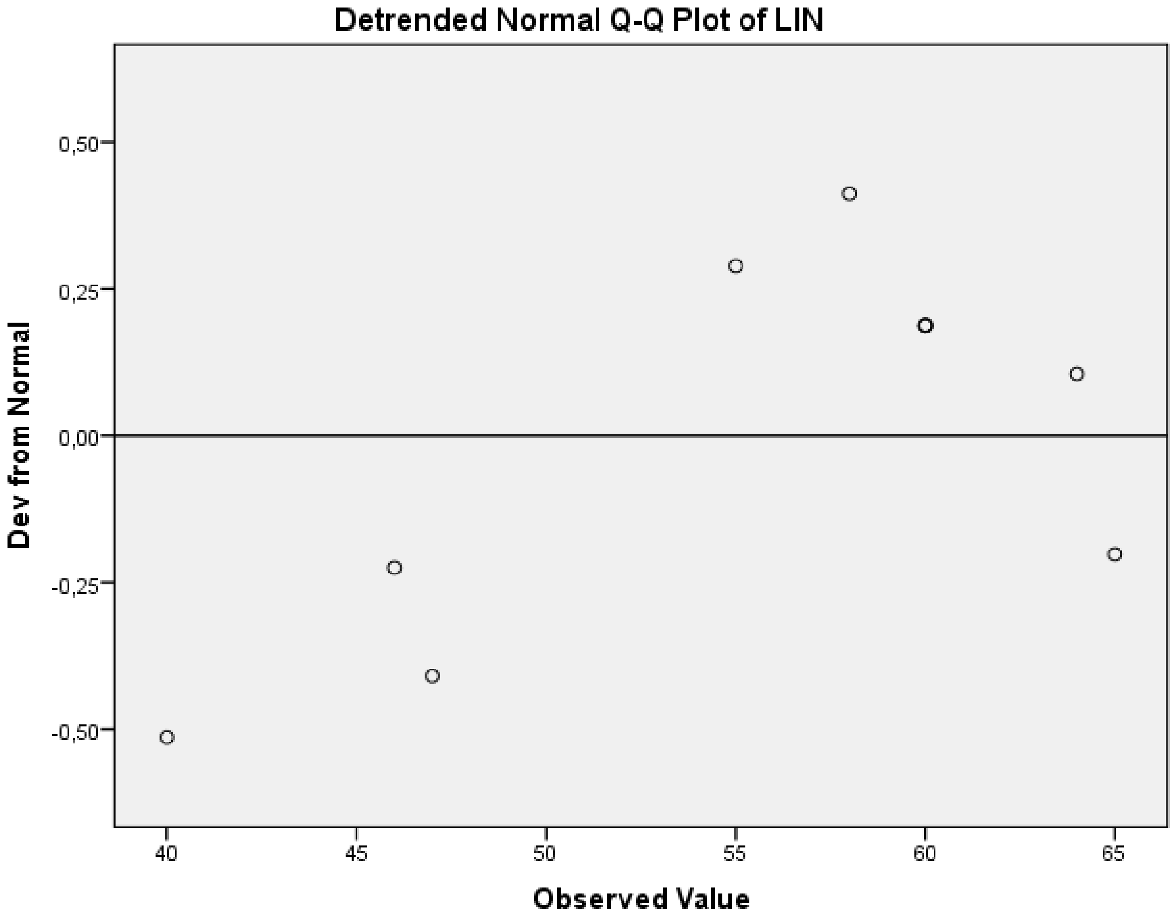

Grup = 9,00

Histograms

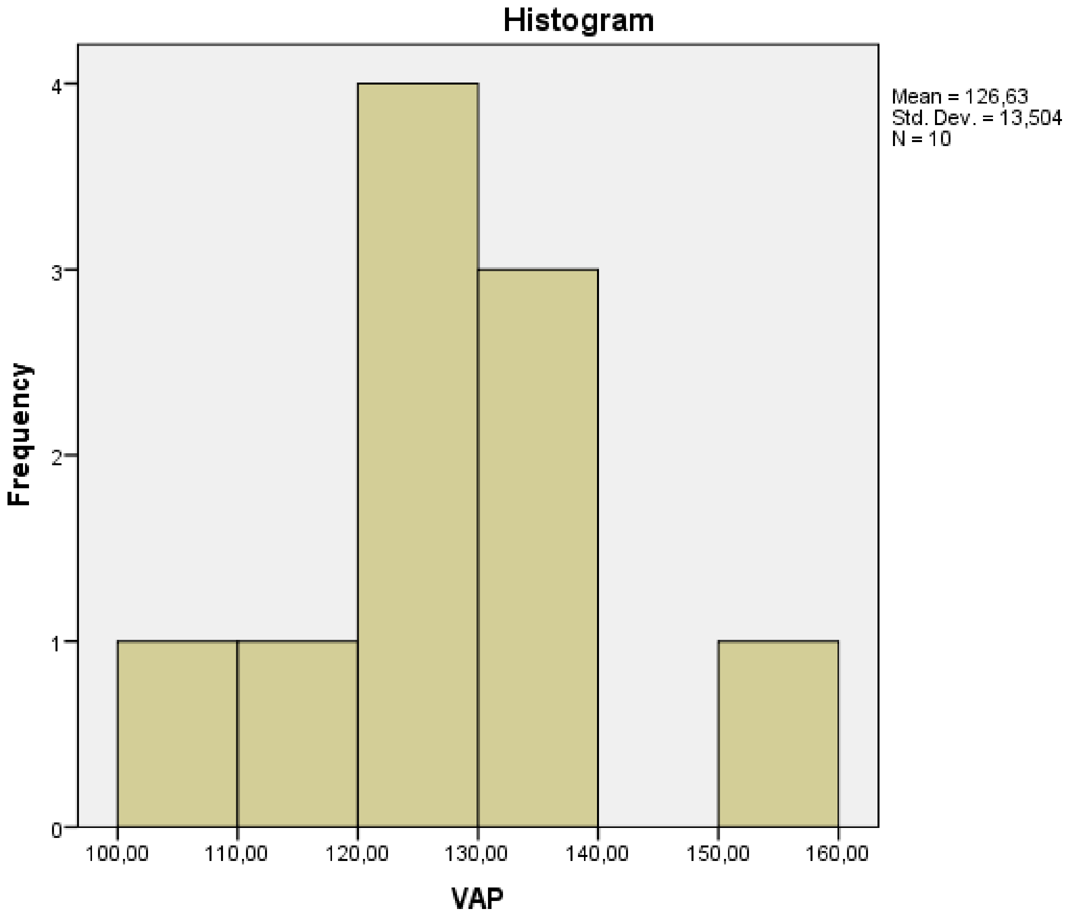

Histogram
Mean = 126,63
Std. Dev. = 13,504
N = 10
Frequency
4
3
2
1
0
100,00
110,00
120,00
130,00
140,00
150,00
160,00
VAP

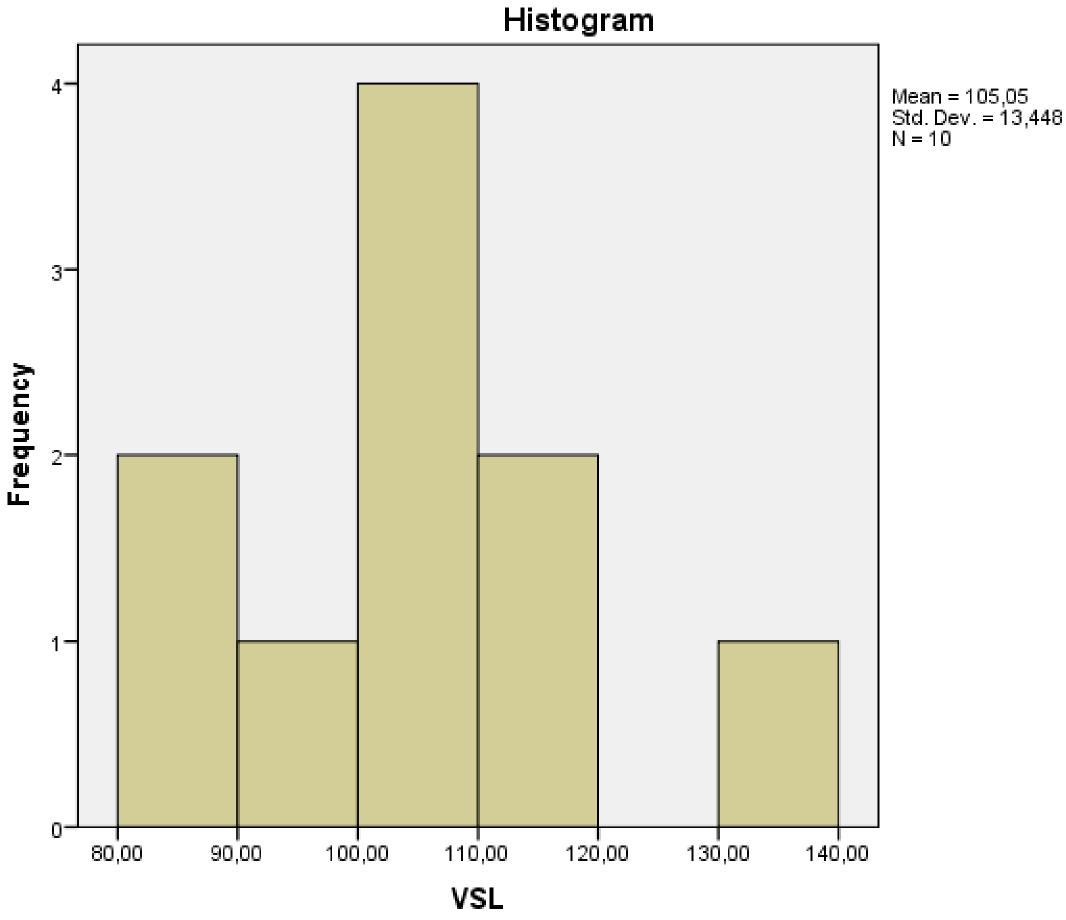
Histogram
Mean = 105,05
Std. Dev. = 13,448
N = 10
Frequency
0
1
2
3
4
80,00
90,00
100,00
110,00
120,00
130,00
140,00
VSL

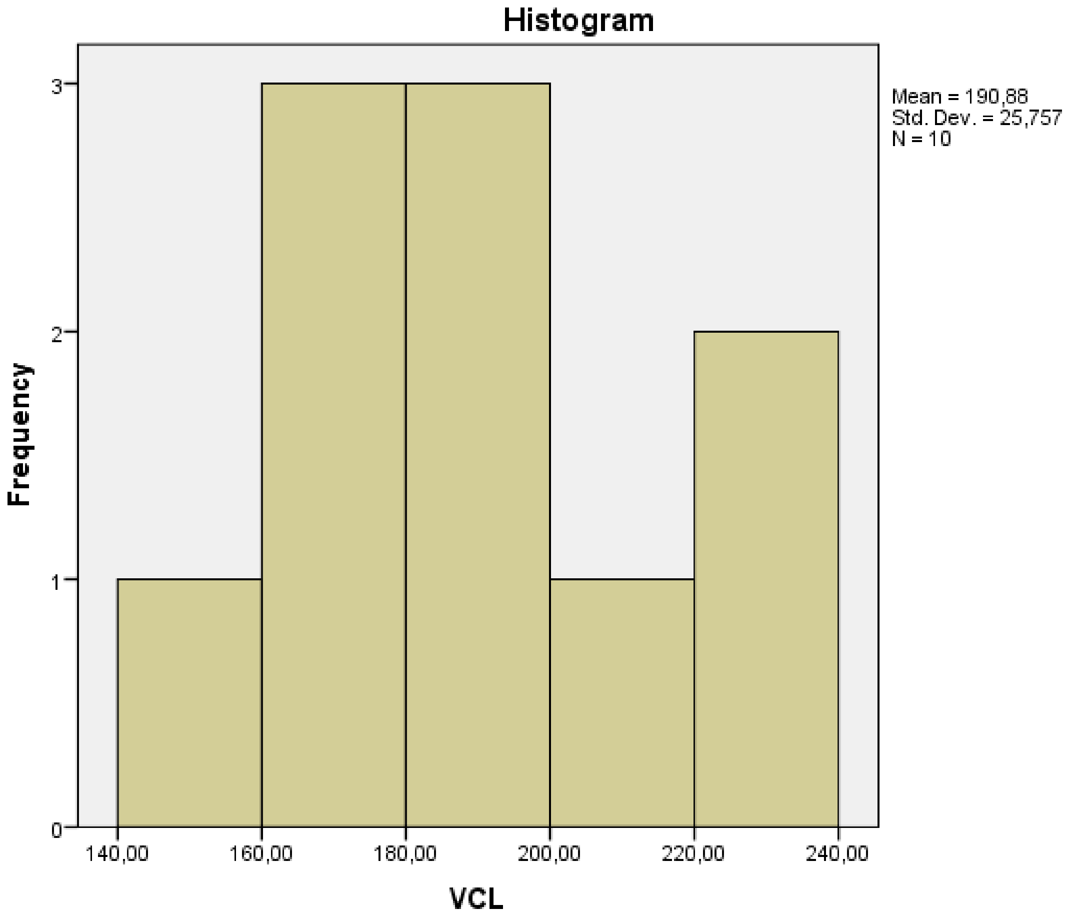

Histogram
Mean = 190,88
Std. Dev. = 25,757
N = 10
Frequency
0
1
2
3
140,00
160,00
180,00
200,00
220,00
240,00
VCL

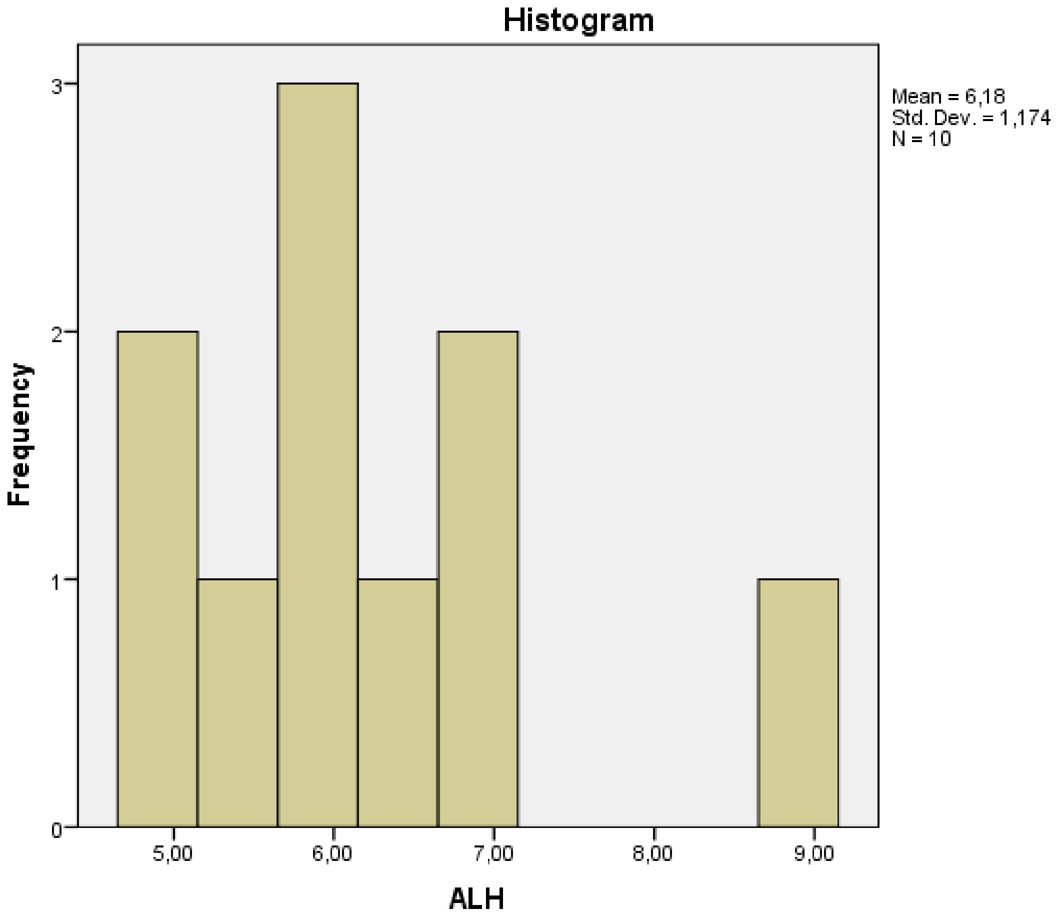
Histogram
Mean = 6,18
Std. Dev. = 1,174
N = 10
Frequency
0
1
2
3
5,00
6,00
7,00
8,00
9,00
ALH

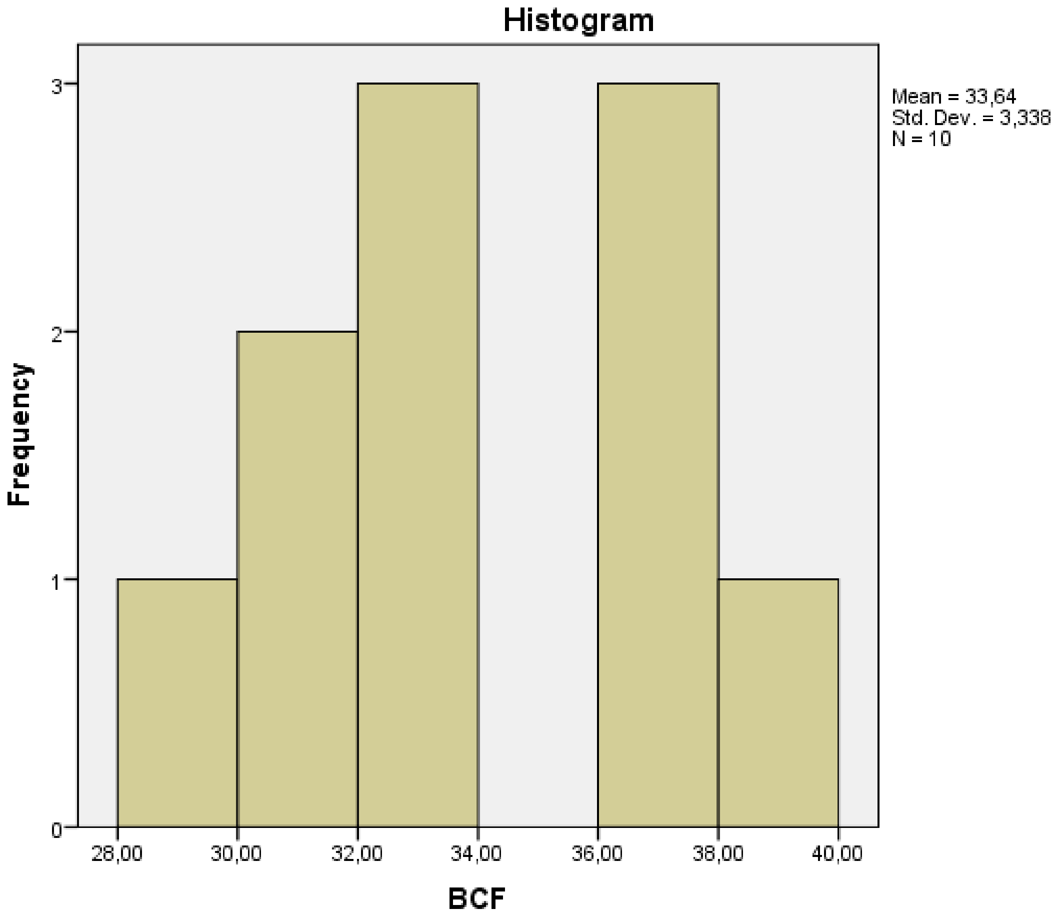
Histogram
Mean = 33,64
Std. Dev. = 3,338
N = 10
Frequency
0
1
2
3
28,00
30,00
32,00
34,00
36,00
38,00
40,00
BCF

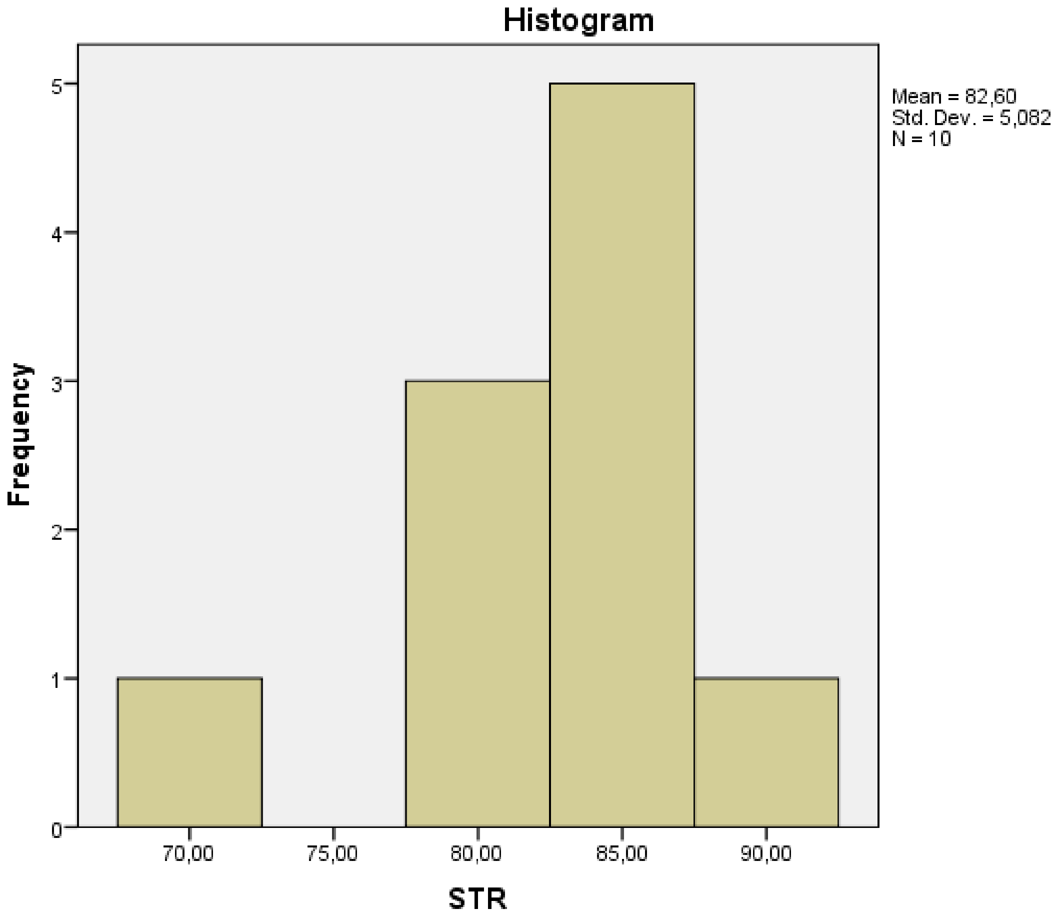
Histogram
Mean = 82,60
Std. Dev. = 5,082
N = 10
Frequency
0
1
2
3
4
5
70,00
75,00
80,00
85,00
90,00
STR

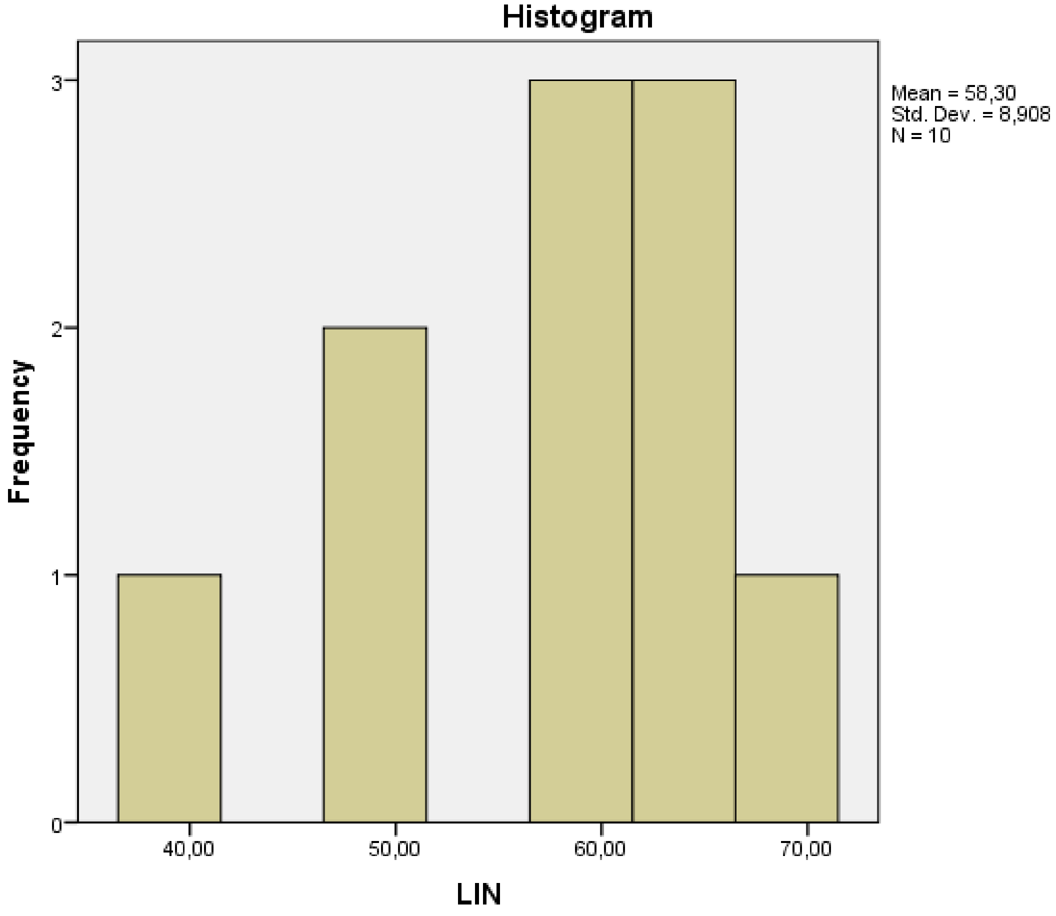

Stem-and-Leaf Plots

```
VAP Stem-and-Leaf Plot for
Grup= 9,00

 Frequency    Stem &  Leaf

     1,00 Extremes    (=<103)
     1,00       11 .  4
      ,00       11 .
     3,00       12 .  134
     1,00       12 .  8
     3,00       13 .  123
     1,00 Extremes    (>=155)

 Stem width:  10,00
 Each leaf:       1 case(s)
```

```
VSL Stem-and-Leaf Plot for
Grup= 9,00

 Frequency    Stem &  Leaf

     2,00        8 .  46
     1,00        9 .  9
     4,00       10 .  0677
     2,00       11 .  34
      ,00       12 .
     1,00       13 .  0

 Stem width:  10,00
 Each leaf:       1 case(s)
```

```
VCL Stem-and-Leaf Plot for
Grup= 9,00

 Frequency    Stem &  Leaf

     7,00        1 .  5777889
     3,00        2 .  123

 Stem width:  100,0
 Each leaf:       1 case(s)
```

```
ALH Stem-and-Leaf Plot for
Grup= 9,00

 Frequency    Stem &  Leaf

     1,00        4 .  9
     5,00        5 .  14789
     2,00        6 .  37
     1,00        7 .  1
     1,00 Extremes    (>=8,9)

 Stem width:   1,00
 Each leaf:       1 case(s)
```

```
BCF Stem-and-Leaf Plot for
Grup= 9,00

 Frequency    Stem &  Leaf
```

```
     1,00        2 .  8
     5,00        3 .  01223
     4,00        3 .  6678

 Stem width:  10,00
 Each leaf:       1 case(s)
```

```
STR Stem-and-Leaf Plot for
Grup= 9,00

 Frequency    Stem &  Leaf

     1,00 Extremes    (=<70)
     1,00        8 .  0
     3,00        8 .  223
     3,00        8 .  455
     1,00        8 .  6
     1,00        8 .  9

 Stem width:  10,00
 Each leaf:       1 case(s)
```

```
LIN Stem-and-Leaf Plot for
Grup= 9,00

 Frequency    Stem &  Leaf

     1,00        3 .  9
      ,00        4 .
     2,00        5 .  11
     6,00        6 .  011235
     1,00        7 .  0

 Stem width:  10,00
 Each leaf:       1 case(s)
```

Normal Q-Q Plots

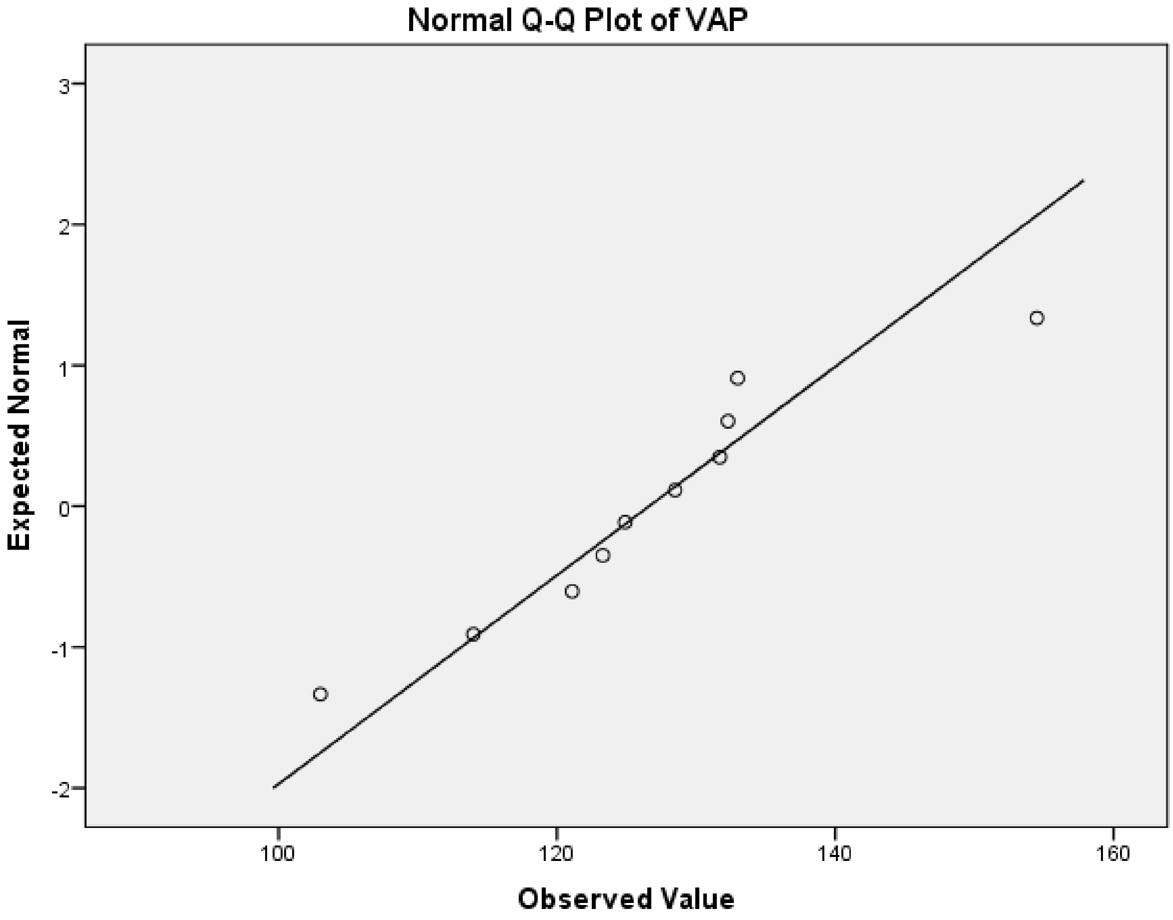

Normal Q-Q Plot of VAP
Expected Normal
3
2
1
0
-1
-2
100
120
140
160
Observed Value

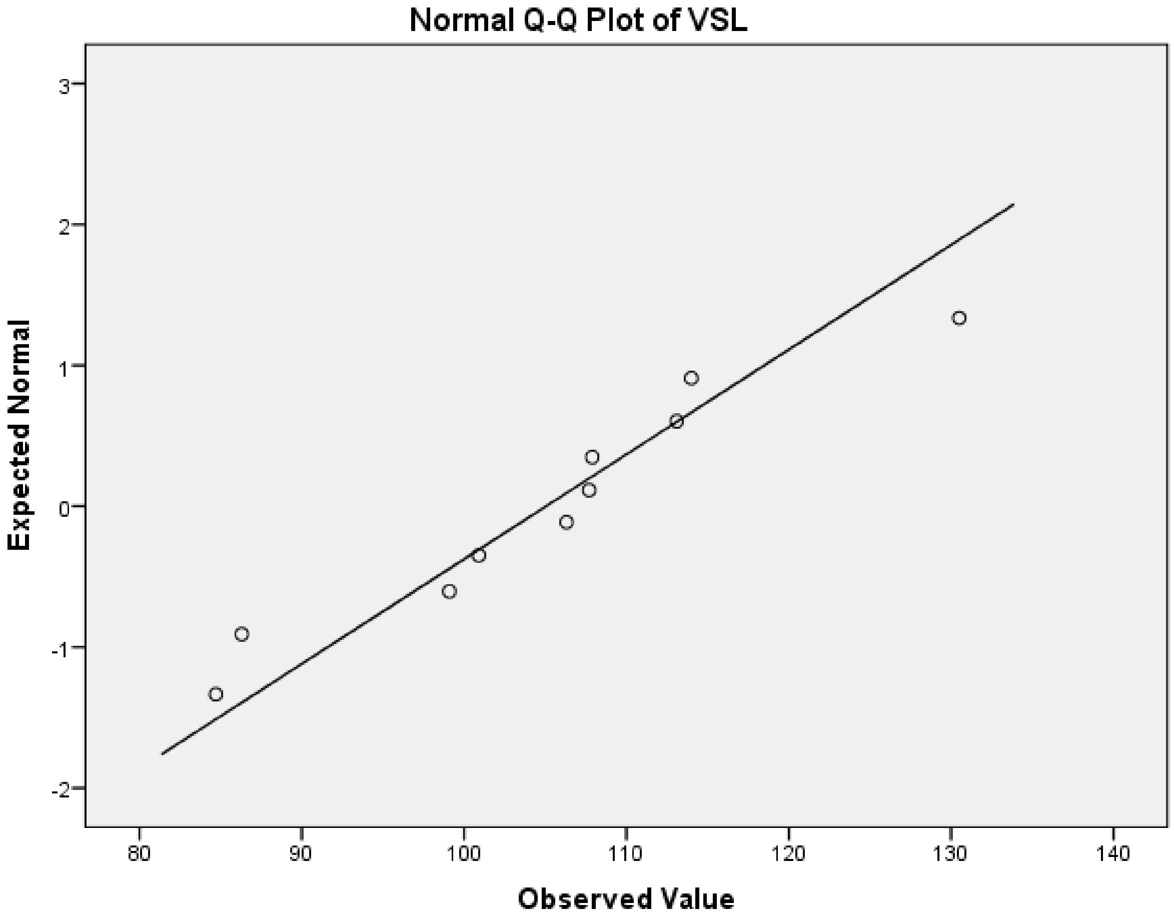

Normal Q-Q Plot of VSL
Expected Normal
Observed Value
3
2
1
0
-1
-2
80
90
100
110
120
130
140

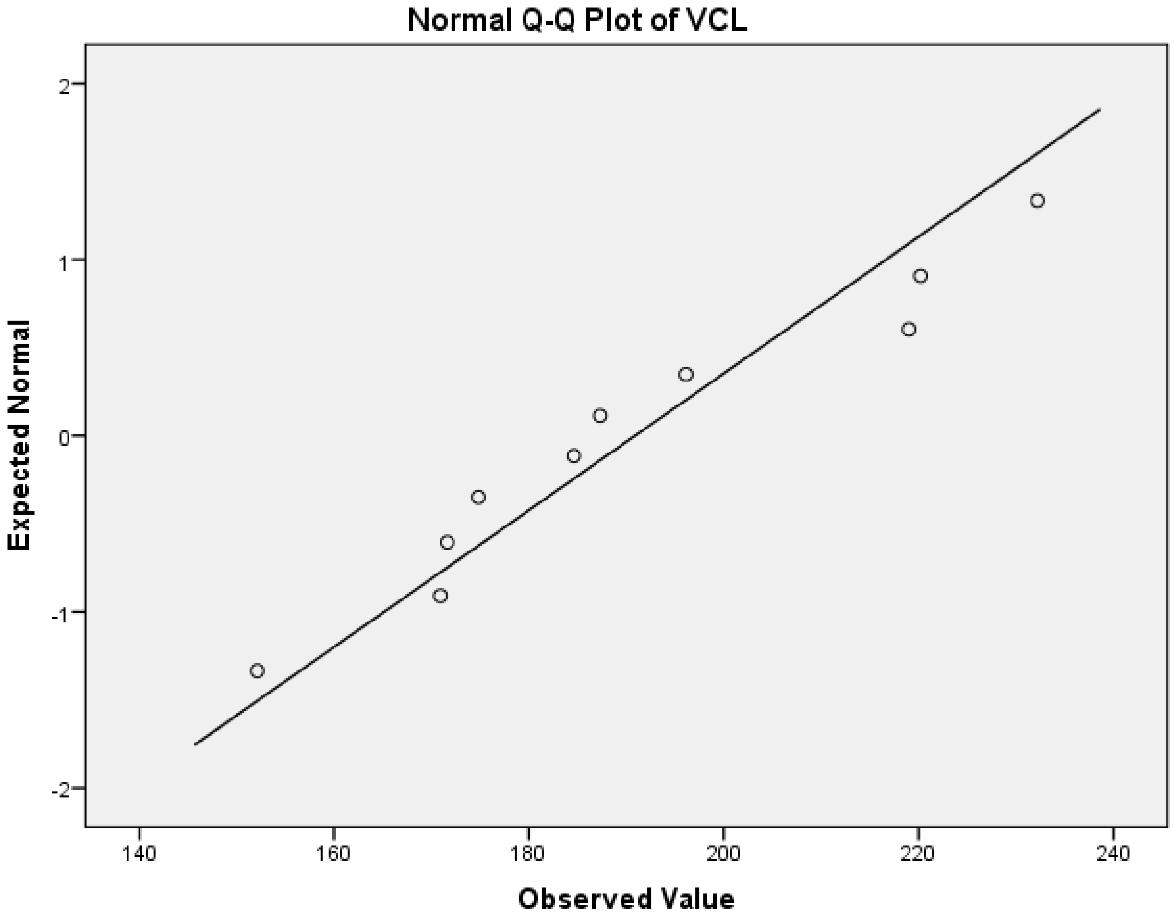
Normal Q-Q Plot of VCL
Expected Normal
2
1
0
-1
-2
140
160
180
200
220
240
Observed Value

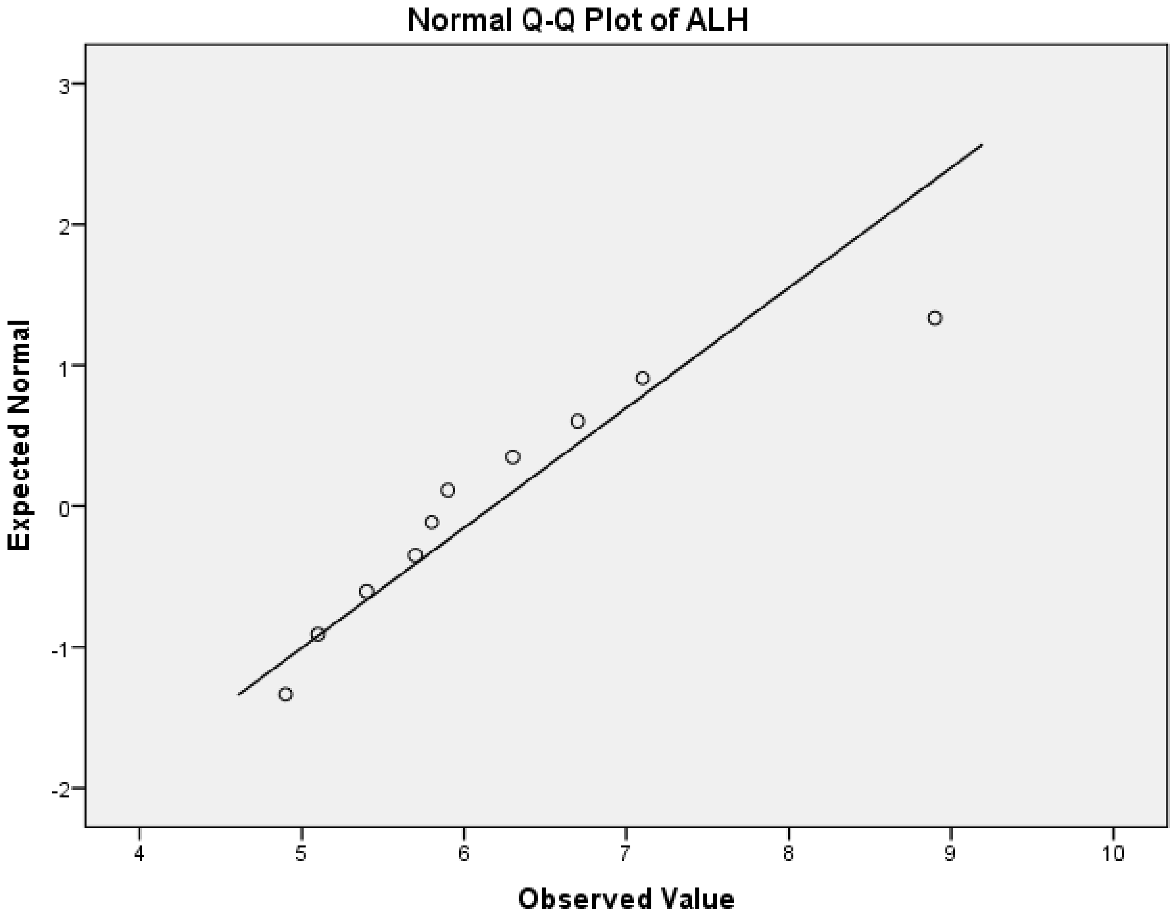

Normal Q-Q Plot of ALH
Expected Normal
3
2
1
0
-1
-2
4
5
6
7
8
9
10
Observed Value

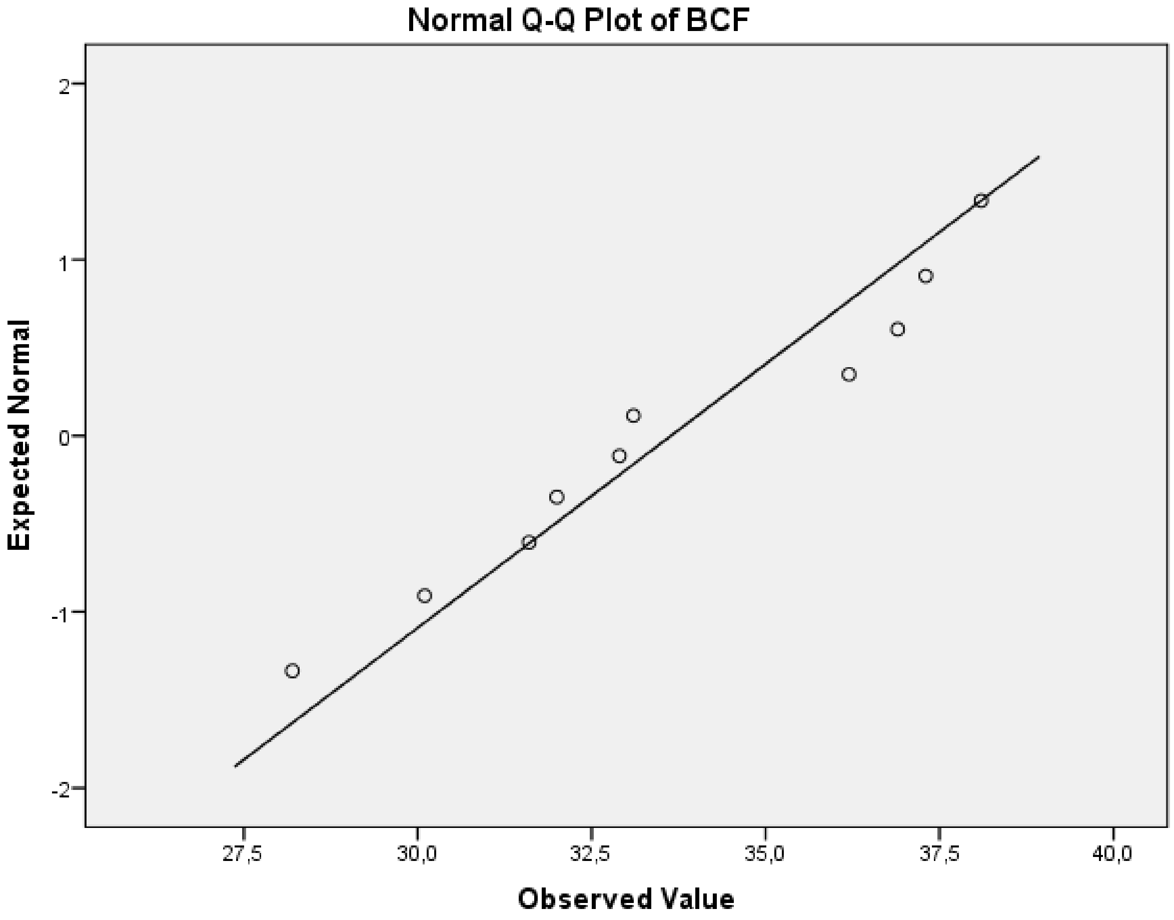
Normal Q-Q Plot of BCF
Expected Normal
Observed Value
2
1
0
-1
-2
27,5
30,0
32,5
35,0
37,5
40,0

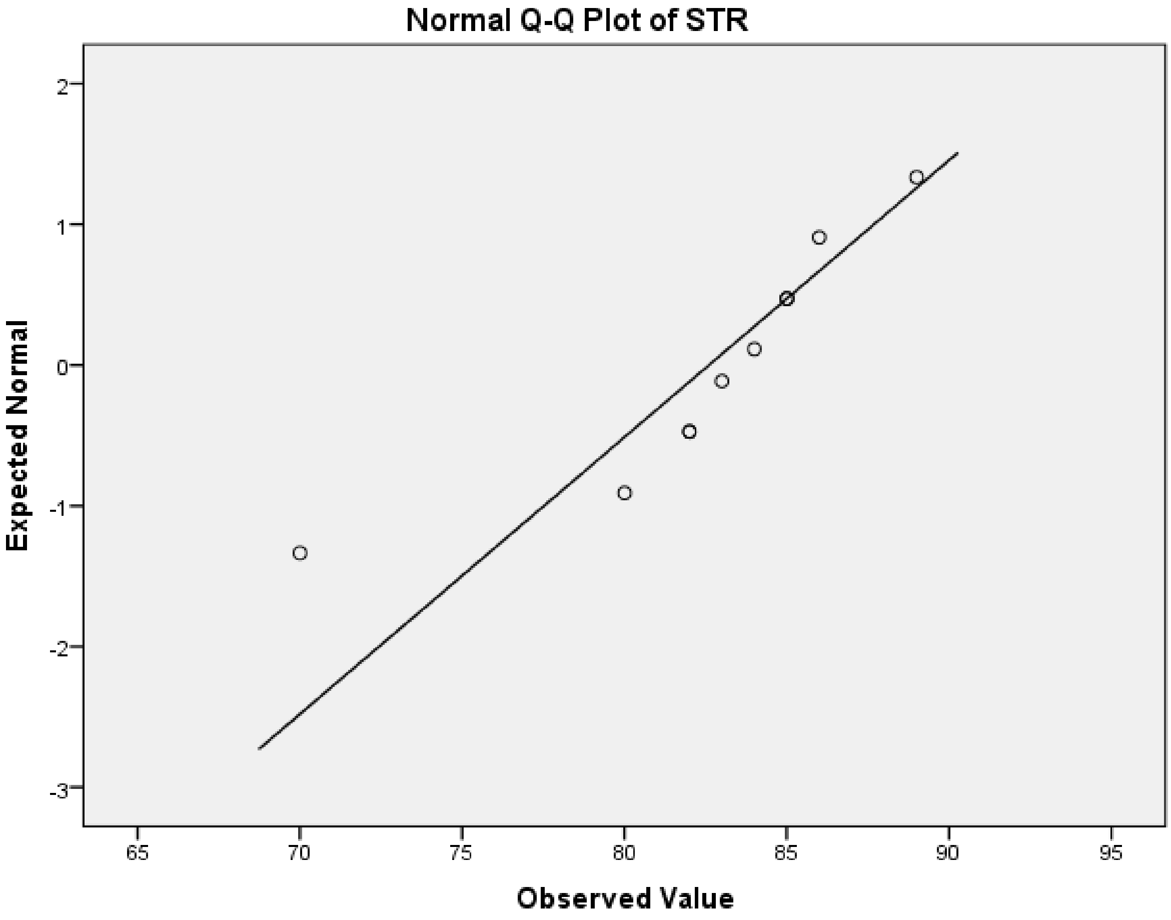
Normal Q-Q Plot of STR
Expected Normal
2
1
0
-1
-2
-3
65
70
75
80
85
90
95
Observed Value

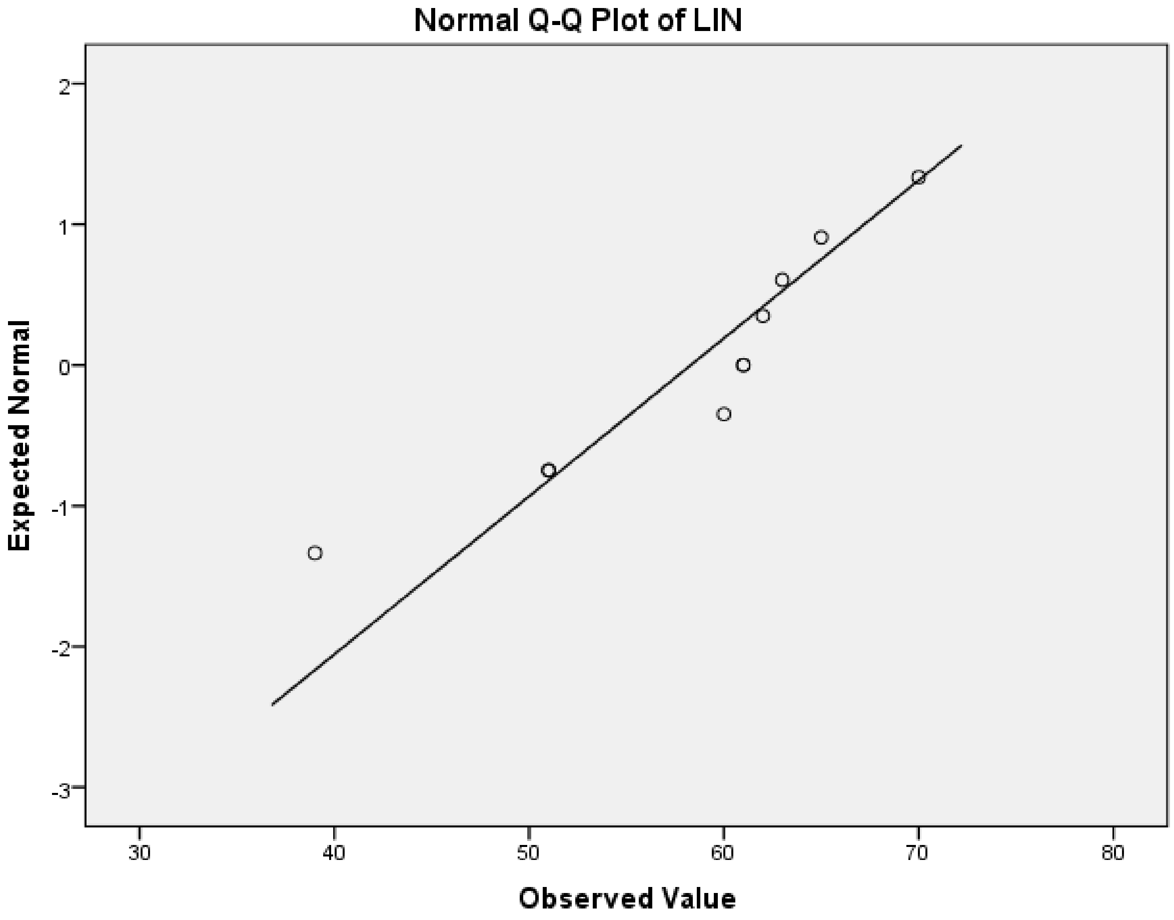

Detrended Normal Q-Q Plots

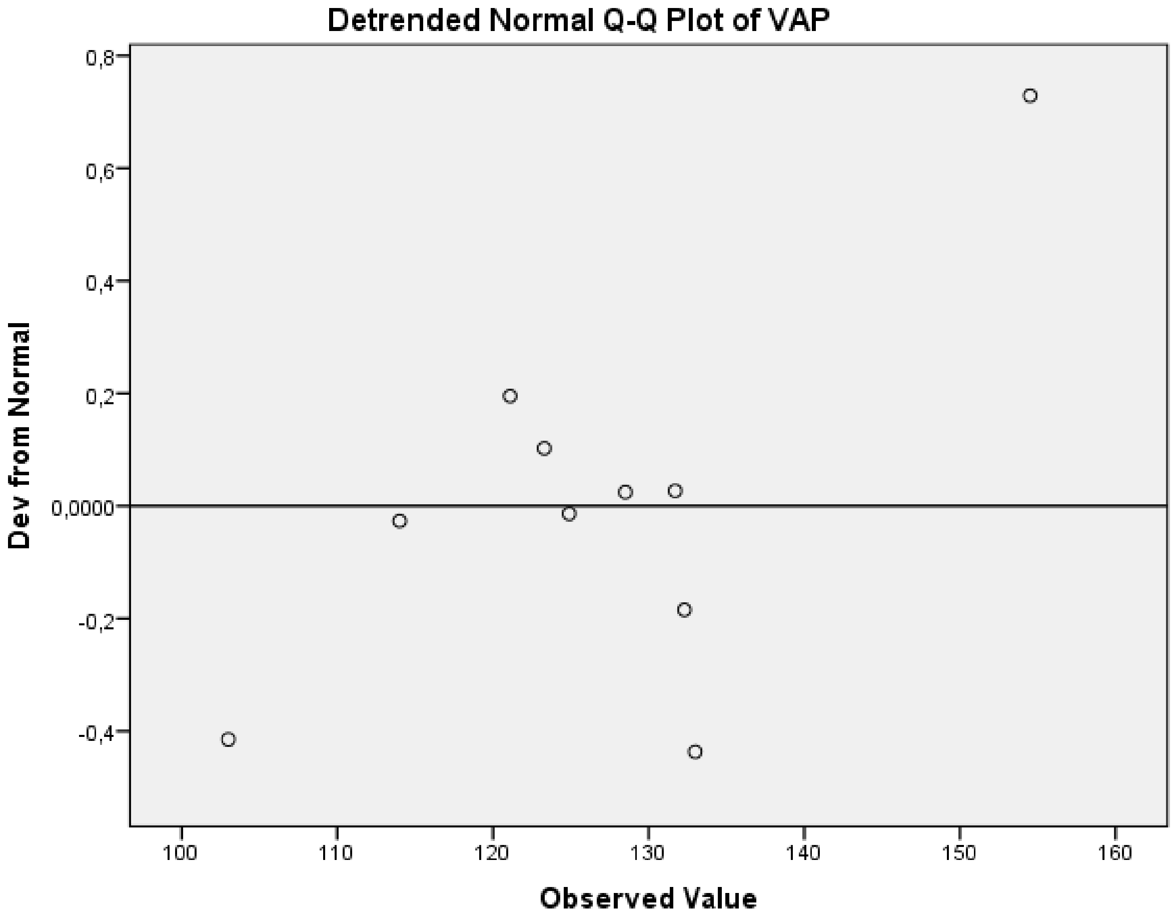

Detrended Normal Q-Q Plot of VAP
Dev from Normal
0,8
0,6
0,4
0,2
0,0000
-0,2
-0,4
100
110
120
130
140
150
160
Observed Value

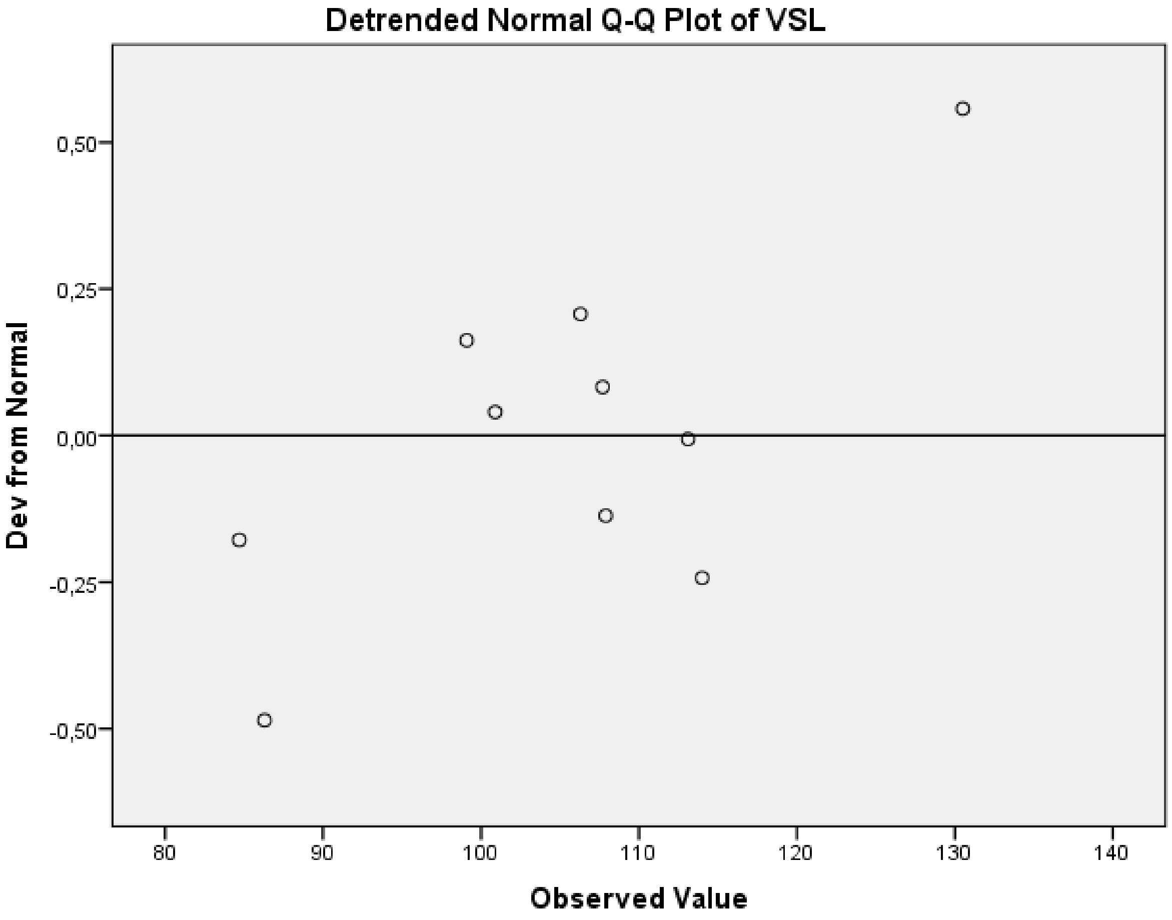

Detrended Normal Q-Q Plot of VSL
Dev from Normal
0,50
0,25
0,00
-0,25
-0,50
80
90
100
110
120
130
140
Observed Value

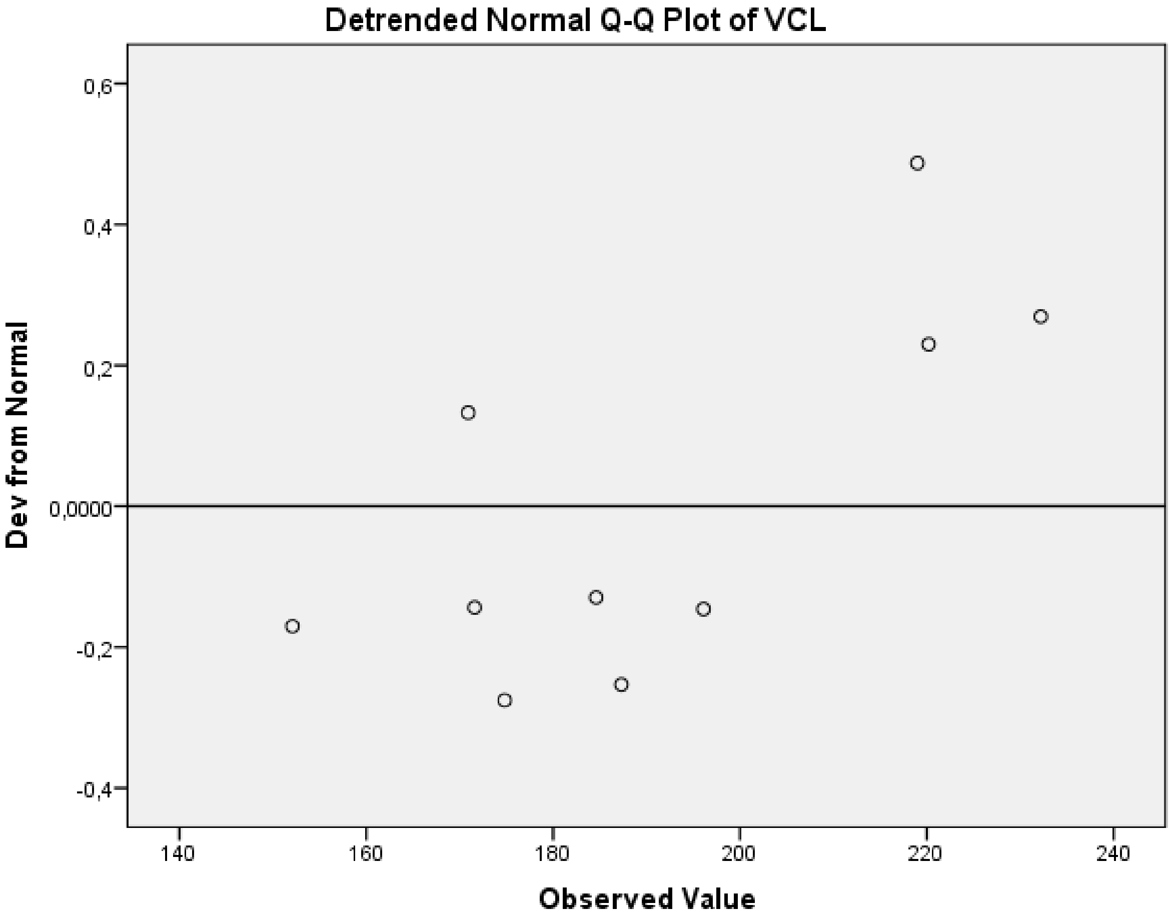
Detrended Normal Q-Q Plot of VCL
Dev from Normal
0,6
0,4
0,2
0,0000
-0,2
-0,4
140
160
180
200
220
240
Observed Value

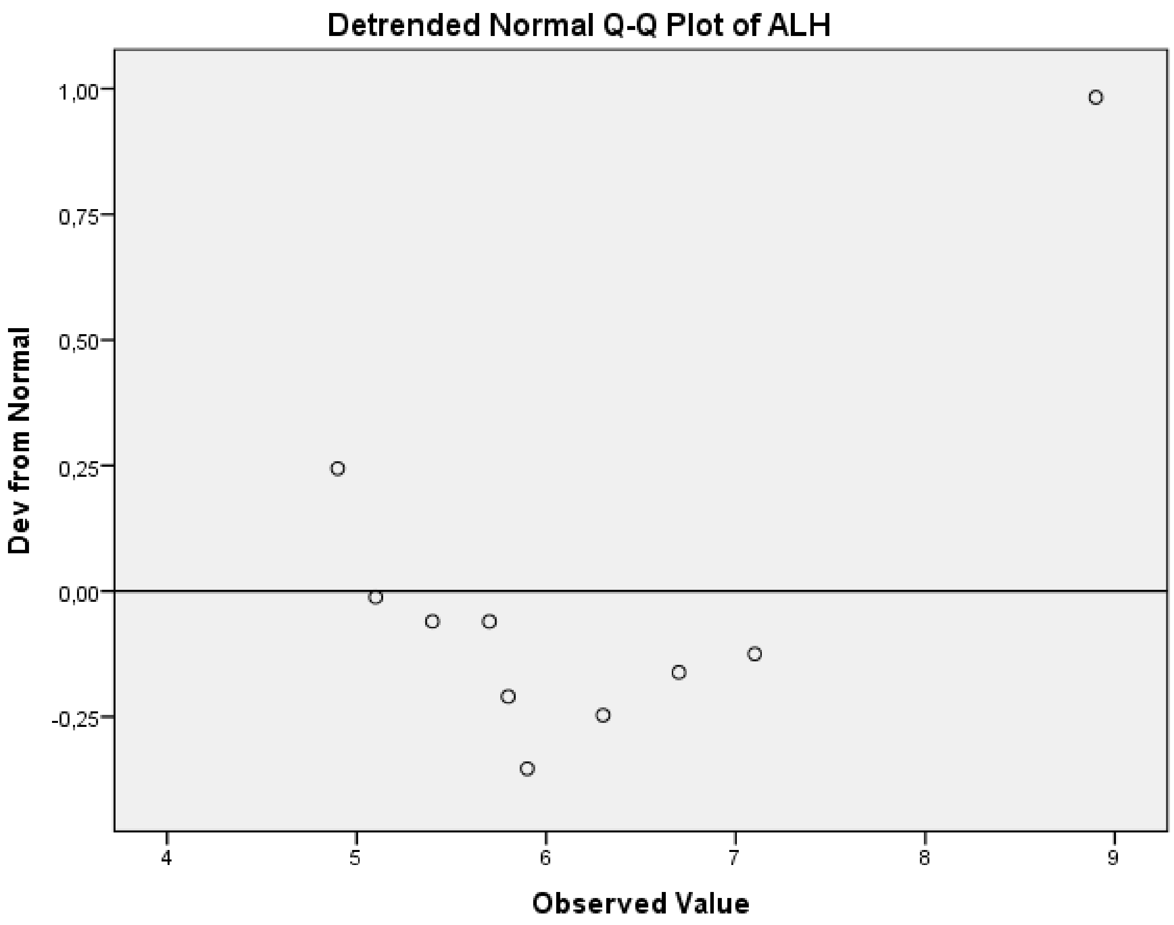
Detrended Normal Q-Q Plot of ALH
Dev from Normal
1,00
0,75
0,50
0,25
0,00
-0,25
4
5
6
7
8
9
Observed Value

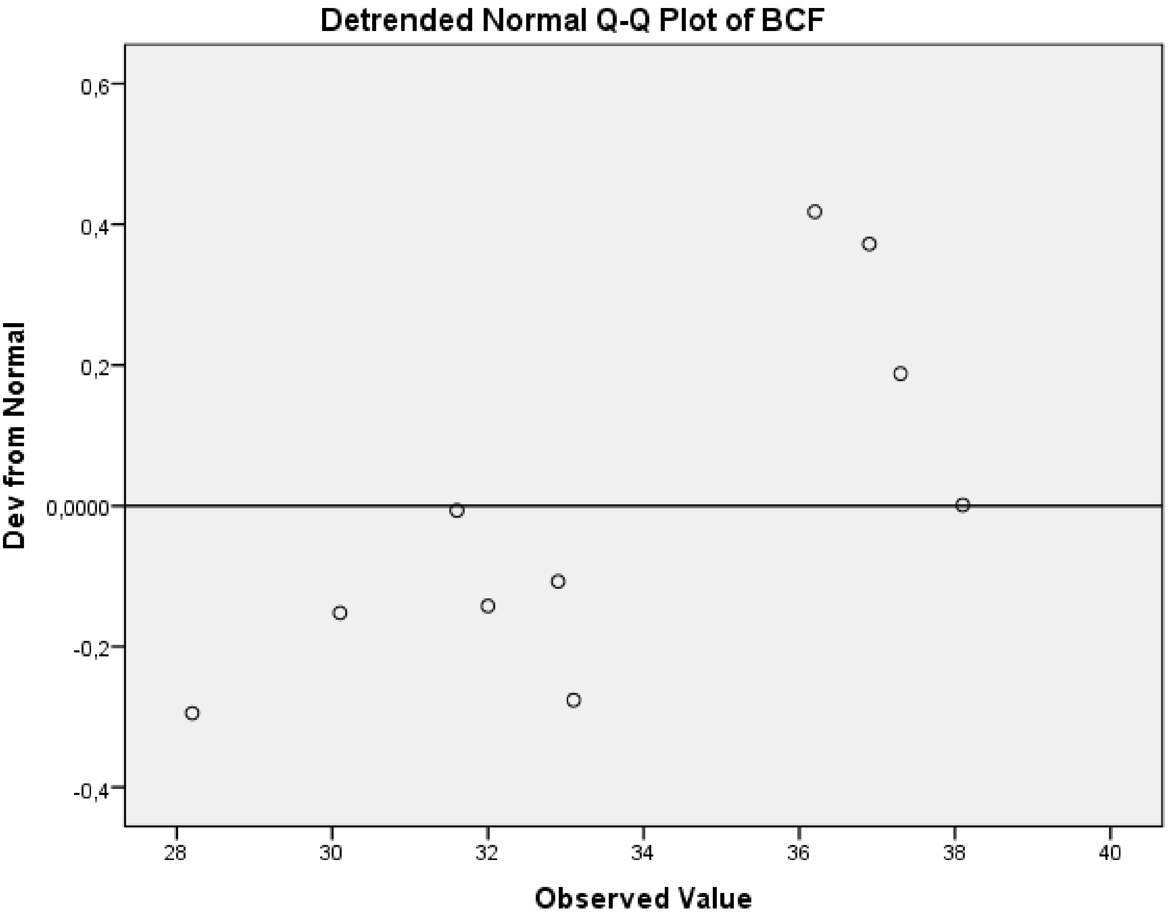
Detrended Normal Q-Q Plot of BCF
Dev from Normal
0,6
0,4
0,2
0,0000
-0,2
-0,4
28
30
32
34
36
38
40
Observed Value

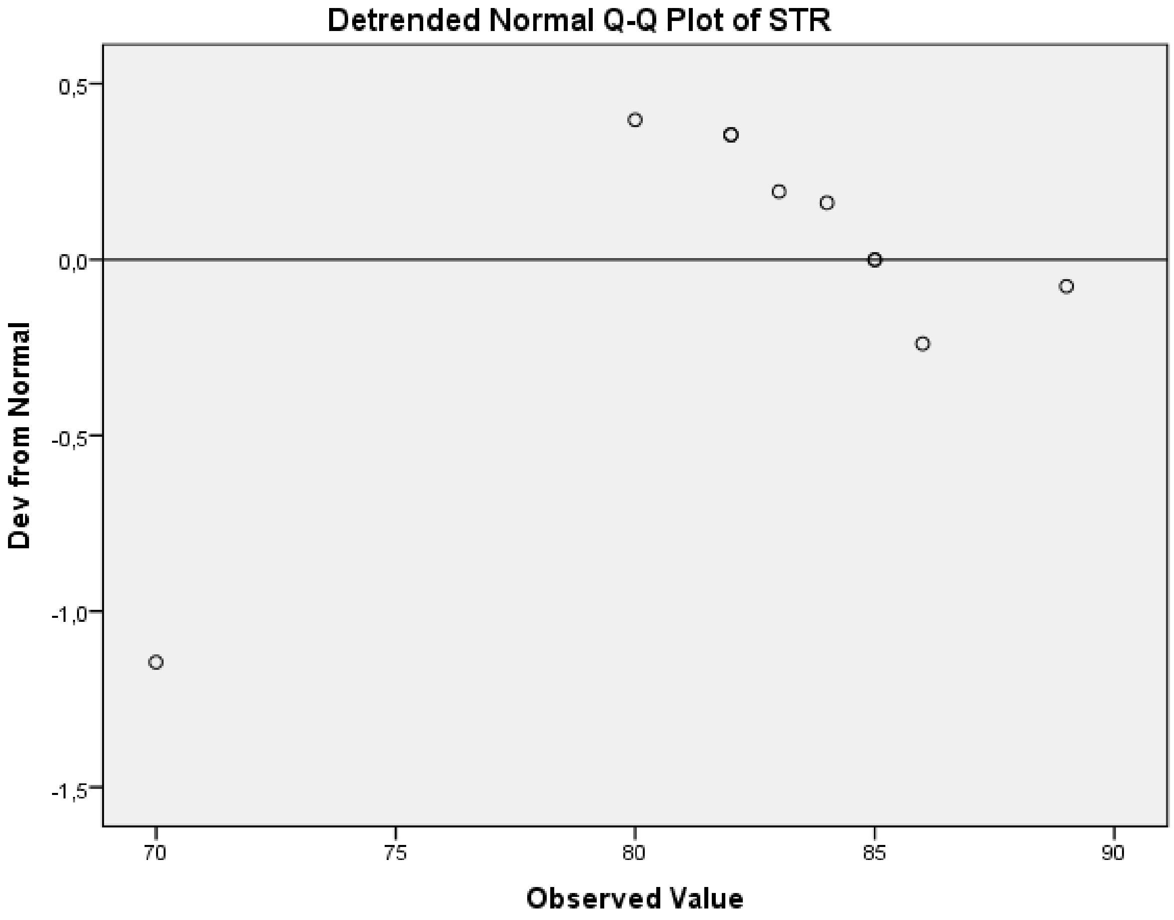

Detrended Normal Q-Q Plot of STR
0,5
0,0
-0,5
-1,0
-1,5
Dev from Normal
70
75
80
85
90
Observed Value

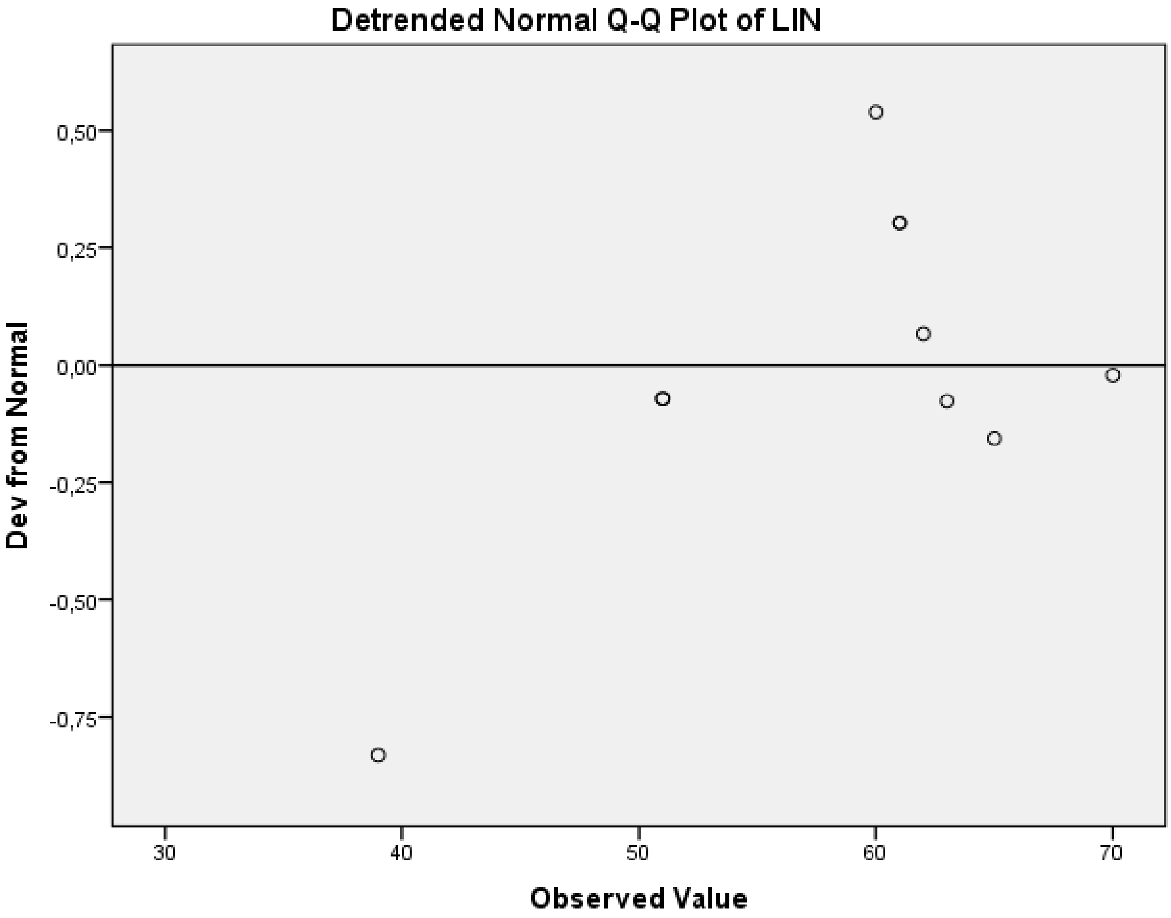

Grup = 10,00

Histograms

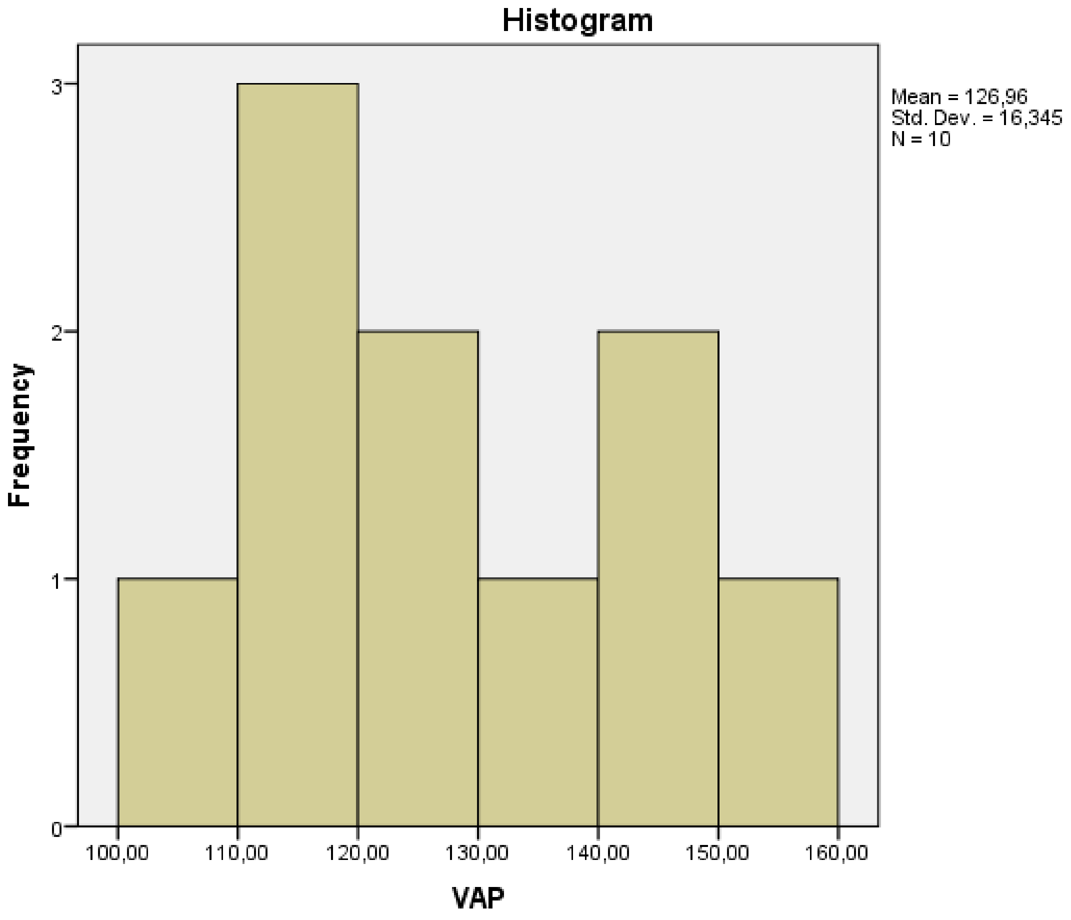
Histogram
Mean = 126,96
Std. Dev. = 16,345
N = 10
Frequency
3
2
1
0
100,00
110,00
120,00
130,00
140,00
150,00
160,00
VAP

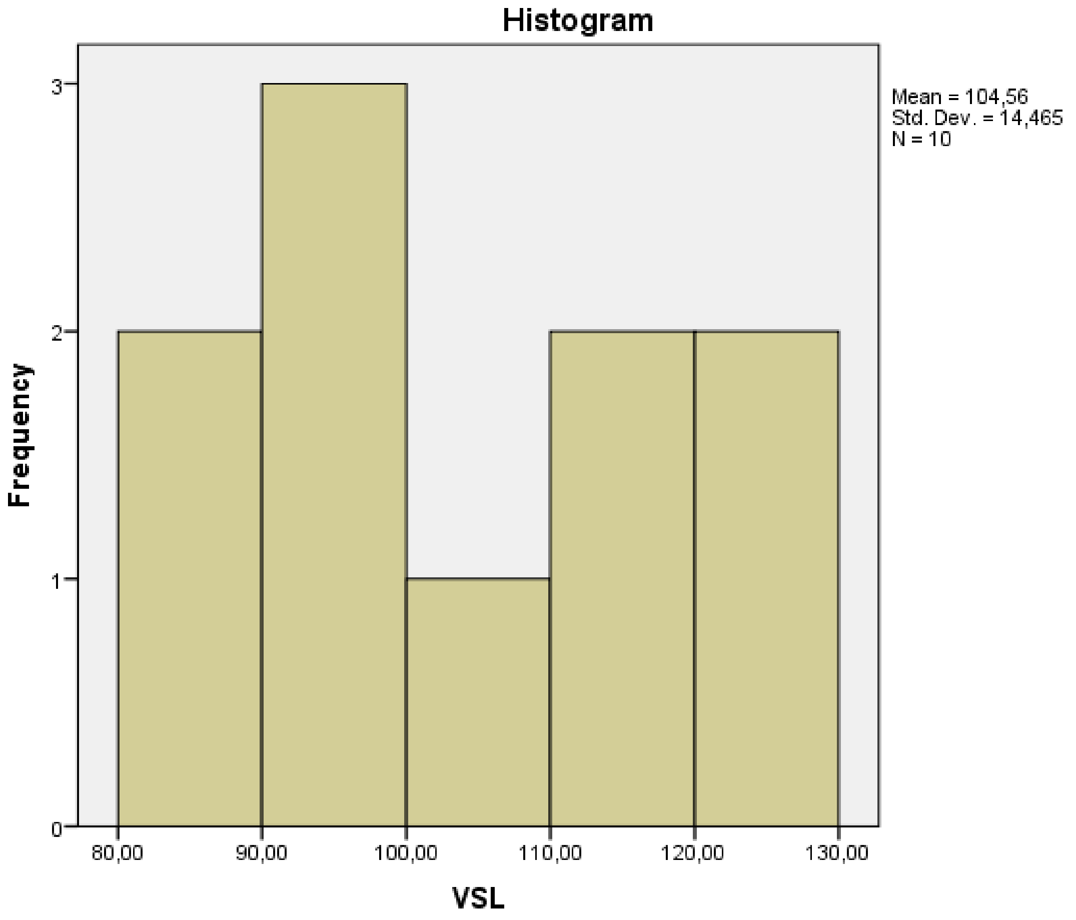

Histogram
Mean = 104,56
Std. Dev. = 14,465
N = 10
Frequency
0
1
2
3
80,00
90,00
100,00
110,00
120,00
130,00
VSL

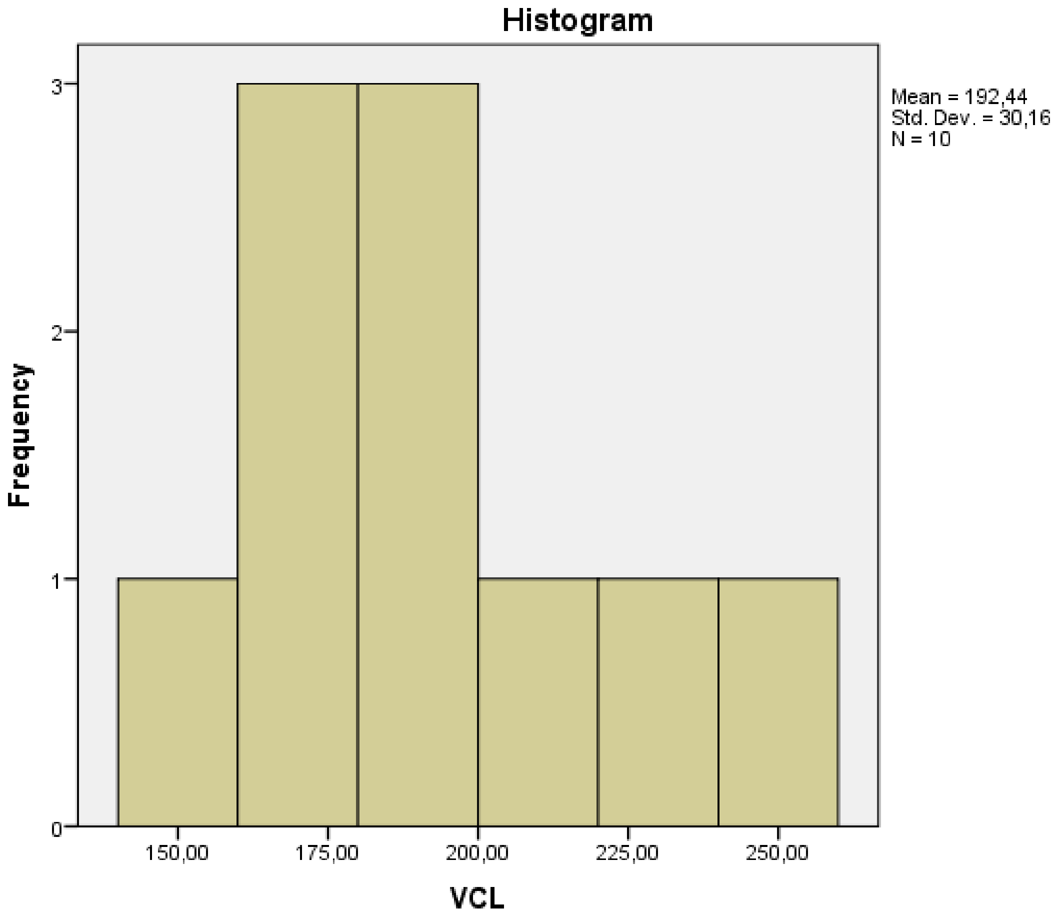

Histogram
Mean = 192,44
Std. Dev. = 30,16
N = 10
Frequency
0
1
2
3
150,00
175,00
200,00
225,00
250,00
VCL

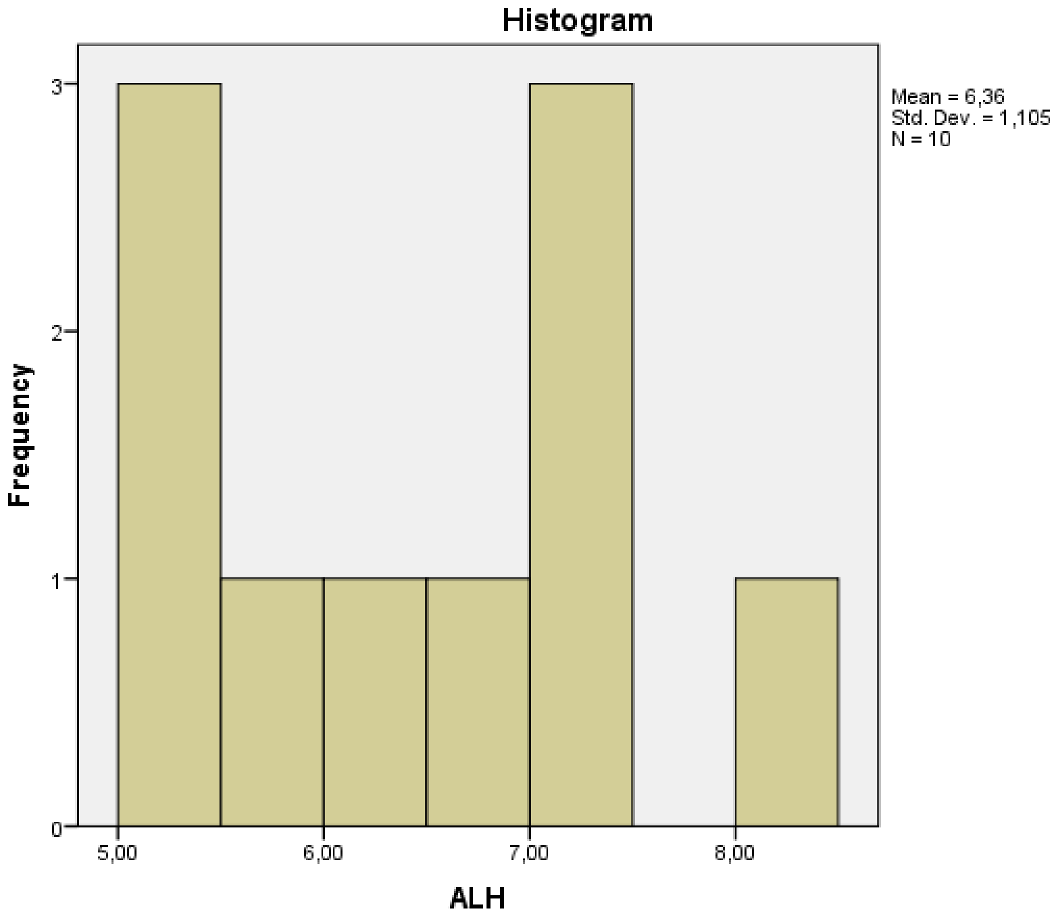
Histogram
Mean = 6,36
Std. Dev. = 1,105
N = 10
Frequency
0
1
2
3
5,00
6,00
7,00
8,00
ALH

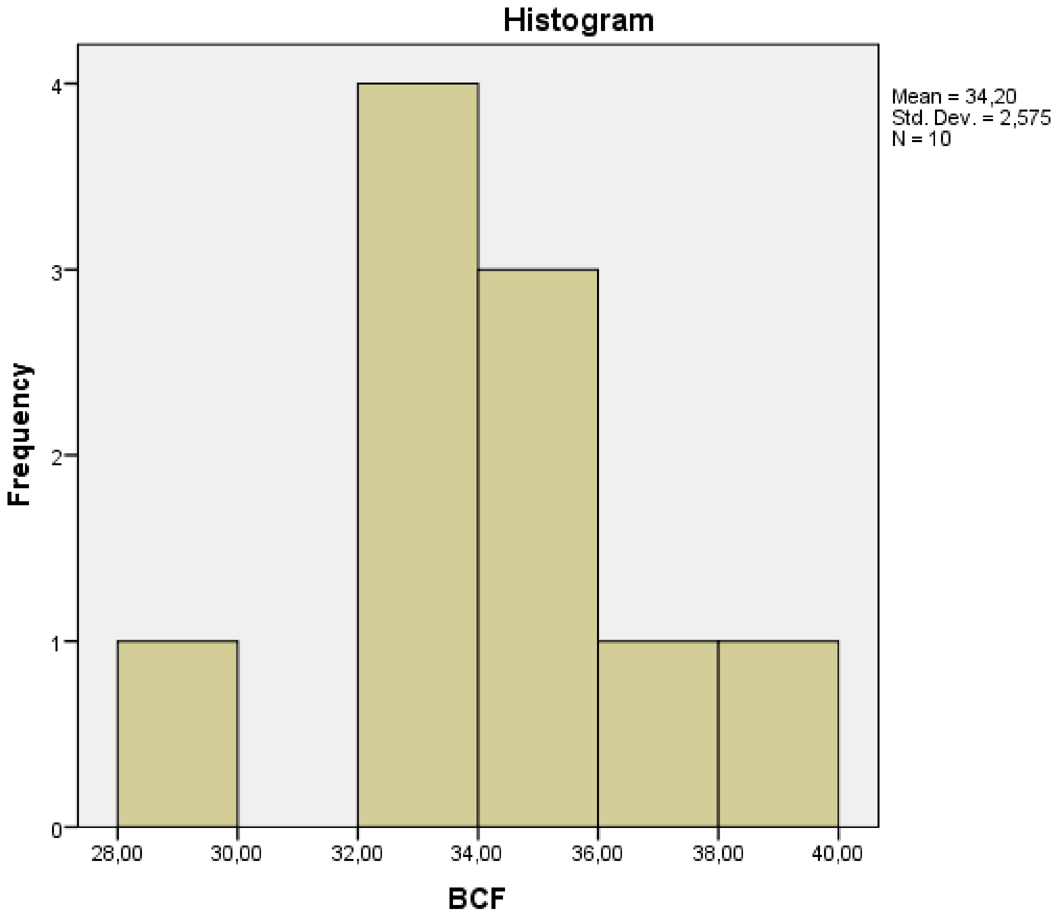

Histogram
Mean = 34,20
Std. Dev. = 2,575
N = 10
Frequency
0
1
2
3
4
28,00
30,00
32,00
34,00
36,00
38,00
40,00
BCF

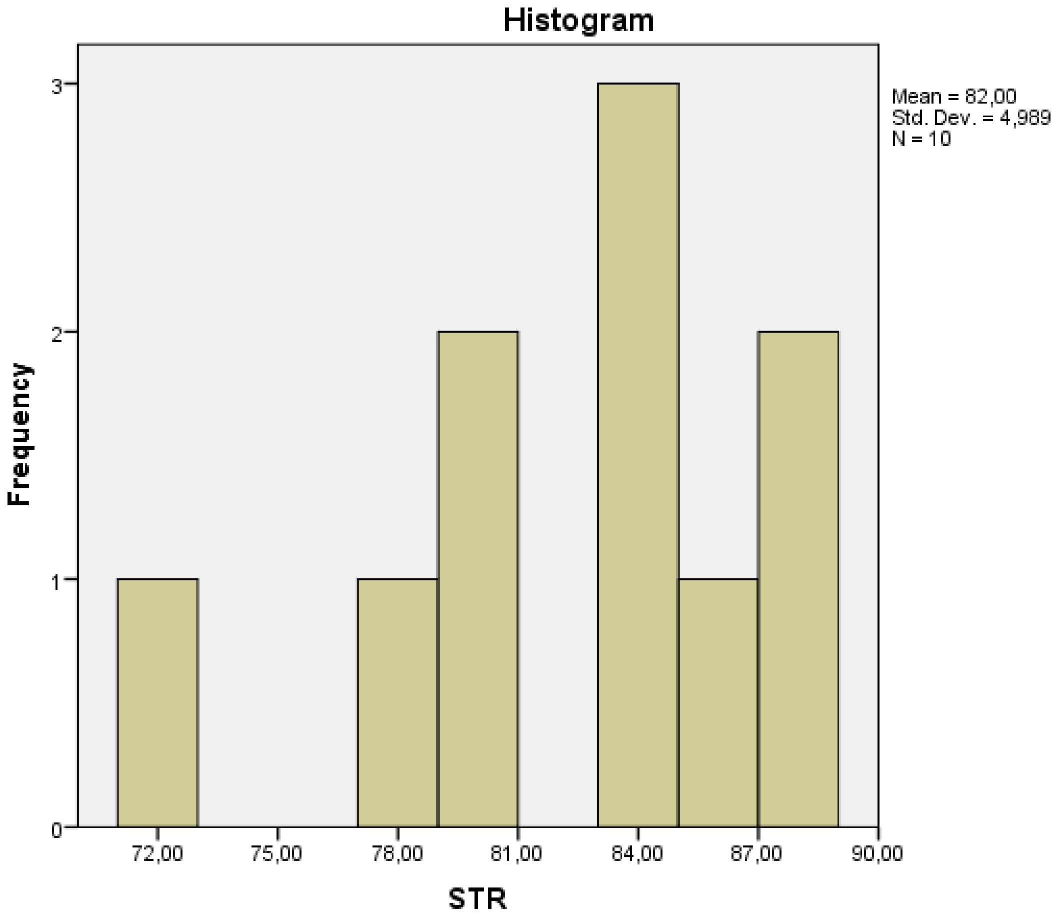

Histogram
Mean = 82,00
Std. Dev. = 4,989
N = 10
Frequency
3
2
1
0
72,00
75,00
78,00
81,00
84,00
87,00
90,00
STR

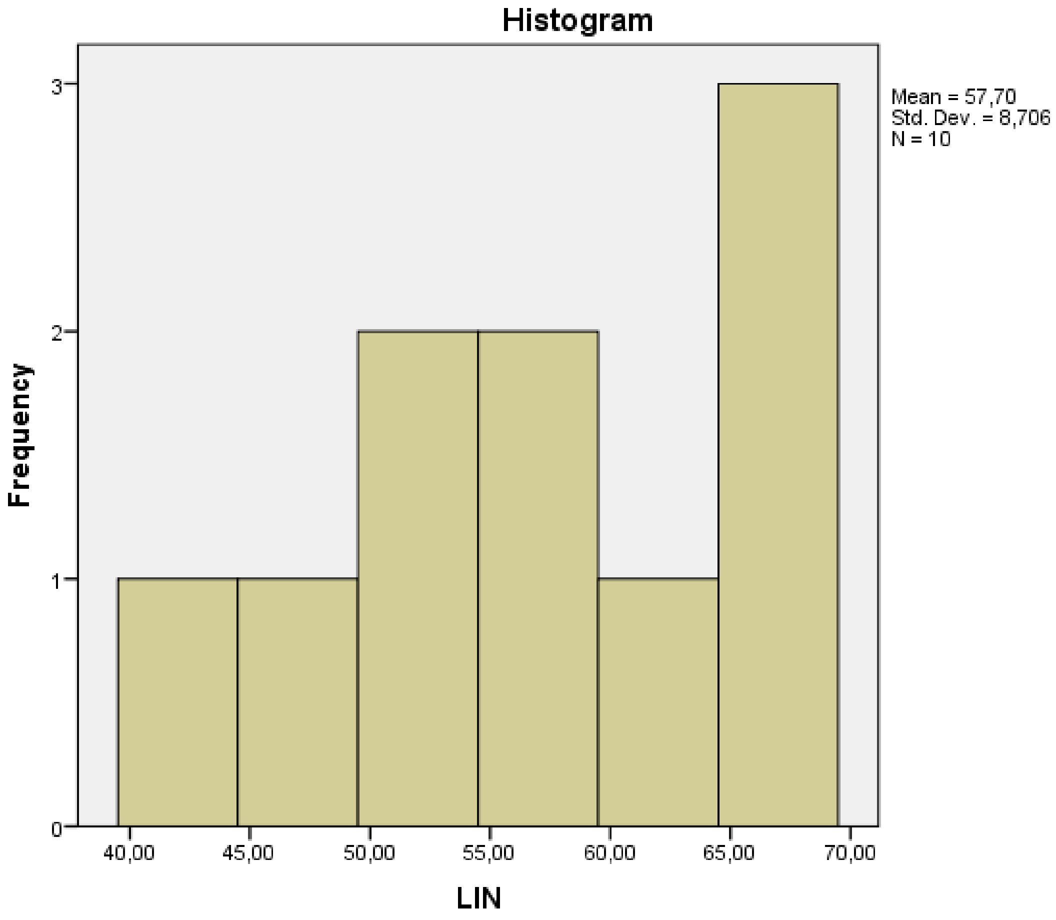

Stem-and-Leaf Plots

```
VAP Stem-and-Leaf Plot for
Grup= 10,00

 Frequency    Stem &  Leaf

     1,00       10 .  2
     3,00       11 .  146
     2,00       12 .  28
     1,00       13 .  5
     2,00       14 .  03
     1,00       15 .  4

 Stem width:  10,00
 Each leaf:       1 case(s)
```

```
VSL Stem-and-Leaf Plot for
Grup= 10,00

 Frequency    Stem &  Leaf

     2,00        8 .  66
     3,00        9 .  778
     1,00       10 .  1
     2,00       11 .  18
     2,00       12 .  45

 Stem width:  10,00
 Each leaf:       1 case(s)
```

```
VCL Stem-and-Leaf Plot for
Grup= 10,00

 Frequency    Stem &  Leaf

     7,00        1 .  5667889
     3,00        2 .  034

 Stem width:  100,0
 Each leaf:       1 case(s)
```

```
ALH Stem-and-Leaf Plot for
Grup= 10,00

 Frequency    Stem &  Leaf

     4,00        5 .  1127
     2,00        6 .  15
     3,00        7 .  114
     1,00        8 .  3

 Stem width:   1,00
 Each leaf:       1 case(s)
```

```
BCF Stem-and-Leaf Plot for
Grup= 10,00

 Frequency    Stem &  Leaf

     1,00        2 .  9
```

```
     4,00        3 .  2223
     5,00        3 .  55568

 Stem width:  10,00
 Each leaf:       1 case(s)
```

```
STR Stem-and-Leaf Plot for
Grup= 10,00

 Frequency    Stem &  Leaf

     1,00        7 .  2
     2,00        7 .  79
     4,00        8 .  0344
     3,00        8 .  678

 Stem width:  10,00
 Each leaf:       1 case(s)
```

```
LIN Stem-and-Leaf Plot for
Grup= 10,00

 Frequency    Stem &  Leaf

     1,00        4 .  2
     1,00        4 .  8
     2,00        5 .  44
     2,00        5 .  59
     1,00        6 .  4
     3,00        6 .  669

 Stem width:  10,00
 Each leaf:       1 case(s)
```

Normal Q-Q Plots

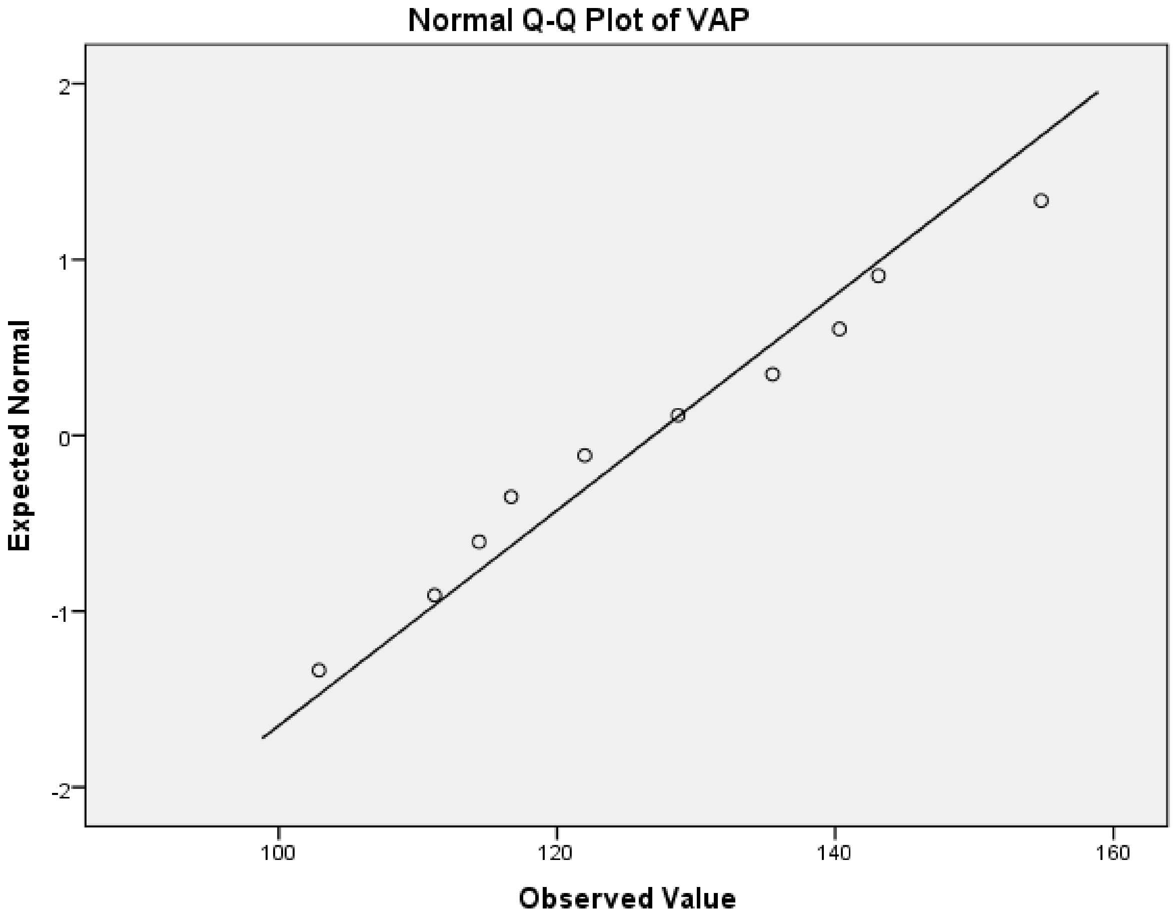

Normal Q-Q Plot of VAP
Expected Normal
2
1
0
-1
-2
100
120
140
160
Observed Value

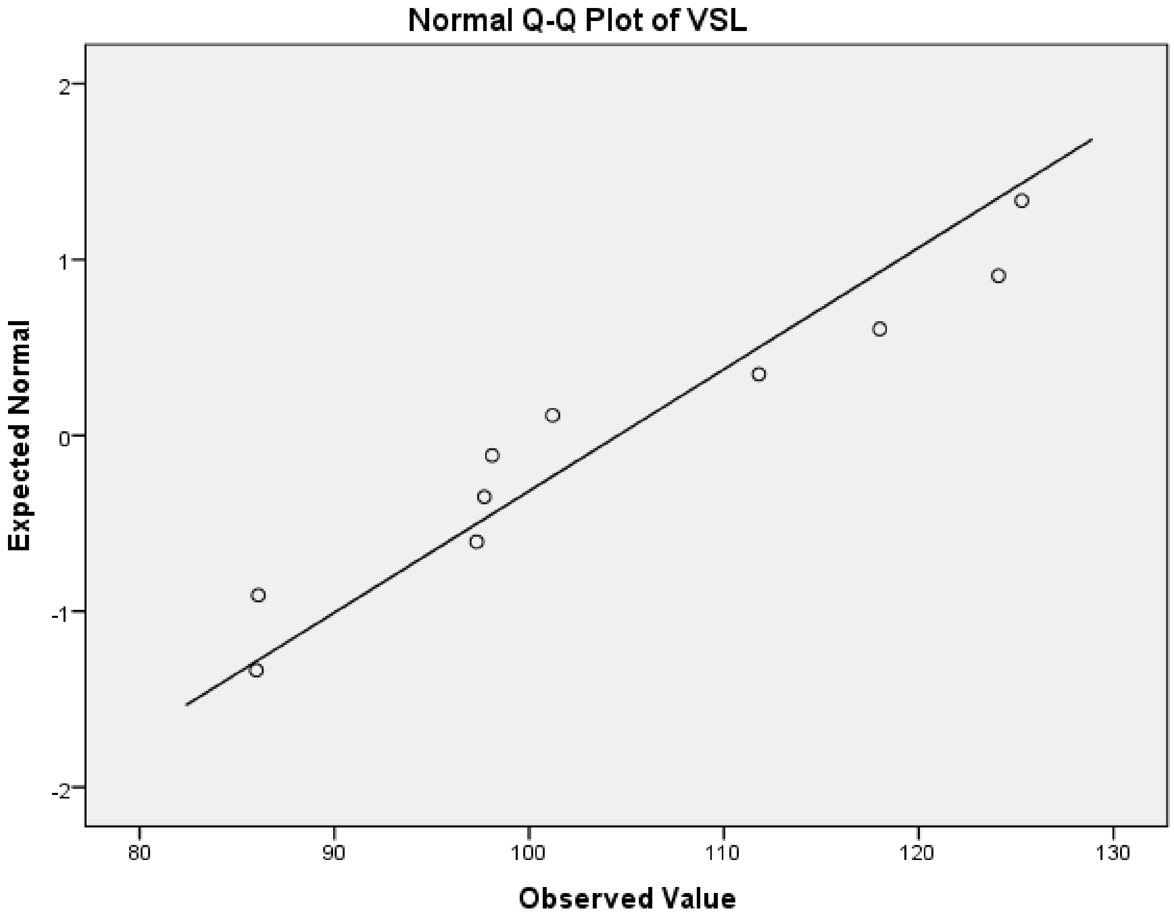
Normal Q-Q Plot of VSL
Expected Normal
Observed Value
2
1
0
-1
-2
80
90
100
110
120
130

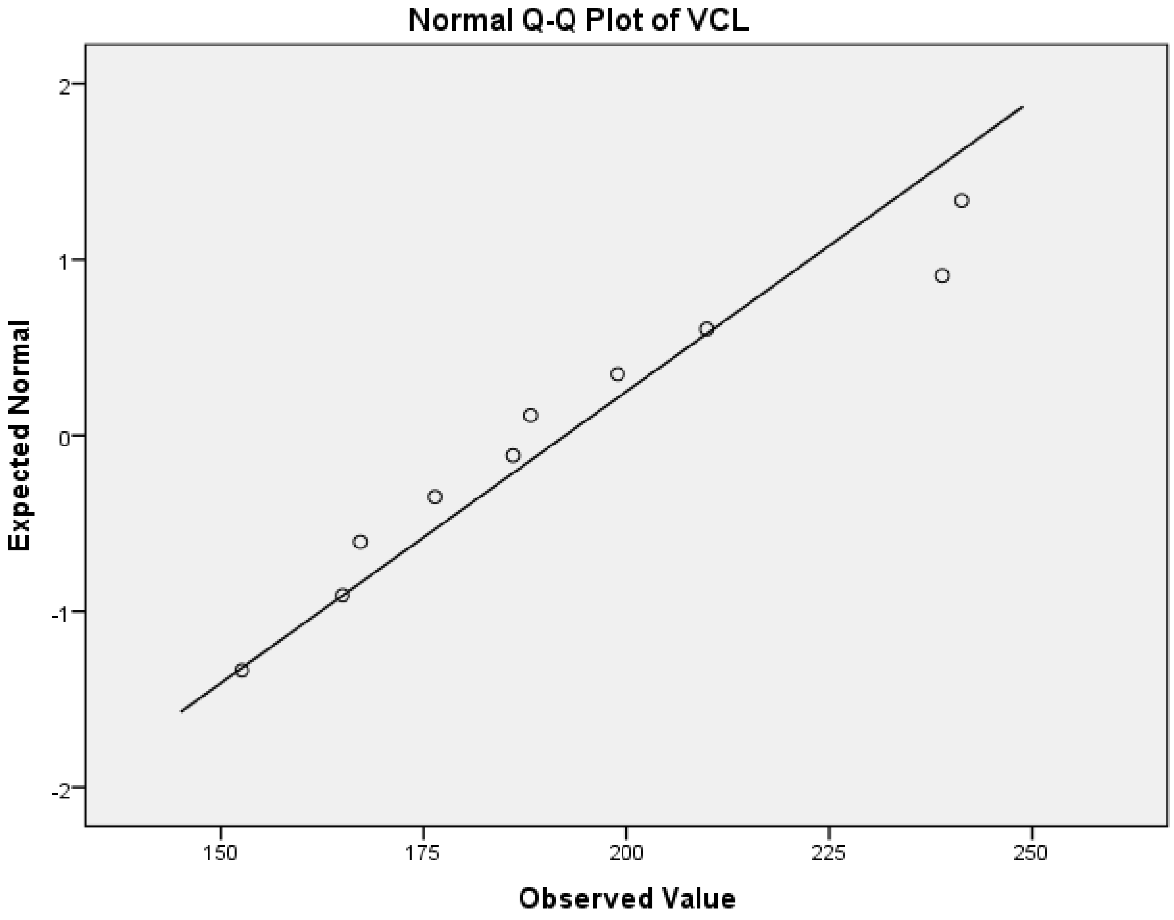
Normal Q-Q Plot of VCL
Expected Normal
2
1
0
-1
-2
150
175
200
225
250
Observed Value

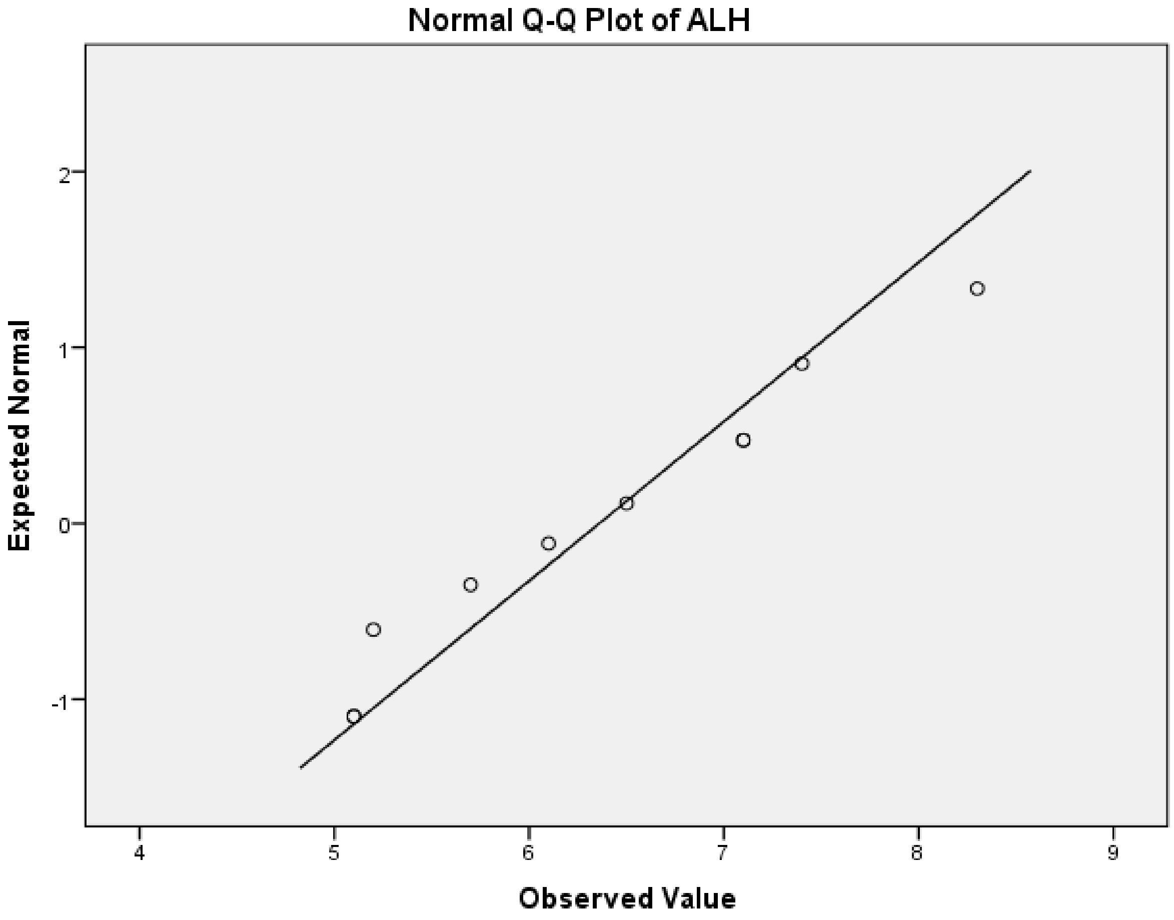
Normal Q-Q Plot of ALH
Expected Normal
2
1
0
-1
4
5
6
7
8
9
Observed Value

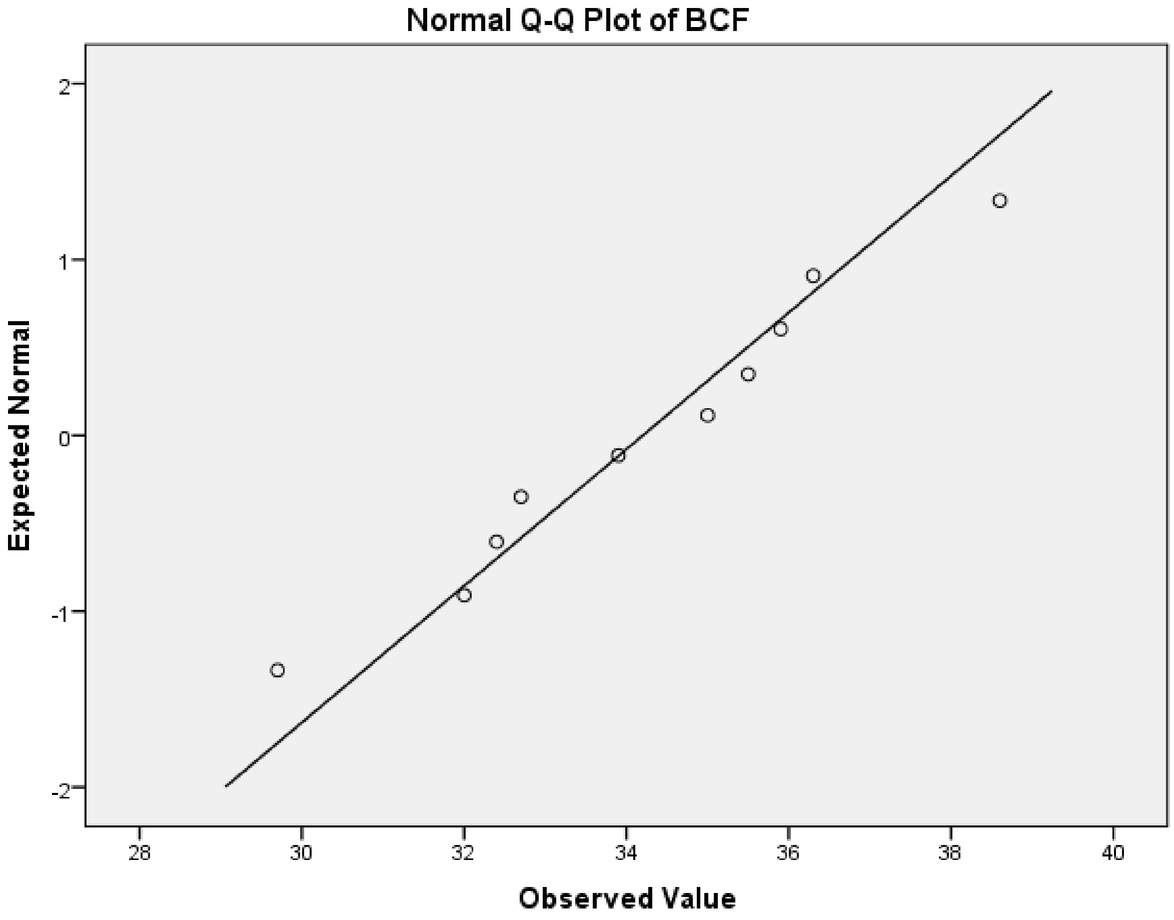

Normal Q-Q Plot of BCF
Expected Normal
2
1
0
-1
-2
28
30
32
34
36
38
40
Observed Value

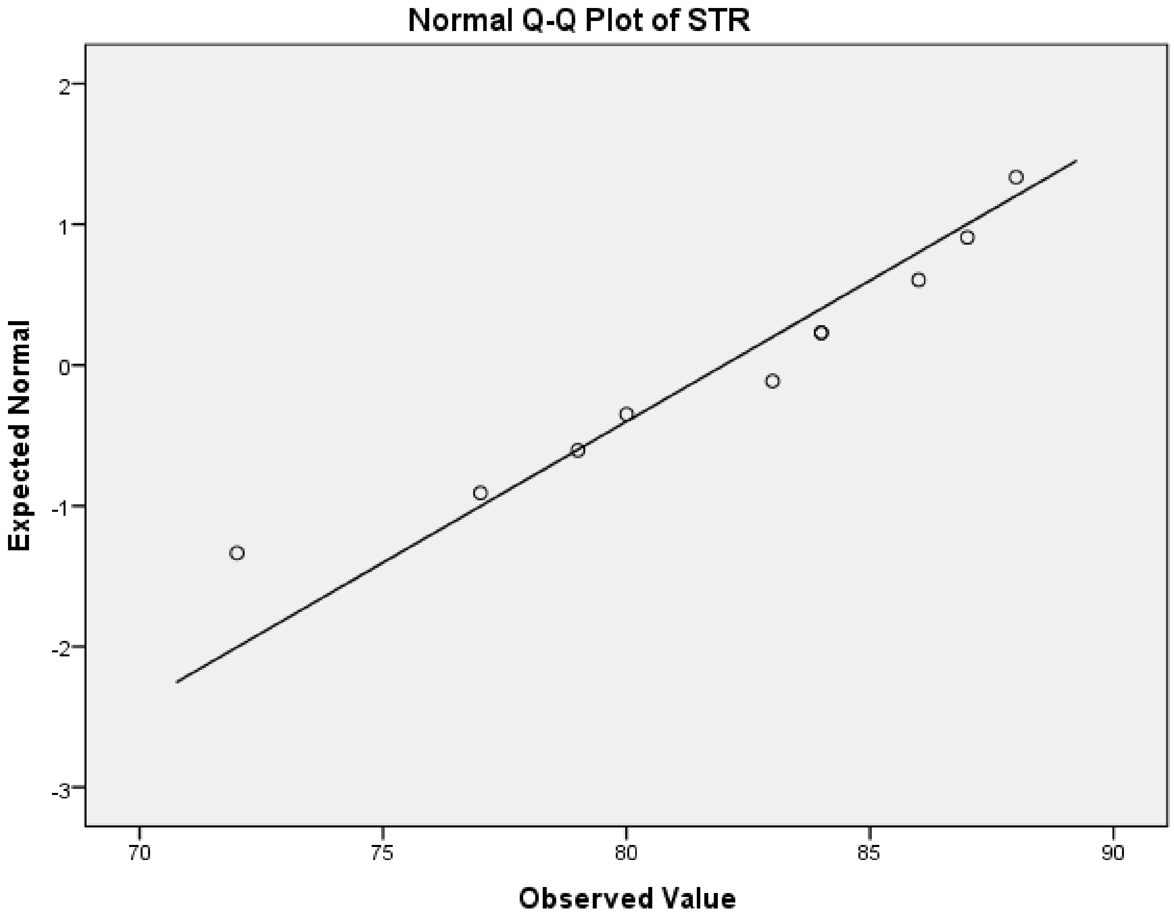

Normal Q-Q Plot of STR
Expected Normal
Observed Value
2
1
0
-1
-2
-3
70
75
80
85
90

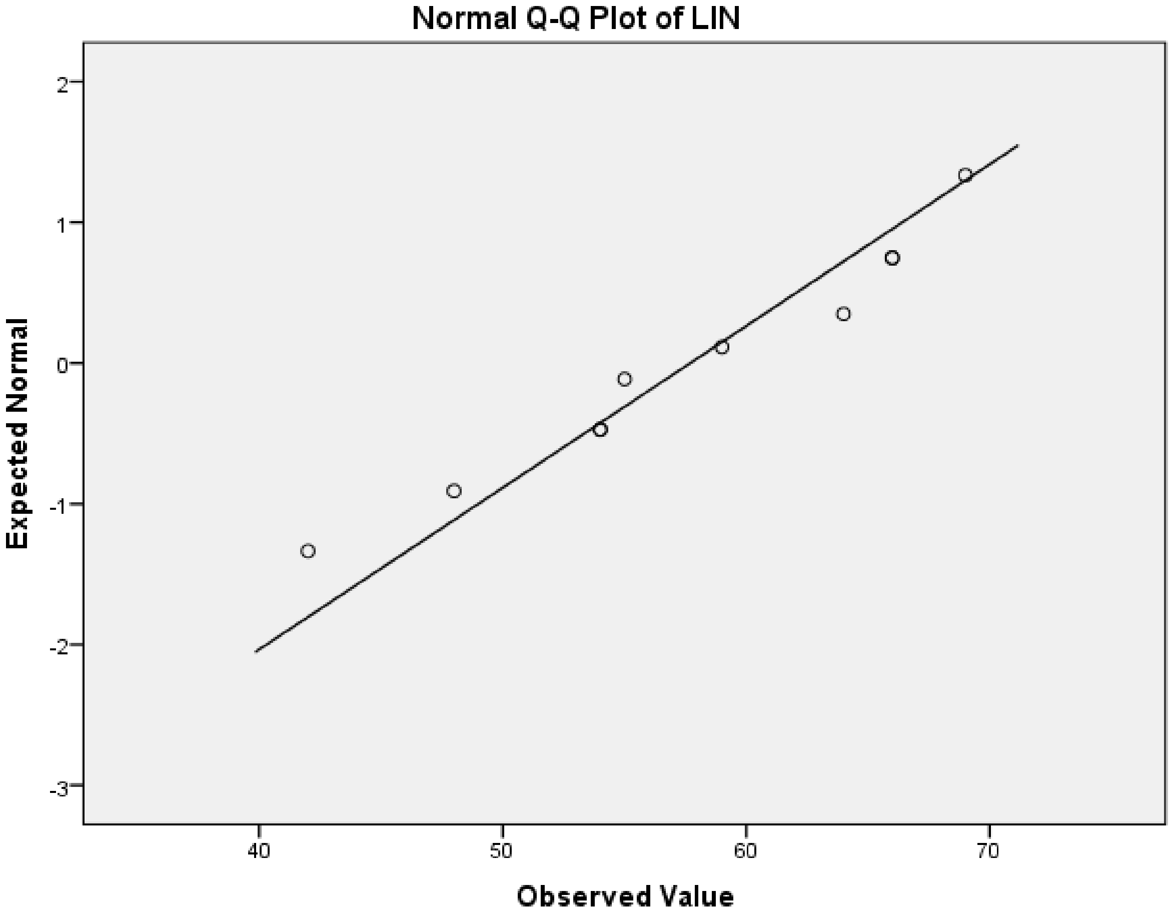

Detrended Normal Q-Q Plots

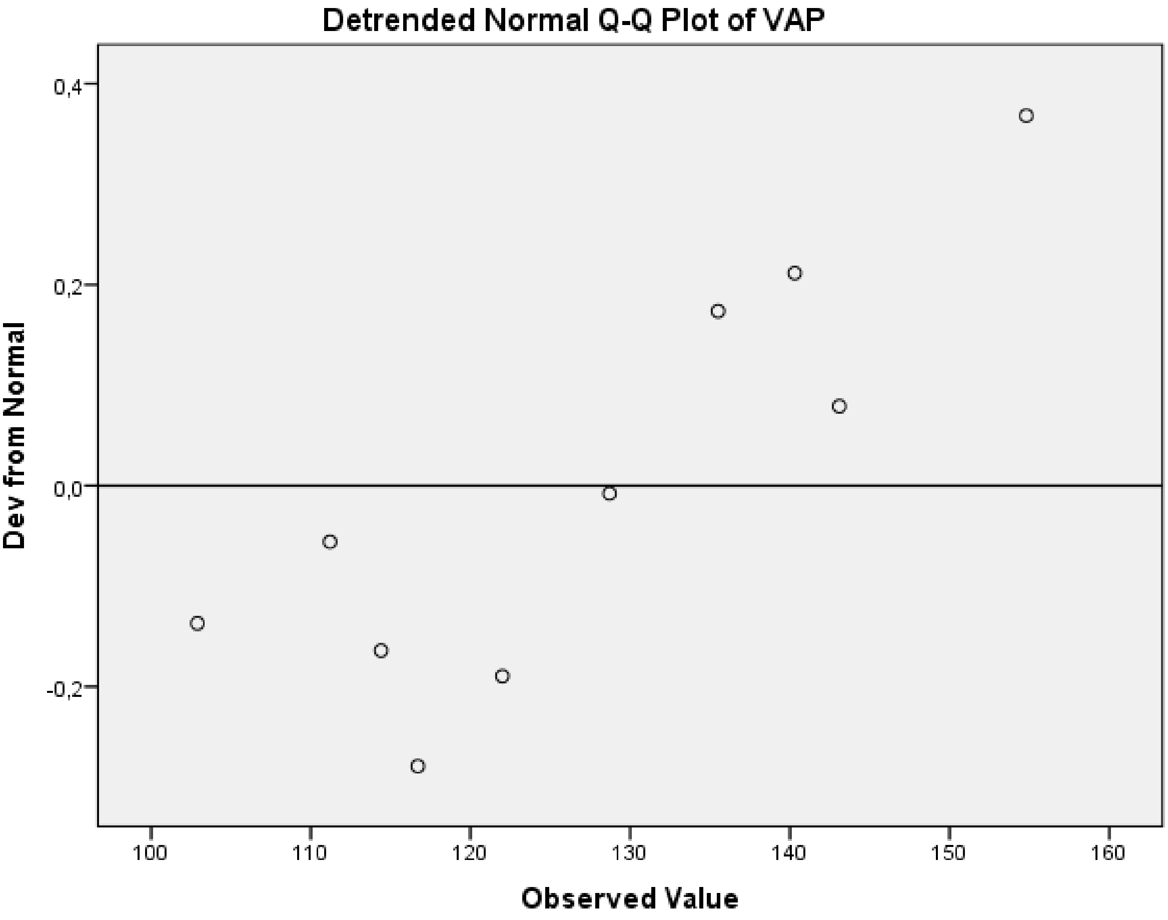
Detrended Normal Q-Q Plot of VAP
Dev from Normal
0,4
0,2
0,0
-0,2
100
110
120
130
140
150
160
Observed Value

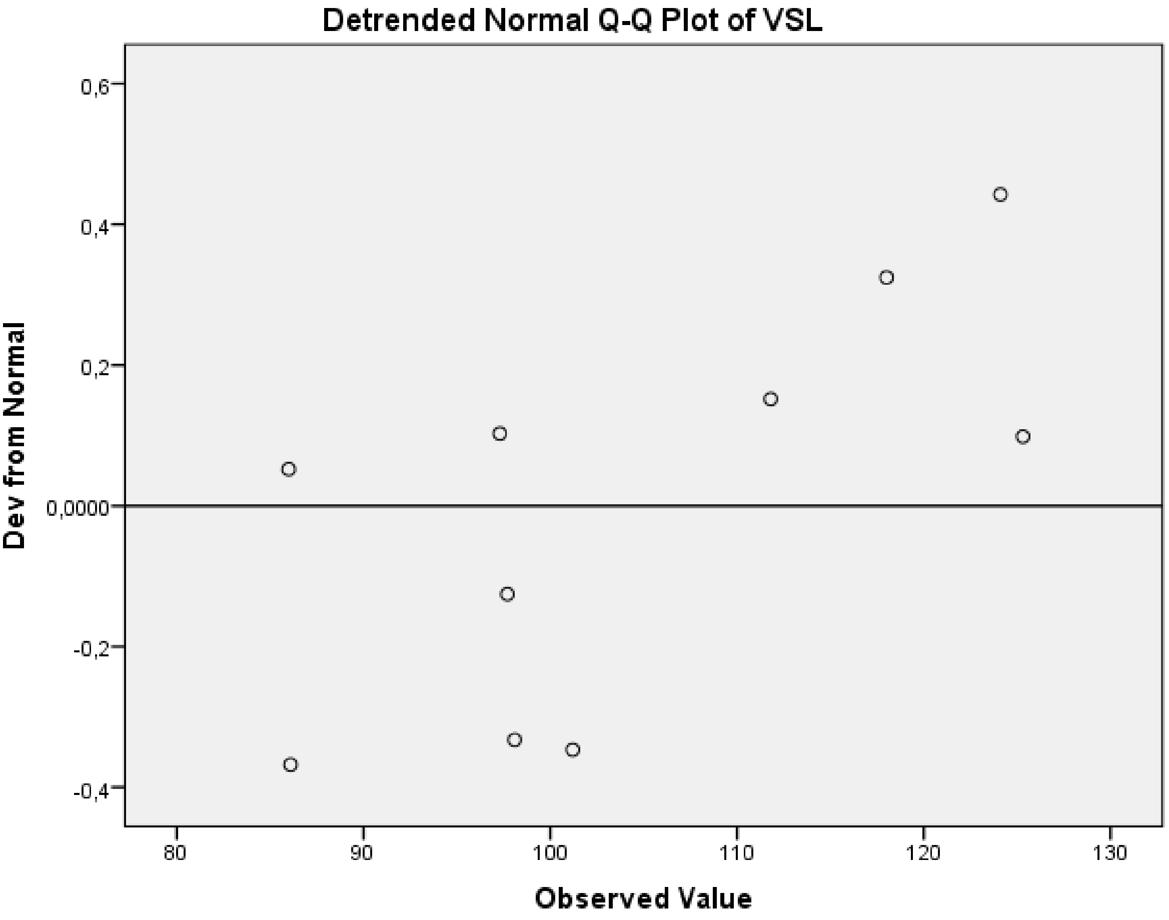

Detrended Normal Q-Q Plot of VSL
Dev from Normal
0,6
0,4
0,2
0,0000
-0,2
-0,4
80
90
100
110
120
130
Observed Value

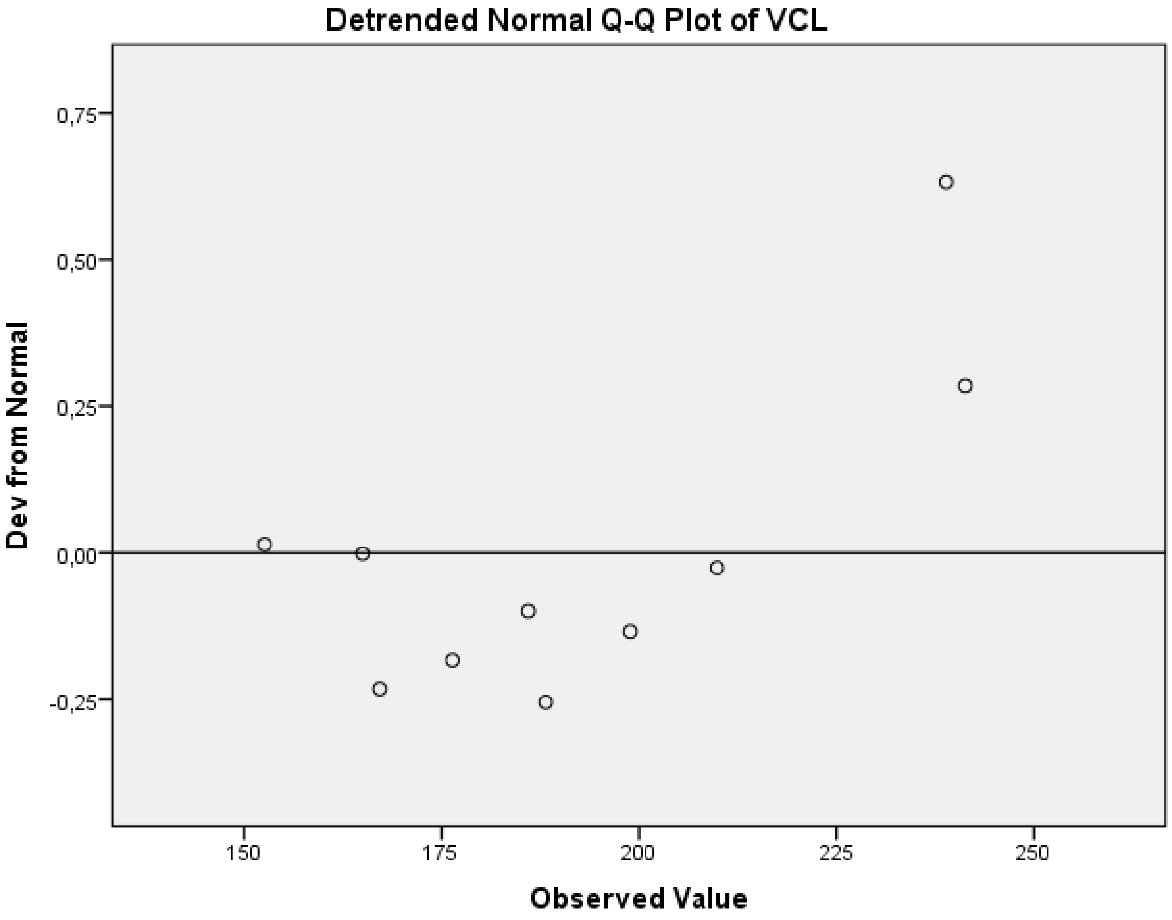
Detrended Normal Q-Q Plot of VCL
Dev from Normal
0,75
0,50
0,25
0,00
-0,25
150
175
200
225
250
Observed Value

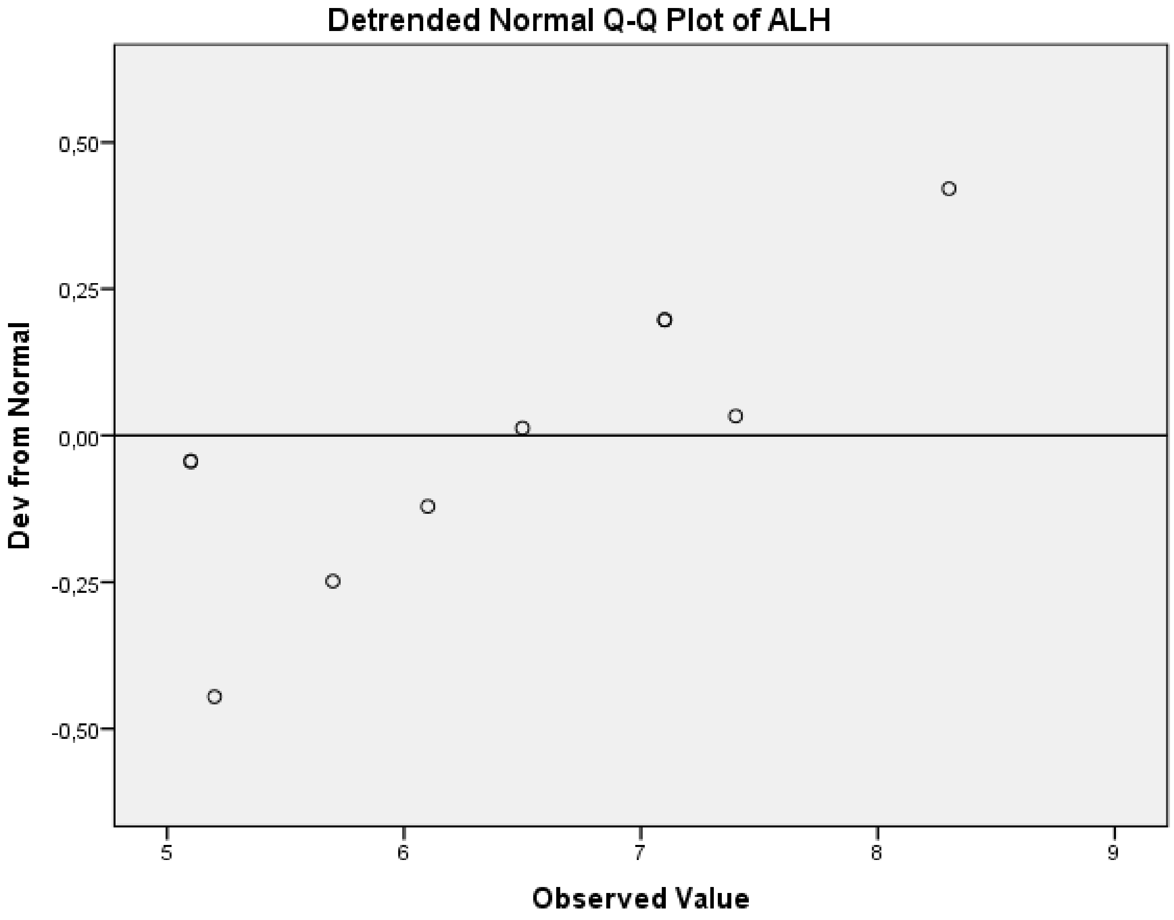

Detrended Normal Q-Q Plot of ALH
Dev from Normal
0,50
0,25
0,00
-0,25
-0,50
5
6
7
8
9
Observed Value

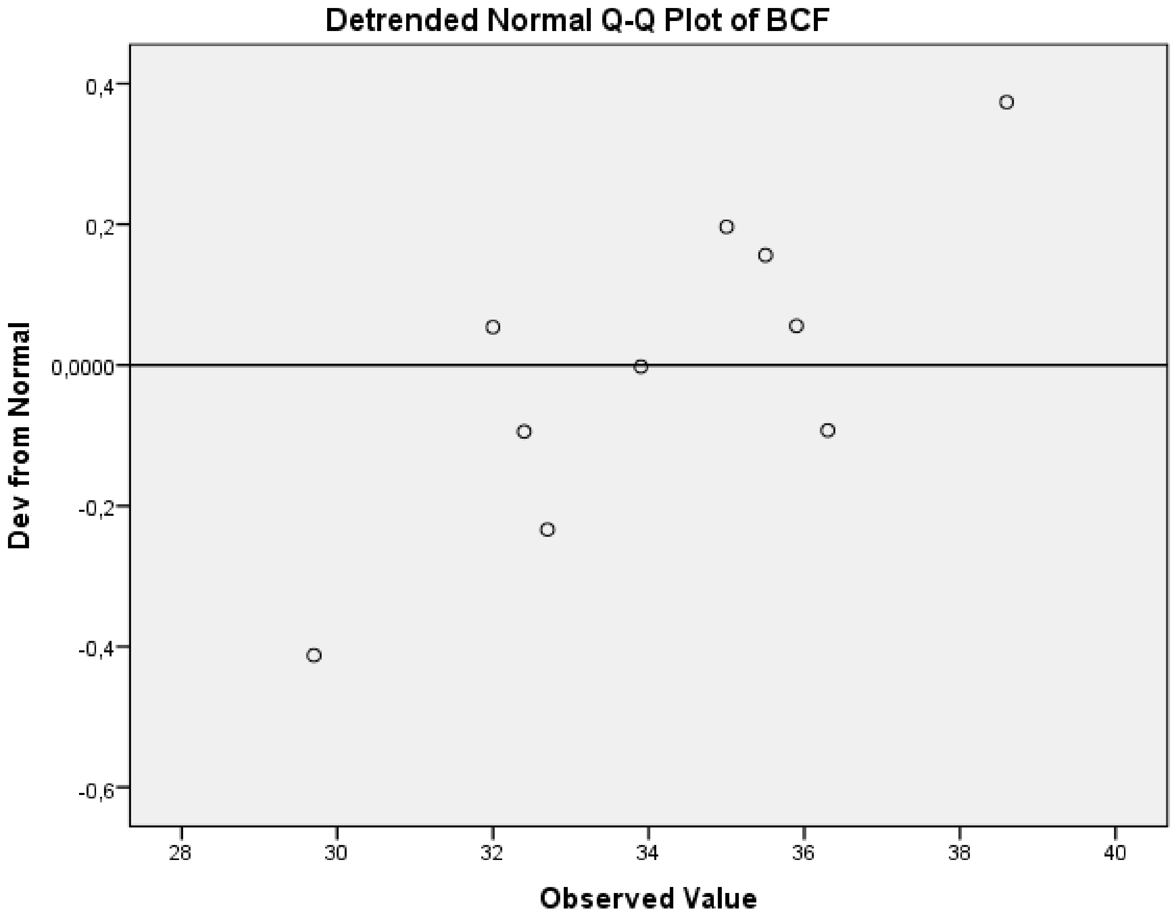
Detrended Normal Q-Q Plot of BCF
Dev from Normal
0,4
0,2
0,0000
-0,2
-0,4
-0,6
28
30
32
34
36
38
40
Observed Value

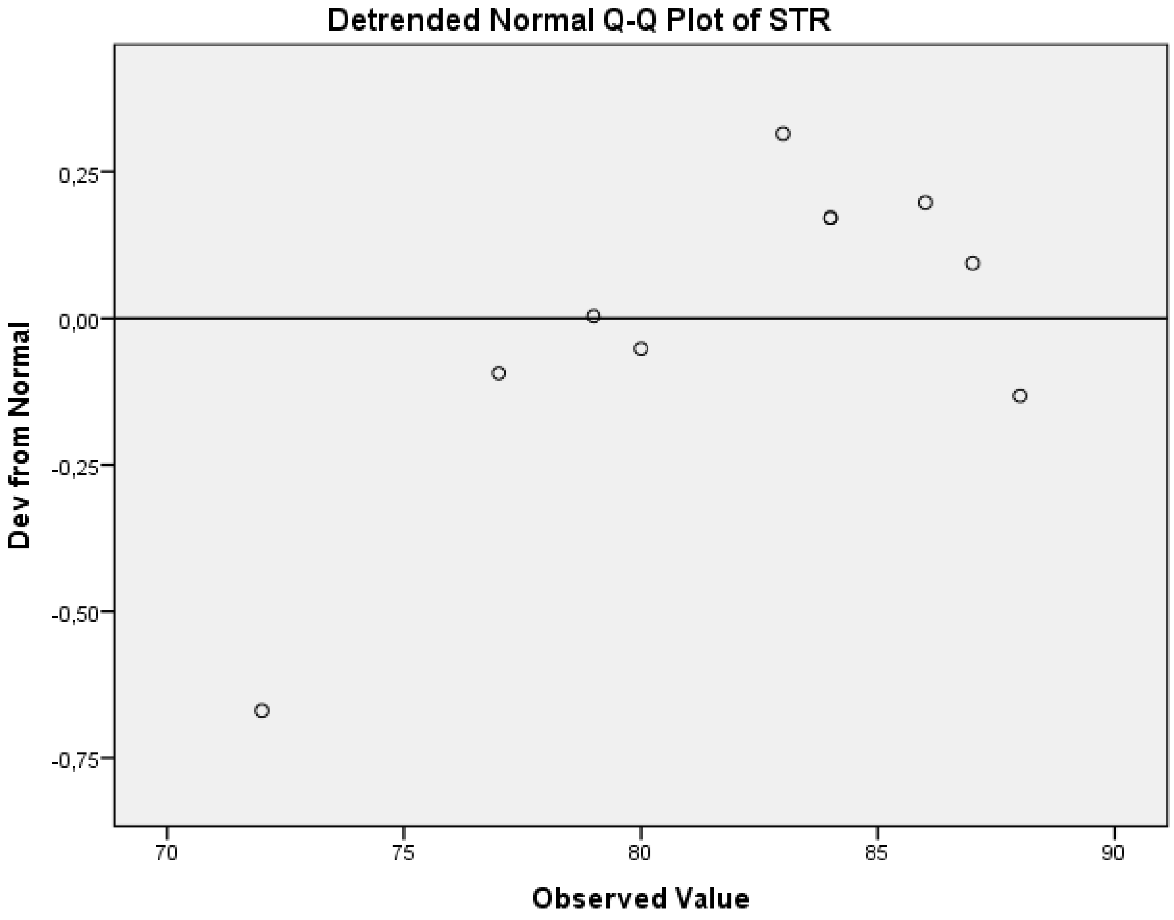

Detrended Normal Q-Q Plot of STR
Dev from Normal
0,25
0,00
-0,25
-0,50
-0,75
70
75
80
85
90
Observed Value

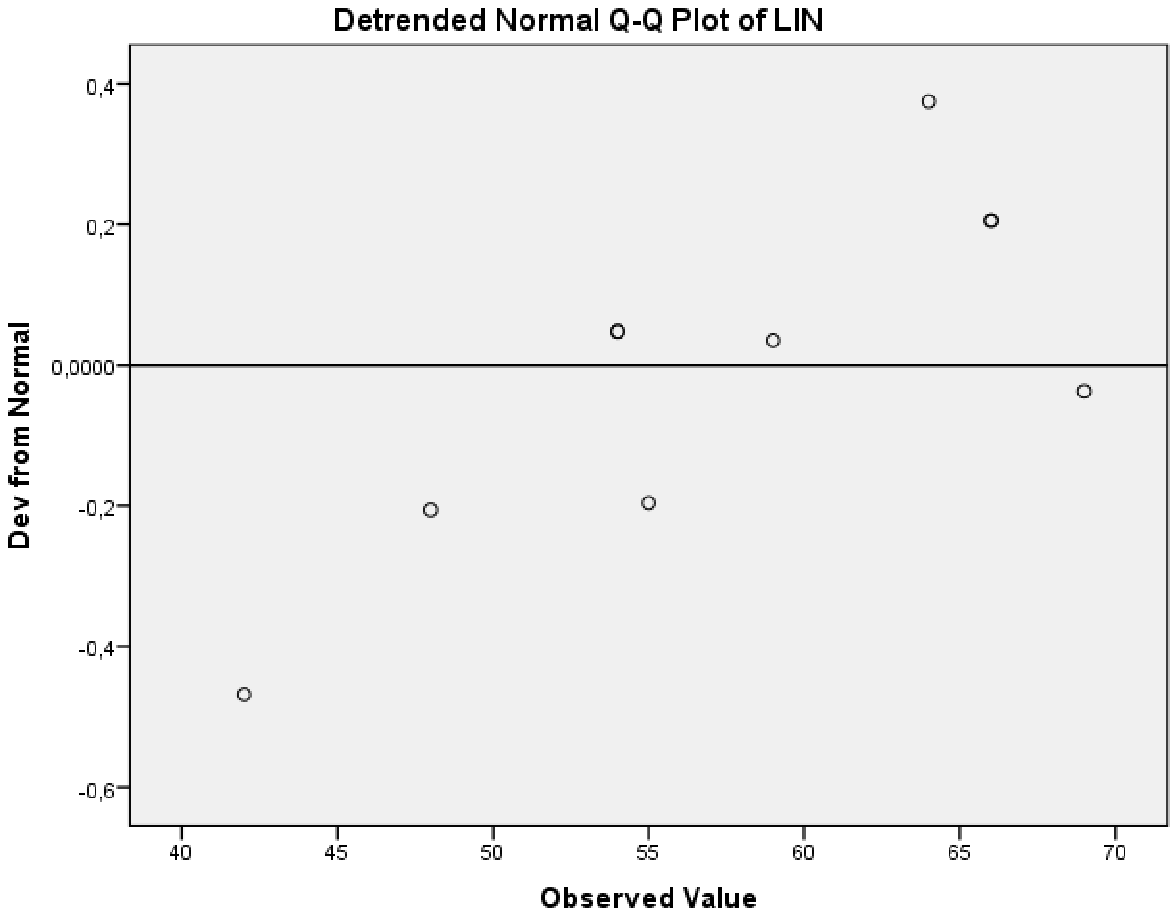
Detrended Normal Q-Q Plot of LIN
Dev from Normal
Observed Value
0,4
0,2
0,0000
-0,2
-0,4
-0,6
40
45
50
55
60
65
70

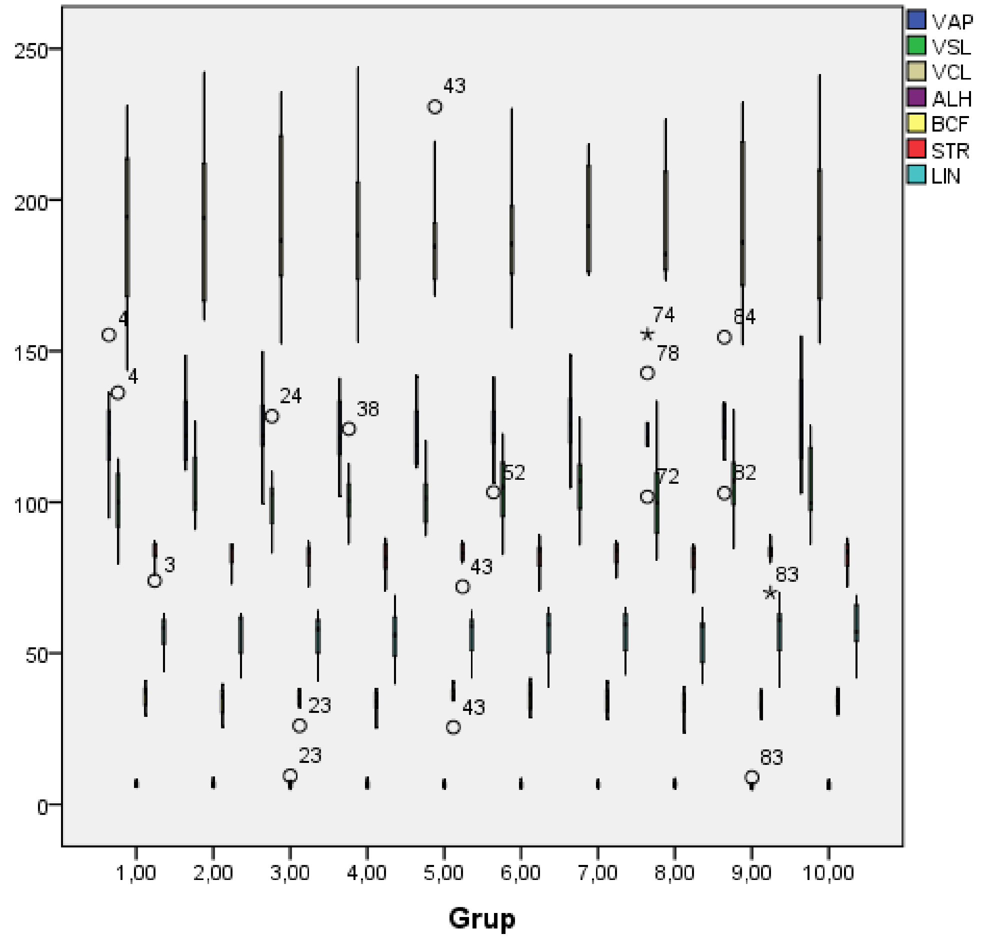


```
ONEWAY VAP VSL VCL ALH BCF STR LIN BY Grup
  /STATISTICS DESCRIPTIVES HOMOGENEITY
  /PLOT MEANS
  /MISSING ANALYSIS
  /POSTHOC=DUNCAN ALPHA(0.05).
```

Oneway

Notes

Output Created		22-DEC-2020 21:42:58
Comments		
Input	Active Dataset	DataSet0
	Filter	<none>
	Weight	<none>

	Split File	<none>
	N of Rows in Working Data File	100
Missing Value Handling	Definition of Missing	User-defined missing values are treated as missing.
	Cases Used	Statistics for each analysis are based on cases with no missing data for any variable in the analysis.
Syntax		ONEWAY VAP VSL VCL ALH BCF STR LIN BY Grup<br>/STATISTICS DESCRIPTIVES HOMOGENEITY<br>/PLOT MEANS<br>/MISSING ANALYSIS<br>/POSTHOC=DUNCAN ALPHA(0.05).
Resources	Processor Time	00:00:01,53
	Elapsed Time	00:00:01,97

Descriptives

						95% Confidence Interval for Mean			
		N	Mean	Std. Deviation	Std. Error	Lower Bound	Upper Bound	Minimum	Maximum
VAP	1,00	10	122,5800	16,67252	5,27231	110,6532	134,5068	94,80	155,30
	2,00	10	124,8700	12,67860	4,00932	115,8003	133,9397	110,70	148,40
	3,00	10	123,9100	13,20542	4,17592	114,4634	133,3566	99,60	149,70
	4,00	10	123,3700	11,70081	3,70012	114,9997	131,7403	102,10	140,90
	5,00	10	122,3800	11,17058	3,53245	114,3891	130,3709	111,40	141,90
	6,00	10	123,2600	12,31000	3,89276	114,4540	132,0660	103,30	141,40
	7,00	10	126,2300	12,23102	3,86779	117,4805	134,9795	104,80	148,80
	8,00	10	124,9700	14,65682	4,63489	114,4851	135,4549	101,80	155,50

	9,00	10	126,6300	13,50400	4,27034	116,9698	136,2902	103,00	154,50
	10,00	10	126,9600	16,34477	5,16867	115,2677	138,6523	102,90	154,80
	Total	100	124,5160	13,03265	1,30326	121,9300	127,1020	94,80	155,50
VSL	1,00	10	102,2500	15,58869	4,92958	91,0985	113,4015	79,60	136,20
	2,00	10	104,0300	11,50266	3,63746	95,8015	112,2585	91,00	126,70
	3,00	10	102,0200	12,14915	3,84190	93,3290	110,7110	83,30	128,40
	4,00	10	101,6000	11,27879	3,56667	93,5316	109,6684	86,30	124,20
	5,00	10	102,0100	9,96265	3,15047	94,8832	109,1368	89,00	120,30
	6,00	10	103,2500	12,46803	3,94274	94,3309	112,1691	82,70	122,40
	7,00	10	105,9800	13,05236	4,12752	96,6429	115,3171	85,90	127,80
	8,00	10	102,4600	16,10550	5,09301	90,9388	113,9812	81,10	133,30
	9,00	10	105,0500	13,44794	4,25261	95,4299	114,6701	84,70	130,50
	10,00	10	104,5600	14,46468	4,57414	94,2126	114,9074	86,00	125,30
	Total	100	103,3210	12,60335	1,26034	100,8202	105,8218	79,60	136,20
VCL	1,00	10	191,8500	28,74761	9,09079	171,2852	212,4148	143,70	231,10
	2,00	10	191,7800	26,13749	8,26540	173,0824	210,4776	160,30	242,10
	3,00	10	192,4900	26,09540	8,25209	173,8225	211,1575	152,50	235,50
	4,00	10	192,0900	25,51842	8,06963	173,8352	210,3448	152,90	243,70
	5,00	10	189,4600	20,36709	6,44064	174,8903	204,0297	168,30	230,80
	6,00	10	189,7400	21,42876	6,77637	174,4108	205,0692	157,70	230,10
	7,00	10	192,9300	16,58380	5,24426	181,0667	204,7933	175,10	218,30

	8,00	10	191,9900	19,71787	6,23534	177,8847	206,0953	173,40	226,50
	9,00	10	190,8800	25,75659	8,14495	172,4548	209,3052	152,10	232,20
	10,00	10	192,4400	30,15955	9,53729	170,8652	214,0148	152,60	241,30
	Total	100	191,5650	23,28918	2,32892	186,9439	196,1861	143,70	243,70
ALH	1,00	10	6,5600	,90333	,28566	5,9138	7,2062	5,70	8,30
	2,00	10	6,5400	1,05325	,33307	5,7866	7,2934	5,50	9,00
	3,00	10	6,6400	1,28599	,40667	5,7201	7,5599	5,10	9,40
	4,00	10	6,5400	1,13353	,35845	5,7291	7,3509	5,50	8,90
	5,00	10	6,4000	,91287	,28868	5,7470	7,0530	5,40	8,30
	6,00	10	6,4200	1,11435	,35239	5,6228	7,2172	5,50	8,70
	7,00	10	6,4400	,91918	,29067	5,7825	7,0975	5,30	8,30
	8,00	10	6,5200	,98860	,31262	5,8128	7,2272	5,30	8,60
	9,00	10	6,1800	1,17360	,37112	5,3405	7,0195	4,90	8,90
	10,00	10	6,3600	1,10474	,34935	5,5697	7,1503	5,10	8,30
	Total	100	6,4600	1,02376	,10238	6,2569	6,6631	4,90	9,40
BCF	1,00	10	35,9300	3,62769	1,14718	33,3349	38,5251	29,50	40,80
	2,00	10	34,5400	4,39677	1,39038	31,3947	37,6853	25,70	39,80
	3,00	10	34,7800	3,73000	1,17953	32,1117	37,4483	26,00	38,30
	4,00	10	33,8400	4,06590	1,28575	30,9314	36,7486	25,30	38,30
	5,00	10	35,3800	4,03727	1,27670	32,4919	38,2681	25,50	40,70
	6,00	10	35,5400	4,51152	1,42667	32,3127	38,7673	28,70	41,80
	7,00	10	34,8500	4,16740	1,31785	31,8688	37,8312	28,20	40,70
	8,00	10	33,0500	4,34211	1,37309	29,9438	36,1562	23,80	38,90
	9,00	10	33,6400	3,33806	1,05559	31,2521	36,0279	28,20	38,10
	10,00	10	34,2000	2,57466	,81418	32,3582	36,0418	29,70	38,60
	Total	100	34,5750	3,83549	,38355	33,8140	35,3360	23,80	41,80
STR	1,00	10	82,1000	4,22821	1,33708	79,0753	85,1247	74,00	87,00
	2,00	10	82,4000	5,05964	1,60000	78,7805	86,0195	73,00	86,00
	3,00	10	81,6000	4,78888	1,51438	78,1742	85,0258	72,00	87,00
	4,00	10	81,4000	5,01553	1,58605	77,8121	84,9879	71,00	88,00
	5,00	10	82,4000	4,37671	1,38404	79,2691	85,5309	72,00	87,00
	6,00	10	82,5000	5,33854	1,68819	78,6810	86,3190	71,00	89,00
	7,00	10	82,7000	4,16467	1,31698	79,7208	85,6792	75,00	87,00
	8,00	10	80,9000	5,32186	1,68292	77,0930	84,7070	70,00	86,00
	9,00	10	82,6000	5,08156	1,60693	78,9649	86,2351	70,00	89,00
	10,00	10	82,0000	4,98888	1,57762	78,4312	85,5688	72,00	88,00
	Total	100	82,0600	4,66195	,46619	81,1350	82,9850	70,00	89,00

LIN	1,00	10	56,1000	6,27960	1,98578	51,6078	60,5922	44,00	63,00
	2,00	10	57,0000	7,85988	2,48551	51,3774	62,6226	42,00	63,00
	3,00	10	55,9000	7,23341	2,28741	50,7255	61,0745	41,00	64,00
	4,00	10	55,5000	8,50163	2,68845	49,4183	61,5817	40,00	69,00
	5,00	10	56,4000	6,78561	2,14580	51,5459	61,2541	42,00	64,00
	6,00	10	56,6000	8,46168	2,67582	50,5469	62,6531	39,00	65,00
	7,00	10	57,1000	7,29459	2,30675	51,8818	62,3182	43,00	65,00
	8,00	10	55,5000	8,38318	2,65100	49,5030	61,4970	40,00	65,00
	9,00	10	58,3000	8,90755	2,81681	51,9279	64,6721	39,00	70,00
	10,00	10	57,7000	8,70568	2,75298	51,4723	63,9277	42,00	69,00
	Total	100	56,6100	7,57174	,75717	55,1076	58,1124	39,00	70,00

Test of Homogeneity of Variances

	Levene Statistic	df1	df2	Sig.
VAP	,332	9	90	,962
VSL	,447	9	90	,905
VCL	,678	9	90	,727
ALH	,320	9	90	,967
BCF	,551	9	90	,833
STR	,289	9	90	,976
LIN	,320	9	90	,966

ANOVA

		Sum of Squares	df	Mean Square	F	Sig.
VAP	Between Groups	252,800	9	28,089	,153	,998
	Within Groups	16562,334	90	184,026		
	Total	16815,134	99			
VSL	Between Groups	203,641	9	22,627	,131	,999
	Within Groups	15521,965	90	172,466		
	Total	15725,606	99			
VCL	Between Groups	122,991	9	13,666	,023	1,000
	Within Groups	53573,217	90	595,258		
	Total	53696,208	99			
ALH	Between Groups	1,528	9	,170	,149	,998
	Within Groups	102,232	90	1,136		
	Total	103,760	99			
BCF	Between Groups	74,149	9	8,239	,536	,844

	Within Groups	1382,239	90	15,358		
	Total	1456,388	99			
STR	Between Groups	31,240	9	3,471	,147	,998
	Within Groups	2120,400	90	23,560		
	Total	2151,640	99			
LIN	Between Groups	77,090	9	8,566	,138	,998
	Within Groups	5598,700	90	62,208		
	Total	5675,790	99			

Post Hoc Tests

Homogeneous Subsets

VAP

Duncan[a]

Grup	N	Subset for alpha = 0.05
		1
5,00	10	122,3800
1,00	10	122,5800
6,00	10	123,2600
4,00	10	123,3700
3,00	10	123,9100
2,00	10	124,8700
8,00	10	124,9700
7,00	10	126,2300
9,00	10	126,6300
10,00	10	126,9600
Sig.		,536

Means for groups in homogeneous subsets are displayed.

a. Uses Harmonic Mean Sample Size = 10,000.

VSL

Duncan[a]

Grup	N	Subset for alpha = 0.05<br>1
4,00	10	101,6000
5,00	10	102,0100
3,00	10	102,0200
1,00	10	102,2500
8,00	10	102,4600
6,00	10	103,2500
2,00	10	104,0300
10,00	10	104,5600
9,00	10	105,0500
7,00	10	105,9800
Sig.		,541

Means for groups in homogeneous subsets are displayed.

a. Uses Harmonic Mean Sample Size = 10,000.

VCL

Duncan[a]

Grup	N	Subset for alpha = 0.05<br>1
5,00	10	189,4600
6,00	10	189,7400
9,00	10	190,8800
2,00	10	191,7800
1,00	10	191,8500
8,00	10	191,9900
4,00	10	192,0900
10,00	10	192,4400
3,00	10	192,4900
7,00	10	192,9300

Sig.		,794

Means for groups in homogeneous subsets are displayed.
a. Uses Harmonic Mean Sample Size = 10,000.

ALH

Duncan[a]

Grup	N	Subset for alpha = 0.05
		1
9,00	10	6,1800
10,00	10	6,3600
5,00	10	6,4000
6,00	10	6,4200
7,00	10	6,4400
8,00	10	6,5200
2,00	10	6,5400
4,00	10	6,5400
1,00	10	6,5600
3,00	10	6,6400
Sig.		,428

Means for groups in homogeneous subsets are displayed.
a. Uses Harmonic Mean Sample Size = 10,000.

BCF

Duncan[a]

Grup	N	Subset for alpha = 0.05
		1
8,00	10	33,0500
9,00	10	33,6400
4,00	10	33,8400
10,00	10	34,2000
2,00	10	34,5400

3,00	10	34,7800
7,00	10	34,8500
5,00	10	35,3800
6,00	10	35,5400
1,00	10	35,9300
Sig.		,175

Means for groups in homogeneous subsets are displayed.

a. Uses Harmonic Mean Sample Size = 10,000.

STR

Duncan[a]

Grup	N	Subset for alpha = 0.05
		1
8,00	10	80,9000
4,00	10	81,4000
3,00	10	81,6000
10,00	10	82,0000
1,00	10	82,1000
2,00	10	82,4000
5,00	10	82,4000
6,00	10	82,5000
9,00	10	82,6000
7,00	10	82,7000
Sig.		,496

Means for groups in homogeneous subsets are displayed.

a. Uses Harmonic Mean Sample Size = 10,000.

LIN

Duncan[a]

Grup	N	Subset for alpha = 0.05
		1

4,00	10	55,5000
8,00	10	55,5000
3,00	10	55,9000
1,00	10	56,1000
5,00	10	56,4000
6,00	10	56,6000
2,00	10	57,0000
7,00	10	57,1000
10,00	10	57,7000
9,00	10	58,3000
Sig.		,515

Means for groups in homogeneous subsets are displayed.

a. Uses Harmonic Mean Sample Size = 10,000.

Means Plots

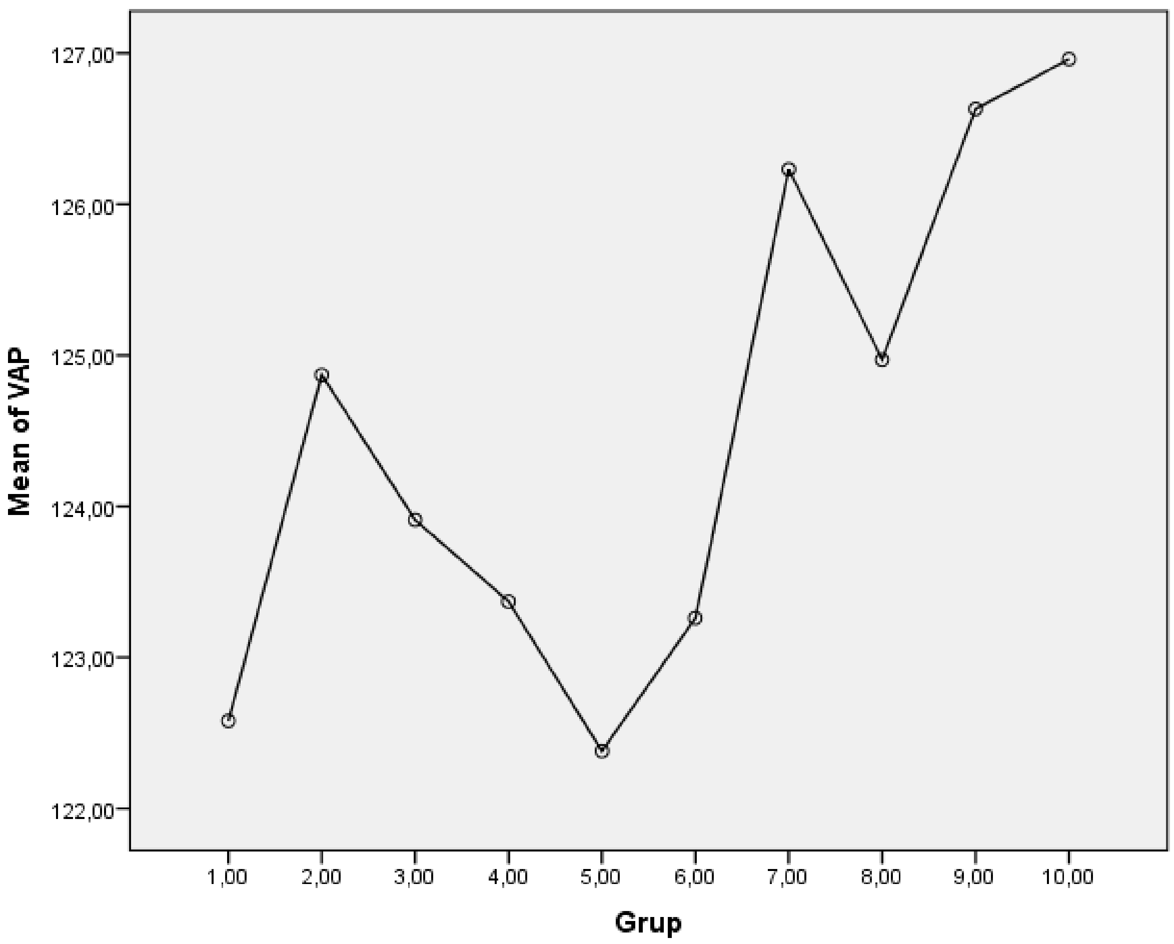

127,00
126,00
125,00
124,00
123,00
122,00
Mean of VAP
1,00
2,00
3,00
4,00
5,00
6,00
7,00
8,00
9,00
10,00
Grup

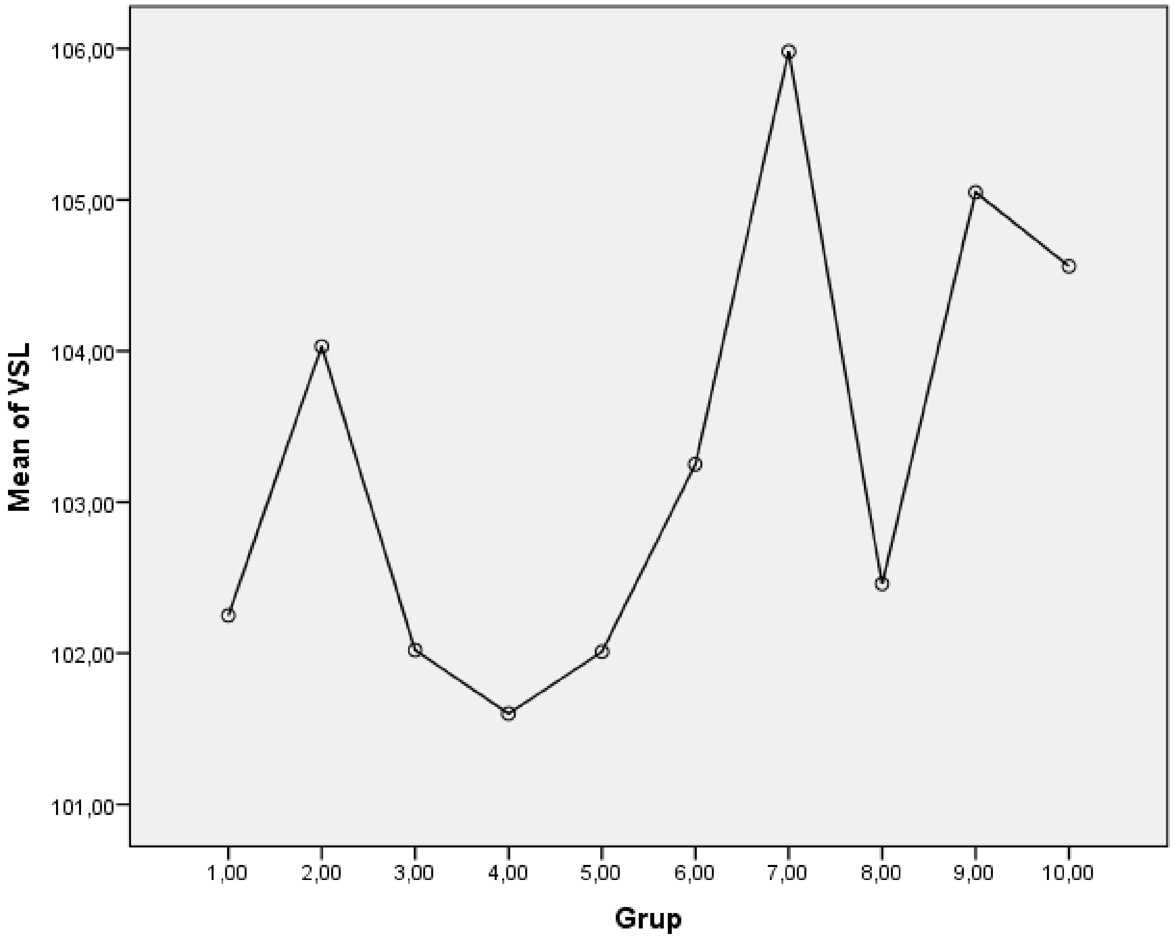
Mean of VSL
106,00
105,00
104,00
103,00
102,00
101,00
1,00
2,00
3,00
4,00
5,00
6,00
7,00
8,00
9,00
10,00
Grup

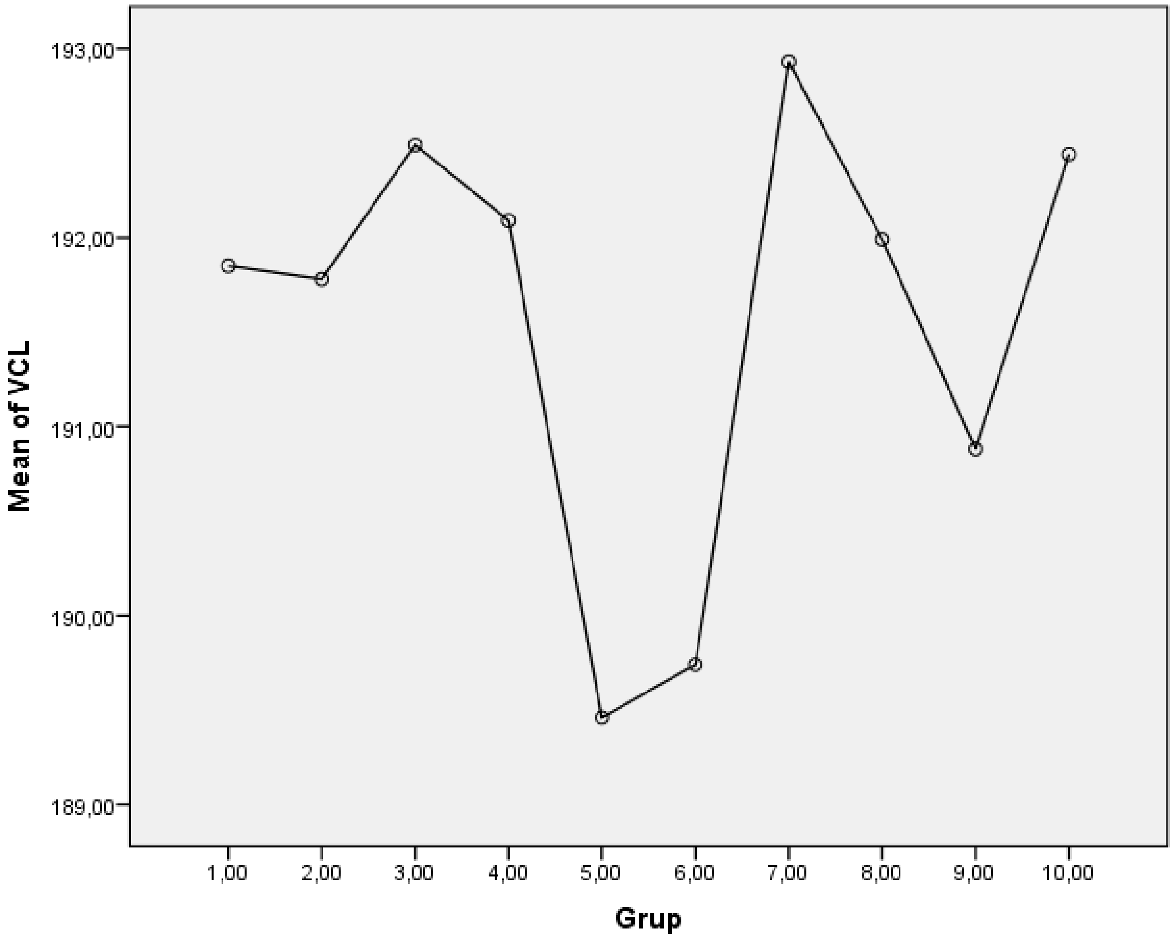

193,00
192,00
191,00
190,00
189,00
Mean of VCL
1,00
2,00
3,00
4,00
5,00
6,00
7,00
8,00
9,00
10,00
Grup

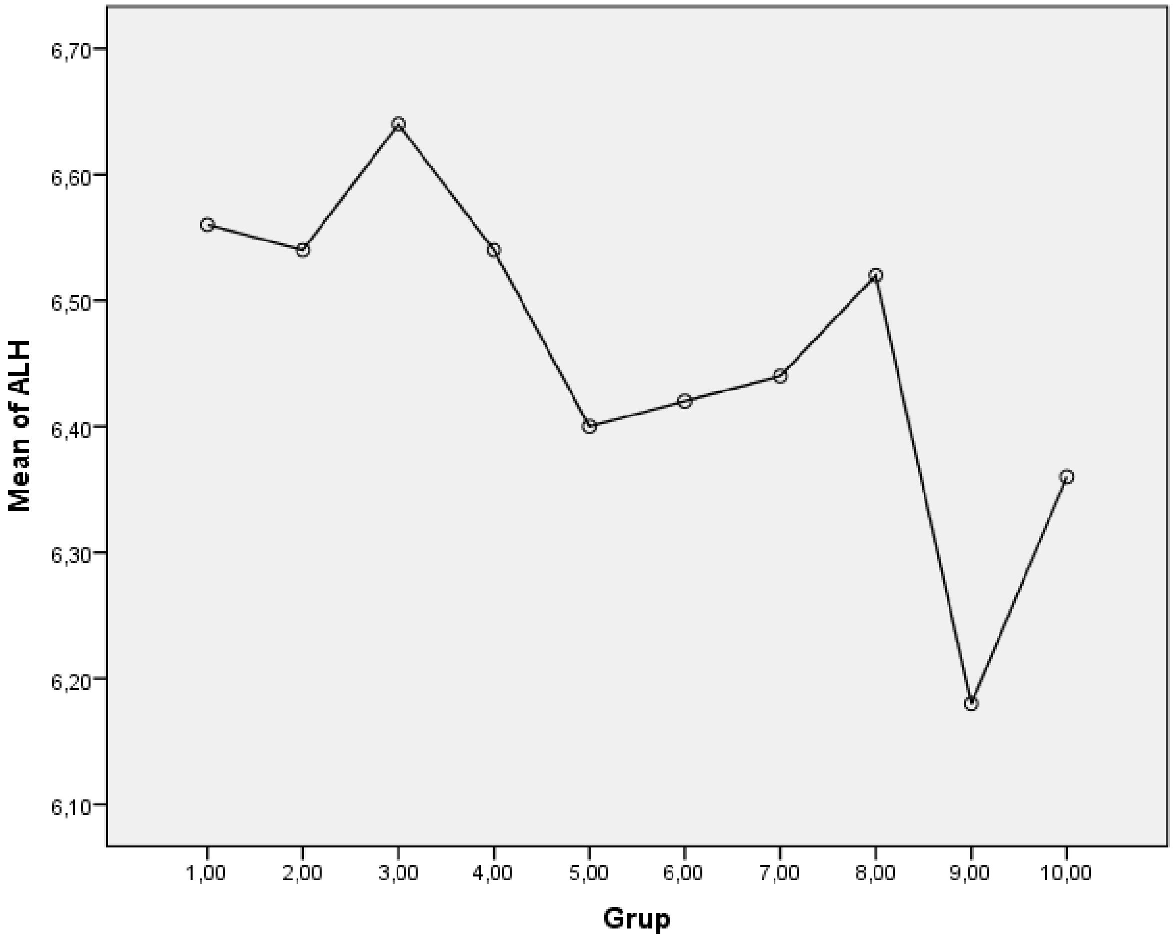

Mean of ALH
6,70
6,60
6,50
6,40
6,30
6,20
6,10
1,00
2,00
3,00
4,00
5,00
6,00
7,00
8,00
9,00
10,00
Grup

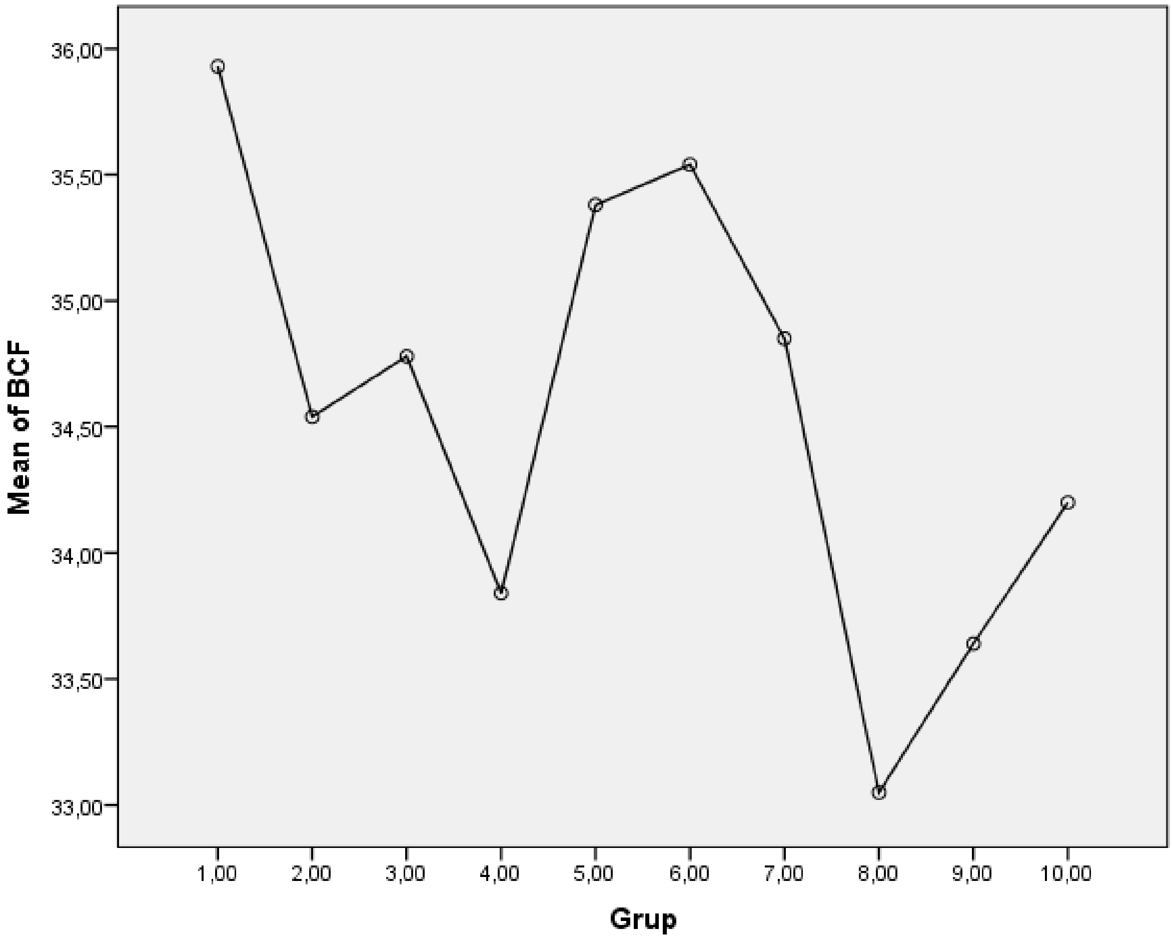
Mean of BCF
36,00
35,50
35,00
34,50
34,00
33,50
33,00
1,00
2,00
3,00
4,00
5,00
6,00
7,00
8,00
9,00
10,00
Grup

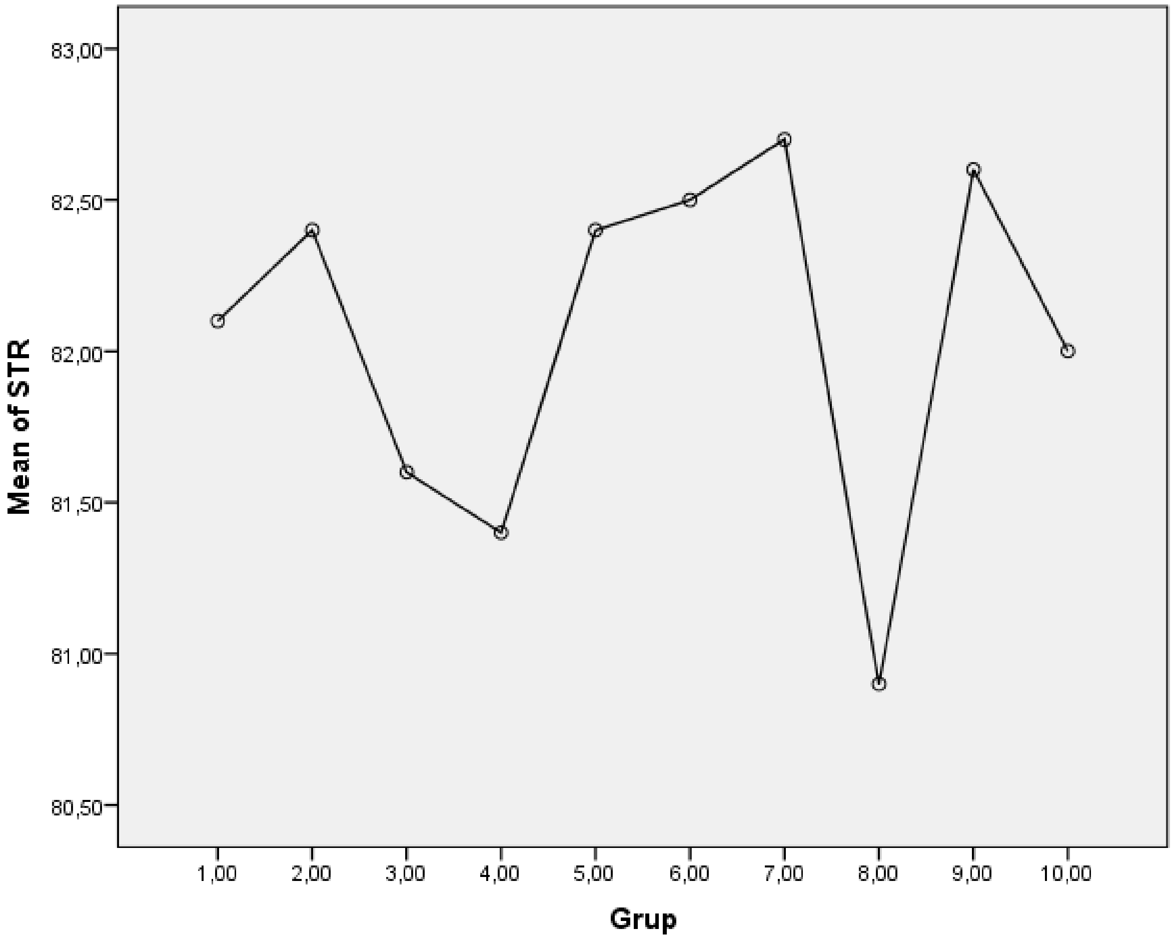

Mean of STR
83,00
82,50
82,00
81,50
81,00
80,50
1,00
2,00
3,00
4,00
5,00
6,00
7,00
8,00
9,00
10,00
Grup

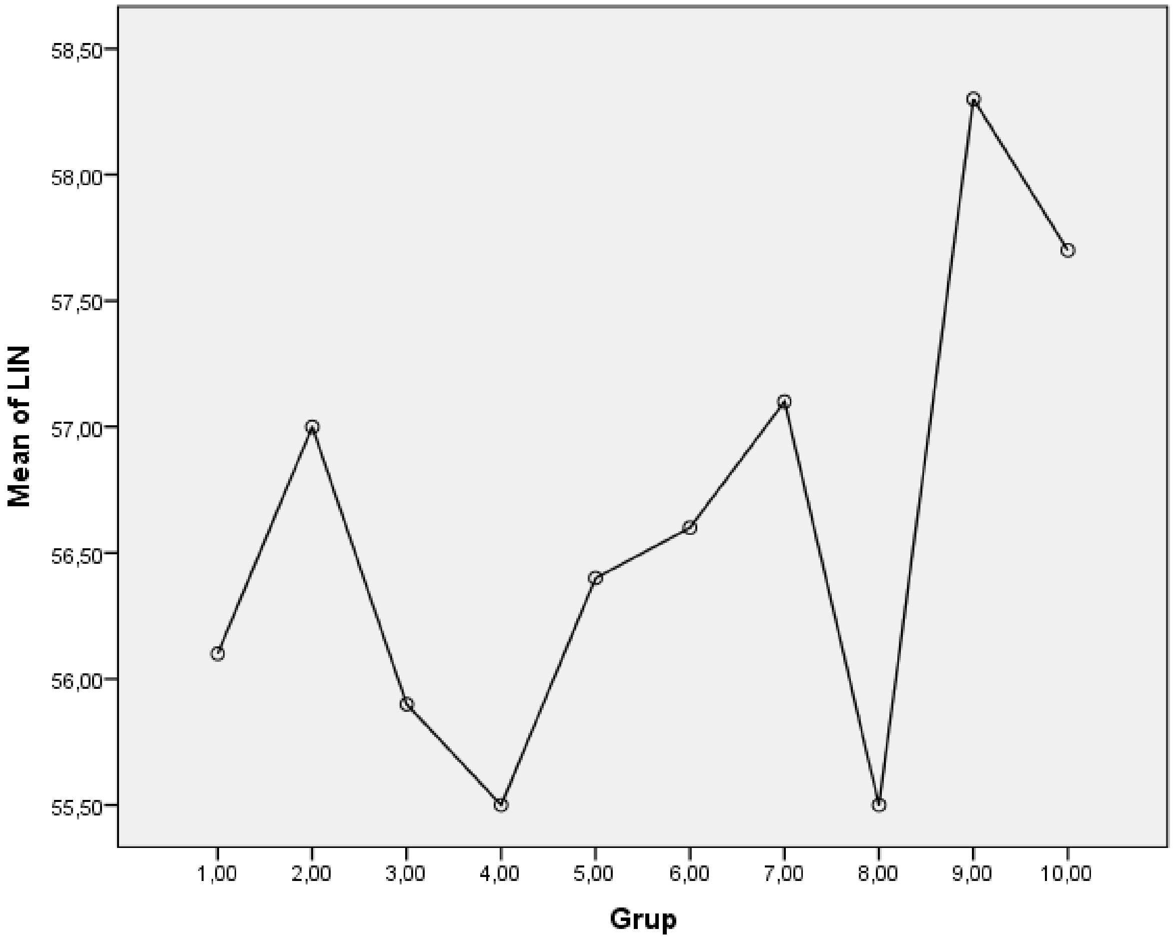

58,50
58,00
57,50
57,00
56,50
56,00
55,50
Mean of LIN
1,00
2,00
3,00
4,00
5,00
6,00
7,00
8,00
9,00
10,00
Grup

Sperm motility parameters of post-thawed ram sperm- ANOVA

CasaMot: Total motility- post-thaw ram sperm

ProgMot: Progresif motility- post-thaw ram sperm

Groups

Kontrol: Control

Res10: RES10

Res20: RES20

Res40: RES40

Sık10: CD10

Sık20: CD10

Sık40: CD40

Rlc10: RLC10

Rlc20: RLC20

Rlc40: RLC40

Explore

Notes

Output Created		22-DEC-2020 12:17:27
Comments		
Input	Active Dataset	DataSet0
	Filter	<none>
	Weight	<none>
	Split File	<none>
	N of Rows in Working Data File	200
Missing Value Handling	Definition of Missing	User-defined missing values for dependent variables are treated as missing.
	Cases Used	Statistics are based on cases with no missing values for any dependent variable or factor used.

Syntax		EXAMINE VARIABLES=SubjMot CasaMot ProgMot BY Grup<br>/PLOT BOXPLOT STEMLEAF HISTOGRAM NPPLOT<br>/COMPARE GROUPS<br>/STATISTICS DESCRIPTIVES<br>/CINTERVAL 95<br>/MISSING LISTWISE<br>/NOTOTAL.
Resources	Processor Time	00:00:10,88
	Elapsed Time	00:00:10,88

Grup

Case Processing Summary

		Cases					
		Valid		Missing		Total	
	Grup	N	Percent	N	Percent	N	Percent
SubjMot	kontrol	20	100,0%	0	0,0%	20	100,0%
	Res10	20	100,0%	0	0,0%	20	100,0%
	Res20	20	100,0%	0	0,0%	20	100,0%
	Res40	20	100,0%	0	0,0%	20	100,0%
	sık10	20	100,0%	0	0,0%	20	100,0%
	sık20	20	100,0%	0	0,0%	20	100,0%
	sık40	20	100,0%	0	0,0%	20	100,0%
	Rlc10	20	100,0%	0	0,0%	20	100,0%
	Rlc20	20	100,0%	0	0,0%	20	100,0%
	Rlc40	20	100,0%	0	0,0%	20	100,0%
CasaMot	kontrol	20	100,0%	0	0,0%	20	100,0%
	Res10	20	100,0%	0	0,0%	20	100,0%
	Res20	20	100,0%	0	0,0%	20	100,0%
	Res40	20	100,0%	0	0,0%	20	100,0%
	sık10	20	100,0%	0	0,0%	20	100,0%
	sık20	20	100,0%	0	0,0%	20	100,0%
	sık40	20	100,0%	0	0,0%	20	100,0%
	Rlc10	20	100,0%	0	0,0%	20	100,0%

	Rlc20	20	100,0%	0	0,0%	20	100,0%
	Rlc40	20	100,0%	0	0,0%	20	100,0%
ProgMot	kontrol	20	100,0%	0	0,0%	20	100,0%
	Res10	20	100,0%	0	0,0%	20	100,0%
	Res20	20	100,0%	0	0,0%	20	100,0%
	Res40	20	100,0%	0	0,0%	20	100,0%
	sık10	20	100,0%	0	0,0%	20	100,0%
	sık20	20	100,0%	0	0,0%	20	100,0%
	sık40	20	100,0%	0	0,0%	20	100,0%
	Rlc10	20	100,0%	0	0,0%	20	100,0%
	Rlc20	20	100,0%	0	0,0%	20	100,0%
	Rlc40	20	100,0%	0	0,0%	20	100,0%

Descriptives

	Grup			Statistic	Std. Error
SubjMot	kontrol	Mean		46,0000	1,93989
		95% Confidence Interval for Mean	Lower Bound	41,9398	
			Upper Bound	50,0602	
		5% Trimmed Mean		46,3889	
		Median		50,0000	
		Variance		75,263	
		Std. Deviation		8,67543	
		Minimum		30,00	
		Maximum		55,00	
		Range		25,00	
		Interquartile Range		17,50	
		Skewness		-,744	,512
		Kurtosis		-,850	,992
	Res10	Mean		46,5000	1,88833
		95% Confidence Interval for Mean	Lower Bound	42,5477	
			Upper Bound	50,4523	
		5% Trimmed Mean		46,9444	
		Median		50,0000	
		Variance		71,316	
		Std. Deviation		8,44487	
		Minimum		30,00	
		Maximum		55,00	

	Range		25,00	
	Interquartile Range		13,75	
	Skewness		-,890	,512
	Kurtosis		-,498	,992
Res20	Mean		50,5000	1,65831
	95% Confidence Interval for Mean	Lower Bound	47,0291	
		Upper Bound	53,9709	
	5% Trimmed Mean		51,1111	
	Median		50,0000	
	Variance		55,000	
	Std. Deviation		7,41620	
	Minimum		30,00	
	Maximum		60,00	
	Range		30,00	
	Interquartile Range		5,00	
	Skewness		-1,372	,512
	Kurtosis		2,701	,992
Res40	Mean		48,2500	1,55068
	95% Confidence Interval for Mean	Lower Bound	45,0044	
		Upper Bound	51,4956	
	5% Trimmed Mean		48,6111	
	Median		50,0000	
	Variance		48,092	
	Std. Deviation		6,93485	
	Minimum		30,00	
	Maximum		60,00	
	Range		30,00	
	Interquartile Range		5,00	
	Skewness		-1,135	,512
	Kurtosis		1,836	,992
sık10	Mean		50,0000	1,84961
	95% Confidence Interval for Mean	Lower Bound	46,1287	
		Upper Bound	53,8713	
	5% Trimmed Mean		50,5556	
	Median		50,0000	
	Variance		68,421	
	Std. Deviation		8,27170	
	Minimum		30,00	

	Maximum		60,00	
	Range		30,00	
	Interquartile Range		5,00	
	Skewness		-1,395	,512
	Kurtosis		2,055	,992
sık20	Mean		46,7500	2,15440
	95% Confidence Interval for Mean	Lower Bound	42,2408	
		Upper Bound	51,2592	
	5% Trimmed Mean		47,2222	
	Median		50,0000	
	Variance		92,829	
	Std. Deviation		9,63478	
	Minimum		25,00	
	Maximum		60,00	
	Range		35,00	
	Interquartile Range		13,75	
	Skewness		-,506	,512
	Kurtosis		-,272	,992
sık40	Mean		41,5000	2,56751
	95% Confidence Interval for Mean	Lower Bound	36,1261	
		Upper Bound	46,8739	
	5% Trimmed Mean		41,6667	
	Median		42,5000	
	Variance		131,842	
	Std. Deviation		11,48225	
	Minimum		20,00	
	Maximum		60,00	
	Range		40,00	
	Interquartile Range		20,00	
	Skewness		-,235	,512
	Kurtosis		-1,144	,992
Rlc10	Mean		47,0000	1,93309
	95% Confidence Interval for Mean	Lower Bound	42,9540	
		Upper Bound	51,0460	
	5% Trimmed Mean		47,2222	
	Median		50,0000	
	Variance		74,737	
	Std. Deviation		8,64505	

		Minimum		30,00	
		Maximum		60,00	
		Range		30,00	
		Interquartile Range		13,75	
		Skewness		-,491	,512
		Kurtosis		-,318	,992
	Rlc20	Mean		48,1000	1,40656
		95% Confidence Interval for Mean	Lower Bound	45,1560	
			Upper Bound	51,0440	
		5% Trimmed Mean		48,1667	
		Median		50,0000	
		Variance		39,568	
		Std. Deviation		6,29034	
		Minimum		35,00	
		Maximum		60,00	
		Range		25,00	
		Interquartile Range		5,00	
		Skewness		-,202	,512
		Kurtosis		-,139	,992
	Rlc40	Mean		46,5000	1,74341
		95% Confidence Interval for Mean	Lower Bound	42,8510	
			Upper Bound	50,1490	
		5% Trimmed Mean		46,6667	
		Median		47,5000	
		Variance		60,789	
		Std. Deviation		7,79676	
		Minimum		30,00	
		Maximum		60,00	
		Range		30,00	
		Interquartile Range		5,00	
		Skewness		-,557	,512
		Kurtosis		-,091	,992
CasaMot	kontrol	Mean		59,6500	3,66295
		95% Confidence Interval for Mean	Lower Bound	51,9833	
			Upper Bound	67,3167	
		5% Trimmed Mean		60,0000	
		Median		66,5000	
		Variance		268,345	
		Std. Deviation		16,38123	

	Minimum		30,00	
	Maximum		83,00	
	Range		53,00	
	Interquartile Range		26,00	
	Skewness		-,627	,512
	Kurtosis		-,912	,992
Res10	Mean		57,2500	3,07783
	95% Confidence Interval for Mean	Lower Bound	50,8080	
		Upper Bound	63,6920	
	5% Trimmed Mean		57,6111	
	Median		63,0000	
	Variance		189,461	
	Std. Deviation		13,76447	
	Minimum		33,00	
	Maximum		75,00	
	Range		42,00	
	Interquartile Range		20,50	
	Skewness		-,583	,512
	Kurtosis		-,902	,992
Res20	Mean		62,6500	3,26567
	95% Confidence Interval for Mean	Lower Bound	55,8149	
		Upper Bound	69,4851	
	5% Trimmed Mean		62,7778	
	Median		65,5000	
	Variance		213,292	
	Std. Deviation		14,60452	
	Minimum		36,00	
	Maximum		87,00	
	Range		51,00	
	Interquartile Range		18,75	
	Skewness		-,297	,512
	Kurtosis		-,560	,992
Res40	Mean		60,9000	2,56792
	95% Confidence Interval for Mean	Lower Bound	55,5253	
		Upper Bound	66,2747	
	5% Trimmed Mean		61,6111	
	Median		62,0000	
	Variance		131,884	
	Std. Deviation		11,48409	
	Minimum		28,00	
	Maximum		81,00	

	Range		53,00	
	Interquartile Range		10,50	
	Skewness		-1,069	,512
	Kurtosis		2,708	,992
sık10	Mean		64,3500	2,64503
	95% Confidence Interval for Mean	Lower Bound	58,8139	
		Upper Bound	69,8861	
	5% Trimmed Mean		64,8889	
	Median		66,0000	
	Variance		139,924	
	Std. Deviation		11,82893	
	Minimum		35,00	
	Maximum		84,00	
	Range		49,00	
	Interquartile Range		14,25	
	Skewness		-,903	,512
	Kurtosis		1,056	,992
sık20	Mean		52,5000	2,97489
	95% Confidence Interval for Mean	Lower Bound	46,2735	
		Upper Bound	58,7265	
	5% Trimmed Mean		52,3889	
	Median		52,5000	
	Variance		177,000	
	Std. Deviation		13,30413	
	Minimum		29,00	
	Maximum		78,00	
	Range		49,00	
	Interquartile Range		20,50	
	Skewness		-,059	,512
	Kurtosis		-,710	,992
sık40	Mean		51,5000	3,70242
	95% Confidence Interval for Mean	Lower Bound	43,7508	
		Upper Bound	59,2492	
	5% Trimmed Mean		51,7222	
	Median		50,5000	
	Variance		274,158	
	Std. Deviation		16,55771	
	Minimum		23,00	
	Maximum		76,00	
	Range		53,00	
	Interquartile Range		28,25	

	Skewness		-,098	,512
	Kurtosis		-1,171	,992
Rlc10	Mean		51,3000	2,62187
	95% Confidence Interval for Mean	Lower Bound	45,8124	
		Upper Bound	56,7876	
	5% Trimmed Mean		50,7222	
	Median		50,0000	
	Variance		137,484	
	Std. Deviation		11,72537	
	Minimum		34,00	
	Maximum		79,00	
	Range		45,00	
	Interquartile Range		19,00	
	Skewness		,552	,512
	Kurtosis		,162	,992
Rlc20	Mean		56,8500	2,96051
	95% Confidence Interval for Mean	Lower Bound	50,6536	
		Upper Bound	63,0464	
	5% Trimmed Mean		56,1111	
	Median		55,0000	
	Variance		175,292	
	Std. Deviation		13,23979	
	Minimum		41,00	
	Maximum		86,00	
	Range		45,00	
	Interquartile Range		22,25	
	Skewness		,522	,512
	Kurtosis		-,626	,992
Rlc40	Mean		57,0000	3,20033
	95% Confidence Interval for Mean	Lower Bound	50,3016	
		Upper Bound	63,6984	
	5% Trimmed Mean		56,8889	
	Median		59,0000	
	Variance		204,842	
	Std. Deviation		14,31231	
	Minimum		30,00	
	Maximum		86,00	
	Range		56,00	
	Interquartile Range		14,50	
	Skewness		,037	,512
	Kurtosis		-,064	,992

ProgMot	kontrol	Mean		29,1500	1,59485
		95% Confidence Interval for Mean	Lower Bound	25,8119	
			Upper Bound	32,4881	
		5% Trimmed Mean		29,1667	
		Median		28,5000	
		Variance		50,871	
		Std. Deviation		7,13239	
		Minimum		14,00	
		Maximum		44,00	
		Range		30,00	
		Interquartile Range		9,25	
		Skewness		,091	,512
		Kurtosis		,512	,992
	Res10	Mean		26,3000	1,90719
		95% Confidence Interval for Mean	Lower Bound	22,3082	
			Upper Bound	30,2918	
		5% Trimmed Mean		26,1667	
		Median		28,0000	
		Variance		72,747	
		Std. Deviation		8,52921	
		Minimum		11,00	
		Maximum		44,00	
		Range		33,00	
		Interquartile Range		9,75	
		Skewness		,172	,512
		Kurtosis		-,322	,992
	Res20	Mean		30,4500	1,23005
		95% Confidence Interval for Mean	Lower Bound	27,8755	
			Upper Bound	33,0245	
		5% Trimmed Mean		30,5000	
		Median		30,5000	
		Variance		30,261	
		Std. Deviation		5,50096	
		Minimum		20,00	
		Maximum		40,00	
		Range		20,00	
		Interquartile Range		8,00	
		Skewness		-,169	,512

	Kurtosis		-,595	,992
Res40	Mean		28,9000	1,60411
	95% Confidence Interval for Mean	Lower Bound	25,5426	
		Upper Bound	32,2574	
	5% Trimmed Mean		28,8333	
	Median		27,5000	
	Variance		51,463	
	Std. Deviation		7,17378	
	Minimum		17,00	
	Maximum		42,00	
	Range		25,00	
	Interquartile Range		10,75	
	Skewness		,415	,512
	Kurtosis		-,698	,992
sık10	Mean		32,6500	1,44964
	95% Confidence Interval for Mean	Lower Bound	29,6159	
		Upper Bound	35,6841	
	5% Trimmed Mean		32,7778	
	Median		33,5000	
	Variance		42,029	
	Std. Deviation		6,48297	
	Minimum		20,00	
	Maximum		43,00	
	Range		23,00	
	Interquartile Range		5,75	
	Skewness		-,152	,512
	Kurtosis		-,155	,992
sık20	Mean		28,9000	1,55072
	95% Confidence Interval for Mean	Lower Bound	25,6543	
		Upper Bound	32,1457	
	5% Trimmed Mean		29,1667	
	Median		28,5000	
	Variance		48,095	
	Std. Deviation		6,93504	
	Minimum		13,00	
	Maximum		40,00	
	Range		27,00	
	Interquartile Range		8,00	

	Skewness		-,632	,512
	Kurtosis		,596	,992
sık40	Mean		26,2000	2,24617
	95% Confidence Interval for Mean	Lower Bound	21,4987	
		Upper Bound	30,9013	
	5% Trimmed Mean		26,1111	
	Median		25,0000	
	Variance		100,905	
	Std. Deviation		10,04516	
	Minimum		9,00	
	Maximum		45,00	
	Range		36,00	
	Interquartile Range		16,75	
	Skewness		,399	,512
	Kurtosis		-,588	,992
Rlc10	Mean		26,7500	1,63655
	95% Confidence Interval for Mean	Lower Bound	23,3247	
		Upper Bound	30,1753	
	5% Trimmed Mean		26,5000	
	Median		27,0000	
	Variance		53,566	
	Std. Deviation		7,31887	
	Minimum		14,00	
	Maximum		44,00	
	Range		30,00	
	Interquartile Range		9,75	
	Skewness		,256	,512
	Kurtosis		,356	,992
Rlc20	Mean		26,7000	1,08361
	95% Confidence Interval for Mean	Lower Bound	24,4320	
		Upper Bound	28,9680	
	5% Trimmed Mean		26,4444	
	Median		26,0000	
	Variance		23,484	
	Std. Deviation		4,84605	
	Minimum		20,00	
	Maximum		38,00	
	Range		18,00	

				Statistic	Std. Error
		Interquartile Range		6,75	
		Skewness		,823	,512
		Kurtosis		,036	,992
	Rlc40	Mean		26,5500	1,64072
		95% Confidence Interval for Mean	Lower Bound	23,1159	
			Upper Bound	29,9841	
		5% Trimmed Mean		26,0000	
		Median		25,5000	
		Variance		53,839	
		Std. Deviation		7,33754	
		Minimum		18,00	
		Maximum		45,00	
		Range		27,00	
		Interquartile Range		12,00	
		Skewness		,838	,512
		Kurtosis		,308	,992

Tests of Normality

		Kolmogorov-Smirnov[a]			Shapiro-Wilk		
	Grup	Statistic	df	Sig.	Statistic	df	Sig.
SubjMot	kontrol	,278	20	,000	,843	20	,004
	Res10	,311	20	,000	,830	20	,002
	Res20	,323	20	,000	,813	20	,001
	Res40	,300	20	,000	,862	20	,009
	sık10	,350	20	,000	,789	20	,001
	sık20	,232	20	,006	,926	20	,127
	sık40	,220	20	,012	,930	20	,156
	Rlc10	,236	20	,005	,915	20	,080
	Rlc20	,219	20	,013	,944	20	,283
	Rlc40	,224	20	,010	,919	20	,093
CasaMot	kontrol	,216	20	,015	,891	20	,028
	Res10	,212	20	,019	,909	20	,062
	Res20	,114	20	,200*	,968	20	,703
	Res40	,217	20	,014	,922	20	,106
	sık10	,205	20	,027	,939	20	,228
	sık20	,114	20	,200*	,971	20	,768
	sık40	,125	20	,200*	,953	20	,416
	Rlc10	,132	20	,200*	,954	20	,437

	Rlc20	,144	20	,200*	,930	20	,156
	Rlc40	,115	20	,200*	,975	20	,856
ProgMot	kontrol	,130	20	,200*	,975	20	,862
	Res10	,143	20	,200*	,975	20	,850
	Res20	,117	20	,200*	,975	20	,846
	Res40	,144	20	,200*	,954	20	,426
	sık10	,168	20	,143	,933	20	,175
	sık20	,144	20	,200*	,927	20	,138
	sık40	,168	20	,140	,945	20	,292
	Rlc10	,087	20	,200*	,978	20	,909
	Rlc20	,161	20	,184	,928	20	,143
	Rlc40	,136	20	,200*	,907	20	,057

*. This is a lower bound of the true significance.

a. Lilliefors Significance Correction

SubjMot

Histograms

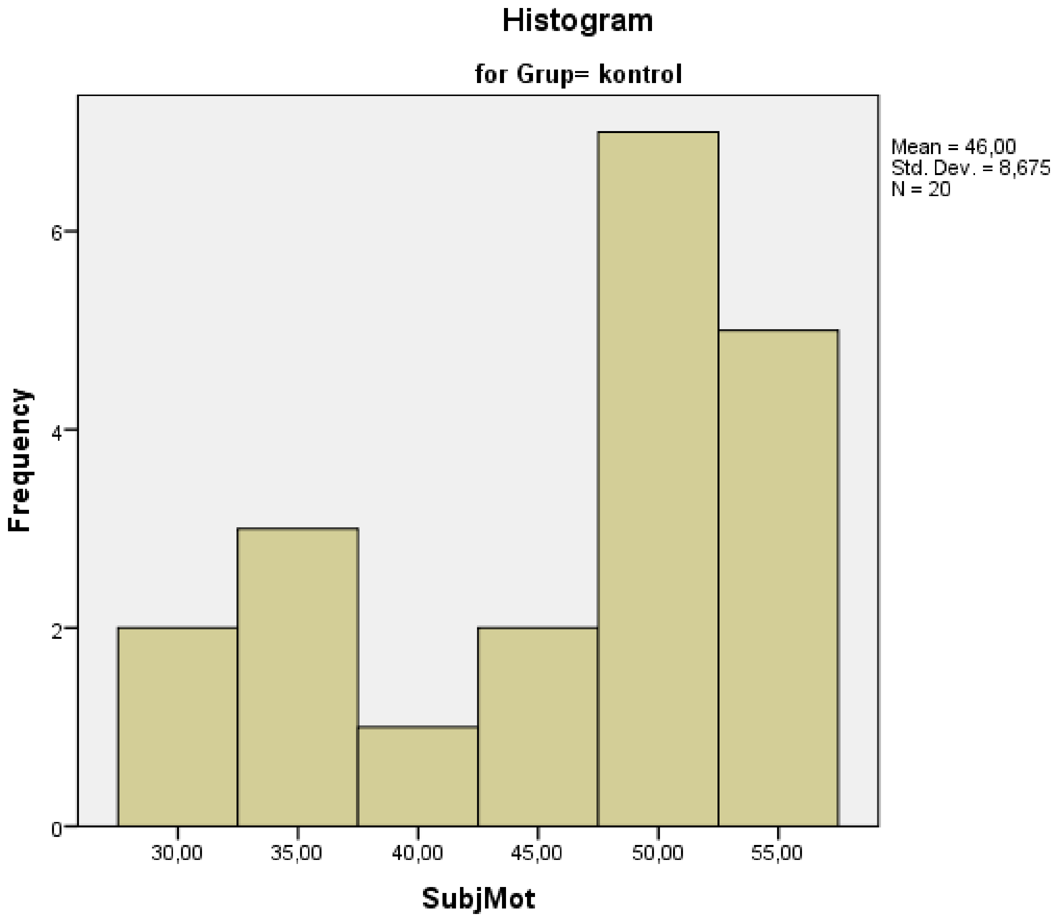
Histogram
for Grup= kontrol
Mean = 46,00
Std. Dev. = 8,675
N = 20
Frequency
0
2
4
6
30,00
35,00
40,00
45,00
50,00
55,00
SubjMot

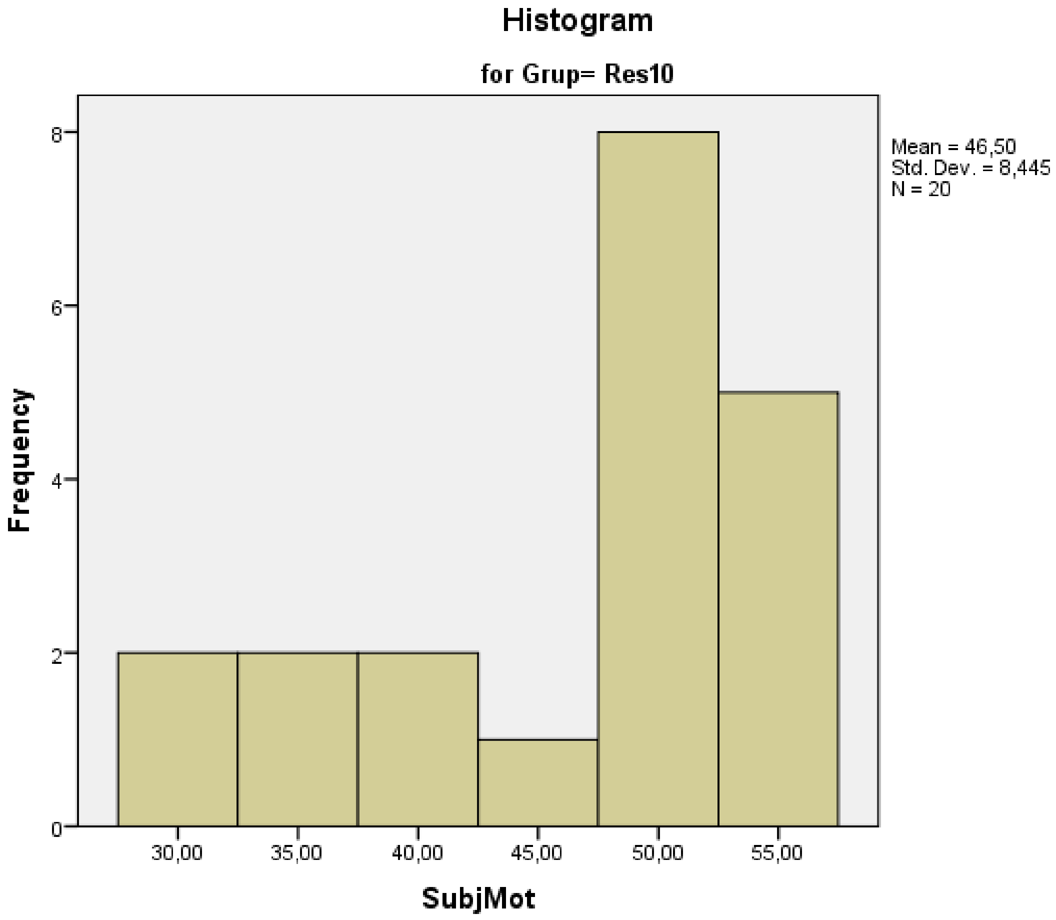

Histogram
for Grup= Res10
Mean = 46,50
Std. Dev. = 8,445
N = 20
Frequency
0
2
4
6
8
30,00
35,00
40,00
45,00
50,00
55,00
SubjMot

Histogram

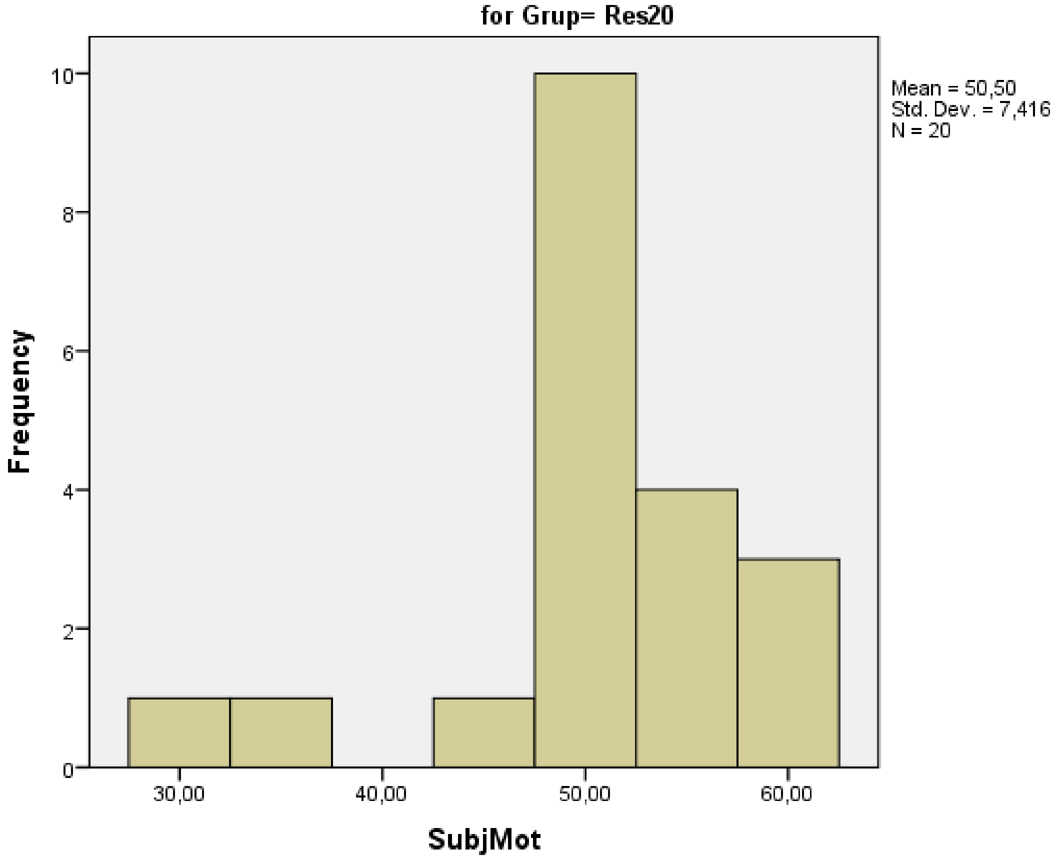

for Grup= Res20
Mean = 50,50
Std. Dev. = 7,416
N = 20
Frequency
0
2
4
6
8
10
30,00
40,00
50,00
60,00
SubjMot

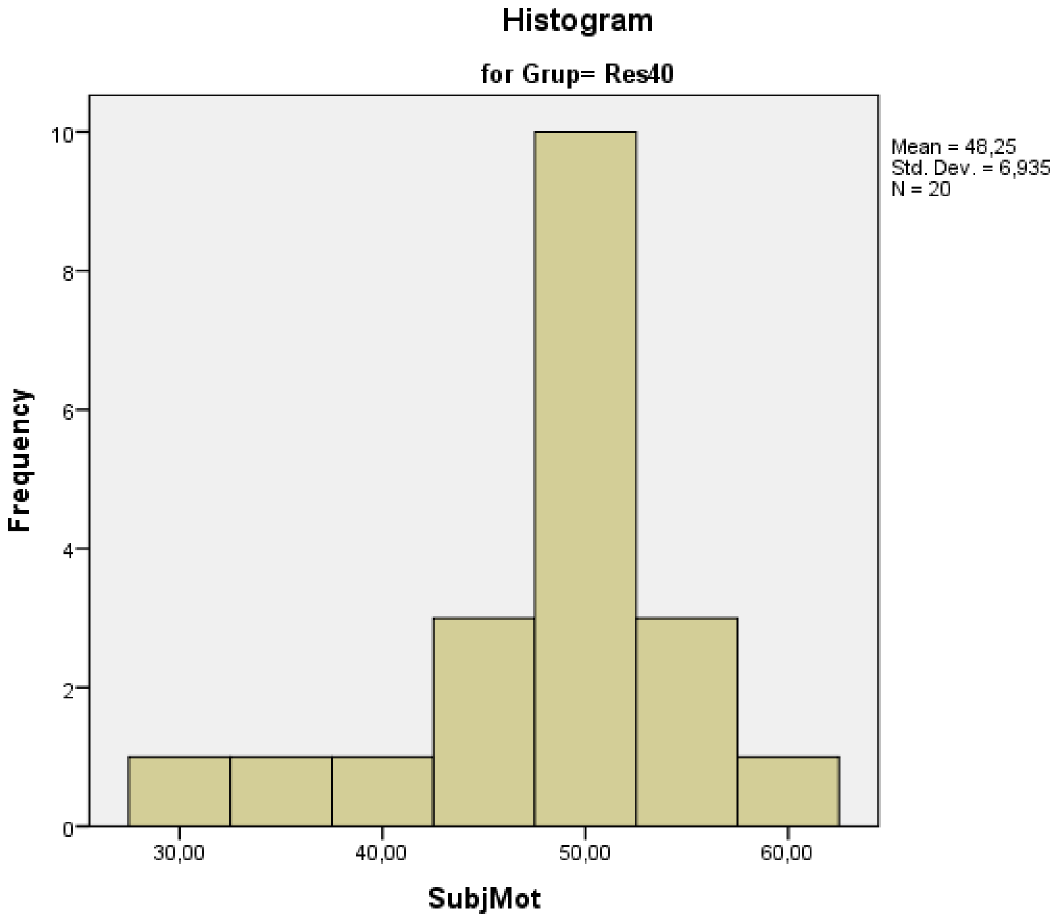
Histogram
for Grup= Res40
Mean = 48,25
Std. Dev. = 6,935
N = 20
Frequency
0
2
4
6
8
10
30,00
40,00
50,00
60,00
SubjMot

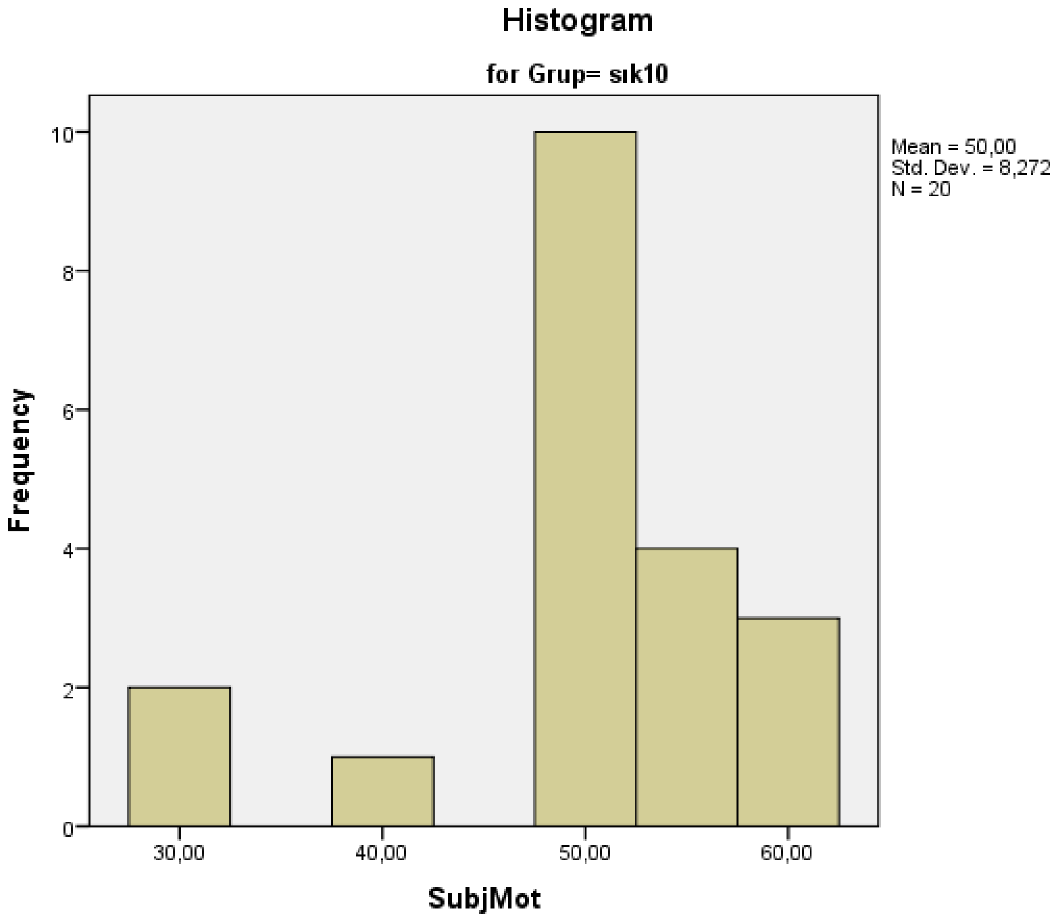
Histogram
for Grup= sık10
Mean = 50,00
Std. Dev. = 8,272
N = 20
Frequency
0
2
4
6
8
10
30,00
40,00
50,00
60,00
SubjMot

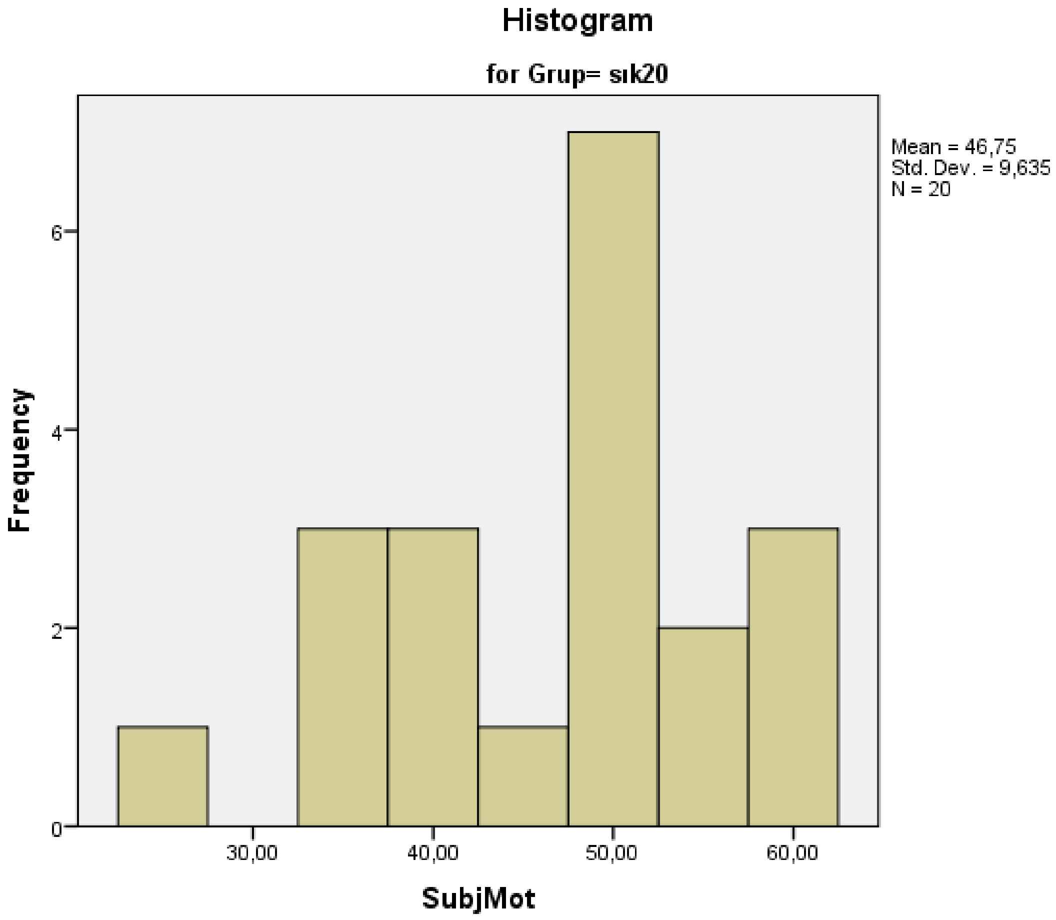

Histogram
for Grup= sık20
Mean = 46,75
Std. Dev. = 9,635
N = 20
Frequency
0
2
4
6
30,00
40,00
50,00
60,00
SubjMot

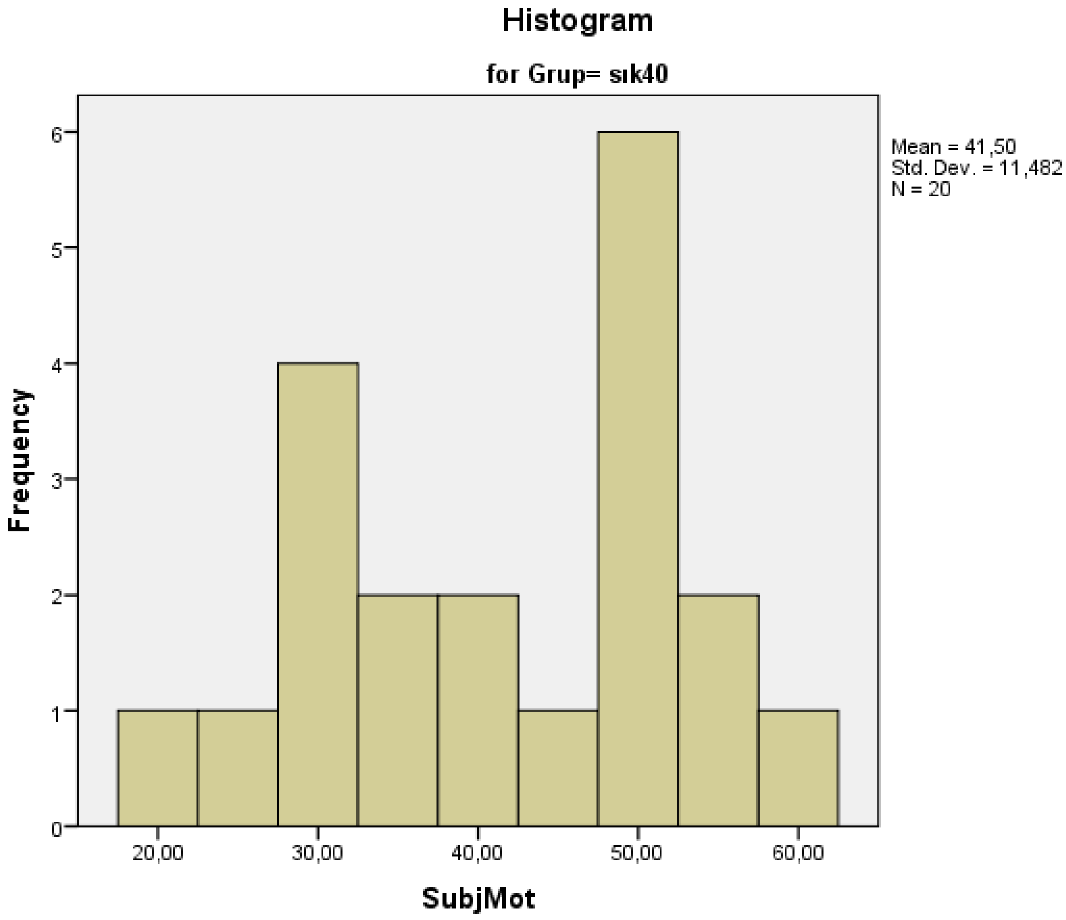
Histogram
for Grup= sık40
Mean = 41,50
Std. Dev. = 11,482
N = 20
Frequency
0
1
2
3
4
5
6
20,00
30,00
40,00
50,00
60,00
SubjMot

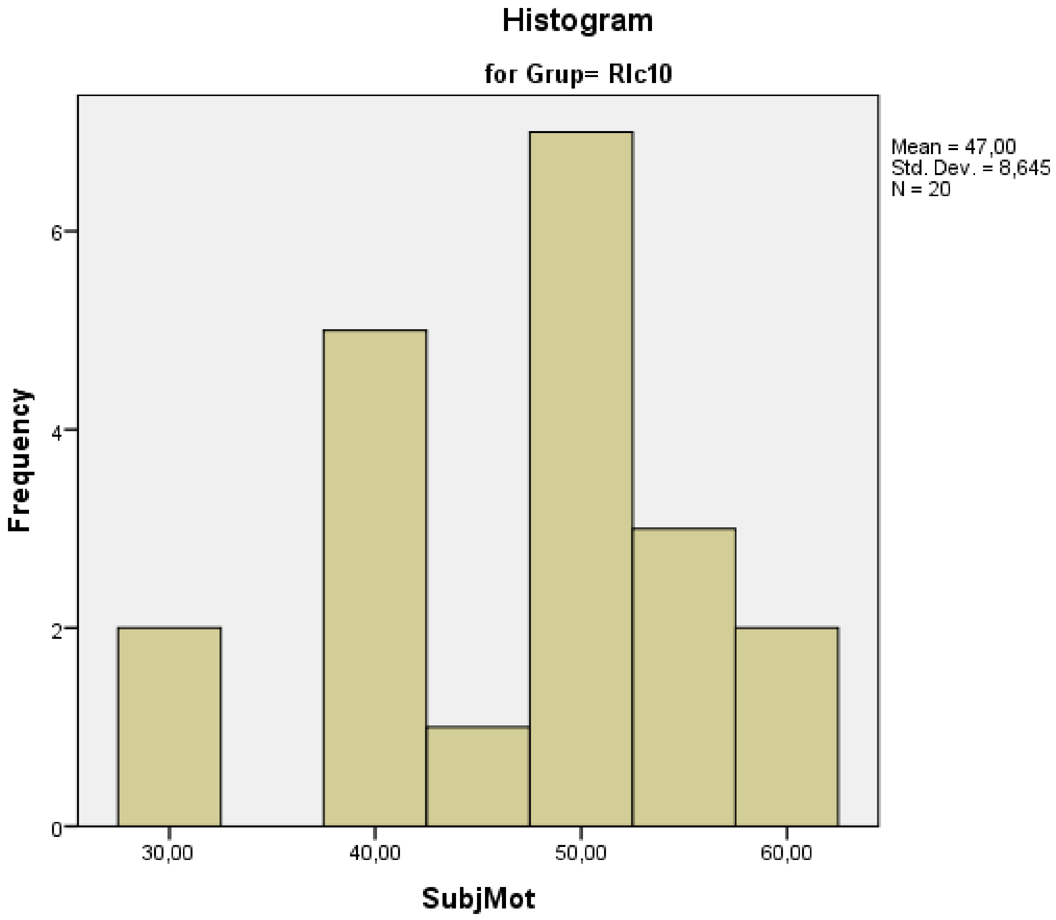
Histogram
for Grup= RIc10
Mean = 47,00
Std. Dev. = 8,645
N = 20
Frequency
0
2
4
6
30,00
40,00
50,00
60,00
SubjMot

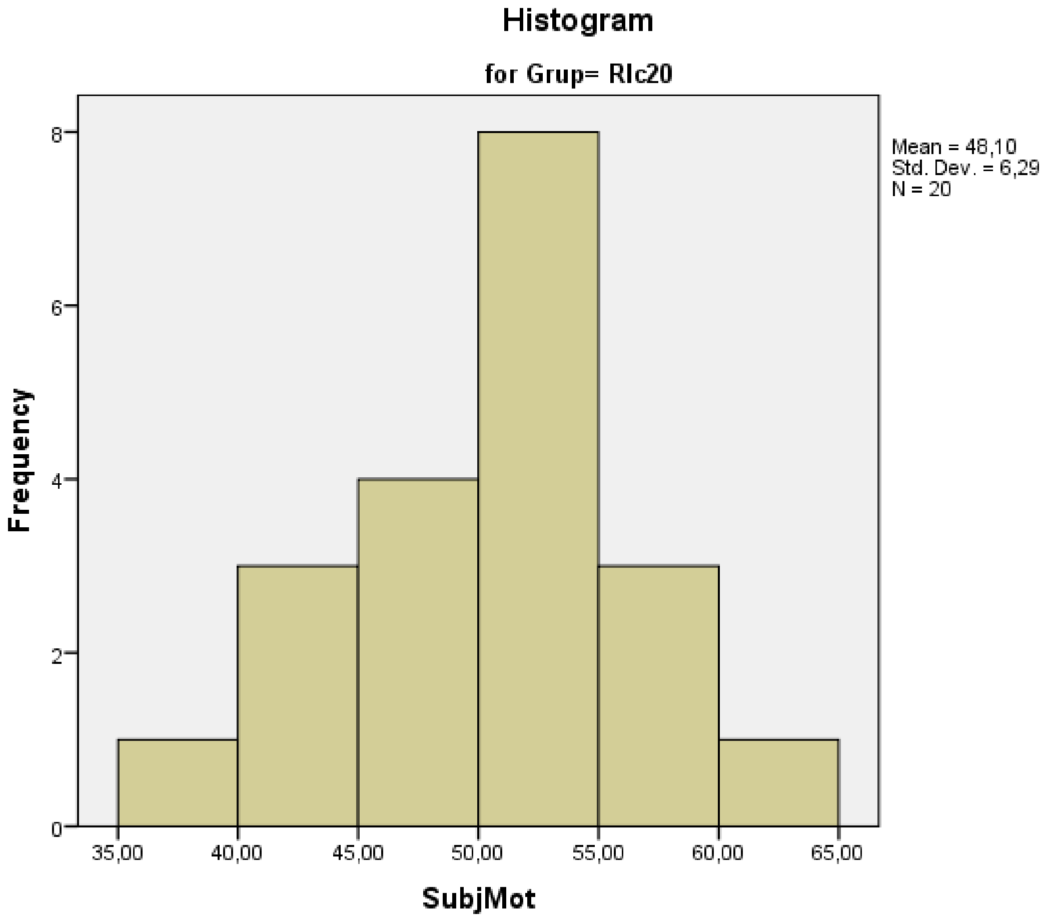
Histogram
for Grup= RIc20
Mean = 48,10
Std. Dev. = 6,29
N = 20
Frequency
0
2
4
6
8
35,00
40,00
45,00
50,00
55,00
60,00
65,00
SubjMot

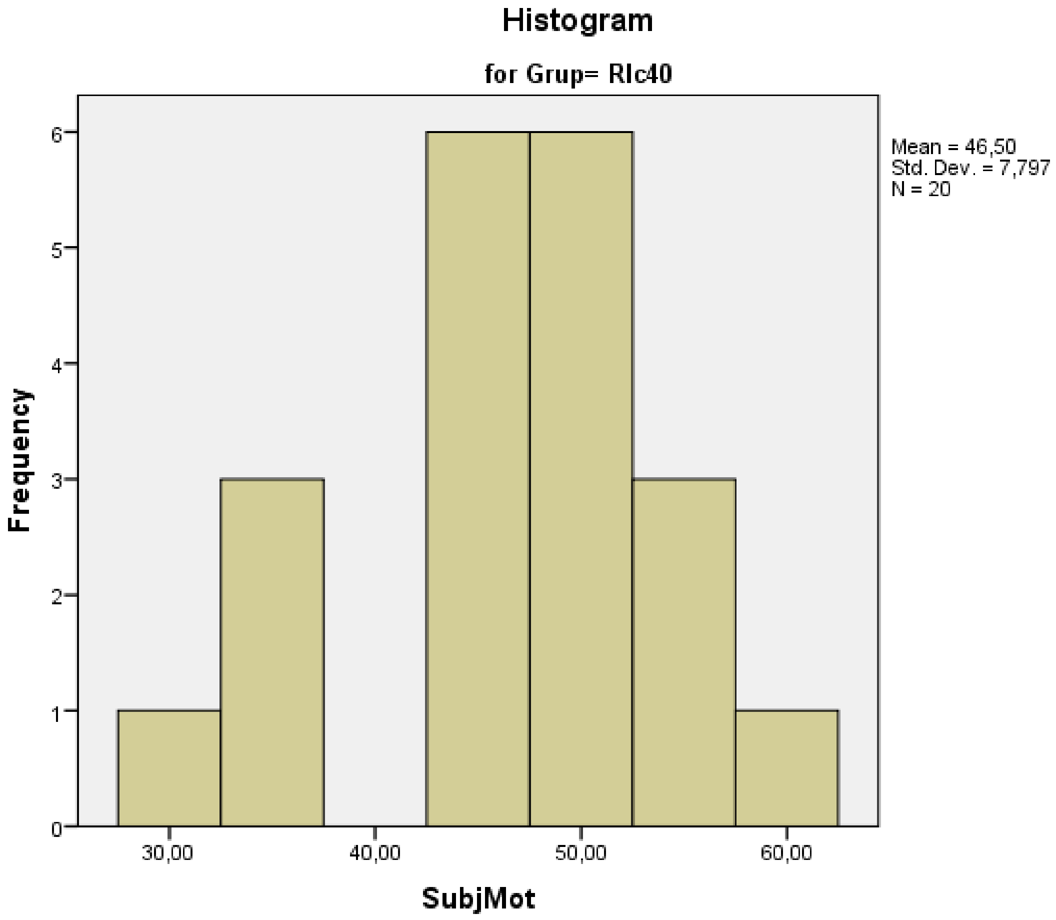

Stem-and-Leaf Plots

```
SubjMot Stem-and-Leaf Plot for
Grup= kontrol

 Frequency    Stem &  Leaf

     2,00        3 .  00
     3,00        3 .  555
     1,00        4 .  0
     2,00        4 .  55
     7,00        5 .  0000000
     5,00        5 .  55555

 Stem width:     10,00
 Each leaf:       1 case(s)
```

SubjMot Stem-and-Leaf Plot for
Grup= Res10

```
 Frequency    Stem &  Leaf

     2,00        3 .  00
     2,00        3 .  55
     2,00        4 .  00
     1,00        4 .  5
     8,00        5 .  00000000
     5,00        5 .  55555

 Stem width:     10,00
 Each leaf:       1 case(s)
```

SubjMot Stem-and-Leaf Plot for
Grup= Res20

```
 Frequency    Stem &  Leaf

     2,00 Extremes    (=<35)
      ,00        4 .
     1,00        4 .  5
    10,00        5 .  0000000000
     4,00        5 .  5555
     3,00        6 .  000

 Stem width:     10,00
 Each leaf:       1 case(s)
```

SubjMot Stem-and-Leaf Plot for
Grup= Res40

```
 Frequency    Stem &  Leaf

     2,00 Extremes    (=<35)
     1,00        4 .  0
     3,00        4 .  555
    10,00        5 .  0000000000
     3,00        5 .  555
     1,00 Extremes    (>=60)

 Stem width:     10,00
 Each leaf:       1 case(s)
```

```
SubjMot Stem-and-Leaf Plot for
Grup= sık10

 Frequency    Stem &  Leaf

     3,00 Extremes    (=<40)
    10,00        5 .  0000000000
     4,00        5 .  5555
     3,00        6 .  000

 Stem width:     10,00
 Each leaf:       1 case(s)
```

```
SubjMot Stem-and-Leaf Plot for
Grup= sık20

 Frequency    Stem &  Leaf

     1,00        2 .  5
     3,00        3 .  555
     4,00        4 .  0005
     9,00        5 .  000000055
     3,00        6 .  000

 Stem width:     10,00
 Each leaf:       1 case(s)
```

```
SubjMot Stem-and-Leaf Plot for
Grup= sık40

 Frequency    Stem &  Leaf

     2,00        2 .  05
     6,00        3 .  000055
     3,00        4 .  005
     8,00        5 .  00000055
     1,00        6 .  0

 Stem width:     10,00
 Each leaf:       1 case(s)
```

```
SubjMot Stem-and-Leaf Plot for
Grup= Rlc10

 Frequency    Stem &  Leaf
```

```
     2,00        3 .  00
      ,00        3 .
     5,00        4 .  00000
     1,00        4 .  5
     7,00        5 .  0000000
     3,00        5 .  555
     2,00        6 .  00

 Stem width:     10,00
 Each leaf:       1 case(s)
```

```
SubjMot Stem-and-Leaf Plot for
Grup= Rlc20

 Frequency    Stem &  Leaf

     1,00 Extremes    (=<35)
     3,00        4 .  000
     4,00        4 .  5555
     8,00        5 .  00000000
     3,00        5 .  557
     1,00 Extremes    (>=60)

 Stem width:     10,00
 Each leaf:       1 case(s)
```

```
SubjMot Stem-and-Leaf Plot for
Grup= Rlc40

 Frequency    Stem &  Leaf

     4,00 Extremes    (=<35)
      ,00        4 .
     6,00        4 .  555555
     6,00        5 .  000000
     3,00        5 .  555
     1,00 Extremes    (>=60)

 Stem width:     10,00
 Each leaf:       1 case(s)
```

Normal Q-Q Plots

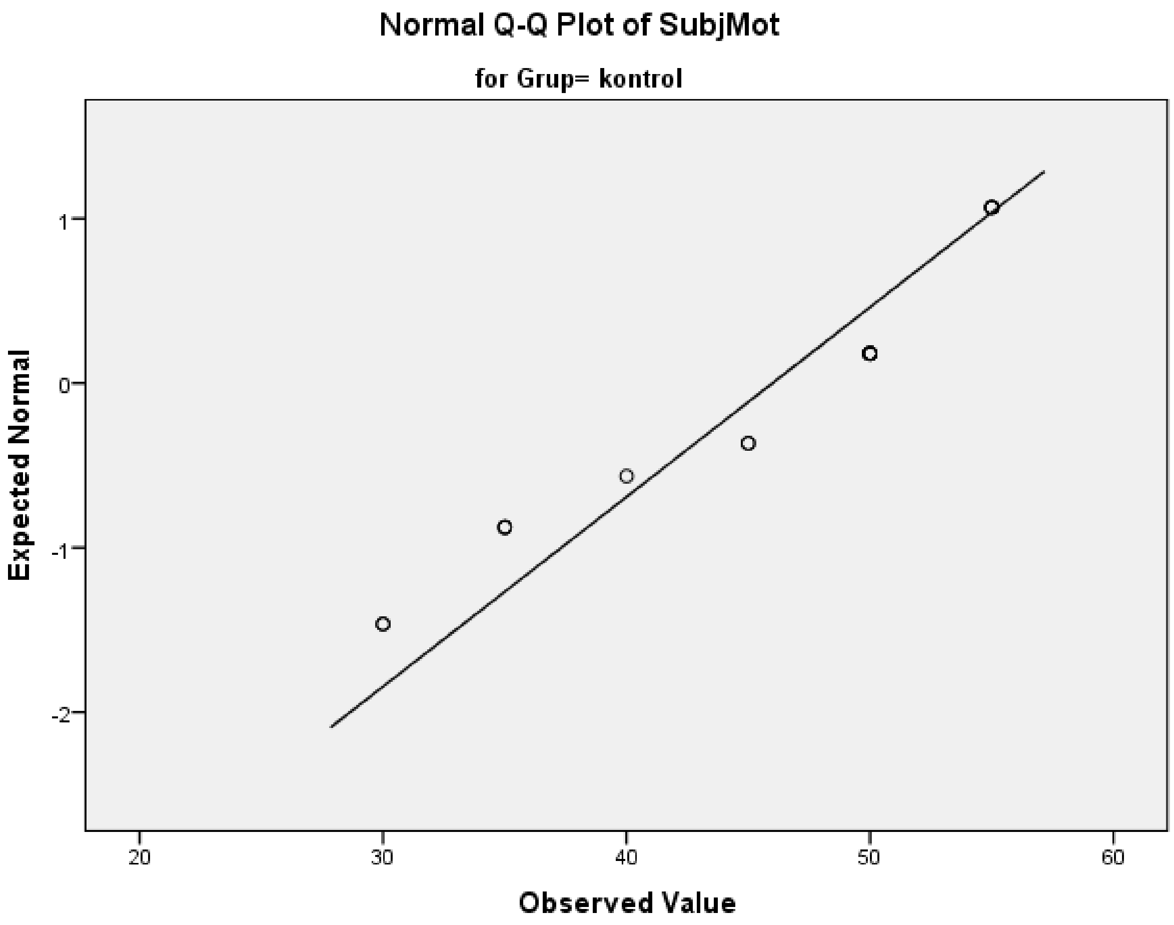
Normal Q-Q Plot of SubjMot
for Grup= kontrol
Expected Normal
1
0
-1
-2
20
30
40
50
60
Observed Value

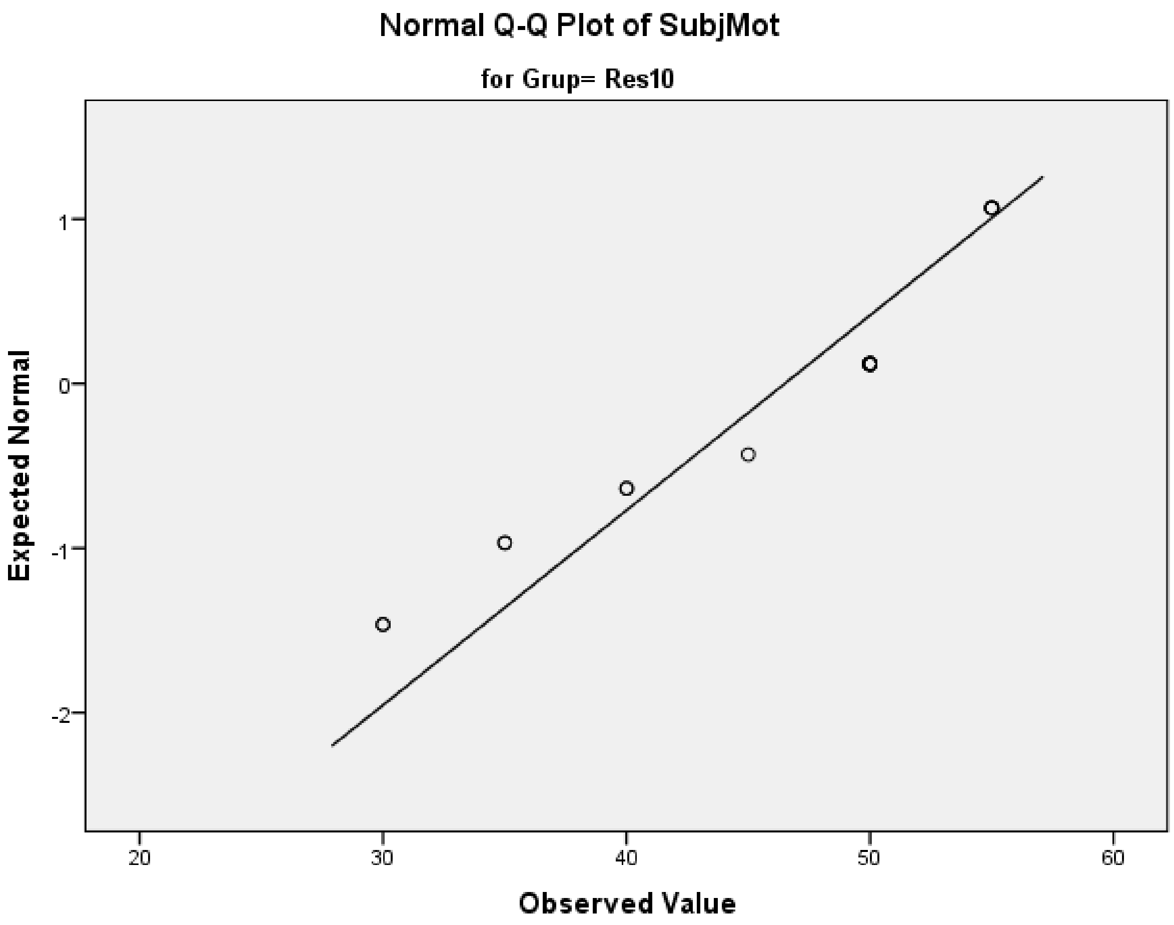
Normal Q-Q Plot of SubjMot
for Grup= Res10
Expected Normal
1
0
-1
-2
20
30
40
50
60
Observed Value

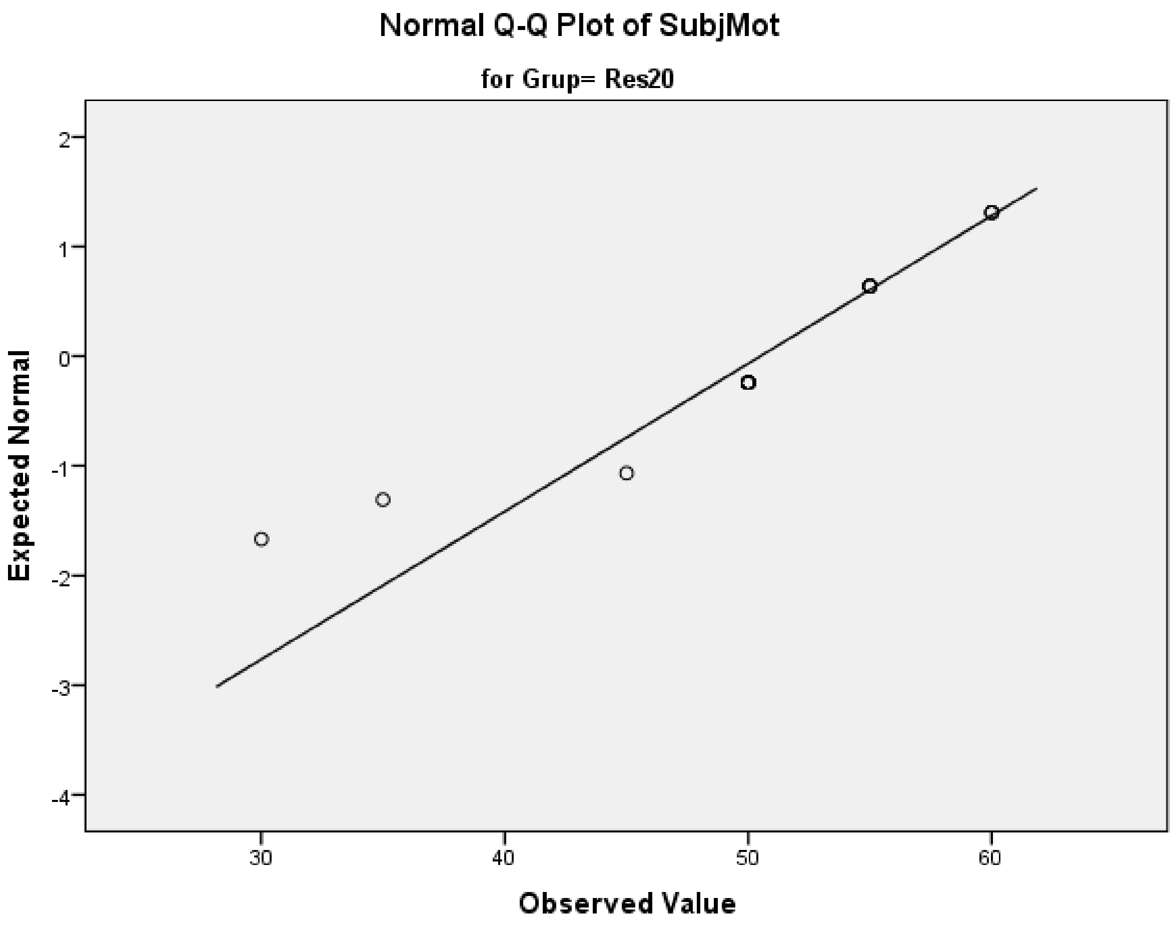
Normal Q-Q Plot of SubjMot
for Grup= Res20
Expected Normal
2
1
0
-1
-2
-3
-4
30
40
50
60
Observed Value

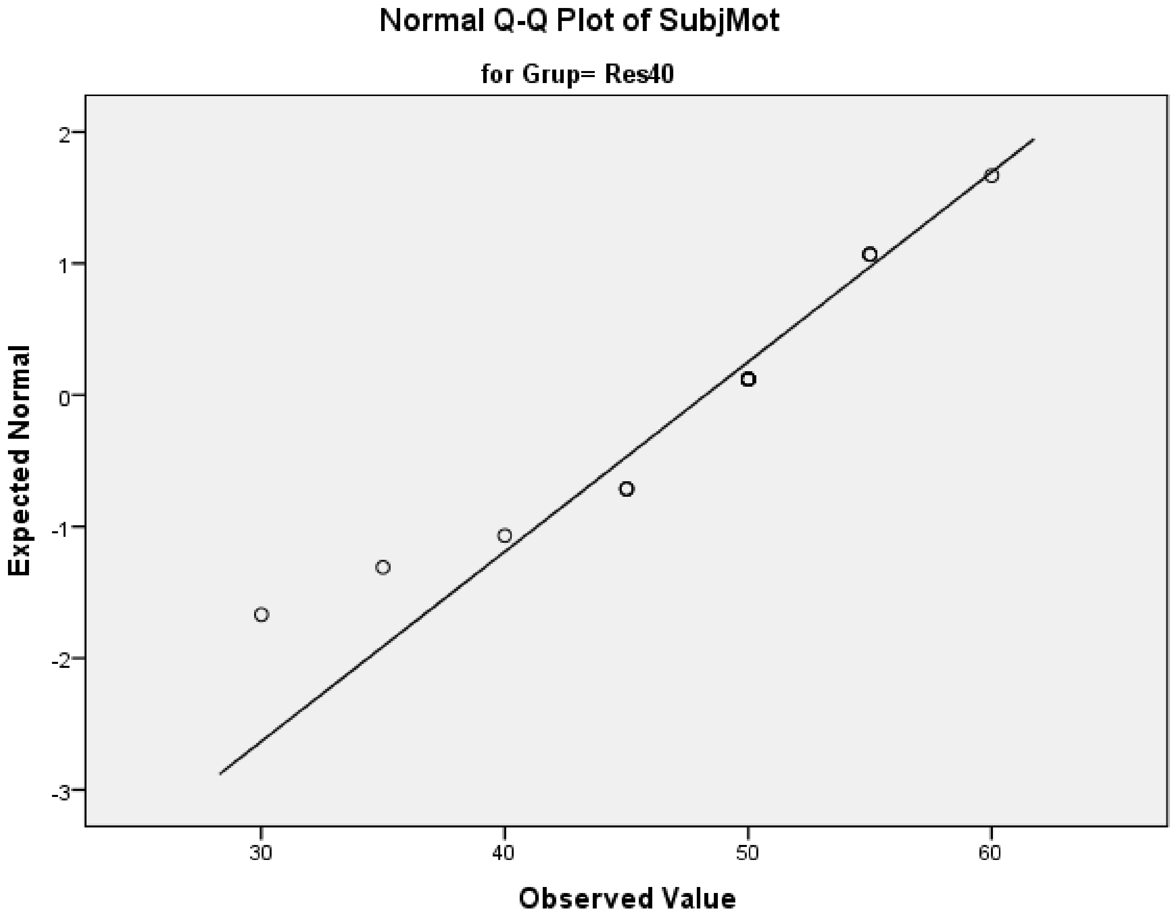

Normal Q-Q Plot of SubjMot
for Grup= Res40
Expected Normal
Observed Value
2
1
0
-1
-2
-3
30
40
50
60

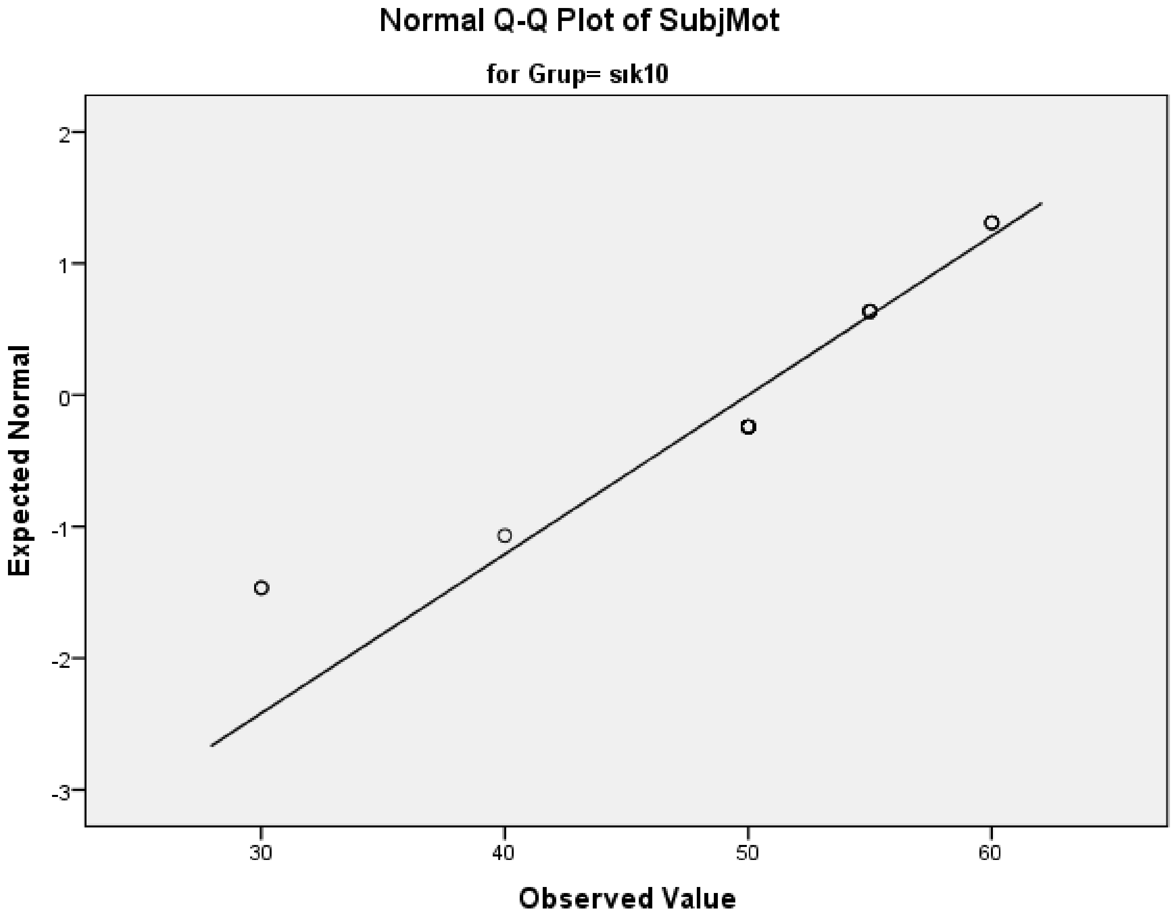
Normal Q-Q Plot of SubjMot
for Grup= sık10
Expected Normal
Observed Value
2
1
0
-1
-2
-3
30
40
50
60

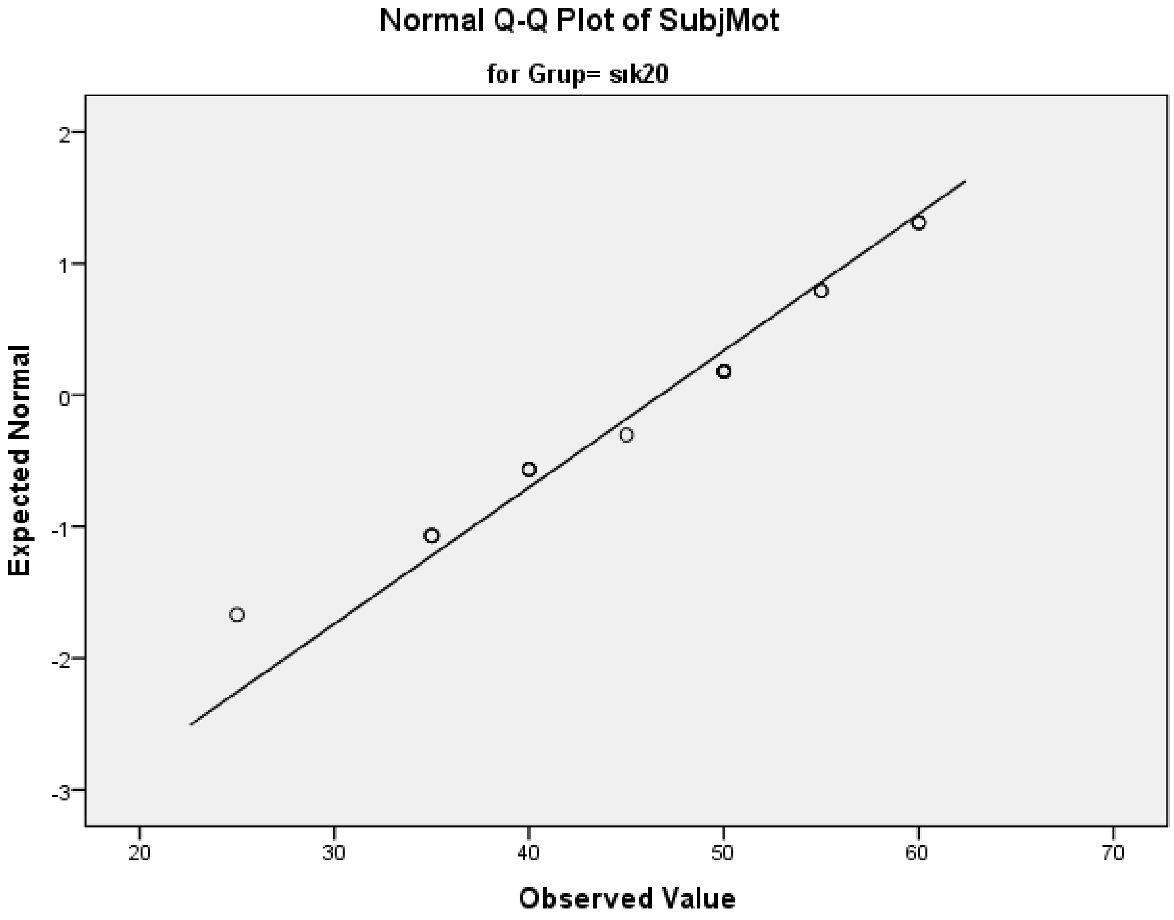
Normal Q-Q Plot of SubjMot
for Grup= sık20
Expected Normal
Observed Value
2
1
0
-1
-2
-3
20
30
40
50
60
70

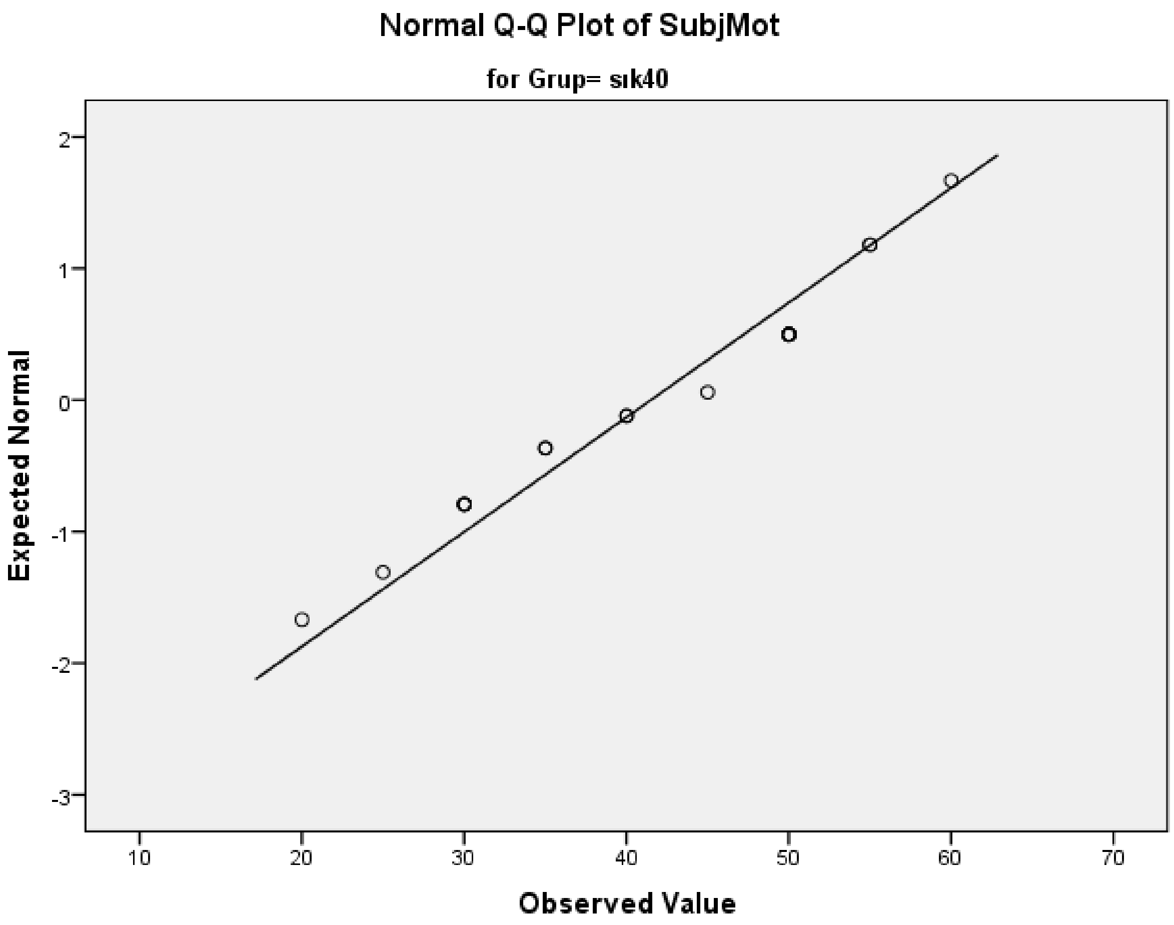

Normal Q-Q Plot of SubjMot
for Grup= sık40
Expected Normal
Observed Value
2
1
0
-1
-2
-3
10
20
30
40
50
60
70

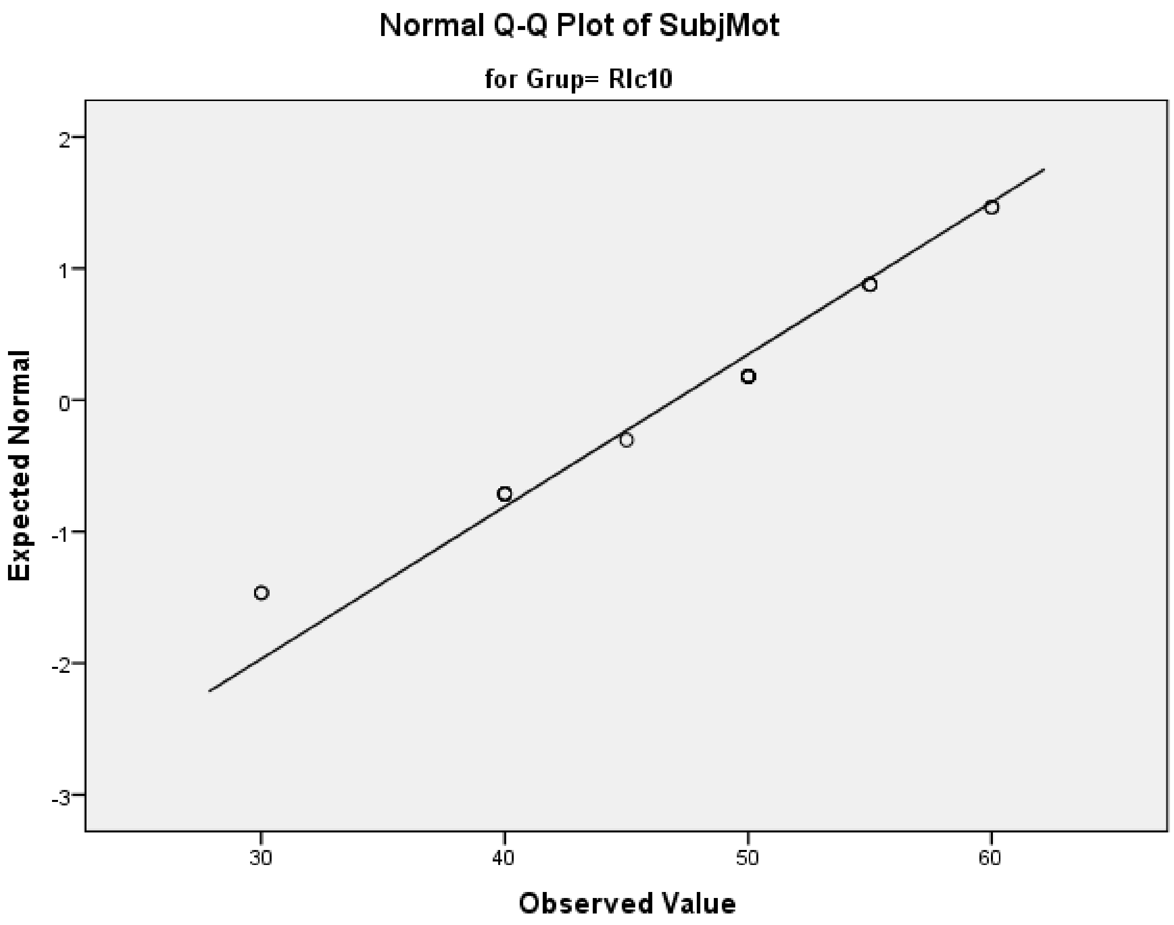
Normal Q-Q Plot of SubjMot
for Grup= Rlc10
Expected Normal
2
1
0
-1
-2
-3
30
40
50
60
Observed Value

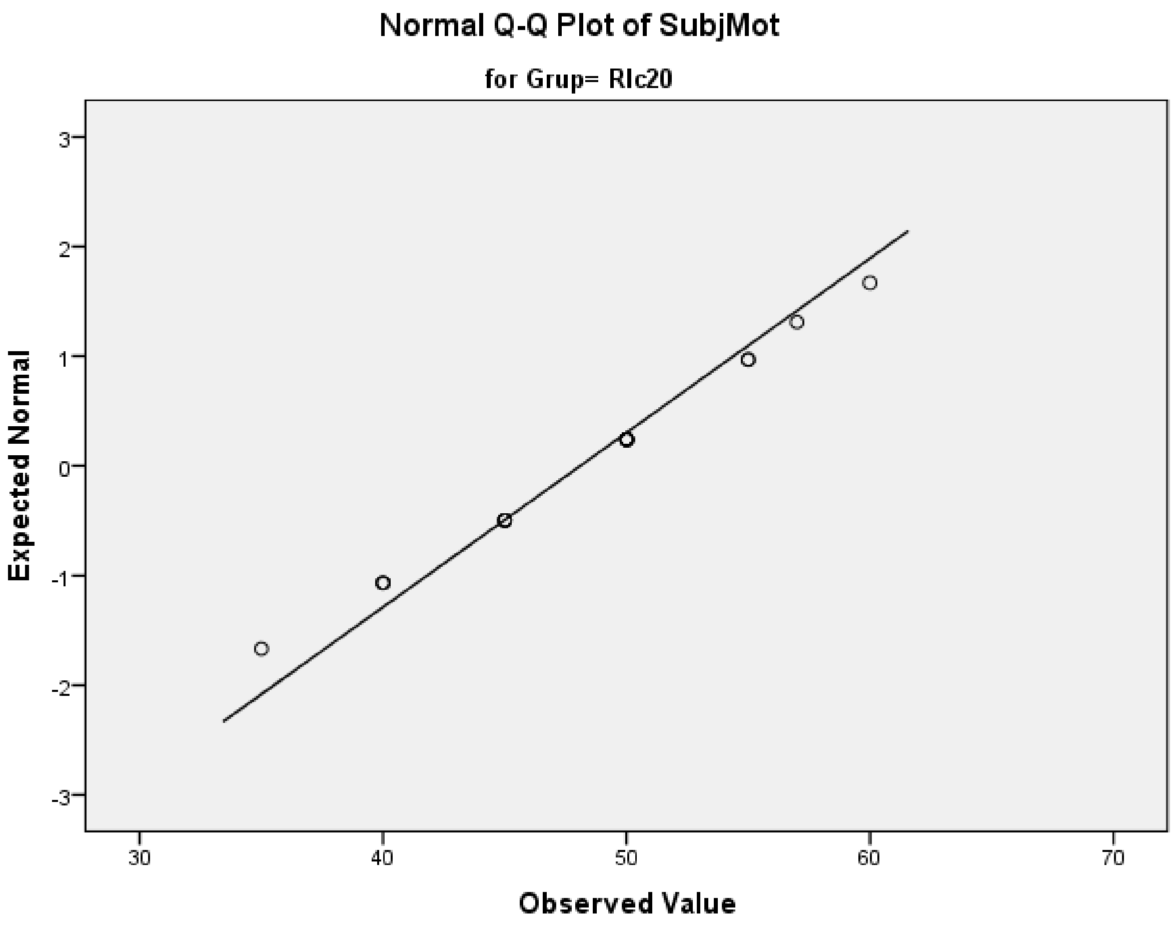
Normal Q-Q Plot of SubjMot
for Grup= Rlc20
Expected Normal
Observed Value
3
2
1
0
-1
-2
-3
30
40
50
60
70

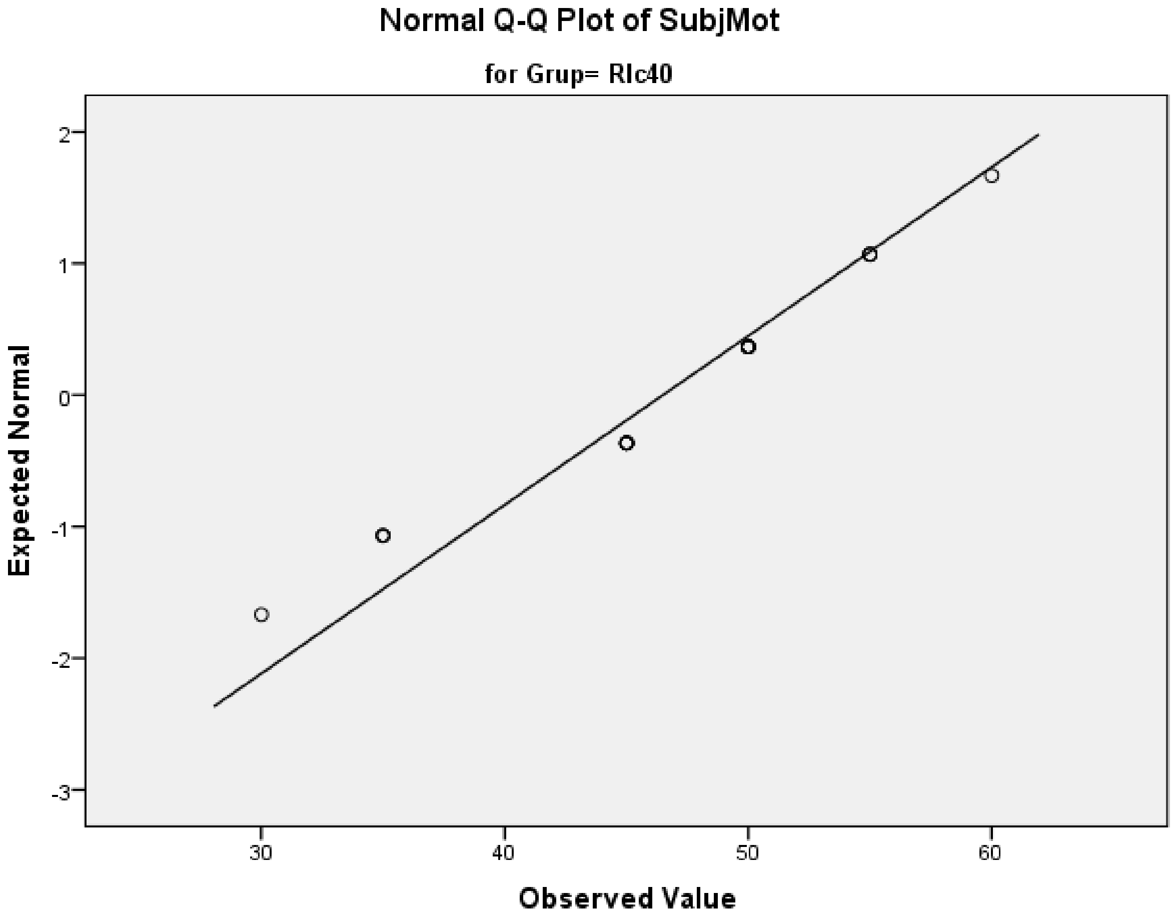

Detrended Normal Q-Q Plots

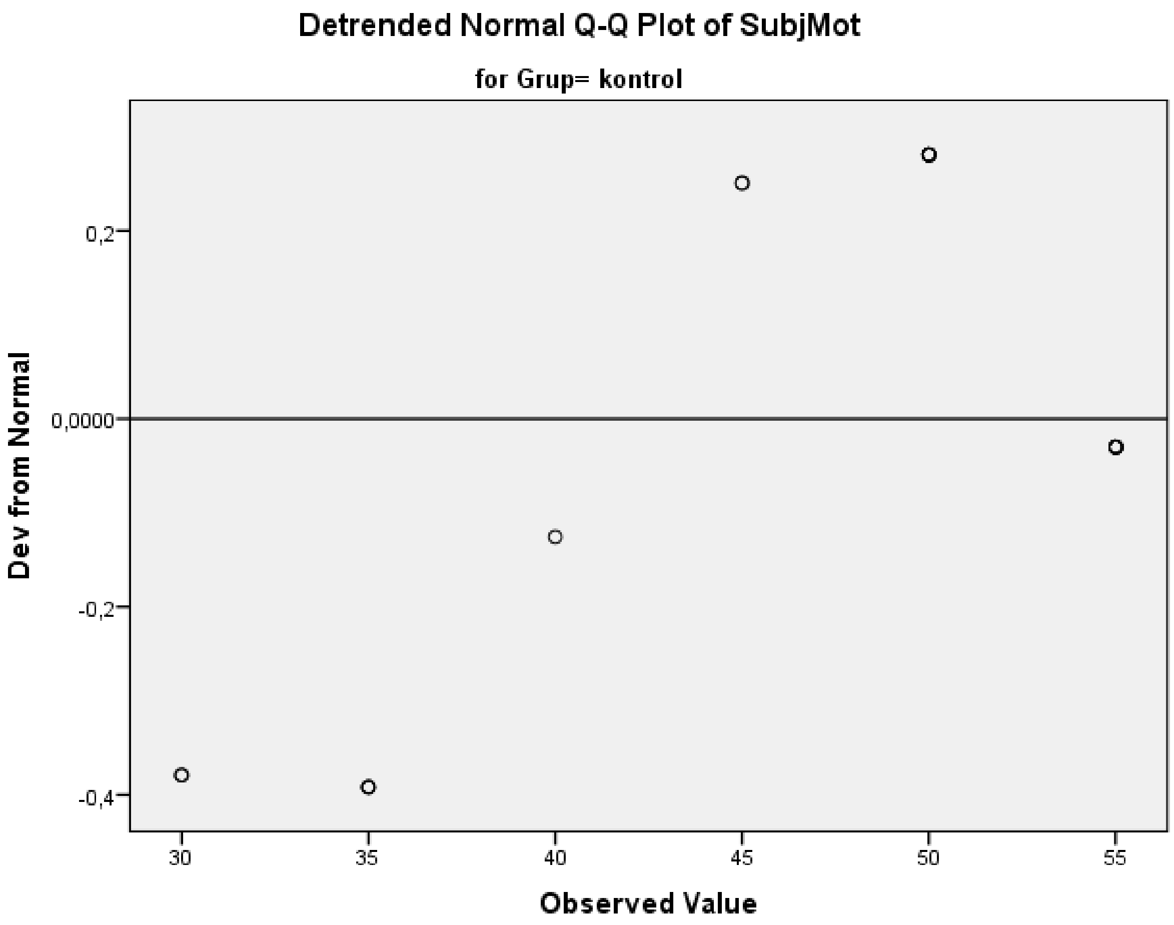
Detrended Normal Q-Q Plot of SubjMot
for Grup= kontrol
Dev from Normal
0,2
0,0000
-0,2
-0,4
30
35
40
45
50
55
Observed Value

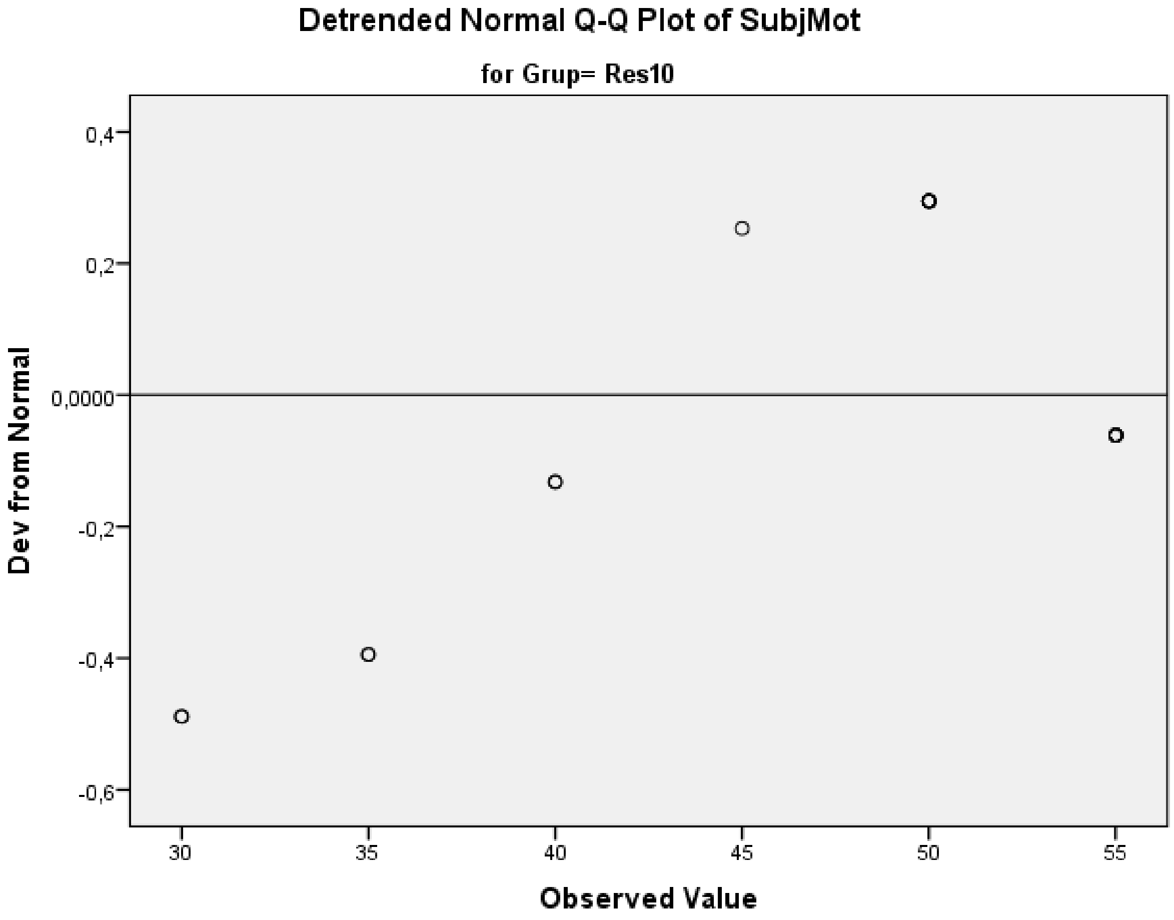

Detrended Normal Q-Q Plot of SubjMot
for Grup= Res10
0,4
0,2
0,0000
-0,2
-0,4
-0,6
Dev from Normal
30
35
40
45
50
55
Observed Value

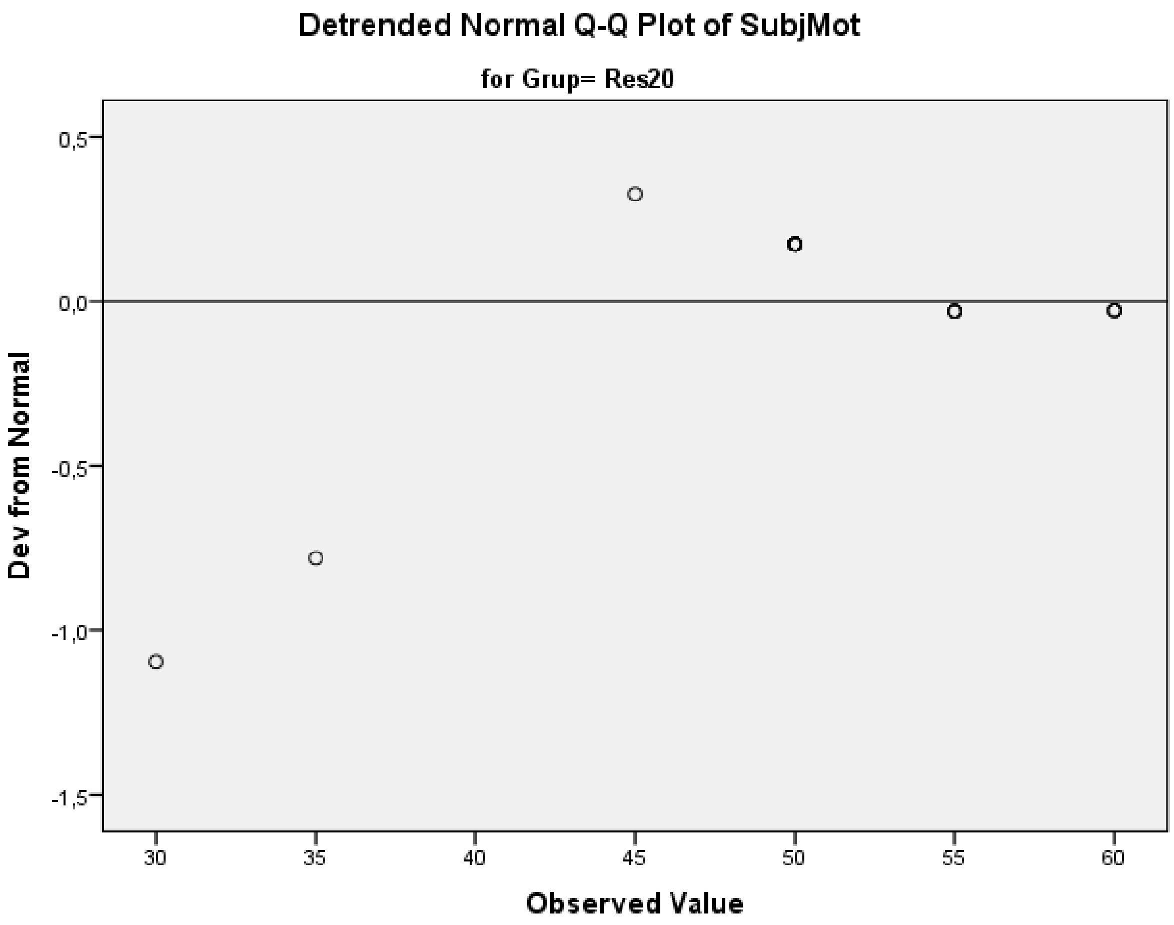
Detrended Normal Q-Q Plot of SubjMot
for Grup= Res20
Dev from Normal
0,5
0,0
-0,5
-1,0
-1,5
30
35
40
45
50
55
60
Observed Value

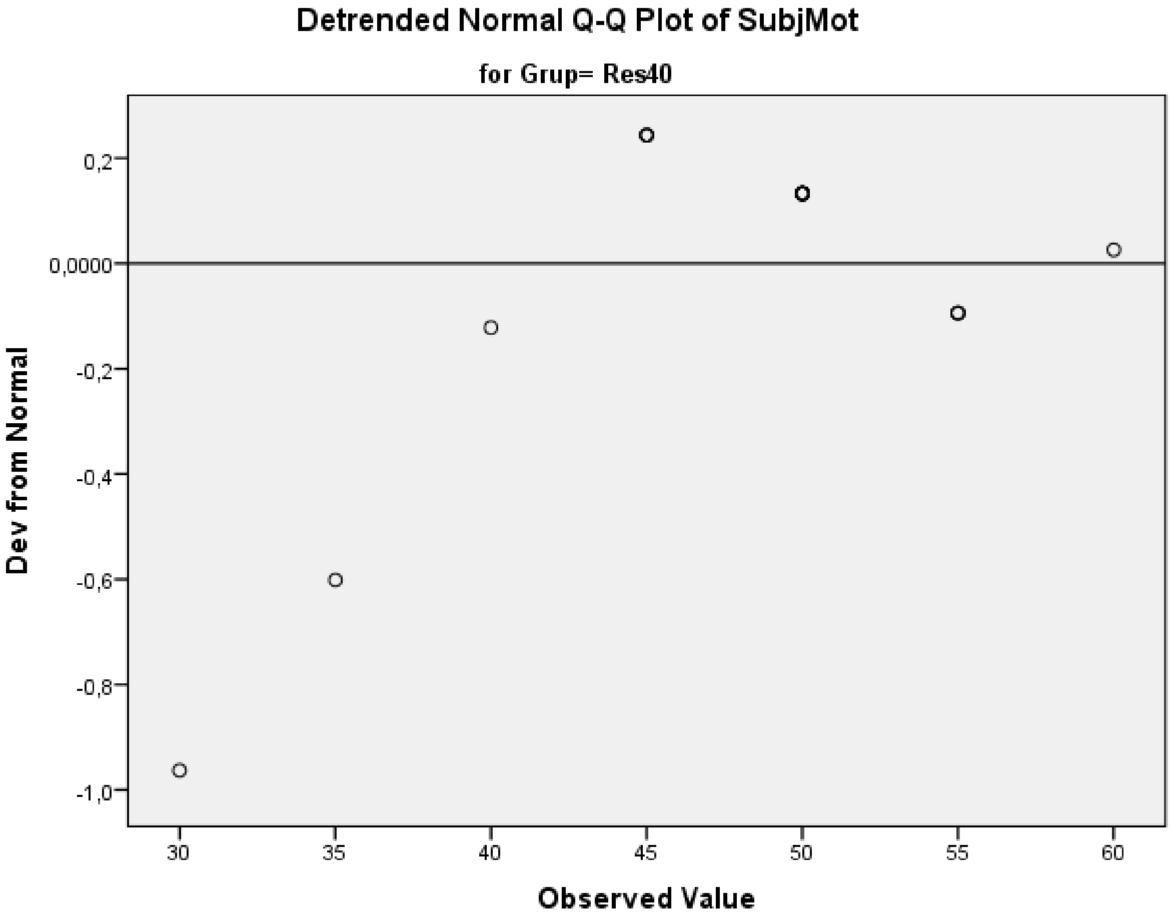
Detrended Normal Q-Q Plot of SubjMot
for Grup= Res40
Dev from Normal
0,2
0,0000
-0,2
-0,4
-0,6
-0,8
-1,0
30
35
40
45
50
55
60
Observed Value

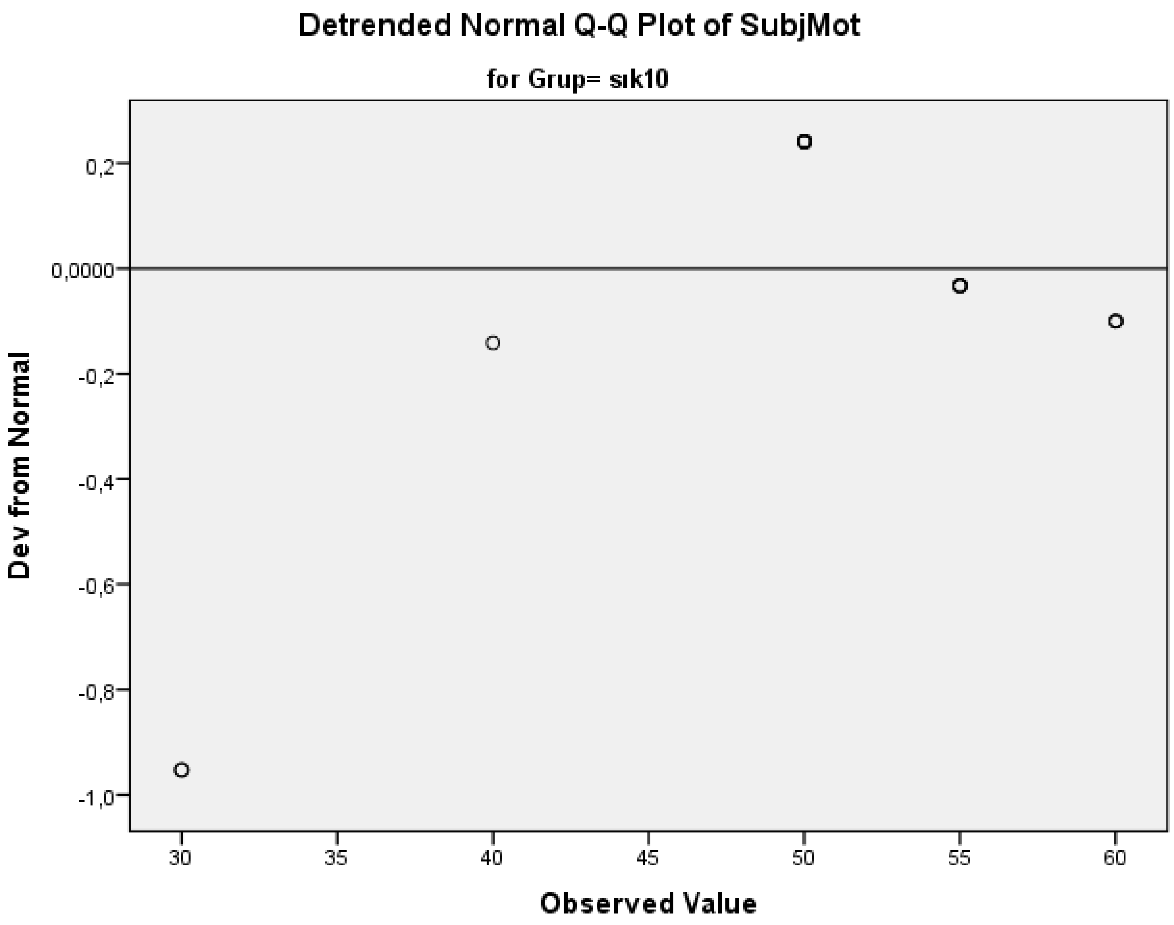

Detrended Normal Q-Q Plot of SubjMot
for Grup= sık10
0,2
0,0000
-0,2
-0,4
-0,6
-0,8
-1,0
Dev from Normal
30
35
40
45
50
55
60
Observed Value

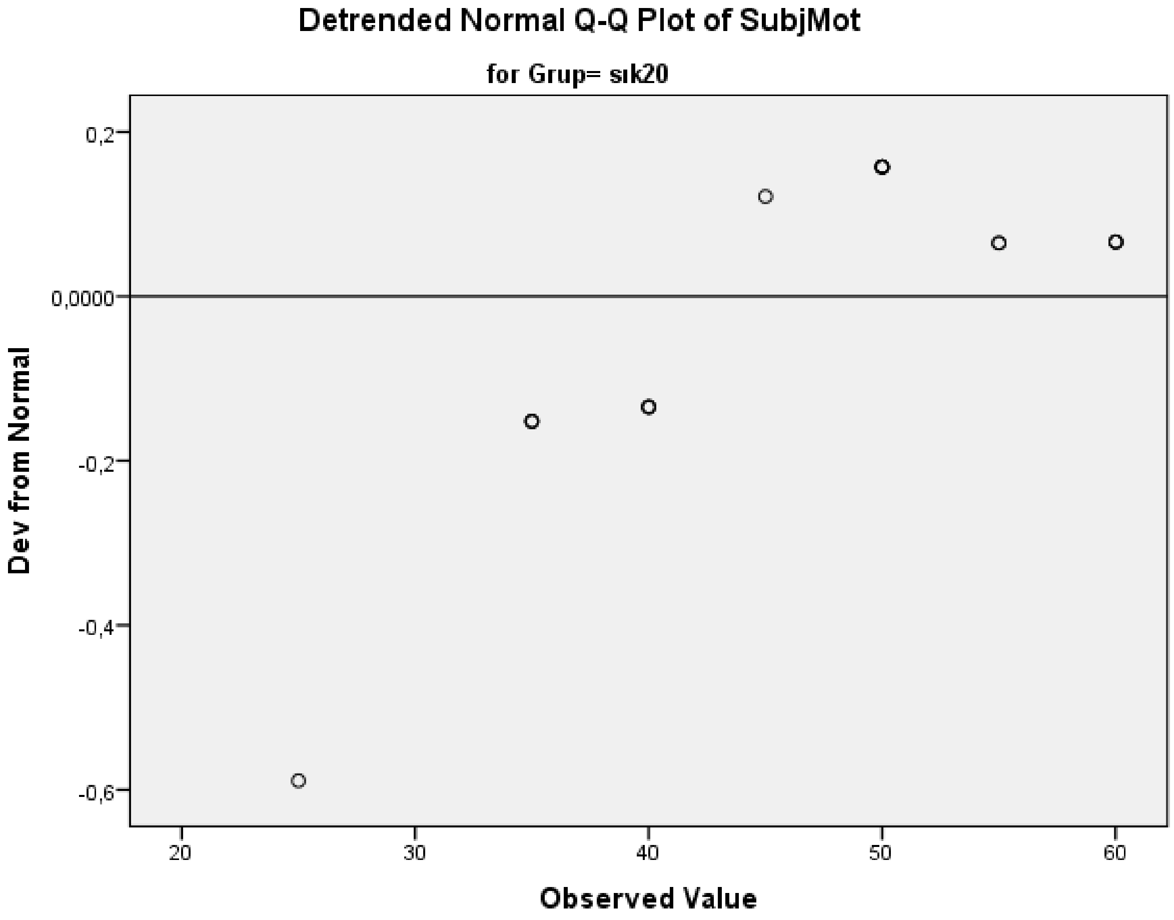

Detrended Normal Q-Q Plot of SubjMot
for Grup= sık20
Dev from Normal
0,2
0,0000
-0,2
-0,4
-0,6
20
30
40
50
60
Observed Value

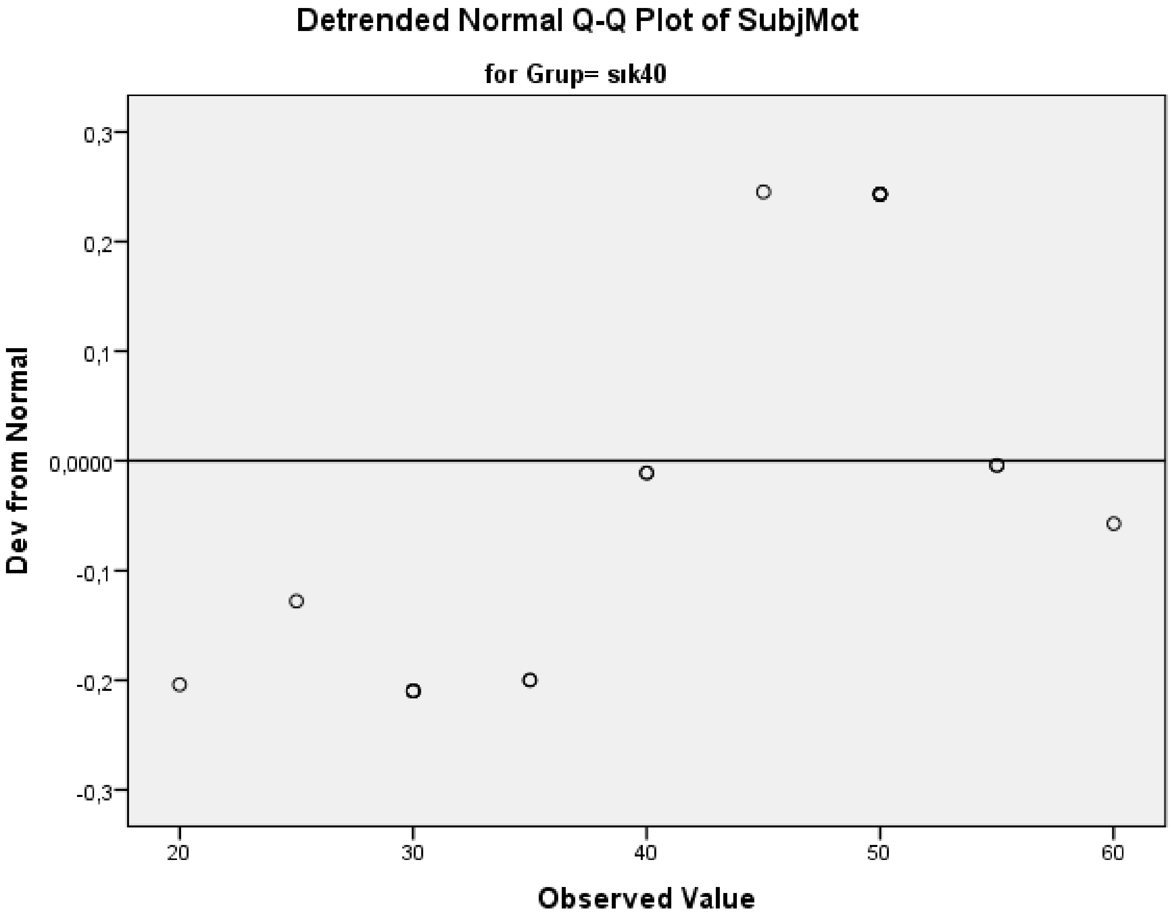
Detrended Normal Q-Q Plot of SubjMot
for Grup= sık40
Dev from Normal
0,3
0,2
0,1
0,0000
-0,1
-0,2
-0,3
20
30
40
50
60
Observed Value

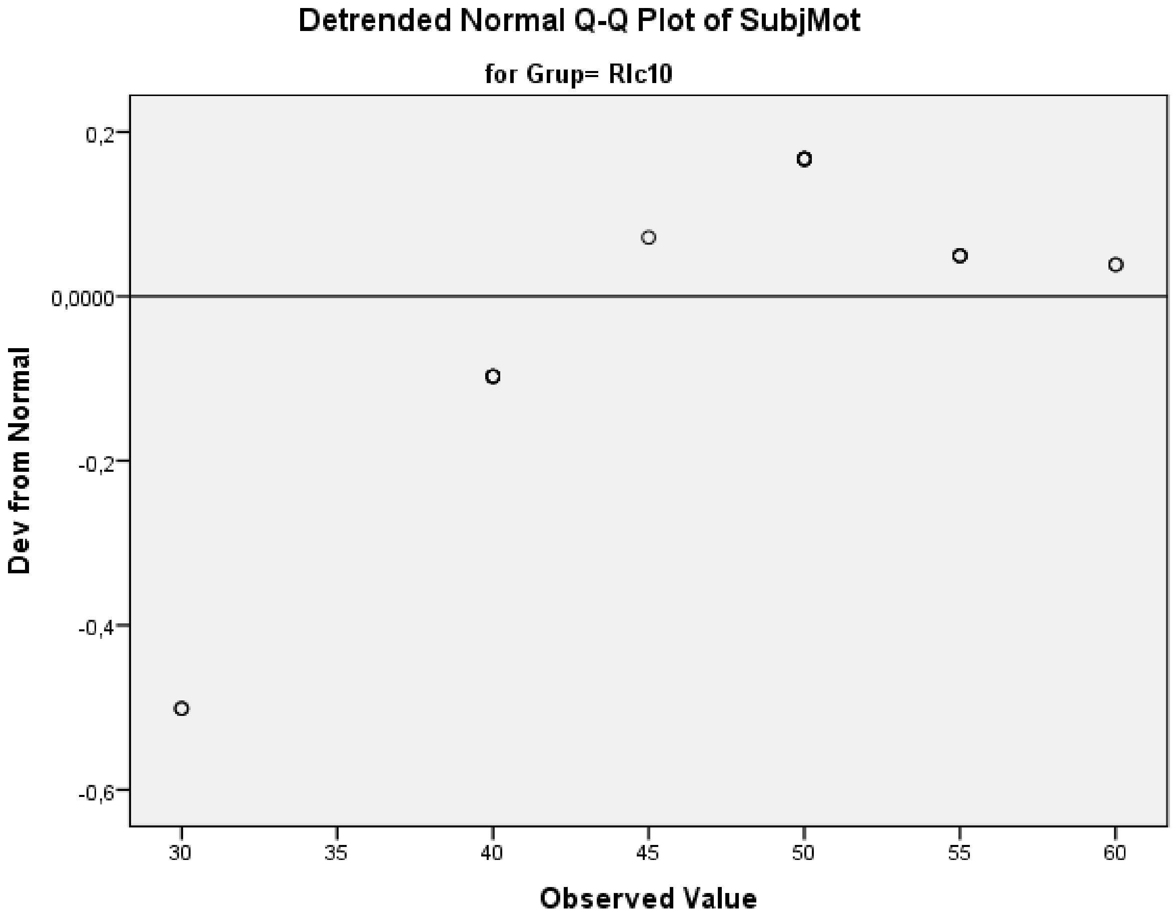
Detrended Normal Q-Q Plot of SubjMot
for Grup= RIc10
0,2
0,0000
-0,2
-0,4
-0,6
Dev from Normal
30
35
40
45
50
55
60
Observed Value

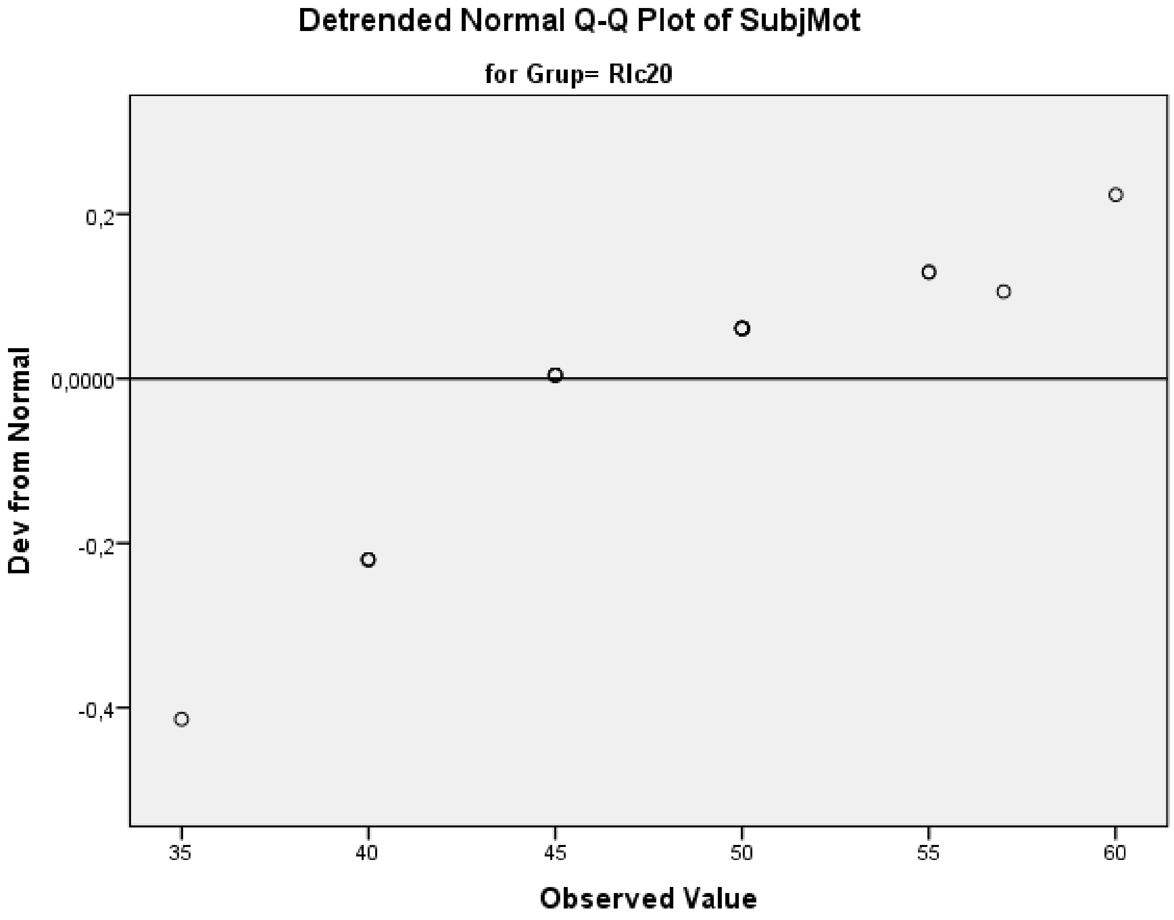

Detrended Normal Q-Q Plot of SubjMot
for Grup= RIc20
Dev from Normal
0,2
0,0000
-0,2
-0,4
35
40
45
50
55
60
Observed Value

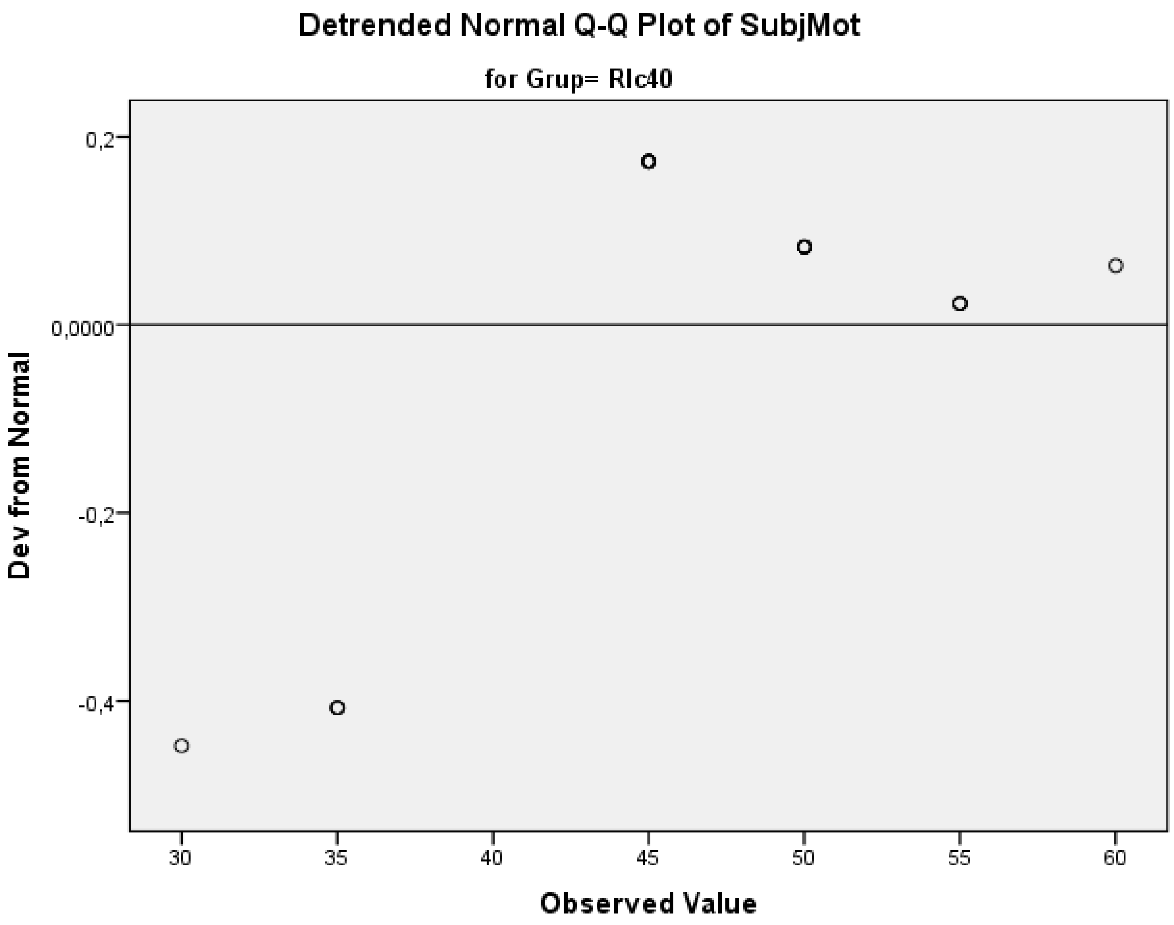
Detrended Normal Q-Q Plot of SubjMot
for Grup= RIc40
Dev from Normal
0,2
0,0000
-0,2
-0,4
30
35
40
45
50
55
60
Observed Value

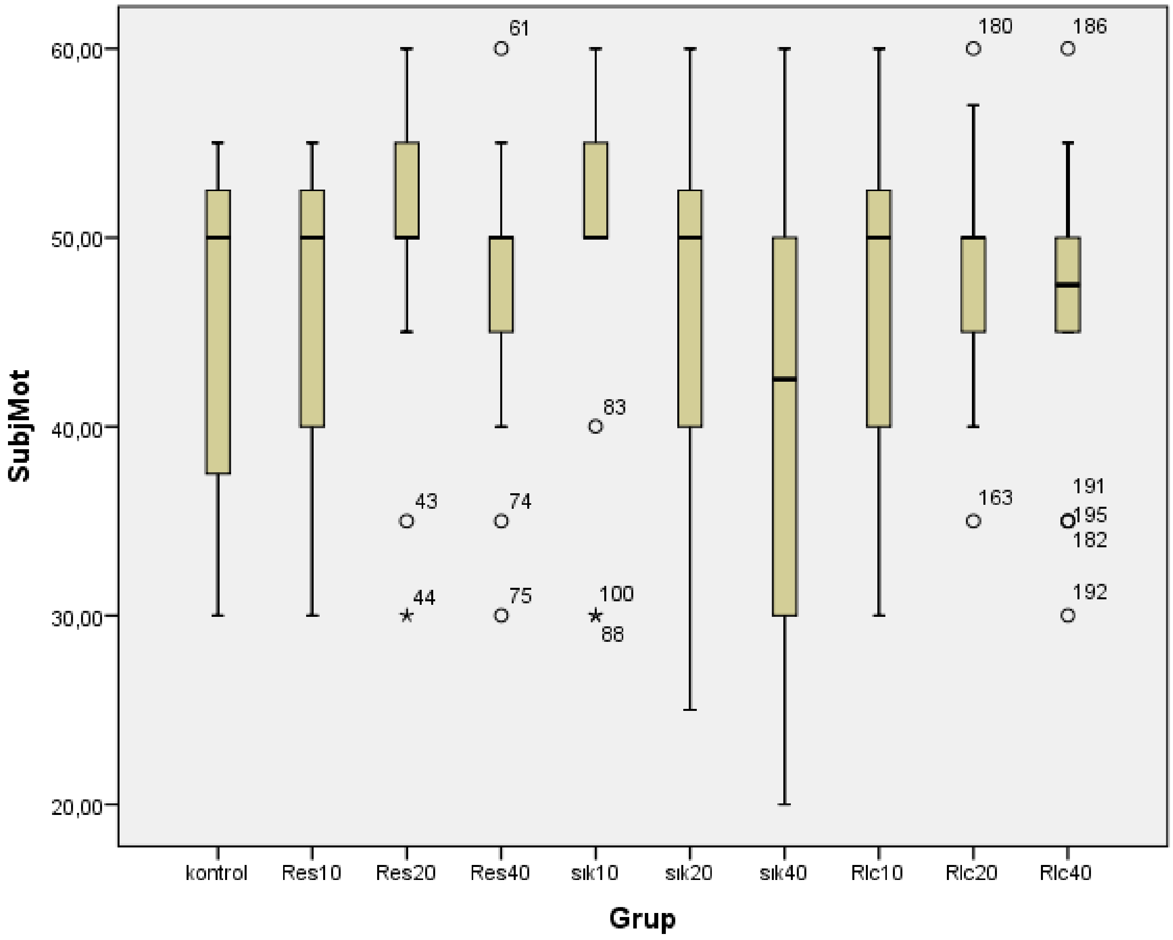

CasaMot

Histograms

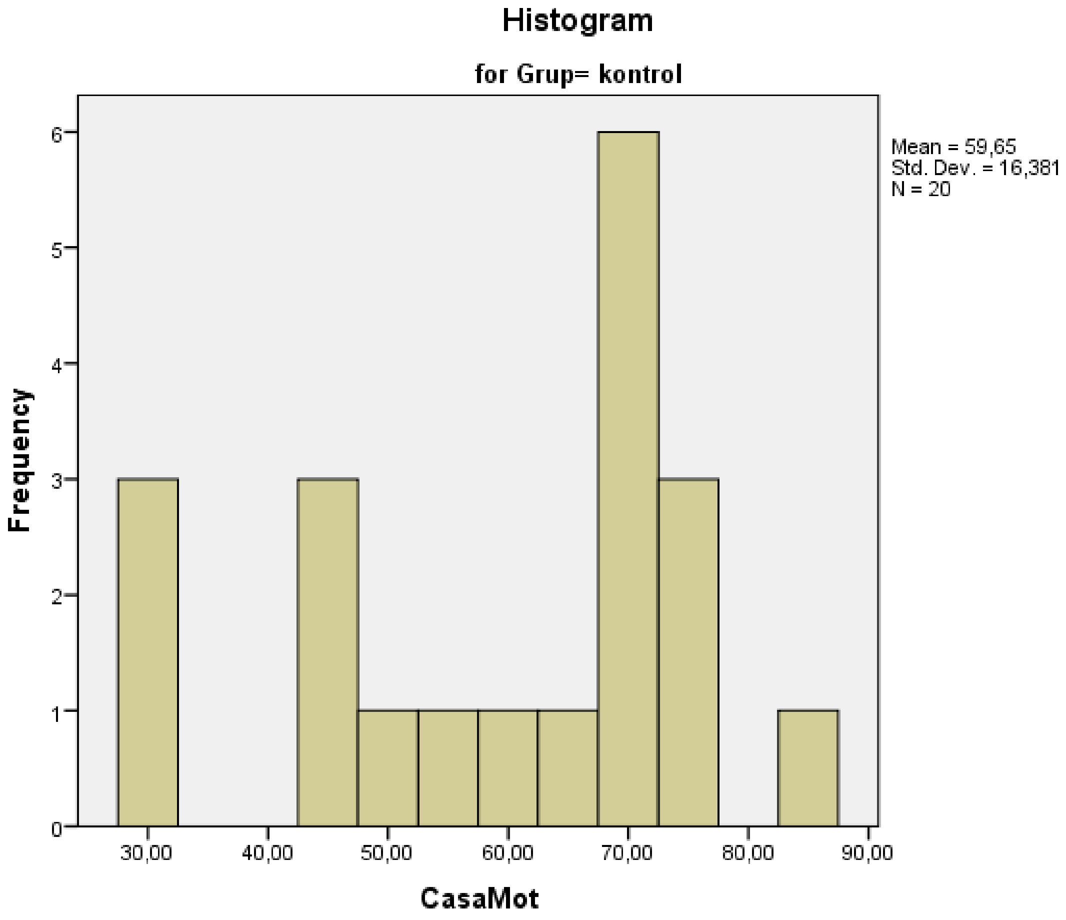
Histogram
for Grup= kontrol
Mean = 59,65
Std. Dev. = 16,381
N = 20
Frequency
0
1
2
3
4
5
6
30,00
40,00
50,00
60,00
70,00
80,00
90,00
CasaMot

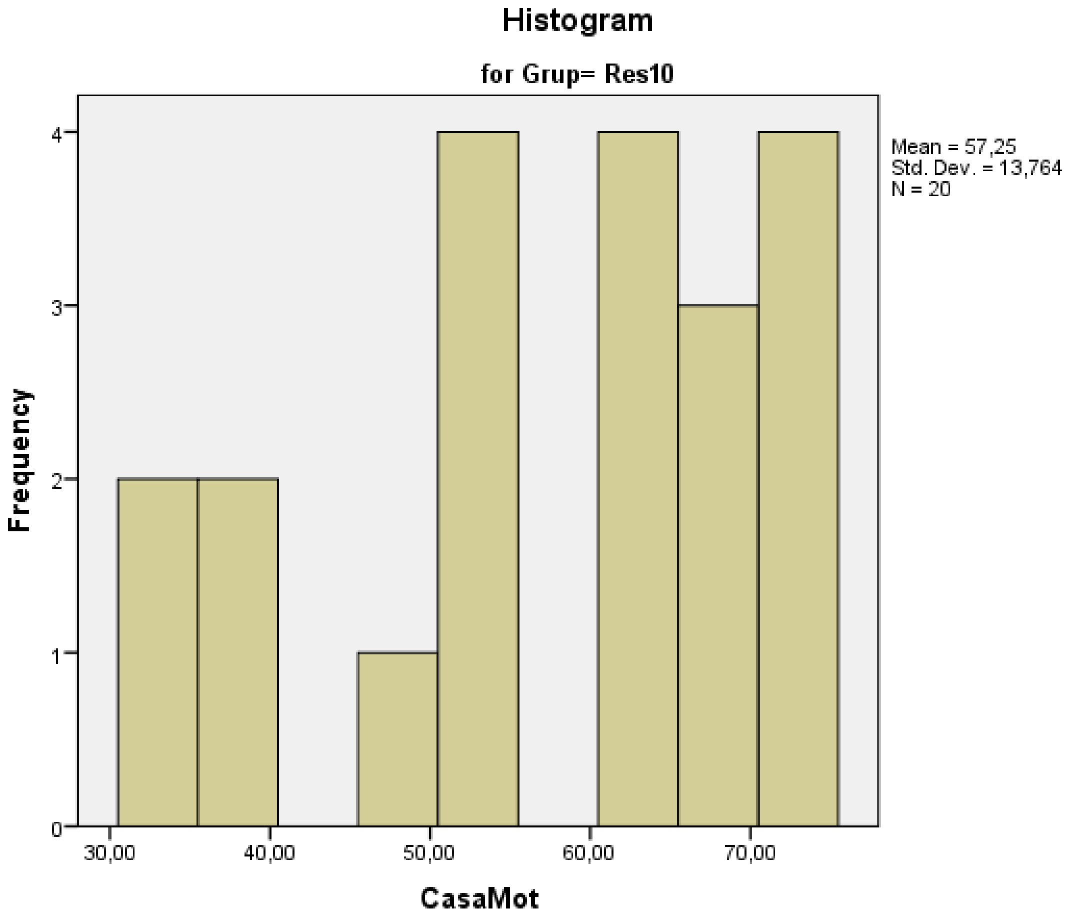

Histogram
for Grup= Res10
Mean = 57,25
Std. Dev. = 13,764
N = 20
Frequency
0
1
2
3
4
30,00
40,00
50,00
60,00
70,00
CasaMot

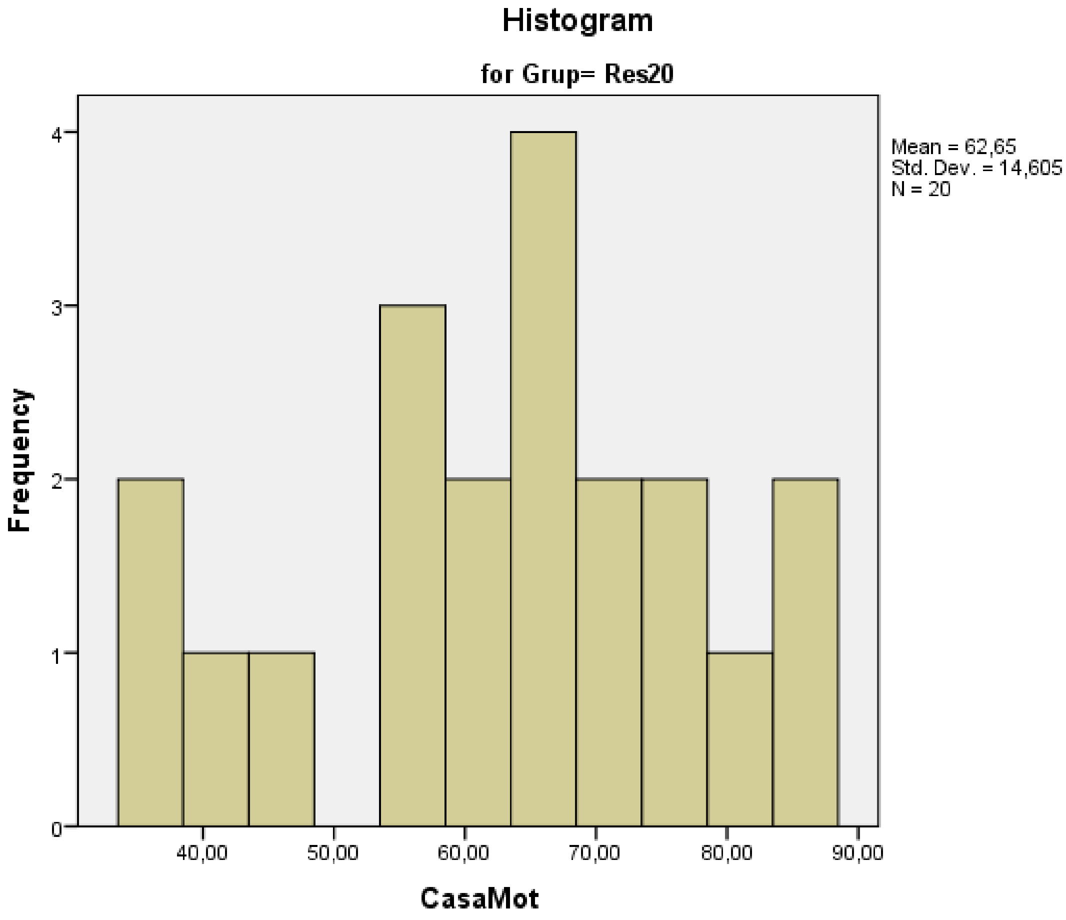
Histogram
for Grup= Res20
Frequency
CasaMot
0
1
2
3
4
40,00
50,00
60,00
70,00
80,00
90,00
Mean = 62,65
Std. Dev. = 14,605
N = 20

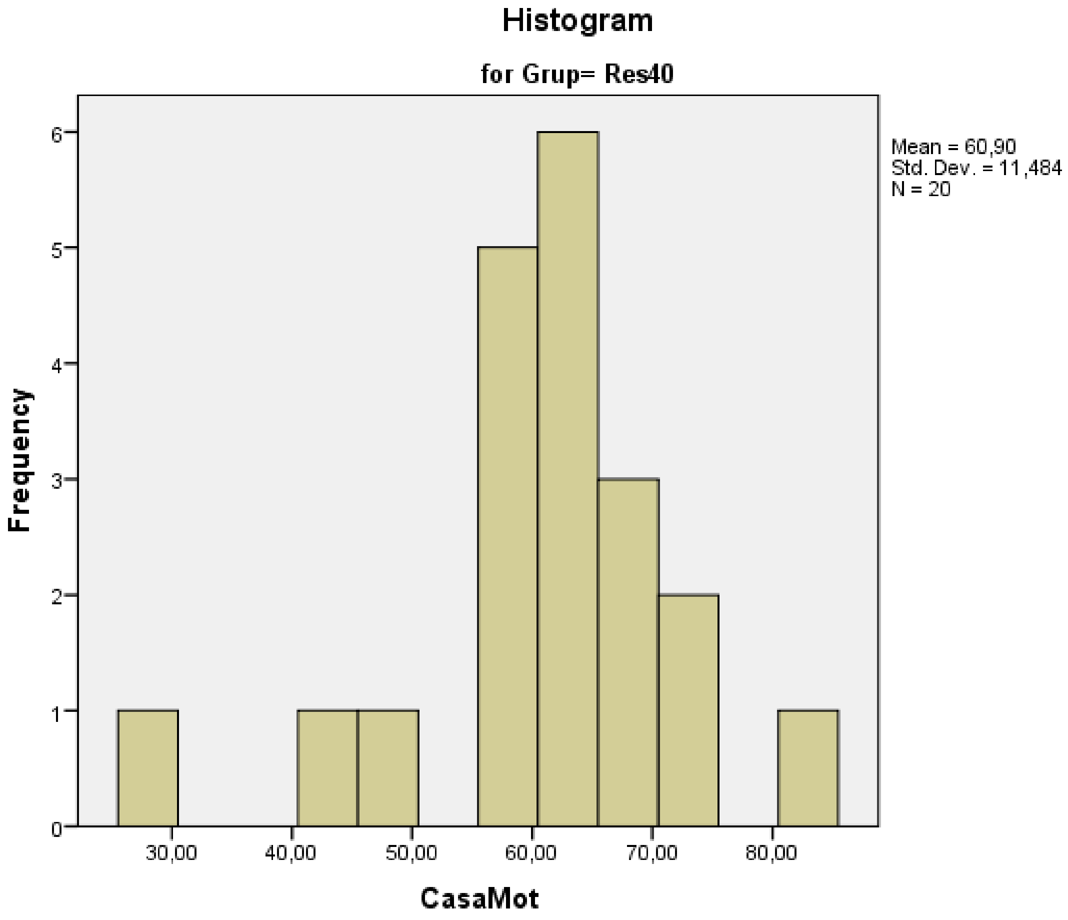

Histogram
for Grup= Res40
Mean = 60,90
Std. Dev. = 11,484
N = 20
Frequency
0
1
2
3
4
5
6
30,00
40,00
50,00
60,00
70,00
80,00
CasaMot

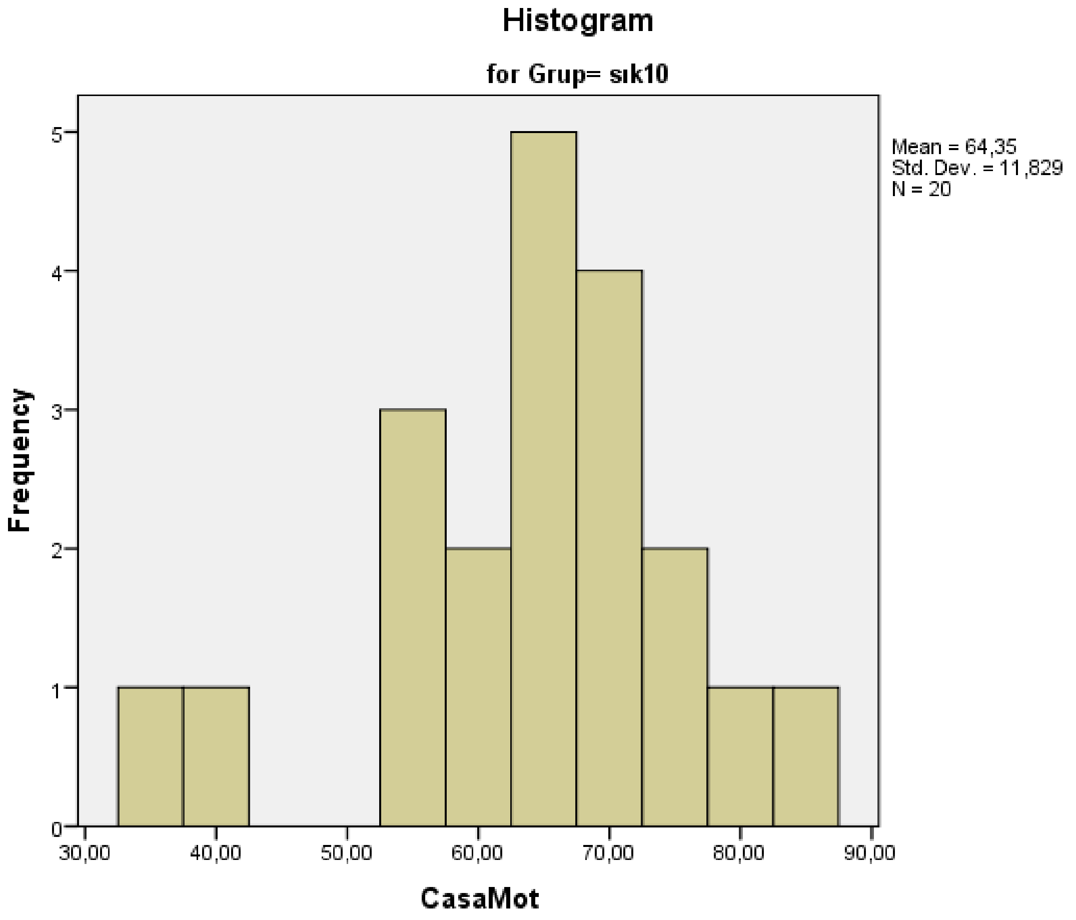
Histogram
for Grup= sık10
Mean = 64,35
Std. Dev. = 11,829
N = 20
Frequency
0
1
2
3
4
5
30,00
40,00
50,00
60,00
70,00
80,00
90,00
CasaMot

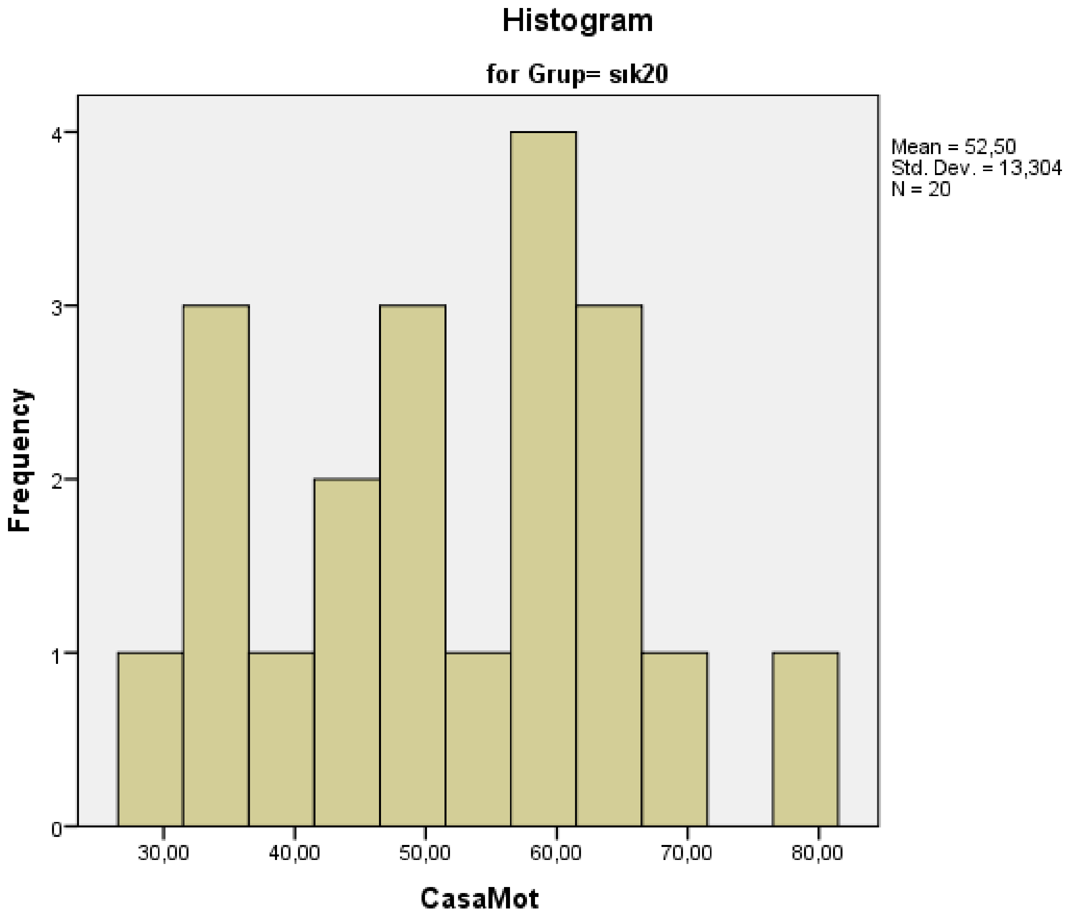
Histogram
for Grup= sık20
Mean = 52,50
Std. Dev. = 13,304
N = 20
Frequency
0
1
2
3
4
30,00
40,00
50,00
60,00
70,00
80,00
CasaMot

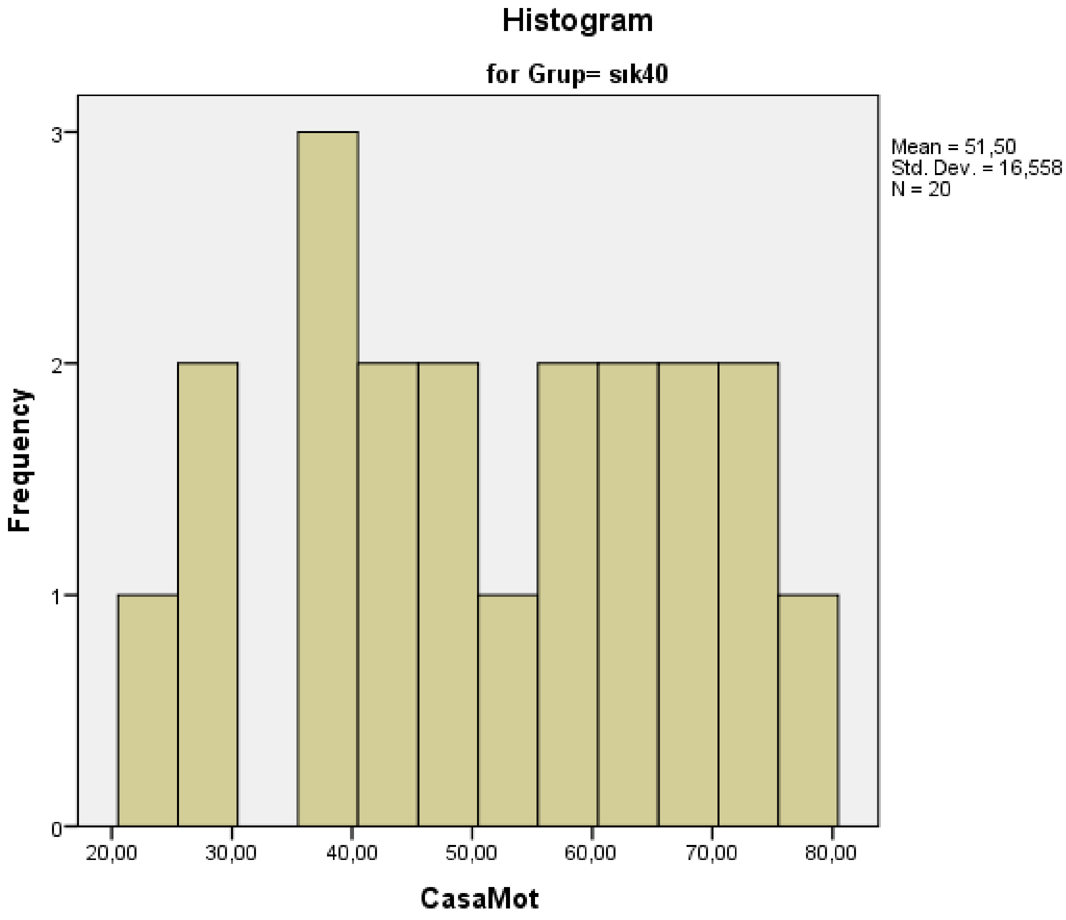
Histogram
for Grup= sık40
Mean = 51,50
Std. Dev. = 16,558
N = 20
Frequency
0
1
2
3
20,00
30,00
40,00
50,00
60,00
70,00
80,00
CasaMot

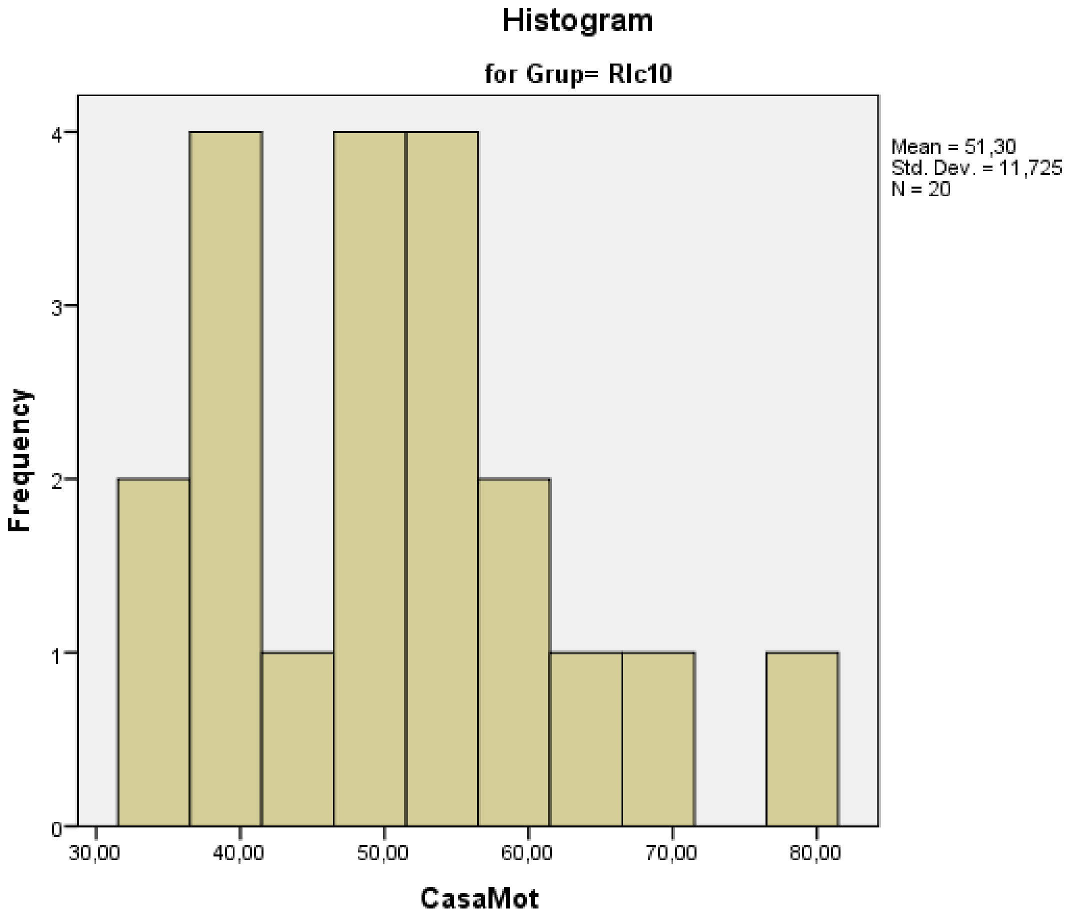
Histogram
for Grup= Rlc10
Mean = 51,30
Std. Dev. = 11,725
N = 20
Frequency
0
1
2
3
4
30,00
40,00
50,00
60,00
70,00
80,00
CasaMot

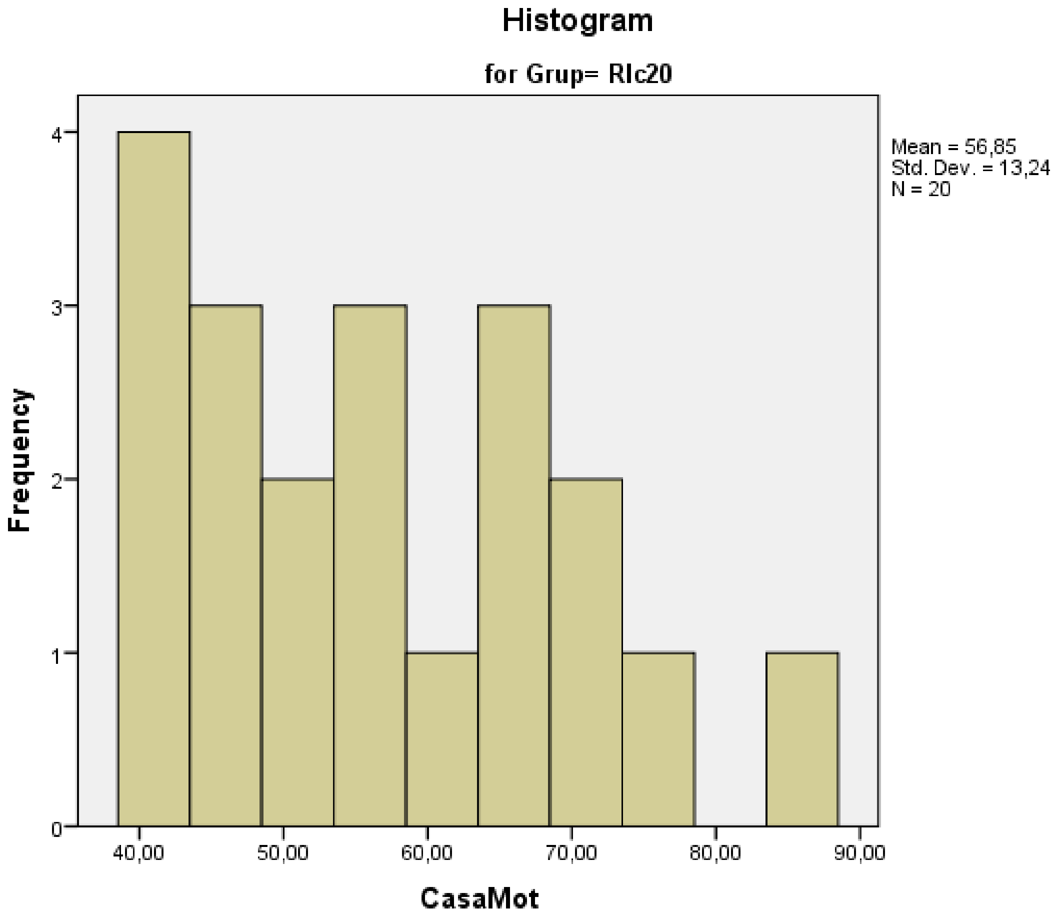
Histogram
for Grup= Rlc20
Mean = 56,85
Std. Dev. = 13,24
N = 20
Frequency
0
1
2
3
4
40,00
50,00
60,00
70,00
80,00
90,00
CasaMot

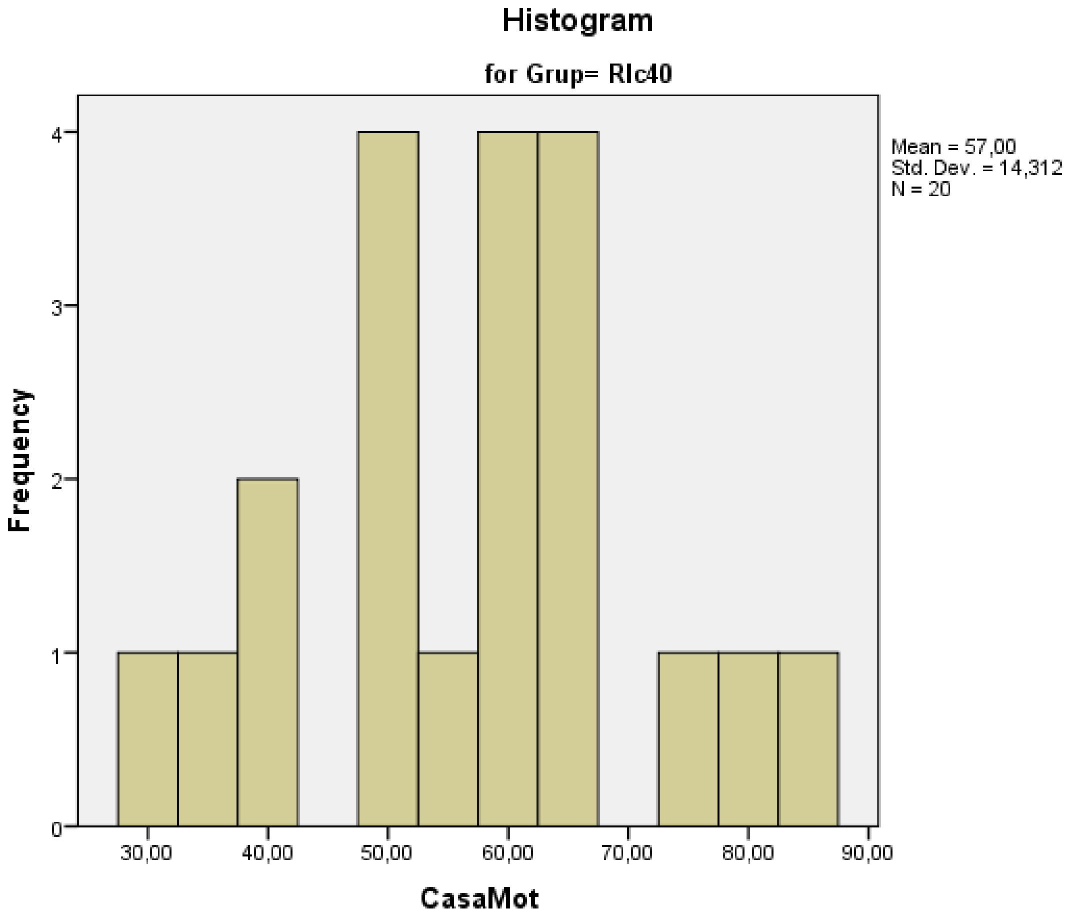

Stem-and-Leaf Plots

```
CasaMot Stem-and-Leaf Plot for
Grup= kontrol

 Frequency    Stem &  Leaf

     3,00        3 .  022
     3,00        4 .  466
     2,00        5 .  07
     3,00        6 .  149
     8,00        7 .  01122355
     1,00        8 .  3

 Stem width:     10,00
 Each leaf:       1 case(s)
```

CasaMot Stem-and-Leaf Plot for
Grup= Res10

```
 Frequency    Stem &  Leaf

     4,00        3 .  3378
     1,00        4 .  6
     4,00        5 .  1245
     7,00        6 .  3334678
     4,00        7 .  1245

 Stem width:     10,00
 Each leaf:       1 case(s)
```

CasaMot Stem-and-Leaf Plot for
Grup= Res20

```
 Frequency    Stem &  Leaf

     2,00        3 .  68
     2,00        4 .  16
     4,00        5 .  4679
     6,00        6 .  056689
     4,00        7 .  1479
     2,00        8 .  47

 Stem width:     10,00
 Each leaf:       1 case(s)
```

CasaMot Stem-and-Leaf Plot for
Grup= Res40

```
 Frequency    Stem &  Leaf

     1,00 Extremes    (=<28)
     2,00        4 .  58
     5,00        5 .  77789
     9,00        6 .  122245689
     2,00        7 .  45
     1,00        8 .  1

 Stem width:     10,00
 Each leaf:       1 case(s)
```

```
CasaMot Stem-and-Leaf Plot for
Grup= sık10

 Frequency    Stem &  Leaf

     1,00 Extremes    (=<35)
     1,00        4 .  2
     3,00        5 .  347
     9,00        6 .  026666788
     5,00        7 .  22568
     1,00        8 .  4

 Stem width:     10,00
 Each leaf:       1 case(s)
```

```
CasaMot Stem-and-Leaf Plot for
Grup= sık20

 Frequency    Stem &  Leaf

     1,00        2 .  9
     3,00        3 .  555
     3,00        4 .  056
     5,00        5 .  00058
     6,00        6 .  001246
     2,00        7 .  18

 Stem width:     10,00
 Each leaf:       1 case(s)
```

```
CasaMot Stem-and-Leaf Plot for
Grup= sık40

 Frequency    Stem &  Leaf

     2,00        2 .  37
     3,00        3 .  066
     5,00        4 .  01578
     2,00        5 .  39
     5,00        6 .  03368
     3,00        7 .  456

 Stem width:     10,00
 Each leaf:       1 case(s)
```

```
CasaMot Stem-and-Leaf Plot for
Grup= Rlc10
```

```
 Frequency    Stem &  Leaf

     2,00        3 .  45
     6,00        4 .  000049
     7,00        5 .  0005566
     3,00        6 .  012
     2,00        7 .  09

 Stem width:     10,00
 Each leaf:       1 case(s)
```

```
CasaMot Stem-and-Leaf Plot for
Grup= Rlc20

 Frequency    Stem &  Leaf

     7,00        4 .  1122556
     5,00        5 .  03467
     4,00        6 .  3458
     3,00        7 .  225
     1,00        8 .  6

 Stem width:     10,00
 Each leaf:       1 case(s)
```

```
CasaMot Stem-and-Leaf Plot for
Grup= Rlc40

 Frequency    Stem &  Leaf

     3,00        3 .  059
     2,00        4 .  19
     6,00        5 .  012699
     6,00        6 .  113346
     2,00        7 .  78
     1,00 Extremes    (>=86)

 Stem width:     10,00
 Each leaf:       1 case(s)
```

Normal Q-Q Plots

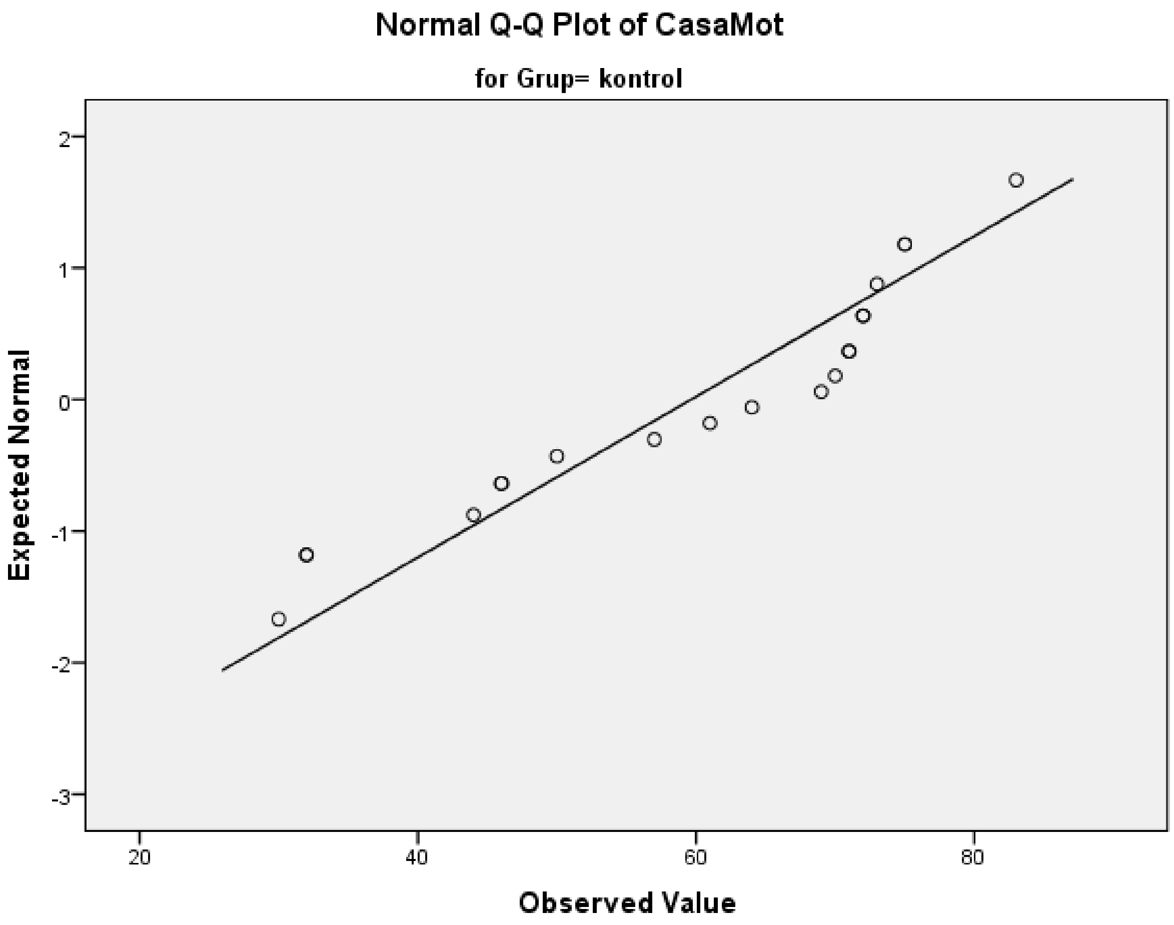
Normal Q-Q Plot of CasaMot
for Grup= kontrol
Expected Normal
2
1
0
-1
-2
-3
20
40
60
80
Observed Value

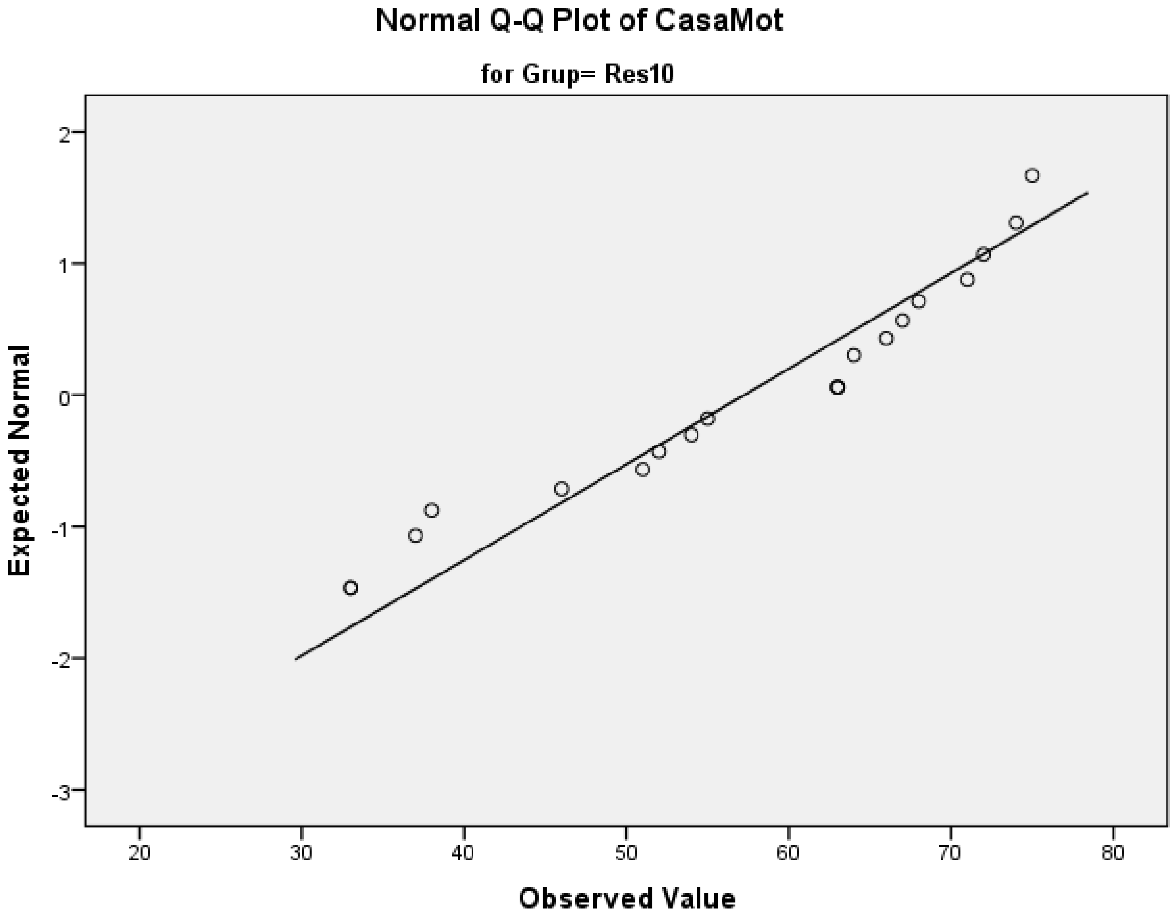
Normal Q-Q Plot of CasaMot
for Grup= Res10
Expected Normal
2
1
0
-1
-2
-3
20
30
40
50
60
70
80
Observed Value

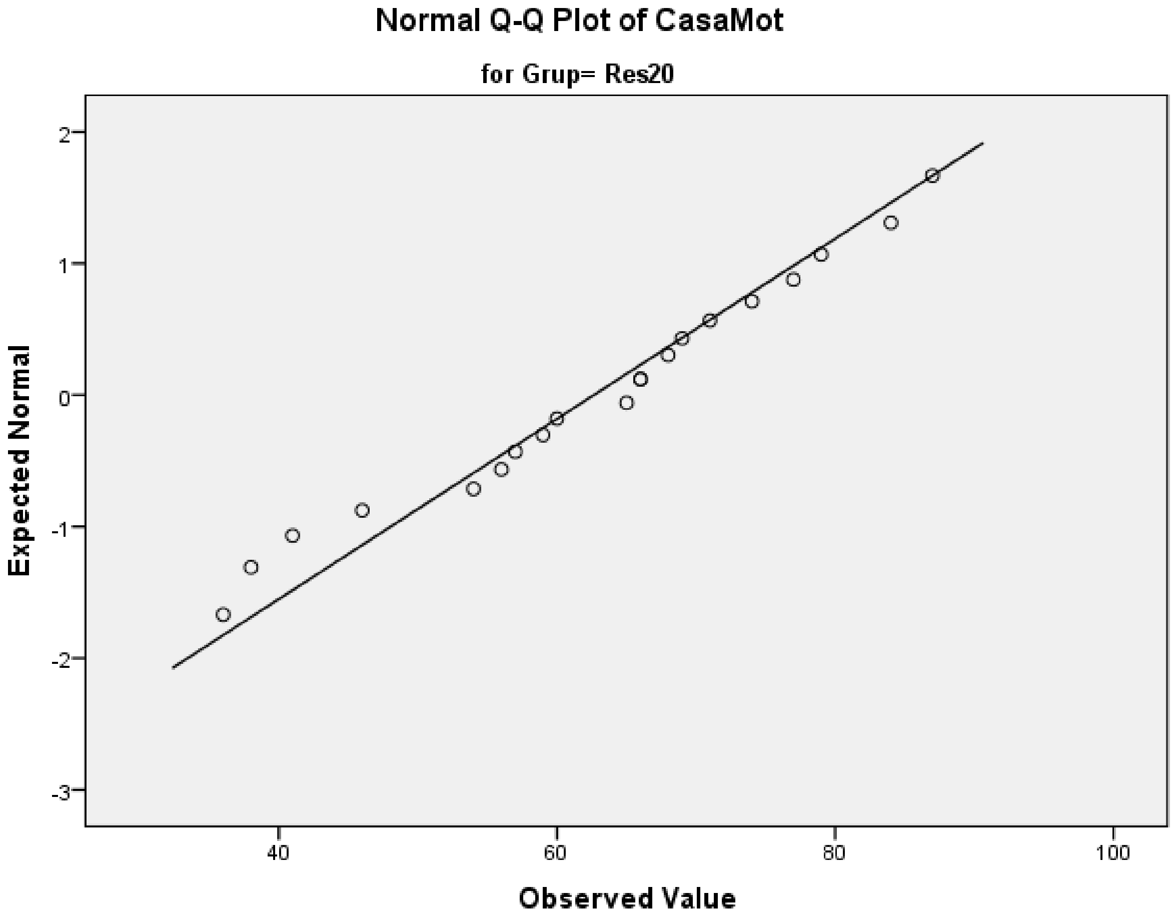

Normal Q-Q Plot of CasaMot
for Grup= Res20
Expected Normal
Observed Value
2
1
0
-1
-2
-3
40
60
80
100

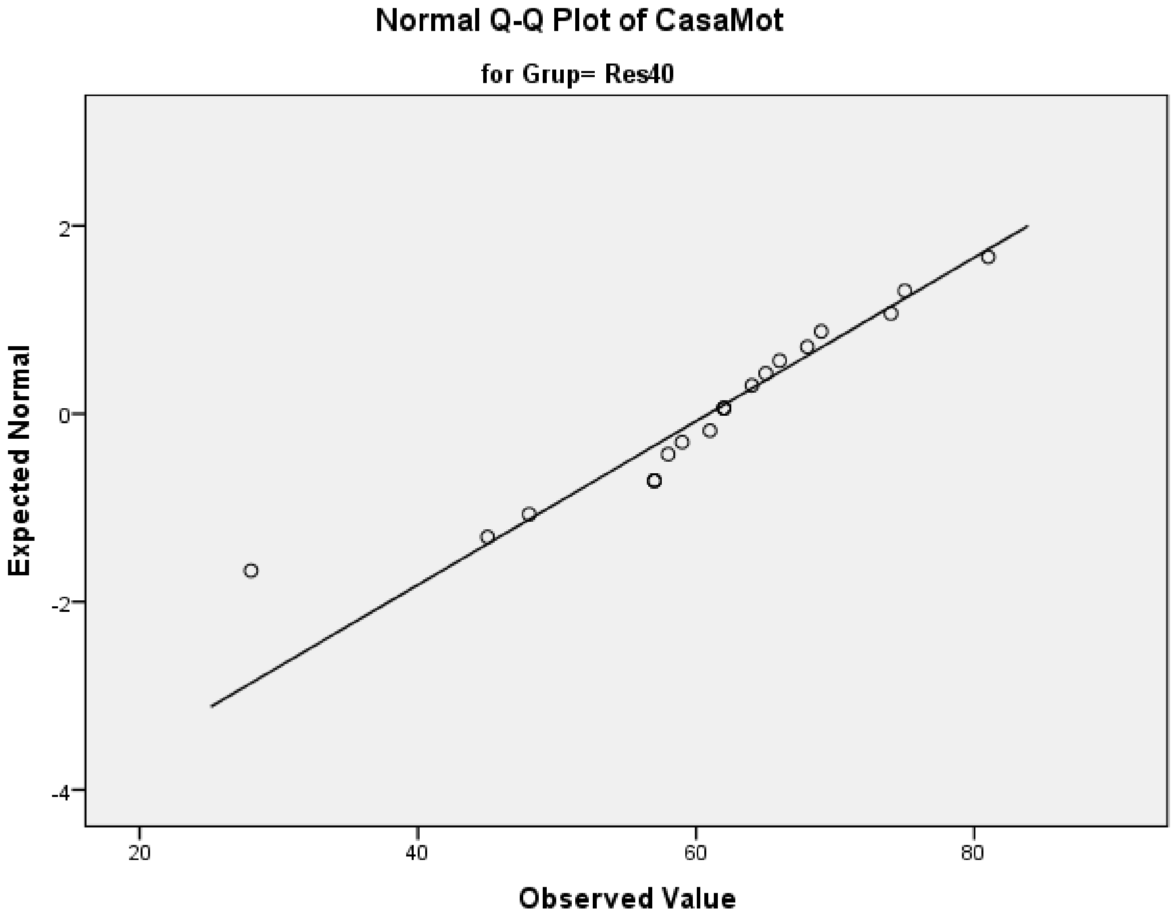

Normal Q-Q Plot of CasaMot
for Grup= Res40
Expected Normal
Observed Value
2
0
-2
-4
20
40
60
80

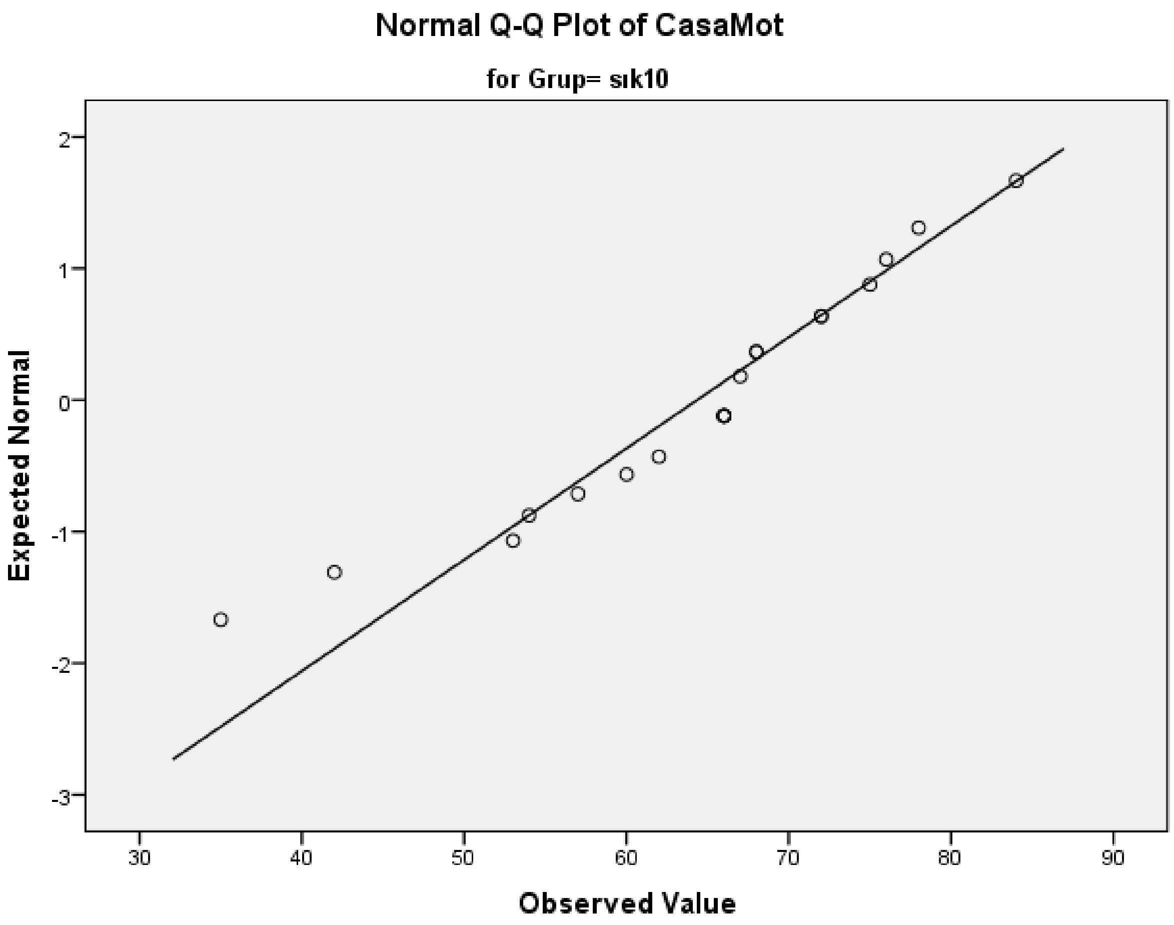
Normal Q-Q Plot of CasaMot
for Grup= sık10
Expected Normal
Observed Value
2
1
0
-1
-2
-3
30
40
50
60
70
80
90

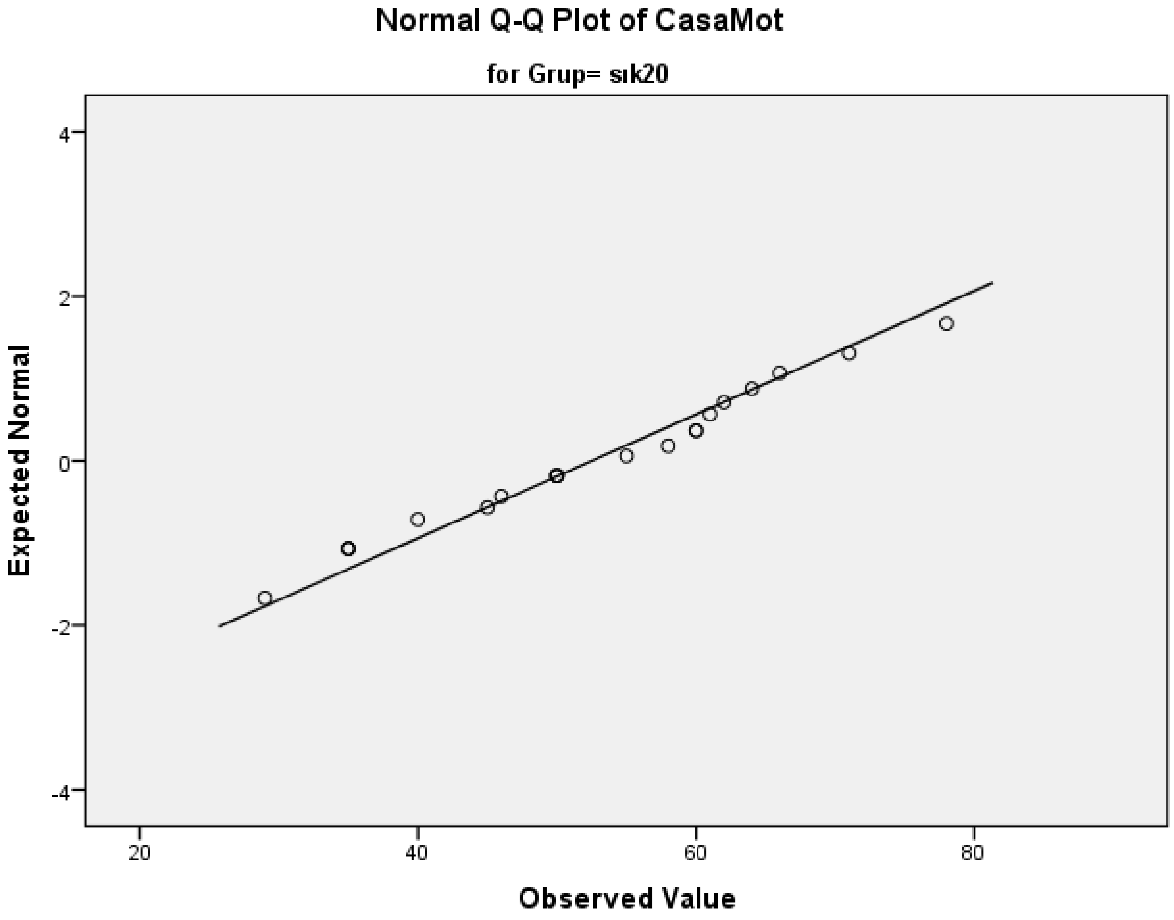
Normal Q-Q Plot of CasaMot
for Grup= sık20
Expected Normal
Observed Value
4
2
0
-2
-4
20
40
60
80

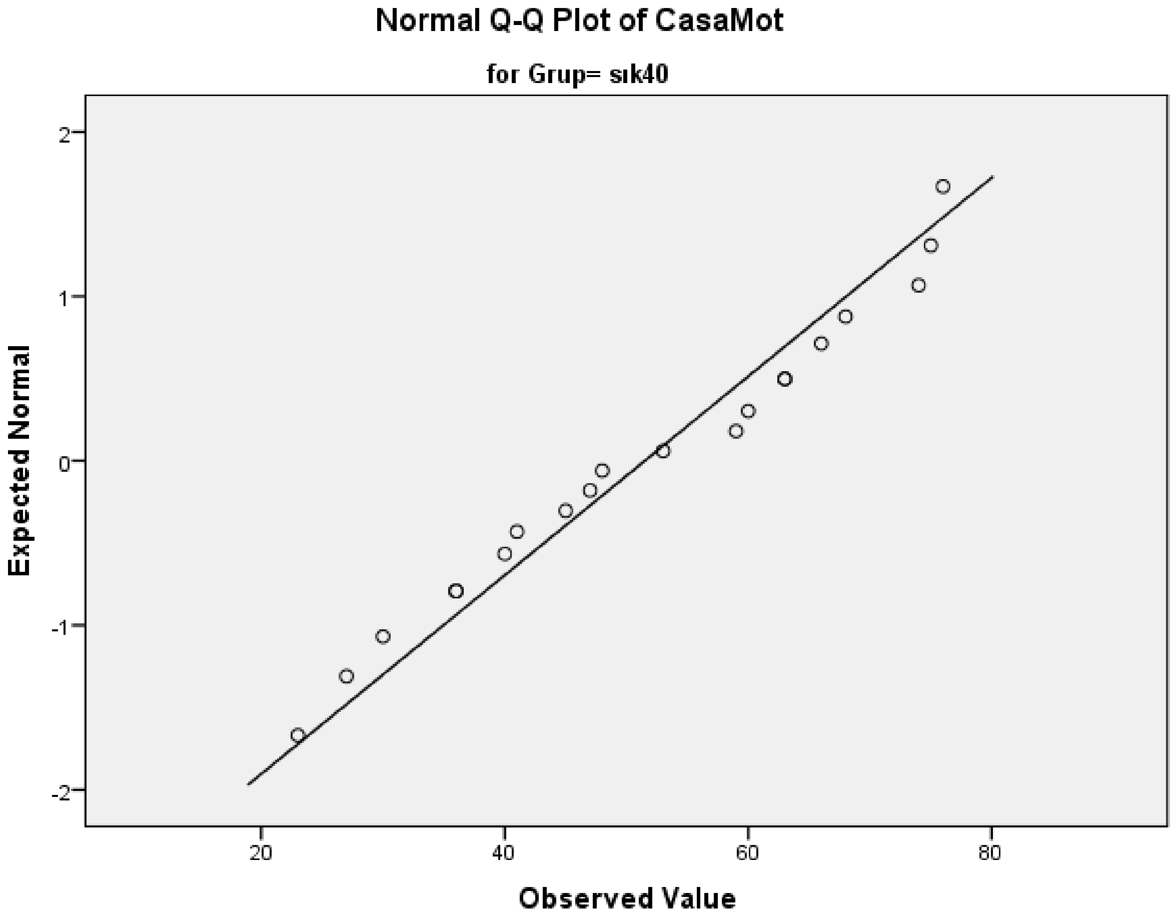
Normal Q-Q Plot of CasaMot
for Grup= sık40
Expected Normal
Observed Value
2
1
0
-1
-2
20
40
60
80

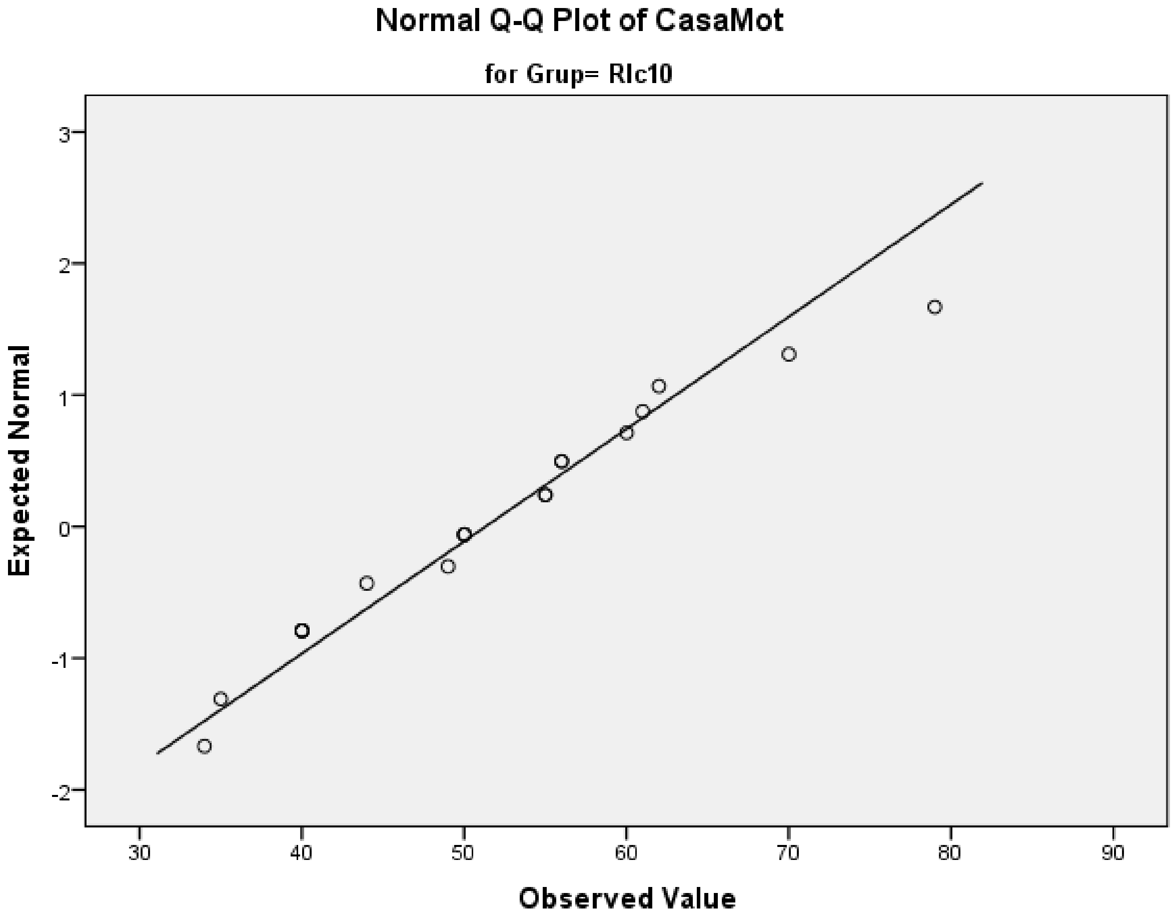

Normal Q-Q Plot of CasaMot
for Grup= RIc10
Expected Normal
Observed Value
3
2
1
0
-1
-2
30
40
50
60
70
80
90

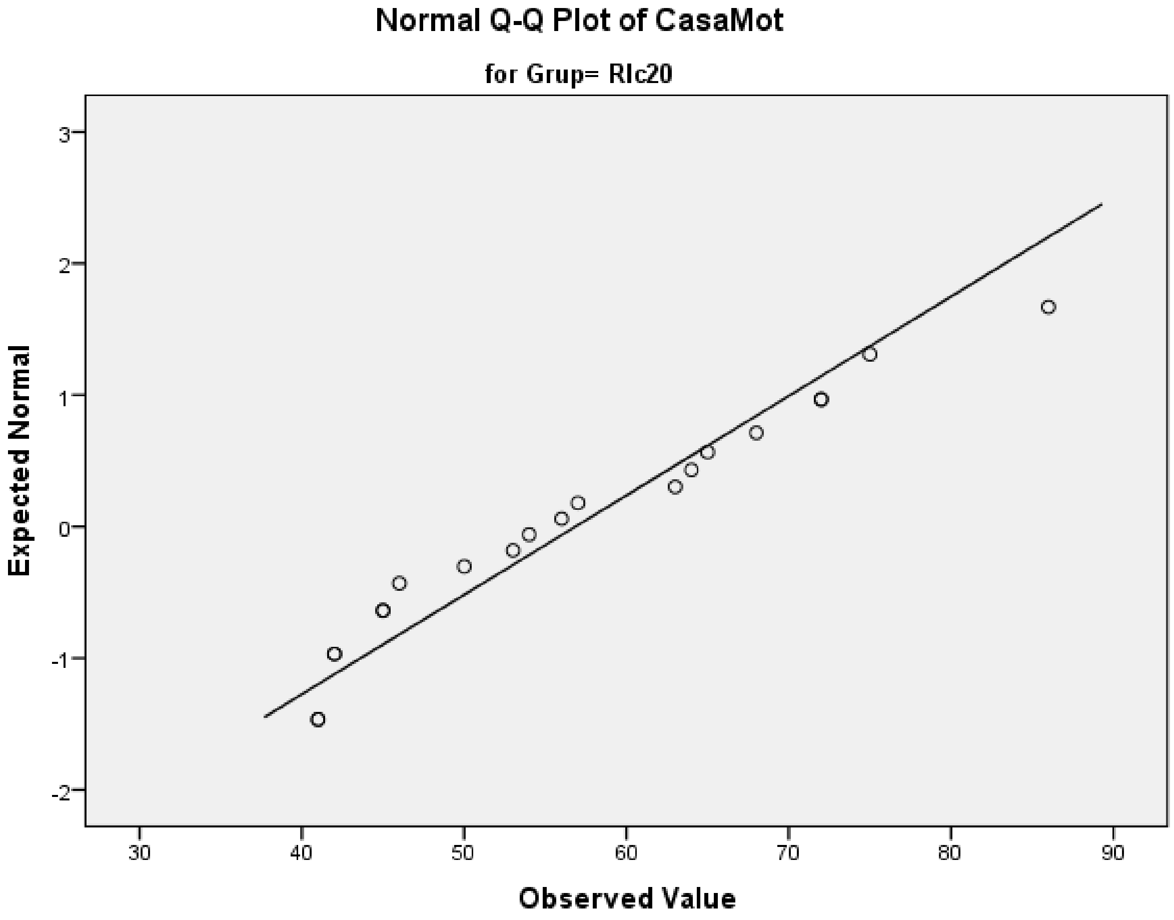

Normal Q-Q Plot of CasaMot
for Grup= RIc20
Expected Normal
Observed Value
3
2
1
0
-1
-2
30
40
50
60
70
80
90

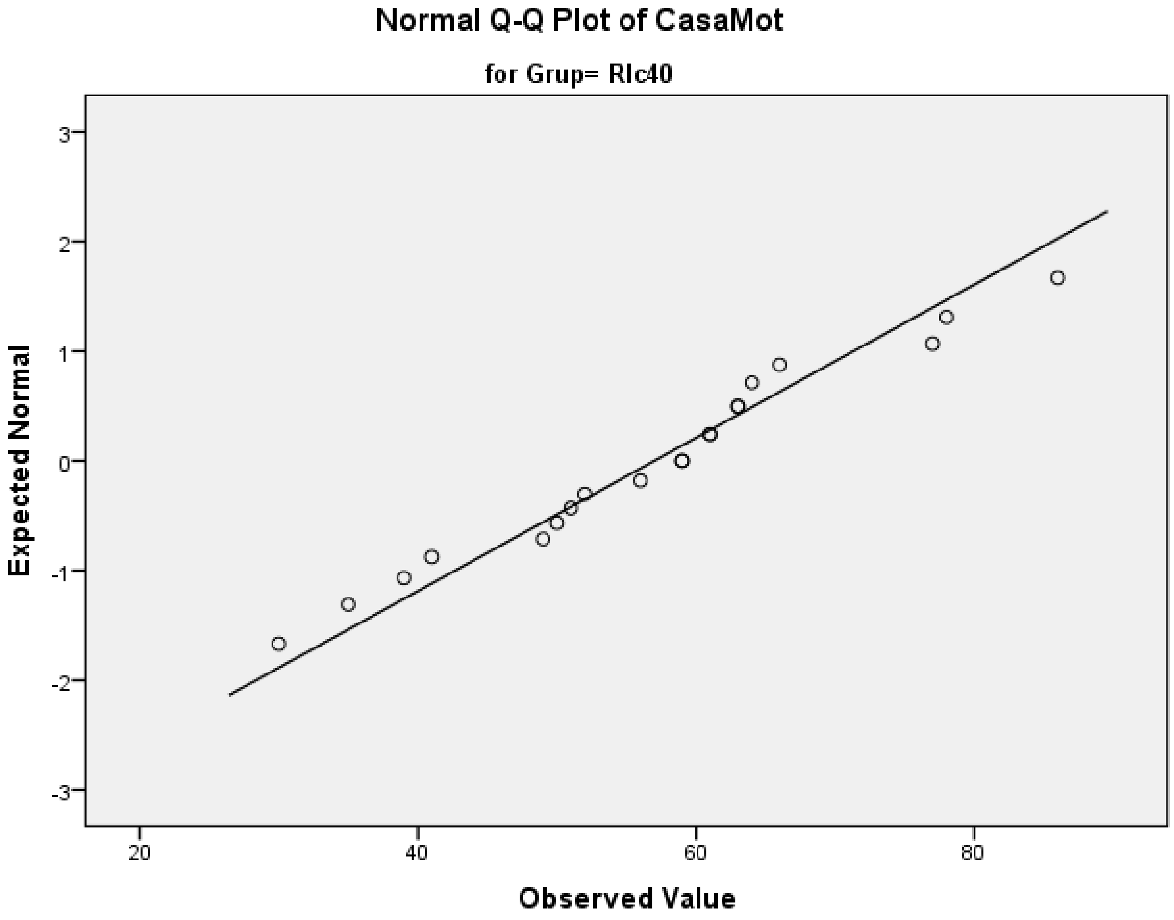

Detrended Normal Q-Q Plots

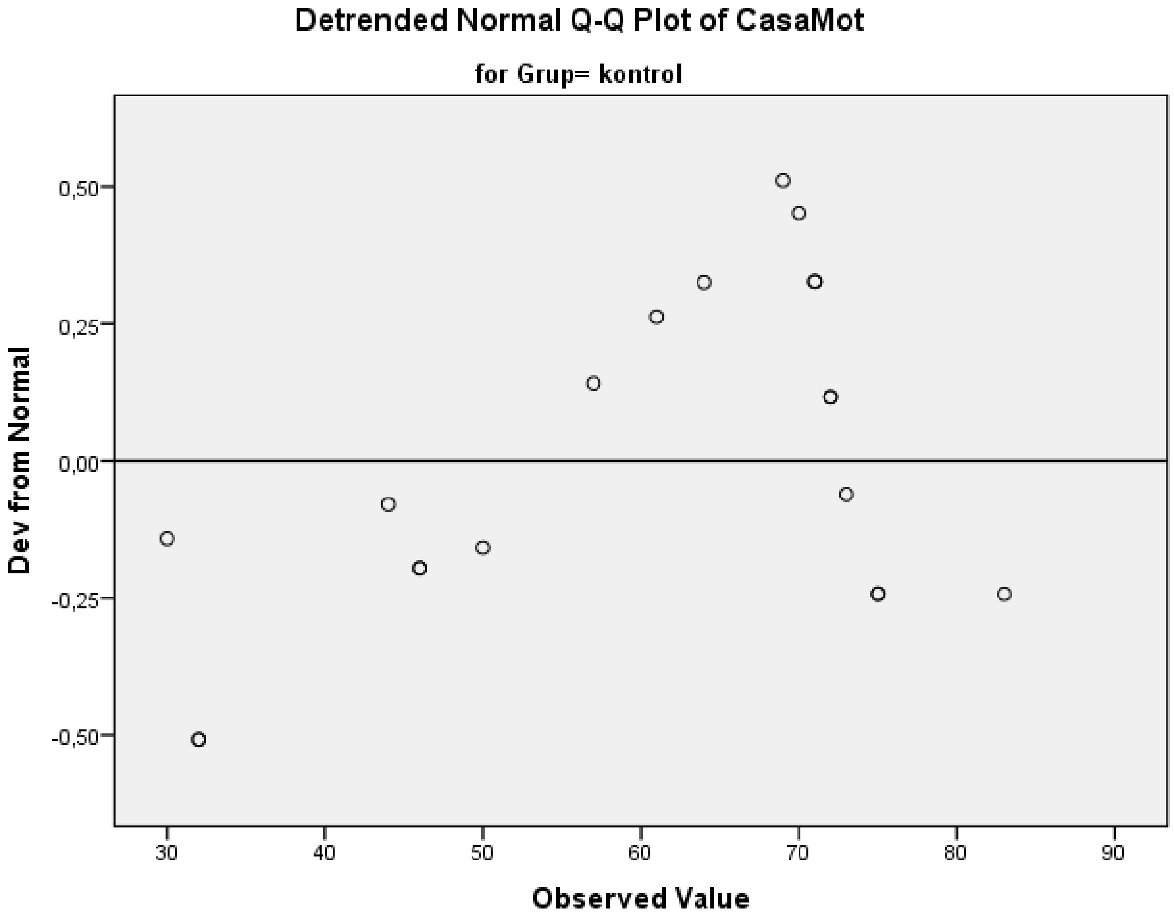

Detrended Normal Q-Q Plot of CasaMot
for Grup= kontrol
Dev from Normal
0,50
0,25
0,00
-0,25
-0,50
30
40
50
60
70
80
90
Observed Value

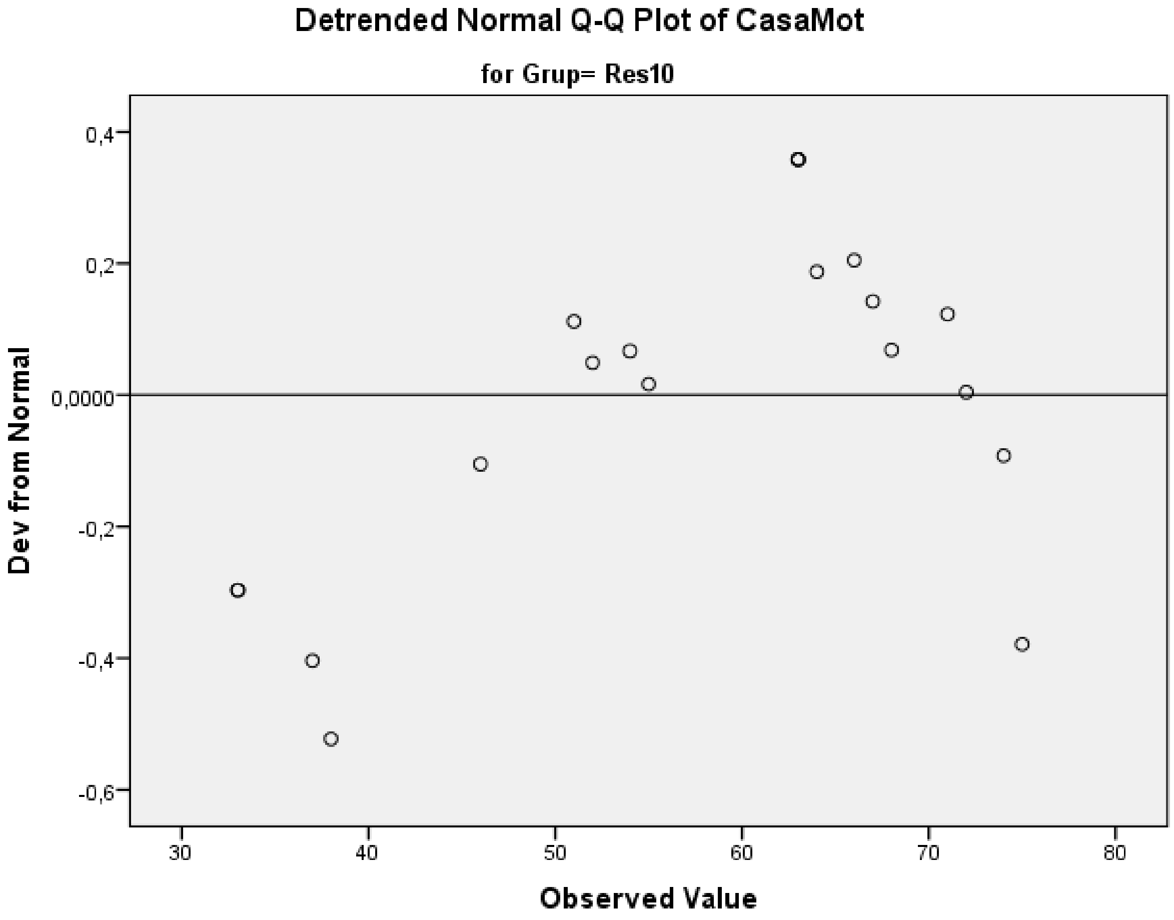
Detrended Normal Q-Q Plot of CasaMot
for Grup= Res10
Dev from Normal
0,4
0,2
0,0000
-0,2
-0,4
-0,6
30
40
50
60
70
80
Observed Value

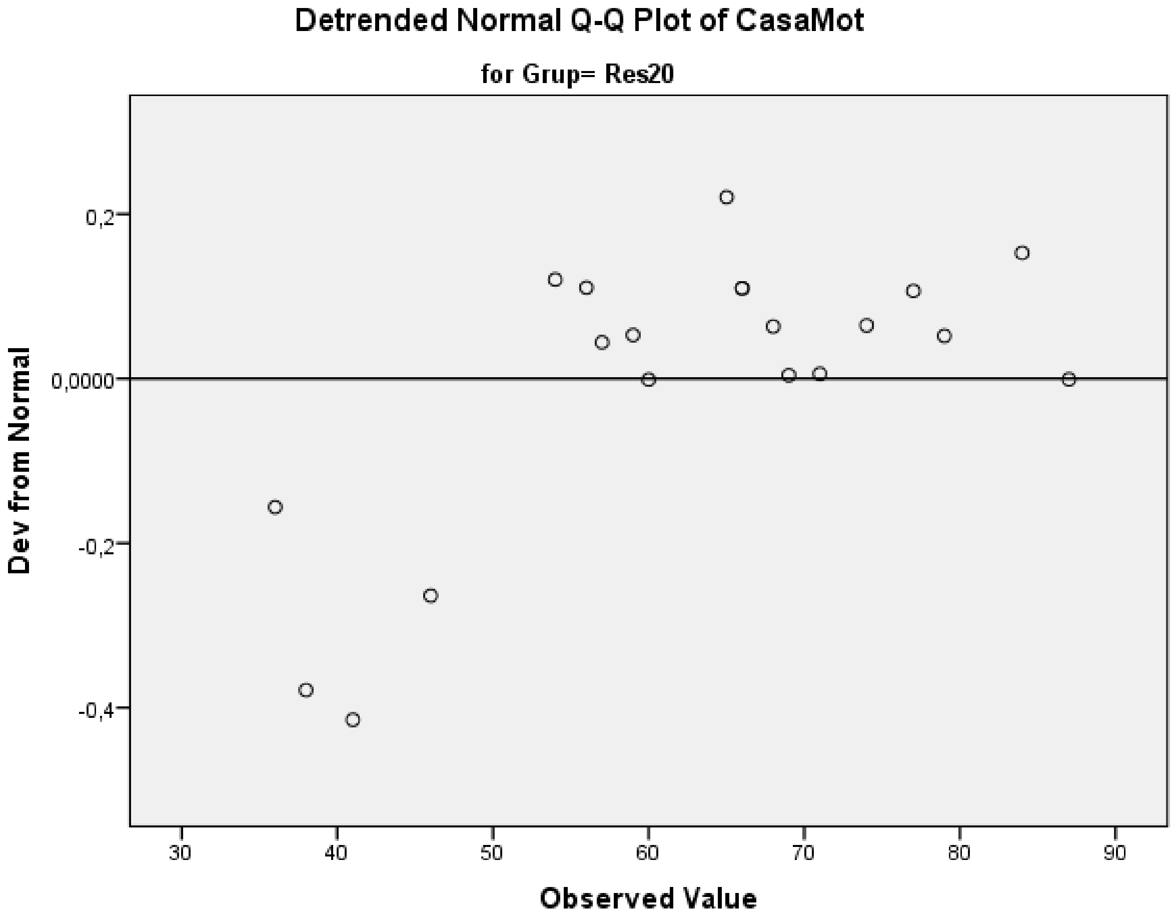
Detrended Normal Q-Q Plot of CasaMot
for Grup= Res20
Dev from Normal
0,2
0,0000
-0,2
-0,4
30
40
50
60
70
80
90
Observed Value

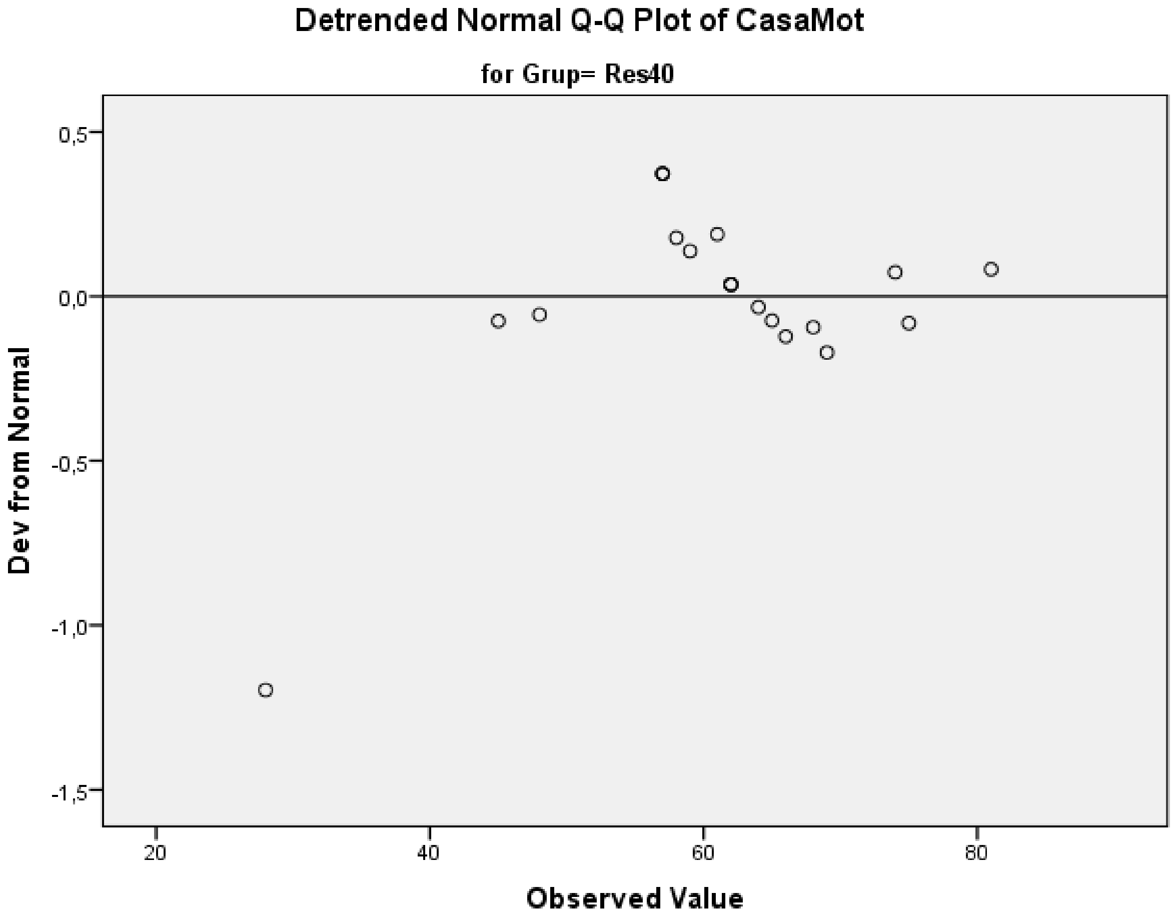

Detrended Normal Q-Q Plot of CasaMot
for Grup= Res40
0,5
0,0
-0,5
-1,0
-1,5
Dev from Normal
20
40
60
80
Observed Value

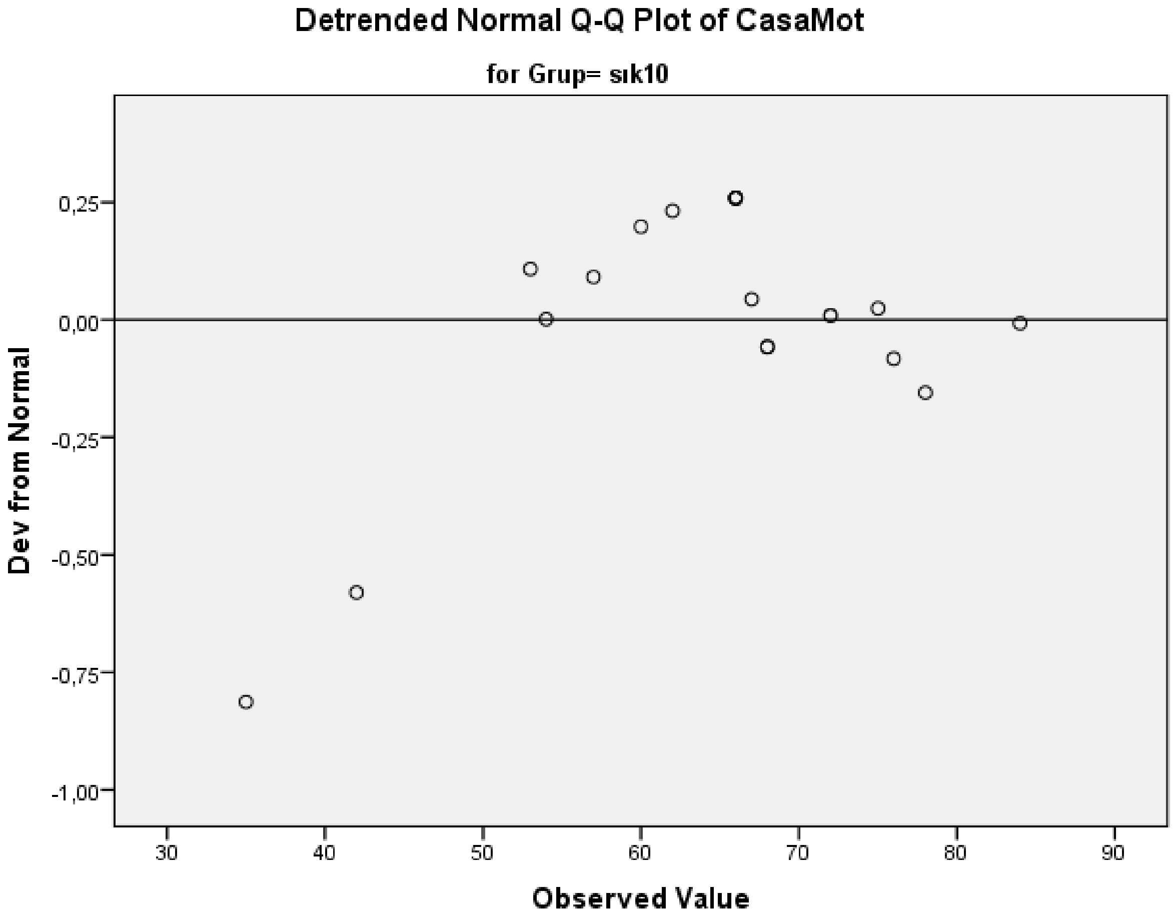
Detrended Normal Q-Q Plot of CasaMot
for Grup= sık10
Dev from Normal
0,25
0,00
-0,25
-0,50
-0,75
-1,00
30
40
50
60
70
80
90
Observed Value

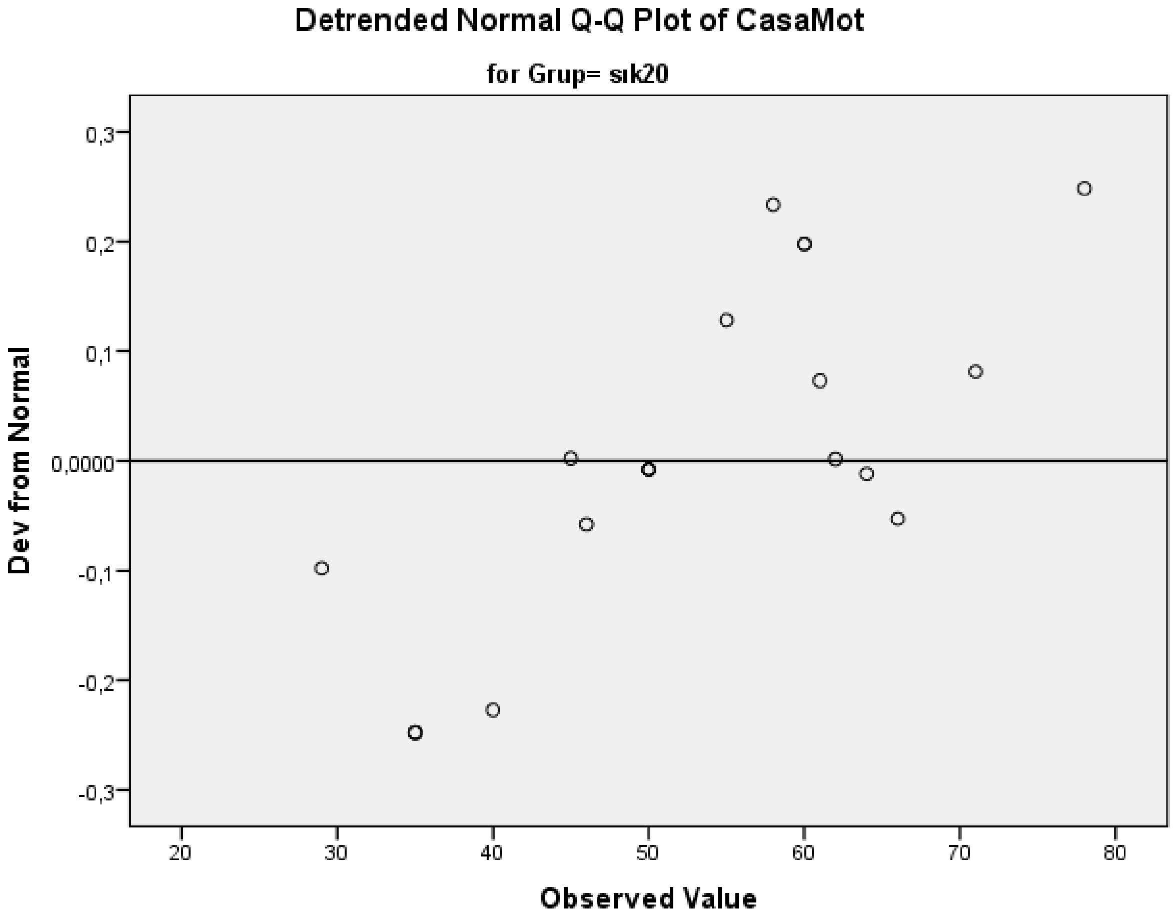

Detrended Normal Q-Q Plot of CasaMot
for Grup= sık20
Dev from Normal
0,3
0,2
0,1
0,0000
-0,1
-0,2
-0,3
20
30
40
50
60
70
80
Observed Value

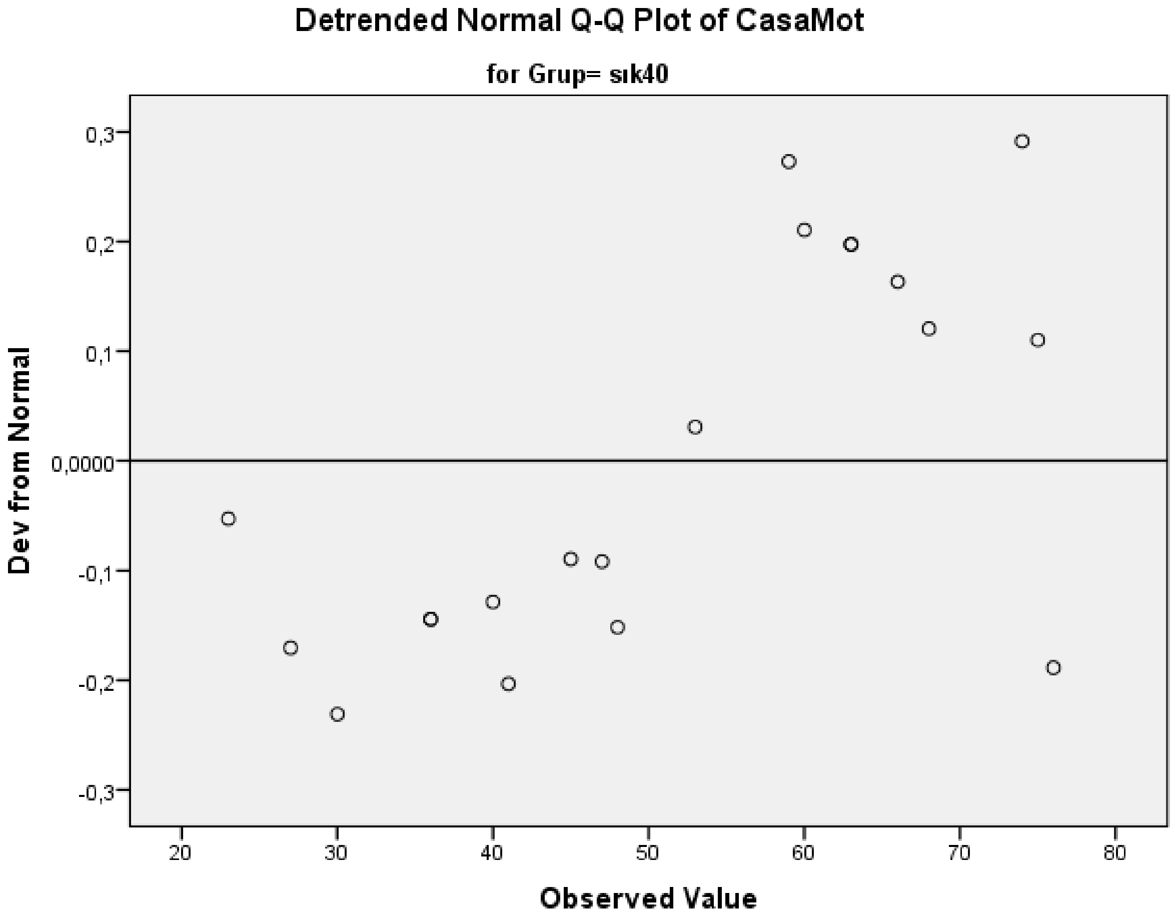
Detrended Normal Q-Q Plot of CasaMot
for Grup= sık40
Dev from Normal
0,3
0,2
0,1
0,0000
-0,1
-0,2
-0,3
20
30
40
50
60
70
80
Observed Value

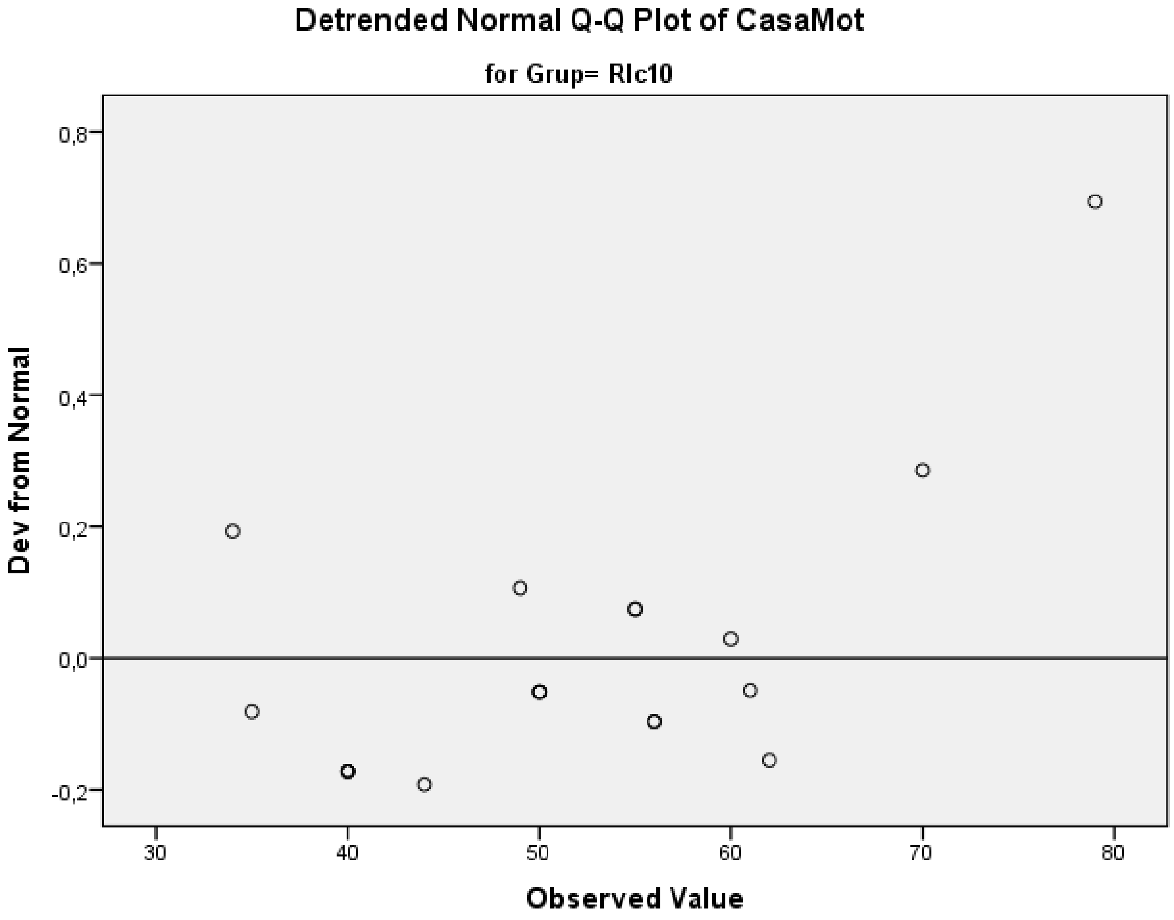

Detrended Normal Q-Q Plot of CasaMot
for Grup= Rlc10
Dev from Normal
Observed Value
0,8
0,6
0,4
0,2
0,0
-0,2
30
40
50
60
70
80

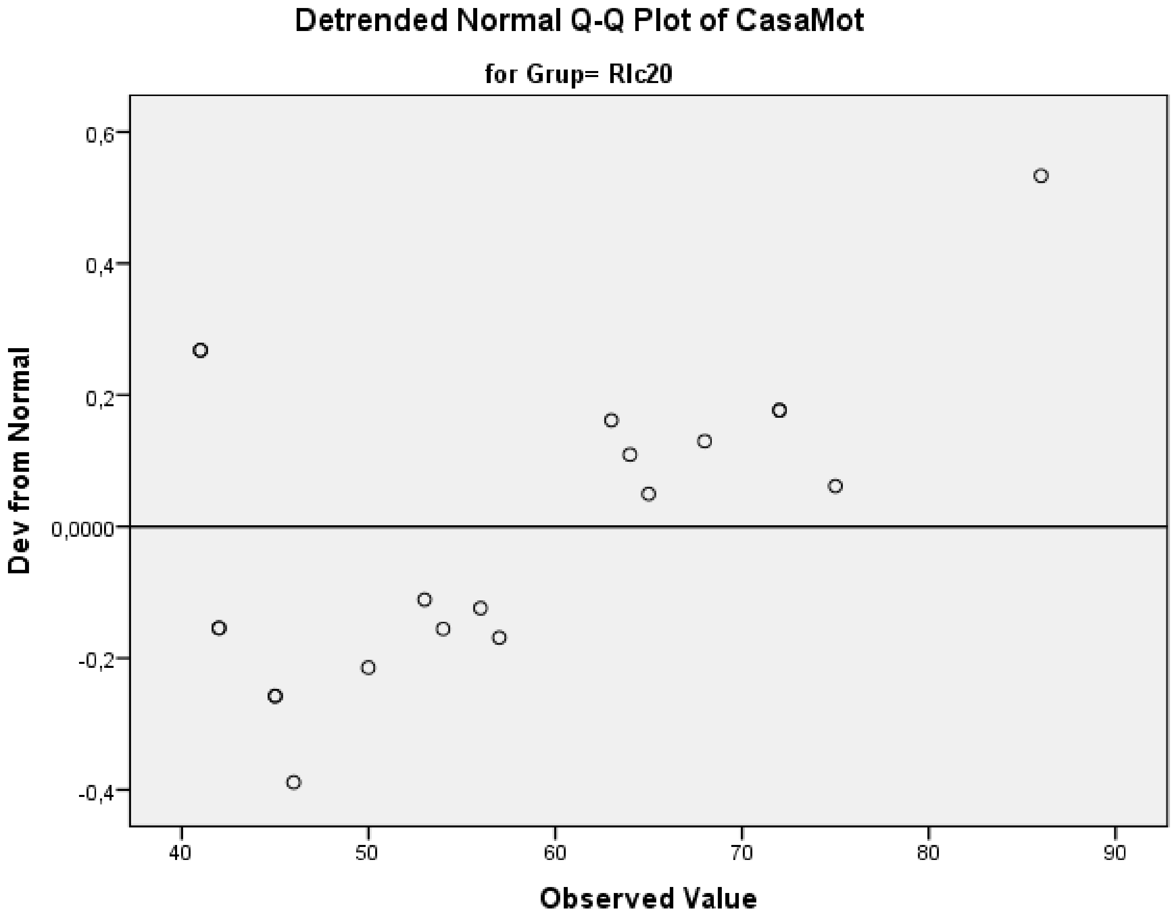

Detrended Normal Q-Q Plot of CasaMot
for Grup= RIc20
Dev from Normal
0,6
0,4
0,2
0,0000
-0,2
-0,4
40
50
60
70
80
90
Observed Value

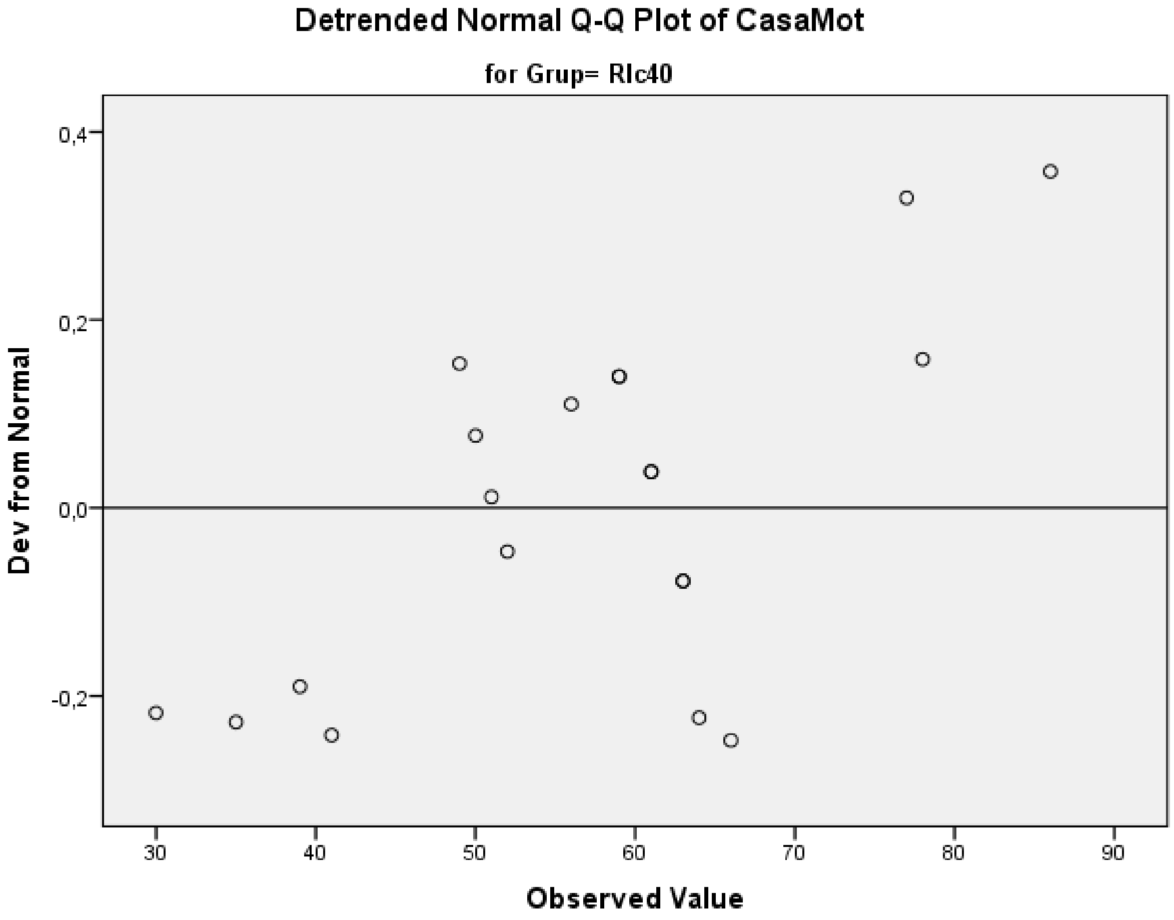

Detrended Normal Q-Q Plot of CasaMot
for Grup= Rlc40
Dev from Normal
0,4
0,2
0,0
-0,2
30
40
50
60
70
80
90
Observed Value

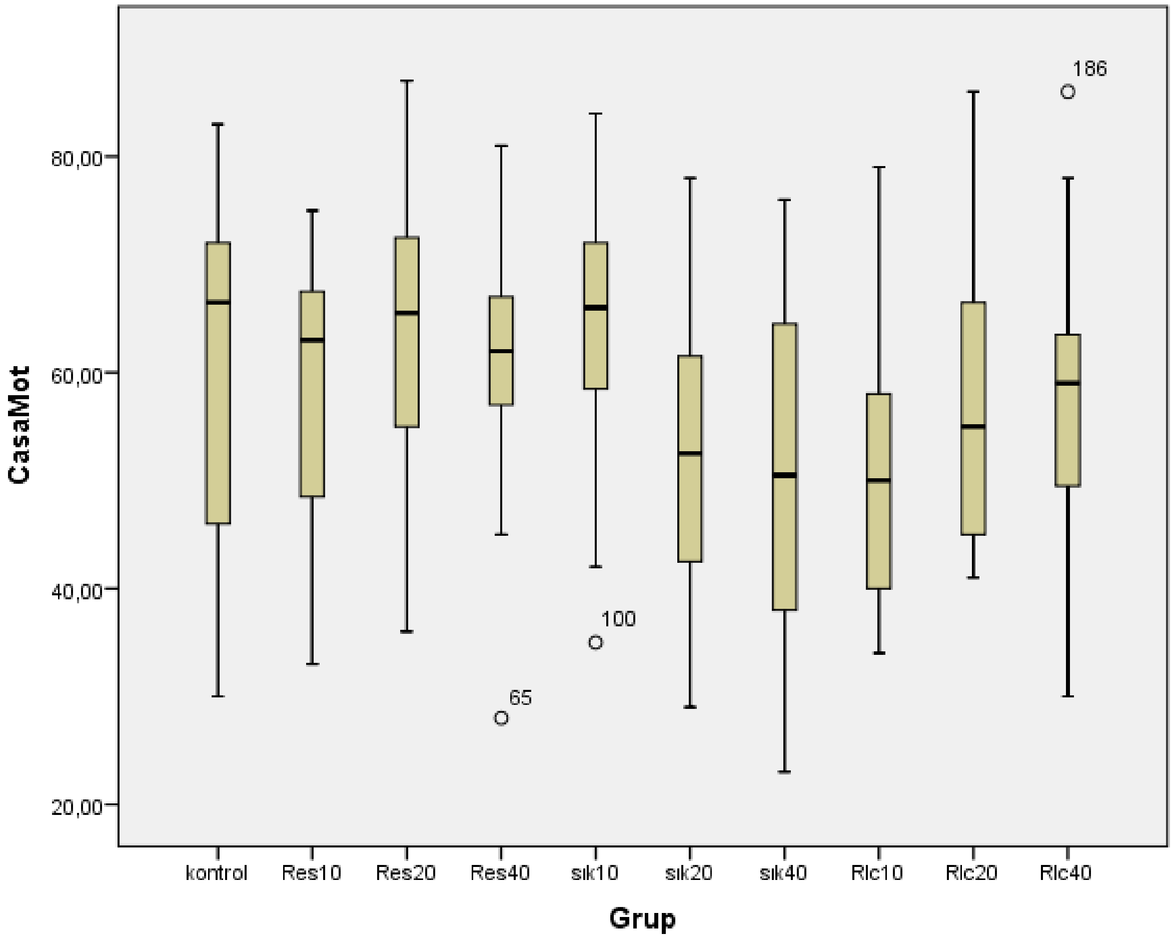

ProgMot

Histograms

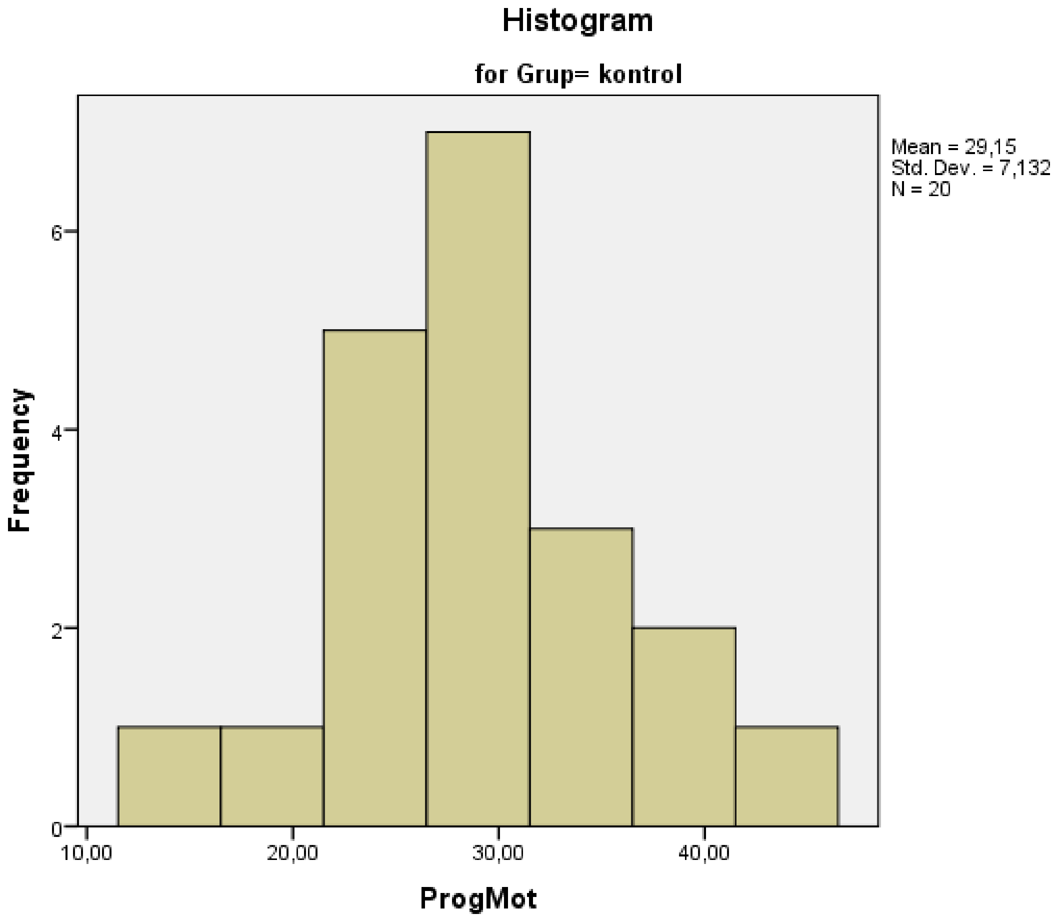
Histogram
for Grup= kontrol
Mean = 29,15
Std. Dev. = 7,132
N = 20
Frequency
0
2
4
6
10,00
20,00
30,00
40,00
ProgMot

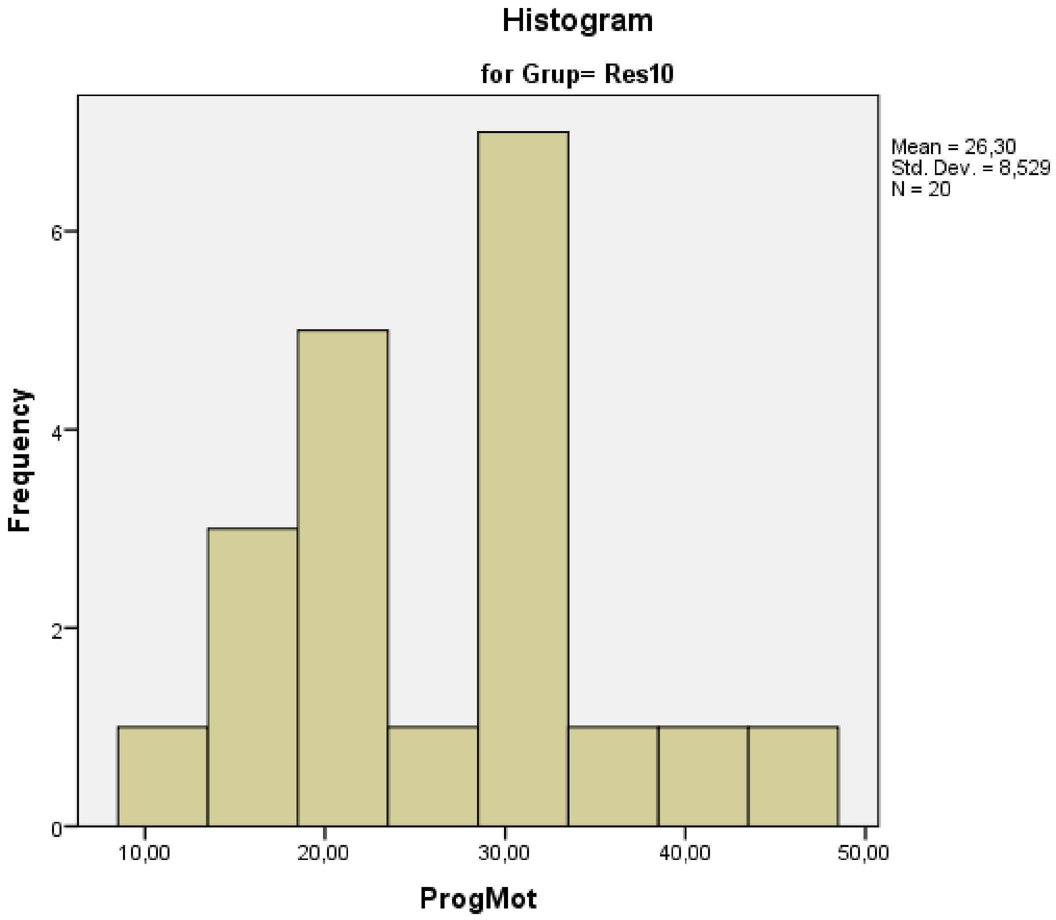

Histogram
for Grup= Res10
Mean = 26,30
Std. Dev. = 8,529
N = 20
Frequency
0
2
4
6
10,00
20,00
30,00
40,00
50,00
ProgMot

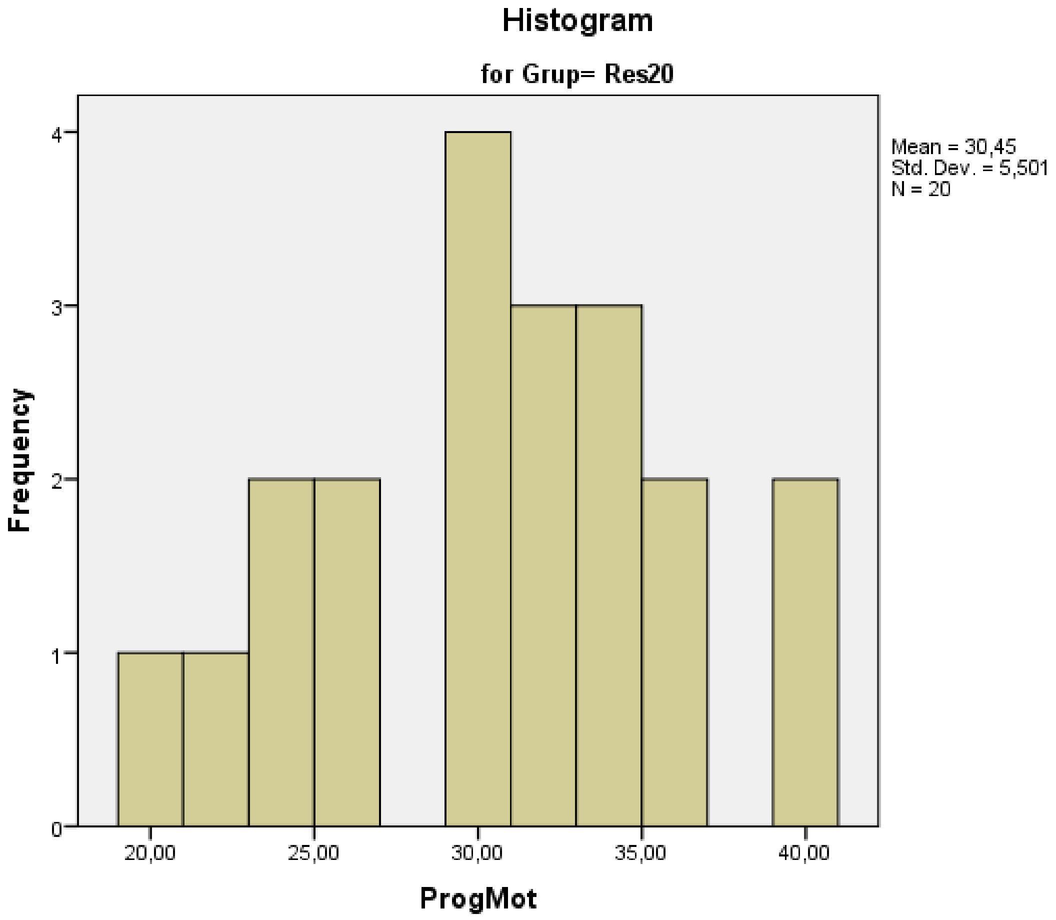
Histogram
for Grup= Res20
Frequency
ProgMot
Mean = 30,45
Std. Dev. = 5,501
N = 20
0
1
2
3
4
20,00
25,00
30,00
35,00
40,00

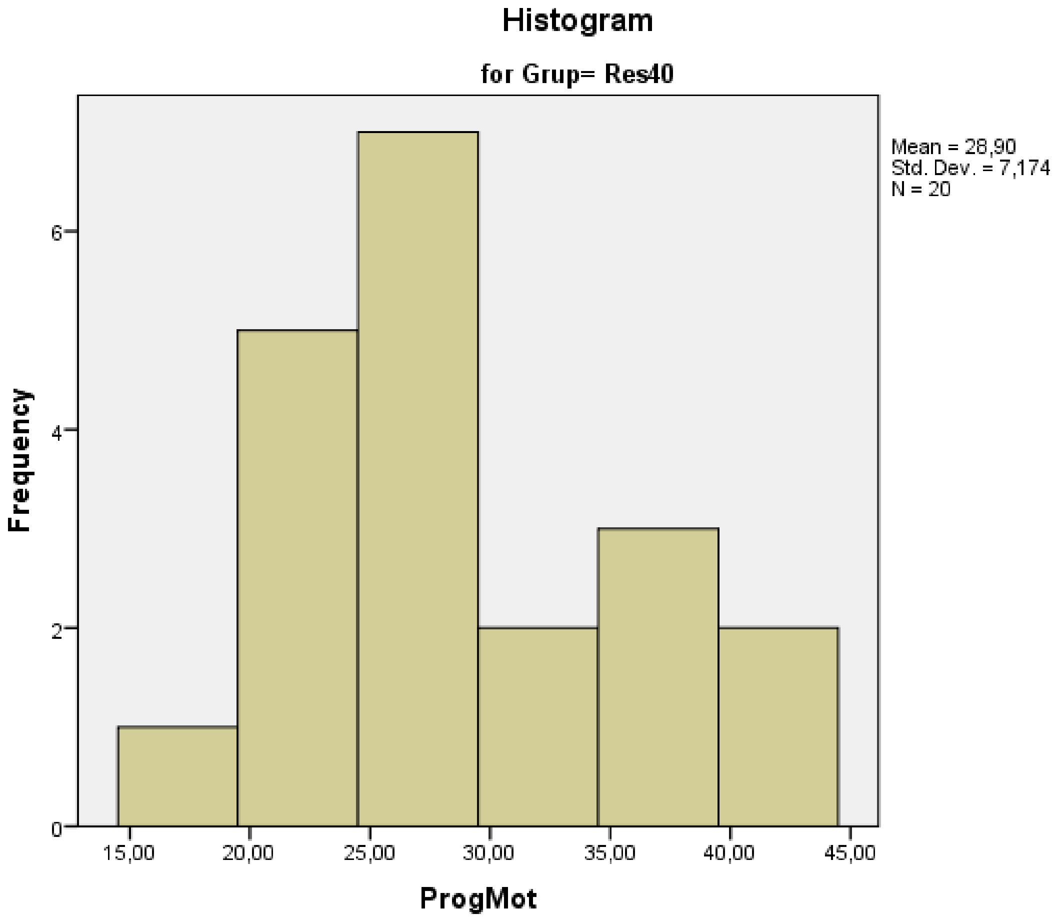
Histogram
for Grup= Res40
Mean = 28,90
Std. Dev. = 7,174
N = 20
Frequency
0
2
4
6
15,00
20,00
25,00
30,00
35,00
40,00
45,00
ProgMot

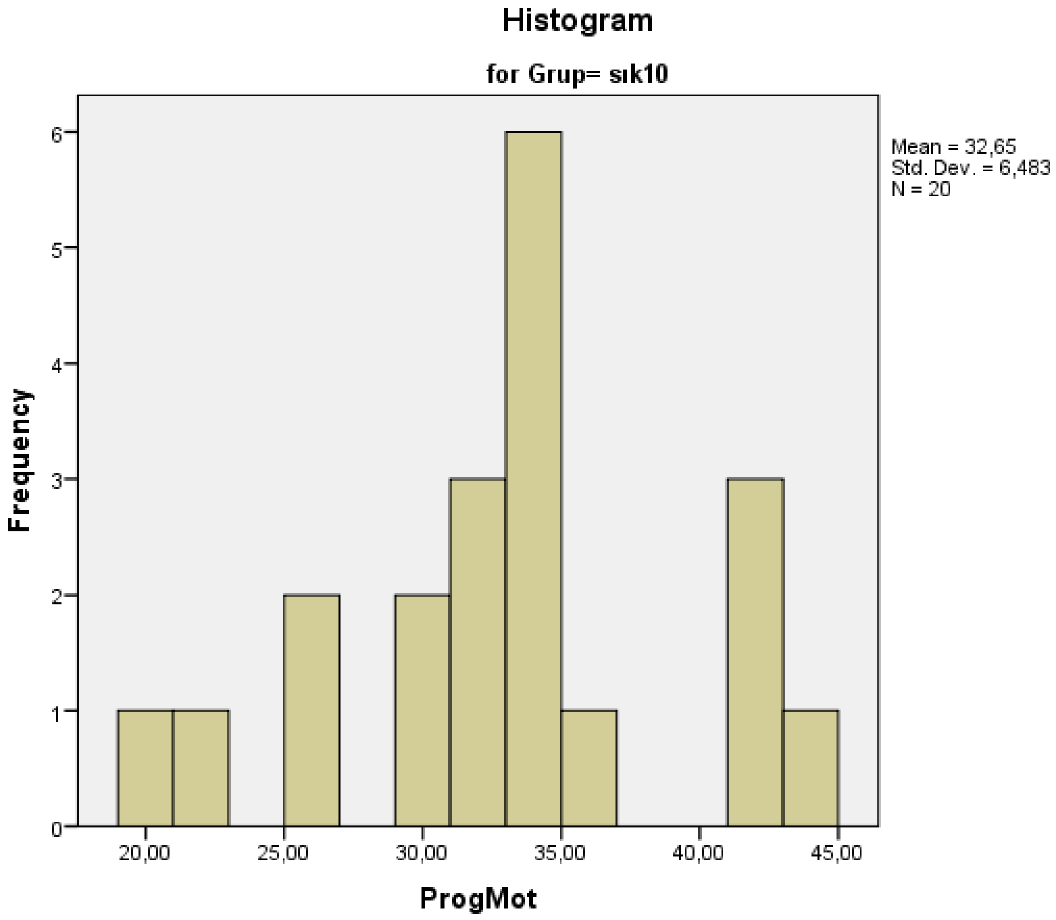
Histogram
for Grup= sık10
Mean = 32,65
Std. Dev. = 6,483
N = 20
Frequency
0
1
2
3
4
5
6
20,00
25,00
30,00
35,00
40,00
45,00
ProgMot

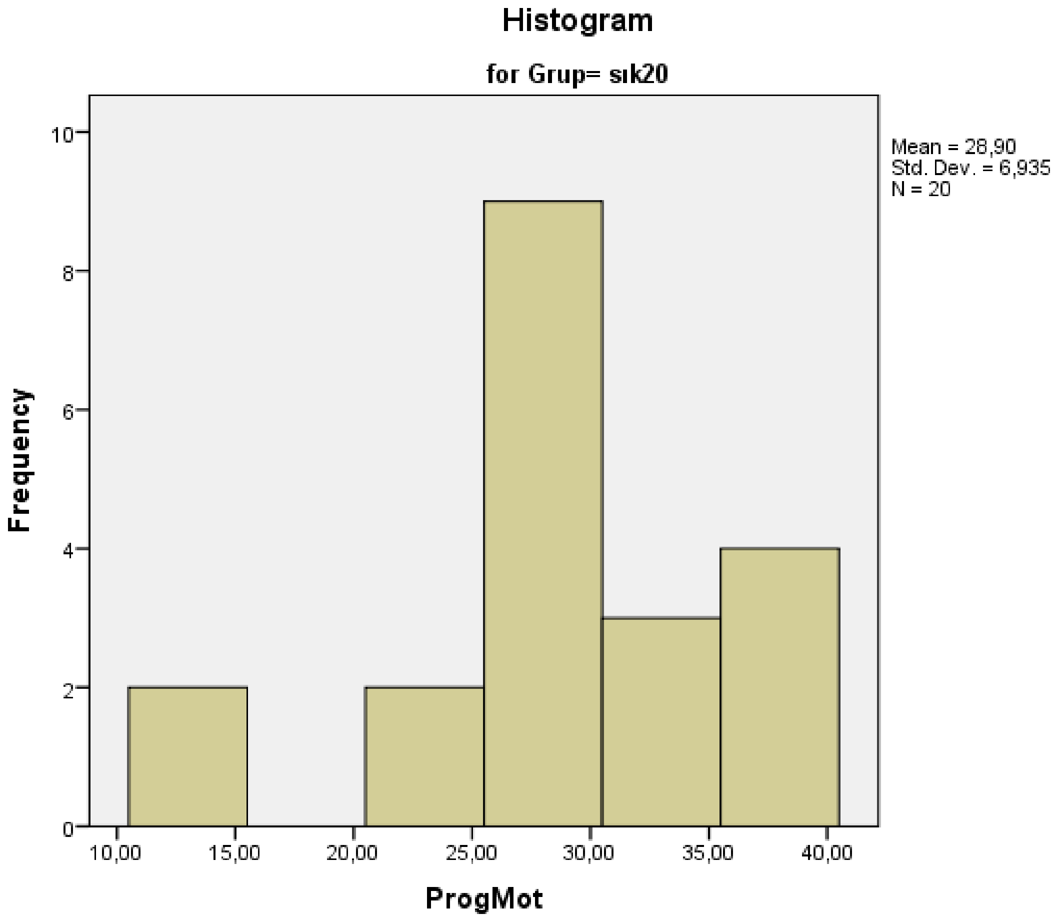
Histogram
for Grup= sık20
Mean = 28,90
Std. Dev. = 6,935
N = 20
Frequency
0
2
4
6
8
10
10,00
15,00
20,00
25,00
30,00
35,00
40,00
ProgMot

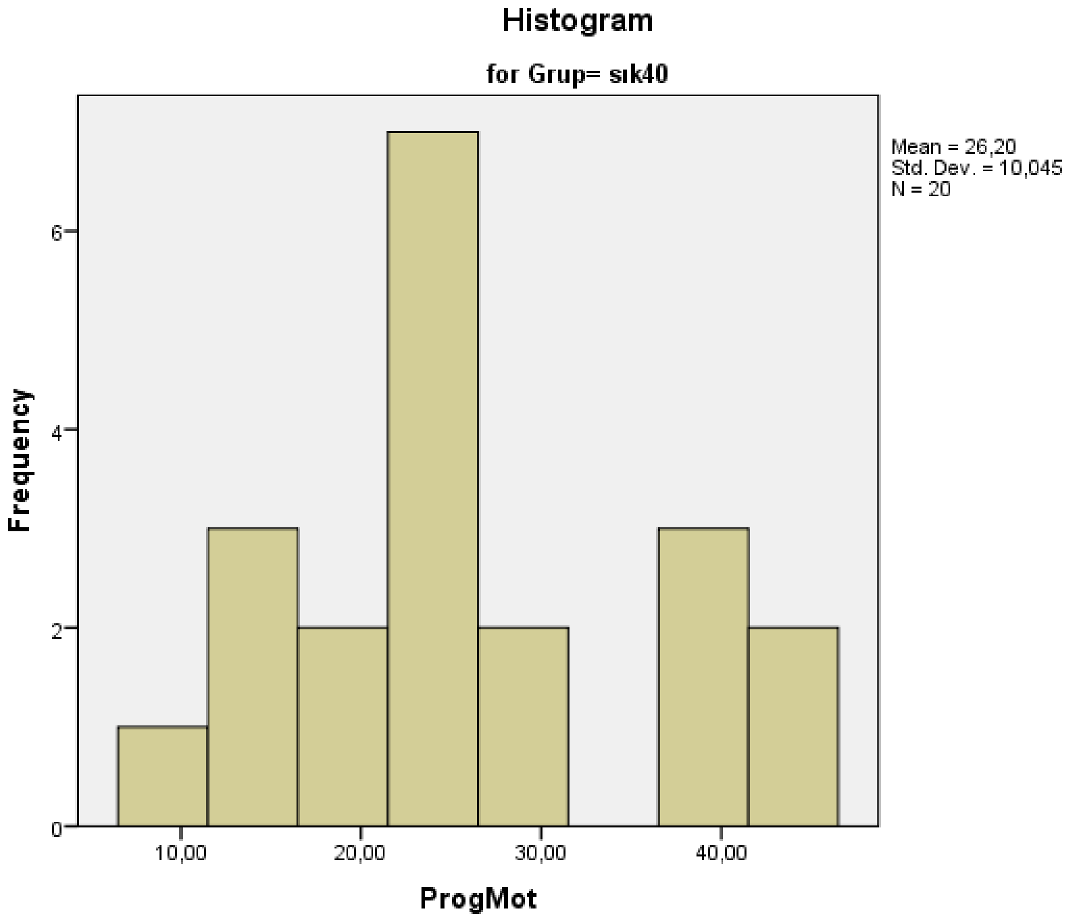
Histogram
for Grup= sık40
Mean = 26,20
Std. Dev. = 10,045
N = 20
Frequency
0
2
4
6
10,00
20,00
30,00
40,00
ProgMot

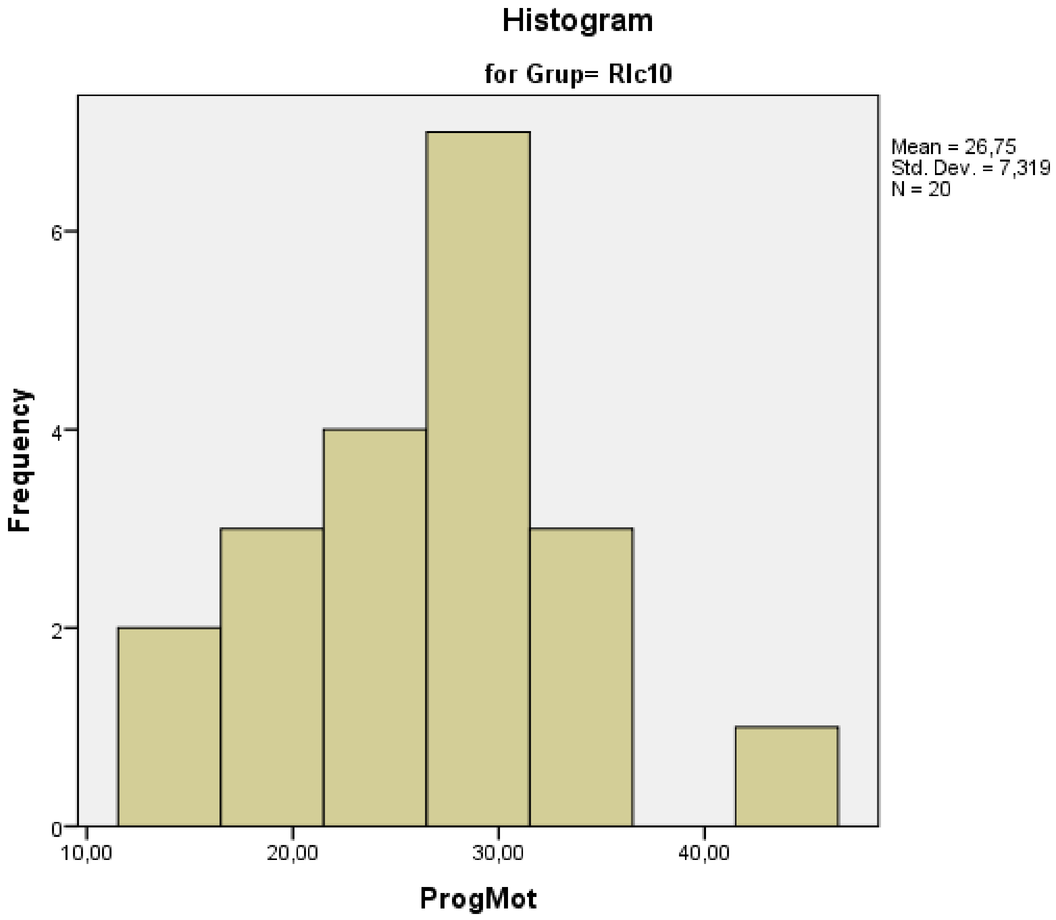
Histogram
for Grup= Rlc10
Mean = 26,75
Std. Dev. = 7,319
N = 20
Frequency
0
2
4
6
10,00
20,00
30,00
40,00
ProgMot

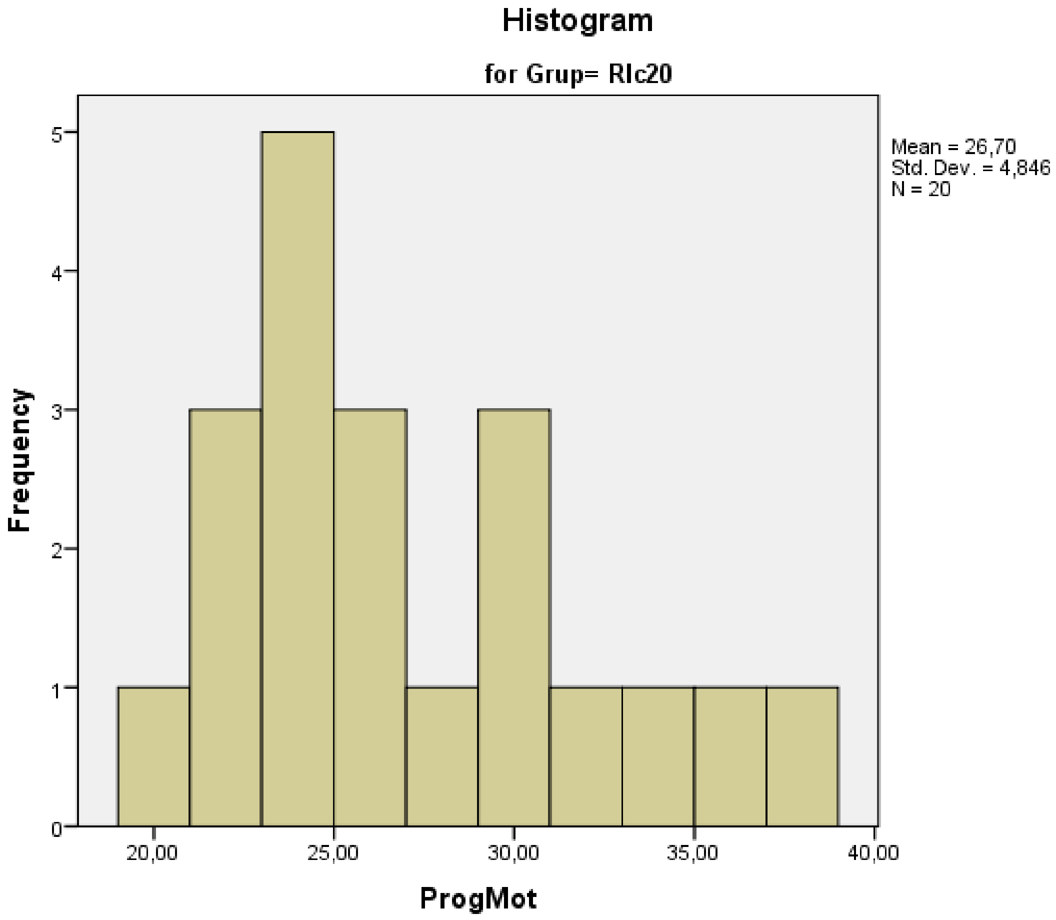

Histogram
for Grup= Rlc20
Mean = 26,70
Std. Dev. = 4,846
N = 20
Frequency
0
1
2
3
4
5
20,00
25,00
30,00
35,00
40,00
ProgMot

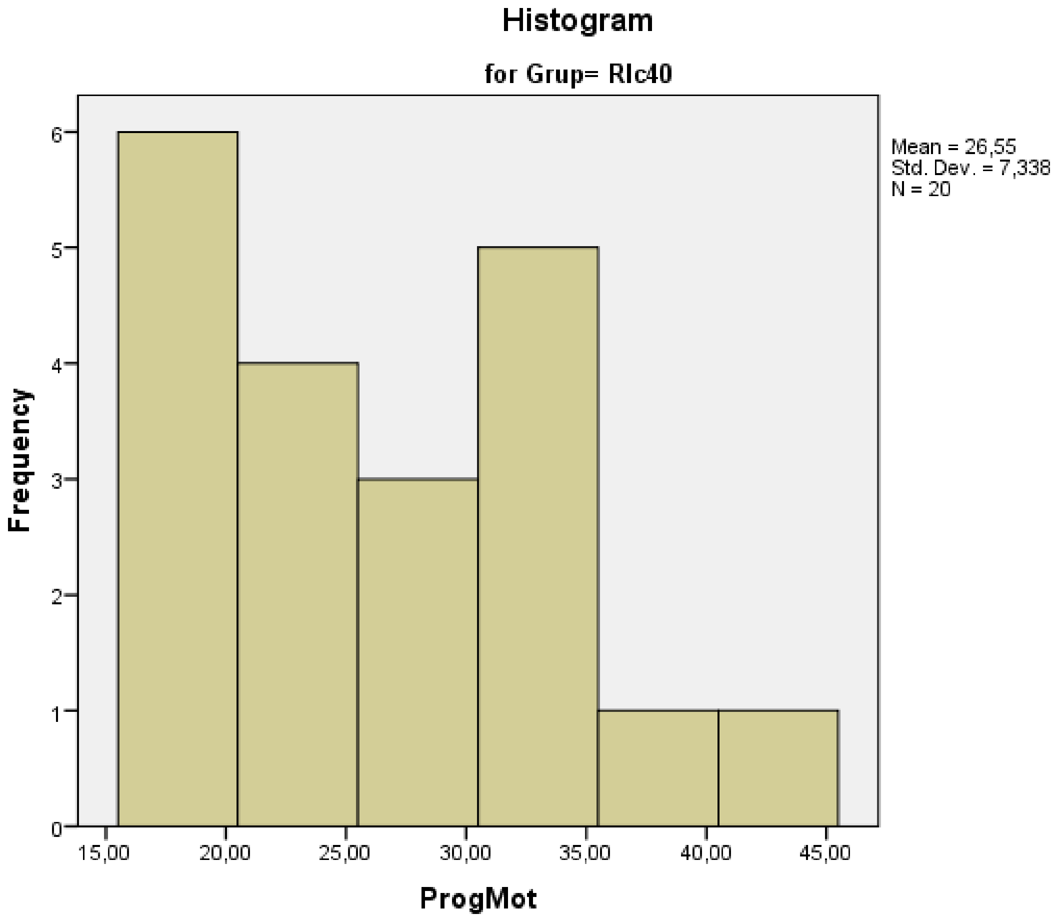

Stem-and-Leaf Plots

```
ProgMot Stem-and-Leaf Plot for
Grup= kontrol

 Frequency    Stem &  Leaf

     1,00        1 .  4
     1,00        1 .  8
     1,00        2 .  3
     9,00        2 .  556677899
     3,00        3 .  013
     3,00        3 .  557
     2,00        4 .  14

 Stem width:     10,00
 Each leaf:       1 case(s)
```

ProgMot Stem-and-Leaf Plot for
Grup= Res10

```
 Frequency    Stem &  Leaf

     2,00        1 .  14
     2,00        1 .  77
     5,00        2 .  11222
     3,00        2 .  799
     5,00        3 .  00013
     2,00        3 .  79
     1,00        4 .  4

 Stem width:     10,00
 Each leaf:       1 case(s)
```

ProgMot Stem-and-Leaf Plot for
Grup= Res20

```
 Frequency    Stem &  Leaf

     4,00        2 .  0244
     3,00        2 .  669
     9,00        3 .  000122444
     3,00        3 .  669
     1,00        4 .  0

 Stem width:     10,00
 Each leaf:       1 case(s)
```

ProgMot Stem-and-Leaf Plot for
Grup= Res40

```
 Frequency    Stem &  Leaf

     1,00        1 .  7
     5,00        2 .  01244
     7,00        2 .  5677899
     2,00        3 .  04
     3,00        3 .  589
     2,00        4 .  12

 Stem width:     10,00
 Each leaf:       1 case(s)
```

```
ProgMot Stem-and-Leaf Plot for
Grup= sık10

 Frequency    Stem &  Leaf

     1,00 Extremes    (=<20)
     1,00        2 .  1
     4,00        2 .  6699
     9,00        3 .  122344444
     1,00        3 .  5
     3,00        4 .  222
     1,00 Extremes    (>=43)

 Stem width:     10,00
 Each leaf:       1 case(s)
```

```
ProgMot Stem-and-Leaf Plot for
Grup= sık20

 Frequency    Stem &  Leaf

     1,00 Extremes    (=<13)
      ,00        1 .
     1,00        1 .  5
     1,00        2 .  4
    10,00        2 .  5666788999
     3,00        3 .  444
     3,00        3 .  777
     1,00        4 .  0

 Stem width:     10,00
 Each leaf:       1 case(s)
```

```
ProgMot Stem-and-Leaf Plot for
Grup= sık40

 Frequency    Stem &  Leaf

     1,00        0 .  9
     5,00        1 .  45699
     9,00        2 .  244556679
     2,00        3 .  88
     3,00        4 .  125

 Stem width:     10,00
 Each leaf:       1 case(s)
```

```
ProgMot Stem-and-Leaf Plot for
Grup= Rlc10

 Frequency    Stem &  Leaf

     1,00        1 .  4
     3,00        1 .  599
     4,00        2 .  1244
     4,00        2 .  6779
     6,00        3 .  001124
     1,00        3 .  6
     1,00        4 .  4

 Stem width:     10,00
 Each leaf:       1 case(s)
```

```
ProgMot Stem-and-Leaf Plot for
Grup= Rlc20

 Frequency    Stem &  Leaf

     9,00        2 .  022233344
     6,00        2 .  666899
     3,00        3 .  013
     2,00        3 .  58

 Stem width:     10,00
 Each leaf:       1 case(s)
```

```
ProgMot Stem-and-Leaf Plot for
Grup= Rlc40

 Frequency    Stem &  Leaf

     4,00        1 .  8999
     5,00        2 .  00123
     3,00        2 .  566
     6,00        3 .  012224
     1,00        3 .  7
      ,00        4 .
     1,00        4 .  5

 Stem width:     10,00
 Each leaf:       1 case(s)
```

Normal Q-Q Plots

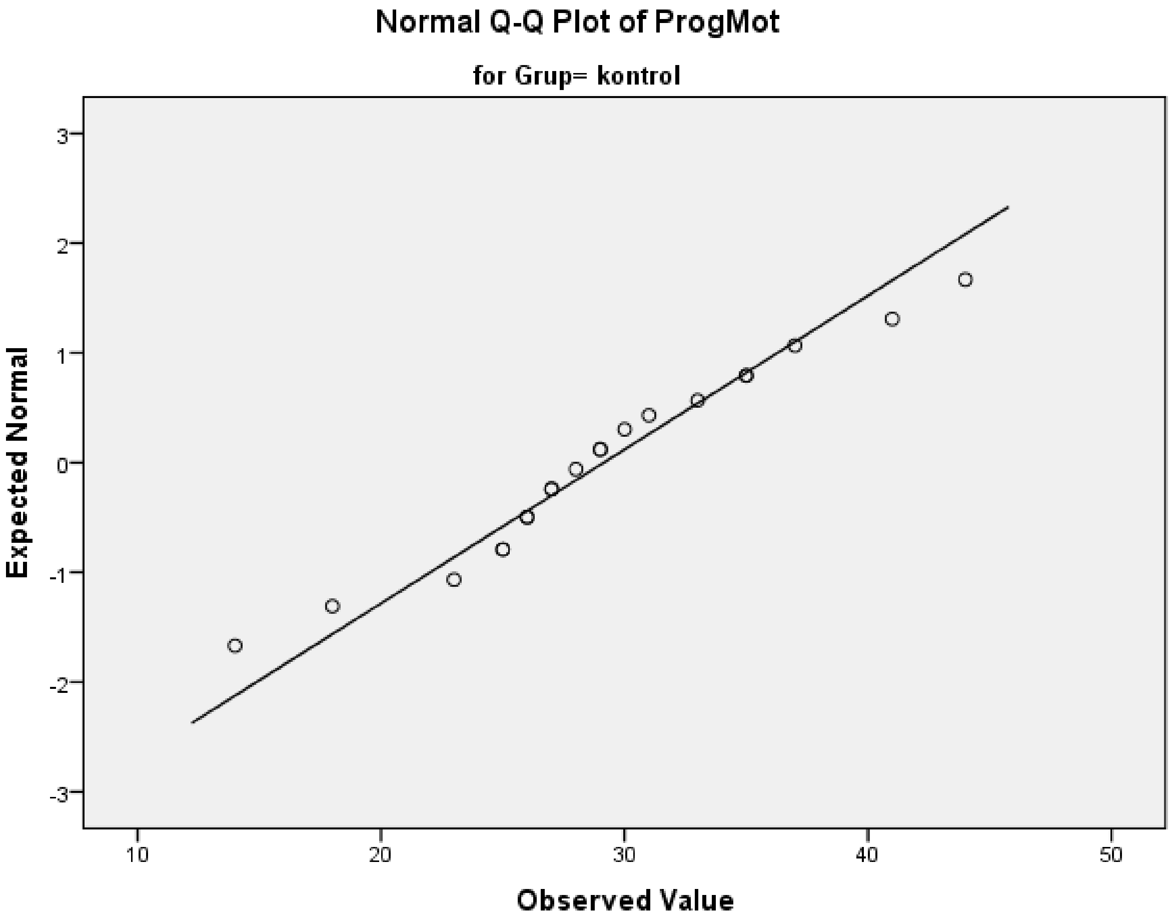

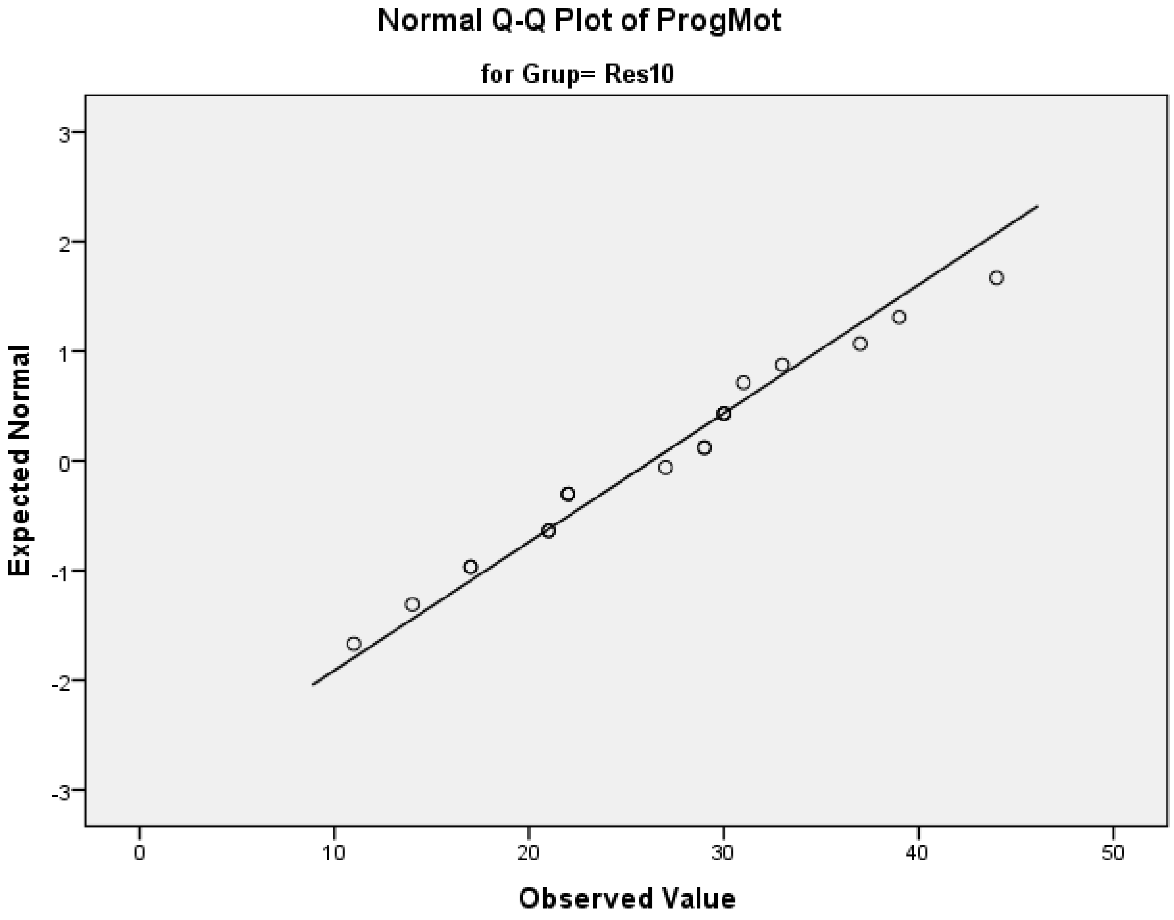
Normal Q-Q Plot of ProgMot
for Grup= Res10
Expected Normal
Observed Value
3
2
1
0
-1
-2
-3
0
10
20
30
40
50

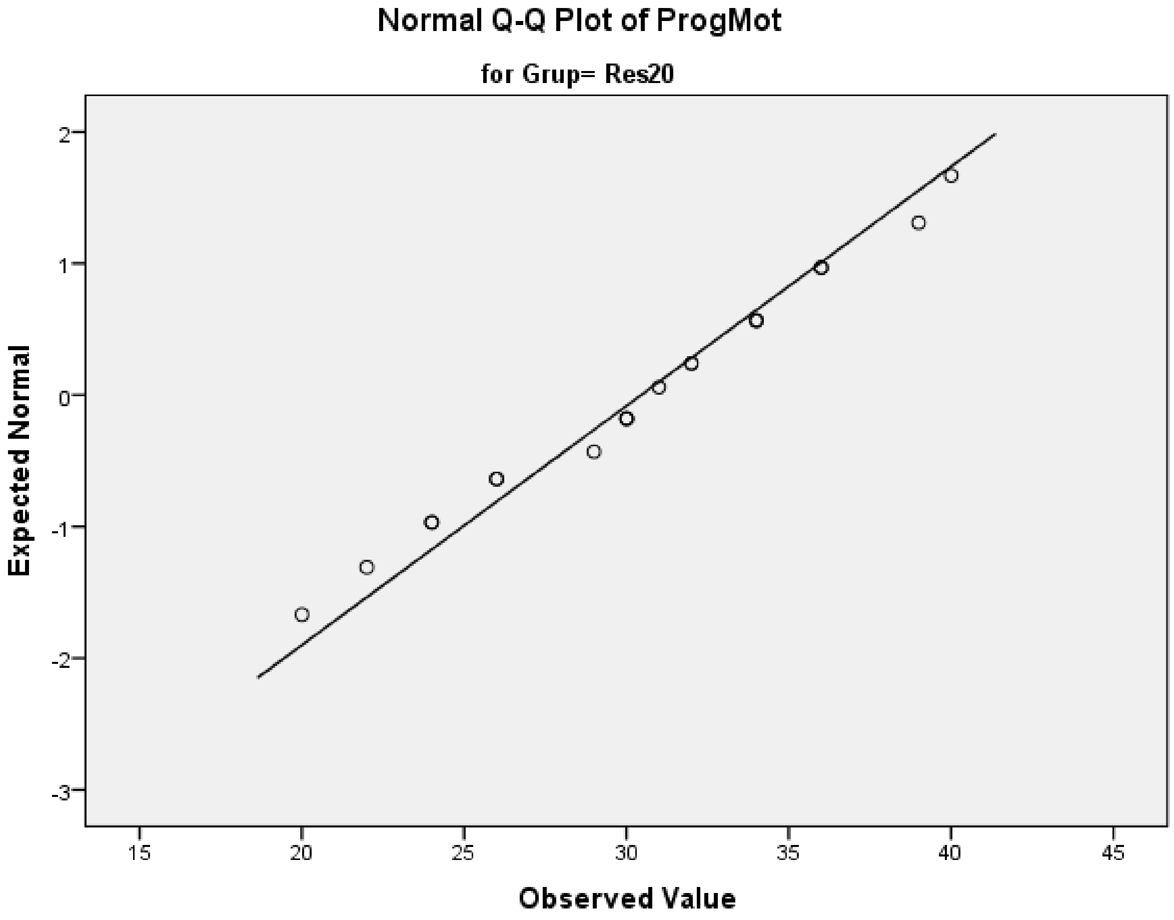
Normal Q-Q Plot of ProgMot
for Grup= Res20
Expected Normal
2
1
0
-1
-2
-3
15
20
25
30
35
40
45
Observed Value

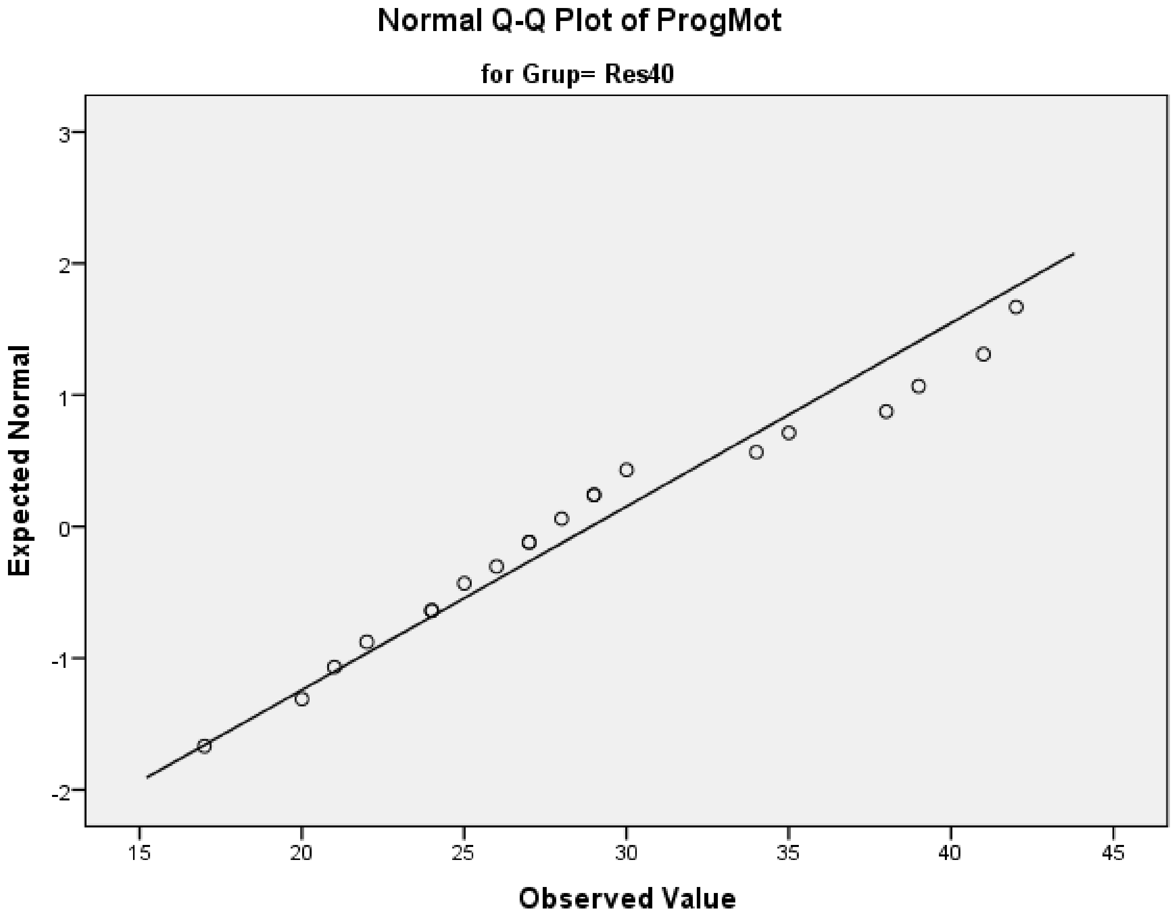
Normal Q-Q Plot of ProgMot
for Grup= Res40
Expected Normal
Observed Value
3
2
1
0
-1
-2
15
20
25
30
35
40
45

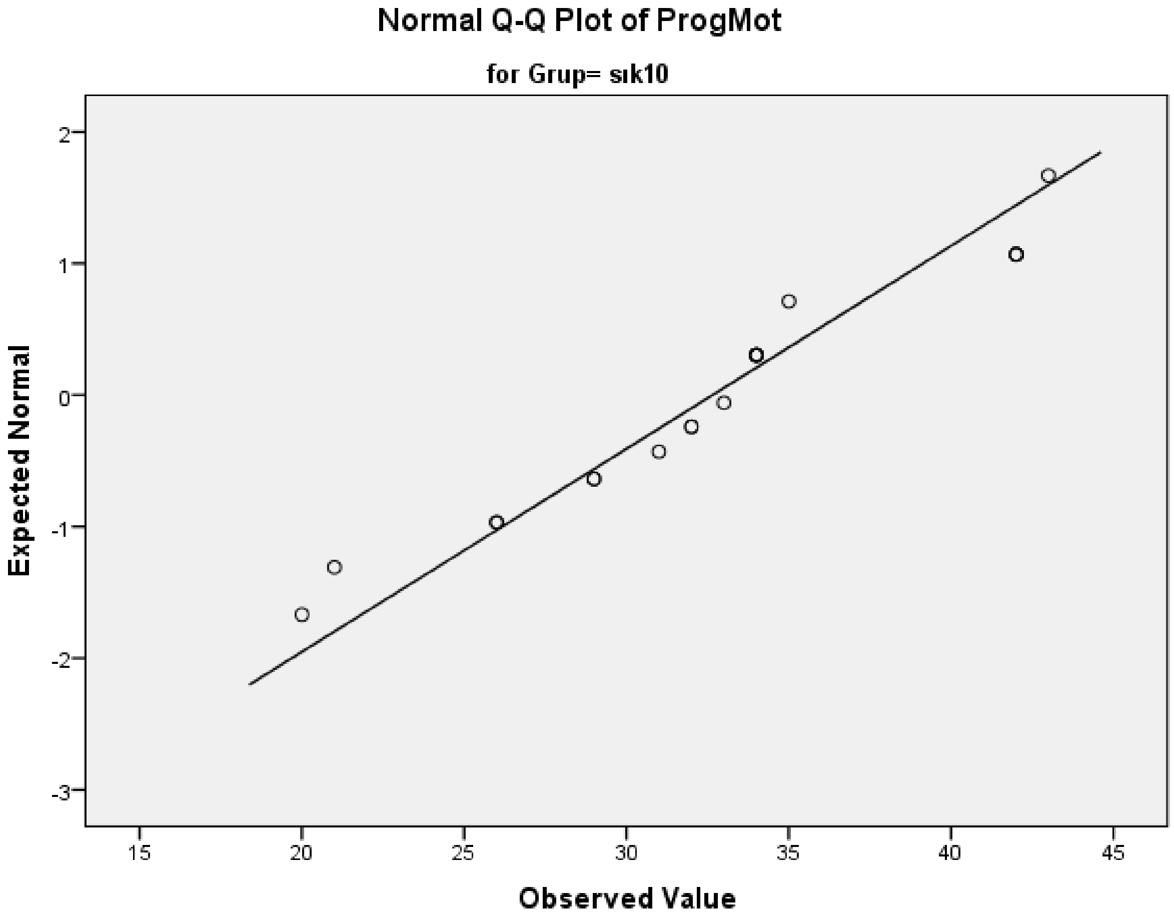

Normal Q-Q Plot of ProgMot
for Grup= sık10
Expected Normal
Observed Value
2
1
0
-1
-2
-3
15
20
25
30
35
40
45

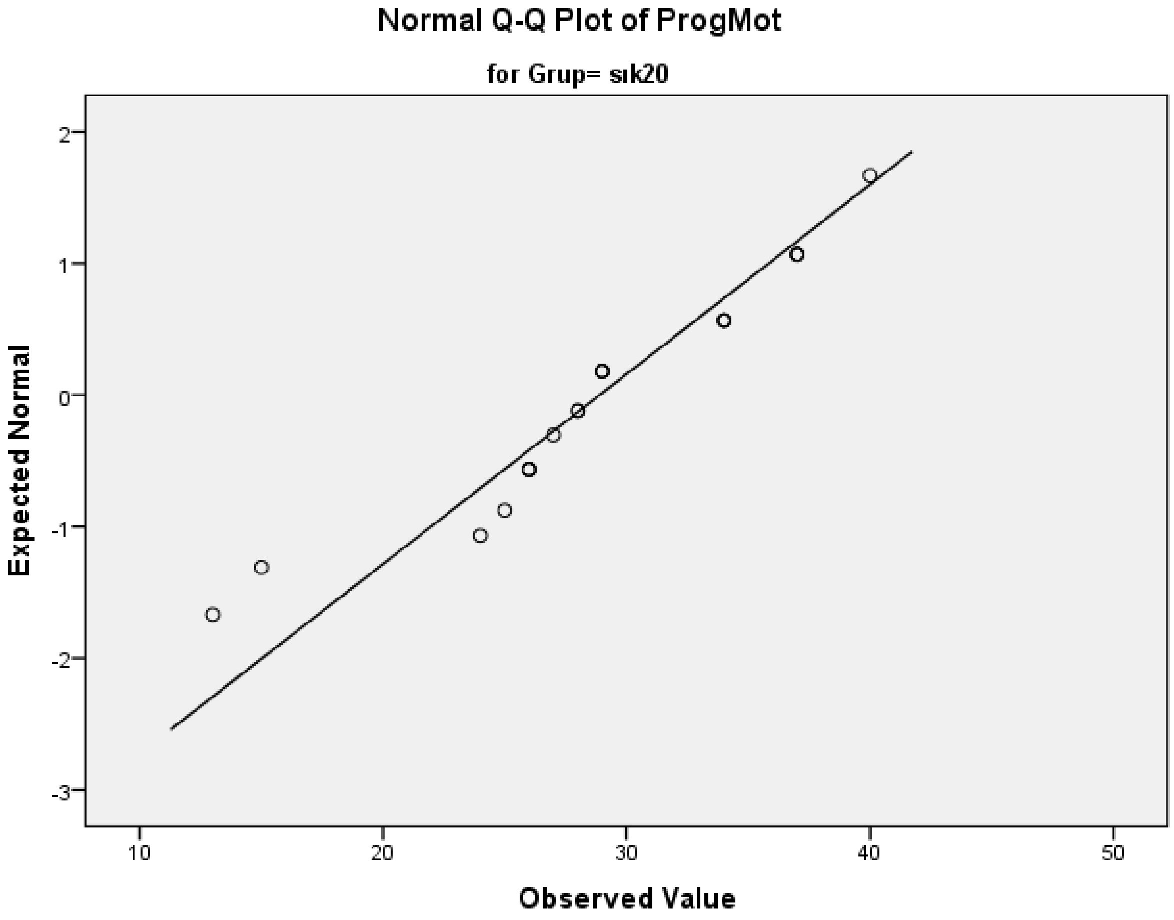
Normal Q-Q Plot of ProgMot
for Grup= sık20
Expected Normal
Observed Value
2
1
0
-1
-2
-3
10
20
30
40
50

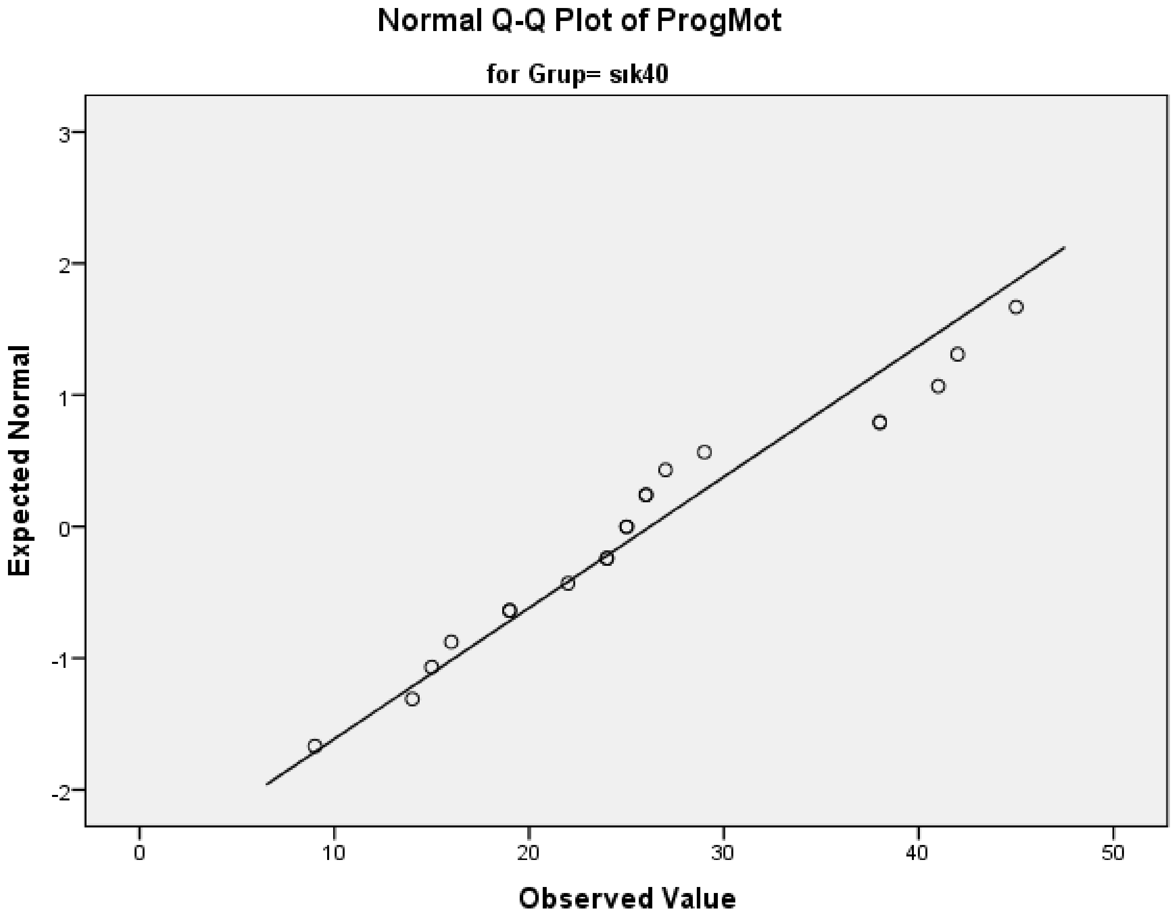
Normal Q-Q Plot of ProgMot
for Grup= sık40
Expected Normal
Observed Value
3
2
1
0
-1
-2
0
10
20
30
40
50

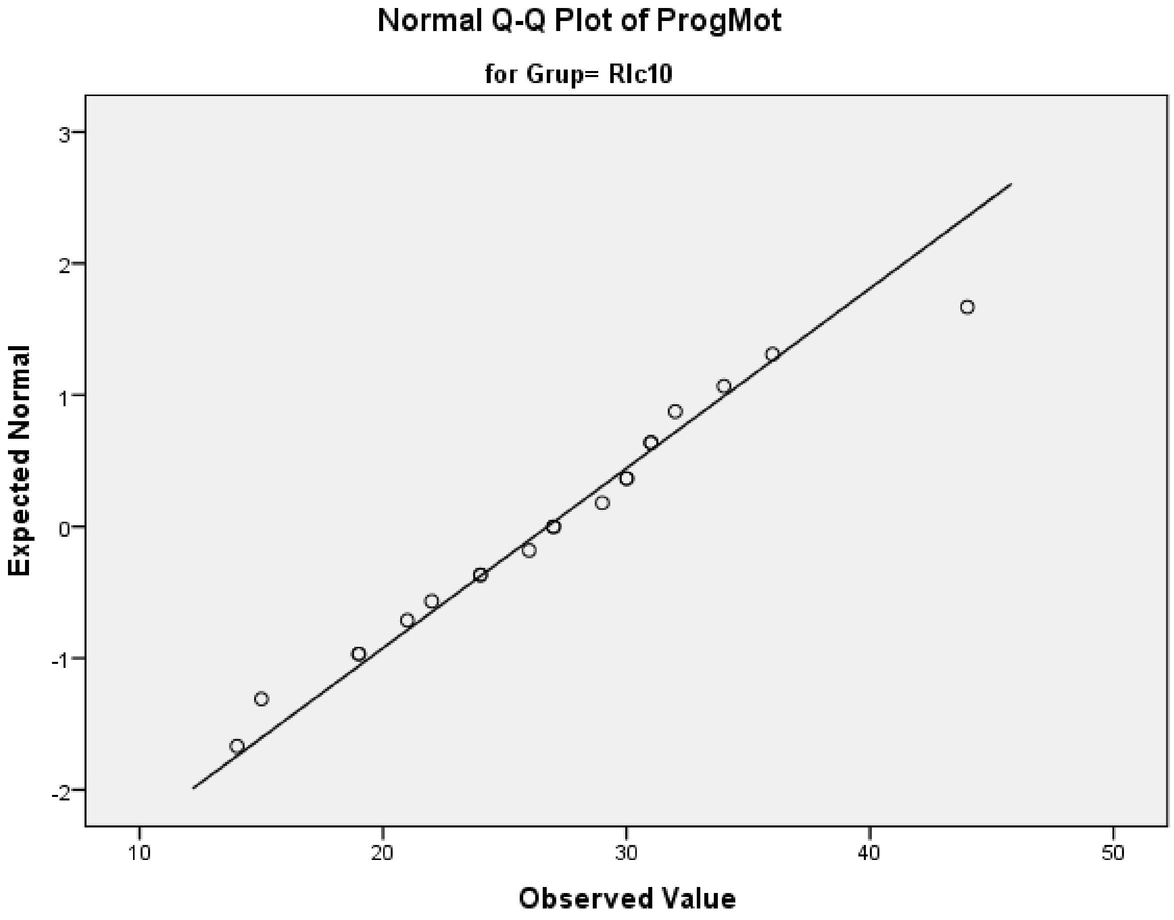

Normal Q-Q Plot of ProgMot
for Grup= Rlc10
Expected Normal
Observed Value
3
2
1
0
-1
-2
10
20
30
40
50

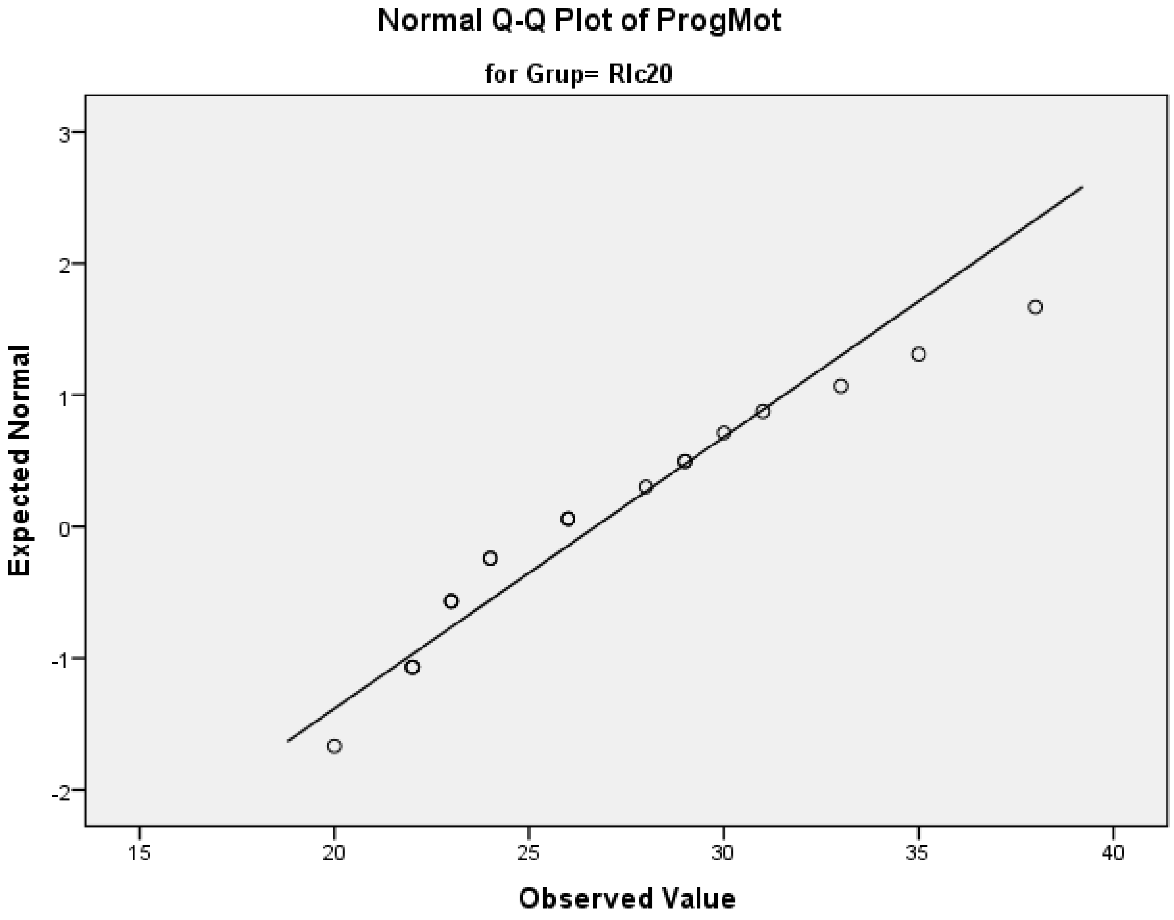
Normal Q-Q Plot of ProgMot
for Grup= RIc20
Expected Normal
3
2
1
0
-1
-2
15
20
25
30
35
40
Observed Value

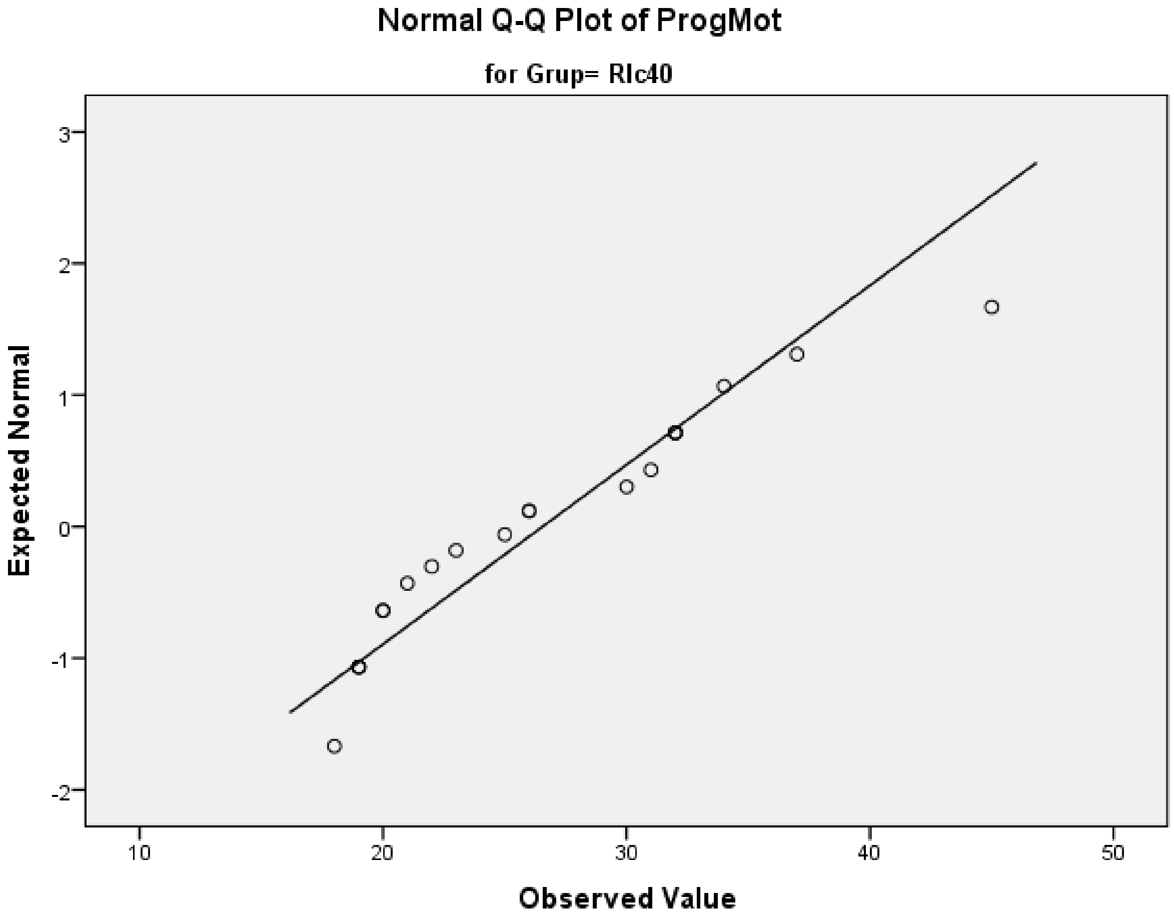

Detrended Normal Q-Q Plots

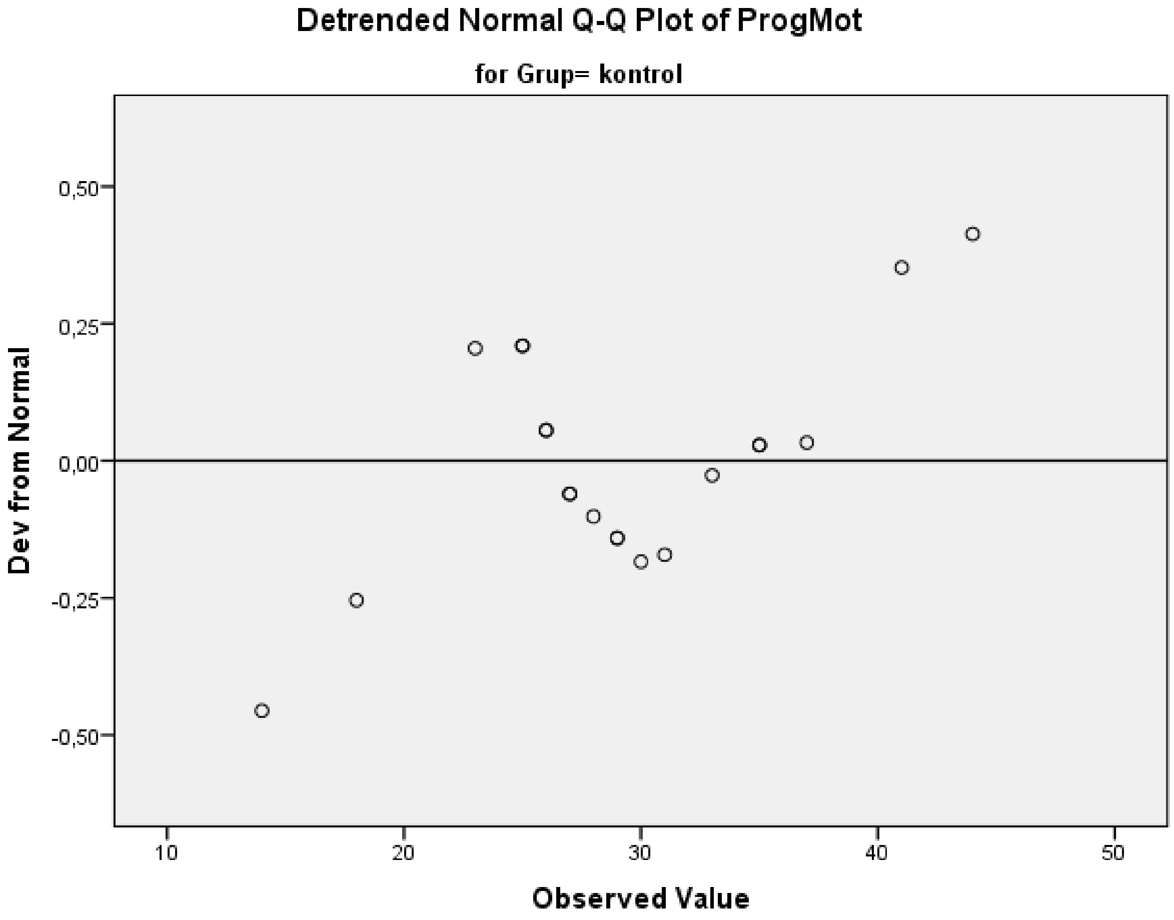

Detrended Normal Q-Q Plot of ProgMot
for Grup= kontrol
Dev from Normal
0,50
0,25
0,00
-0,25
-0,50
10
20
30
40
50
Observed Value

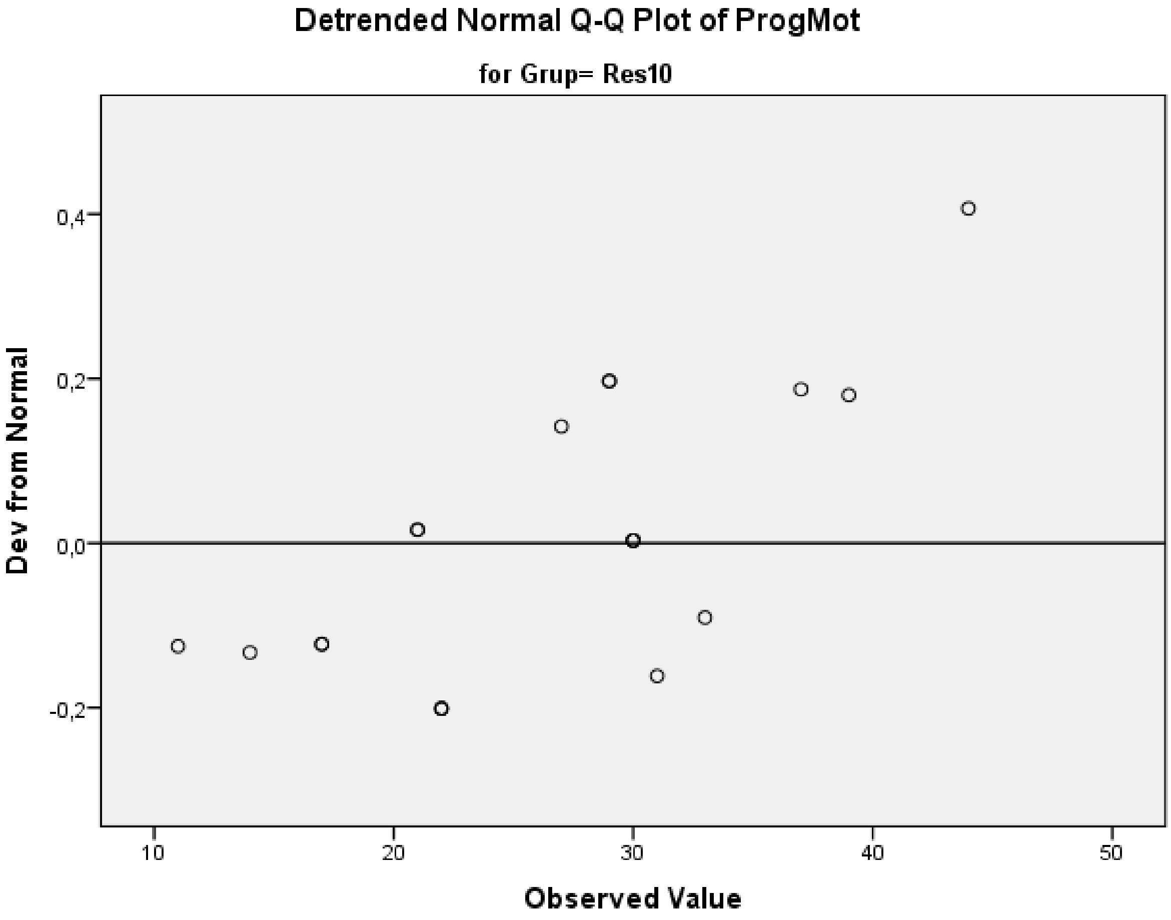
Detrended Normal Q-Q Plot of ProgMot
for Grup= Res10
Dev from Normal
0,4
0,2
0,0
-0,2
10
20
30
40
50
Observed Value

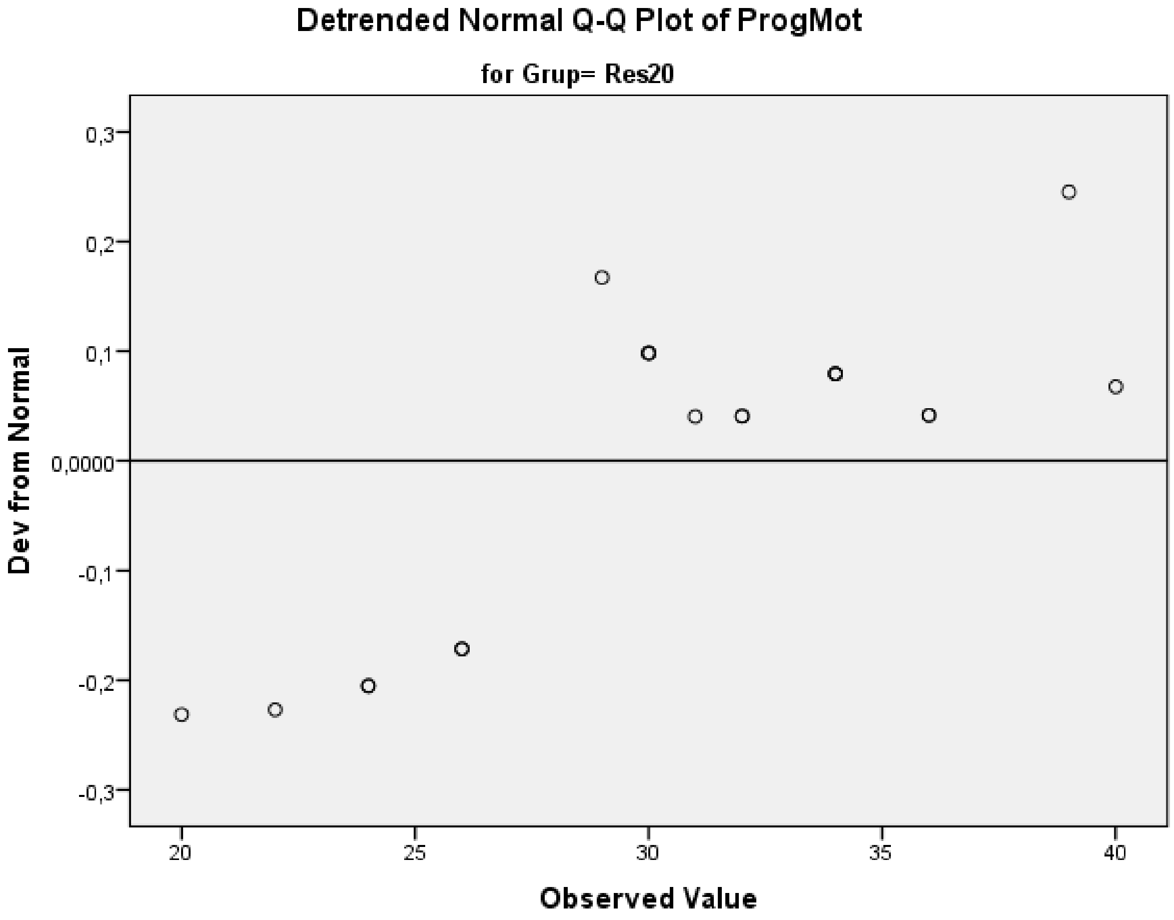

Detrended Normal Q-Q Plot of ProgMot
for Grup= Res20
Dev from Normal
0,3
0,2
0,1
0,0000
-0,1
-0,2
-0,3
20
25
30
35
40
Observed Value

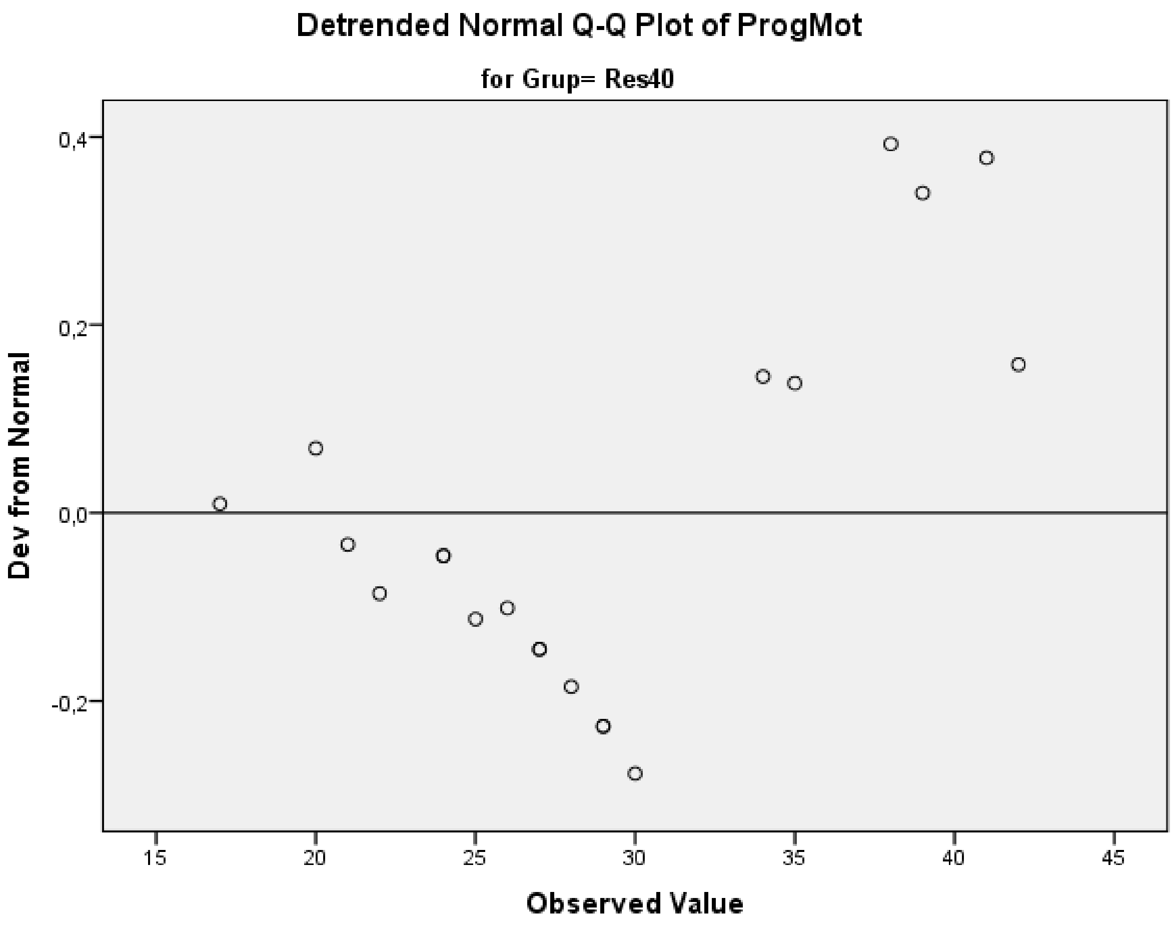
Detrended Normal Q-Q Plot of ProgMot
for Grup= Res40
Dev from Normal
0,4
0,2
0,0
-0,2
15
20
25
30
35
40
45
Observed Value

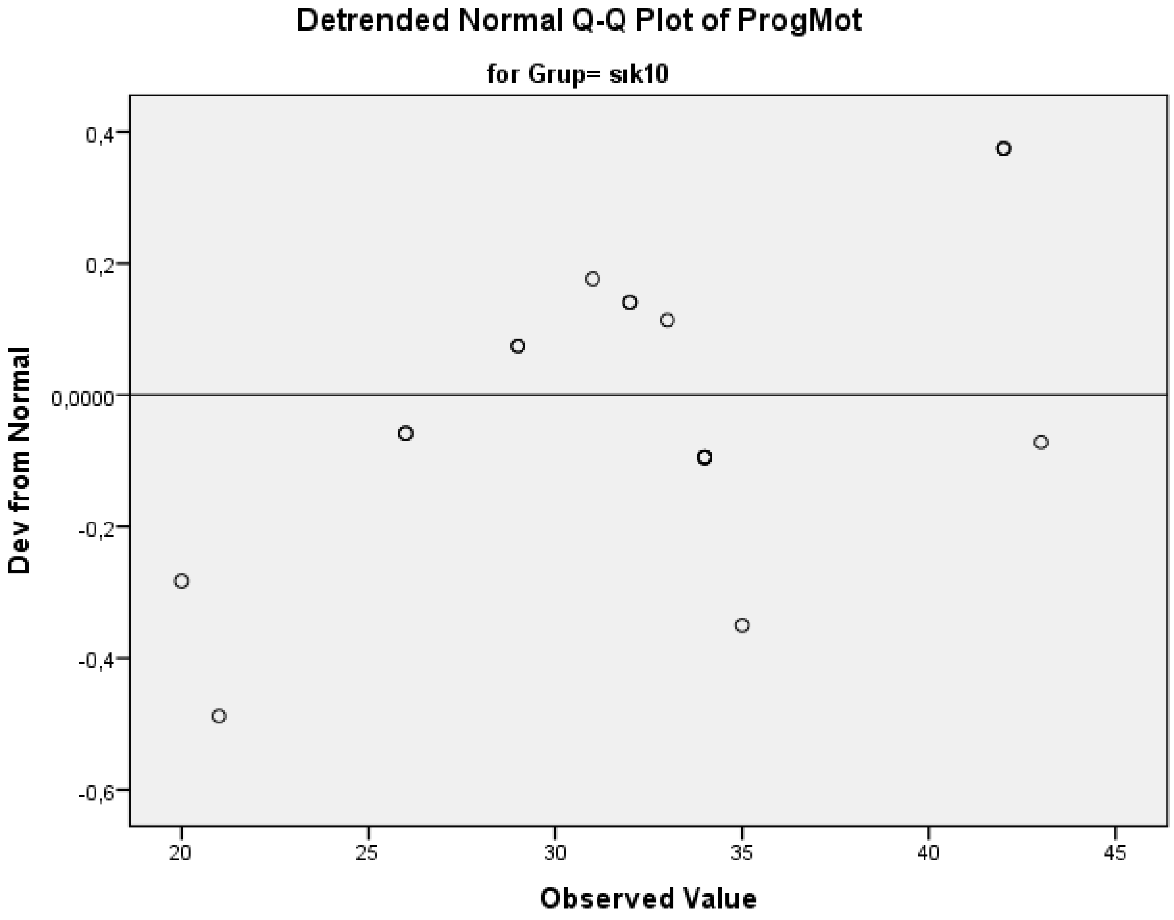

Detrended Normal Q-Q Plot of ProgMot
for Grup= sık10
Dev from Normal
0,4
0,2
0,0000
-0,2
-0,4
-0,6
20
25
30
35
40
45
Observed Value

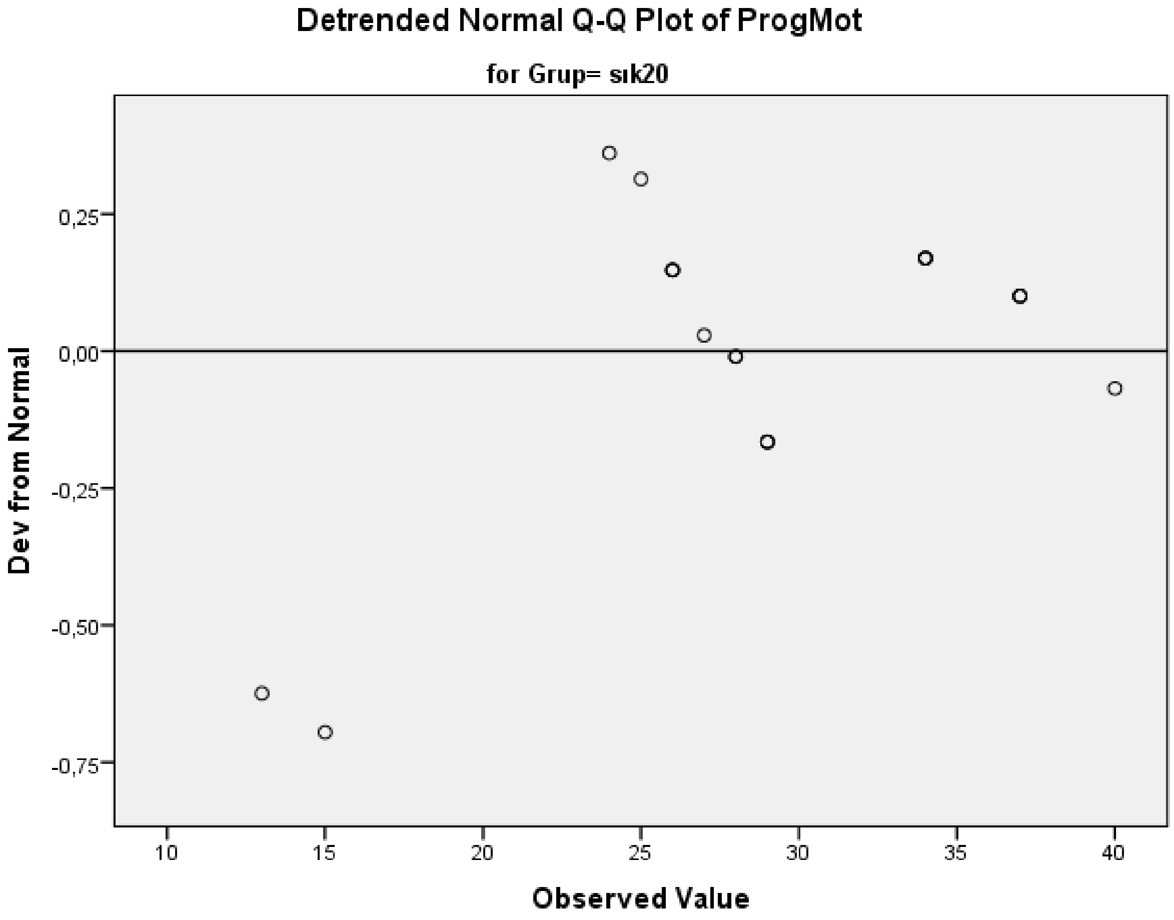
Detrended Normal Q-Q Plot of ProgMot
for Grup= sık20
Dev from Normal
0,25
0,00
-0,25
-0,50
-0,75
10
15
20
25
30
35
40
Observed Value

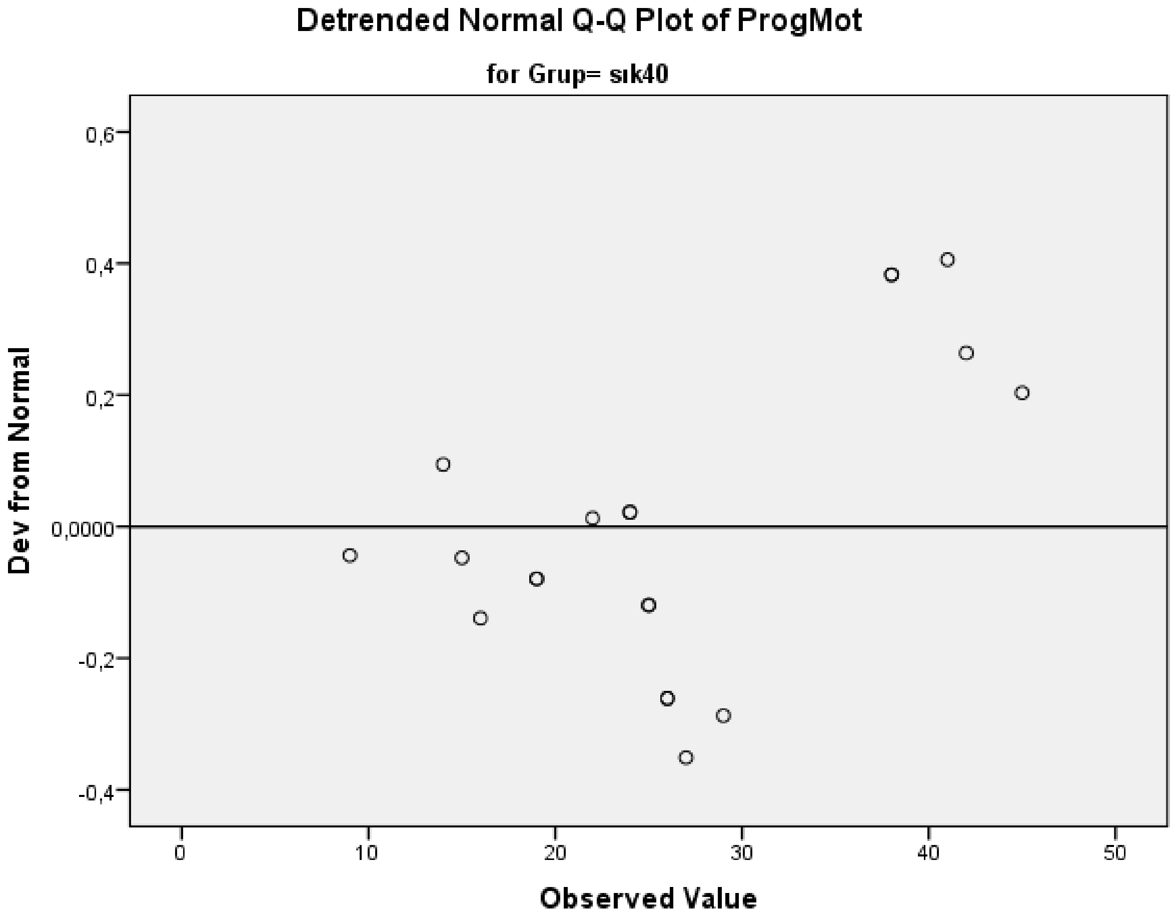

Detrended Normal Q-Q Plot of ProgMot
for Grup= sık40
Dev from Normal
0,6
0,4
0,2
0,0000
-0,2
-0,4
0
10
20
30
40
50
Observed Value

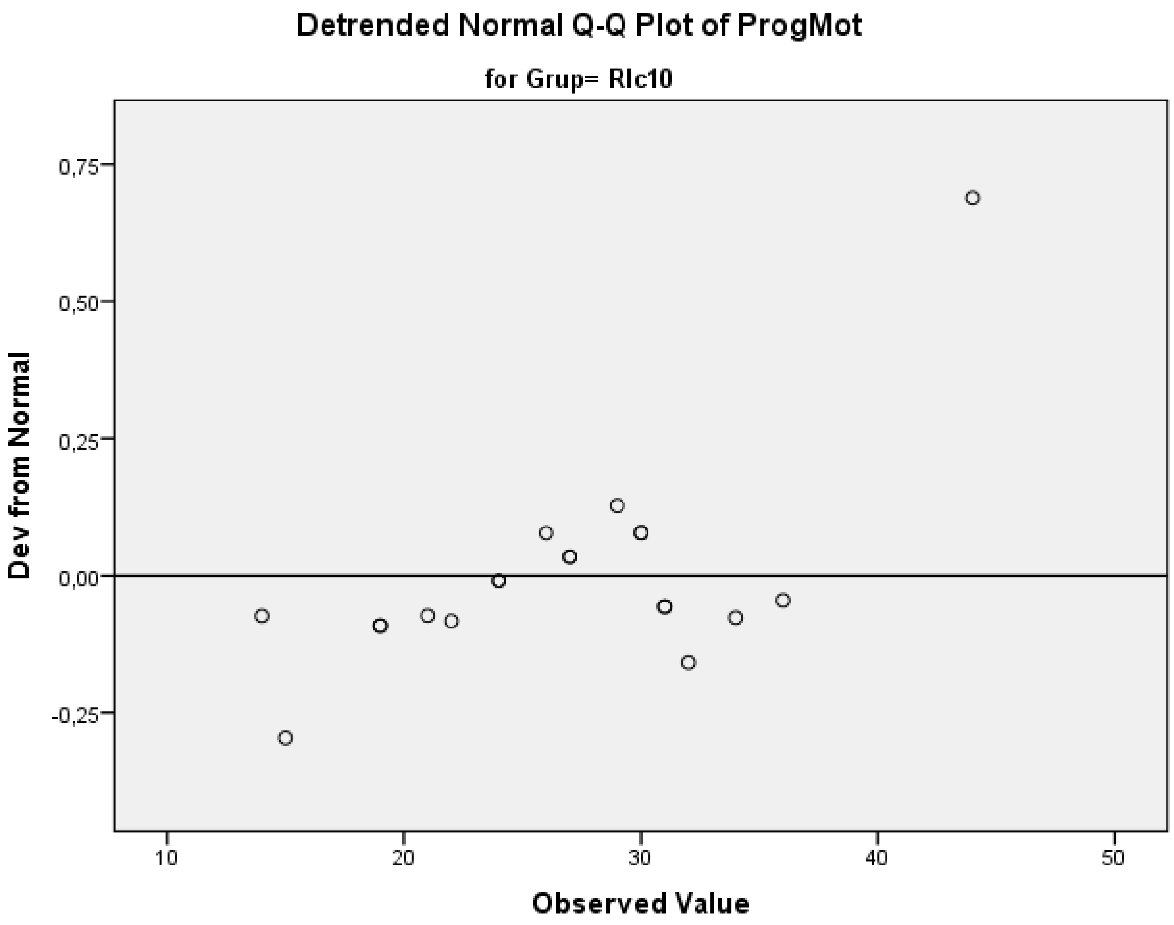

Detrended Normal Q-Q Plot of ProgMot
for Grup= RIc10
Dev from Normal
0,75
0,50
0,25
0,00
-0,25
10
20
30
40
50
Observed Value

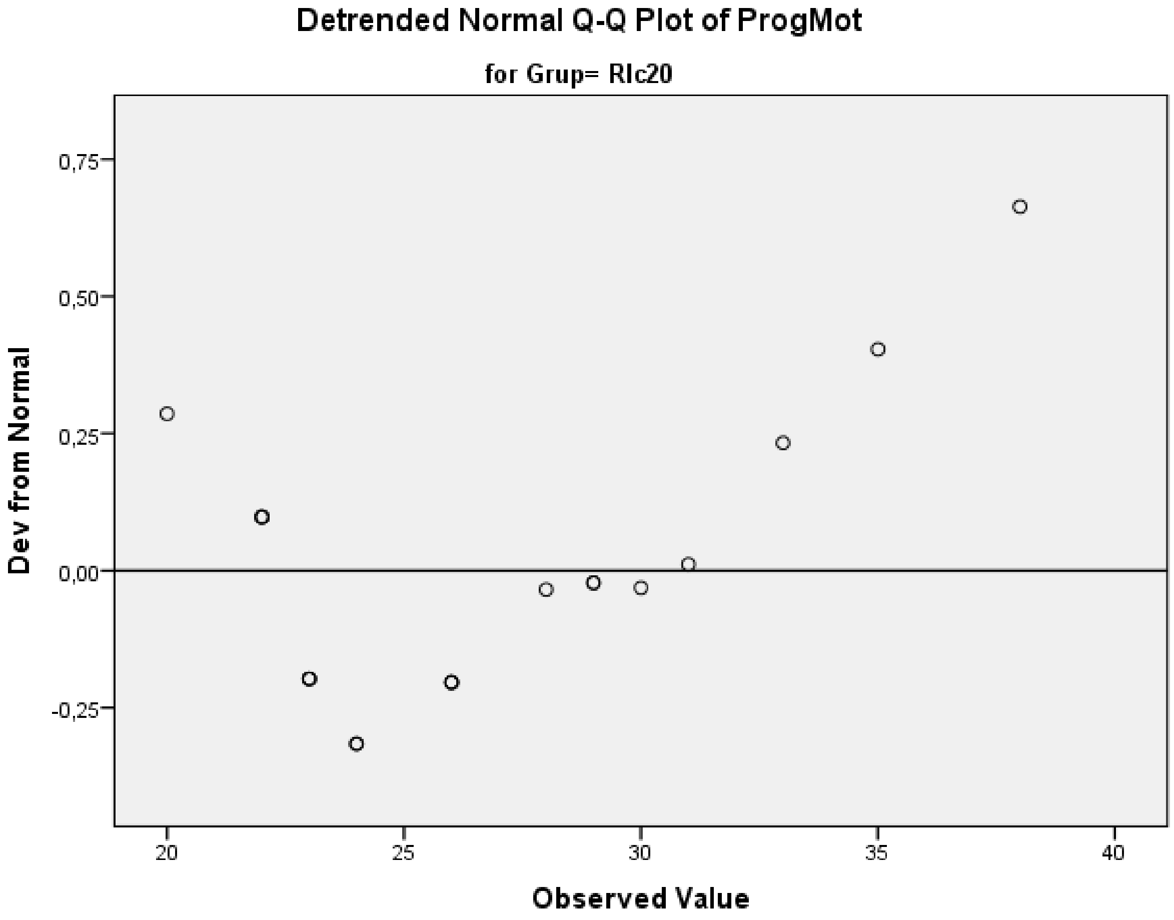

Detrended Normal Q-Q Plot of ProgMot
for Grup= RIc20
Dev from Normal
0,75
0,50
0,25
0,00
-0,25
20
25
30
35
40
Observed Value

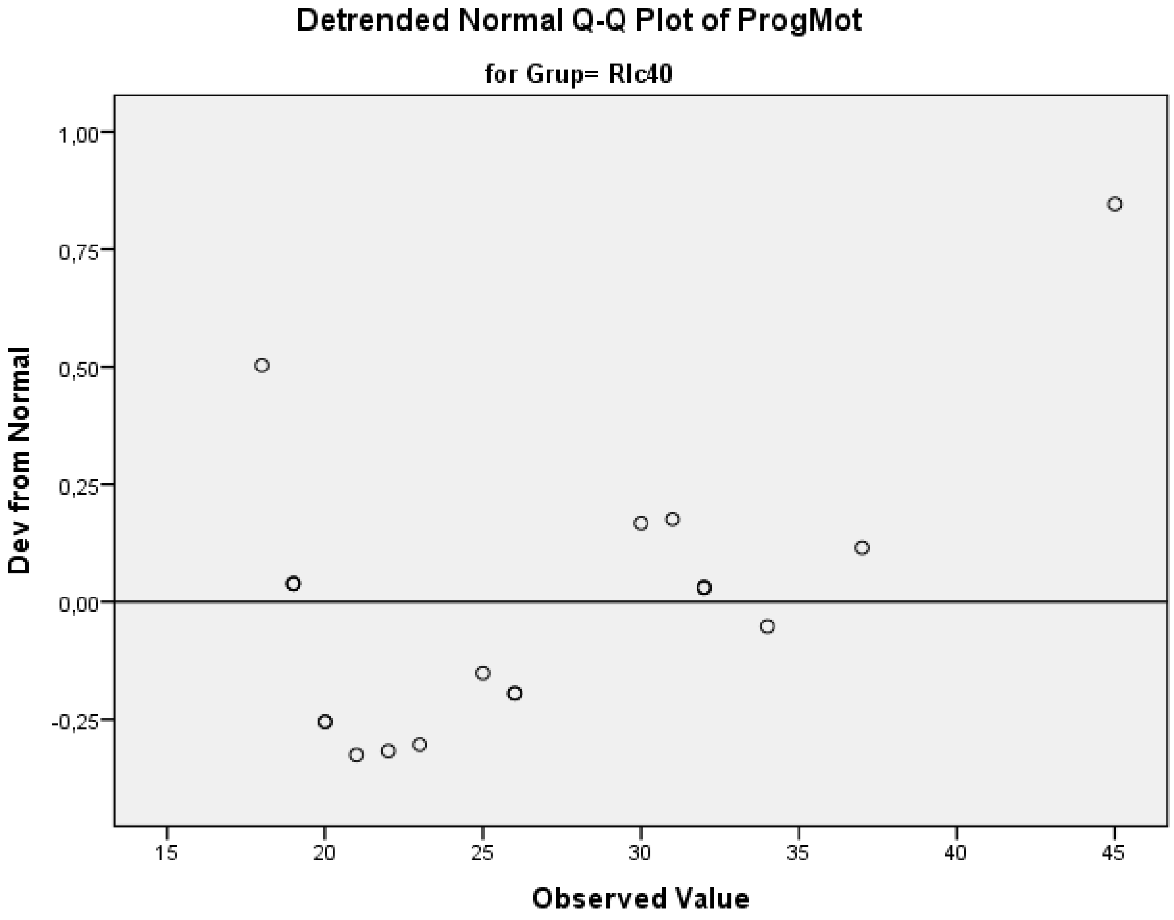
Detrended Normal Q-Q Plot of ProgMot
for Grup= RIc40
Dev from Normal
1,00
0,75
0,50
0,25
0,00
-0,25
15
20
25
30
35
40
45
Observed Value

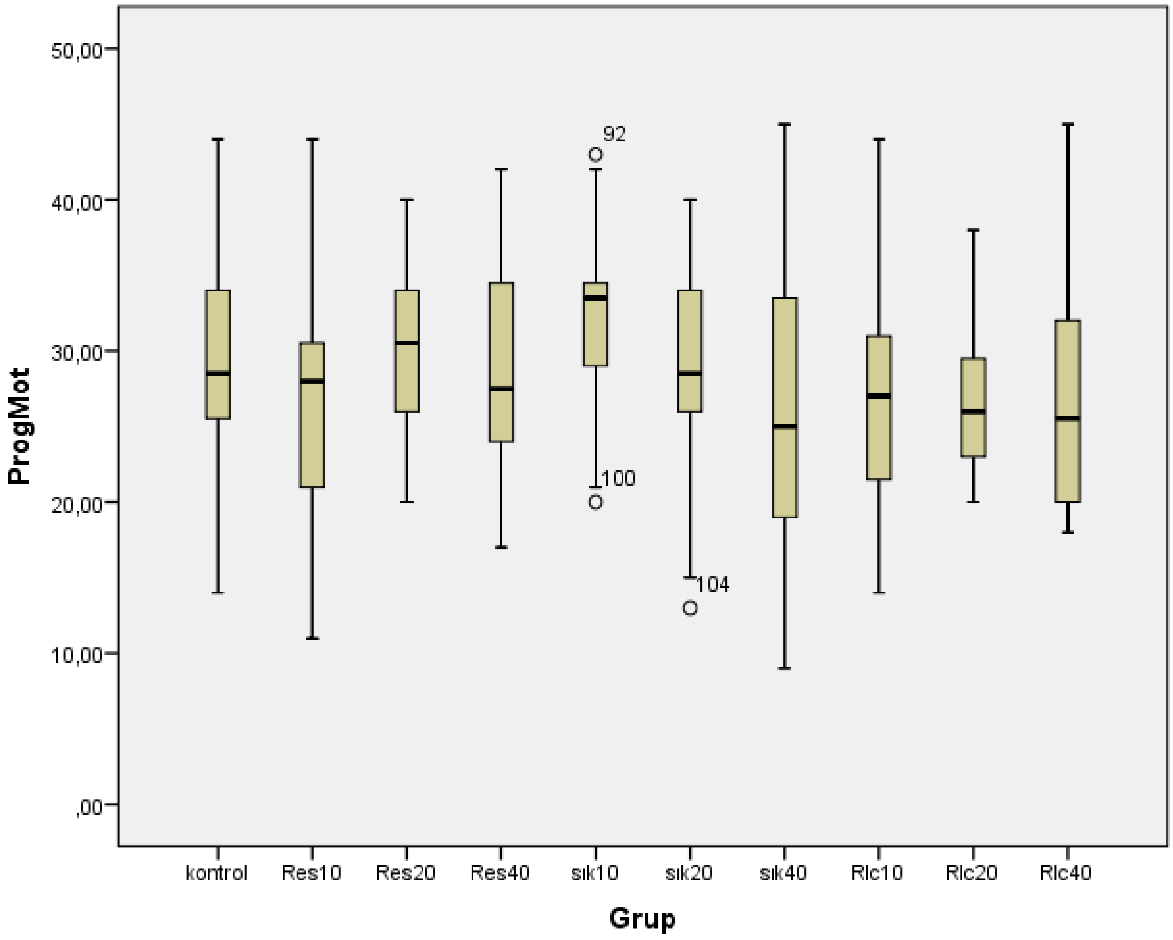


```
ONEWAY SubjMot CasaMot ProgMot BY Grup
  /STATISTICS DESCRIPTIVES HOMOGENEITY
  /PLOT MEANS
  /MISSING ANALYSIS
  /POSTHOC=DUNCAN ALPHA(0.05).
```

Oneway

Notes

Output Created		22-DEC-2020 12:22:00
Comments		
Input	Active Dataset	DataSet0
	Filter	<none>
	Weight	<none>

	Split File	<none>
	N of Rows in Working Data File	200
Missing Value Handling	Definition of Missing	User-defined missing values are treated as missing.
	Cases Used	Statistics for each analysis are based on cases with no missing data for any variable in the analysis.
Syntax		ONEWAY SubjMot CasaMot ProgMot BY Grup /STATISTICS DESCRIPTIVES HOMOGENEITY /PLOT MEANS /MISSING ANALYSIS /POSTHOC=DUNCAN ALPHA(0.05).
Resources	Processor Time	00:00:00,45
	Elapsed Time	00:00:00,46

Descriptives

		N	Mean	Std. Deviation	Std. Error	95% Confidence Interval for Mean
						Lower Bound
SubjMot	kontrol	20	46,0000	8,67543	1,93989	41,9398
	Res10	20	46,5000	8,44487	1,88833	42,5477
	Res20	20	50,5000	7,41620	1,65831	47,0291
	Res40	20	48,2500	6,93485	1,55068	45,0044
	sık10	20	50,0000	8,27170	1,84961	46,1287
	sık20	20	46,7500	9,63478	2,15440	42,2408
	sık40	20	41,5000	11,48225	2,56751	36,1261
	Rlc10	20	47,0000	8,64505	1,93309	42,9540
	Rlc20	20	48,1000	6,29034	1,40656	45,1560
	Rlc40	20	46,5000	7,79676	1,74341	42,8510
	Total	200	47,1100	8,61038	,60885	45,9094
CasaMot	kontrol	20	59,6500	16,38123	3,66295	51,9833
	Res10	20	57,2500	13,76447	3,07783	50,8080
	Res20	20	62,6500	14,60452	3,26567	55,8149
	Res40	20	60,9000	11,48409	2,56792	55,5253
	sık10	20	64,3500	11,82893	2,64503	58,8139

	sık20	20	52,5000	13,30413	2,97489	46,2735
	sık40	20	51,5000	16,55771	3,70242	43,7508
	Rlc10	20	51,3000	11,72537	2,62187	45,8124
	Rlc20	20	56,8500	13,23979	2,96051	50,6536
	Rlc40	20	57,0000	14,31231	3,20033	50,3016
	Total	200	57,3950	14,19813	1,00396	55,4152
ProgMot	kontrol	20	29,1500	7,13239	1,59485	25,8119
	Res10	20	26,3000	8,52921	1,90719	22,3082
	Res20	20	30,4500	5,50096	1,23005	27,8755
	Res40	20	28,9000	7,17378	1,60411	25,5426
	sık10	20	32,6500	6,48297	1,44964	29,6159
	sık20	20	28,9000	6,93504	1,55072	25,6543
	sık40	20	26,2000	10,04516	2,24617	21,4987
	Rlc10	20	26,7500	7,31887	1,63655	23,3247
	Rlc20	20	26,7000	4,84605	1,08361	24,4320
	Rlc40	20	26,5500	7,33754	1,64072	23,1159
	Total	200	28,2550	7,38258	,52203	27,2256

Descriptives

		95% Confidence Interval for Mean		
		Upper Bound	Minimum	Maximum
SubjMot	kontrol	50,0602	30,00	55,00
	Res10	50,4523	30,00	55,00
	Res20	53,9709	30,00	60,00
	Res40	51,4956	30,00	60,00
	sık10	53,8713	30,00	60,00
	sık20	51,2592	25,00	60,00
	sık40	46,8739	20,00	60,00
	Rlc10	51,0460	30,00	60,00
	Rlc20	51,0440	35,00	60,00
	Rlc40	50,1490	30,00	60,00
	Total	48,3106	20,00	60,00
CasaMot	kontrol	67,3167	30,00	83,00
	Res10	63,6920	33,00	75,00
	Res20	69,4851	36,00	87,00
	Res40	66,2747	28,00	81,00
	sık10	69,8861	35,00	84,00
	sık20	58,7265	29,00	78,00
	sık40	59,2492	23,00	76,00

	Rlc10	56,7876	34,00	79,00
	Rlc20	63,0464	41,00	86,00
	Rlc40	63,6984	30,00	86,00
	Total	59,3748	23,00	87,00
ProgMot	kontrol	32,4881	14,00	44,00
	Res10	30,2918	11,00	44,00
	Res20	33,0245	20,00	40,00
	Res40	32,2574	17,00	42,00
	sık10	35,6841	20,00	43,00
	sık20	32,1457	13,00	40,00
	sık40	30,9013	9,00	45,00
	Rlc10	30,1753	14,00	44,00
	Rlc20	28,9680	20,00	38,00
	Rlc40	29,9841	18,00	45,00
	Total	29,2844	9,00	45,00

Test of Homogeneity of Variances

	Levene Statistic	df1	df2	Sig.
SubjMot	2,357	9	190	,015
CasaMot	1,454	9	190	,168
ProgMot	1,359	9	190	,210

ANOVA

		Sum of Squares	df	Mean Square	F	Sig.
SubjMot	Between Groups	1114,280	9	123,809	1,725	,086
	Within Groups	13639,300	190	71,786		
	Total	14753,580	199			
CasaMot	Between Groups	3793,845	9	421,538	2,205	,023
	Within Groups	36321,950	190	191,168		
	Total	40115,795	199			
ProgMot	Between Groups	828,045	9	92,005	1,745	,081
	Within Groups	10017,950	190	52,726		
	Total	10845,995	199			

Post Hoc Tests

Homogeneous Subsets

SubjMot

Duncan[a]

Grup	N	Subset for alpha = 0.05	
		1	2
sık40	20	41,5000	
kontrol	20	46,0000	46,0000
Res10	20	46,5000	46,5000
Rlc40	20	46,5000	46,5000
sık20	20	46,7500	46,7500
Rlc10	20	47,0000	47,0000
Rlc20	20		48,1000
Res40	20		48,2500
sık10	20		50,0000
Res20	20		50,5000
Sig.		,073	,163

Means for groups in homogeneous subsets are displayed.

a. Uses Harmonic Mean Sample Size = 20,000.

CasaMot

Duncan[a]

Grup	N	Subset for alpha = 0.05	
		1	2
Rlc10	20	51,3000	
sık40	20	51,5000	
sık20	20	52,5000	
Rlc20	20	56,8500	56,8500
Rlc40	20	57,0000	57,0000
Res10	20	57,2500	57,2500
kontrol	20	59,6500	59,6500

Res40	20	60,9000	60,9000
Res20	20		62,6500
sık10	20		64,3500
Sig.		,062	,144

Means for groups in homogeneous subsets are displayed.

a. Uses Harmonic Mean Sample Size = 20,000.

ProgMot

Duncan[a]

Grup	N	Subset for alpha = 0.05	
		1	2
sık40	20	26,2000	
Res10	20	26,3000	
Rlc40	20	26,5500	
Rlc20	20	26,7000	
Rlc10	20	26,7500	
Res40	20	28,9000	28,9000
sık20	20	28,9000	28,9000
kontrol	20	29,1500	29,1500
Res20	20	30,4500	30,4500
sık10	20		32,6500
Sig.		,122	,150

Means for groups in homogeneous subsets are displayed.

a. Uses Harmonic Mean Sample Size = 20,000.

Means Plots

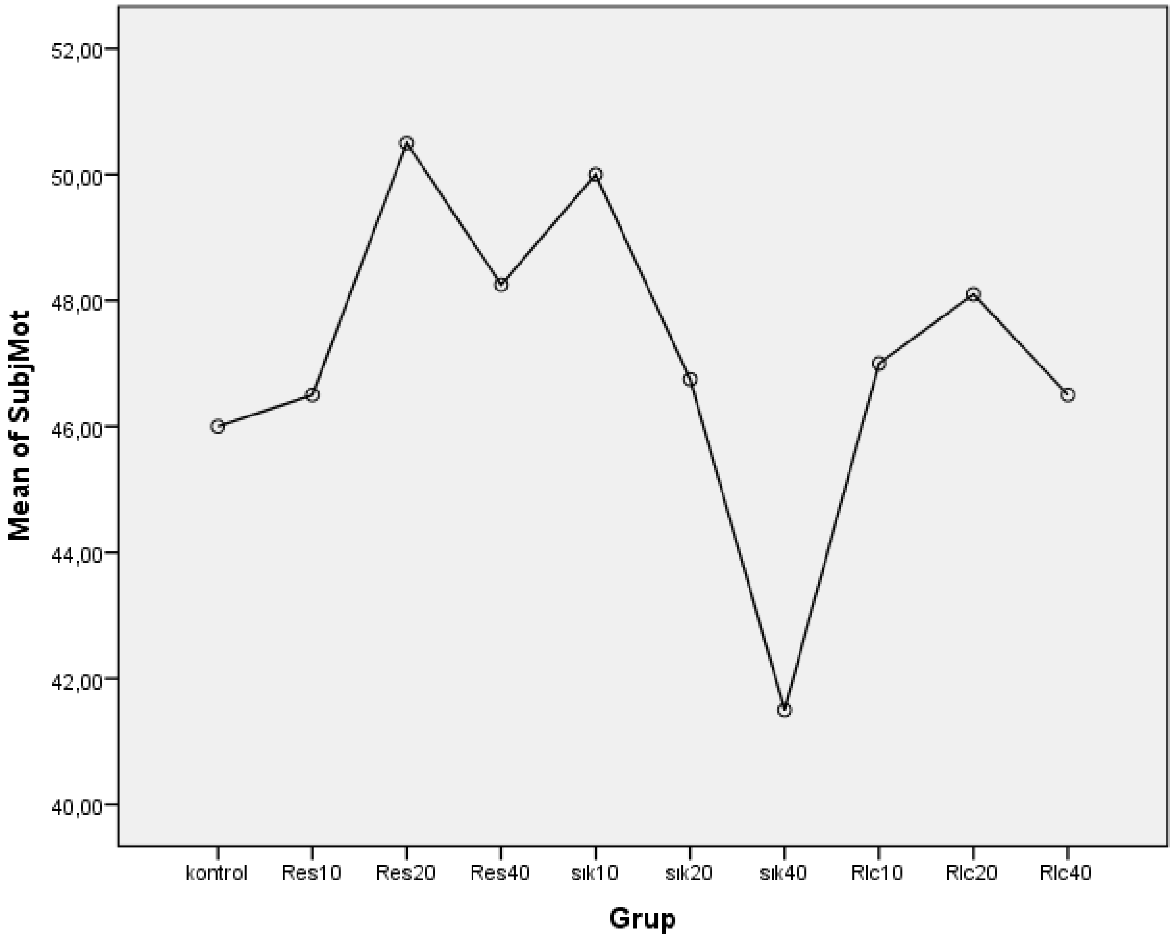
52,00
50,00
48,00
46,00
44,00
42,00
40,00
Mean of SubjMot
kontrol
Res10
Res20
Res40
sık10
sık20
sık40
Rlc10
Rlc20
Rlc40
Grup

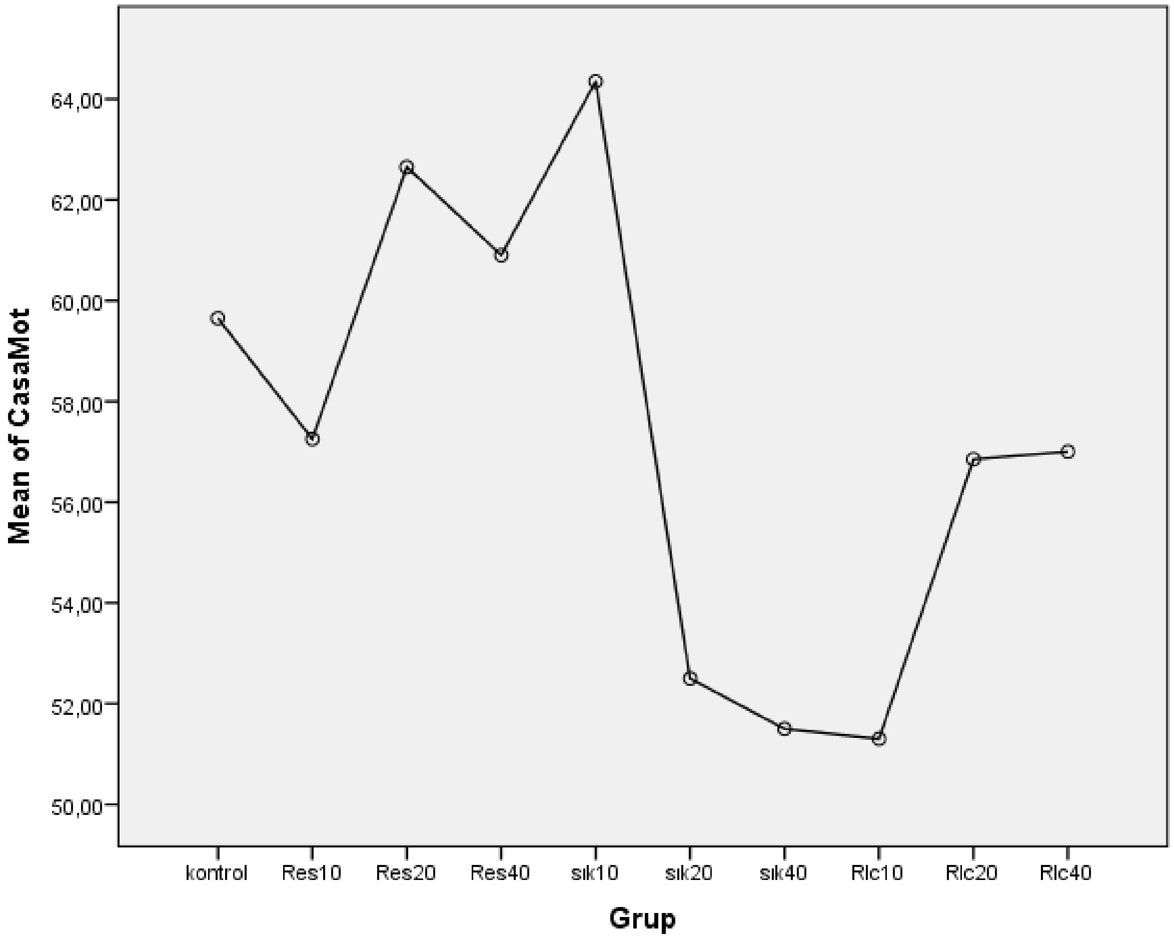
Mean of CasaMot
64,00
62,00
60,00
58,00
56,00
54,00
52,00
50,00
kontrol
Res10
Res20
Res40
sık10
sık20
sık40
Rlc10
Rlc20
Rlc40
Grup

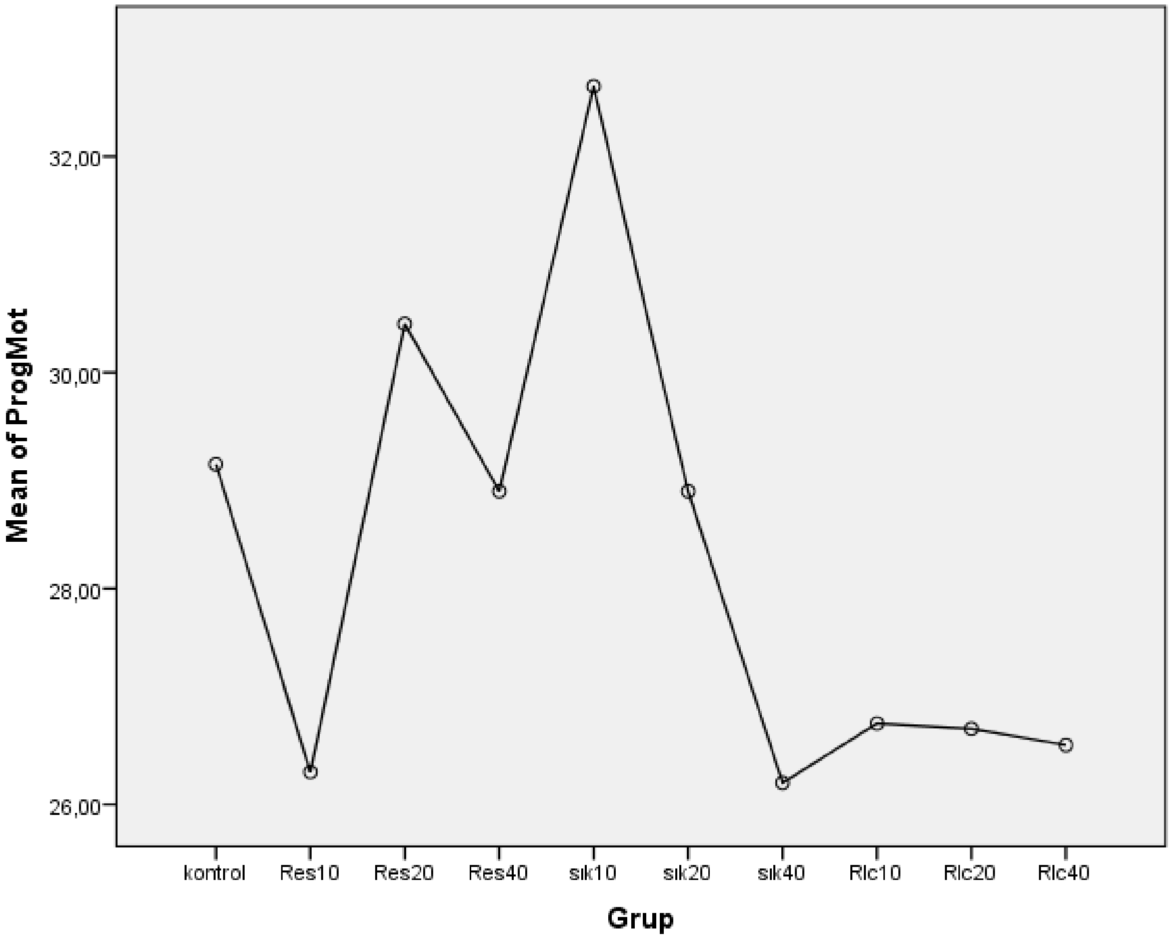
Mean of ProgMot
32,00
30,00
28,00
26,00
kontrol
Res10
Res20
Res40
sık10
sık20
sık40
Rlc10
Rlc20
Rlc40
Grup

Sperm velocity parameters of post-thawed ram sperm- ANOVA

Groups

1: Control

2: RES10

3: RES20

4: RES40

5: CD10

6: CD20

7: CD40

8: RLC10

9: RLC20

10: RLC40

Explore

Notes

Output Created		23-DEC-2020 21:40:35
Comments		
Input	Active Dataset	DataSet0
	Filter	<none>
	Weight	<none>
	Split File	<none>
	N of Rows in Working Data File	200
Missing Value Handling	Definition of Missing	User-defined missing values for dependent variables are treated as missing.
	Cases Used	Statistics are based on cases with no missing values for any dependent variable or factor used.

Syntax		EXAMINE VARIABLES=VAP VSL VCL ALH BCF STR LIN BY Gruplar<br>/PLOT BOXPLOT STEMLEAF HISTOGRAM NPPLOT<br>/COMPARE VARIABLES<br>/STATISTICS DESCRIPTIVES<br>/CINTERVAL 95<br>/MISSING LISTWISE<br>/NOTOTAL.
Resources	Processor Time	00:00:31,70
	Elapsed Time	00:00:28,66

[DataSet0]

Gruplar

Case Processing Summary

		Cases					
		Valid		Missing		Total	
	Gruplar	N	Percent	N	Percent	N	Percent
VAP	1,00	20	100,0%	0	0,0%	20	100,0%
	2,00	20	100,0%	0	0,0%	20	100,0%
	3,00	20	100,0%	0	0,0%	20	100,0%
	4,00	20	100,0%	0	0,0%	20	100,0%
	5,00	20	100,0%	0	0,0%	20	100,0%
	6,00	20	100,0%	0	0,0%	20	100,0%
	7,00	20	100,0%	0	0,0%	20	100,0%
	8,00	20	100,0%	0	0,0%	20	100,0%
	9,00	20	100,0%	0	0,0%	20	100,0%
	10,00	20	100,0%	0	0,0%	20	100,0%
VSL	1,00	20	100,0%	0	0,0%	20	100,0%
	2,00	20	100,0%	0	0,0%	20	100,0%
	3,00	20	100,0%	0	0,0%	20	100,0%
	4,00	20	100,0%	0	0,0%	20	100,0%
	5,00	20	100,0%	0	0,0%	20	100,0%

	6,00	20	100,0%	0	0,0%	20	100,0%
	7,00	20	100,0%	0	0,0%	20	100,0%
	8,00	20	100,0%	0	0,0%	20	100,0%
	9,00	20	100,0%	0	0,0%	20	100,0%
	10,00	20	100,0%	0	0,0%	20	100,0%
VCL	1,00	20	100,0%	0	0,0%	20	100,0%
	2,00	20	100,0%	0	0,0%	20	100,0%
	3,00	20	100,0%	0	0,0%	20	100,0%
	4,00	20	100,0%	0	0,0%	20	100,0%
	5,00	20	100,0%	0	0,0%	20	100,0%
	6,00	20	100,0%	0	0,0%	20	100,0%
	7,00	20	100,0%	0	0,0%	20	100,0%
	8,00	20	100,0%	0	0,0%	20	100,0%
	9,00	20	100,0%	0	0,0%	20	100,0%
	10,00	20	100,0%	0	0,0%	20	100,0%
ALH	1,00	20	100,0%	0	0,0%	20	100,0%
	2,00	20	100,0%	0	0,0%	20	100,0%
	3,00	20	100,0%	0	0,0%	20	100,0%
	4,00	20	100,0%	0	0,0%	20	100,0%
	5,00	20	100,0%	0	0,0%	20	100,0%
	6,00	20	100,0%	0	0,0%	20	100,0%
	7,00	20	100,0%	0	0,0%	20	100,0%
	8,00	20	100,0%	0	0,0%	20	100,0%
	9,00	20	100,0%	0	0,0%	20	100,0%
	10,00	20	100,0%	0	0,0%	20	100,0%
BCF	1,00	20	100,0%	0	0,0%	20	100,0%
	2,00	20	100,0%	0	0,0%	20	100,0%
	3,00	20	100,0%	0	0,0%	20	100,0%
	4,00	20	100,0%	0	0,0%	20	100,0%
	5,00	20	100,0%	0	0,0%	20	100,0%
	6,00	20	100,0%	0	0,0%	20	100,0%
	7,00	20	100,0%	0	0,0%	20	100,0%
	8,00	20	100,0%	0	0,0%	20	100,0%
	9,00	20	100,0%	0	0,0%	20	100,0%
	10,00	20	100,0%	0	0,0%	20	100,0%
STR	1,00	20	100,0%	0	0,0%	20	100,0%
	2,00	20	100,0%	0	0,0%	20	100,0%
	3,00	20	100,0%	0	0,0%	20	100,0%
	4,00	20	100,0%	0	0,0%	20	100,0%
	5,00	20	100,0%	0	0,0%	20	100,0%
	6,00	20	100,0%	0	0,0%	20	100,0%

	7,00	20	100,0%	0	0,0%	20	100,0%
	8,00	20	100,0%	0	0,0%	20	100,0%
	9,00	20	100,0%	0	0,0%	20	100,0%
	10,00	20	100,0%	0	0,0%	20	100,0%
LIN	1,00	20	100,0%	0	0,0%	20	100,0%
	2,00	20	100,0%	0	0,0%	20	100,0%
	3,00	20	100,0%	0	0,0%	20	100,0%
	4,00	20	100,0%	0	0,0%	20	100,0%
	5,00	20	100,0%	0	0,0%	20	100,0%
	6,00	20	100,0%	0	0,0%	20	100,0%
	7,00	20	100,0%	0	0,0%	20	100,0%
	8,00	20	100,0%	0	0,0%	20	100,0%
	9,00	20	100,0%	0	0,0%	20	100,0%
	10,00	20	100,0%	0	0,0%	20	100,0%

Descriptives

	Gruplar			Statistic	Std. Error
VAP	1,00	Mean		95,3500	2,84999
		95% Confidence Interval for Mean	Lower Bound	89,3849	
			Upper Bound	101,3151	
		5% Trimmed Mean		94,5111	
		Median		91,2000	
		Variance		162,449	
		Std. Deviation		12,74555	
		Minimum		80,90	
		Maximum		124,90	
		Range		44,00	
		Interquartile Range		16,83	
		Skewness		1,091	,512
		Kurtosis		,237	,992
	2,00	Mean		90,5250	2,27798
		95% Confidence Interval for Mean	Lower Bound	85,7571	
			Upper Bound	95,2929	
		5% Trimmed Mean		90,0889	
		Median		88,4500	
		Variance		103,784	
		Std. Deviation		10,18745	

	Minimum		77,80	
	Maximum		111,10	
	Range		33,30	
	Interquartile Range		17,75	
	Skewness		,365	,512
	Kurtosis		-1,084	,992
3,00	Mean		92,7400	1,92189
	95% Confidence Interval for Mean	Lower Bound	88,7174	
		Upper Bound	96,7626	
	5% Trimmed Mean		92,4611	
	Median		92,8500	
	Variance		73,873	
	Std. Deviation		8,59494	
	Minimum		79,10	
	Maximum		111,40	
	Range		32,30	
	Interquartile Range		8,80	
	Skewness		,619	,512
	Kurtosis		,678	,992
4,00	Mean		93,1000	2,16577
	95% Confidence Interval for Mean	Lower Bound	88,5670	
		Upper Bound	97,6330	
	5% Trimmed Mean		93,1778	
	Median		94,1000	
	Variance		93,812	
	Std. Deviation		9,68564	
	Minimum		75,70	
	Maximum		109,10	
	Range		33,40	
	Interquartile Range		13,88	
	Skewness		,093	,512
	Kurtosis		-,857	,992
5,00	Mean		98,1550	1,72918
	95% Confidence Interval for Mean	Lower Bound	94,5358	
		Upper Bound	101,7742	
	5% Trimmed Mean		98,1222	
	Median		97,0500	
	Variance		59,802	

	Std. Deviation		7,73315	
	Minimum		82,30	
	Maximum		114,60	
	Range		32,30	
	Interquartile Range		10,13	
	Skewness		,187	,512
	Kurtosis		,207	,992
6,00	Mean		95,4350	2,54970
	95% Confidence Interval for Mean	Lower Bound	90,0984	
		Upper Bound	100,7716	
	5% Trimmed Mean		94,4889	
	Median		92,8500	
	Variance		130,019	
	Std. Deviation		11,40260	
	Minimum		81,40	
	Maximum		126,50	
	Range		45,10	
	Interquartile Range		16,80	
	Skewness		1,187	,512
	Kurtosis		1,401	,992
7,00	Mean		95,5050	1,94227
	95% Confidence Interval for Mean	Lower Bound	91,4398	
		Upper Bound	99,5702	
	5% Trimmed Mean		95,5667	
	Median		94,8000	
	Variance		75,448	
	Std. Deviation		8,68607	
	Minimum		80,30	
	Maximum		109,60	
	Range		29,30	
	Interquartile Range		15,15	
	Skewness		,013	,512
	Kurtosis		-1,035	,992
8,00	Mean		92,3750	1,99479
	95% Confidence Interval for Mean	Lower Bound	88,1998	
		Upper Bound	96,5502	
	5% Trimmed Mean		92,4222	
	Median		92,8500	

		Variance		79,584	
		Std. Deviation		8,92099	
		Minimum		76,70	
		Maximum		107,20	
		Range		30,50	
		Interquartile Range		15,53	
		Skewness		-,106	,512
		Kurtosis		-,827	,992
	9,00	Mean		94,6100	2,59216
		95% Confidence Interval for Mean	Lower Bound	89,1845	
			Upper Bound	100,0355	
		5% Trimmed Mean		93,9278	
		Median		93,1000	
		Variance		134,386	
		Std. Deviation		11,59251	
		Minimum		76,00	
		Maximum		125,50	
		Range		49,50	
		Interquartile Range		13,00	
		Skewness		1,022	,512
		Kurtosis		1,379	,992
	10,00	Mean		92,7850	2,35121
		95% Confidence Interval for Mean	Lower Bound	87,8639	
			Upper Bound	97,7061	
		5% Trimmed Mean		91,8444	
		Median		91,5000	
		Variance		110,563	
		Std. Deviation		10,51492	
		Minimum		79,10	
		Maximum		123,40	
		Range		44,30	
		Interquartile Range		13,38	
		Skewness		1,173	,512
		Kurtosis		2,509	,992
VSL	1,00	Mean		84,8450	3,14361
		95% Confidence Interval for Mean	Lower Bound	78,2653	
			Upper Bound	91,4247	
		5% Trimmed Mean		83,8556	

	Median		81,1500	
	Variance		197,646	
	Std. Deviation		14,05865	
	Minimum		69,90	
	Maximum		117,60	
	Range		47,70	
	Interquartile Range		20,70	
	Skewness		1,035	,512
	Kurtosis		,164	,992
2,00	Mean		79,7850	2,34138
	95% Confidence Interval for Mean	Lower Bound	74,8844	
		Upper Bound	84,6856	
	5% Trimmed Mean		79,4611	
	Median		79,4000	
	Variance		109,641	
	Std. Deviation		10,47098	
	Minimum		63,90	
	Maximum		101,50	
	Range		37,60	
	Interquartile Range		16,75	
	Skewness		,371	,512
	Kurtosis		-,734	,992
3,00	Mean		82,1450	2,30655
	95% Confidence Interval for Mean	Lower Bound	77,3173	
		Upper Bound	86,9727	
	5% Trimmed Mean		81,9500	
	Median		80,6000	
	Variance		106,404	
	Std. Deviation		10,31521	
	Minimum		62,90	
	Maximum		104,90	
	Range		42,00	
	Interquartile Range		12,80	
	Skewness		,678	,512
	Kurtosis		,736	,992
4,00	Mean		82,0850	2,51691
	95% Confidence Interval for Mean	Lower Bound	76,8170	
		Upper Bound	87,3530	
	5% Trimmed Mean		82,1944	
	Median		79,7500	
	Variance		126,697	

	Std. Deviation		11,25598	
	Minimum		62,20	
	Maximum		100,00	
	Range		37,80	
	Interquartile Range		19,22	
	Skewness		,188	,512
	Kurtosis		-,939	,992
5,00	Mean		87,6150	2,00244
	95% Confidence Interval for Mean	Lower Bound	83,4239	
		Upper Bound	91,8061	
	5% Trimmed Mean		87,6611	
	Median		87,7000	
	Variance		80,195	
	Std. Deviation		8,95517	
	Minimum		68,80	
	Maximum		105,60	
	Range		36,80	
	Interquartile Range		12,00	
	Skewness		,072	,512
	Kurtosis		-,061	,992
6,00	Mean		85,0250	2,73696
	95% Confidence Interval for Mean	Lower Bound	79,2965	
		Upper Bound	90,7535	
	5% Trimmed Mean		84,1611	
	Median		81,0000	
	Variance		149,819	
	Std. Deviation		12,24005	
	Minimum		70,30	
	Maximum		115,30	
	Range		45,00	
	Interquartile Range		20,38	
	Skewness		,940	,512
	Kurtosis		,170	,992
7,00	Mean		85,2150	2,28784
	95% Confidence Interval for Mean	Lower Bound	80,4265	
		Upper Bound	90,0035	
	5% Trimmed Mean		85,1722	
	Median		82,4000	
	Variance		104,685	
	Std. Deviation		10,23154	
	Minimum		68,50	

	Maximum		102,70	
	Range		34,20	
	Interquartile Range		17,48	
	Skewness		,292	,512
	Kurtosis		-1,109	,992
8,00	Mean		81,4550	2,56364
	95% Confidence Interval for Mean	Lower Bound	76,0892	
		Upper Bound	86,8208	
	5% Trimmed Mean		81,4722	
	Median		84,7000	
	Variance		131,445	
	Std. Deviation		11,46493	
	Minimum		61,90	
	Maximum		100,70	
	Range		38,80	
	Interquartile Range		20,80	
	Skewness		-,108	,512
	Kurtosis		-,966	,992
9,00	Mean		83,5250	2,98930
	95% Confidence Interval for Mean	Lower Bound	77,2683	
		Upper Bound	89,7817	
	5% Trimmed Mean		82,9667	
	Median		81,3500	
	Variance		178,719	
	Std. Deviation		13,36858	
	Minimum		62,30	
	Maximum		114,80	
	Range		52,50	
	Interquartile Range		19,30	
	Skewness		,658	,512
	Kurtosis		,177	,992
10,00	Mean		81,1200	2,92932
	95% Confidence Interval for Mean	Lower Bound	74,9889	
		Upper Bound	87,2511	
	5% Trimmed Mean		80,2556	
	Median		80,8000	
	Variance		171,619	
	Std. Deviation		13,10033	
	Minimum		62,50	
	Maximum		115,30	
	Range		52,80	

		Interquartile Range		20,17	
		Skewness		,675	,512
		Kurtosis		,948	,992
VCL	1,00	Mean		146,8450	3,39984
		95% Confidence Interval for Mean	Lower Bound	139,7291	
			Upper Bound	153,9609	
		5% Trimmed Mean		146,0000	
		Median		144,9000	
		Variance		231,178	
		Std. Deviation		15,20455	
		Minimum		125,20	
		Maximum		183,70	
		Range		58,50	
		Interquartile Range		20,25	
		Skewness		,869	,512
		Kurtosis		,536	,992
	2,00	Mean		140,7750	3,38213
		95% Confidence Interval for Mean	Lower Bound	133,6961	
			Upper Bound	147,8539	
		5% Trimmed Mean		140,1056	
		Median		135,4500	
		Variance		228,776	
		Std. Deviation		15,12533	
		Minimum		121,20	
		Maximum		172,40	
		Range		51,20	
		Interquartile Range		19,33	
		Skewness		,938	,512
		Kurtosis		-,085	,992
	3,00	Mean		142,2750	2,66321
		95% Confidence Interval for Mean	Lower Bound	136,7008	
			Upper Bound	147,8492	
		5% Trimmed Mean		142,3667	
		Median		140,7000	
		Variance		141,854	
		Std. Deviation		11,91023	
		Minimum		119,60	
		Maximum		163,30	
		Range		43,70	
		Interquartile Range		14,78	
		Skewness		-,277	,512

	Kurtosis		-,249	,992
4,00	Mean		143,6550	2,90537
	95% Confidence Interval for Mean	Lower Bound	137,5740	
		Upper Bound	149,7360	
	5% Trimmed Mean		143,7667	
	Median		142,8500	
	Variance		168,824	
	Std. Deviation		12,99322	
	Minimum		123,20	
	Maximum		162,10	
	Range		38,90	
	Interquartile Range		26,00	
	Skewness		-,016	,512
	Kurtosis		-1,478	,992
5,00	Mean		148,1050	2,84206
	95% Confidence Interval for Mean	Lower Bound	142,1565	
		Upper Bound	154,0535	
	5% Trimmed Mean		147,4611	
	Median		150,0500	
	Variance		161,546	
	Std. Deviation		12,71007	
	Minimum		128,70	
	Maximum		179,10	
	Range		50,40	
	Interquartile Range		18,65	
	Skewness		,423	,512
	Kurtosis		,457	,992
6,00	Mean		149,0550	3,90230
	95% Confidence Interval for Mean	Lower Bound	140,8874	
		Upper Bound	157,2226	
	5% Trimmed Mean		147,7389	
	Median		147,8000	
	Variance		304,559	
	Std. Deviation		17,45163	
	Minimum		121,80	
	Maximum		200,00	
	Range		78,20	
	Interquartile Range		16,57	
	Skewness		1,468	,512
	Kurtosis		3,315	,992
7,00	Mean		147,1400	2,77532

	95% Confidence Interval for Mean	Lower Bound	141,3312	
		Upper Bound	152,9488	
	5% Trimmed Mean		146,9944	
	Median		148,8500	
	Variance		154,048	
	Std. Deviation		12,41160	
	Minimum		126,20	
	Maximum		170,70	
	Range		44,50	
	Interquartile Range		19,50	
	Skewness		-,132	,512
	Kurtosis		-,679	,992
8,00	Mean		142,2500	2,24227
	95% Confidence Interval for Mean	Lower Bound	137,5569	
		Upper Bound	146,9431	
	5% Trimmed Mean		142,2000	
	Median		142,5500	
	Variance		100,555	
	Std. Deviation		10,02772	
	Minimum		126,10	
	Maximum		159,30	
	Range		33,20	
	Interquartile Range		16,07	
	Skewness		,066	,512
	Kurtosis		-1,017	,992
9,00	Mean		146,1800	3,01605
	95% Confidence Interval for Mean	Lower Bound	139,8673	
		Upper Bound	152,4927	
	5% Trimmed Mean		146,1444	
	Median		146,6000	
	Variance		181,931	
	Std. Deviation		13,48819	
	Minimum		120,90	
	Maximum		172,10	
	Range		51,20	
	Interquartile Range		16,13	
	Skewness		,027	,512
	Kurtosis		-,312	,992
10,00	Mean		143,7150	2,64854
	95% Confidence Interval for Mean	Lower Bound	138,1715	
		Upper Bound	149,2585	

		5% Trimmed Mean		143,2556	
		Median		142,3500	
		Variance		140,295	
		Std. Deviation		11,84462	
		Minimum		119,70	
		Maximum		176,00	
		Range		56,30	
		Interquartile Range		13,45	
		Skewness		,806	,512
		Kurtosis		2,226	,992
ALH	1,00	Mean		5,7950	,13907
		95% Confidence Interval for Mean	Lower Bound	5,5039	
			Upper Bound	6,0861	
		5% Trimmed Mean		5,7778	
		Median		5,9000	
		Variance		,387	
		Std. Deviation		,62195	
		Minimum		4,90	
		Maximum		7,00	
		Range		2,10	
		Interquartile Range		,95	
		Skewness		,328	,512
		Kurtosis		-,601	,992
	2,00	Mean		5,9900	,16074
		95% Confidence Interval for Mean	Lower Bound	5,6536	
			Upper Bound	6,3264	
		5% Trimmed Mean		5,9500	
		Median		5,8000	
		Variance		,517	
		Std. Deviation		,71884	
		Minimum		5,20	
		Maximum		7,50	
		Range		2,30	
		Interquartile Range		1,05	
		Skewness		,884	,512
		Kurtosis		-,089	,992
	3,00	Mean		5,8100	,20824
		95% Confidence Interval for Mean	Lower Bound	5,3742	
			Upper Bound	6,2458	
		5% Trimmed Mean		5,7556	
		Median		5,7500	

	Variance		,867	
	Std. Deviation		,93127	
	Minimum		4,50	
	Maximum		8,10	
	Range		3,60	
	Interquartile Range		1,42	
	Skewness		,496	,512
	Kurtosis		,327	,992
4,00	Mean		5,9000	,21325
	95% Confidence Interval for Mean	Lower Bound	5,4537	
		Upper Bound	6,3463	
	5% Trimmed Mean		5,9000	
	Median		5,5500	
	Variance		,909	
	Std. Deviation		,95366	
	Minimum		4,30	
	Maximum		7,50	
	Range		3,20	
	Interquartile Range		1,65	
	Skewness		,237	,512
	Kurtosis		-1,192	,992
5,00	Mean		5,7050	,18460
	95% Confidence Interval for Mean	Lower Bound	5,3186	
		Upper Bound	6,0914	
	5% Trimmed Mean		5,7222	
	Median		5,8000	
	Variance		,682	
	Std. Deviation		,82556	
	Minimum		4,20	
	Maximum		6,90	
	Range		2,70	
	Interquartile Range		1,38	
	Skewness		-,350	,512
	Kurtosis		-,975	,992
6,00	Mean		5,9000	,16075
	95% Confidence Interval for Mean	Lower Bound	5,5635	
		Upper Bound	6,2365	
	5% Trimmed Mean		5,8889	
	Median		5,7500	
	Variance		,517	
	Std. Deviation		,71892	

	Minimum		4,70	
	Maximum		7,30	
	Range		2,60	
	Interquartile Range		1,38	
	Skewness		,327	,512
	Kurtosis		-1,018	,992
7,00	Mean		5,7550	,18588
	95% Confidence Interval for Mean	Lower Bound	5,3659	
		Upper Bound	6,1441	
	5% Trimmed Mean		5,7222	
	Median		5,6000	
	Variance		,691	
	Std. Deviation		,83128	
	Minimum		4,70	
	Maximum		7,40	
	Range		2,70	
	Interquartile Range		1,35	
	Skewness		,531	,512
	Kurtosis		-,740	,992
8,00	Mean		5,8500	,19728
	95% Confidence Interval for Mean	Lower Bound	5,4371	
		Upper Bound	6,2629	
	5% Trimmed Mean		5,8611	
	Median		5,5000	
	Variance		,778	
	Std. Deviation		,88228	
	Minimum		4,30	
	Maximum		7,20	
	Range		2,90	
	Interquartile Range		1,55	
	Skewness		,131	,512
	Kurtosis		-1,318	,992
9,00	Mean		5,9600	,17747
	95% Confidence Interval for Mean	Lower Bound	5,5886	
		Upper Bound	6,3314	
	5% Trimmed Mean		5,9556	
	Median		5,8000	
	Variance		,630	
	Std. Deviation		,79366	
	Minimum		4,90	
	Maximum		7,10	

		Range		2,20	
		Interquartile Range		1,65	
		Skewness		,185	,512
		Kurtosis		-1,749	,992
	10,00	Mean		5,9400	,20071
		95% Confidence Interval for Mean	Lower Bound	5,5199	
			Upper Bound	6,3601	
		5% Trimmed Mean		5,9056	
		Median		5,6500	
		Variance		,806	
		Std. Deviation		,89760	
		Minimum		4,80	
		Maximum		7,70	
		Range		2,90	
		Interquartile Range		1,47	
		Skewness		,569	,512
		Kurtosis		-,950	,992
BCF	1,00	Mean		37,1550	,71460
		95% Confidence Interval for Mean	Lower Bound	35,6593	
			Upper Bound	38,6507	
		5% Trimmed Mean		37,1556	
		Median		36,9000	
		Variance		10,213	
		Std. Deviation		3,19580	
		Minimum		31,30	
		Maximum		43,00	
		Range		11,70	
		Interquartile Range		5,52	
		Skewness		,071	,512
		Kurtosis		-,821	,992
	2,00	Mean		34,9750	,64579
		95% Confidence Interval for Mean	Lower Bound	33,6233	
			Upper Bound	36,3267	
		5% Trimmed Mean		35,0167	
		Median		34,6000	
		Variance		8,341	
		Std. Deviation		2,88807	
		Minimum		29,00	
		Maximum		40,20	
		Range		11,20	
		Interquartile Range		3,88	

	Skewness		,085	,512
	Kurtosis		-,159	,992
3,00	Mean		35,6900	,72351
	95% Confidence Interval for Mean	Lower Bound	34,1757	
		Upper Bound	37,2043	
	5% Trimmed Mean		35,8389	
	Median		35,4500	
	Variance		10,469	
	Std. Deviation		3,23564	
	Minimum		26,60	
	Maximum		42,10	
	Range		15,50	
	Interquartile Range		3,05	
	Skewness		-,739	,512
	Kurtosis		2,643	,992
4,00	Mean		35,3600	,75997
	95% Confidence Interval for Mean	Lower Bound	33,7694	
		Upper Bound	36,9506	
	5% Trimmed Mean		35,3611	
	Median		35,2500	
	Variance		11,551	
	Std. Deviation		3,39867	
	Minimum		28,70	
	Maximum		42,00	
	Range		13,30	
	Interquartile Range		5,60	
	Skewness		,087	,512
	Kurtosis		-,491	,992
5,00	Mean		37,2500	,52683
	95% Confidence Interval for Mean	Lower Bound	36,1473	
		Upper Bound	38,3527	
	5% Trimmed Mean		37,0833	
	Median		36,7500	
	Variance		5,551	
	Std. Deviation		2,35607	
	Minimum		33,80	
	Maximum		43,70	
	Range		9,90	
	Interquartile Range		2,02	
	Skewness		1,321	,512
	Kurtosis		2,129	,992

6,00	Mean		37,3950	,60313
	95% Confidence Interval for Mean	Lower Bound	36,1326	
		Upper Bound	38,6574	
	5% Trimmed Mean		37,4222	
	Median		37,4000	
	Variance		7,275	
	Std. Deviation		2,69726	
	Minimum		32,10	
	Maximum		42,20	
	Range		10,10	
	Interquartile Range		3,85	
	Skewness		-,181	,512
	Kurtosis		-,359	,992
7,00	Mean		36,8950	,70144
	95% Confidence Interval for Mean	Lower Bound	35,4269	
		Upper Bound	38,3631	
	5% Trimmed Mean		36,8667	
	Median		36,9000	
	Variance		9,840	
	Std. Deviation		3,13696	
	Minimum		32,60	
	Maximum		41,70	
	Range		9,10	
	Interquartile Range		6,23	
	Skewness		,089	,512
	Kurtosis		-1,254	,992
8,00	Mean		36,2750	,89369
	95% Confidence Interval for Mean	Lower Bound	34,4045	
		Upper Bound	38,1455	
	5% Trimmed Mean		36,2889	
	Median		37,1500	
	Variance		15,974	
	Std. Deviation		3,99669	
	Minimum		29,50	
	Maximum		42,80	
	Range		13,30	
	Interquartile Range		6,30	
	Skewness		-,076	,512
	Kurtosis		-1,004	,992
9,00	Mean		35,4250	,84183
	95% Confidence Interval for	Lower Bound	33,6630	

		Mean	Upper Bound	37,1870	
		5% Trimmed Mean		35,5278	
		Median		35,9000	
		Variance		14,174	
		Std. Deviation		3,76478	
		Minimum		27,10	
		Maximum		41,90	
		Range		14,80	
		Interquartile Range		6,02	
		Skewness		-,266	,512
		Kurtosis		-,305	,992
	10,00	Mean		35,4250	1,02910
		95% Confidence Interval for Mean	Lower Bound	33,2711	
			Upper Bound	37,5789	
		5% Trimmed Mean		35,5944	
		Median		35,3000	
		Variance		21,181	
		Std. Deviation		4,60227	
		Minimum		25,40	
		Maximum		42,40	
		Range		17,00	
		Interquartile Range		7,38	
		Skewness		-,426	,512
		Kurtosis		-,338	,992
STR	1,00	Mean		85,8500	,85000
		95% Confidence Interval for Mean	Lower Bound	84,0709	
			Upper Bound	87,6291	
		5% Trimmed Mean		85,8889	
		Median		85,5000	
		Variance		14,450	
		Std. Deviation		3,80132	
		Minimum		78,00	
		Maximum		93,00	
		Range		15,00	
		Interquartile Range		4,75	
		Skewness		-,133	,512
		Kurtosis		-,249	,992
	2,00	Mean		84,9500	,78965
		95% Confidence Interval for Mean	Lower Bound	83,2972	
			Upper Bound	86,6028	
		5% Trimmed Mean		85,0556	

	Median		85,0000	
	Variance		12,471	
	Std. Deviation		3,53144	
	Minimum		77,00	
	Maximum		91,00	
	Range		14,00	
	Interquartile Range		4,75	
	Skewness		-,368	,512
	Kurtosis		,088	,992
3,00	Mean		85,9000	,94284
	95% Confidence Interval for Mean	Lower Bound	83,9266	
		Upper Bound	87,8734	
	5% Trimmed Mean		85,8333	
	Median		86,5000	
	Variance		17,779	
	Std. Deviation		4,21651	
	Minimum		80,00	
	Maximum		93,00	
	Range		13,00	
	Interquartile Range		7,50	
	Skewness		-,049	,512
	Kurtosis		-1,128	,992
4,00	Mean		85,1000	1,06598
	95% Confidence Interval for Mean	Lower Bound	82,8689	
		Upper Bound	87,3311	
	5% Trimmed Mean		85,1667	
	Median		85,0000	
	Variance		22,726	
	Std. Deviation		4,76721	
	Minimum		77,00	
	Maximum		92,00	
	Range		15,00	
	Interquartile Range		8,75	
	Skewness		-,150	,512
	Kurtosis		-1,330	,992
5,00	Mean		85,9500	,87802
	95% Confidence Interval for Mean	Lower Bound	84,1123	
		Upper Bound	87,7877	
	5% Trimmed Mean		85,8333	
	Median		86,0000	
	Variance		15,418	

	Std. Deviation		3,92663	
	Minimum		81,00	
	Maximum		93,00	
	Range		12,00	
	Interquartile Range		6,25	
	Skewness		,459	,512
	Kurtosis		-,775	,992
6,00	Mean		85,6000	,89560
	95% Confidence Interval for Mean	Lower Bound	83,7255	
		Upper Bound	87,4745	
	5% Trimmed Mean		85,6667	
	Median		86,0000	
	Variance		16,042	
	Std. Deviation		4,00526	
	Minimum		79,00	
	Maximum		91,00	
	Range		12,00	
	Interquartile Range		6,75	
	Skewness		-,285	,512
	Kurtosis		-1,149	,992
7,00	Mean		86,1000	,92310
	95% Confidence Interval for Mean	Lower Bound	84,1679	
		Upper Bound	88,0321	
	5% Trimmed Mean		86,3333	
	Median		86,5000	
	Variance		17,042	
	Std. Deviation		4,12821	
	Minimum		77,00	
	Maximum		91,00	
	Range		14,00	
	Interquartile Range		7,25	
	Skewness		-,533	,512
	Kurtosis		-,340	,992
8,00	Mean		85,2500	1,12828
	95% Confidence Interval for Mean	Lower Bound	82,8885	
		Upper Bound	87,6115	
	5% Trimmed Mean		85,2778	
	Median		85,5000	
	Variance		25,461	
	Std. Deviation		5,04584	
	Minimum		76,00	

		Maximum		94,00	
		Range		18,00	
		Interquartile Range		9,50	
		Skewness		-,133	,512
		Kurtosis		-1,019	,992
	9,00	Mean		85,2500	1,09033
		95% Confidence Interval for Mean	Lower Bound	82,9679	
			Upper Bound	87,5321	
		5% Trimmed Mean		85,2778	
		Median		86,0000	
		Variance		23,776	
		Std. Deviation		4,87610	
		Minimum		76,00	
		Maximum		94,00	
		Range		18,00	
		Interquartile Range		7,00	
		Skewness		-,169	,512
		Kurtosis		-,672	,992
	10,00	Mean		84,0500	1,21498
		95% Confidence Interval for Mean	Lower Bound	81,5070	
			Upper Bound	86,5930	
		5% Trimmed Mean		84,2222	
		Median		84,5000	
		Variance		29,524	
		Std. Deviation		5,43357	
		Minimum		74,00	
		Maximum		91,00	
		Range		17,00	
		Interquartile Range		9,75	
		Skewness		-,295	,512
		Kurtosis		-1,072	,992
LIN	1,00	Mean		57,7500	1,16048
		95% Confidence Interval for Mean	Lower Bound	55,3211	
			Upper Bound	60,1789	
		5% Trimmed Mean		57,7778	
		Median		58,0000	
		Variance		26,934	
		Std. Deviation		5,18982	
		Minimum		48,00	
		Maximum		67,00	

	Range		19,00	
	Interquartile Range		9,00	
	Skewness		-,042	,512
	Kurtosis		-,609	,992
2,00	Mean		56,7000	1,08604
	95% Confidence Interval for Mean	Lower Bound	54,4269	
		Upper Bound	58,9731	
	5% Trimmed Mean		56,6667	
	Median		57,5000	
	Variance		23,589	
	Std. Deviation		4,85690	
	Minimum		48,00	
	Maximum		66,00	
	Range		18,00	
	Interquartile Range		8,25	
	Skewness		-,088	,512
	Kurtosis		-,821	,992
3,00	Mean		58,0500	1,46983
	95% Confidence Interval for Mean	Lower Bound	54,9736	
		Upper Bound	61,1264	
	5% Trimmed Mean		57,9444	
	Median		58,0000	
	Variance		43,208	
	Std. Deviation		6,57327	
	Minimum		47,00	
	Maximum		71,00	
	Range		24,00	
	Interquartile Range		9,75	
	Skewness		,200	,512
	Kurtosis		-,712	,992
4,00	Mean		57,1500	1,59155
	95% Confidence Interval for Mean	Lower Bound	53,8189	
		Upper Bound	60,4811	
	5% Trimmed Mean		56,8333	
	Median		56,5000	
	Variance		50,661	
	Std. Deviation		7,11762	
	Minimum		48,00	

			Statistic	Std. Error
	Maximum		72,00	
	Range		24,00	
	Interquartile Range		12,00	
	Skewness		,411	,512
	Kurtosis		-,722	,992
5,00	Mean		59,2500	1,62363
	95% Confidence Interval for Mean	Lower Bound	55,8517	
		Upper Bound	62,6483	
	5% Trimmed Mean		59,0556	
	Median		58,0000	
	Variance		52,724	
	Std. Deviation		7,26111	
	Minimum		49,00	
	Maximum		73,00	
	Range		24,00	
	Interquartile Range		13,00	
	Skewness		,502	,512
	Kurtosis		-,908	,992
6,00	Mean		56,8000	1,23672
	95% Confidence Interval for Mean	Lower Bound	54,2115	
		Upper Bound	59,3885	
	5% Trimmed Mean		56,5000	
	Median		58,0000	
	Variance		30,589	
	Std. Deviation		5,53078	
	Minimum		49,00	
	Maximum		70,00	
	Range		21,00	
	Interquartile Range		9,50	
	Skewness		,418	,512
	Kurtosis		,021	,992
7,00	Mean		58,0000	1,37075
	95% Confidence Interval for Mean	Lower Bound	55,1310	
		Upper Bound	60,8690	
	5% Trimmed Mean		58,1667	
	Median		58,5000	
	Variance		37,579	
	Std. Deviation		6,13017	

	Minimum		46,00	
	Maximum		67,00	
	Range		21,00	
	Interquartile Range		9,75	
	Skewness		-,329	,512
	Kurtosis		-,983	,992
8,00	Mean		57,4000	1,67552
	95% Confidence Interval for Mean	Lower Bound	53,8931	
		Upper Bound	60,9069	
	5% Trimmed Mean		57,2222	
	Median		58,0000	
	Variance		56,147	
	Std. Deviation		7,49315	
	Minimum		46,00	
	Maximum		72,00	
	Range		26,00	
	Interquartile Range		12,75	
	Skewness		,078	,512
	Kurtosis		-,955	,992
9,00	Mean		57,3000	1,55952
	95% Confidence Interval for Mean	Lower Bound	54,0359	
		Upper Bound	60,5641	
	5% Trimmed Mean		57,3333	
	Median		59,0000	
	Variance		48,642	
	Std. Deviation		6,97439	
	Minimum		46,00	
	Maximum		68,00	
	Range		22,00	
	Interquartile Range		11,75	
	Skewness		-,206	,512
	Kurtosis		-1,235	,992
10,00	Mean		56,2000	1,53314
	95% Confidence Interval for Mean	Lower Bound	52,9911	
		Upper Bound	59,4089	
	5% Trimmed Mean		56,1111	
	Median		57,0000	
	Variance		47,011	

	Std. Deviation	6,85642	
	Minimum	46,00	
	Maximum	68,00	
	Range	22,00	
	Interquartile Range	13,00	
	Skewness	-,001	,512
	Kurtosis	-1,294	,992

Tests of Normality

	Gruplar	Kolmogorov-Smirnov[a]			Shapiro-Wilk		
		Statistic	df	Sig.	Statistic	df	Sig.
VAP	1,00	,189	20	,059	,878	20	,016
	2,00	,176	20	,106	,924	20	,120
	3,00	,192	20	,052	,935	20	,193
	4,00	,158	20	,200*	,962	20	,592
	5,00	,113	20	,200*	,984	20	,972
	6,00	,179	20	,092	,899	20	,040
	7,00	,123	20	,200*	,967	20	,682
	8,00	,102	20	,200*	,968	20	,717
	9,00	,145	20	,200*	,934	20	,186
	10,00	,110	20	,200*	,918	20	,090
VSL	1,00	,161	20	,188	,878	20	,017
	2,00	,163	20	,174	,939	20	,229
	3,00	,125	20	,200*	,950	20	,366
	4,00	,127	20	,200*	,955	20	,457
	5,00	,093	20	,200*	,978	20	,913
	6,00	,170	20	,134	,897	20	,037
	7,00	,160	20	,194	,943	20	,267
	8,00	,184	20	,073	,940	20	,235
	9,00	,142	20	,200*	,964	20	,636
	10,00	,107	20	,200*	,941	20	,248
VCL	1,00	,172	20	,122	,940	20	,236
	2,00	,202	20	,032	,889	20	,026
	3,00	,141	20	,200*	,963	20	,603
	4,00	,187	20	,066	,919	20	,096
	5,00	,130	20	,200*	,959	20	,518
	6,00	,217	20	,015	,875	20	,014
	7,00	,108	20	,200*	,969	20	,739

	8,00	,107	20	,200*	,965	20	,640
	9,00	,088	20	,200*	,981	20	,952
	10,00	,122	20	,200*	,941	20	,255
ALH	1,00	,117	20	,200*	,955	20	,441
	2,00	,156	20	,200*	,886	20	,023
	3,00	,094	20	,200*	,952	20	,406
	4,00	,183	20	,078	,926	20	,128
	5,00	,134	20	,200*	,951	20	,385
	6,00	,161	20	,186	,934	20	,183
	7,00	,165	20	,155	,923	20	,114
	8,00	,195	20	,045	,913	20	,073
	9,00	,190	20	,057	,864	20	,009
	10,00	,188	20	,062	,903	20	,048
BCF	1,00	,109	20	,200*	,972	20	,798
	2,00	,133	20	,200*	,972	20	,803
	3,00	,162	20	,182	,932	20	,167
	4,00	,100	20	,200*	,979	20	,922
	5,00	,225	20	,009	,889	20	,025
	6,00	,098	20	,200*	,983	20	,969
	7,00	,131	20	,200*	,925	20	,121
	8,00	,122	20	,200*	,957	20	,482
	9,00	,112	20	,200*	,969	20	,731
	10,00	,112	20	,200*	,968	20	,708
STR	1,00	,113	20	,200*	,985	20	,983
	2,00	,144	20	,200*	,970	20	,755
	3,00	,123	20	,200*	,936	20	,199
	4,00	,179	20	,095	,933	20	,173
	5,00	,145	20	,200*	,916	20	,085
	6,00	,140	20	,200*	,930	20	,155
	7,00	,132	20	,200*	,925	20	,125
	8,00	,127	20	,200*	,958	20	,511
	9,00	,161	20	,185	,971	20	,784
	10,00	,119	20	,200*	,934	20	,187
LIN	1,00	,120	20	,200*	,976	20	,872
	2,00	,132	20	,200*	,965	20	,638
	3,00	,081	20	,200*	,980	20	,929
	4,00	,142	20	,200*	,929	20	,146
	5,00	,124	20	,200*	,933	20	,175
	6,00	,136	20	,200*	,938	20	,224
	7,00	,188	20	,063	,933	20	,173

8,00	,153	20	,200*	,957	20	,491
9,00	,152	20	,200*	,938	20	,219
10,00	,153	20	,200*	,939	20	,225

*. This is a lower bound of the true significance.

a. Lilliefors Significance Correction

Gruplar = 1,00

Histograms

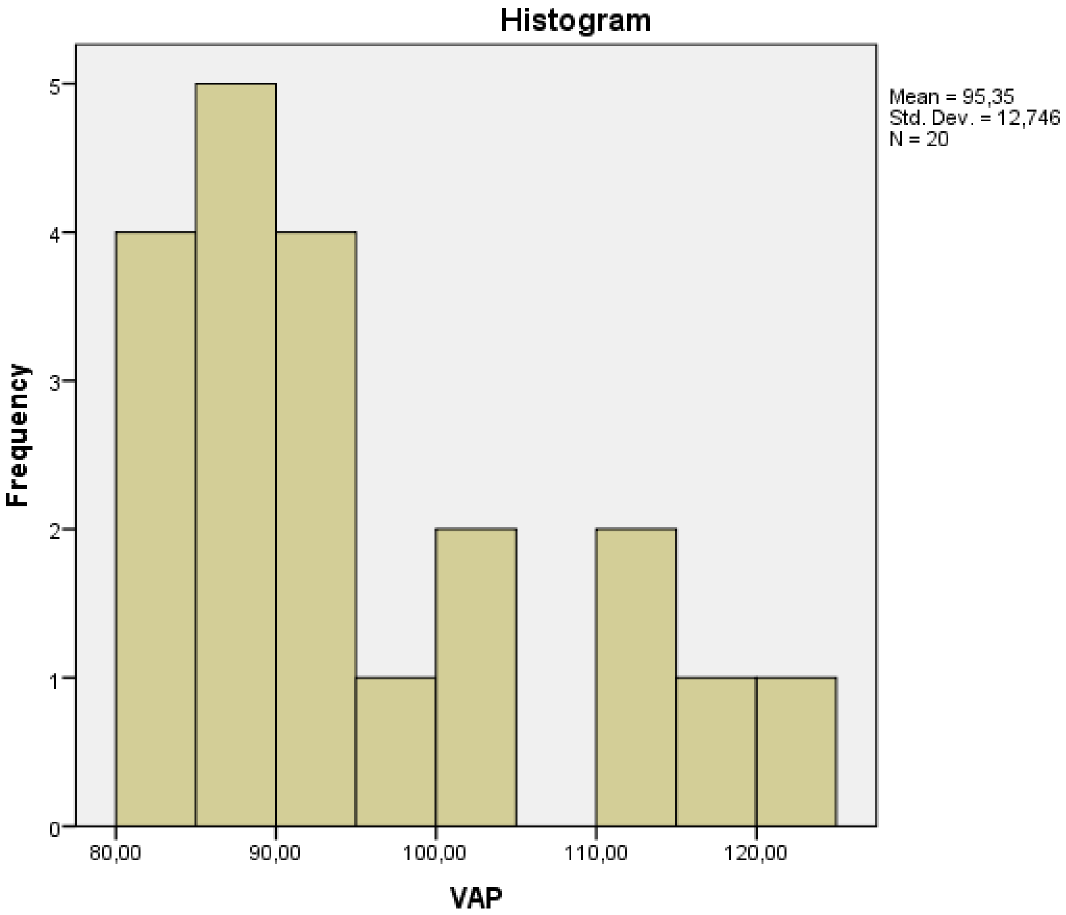

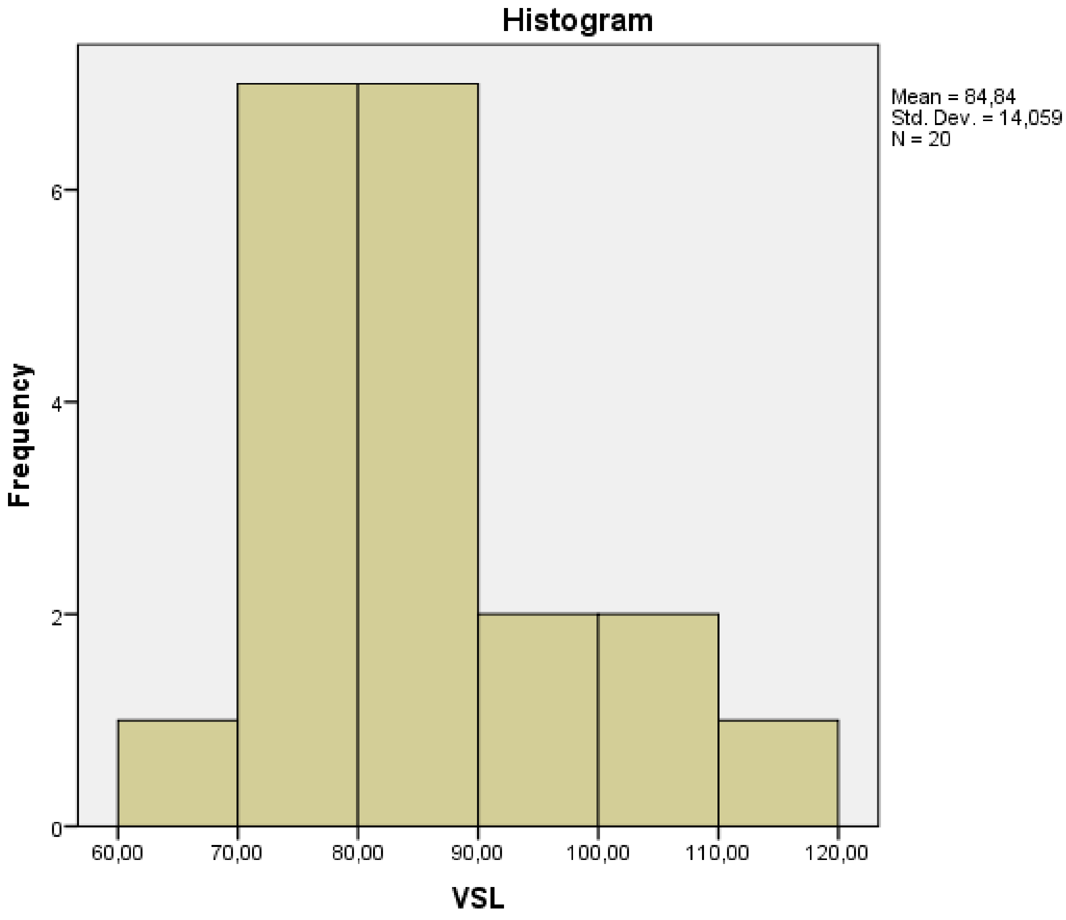
Histogram
Mean = 84,84
Std. Dev. = 14,059
N = 20
Frequency
0
2
4
6
60,00
70,00
80,00
90,00
100,00
110,00
120,00
VSL

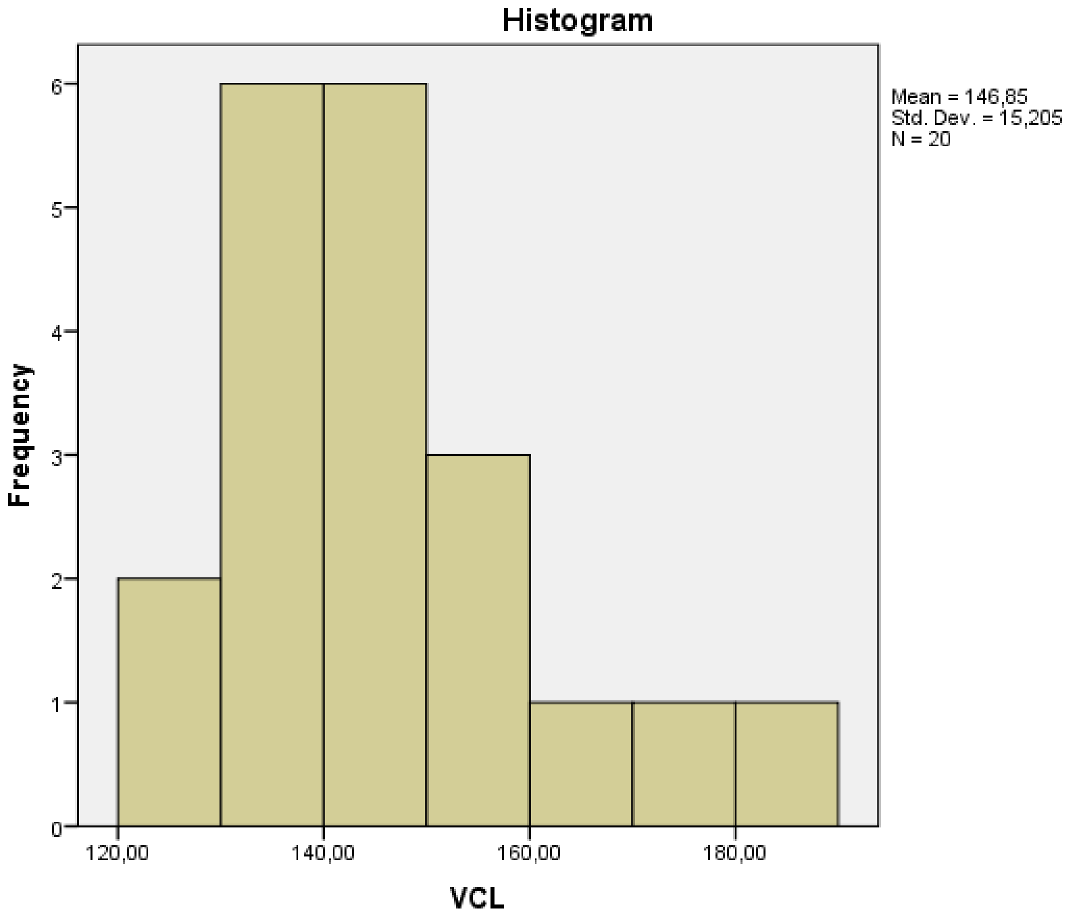
Histogram
Mean = 146,85
Std. Dev. = 15,205
N = 20
Frequency
0
1
2
3
4
5
6
120,00
140,00
160,00
180,00
VCL

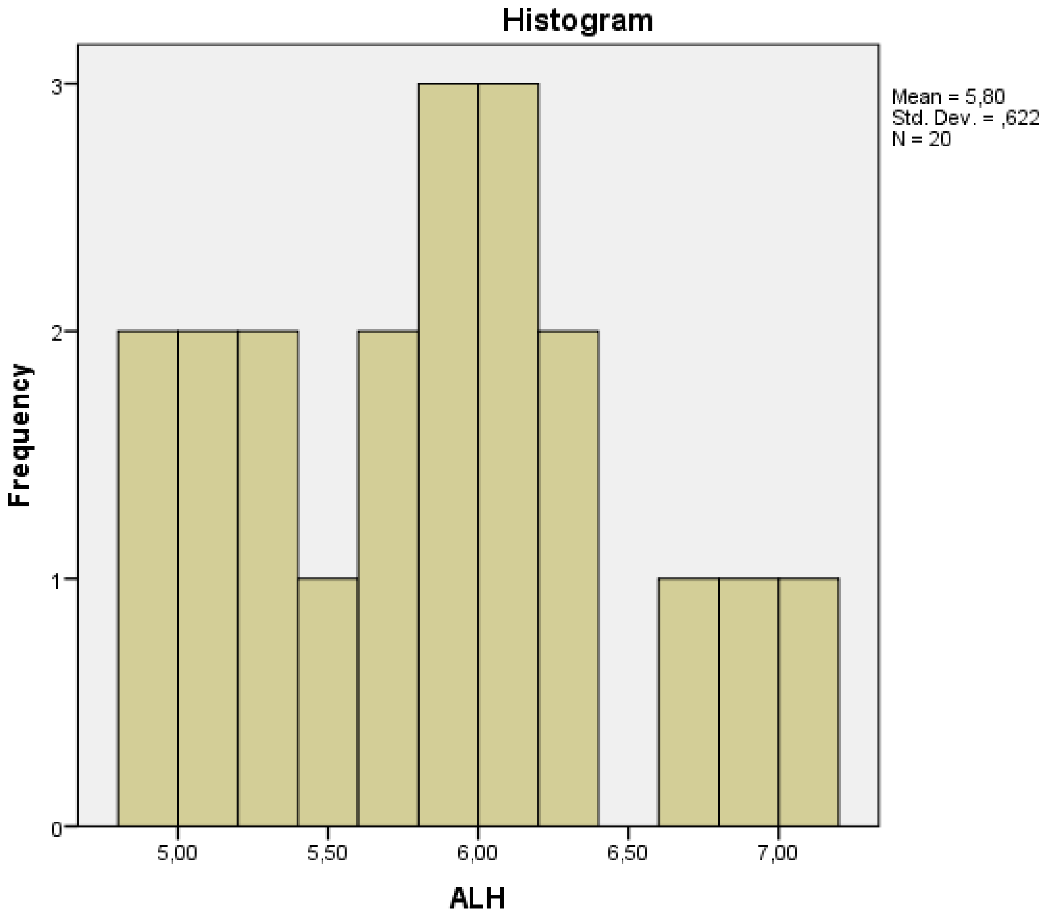
Histogram
Mean = 5,80
Std. Dev. = ,622
N = 20
Frequency
0
1
2
3
5,00
5,50
6,00
6,50
7,00
ALH

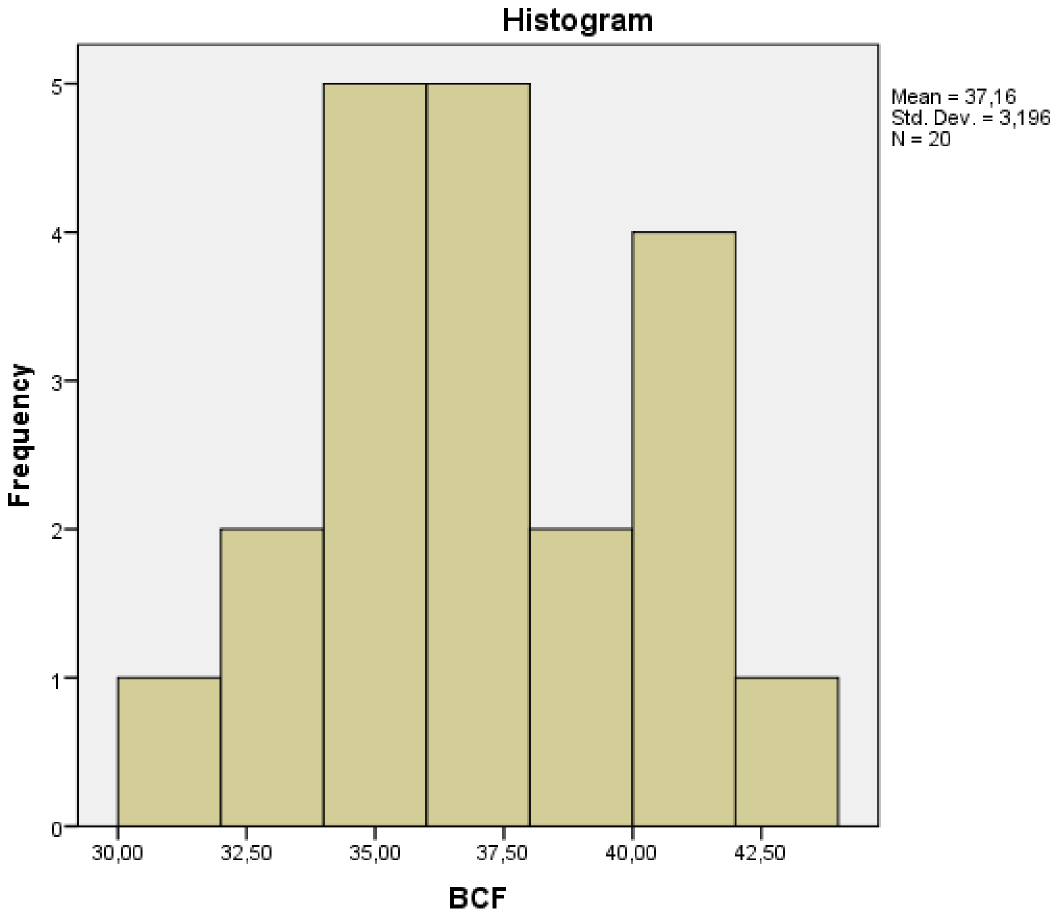

Histogram
Mean = 37,16
Std. Dev. = 3,196
N = 20
Frequency
0
1
2
3
4
5
30,00
32,50
35,00
37,50
40,00
42,50
BCF

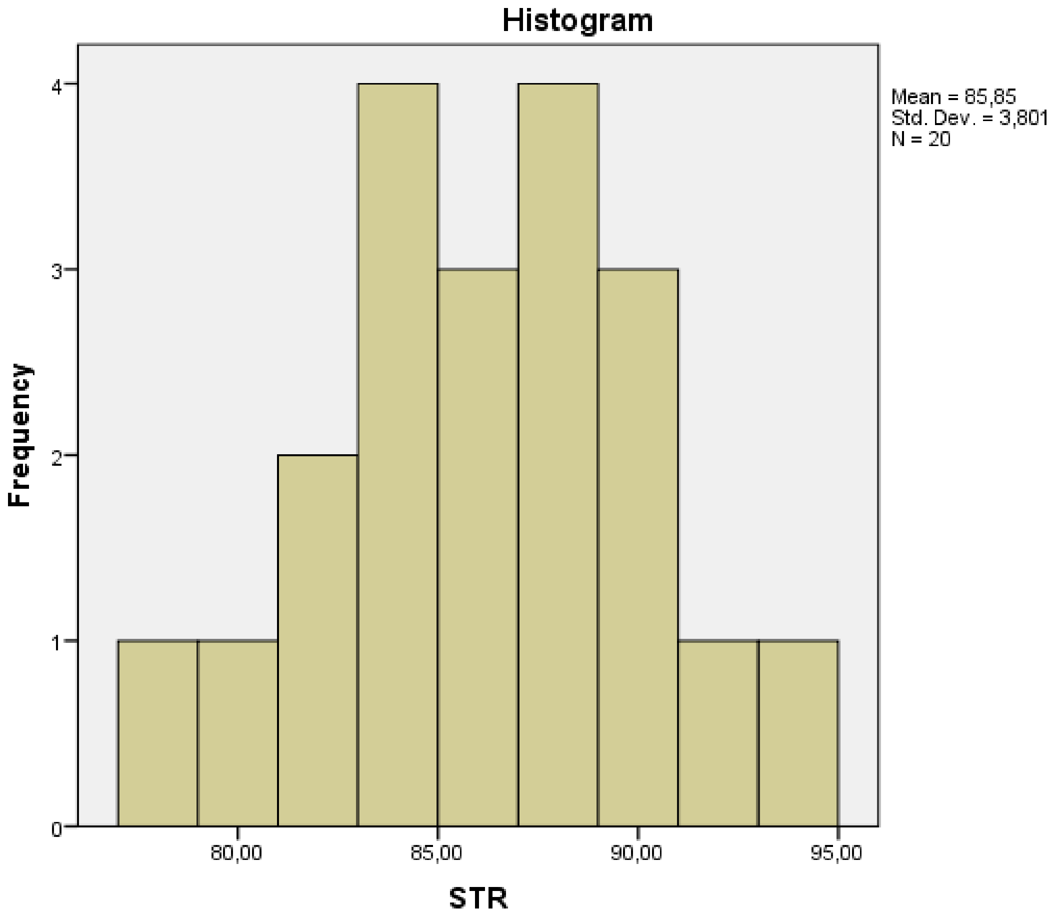
Histogram
Mean = 85,85
Std. Dev. = 3,801
N = 20
Frequency
0
1
2
3
4
80,00
85,00
90,00
95,00
STR

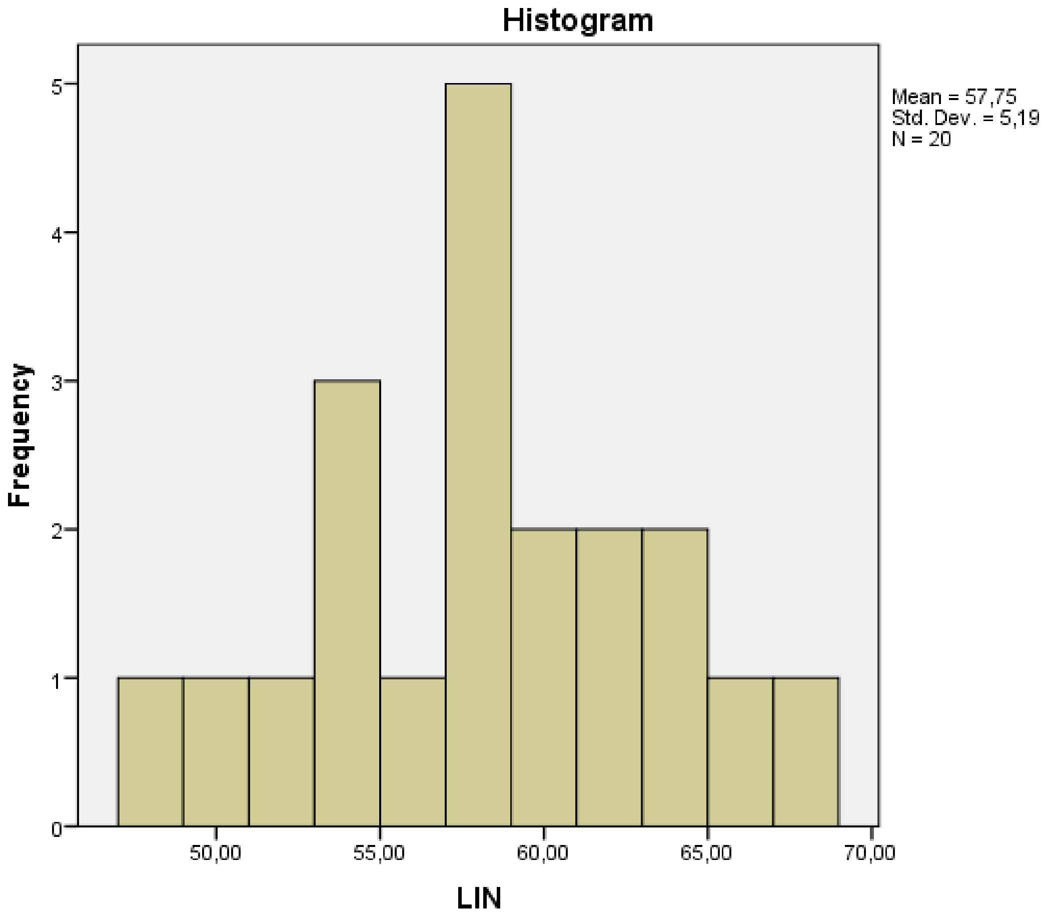

Stem-and-Leaf Plots

```
VAP Stem-and-Leaf Plot for
Gruplar= 1,00

 Frequency    Stem &  Leaf

     4,00        8 .  0234
     5,00        8 .  57788
     4,00        9 .  1134
     1,00        9 .  5
     2,00       10 .  03
      ,00       10 .
     2,00       11 .  04
     1,00       11 .  8
     1,00 Extremes    (>=125)

 Stem width:  10,00
 Each leaf:       1 case(s)
```

VSL Stem-and-Leaf Plot for
Gruplar= 1,00

Frequency	Stem &	Leaf
1,00	6 .	9
7,00	7 .	1122345
7,00	8 .	0112578
2,00	9 .	59
2,00	10 .	69
1,00	11 .	7

Stem width: 10,00
Each leaf: 1 case(s)

VCL Stem-and-Leaf Plot for
Gruplar= 1,00

Frequency	Stem &	Leaf
2,00	12 .	56
6,00	13 .	155899
6,00	14 .	045777
3,00	15 .	468
1,00	16 .	9
1,00	17 .	1
1,00	18 .	3

Stem width: 10,00
Each leaf: 1 case(s)

ALH Stem-and-Leaf Plot for
Gruplar= 1,00

Frequency	Stem &	Leaf
2,00	4 .	99
5,00	5 .	11234
5,00	5 .	66999
5,00	6 .	00123
2,00	6 .	69
1,00	7 .	0

Stem width: 1,00
Each leaf: 1 case(s)

```
BCF Stem-and-Leaf Plot for
Gruplar= 1,00

 Frequency    Stem &  Leaf

     5,00        3 .  12344
    10,00        3 .  5556677799
     5,00        4 .  00113

 Stem width:  10,00
 Each leaf:       1 case(s)
```

```
STR Stem-and-Leaf Plot for
Gruplar= 1,00

 Frequency    Stem &  Leaf

     1,00        7 .  8
     7,00        8 .  0224444
     8,00        8 .  55677889
     4,00        9 .  0013

 Stem width:  10,00
 Each leaf:       1 case(s)
```

```
LIN Stem-and-Leaf Plot for
Gruplar= 1,00

 Frequency    Stem &  Leaf

     1,00        4 .  8
     5,00        5 .  02333
     7,00        5 .  6778889
     5,00        6 .  02233
     2,00        6 .  67

 Stem width:  10,00
 Each leaf:       1 case(s)
```

Normal Q-Q Plots

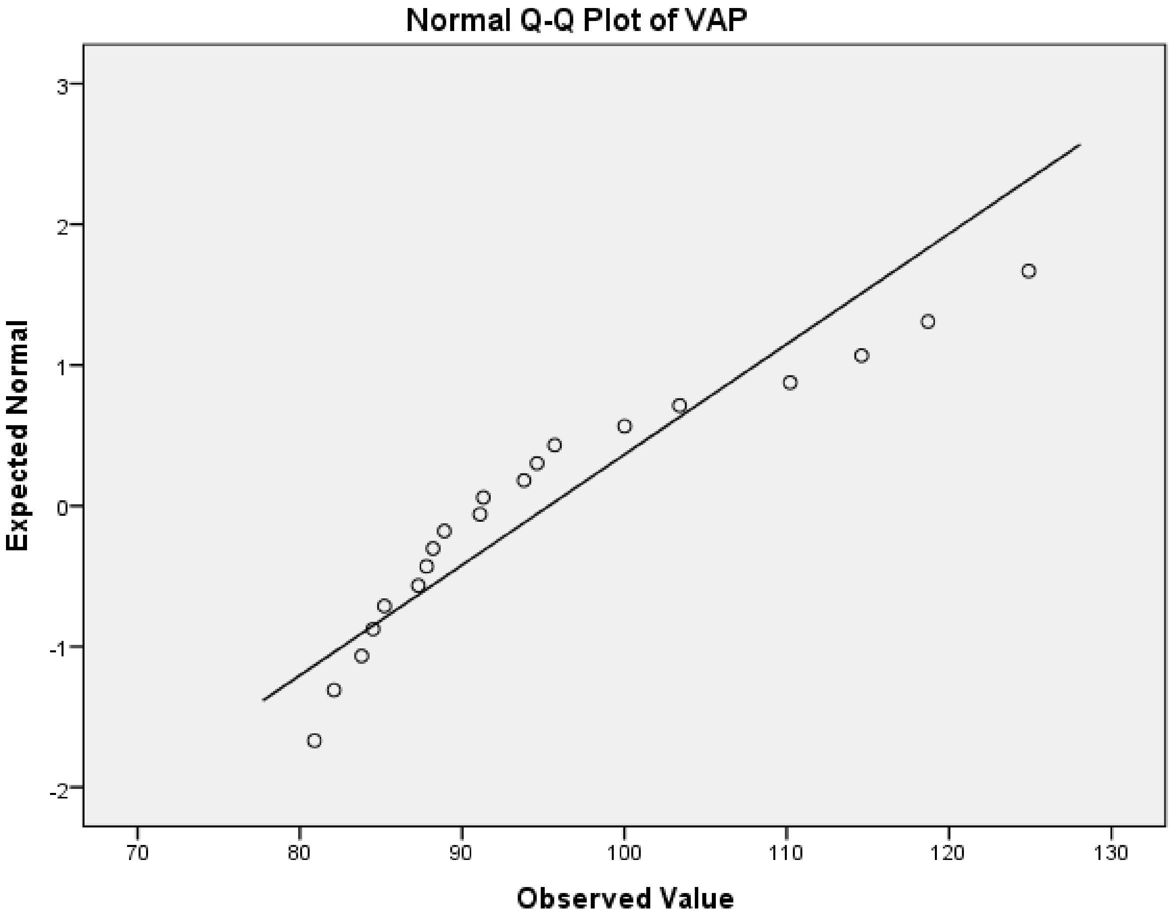
Normal Q-Q Plot of VAP
Expected Normal
Observed Value
3
2
1
0
-1
-2
70
80
90
100
110
120
130

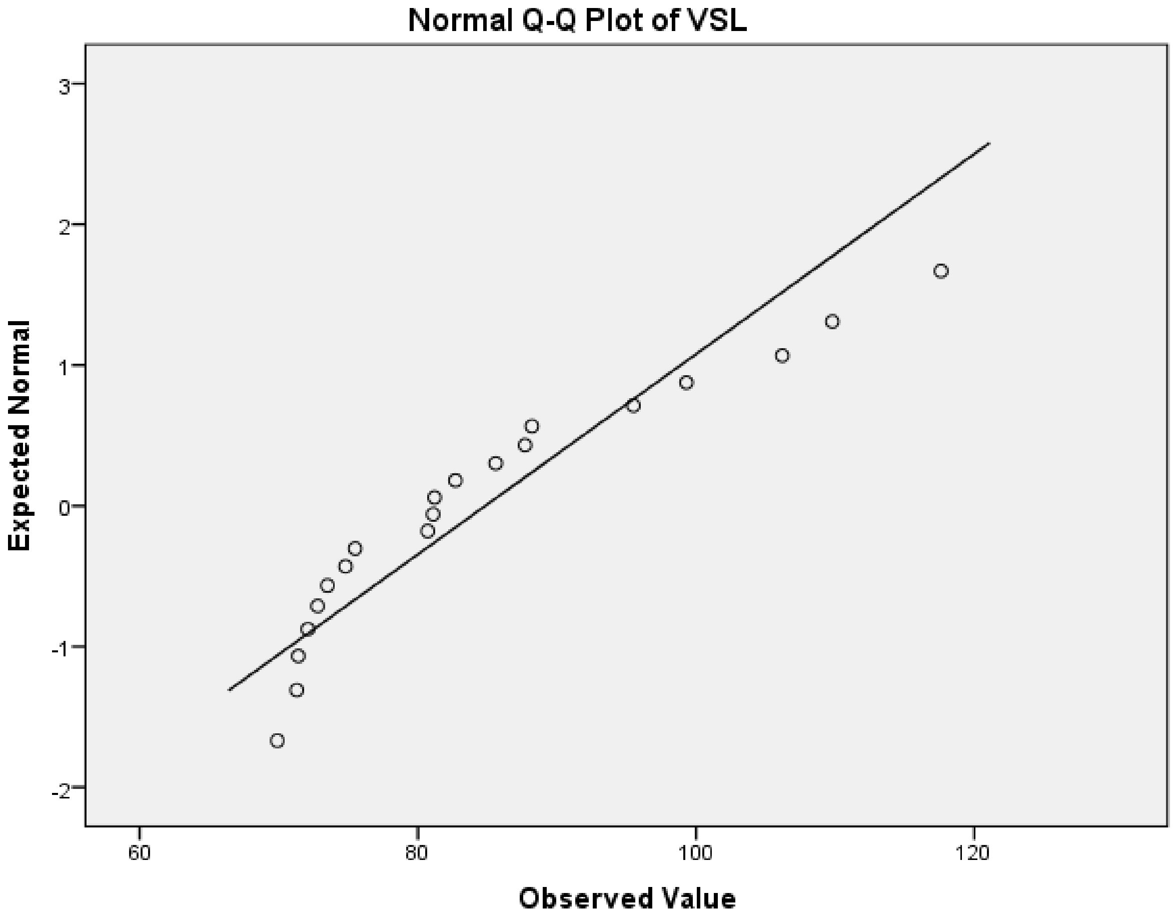
Normal Q-Q Plot of VSL
Expected Normal
3
2
1
0
-1
-2
60
80
100
120
Observed Value

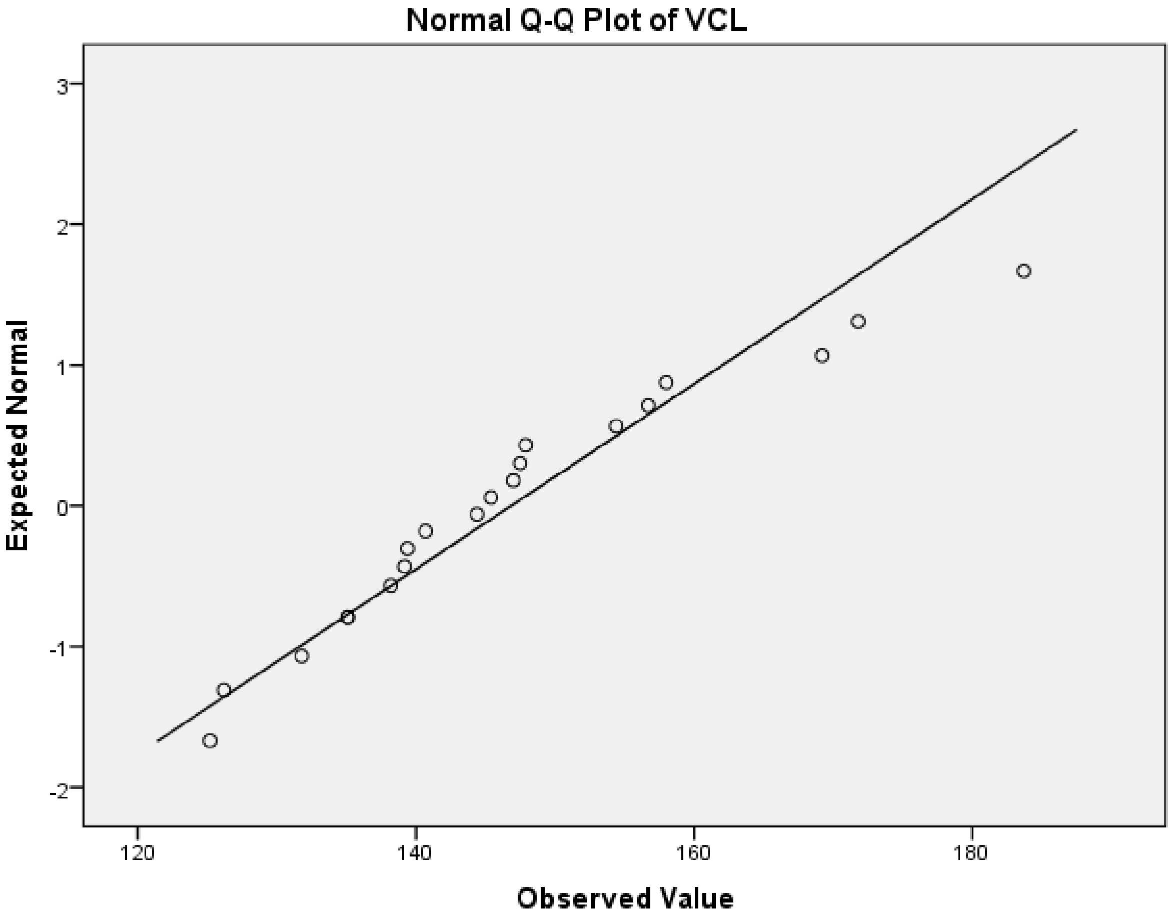
Normal Q-Q Plot of VCL
Expected Normal
Observed Value
3
2
1
0
-1
-2
120
140
160
180

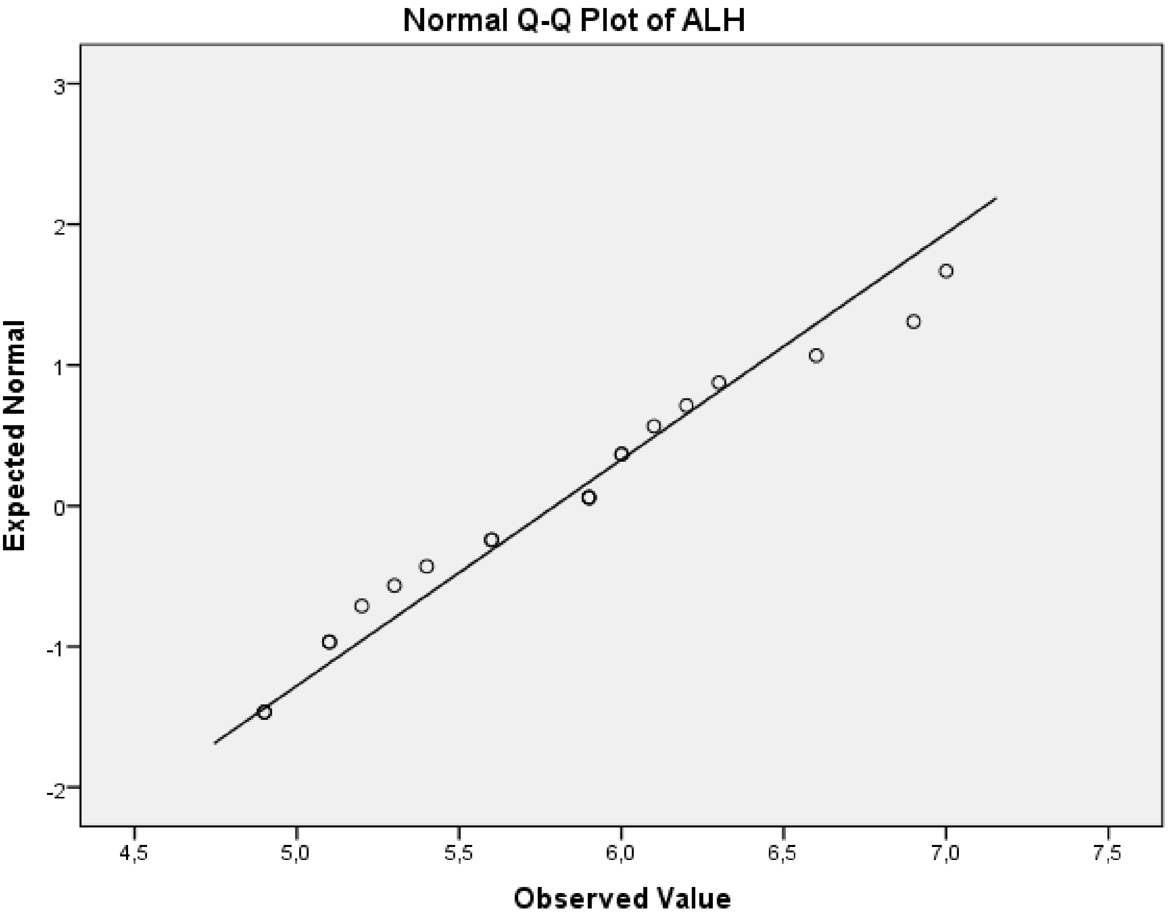

Normal Q-Q Plot of ALH
Expected Normal
Observed Value
3
2
1
0
-1
-2
4,5
5,0
5,5
6,0
6,5
7,0
7,5

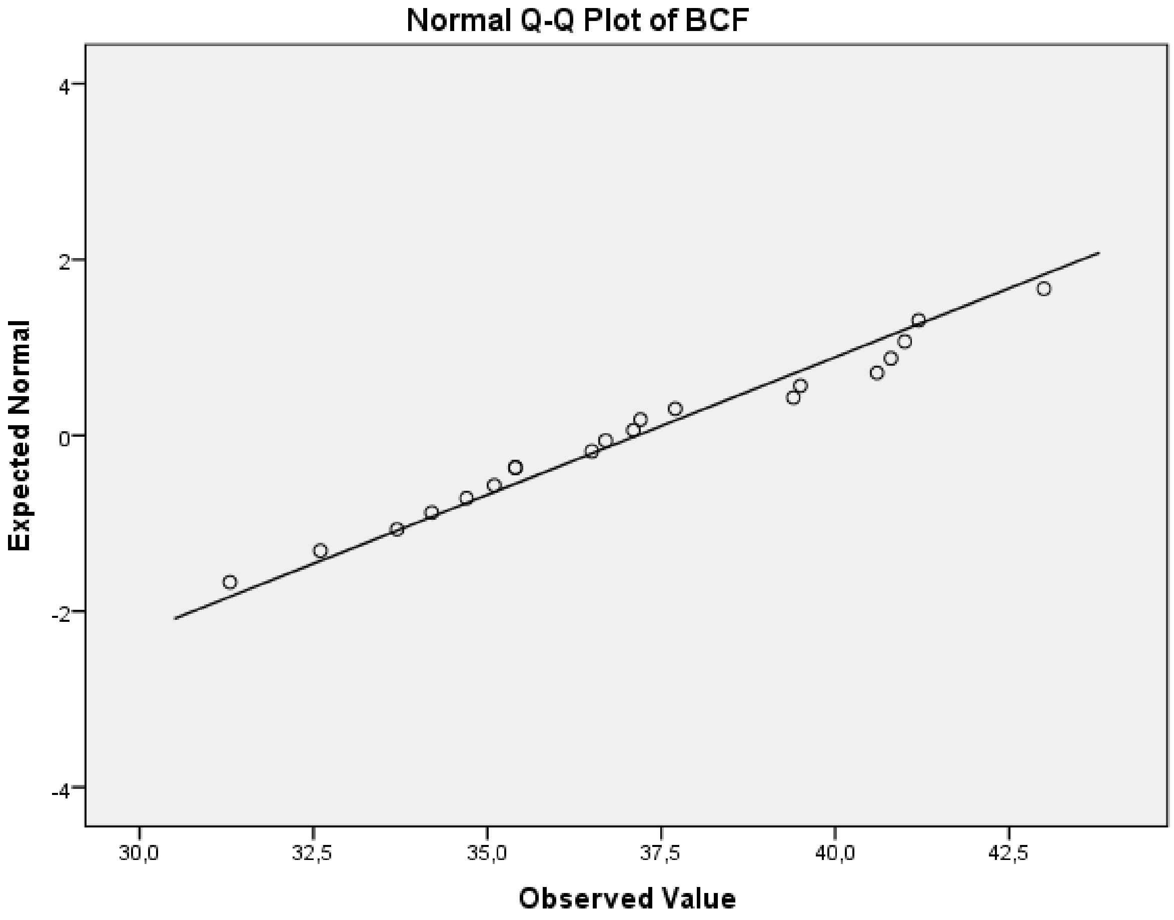

Normal Q-Q Plot of BCF
Expected Normal
Observed Value
4
2
0
-2
-4
30,0
32,5
35,0
37,5
40,0
42,5

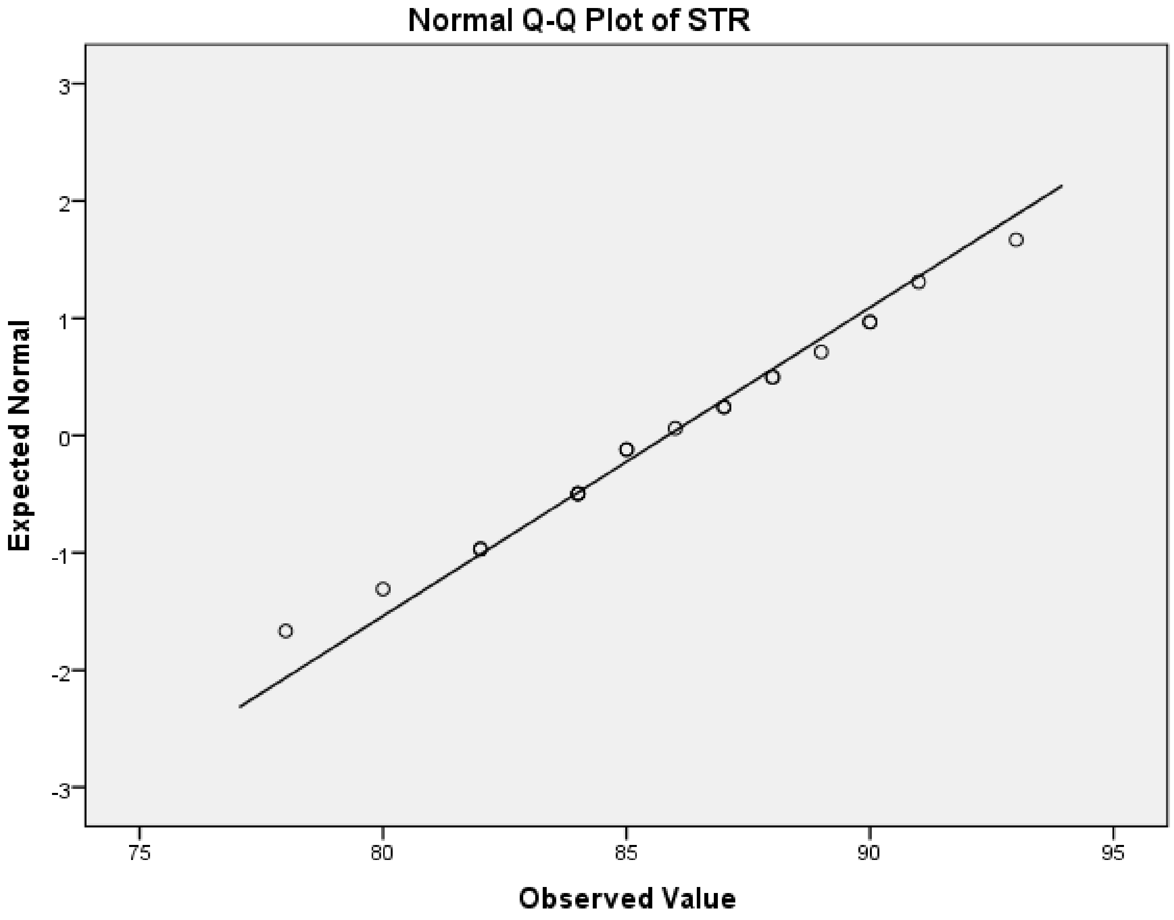
Normal Q-Q Plot of STR
Expected Normal
Observed Value
3
2
1
0
-1
-2
-3
75
80
85
90
95

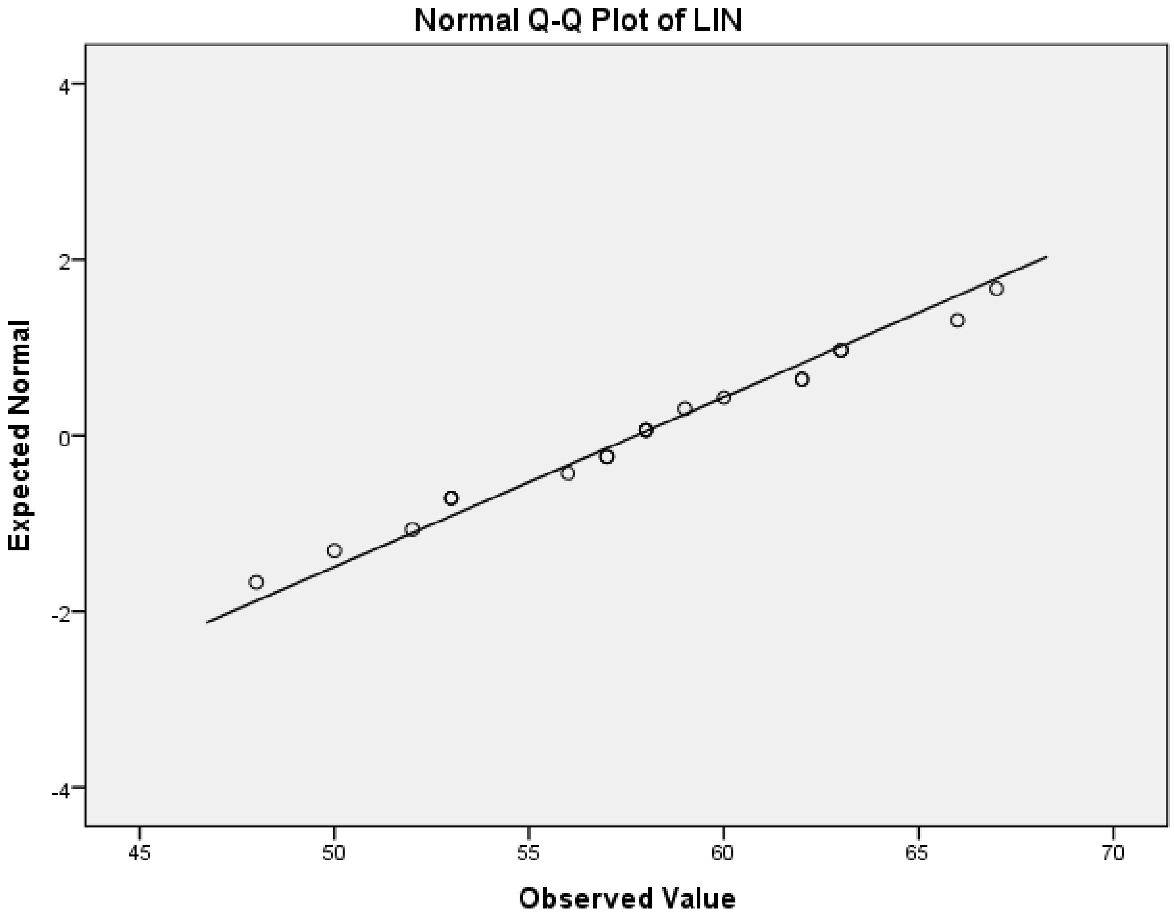

Detrended Normal Q-Q Plots

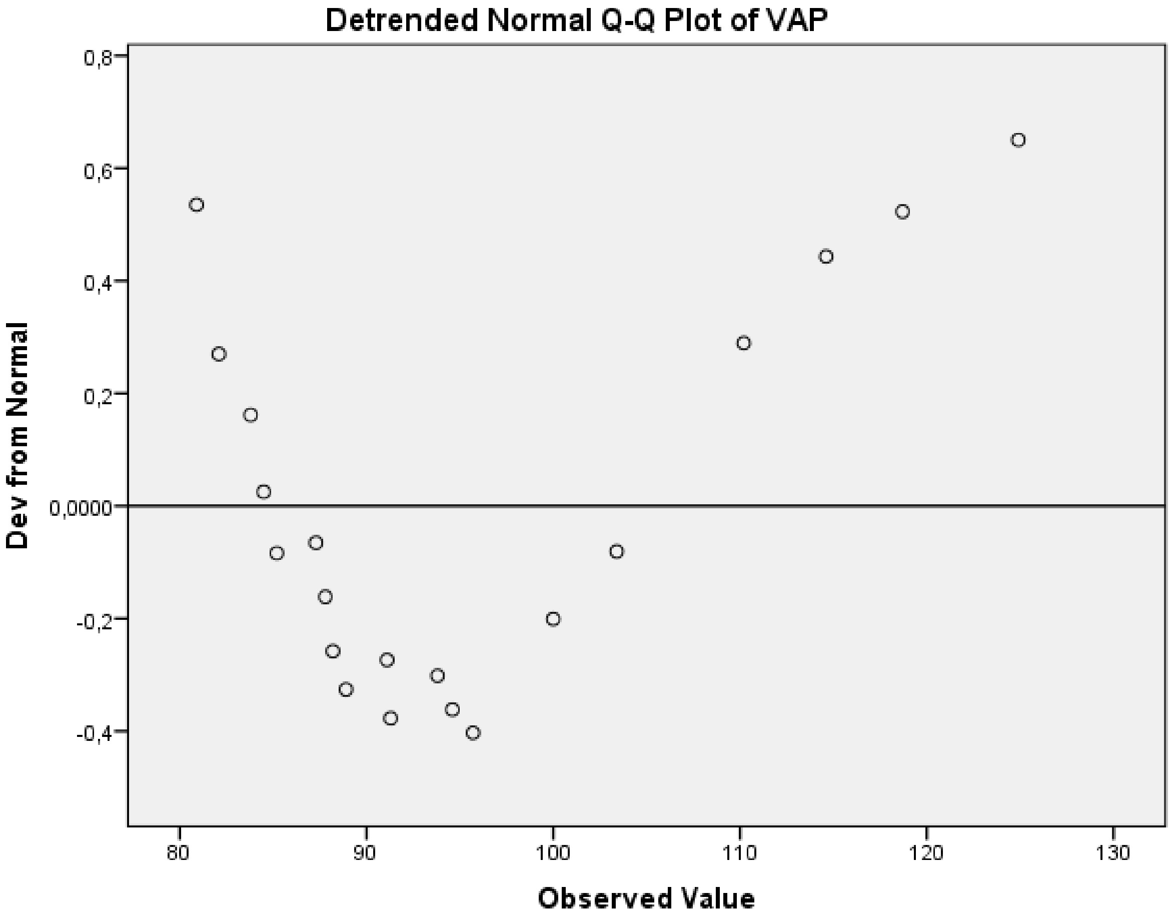
Detrended Normal Q-Q Plot of VAP
Dev from Normal
0,8
0,6
0,4
0,2
0,0000
-0,2
-0,4
80
90
100
110
120
130
Observed Value

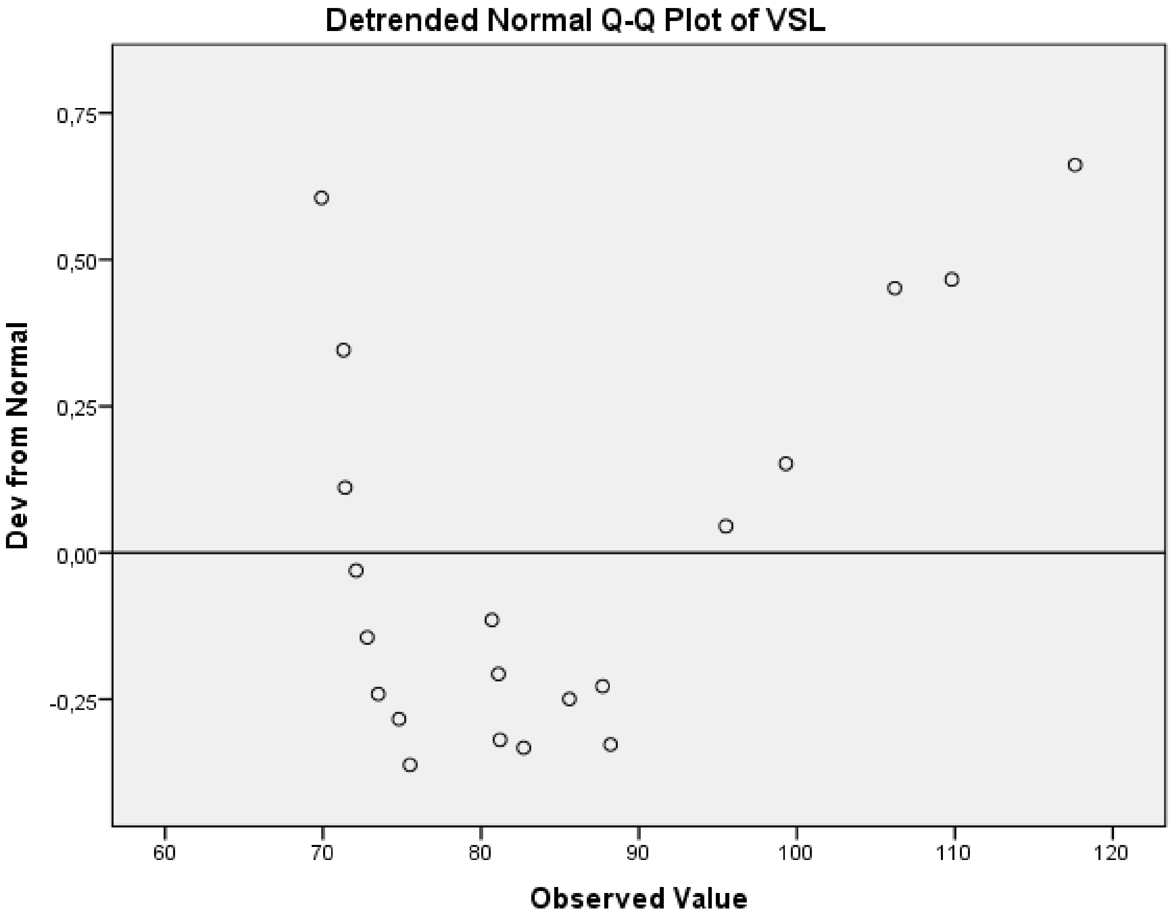

Detrended Normal Q-Q Plot of VSL
Dev from Normal
0,75
0,50
0,25
0,00
-0,25
60
70
80
90
100
110
120
Observed Value

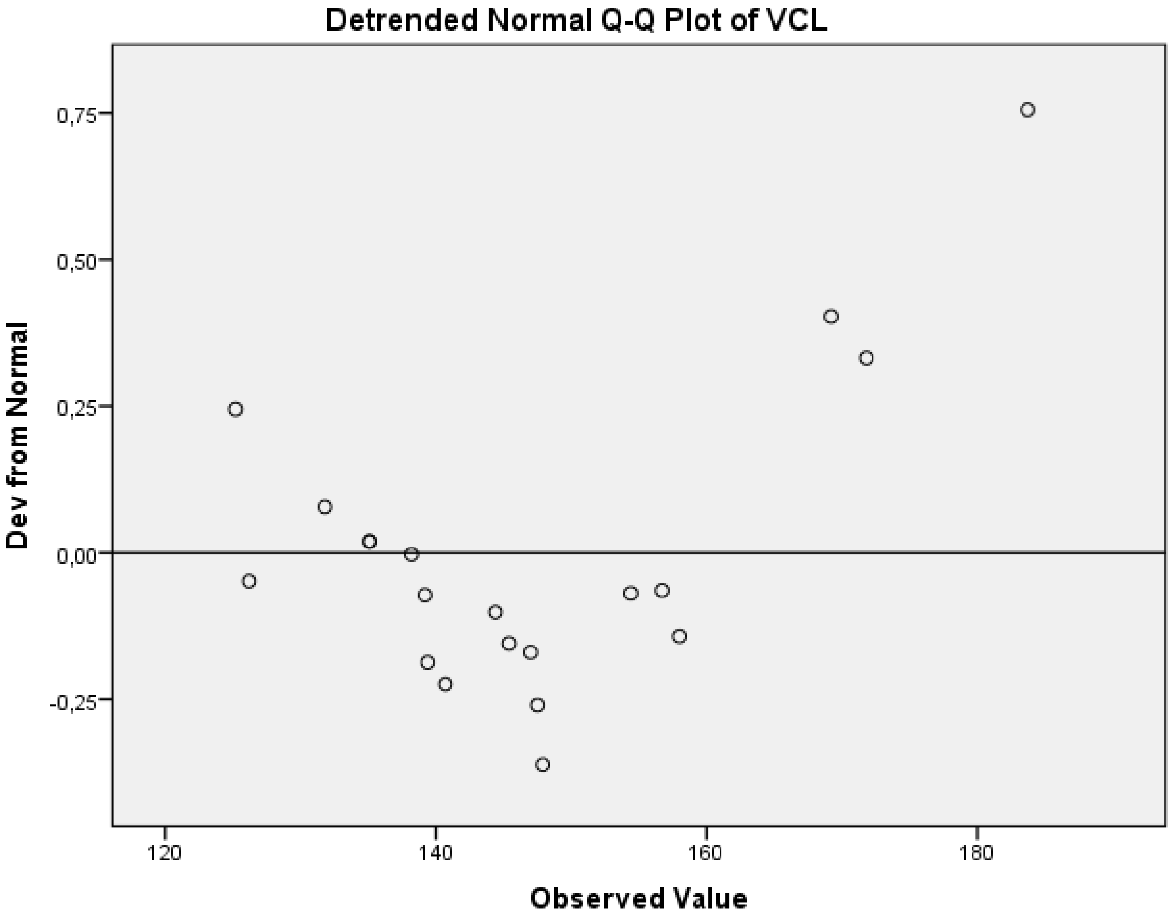
Detrended Normal Q-Q Plot of VCL
Dev from Normal
0,75
0,50
0,25
0,00
-0,25
120
140
160
180
Observed Value

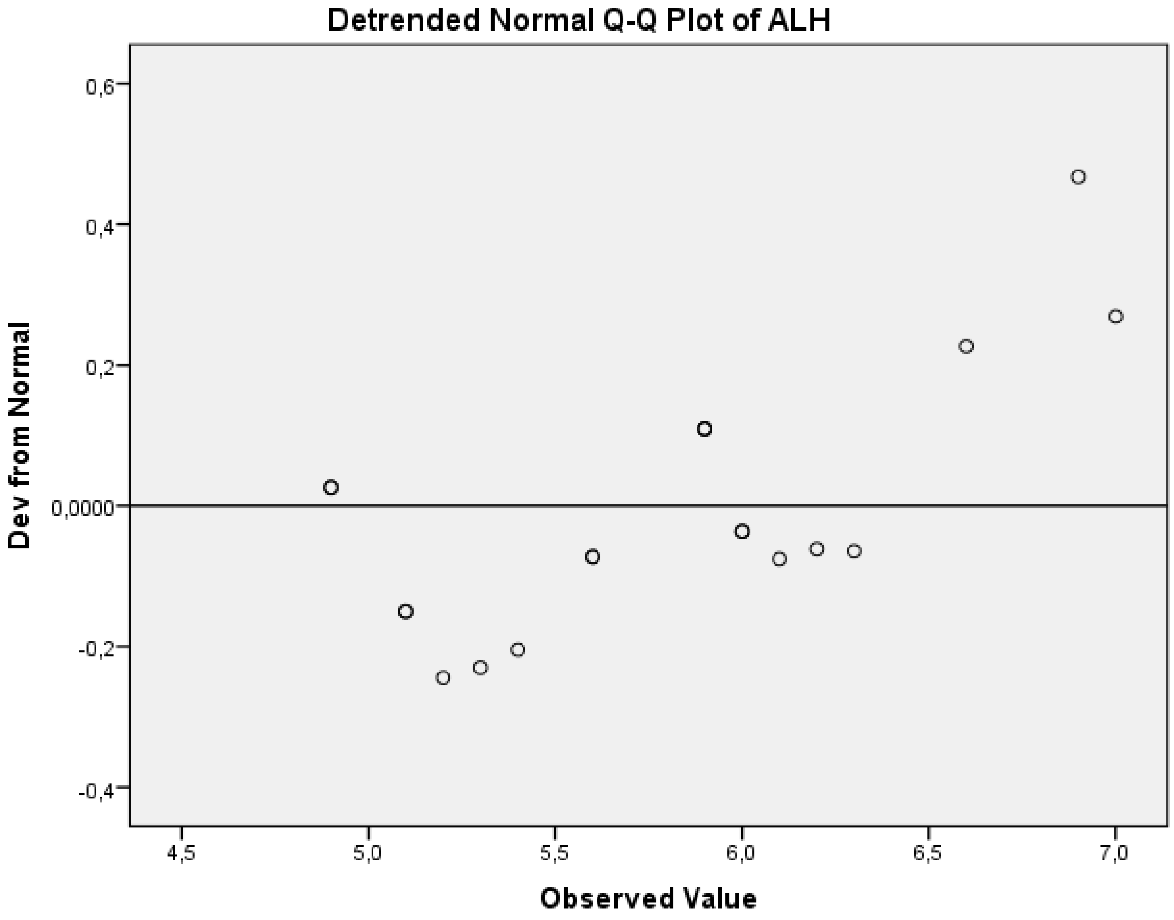
Detrended Normal Q-Q Plot of ALH
Dev from Normal
Observed Value
0,6
0,4
0,2
0,0000
-0,2
-0,4
4,5
5,0
5,5
6,0
6,5
7,0

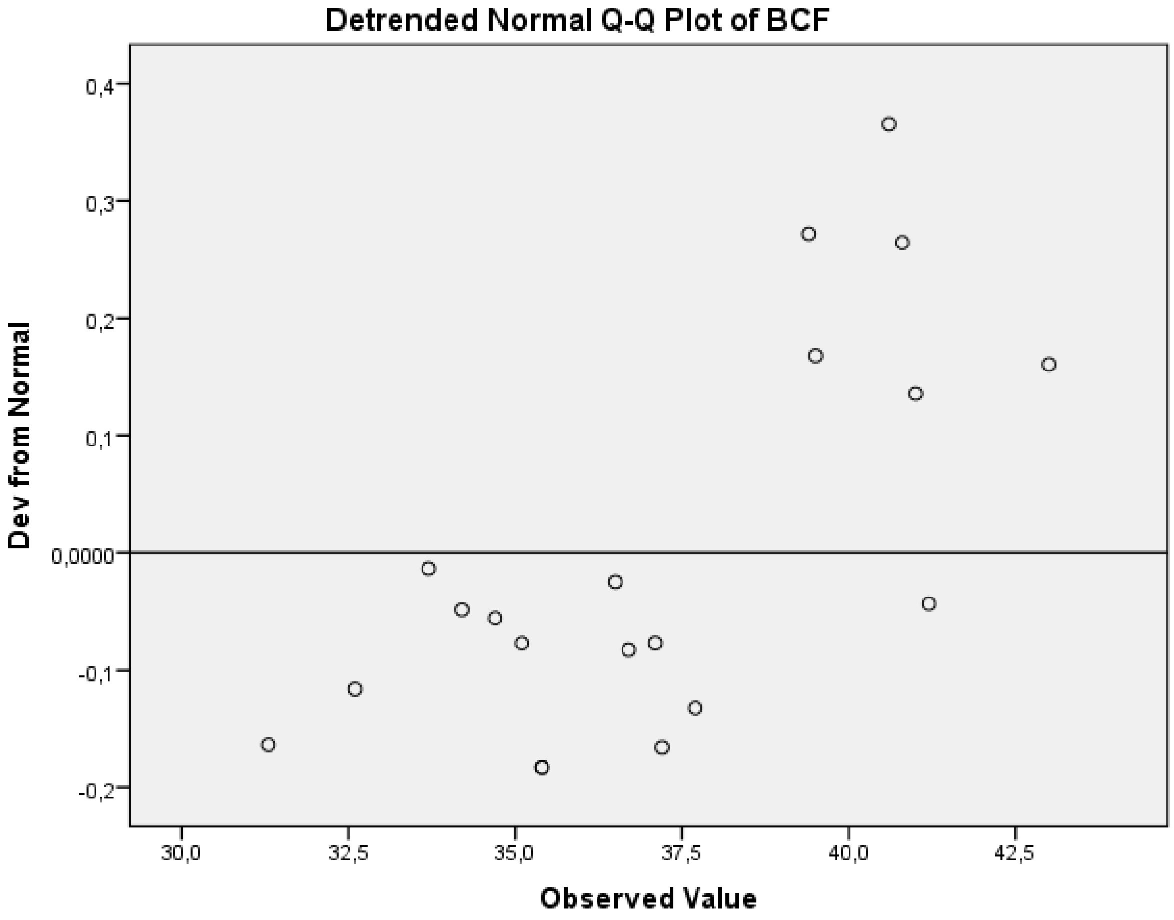

Detrended Normal Q-Q Plot of BCF
Dev from Normal
0,4
0,3
0,2
0,1
0,0000
-0,1
-0,2
30,0
32,5
35,0
37,5
40,0
42,5
Observed Value

Detrended Normal Q-Q Plot of STR

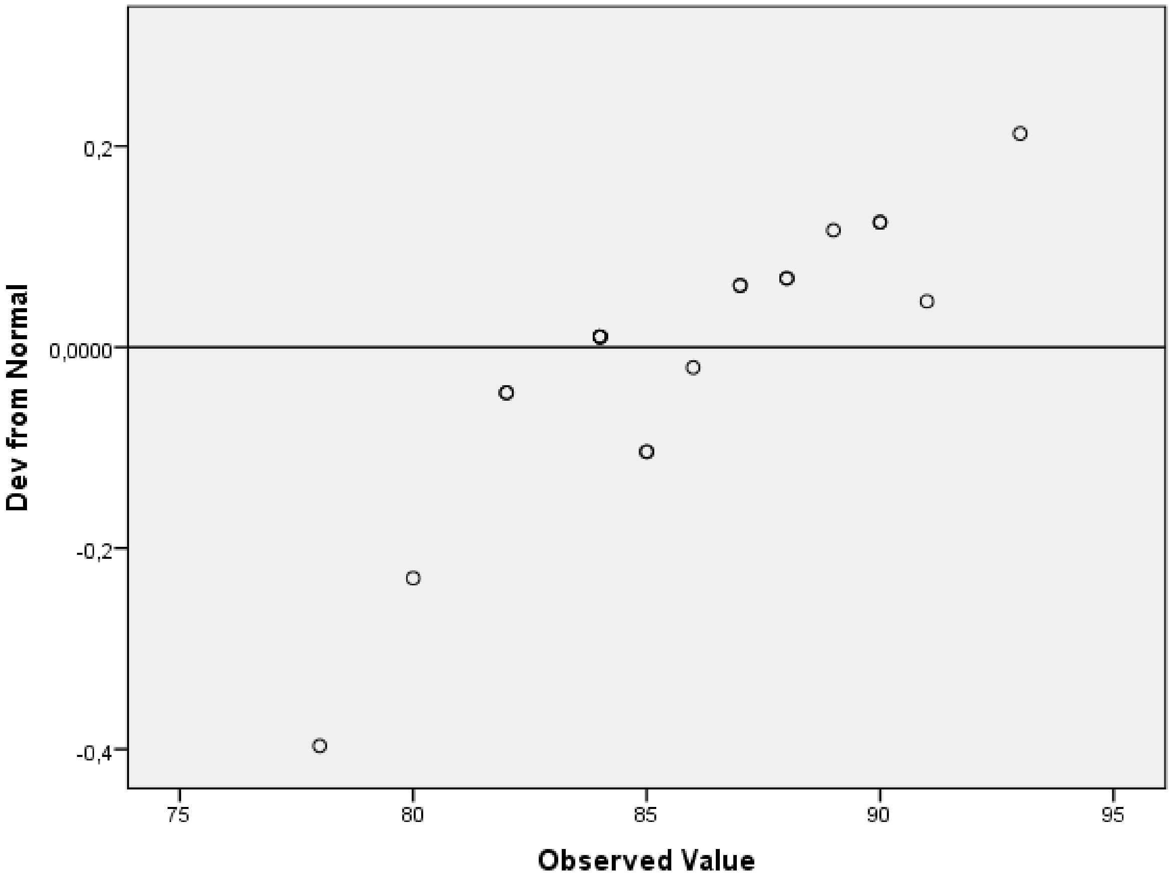

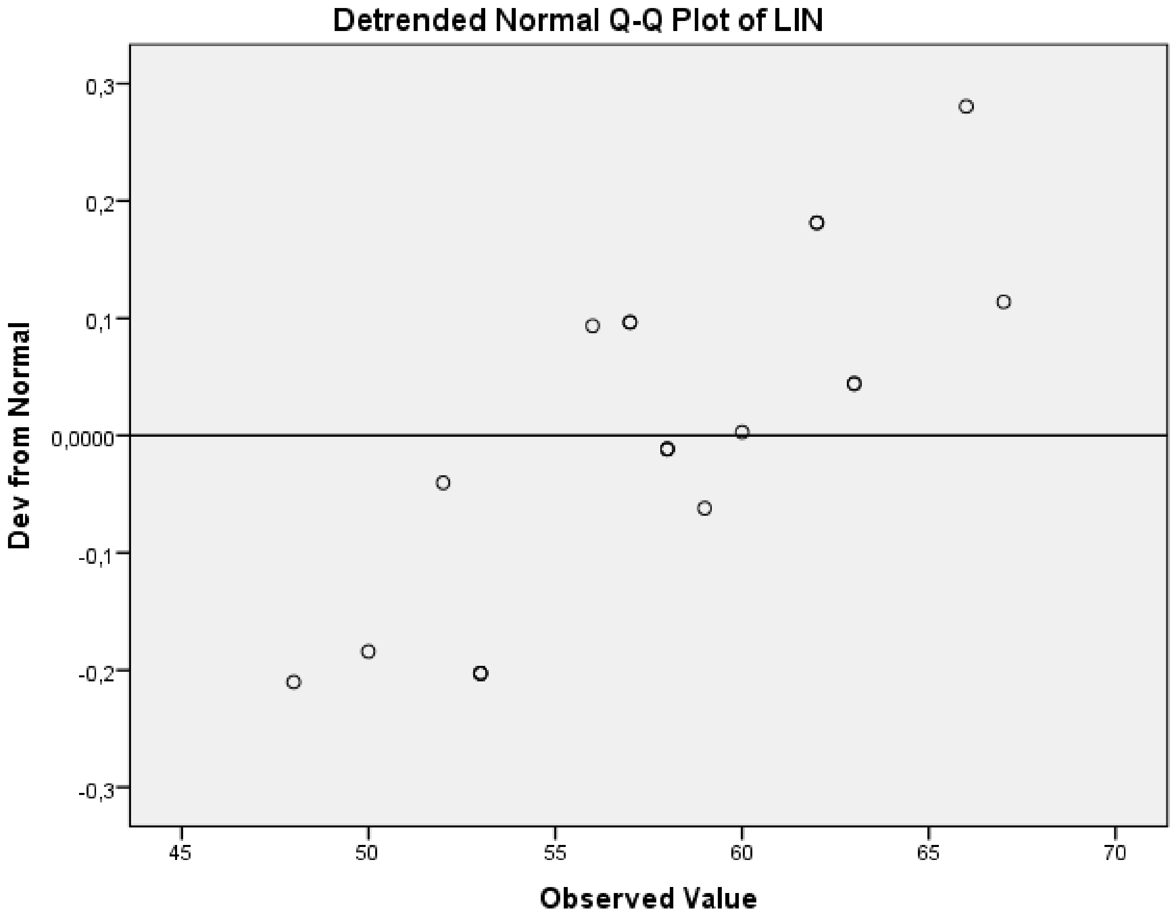

Gruplar = 2,00

Histograms

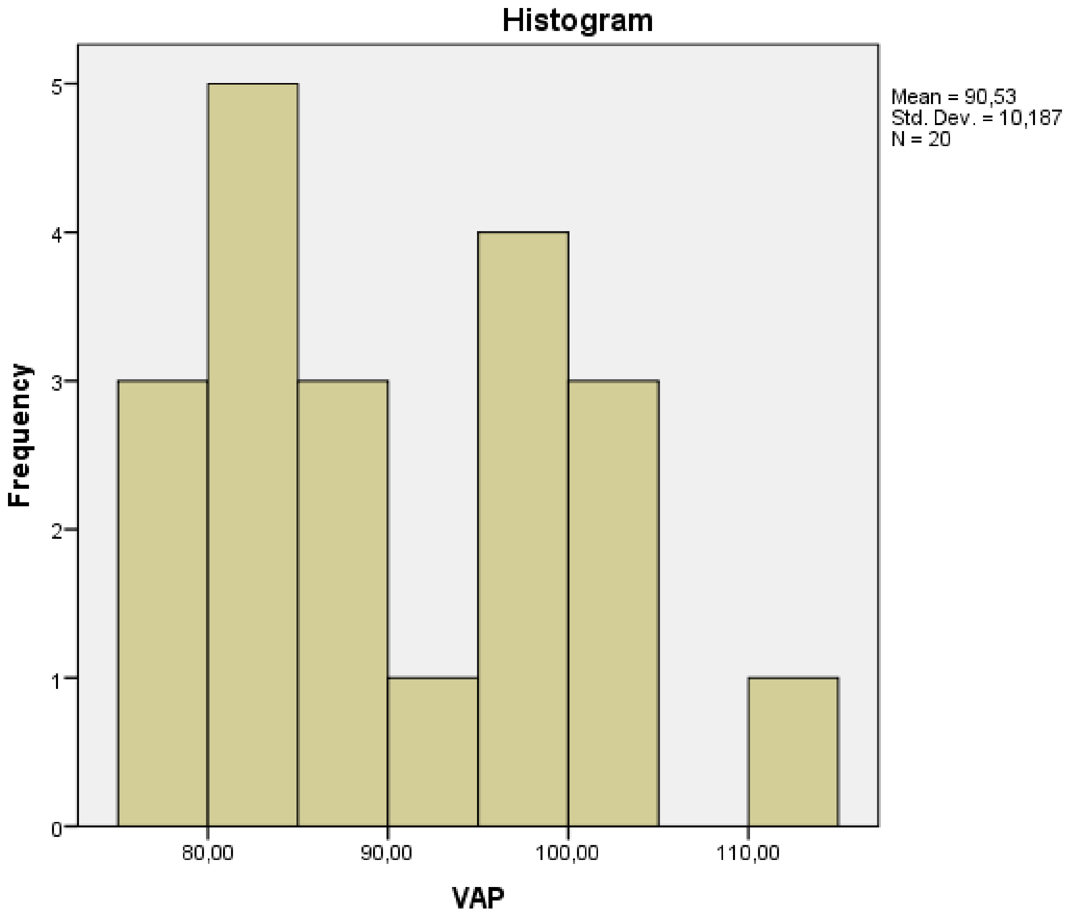
Histogram
Mean = 90,53
Std. Dev. = 10,187
N = 20
Frequency
0
1
2
3
4
5
80,00
90,00
100,00
110,00
VAP

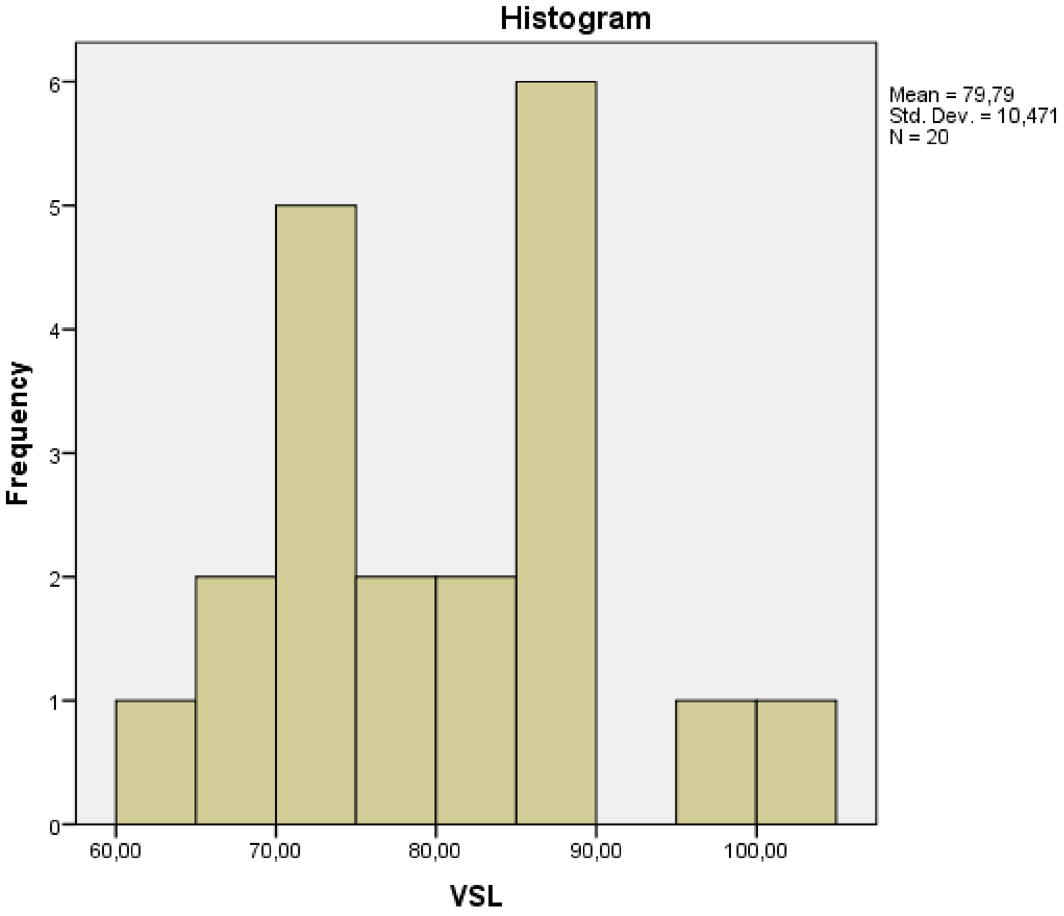

Histogram
Mean = 79,79
Std. Dev. = 10,471
N = 20
Frequency
VSL
0
1
2
3
4
5
6
60,00
70,00
80,00
90,00
100,00

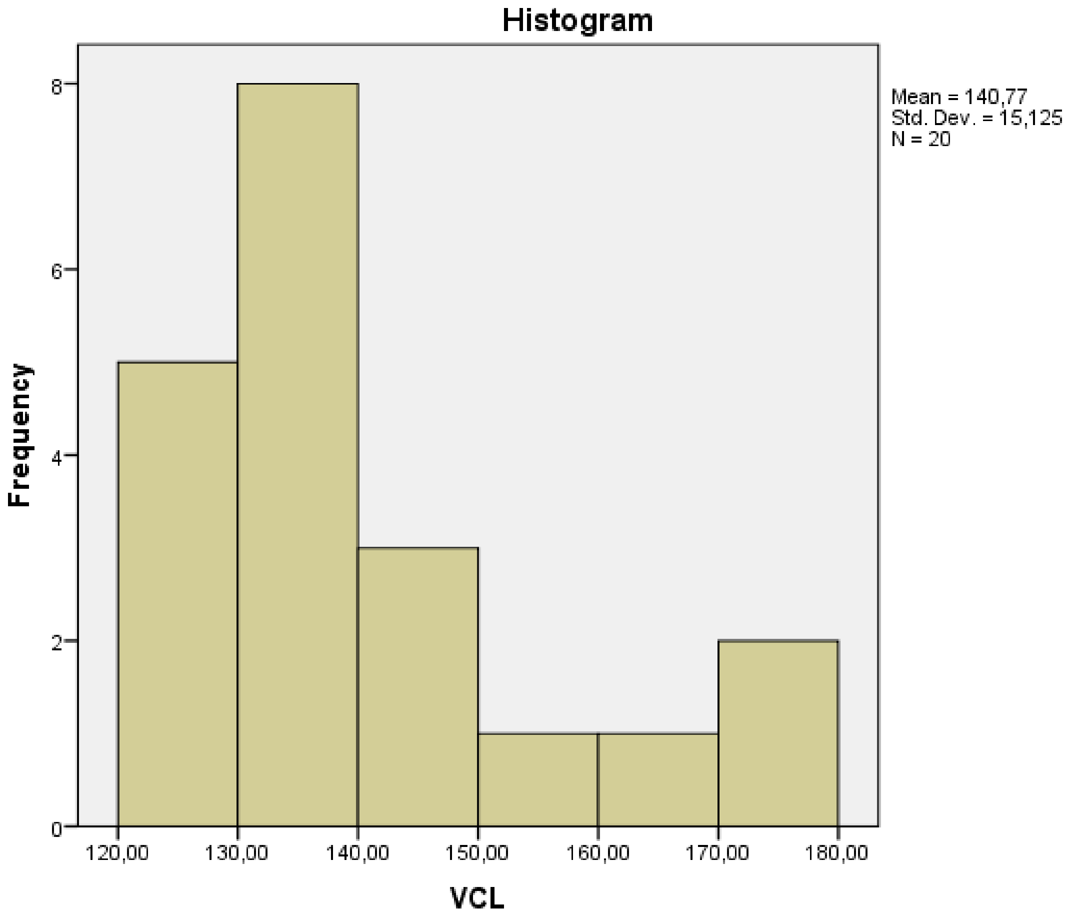
Histogram
Mean = 140,77
Std. Dev. = 15,125
N = 20
Frequency
0
2
4
6
8
120,00
130,00
140,00
150,00
160,00
170,00
180,00
VCL

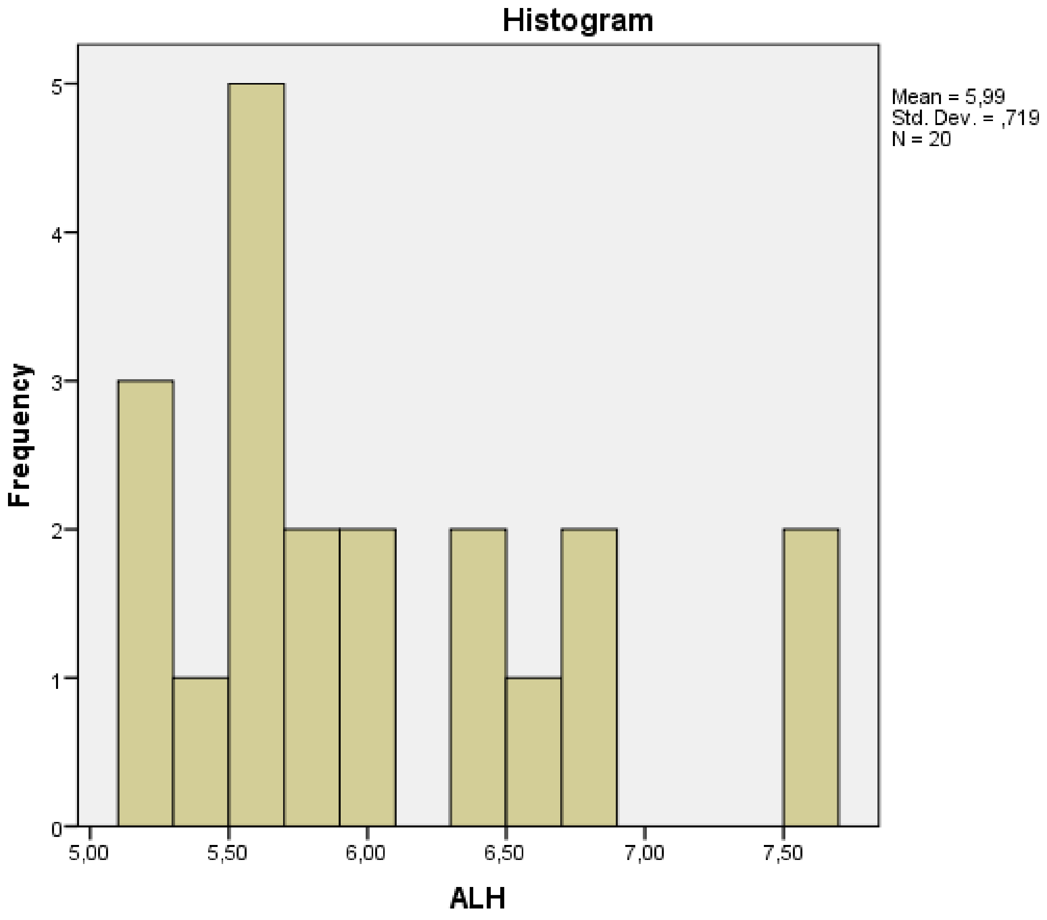
Histogram
Frequency
0
1
2
3
4
5
5,00
5,50
6,00
6,50
7,00
7,50
ALH
Mean = 5,99
Std. Dev. = ,719
N = 20

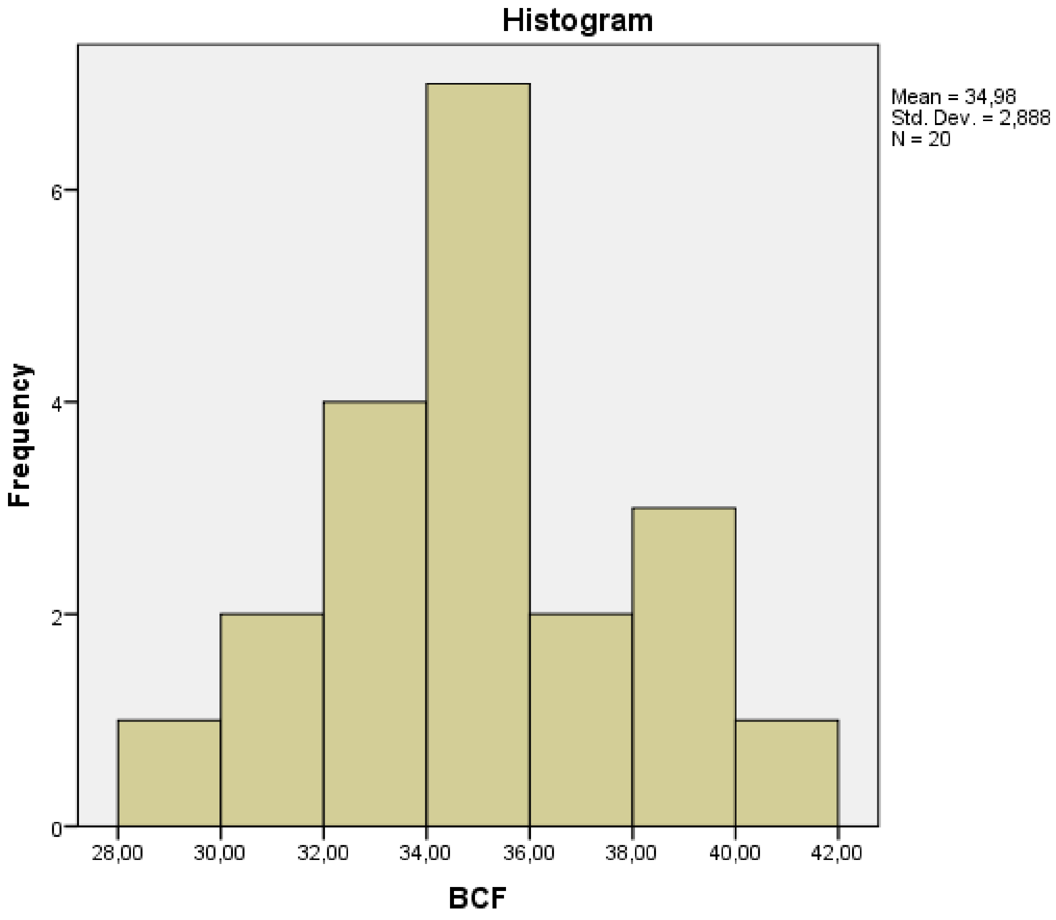
Histogram
Mean = 34,98
Std. Dev. = 2,888
N = 20
Frequency
0
2
4
6
28,00
30,00
32,00
34,00
36,00
38,00
40,00
42,00
BCF

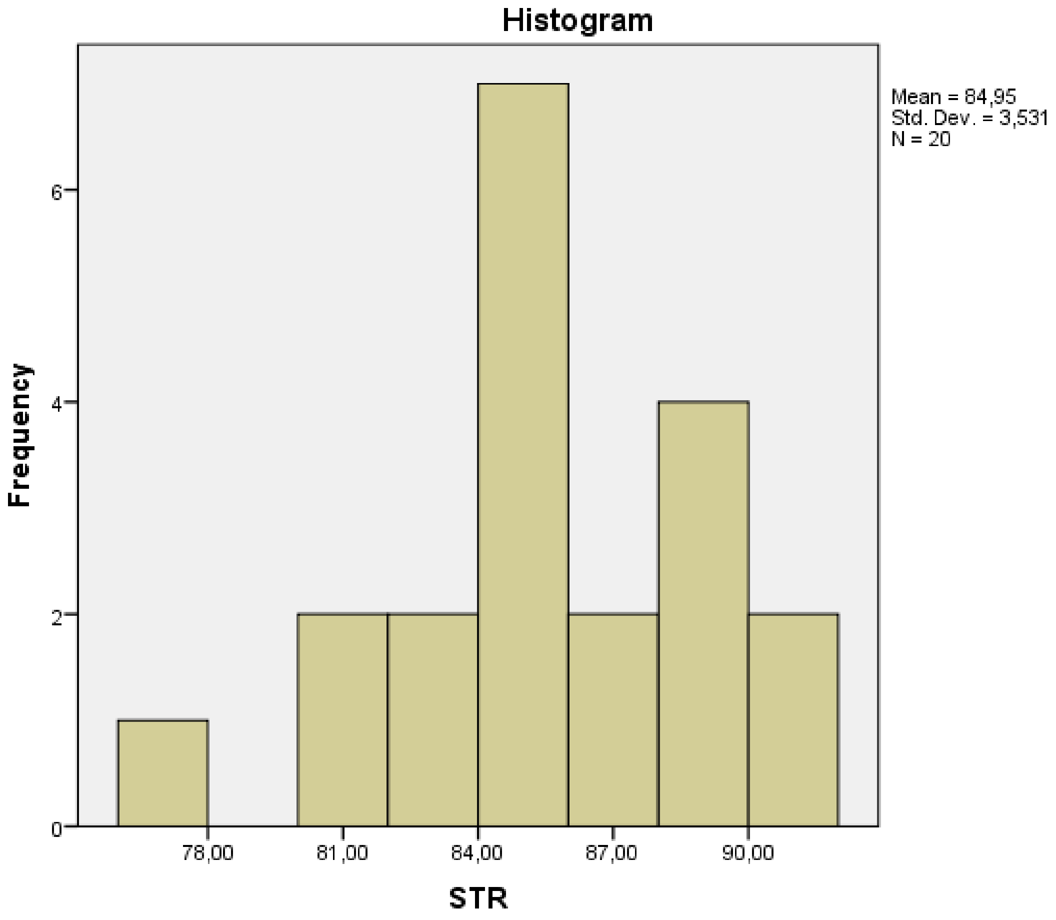
Histogram
Mean = 84,95
Std. Dev. = 3,531
N = 20
Frequency
0
2
4
6
78,00
81,00
84,00
87,00
90,00
STR

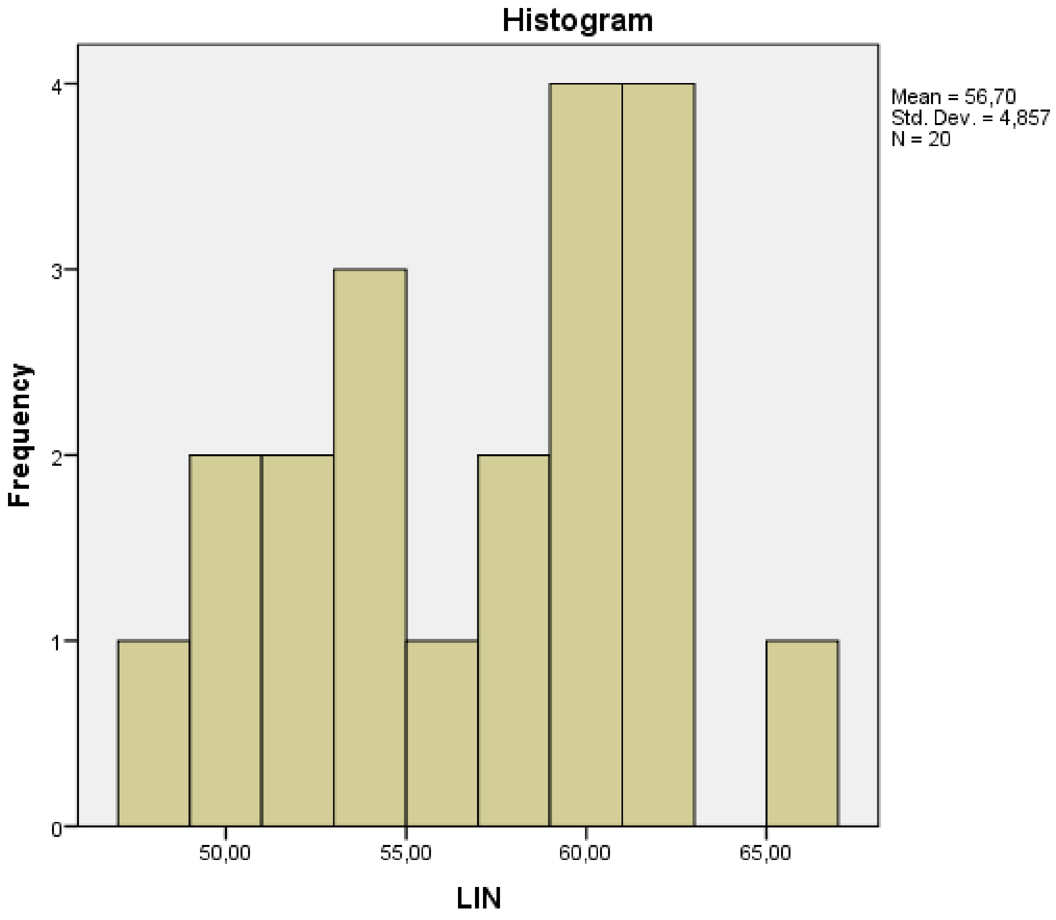

Stem-and-Leaf Plots

```
VAP Stem-and-Leaf Plot for
Gruplar= 2,00

 Frequency    Stem &  Leaf

     3,00        7 .  788
     8,00        8 .  01122679
     5,00        9 .  46689
     3,00       10 .  024
     1,00       11 .  1

 Stem width:  10,00
 Each leaf:       1 case(s)
```

```
VSL Stem-and-Leaf Plot for
Gruplar= 2,00

 Frequency    Stem &  Leaf

     3,00        6 .  378
     7,00        7 .  0001258
     8,00        8 .  04666779
     1,00        9 .  7
     1,00       10 .  1

 Stem width:  10,00
 Each leaf:       1 case(s)
```

```
VCL Stem-and-Leaf Plot for
Gruplar= 2,00

 Frequency    Stem &  Leaf

     5,00       12 .  12999
     8,00       13 .  01234688
     3,00       14 .  479
     1,00       15 .  7
     1,00       16 .  4
     2,00       17 .  02

 Stem width:  10,00
 Each leaf:       1 case(s)
```

```
ALH Stem-and-Leaf Plot for
Gruplar= 2,00

 Frequency    Stem &  Leaf

     4,00        5 .  2223
     8,00        5 .  55556889
     3,00        6 .  044
     3,00        6 .  677
      ,00        7 .
     2,00        7 .  55

 Stem width:   1,00
 Each leaf:       1 case(s)
```

```
BCF Stem-and-Leaf Plot for
Gruplar= 2,00
```

```
 Frequency    Stem &  Leaf

     1,00        2 .  9
    10,00        3 .  1123334444
     8,00        3 .  55567899
     1,00        4 .  0

 Stem width:  10,00
 Each leaf:       1 case(s)
```

```
STR Stem-and-Leaf Plot for
Gruplar= 2,00

 Frequency    Stem &  Leaf

     1,00        7 .  7
     8,00        8 .  00234444
     9,00        8 .  555668889
     2,00        9 .  01

 Stem width:  10,00
 Each leaf:       1 case(s)
```

```
LIN Stem-and-Leaf Plot for
Gruplar= 2,00

 Frequency    Stem &  Leaf

     1,00        4 .  8
     7,00        5 .  0022444
     5,00        5 .  57899
     6,00        6 .  001122
     1,00        6 .  6

 Stem width:  10,00
 Each leaf:       1 case(s)
```

Normal Q-Q Plots

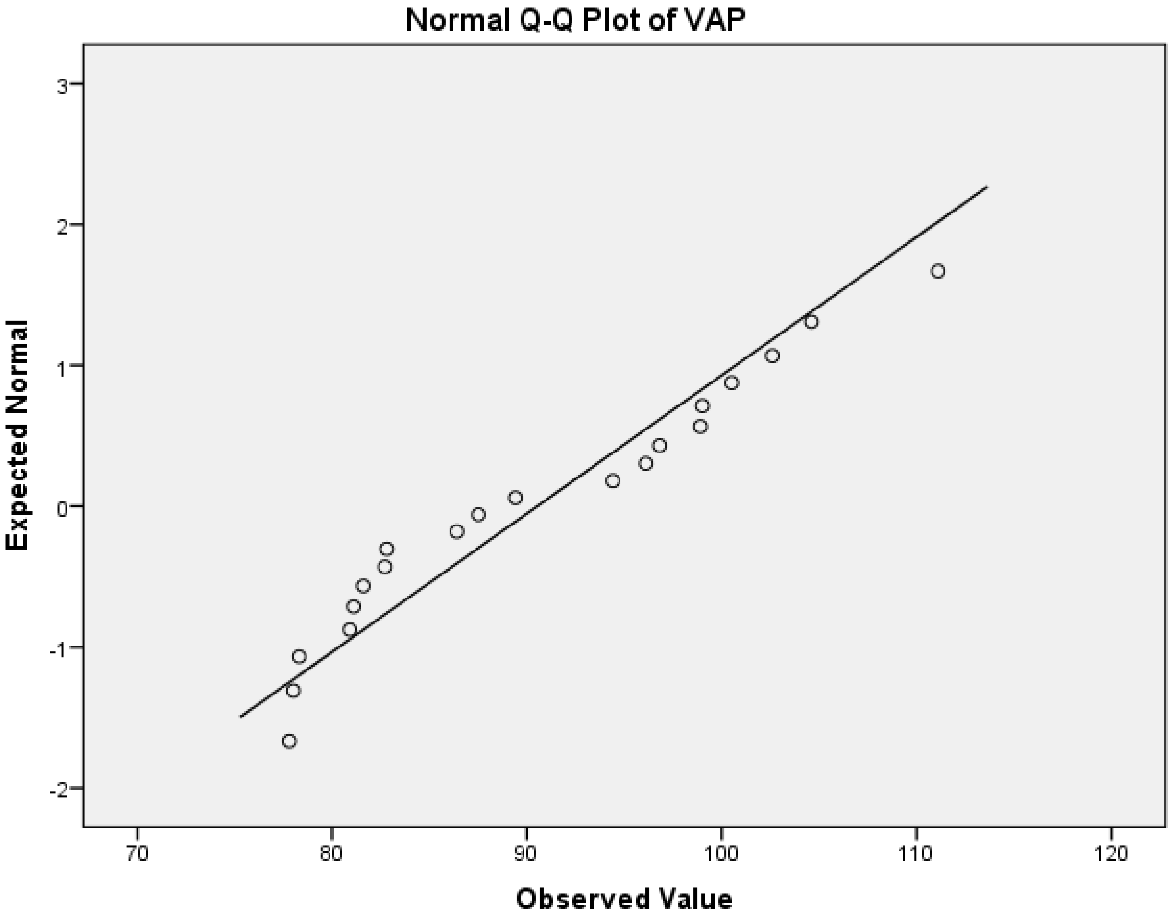
Normal Q-Q Plot of VAP
Expected Normal
Observed Value
3
2
1
0
-1
-2
70
80
90
100
110
120

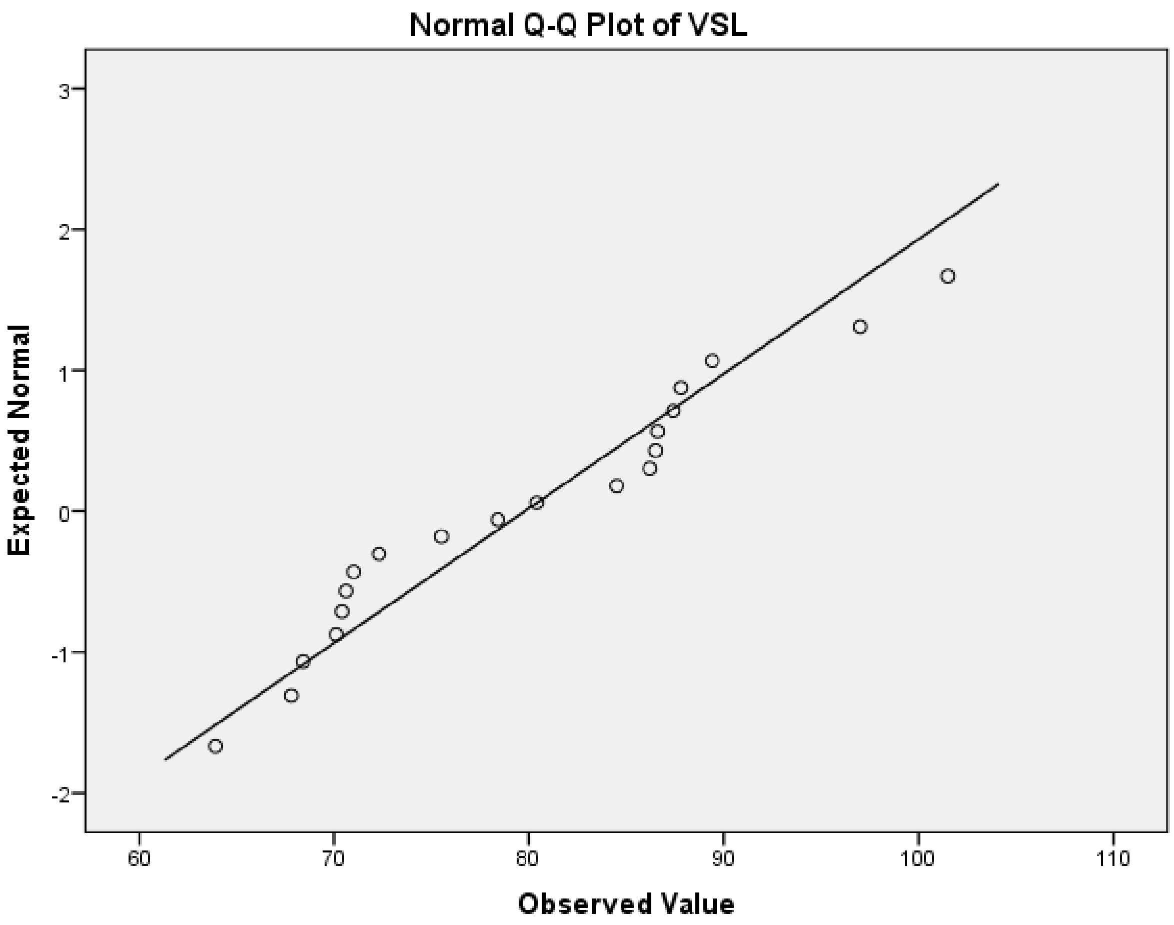
Normal Q-Q Plot of VSL
Expected Normal
Observed Value
3
2
1
0
-1
-2
60
70
80
90
100
110

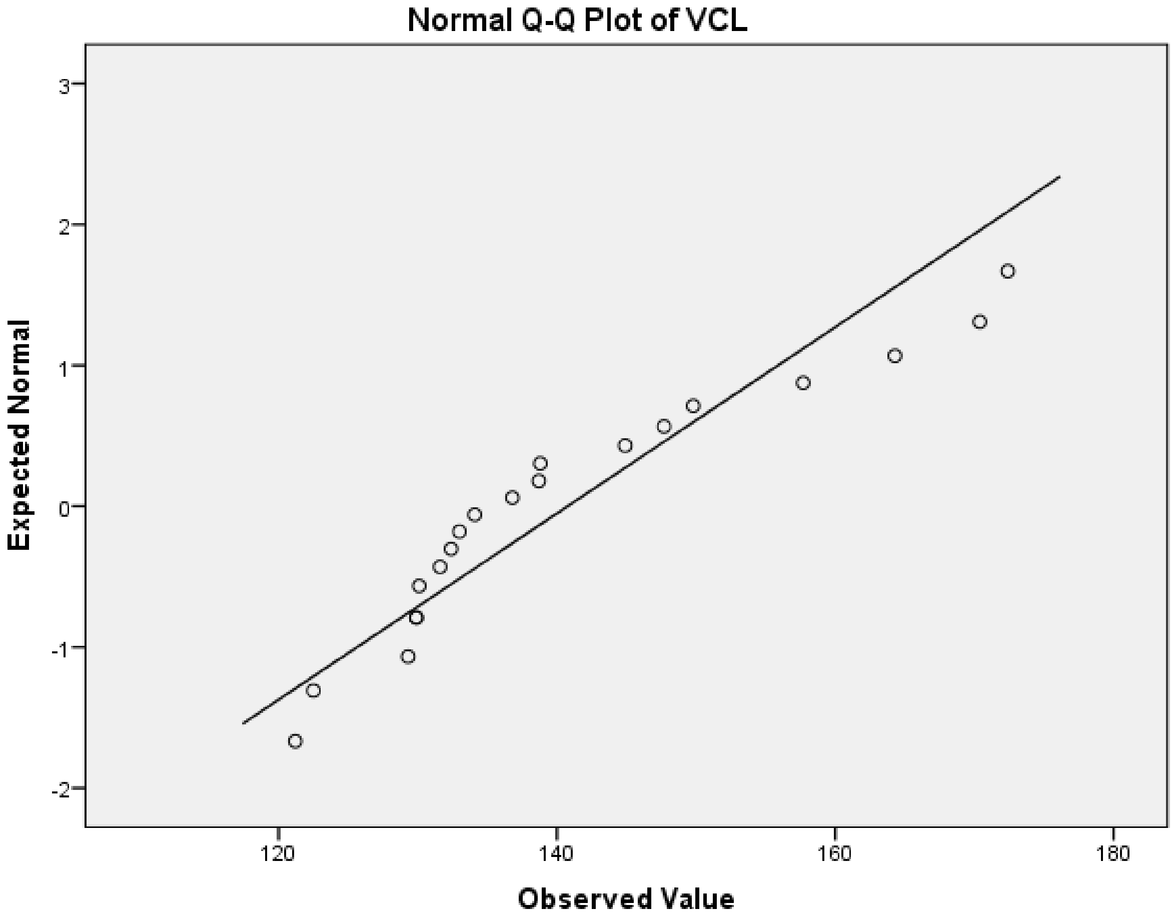
Normal Q-Q Plot of VCL
Expected Normal
Observed Value
3
2
1
0
-1
-2
120
140
160
180

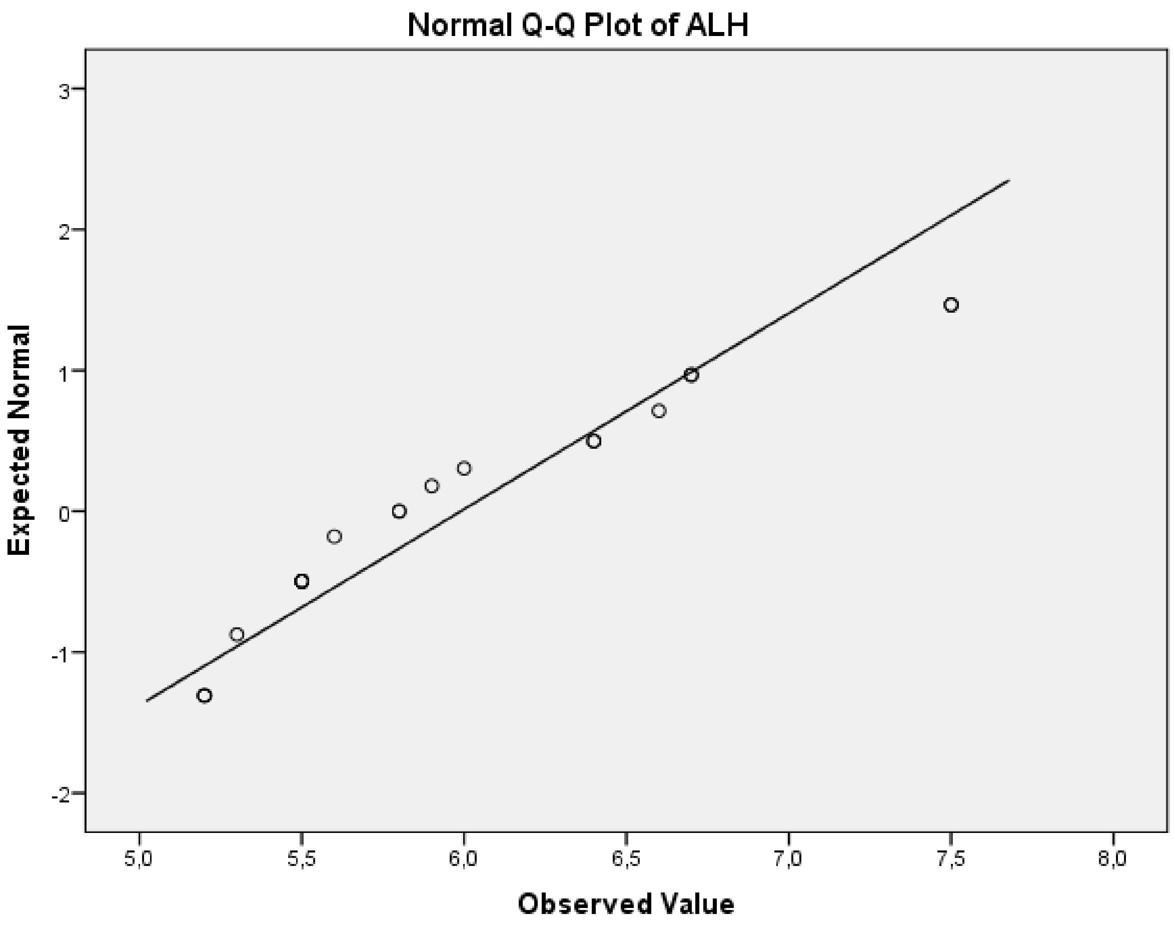
Normal Q-Q Plot of ALH
Expected Normal
3
2
1
0
-1
-2
5,0
5,5
6,0
6,5
7,0
7,5
8,0
Observed Value

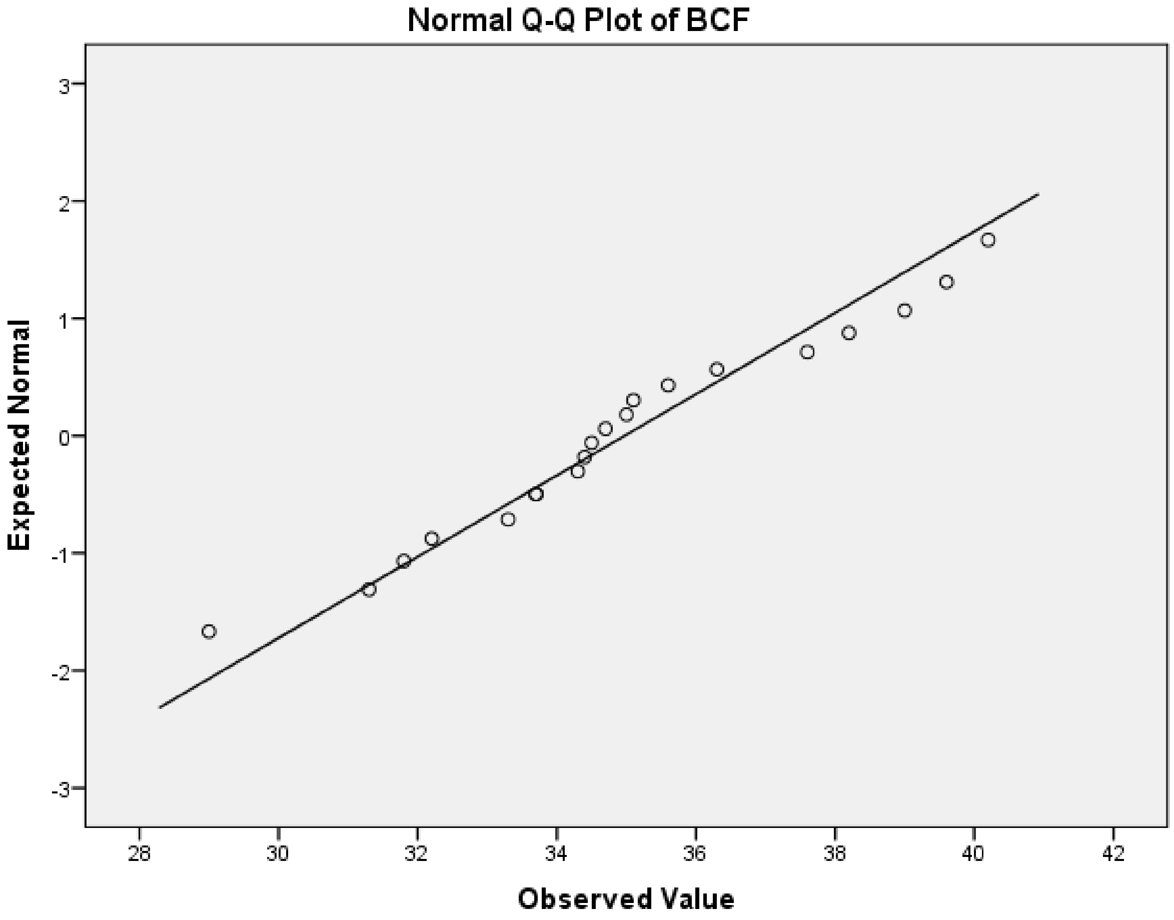
Normal Q-Q Plot of BCF
Expected Normal
Observed Value
3
2
1
0
-1
-2
-3
28
30
32
34
36
38
40
42

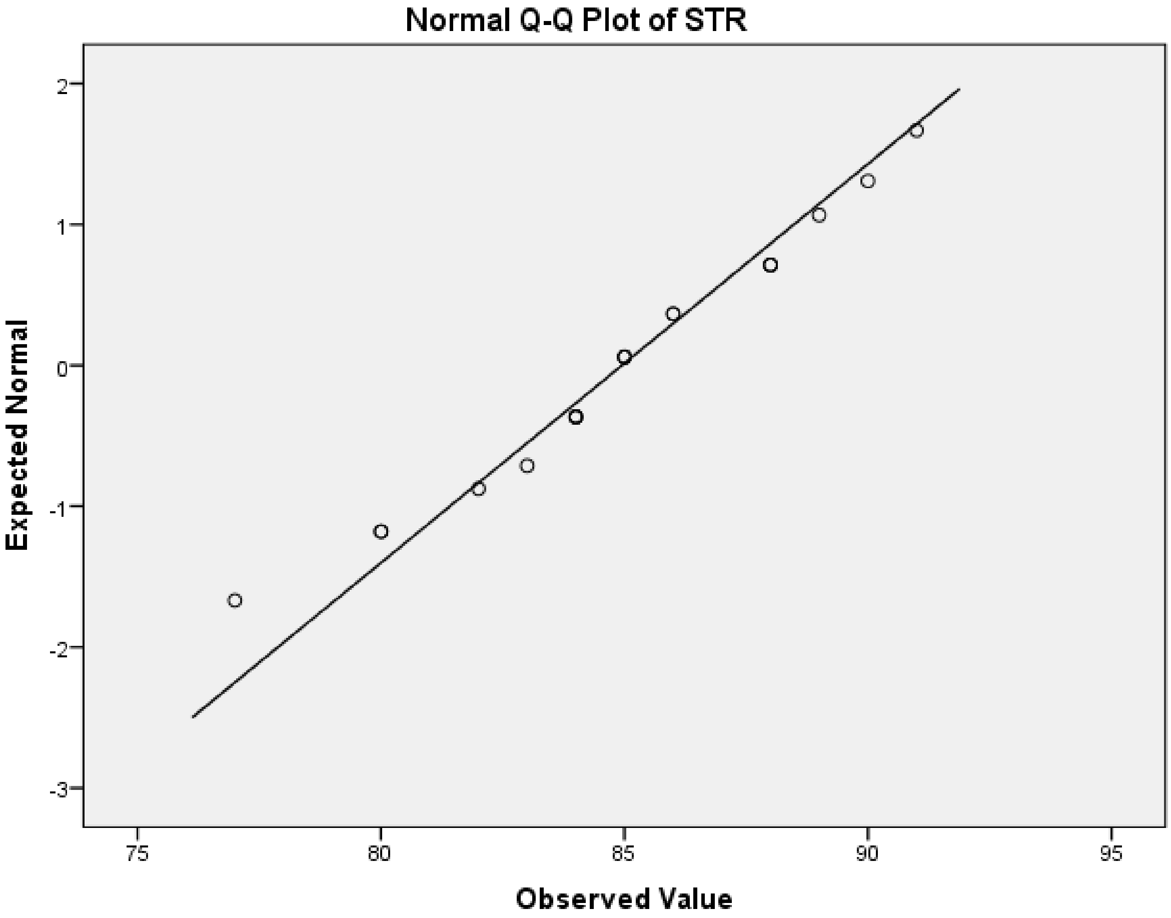

Normal Q-Q Plot of STR
Expected Normal
Observed Value
2
1
0
-1
-2
-3
75
80
85
90
95

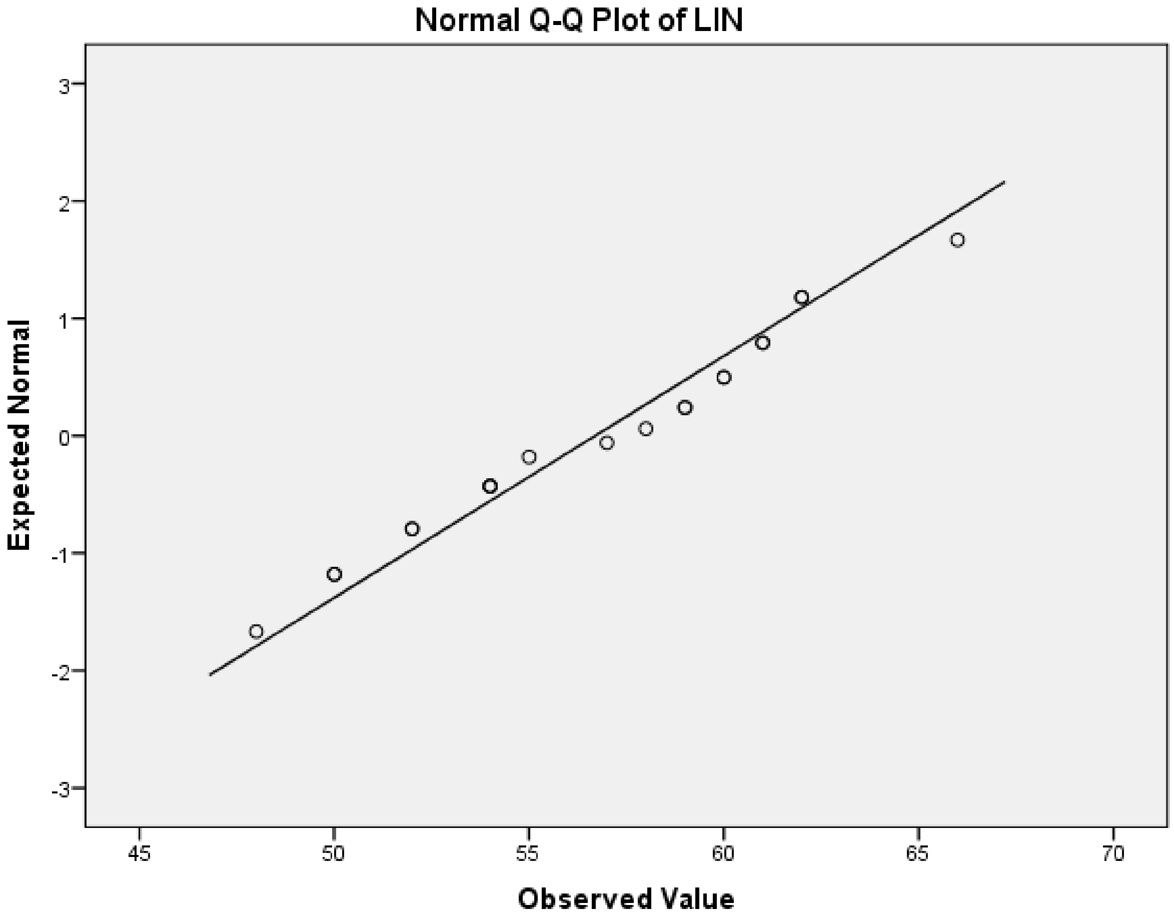

Detrended Normal Q-Q Plots

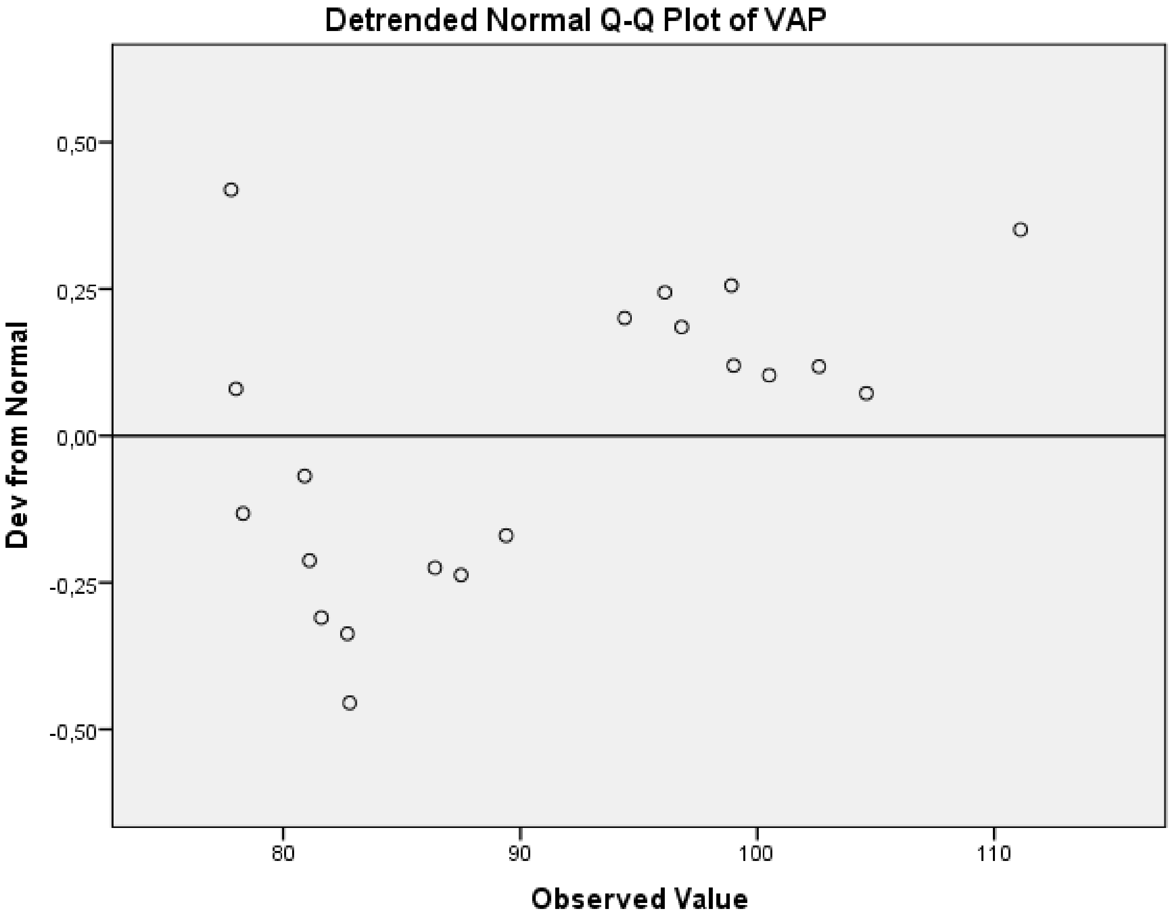

Detrended Normal Q-Q Plot of VAP
0,50
0,25
0,00
-0,25
-0,50
Dev from Normal
80
90
100
110
Observed Value

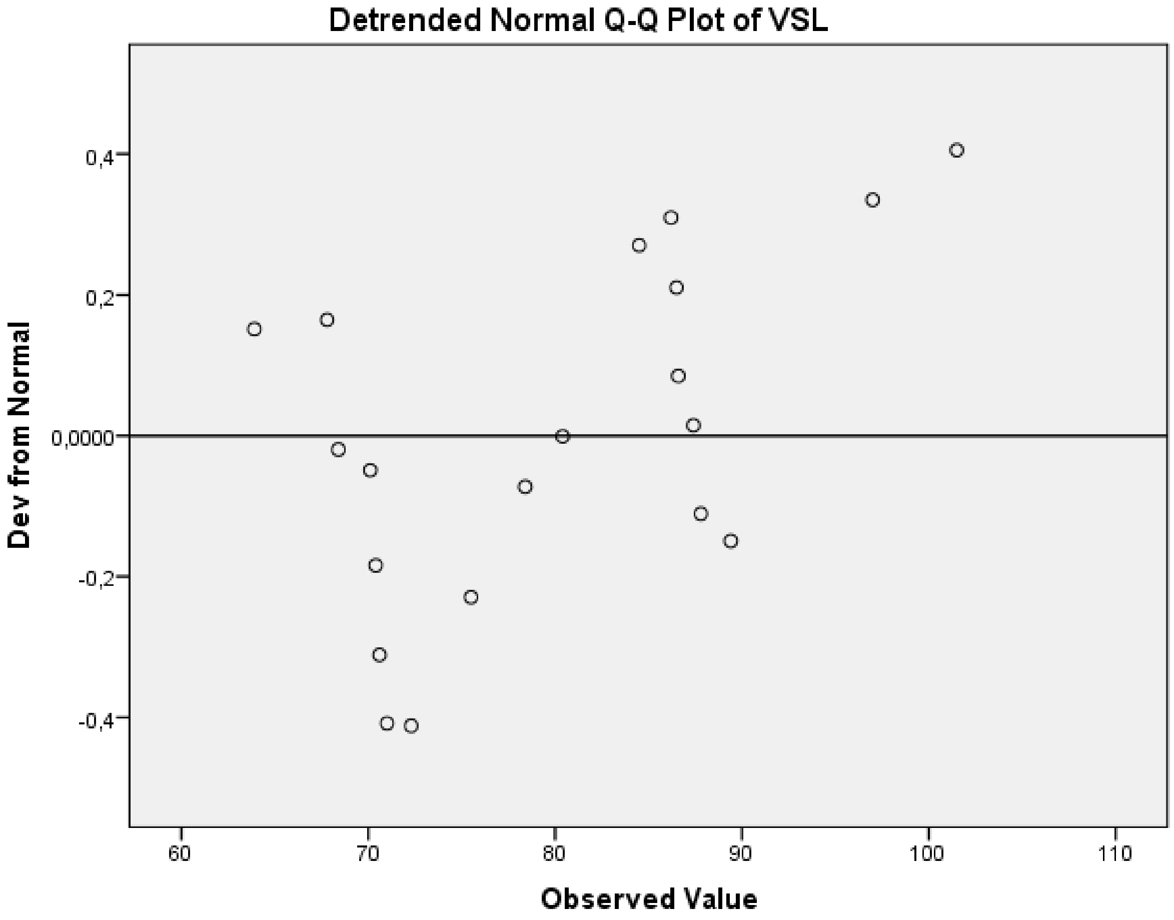

Detrended Normal Q-Q Plot of VSL
0,4
0,2
0,0000
-0,2
-0,4
Dev from Normal
60
70
80
90
100
110
Observed Value

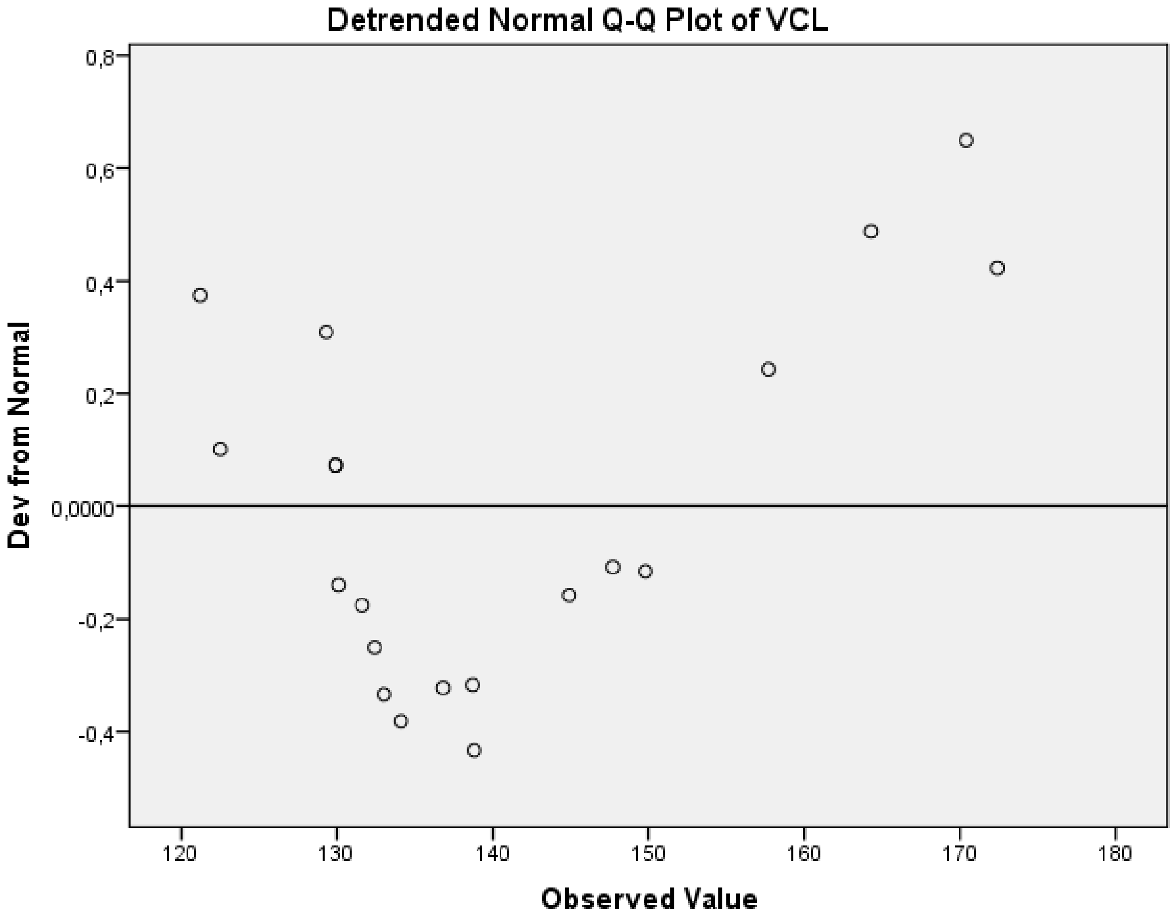
Detrended Normal Q-Q Plot of VCL
Dev from Normal
0,8
0,6
0,4
0,2
0,0000
-0,2
-0,4
120
130
140
150
160
170
180
Observed Value

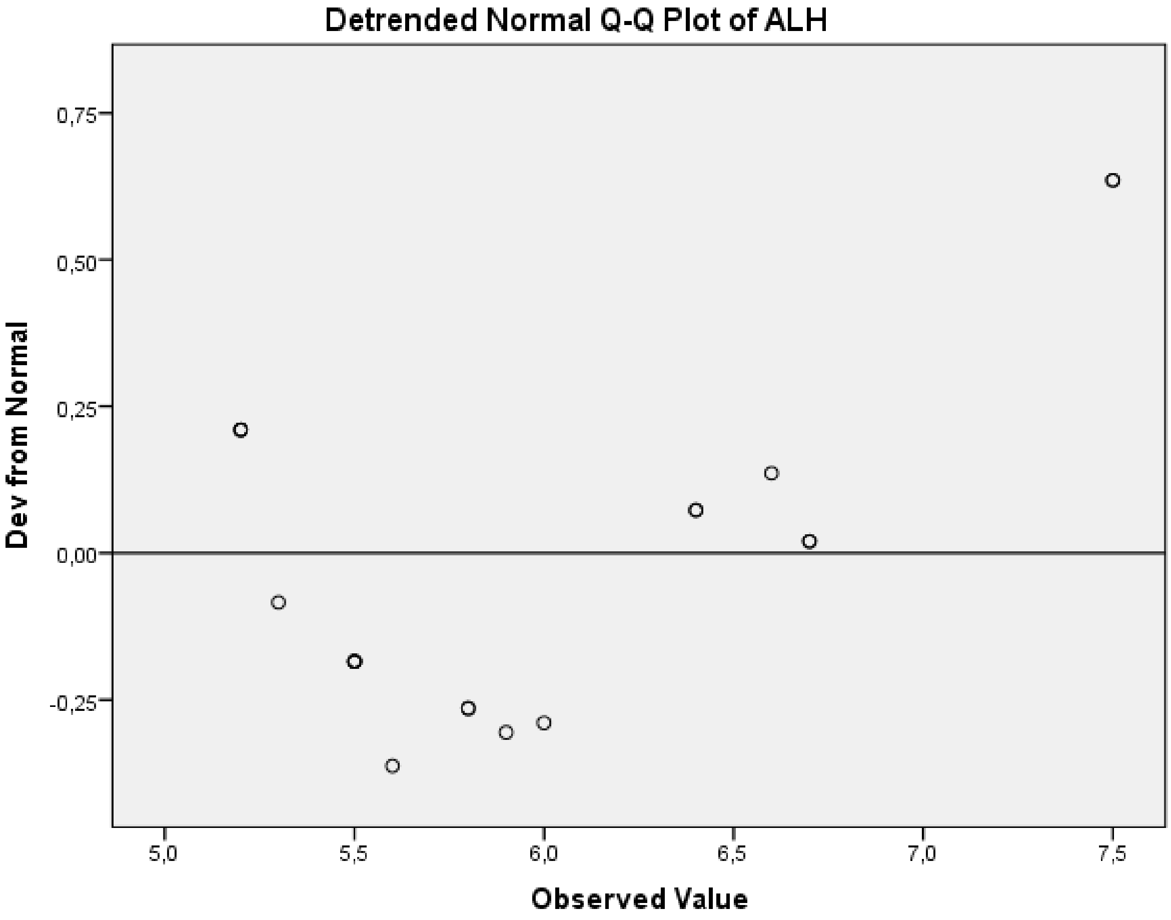
Detrended Normal Q-Q Plot of ALH
Dev from Normal
0,75
0,50
0,25
0,00
-0,25
5,0
5,5
6,0
6,5
7,0
7,5
Observed Value

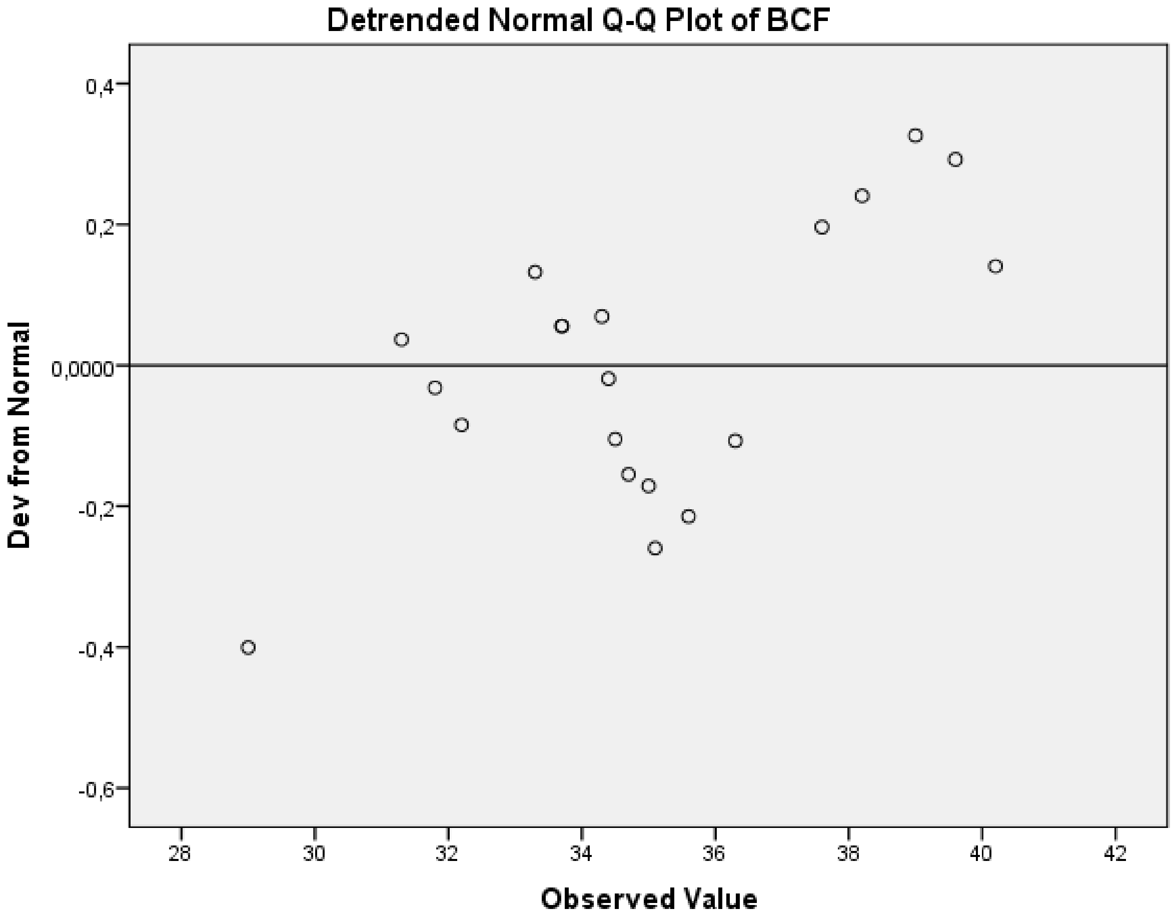
Detrended Normal Q-Q Plot of BCF
Dev from Normal
0,4
0,2
0,0000
-0,2
-0,4
-0,6
28
30
32
34
36
38
40
42
Observed Value

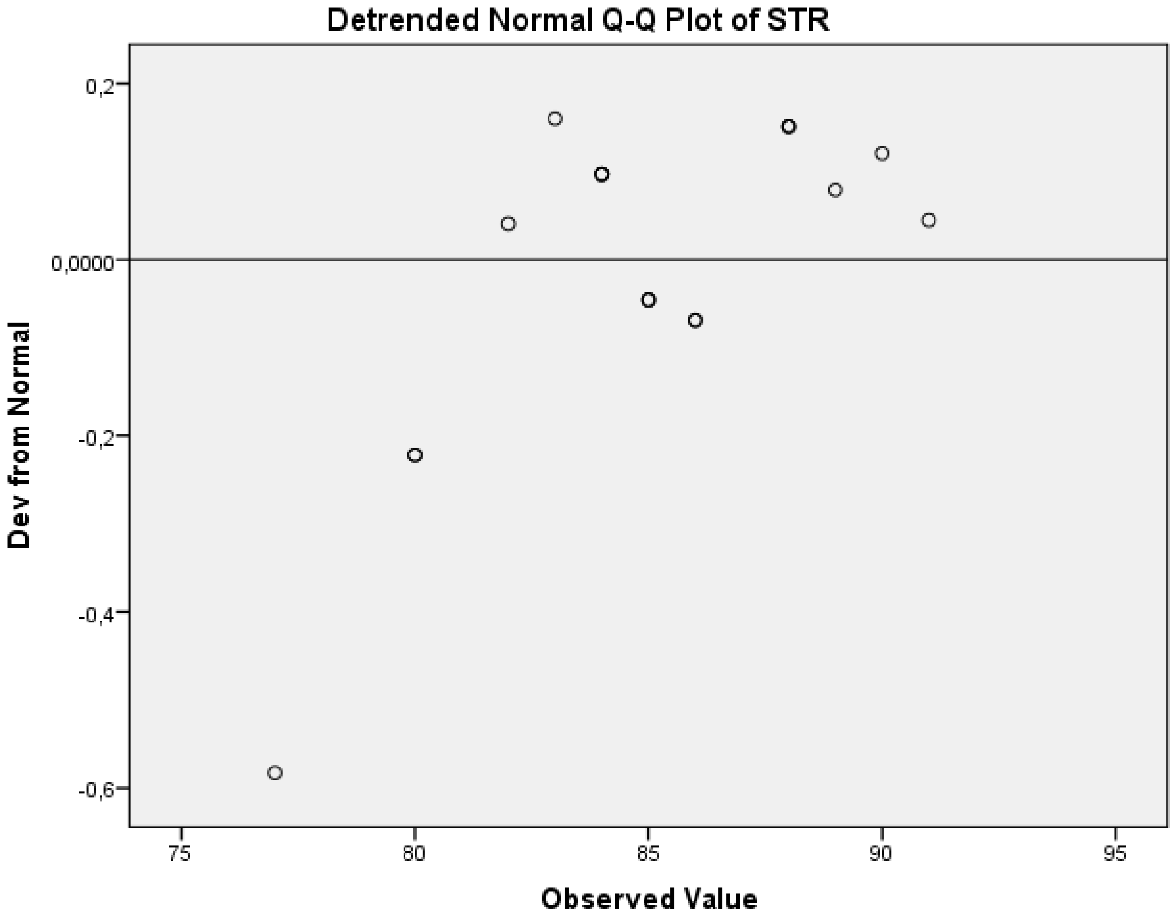
Detrended Normal Q-Q Plot of STR
Dev from Normal
0,2
0,0000
-0,2
-0,4
-0,6
75
80
85
90
95
Observed Value

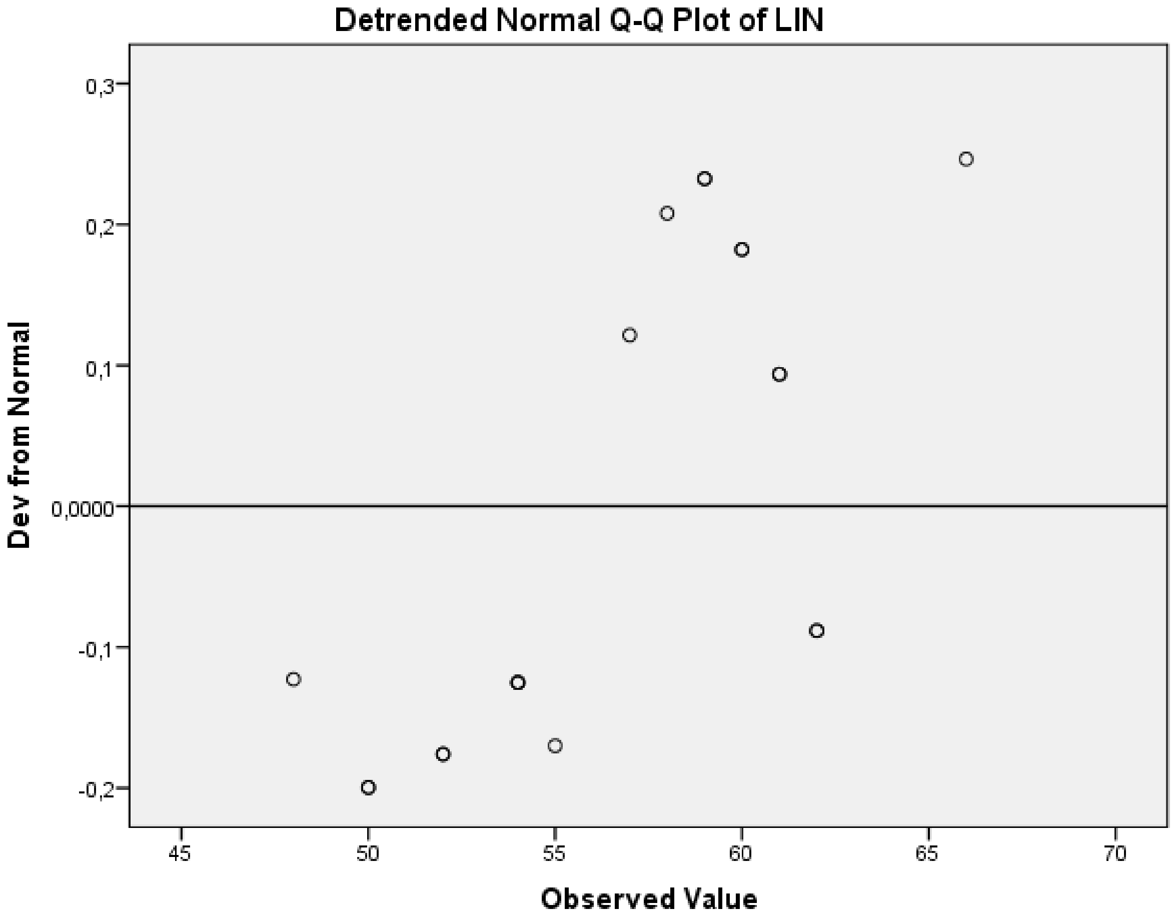

Gruplar = 3,00

Histograms

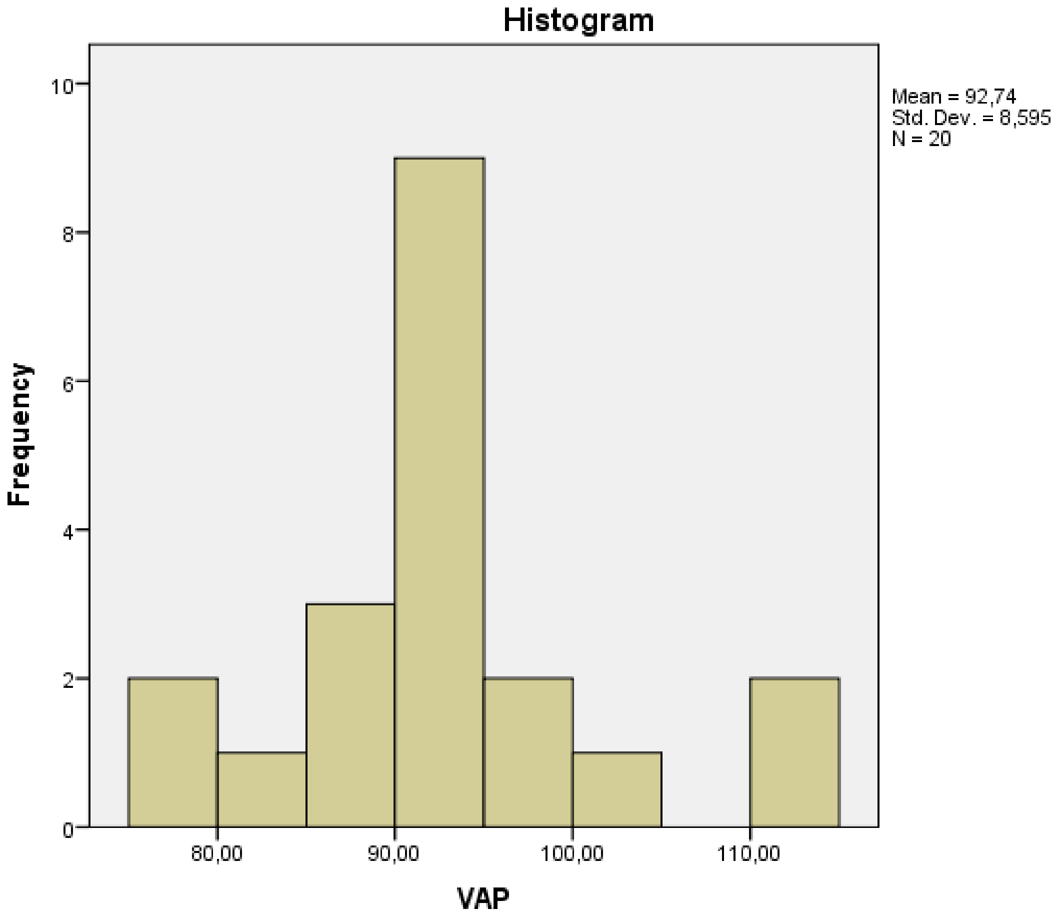
Histogram
Mean = 92,74
Std. Dev. = 8,595
N = 20
Frequency
0
2
4
6
8
10
80,00
90,00
100,00
110,00
VAP

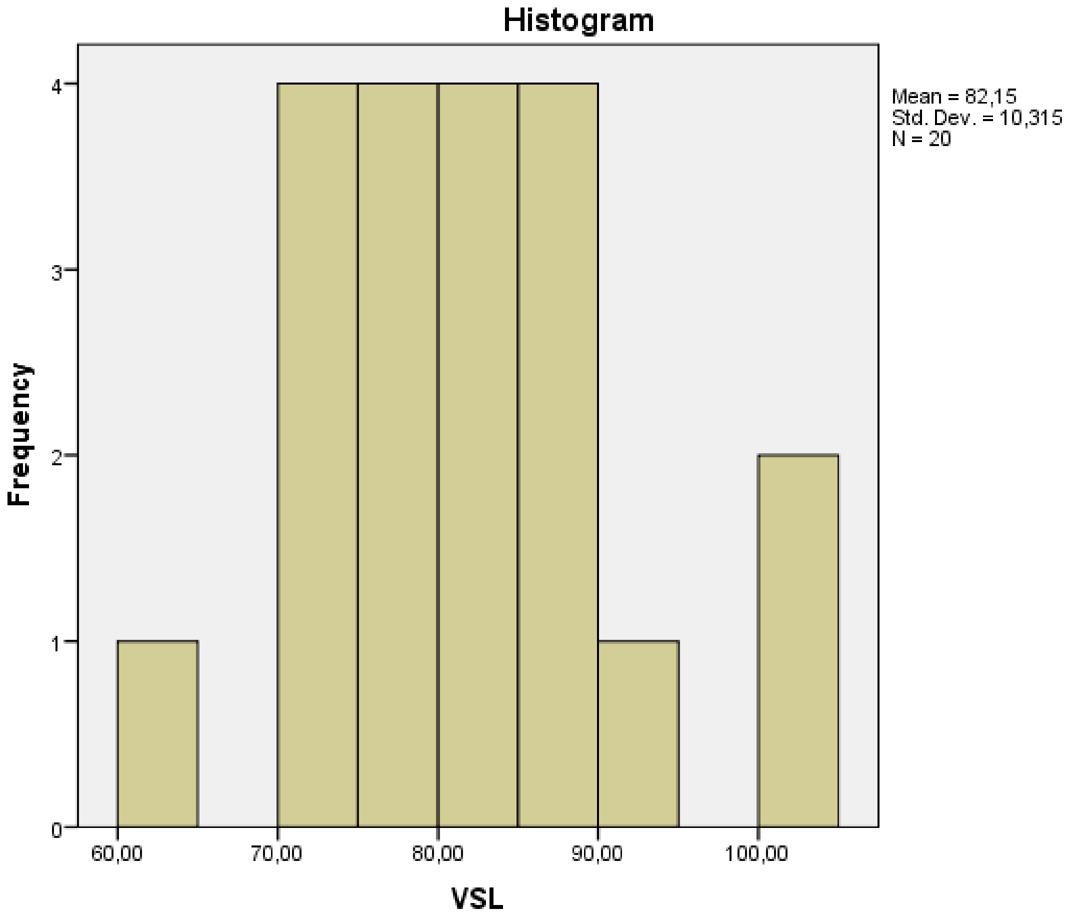
Histogram
Mean = 82,15
Std. Dev. = 10,315
N = 20
Frequency
0
1
2
3
4
60,00
70,00
80,00
90,00
100,00
VSL

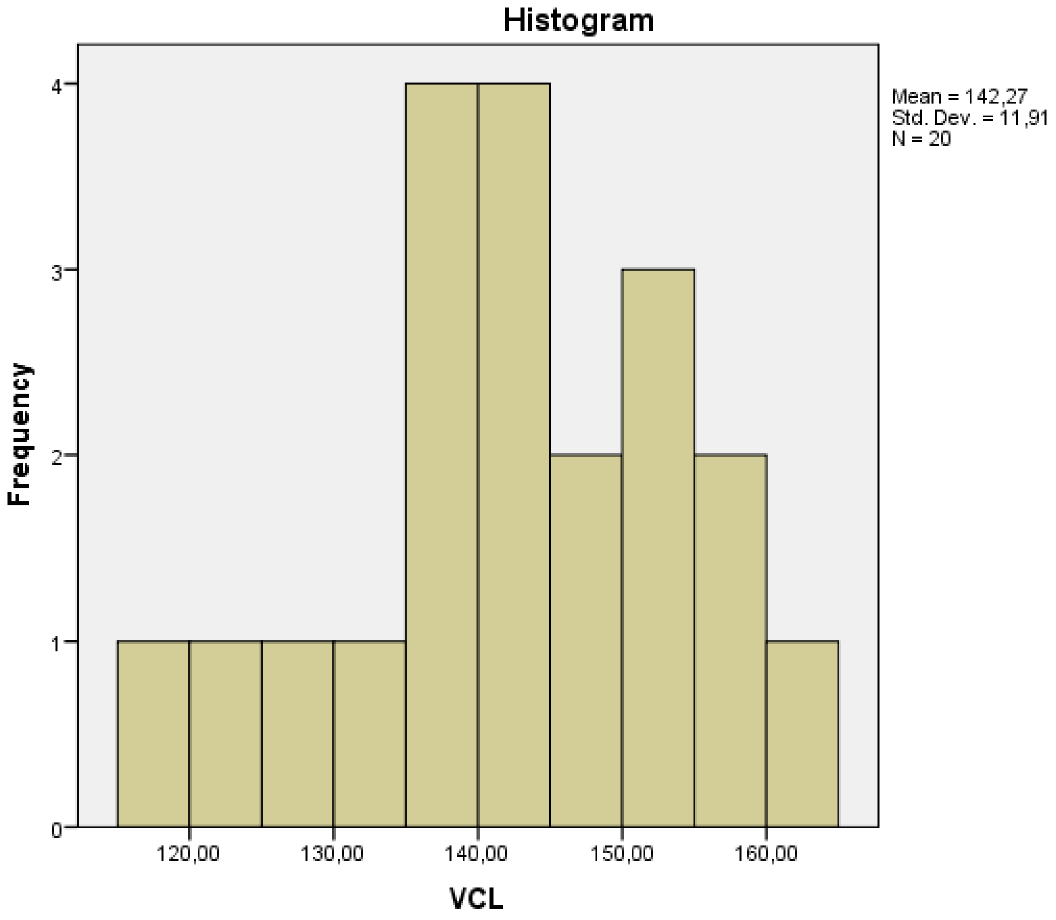
Histogram
Mean = 142,27
Std. Dev. = 11,91
N = 20
Frequency
0
1
2
3
4
120,00
130,00
140,00
150,00
160,00
VCL

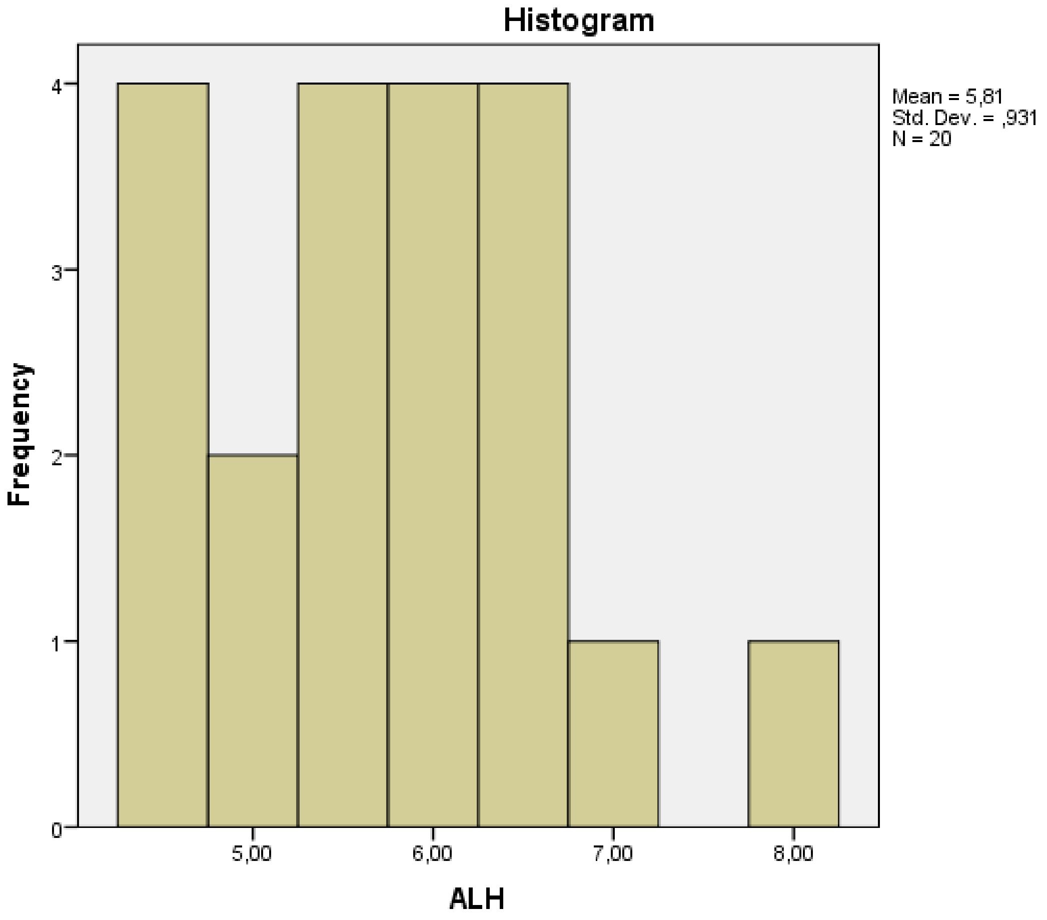

Histogram
Mean = 5,81
Std. Dev. = ,931
N = 20
Frequency
0
1
2
3
4
5,00
6,00
7,00
8,00
ALH

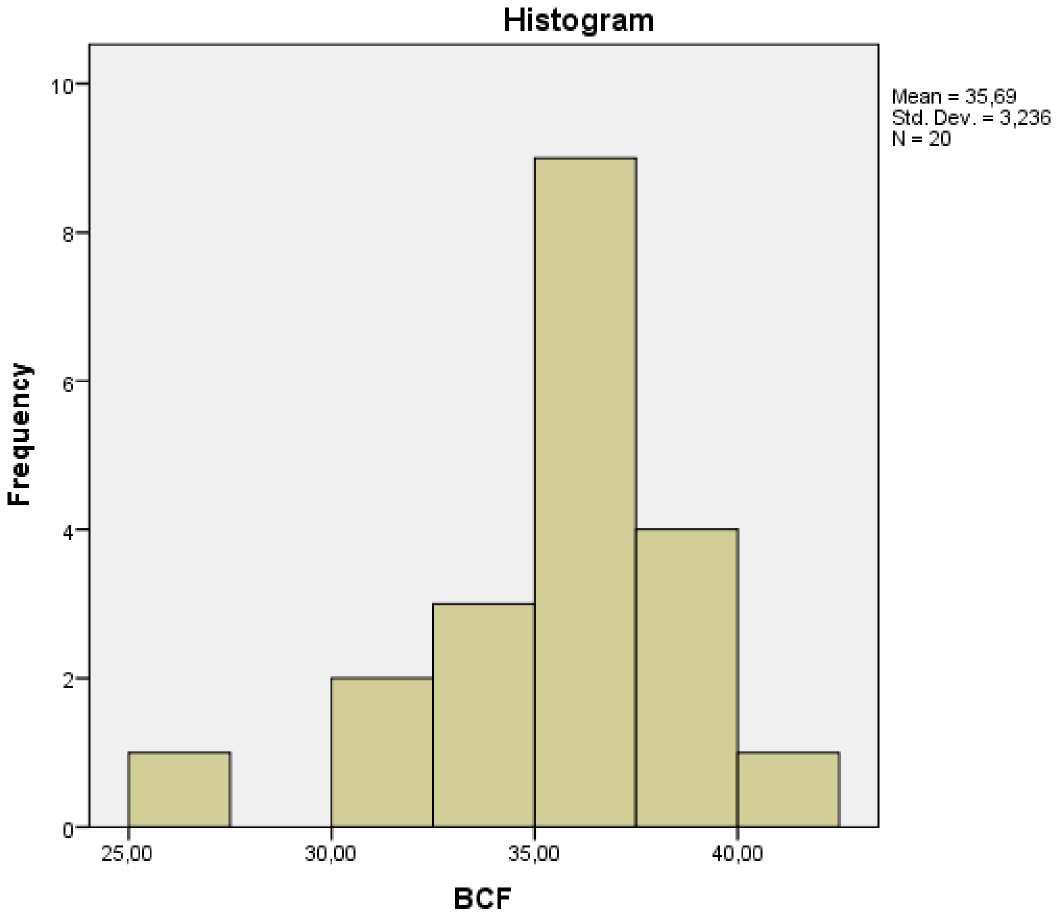
Histogram
Mean = 35,69
Std. Dev. = 3,236
N = 20
Frequency
10
8
6
4
2
0
25,00
30,00
35,00
40,00
BCF

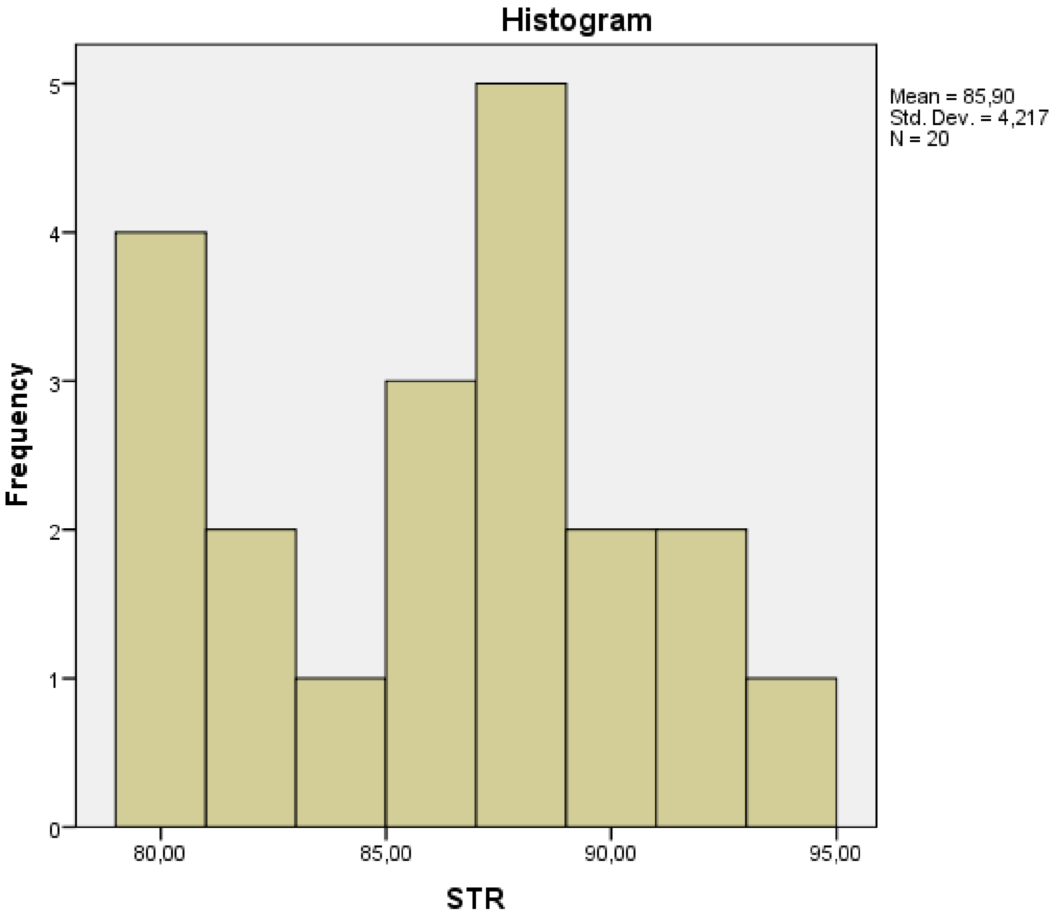

Histogram
Mean = 85,90
Std. Dev. = 4,217
N = 20
Frequency
0
1
2
3
4
5
80,00
85,00
90,00
95,00
STR

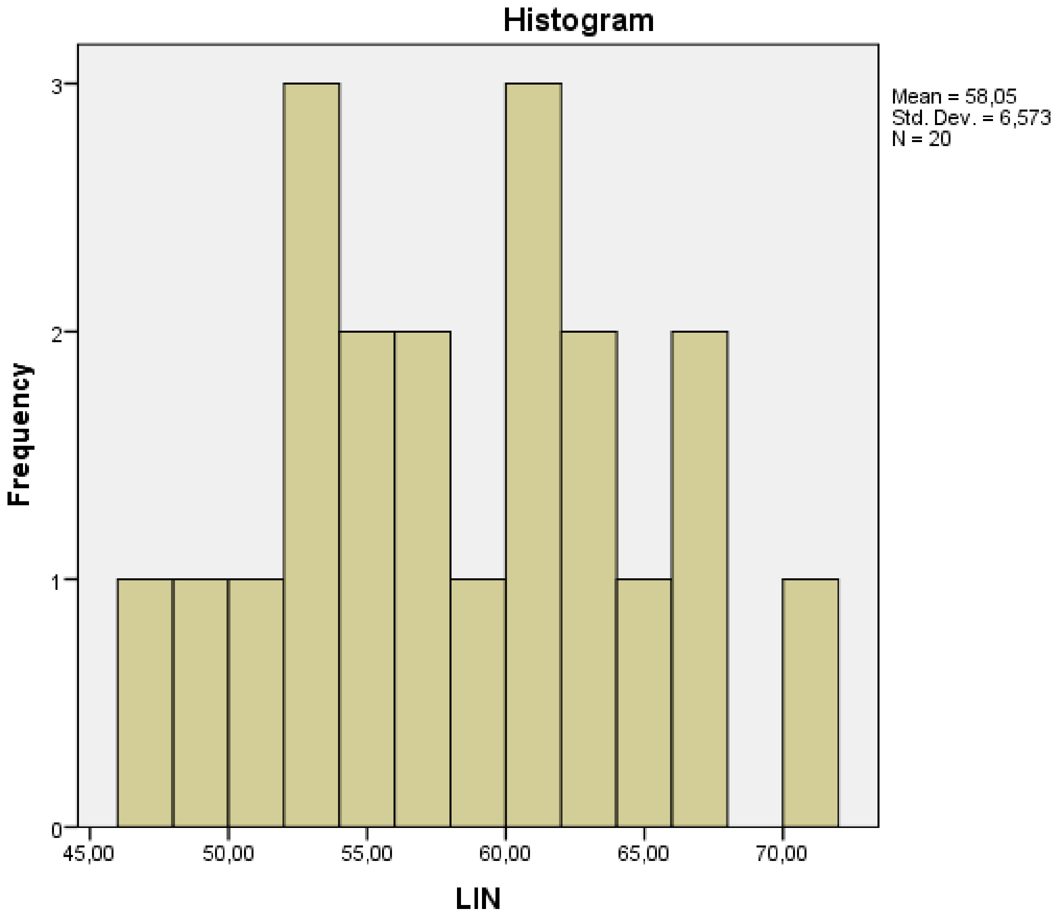

Stem-and-Leaf Plots

```
VAP Stem-and-Leaf Plot for
Gruplar= 3,00

 Frequency    Stem &  Leaf

     2,00        7 .  99
     1,00        8 .  3
     3,00        8 .  557
     9,00        9 .  011233444
     2,00        9 .  59
     1,00       10 .  1
     2,00 Extremes    (>=111)

 Stem width:  10,00
 Each leaf:       1 case(s)
```

VSL Stem-and-Leaf Plot for
Gruplar= 3,00

Frequency	Stem &	Leaf
1,00	6 .	2
8,00	7 .	12336789
8,00	8 .	01145679
1,00	9 .	3
1,00	10 .	3
1,00	Extremes	(>=105)

Stem width: 10,00
Each leaf: 1 case(s)

VCL Stem-and-Leaf Plot for
Gruplar= 3,00

Frequency	Stem &	Leaf
1,00	11 .	9
2,00	12 .	05
5,00	13 .	37789
6,00	14 .	000456
5,00	15 .	12467
1,00	16 .	3

Stem width: 10,00
Each leaf: 1 case(s)

ALH Stem-and-Leaf Plot for
Gruplar= 3,00

Frequency	Stem &	Leaf
4,00	4 .	5567
7,00	5 .	1245578
8,00	6 .	11246778
,00	7 .	
1,00	8 .	1

Stem width: 1,00
Each leaf: 1 case(s)

```
BCF Stem-and-Leaf Plot for
Gruplar= 3,00

 Frequency    Stem &  Leaf

     1,00 Extremes    (=<26,6)
     2,00       32 .  13
      ,00       33 .
     3,00       34 .  139
     6,00       35 .  133457
     2,00       36 .  08
     2,00       37 .  26
     1,00       38 .  2
     2,00       39 .  58
     1,00 Extremes    (>=42,1)

 Stem width:   1,00
 Each leaf:       1 case(s)
```

```
STR Stem-and-Leaf Plot for
Gruplar= 3,00

 Frequency    Stem &  Leaf

     7,00        8 .  0000224
     8,00        8 .  55677888
     5,00        9 .  00123

 Stem width:  10,00
 Each leaf:       1 case(s)
```

```
LIN Stem-and-Leaf Plot for
Gruplar= 3,00

 Frequency    Stem &  Leaf

     2,00        4 .  79
     5,00        5 .  02334
     4,00        5 .  5679
     5,00        6 .  00123
     3,00        6 .  577
     1,00        7 .  1

 Stem width:  10,00
 Each leaf:       1 case(s)
```

Normal Q-Q Plots

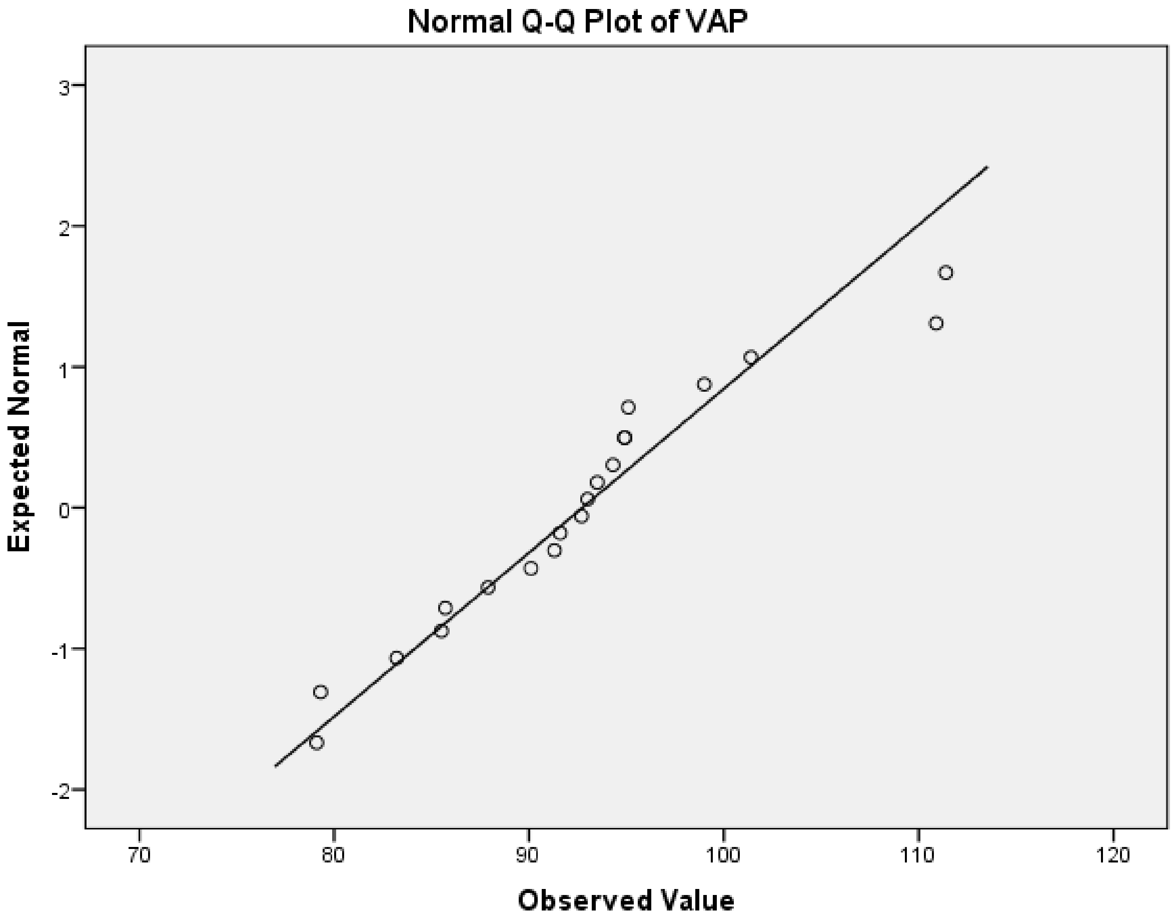

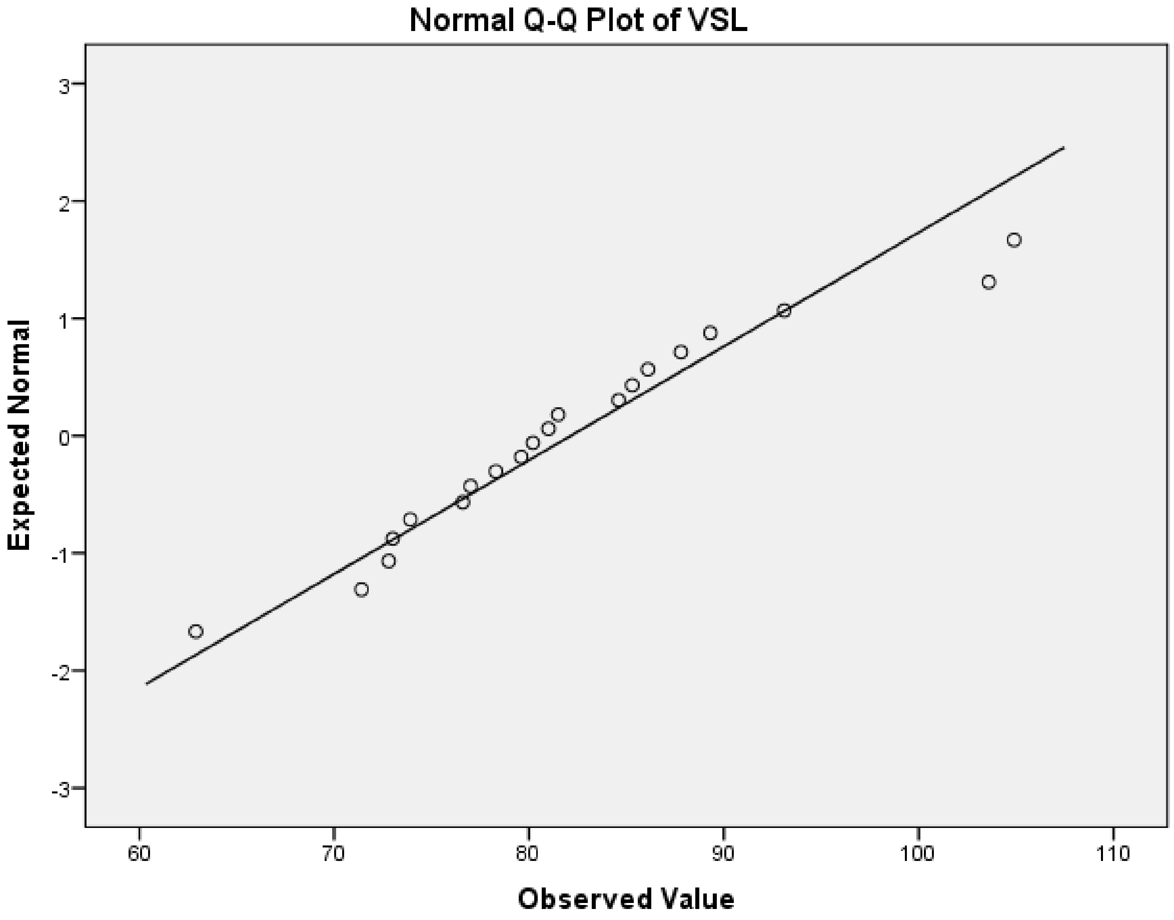

Normal Q-Q Plot of VSL
Expected Normal
Observed Value
3
2
1
0
-1
-2
-3
60
70
80
90
100
110

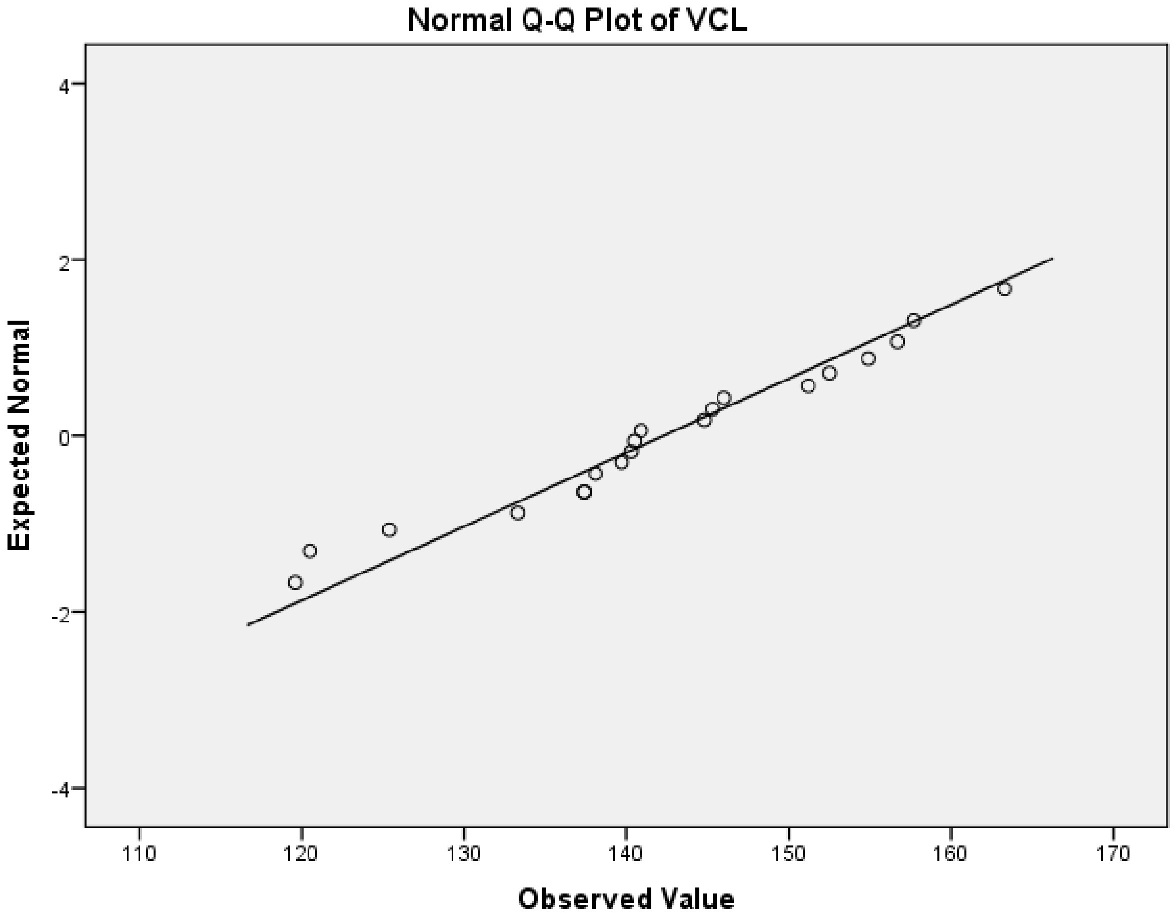

Normal Q-Q Plot of VCL
Expected Normal
Observed Value
4
2
0
-2
-4
110
120
130
140
150
160
170

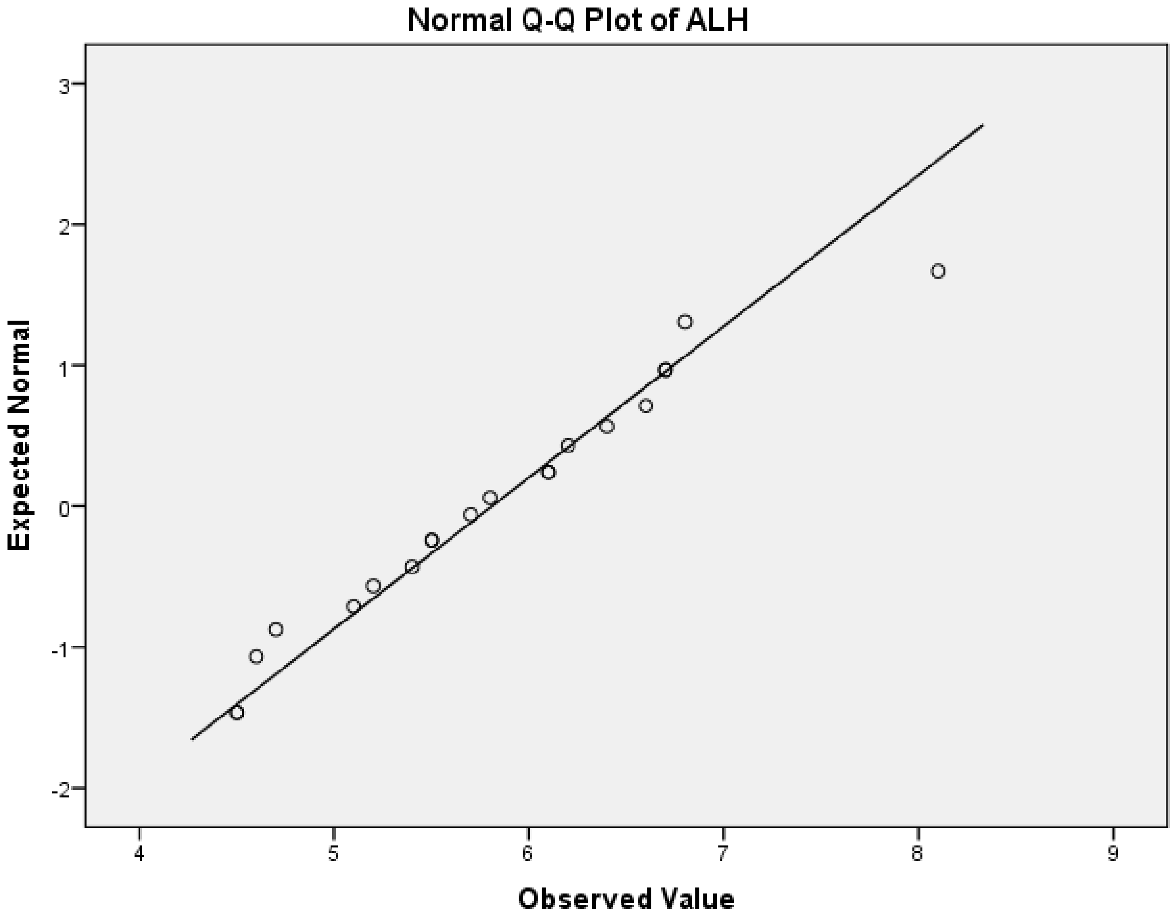
Normal Q-Q Plot of ALH
Expected Normal
Observed Value
3
2
1
0
-1
-2
4
5
6
7
8
9

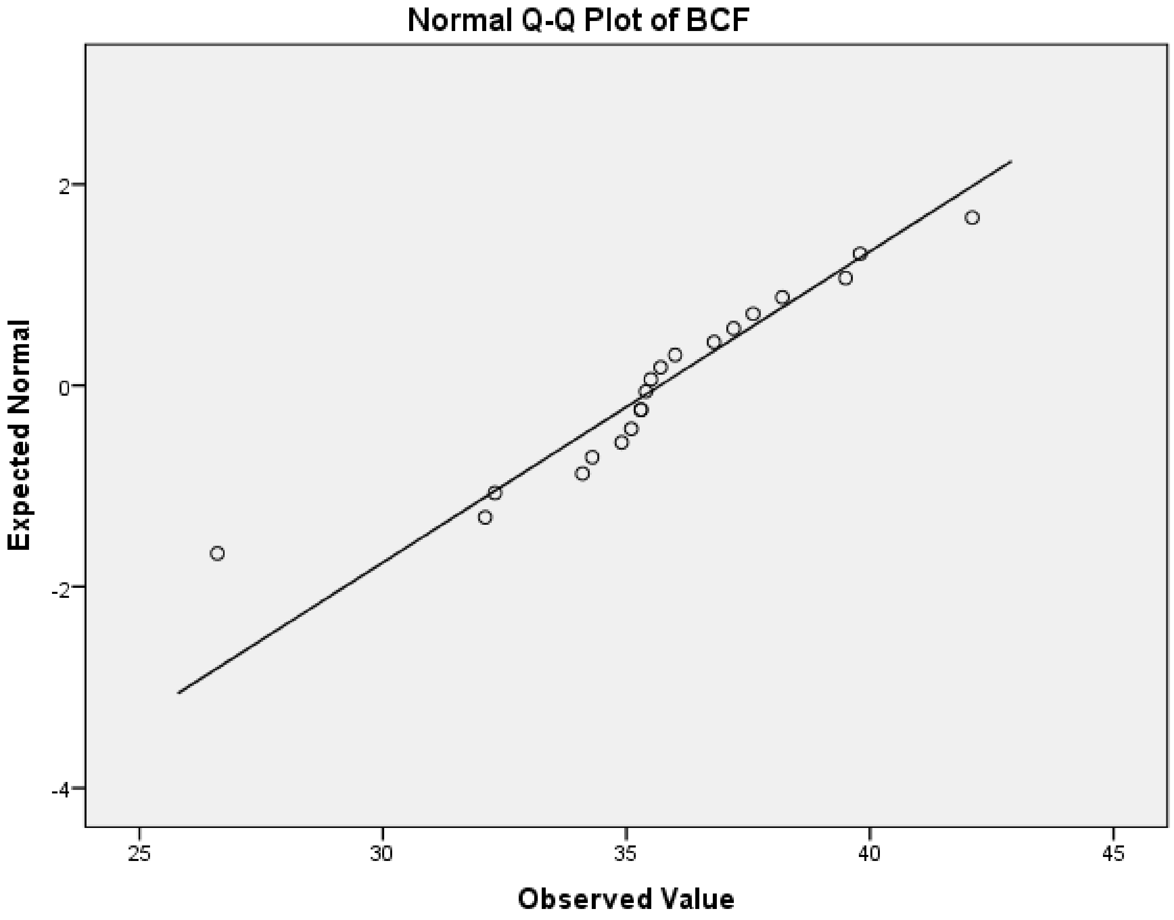

Normal Q-Q Plot of BCF
Expected Normal
2
0
-2
-4
25
30
35
40
45
Observed Value

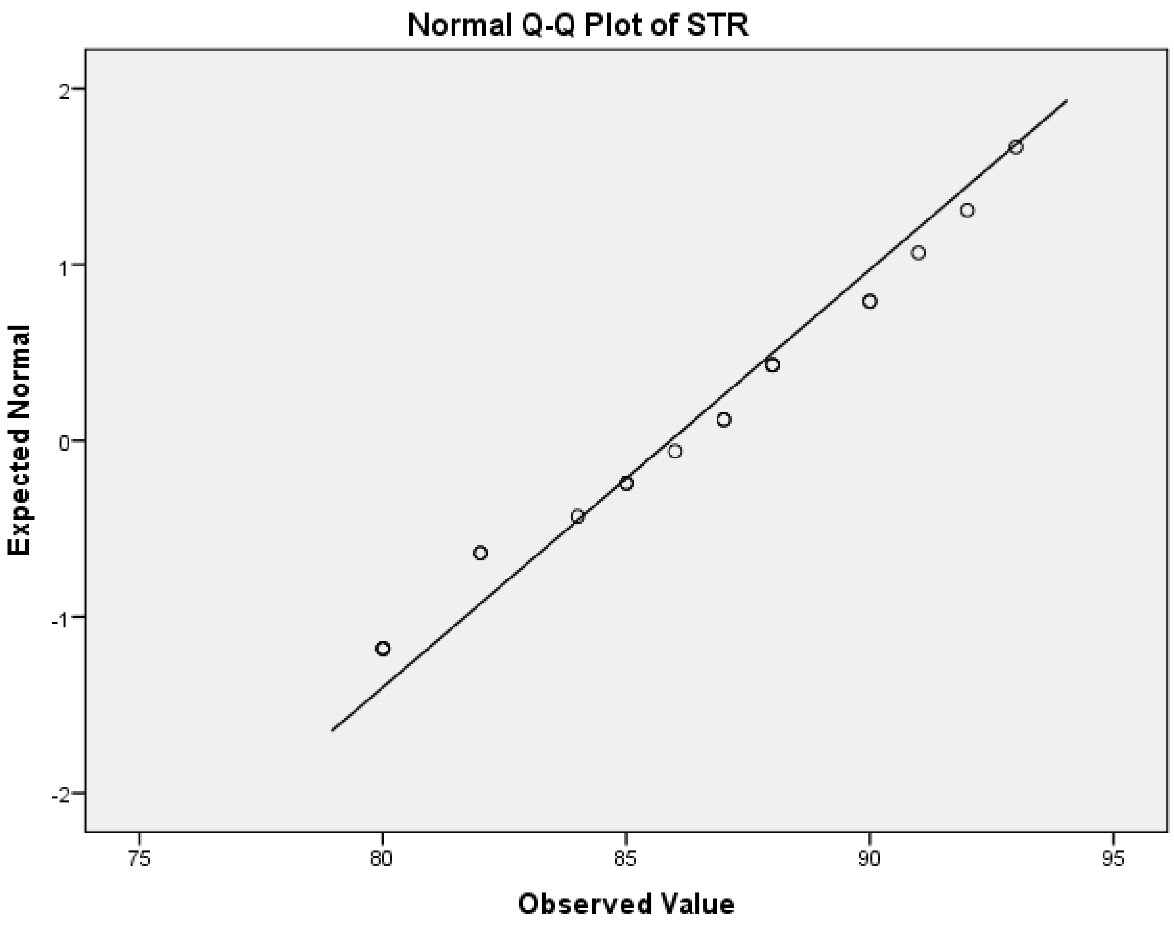

Normal Q-Q Plot of STR
Expected Normal
Observed Value
2
1
0
-1
-2
75
80
85
90
95

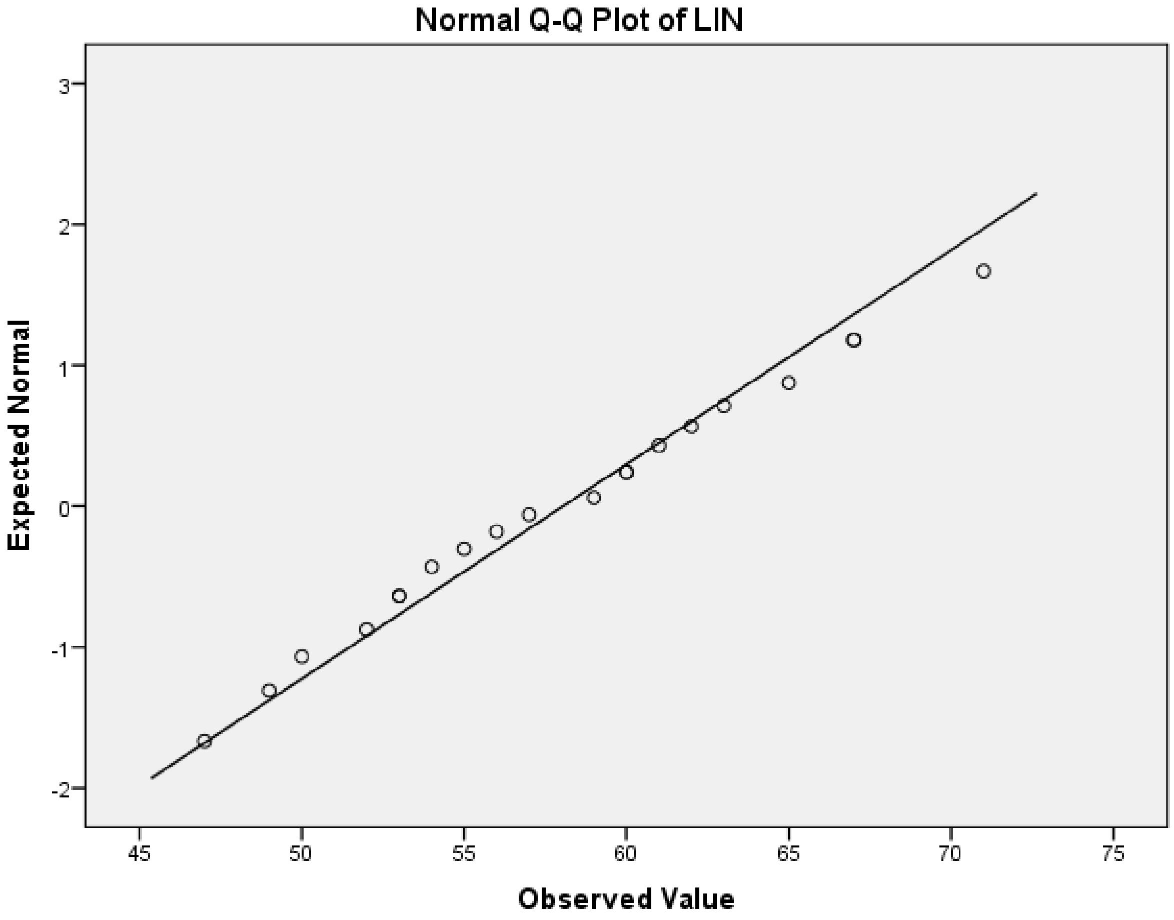

Detrended Normal Q-Q Plots

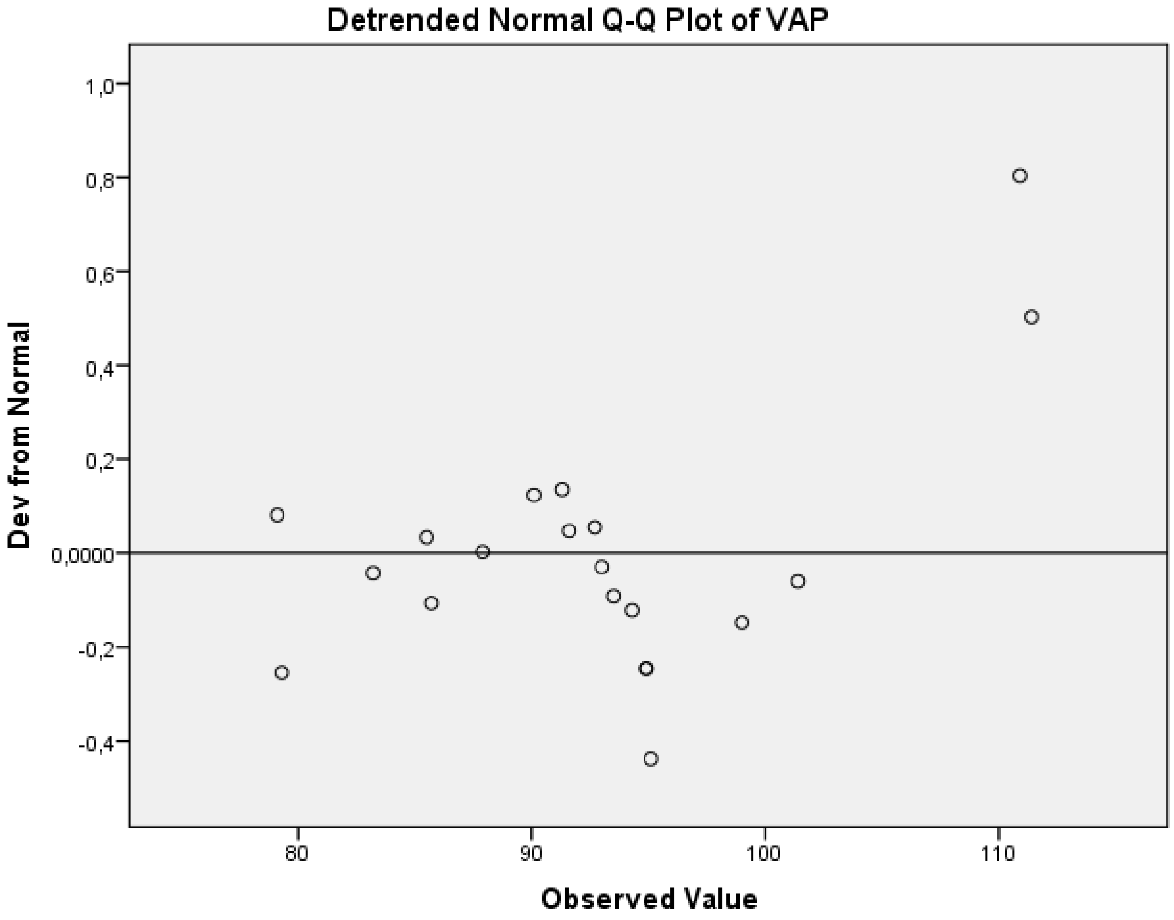

Detrended Normal Q-Q Plot of VAP
Dev from Normal
1,0
0,8
0,6
0,4
0,2
0,0000
-0,2
-0,4
80
90
100
110
Observed Value

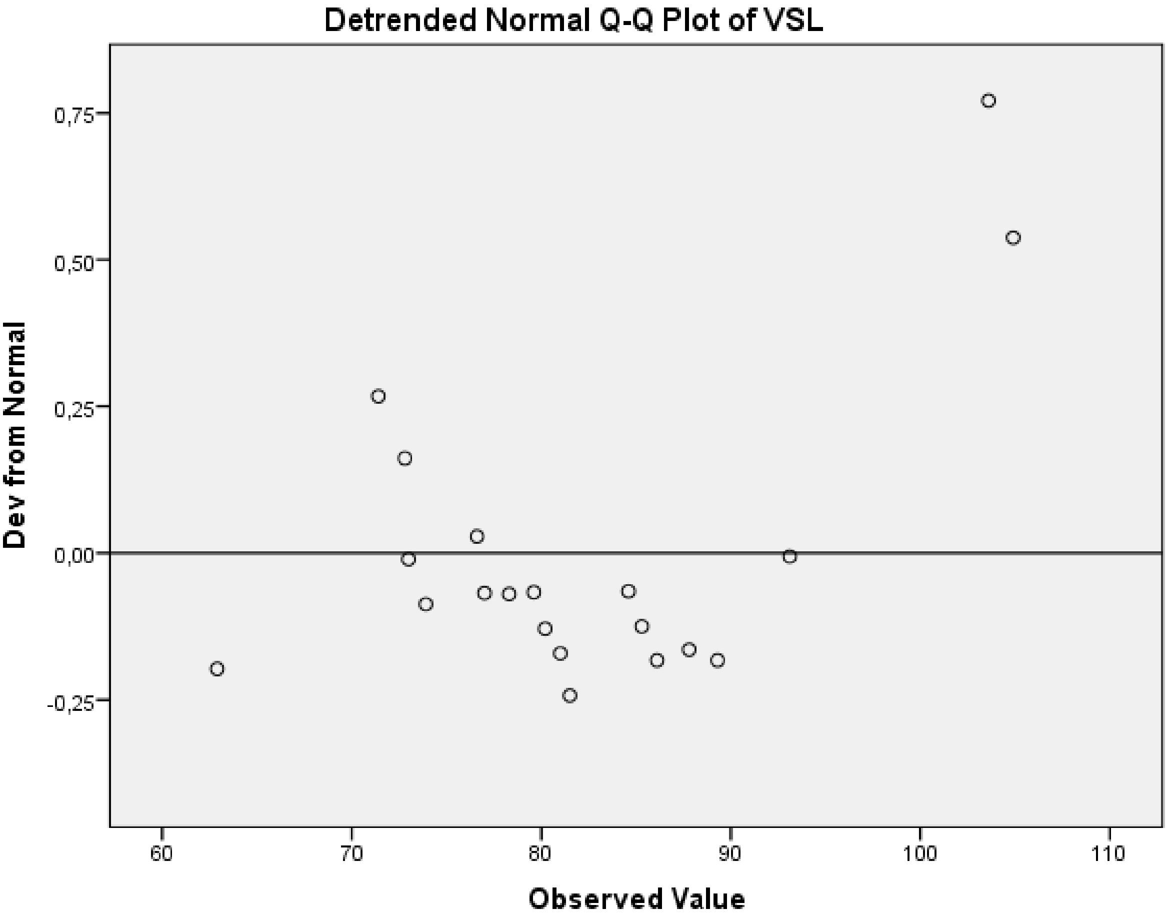
Detrended Normal Q-Q Plot of VSL
Dev from Normal
0,75
0,50
0,25
0,00
-0,25
60
70
80
90
100
110
Observed Value

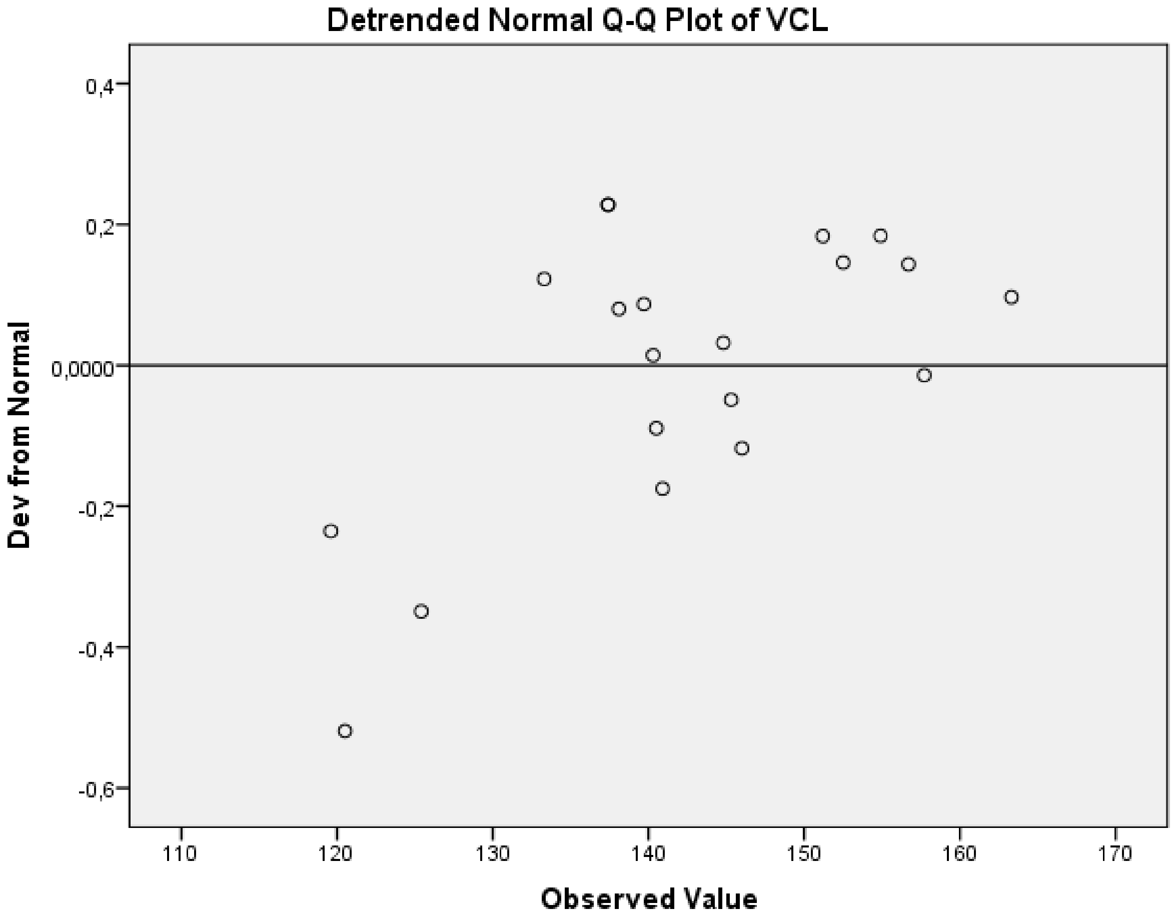

Detrended Normal Q-Q Plot of VCL
0,4
0,2
0,0000
-0,2
-0,4
-0,6
Dev from Normal
110
120
130
140
150
160
170
Observed Value

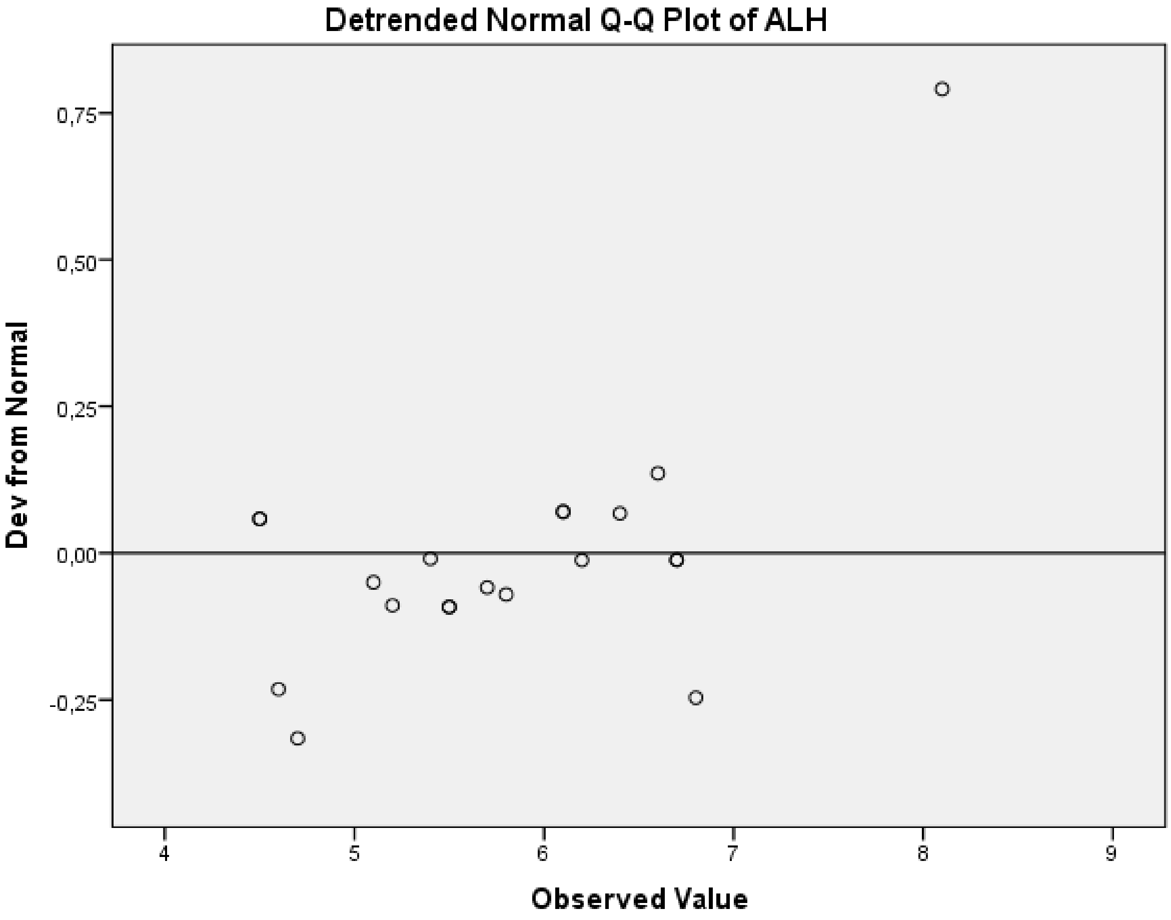
Detrended Normal Q-Q Plot of ALH
0,75
0,50
0,25
0,00
-0,25
Dev from Normal
4
5
6
7
8
9
Observed Value

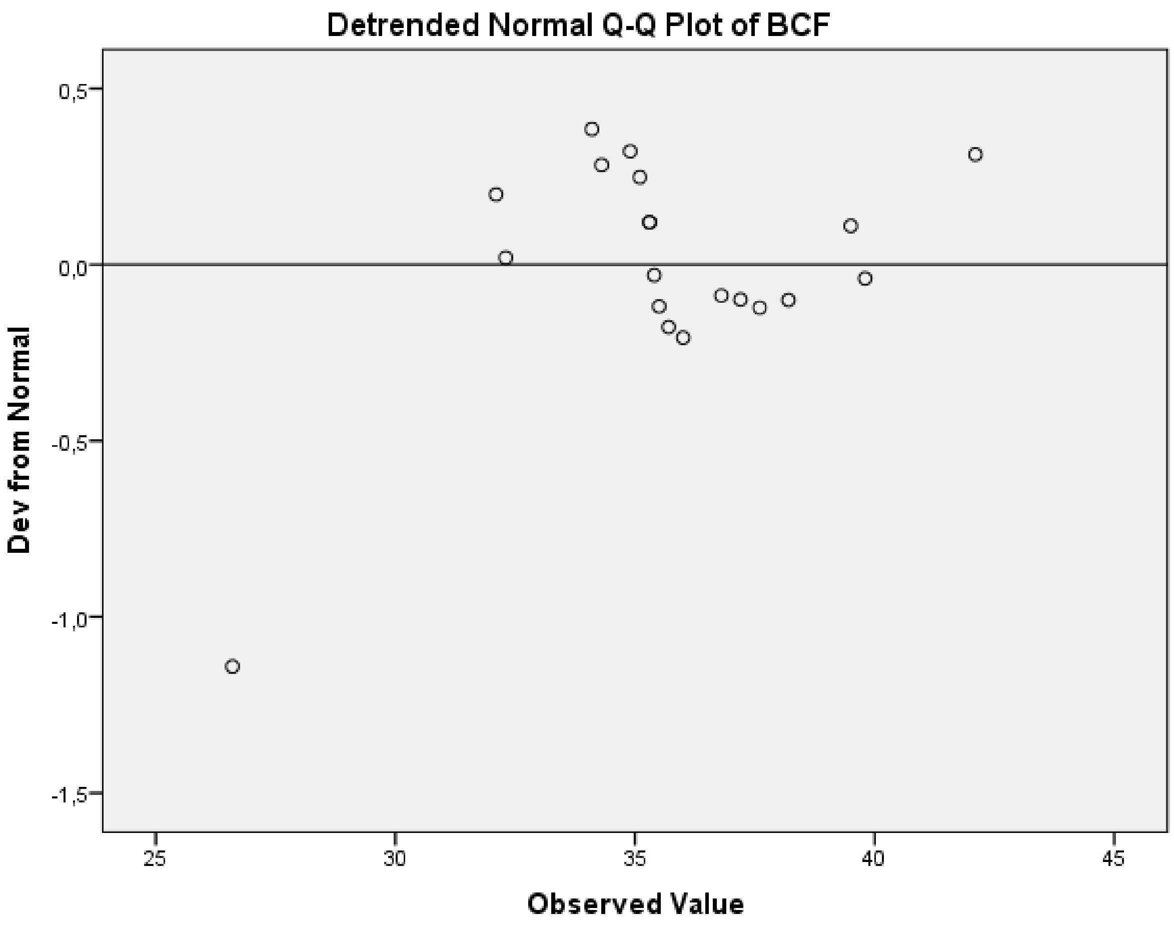

Detrended Normal Q-Q Plot of BCF
Dev from Normal
0,5
0,0
-0,5
-1,0
-1,5
25
30
35
40
45
Observed Value

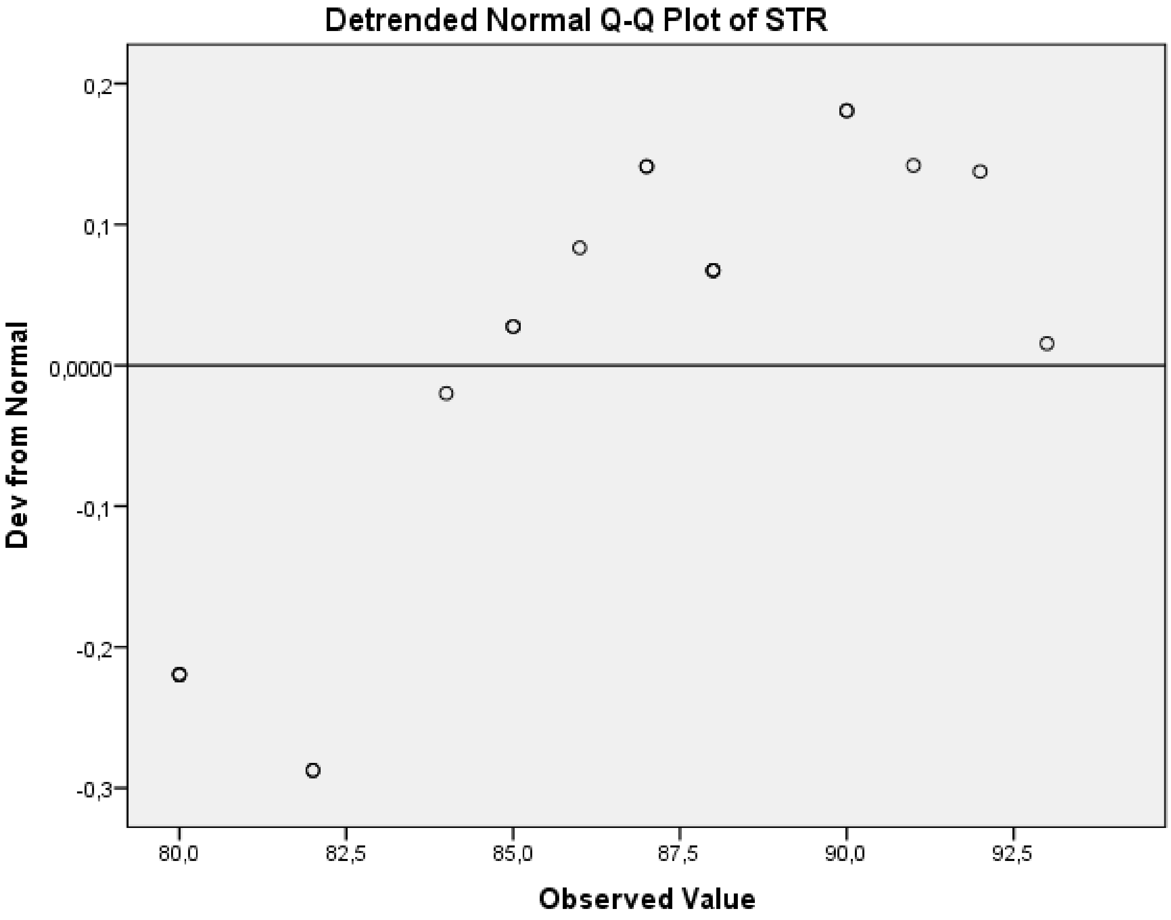
Detrended Normal Q-Q Plot of STR
Dev from Normal
0,2
0,1
0,0000
-0,1
-0,2
-0,3
80,0
82,5
85,0
87,5
90,0
92,5
Observed Value

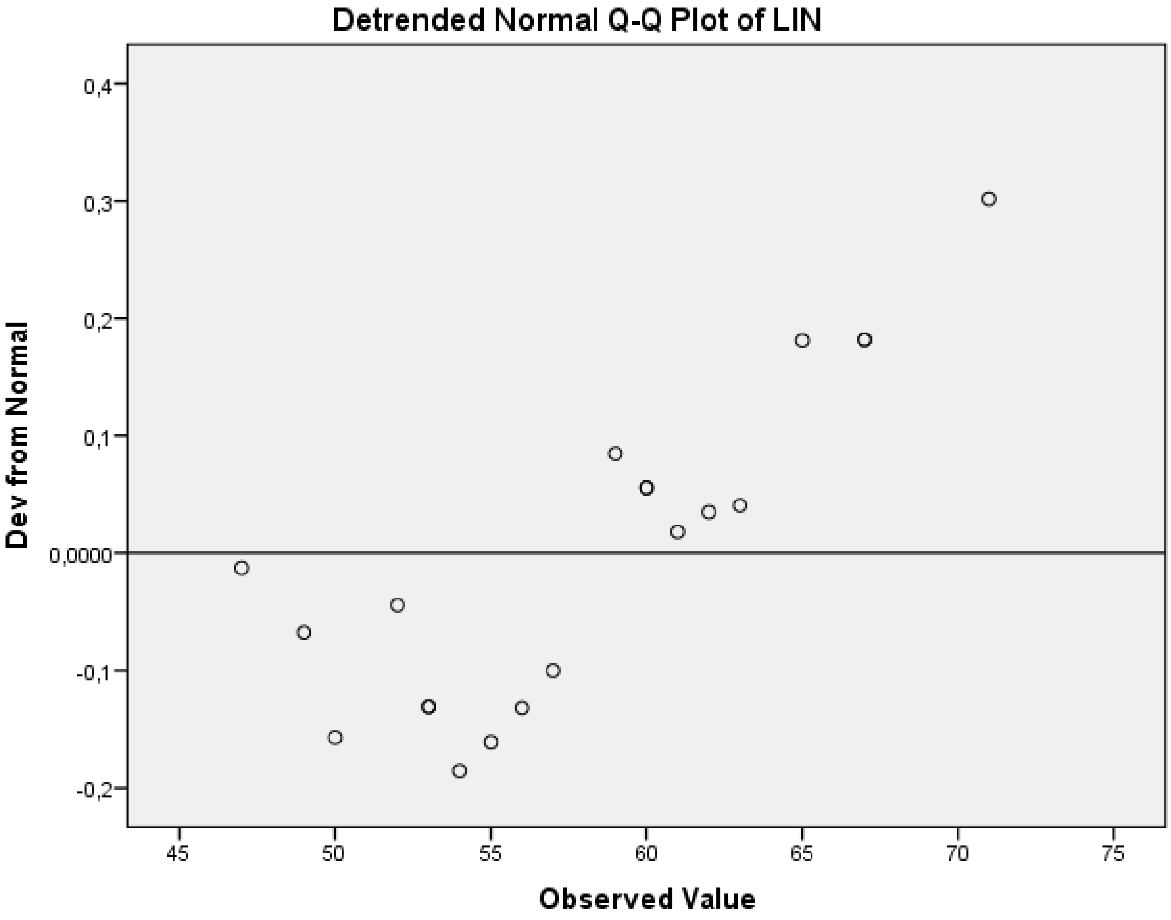

Gruplar = 4,00

Histograms

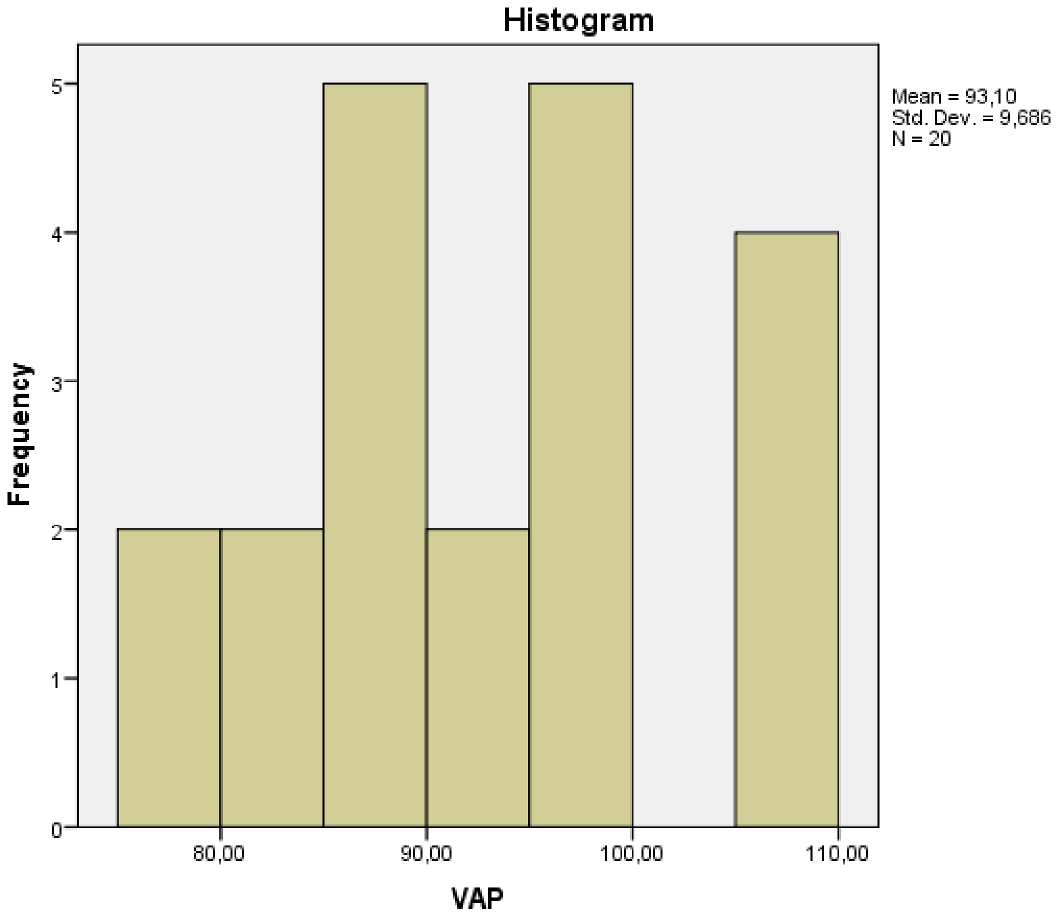
Histogram
Frequency
VAP
Mean = 93,10
Std. Dev. = 9,686
N = 20
0
1
2
3
4
5
80,00
90,00
100,00
110,00

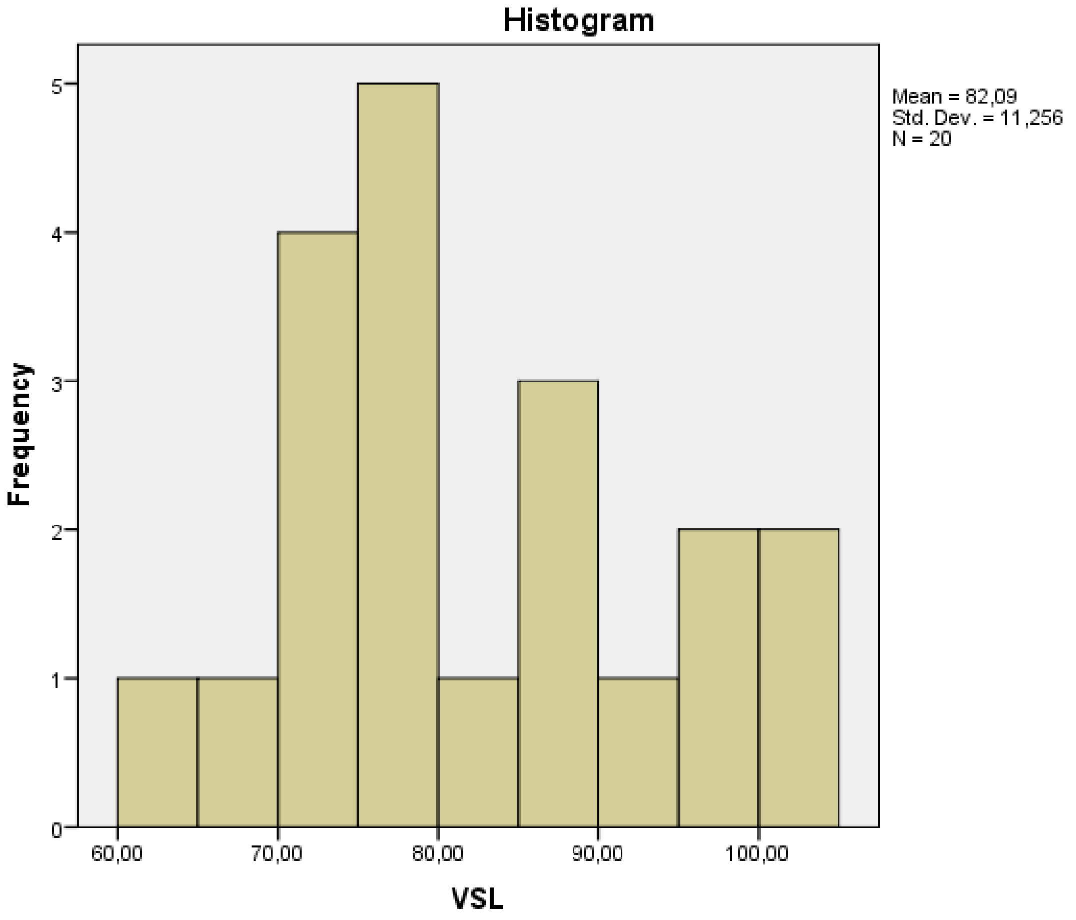
Histogram
Mean = 82,09
Std. Dev. = 11,256
N = 20
Frequency
5
4
3
2
1
0
60,00
70,00
80,00
90,00
100,00
VSL

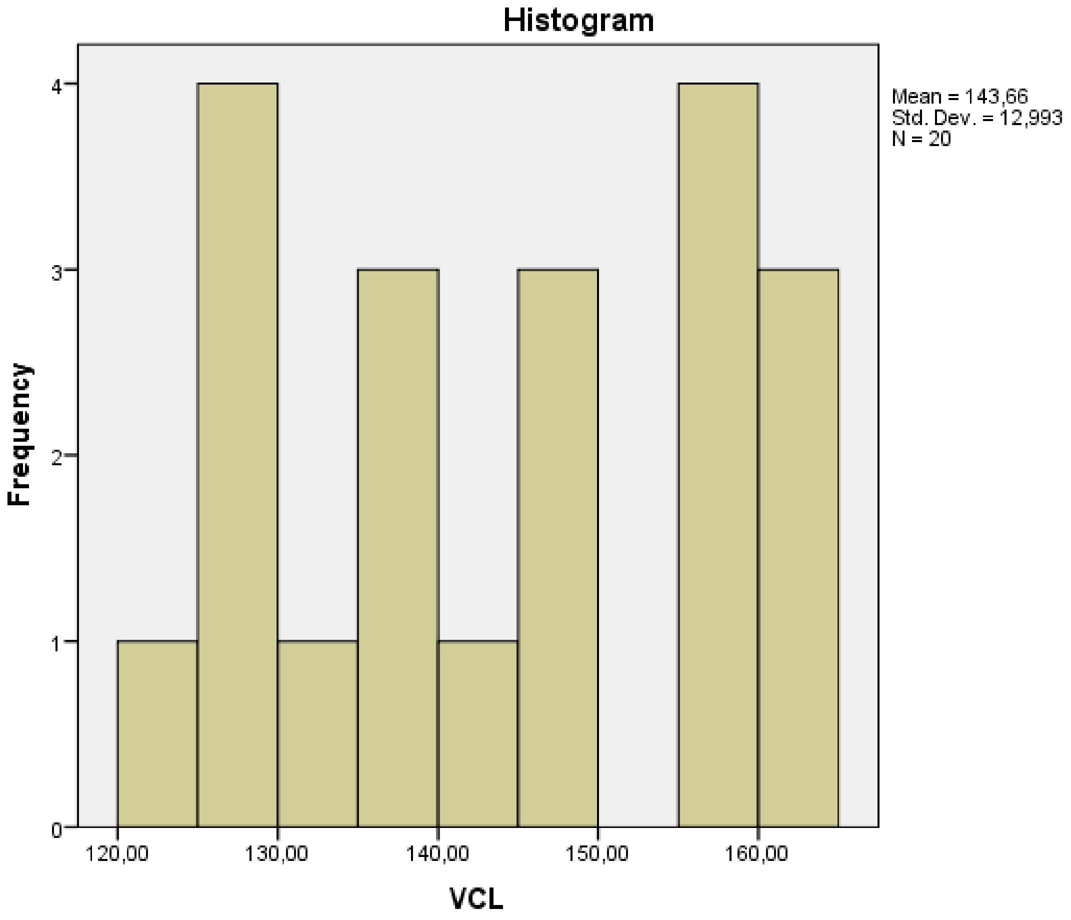
Histogram
Mean = 143,66
Std. Dev. = 12,993
N = 20
Frequency
0
1
2
3
4
120,00
130,00
140,00
150,00
160,00
VCL

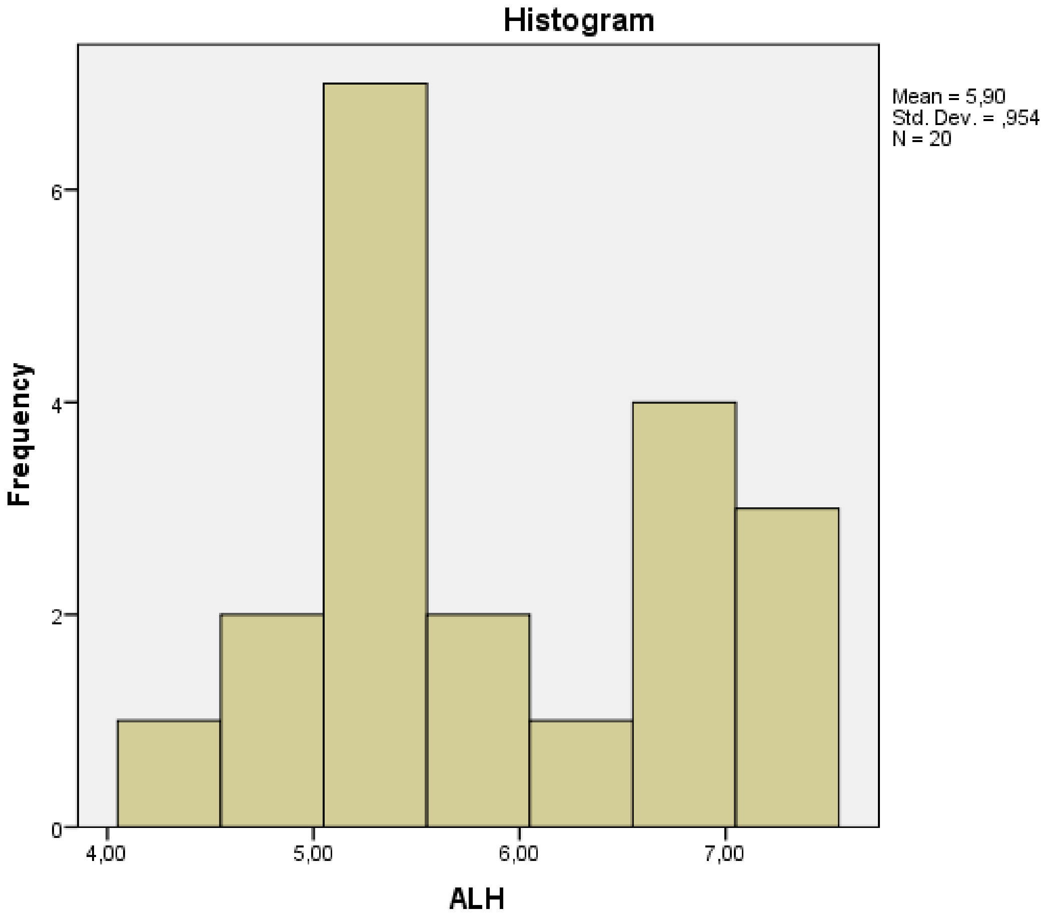
Histogram
Mean = 5,90
Std. Dev. = ,954
N = 20
Frequency
0
2
4
6
4,00
5,00
6,00
7,00
ALH

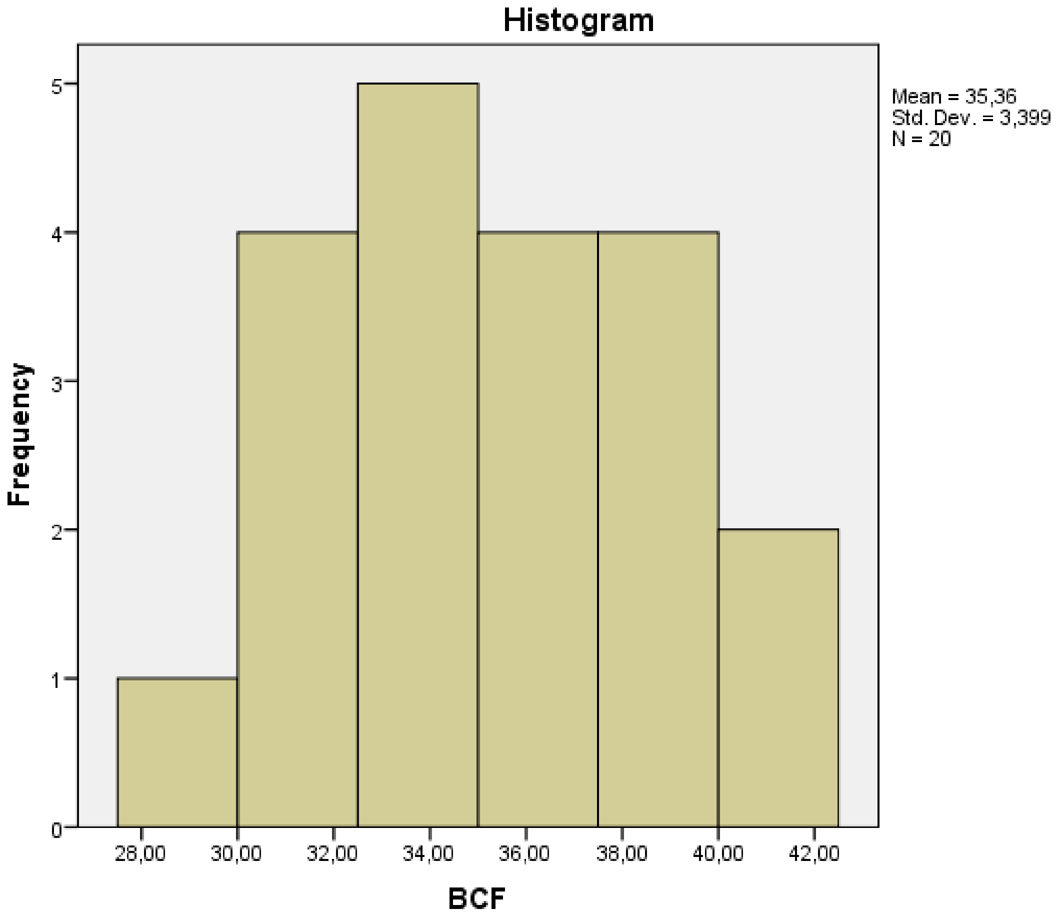
Histogram
Mean = 35,36
Std. Dev. = 3,399
N = 20
Frequency
0
1
2
3
4
5
28,00
30,00
32,00
34,00
36,00
38,00
40,00
42,00
BCF

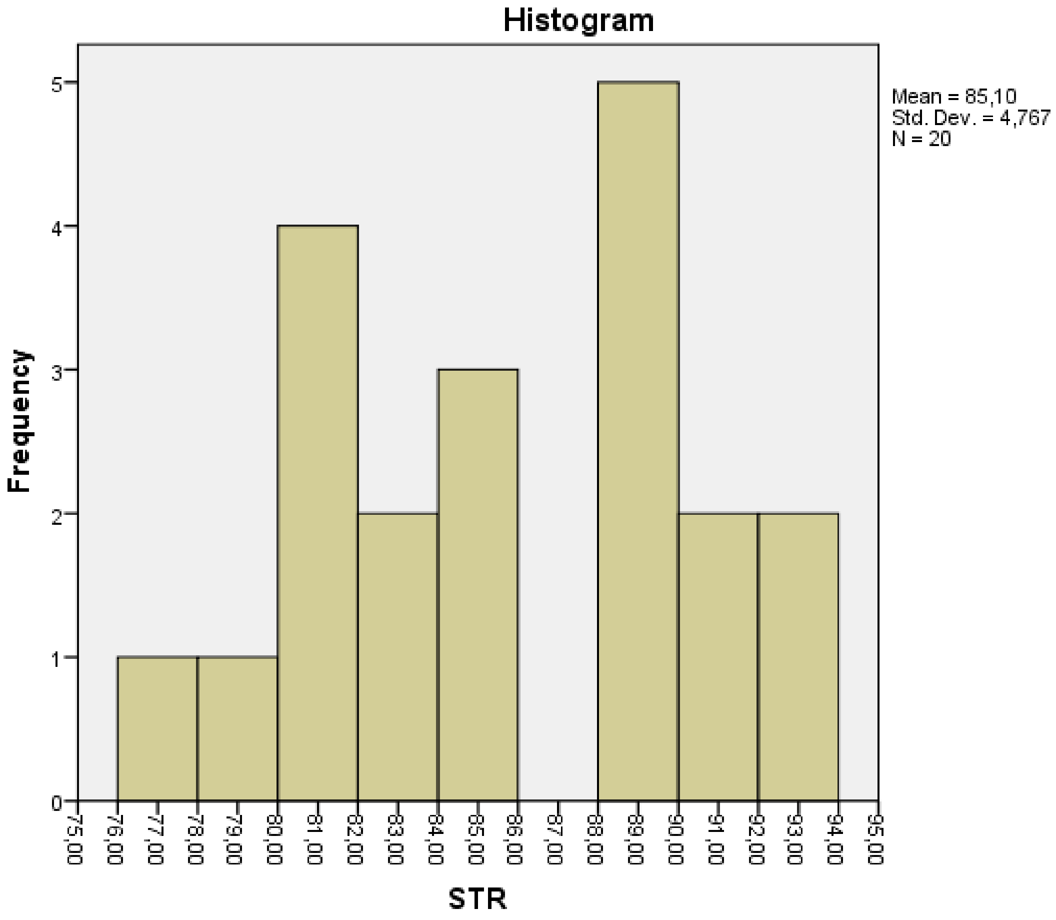
Histogram
Mean = 85,10
Std. Dev. = 4,767
N = 20
Frequency
0
1
2
3
4
5
75,00
76,00
77,00
78,00
79,00
80,00
81,00
82,00
83,00
84,00
85,00
86,00
87,00
88,00
89,00
90,00
91,00
92,00
93,00
94,00
95,00
STR

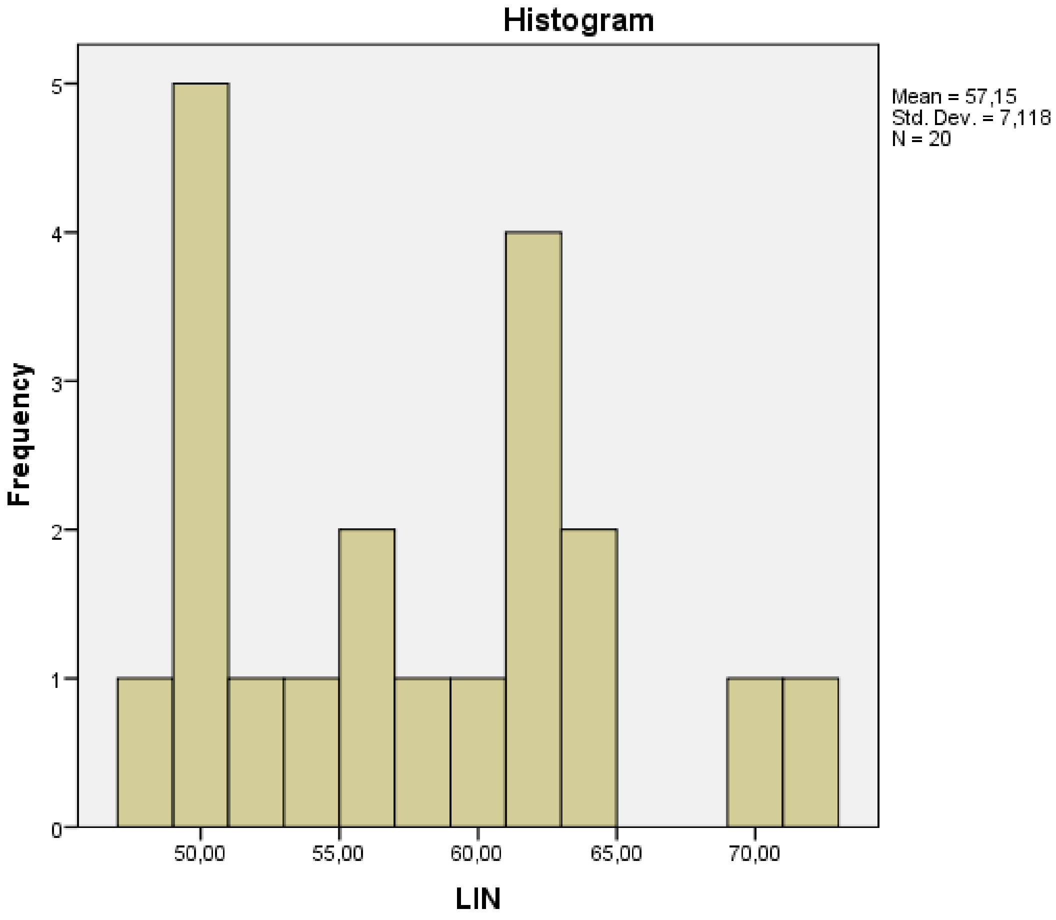

Stem-and-Leaf Plots

```
VAP Stem-and-Leaf Plot for
Gruplar= 4,00

 Frequency    Stem &  Leaf

     2,00        7 .  59
     2,00        8 .  34
     5,00        8 .  56777
     2,00        9 .  34
     5,00        9 .  56699
      ,00       10 .
     4,00       10 .  5689

 Stem width:  10,00
 Each leaf:       1 case(s)
```

```
VSL Stem-and-Leaf Plot for
Gruplar= 4,00

 Frequency    Stem &  Leaf

     2,00        6 .  27
     9,00        7 .  002367999
     4,00        8 .  4667
     3,00        9 .  368
     2,00       10 .  00

 Stem width:  10,00
 Each leaf:       1 case(s)
```

```
VCL Stem-and-Leaf Plot for
Gruplar= 4,00

 Frequency    Stem &  Leaf

     5,00       12 .  35899
     4,00       13 .  4579
     4,00       14 .  0559
     4,00       15 .  6667
     3,00       16 .  002

 Stem width:  10,00
 Each leaf:       1 case(s)
```

```
ALH Stem-and-Leaf Plot for
Gruplar= 4,00

 Frequency    Stem &  Leaf

     1,00        4 .  3
     1,00        4 .  6
     7,00        5 .  0123444
     3,00        5 .  567
     1,00        6 .  2
     3,00        6 .  789
     3,00        7 .  022
     1,00        7 .  5

 Stem width:   1,00
 Each leaf:       1 case(s)
```

```
BCF Stem-and-Leaf Plot for
Gruplar= 4,00

 Frequency    Stem &  Leaf

     1,00        2 .  8
     9,00        3 .  112233344
     8,00        3 .  66678888
     2,00        4 .  02

 Stem width:  10,00
 Each leaf:       1 case(s)
```

```
STR Stem-and-Leaf Plot for
Gruplar= 4,00

 Frequency    Stem &  Leaf

     2,00        7 .  78
     7,00        8 .  0001234
     7,00        8 .  5588999
     4,00        9 .  0022

 Stem width:  10,00
 Each leaf:       1 case(s)
```

```
LIN Stem-and-Leaf Plot for
Gruplar= 4,00

 Frequency    Stem &  Leaf

     4,00        4 .  8999
     4,00        5 .  0023
     4,00        5 .  6679
     6,00        6 .  122234
     1,00        6 .  9
     1,00        7 .  2

 Stem width:  10,00
 Each leaf:       1 case(s)
```

Normal Q-Q Plots

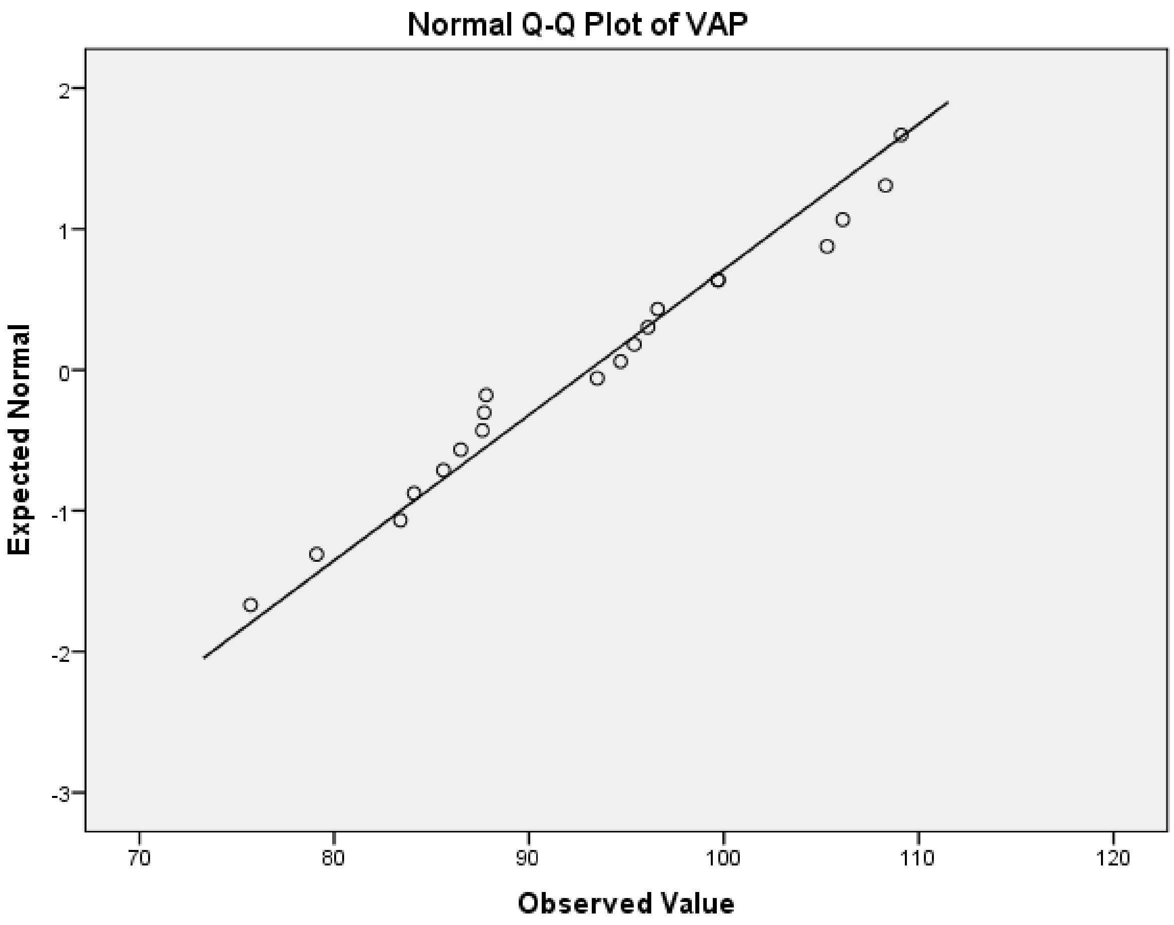
Normal Q-Q Plot of VAP
Expected Normal
Observed Value
2
1
0
-1
-2
-3
70
80
90
100
110
120

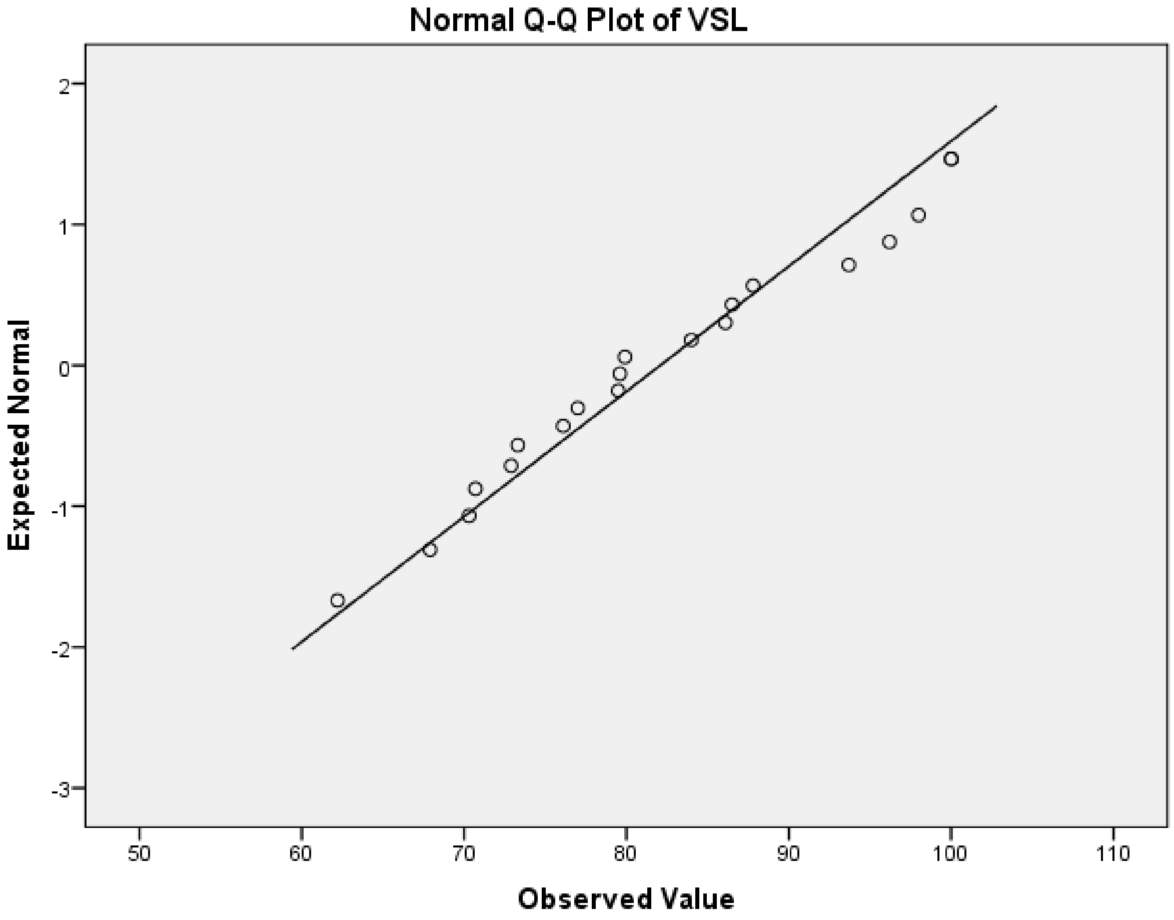
Normal Q-Q Plot of VSL
Expected Normal
Observed Value
2
1
0
-1
-2
-3
50
60
70
80
90
100
110

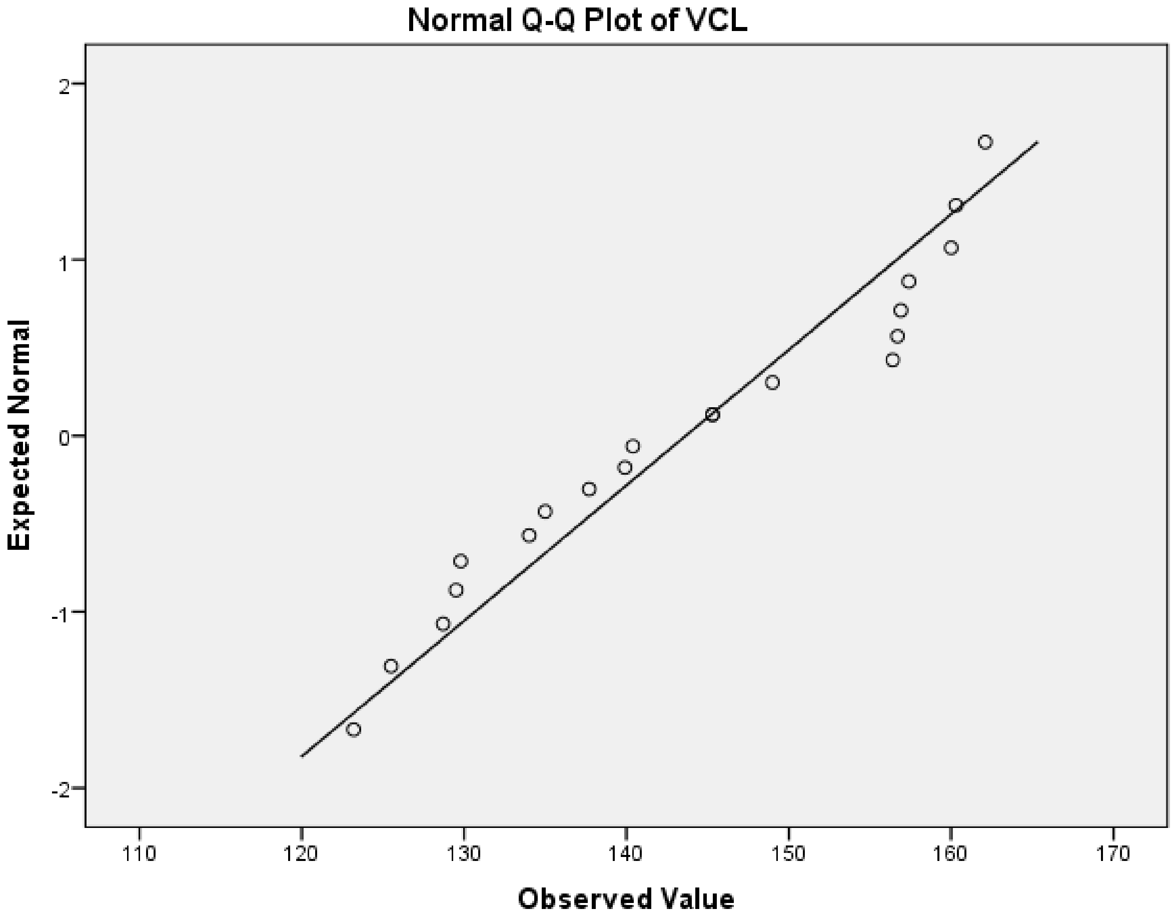
Normal Q-Q Plot of VCL
Expected Normal
2
1
0
-1
-2
110
120
130
140
150
160
170
Observed Value

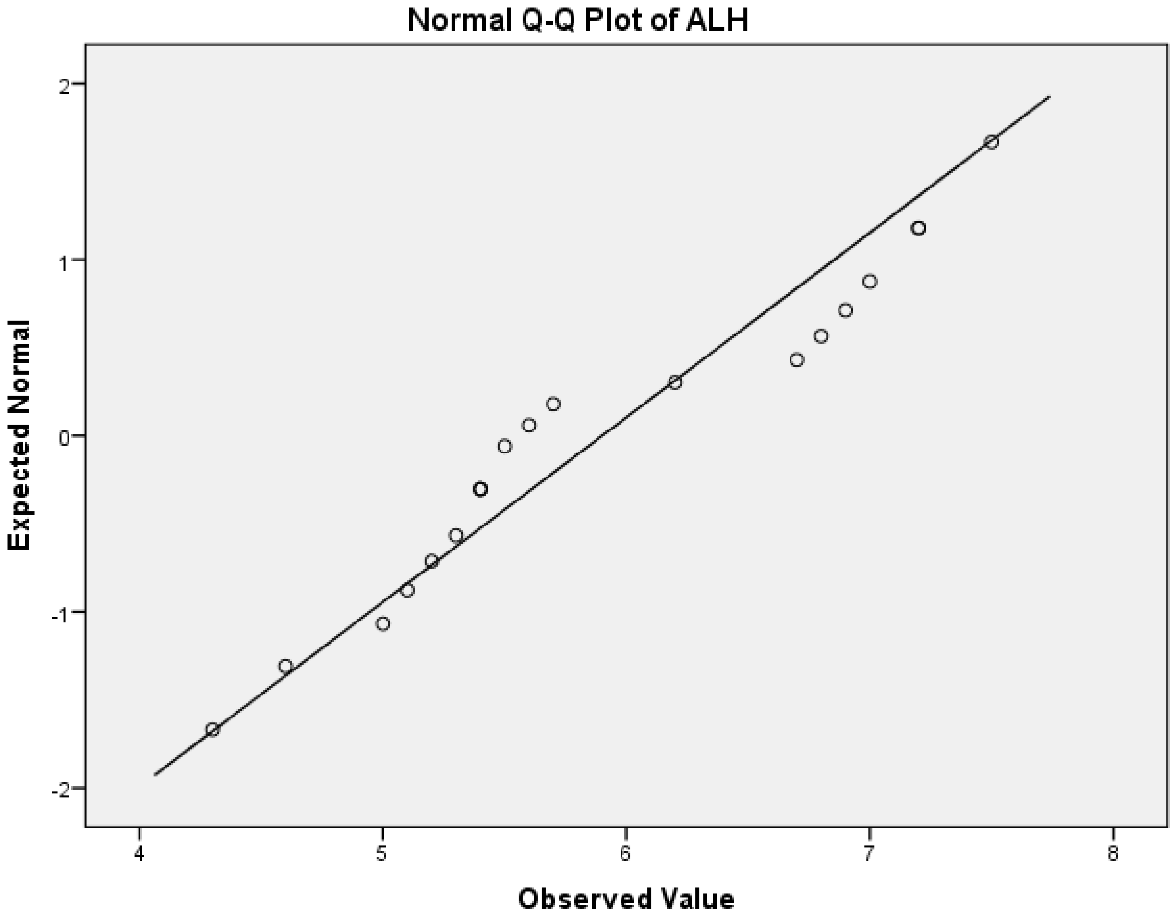
Normal Q-Q Plot of ALH
Expected Normal
2
1
0
-1
-2
4
5
6
7
8
Observed Value

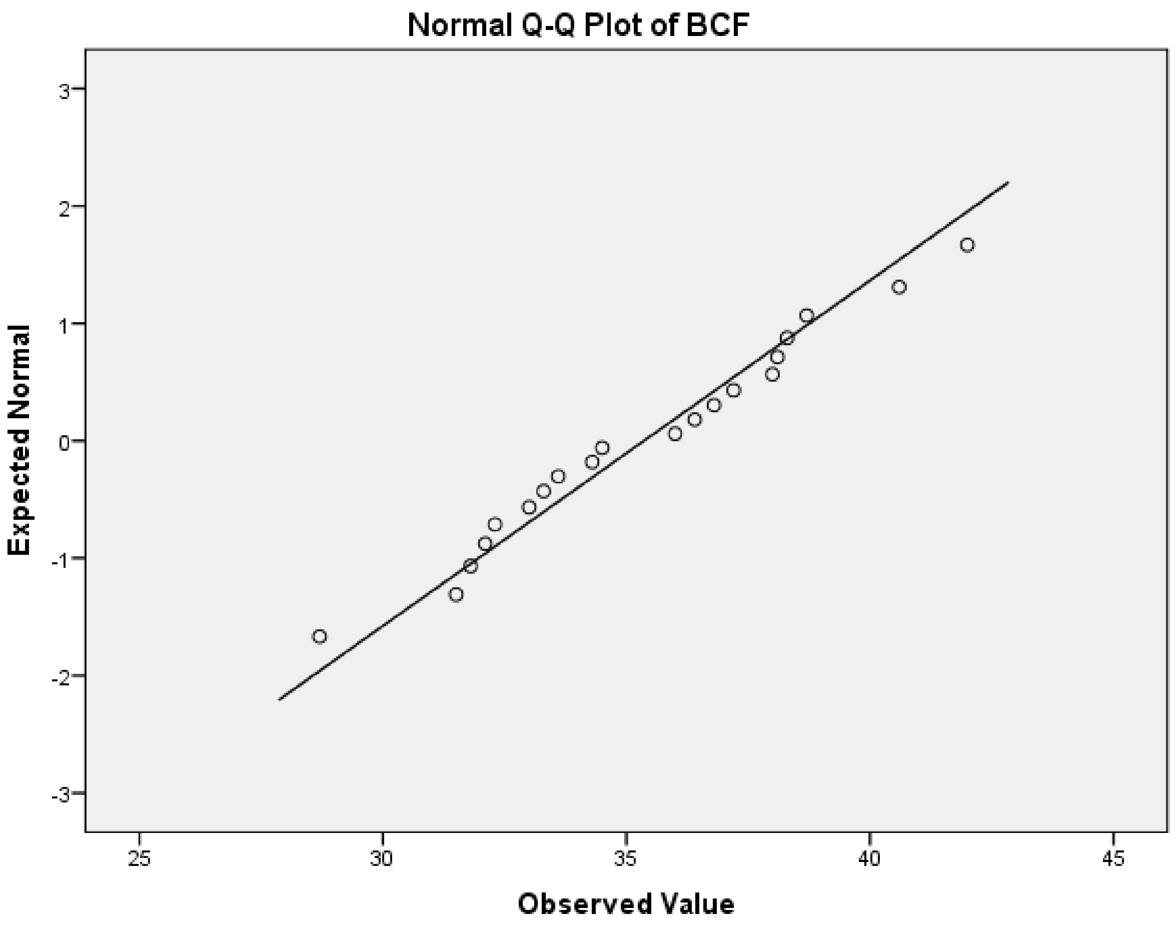

Normal Q-Q Plot of BCF
Expected Normal
Observed Value
3
2
1
0
-1
-2
-3
25
30
35
40
45

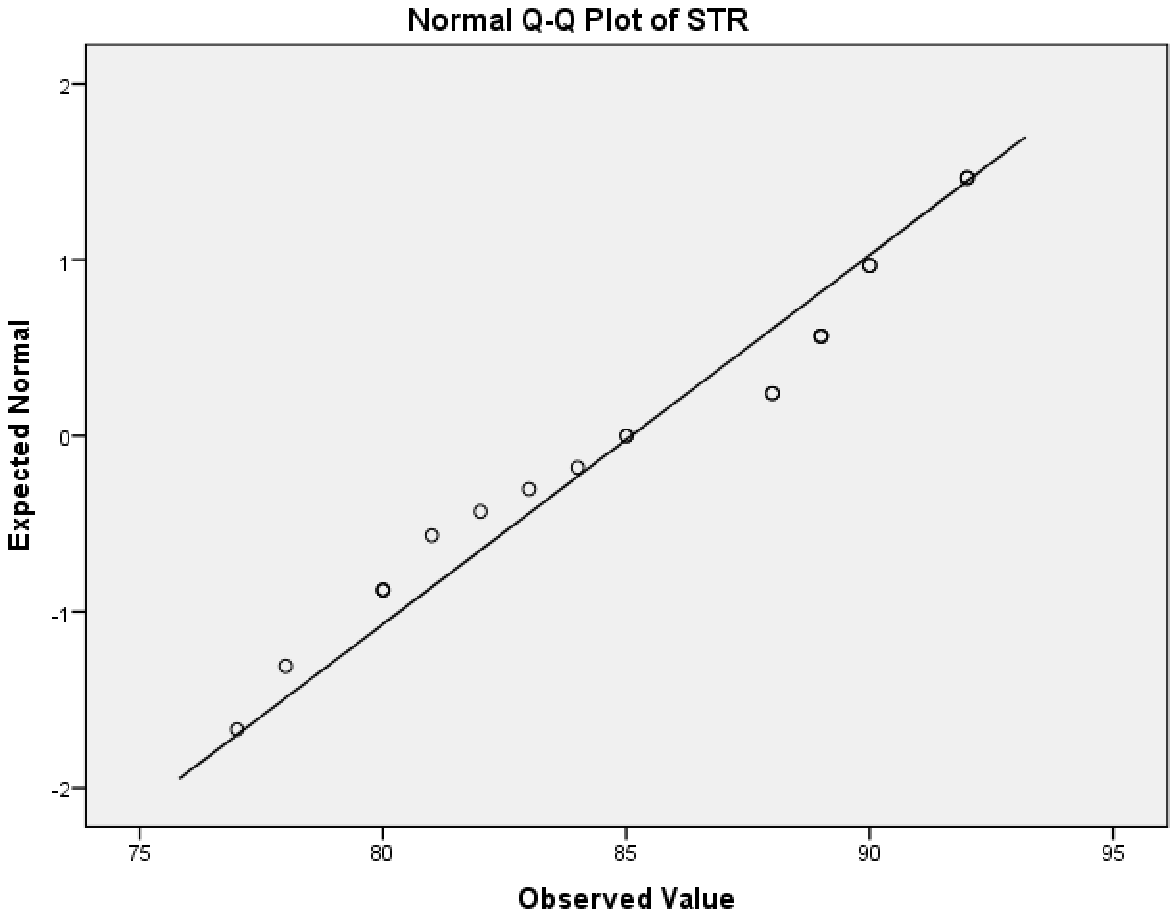
Normal Q-Q Plot of STR
Expected Normal
2
1
0
-1
-2
75
80
85
90
95
Observed Value

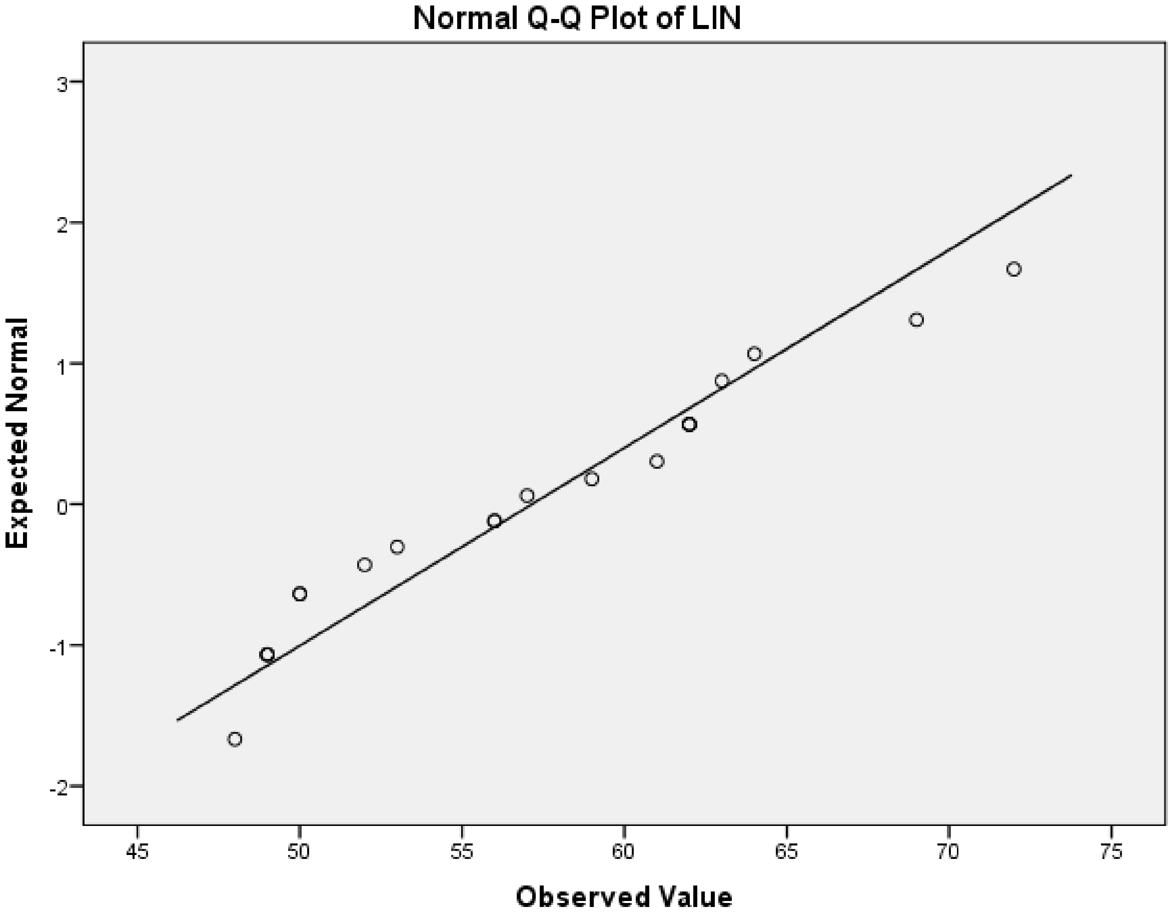

Detrended Normal Q-Q Plots

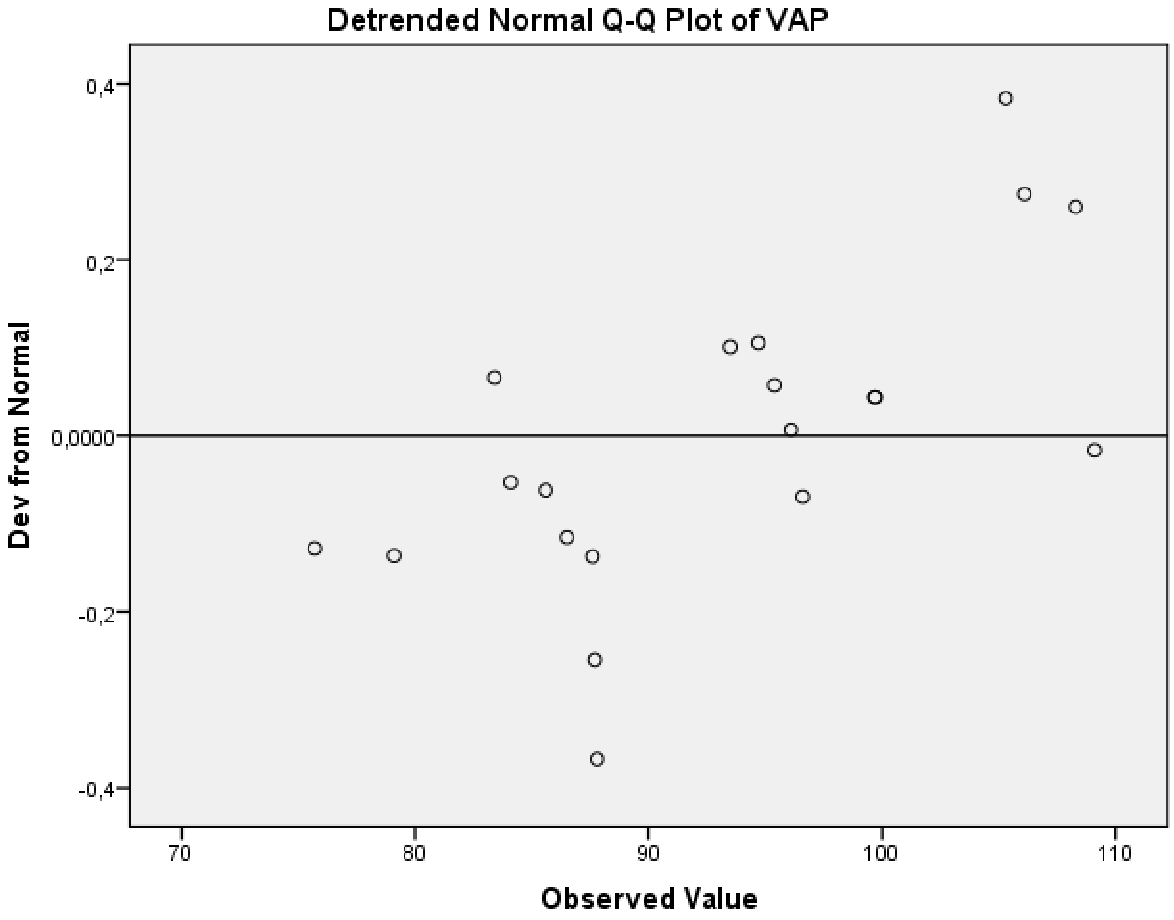
Detrended Normal Q-Q Plot of VAP
Dev from Normal
0,4
0,2
0,0000
-0,2
-0,4
70
80
90
100
110
Observed Value

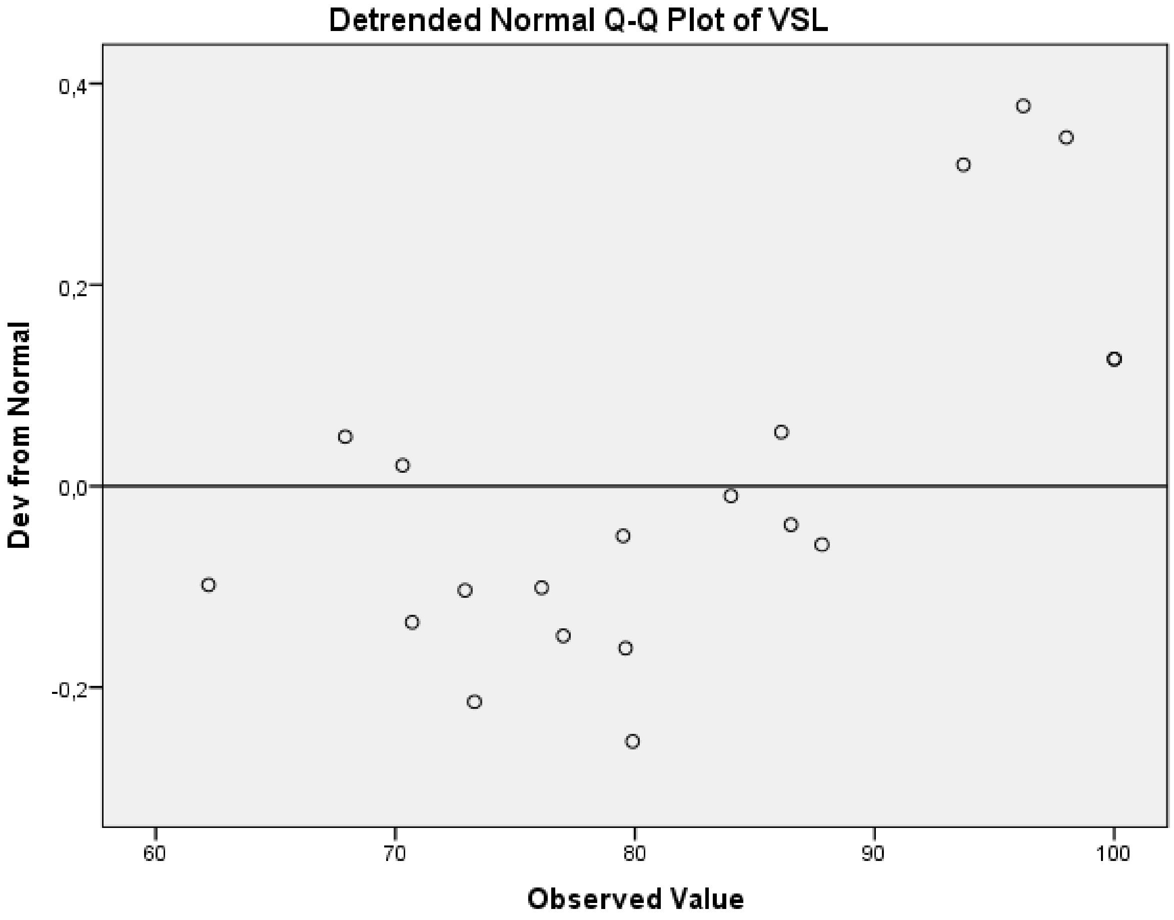

Detrended Normal Q-Q Plot of VSL
Dev from Normal
0,4
0,2
0,0
-0,2
60
70
80
90
100
Observed Value

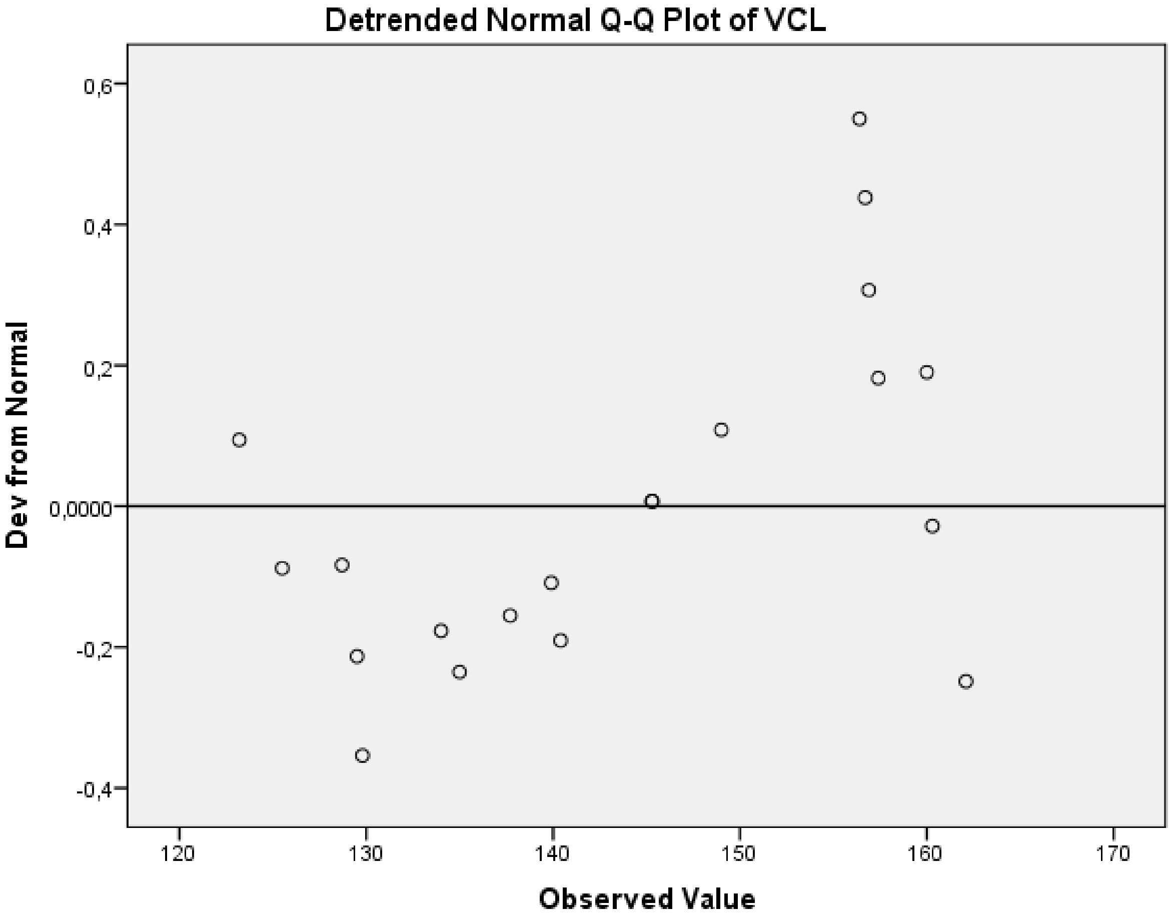
Detrended Normal Q-Q Plot of VCL
Dev from Normal
0,6
0,4
0,2
0,0000
-0,2
-0,4
120
130
140
150
160
170
Observed Value

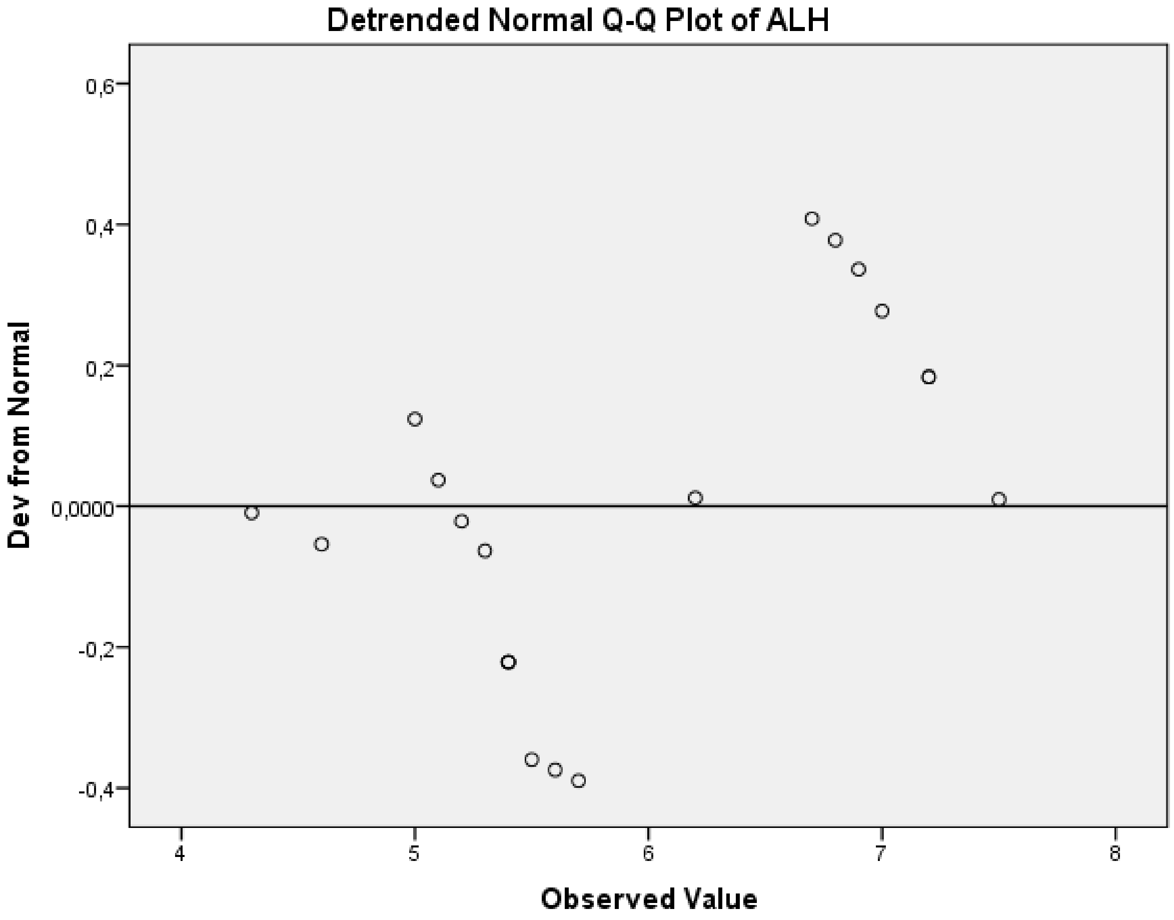

Detrended Normal Q-Q Plot of ALH
0,6
0,4
0,2
0,0000
-0,2
-0,4
Dev from Normal
4
5
6
7
8
Observed Value

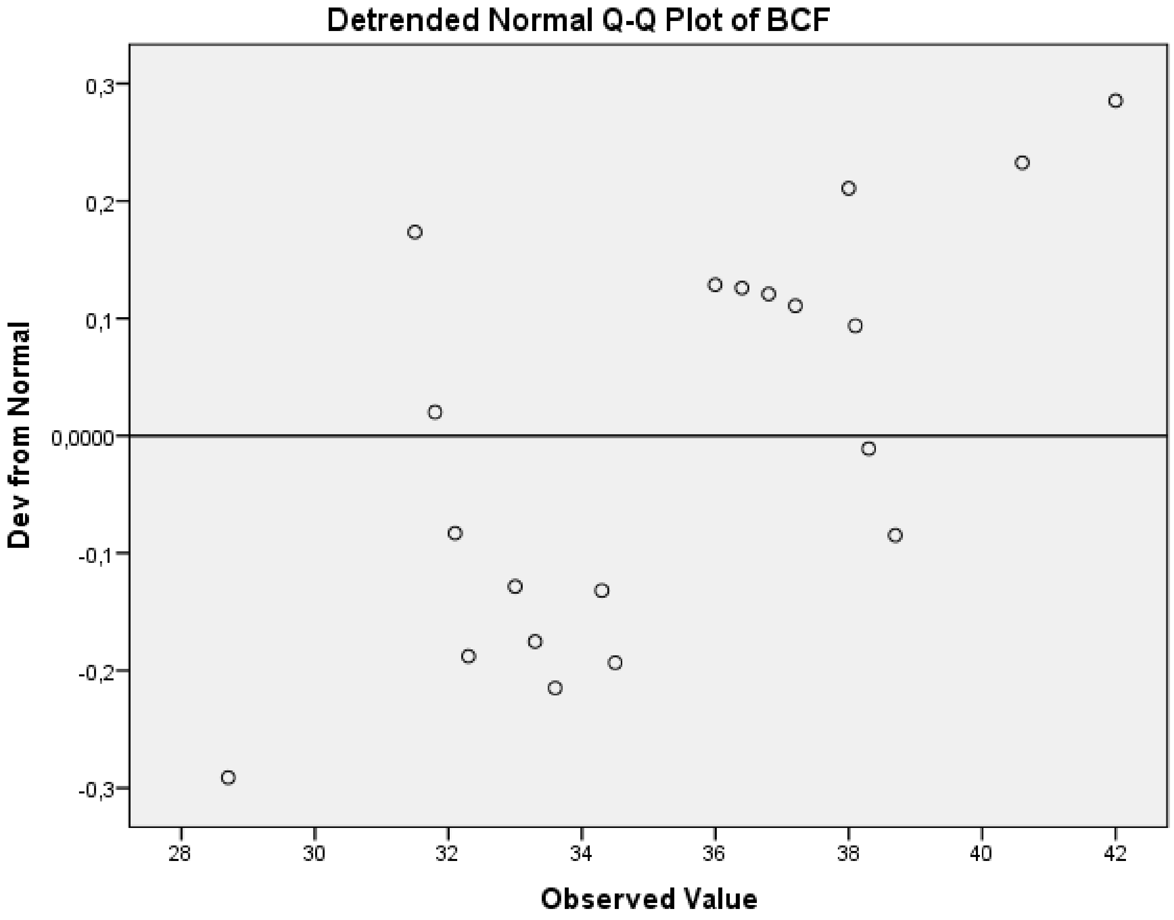

Detrended Normal Q-Q Plot of BCF
Dev from Normal
0,3
0,2
0,1
0,0000
-0,1
-0,2
-0,3
28
30
32
34
36
38
40
42
Observed Value

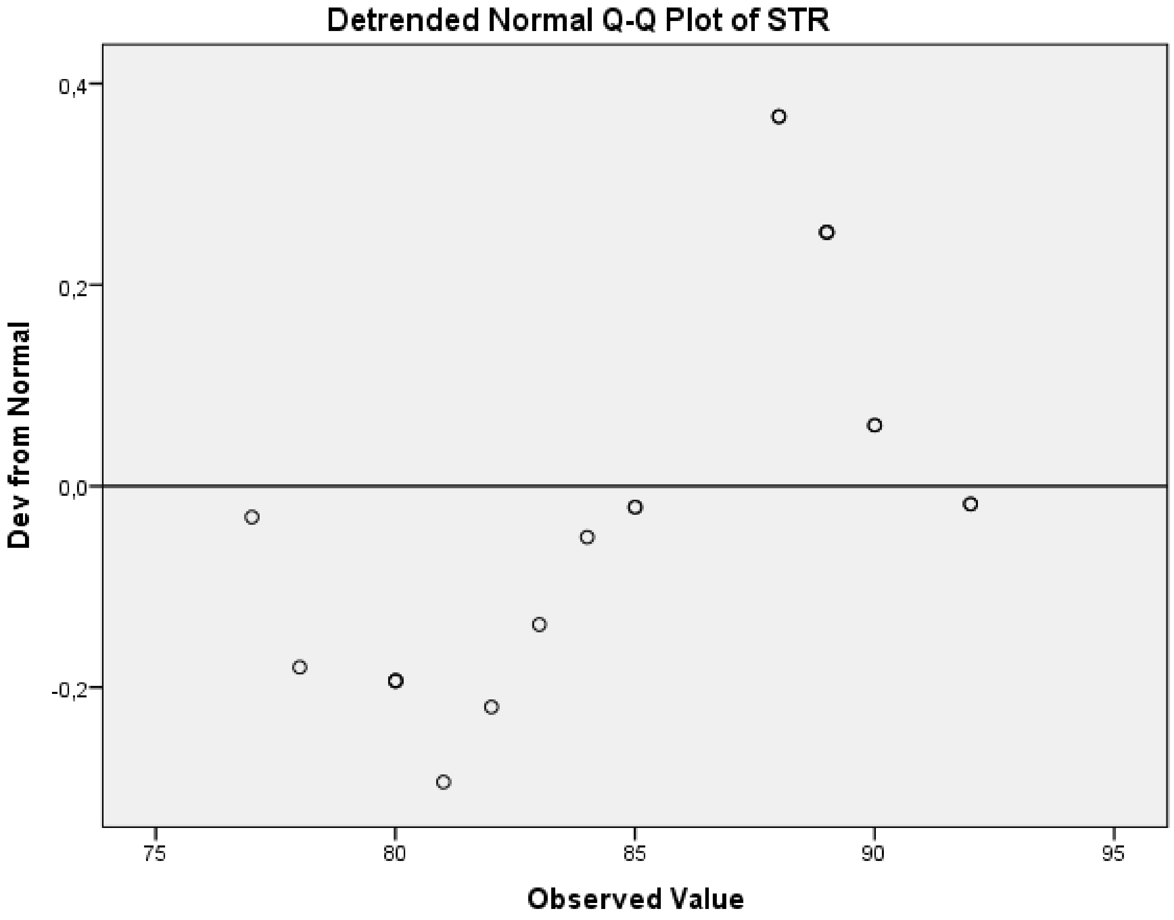

Detrended Normal Q-Q Plot of STR
0,4
0,2
0,0
-0,2
Dev from Normal
75
80
85
90
95
Observed Value

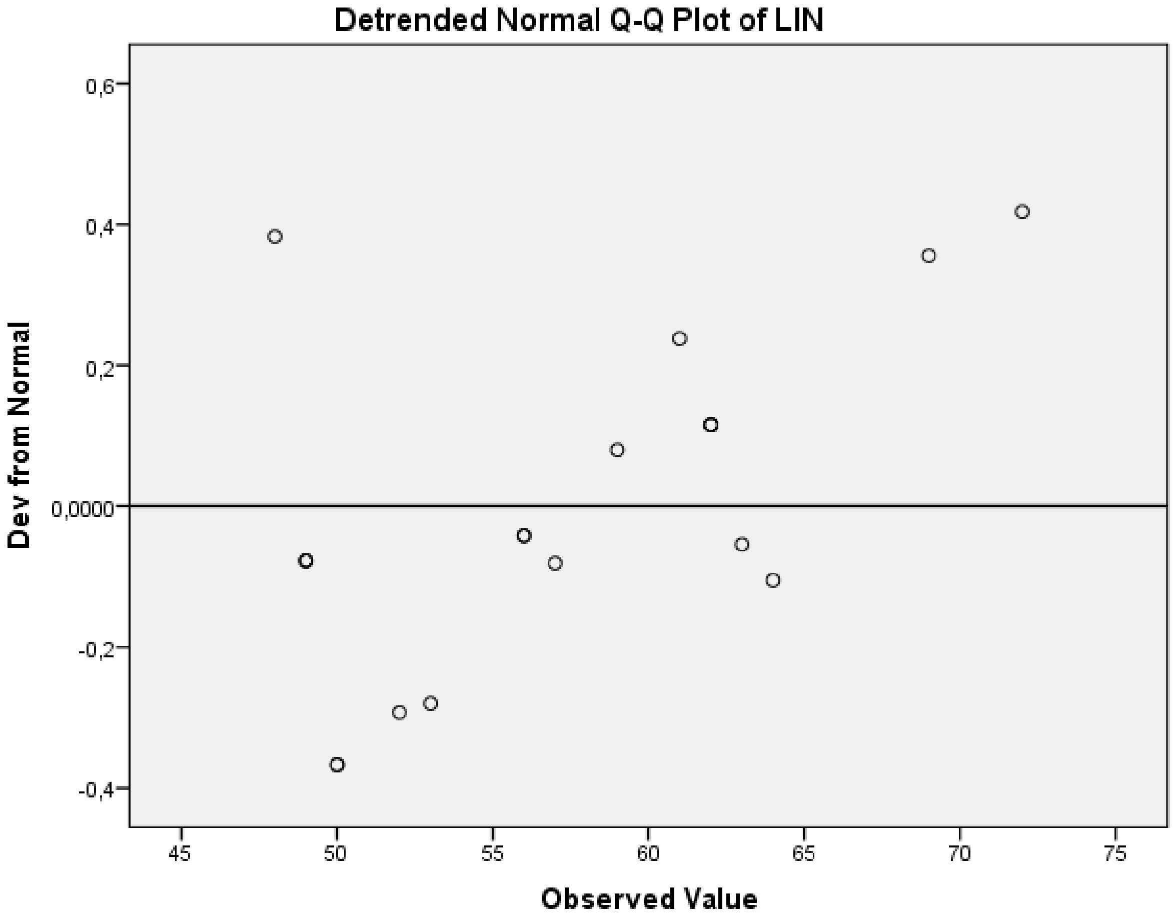

Gruplar = 5,00

Histograms

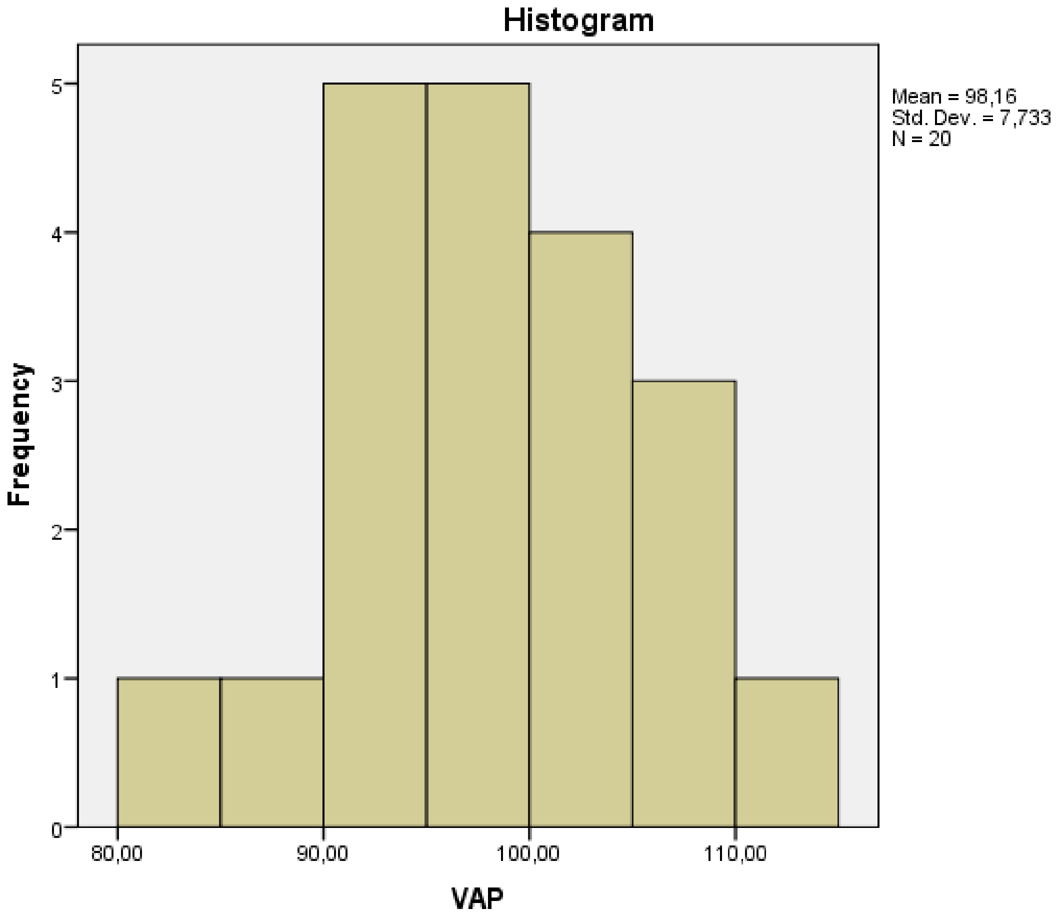
Histogram
Mean = 98,16
Std. Dev. = 7,733
N = 20
Frequency
0
1
2
3
4
5
80,00
90,00
100,00
110,00
VAP

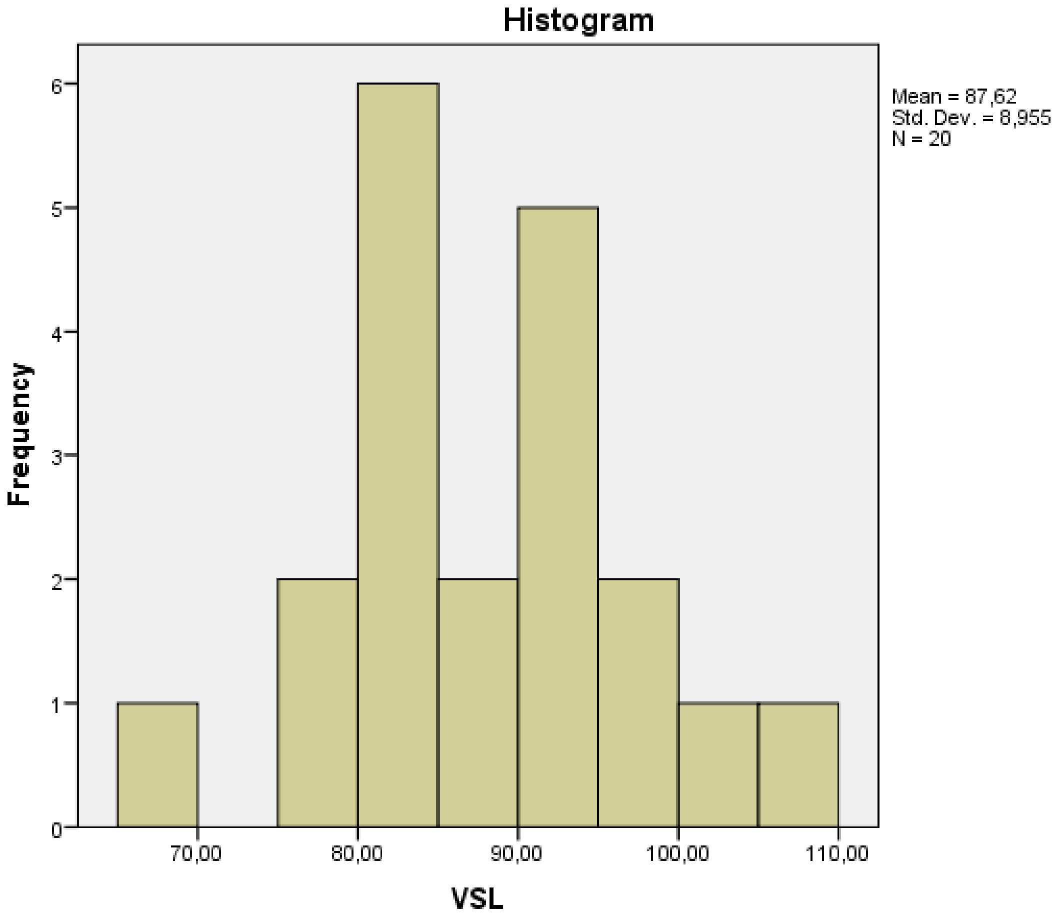
Histogram
Mean = 87,62
Std. Dev. = 8,955
N = 20
Frequency
0
1
2
3
4
5
6
70,00
80,00
90,00
100,00
110,00
VSL

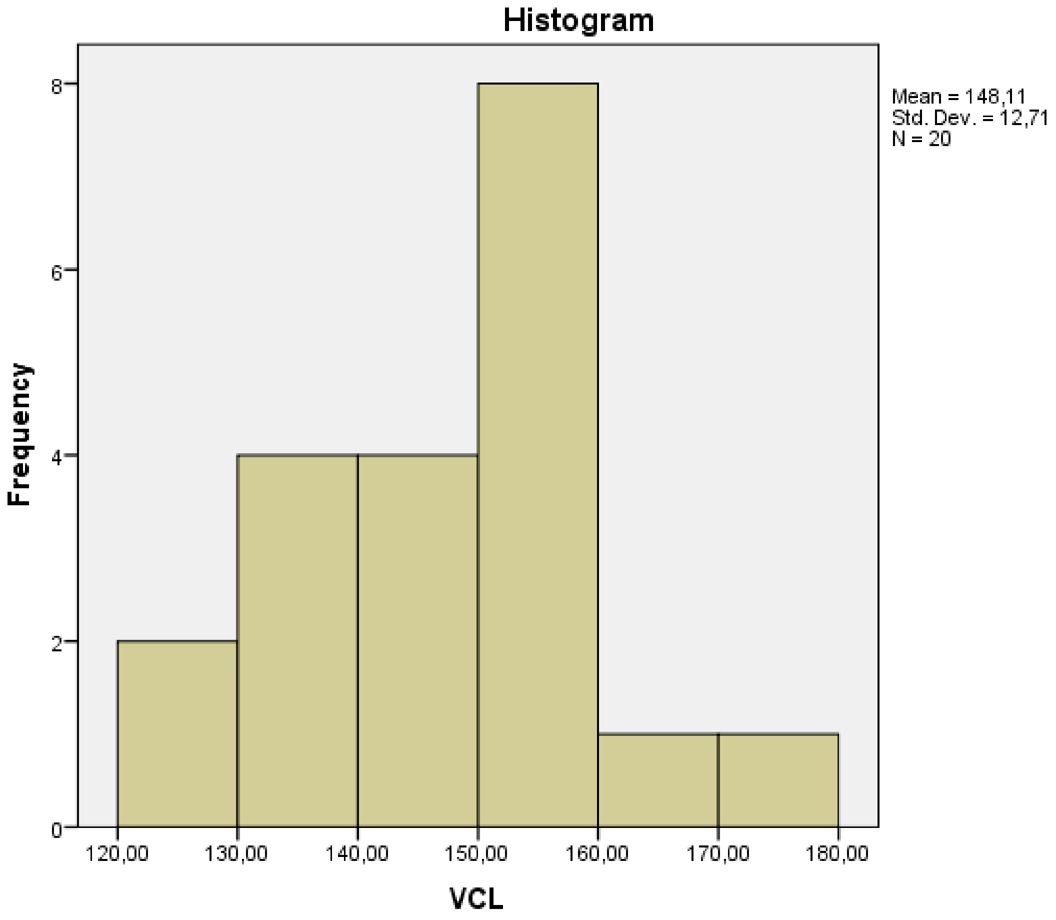
Histogram
Frequency
8
6
4
2
0
120,00
130,00
140,00
150,00
160,00
170,00
180,00
VCL
Mean = 148,11
Std. Dev. = 12,71
N = 20

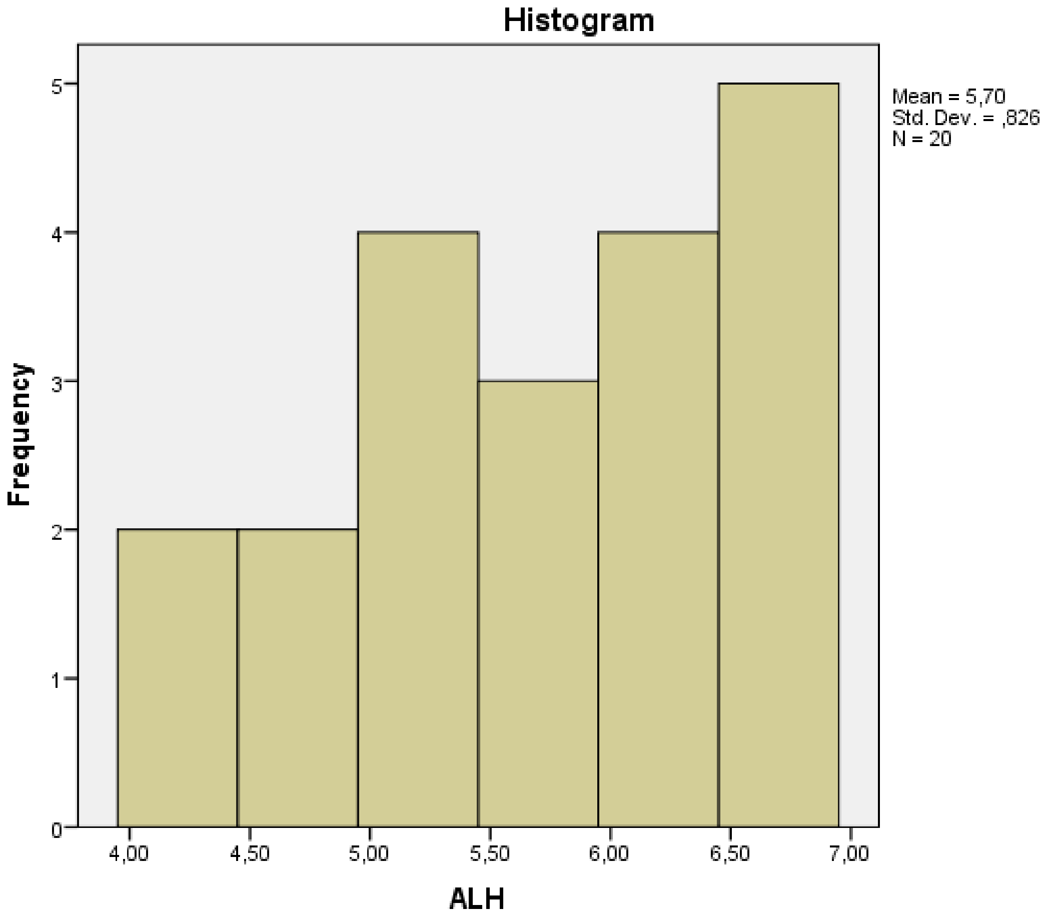
Histogram
Mean = 5,70
Std. Dev. = ,826
N = 20
Frequency
0
1
2
3
4
5
4,00
4,50
5,00
5,50
6,00
6,50
7,00
ALH

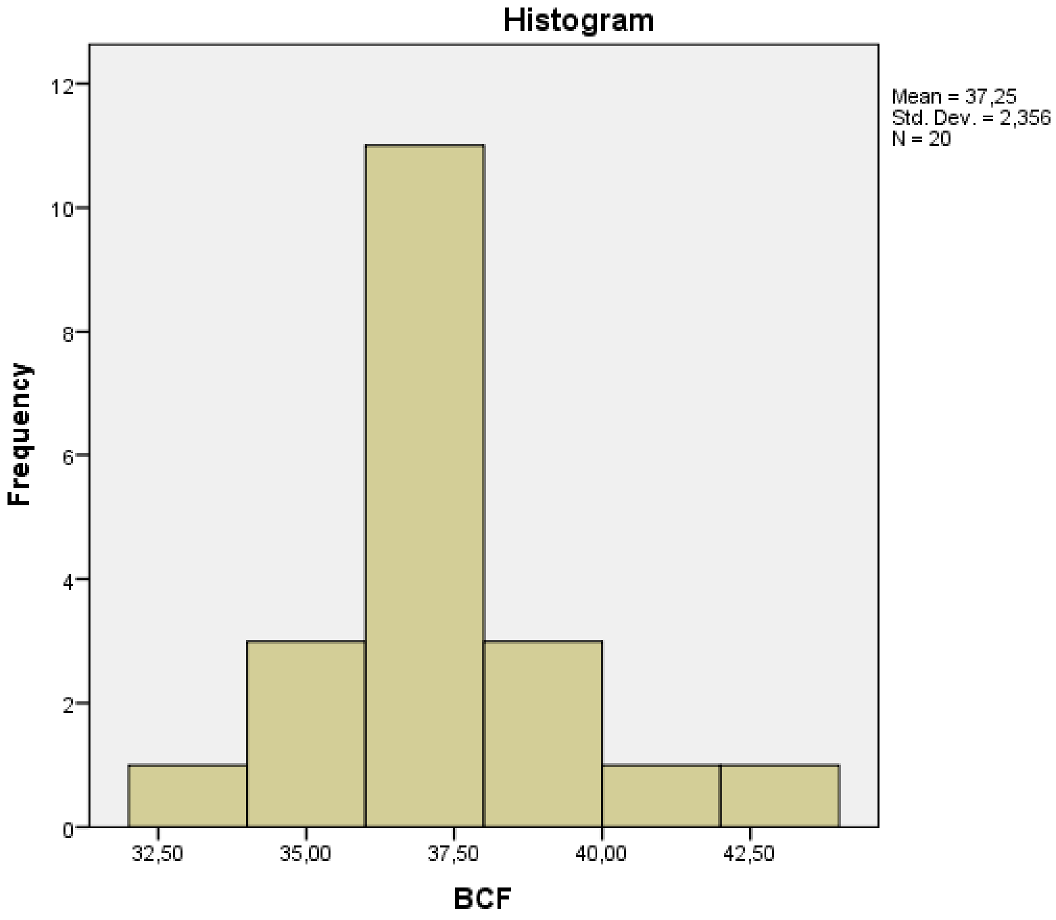
Histogram
Mean = 37,25
Std. Dev. = 2,356
N = 20
Frequency
0
2
4
6
8
10
12
32,50
35,00
37,50
40,00
42,50
BCF

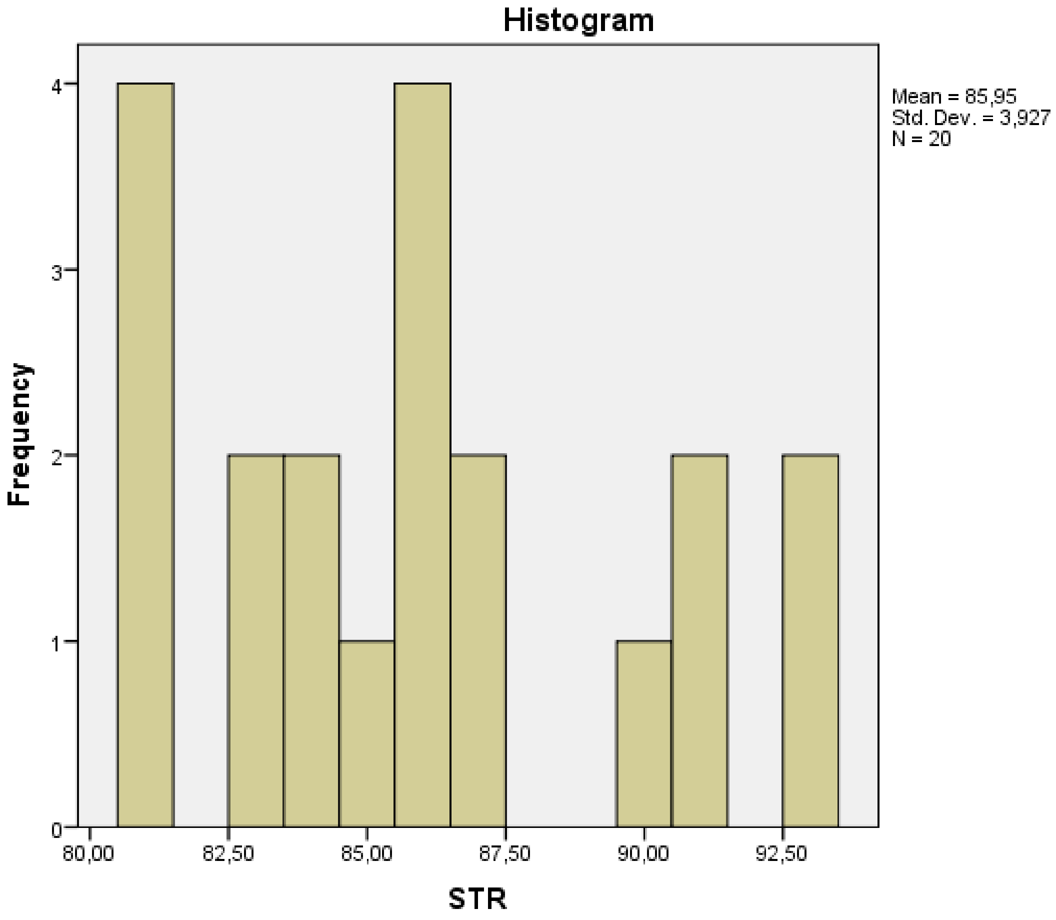

Histogram
Mean = 85,95
Std. Dev. = 3,927
N = 20
Frequency
0
1
2
3
4
80,00
82,50
85,00
87,50
90,00
92,50
STR

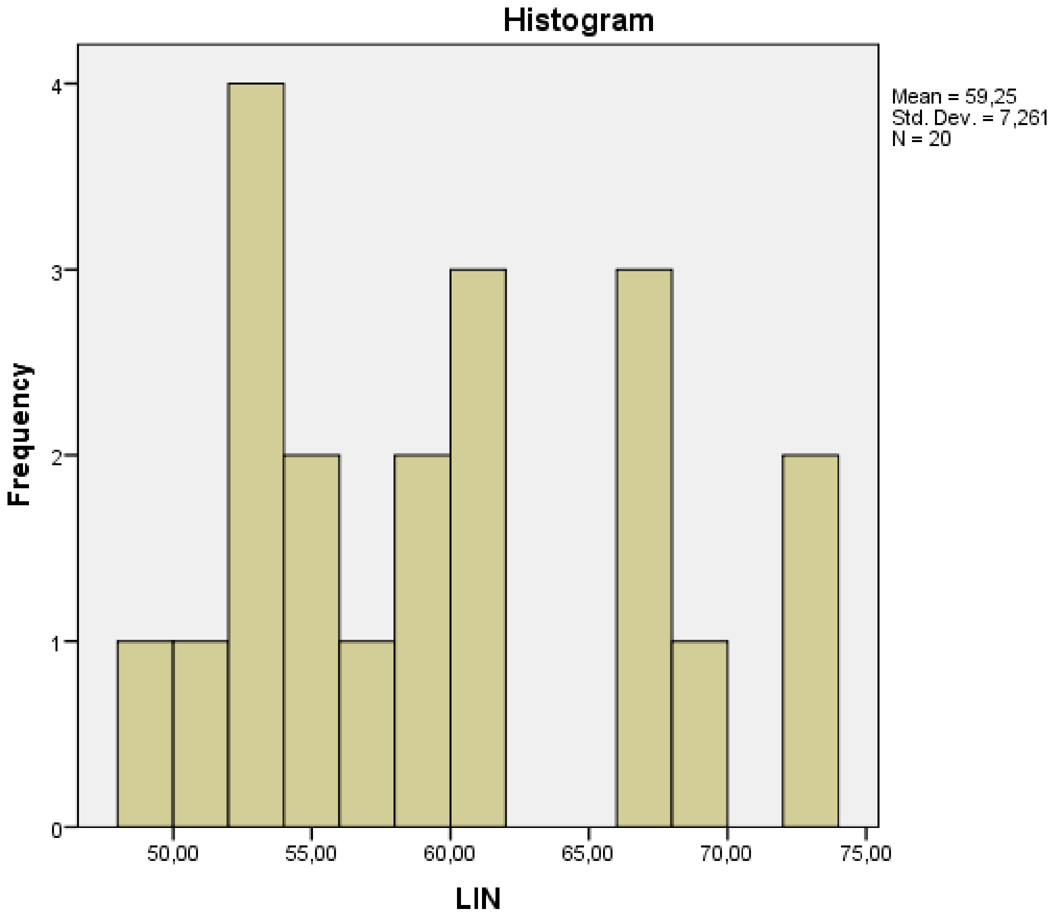

Stem-and-Leaf Plots

```
VAP Stem-and-Leaf Plot for
Gruplar= 5,00

 Frequency    Stem &  Leaf

     1,00        8 .  2
     1,00        8 .  7
     5,00        9 .  22244
     5,00        9 .  55678
     4,00       10 .  0123
     3,00       10 .  589
     1,00       11 .  4

 Stem width:  10,00
 Each leaf:       1 case(s)
```

```
VSL Stem-and-Leaf Plot for
Gruplar= 5,00

 Frequency    Stem &  Leaf

     1,00        6 .  8
     2,00        7 .  79
     8,00        8 .  00123459
     7,00        9 .  0122267
     2,00       10 .  15

 Stem width:  10,00
 Each leaf:       1 case(s)
```

```
VCL Stem-and-Leaf Plot for
Gruplar= 5,00

 Frequency    Stem &  Leaf

     2,00       12 .  88
     4,00       13 .  2569
     4,00       14 .  2369
     8,00       15 .  01344577
     1,00       16 .  5
     1,00       17 .  9

 Stem width:  10,00
 Each leaf:       1 case(s)
```

```
ALH Stem-and-Leaf Plot for
Gruplar= 5,00

 Frequency    Stem &  Leaf

     2,00        4 .  24
     2,00        4 .  58
     4,00        5 .  0234
     3,00        5 .  779
     4,00        6 .  1122
     5,00        6 .  56689

 Stem width:   1,00
 Each leaf:       1 case(s)
```

BCF Stem-and-Leaf Plot for
Gruplar= 5,00

Frequency	Stem &	Leaf
1,00	33 .	8
1,00	34 .	3
2,00	35 .	38
7,00	36 .	0122378
4,00	37 .	0119
1,00	38 .	1
2,00	39 .	27
2,00	Extremes	(>=41,7)

Stem width: 1,00
Each leaf: 1 case(s)

STR Stem-and-Leaf Plot for
Gruplar= 5,00

Frequency	Stem &	Leaf
8,00	8 .	11113344
7,00	8 .	5666677
5,00	9 .	01133

Stem width: 10,00
Each leaf: 1 case(s)

LIN Stem-and-Leaf Plot for
Gruplar= 5,00

Frequency	Stem &	Leaf
1,00	4 .	9
6,00	5 .	122334
4,00	5 .	5688
3,00	6 .	011
4,00	6 .	6678
2,00	7 .	23

Stem width: 10,00
Each leaf: 1 case(s)

Normal Q-Q Plots

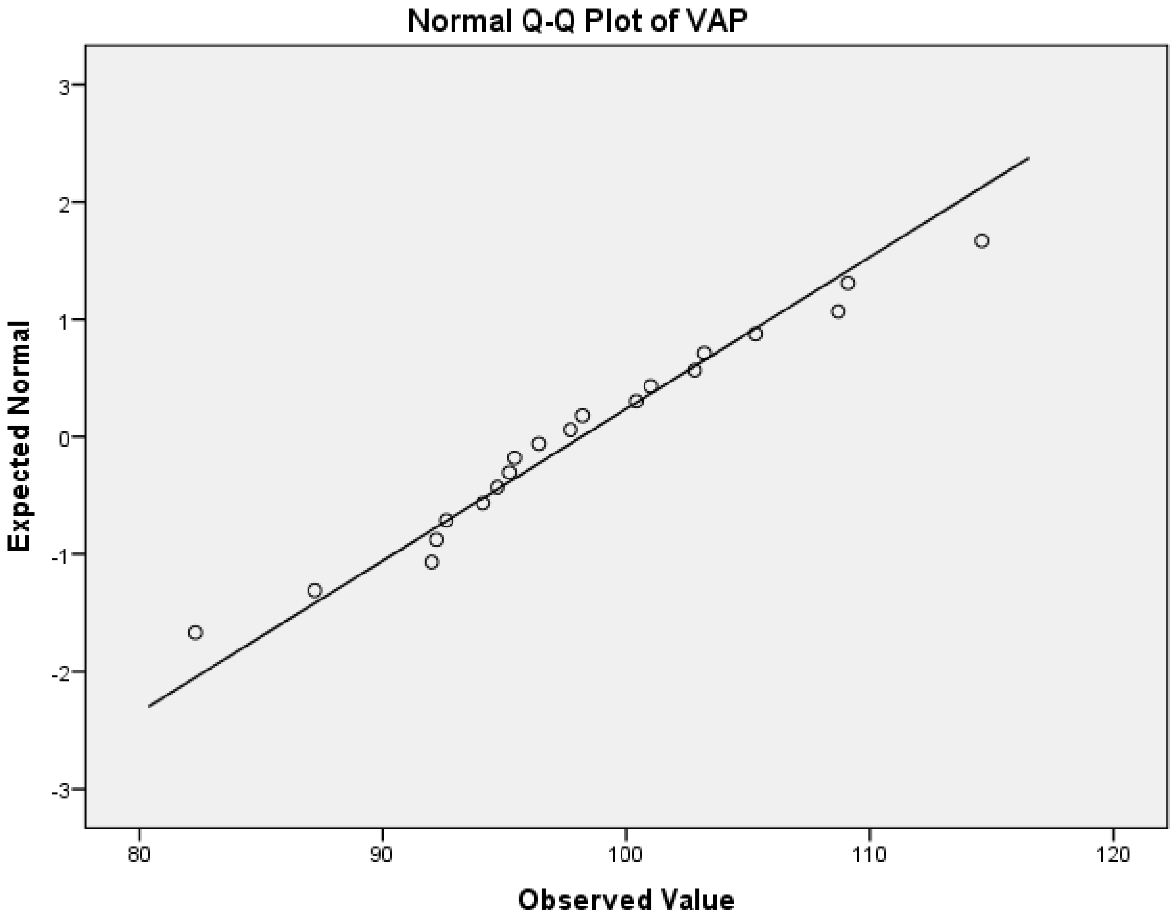

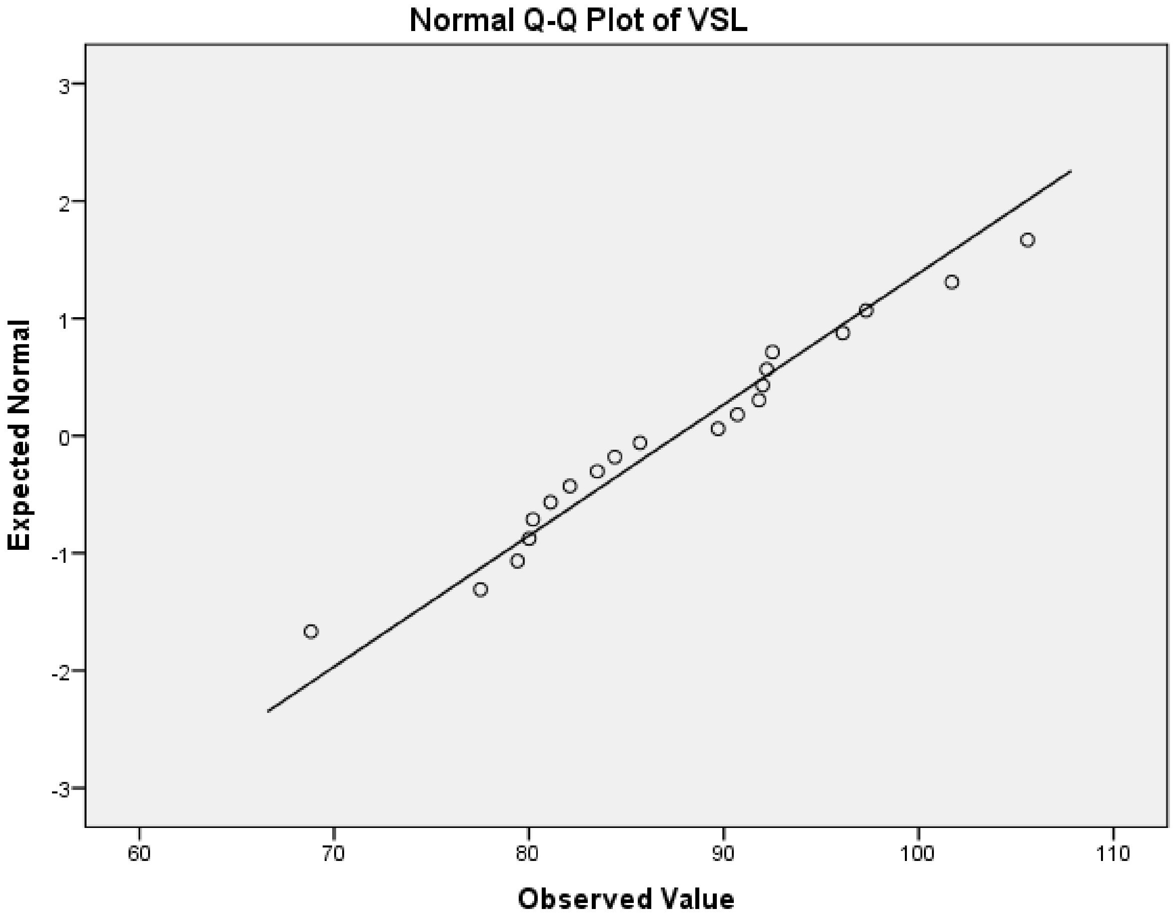
Normal Q-Q Plot of VSL
Expected Normal
Observed Value
3
2
1
0
-1
-2
-3
60
70
80
90
100
110

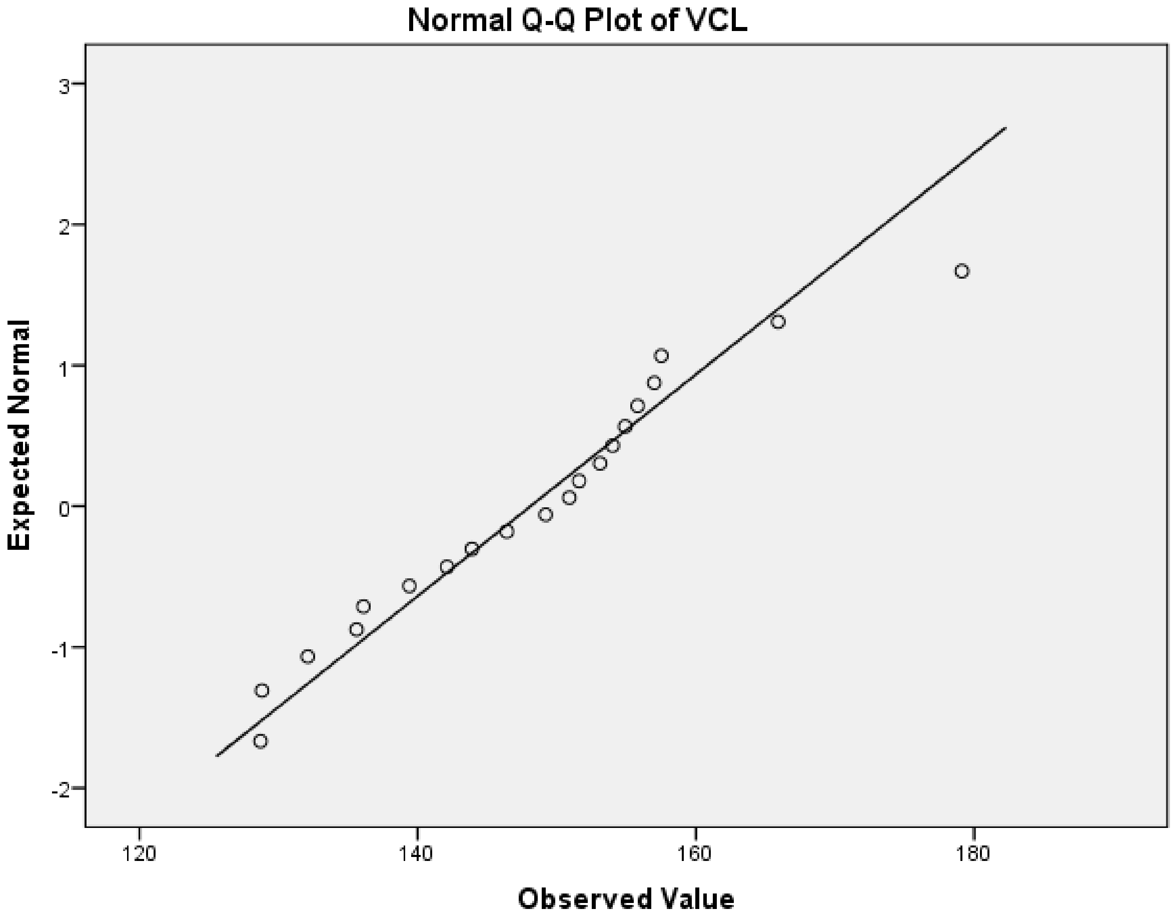
Normal Q-Q Plot of VCL
Expected Normal
3
2
1
0
-1
-2
120
140
160
180
Observed Value

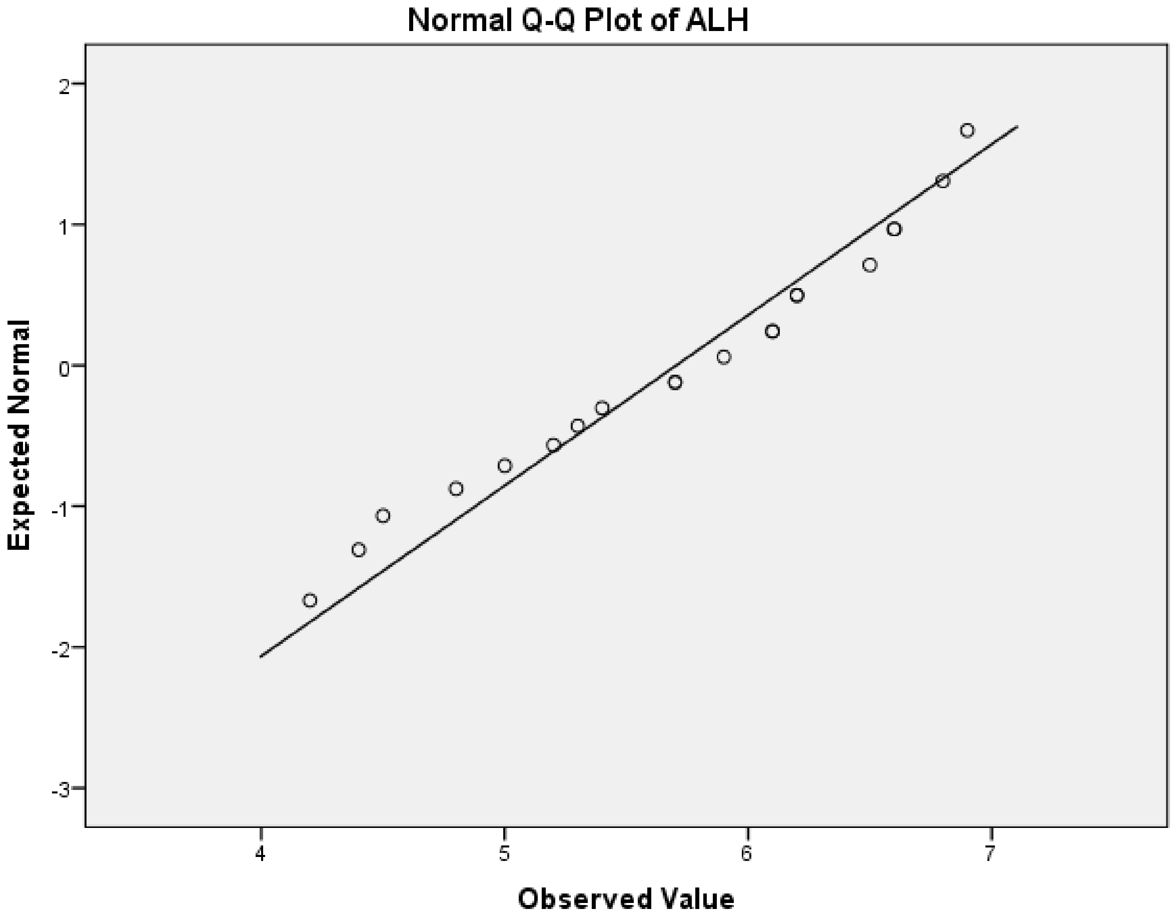

Normal Q-Q Plot of ALH
Expected Normal
2
1
0
-1
-2
-3
4
5
6
7
Observed Value

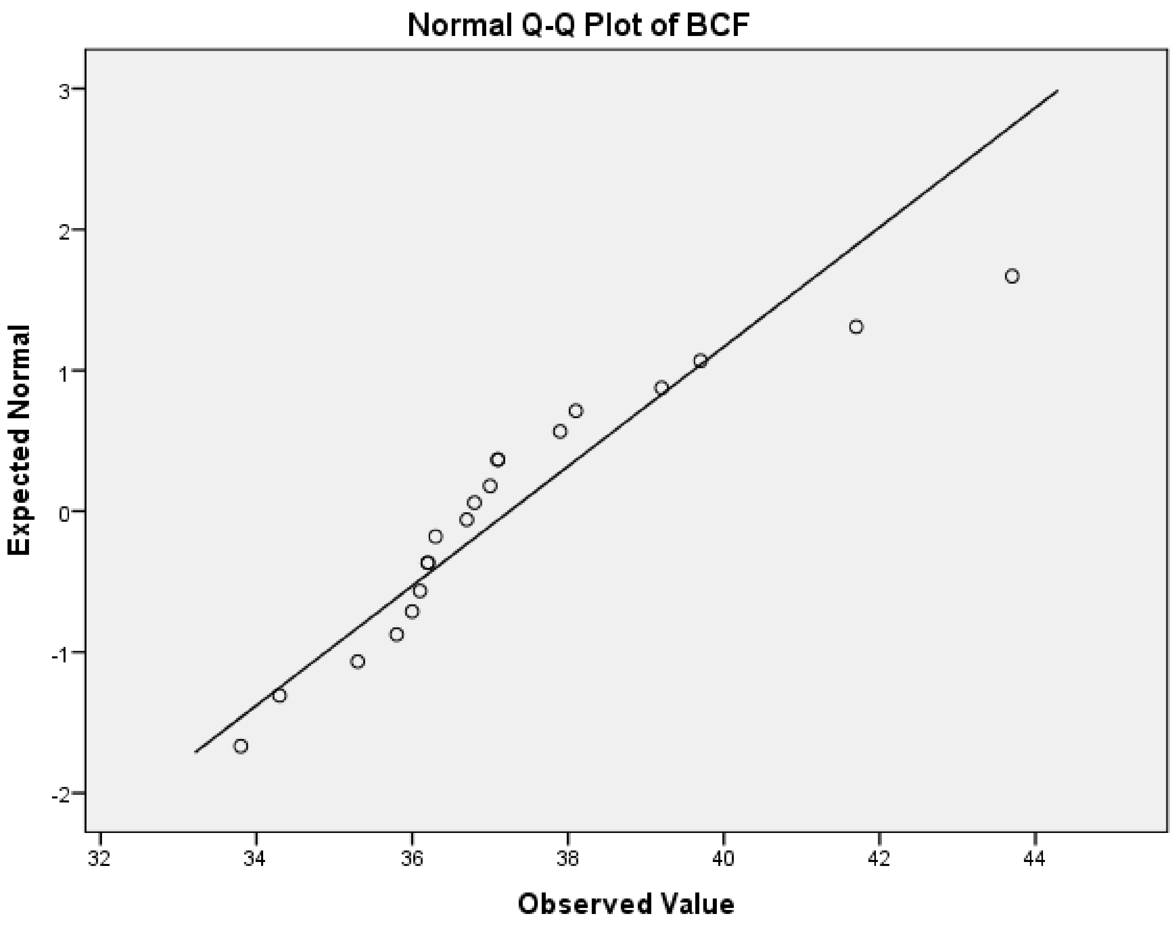

Normal Q-Q Plot of BCF
Expected Normal
Observed Value
3
2
1
0
-1
-2
32
34
36
38
40
42
44

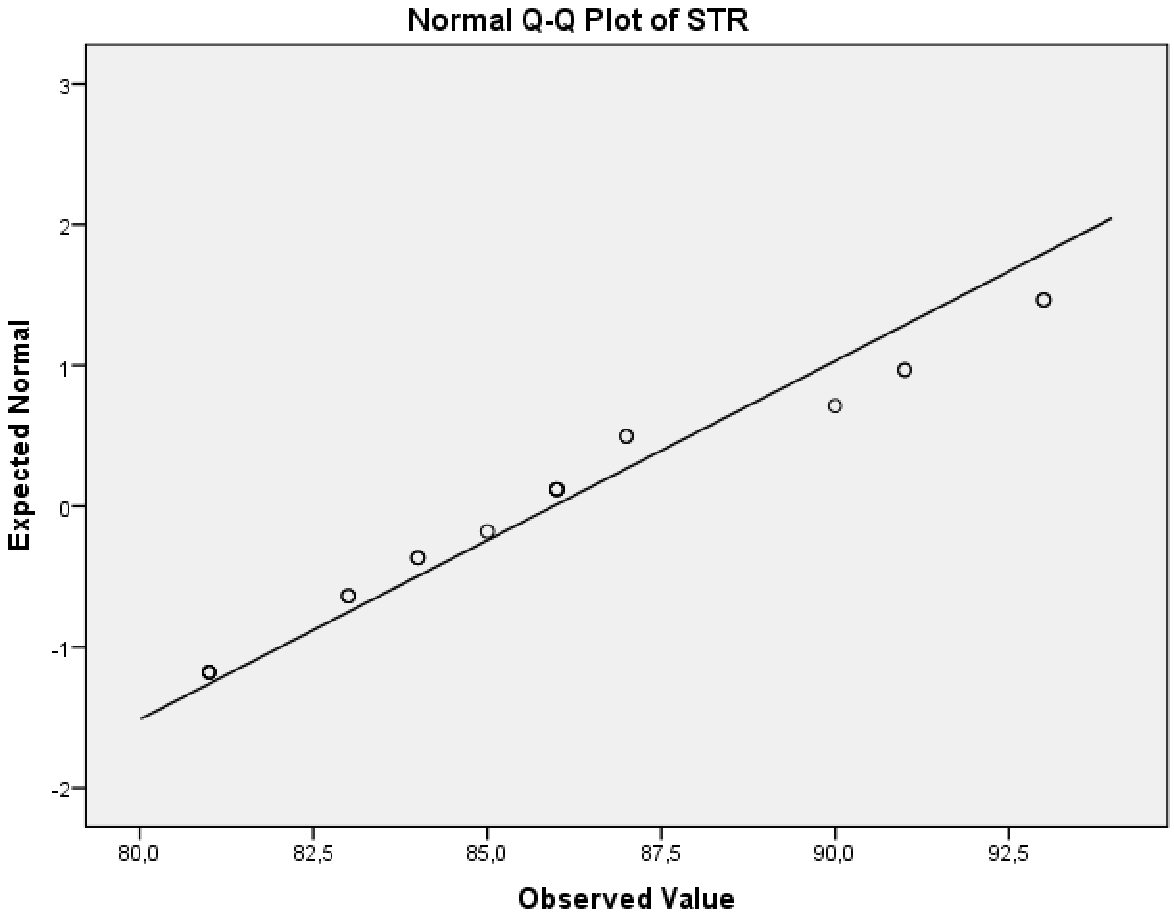

Normal Q-Q Plot of STR
Expected Normal
Observed Value
3
2
1
0
-1
-2
80,0
82,5
85,0
87,5
90,0
92,5

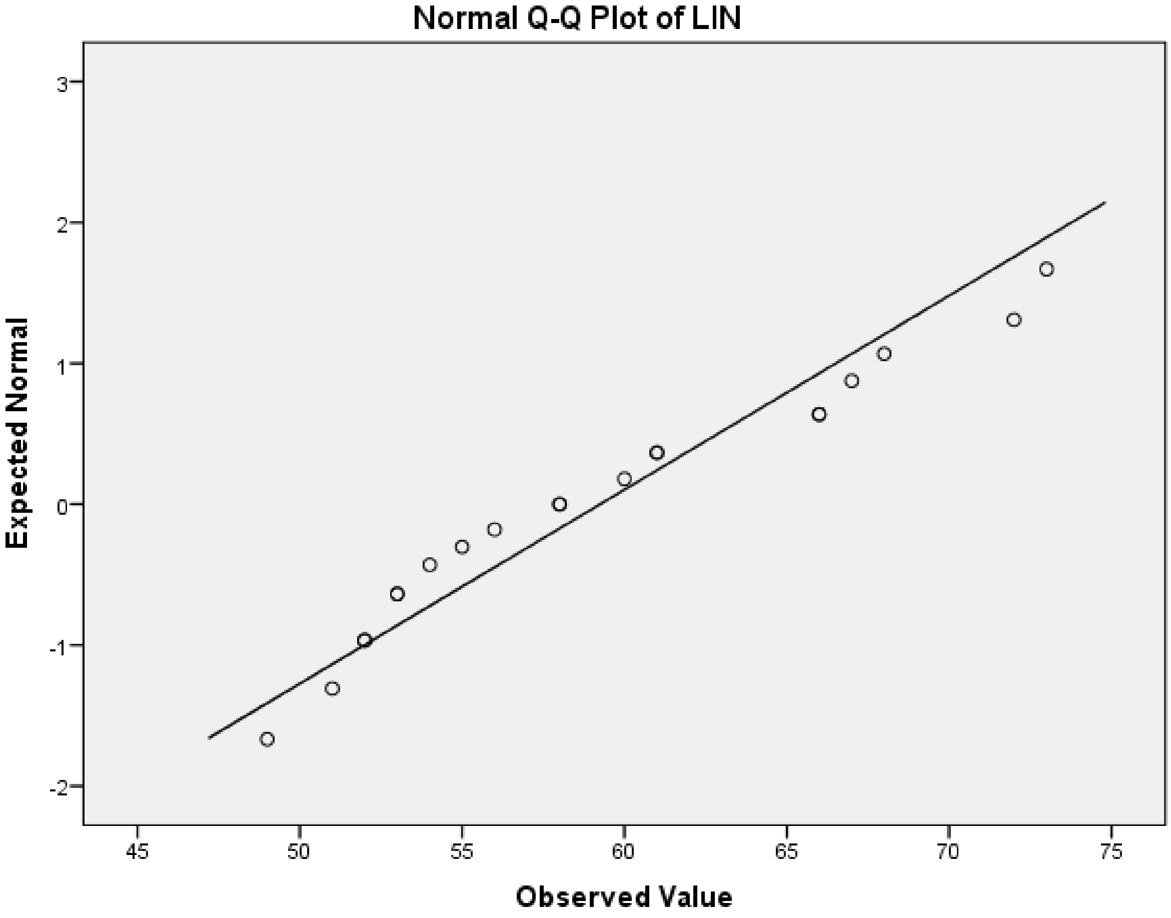

Detrended Normal Q-Q Plots

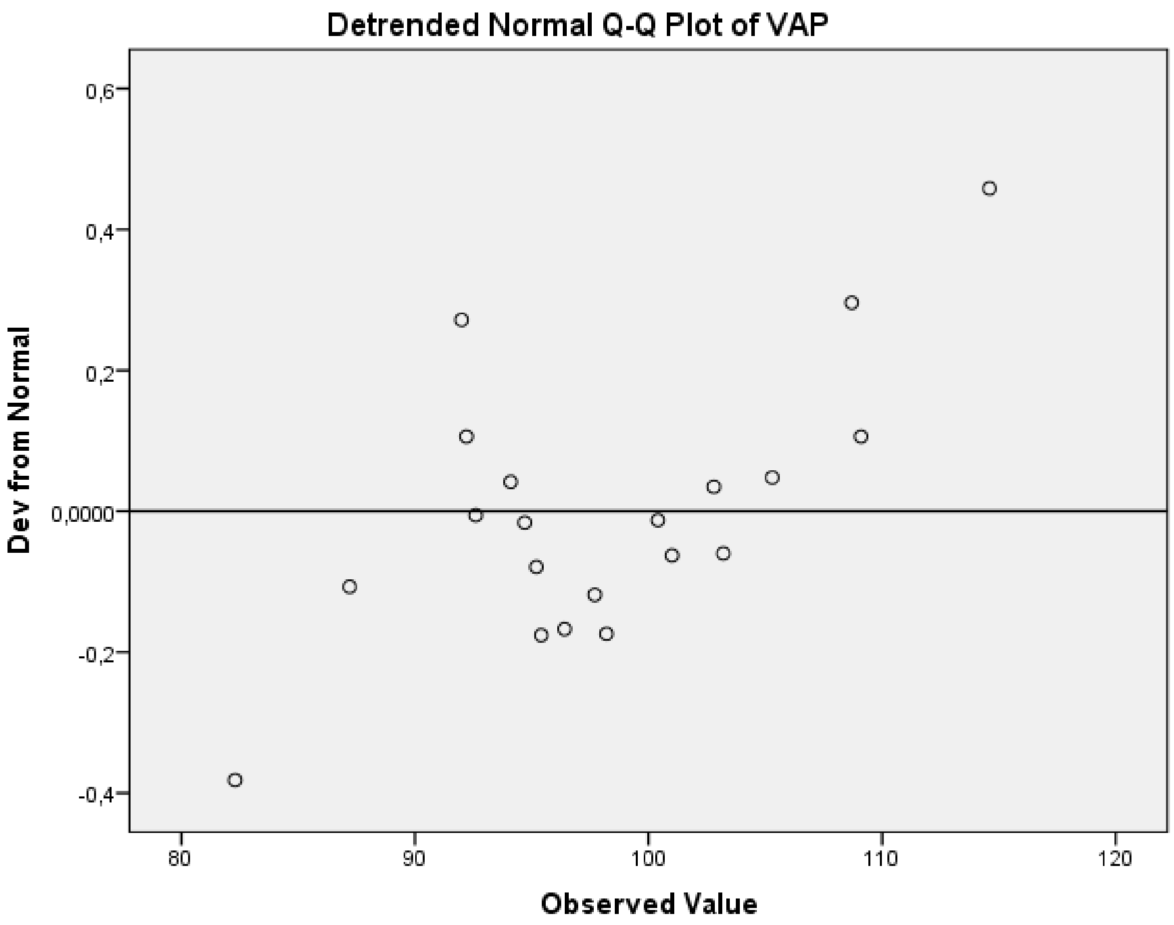
Detrended Normal Q-Q Plot of VAP
0,6
0,4
0,2
0,0000
-0,2
-0,4
Dev from Normal
80
90
100
110
120
Observed Value

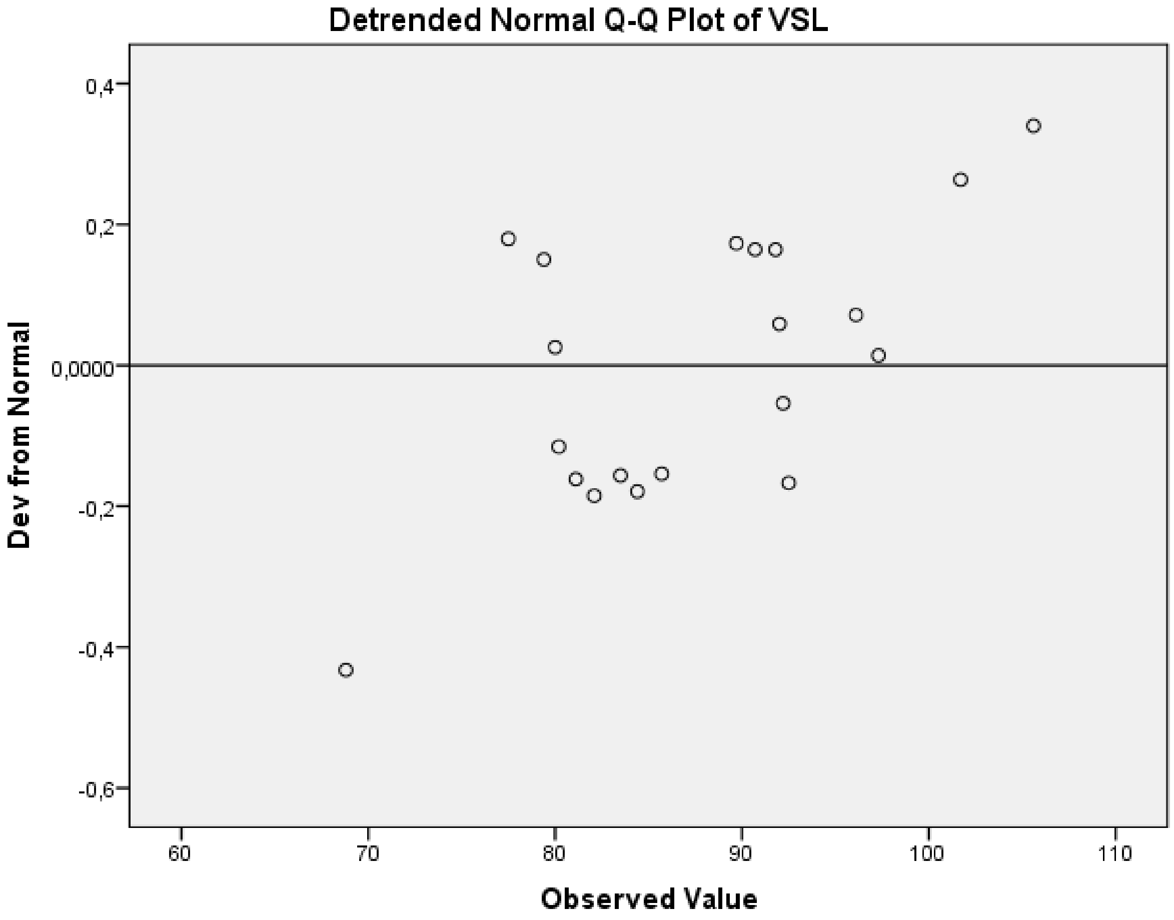

Detrended Normal Q-Q Plot of VSL
Dev from Normal
0,4
0,2
0,0000
-0,2
-0,4
-0,6
60
70
80
90
100
110
Observed Value

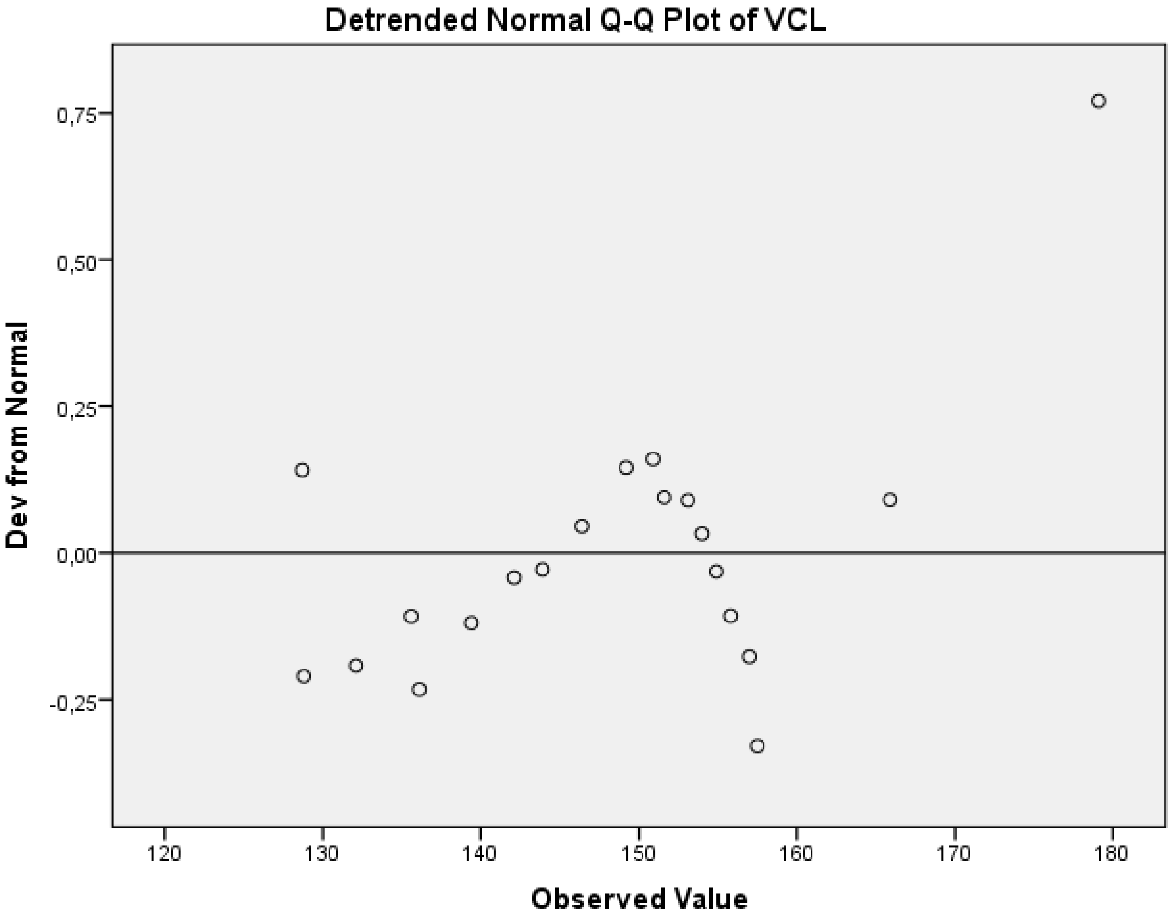
Detrended Normal Q-Q Plot of VCL
Dev from Normal
0,75
0,50
0,25
0,00
-0,25
120
130
140
150
160
170
180
Observed Value

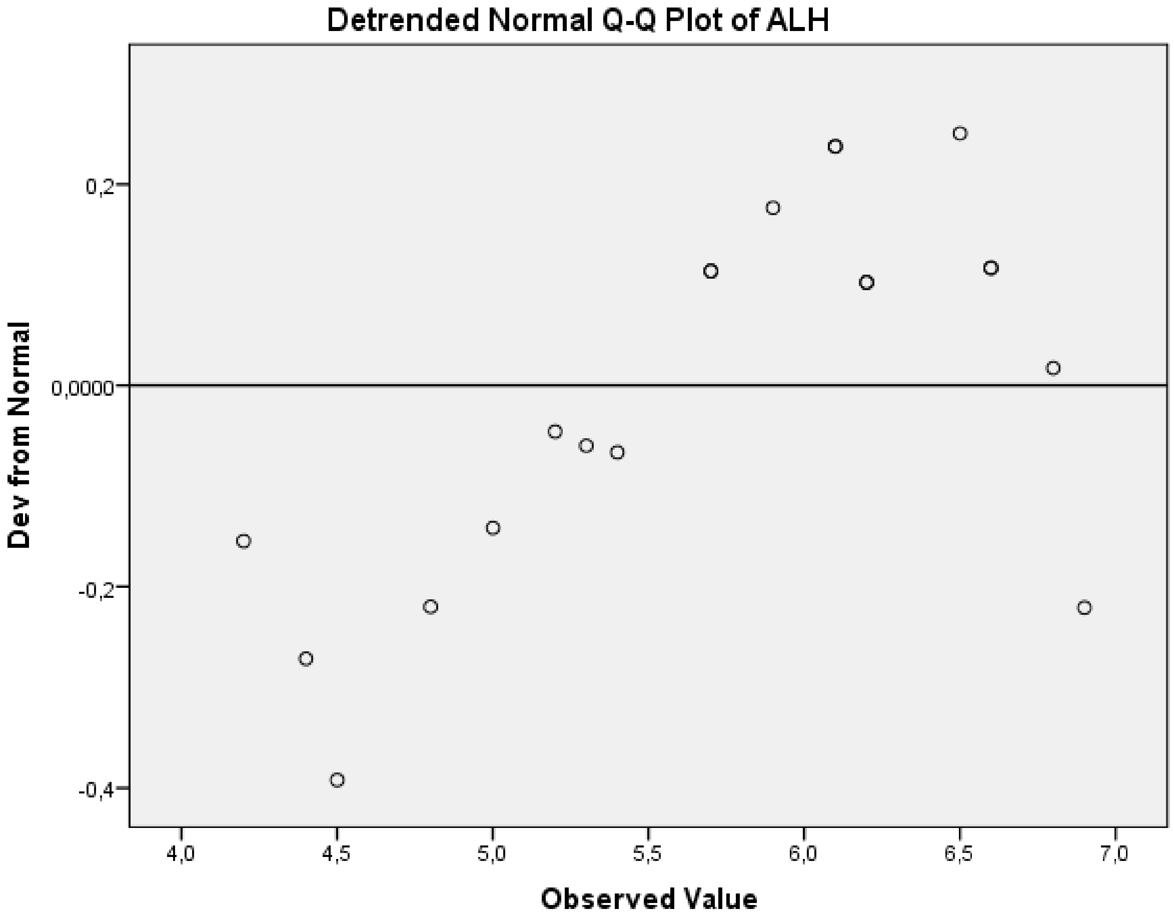
Detrended Normal Q-Q Plot of ALH
Dev from Normal
0,2
0,0000
-0,2
-0,4
4,0
4,5
5,0
5,5
6,0
6,5
7,0
Observed Value

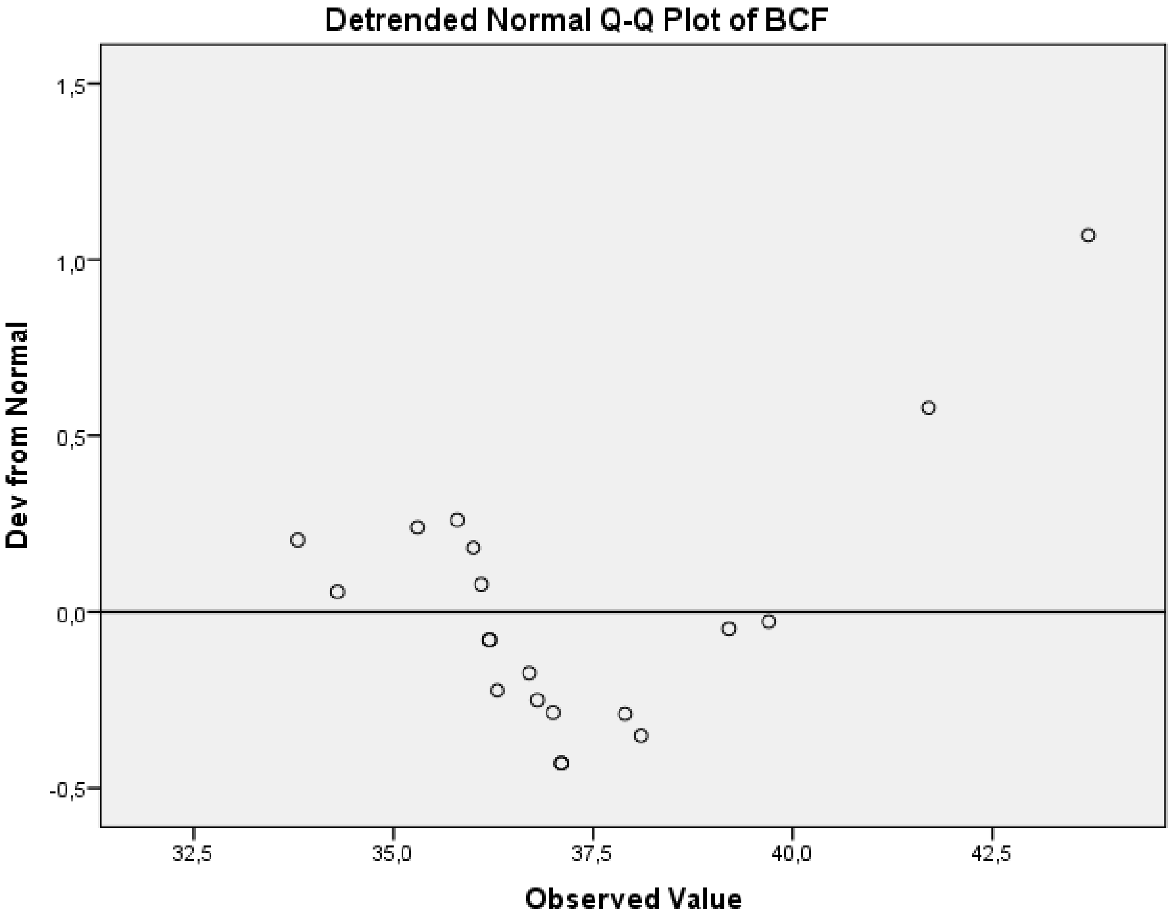

Detrended Normal Q-Q Plot of BCF
Dev from Normal
1,5
1,0
0,5
0,0
-0,5
32,5
35,0
37,5
40,0
42,5
Observed Value

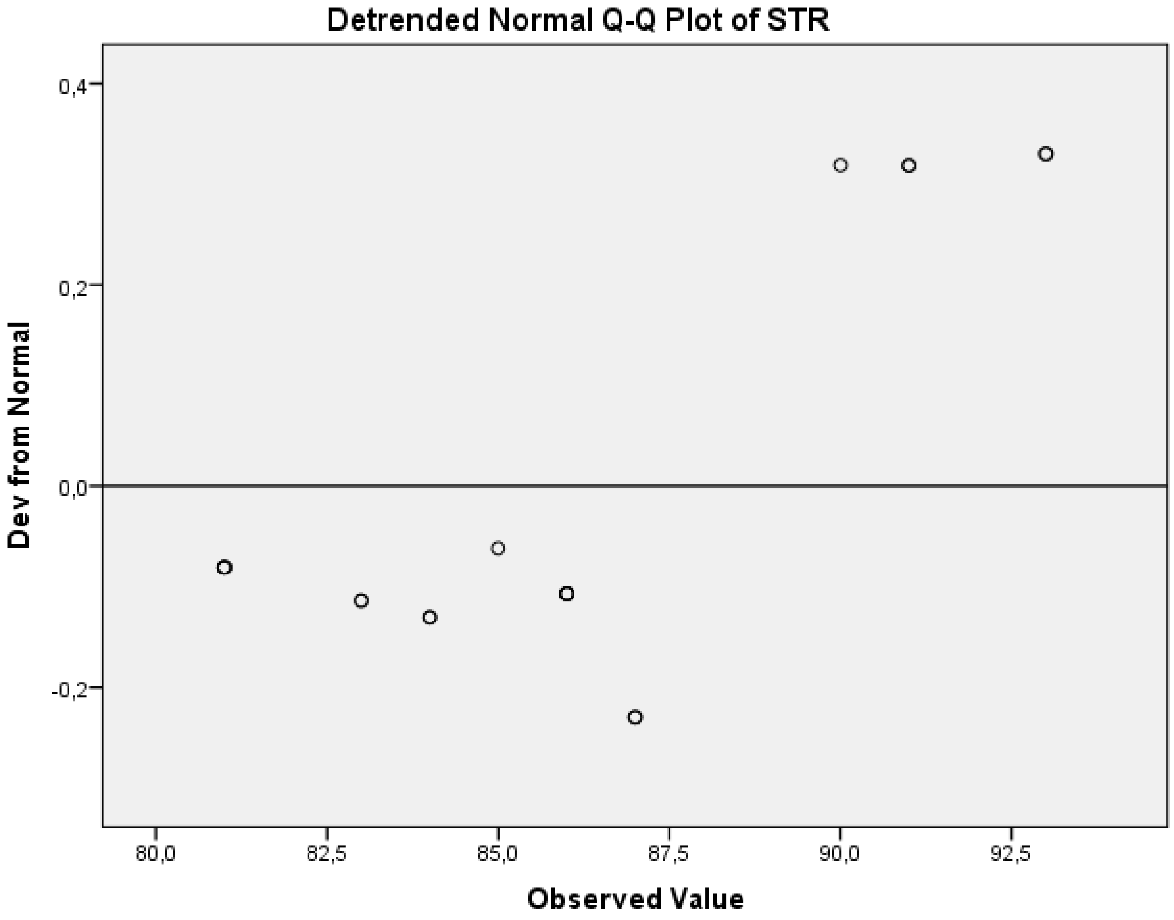
Detrended Normal Q-Q Plot of STR
Dev from Normal
0,4
0,2
0,0
-0,2
80,0
82,5
85,0
87,5
90,0
92,5
Observed Value

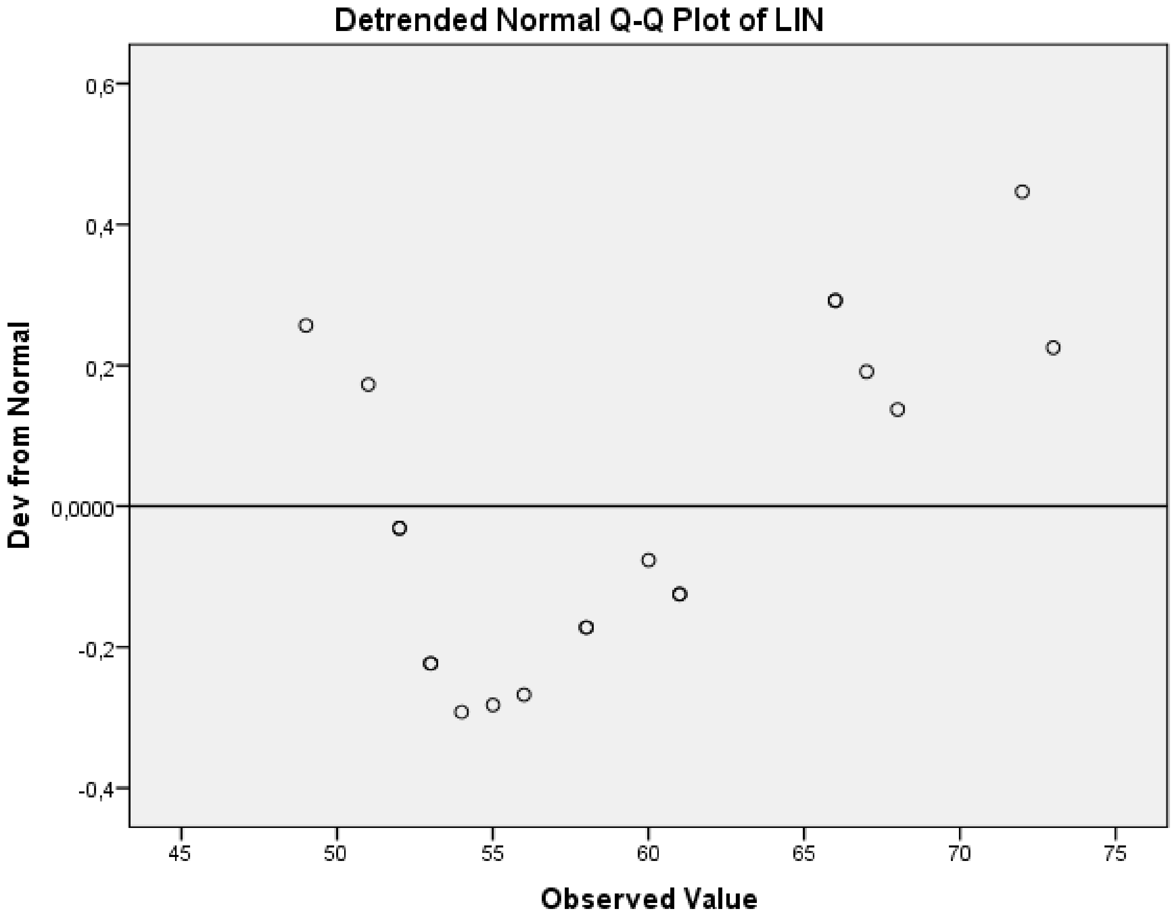

Gruplar = 6,00

Histograms

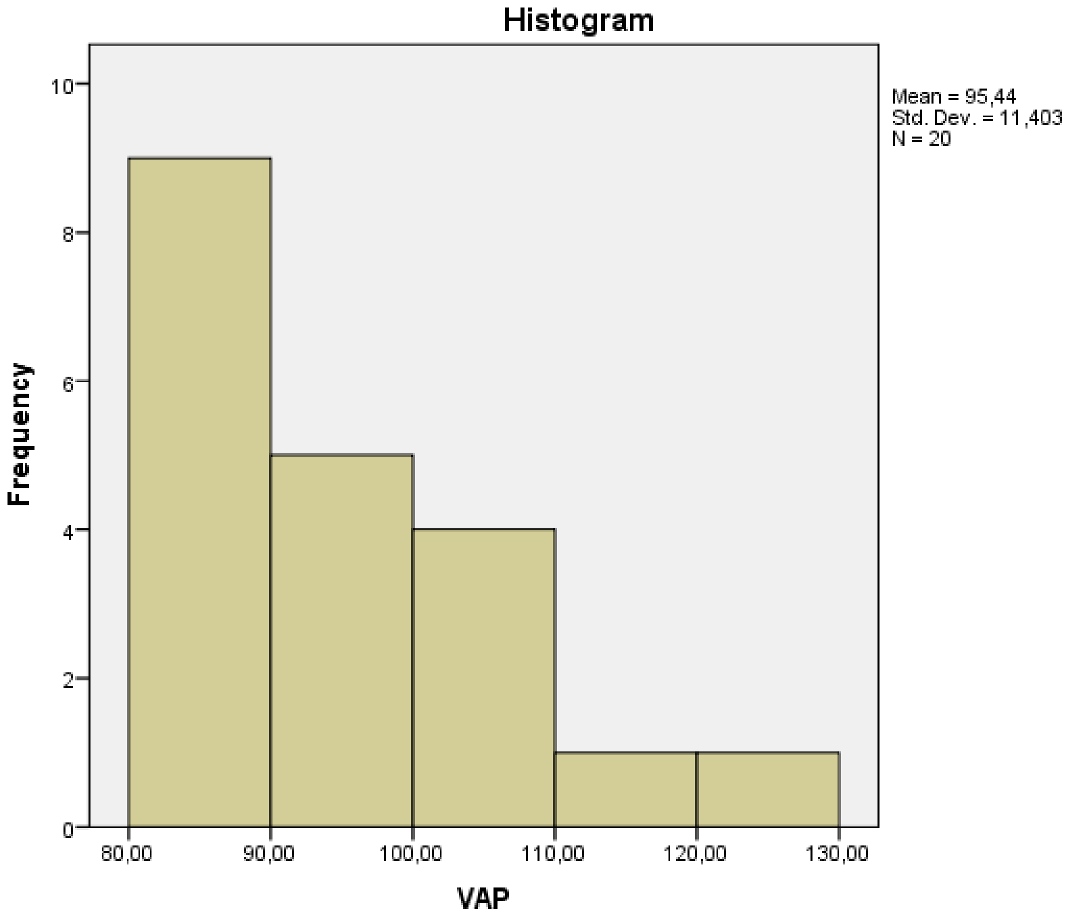

Histogram
Mean = 95,44
Std. Dev. = 11,403
N = 20
Frequency
0
2
4
6
8
10
80,00
90,00
100,00
110,00
120,00
130,00
VAP

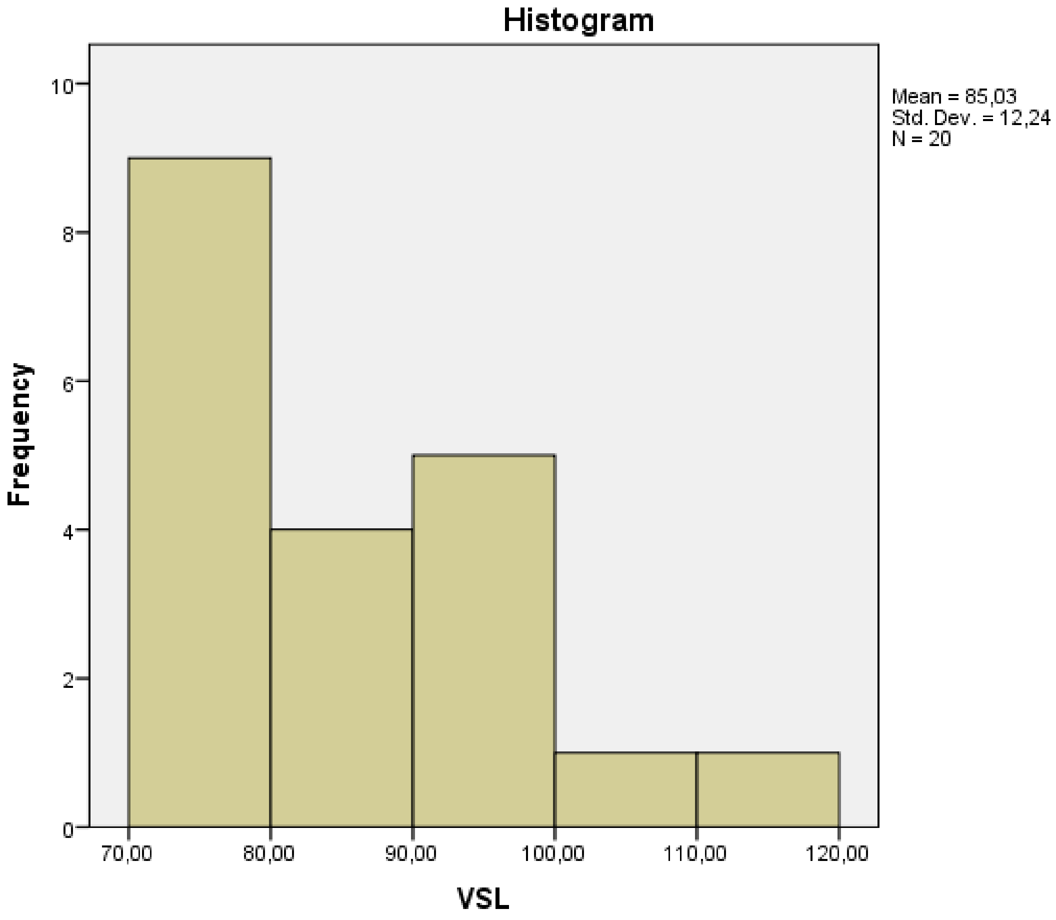
Histogram
Mean = 85,03
Std. Dev. = 12,24
N = 20
Frequency
0
2
4
6
8
10
70,00
80,00
90,00
100,00
110,00
120,00
VSL

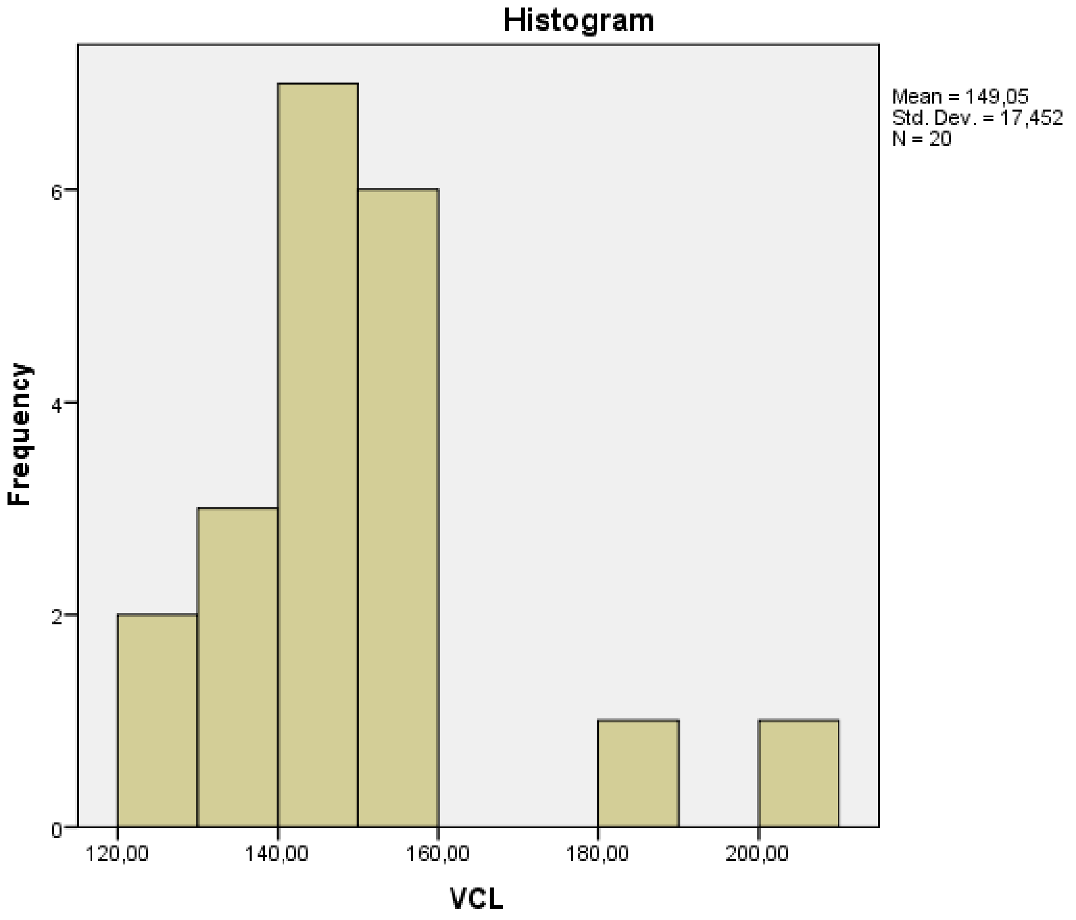
Histogram
Mean = 149,05
Std. Dev. = 17,452
N = 20
Frequency
0
2
4
6
120,00
140,00
160,00
180,00
200,00
VCL

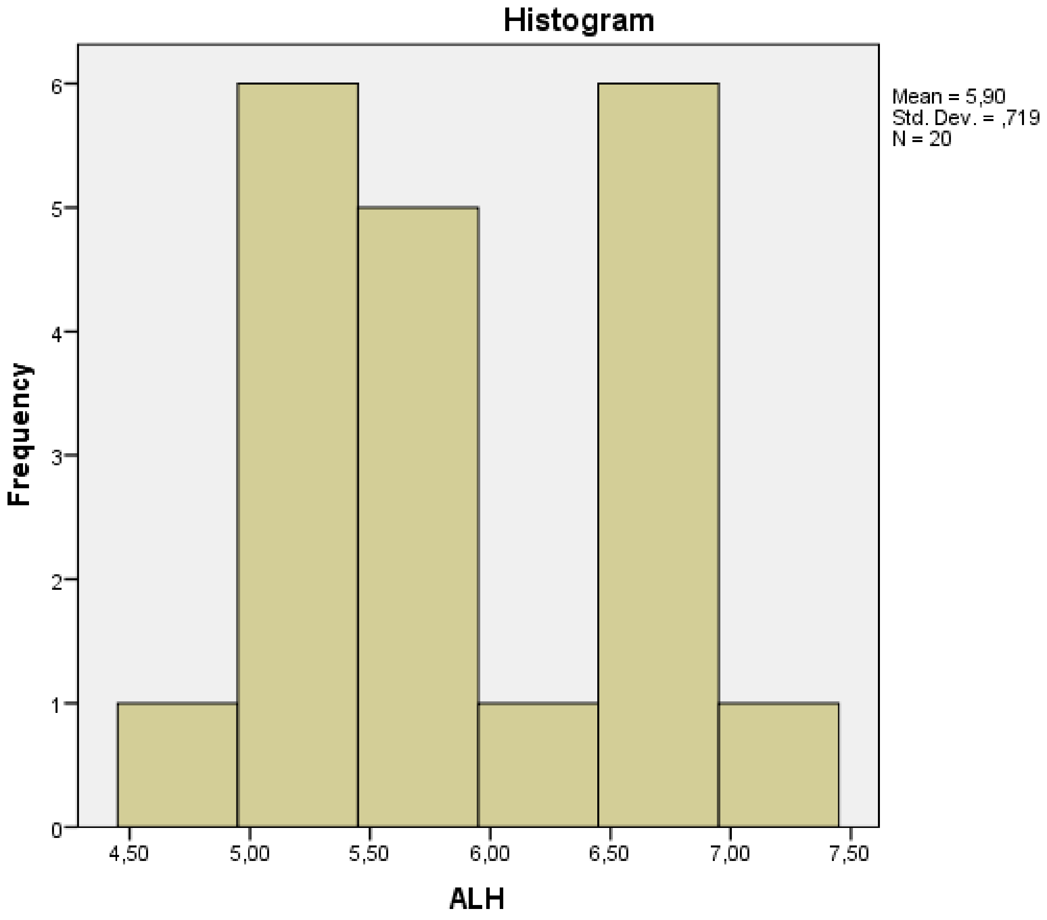

Histogram
Mean = 5,90
Std. Dev. = ,719
N = 20
Frequency
0
1
2
3
4
5
6
4,50
5,00
5,50
6,00
6,50
7,00
7,50
ALH

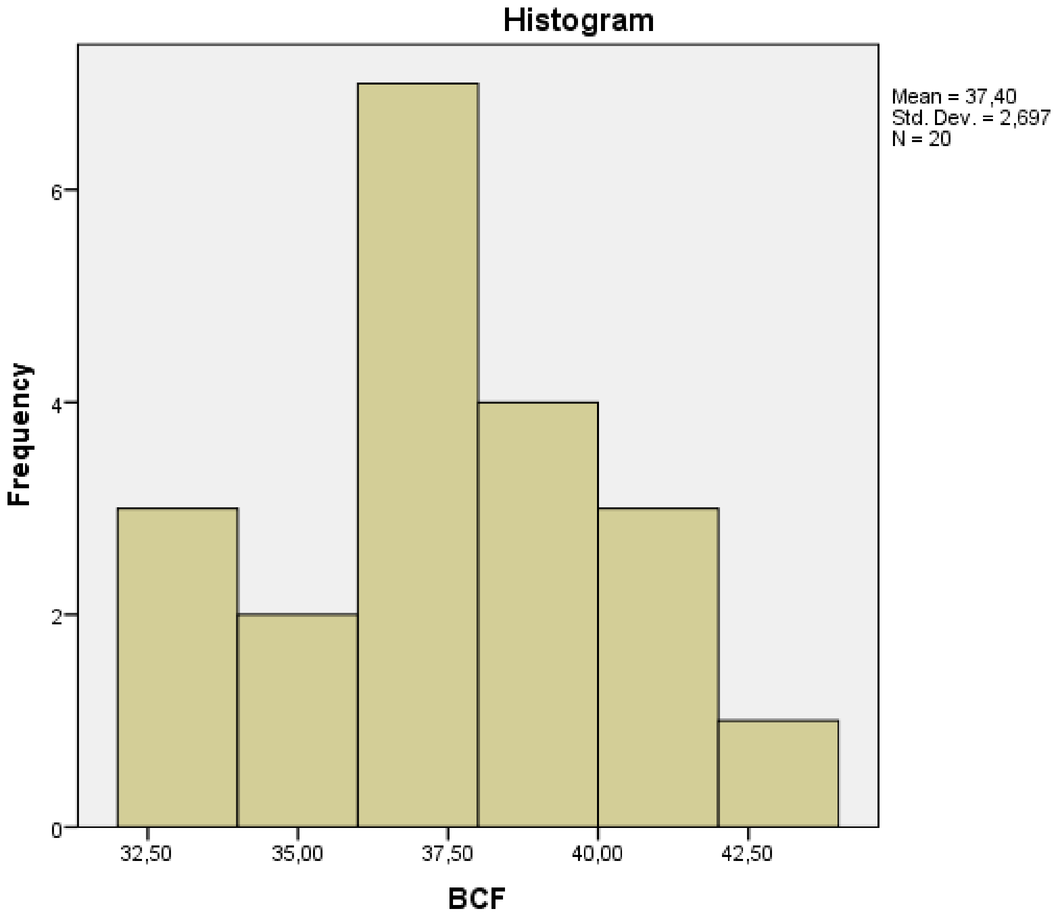
Histogram
Mean = 37,40
Std. Dev. = 2,697
N = 20
Frequency
0
2
4
6
32,50
35,00
37,50
40,00
42,50
BCF

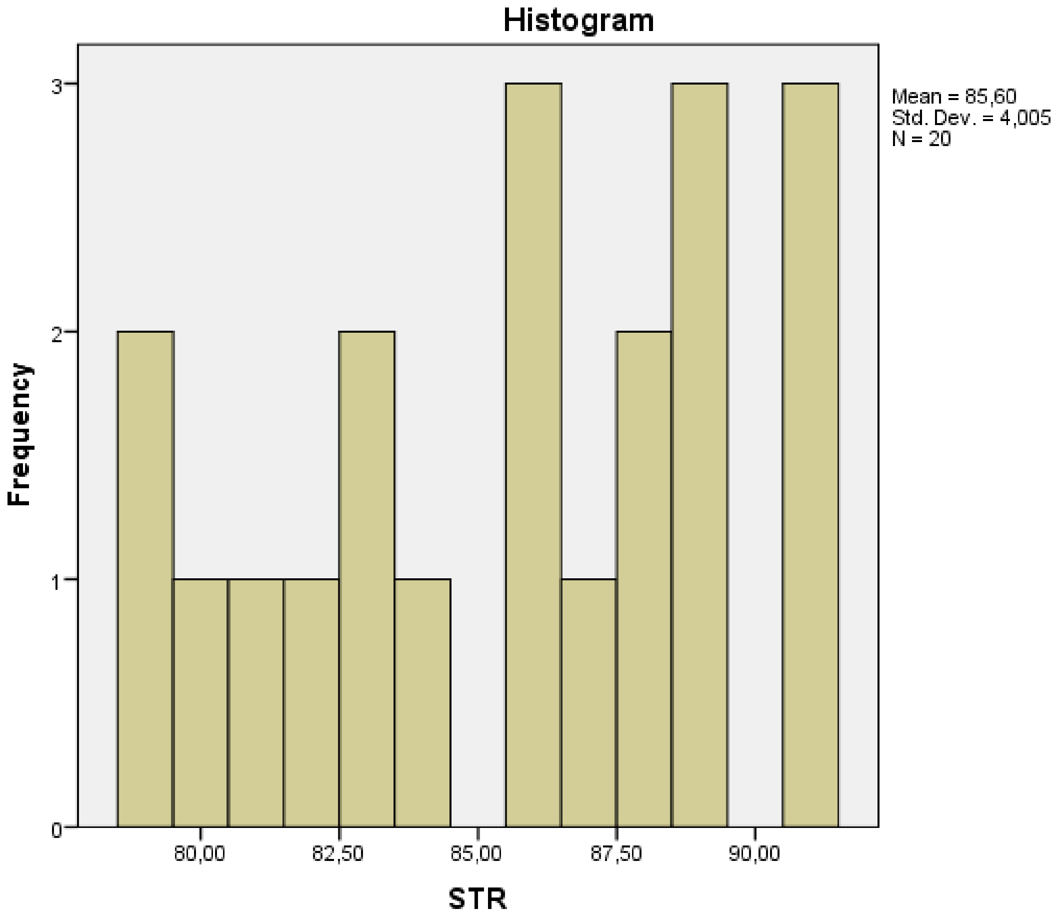
Histogram
Mean = 85,60
Std. Dev. = 4,005
N = 20
Frequency
0
1
2
3
80,00
82,50
85,00
87,50
90,00
STR

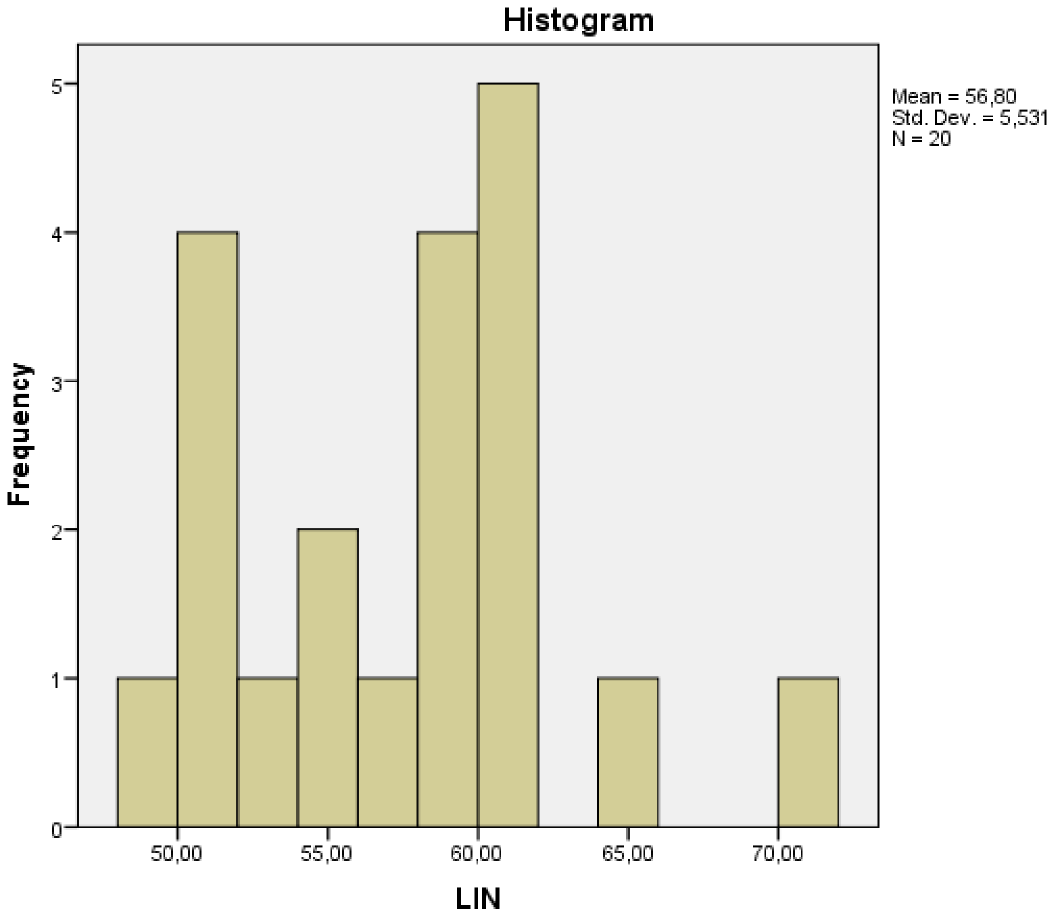

Stem-and-Leaf Plots

```
VAP Stem-and-Leaf Plot for
Gruplar= 6,00

 Frequency    Stem &  Leaf

     9,00        8 .  133677889
     5,00        9 .  23349
     4,00       10 .  3556
     1,00       11 .  2
     1,00       12 .  6

 Stem width:  10,00
 Each leaf:       1 case(s)
```

VSL Stem-and-Leaf Plot for
Gruplar= 6,00

Frequency	Stem &	Leaf
9,00	7 .	023456668
4,00	8 .	0134
5,00	9 .	13689
1,00	10 .	2
1,00	11 .	5

Stem width: 10,00
Each leaf: 1 case(s)

VCL Stem-and-Leaf Plot for
Gruplar= 6,00

Frequency	Stem &	Leaf
1,00	12 .	1
1,00	12 .	9
1,00	13 .	4
2,00	13 .	56
4,00	14 .	1233
3,00	14 .	689
4,00	15 .	1234
2,00	15 .	59
2,00	Extremes	(>=182)

Stem width: 10,00
Each leaf: 1 case(s)

ALH Stem-and-Leaf Plot for
Gruplar= 6,00

Frequency	Stem &	Leaf
1,00	4 .	7
6,00	5 .	123334
5,00	5 .	55789
1,00	6 .	0
6,00	6 .	567778
1,00	7 .	3

Stem width: 1,00
Each leaf: 1 case(s)

```
BCF Stem-and-Leaf Plot for
Gruplar= 6,00

 Frequency    Stem &  Leaf

     3,00        3 .  233
    13,00        3 .  5566667778899
     4,00        4 .  0012

 Stem width:  10,00
 Each leaf:       1 case(s)
```

```
STR Stem-and-Leaf Plot for
Gruplar= 6,00

 Frequency    Stem &  Leaf

     2,00        7 .  99
     6,00        8 .  012334
     9,00        8 .  666788999
     3,00        9 .  111

 Stem width:  10,00
 Each leaf:       1 case(s)
```

```
LIN Stem-and-Leaf Plot for
Gruplar= 6,00

 Frequency    Stem &  Leaf

     1,00        4 .  9
     7,00        5 .  0001244
     5,00        5 .  68889
     6,00        6 .  001114
      ,00        6 .
     1,00        7 .  0

 Stem width:  10,00
 Each leaf:       1 case(s)
```

Normal Q-Q Plots

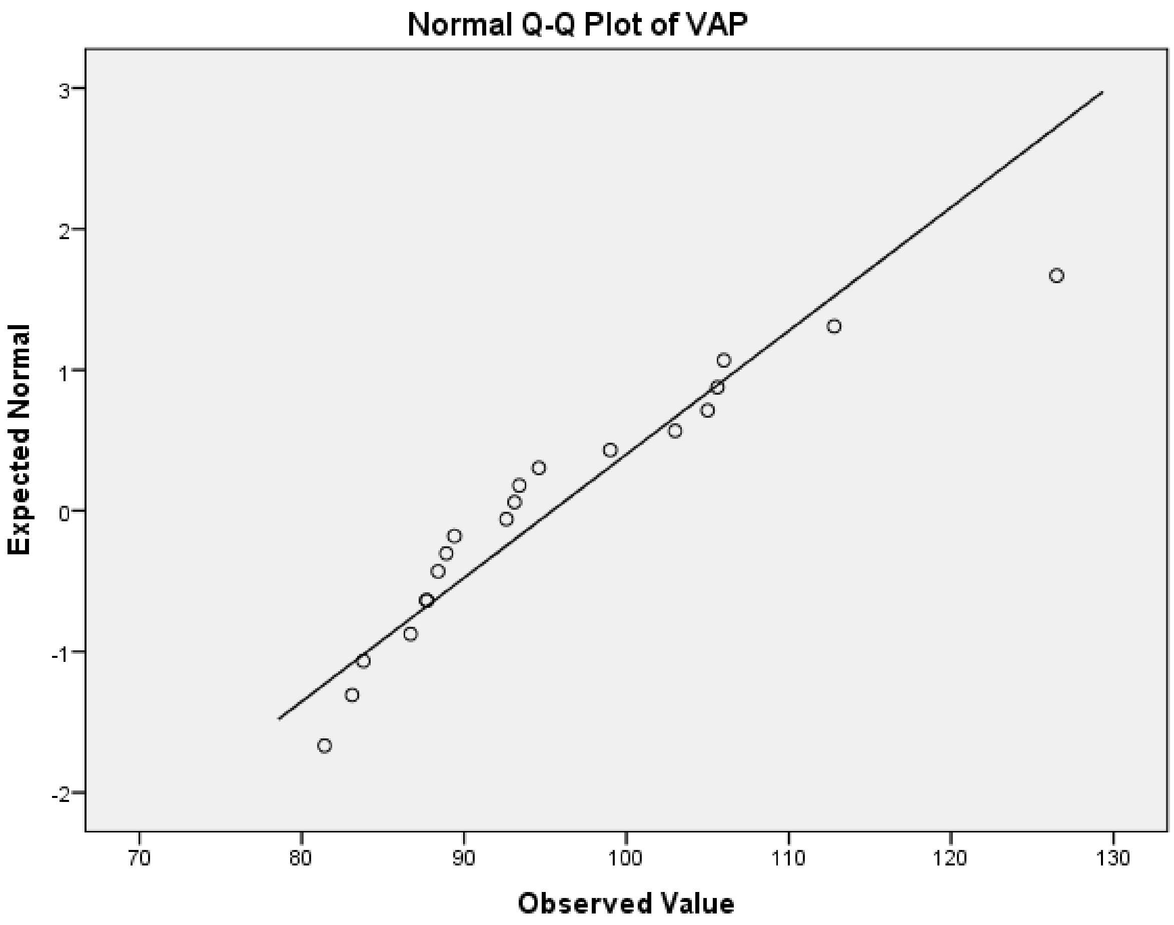

Normal Q-Q Plot of VAP
Expected Normal
3
2
1
0
-1
-2
70
80
90
100
110
120
130
Observed Value

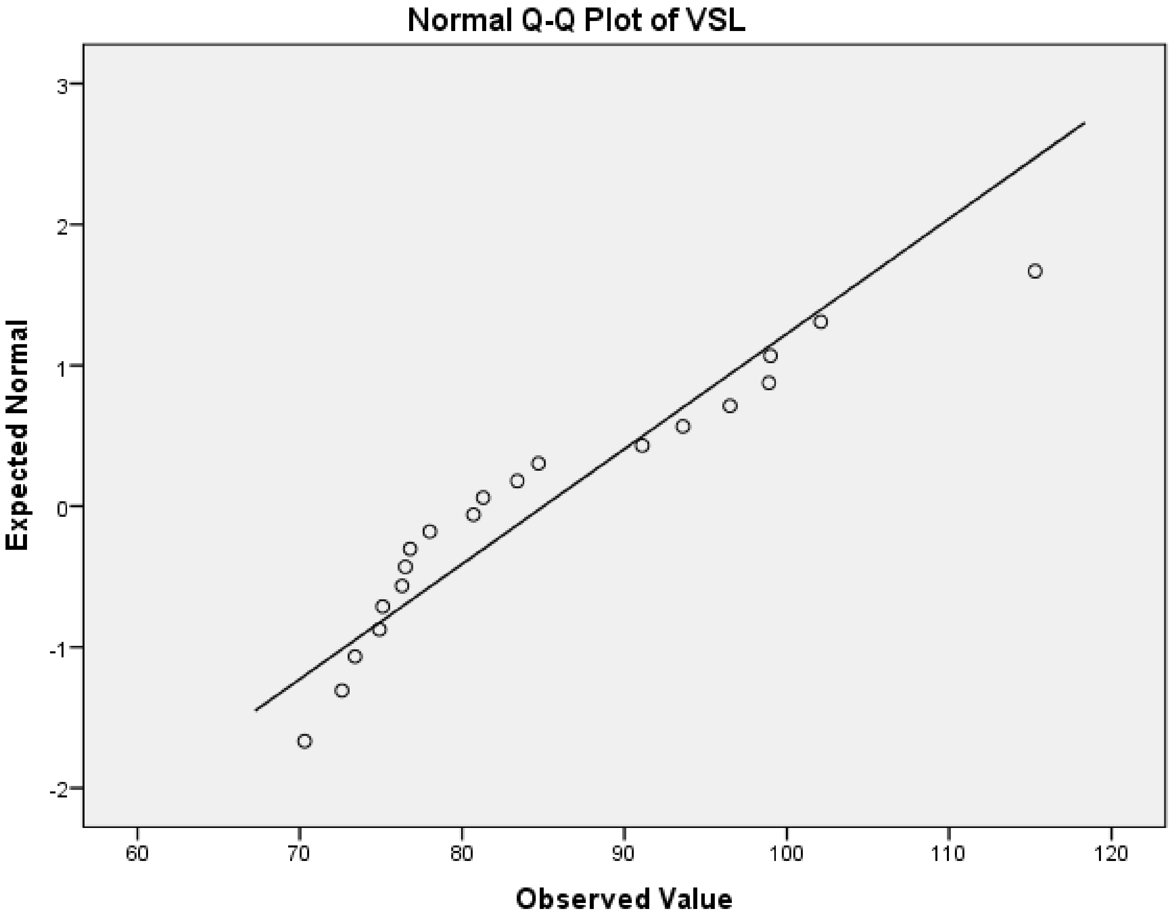
Normal Q-Q Plot of VSL
Expected Normal
3
2
1
0
-1
-2
60
70
80
90
100
110
120
Observed Value

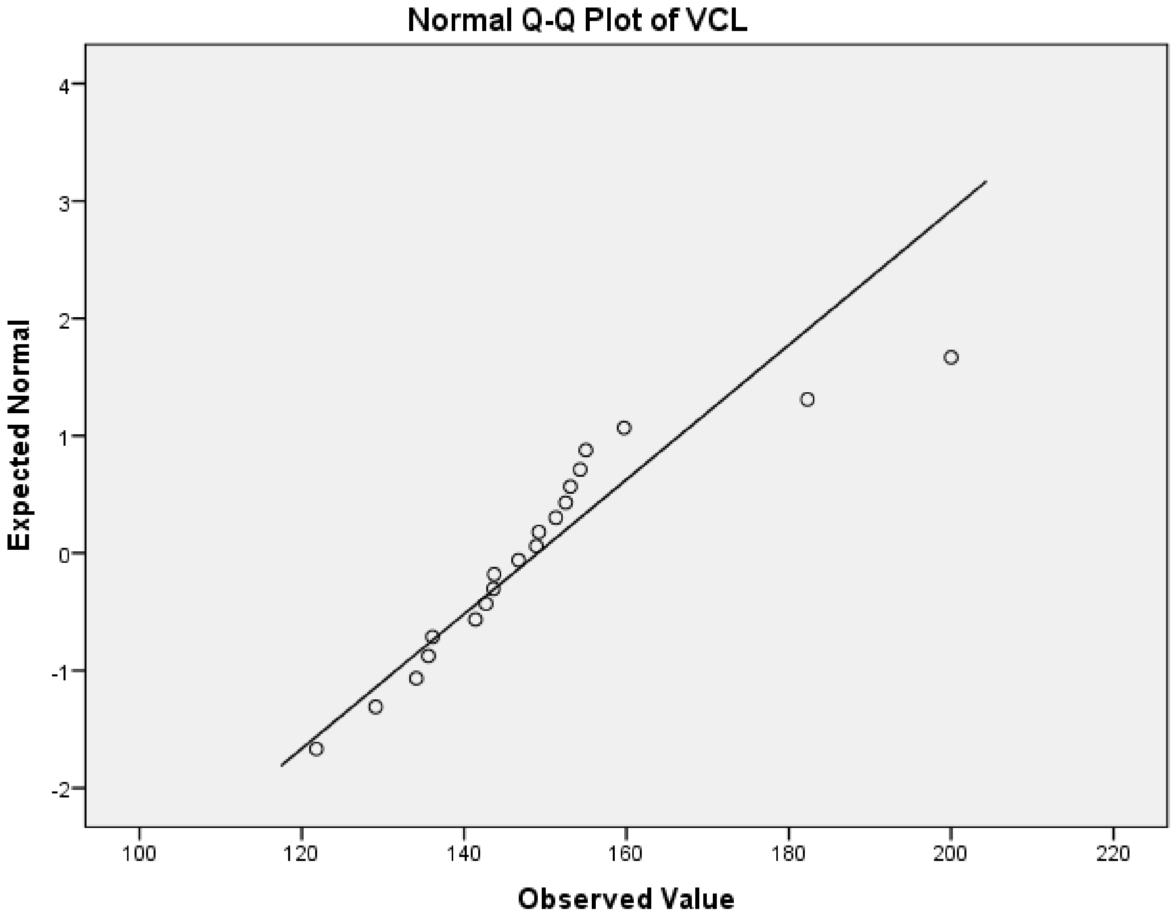
Normal Q-Q Plot of VCL
Expected Normal
Observed Value
4
3
2
1
0
-1
-2
100
120
140
160
180
200
220

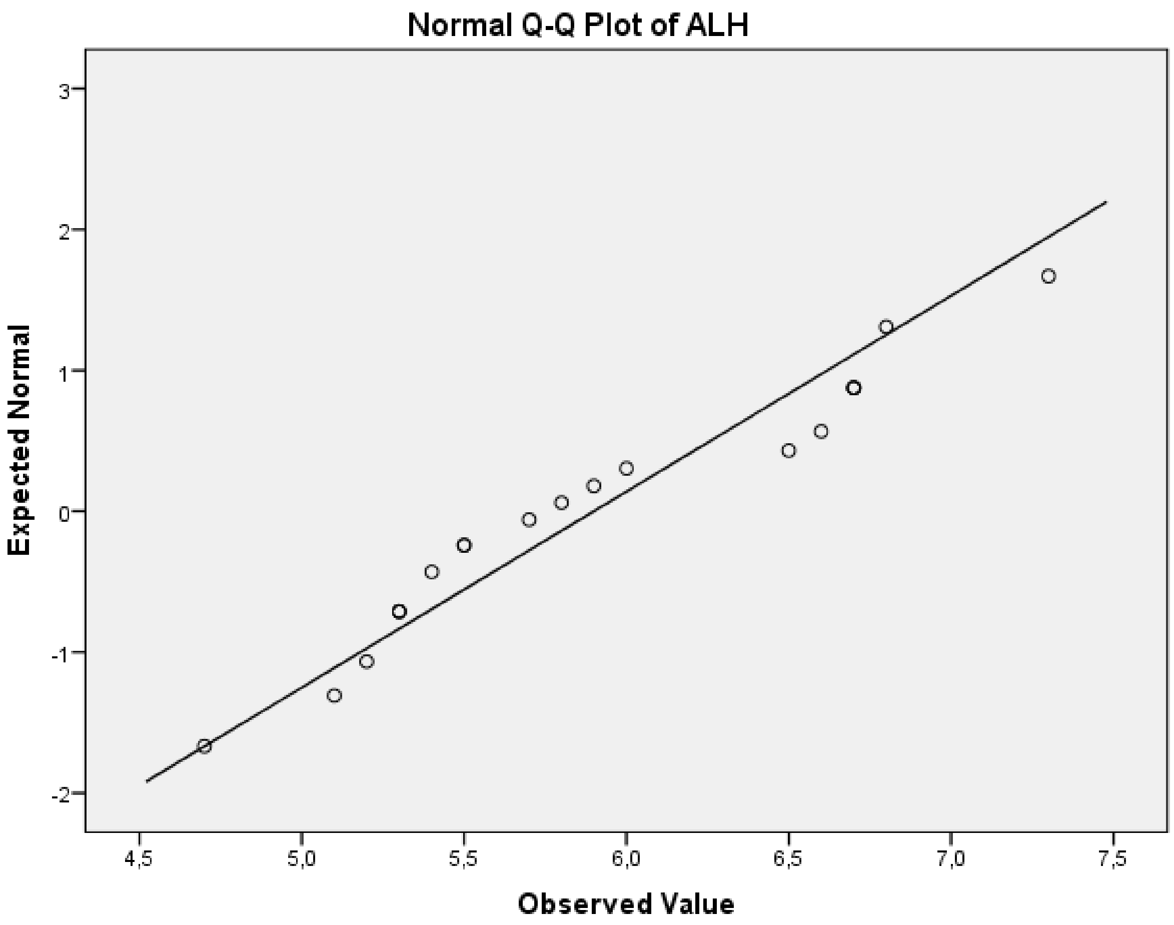
Normal Q-Q Plot of ALH
Expected Normal
3
2
1
0
-1
-2
4,5
5,0
5,5
6,0
6,5
7,0
7,5
Observed Value

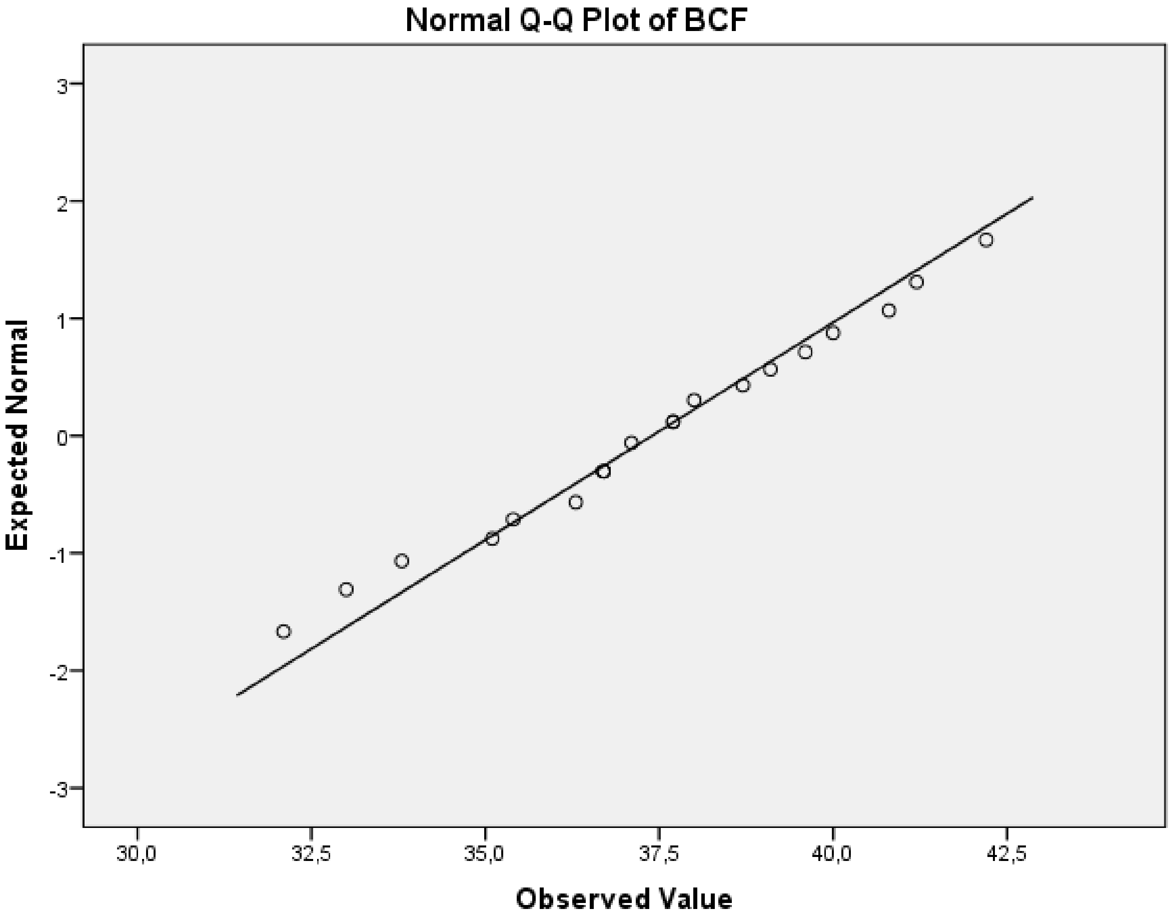

Normal Q-Q Plot of BCF
Expected Normal
3
2
1
0
-1
-2
-3
30,0
32,5
35,0
37,5
40,0
42,5
Observed Value

Normal Q-Q Plot of STR

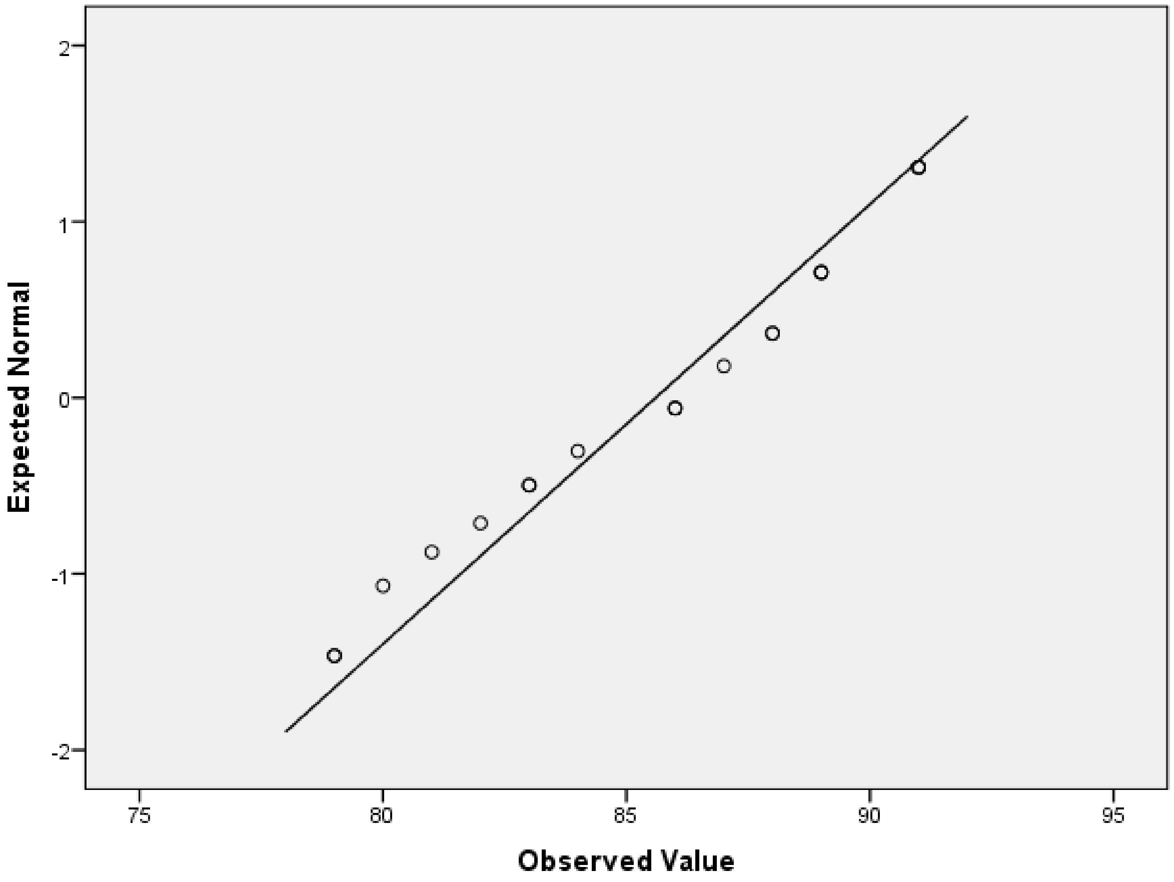

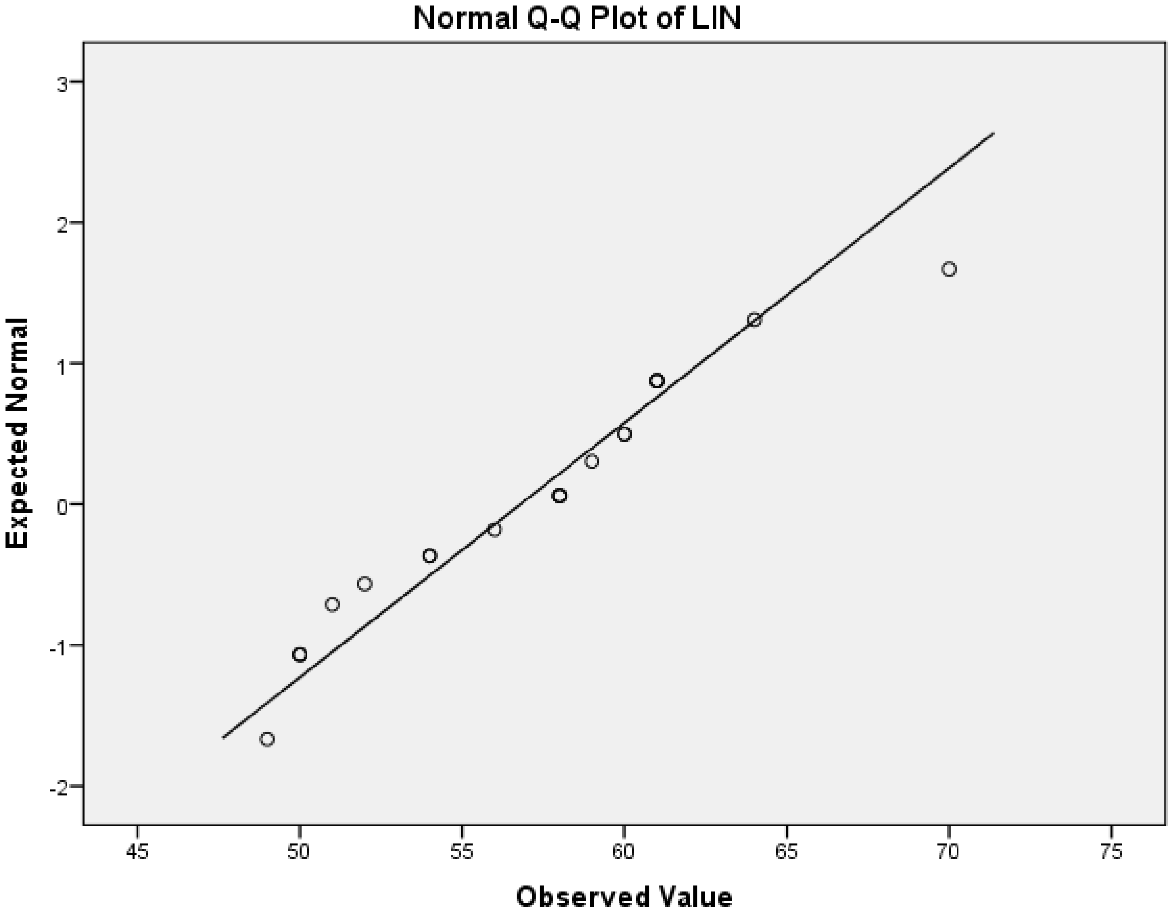

Detrended Normal Q-Q Plots

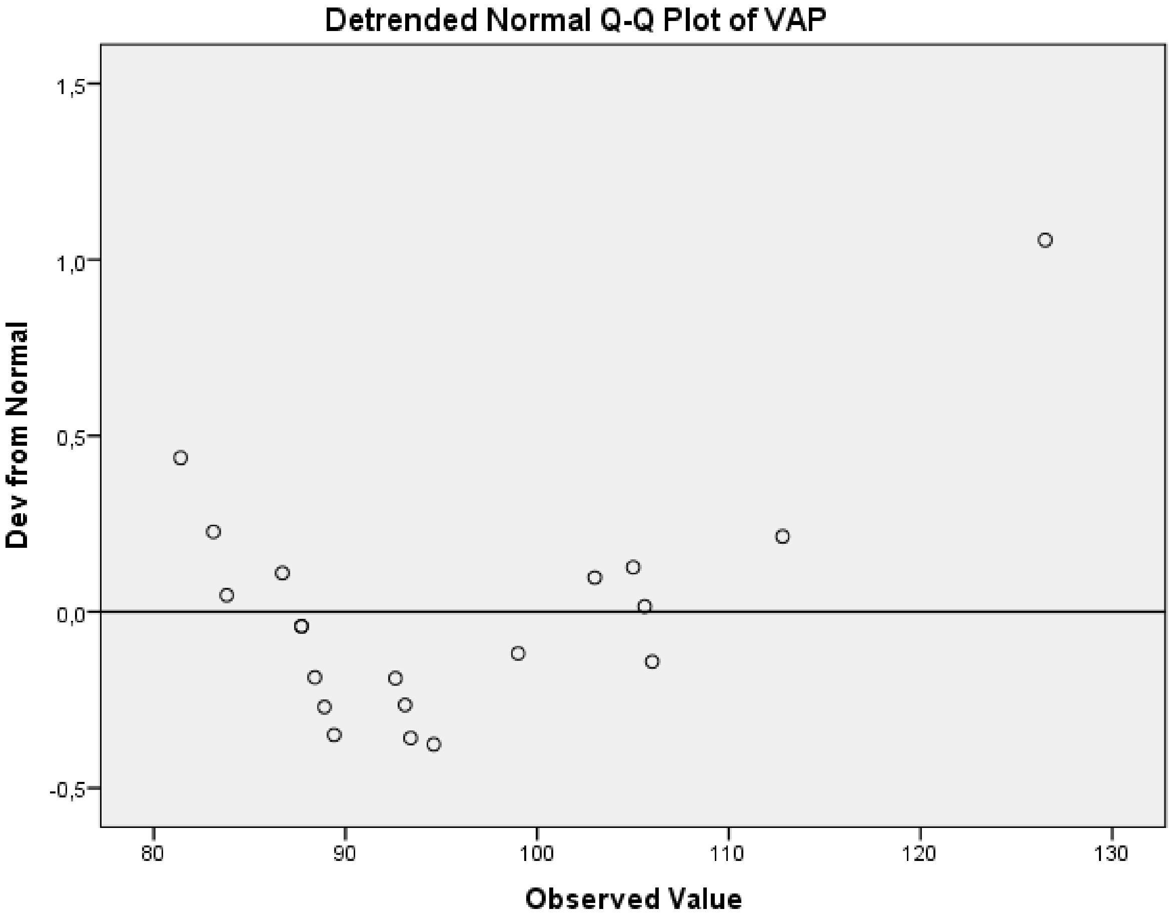

Detrended Normal Q-Q Plot of VAP
Dev from Normal
1,5
1,0
0,5
0,0
-0,5
80
90
100
110
120
130
Observed Value

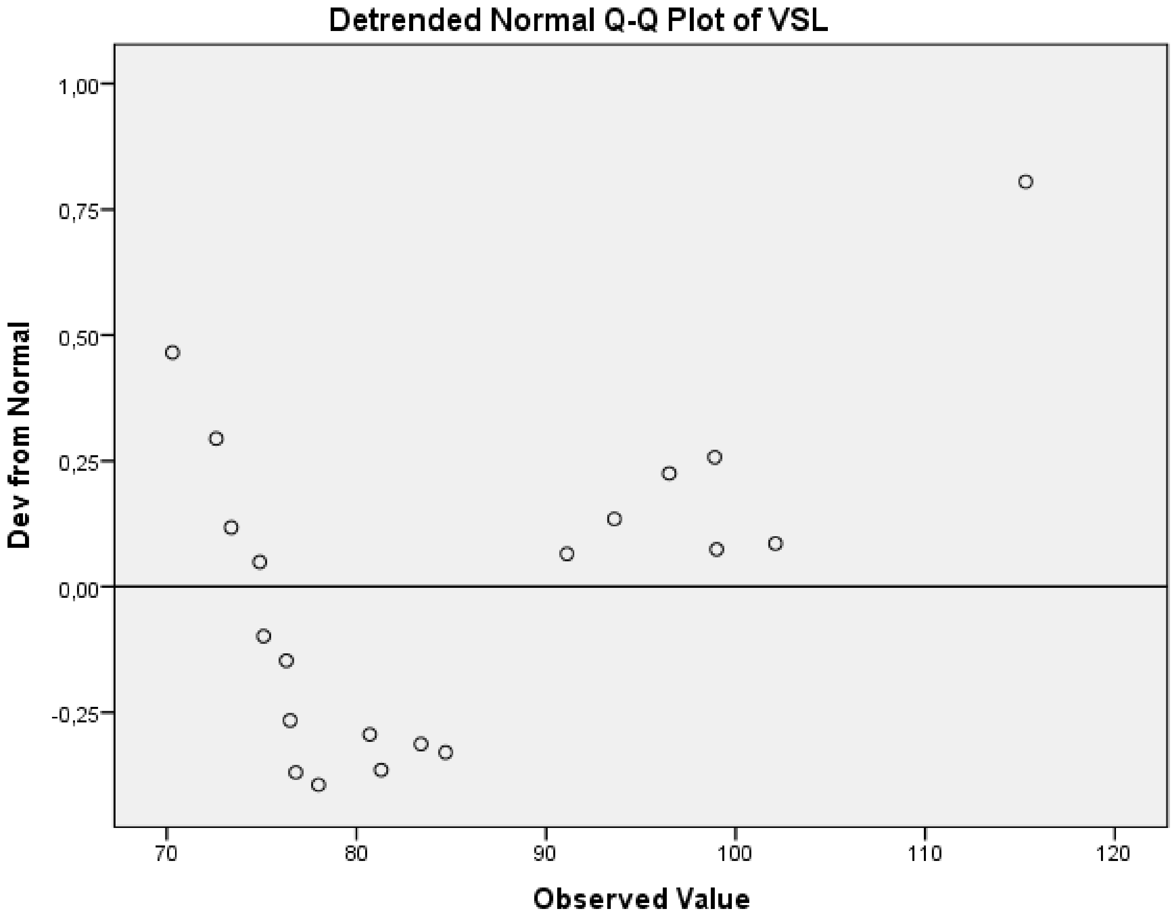

Detrended Normal Q-Q Plot of VSL
Dev from Normal
1,00
0,75
0,50
0,25
0,00
-0,25
70
80
90
100
110
120
Observed Value

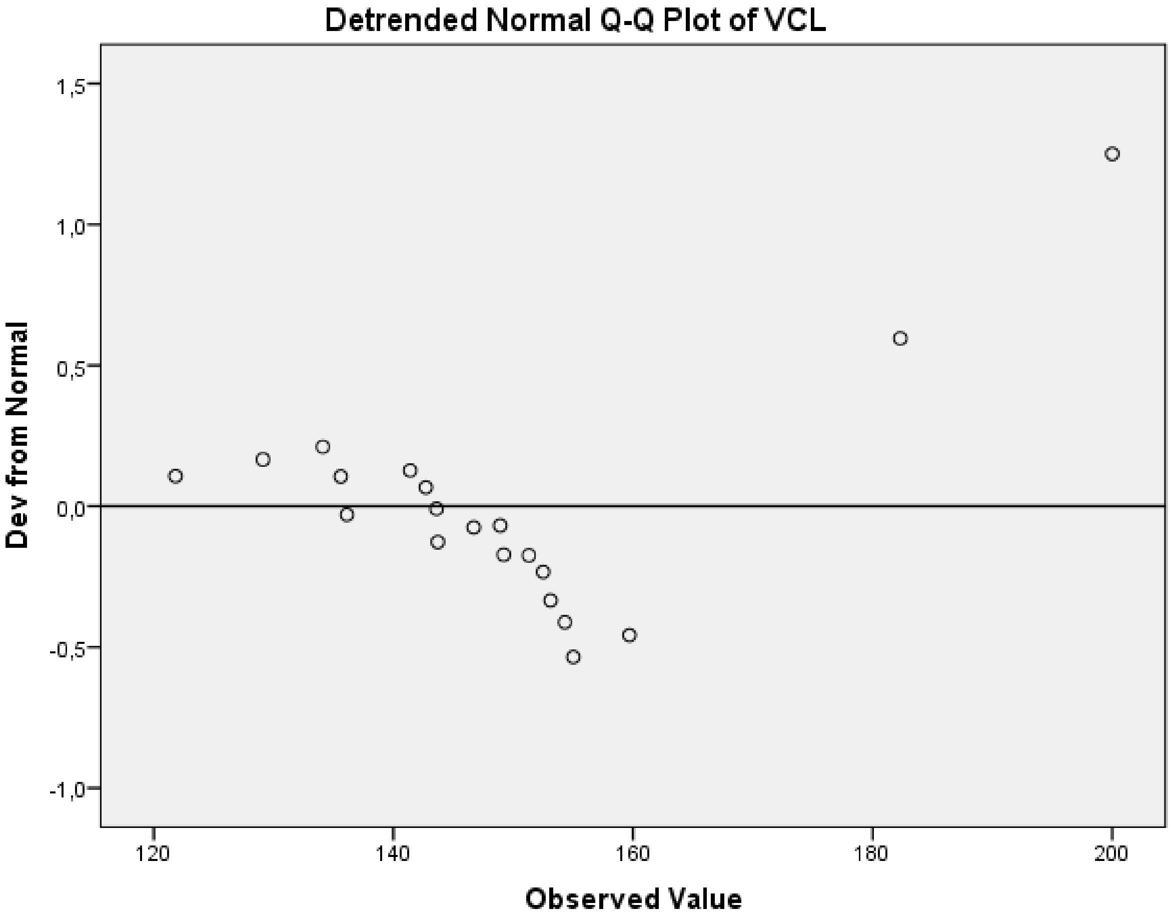
Detrended Normal Q-Q Plot of VCL
Dev from Normal
1,5
1,0
0,5
0,0
-0,5
-1,0
120
140
160
180
200
Observed Value

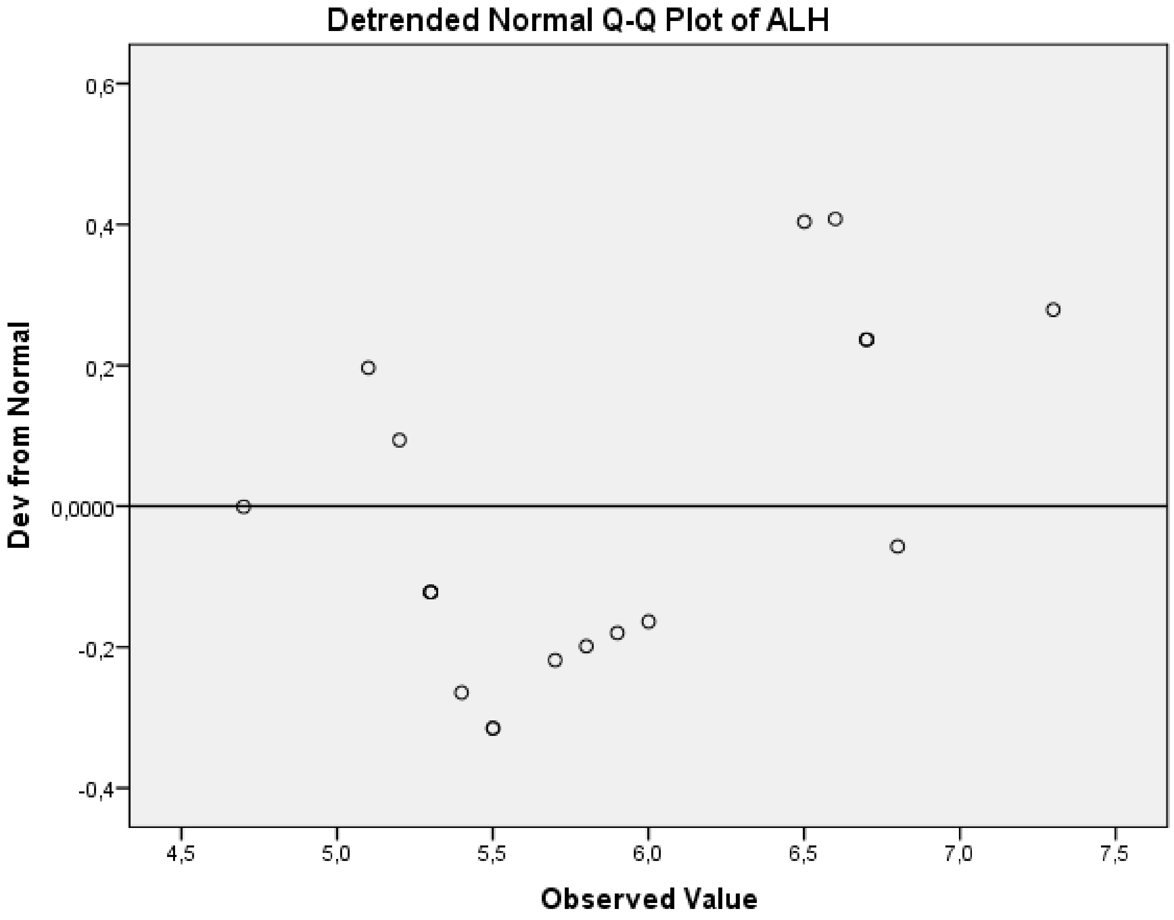
Detrended Normal Q-Q Plot of ALH
Dev from Normal
0,6
0,4
0,2
0,0000
-0,2
-0,4
4,5
5,0
5,5
6,0
6,5
7,0
7,5
Observed Value

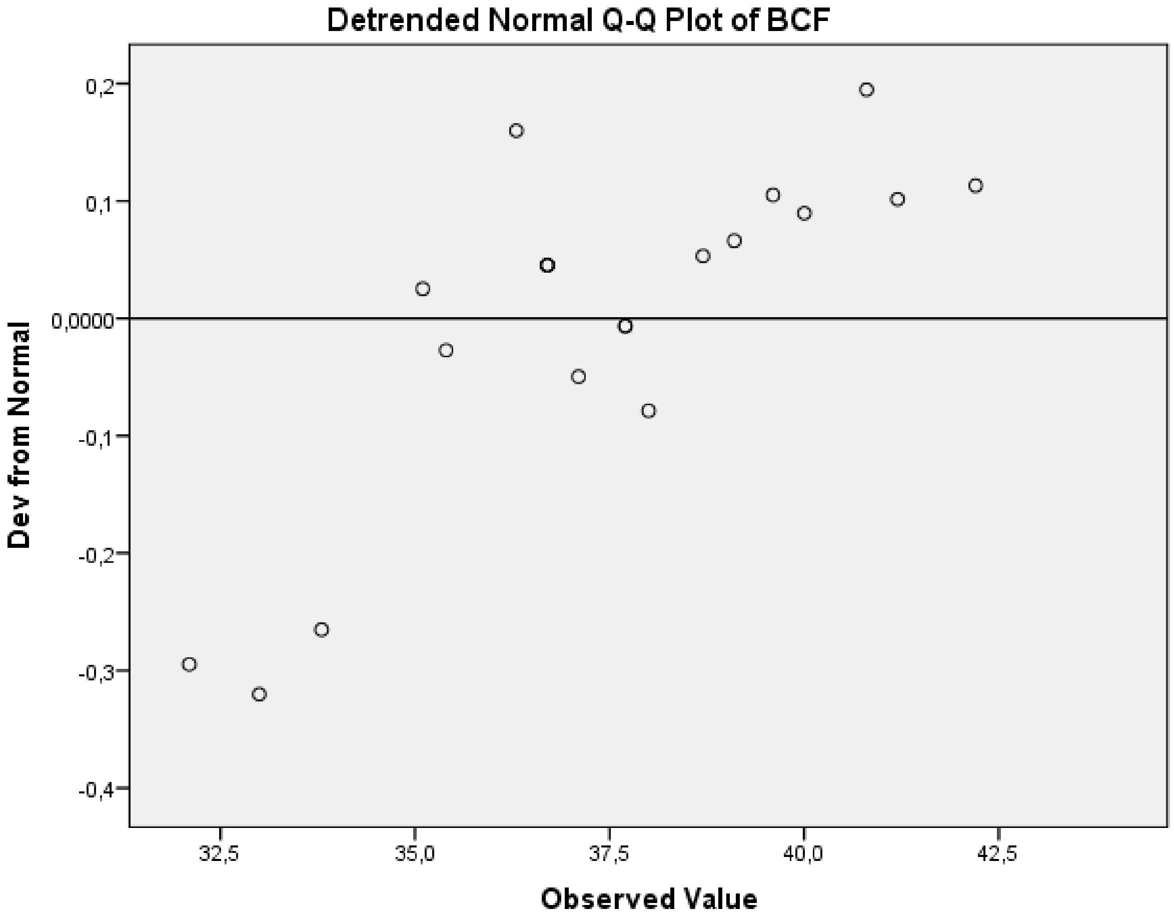

Detrended Normal Q-Q Plot of BCF
Dev from Normal
0,2
0,1
0,0000
-0,1
-0,2
-0,3
-0,4
32,5
35,0
37,5
40,0
42,5
Observed Value

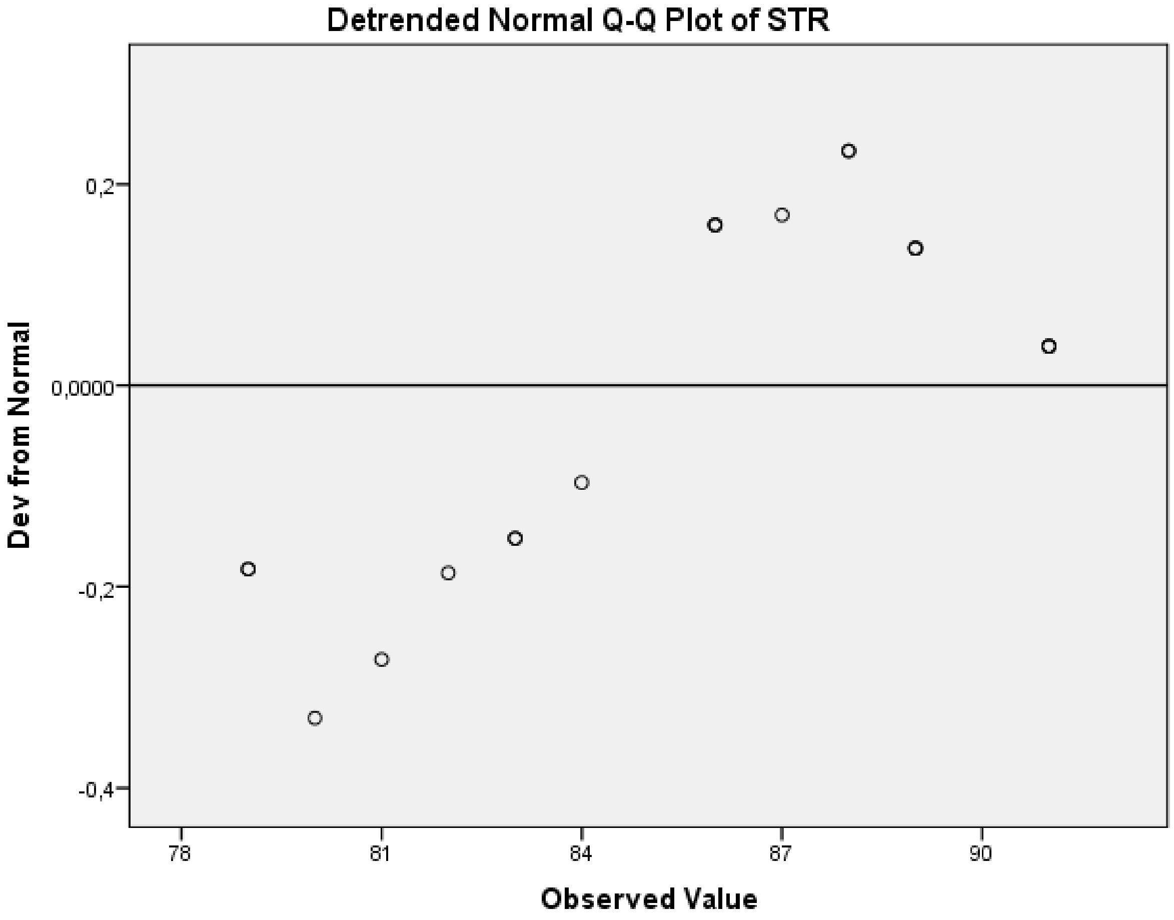
Detrended Normal Q-Q Plot of STR
Dev from Normal
0,2
0,0000
-0,2
-0,4
78
81
84
87
90
Observed Value

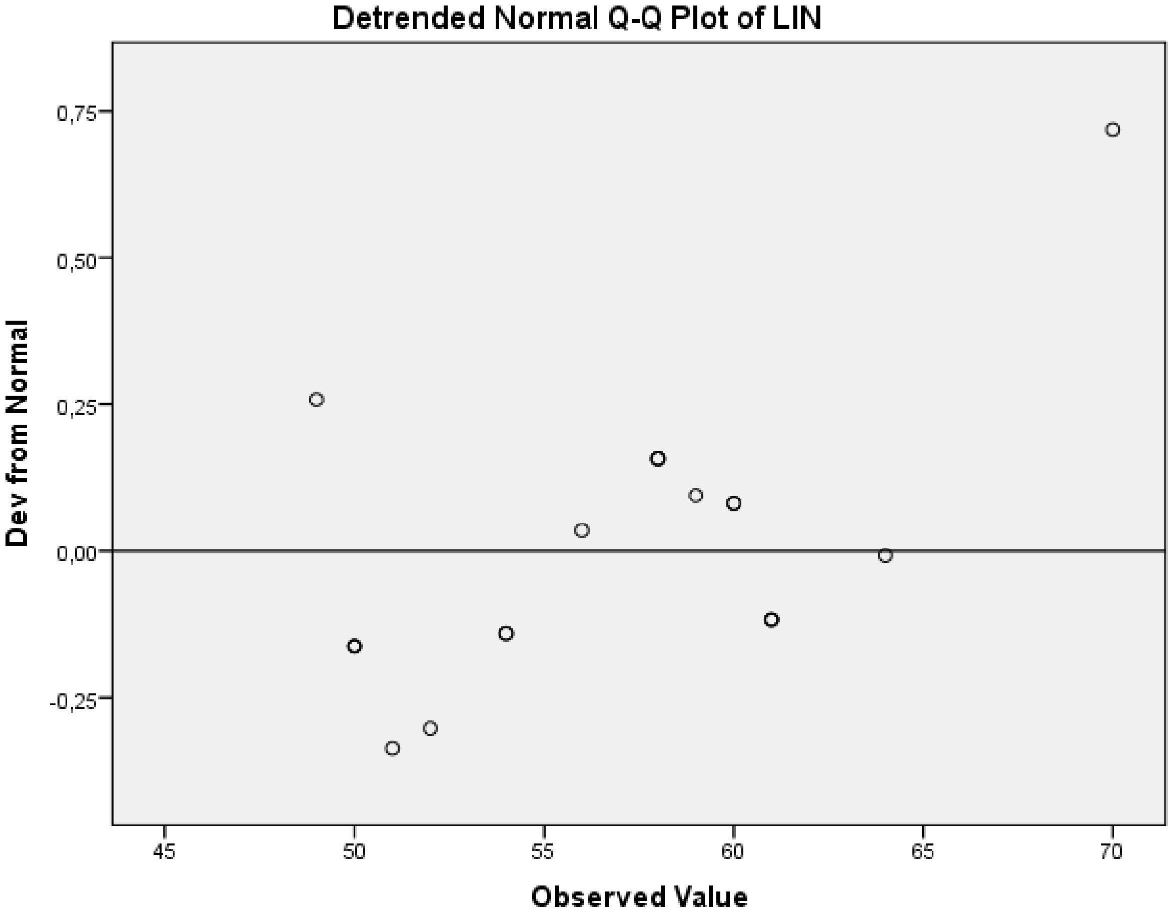

Gruplar = 7,00

Histograms

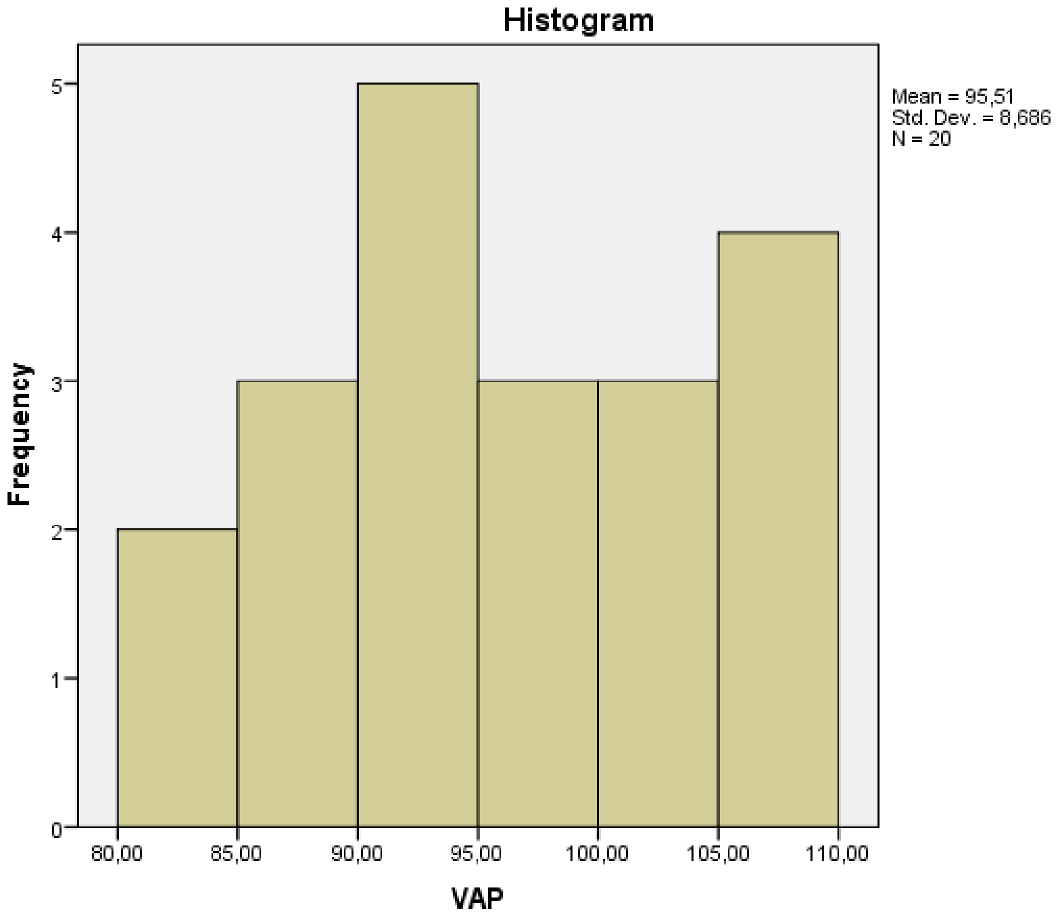
Histogram
Mean = 95,51
Std. Dev. = 8,686
N = 20
Frequency
0
1
2
3
4
5
80,00
85,00
90,00
95,00
100,00
105,00
110,00
VAP

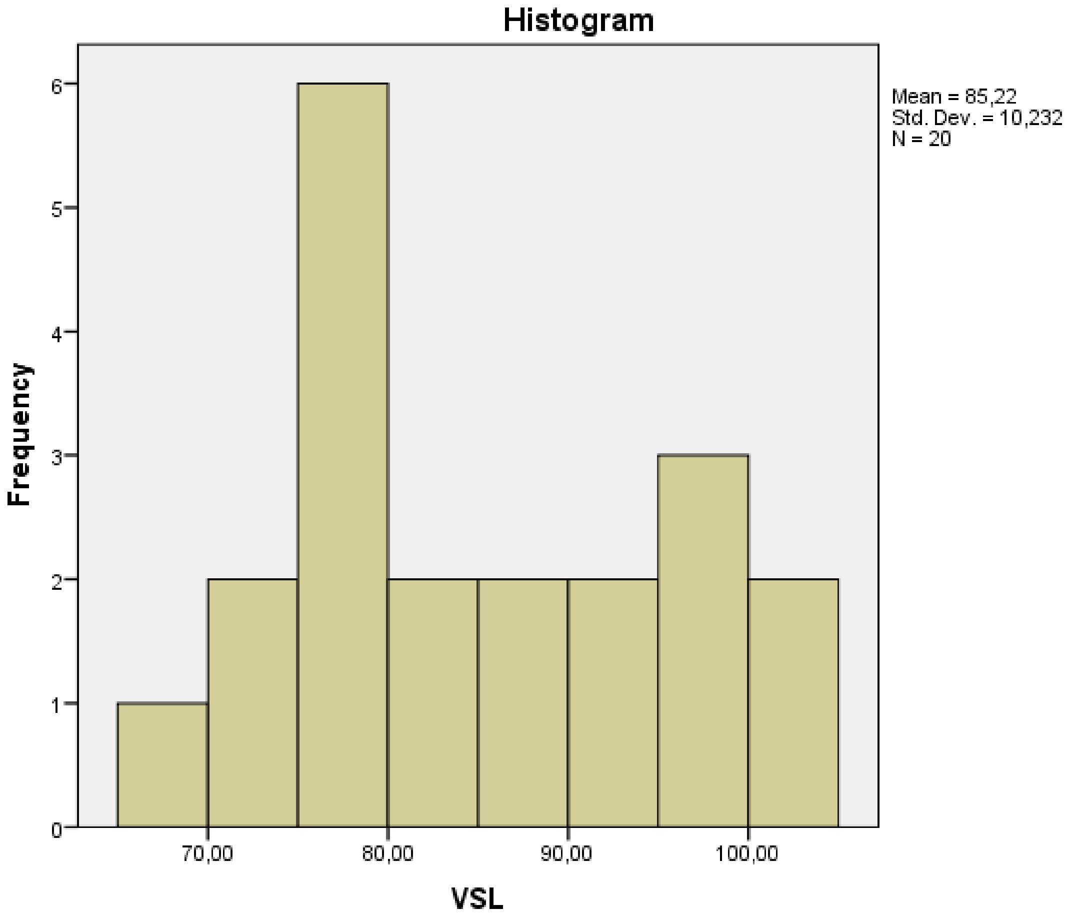
Histogram
Mean = 85,22
Std. Dev. = 10,232
N = 20
Frequency
0
1
2
3
4
5
6
70,00
80,00
90,00
100,00
VSL

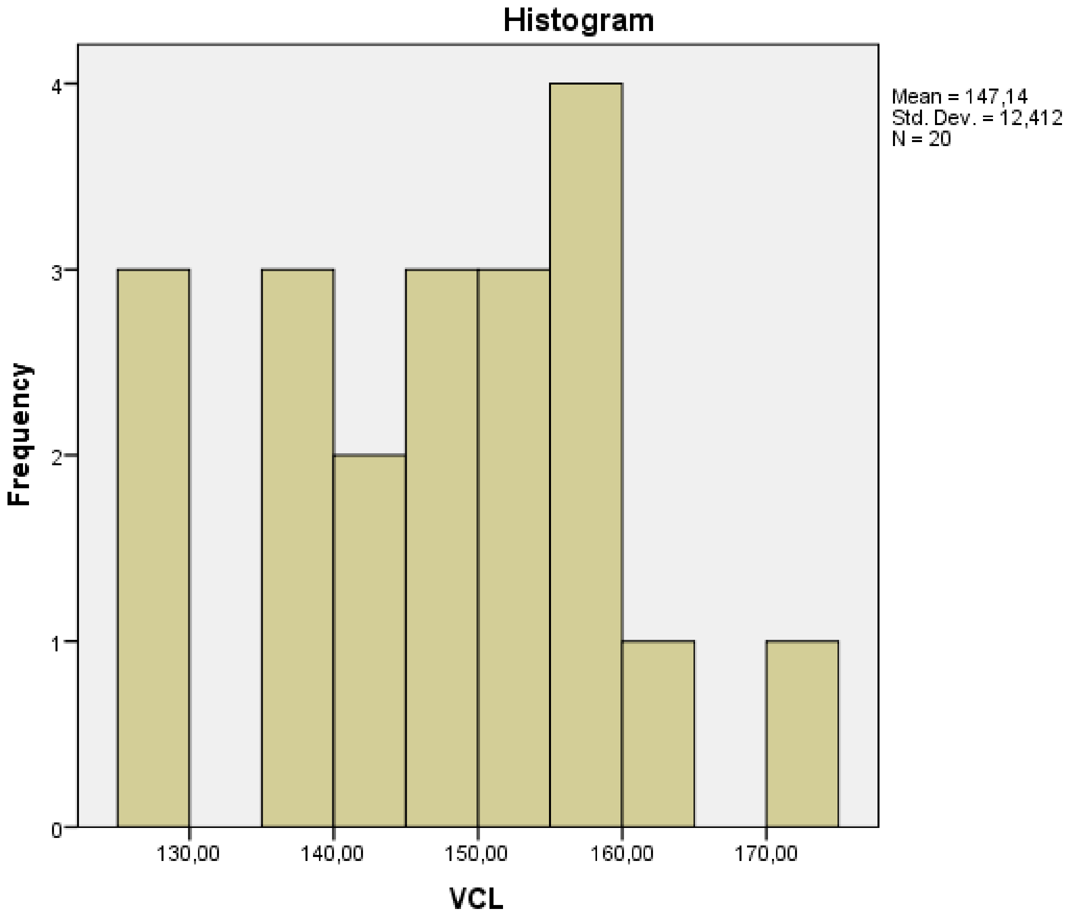

Histogram
Mean = 147,14
Std. Dev. = 12,412
N = 20
Frequency
0
1
2
3
4
130,00
140,00
150,00
160,00
170,00
VCL

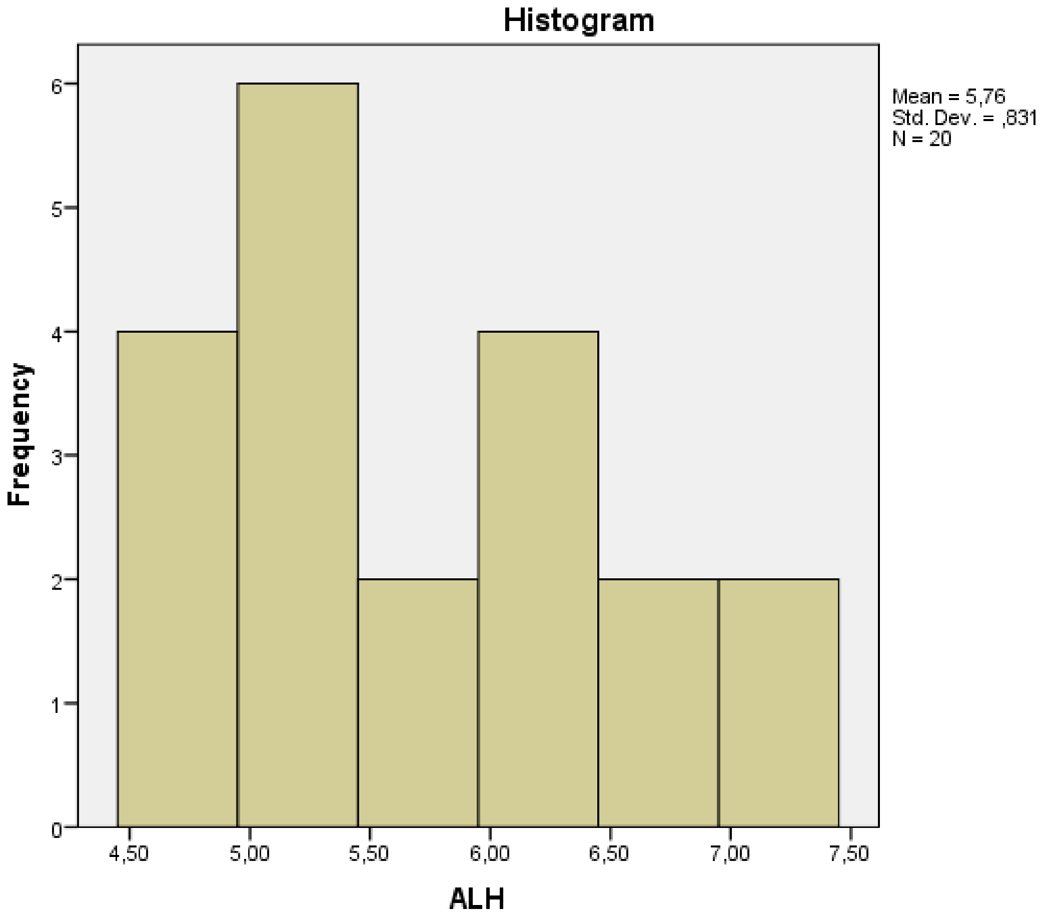
Histogram
Mean = 5,76
Std. Dev. = ,831
N = 20
Frequency
0
1
2
3
4
5
6
4,50
5,00
5,50
6,00
6,50
7,00
7,50
ALH

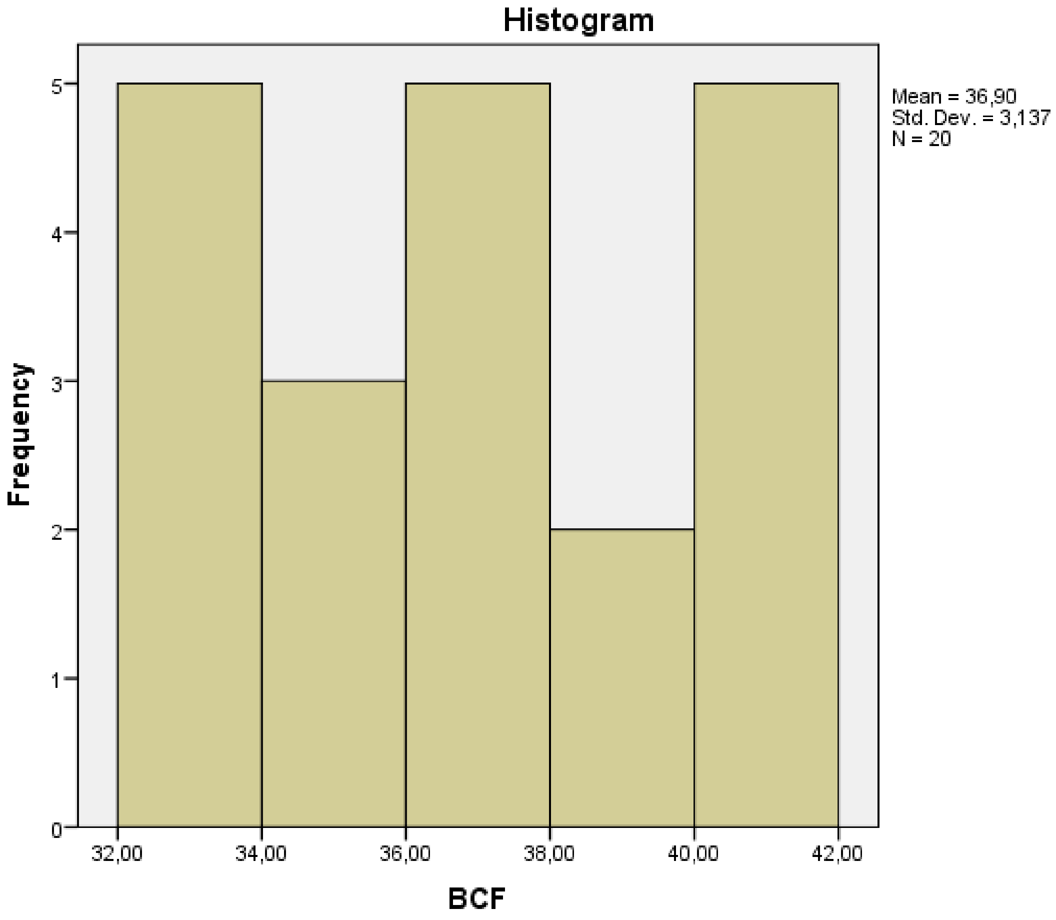
Histogram
Mean = 36,90
Std. Dev. = 3,137
N = 20
Frequency
0
1
2
3
4
5
32,00
34,00
36,00
38,00
40,00
42,00
BCF

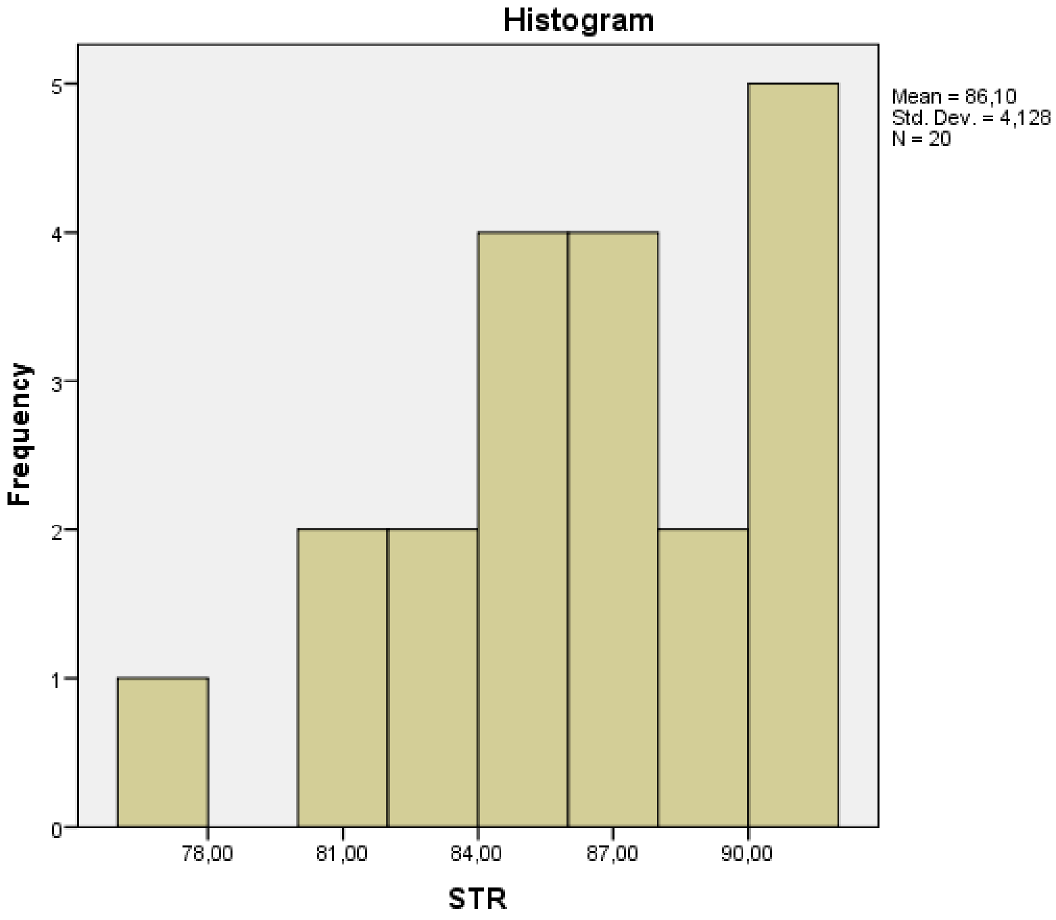
Histogram
Mean = 86,10
Std. Dev. = 4,128
N = 20
Frequency
0
1
2
3
4
5
78,00
81,00
84,00
87,00
90,00
STR

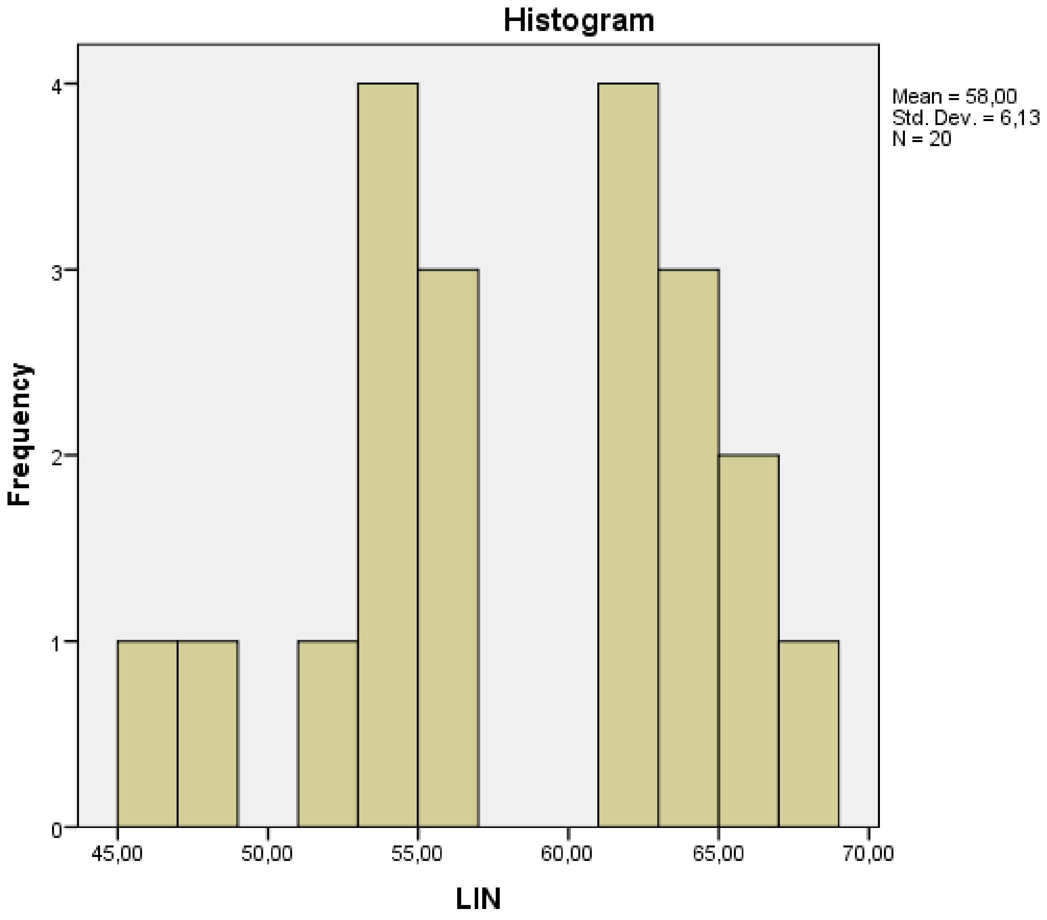

Stem-and-Leaf Plots

```
VAP Stem-and-Leaf Plot for
Gruplar= 7,00

 Frequency    Stem &  Leaf

     2,00        8 .  03
     3,00        8 .  577
     5,00        9 .  11114
     3,00        9 .  579
     3,00       10 .  013
     4,00       10 .  5689

 Stem width:  10,00
 Each leaf:       1 case(s)
```

```
VSL Stem-and-Leaf Plot for
Gruplar= 7,00

 Frequency    Stem &  Leaf

     1,00        6 .  8
     8,00        7 .  24678889
     4,00        8 .  1378
     5,00        9 .  01669
     2,00       10 .  02

 Stem width:  10,00
 Each leaf:       1 case(s)
```

```
VCL Stem-and-Leaf Plot for
Gruplar= 7,00

 Frequency    Stem &  Leaf

     3,00       12 .  678
     3,00       13 .  578
     5,00       14 .  02789
     7,00       15 .  0135799
     1,00       16 .  2
     1,00       17 .  0

 Stem width:  10,00
 Each leaf:       1 case(s)
```

```
ALH Stem-and-Leaf Plot for
Gruplar= 7,00

 Frequency    Stem &  Leaf

     4,00        4 .  7799
     6,00        5 .  022234
     2,00        5 .  88
     4,00        6 .  1344
     2,00        6 .  56
     2,00        7 .  34

 Stem width:   1,00
 Each leaf:       1 case(s)
```

```
BCF Stem-and-Leaf Plot for
Gruplar= 7,00

 Frequency    Stem &  Leaf

     6,00        3 .  222334
     9,00        3 .  556677799
     5,00        4 .  00111

 Stem width:  10,00
 Each leaf:       1 case(s)
```

```
STR Stem-and-Leaf Plot for
Gruplar= 7,00

 Frequency    Stem &  Leaf

     1,00        7 .  7
     5,00        8 .  00334
     9,00        8 .  555677799
     5,00        9 .  11111

 Stem width:  10,00
 Each leaf:       1 case(s)
```

```
LIN Stem-and-Leaf Plot for
Gruplar= 7,00

 Frequency    Stem &  Leaf

     2,00        4 .  68
     5,00        5 .  13444
     3,00        5 .  556
     7,00        6 .  1122344
     3,00        6 .  557

 Stem width:  10,00
 Each leaf:       1 case(s)
```

Normal Q-Q Plots

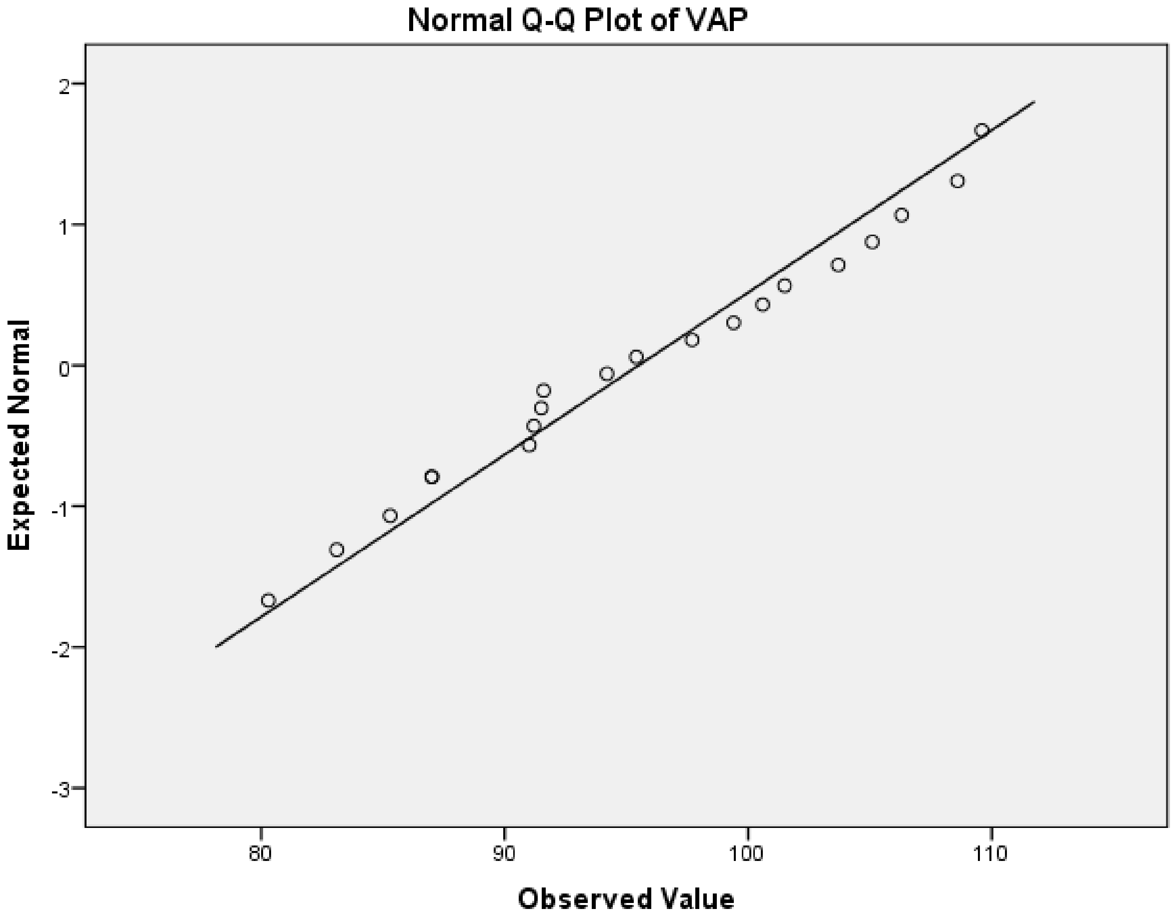
Normal Q-Q Plot of VAP
Expected Normal
Observed Value
2
1
0
-1
-2
-3
80
90
100
110

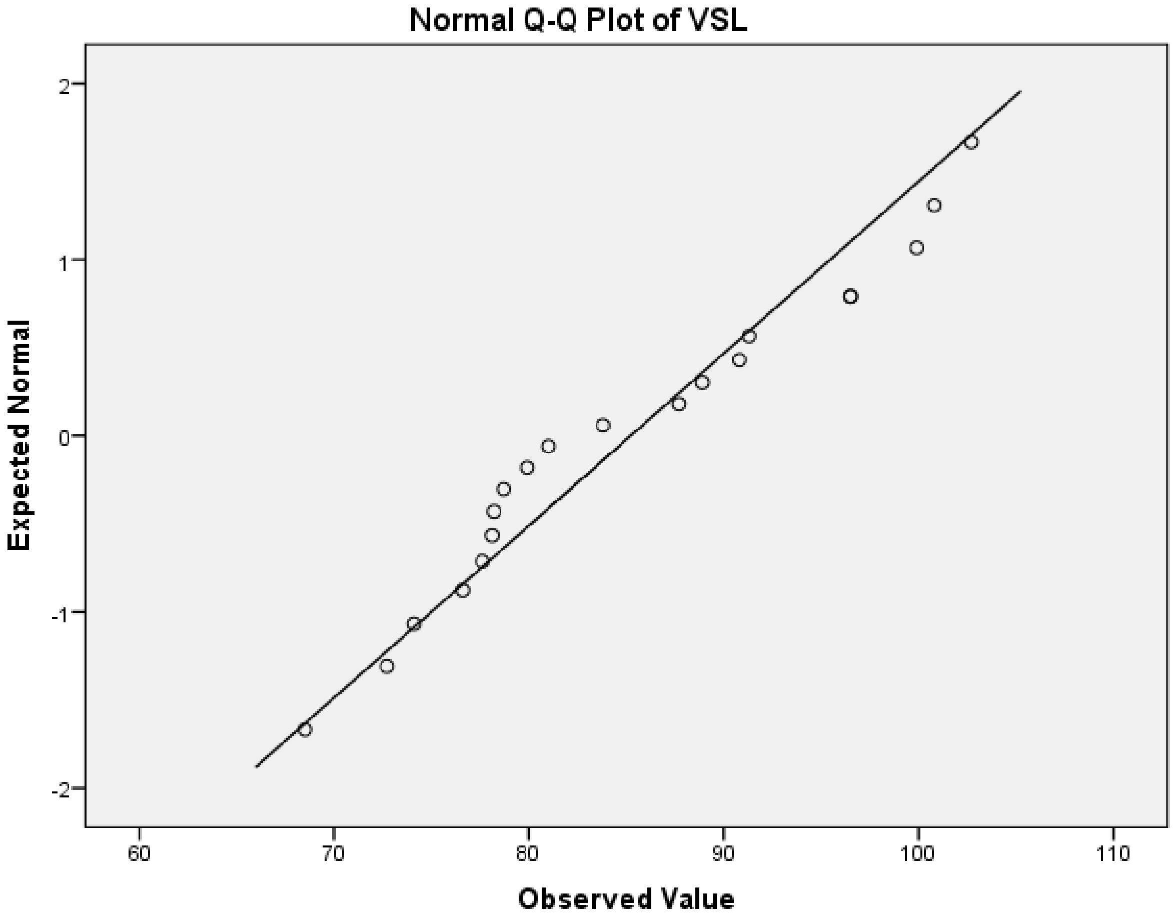

Normal Q-Q Plot of VSL
Expected Normal
Observed Value
2
1
0
-1
-2
60
70
80
90
100
110

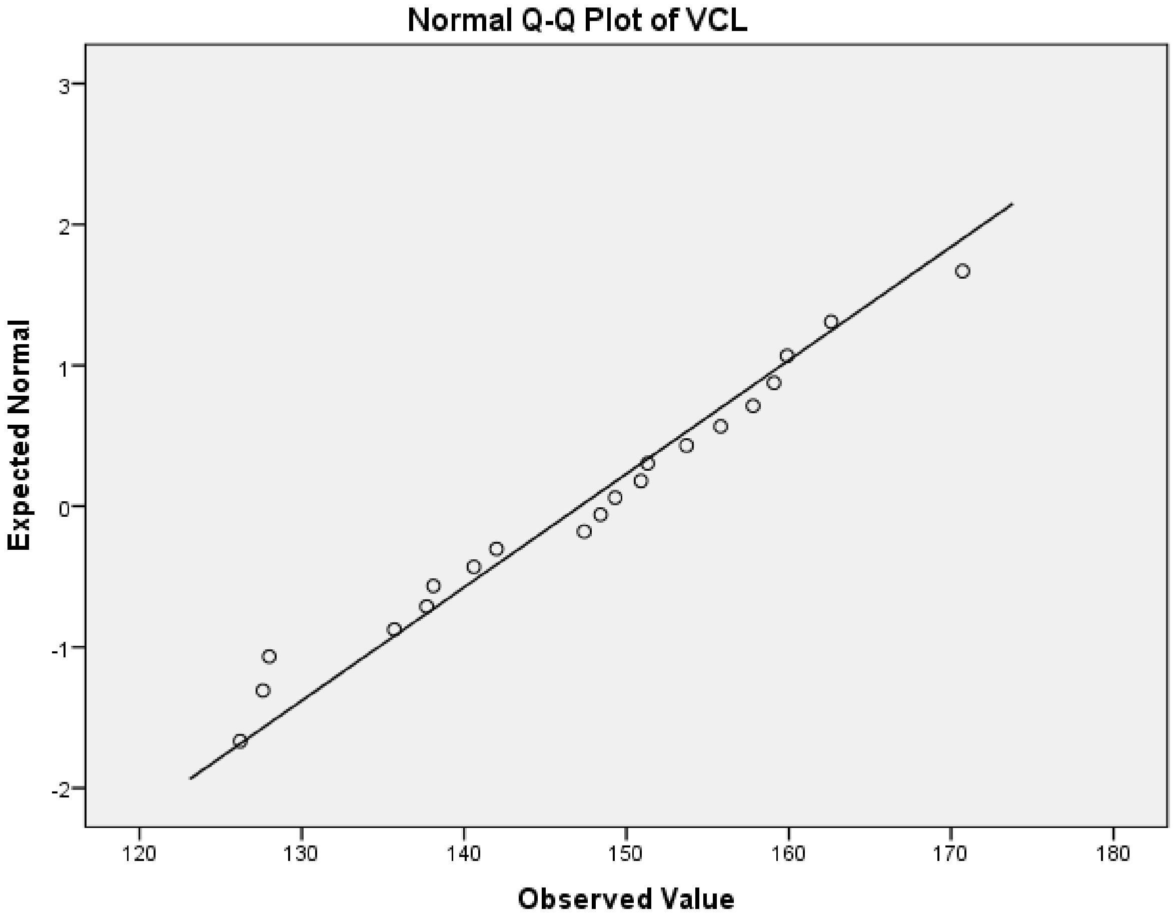

Normal Q-Q Plot of VCL
Expected Normal
3
2
1
0
-1
-2
120
130
140
150
160
170
180
Observed Value

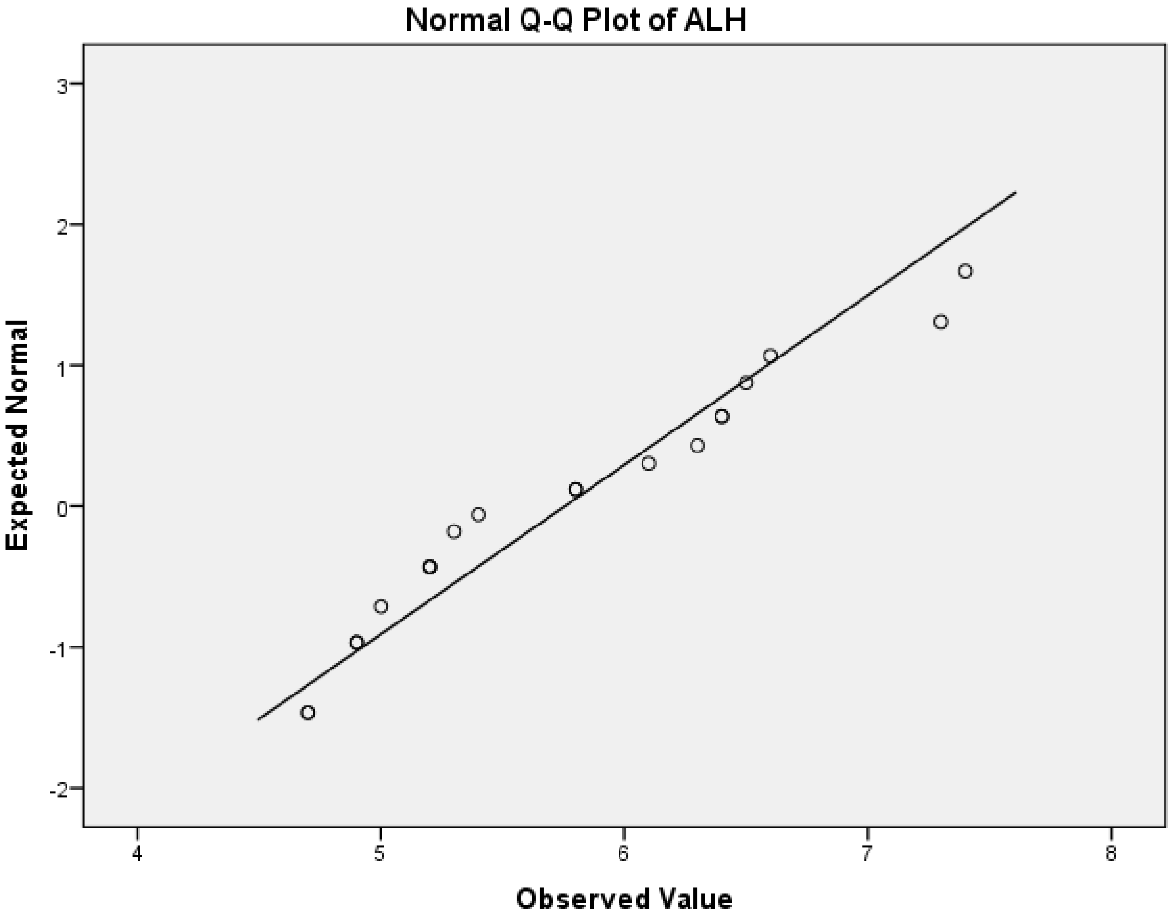
Normal Q-Q Plot of ALH
Expected Normal
Observed Value
3
2
1
0
-1
-2
4
5
6
7
8

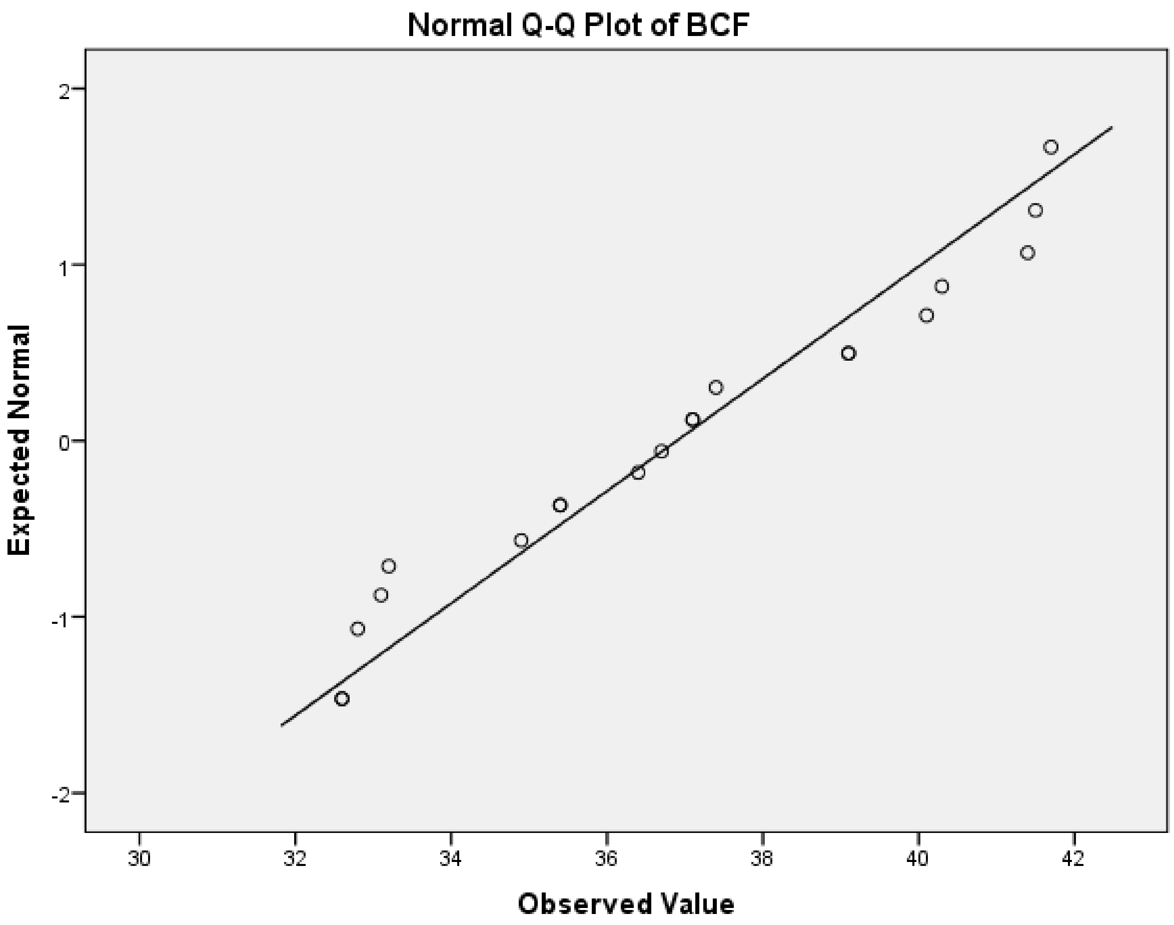
Normal Q-Q Plot of BCF
Expected Normal
Observed Value
2
1
0
-1
-2
30
32
34
36
38
40
42

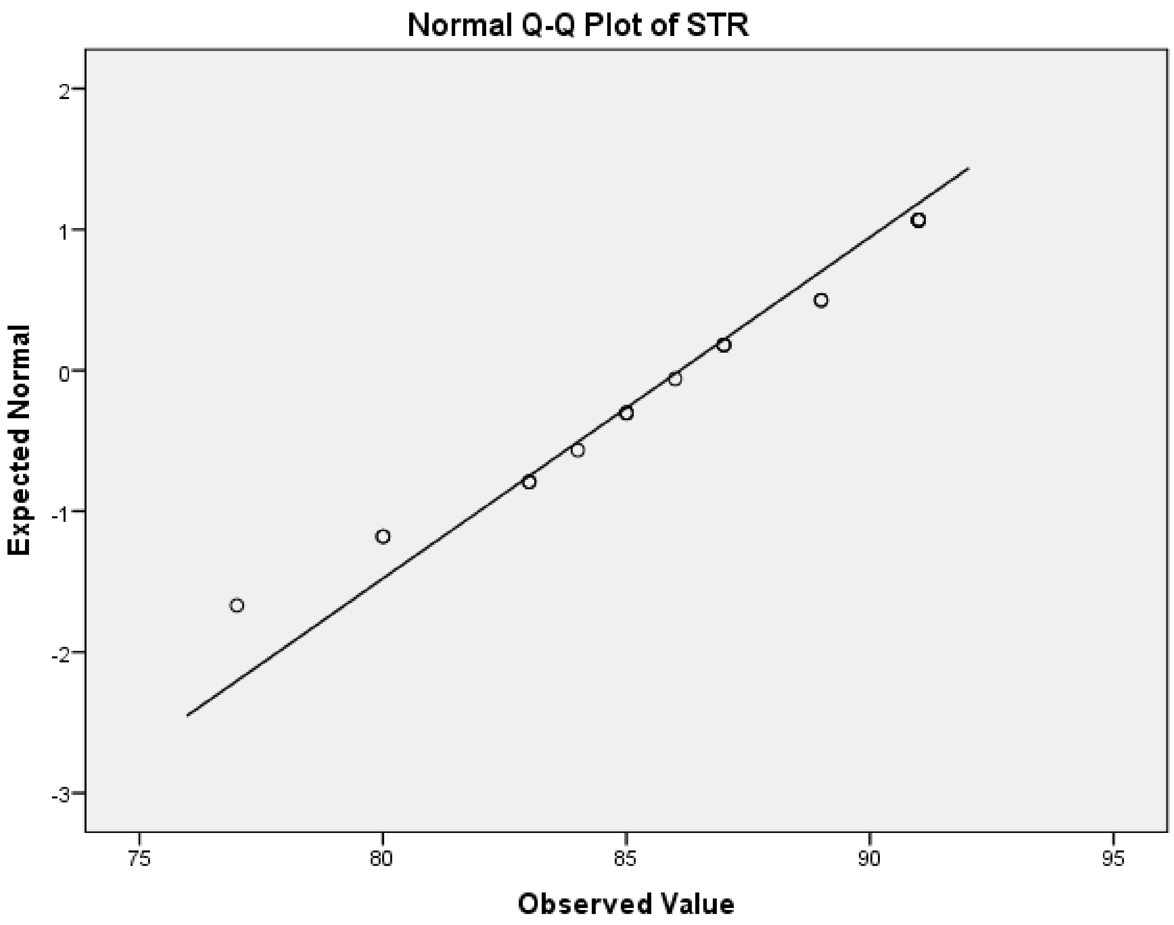

Normal Q-Q Plot of STR
Expected Normal
2
1
0
-1
-2
-3
75
80
85
90
95
Observed Value

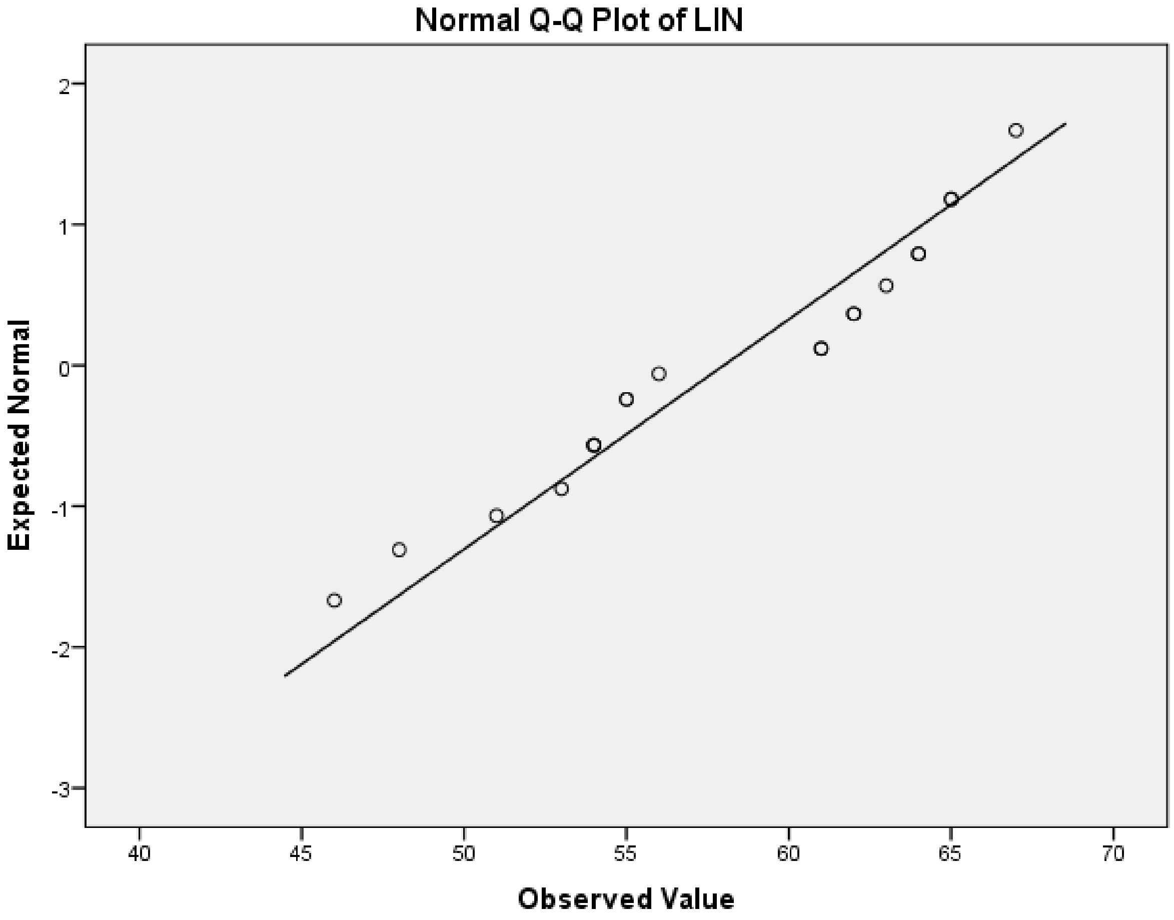

Detrended Normal Q-Q Plots

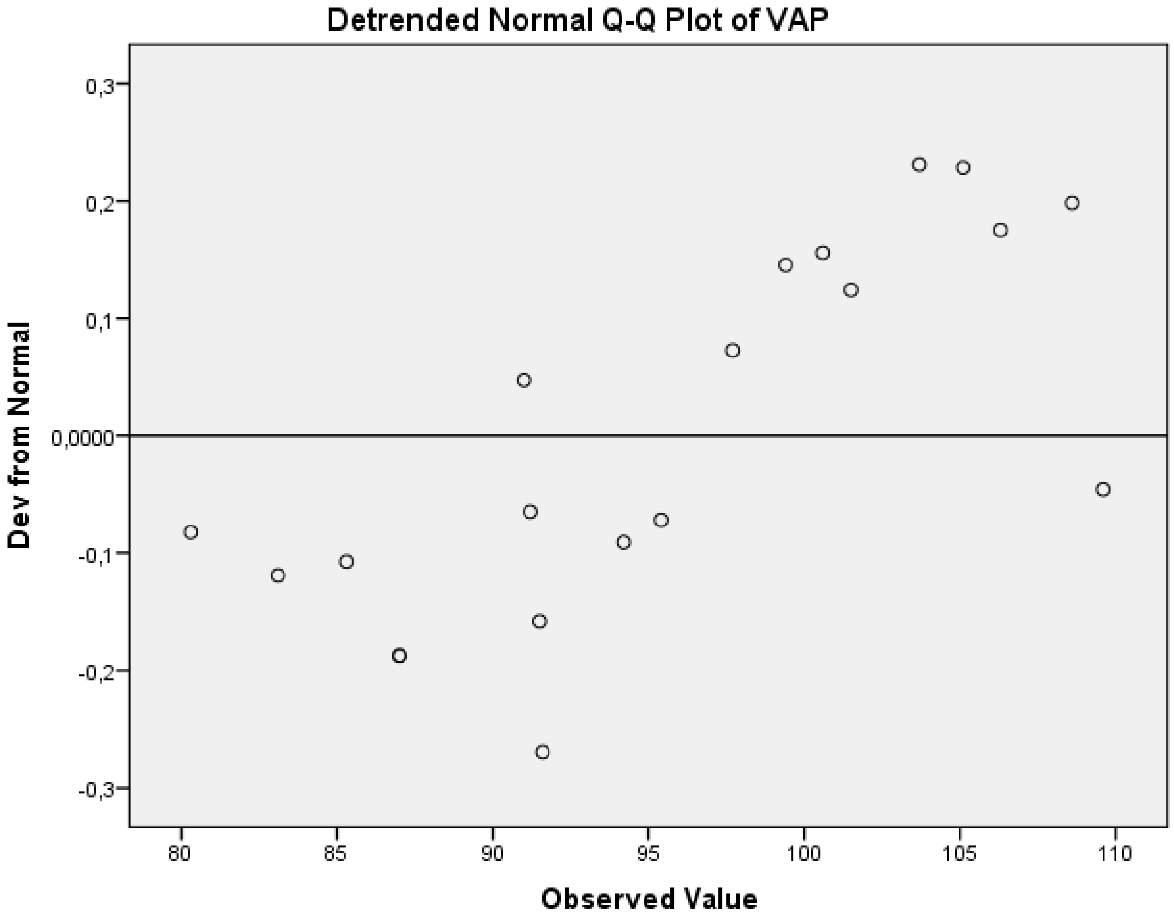

Detrended Normal Q-Q Plot of VAP
Dev from Normal
0,3
0,2
0,1
0,0000
-0,1
-0,2
-0,3
80
85
90
95
100
105
110
Observed Value

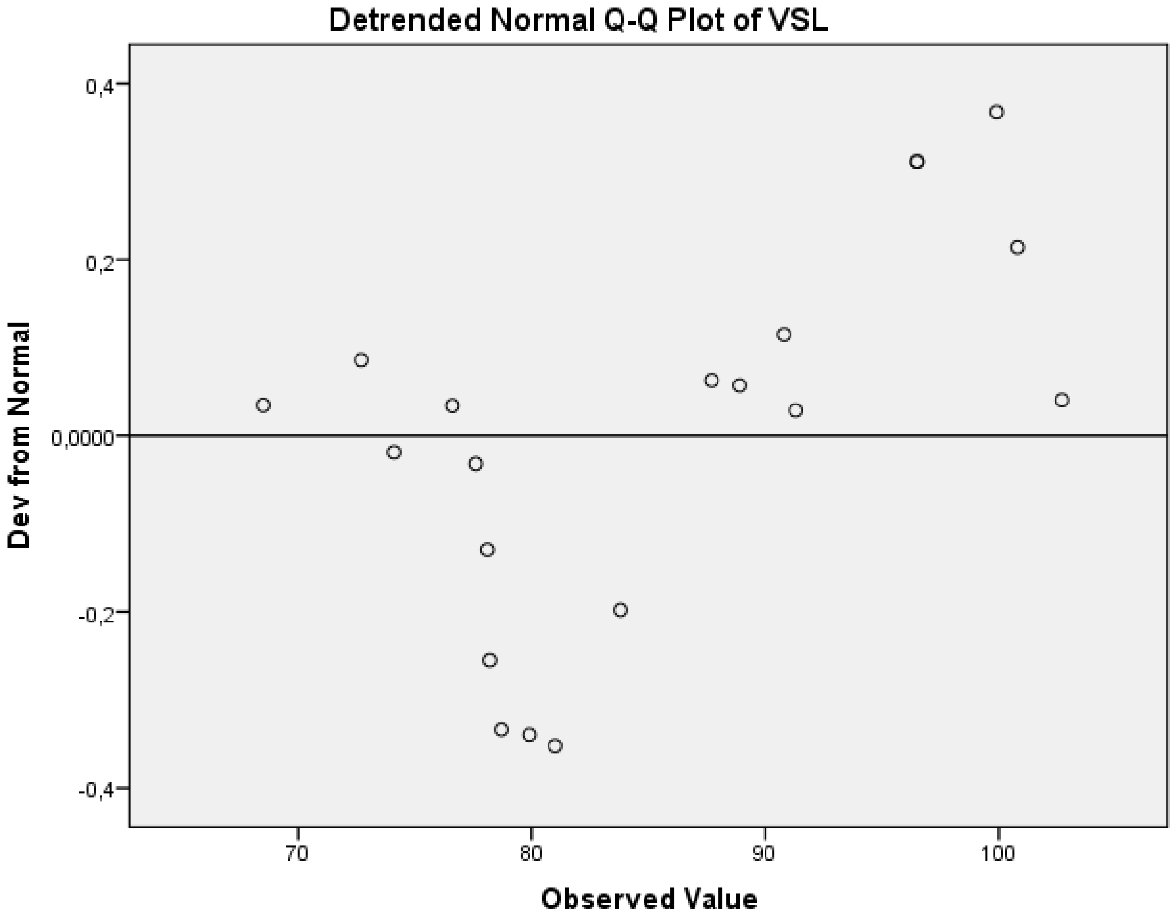

Detrended Normal Q-Q Plot of VSL
0,4
0,2
0,0000
-0,2
-0,4
Dev from Normal
70
80
90
100
Observed Value

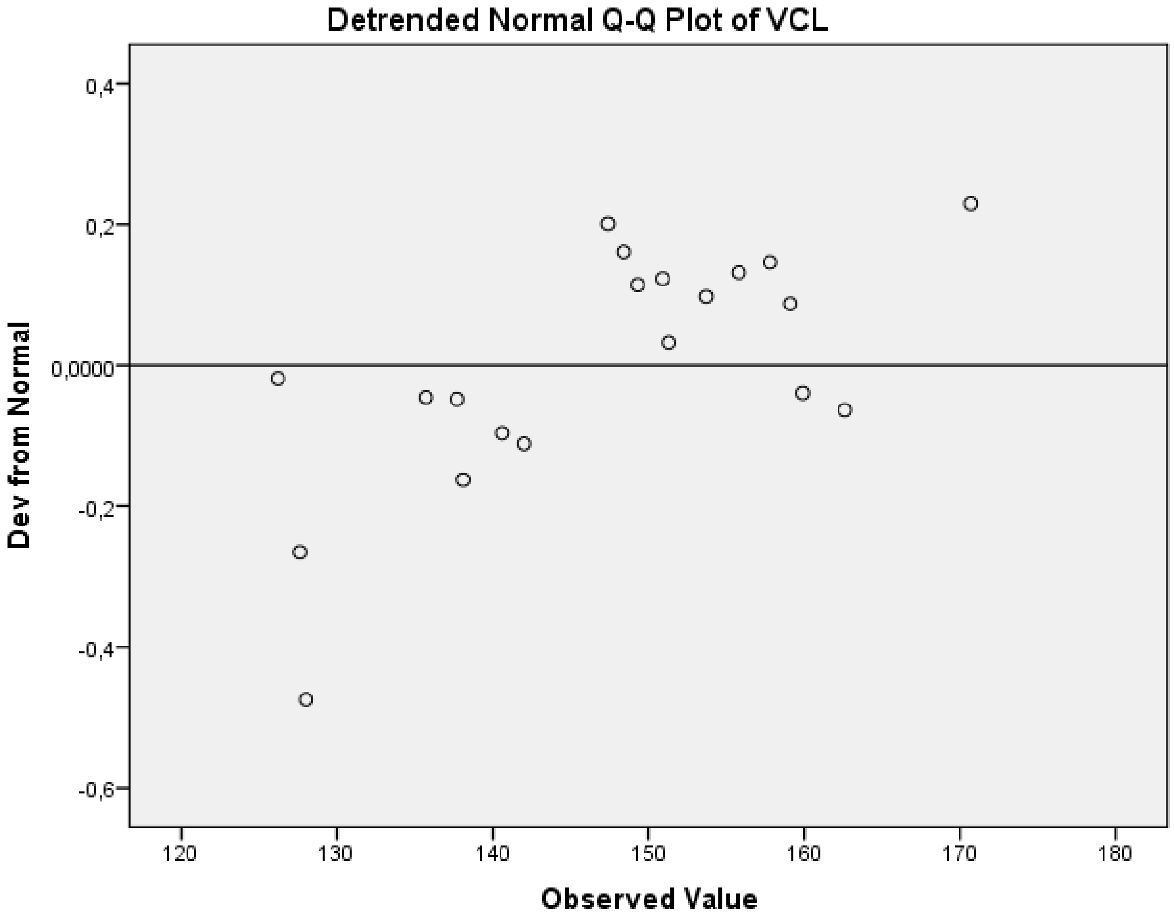
Detrended Normal Q-Q Plot of VCL
Dev from Normal
0,4
0,2
0,0000
-0,2
-0,4
-0,6
120
130
140
150
160
170
180
Observed Value

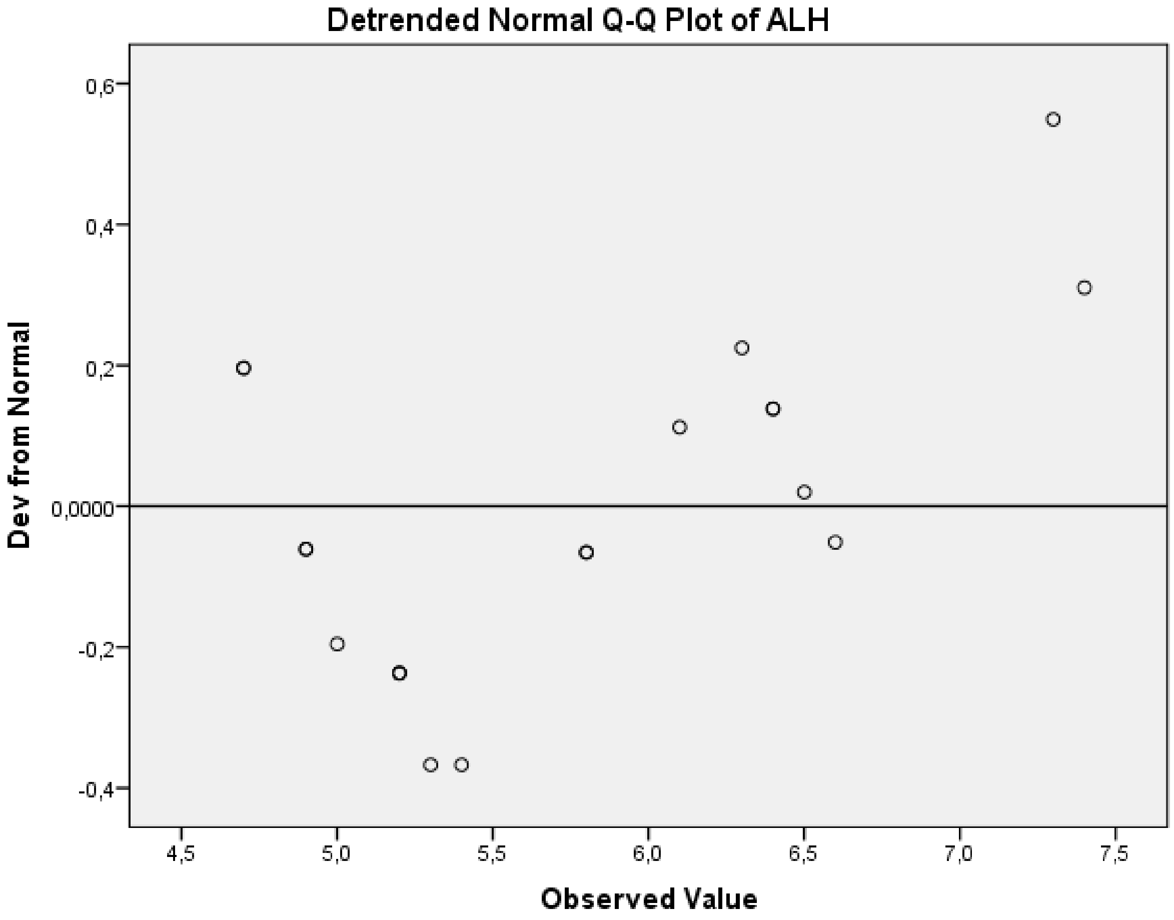
Detrended Normal Q-Q Plot of ALH
Dev from Normal
0,6
0,4
0,2
0,0000
-0,2
-0,4
4,5
5,0
5,5
6,0
6,5
7,0
7,5
Observed Value

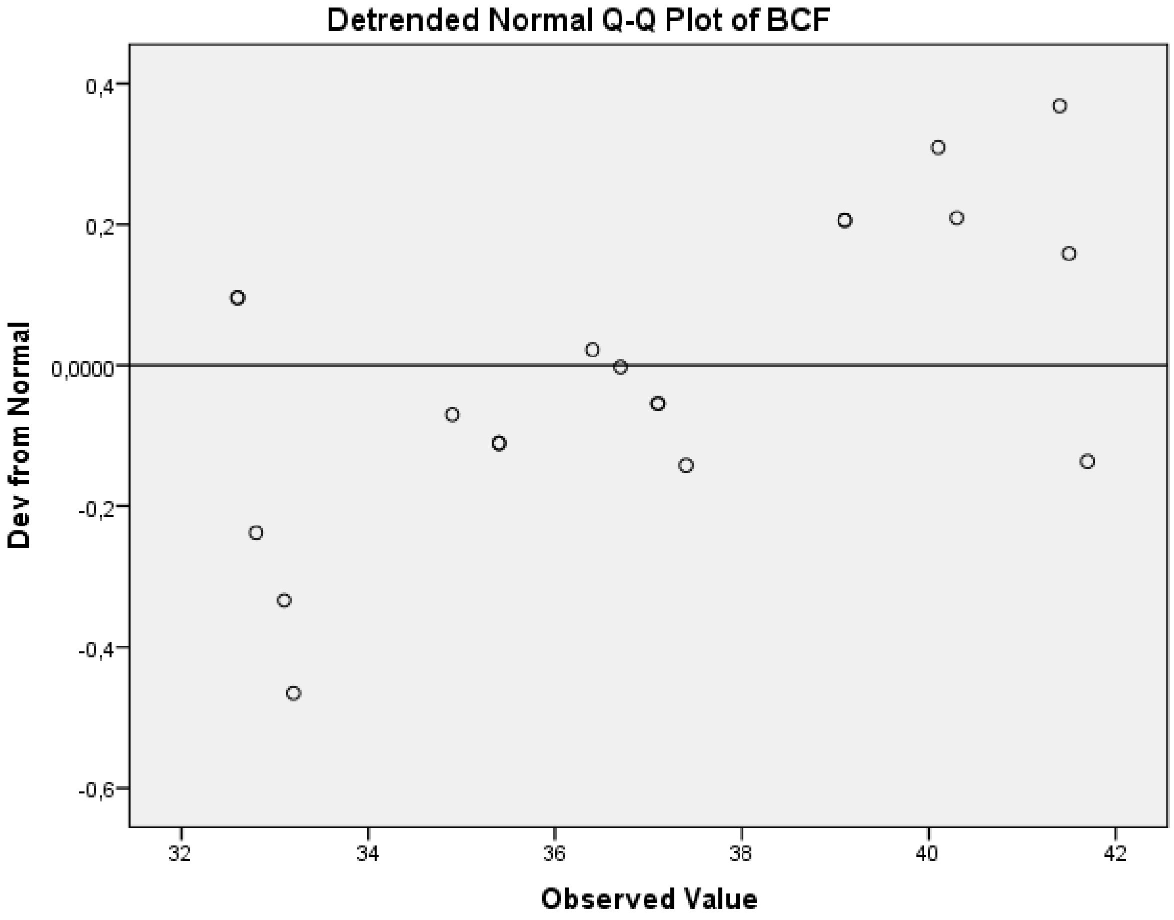

Detrended Normal Q-Q Plot of BCF
Dev from Normal
Observed Value
0,4
0,2
0,0000
-0,2
-0,4
-0,6
32
34
36
38
40
42

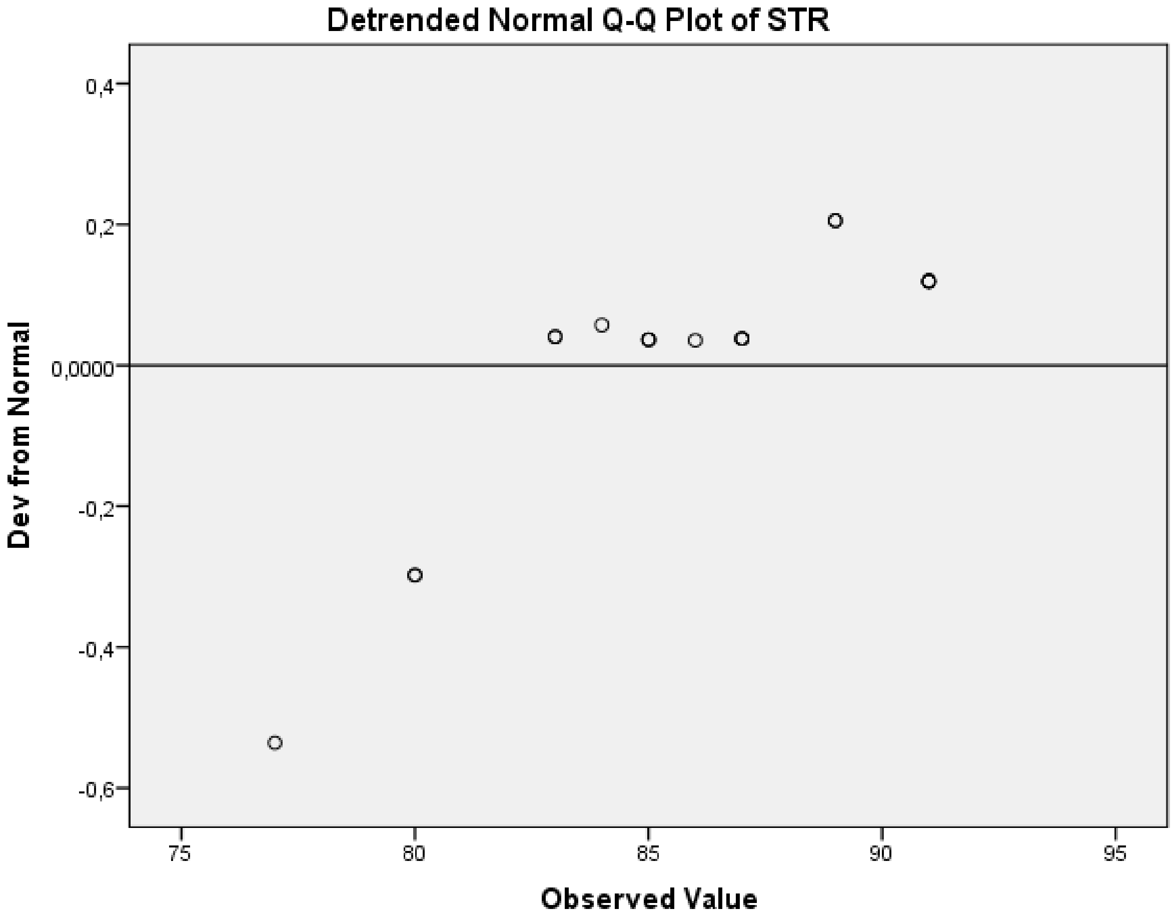

Detrended Normal Q-Q Plot of STR
Dev from Normal
0,4
0,2
0,0000
-0,2
-0,4
-0,6
75
80
85
90
95
Observed Value

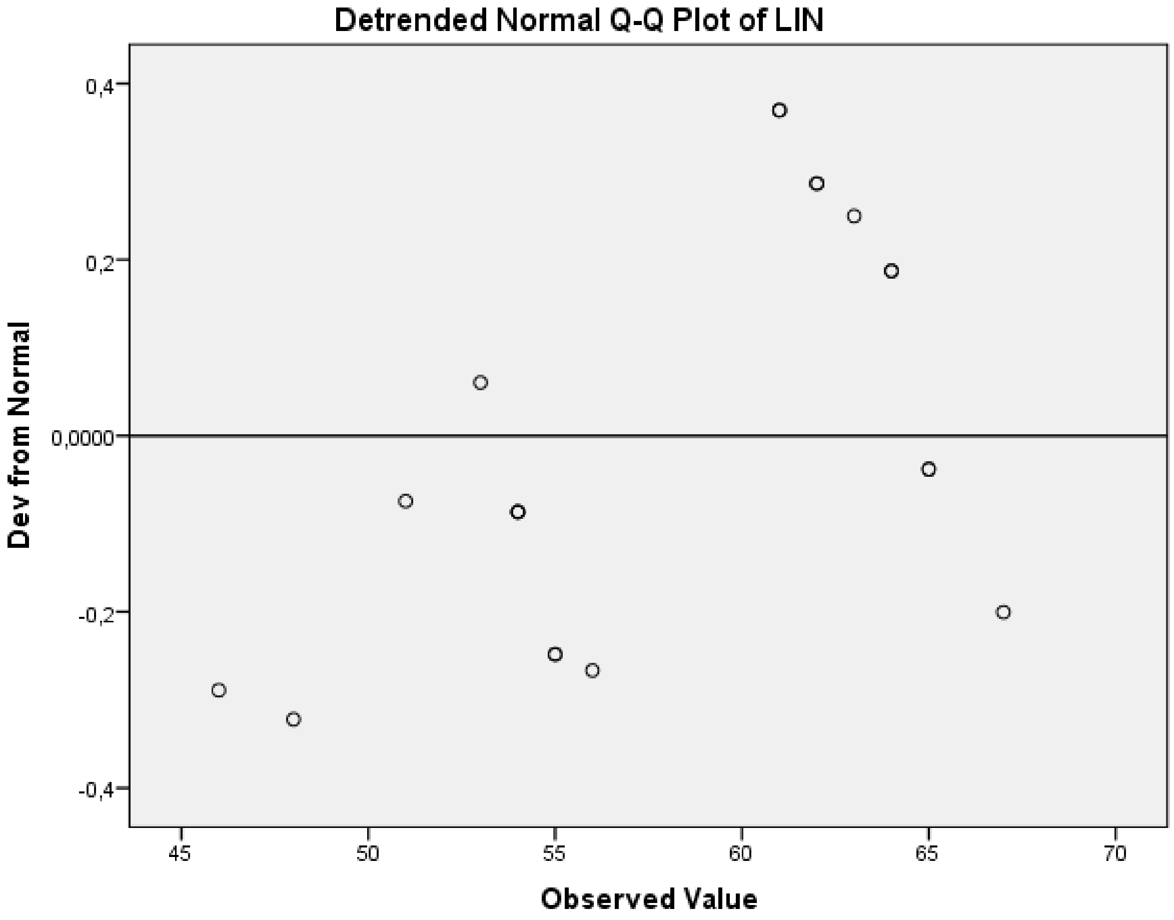

Gruplar = 8,00

Histograms

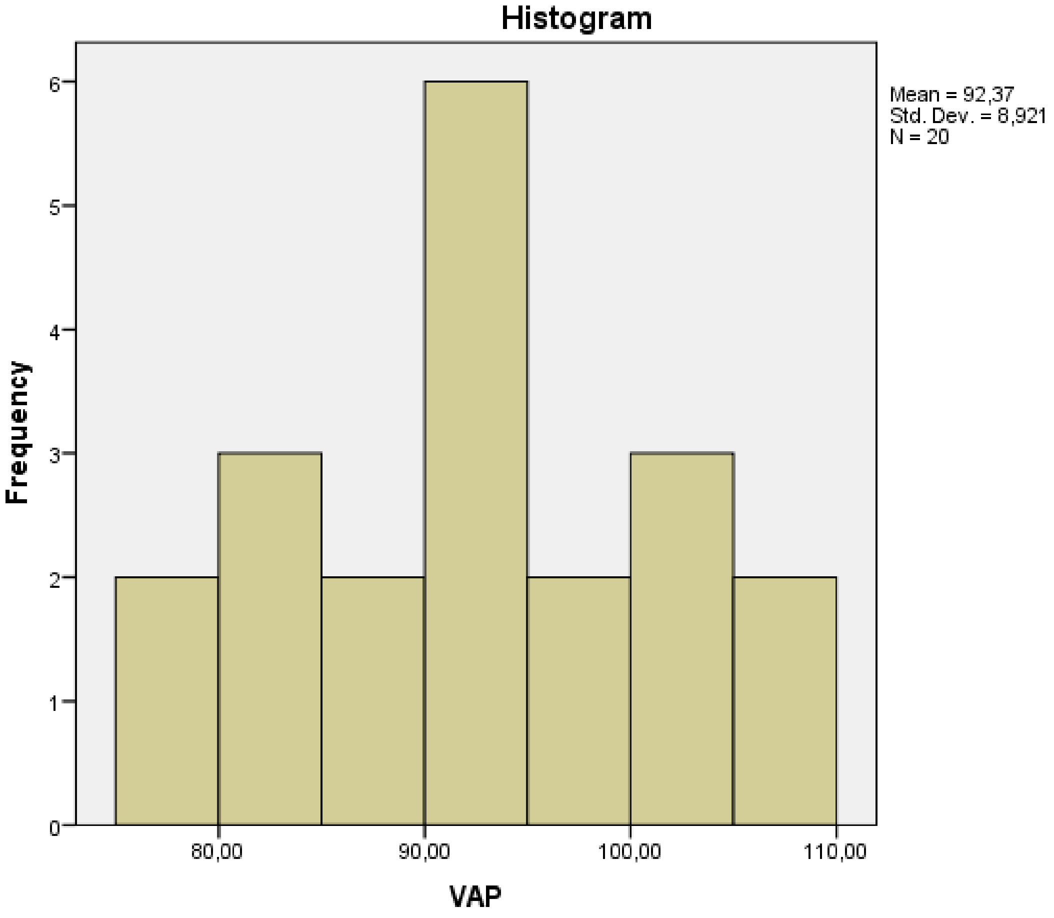

Histogram
Mean = 92,37
Std. Dev. = 8,921
N = 20
Frequency
0
1
2
3
4
5
6
80,00
90,00
100,00
110,00
VAP

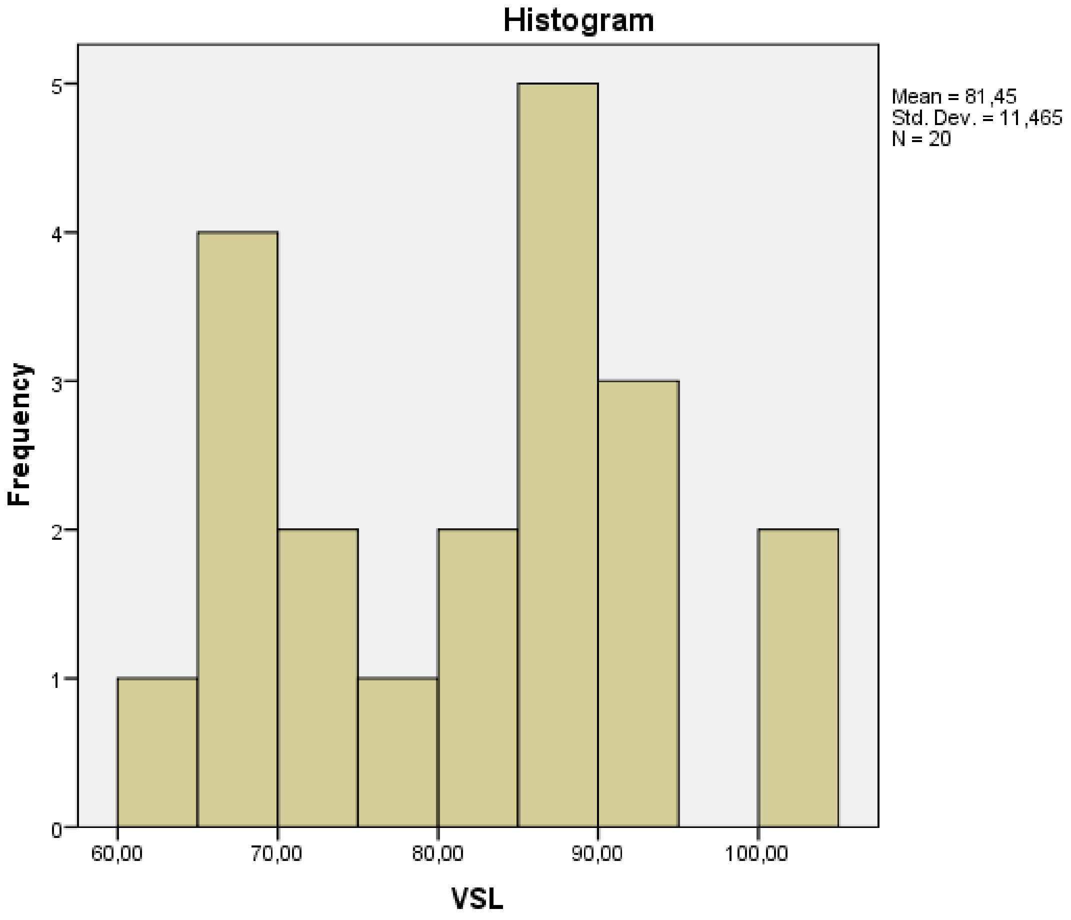

Histogram
Mean = 81,45
Std. Dev. = 11,465
N = 20
Frequency
0
1
2
3
4
5
60,00
70,00
80,00
90,00
100,00
VSL

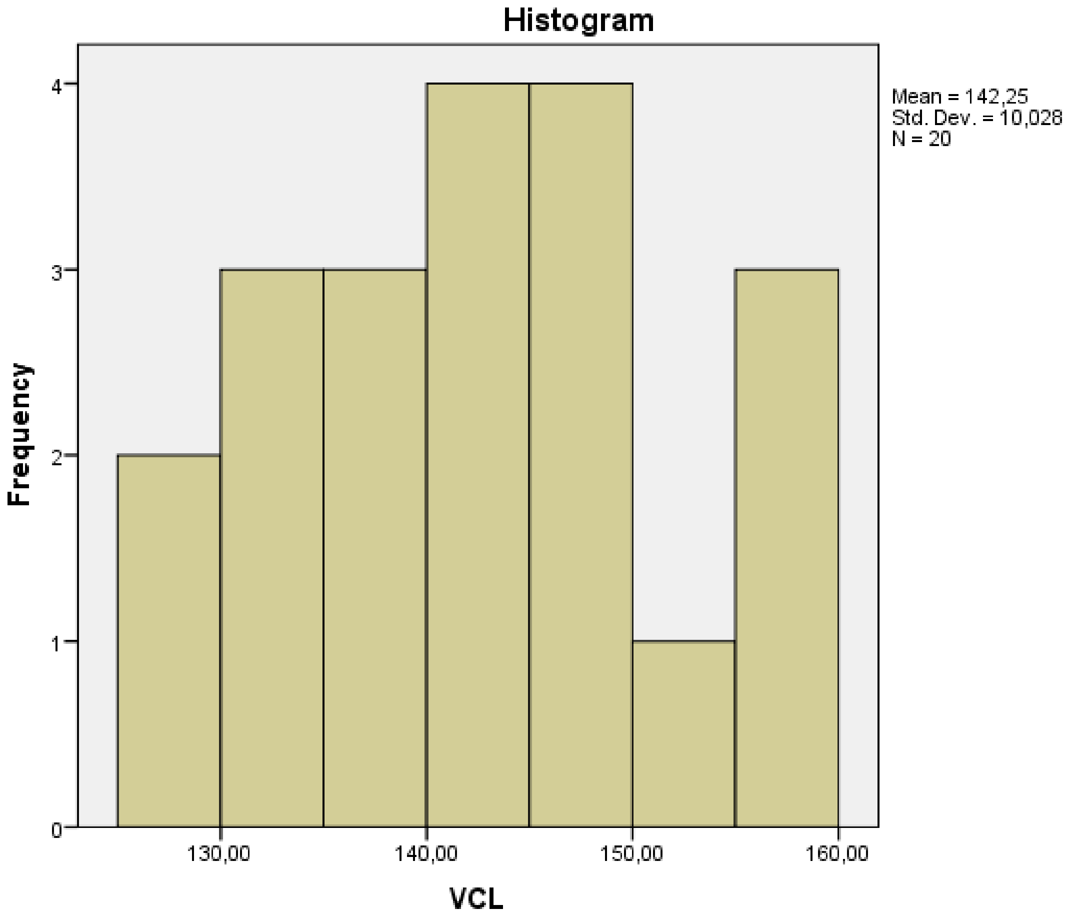
Histogram
Mean = 142,25
Std. Dev. = 10,028
N = 20
Frequency
0
1
2
3
4
130,00
140,00
150,00
160,00
VCL

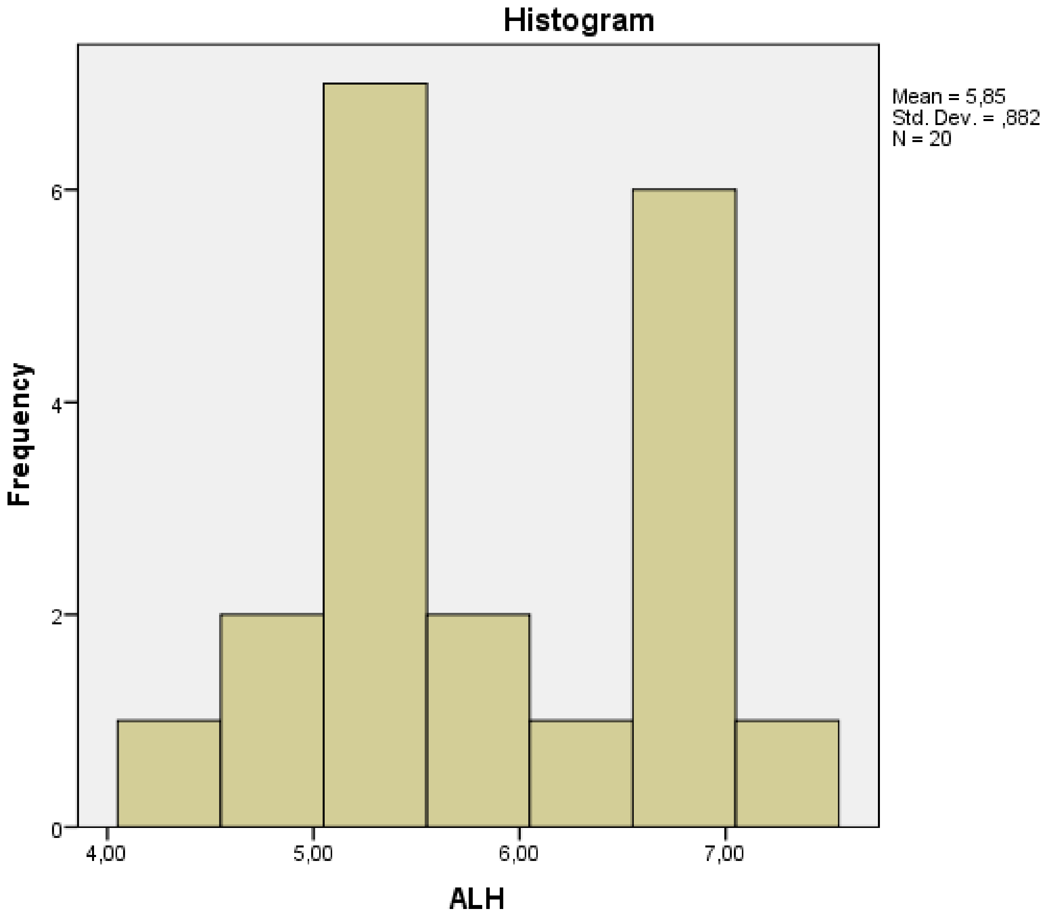

Histogram
Mean = 5,85
Std. Dev. = ,882
N = 20
Frequency
0
2
4
6
4,00
5,00
6,00
7,00
ALH

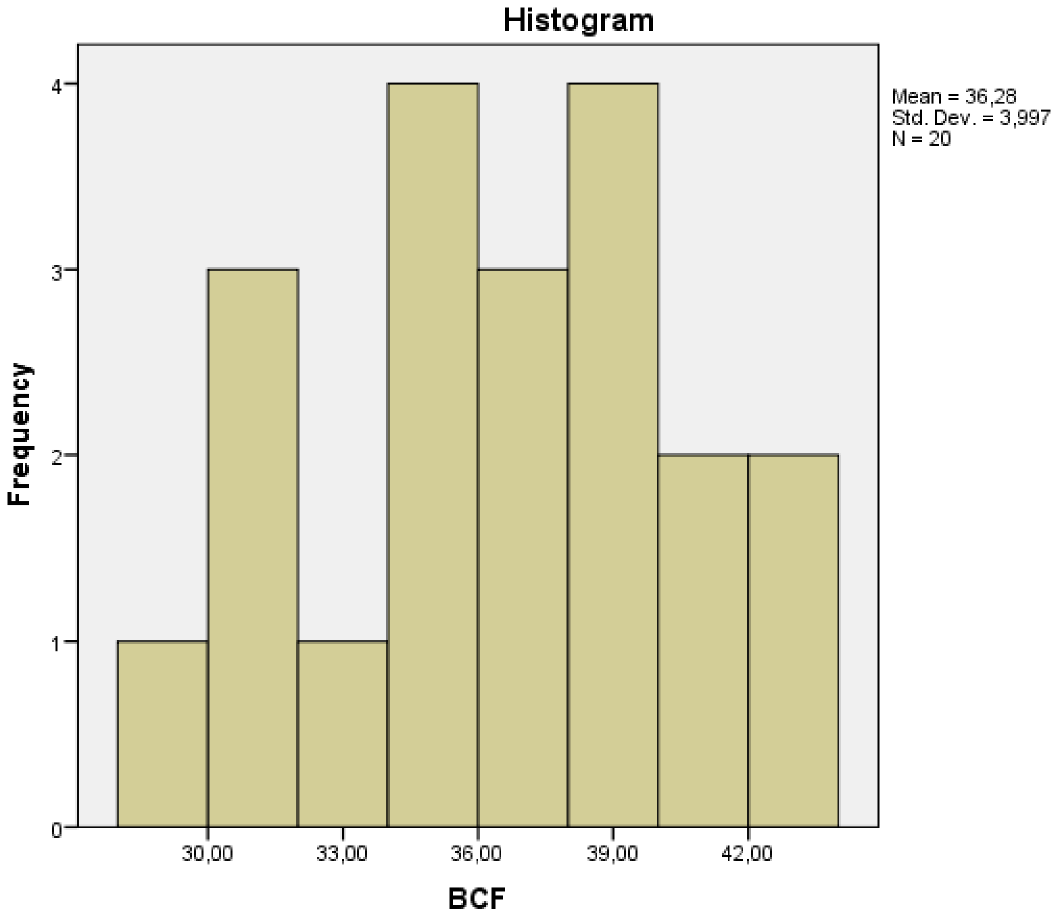
Histogram
Mean = 36,28
Std. Dev. = 3,997
N = 20
Frequency
0
1
2
3
4
30,00
33,00
36,00
39,00
42,00
BCF

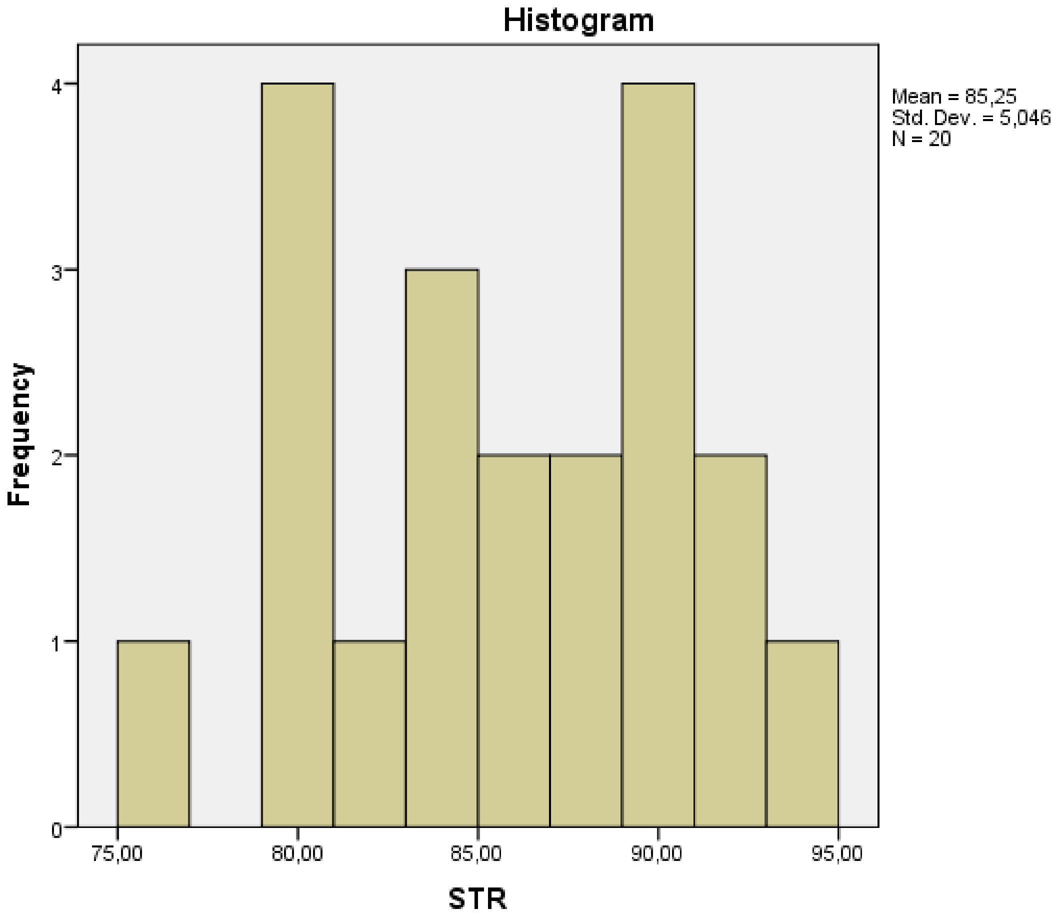

Histogram
Mean = 85,25
Std. Dev. = 5,046
N = 20
Frequency
0
1
2
3
4
75,00
80,00
85,00
90,00
95,00
STR

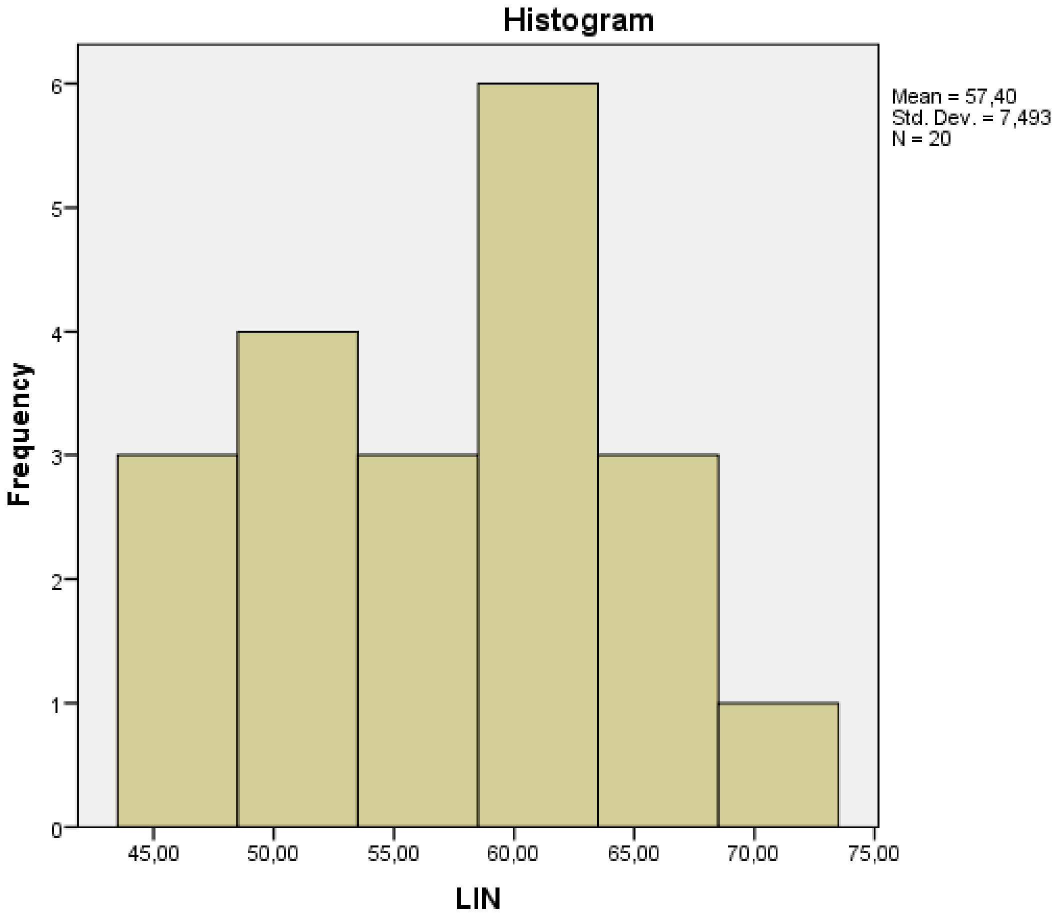

Stem-and-Leaf Plots

```
VAP Stem-and-Leaf Plot for
Gruplar= 8,00

 Frequency    Stem &  Leaf

     2,00        7 .  68
     3,00        8 .  024
     2,00        8 .  77
     6,00        9 .  112344
     2,00        9 .  58
     3,00       10 .  122
     2,00       10 .  57

 Stem width:  10,00
 Each leaf:       1 case(s)
```

```
VSL Stem-and-Leaf Plot for
Gruplar= 8,00

 Frequency    Stem &  Leaf

     5,00        6 .  16678
     3,00        7 .  229
     7,00        8 .  3455558
     3,00        9 .  112
     2,00       10 .  00

 Stem width:  10,00
 Each leaf:       1 case(s)
```

```
VCL Stem-and-Leaf Plot for
Gruplar= 8,00

 Frequency    Stem &  Leaf

     2,00       12 .  67
     3,00       13 .  002
     3,00       13 .  666
     4,00       14 .  0134
     4,00       14 .  5899
     1,00       15 .  1
     3,00       15 .  679

 Stem width:  10,00
 Each leaf:       1 case(s)
```

```
ALH Stem-and-Leaf Plot for
Gruplar= 8,00

 Frequency    Stem &  Leaf

     1,00        4 .  3
     1,00        4 .  7
     8,00        5 .  01233444
     2,00        5 .  67
     1,00        6 .  4
     4,00        6 .  6789
     3,00        7 .  002

 Stem width:   1,00
 Each leaf:       1 case(s)
```

```
BCF Stem-and-Leaf Plot for
Gruplar= 8,00

 Frequency    Stem &  Leaf

     1,00        2 .  9
     8,00        3 .  01124444
     7,00        3 .  7778889
     4,00        4 .  0122

 Stem width:  10,00
 Each leaf:       1 case(s)
```

```
STR Stem-and-Leaf Plot for
Gruplar= 8,00

 Frequency    Stem &  Leaf

     4,00        7 .  6999
     5,00        8 .  02334
     5,00        8 .  56779
     6,00        9 .  000114

 Stem width:  10,00
 Each leaf:       1 case(s)
```

```
LIN Stem-and-Leaf Plot for
Gruplar= 8,00

 Frequency    Stem &  Leaf

     4,00        4 .  6789
     3,00        5 .  011
     5,00        5 .  67799
     4,00        6 .  1133
     3,00        6 .  567
     1,00        7 .  2

 Stem width:  10,00
 Each leaf:       1 case(s)
```

Normal Q-Q Plots

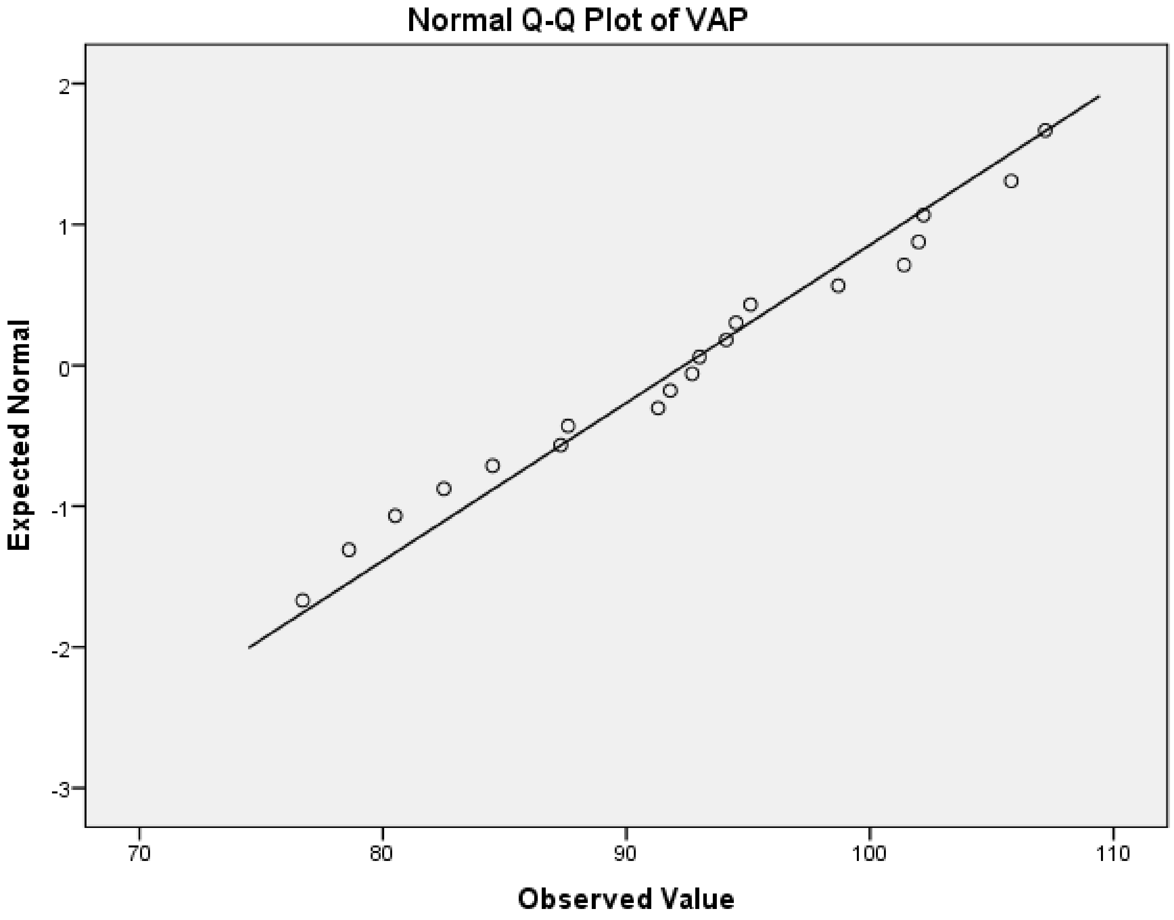
Normal Q-Q Plot of VAP
Expected Normal
2
1
0
-1
-2
-3
70
80
90
100
110
Observed Value

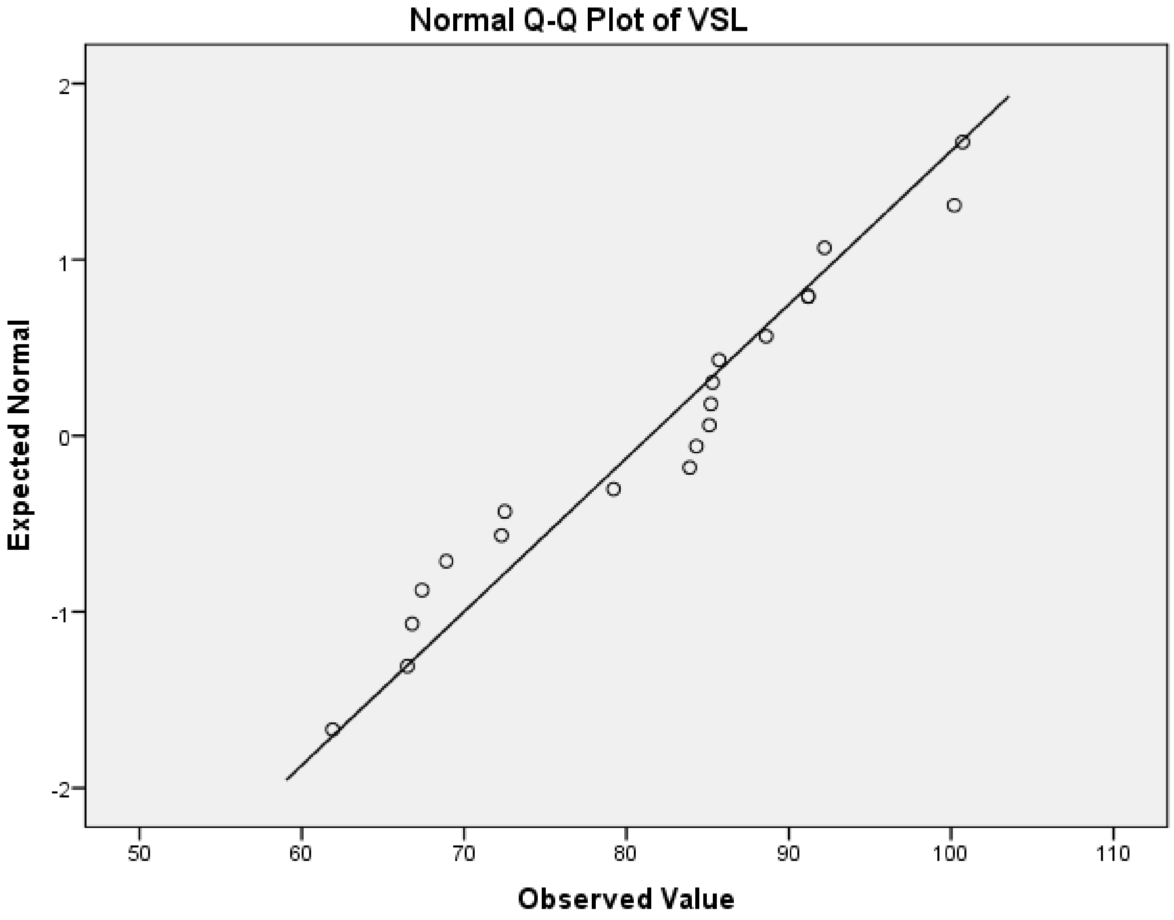
Normal Q-Q Plot of VSL
Expected Normal
2
1
0
-1
-2
50
60
70
80
90
100
110
Observed Value

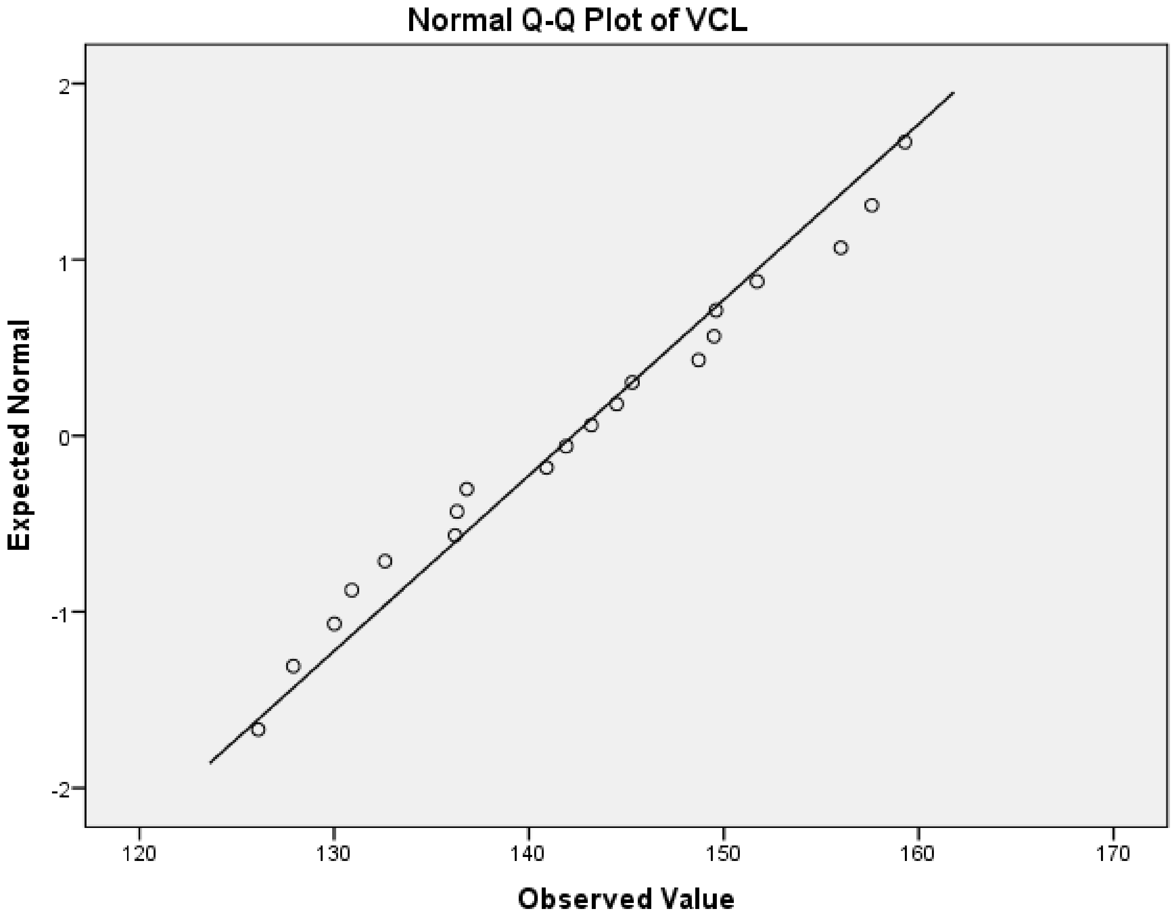
Normal Q-Q Plot of VCL
Expected Normal
2
1
0
-1
-2
120
130
140
150
160
170
Observed Value

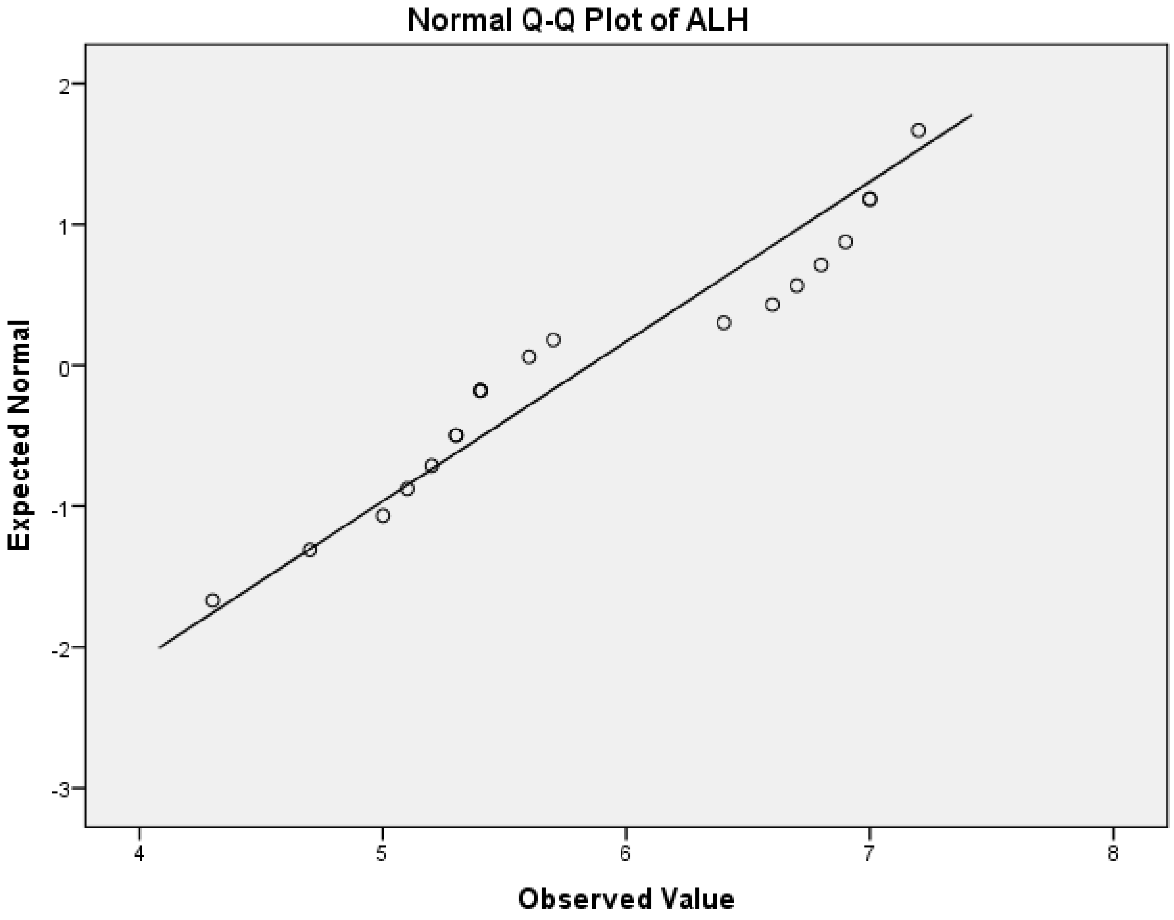
Normal Q-Q Plot of ALH
Expected Normal
Observed Value
2
1
0
-1
-2
-3
4
5
6
7
8

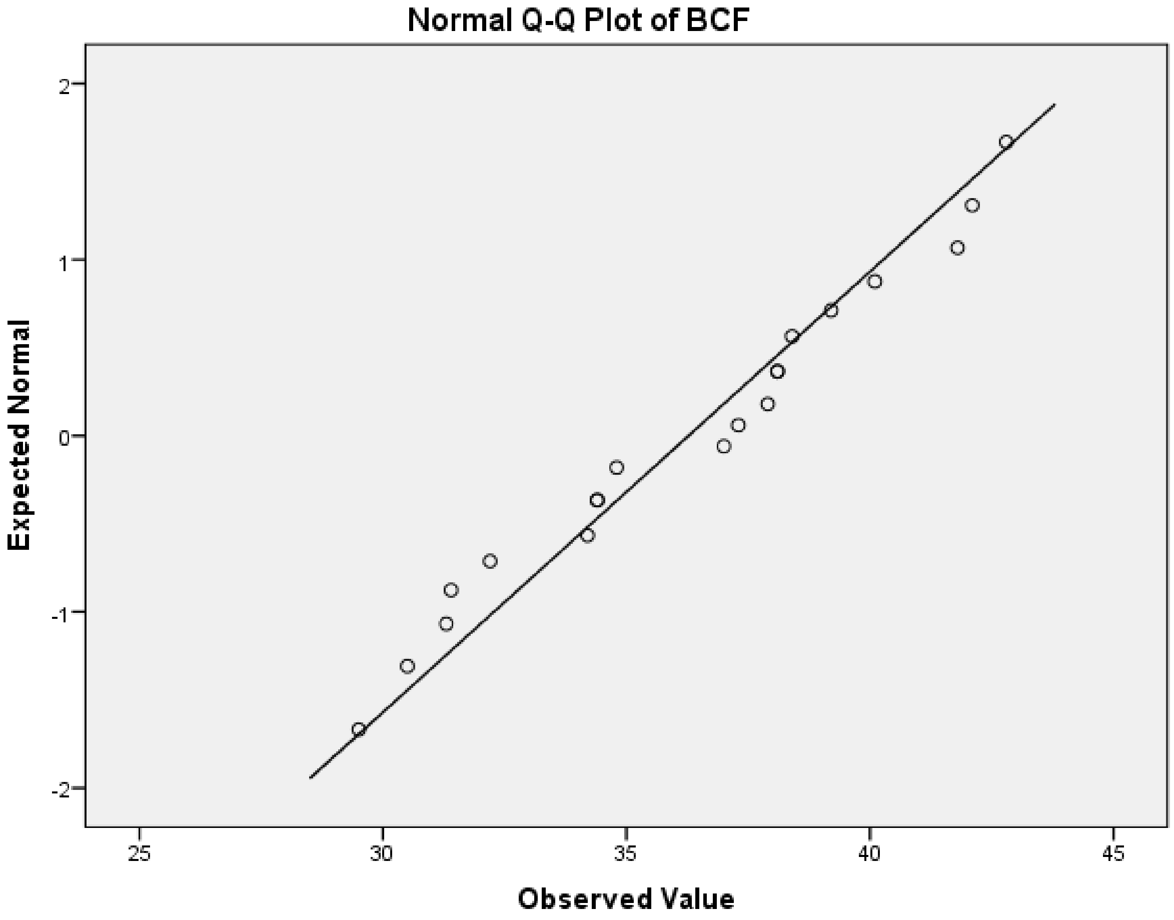
Normal Q-Q Plot of BCF
Expected Normal
Observed Value
2
1
0
-1
-2
25
30
35
40
45

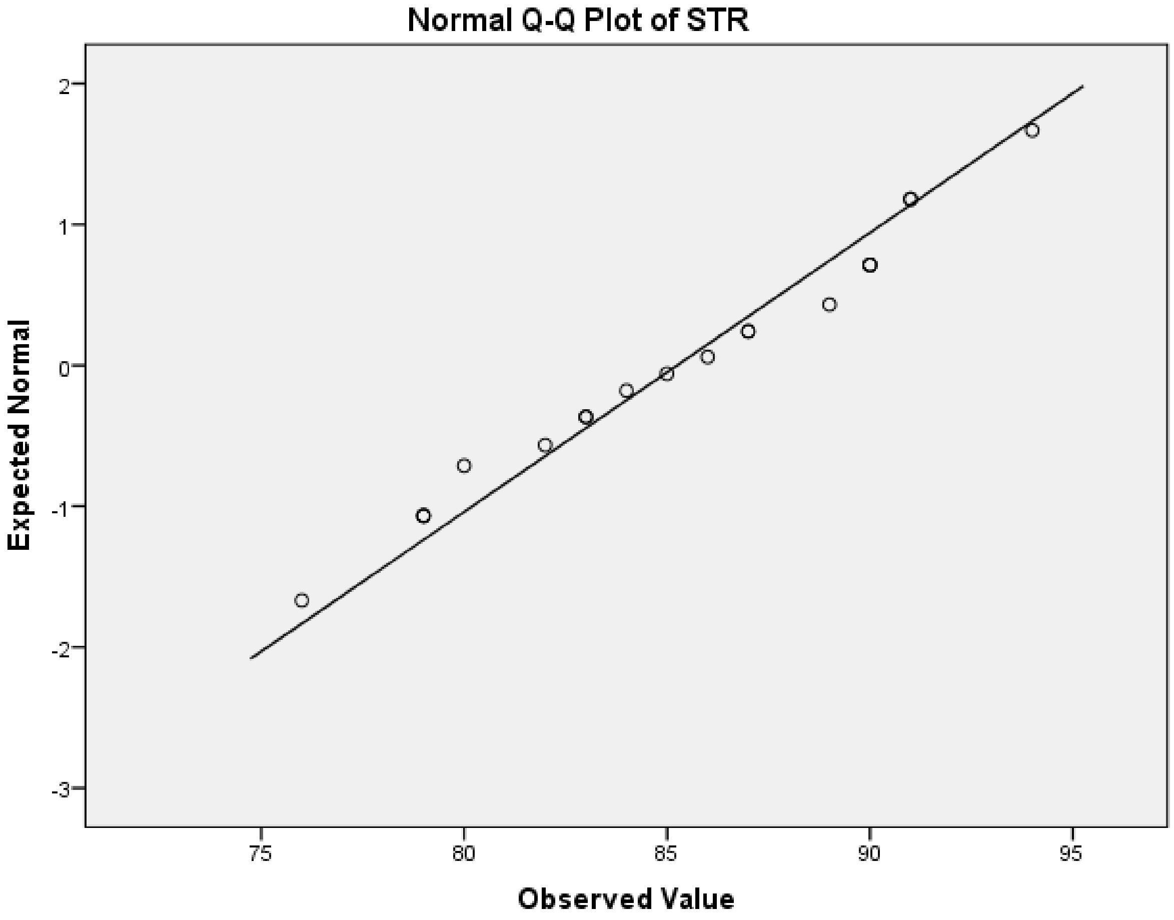
Normal Q-Q Plot of STR
Expected Normal
2
1
0
-1
-2
-3
75
80
85
90
95
Observed Value

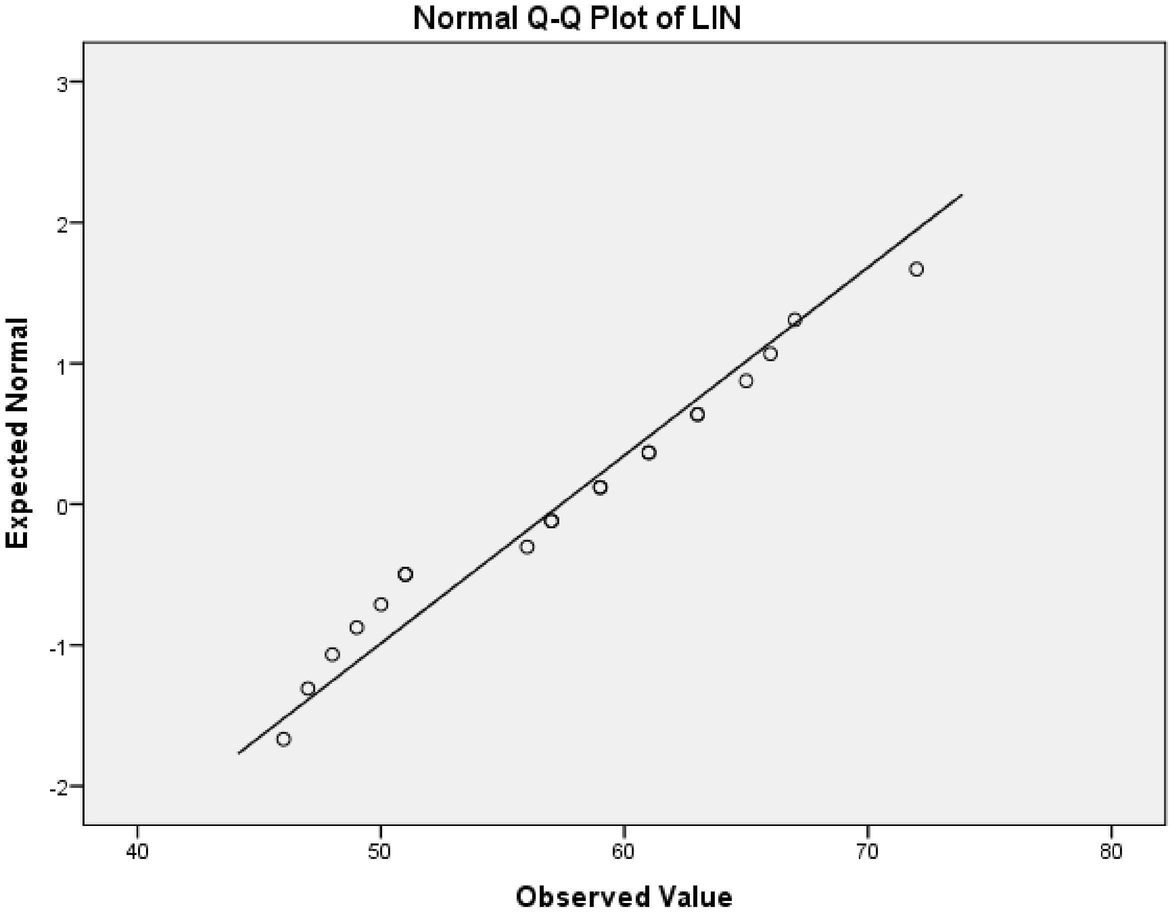

Detrended Normal Q-Q Plots

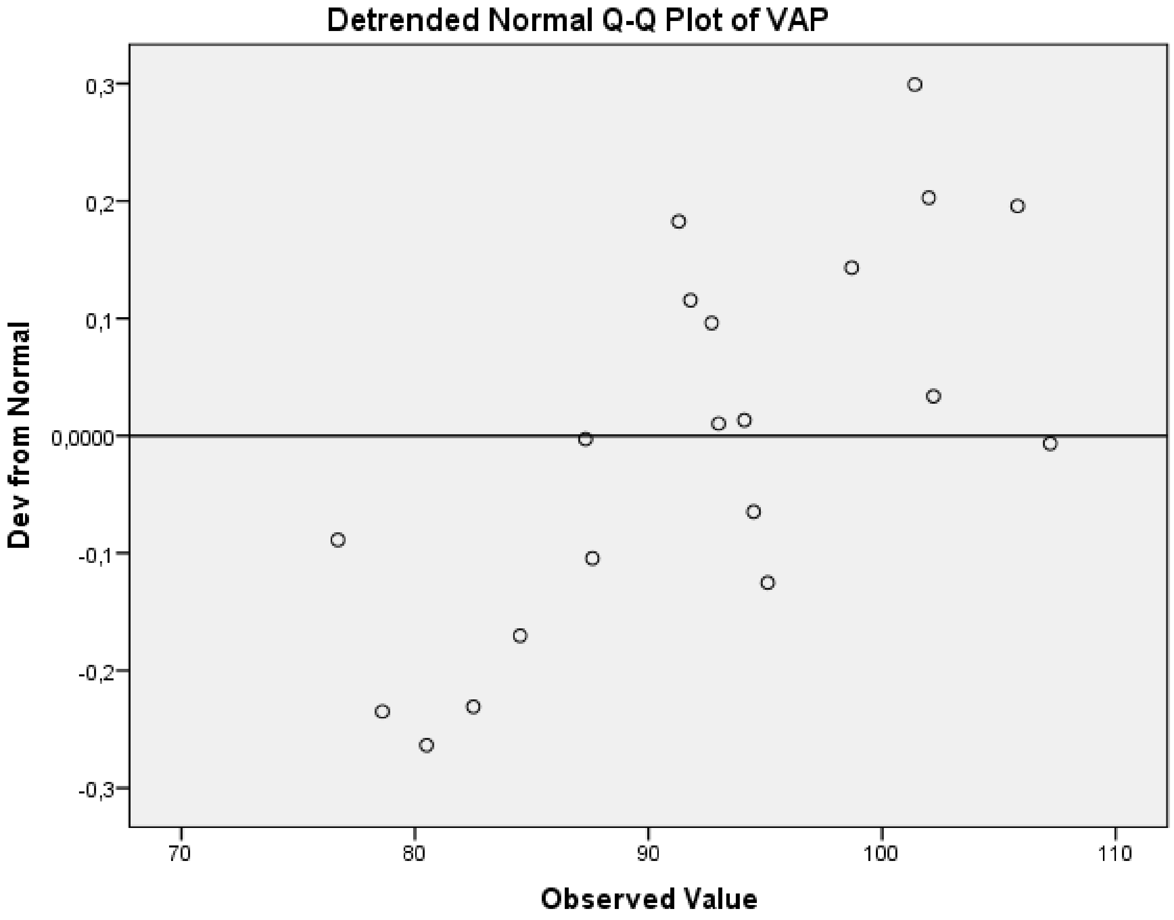
Detrended Normal Q-Q Plot of VAP
Dev from Normal
0,3
0,2
0,1
0,0000
-0,1
-0,2
-0,3
70
80
90
100
110
Observed Value

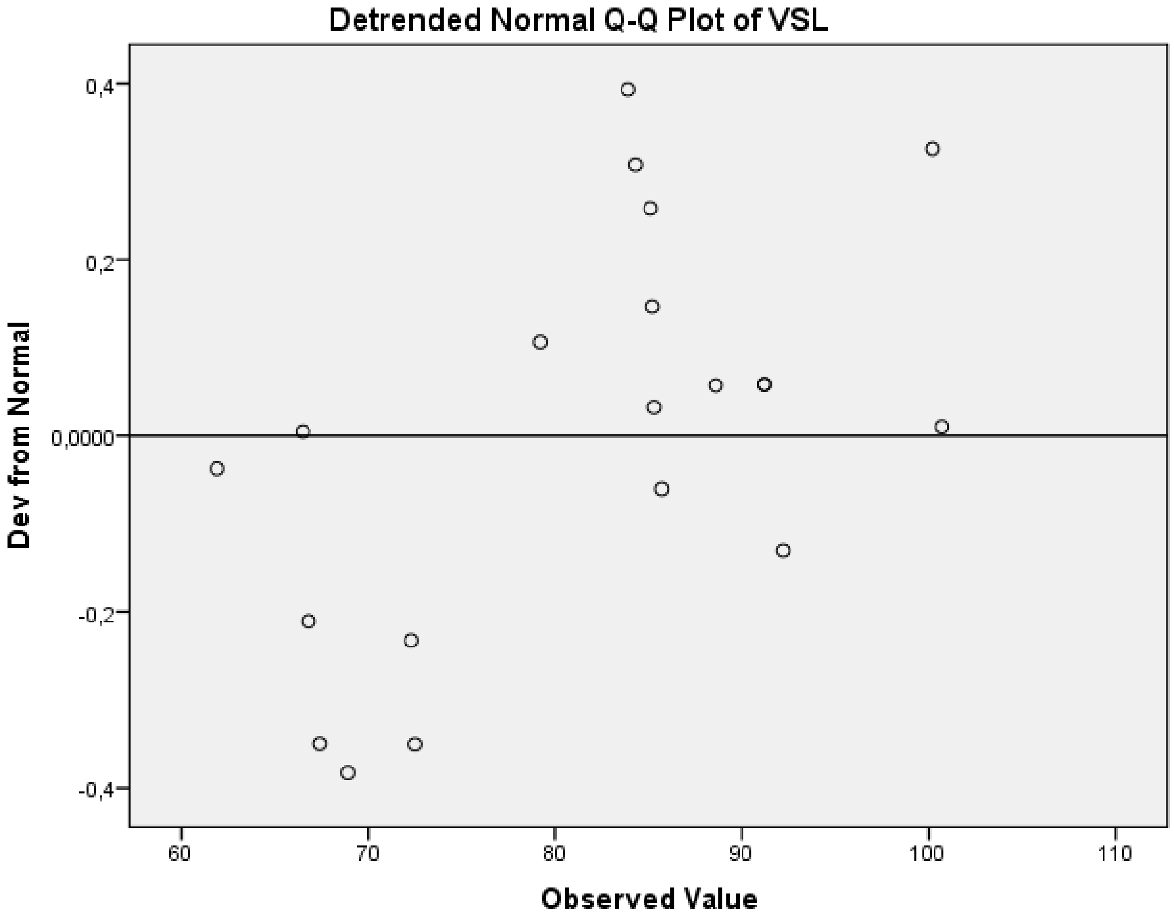

Detrended Normal Q-Q Plot of VSL
0,4
0,2
0,0000
-0,2
-0,4
Dev from Normal
60
70
80
90
100
110
Observed Value

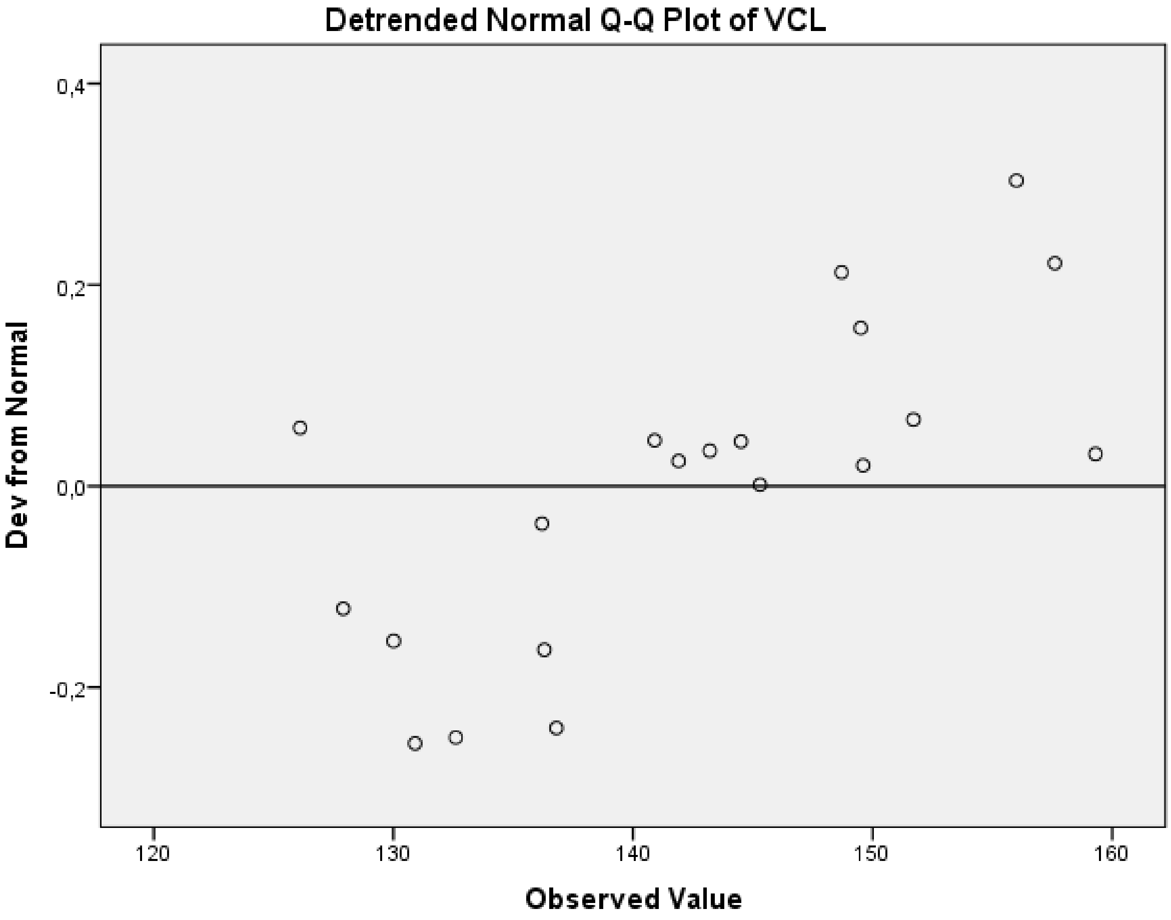
Detrended Normal Q-Q Plot of VCL
Dev from Normal
0,4
0,2
0,0
-0,2
120
130
140
150
160
Observed Value

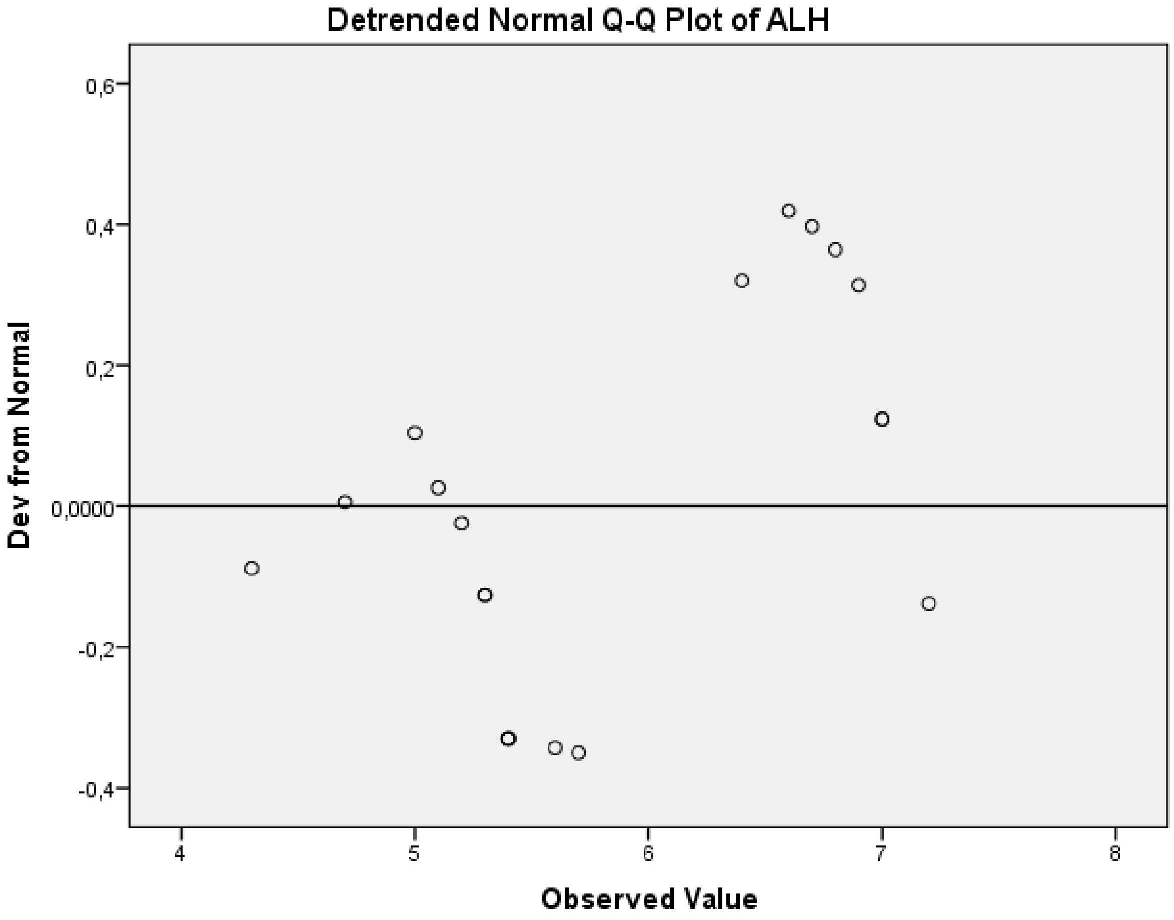

Detrended Normal Q-Q Plot of ALH
Dev from Normal
0,6
0,4
0,2
0,0000
-0,2
-0,4
4
5
6
7
8
Observed Value

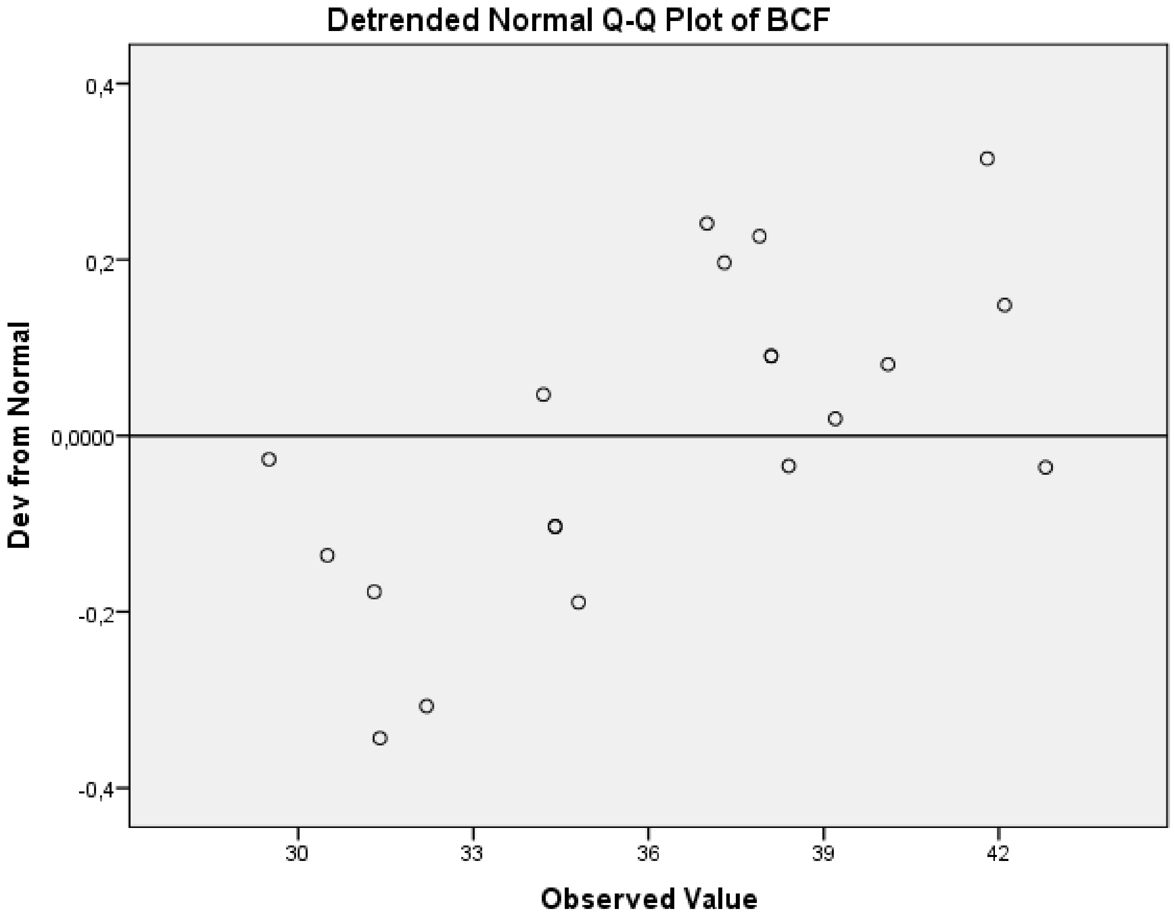

Detrended Normal Q-Q Plot of BCF
Dev from Normal
Observed Value
0,4
0,2
0,0000
-0,2
-0,4
30
33
36
39
42

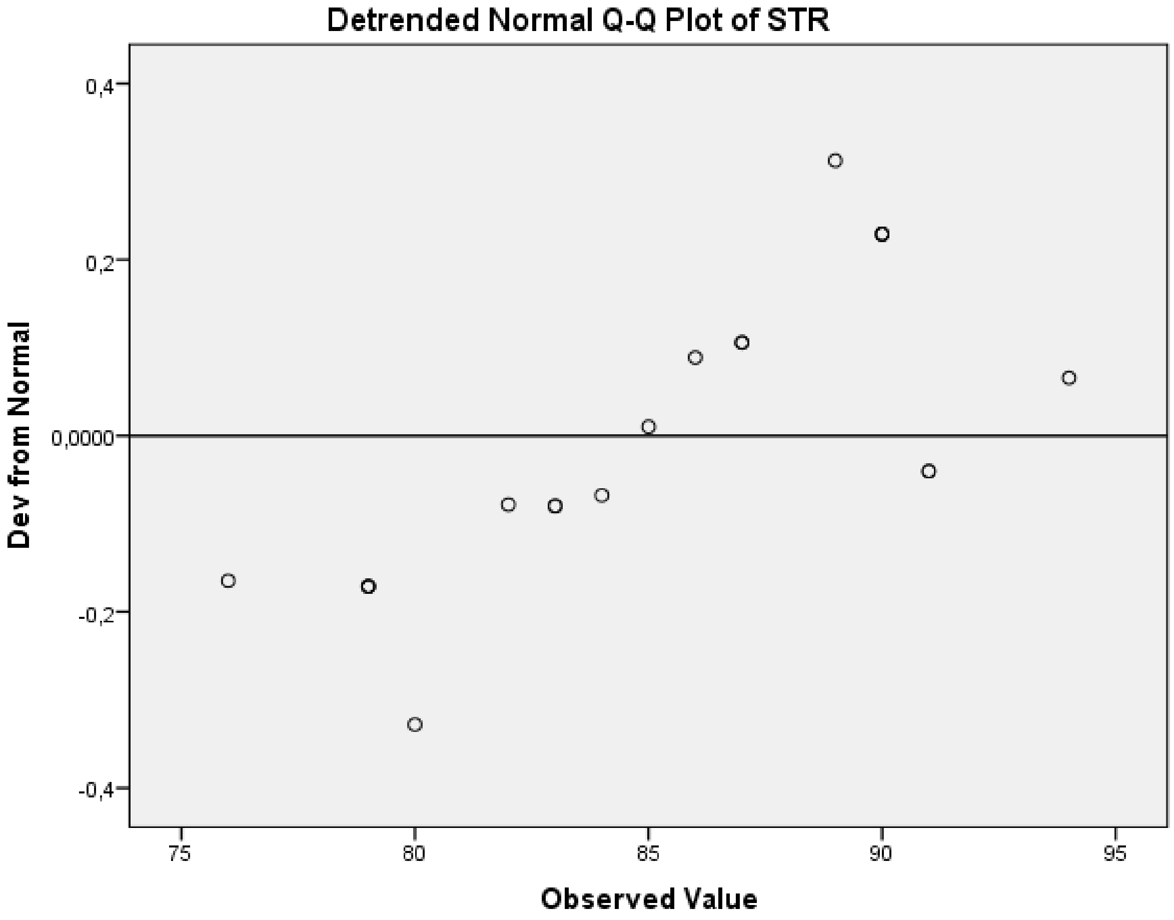

Detrended Normal Q-Q Plot of STR
Dev from Normal
0,4
0,2
0,0000
-0,2
-0,4
75
80
85
90
95
Observed Value

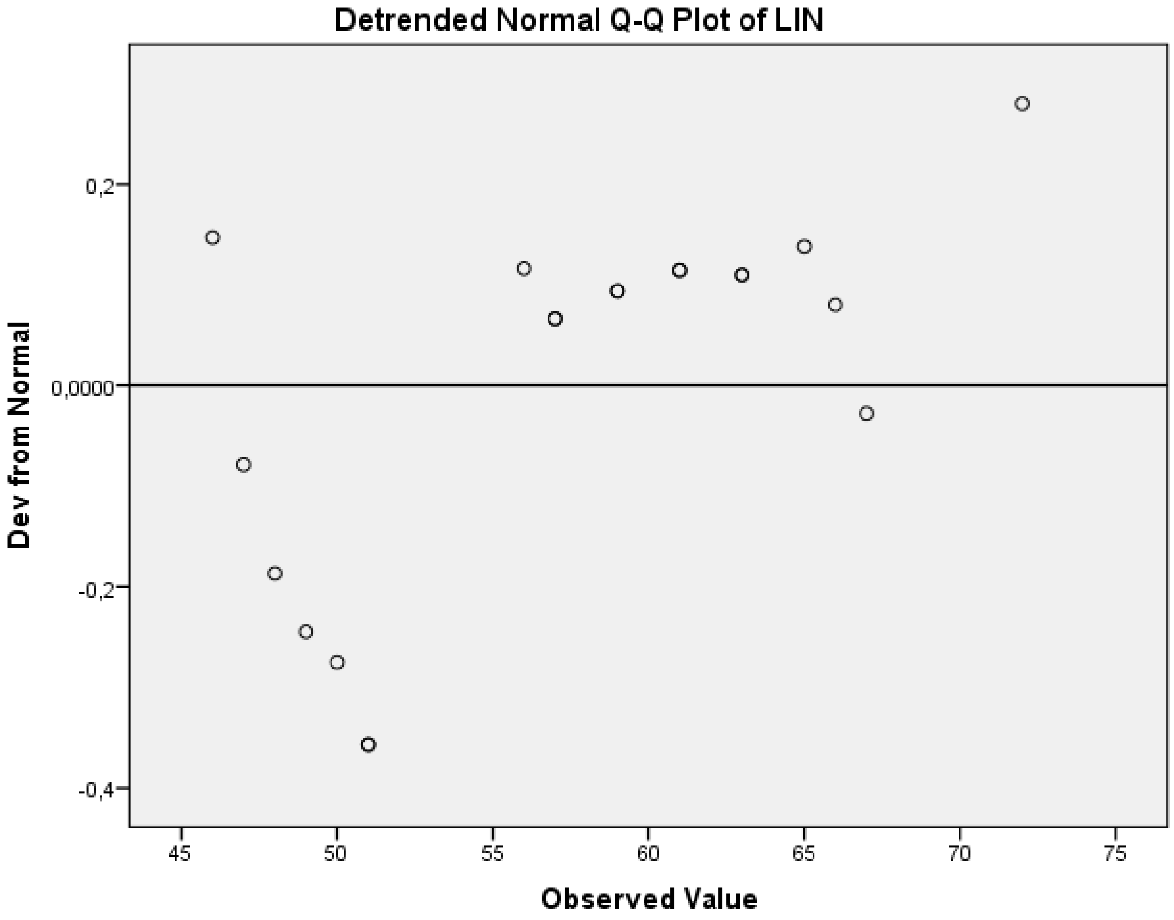

Gruplar = 9,00

Histograms

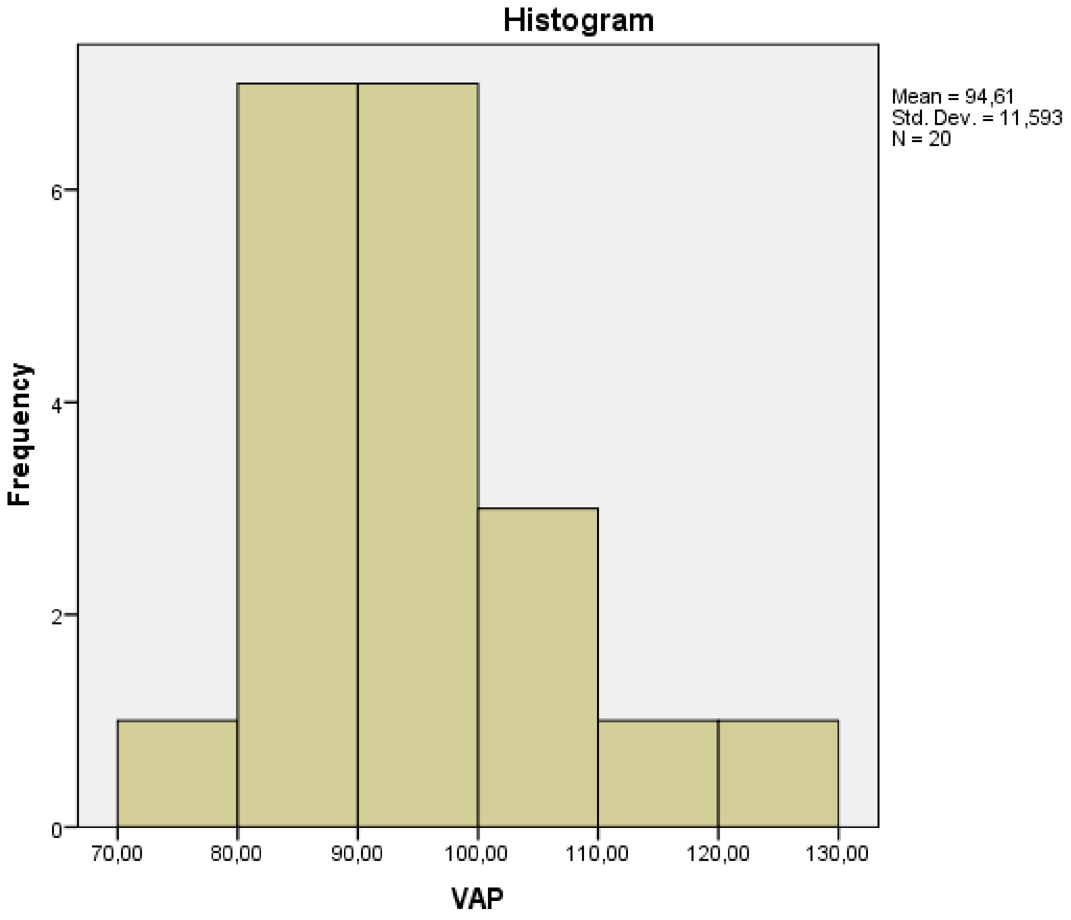
Histogram
Mean = 94,61
Std. Dev. = 11,593
N = 20
Frequency
0
2
4
6
70,00
80,00
90,00
100,00
110,00
120,00
130,00
VAP

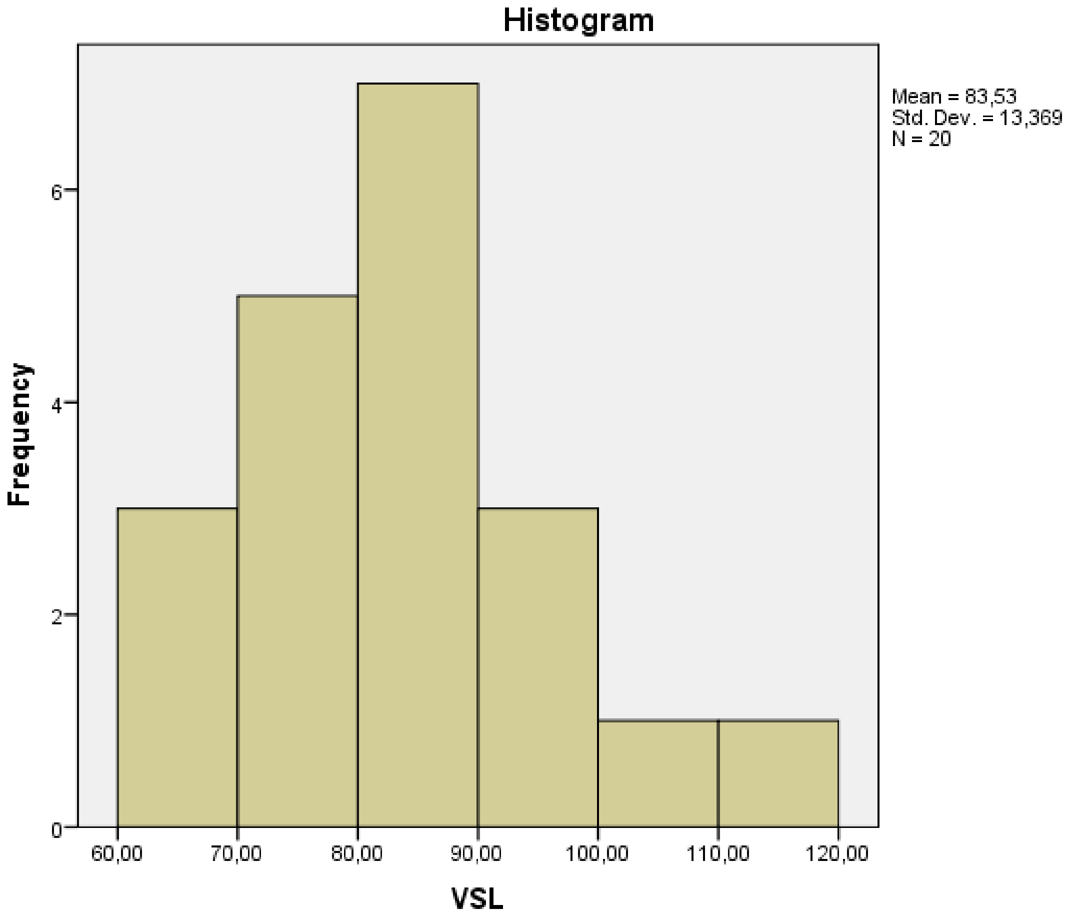
Histogram
Mean = 83,53
Std. Dev. = 13,369
N = 20
Frequency
0
2
4
6
60,00
70,00
80,00
90,00
100,00
110,00
120,00
VSL

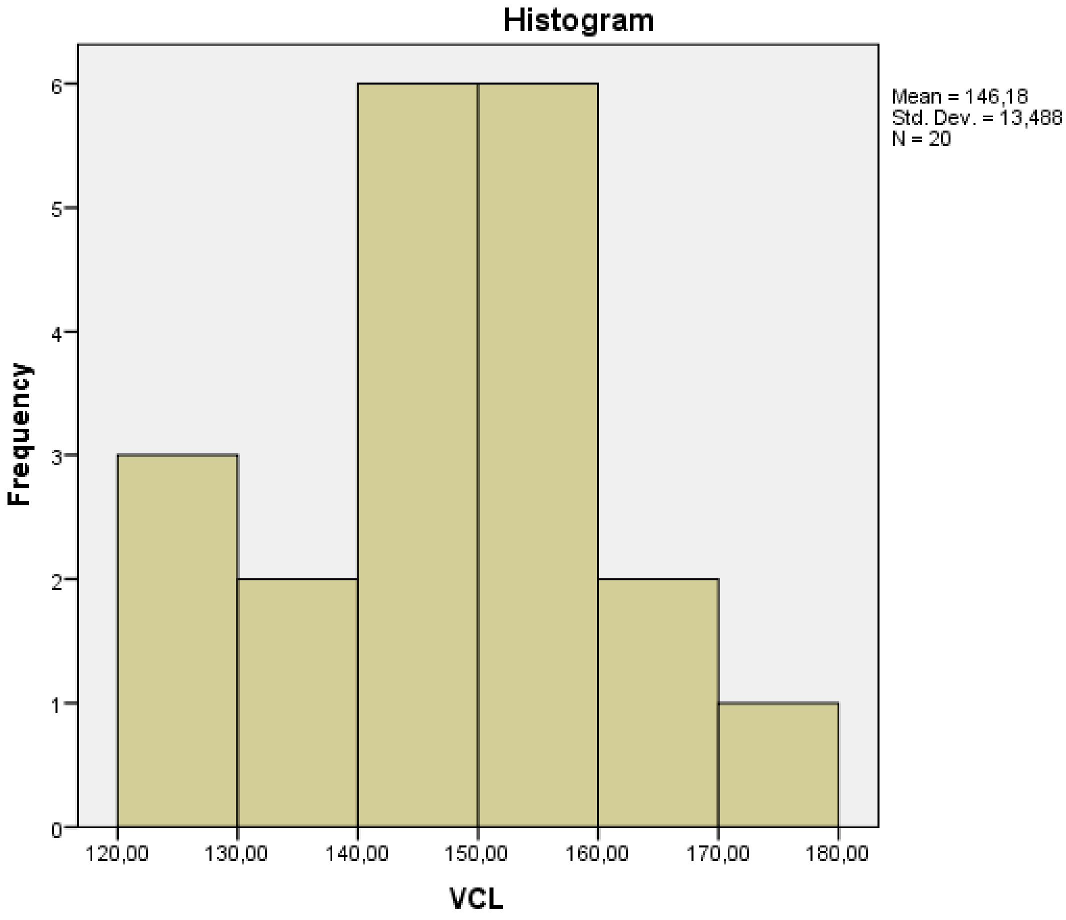
Histogram
Mean = 146,18
Std. Dev. = 13,488
N = 20
Frequency
0
1
2
3
4
5
6
120,00
130,00
140,00
150,00
160,00
170,00
180,00
VCL

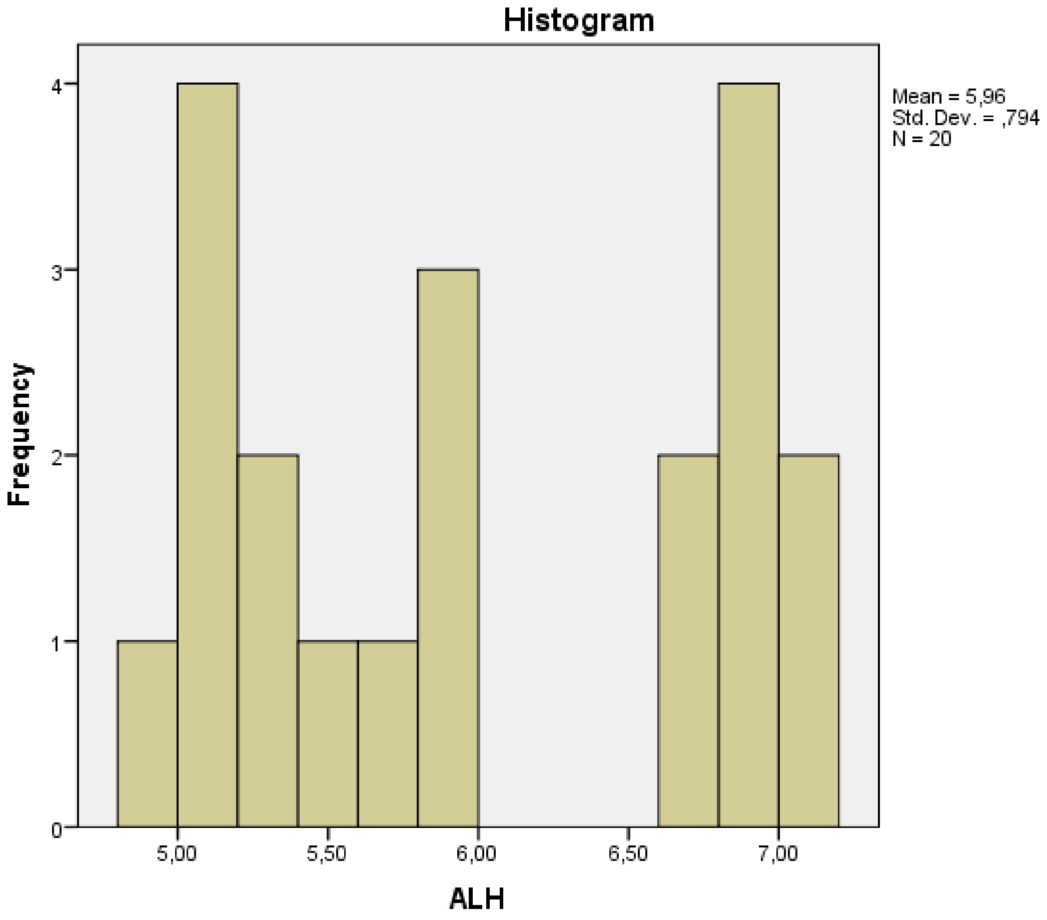
Histogram
Mean = 5,96
Std. Dev. = ,794
N = 20
Frequency
4
3
2
1
0
5,00
5,50
6,00
6,50
7,00
ALH

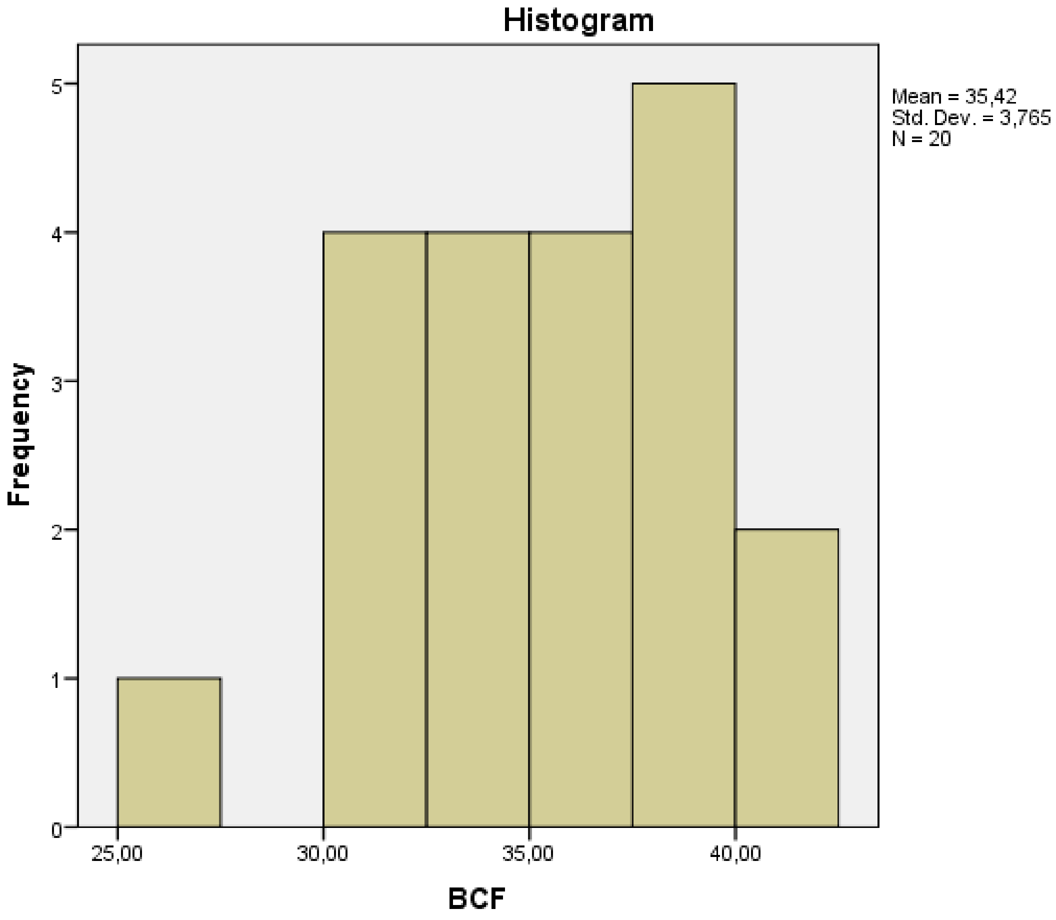

Histogram
Mean = 35,42
Std. Dev. = 3,765
N = 20
Frequency
0
1
2
3
4
5
25,00
30,00
35,00
40,00
BCF

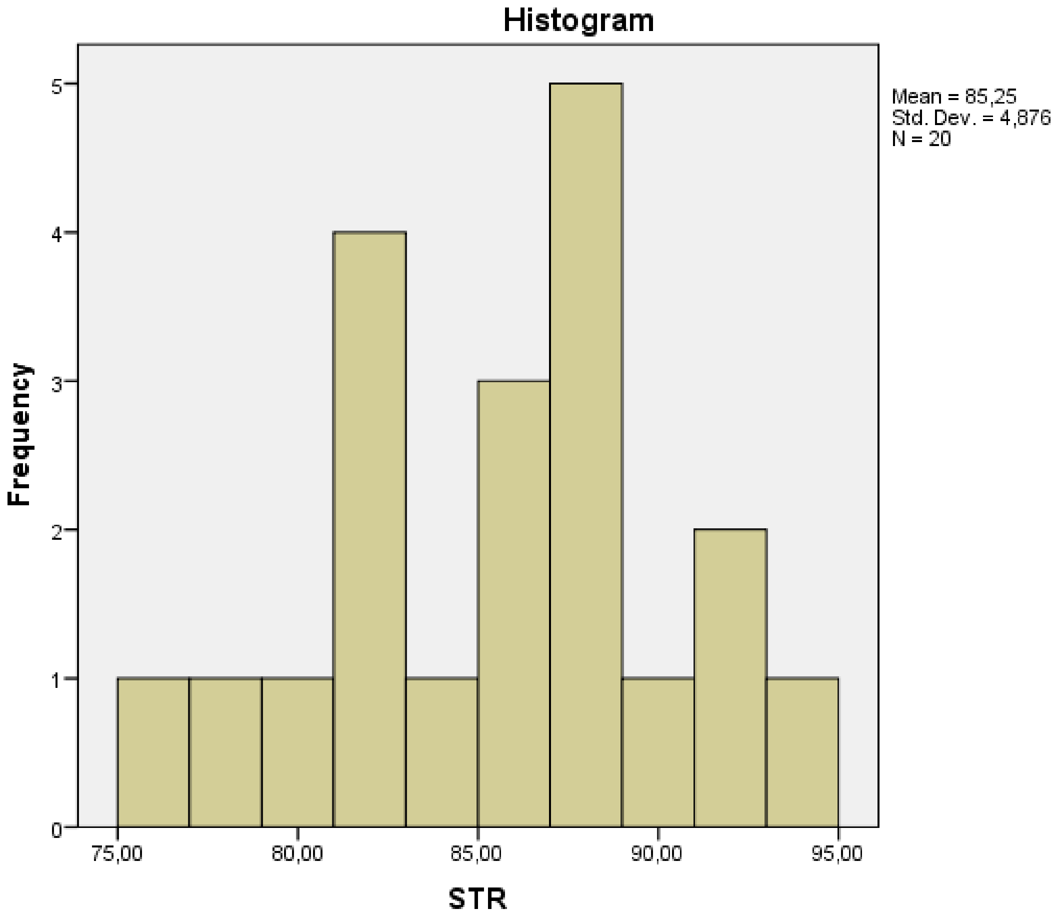

Histogram
Mean = 85,25
Std. Dev. = 4,876
N = 20
Frequency
0
1
2
3
4
5
75,00
80,00
85,00
90,00
95,00
STR

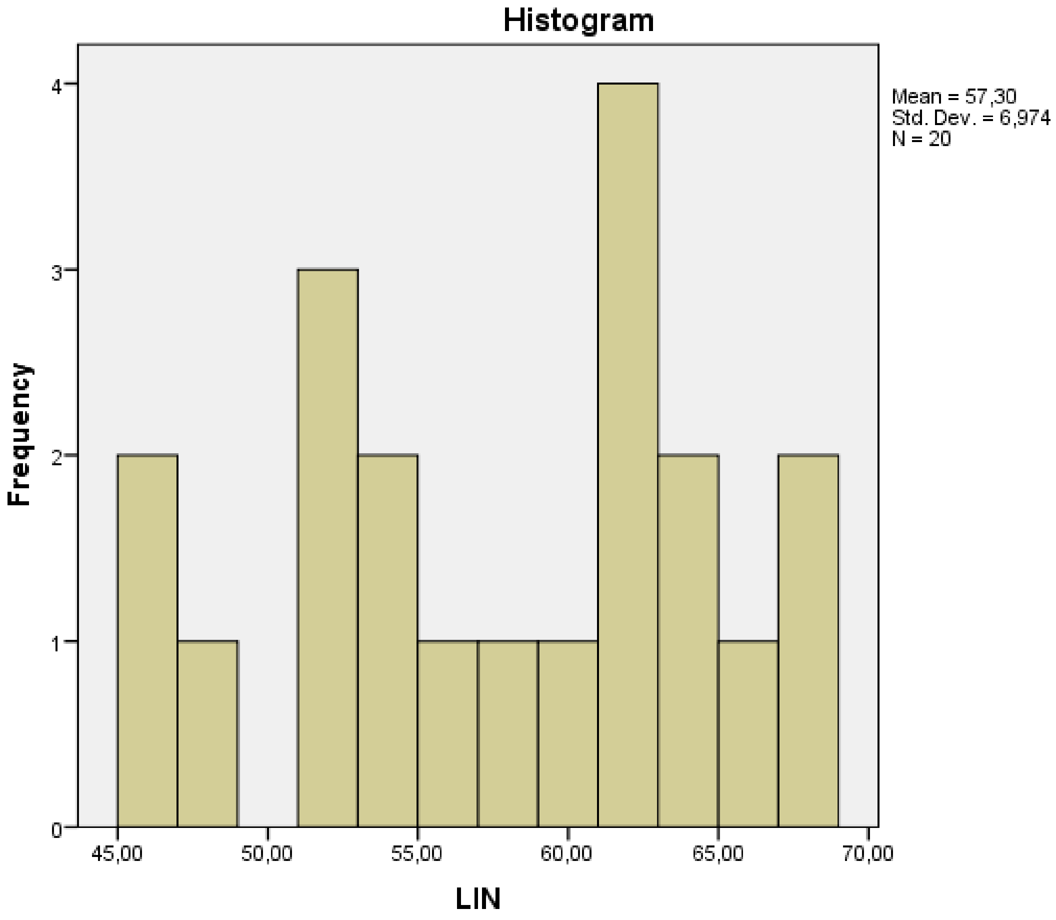

Stem-and-Leaf Plots

```
VAP Stem-and-Leaf Plot for
Gruplar= 9,00

 Frequency    Stem &  Leaf

     1,00        7 .  6
     7,00        8 .  1557788
     7,00        9 .  0333468
     3,00       10 .  078
     1,00       11 .  1
     1,00 Extremes    (>=126)

 Stem width:  10,00
 Each leaf:       1 case(s)
```

VSL Stem-and-Leaf Plot for
Gruplar= 9,00

Frequency	Stem &	Leaf
3,00	6 .	259
5,00	7 .	23477
7,00	8 .	0113368
3,00	9 .	489
1,00	10 .	4
1,00	11 .	4

Stem width: 10,00
Each leaf: 1 case(s)

VCL Stem-and-Leaf Plot for
Gruplar= 9,00

Frequency	Stem &	Leaf
3,00	12 .	078
2,00	13 .	18
6,00	14 .	001248
6,00	15 .	011156
2,00	16 .	28
1,00	17 .	2

Stem width: 10,00
Each leaf: 1 case(s)

ALH Stem-and-Leaf Plot for
Gruplar= 9,00

Frequency	Stem &	Leaf
1,00	4 .	9
7,00	5 .	1111334
4,00	5 .	6889
,00	6 .	
6,00	6 .	678899
2,00	7 .	01

Stem width: 1,00
Each leaf: 1 case(s)

```
BCF Stem-and-Leaf Plot for
Gruplar= 9,00

 Frequency    Stem &  Leaf

     1,00        2 .  7
     8,00        3 .  11222334
     9,00        3 .  567778888
     2,00        4 .  11

 Stem width:  10,00
 Each leaf:       1 case(s)
```

```
STR Stem-and-Leaf Plot for
Gruplar= 9,00

 Frequency    Stem &  Leaf

     3,00        7 .  689
     5,00        8 .  11124
     8,00        8 .  66677888
     4,00        9 .  0124

 Stem width:  10,00
 Each leaf:       1 case(s)
```

```
LIN Stem-and-Leaf Plot for
Gruplar= 9,00

 Frequency    Stem &  Leaf

     3,00        4 .  668
     5,00        5 .  11133
     2,00        5 .  68
     7,00        6 .  0112234
     3,00        6 .  578

 Stem width:  10,00
 Each leaf:       1 case(s)
```

Normal Q-Q Plots

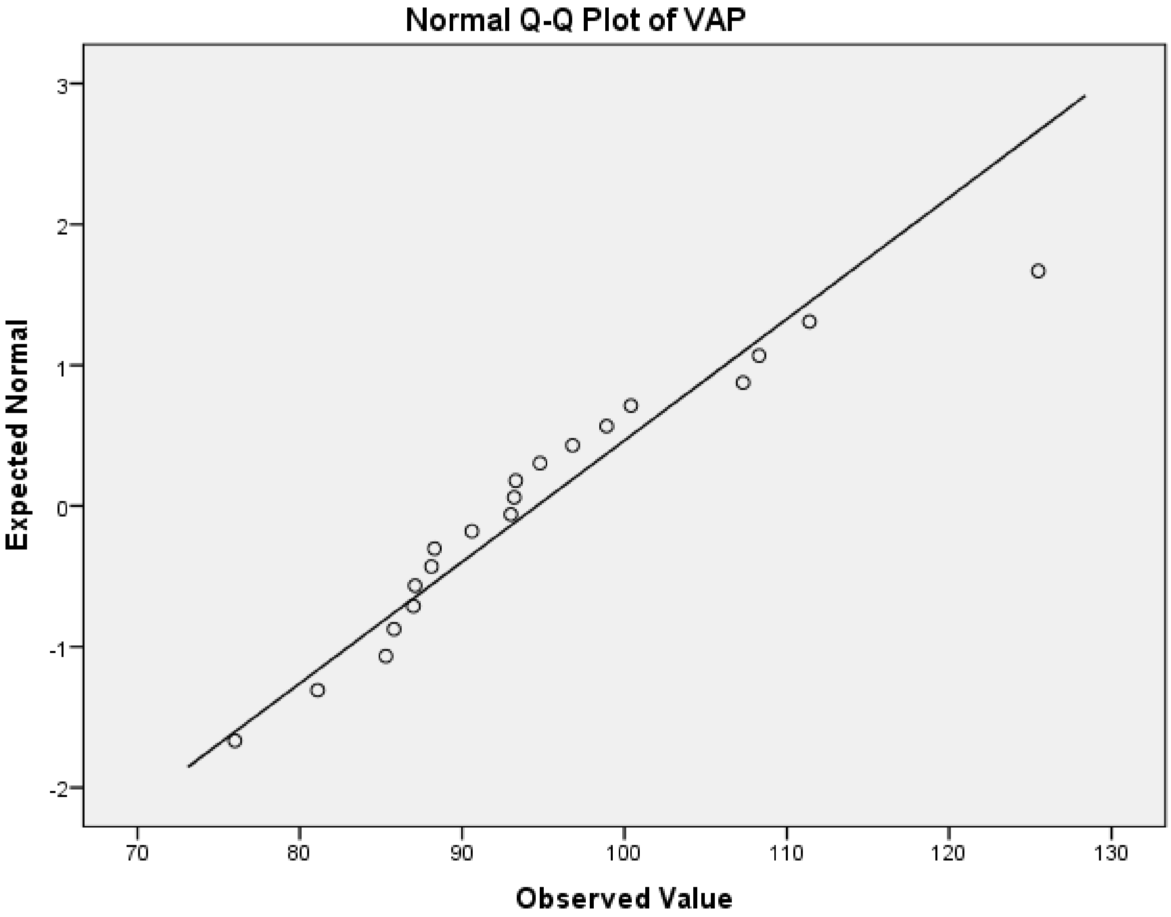
Normal Q-Q Plot of VAP
Expected Normal
3
2
1
0
-1
-2
70
80
90
100
110
120
130
Observed Value

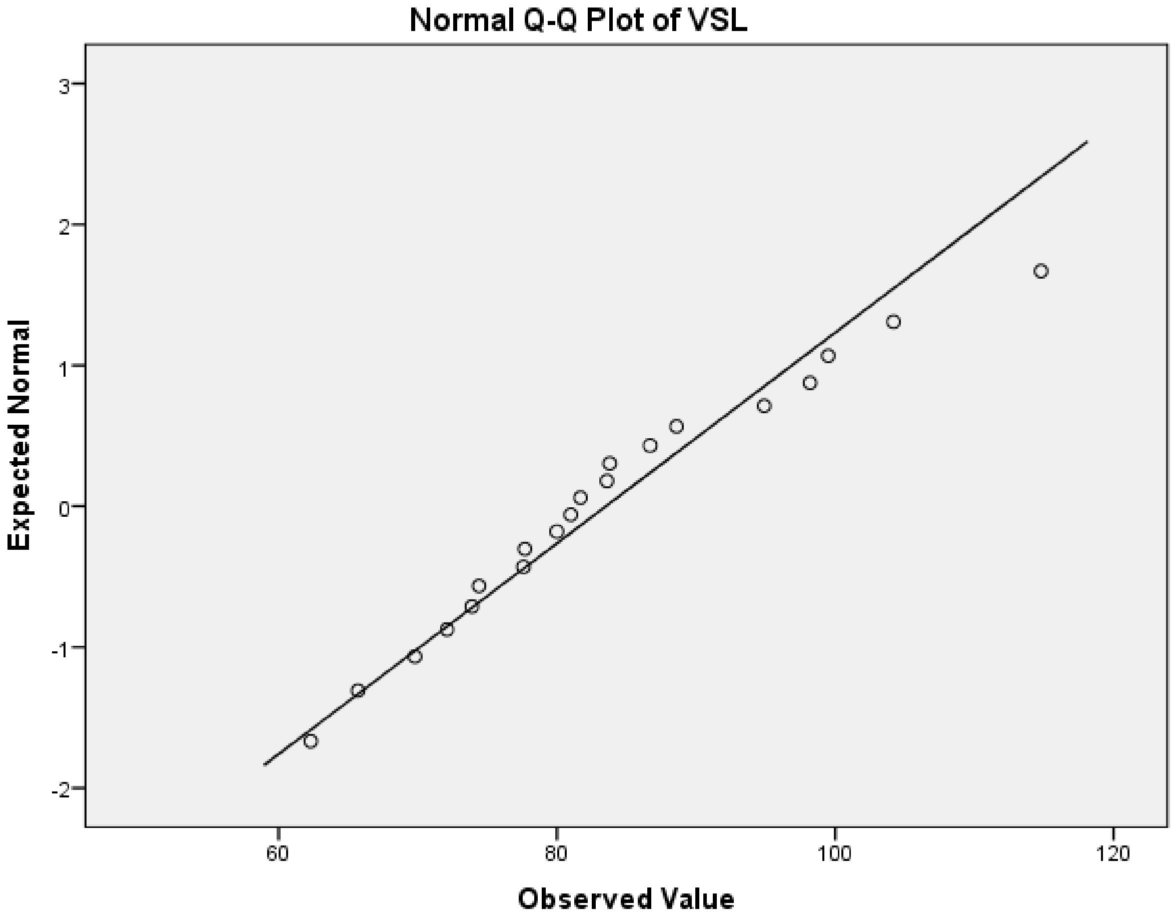

Normal Q-Q Plot of VSL
Expected Normal
3
2
1
0
-1
-2
60
80
100
120
Observed Value

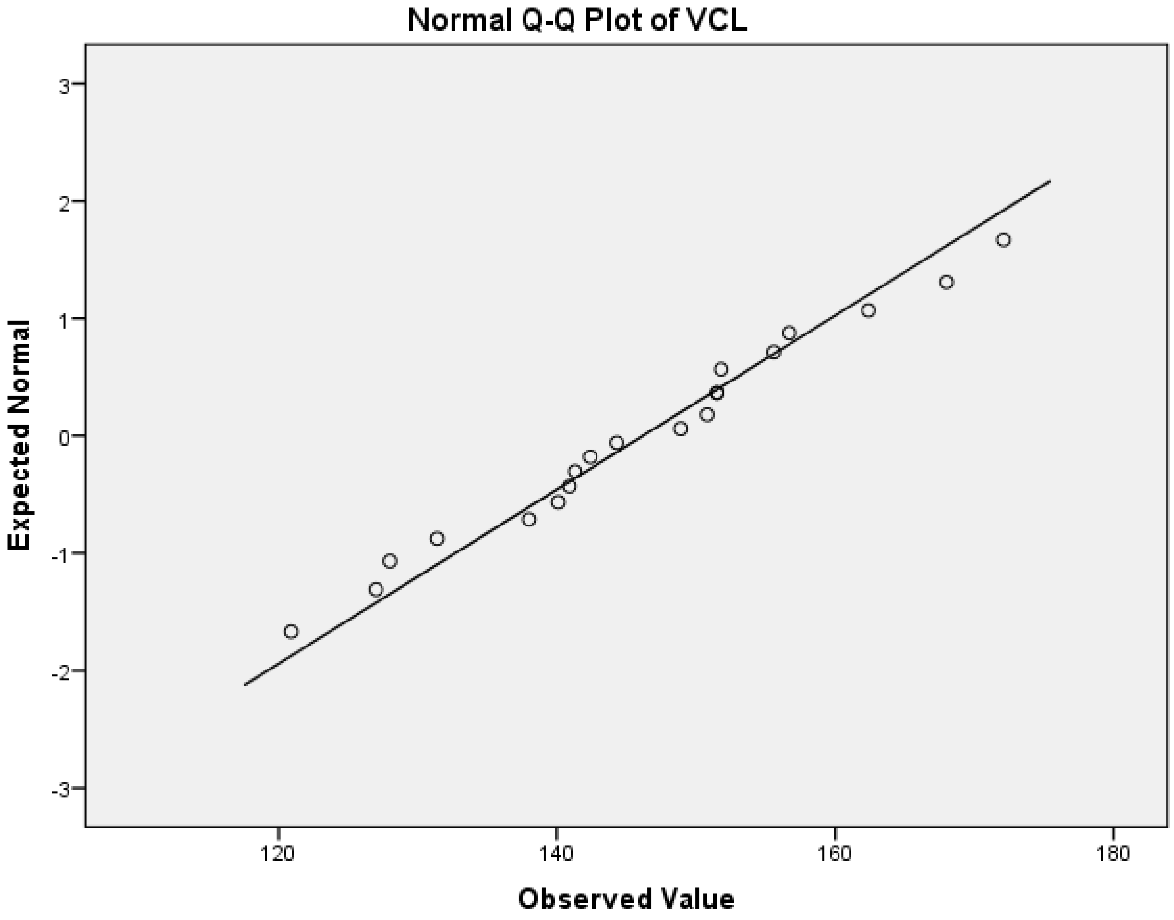
Normal Q-Q Plot of VCL
Expected Normal
Observed Value
3
2
1
0
-1
-2
-3
120
140
160
180

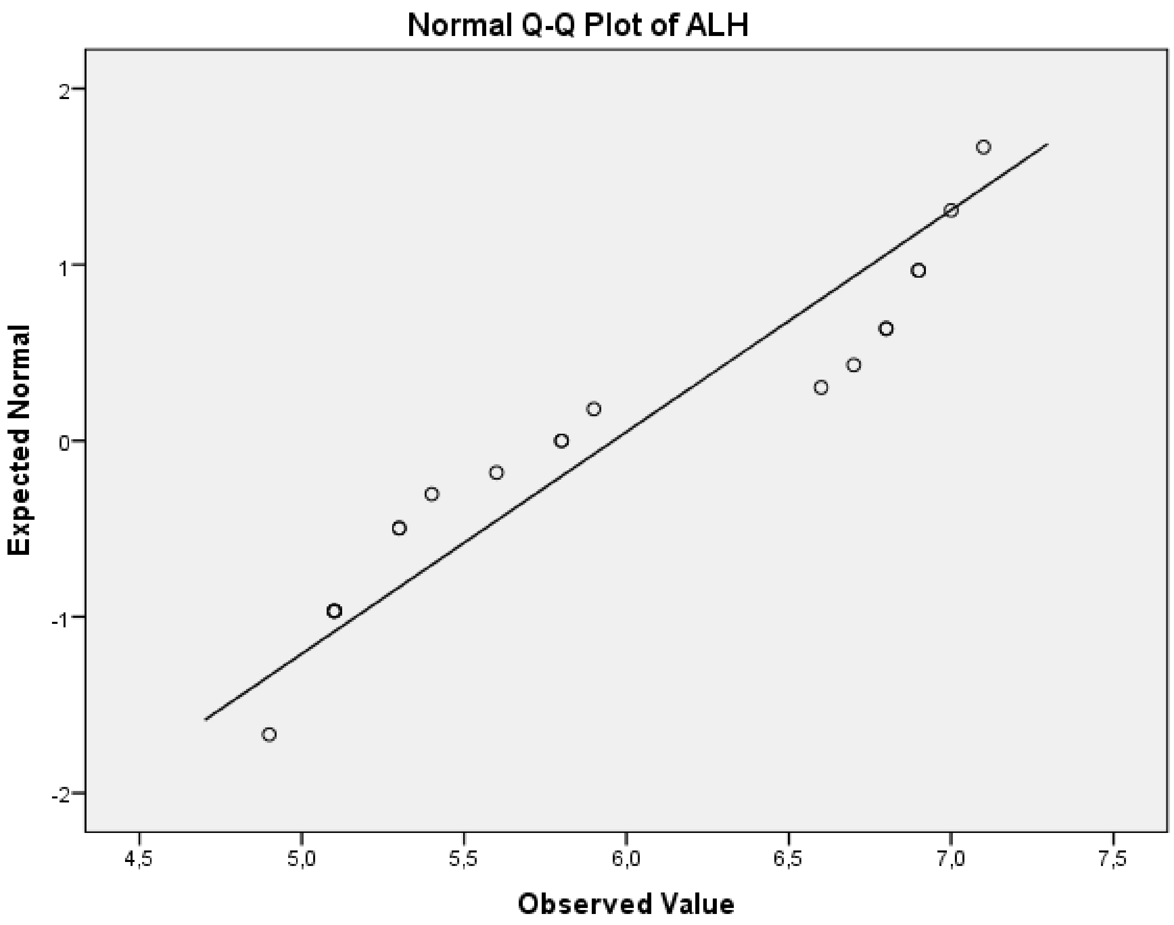

Normal Q-Q Plot of ALH
Expected Normal
2
1
0
-1
-2
4,5
5,0
5,5
6,0
6,5
7,0
7,5
Observed Value

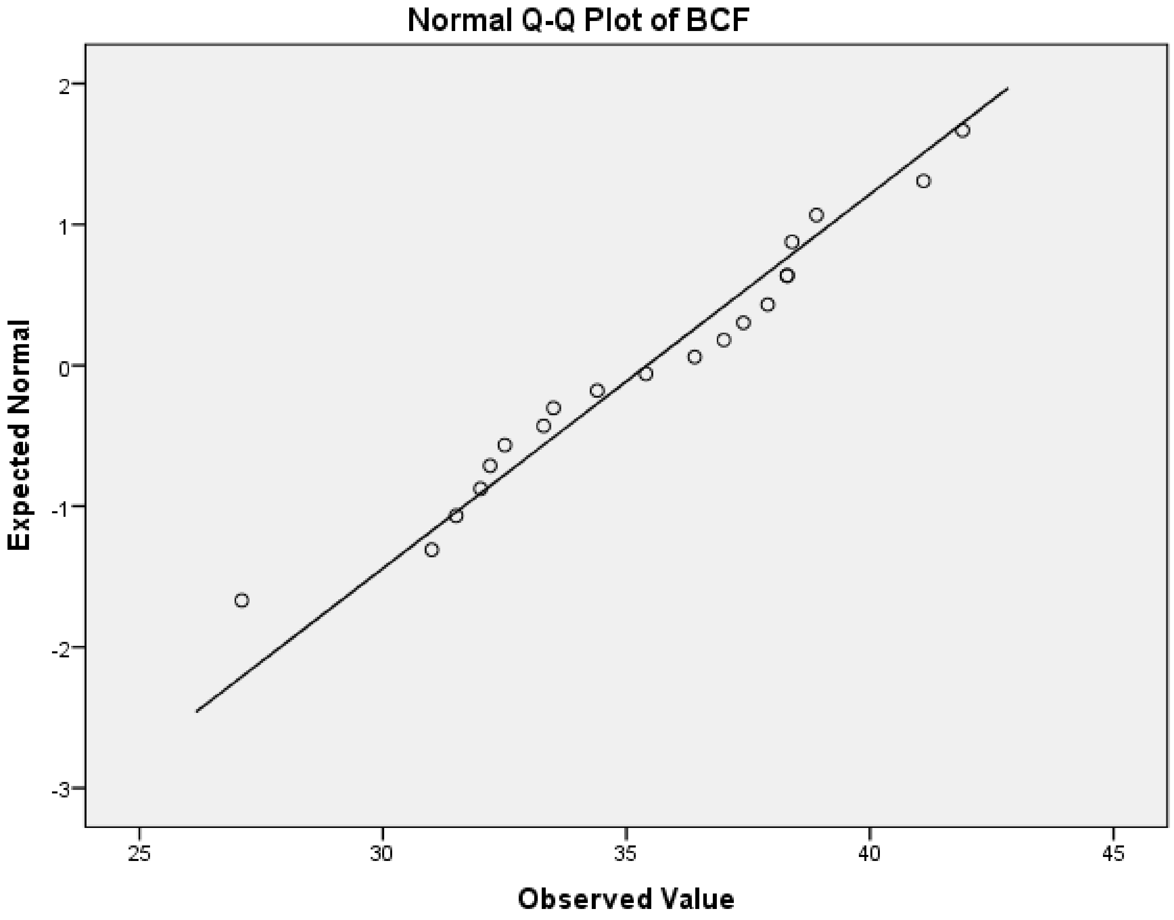
Normal Q-Q Plot of BCF
Expected Normal
Observed Value
2
1
0
-1
-2
-3
25
30
35
40
45

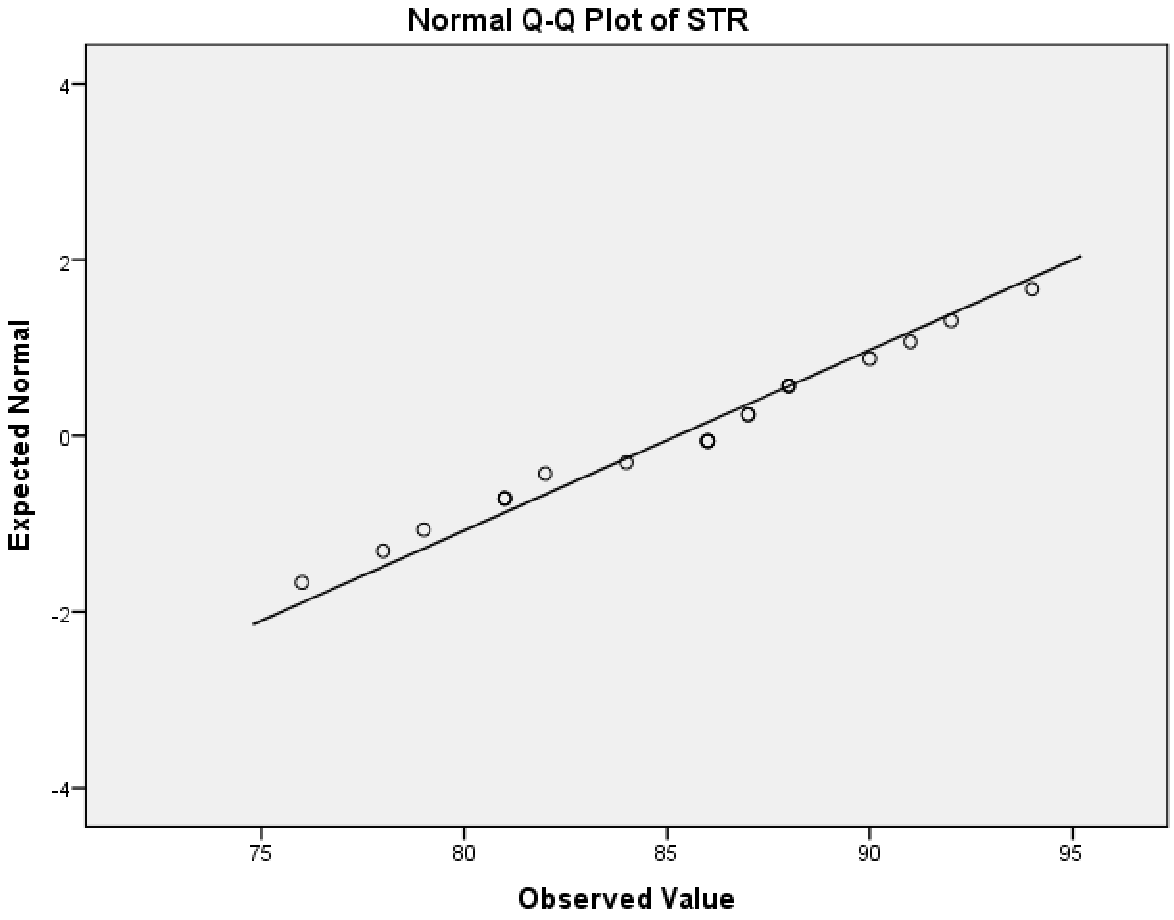

Normal Q-Q Plot of STR
Expected Normal
4
2
0
-2
-4
75
80
85
90
95
Observed Value

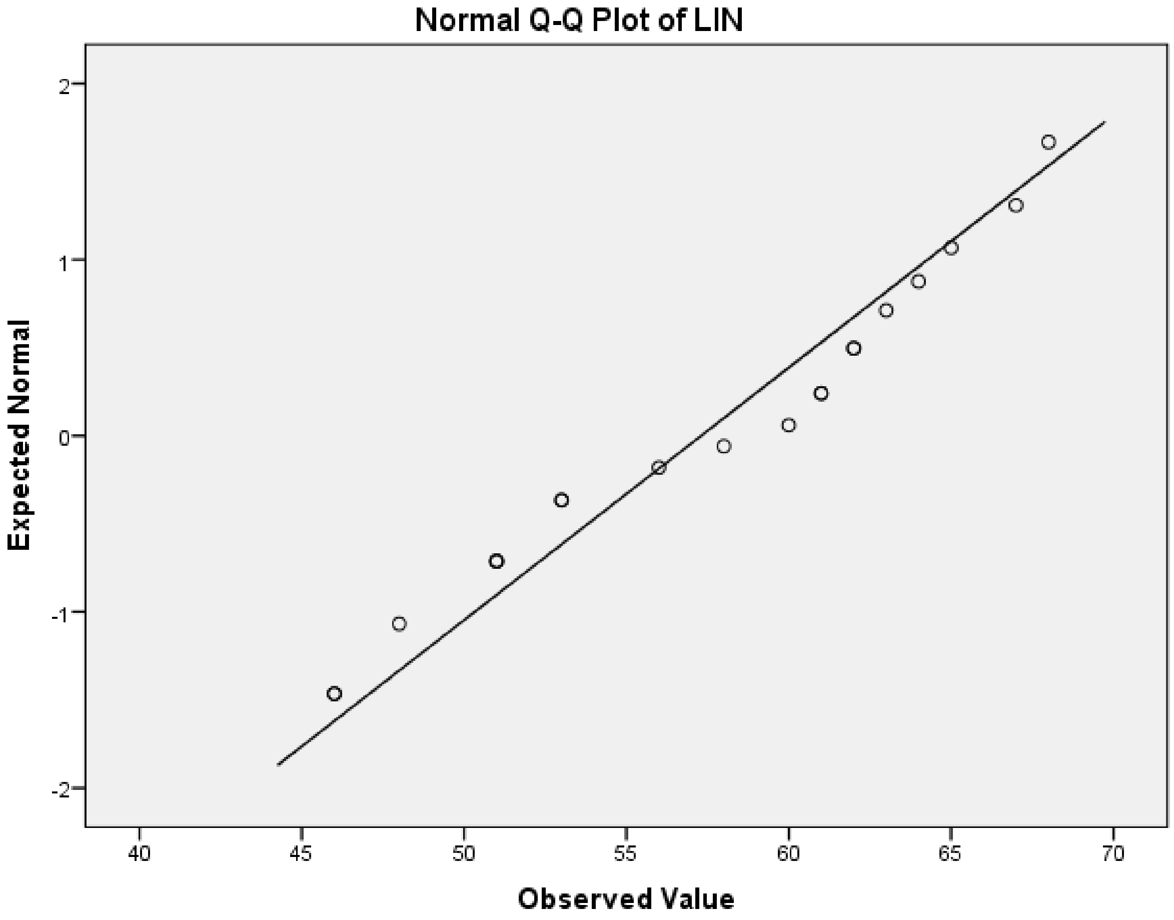

Detrended Normal Q-Q Plots

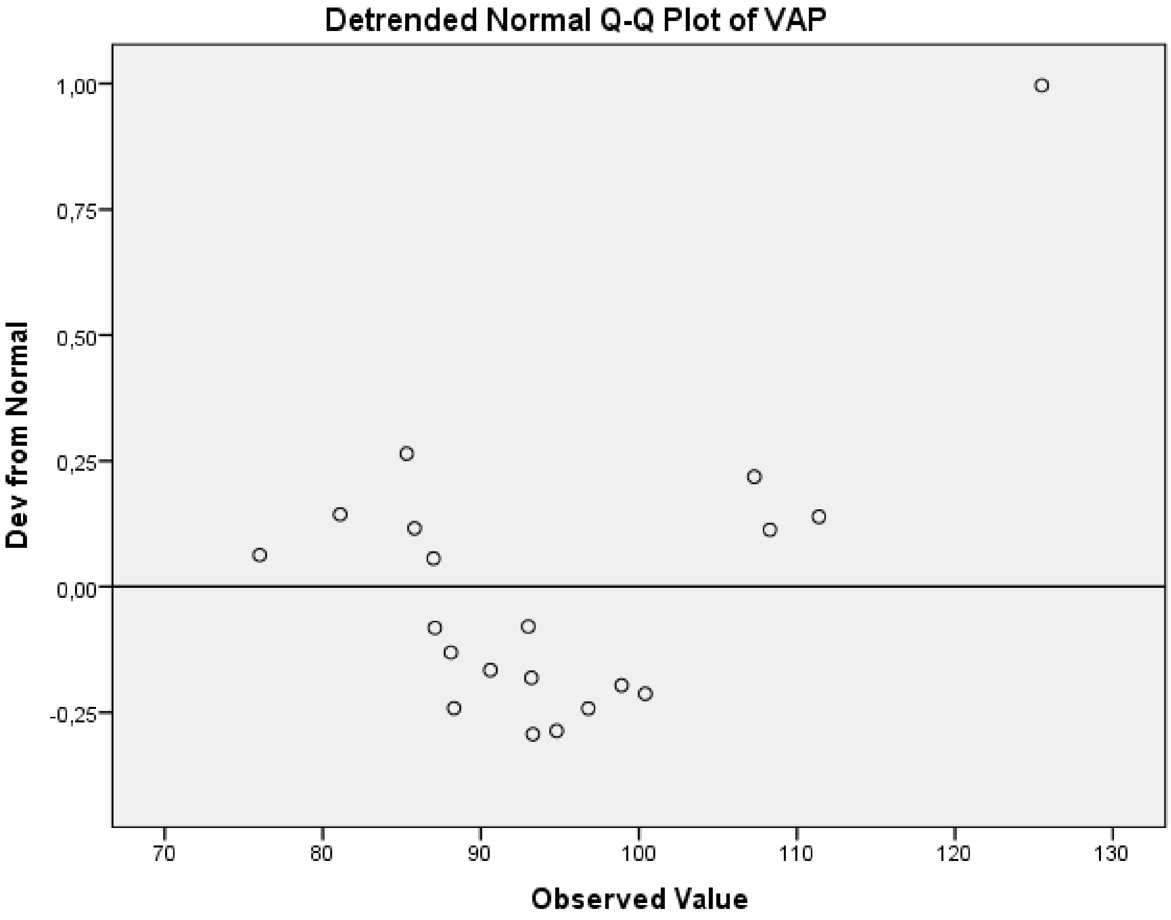

Detrended Normal Q-Q Plot of VAP
Dev from Normal
1,00
0,75
0,50
0,25
0,00
-0,25
70
80
90
100
110
120
130
Observed Value

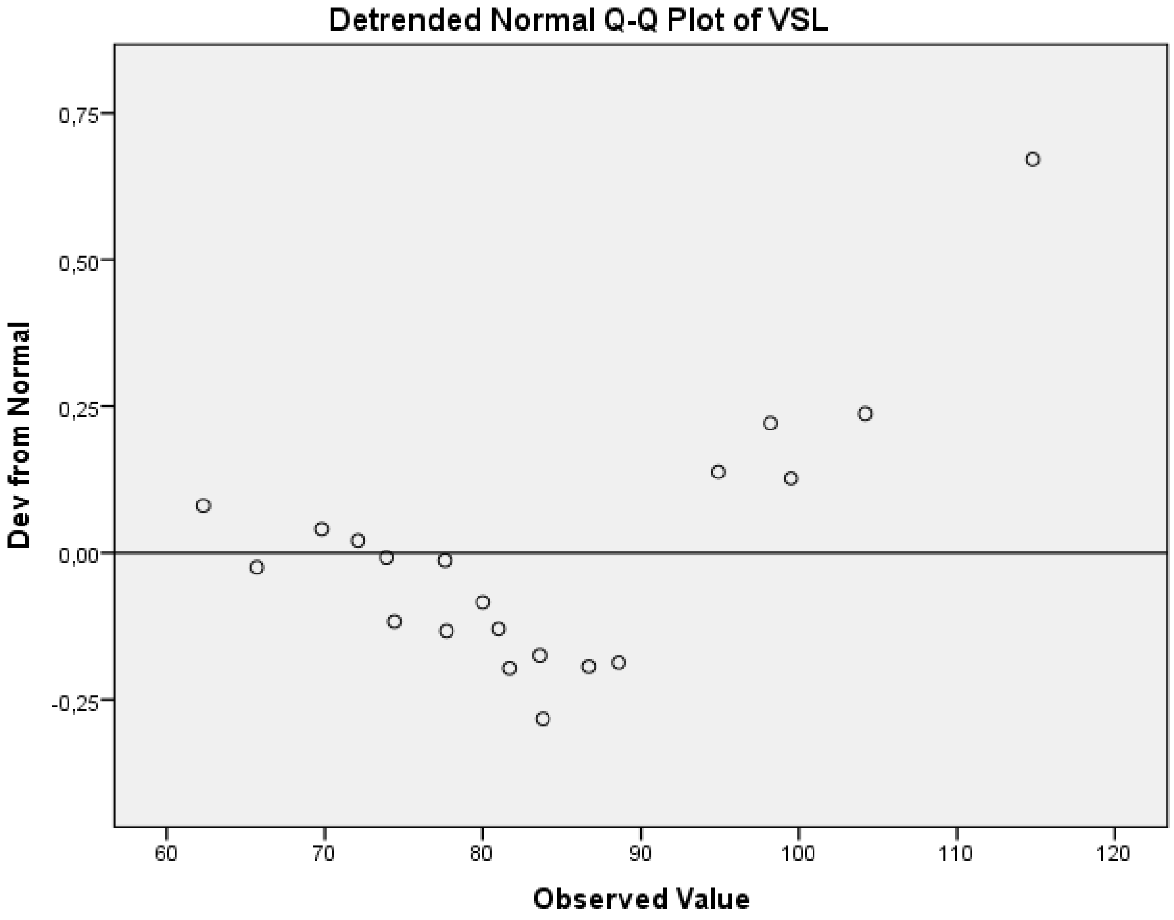
Detrended Normal Q-Q Plot of VSL
Dev from Normal
0,75
0,50
0,25
0,00
-0,25
60
70
80
90
100
110
120
Observed Value

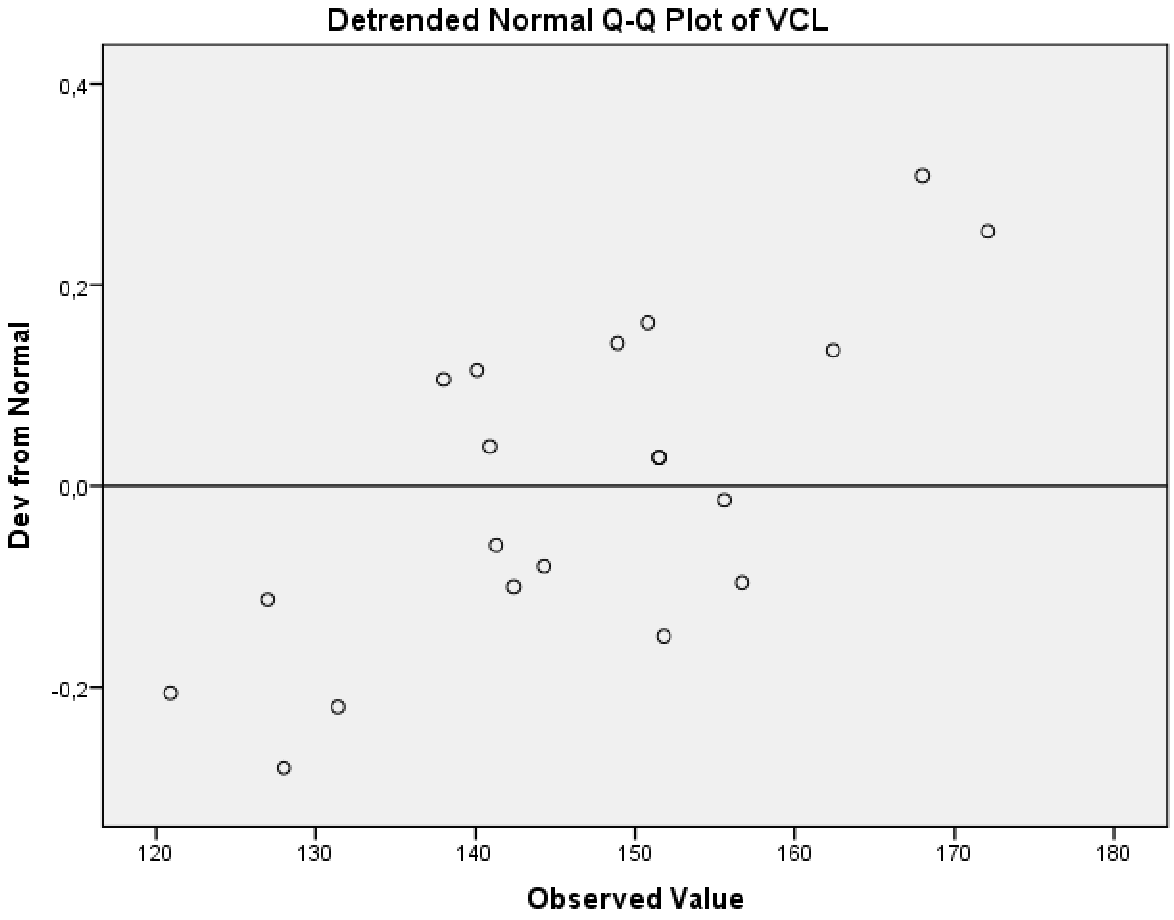
Detrended Normal Q-Q Plot of VCL
Dev from Normal
0,4
0,2
0,0
-0,2
120
130
140
150
160
170
180
Observed Value

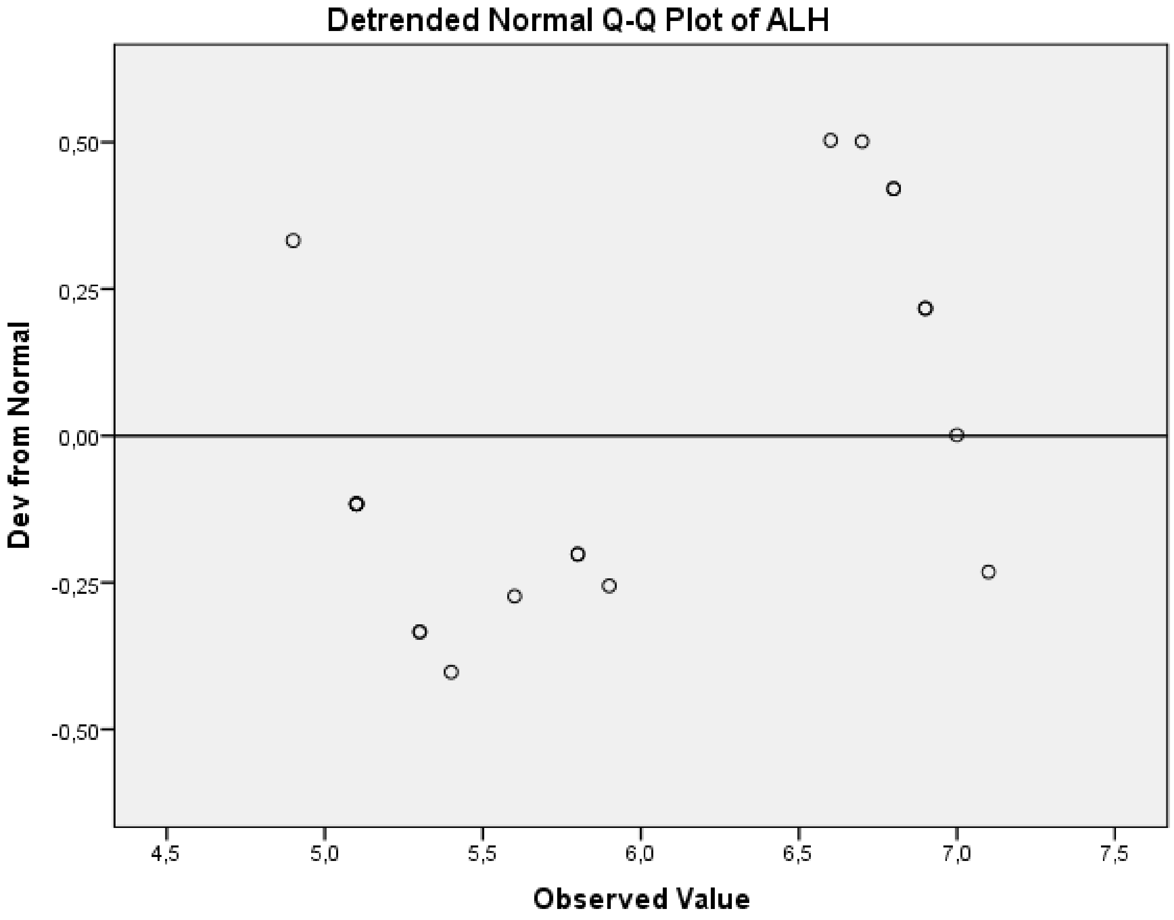
Detrended Normal Q-Q Plot of ALH
Dev from Normal
0,50
0,25
0,00
-0,25
-0,50
4,5
5,0
5,5
6,0
6,5
7,0
7,5
Observed Value

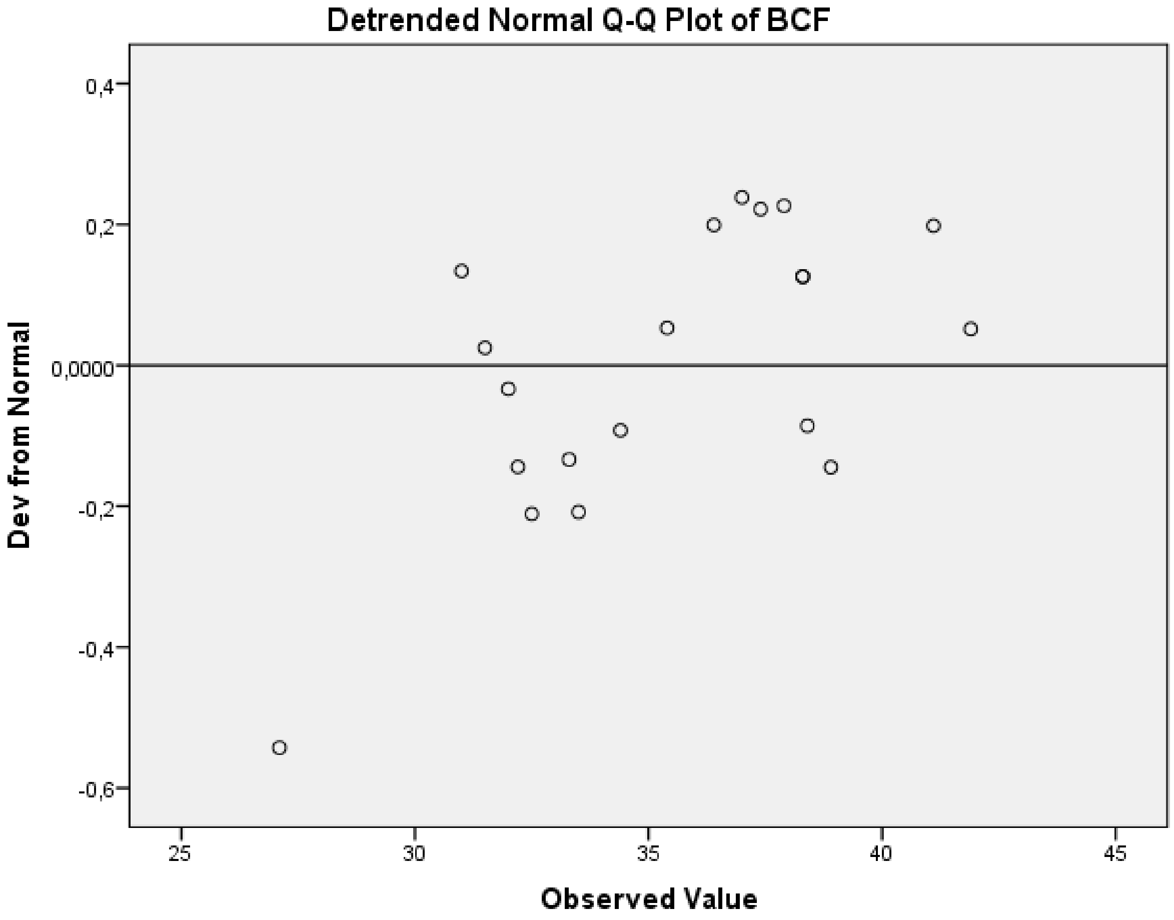

Detrended Normal Q-Q Plot of BCF
Dev from Normal
0,4
0,2
0,0000
-0,2
-0,4
-0,6
25
30
35
40
45
Observed Value

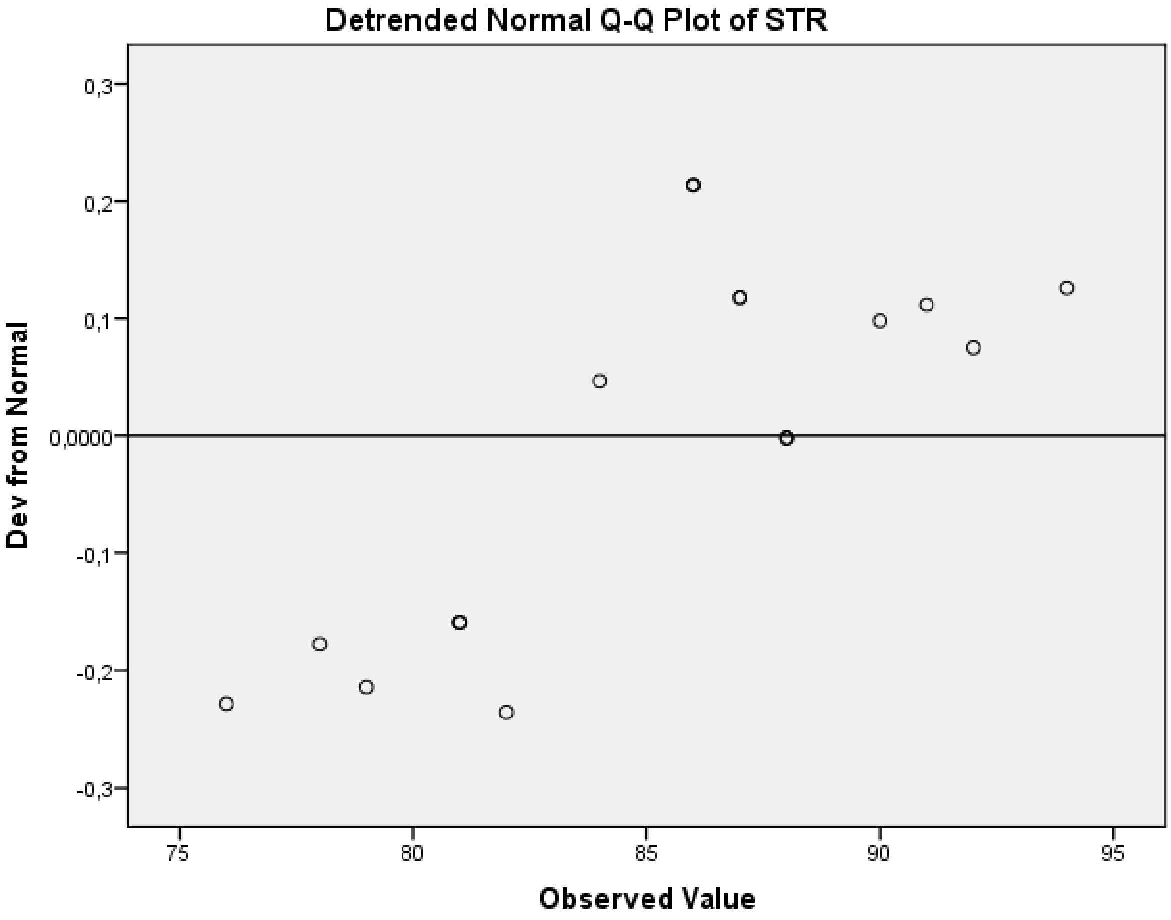
Detrended Normal Q-Q Plot of STR
Dev from Normal
0,3
0,2
0,1
0,0000
-0,1
-0,2
-0,3
75
80
85
90
95
Observed Value

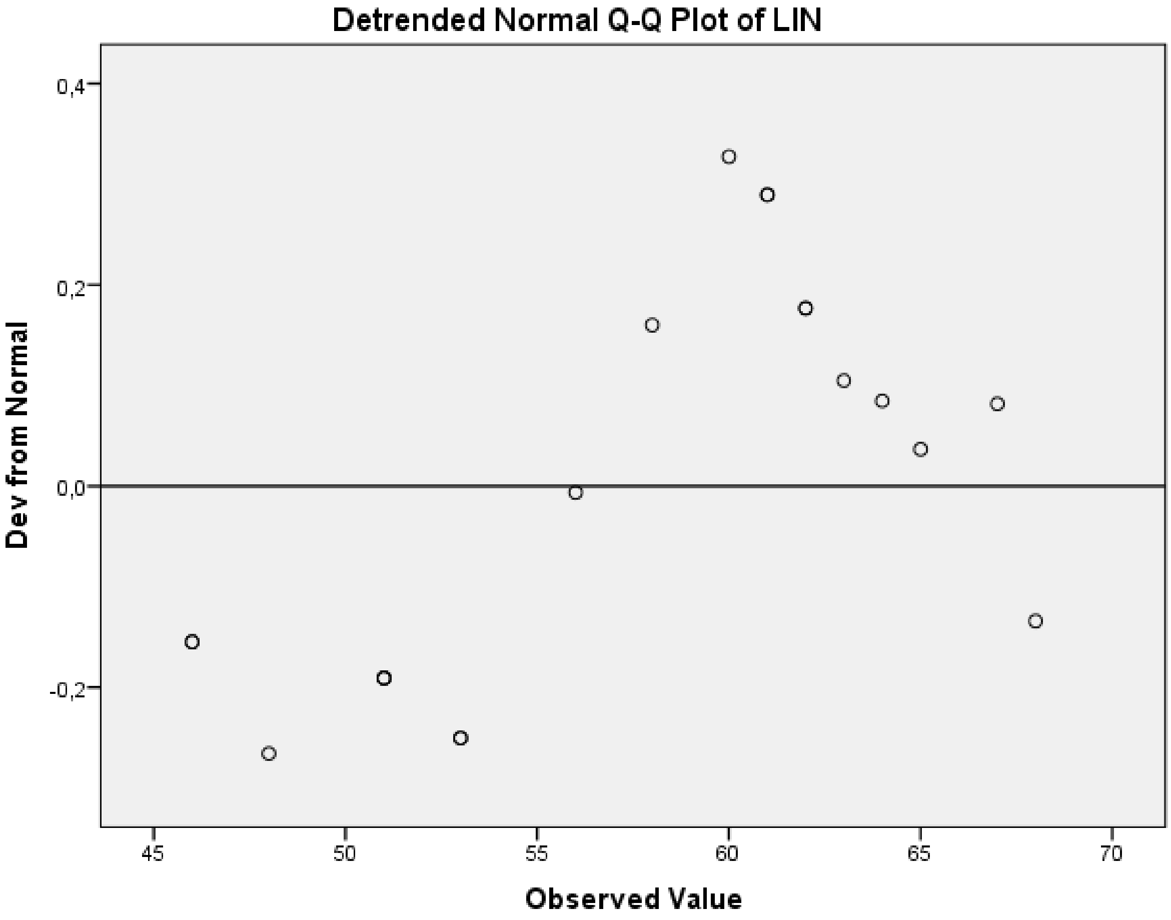

Gruplar = 10,00

Histograms

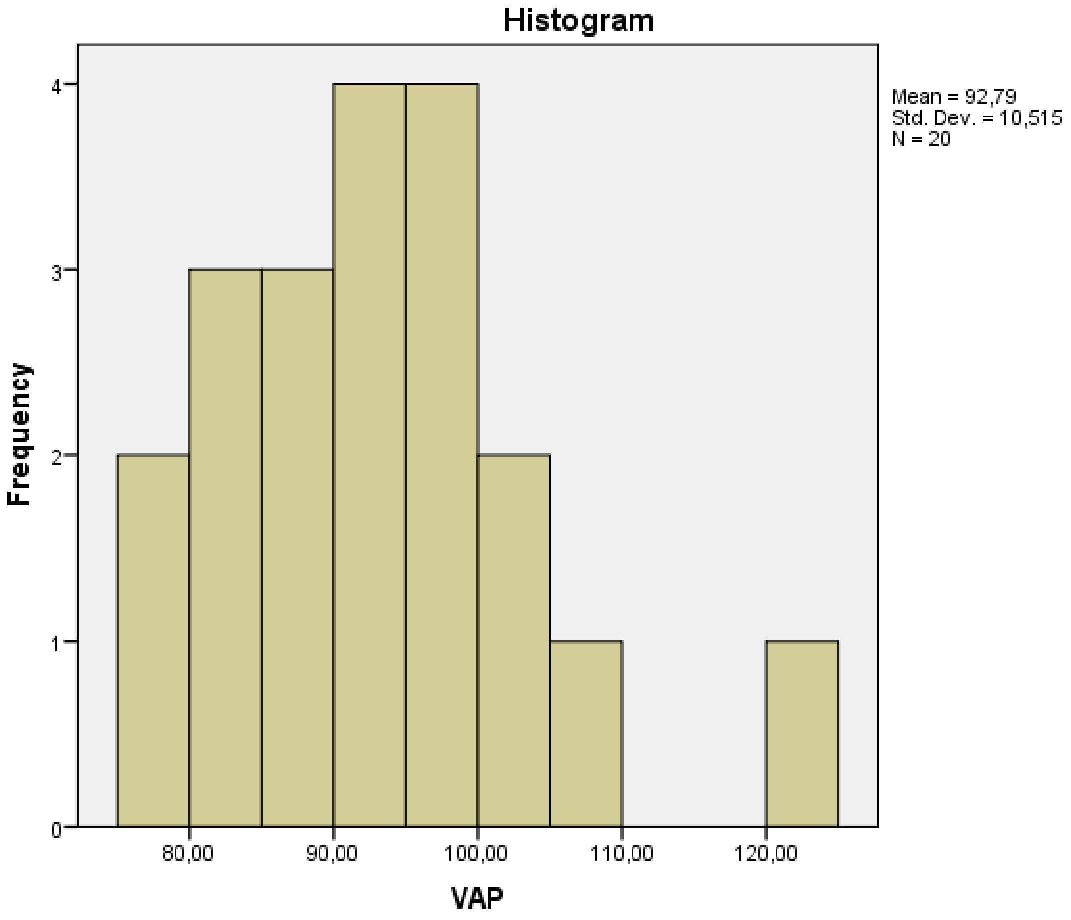
Histogram
Mean = 92,79
Std. Dev. = 10,515
N = 20
Frequency
0
1
2
3
4
80,00
90,00
100,00
110,00
120,00
VAP

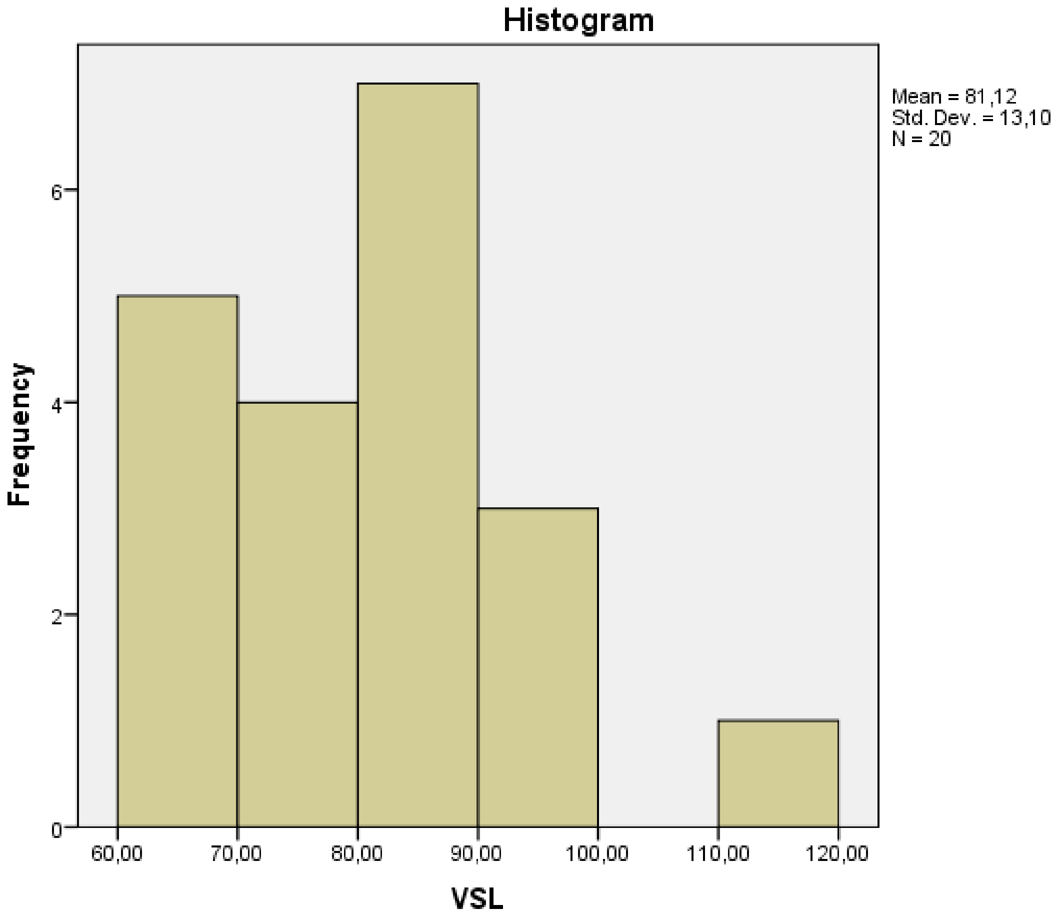

Histogram
Mean = 81,12
Std. Dev. = 13,10
N = 20
Frequency
0
2
4
6
60,00
70,00
80,00
90,00
100,00
110,00
120,00
VSL

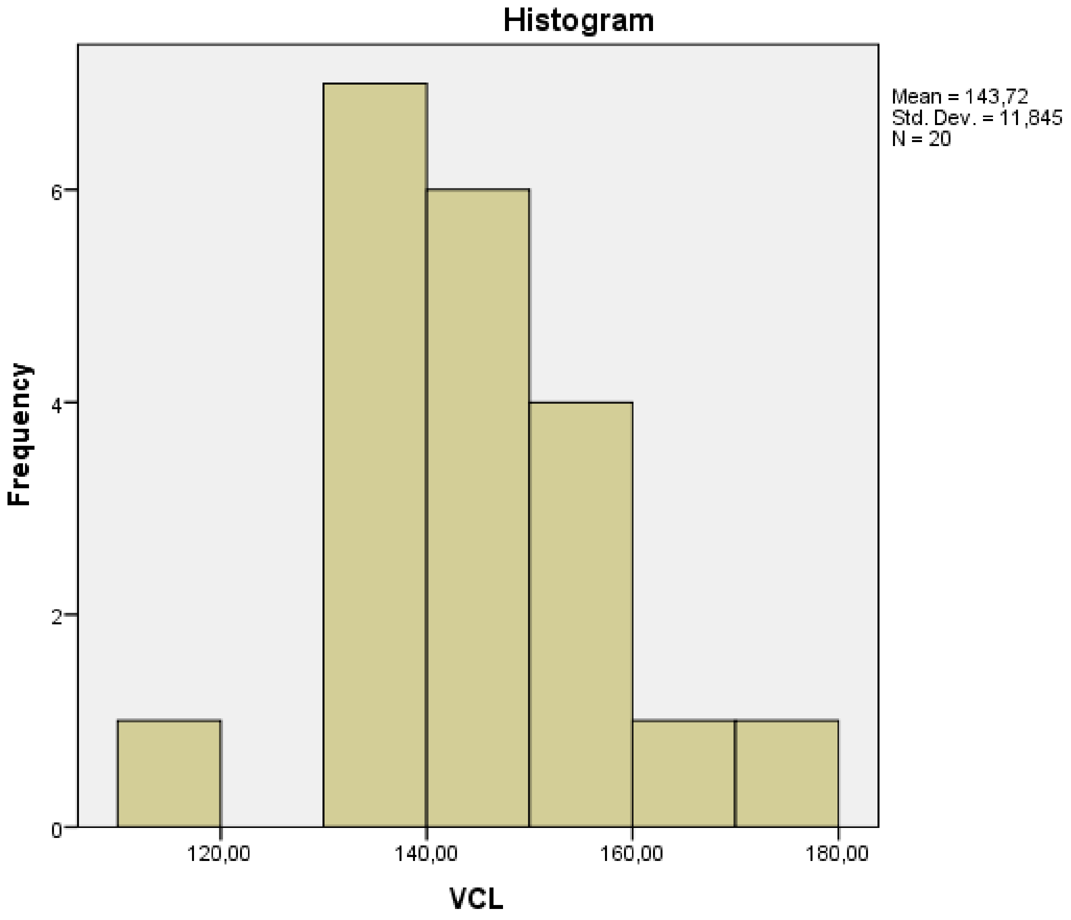

Histogram
Mean = 143,72
Std. Dev. = 11,845
N = 20
Frequency
0
2
4
6
120,00
140,00
160,00
180,00
VCL

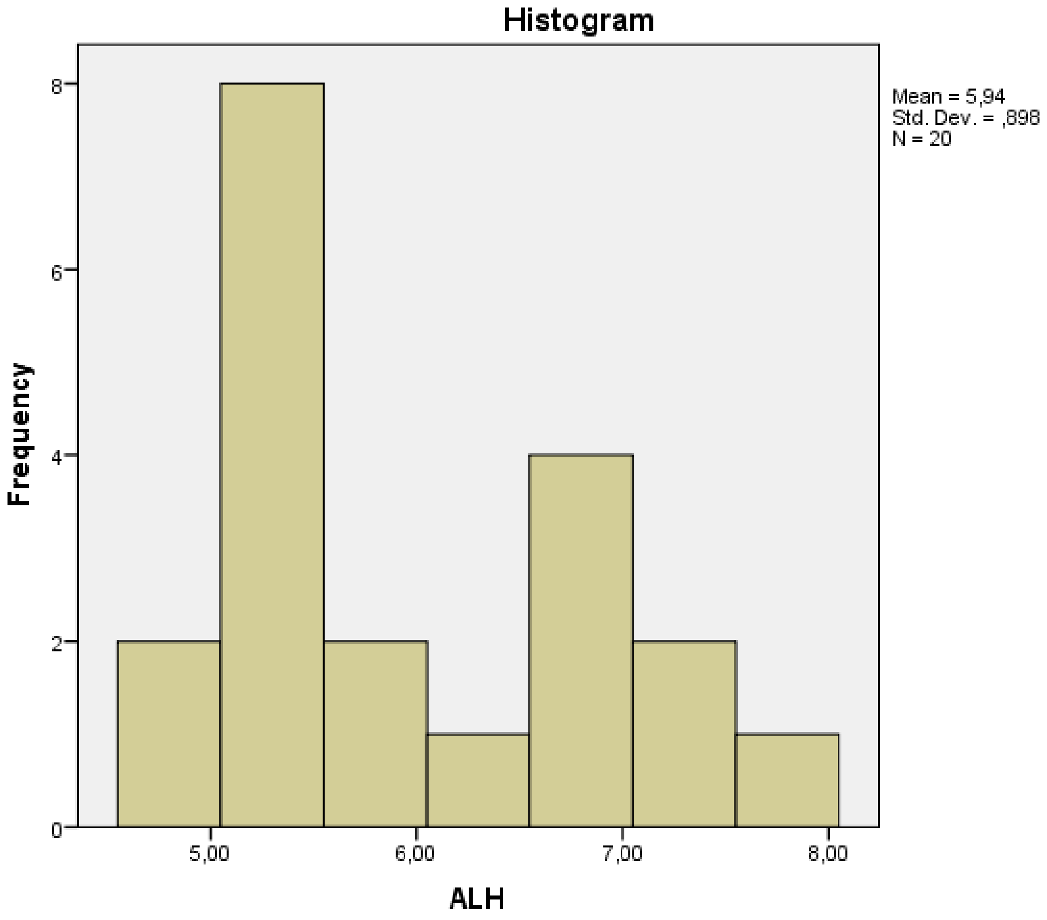

Histogram
Mean = 5,94
Std. Dev. = ,898
N = 20
Frequency
0
2
4
6
8
5,00
6,00
7,00
8,00
ALH

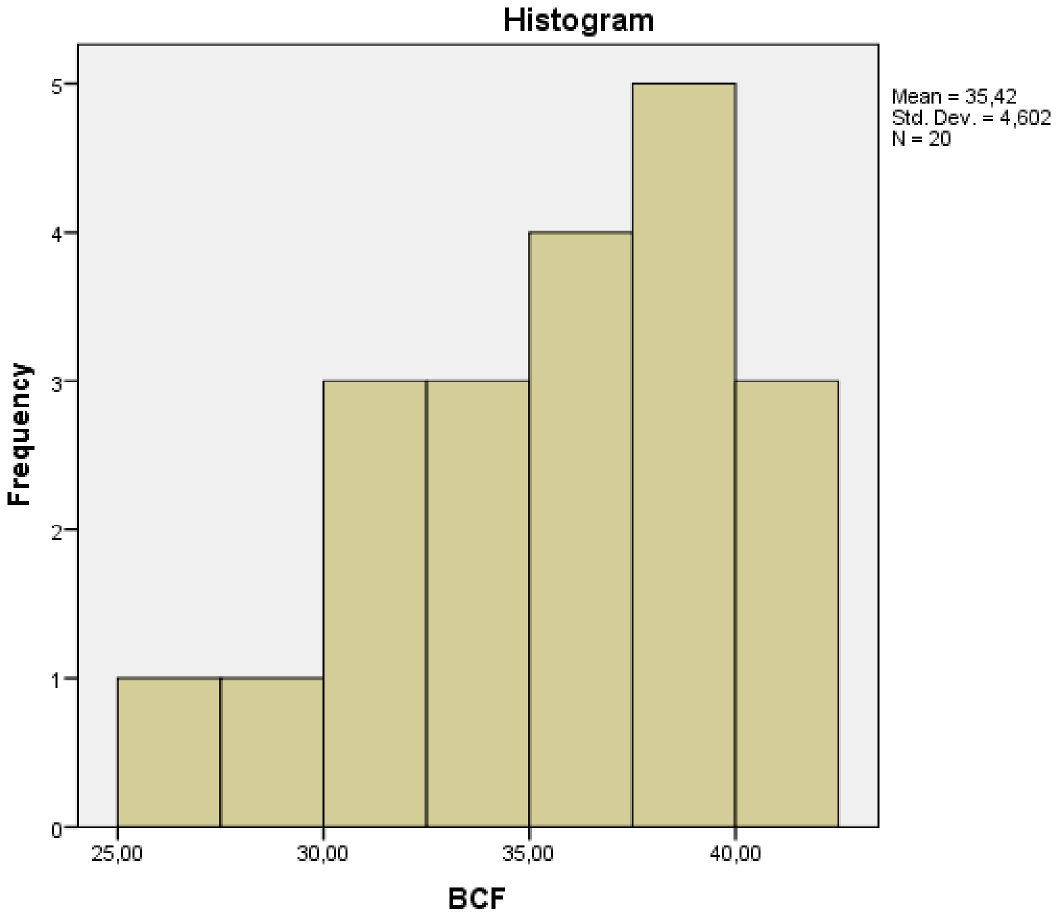
Histogram
Mean = 35,42
Std. Dev. = 4,602
N = 20
Frequency
0
1
2
3
4
5
25,00
30,00
35,00
40,00
BCF

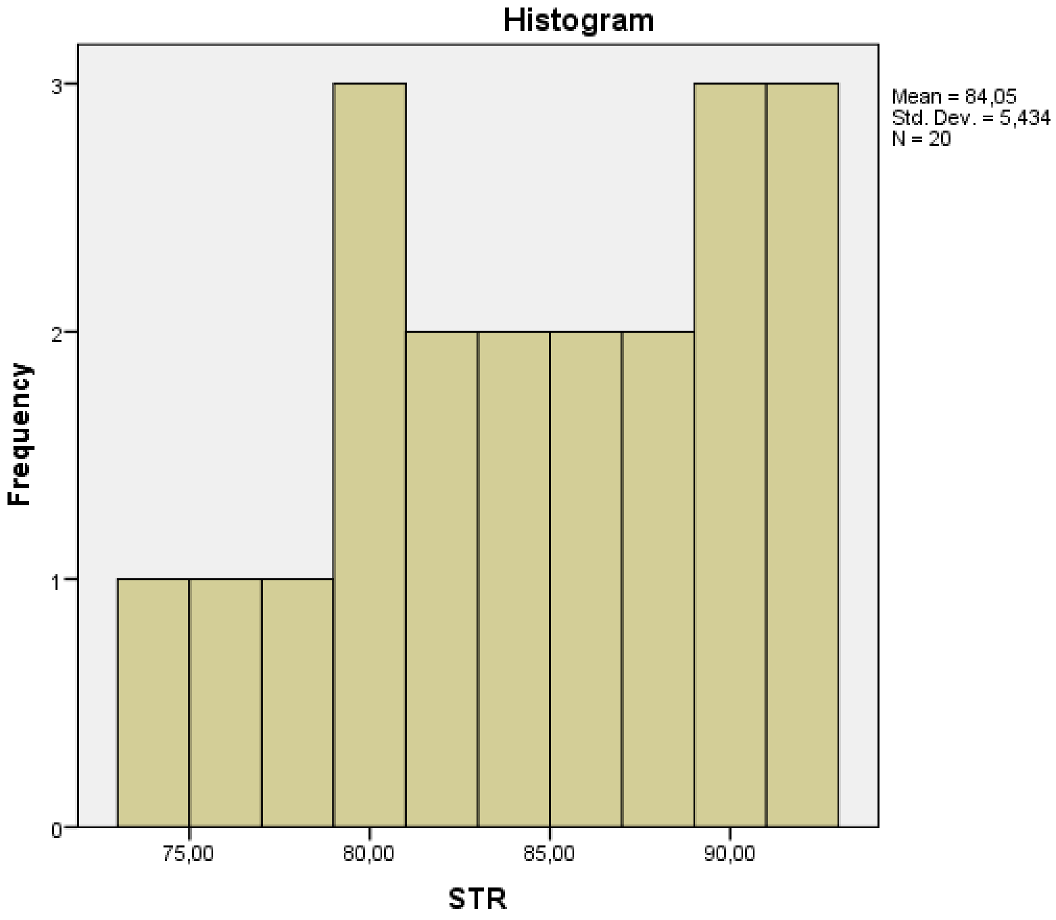
Histogram
Mean = 84,05
Std. Dev. = 5,434
N = 20
Frequency
0
1
2
3
75,00
80,00
85,00
90,00
STR

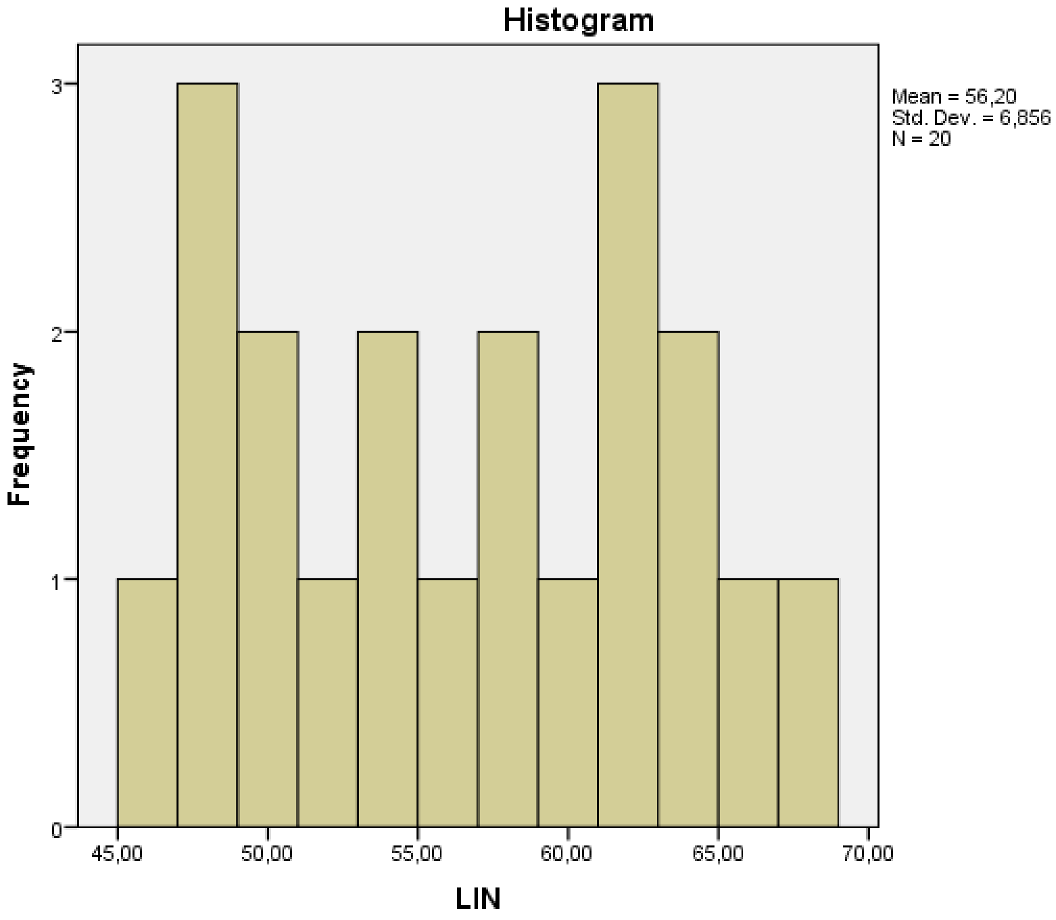

Stem-and-Leaf Plots

```
VAP Stem-and-Leaf Plot for
Gruplar= 10,00

 Frequency    Stem &  Leaf

     2,00        7 .  99
     3,00        8 .  033
     3,00        8 .  678
     4,00        9 .  0123
     4,00        9 .  5678
     2,00       10 .  12
     1,00       10 .  5
     1,00 Extremes    (>=123)

 Stem width:  10,00
 Each leaf:       1 case(s)
```

VSL Stem-and-Leaf Plot for
Gruplar= 10,00

Frequency	Stem &	Leaf
5,00	6 .	25558
4,00	7 .	0699
7,00	8 .	0015689
3,00	9 .	037
,00	10 .	
1,00	11 .	5

Stem width: 10,00
Each leaf: 1 case(s)

VCL Stem-and-Leaf Plot for
Gruplar= 10,00

Frequency	Stem &	Leaf
1,00	11 .	9
,00	12 .	
7,00	13 .	1456777
6,00	14 .	003457
4,00	15 .	0003
1,00	16 .	1
1,00	Extremes	(>=176)

Stem width: 10,00
Each leaf: 1 case(s)

ALH Stem-and-Leaf Plot for
Gruplar= 10,00

Frequency	Stem &	Leaf
2,00	4 .	89
7,00	5 .	1222234
3,00	5 .	589
1,00	6 .	4
4,00	6 .	6677
1,00	7 .	1
2,00	7 .	57

Stem width: 1,00
Each leaf: 1 case(s)

```
BCF Stem-and-Leaf Plot for
Gruplar= 10,00

 Frequency    Stem &  Leaf

     2,00        2 .  58
     6,00        3 .  000234
     9,00        3 .  555788889
     3,00        4 .  022

 Stem width:  10,00
 Each leaf:       1 case(s)
```

```
STR Stem-and-Leaf Plot for
Gruplar= 10,00

 Frequency    Stem &  Leaf

     1,00        7 .  4
     4,00        7 .  5899
     5,00        8 .  01144
     6,00        8 .  557899
     4,00        9 .  0111

 Stem width:  10,00
 Each leaf:       1 case(s)
```

```
LIN Stem-and-Leaf Plot for
Gruplar= 10,00

 Frequency    Stem &  Leaf

     6,00        4 .  678899
     3,00        5 .  244
     3,00        5 .  688
     6,00        6 .  012233
     2,00        6 .  68

 Stem width:  10,00
 Each leaf:       1 case(s)
```

Normal Q-Q Plots

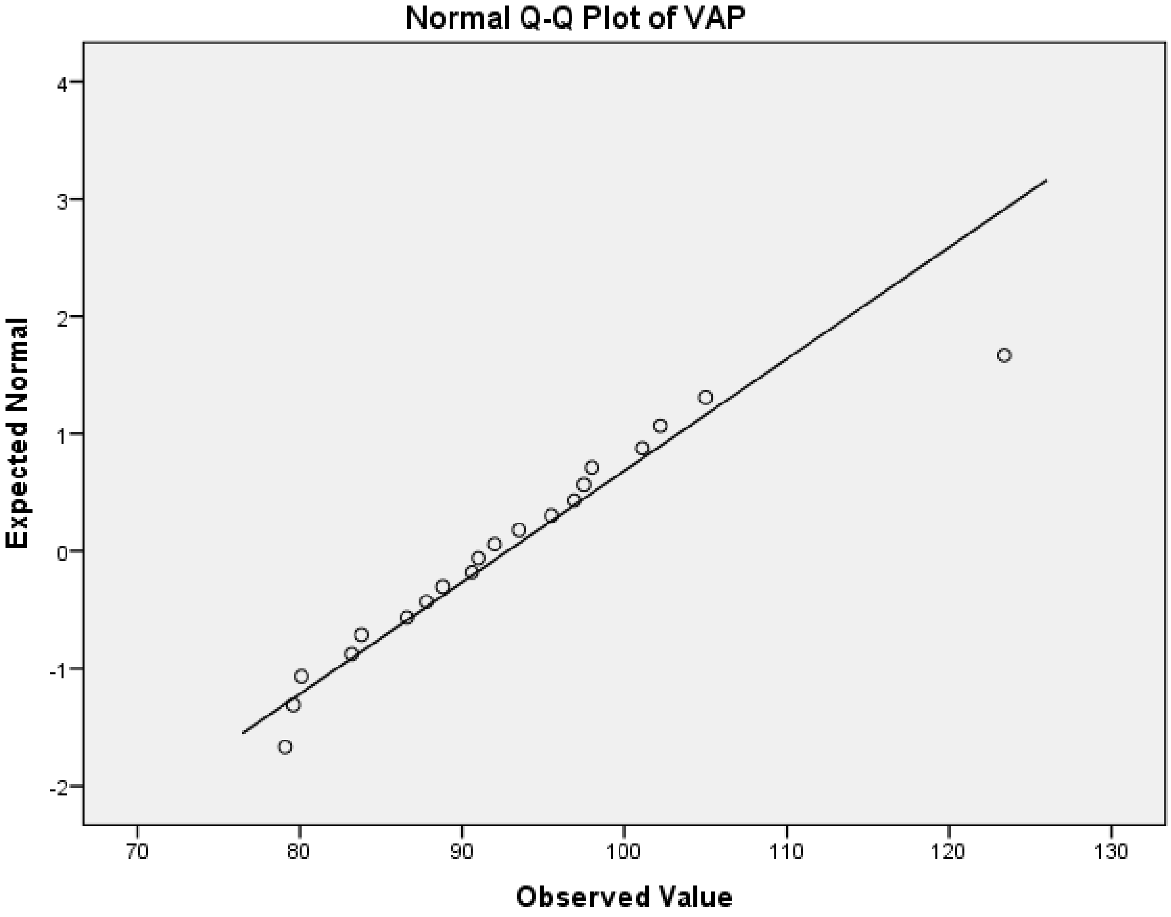

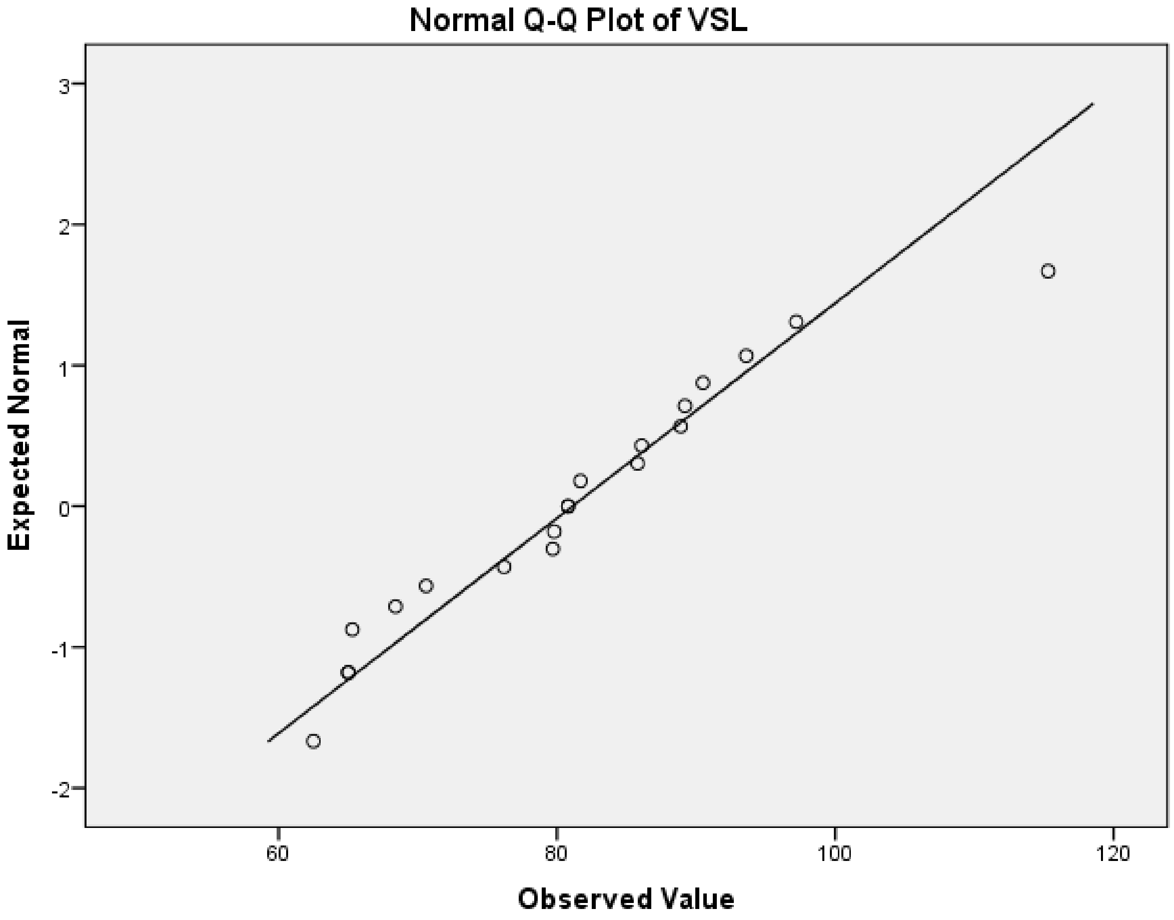

Normal Q-Q Plot of VSL
Expected Normal
3
2
1
0
-1
-2
60
80
100
120
Observed Value

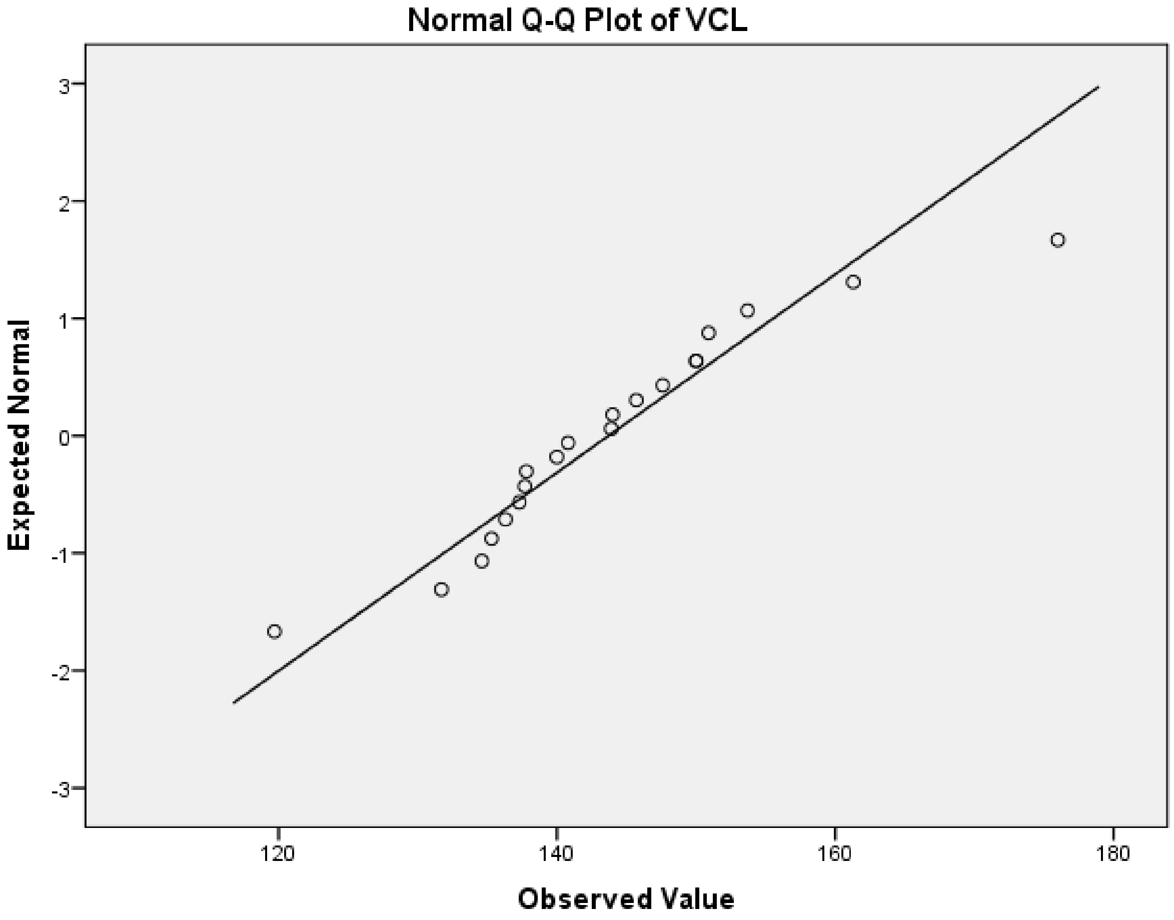

Normal Q-Q Plot of VCL
Expected Normal
Observed Value
3
2
1
0
-1
-2
-3
120
140
160
180

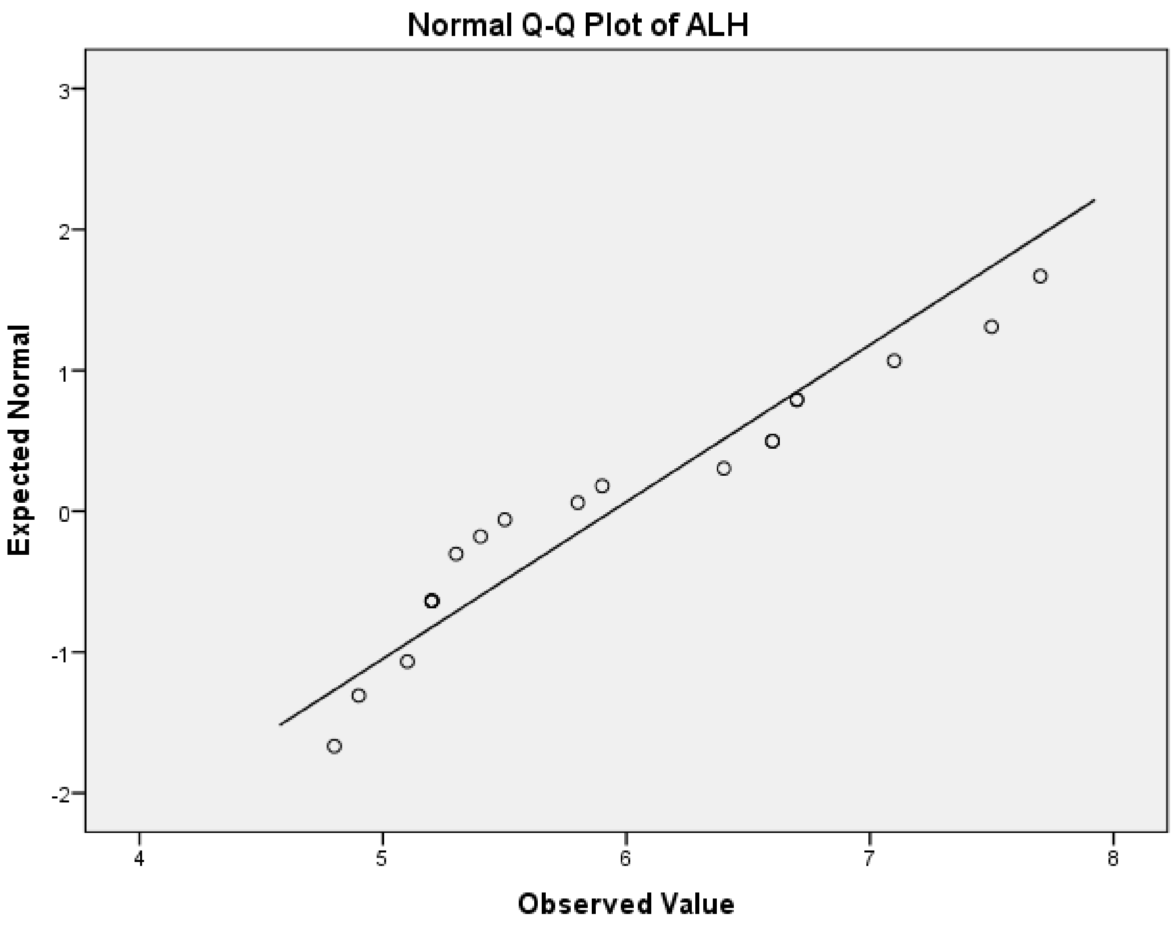
Normal Q-Q Plot of ALH
Expected Normal
Observed Value
3
2
1
0
-1
-2
4
5
6
7
8

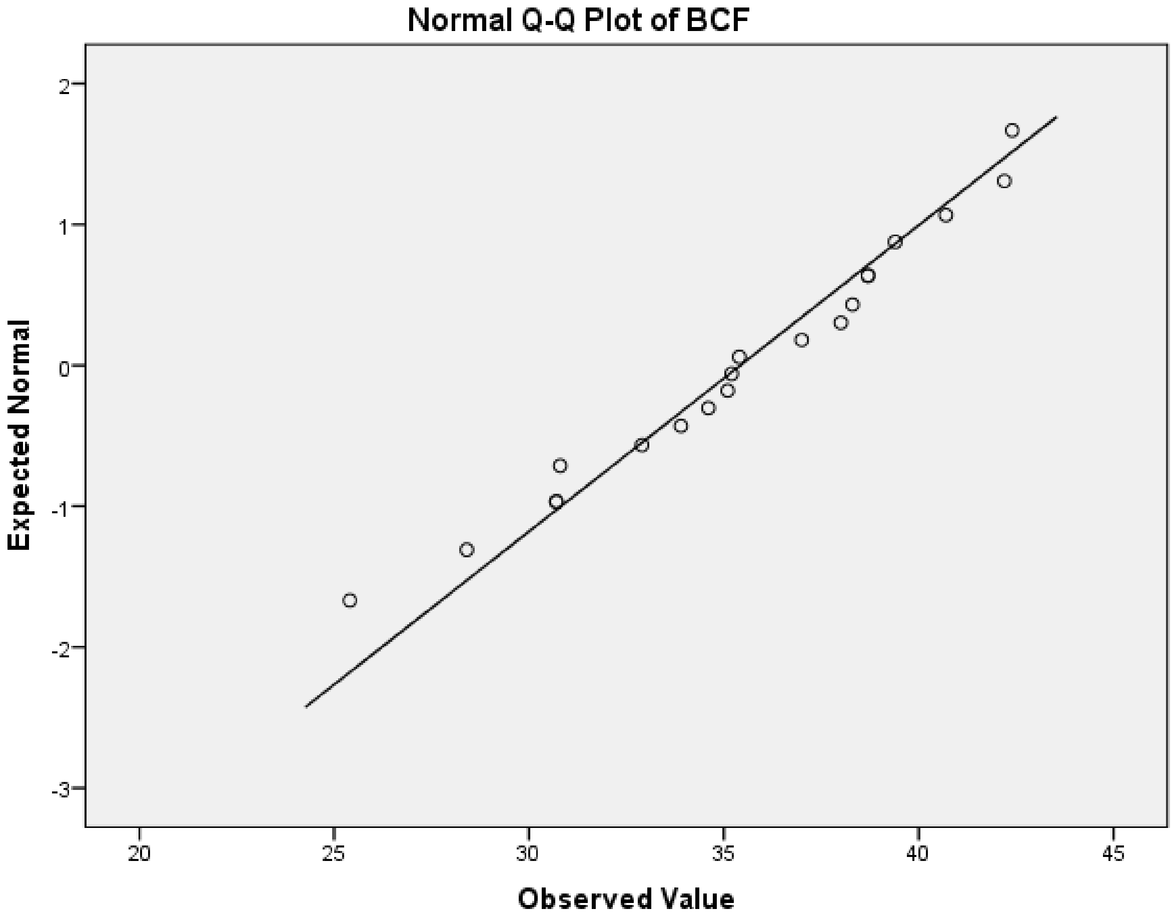
Normal Q-Q Plot of BCF
Expected Normal
Observed Value
2
1
0
-1
-2
-3
20
25
30
35
40
45

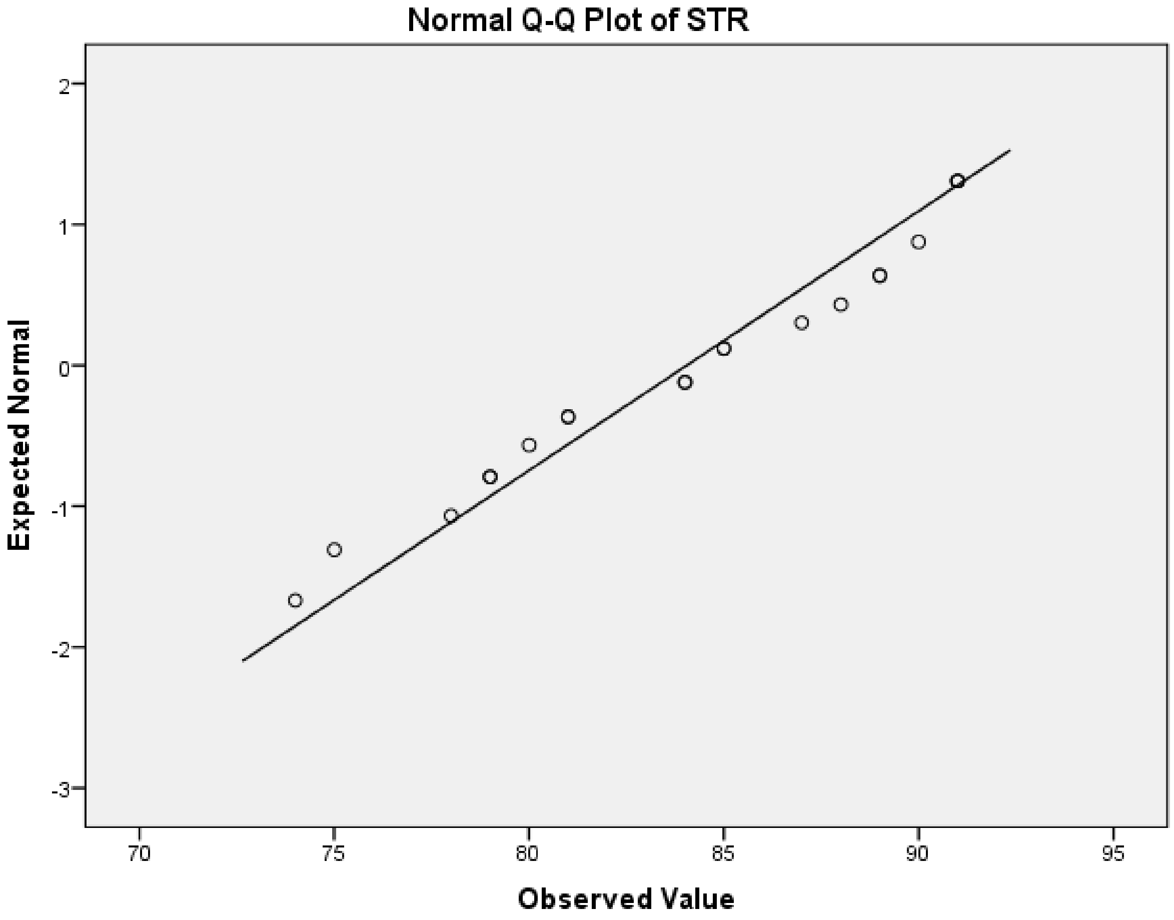

Normal Q-Q Plot of STR
Expected Normal
Observed Value
2
1
0
-1
-2
-3
70
75
80
85
90
95

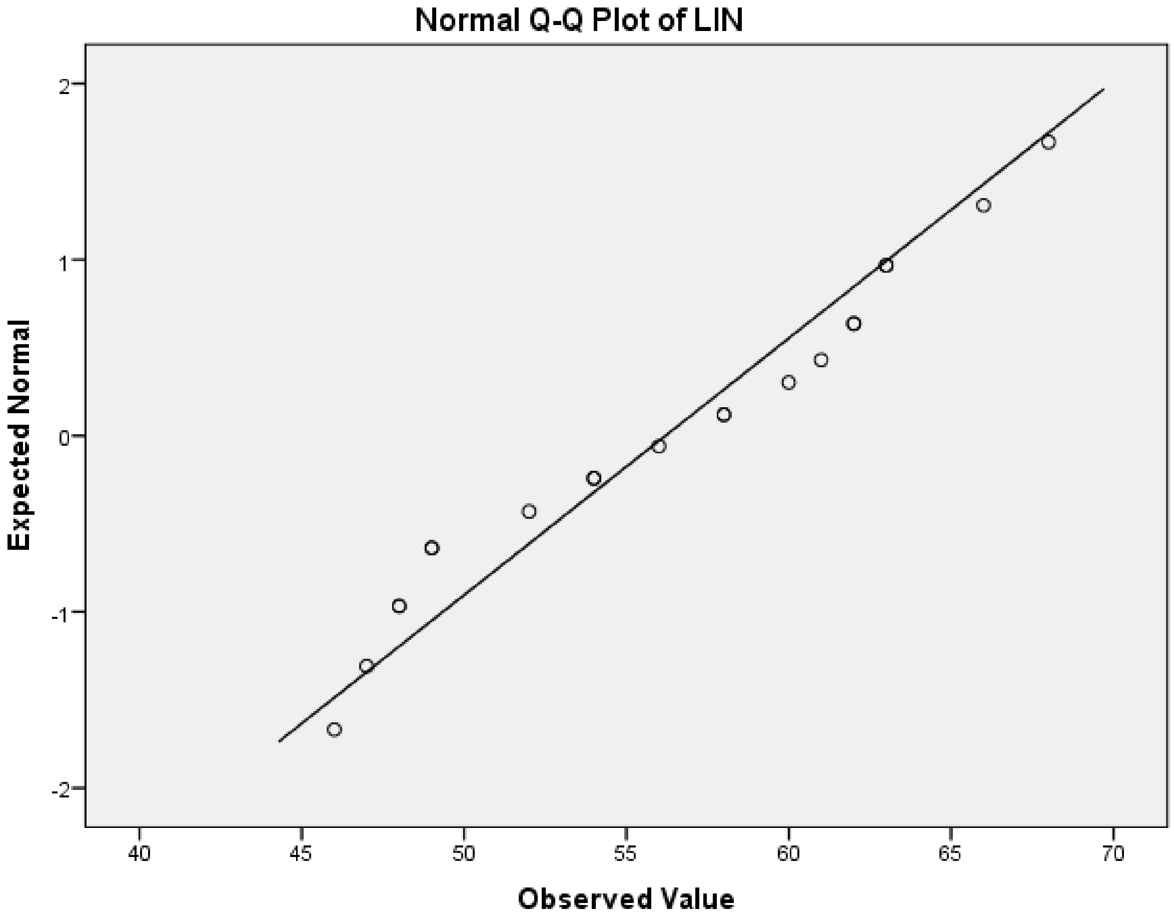

Detrended Normal Q-Q Plots

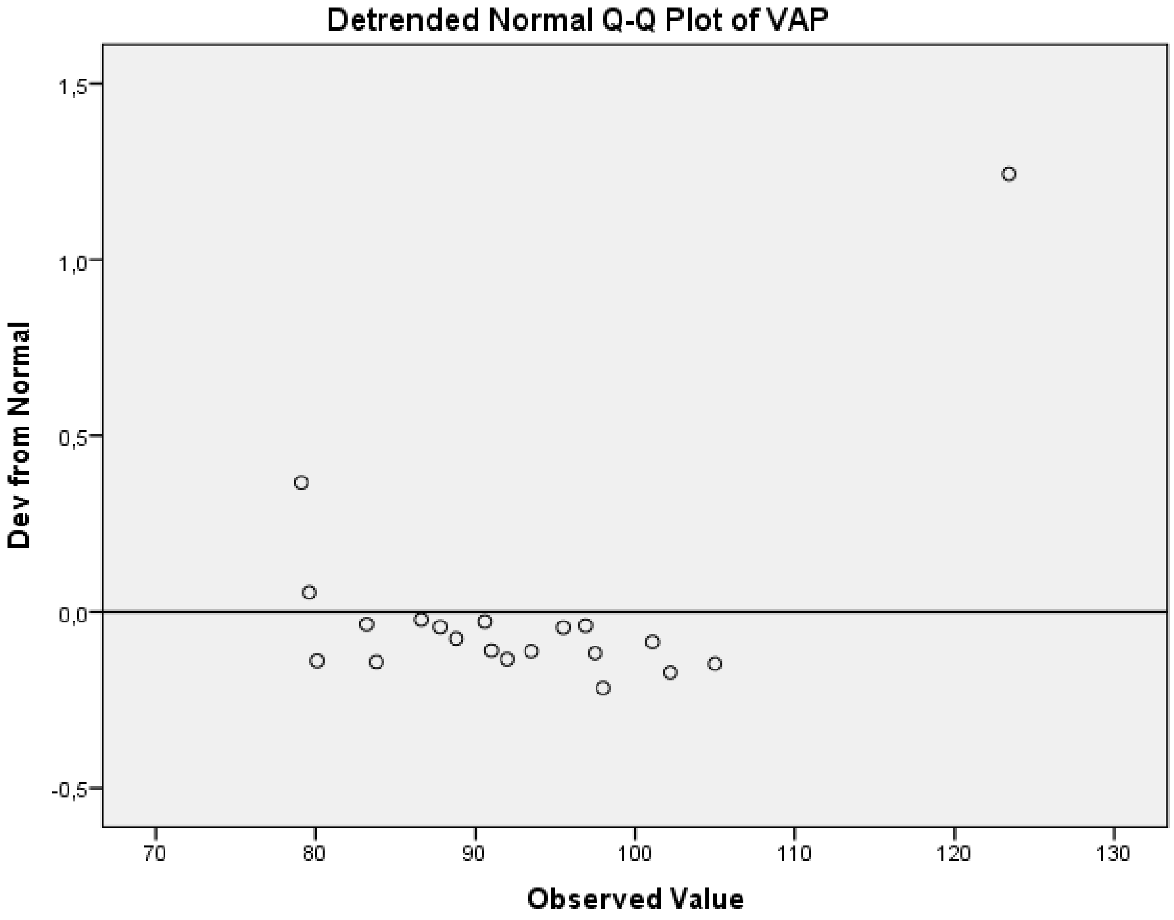
Detrended Normal Q-Q Plot of VAP
Dev from Normal
1,5
1,0
0,5
0,0
-0,5
70
80
90
100
110
120
130
Observed Value

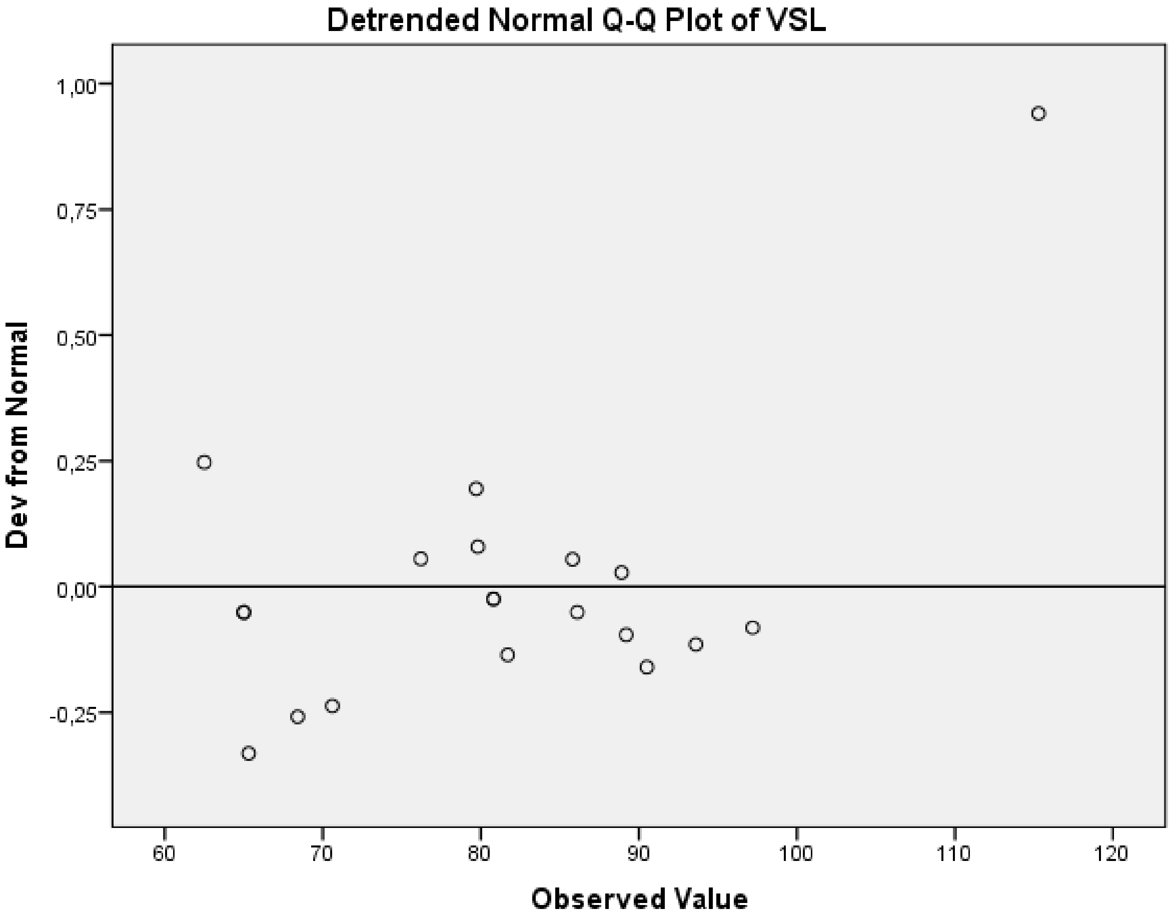
Detrended Normal Q-Q Plot of VSL
Dev from Normal
1,00
0,75
0,50
0,25
0,00
-0,25
60
70
80
90
100
110
120
Observed Value

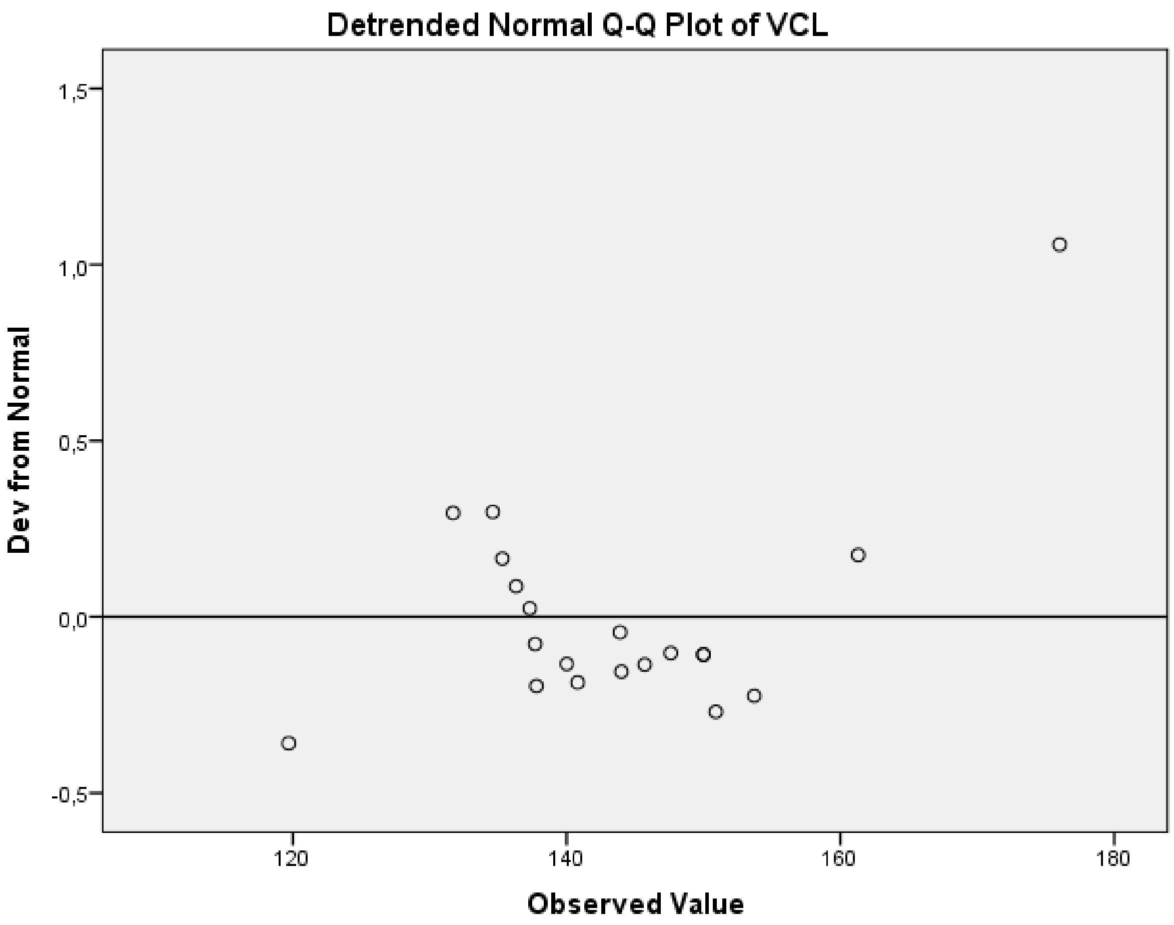

Detrended Normal Q-Q Plot of VCL
Dev from Normal
1,5
1,0
0,5
0,0
-0,5
120
140
160
180
Observed Value

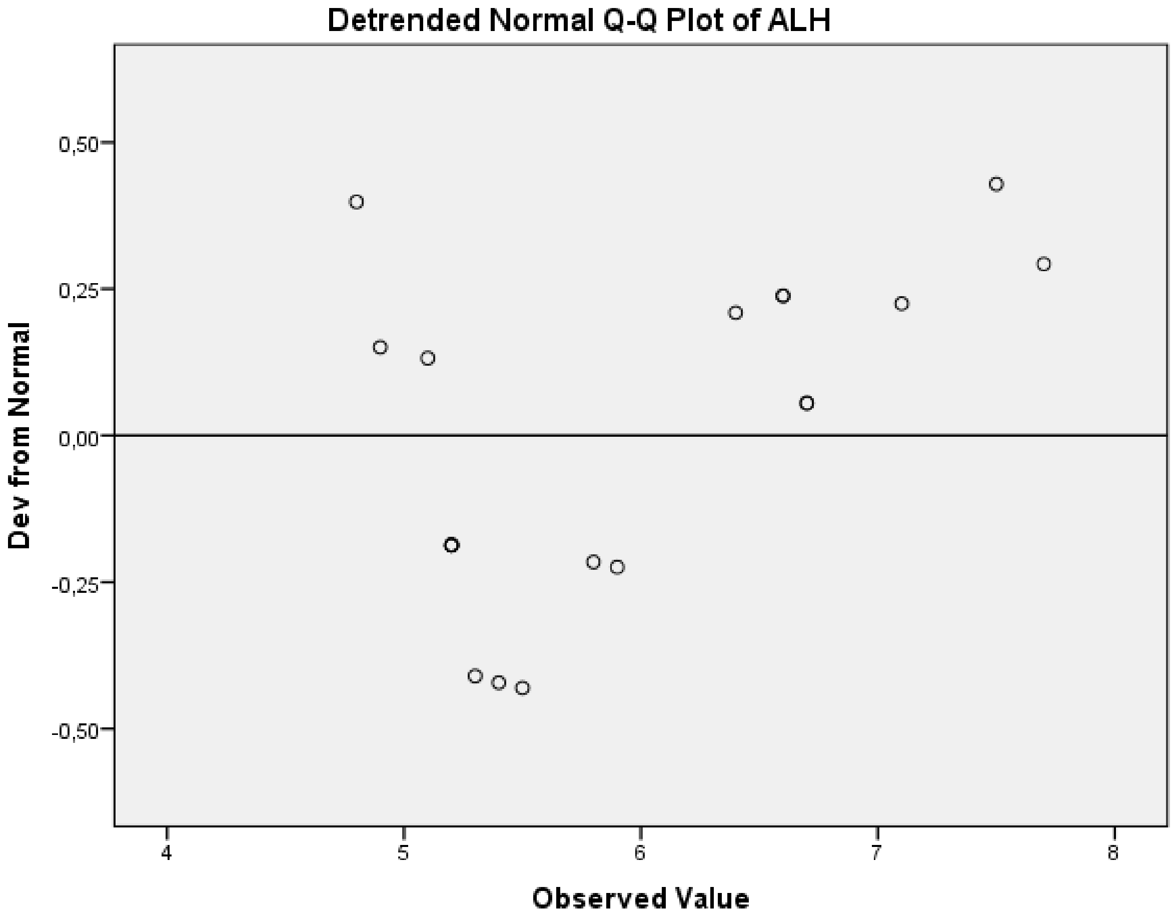

Detrended Normal Q-Q Plot of ALH
Dev from Normal
0,50
0,25
0,00
-0,25
-0,50
4
5
6
7
8
Observed Value

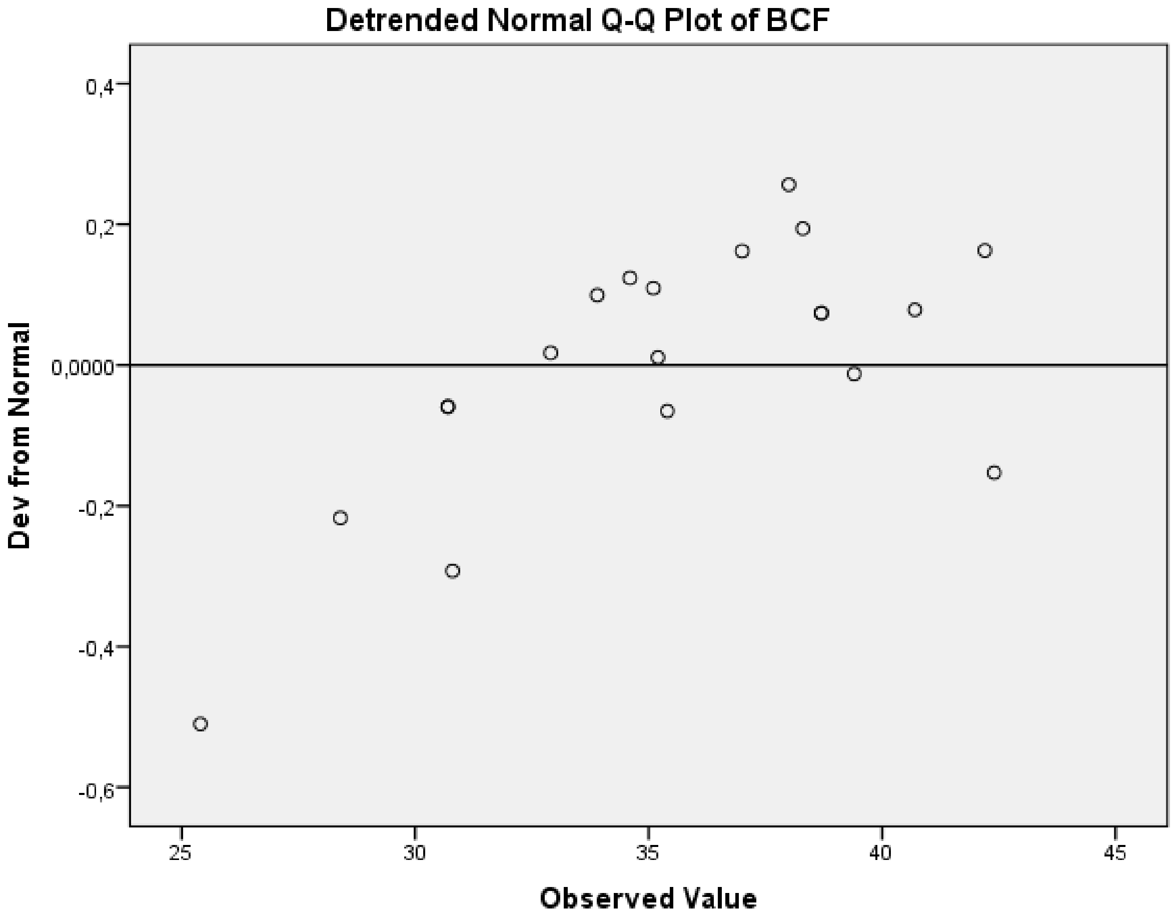

Detrended Normal Q-Q Plot of BCF
0,4
0,2
0,0000
-0,2
-0,4
-0,6
Dev from Normal
25
30
35
40
45
Observed Value

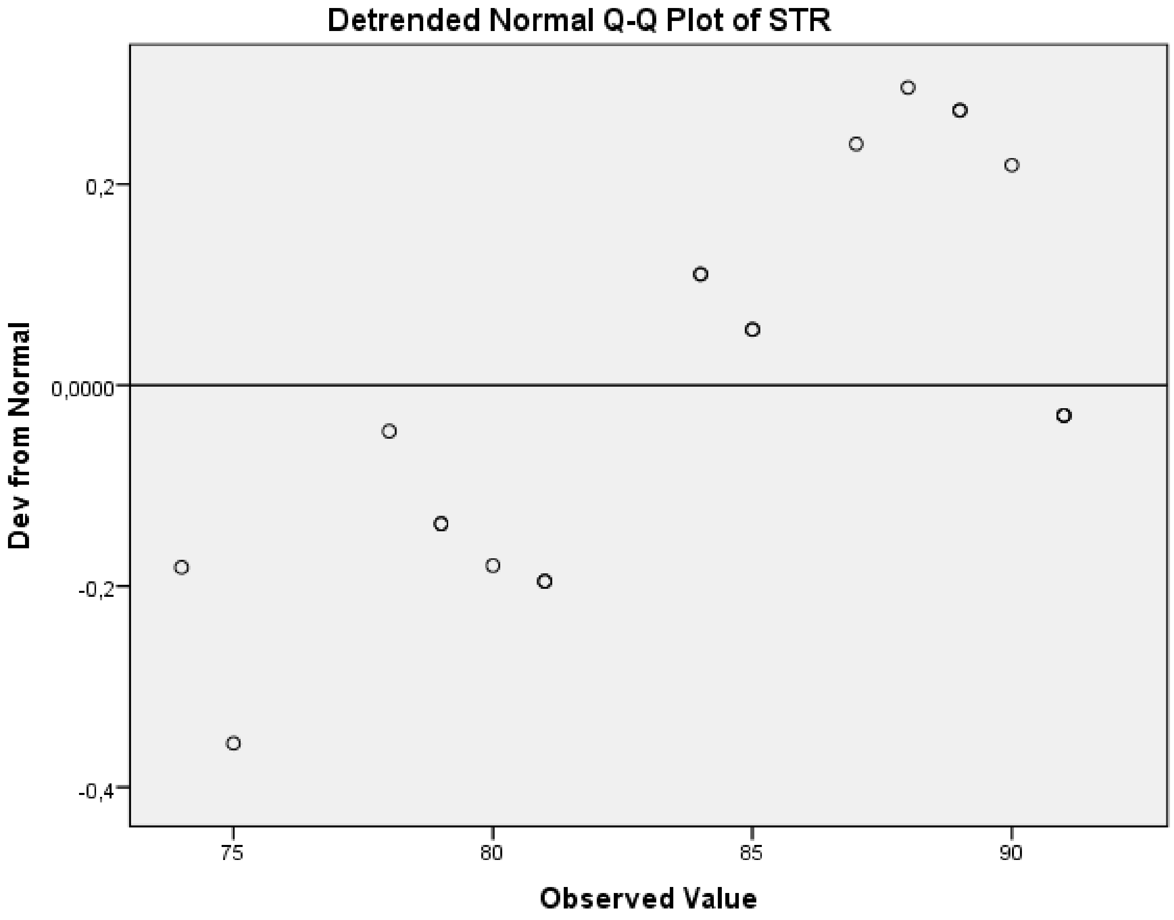
Detrended Normal Q-Q Plot of STR
Dev from Normal
0,2
0,0000
-0,2
-0,4
75
80
85
90
Observed Value

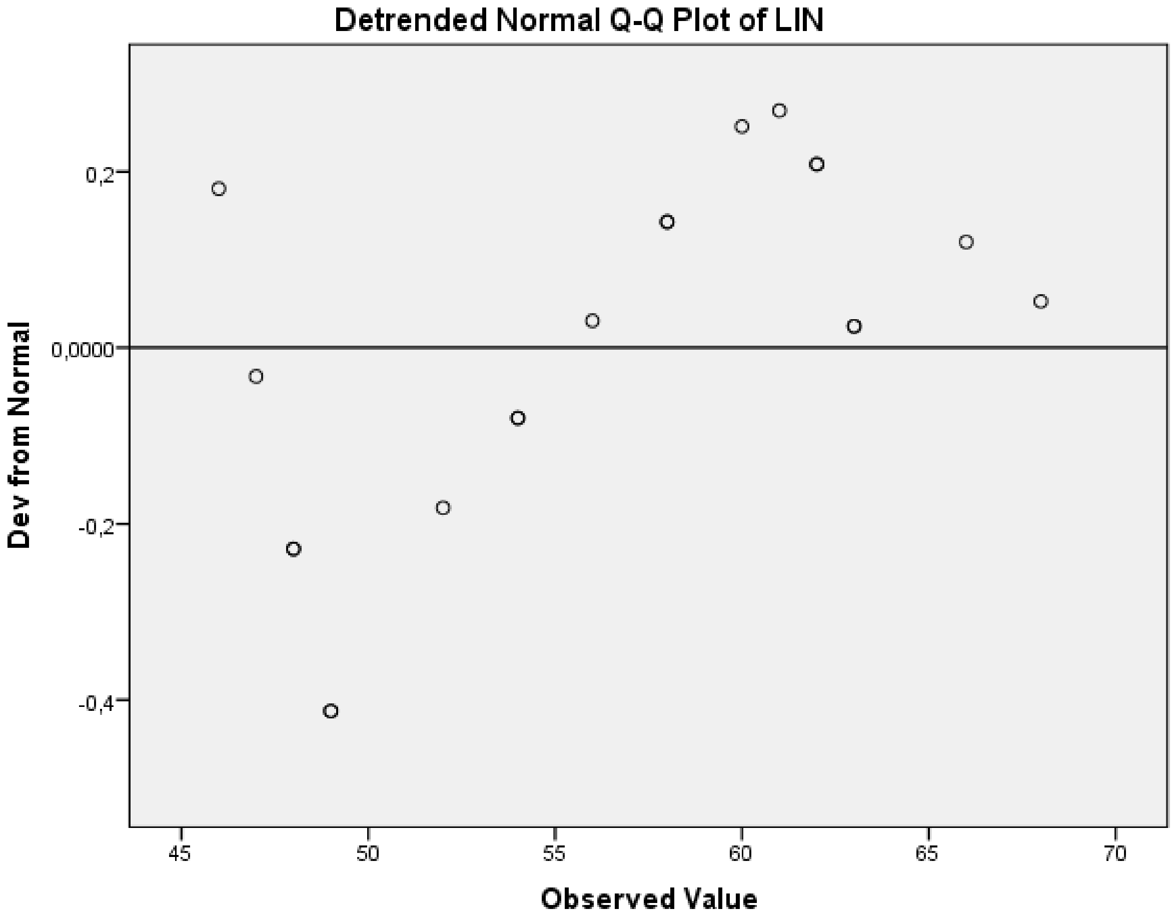
Detrended Normal Q-Q Plot of LIN
Dev from Normal
0,2
0,0000
-0,2
-0,4
45
50
55
60
65
70
Observed Value

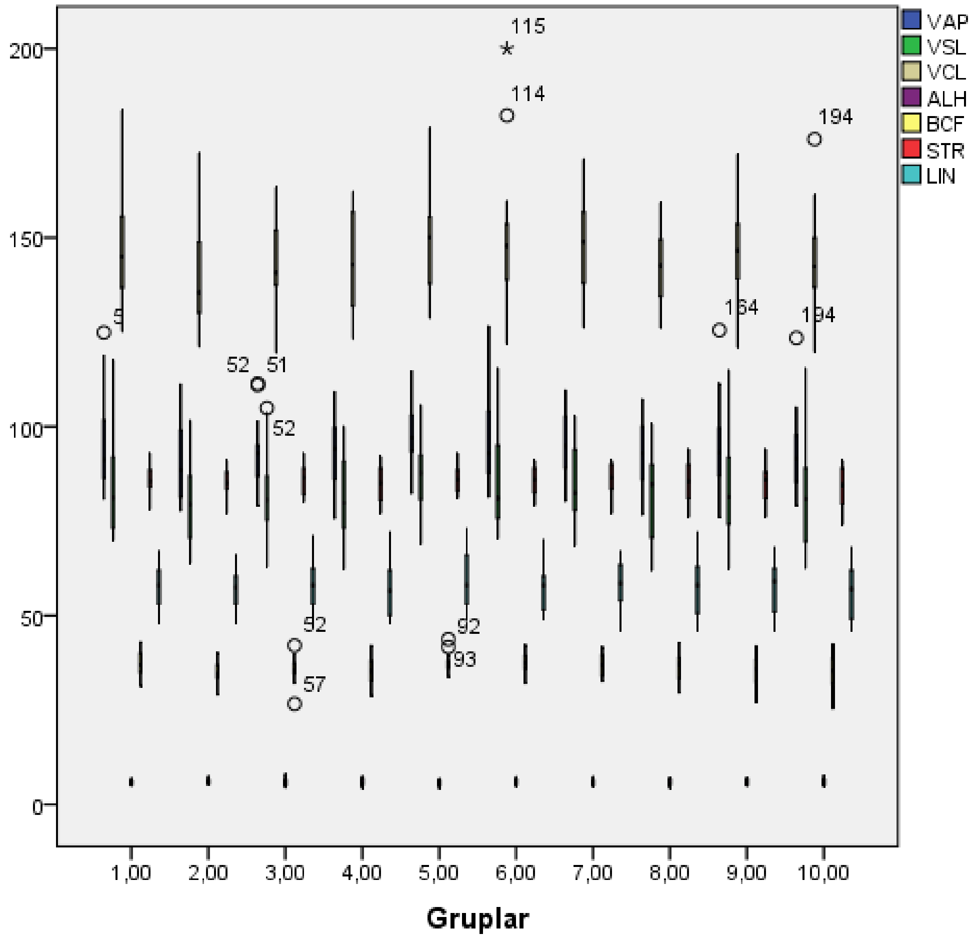


```
ONEWAY VAP VSL VCL ALH BCF STR LIN BY Gruplar
  /STATISTICS DESCRIPTIVES HOMOGENEITY
  /PLOT MEANS
  /MISSING ANALYSIS
  /POSTHOC=DUNCAN ALPHA(0.05).
```

Oneway

Notes

Output Created		23-DEC-2020 21:44:28
Comments		
Input	Active Dataset	DataSet0
	Filter	<none>
	Weight	<none>

	Split File	<none>
	N of Rows in Working Data File	200
Missing Value Handling	Definition of Missing	User-defined missing values are treated as missing.
	Cases Used	Statistics for each analysis are based on cases with no missing data for any variable in the analysis.
Syntax		ONEWAY VAP VSL VCL ALH BCF STR LIN BY Gruplar<br>/STATISTICS DESCRIPTIVES HOMOGENEITY<br>/PLOT MEANS<br>/MISSING ANALYSIS<br>/POSTHOC=DUNCAN ALPHA(0.05).
Resources	Processor Time	00:00:01,09
	Elapsed Time	00:00:01,22

Descriptives

						95% Confidence Interval for Mean			
		N	Mean	Std. Deviation	Std. Error	Lower Bound	Upper Bound	Minimum	Maximum
VAP	1,00	20	95,3500	12,74555	2,84999	89,3849	101,3151	80,90	124,90
	2,00	20	90,5250	10,18745	2,27798	85,7571	95,2929	77,80	111,10
	3,00	20	92,7400	8,59494	1,92189	88,7174	96,7626	79,10	111,40
	4,00	20	93,1000	9,68564	2,16577	88,5670	97,6330	75,70	109,10
	5,00	20	98,1550	7,73315	1,72918	94,5358	101,7742	82,30	114,60
	6,00	20	95,4350	11,40260	2,54970	90,0984	100,7716	81,40	126,50
	7,00	20	95,5050	8,68607	1,94227	91,4398	99,5702	80,30	109,60
	8,00	20	92,3750	8,92099	1,99479	88,1998	96,5502	76,70	107,20
	9,00	20	94,6100	11,59251	2,59216	89,1845	100,0355	76,00	125,50
	10,00	20	92,7850	10,51492	2,35121	87,8639	97,7061	79,10	123,40
	Total	200	94,0580	10,09937	,71413	92,6498	95,4662	75,70	126,50
VSL	1,00	20	84,8450	14,05865	3,14361	78,2653	91,4247	69,90	117,60
	2,00	20	79,7850	10,47098	2,34138	74,8844	84,6856	63,90	101,50
	3,00	20	82,1450	10,31521	2,30655	77,3173	86,9727	62,90	104,90
	4,00	20	82,0850	11,25598	2,51691	76,8170	87,3530	62,20	100,00
	5,00	20	87,6150	8,95517	2,00244	83,4239	91,8061	68,80	105,60

	6,00	20	85,0250	12,24005	2,73696	79,2965	90,7535	70,30	115,30
	7,00	20	85,2150	10,23154	2,28784	80,4265	90,0035	68,50	102,70
	8,00	20	81,4550	11,46493	2,56364	76,0892	86,8208	61,90	100,70
	9,00	20	83,5250	13,36858	2,98930	77,2683	89,7817	62,30	114,80
	10,00	20	81,1200	13,10033	2,92932	74,9889	87,2511	62,50	115,30
	Total	200	83,2815	11,60507	,82060	81,6633	84,8997	61,90	117,60
VCL	1,00	20	146,8450	15,20455	3,39984	139,7291	153,9609	125,20	183,70
	2,00	20	140,7750	15,12533	3,38213	133,6961	147,8539	121,20	172,40
	3,00	20	142,2750	11,91023	2,66321	136,7008	147,8492	119,60	163,30
	4,00	20	143,6550	12,99322	2,90537	137,5740	149,7360	123,20	162,10
	5,00	20	148,1050	12,71007	2,84206	142,1565	154,0535	128,70	179,10
	6,00	20	149,0550	17,45163	3,90230	140,8874	157,2226	121,80	200,00
	7,00	20	147,1400	12,41160	2,77532	141,3312	152,9488	126,20	170,70
	8,00	20	142,2500	10,02772	2,24227	137,5569	146,9431	126,10	159,30
	9,00	20	146,1800	13,48819	3,01605	139,8673	152,4927	120,90	172,10
	10,00	20	143,7150	11,84462	2,64854	138,1715	149,2585	119,70	176,00
	Total	200	144,9995	13,43006	,94965	143,1268	146,8722	119,60	200,00
ALH	1,00	20	5,7950	,62195	,13907	5,5039	6,0861	4,90	7,00
	2,00	20	5,9900	,71884	,16074	5,6536	6,3264	5,20	7,50
	3,00	20	5,8100	,93127	,20824	5,3742	6,2458	4,50	8,10
	4,00	20	5,9000	,95366	,21325	5,4537	6,3463	4,30	7,50
	5,00	20	5,7050	,82556	,18460	5,3186	6,0914	4,20	6,90
	6,00	20	5,9000	,71892	,16075	5,5635	6,2365	4,70	7,30
	7,00	20	5,7550	,83128	,18588	5,3659	6,1441	4,70	7,40
	8,00	20	5,8500	,88228	,19728	5,4371	6,2629	4,30	7,20
	9,00	20	5,9600	,79366	,17747	5,5886	6,3314	4,90	7,10
	10,00	20	5,9400	,89760	,20071	5,5199	6,3601	4,80	7,70
	Total	200	5,8605	,80967	,05725	5,7476	5,9734	4,20	8,10
BCF	1,00	20	37,1550	3,19580	,71460	35,6593	38,6507	31,30	43,00
	2,00	20	34,9750	2,88807	,64579	33,6233	36,3267	29,00	40,20

	3,00	20	35,6900	3,23564	,72351	34,1757	37,2043	26,60	42,10
	4,00	20	35,3600	3,39867	,75997	33,7694	36,9506	28,70	42,00
	5,00	20	37,2500	2,35607	,52683	36,1473	38,3527	33,80	43,70
	6,00	20	37,3950	2,69726	,60313	36,1326	38,6574	32,10	42,20
	7,00	20	36,8950	3,13696	,70144	35,4269	38,3631	32,60	41,70
	8,00	20	36,2750	3,99669	,89369	34,4045	38,1455	29,50	42,80
	9,00	20	35,4250	3,76478	,84183	33,6630	37,1870	27,10	41,90
	10,00	20	35,4250	4,60227	1,02910	33,2711	37,5789	25,40	42,40
	Total	200	36,1845	3,42085	,24189	35,7075	36,6615	25,40	43,70
STR	1,00	20	85,8500	3,80132	,85000	84,0709	87,6291	78,00	93,00
	2,00	20	84,9500	3,53144	,78965	83,2972	86,6028	77,00	91,00
	3,00	20	85,9000	4,21651	,94284	83,9266	87,8734	80,00	93,00
	4,00	20	85,1000	4,76721	1,06598	82,8689	87,3311	77,00	92,00
	5,00	20	85,9500	3,92663	,87802	84,1123	87,7877	81,00	93,00
	6,00	20	85,6000	4,00526	,89560	83,7255	87,4745	79,00	91,00
	7,00	20	86,1000	4,12821	,92310	84,1679	88,0321	77,00	91,00
	8,00	20	85,2500	5,04584	1,12828	82,8885	87,6115	76,00	94,00
	9,00	20	85,2500	4,87610	1,09033	82,9679	87,5321	76,00	94,00
	10,00	20	84,0500	5,43357	1,21498	81,5070	86,5930	74,00	91,00
	Total	200	85,4000	4,35140	,30769	84,7932	86,0068	74,00	94,00
LIN	1,00	20	57,7500	5,18982	1,16048	55,3211	60,1789	48,00	67,00
	2,00	20	56,7000	4,85690	1,08604	54,4269	58,9731	48,00	66,00
	3,00	20	58,0500	6,57327	1,46983	54,9736	61,1264	47,00	71,00
	4,00	20	57,1500	7,11762	1,59155	53,8189	60,4811	48,00	72,00
	5,00	20	59,2500	7,26111	1,62363	55,8517	62,6483	49,00	73,00
	6,00	20	56,8000	5,53078	1,23672	54,2115	59,3885	49,00	70,00
	7,00	20	58,0000	6,13017	1,37075	55,1310	60,8690	46,00	67,00
	8,00	20	57,4000	7,49315	1,67552	53,8931	60,9069	46,00	72,00
	9,00	20	57,3000	6,97439	1,55952	54,0359	60,5641	46,00	68,00
	10,00	20	56,2000	6,85642	1,53314	52,9911	59,4089	46,00	68,00
	Total	200	57,4600	6,36324	,44995	56,5727	58,3473	46,00	73,00

Test of Homogeneity of Variances

	Levene Statistic	df1	df2	Sig.
VAP	,958	9	190	,477
VSL	,611	9	190	,787
VCL	,530	9	190	,851
ALH	1,222	9	190	,284

BCF	2,063	9	190	,035
STR	1,348	9	190	,215
LIN	1,311	9	190	,233

ANOVA

		Sum of Squares	df	Mean Square	F	Sig.
VAP	Between Groups	846,786	9	94,087	,919	,510
	Within Groups	19450,681	190	102,372		
	Total	20297,467	199			
VSL	Between Groups	1020,364	9	113,374	,836	,584
	Within Groups	25780,497	190	135,687		
	Total	26800,862	199			
VCL	Between Groups	1435,181	9	159,465	,879	,545
	Within Groups	34457,749	190	181,357		
	Total	35892,930	199			
ALH	Between Groups	1,567	9	,174	,257	,985
	Within Groups	128,890	190	,678		
	Total	130,458	199			
BCF	Between Groups	151,927	9	16,881	1,473	,160
	Within Groups	2176,815	190	11,457		
	Total	2328,742	199			
STR	Between Groups	68,900	9	7,656	,393	,937
	Within Groups	3699,100	190	19,469		
	Total	3768,000	199			
LIN	Between Groups	133,080	9	14,787	,355	,955
	Within Groups	7924,600	190	41,708		
	Total	8057,680	199			

Post Hoc Tests

Homogeneous Subsets

VAP

Duncan[a]

Gruplar	N	Subset for alpha = 0.05	
		1	2
2,00	20	90,5250	
8,00	20	92,3750	92,3750
3,00	20	92,7400	92,7400
10,00	20	92,7850	92,7850
4,00	20	93,1000	93,1000
9,00	20	94,6100	94,6100
1,00	20	95,3500	95,3500
6,00	20	95,4350	95,4350
7,00	20	95,5050	95,5050
5,00	20		98,1550
Sig.		,197	,132

Means for groups in homogeneous subsets are displayed.
a. Uses Harmonic Mean Sample Size = 20,000.

VSL

Duncan[a]

Gruplar	N	Subset for alpha = 0.05
		1
2,00	20	79,7850
10,00	20	81,1200
8,00	20	81,4550
4,00	20	82,0850
3,00	20	82,1450
9,00	20	83,5250
1,00	20	84,8450
6,00	20	85,0250
7,00	20	85,2150
5,00	20	87,6150
Sig.		,077

Means for groups in homogeneous subsets are displayed.

a. Uses Harmonic Mean Sample Size = 20,000.

VCL

Duncan[a]

Gruplar	N	Subset for alpha = 0.05
		1
2,00	20	140,7750
8,00	20	142,2500
3,00	20	142,2750
4,00	20	143,6550
10,00	20	143,7150
9,00	20	146,1800
1,00	20	146,8450
7,00	20	147,1400
5,00	20	148,1050
6,00	20	149,0550
Sig.		,107

Means for groups in homogeneous subsets are displayed.
a. Uses Harmonic Mean Sample Size = 20,000.

ALH

Duncan[a]

Gruplar	N	Subset for alpha = 0.05
		1
5,00	20	5,7050
7,00	20	5,7550
1,00	20	5,7950
3,00	20	5,8100
8,00	20	5,8500
4,00	20	5,9000
6,00	20	5,9000
10,00	20	5,9400

9,00	20	5,9600
2,00	20	5,9900
Sig.		,372

Means for groups in homogeneous subsets are displayed.
a. Uses Harmonic Mean Sample Size = 20,000.

BCF

Duncan[a]

Gruplar	N	Subset for alpha = 0.05
		1
2,00	20	34,9750
4,00	20	35,3600
9,00	20	35,4250
10,00	20	35,4250
3,00	20	35,6900
8,00	20	36,2750
7,00	20	36,8950
1,00	20	37,1550
5,00	20	37,2500
6,00	20	37,3950
Sig.		,059

Means for groups in homogeneous subsets are displayed.
a. Uses Harmonic Mean Sample Size = 20,000.

STR

Duncan[a]

Gruplar	N	Subset for alpha = 0.05
		1
10,00	20	84,0500
2,00	20	84,9500
4,00	20	85,1000

8,00	20	85,2500
9,00	20	85,2500
6,00	20	85,6000
1,00	20	85,8500
3,00	20	85,9000
5,00	20	85,9500
7,00	20	86,1000
Sig.		,228

Means for groups in homogeneous subsets are displayed.
a. Uses Harmonic Mean Sample Size = 20,000.

LIN

Duncan[a]

Gruplar	N	Subset for alpha = 0.05
		1
10,00	20	56,2000
2,00	20	56,7000
6,00	20	56,8000
4,00	20	57,1500
9,00	20	57,3000
8,00	20	57,4000
1,00	20	57,7500
7,00	20	58,0000
3,00	20	58,0500
5,00	20	59,2500
Sig.		,220

Means for groups in homogeneous subsets are displayed.
a. Uses Harmonic Mean Sample Size = 20,000.

Means Plots

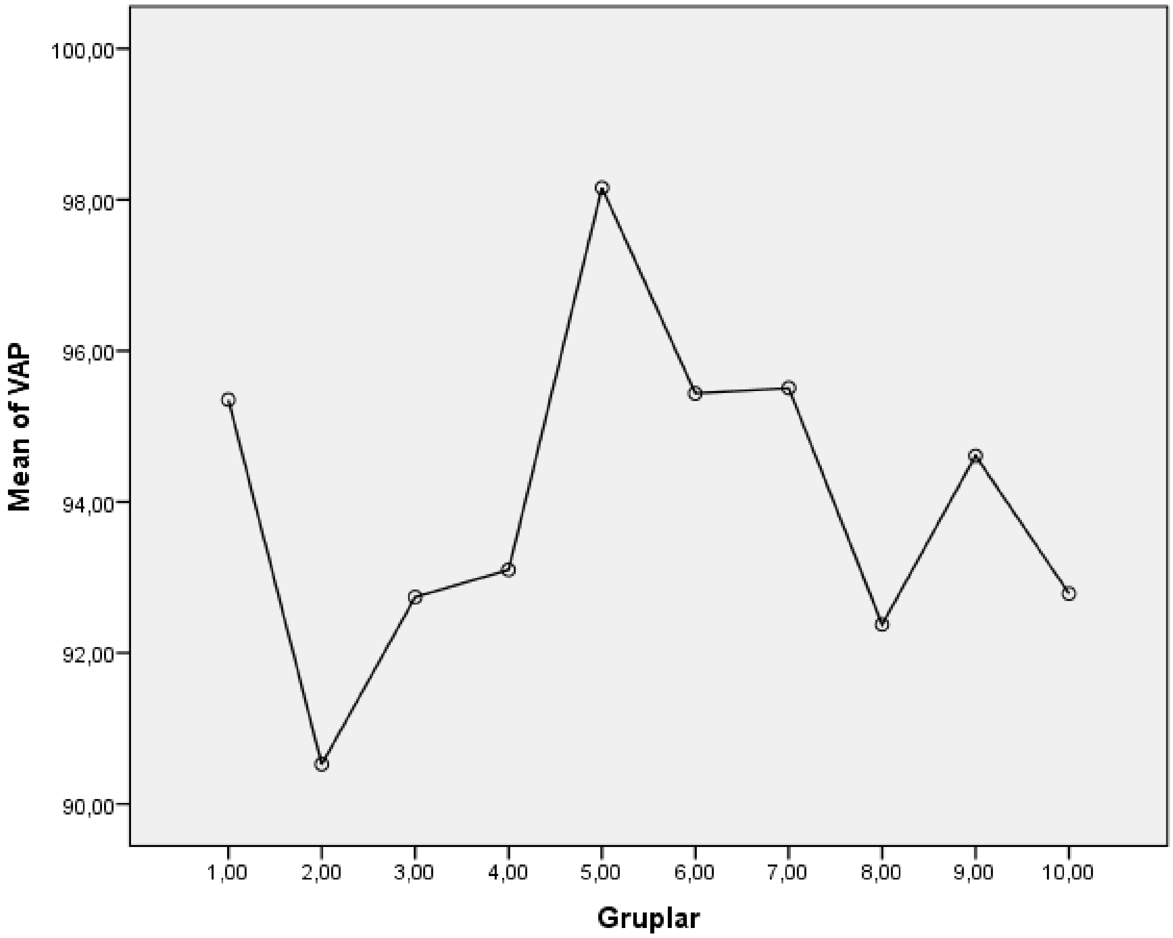
100,00
98,00
96,00
94,00
92,00
90,00
Mean of VAP
1,00
2,00
3,00
4,00
5,00
6,00
7,00
8,00
9,00
10,00
Gruplar

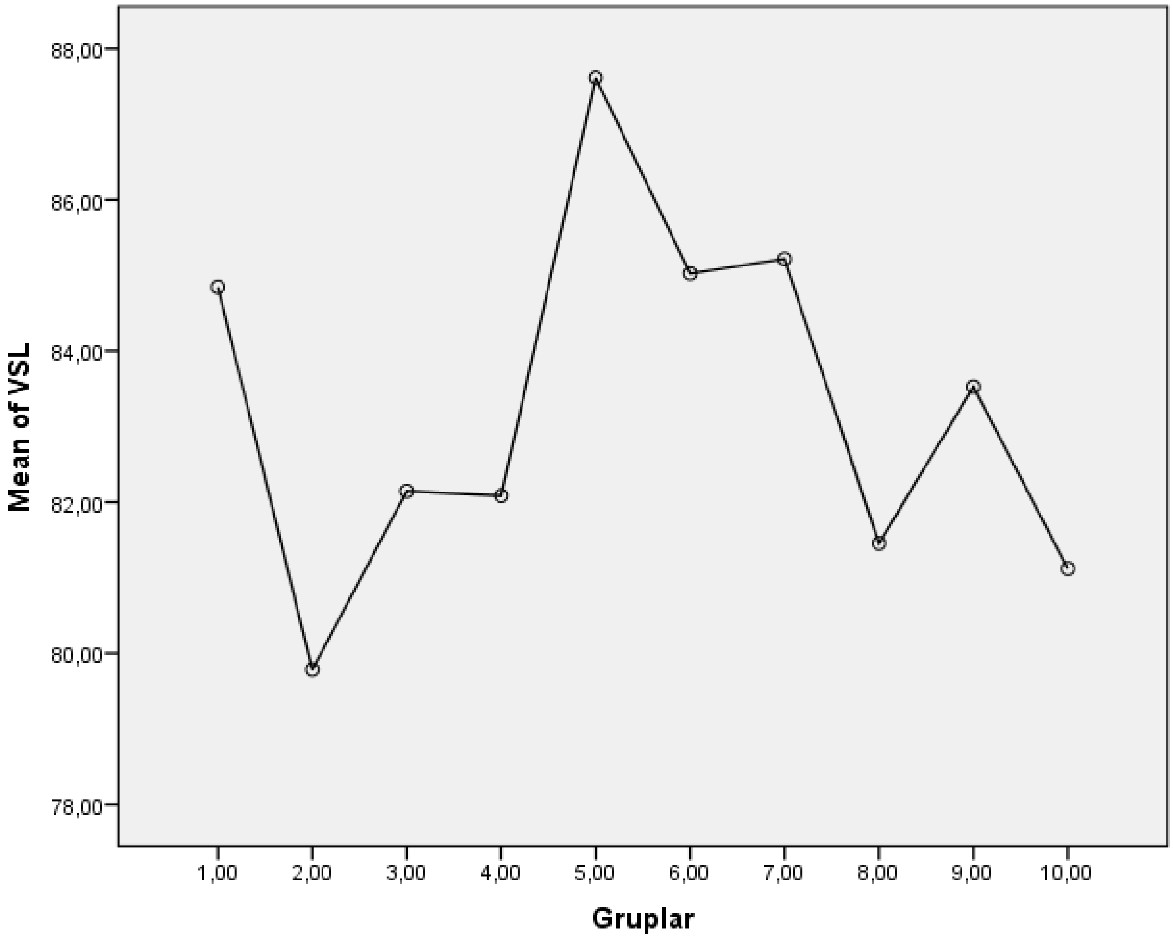

88,00
86,00
84,00
82,00
80,00
78,00
Mean of VSL
1,00
2,00
3,00
4,00
5,00
6,00
7,00
8,00
9,00
10,00
Gruplar

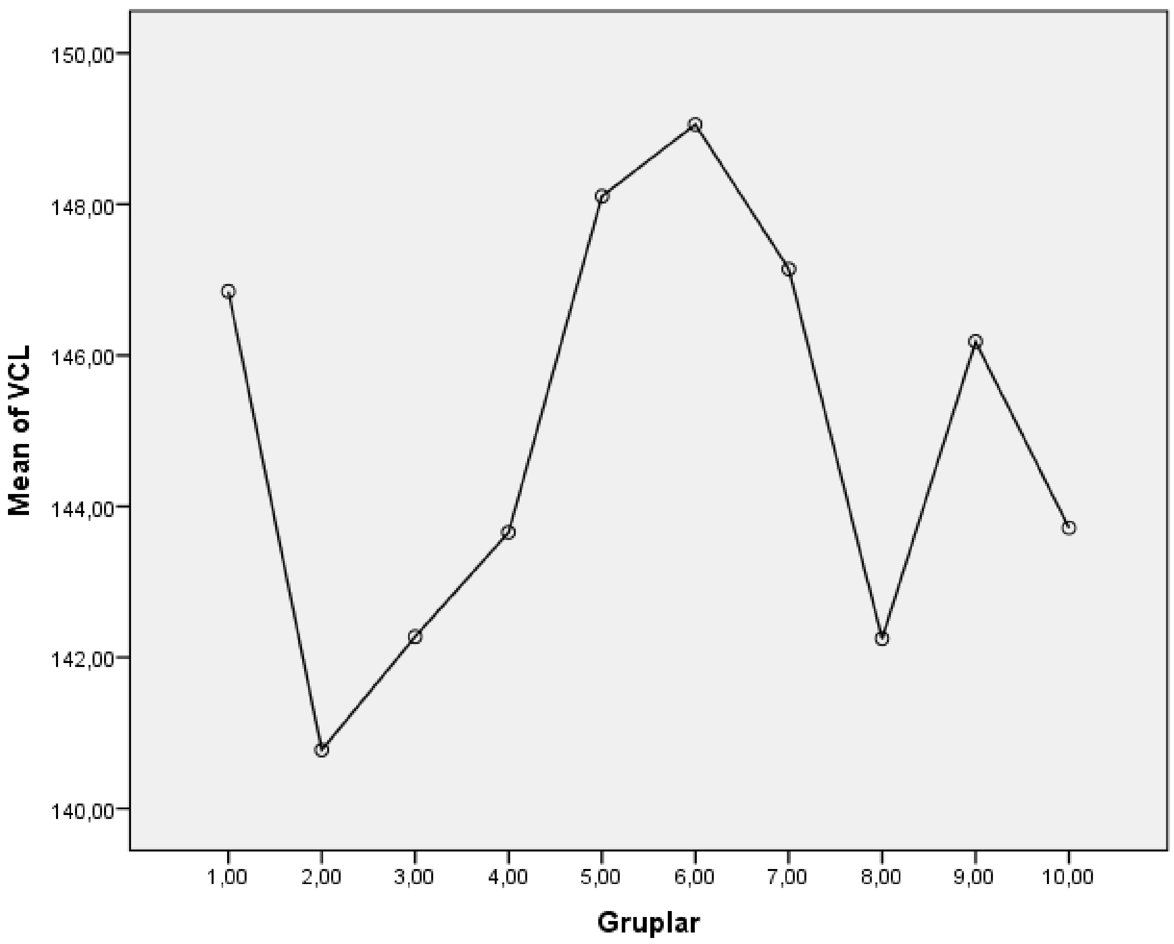

150,00
148,00
146,00
144,00
142,00
140,00
Mean of VCL
1,00
2,00
3,00
4,00
5,00
6,00
7,00
8,00
9,00
10,00
Gruplar

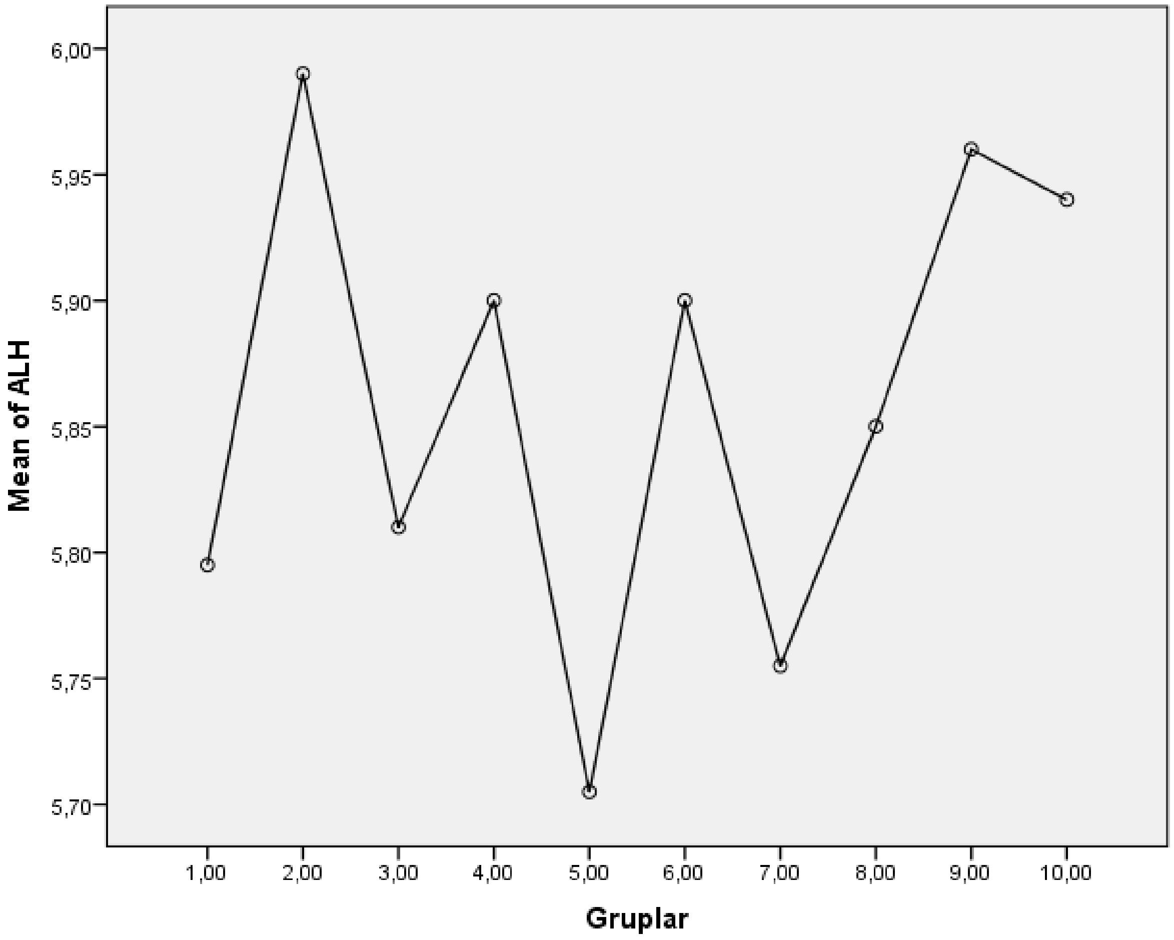

Mean of ALH
6,00
5,95
5,90
5,85
5,80
5,75
5,70
1,00
2,00
3,00
4,00
5,00
6,00
7,00
8,00
9,00
10,00
Gruplar

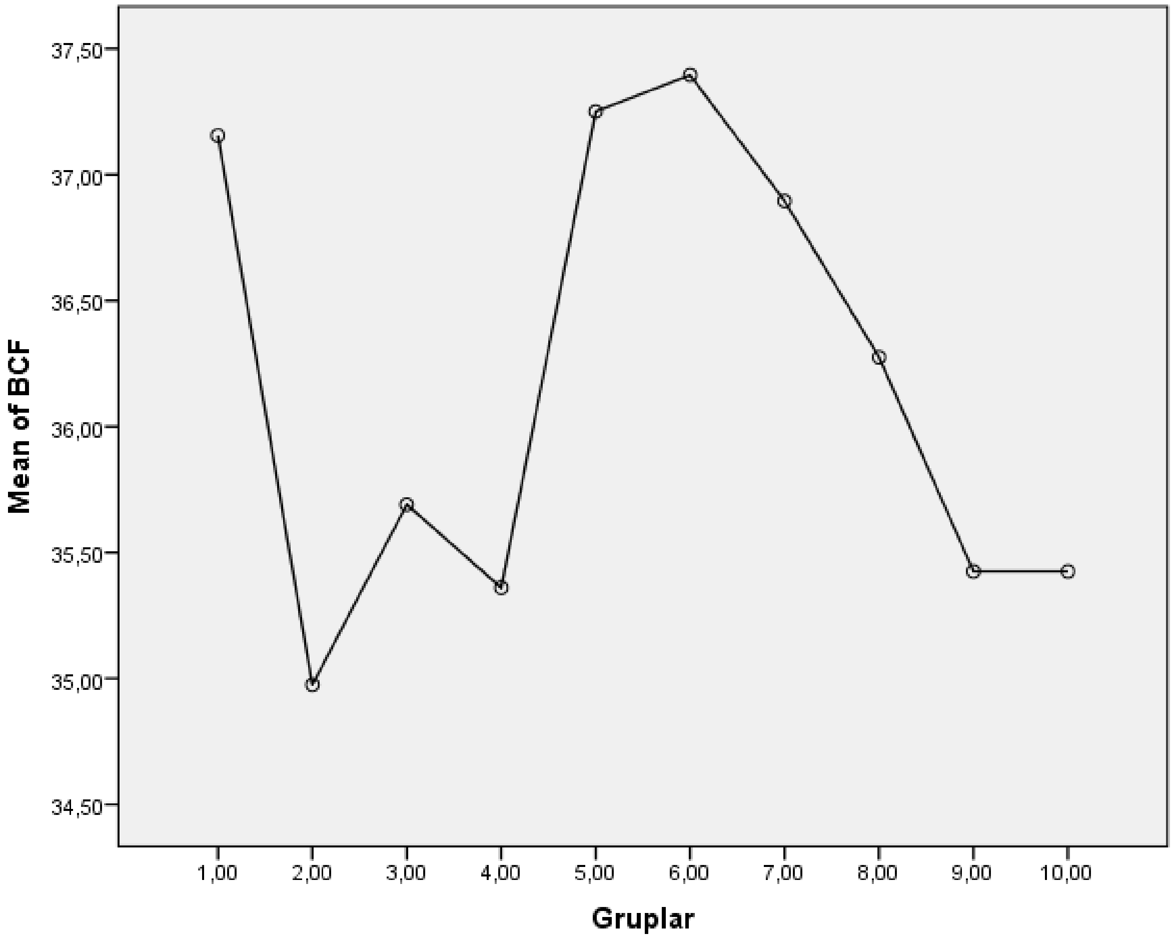

Mean of BCF
37,50
37,00
36,50
36,00
35,50
35,00
34,50
1,00
2,00
3,00
4,00
5,00
6,00
7,00
8,00
9,00
10,00
Gruplar

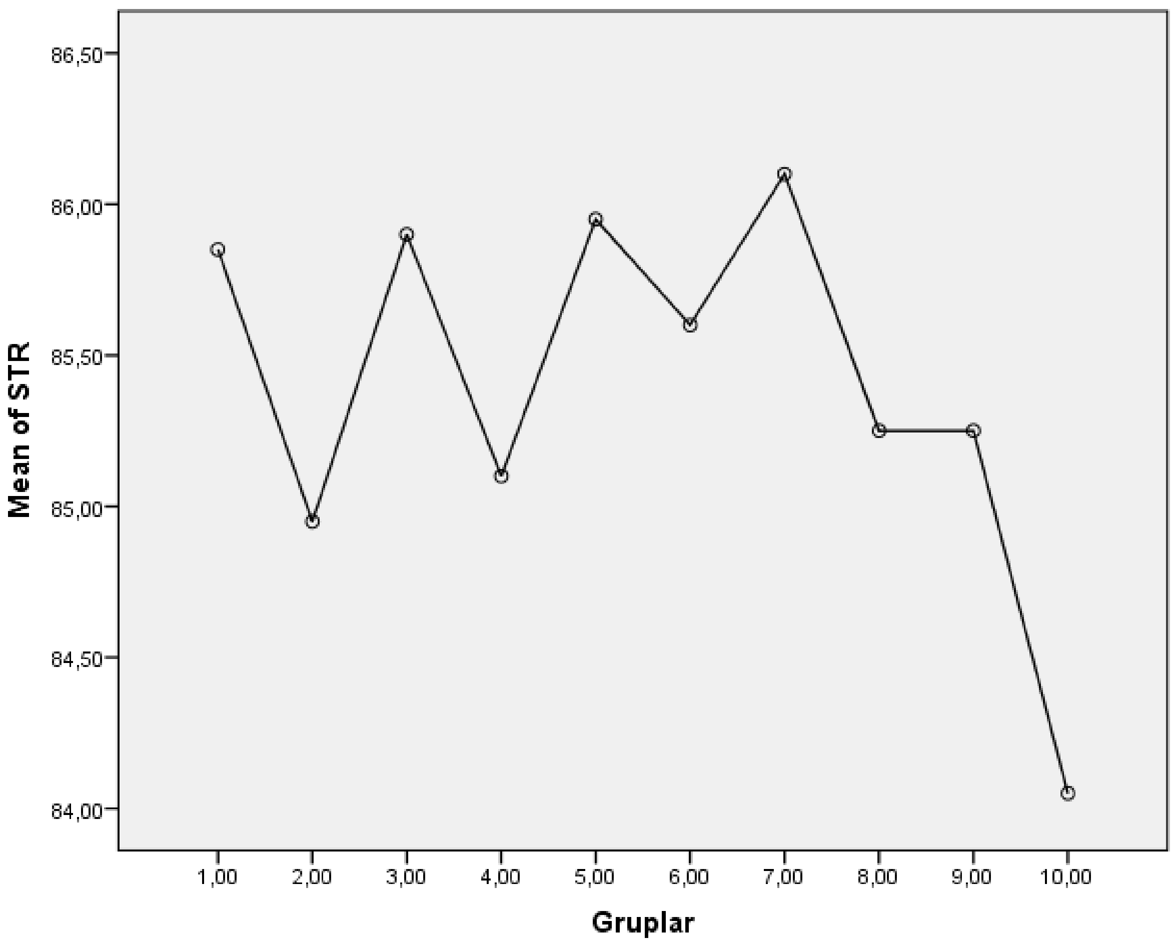

86,50
86,00
85,50
85,00
84,50
84,00
Mean of STR
1,00
2,00
3,00
4,00
5,00
6,00
7,00
8,00
9,00
10,00
Gruplar

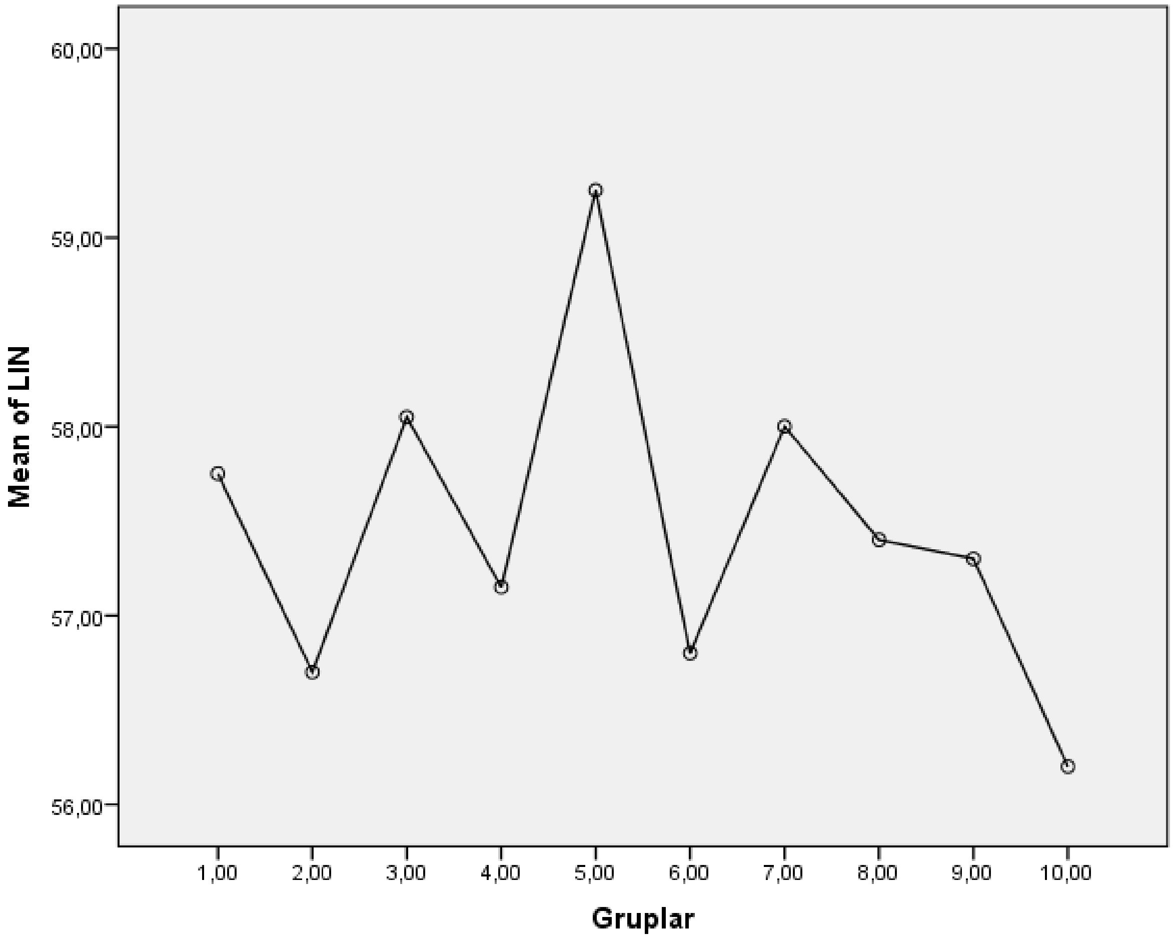

60,00
59,00
58,00
57,00
56,00
Mean of LIN
1,00
2,00
3,00
4,00
5,00
6,00
7,00
8,00
9,00
10,00
Gruplar

F pattern rates- ANOVA

Groups

1: Control

2: RES10

3: RES20

4: RES40

5: CD10

6: CD20

7: CD40

8: RLC10

9: RLC20

10: RLC40

Explore

Notes

Output Created		24-DEC-2020 13:27:16
Comments		
Input	Active Dataset	DataSet0
	Filter	<none>
	Weight	<none>
	Split File	<none>
	N of Rows in Working Data File	100
Missing Value Handling	Definition of Missing	User-defined missing values for dependent variables are treated as missing.
	Cases Used	Statistics are based on cases with no missing values for any dependent variable or factor used.
Syntax		EXAMINE VARIABLES=F BY Gruplar /PLOT BOXPLOT STEMLEAF HISTOGRAM NPPLOT /COMPARE GROUPS /STATISTICS DESCRIPTIVES /CINTERVAL 95 /MISSING LISTWISE /NOTOTAL.

Resources	Processor Time	00:00:12,44
	Elapsed Time	00:00:12,57

Gruplar

Case Processing Summary

		Cases					
		Valid		Missing		Total	
	Gruplar	N	Percent	N	Percent	N	Percent
F	1,00	10	100,0%	0	0,0%	10	100,0%
	2,00	10	100,0%	0	0,0%	10	100,0%
	3,00	10	100,0%	0	0,0%	10	100,0%
	4,00	10	100,0%	0	0,0%	10	100,0%
	5,00	10	100,0%	0	0,0%	10	100,0%
	6,00	10	100,0%	0	0,0%	10	100,0%
	7,00	10	100,0%	0	0,0%	10	100,0%
	8,00	10	100,0%	0	0,0%	10	100,0%
	9,00	10	100,0%	0	0,0%	10	100,0%
	10,00	10	100,0%	0	0,0%	10	100,0%

Descriptives

	Gruplar			Statistic	Std. Error
F	1,00	Mean		44,0000	1,94936
		95% Confidence Interval for Mean	Lower Bound	39,5902	
			Upper Bound	48,4098	
		5% Trimmed Mean		44,0000	
		Median		45,0000	
		Variance		38,000	
		Std. Deviation		6,16441	
		Minimum		36,00	
		Maximum		52,00	
		Range		16,00	
		Interquartile Range		12,75	

	Skewness		-,324	,687
	Kurtosis		-1,420	1,334
2,00	Mean		48,4000	2,64239
	95% Confidence Interval for Mean	Lower Bound	42,4225	
		Upper Bound	54,3775	
	5% Trimmed Mean		48,3889	
	Median		46,0000	
	Variance		69,822	
	Std. Deviation		8,35597	
	Minimum		35,00	
	Maximum		62,00	
	Range		27,00	
	Interquartile Range		13,75	
	Skewness		,226	,687
	Kurtosis		-,694	1,334
3,00	Mean		59,1000	2,08407
	95% Confidence Interval for Mean	Lower Bound	54,3855	
		Upper Bound	63,8145	
	5% Trimmed Mean		59,1111	
	Median		58,5000	
	Variance		43,433	
	Std. Deviation		6,59040	
	Minimum		48,00	
	Maximum		70,00	
	Range		22,00	
	Interquartile Range		7,50	
	Skewness		,345	,687
	Kurtosis		,263	1,334
4,00	Mean		46,8000	2,57682
	95% Confidence Interval for Mean	Lower Bound	40,9708	
		Upper Bound	52,6292	
	5% Trimmed Mean		47,0556	
	Median		49,0000	
	Variance		66,400	
	Std. Deviation		8,14862	
	Minimum		31,00	
	Maximum		58,00	
	Range		27,00	

	Interquartile Range		13,50	
	Skewness		-,746	,687
	Kurtosis		,071	1,334
5,00	Mean		31,0000	2,45402
	95% Confidence Interval for Mean	Lower Bound	25,4486	
		Upper Bound	36,5514	
	5% Trimmed Mean		30,7778	
	Median		30,0000	
	Variance		60,222	
	Std. Deviation		7,76030	
	Minimum		21,00	
	Maximum		45,00	
	Range		24,00	
	Interquartile Range		13,25	
	Skewness		,374	,687
	Kurtosis		-,486	1,334
6,00	Mean		29,7000	2,99648
	95% Confidence Interval for Mean	Lower Bound	22,9215	
		Upper Bound	36,4785	
	5% Trimmed Mean		29,8889	
	Median		32,5000	
	Variance		89,789	
	Std. Deviation		9,47570	
	Minimum		15,00	
	Maximum		41,00	
	Range		26,00	
	Interquartile Range		19,25	
	Skewness		-,496	,687
	Kurtosis		-1,449	1,334
7,00	Mean		25,3000	2,74084
	95% Confidence Interval for Mean	Lower Bound	19,0998	
		Upper Bound	31,5002	
	5% Trimmed Mean		24,7778	
	Median		21,5000	
	Variance		75,122	
	Std. Deviation		8,66731	
	Minimum		17,00	
	Maximum		43,00	

	Range		26,00	
	Interquartile Range		14,00	
	Skewness		1,117	,687
	Kurtosis		,293	1,334
8,00	Mean		52,4000	4,05024
	95% Confidence Interval for Mean	Lower Bound	43,2377	
		Upper Bound	61,5623	
	5% Trimmed Mean		53,0556	
	Median		55,0000	
	Variance		164,044	
	Std. Deviation		12,80798	
	Minimum		25,00	
	Maximum		68,00	
	Range		43,00	
	Interquartile Range		13,75	
	Skewness		-1,220	,687
	Kurtosis		1,305	1,334
9,00	Mean		51,2000	2,59829
	95% Confidence Interval for Mean	Lower Bound	45,3223	
		Upper Bound	57,0777	
	5% Trimmed Mean		51,0000	
	Median		51,5000	
	Variance		67,511	
	Std. Deviation		8,21651	
	Minimum		38,00	
	Maximum		68,00	
	Range		30,00	
	Interquartile Range		8,75	
	Skewness		,447	,687
	Kurtosis		1,321	1,334
10,00	Mean		46,1000	2,72621
	95% Confidence Interval for Mean	Lower Bound	39,9329	
		Upper Bound	52,2671	
	5% Trimmed Mean		46,8333	
	Median		49,0000	
	Variance		74,322	
	Std. Deviation		8,62103	
	Minimum		26,00	

	Maximum	53,00	
	Range	27,00	
	Interquartile Range	8,00	
	Skewness	-1,776	,687
	Kurtosis	2,755	1,334

Tests of Normality

		Kolmogorov-Smirnov[a]			Shapiro-Wilk		
	Gruplar	Statistic	df	Sig.	Statistic	df	Sig.
F	1,00	,203	10	,200*	,883	10	,142
	2,00	,213	10	,200*	,957	10	,751
	3,00	,187	10	,200*	,939	10	,543
	4,00	,210	10	,200*	,937	10	,517
	5,00	,150	10	,200*	,951	10	,685
	6,00	,212	10	,200*	,894	10	,187
	7,00	,230	10	,144	,863	10	,082
	8,00	,256	10	,061	,891	10	,172
	9,00	,180	10	,200*	,955	10	,733
	10,00	,295	10	,014	,772	10	,007
	7,00	,189	10	,200*	,878	10	,124
	8,00	,190	10	,200*	,875	10	,114
	9,00	,145	10	,200*	,934	10	,491
	10,00	,176	10	,200*	,932	10	,466

*. This is a lower bound of the true significance.

a. Lilliefors Significance Correction

F

Histograms

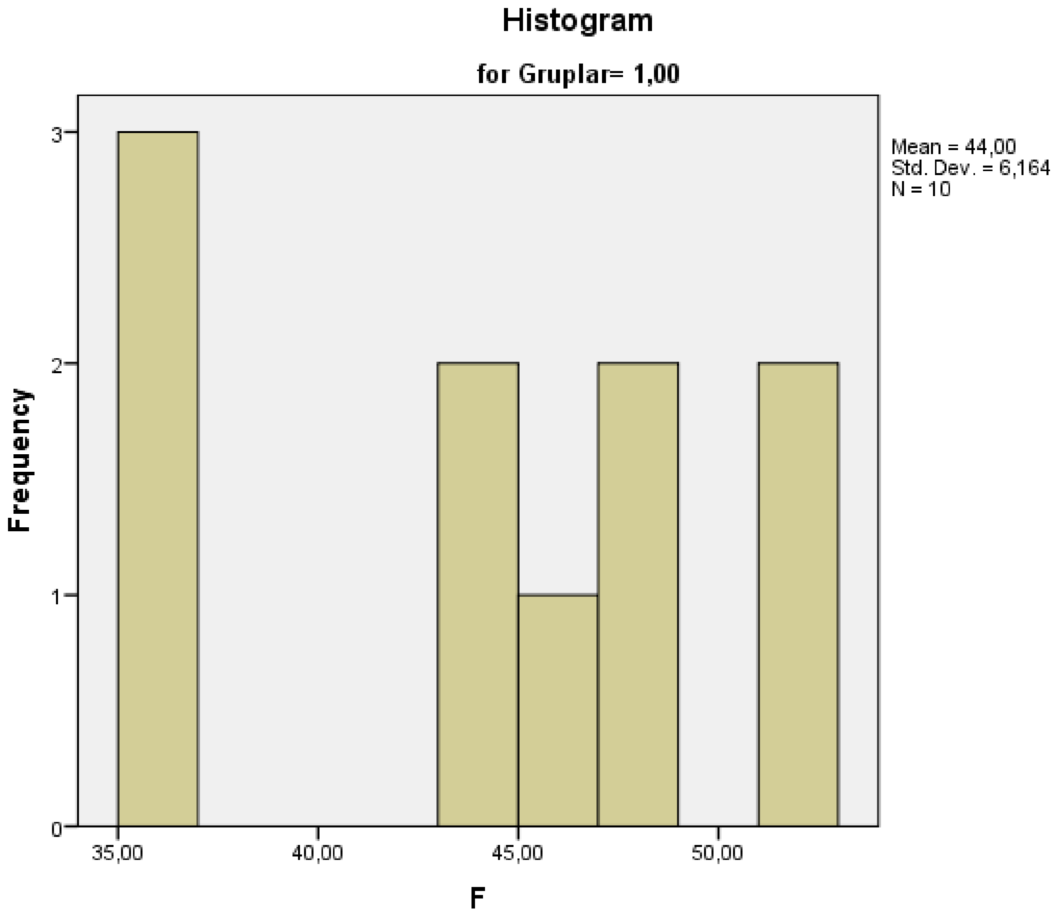

Histogram
for Gruplar= 1,00
Mean = 44,00
Std. Dev. = 6,164
N = 10
Frequency
3
2
1
0
35,00
40,00
45,00
50,00
F

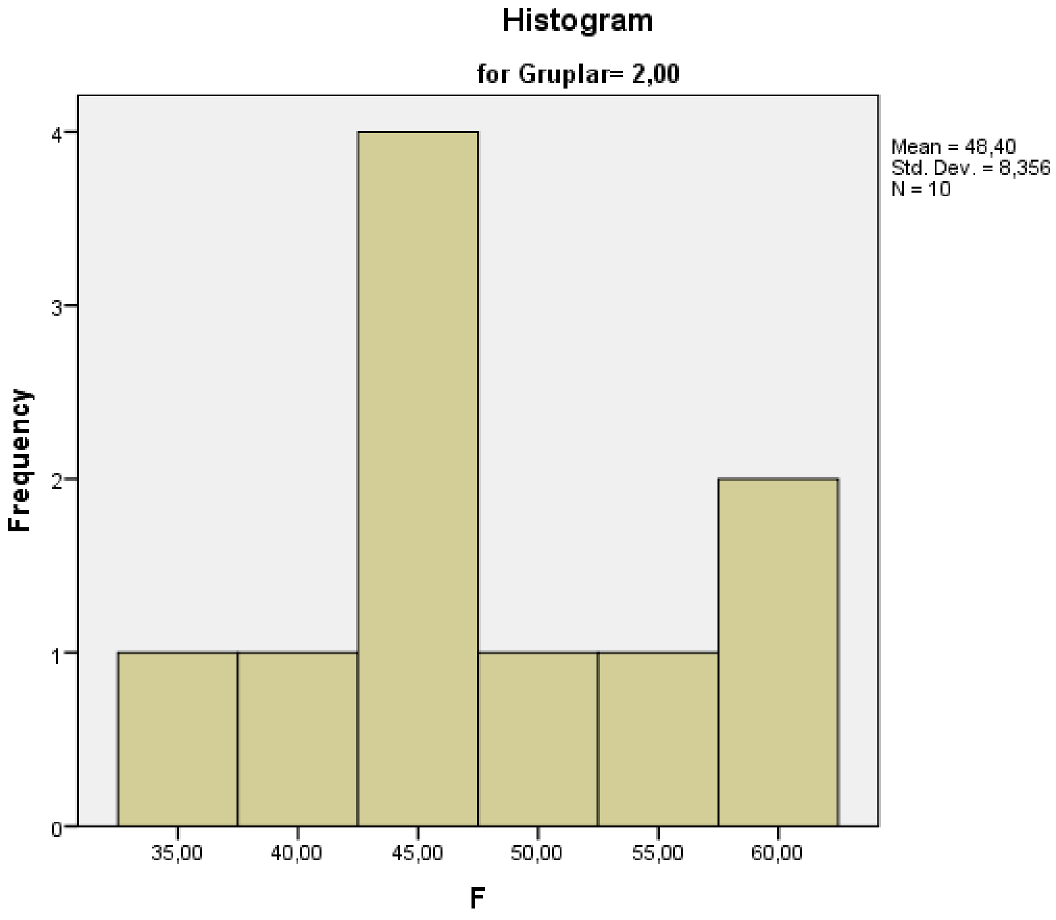
Histogram
for Gruplar= 2,00
Mean = 48,40
Std. Dev. = 8,356
N = 10
Frequency
0
1
2
3
4
35,00
40,00
45,00
50,00
55,00
60,00
F

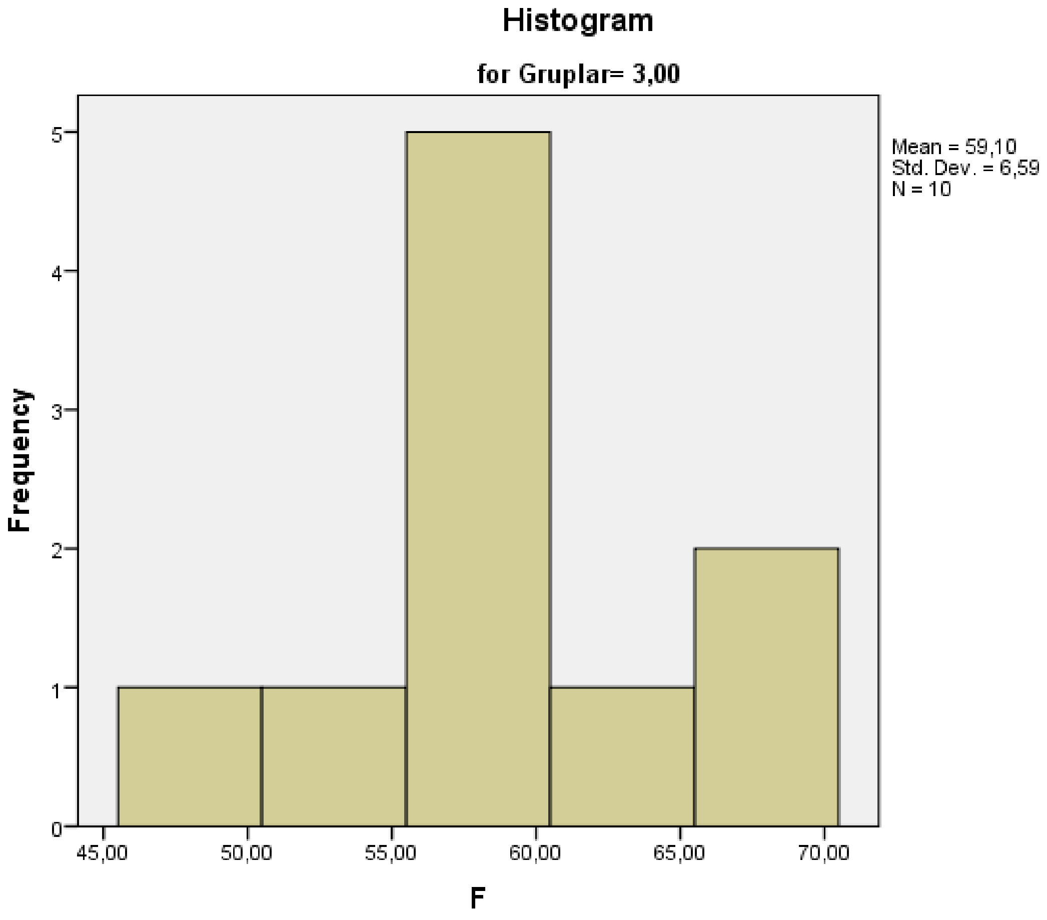
Histogram
for Gruplar= 3,00
Mean = 59,10
Std. Dev. = 6,59
N = 10
Frequency
0
1
2
3
4
5
45,00
50,00
55,00
60,00
65,00
70,00
F

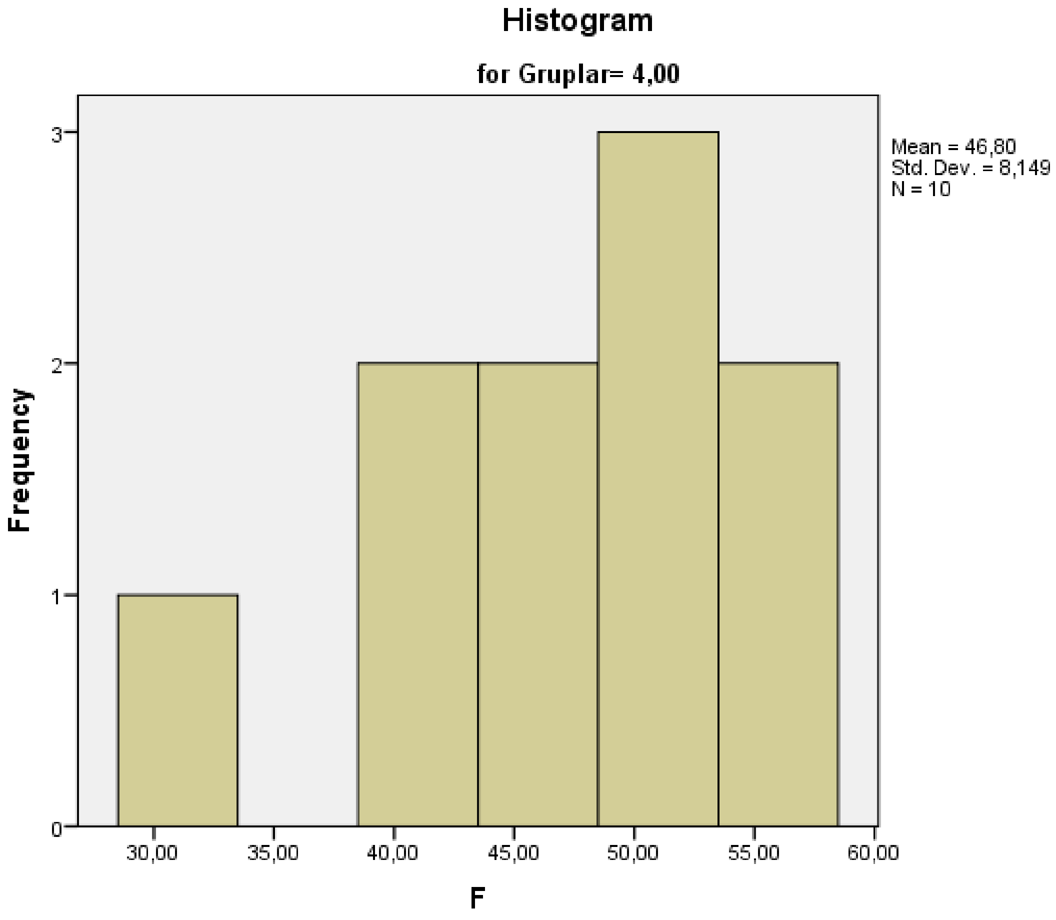

Histogram
for Gruplar= 4,00
Mean = 46,80
Std. Dev. = 8,149
N = 10
Frequency
0
1
2
3
30,00
35,00
40,00
45,00
50,00
55,00
60,00
F

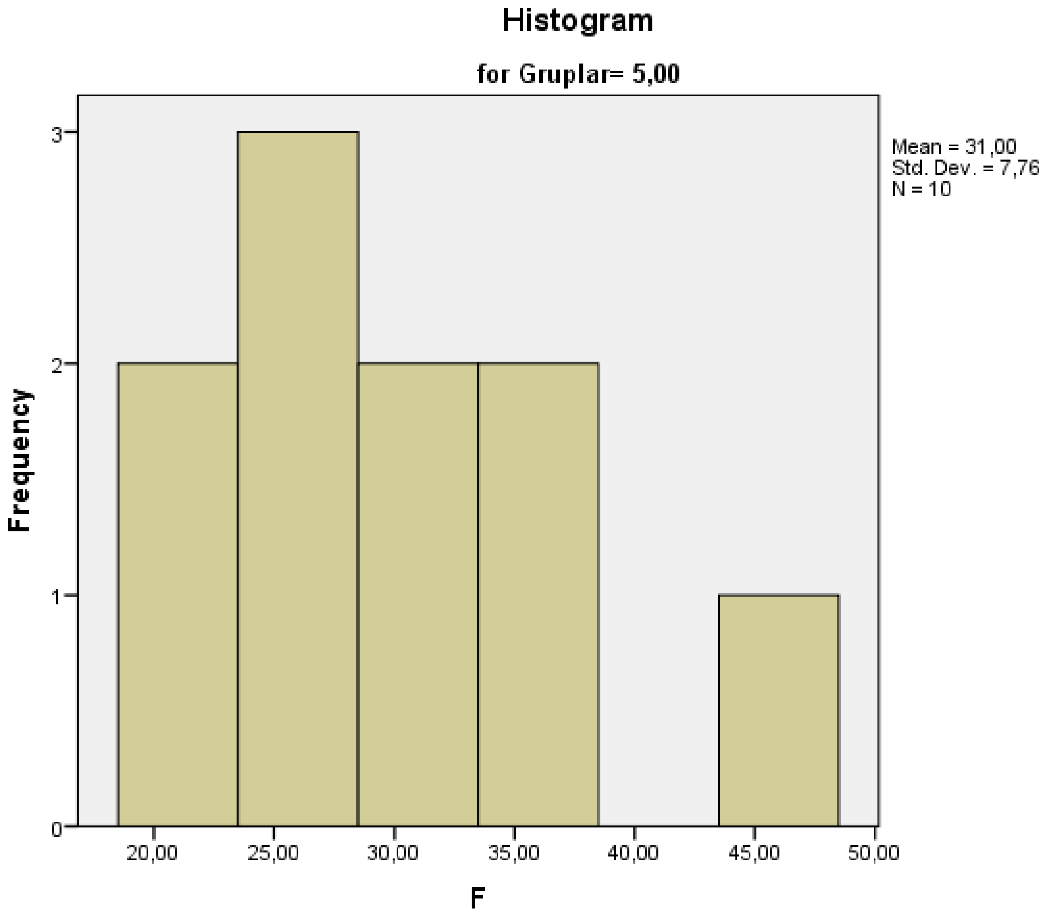

Histogram
for Gruplar= 5,00
Mean = 31,00
Std. Dev. = 7,76
N = 10
Frequency
0
1
2
3
20,00
25,00
30,00
35,00
40,00
45,00
50,00
F

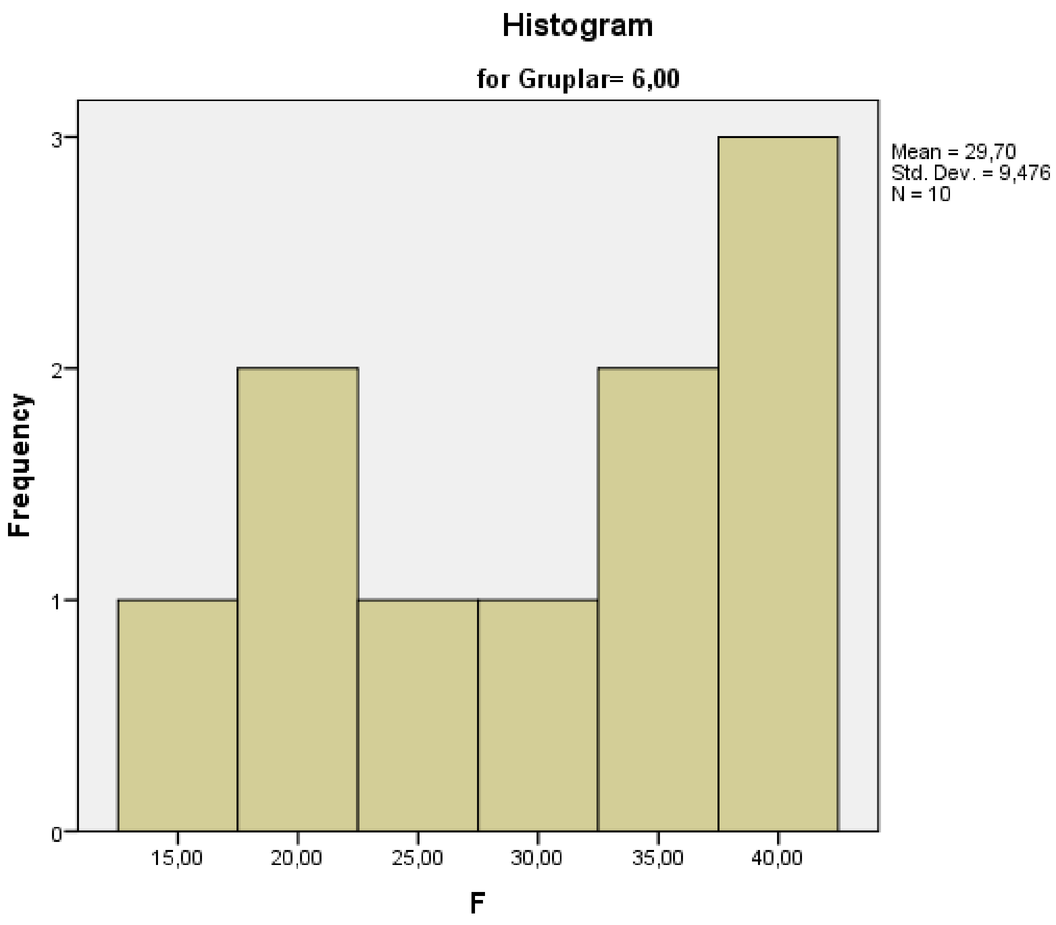

Histogram
for Gruplar= 6,00
Mean = 29,70
Std. Dev. = 9,476
N = 10
Frequency
0
1
2
3
15,00
20,00
25,00
30,00
35,00
40,00
F

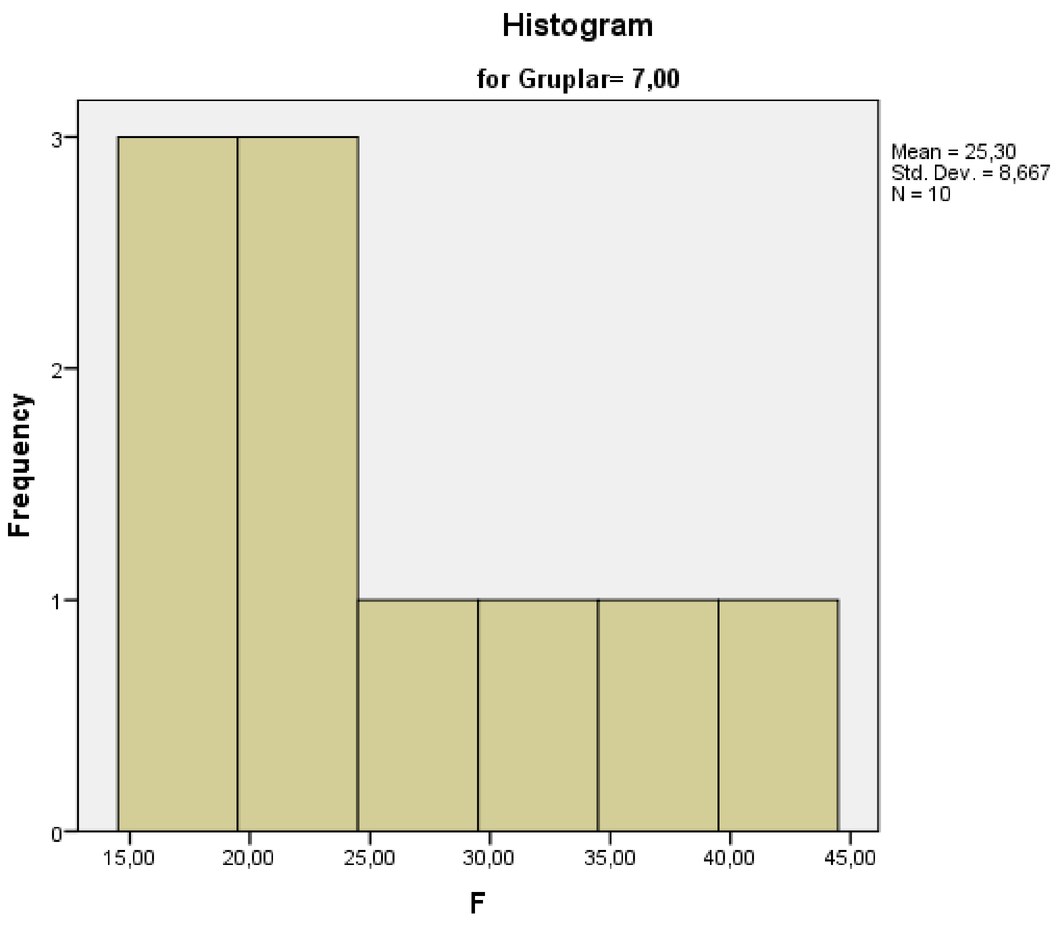
Histogram
for Gruplar= 7,00
Mean = 25,30
Std. Dev. = 8,667
N = 10
Frequency
3
2
1
0
15,00
20,00
25,00
30,00
35,00
40,00
45,00
F

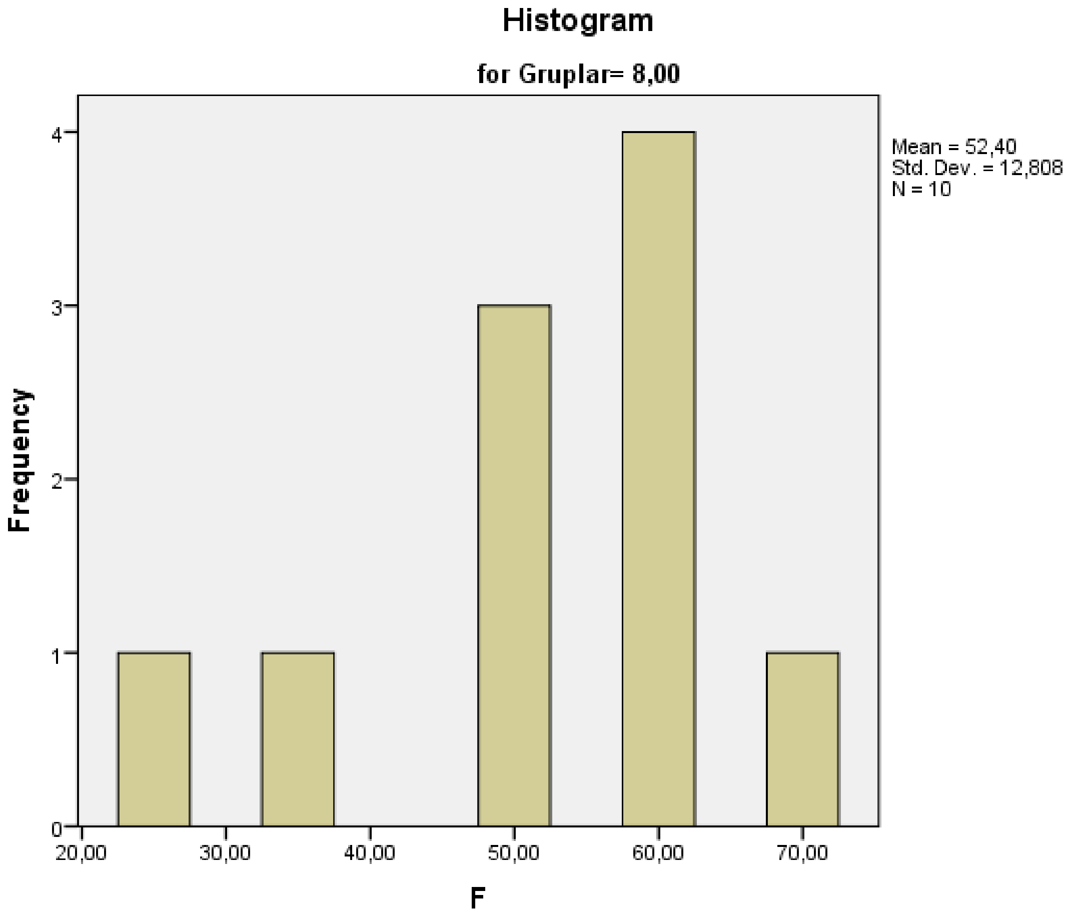
Histogram
for Gruplar= 8,00
Mean = 52,40
Std. Dev. = 12,808
N = 10
Frequency
0
1
2
3
4
20,00
30,00
40,00
50,00
60,00
70,00
F

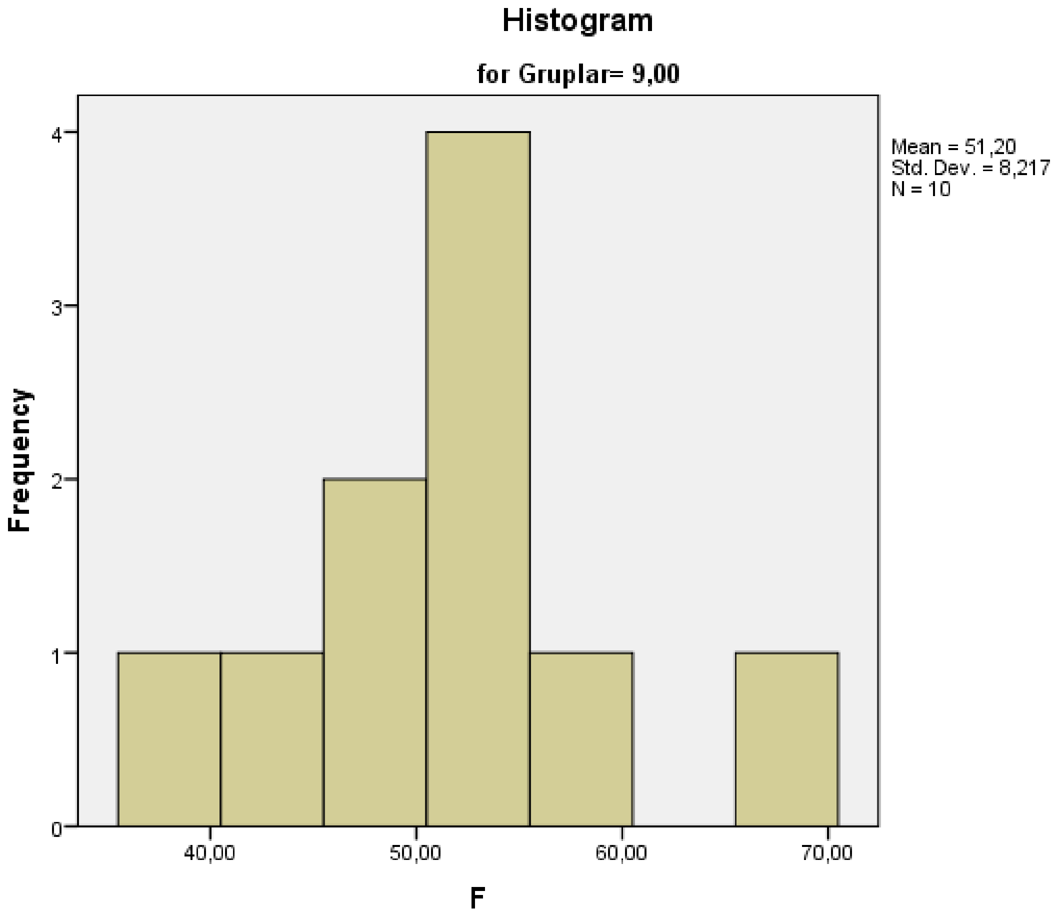
Histogram
for Gruplar= 9,00
Mean = 51,20
Std. Dev. = 8,217
N = 10
Frequency
4
3
2
1
0
40,00
50,00
60,00
70,00
F

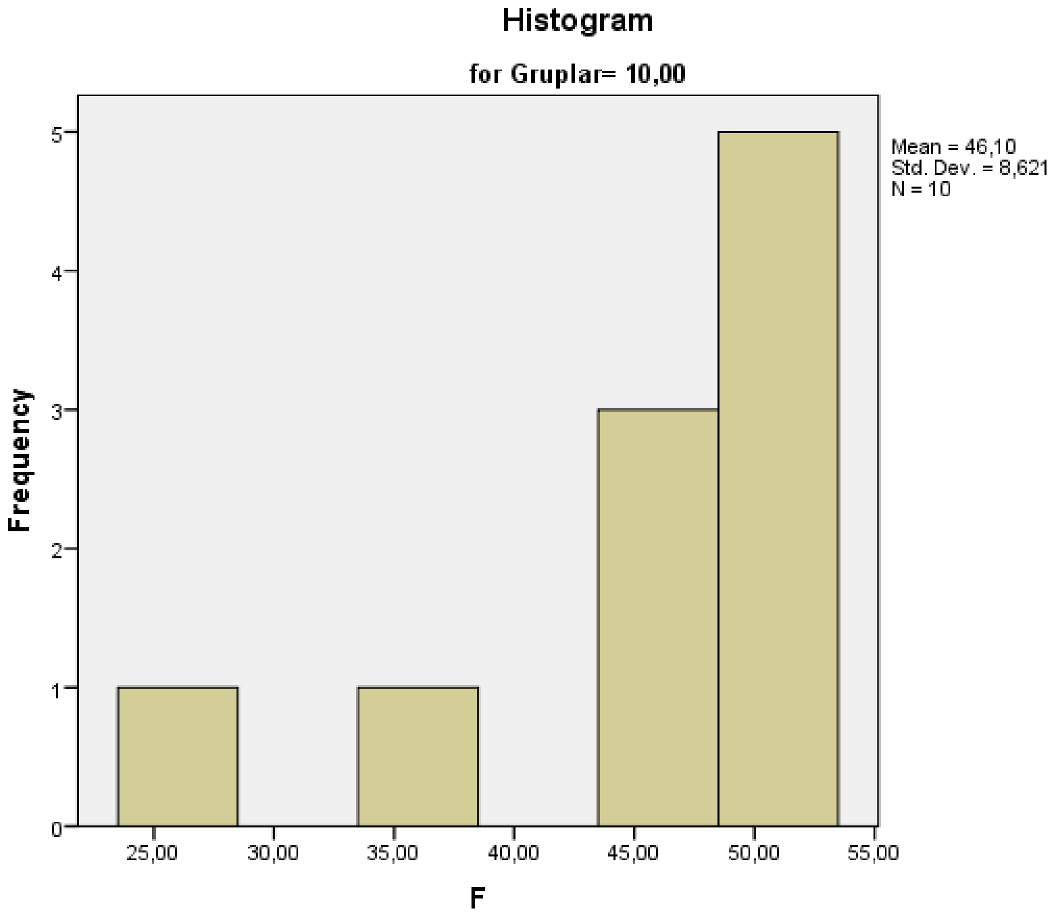

Stem-and-Leaf Plots

```
F Stem-and-Leaf Plot for
Gruplar= 1,00

 Frequency    Stem &  Leaf

     3,00        3 .  666
     2,00        4 .  34
     3,00        4 .  688
     2,00        5 .  12

 Stem width:  10,00
 Each leaf:       1 case(s)
```

```
F Stem-and-Leaf Plot for
Gruplar= 2,00

 Frequency    Stem &  Leaf

     1,00        3 .  5
     5,00        4 .  23466
     3,00        5 .  268
     1,00        6 .  2

 Stem width:  10,00
 Each leaf:       1 case(s)
```

```
F Stem-and-Leaf Plot for
Gruplar= 3,00

 Frequency    Stem &  Leaf

     1,00 Extremes    (=<48)
     1,00        5 .  4
     4,00        5 .  6689
     2,00        6 .  01
     2,00 Extremes    (>=69)

 Stem width:  10,00
 Each leaf:       1 case(s)
```

```
F Stem-and-Leaf Plot for
Gruplar= 4,00

 Frequency    Stem &  Leaf

     1,00        3 .  1
     2,00        3 .  99
      ,00        4 .
     2,00        4 .  78
     4,00        5 .  0024
     1,00        5 .  8

 Stem width:  10,00
 Each leaf:       1 case(s)
```

```
F Stem-and-Leaf Plot for
Gruplar= 5,00

 Frequency    Stem &  Leaf
```

```
     2,00        2 .  11
     3,00        2 .  688
     2,00        3 .  23
     2,00        3 .  88
      ,00        4 .
     1,00        4 .  5

 Stem width:  10,00
 Each leaf:       1 case(s)
```

F Stem-and-Leaf Plot for
Gruplar= 6,00

```
 Frequency    Stem &  Leaf

     3,00        1 .  589
     1,00        2 .  7
     5,00        3 .  05688
     1,00        4 .  1

 Stem width:  10,00
 Each leaf:       1 case(s)
```

F Stem-and-Leaf Plot for
Gruplar= 7,00

```
 Frequency    Stem &  Leaf

     3,00        1 .  789
     4,00        2 .  0036
     2,00        3 .  25
     1,00        4 .  3

 Stem width:  10,00
 Each leaf:       1 case(s)
```

F Stem-and-Leaf Plot for
Gruplar= 8,00

```
 Frequency    Stem &  Leaf

     1,00 Extremes    (=<25)
     1,00        3 .  7
      ,00        4 .
     5,00        5 .  11289
     3,00        6 .  128
```

```
 Stem width:  10,00
 Each leaf:       1 case(s)
```

```
F Stem-and-Leaf Plot for
Gruplar= 9,00

 Frequency    Stem &  Leaf

     1,00        3 .  8
     1,00        4 .  2
     2,00        4 .  88
     3,00        5 .  124
     2,00        5 .  56
     1,00 Extremes    (>=68)

 Stem width:  10,00
 Each leaf:       1 case(s)
```

```
F Stem-and-Leaf Plot for
Gruplar= 10,00

 Frequency    Stem &  Leaf

     2,00 Extremes    (=<36)
     3,00        4 .  678
     5,00        5 .  01133

 Stem width:  10,00
 Each leaf:       1 case(s)
```

Normal Q-Q Plots

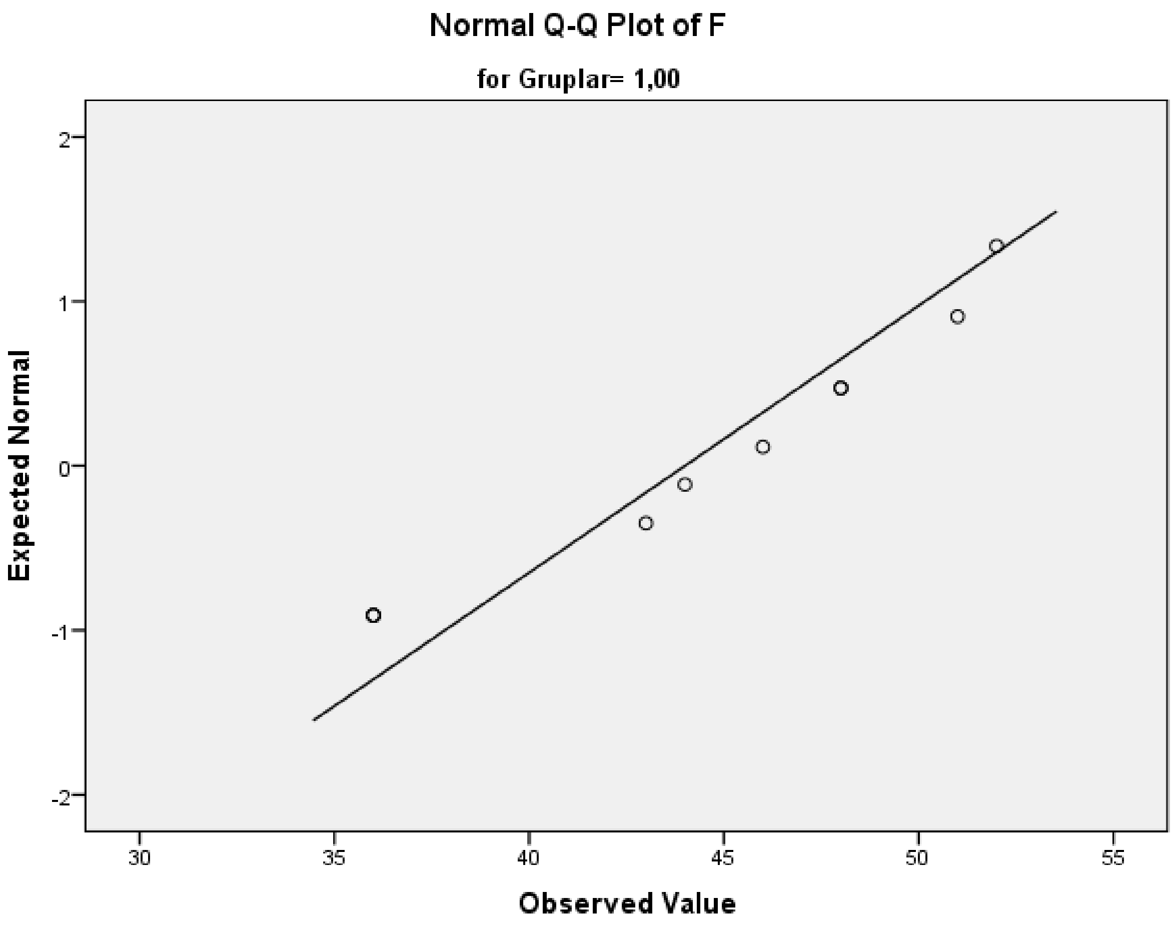

Normal Q-Q Plot of F
for Gruplar= 1,00
Expected Normal
2
1
0
-1
-2
30
35
40
45
50
55
Observed Value

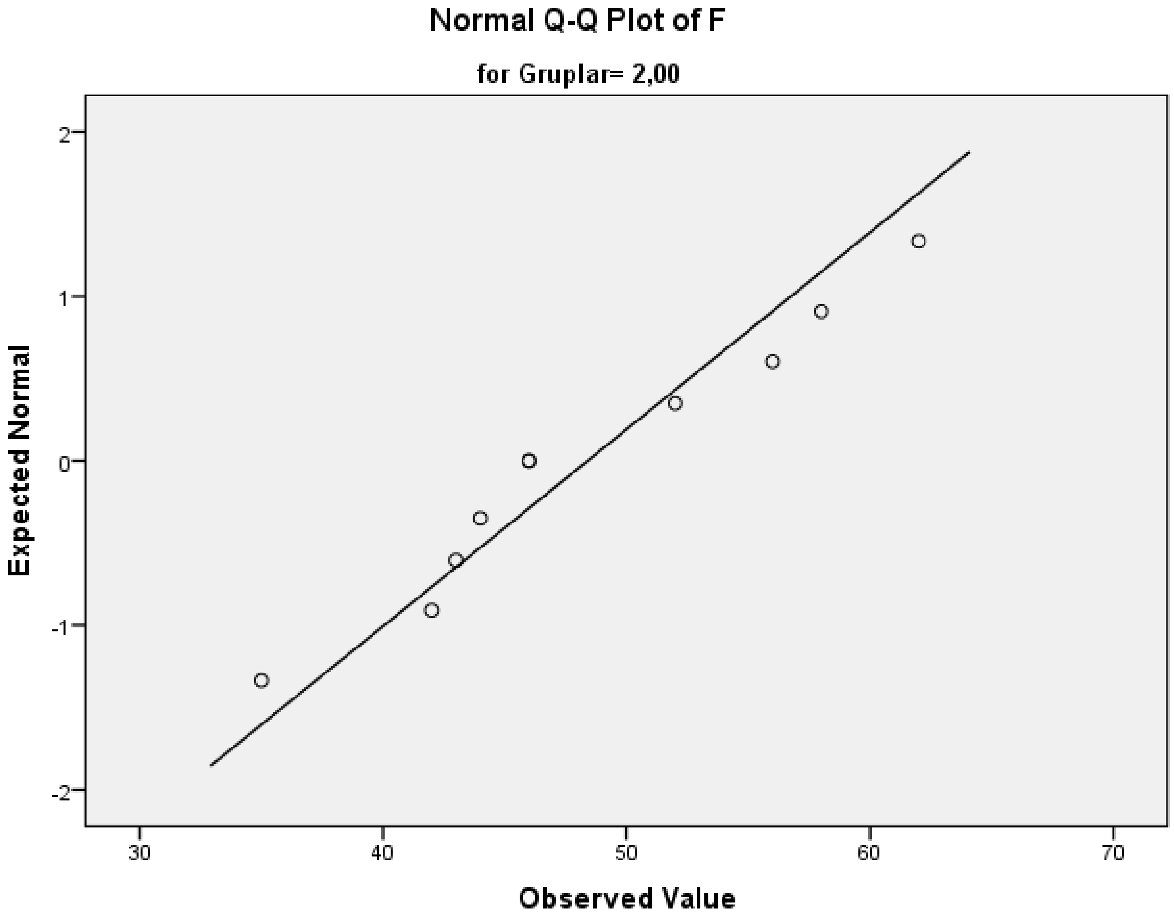
Normal Q-Q Plot of F
for Gruplar= 2,00
Expected Normal
Observed Value
2
1
0
-1
-2
30
40
50
60
70

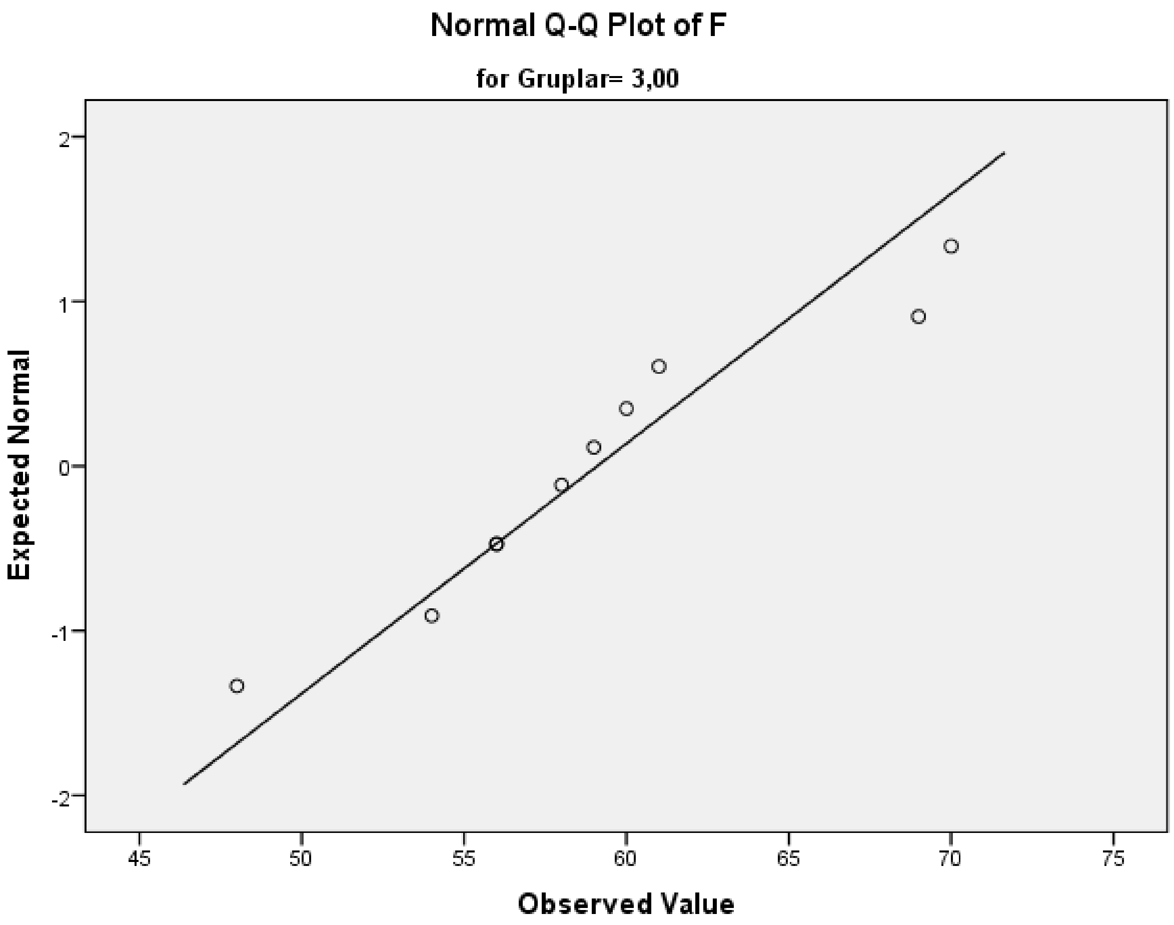
Normal Q-Q Plot of F
for Gruplar= 3,00
Expected Normal
Observed Value
2
1
0
-1
-2
45
50
55
60
65
70
75

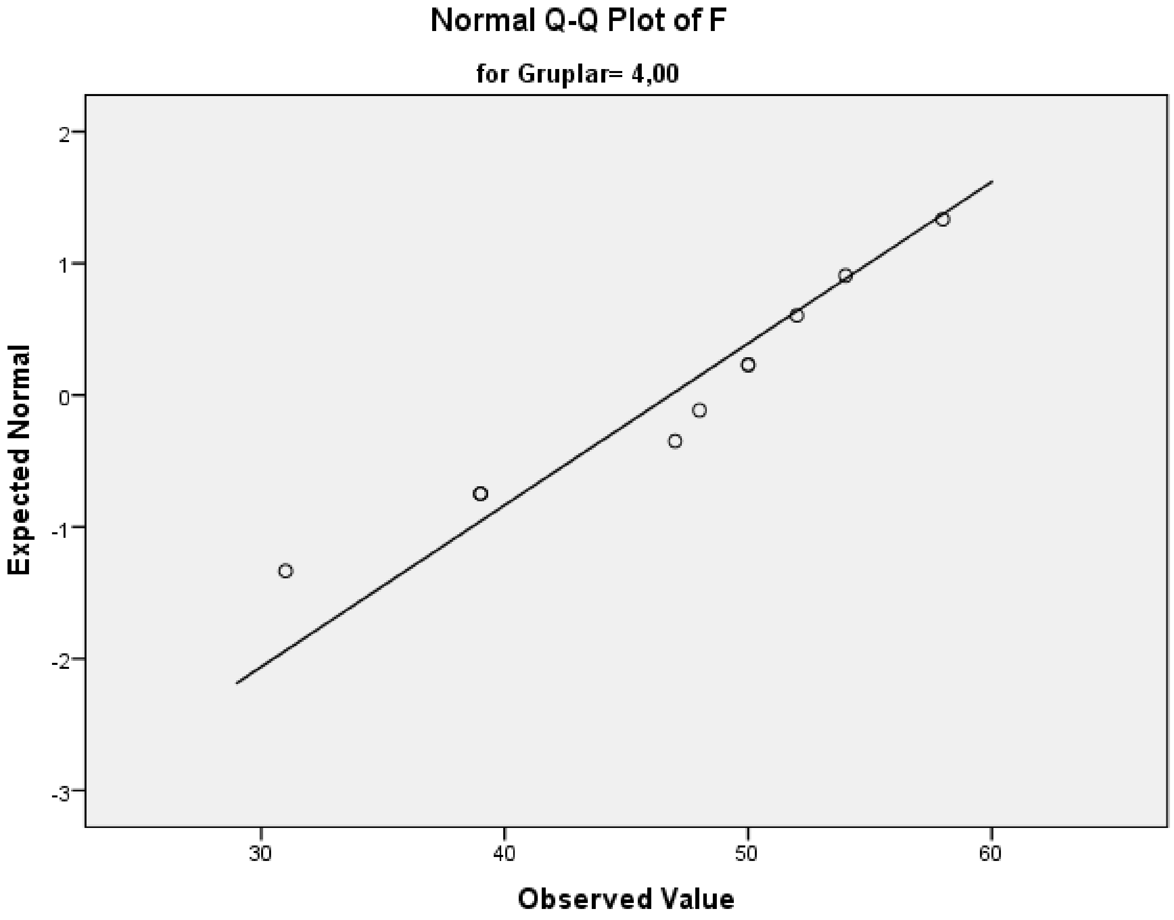

Normal Q-Q Plot of F
for Gruplar= 4,00
Expected Normal
Observed Value
2
1
0
-1
-2
-3
30
40
50
60

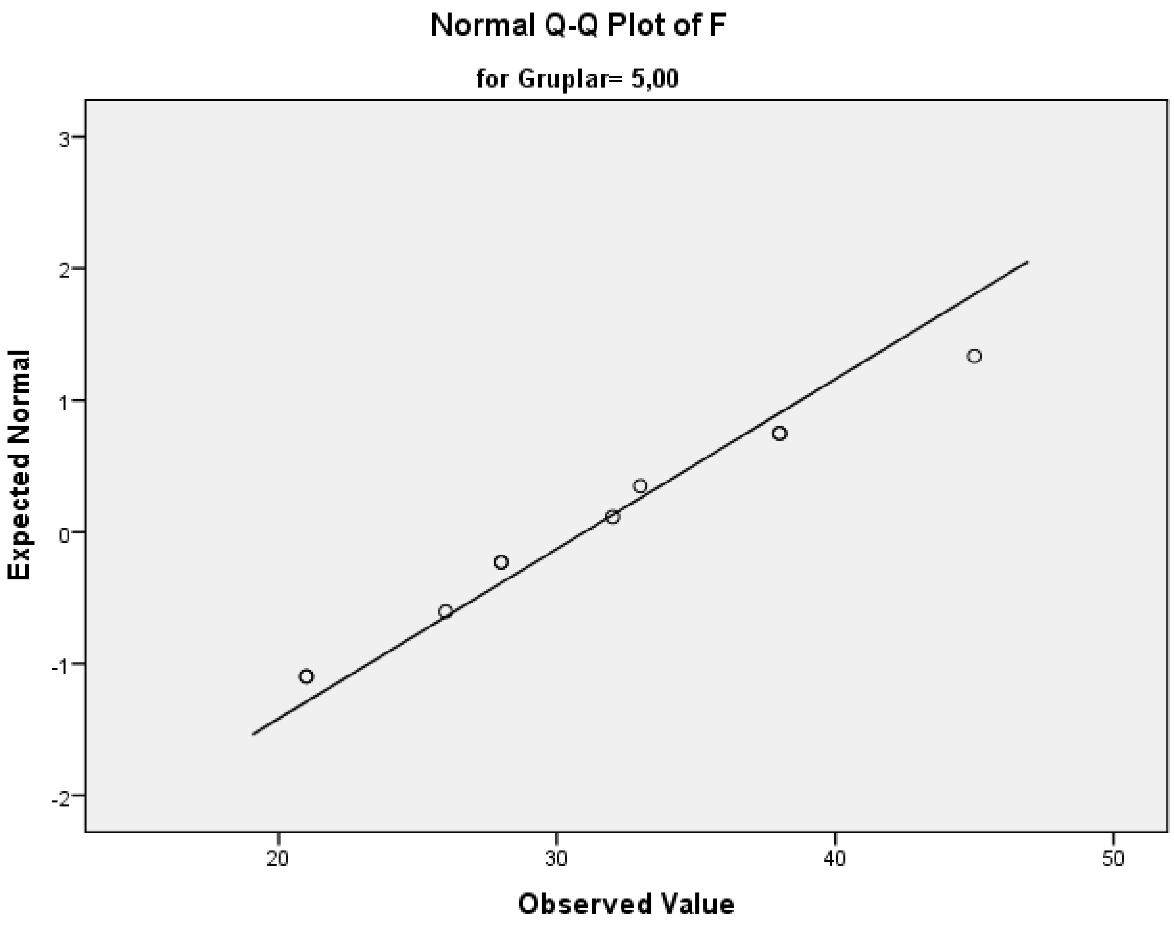
Normal Q-Q Plot of F
for Gruplar= 5,00
Expected Normal
3
2
1
0
-1
-2
20
30
40
50
Observed Value

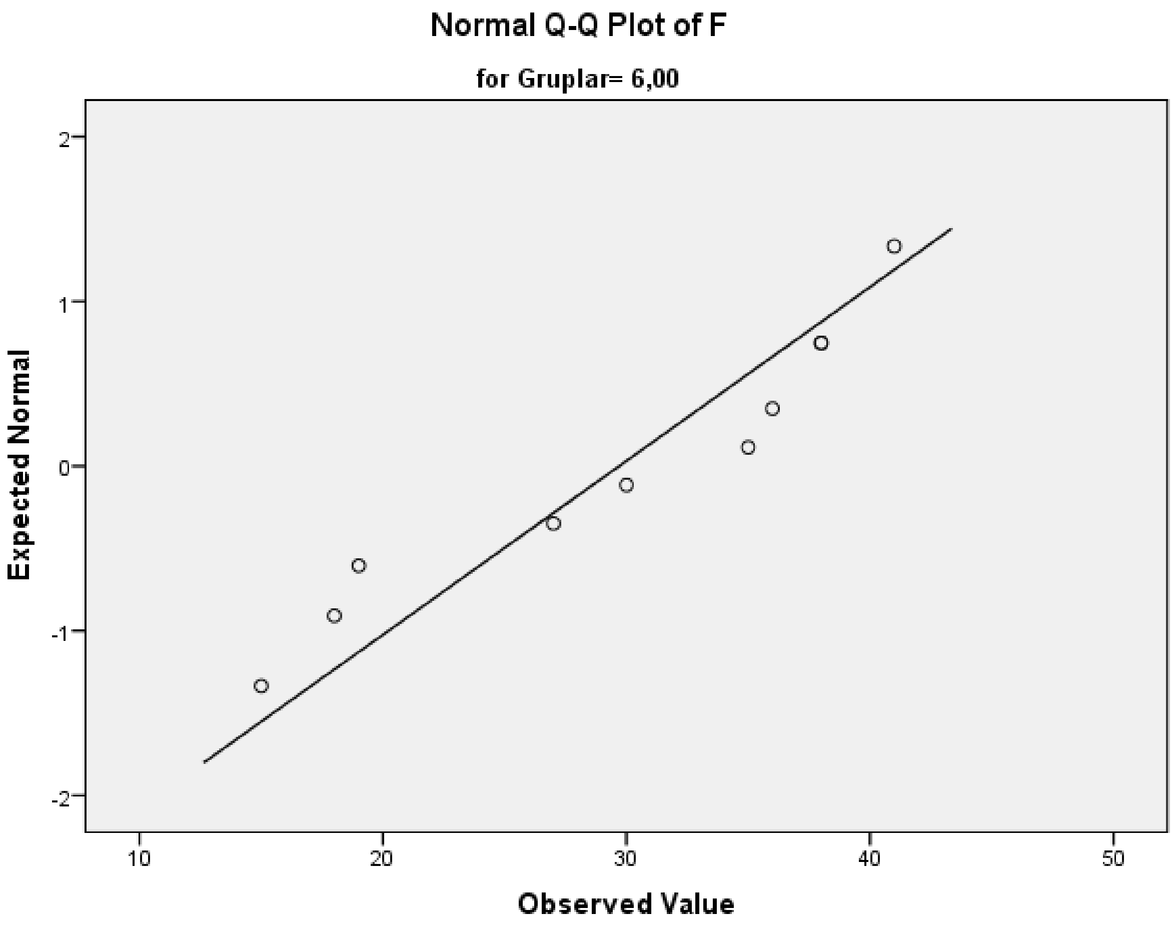
Normal Q-Q Plot of F
for Gruplar= 6,00
Expected Normal
2
1
0
-1
-2
10
20
30
40
50
Observed Value

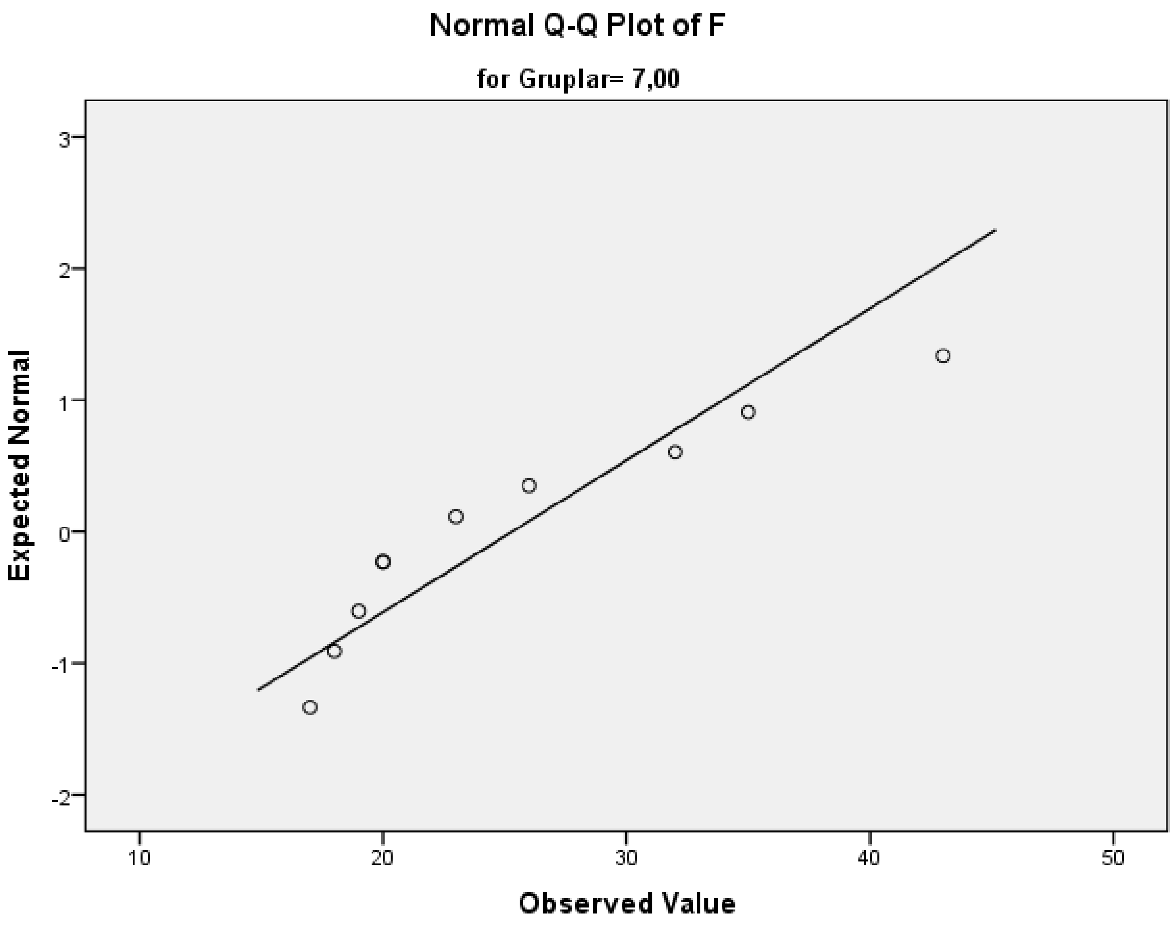
Normal Q-Q Plot of F
for Gruplar= 7,00
Expected Normal
3
2
1
0
-1
-2
10
20
30
40
50
Observed Value

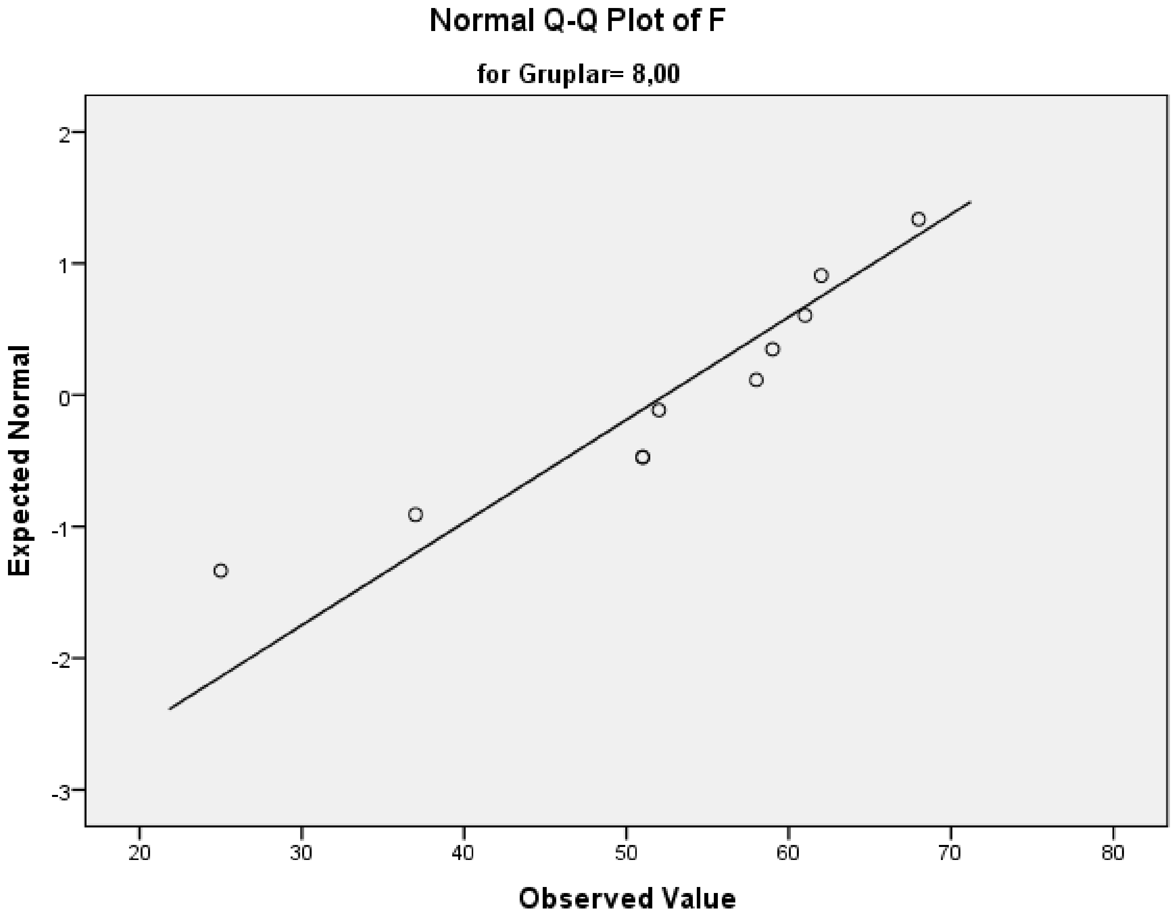
Normal Q-Q Plot of F
for Gruplar= 8,00
Expected Normal
2
1
0
-1
-2
-3
20
30
40
50
60
70
80
Observed Value

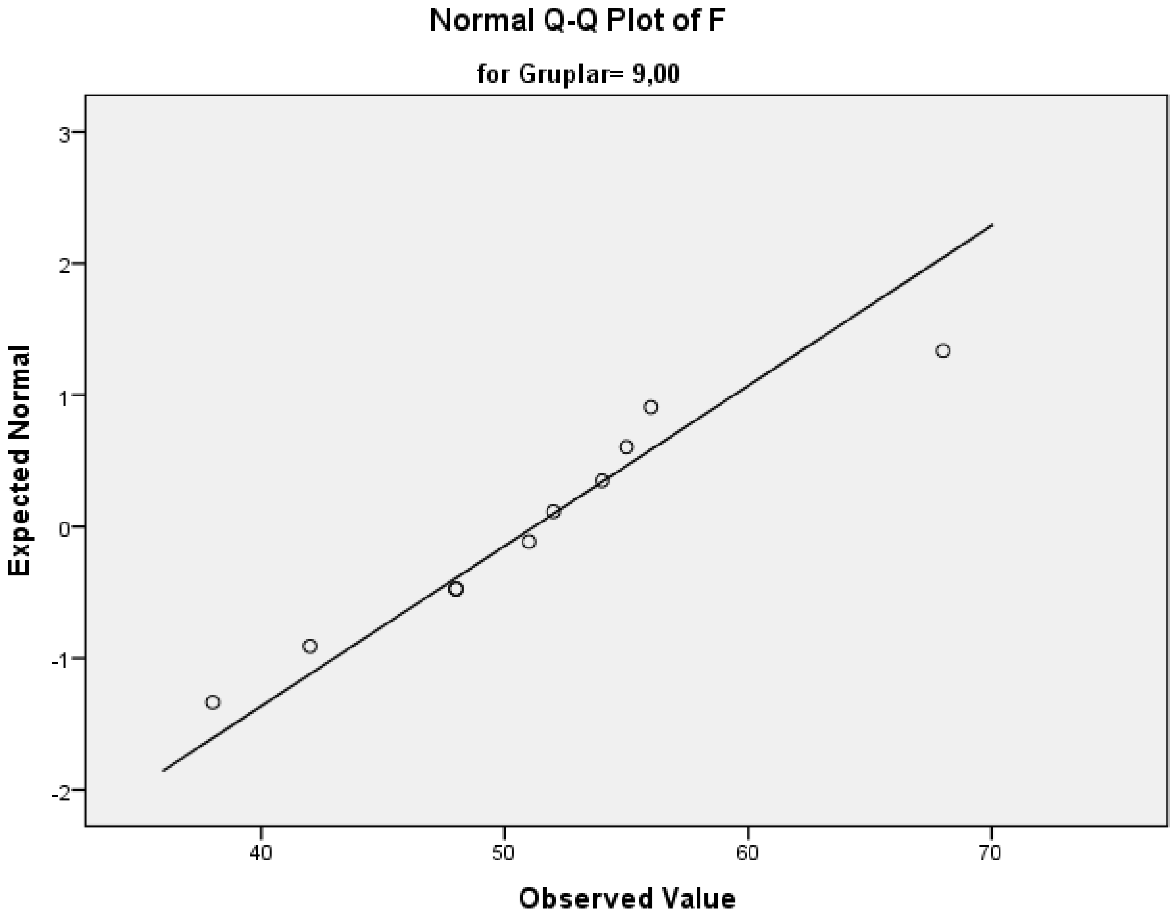
Normal Q-Q Plot of F
for Gruplar= 9,00
Expected Normal
Observed Value
3
2
1
0
-1
-2
40
50
60
70

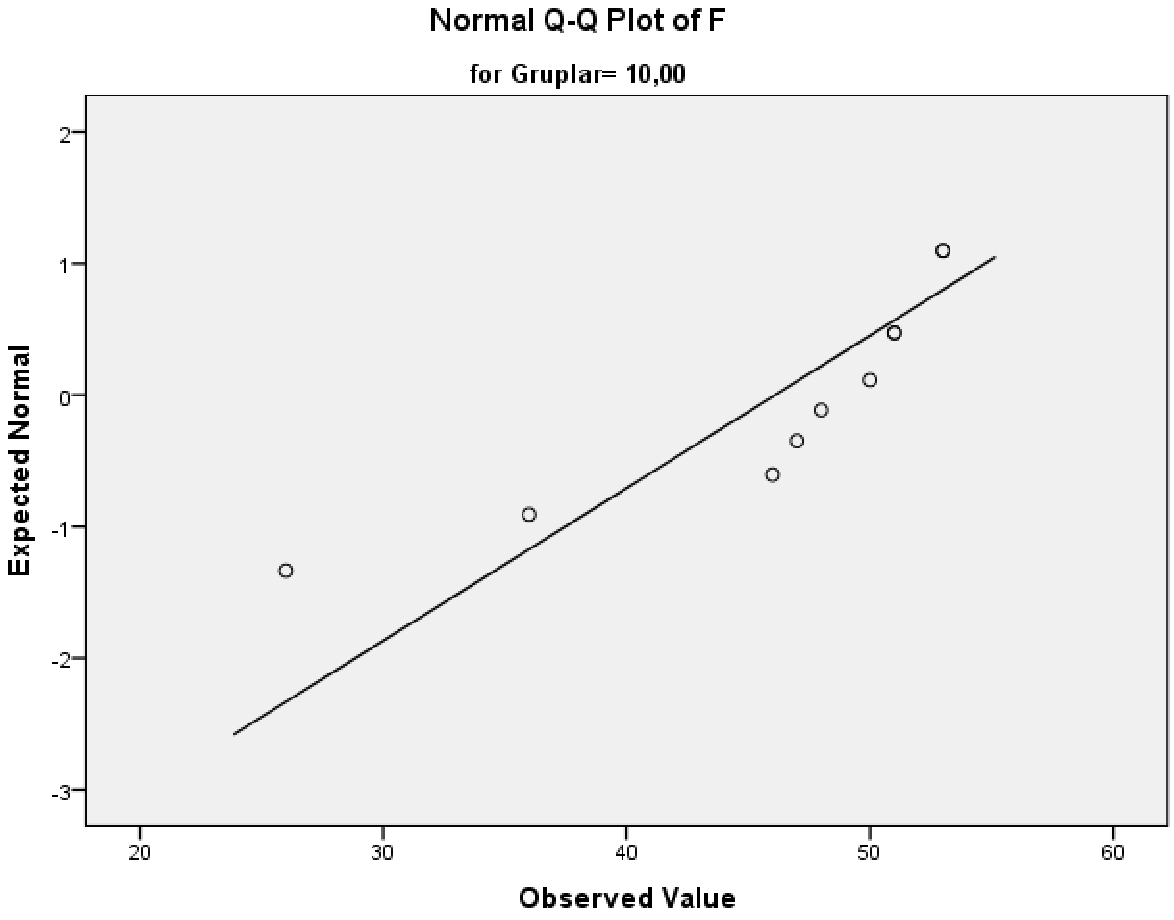

Detrended Normal Q-Q Plots

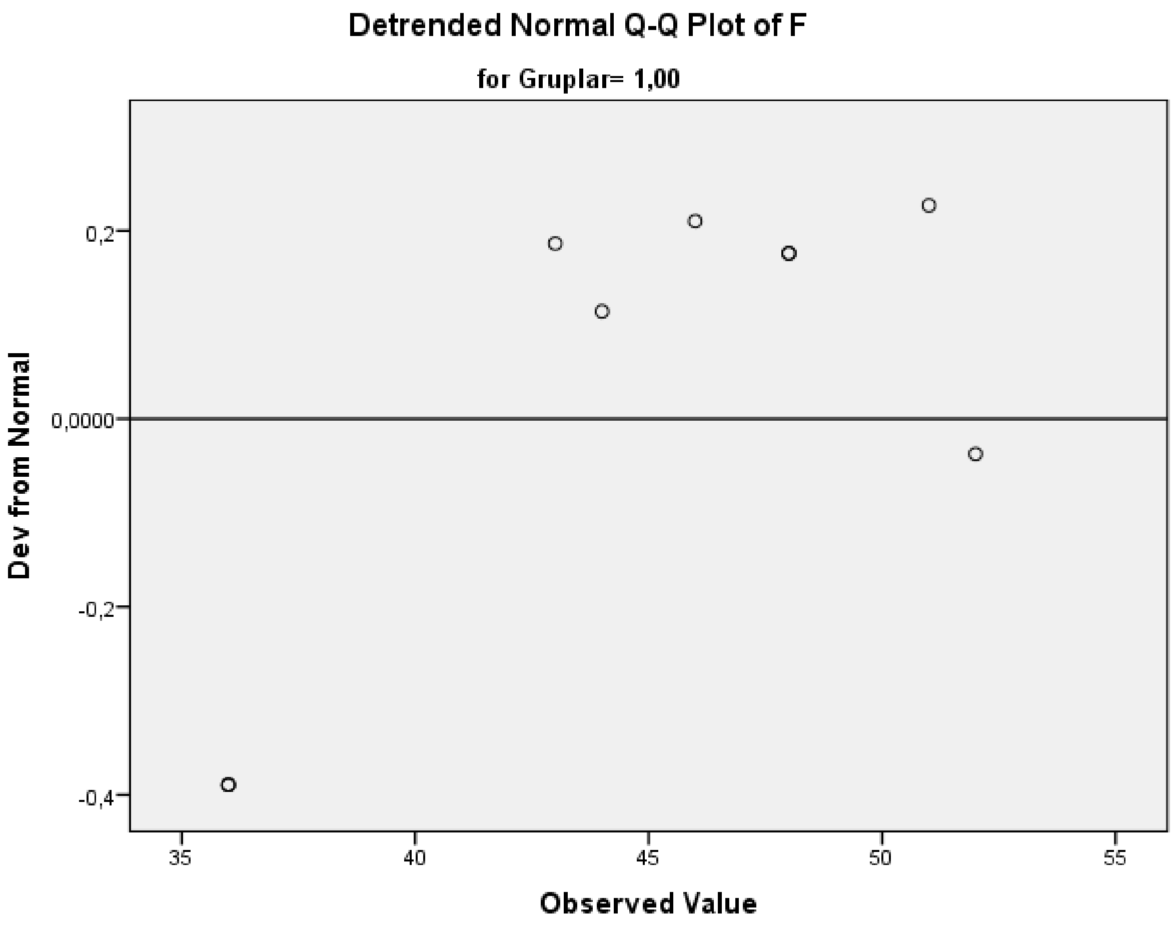
Detrended Normal Q-Q Plot of F
for Gruplar= 1,00
Dev from Normal
0,2
0,0000
-0,2
-0,4
35
40
45
50
55
Observed Value

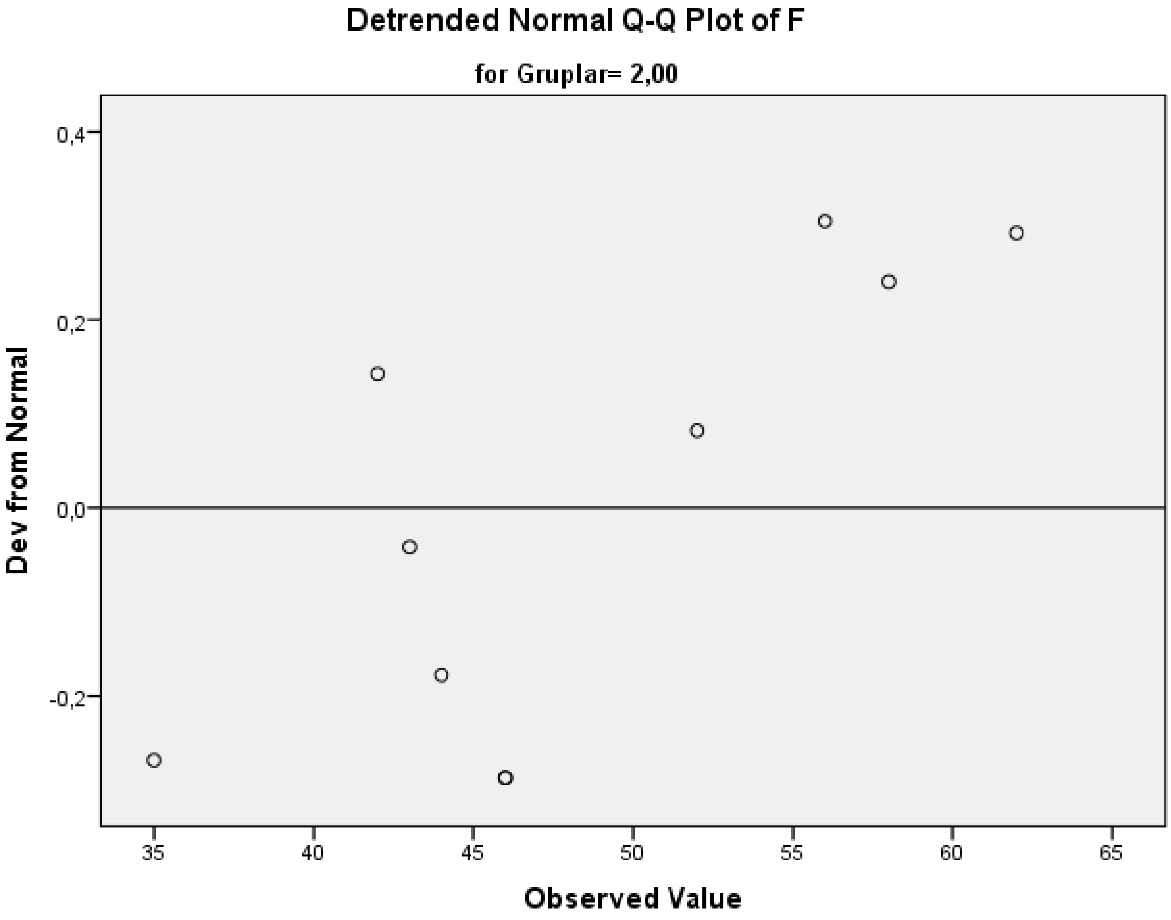
Detrended Normal Q-Q Plot of F
for Gruplar= 2,00
Dev from Normal
0,4
0,2
0,0
-0,2
35
40
45
50
55
60
65
Observed Value

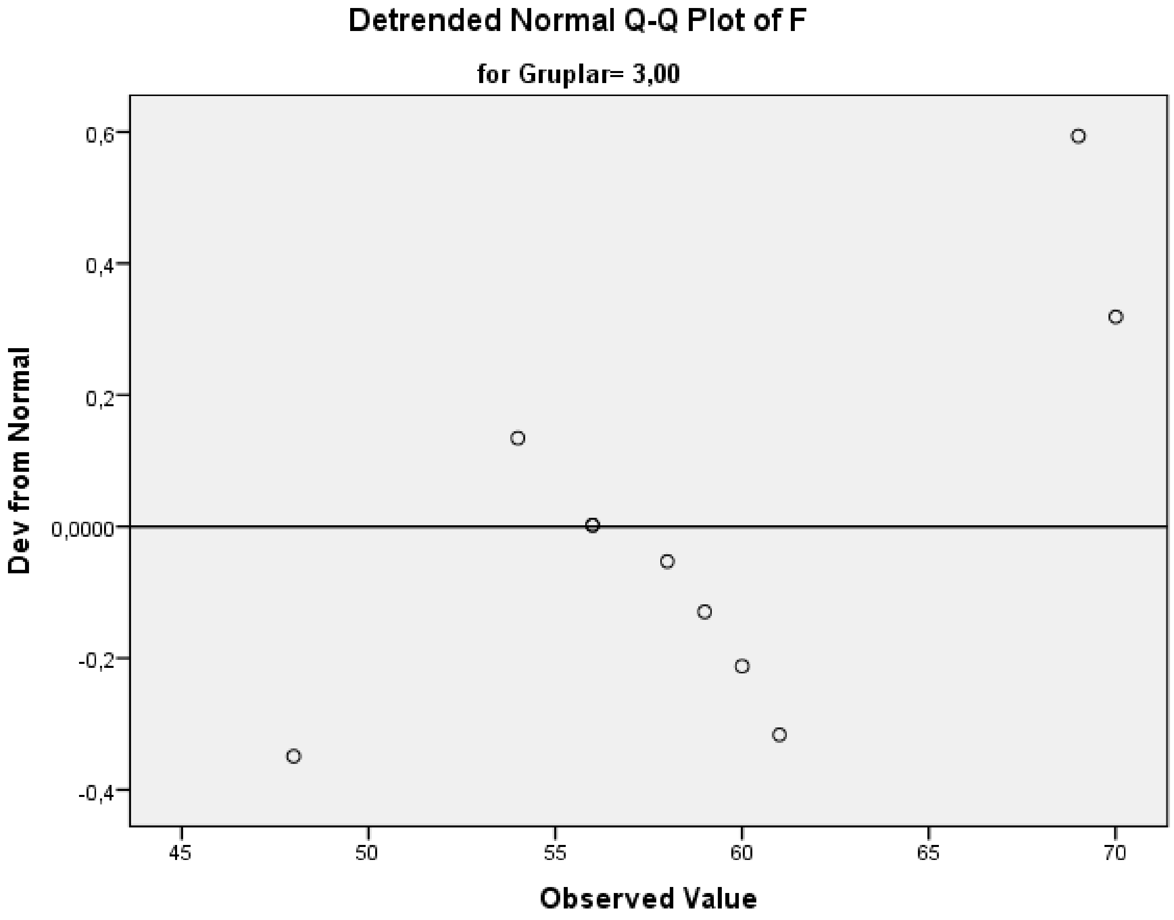

Detrended Normal Q-Q Plot of F
for Gruplar= 3,00
Dev from Normal
0,6
0,4
0,2
0,0000
-0,2
-0,4
45
50
55
60
65
70
Observed Value

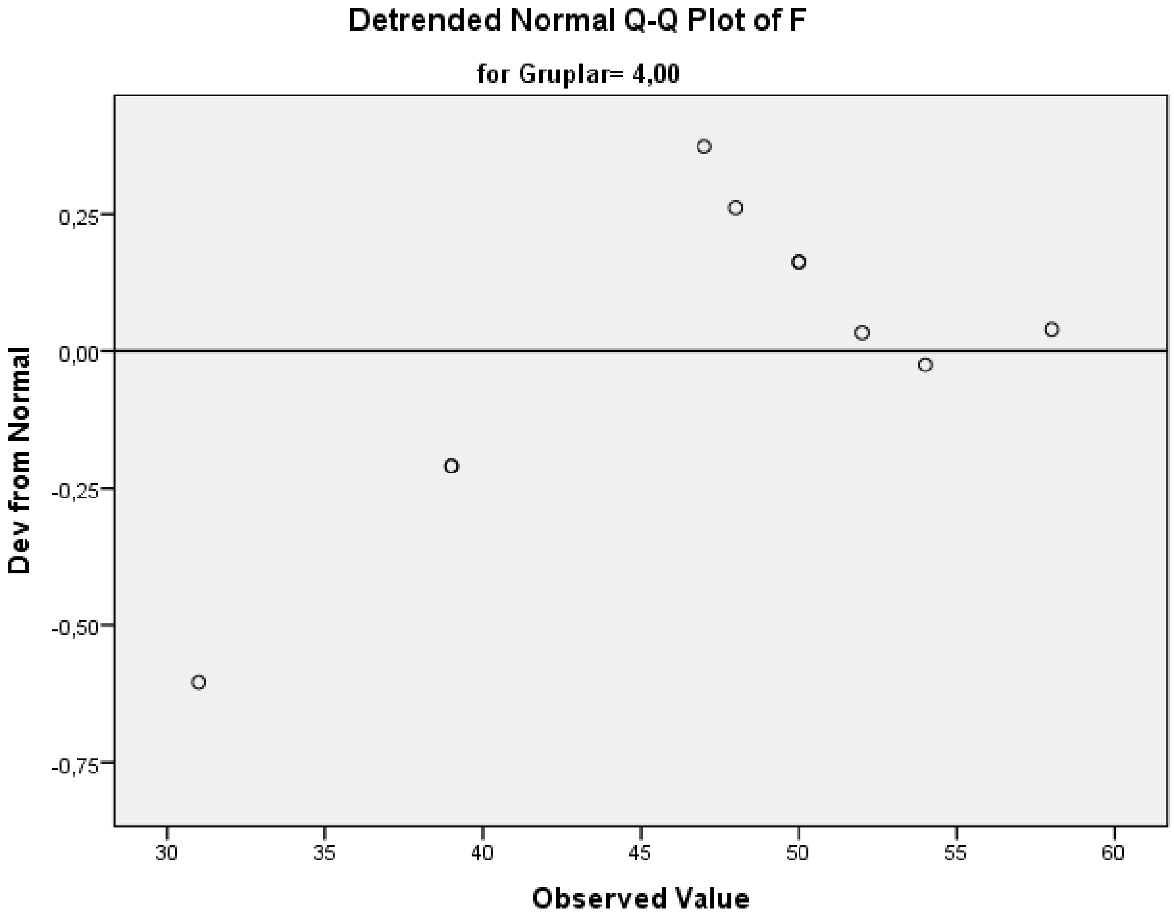

Detrended Normal Q-Q Plot of F
for Gruplar= 4,00
Dev from Normal
0,25
0,00
-0,25
-0,50
-0,75
30
35
40
45
50
55
60
Observed Value

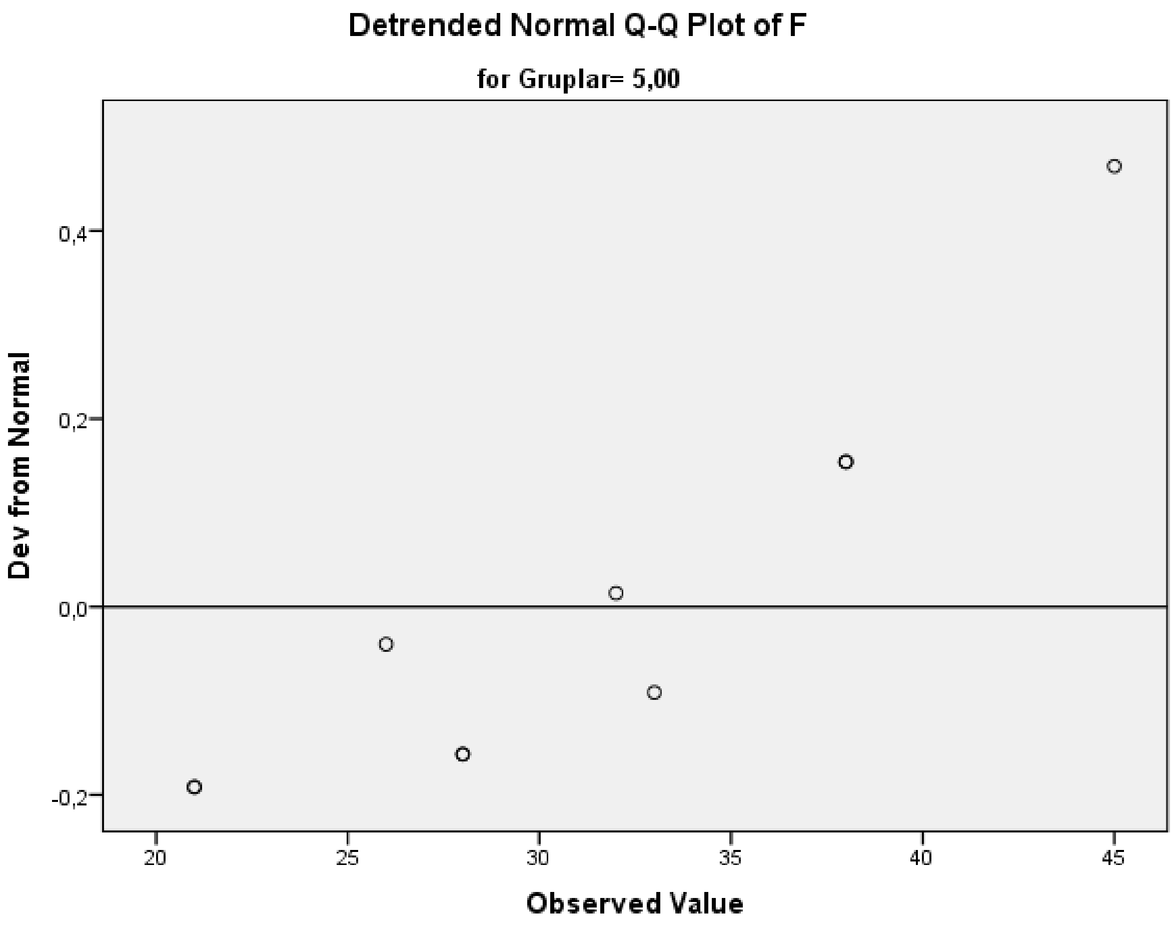
Detrended Normal Q-Q Plot of F
for Gruplar= 5,00
Dev from Normal
0,4
0,2
0,0
-0,2
20
25
30
35
40
45
Observed Value

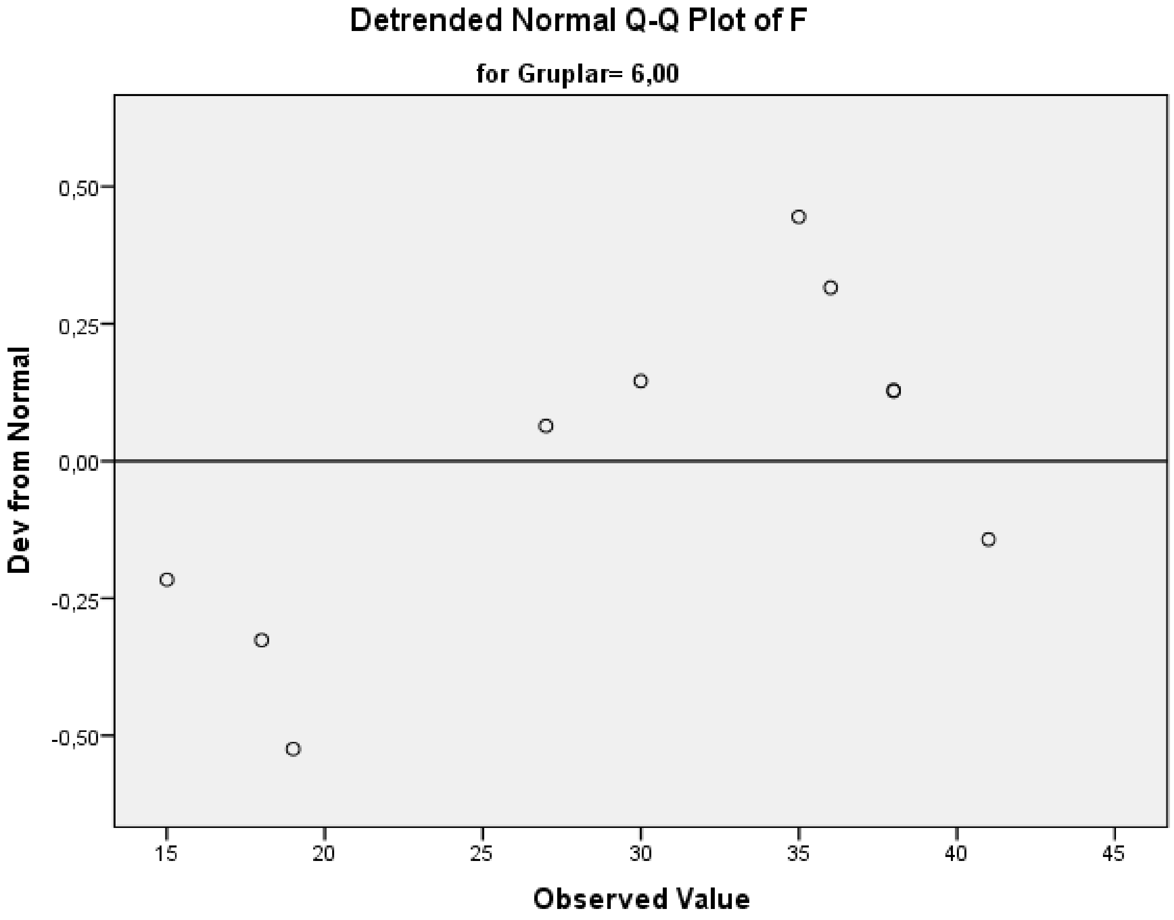
Detrended Normal Q-Q Plot of F
for Gruplar= 6,00
Dev from Normal
0,50
0,25
0,00
-0,25
-0,50
15
20
25
30
35
40
45
Observed Value

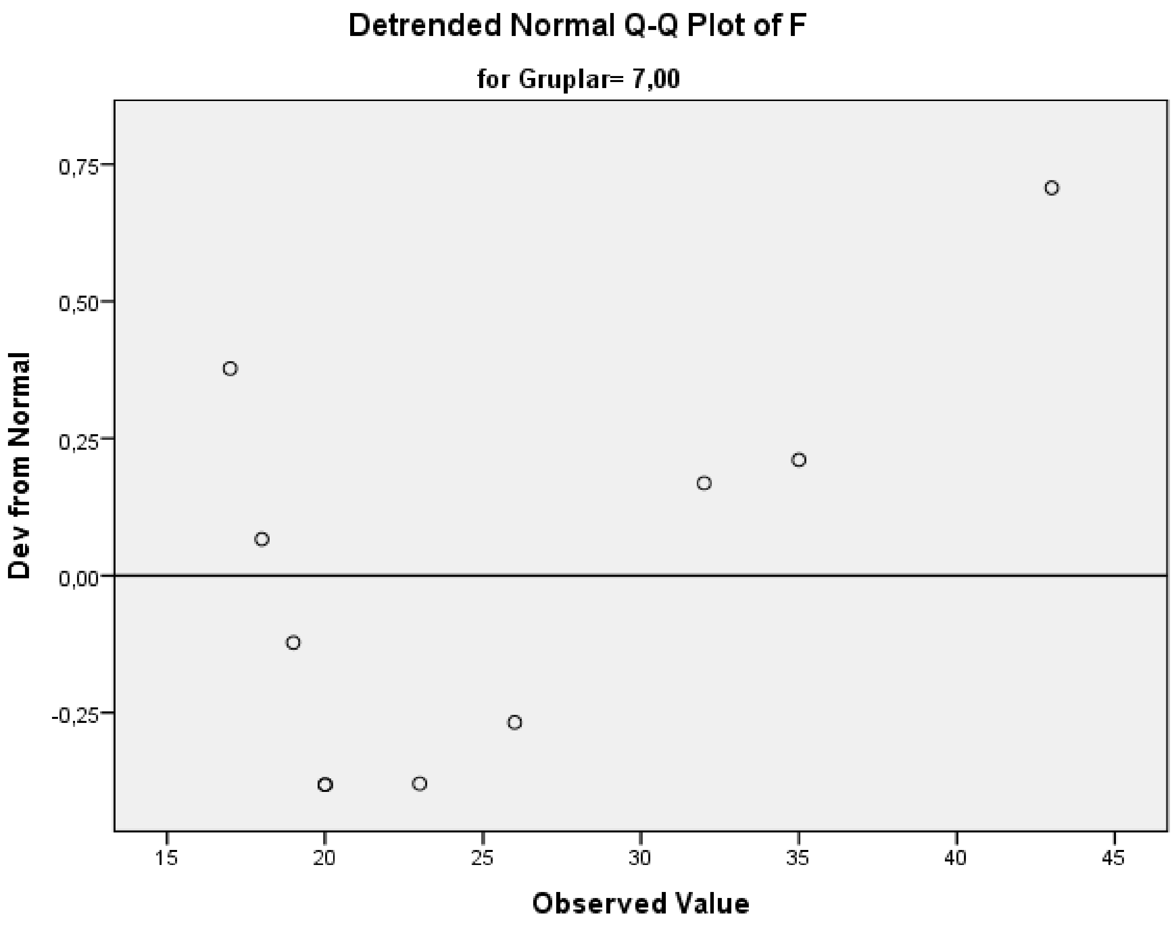

Detrended Normal Q-Q Plot of F
for Gruplar= 7,00
Dev from Normal
0,75
0,50
0,25
0,00
-0,25
15
20
25
30
35
40
45
Observed Value

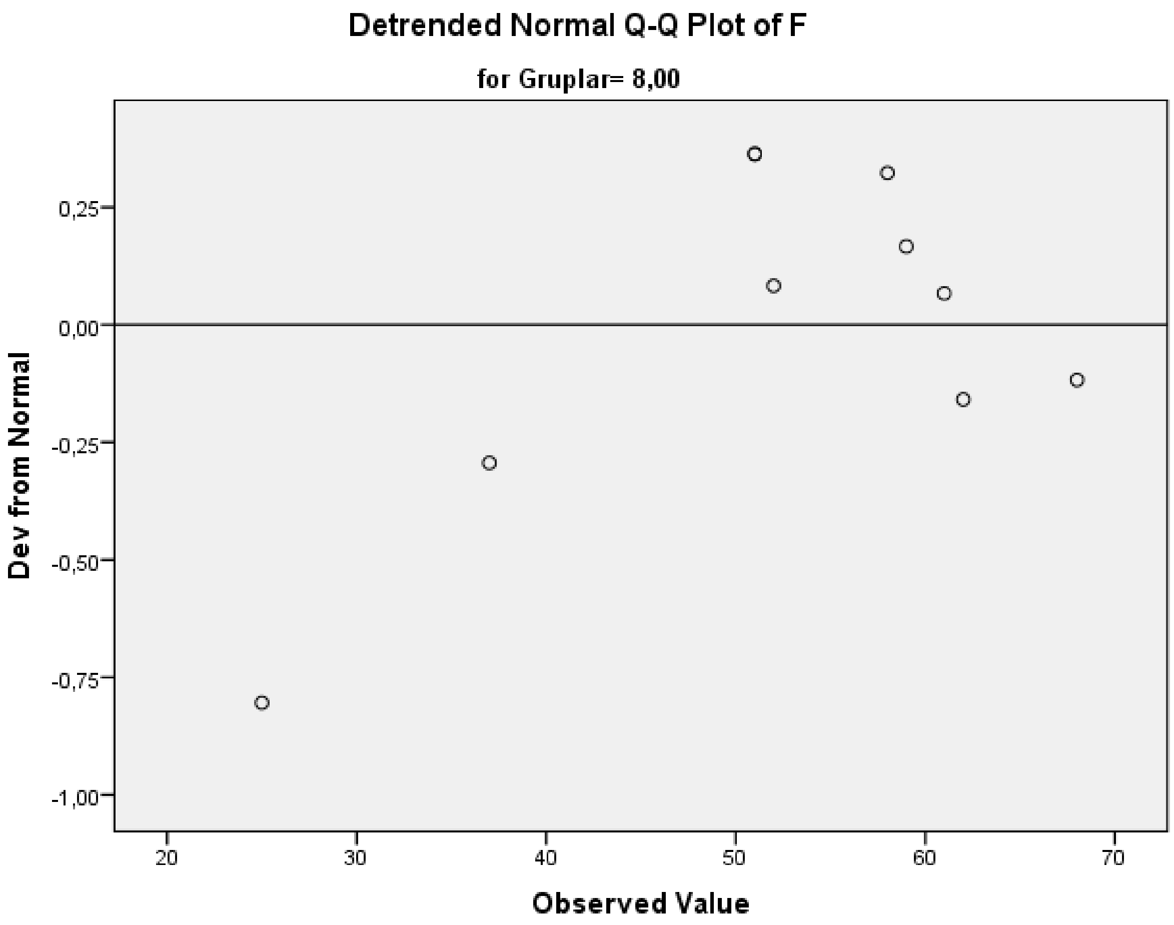
Detrended Normal Q-Q Plot of F
for Gruplar= 8,00
Dev from Normal
0,25
0,00
-0,25
-0,50
-0,75
-1,00
20
30
40
50
60
70
Observed Value

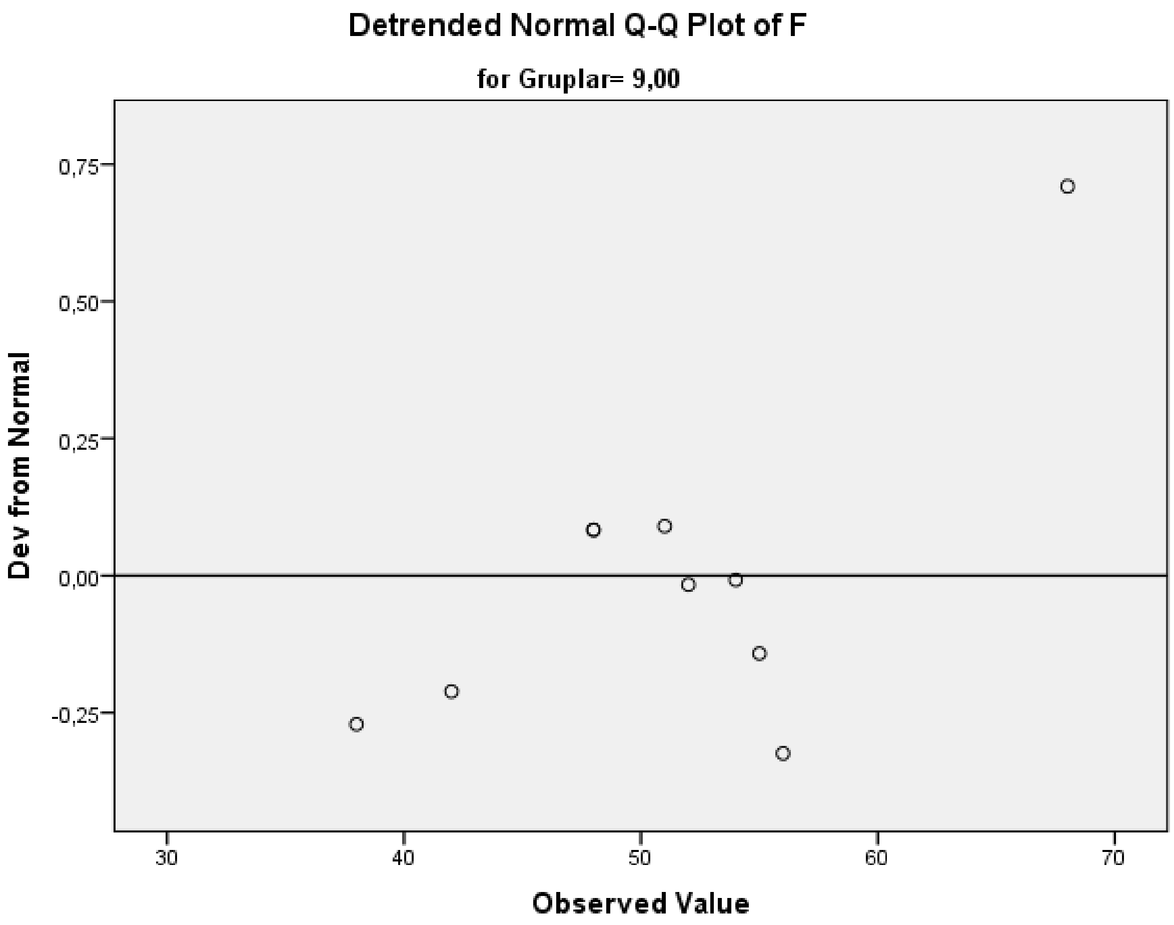
Detrended Normal Q-Q Plot of F
for Gruplar= 9,00
Dev from Normal
0,75
0,50
0,25
0,00
-0,25
30
40
50
60
70
Observed Value

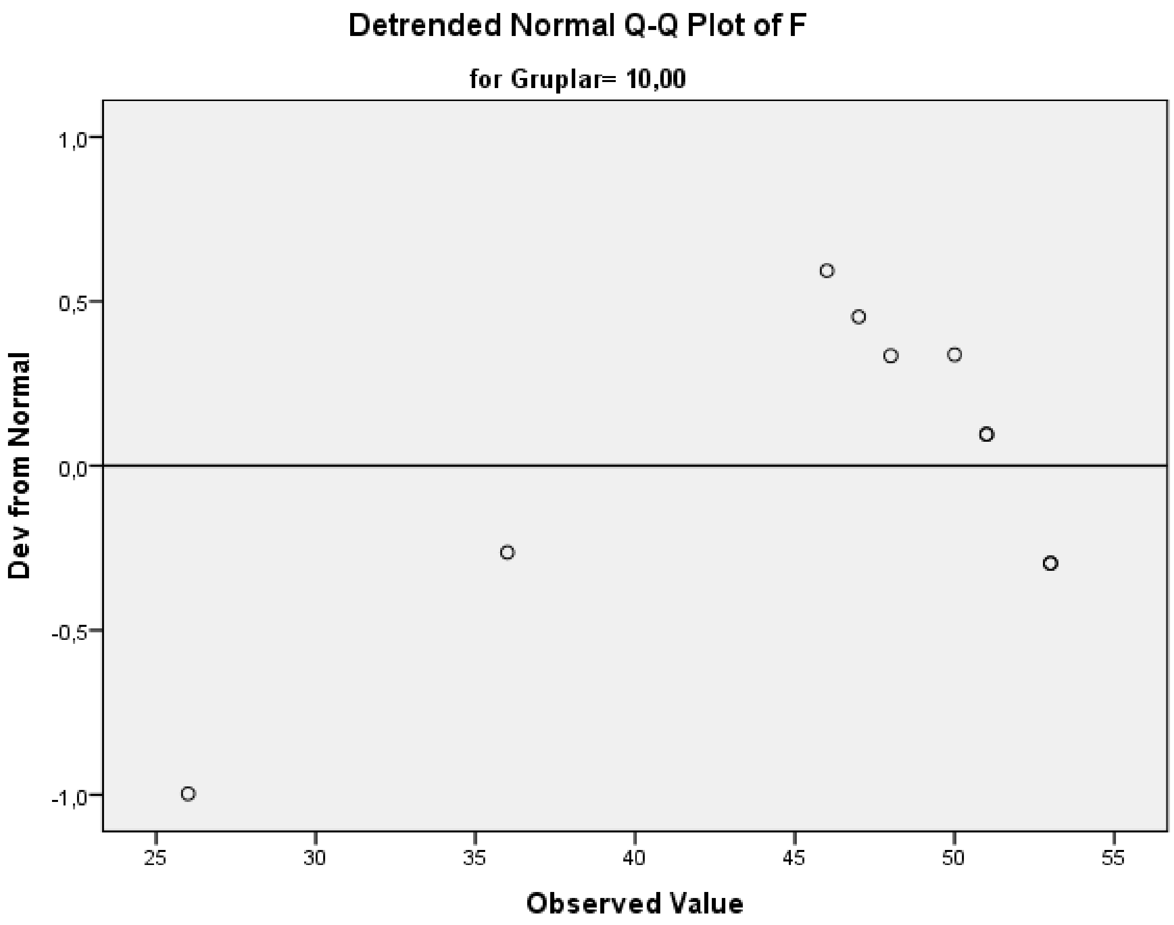

Detrended Normal Q-Q Plot of F
for Gruplar= 10,00
Dev from Normal
1,0
0,5
0,0
-0,5
-1,0
25
30
35
40
45
50
55
Observed Value

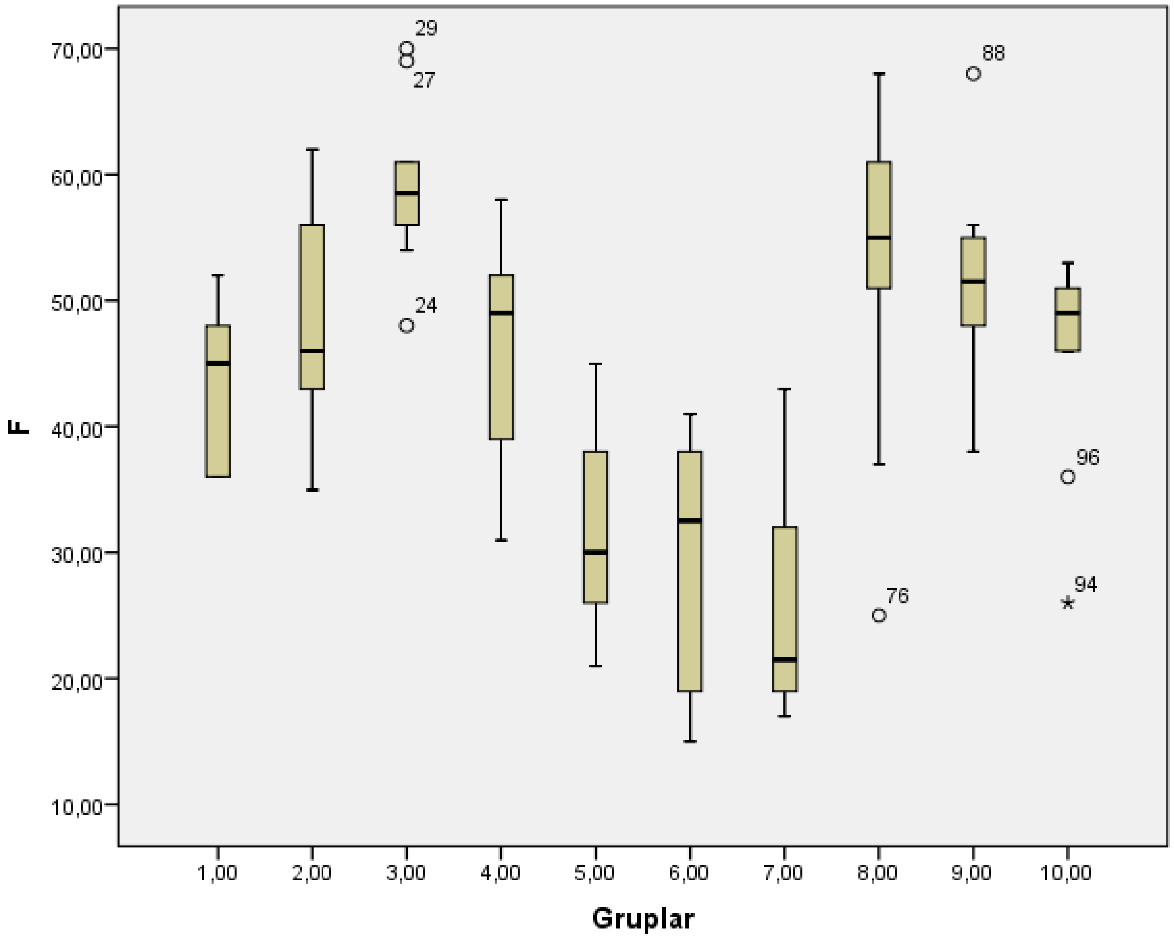


```
ONEWAY F BY Gruplar
  /STATISTICS DESCRIPTIVES HOMOGENEITY
  /PLOT MEANS
  /MISSING ANALYSIS
  /POSTHOC=DUNCAN ALPHA(0.05).
```

Oneway

Notes

Output Created		24-DEC-2020 13:28:46
Comments		
Input	Active Dataset	DataSet0
	Filter	<none>
	Weight	<none>
	Split File	<none>

	N of Rows in Working Data File	100
Missing Value Handling	Definition of Missing	User-defined missing values are treated as missing.
	Cases Used	Statistics for each analysis are based on cases with no missing data for any variable in the analysis.
Syntax		ONEWAY F BY Gruplar<br>/STATISTICS DESCRIPTIVES HOMOGENEITY<br>/PLOT MEANS<br>/MISSING ANALYSIS<br>/POSTHOC=DUNCAN ALPHA(0.05).
Resources	Processor Time	00:00:00,58
	Elapsed Time	00:00:00,63

Descriptives

		N	Mean	Std. Deviation	Std. Error	95% Confidence Interval for Mean	
						Lower Bound	Upper Bound
F	1,00	10	44,0000	6,16441	1,94936	39,5902	48,4098
	2,00	10	48,4000	8,35597	2,64239	42,4225	54,3775
	3,00	10	59,1000	6,59040	2,08407	54,3855	63,8145
	4,00	10	46,8000	8,14862	2,57682	40,9708	52,6292
	5,00	10	31,0000	7,76030	2,45402	25,4486	36,5514
	6,00	10	29,7000	9,47570	2,99648	22,9215	36,4785
	7,00	10	25,3000	8,66731	2,74084	19,0998	31,5002
	8,00	10	52,4000	12,80798	4,05024	43,2377	61,5623
	9,00	10	51,2000	8,21651	2,59829	45,3223	57,0777
	10,00	10	46,1000	8,62103	2,72621	39,9329	52,2671
	Total	100	43,4000	13,39154	1,33915	40,7428	46,0572

Descriptives

		Minimum	Maximum
F	1,00	36,00	52,00
	2,00	35,00	62,00
	3,00	48,00	70,00
	4,00	31,00	58,00
	5,00	21,00	45,00

	6,00	15,00	41,00
	7,00	17,00	43,00
	8,00	25,00	68,00
	9,00	38,00	68,00
	10,00	26,00	53,00
	Total	15,00	70,00

Test of Homogeneity of Variances

	Levene Statistic	df1	df2	Sig.
F	,683	9	90	,722

ANOVA

		Sum of Squares	df	Mean Square	F	Sig.
F	Between Groups	11016,000	9	1224,000	16,349	,000
	Within Groups	6738,000	90	74,867		
	Total	17754,000	99			

Post Hoc Tests

Homogeneous Subsets

F

Duncan[a]

Gruplar	N	Subset for alpha = 0.05		
		1	2	3
7,00	10	25,3000		
6,00	10	29,7000		
5,00	10	31,0000		
1,00	10		44,0000	
10,00	10		46,1000	

4,00	10		46,8000	
2,00	10		48,4000	
9,00	10		51,2000	51,2000
8,00	10		52,4000	52,4000
3,00	10			59,1000
Sig.		,169	,059	,056

Means for groups in homogeneous subsets are displayed.

a. Uses Harmonic Mean Sample Size = 10,000.

Means Plots

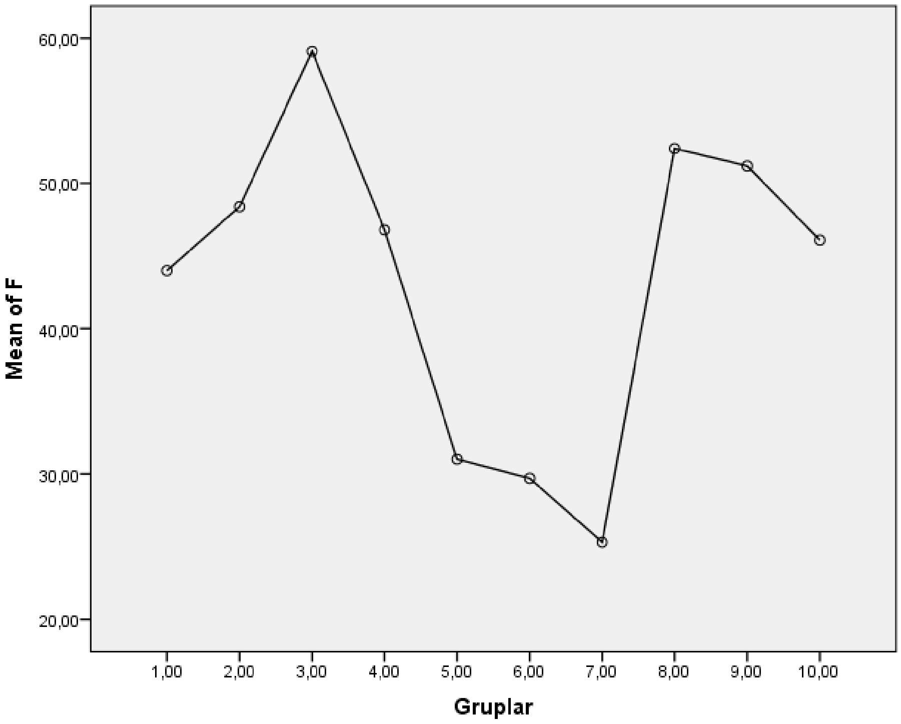

HOST positive rates-ANOVA

HOSTpoz: HOST positive

Groups

1: Control

2: RES10

3: RES20

4: RES40

5: CD10

6: CD20

7: CD40

8: RLC10

9: RLC20

10: RLC40

Explore

Notes

Output Created		24-DEC-2020 14:16:19
Comments		
Input	Active Dataset	DataSet0
	Filter	<none>
	Weight	<none>
	Split File	<none>
	N of Rows in Working Data File	200
Missing Value Handling	Definition of Missing	User-defined missing values for dependent variables are treated as missing.
	Cases Used	Statistics are based on cases with no missing values for any dependent variable or factor used.

Syntax		EXAMINE VARIABLES=HOSTpoz BY Gruplar<br>/PLOT BOXPLOT STEMLEAF HISTOGRAM NPPLOT<br>/COMPARE GROUPS<br>/STATISTICS DESCRIPTIVES<br>/CINTERVAL 95<br>/MISSING LISTWISE<br>/NOTOTAL.
Resources	Processor Time	00:00:04,14
	Elapsed Time	00:00:04,14

Gruplar

Case Processing Summary

		Cases					
		Valid		Missing		Total	
	Gruplar	N	Percent	N	Percent	N	Percent
HOSTpoz	1,00	20	100,0%	0	0,0%	20	100,0%
	2,00	20	100,0%	0	0,0%	20	100,0%
	3,00	20	100,0%	0	0,0%	20	100,0%
	4,00	20	100,0%	0	0,0%	20	100,0%
	5,00	20	100,0%	0	0,0%	20	100,0%
	6,00	20	100,0%	0	0,0%	20	100,0%
	7,00	20	100,0%	0	0,0%	20	100,0%
	8,00	20	100,0%	0	0,0%	20	100,0%
	9,00	20	100,0%	0	0,0%	20	100,0%
	10,00	20	100,0%	0	0,0%	20	100,0%

Descriptives

	Gruplar			Statistic	Std. Error
HOSTpoz	1,00	Mean		35,1500	1,03689
		95% Confidence Interval for Mean	Lower Bound	32,9798	
			Upper Bound	37,3202	

	5% Trimmed Mean		35,2222	
	Median		36,0000	
	Variance		21,503	
	Std. Deviation		4,63709	
	Minimum		26,00	
	Maximum		43,00	
	Range		17,00	
	Interquartile Range		7,75	
	Skewness		-,391	,512
	Kurtosis		-,476	,992
2,00	Mean		43,8000	1,40974
	95% Confidence Interval for Mean	Lower Bound	40,8494	
		Upper Bound	46,7506	
	5% Trimmed Mean		43,7778	
	Median		42,5000	
	Variance		39,747	
	Std. Deviation		6,30455	
	Minimum		32,00	
	Maximum		56,00	
	Range		24,00	
	Interquartile Range		8,50	
	Skewness		,386	,512
	Kurtosis		-,099	,992
3,00	Mean		44,6500	1,11986
	95% Confidence Interval for Mean	Lower Bound	42,3061	
		Upper Bound	46,9939	
	5% Trimmed Mean		44,6667	
	Median		45,0000	
	Variance		25,082	
	Std. Deviation		5,00815	
	Minimum		36,00	
	Maximum		53,00	
	Range		17,00	
	Interquartile Range		9,75	
	Skewness		,071	,512
	Kurtosis		-1,062	,992
4,00	Mean		37,4500	1,39449
	95% Confidence Interval for	Lower Bound	34,5313	

	Mean	Upper Bound	40,3687	
	5% Trimmed Mean		37,5556	
	Median		38,0000	
	Variance		38,892	
	Std. Deviation		6,23635	
	Minimum		24,00	
	Maximum		49,00	
	Range		25,00	
	Interquartile Range		6,00	
	Skewness		-,043	,512
	Kurtosis		,425	,992
5,00	Mean		36,0000	1,40300
	95% Confidence Interval for Mean	Lower Bound	33,0635	
		Upper Bound	38,9365	
	5% Trimmed Mean		35,8889	
	Median		37,5000	
	Variance		39,368	
	Std. Deviation		6,27443	
	Minimum		26,00	
	Maximum		48,00	
	Range		22,00	
	Interquartile Range		7,75	
	Skewness		,061	,512
	Kurtosis		-,562	,992
6,00	Mean		33,6000	1,38488
	95% Confidence Interval for Mean	Lower Bound	30,7014	
		Upper Bound	36,4986	
	5% Trimmed Mean		33,5556	
	Median		32,0000	
	Variance		38,358	
	Std. Deviation		6,19338	
	Minimum		24,00	
	Maximum		44,00	
	Range		20,00	
	Interquartile Range		11,00	
	Skewness		,298	,512
	Kurtosis		-,916	,992
7,00	Mean		33,6000	1,75978

	95% Confidence Interval for Mean	Lower Bound	29,9167	
		Upper Bound	37,2833	
	5% Trimmed Mean		33,2778	
	Median		34,5000	
	Variance		61,937	
	Std. Deviation		7,87000	
	Minimum		23,00	
	Maximum		50,00	
	Range		27,00	
	Interquartile Range		9,75	
	Skewness		,651	,512
	Kurtosis		,003	,992
8,00	Mean		41,4000	1,53623
	95% Confidence Interval for Mean	Lower Bound	38,1846	
		Upper Bound	44,6154	
	5% Trimmed Mean		41,1667	
	Median		40,5000	
	Variance		47,200	
	Std. Deviation		6,87023	
	Minimum		32,00	
	Maximum		55,00	
	Range		23,00	
	Interquartile Range		12,50	
	Skewness		,311	,512
	Kurtosis		-,797	,992
9,00	Mean		43,2500	1,23518
	95% Confidence Interval for Mean	Lower Bound	40,6647	
		Upper Bound	45,8353	
	5% Trimmed Mean		43,3889	
	Median		43,0000	
	Variance		30,513	
	Std. Deviation		5,52387	
	Minimum		32,00	
	Maximum		52,00	
	Range		20,00	
	Interquartile Range		6,00	
	Skewness		-,264	,512
	Kurtosis		-,321	,992

	10,00	Mean		42,9500	1,25126
		95% Confidence Interval for Mean	Lower Bound	40,3311	
			Upper Bound	45,5689	
		5% Trimmed Mean		42,9444	
		Median		42,5000	
		Variance		31,313	
		Std. Deviation		5,59582	
		Minimum		31,00	
		Maximum		55,00	
		Range		24,00	
		Interquartile Range		6,00	
		Skewness		,274	,512
		Kurtosis		,638	,992

Tests of Normality

		Kolmogorov-Smirnov[a]			Shapiro-Wilk		
	Gruplar	Statistic	df	Sig.	Statistic	df	Sig.
HOSTpoz	1,00	,123	20	,200*	,960	20	,543
	2,00	,112	20	,200*	,970	20	,753
	3,00	,123	20	,200*	,957	20	,485
	4,00	,141	20	,200*	,962	20	,593
	5,00	,125	20	,200*	,957	20	,488
	6,00	,152	20	,200*	,934	20	,182
	7,00	,171	20	,130	,925	20	,121
	8,00	,132	20	,200*	,951	20	,376
	9,00	,142	20	,200*	,967	20	,700
	10,00	,151	20	,200*	,958	20	,500

*. This is a lower bound of the true significance.

a. Lilliefors Significance Correction

HOSTpoz

Histograms

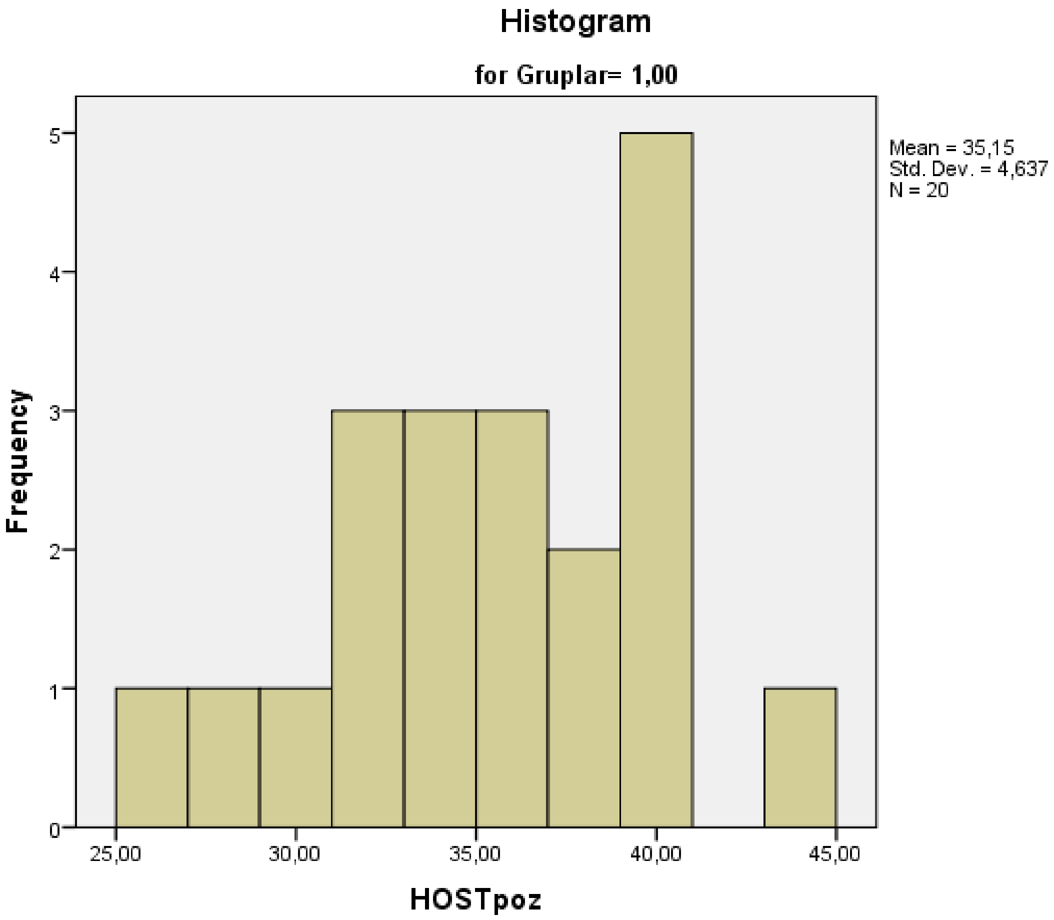

Histogram
for Gruplar= 1,00
Mean = 35,15
Std. Dev. = 4,637
N = 20
Frequency
0
1
2
3
4
5
25,00
30,00
35,00
40,00
45,00
HOSTpoz

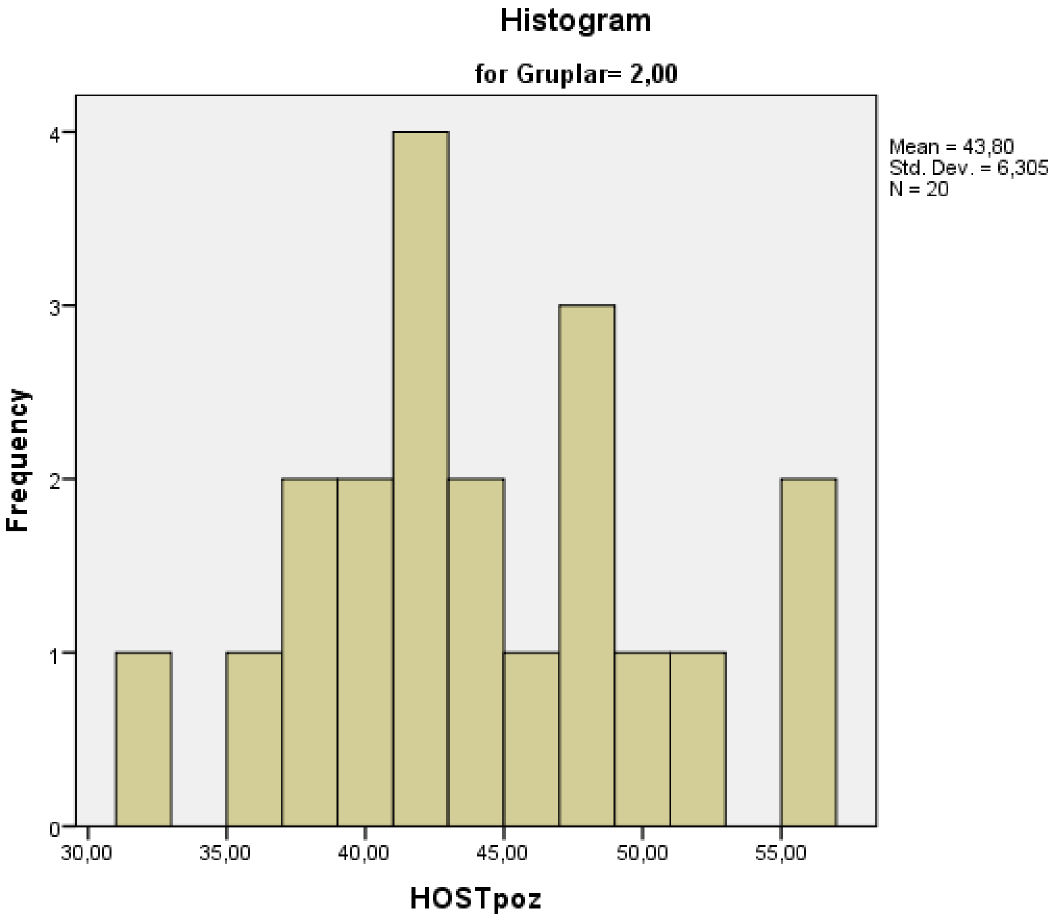

Histogram
for Gruplar= 2,00
Mean = 43,80
Std. Dev. = 6,305
N = 20
Frequency
0
1
2
3
4
30,00
35,00
40,00
45,00
50,00
55,00
HOSTpoz

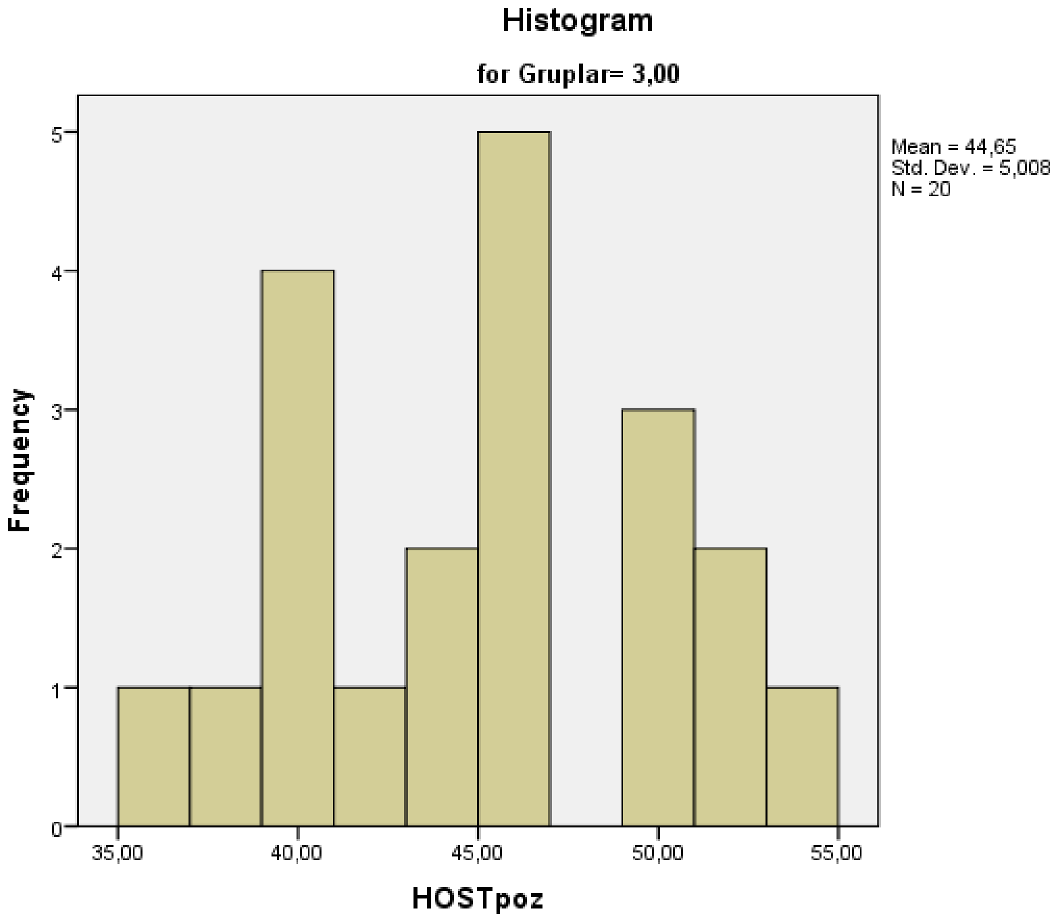
Histogram
for Gruplar= 3,00
Mean = 44,65
Std. Dev. = 5,008
N = 20
Frequency
0
1
2
3
4
5
35,00
40,00
45,00
50,00
55,00
HOSTpoz

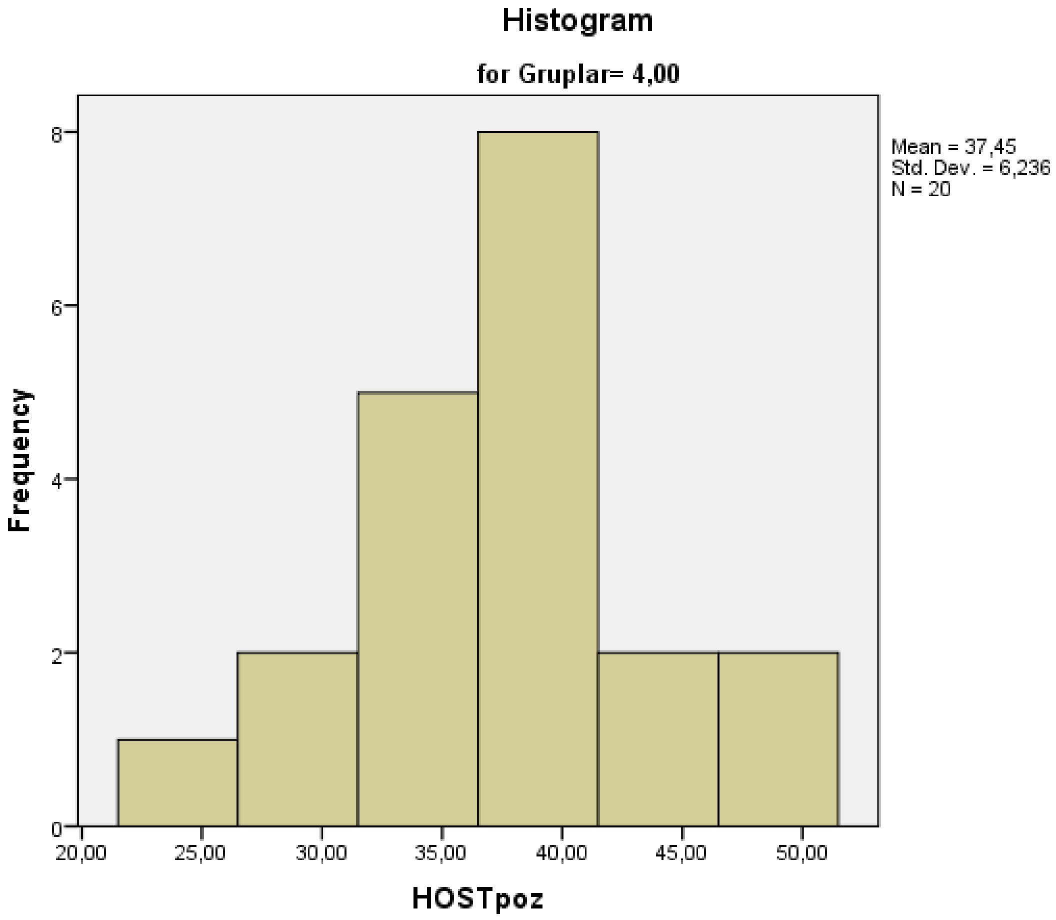
Histogram
for Gruplar= 4,00
Mean = 37,45
Std. Dev. = 6,236
N = 20
Frequency
HOSTpoz
0
2
4
6
8
20,00
25,00
30,00
35,00
40,00
45,00
50,00

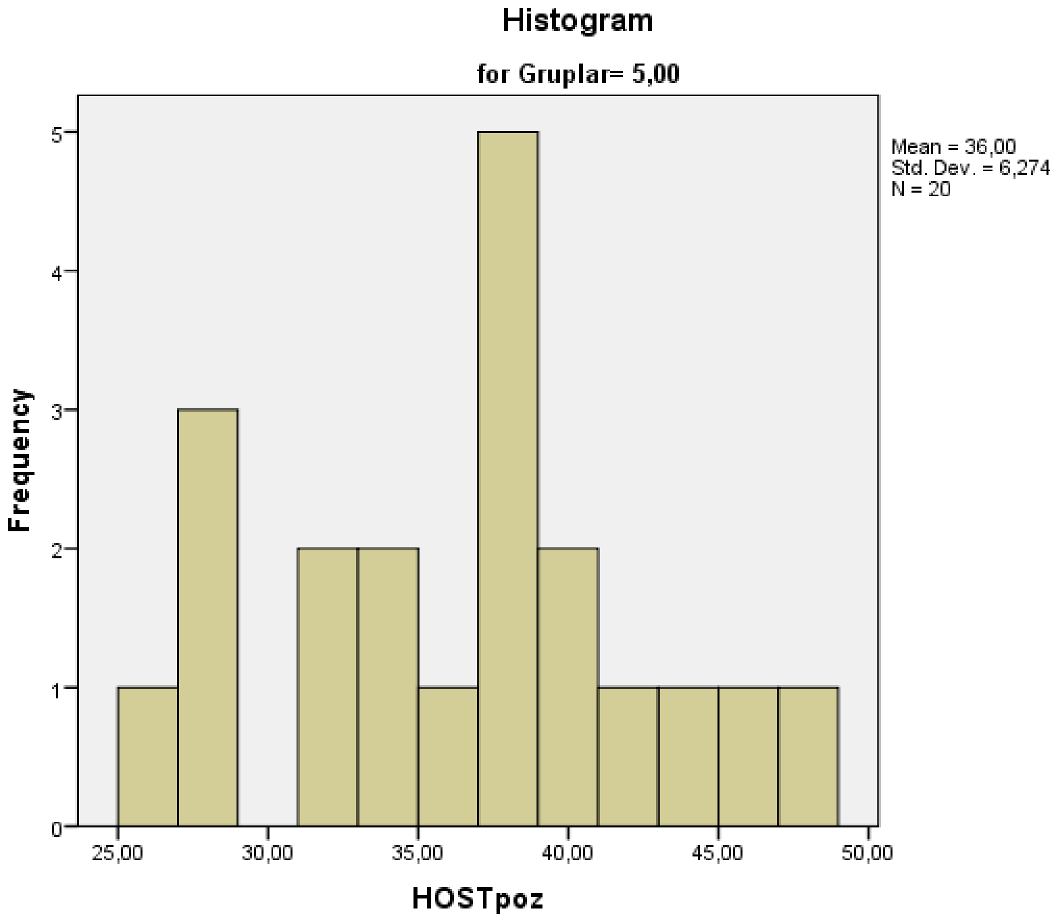
Histogram
for Gruplar= 5,00
Mean = 36,00
Std. Dev. = 6,274
N = 20
Frequency
0
1
2
3
4
5
25,00
30,00
35,00
40,00
45,00
50,00
HOSTpoz

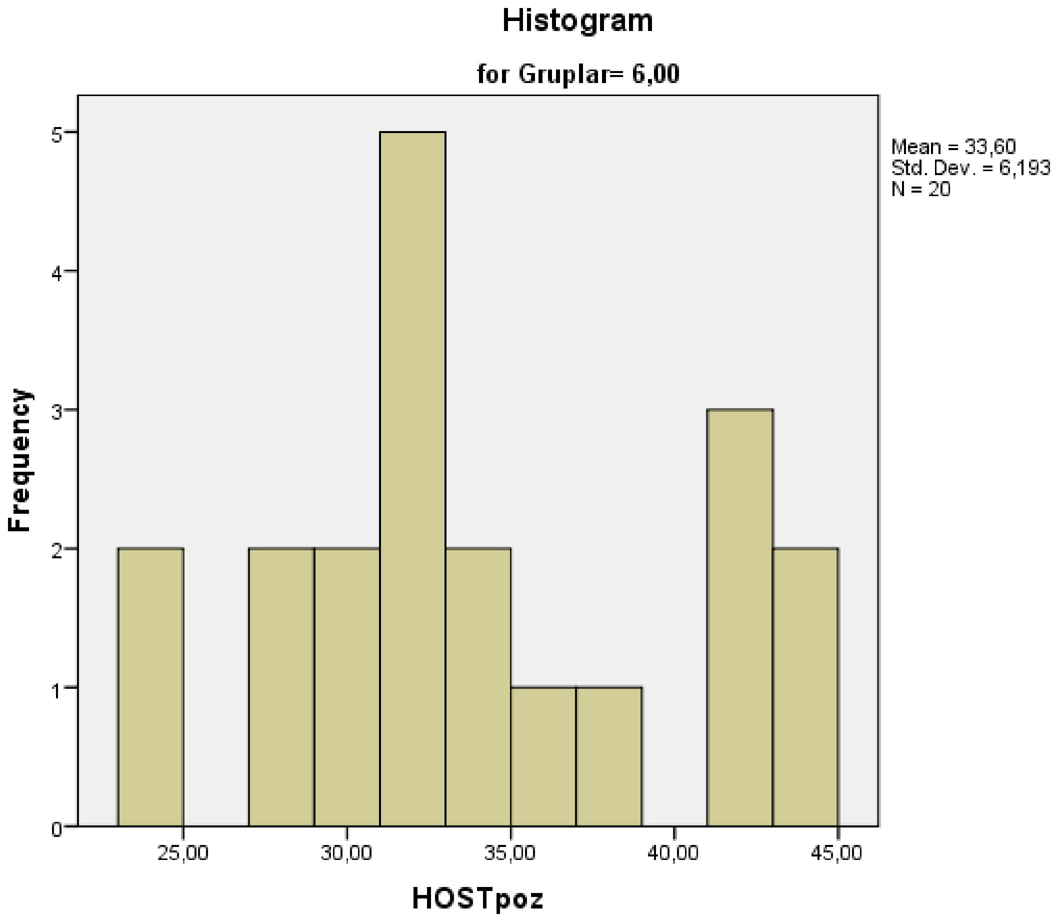

Histogram
for Gruplar= 6,00
Mean = 33,60
Std. Dev. = 6,193
N = 20
Frequency
0
1
2
3
4
5
25,00
30,00
35,00
40,00
45,00
HOSTpoz

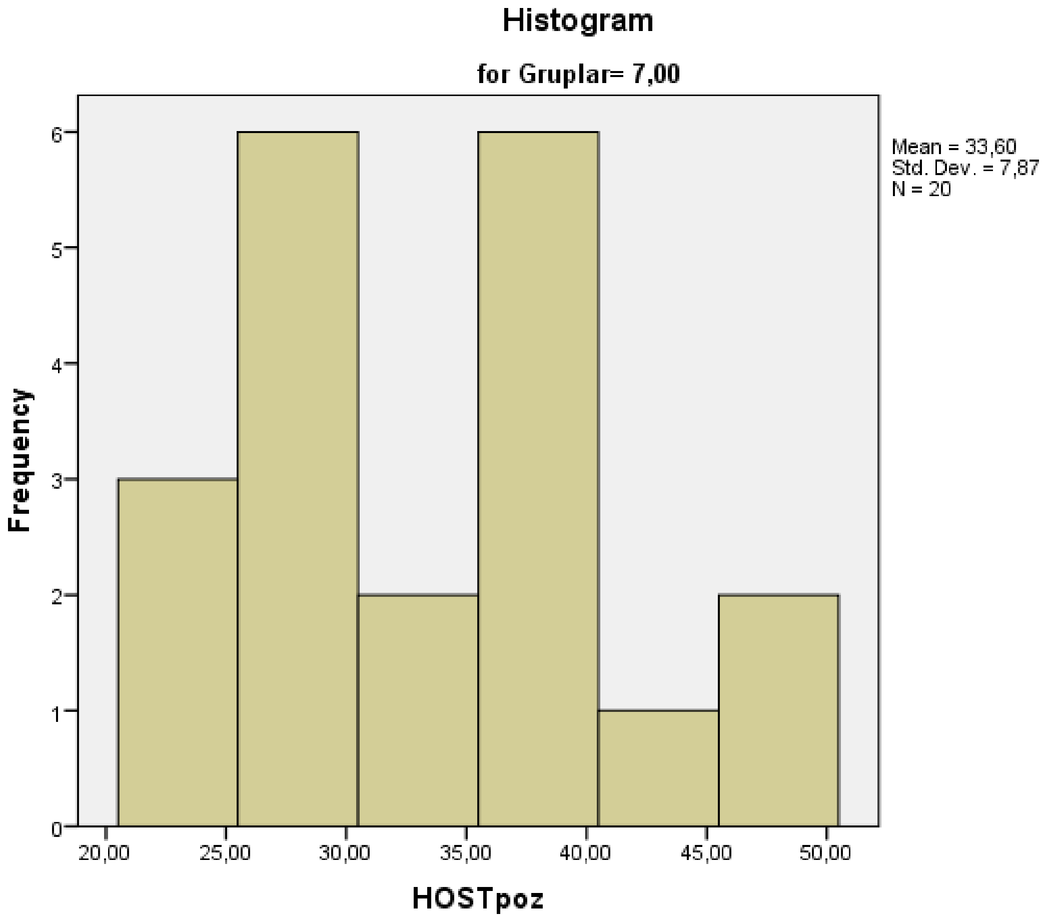
Histogram
for Gruplar= 7,00
Mean = 33,60
Std. Dev. = 7,87
N = 20
Frequency
0
1
2
3
4
5
6
20,00
25,00
30,00
35,00
40,00
45,00
50,00
HOSTpoz

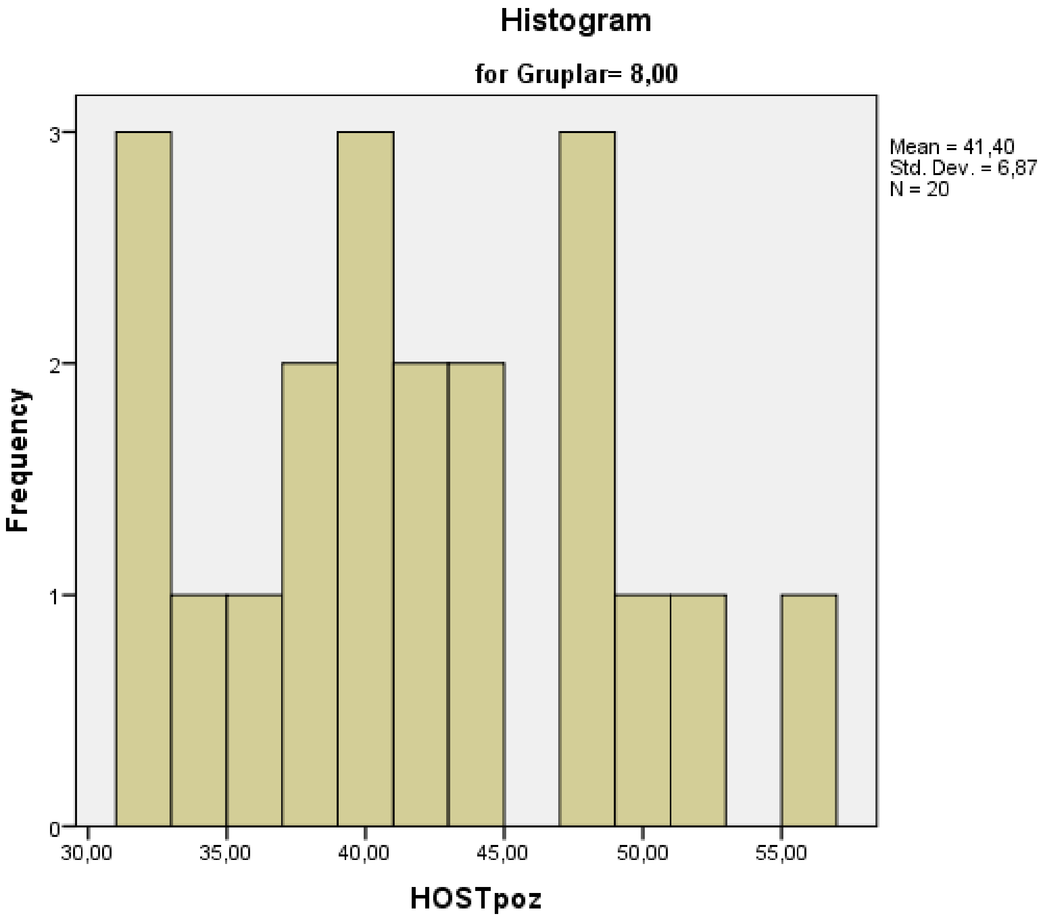

Histogram
for Gruplar= 8,00
Mean = 41,40
Std. Dev. = 6,87
N = 20
Frequency
0
1
2
3
30,00
35,00
40,00
45,00
50,00
55,00
HOSTpoz

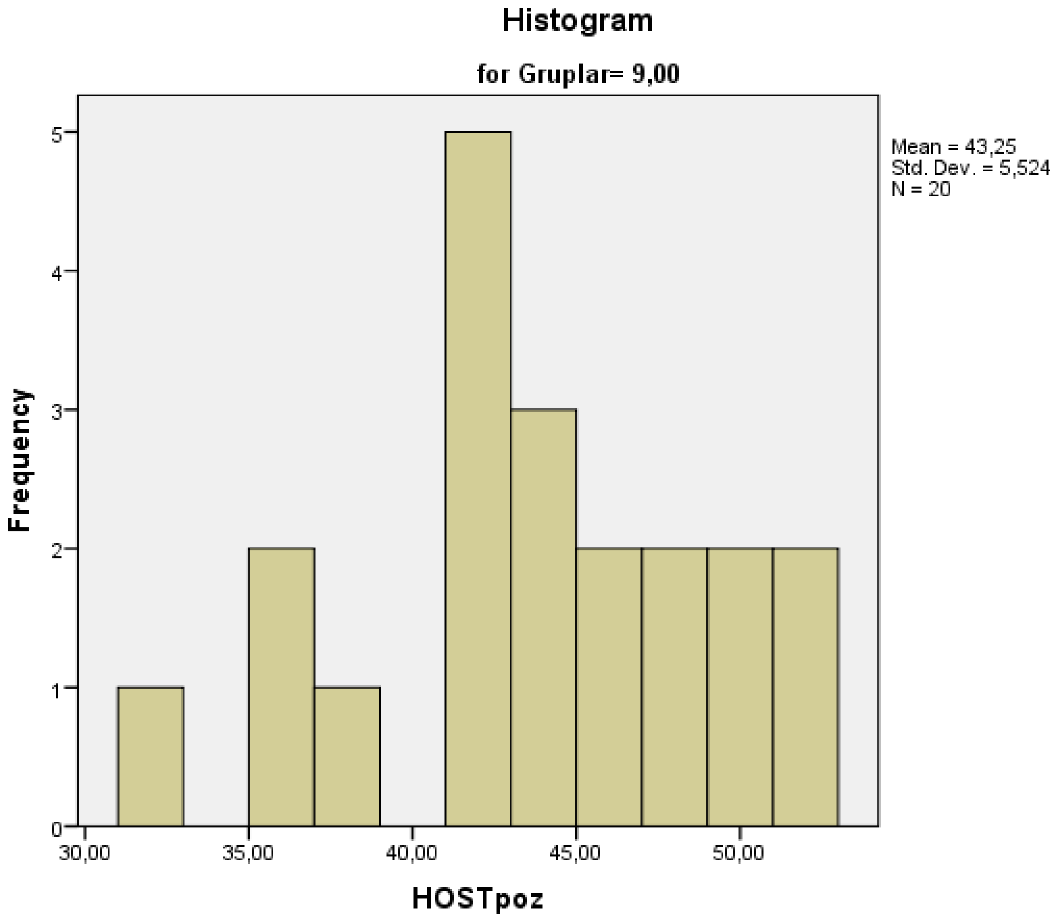
Histogram
for Gruplar= 9,00
Mean = 43,25
Std. Dev. = 5,524
N = 20
Frequency
0
1
2
3
4
5
30,00
35,00
40,00
45,00
50,00
HOSTpoz

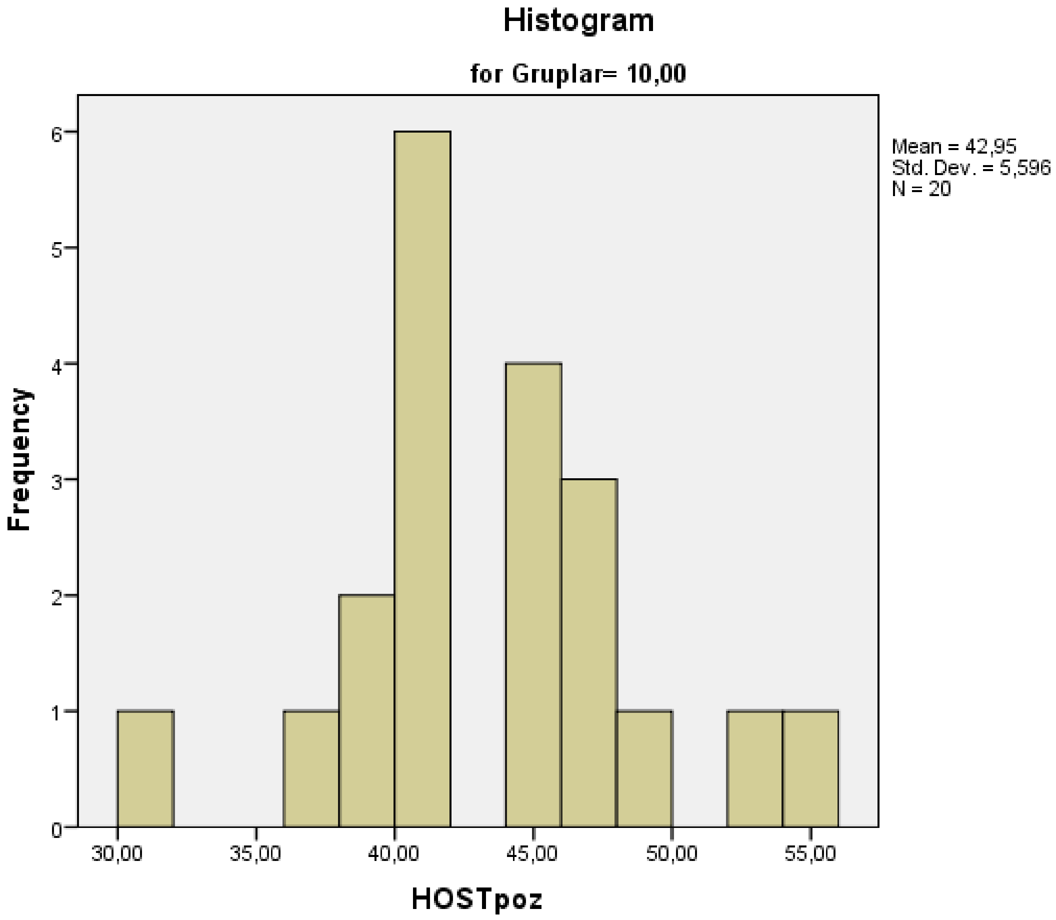

Stem-and-Leaf Plots

```
HOSTpoz Stem-and-Leaf Plot for
Gruplar= 1,00

 Frequency    Stem &  Leaf

     3,00        2 .  679
     6,00        3 .  122444
     6,00        3 .  666779
     5,00        4 .  00003

 Stem width:  10,00
 Each leaf:       1 case(s)
```

```
HOSTpoz Stem-and-Leaf Plot for
Gruplar= 2,00

 Frequency    Stem &  Leaf

     1,00        3 .  2
     4,00        3 .  6889
     7,00        4 .  0112234
     4,00        4 .  5778
     2,00        5 .  01
     2,00        5 .  66

 Stem width:  10,00
 Each leaf:       1 case(s)
```

```
HOSTpoz Stem-and-Leaf Plot for
Gruplar= 3,00

 Frequency    Stem &  Leaf

     3,00        3 .  689
     6,00        4 .  000134
     6,00        4 .  555669
     5,00        5 .  00123

 Stem width:  10,00
 Each leaf:       1 case(s)
```

```
HOSTpoz Stem-and-Leaf Plot for
Gruplar= 4,00

 Frequency    Stem &  Leaf

     1,00 Extremes    (=<24)
     2,00        2 .  99
     3,00        3 .  344
     8,00        3 .  56888899
     3,00        4 .  002
     3,00        4 .  599

 Stem width:  10,00
 Each leaf:       1 case(s)
```

```
HOSTpoz Stem-and-Leaf Plot for
Gruplar= 5,00

 Frequency    Stem &  Leaf
```

```
     4,00        2 .  6778
     4,00        3 .  1234
     8,00        3 .  67888899
     2,00        4 .  14
     2,00        4 .  68

 Stem width:  10,00
 Each leaf:       1 case(s)
```

HOSTpoz Stem-and-Leaf Plot for
Gruplar= 6,00

```
 Frequency    Stem &  Leaf

     2,00        2 .  44
     3,00        2 .  789
     8,00        3 .  01122234
     2,00        3 .  58
     5,00        4 .  12234

 Stem width:  10,00
 Each leaf:       1 case(s)
```

HOSTpoz Stem-and-Leaf Plot for
Gruplar= 7,00

```
 Frequency    Stem &  Leaf

     2,00        2 .  33
     7,00        2 .  5678999
     1,00        3 .  4
     6,00        3 .  566777
     2,00        4 .  01
      ,00        4 .
     2,00        5 .  00

 Stem width:  10,00
 Each leaf:       1 case(s)
```

HOSTpoz Stem-and-Leaf Plot for
Gruplar= 8,00

```
 Frequency    Stem &  Leaf

     4,00        3 .  2224
     4,00        3 .  5789
```

```
     6,00        4 .  001134
     4,00        4 .  8889
     1,00        5 .  2
     1,00        5 .  5

 Stem width:  10,00
 Each leaf:       1 case(s)
```

```
HOSTpoz Stem-and-Leaf Plot for
Gruplar= 9,00

 Frequency    Stem &  Leaf

     1,00        3 .  2
     3,00        3 .  558
     8,00        4 .  11122334
     5,00        4 .  56779
     3,00        5 .  022

 Stem width:  10,00
 Each leaf:       1 case(s)
```

```
HOSTpoz Stem-and-Leaf Plot for
Gruplar= 10,00

 Frequency    Stem &  Leaf

     1,00        3 .  1
     3,00        3 .  699
     9,00        4 .  000001444
     5,00        4 .  56679
     1,00        5 .  3
     1,00        5 .  5

 Stem width:  10,00
 Each leaf:       1 case(s)
```

Normal Q-Q Plots

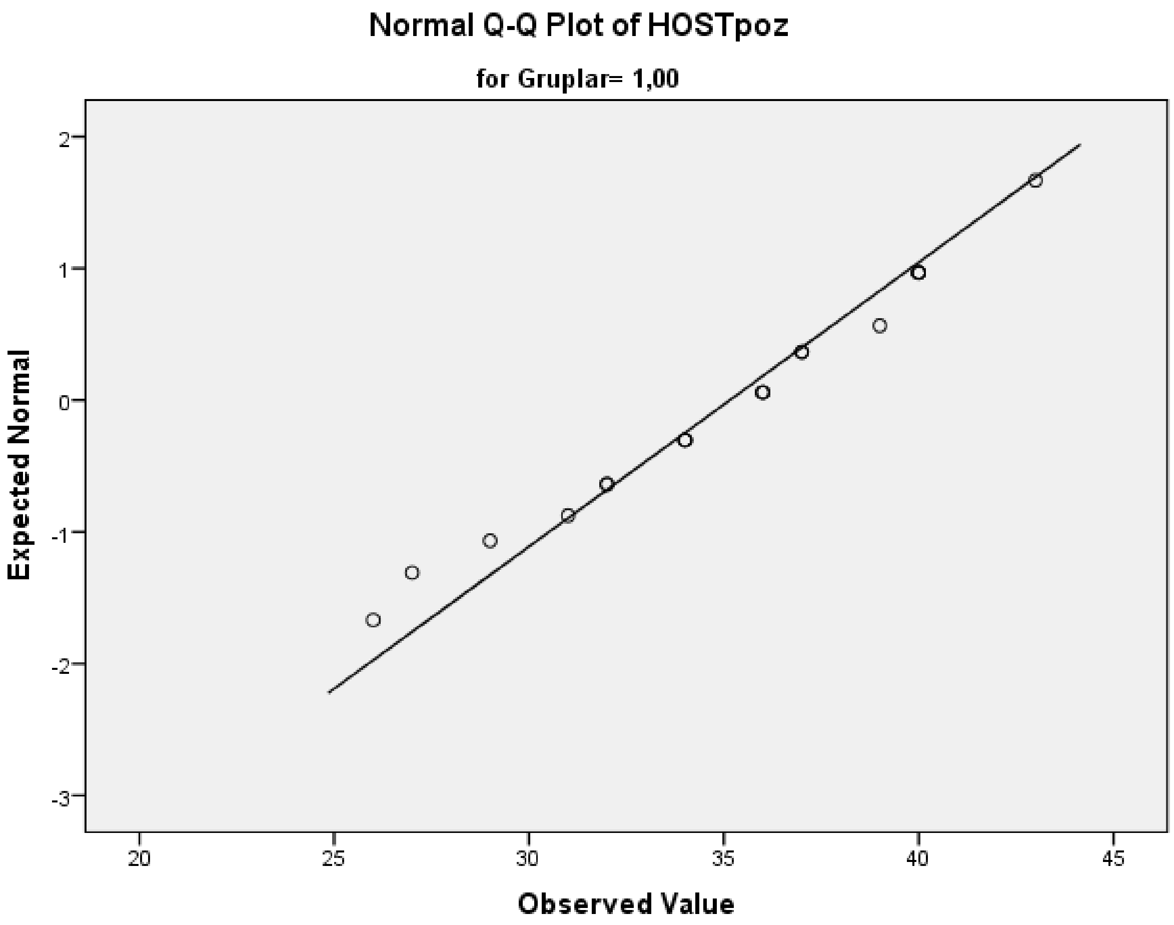
Normal Q-Q Plot of HOSTpoz
for Gruplar= 1,00
Expected Normal
2
1
0
-1
-2
-3
20
25
30
35
40
45
Observed Value

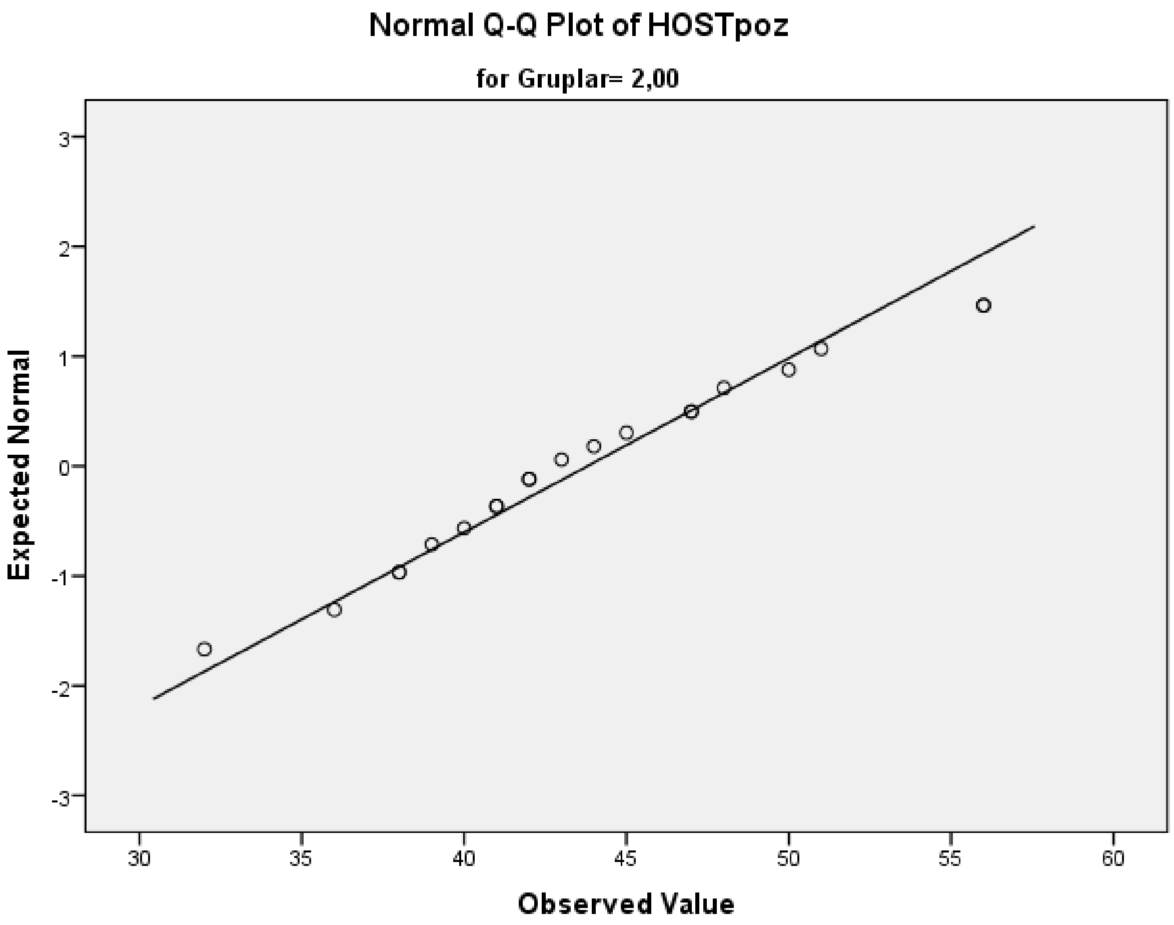
Normal Q-Q Plot of HOSTpoz
for Gruplar= 2,00
Expected Normal
Observed Value
3
2
1
0
-1
-2
-3
30
35
40
45
50
55
60

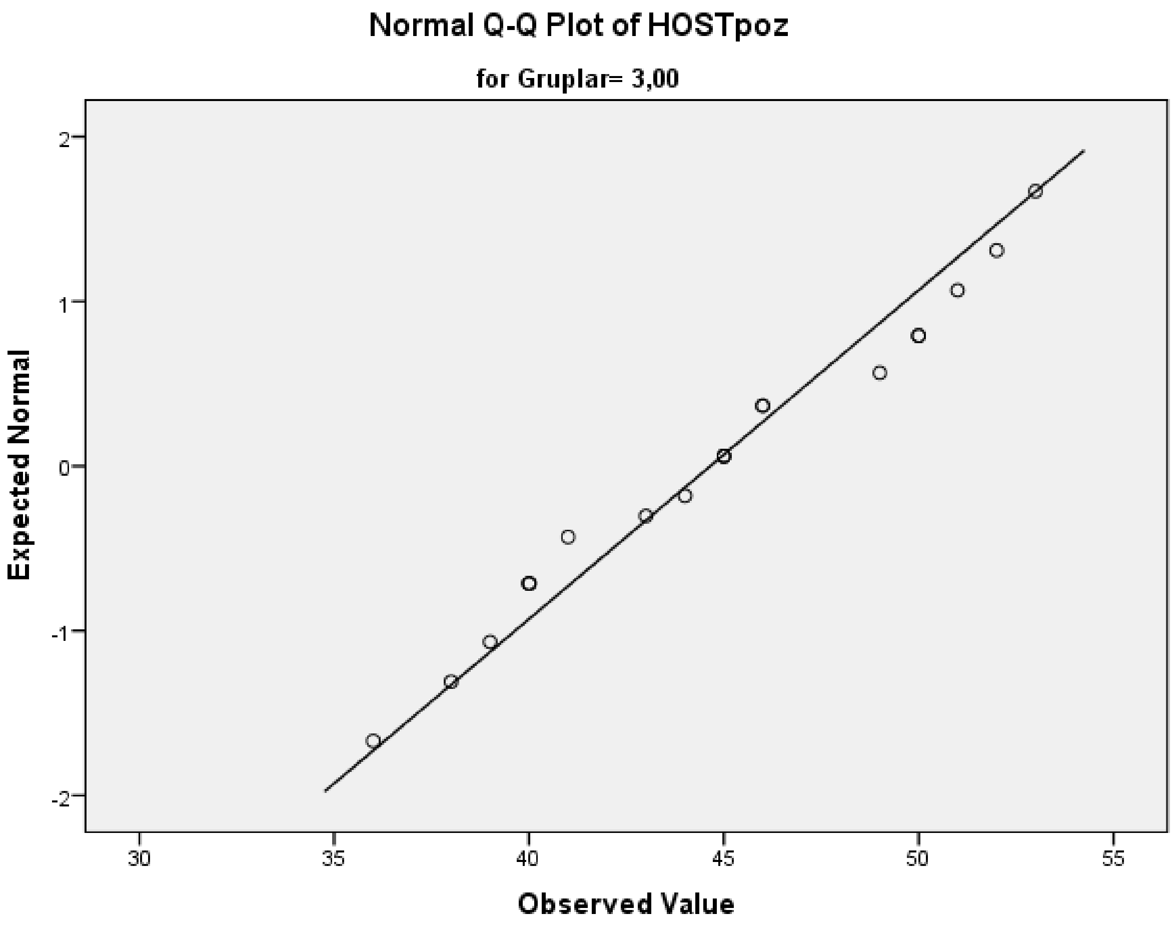
Normal Q-Q Plot of HOSTpoz
for Gruplar= 3,00
Expected Normal
2
1
0
-1
-2
30
35
40
45
50
55
Observed Value

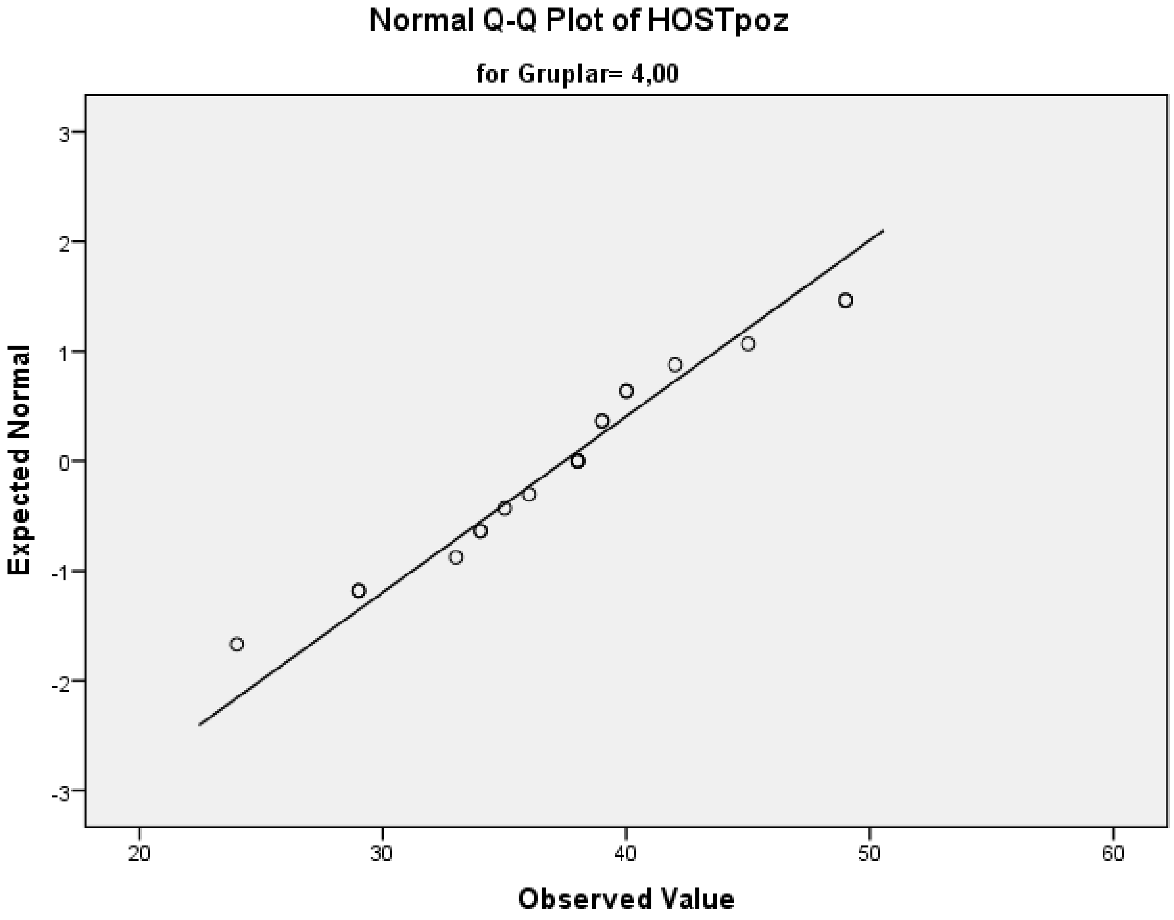
Normal Q-Q Plot of HOSTpoz
for Gruplar= 4,00
Expected Normal
Observed Value
3
2
1
0
-1
-2
-3
20
30
40
50
60

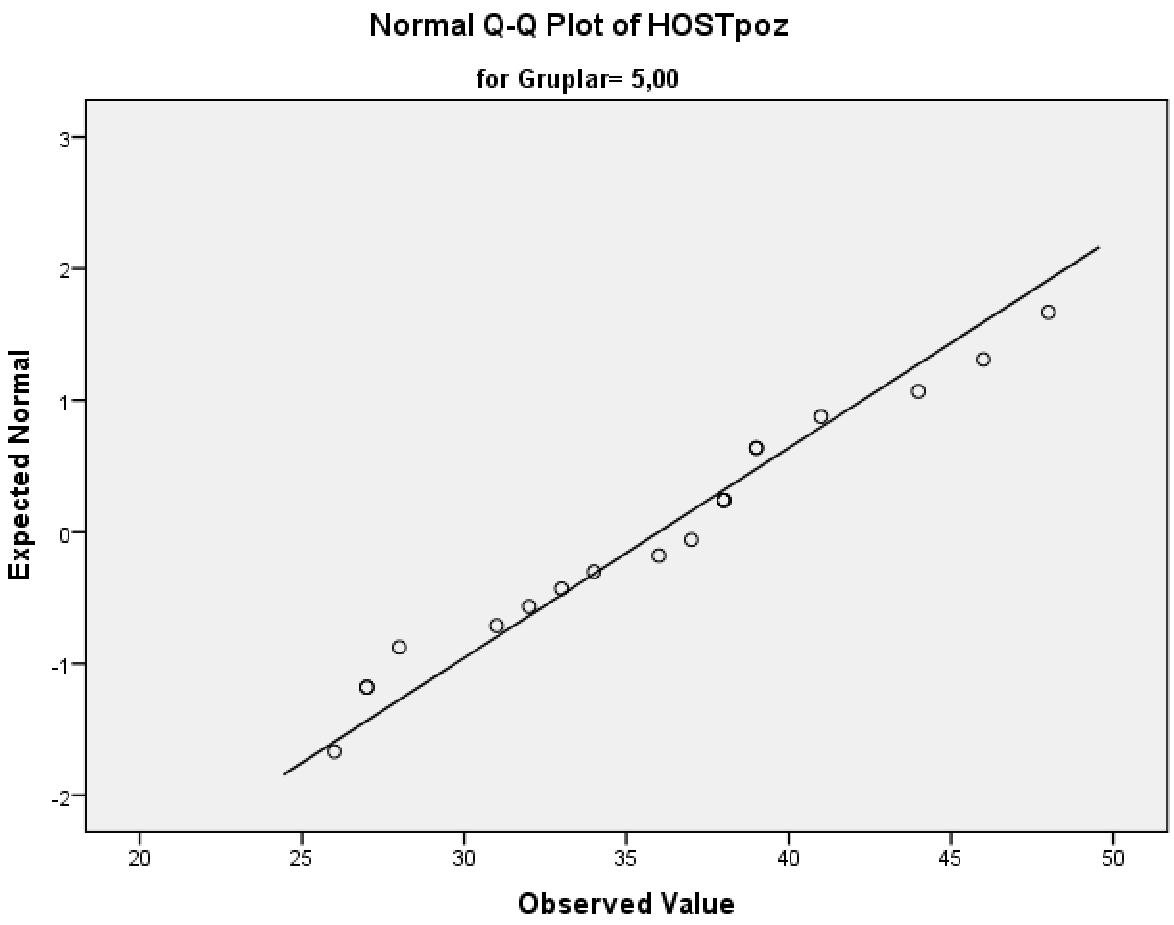

Normal Q-Q Plot of HOSTpoz
for Gruplar= 5,00
Expected Normal
3
2
1
0
-1
-2
20
25
30
35
40
45
50
Observed Value

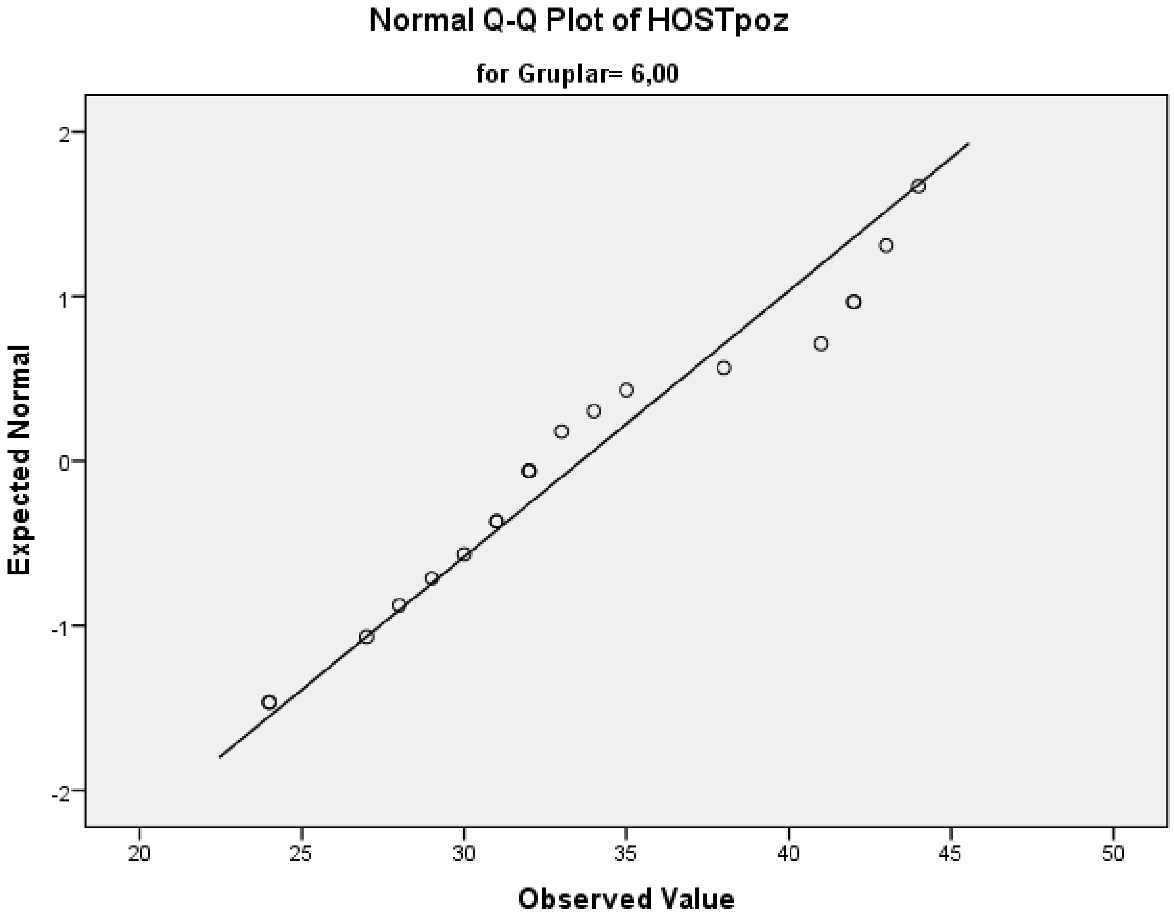
Normal Q-Q Plot of HOSTpoz
for Gruplar= 6,00
Expected Normal
2
1
0
-1
-2
20
25
30
35
40
45
50
Observed Value

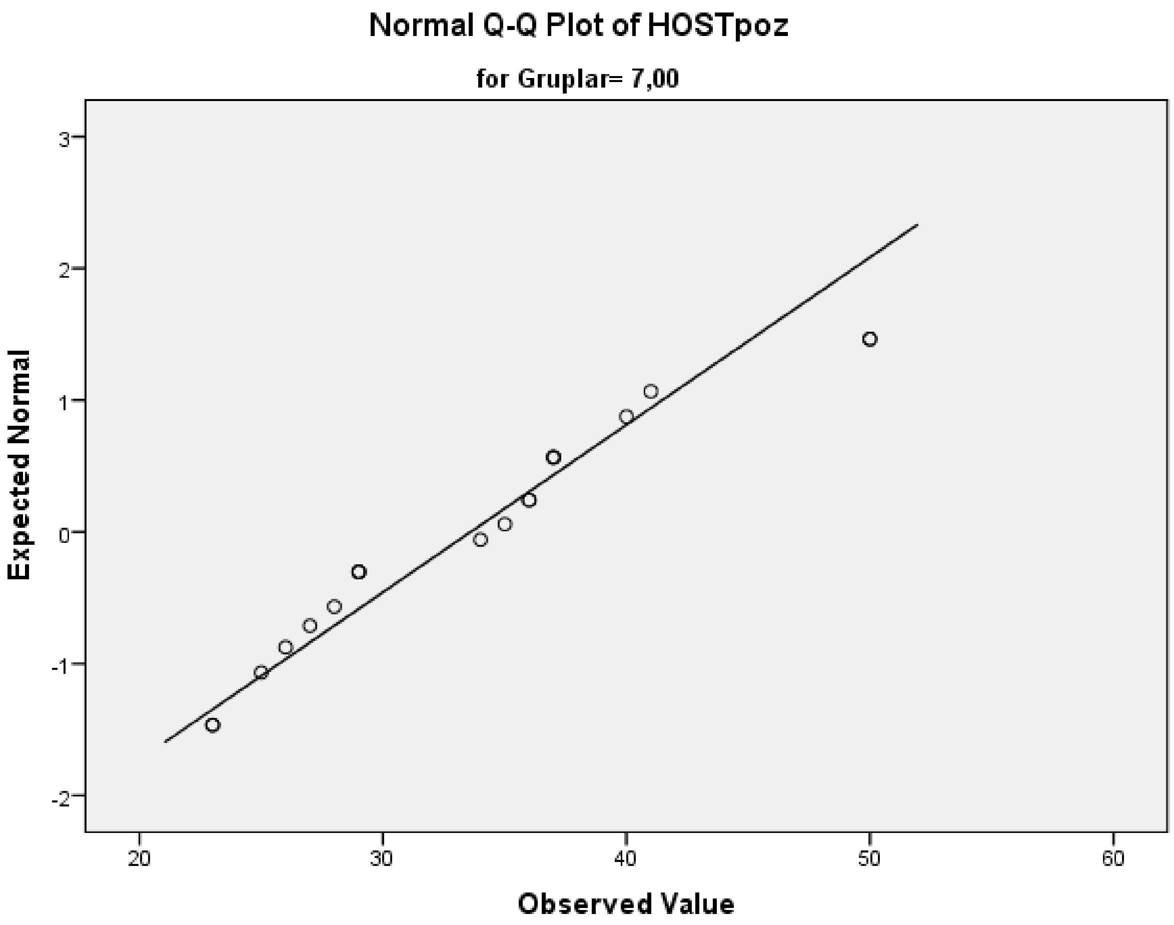
Normal Q-Q Plot of HOSTpoz
for Gruplar= 7,00
Expected Normal
Observed Value
3
2
1
0
-1
-2
20
30
40
50
60

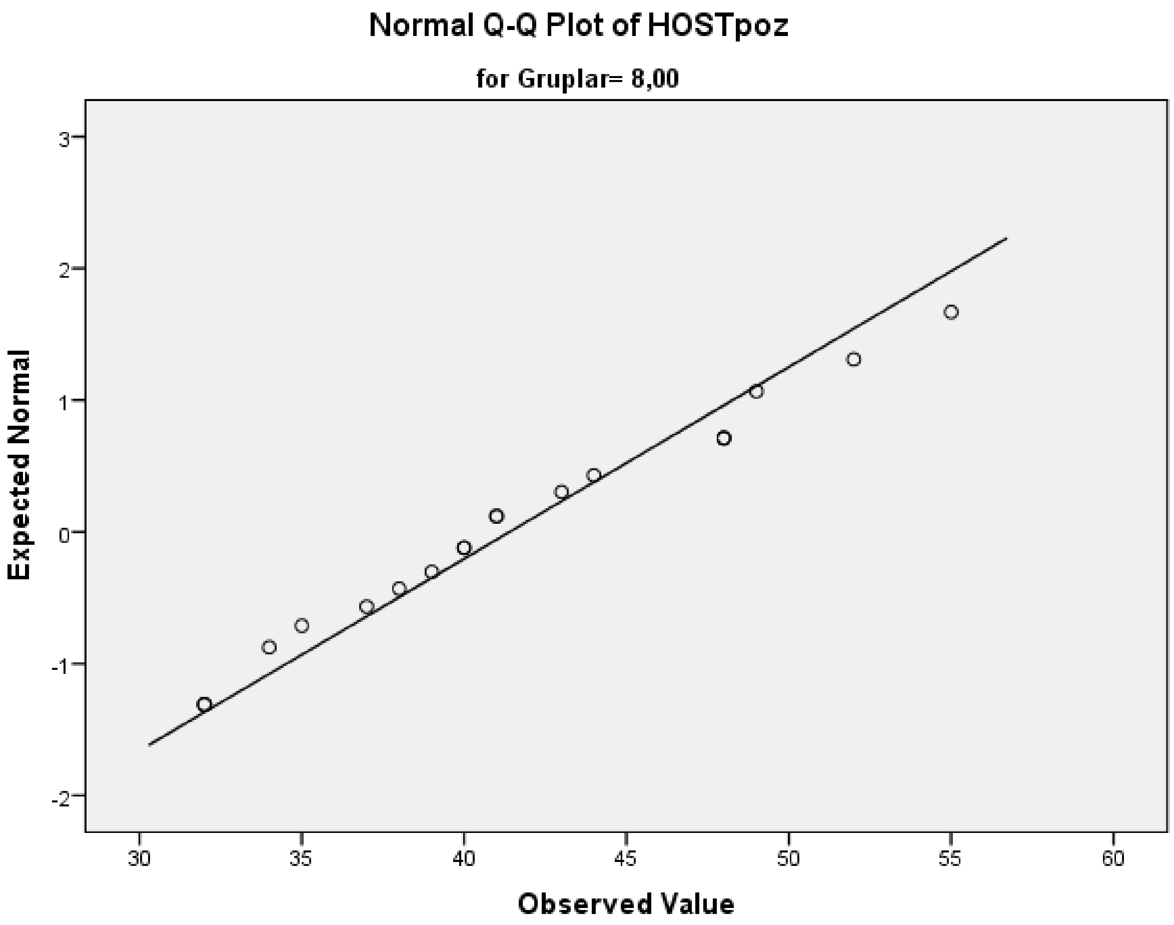

Normal Q-Q Plot of HOSTpoz
for Gruplar= 8,00
Expected Normal
Observed Value
3
2
1
0
-1
-2
30
35
40
45
50
55
60

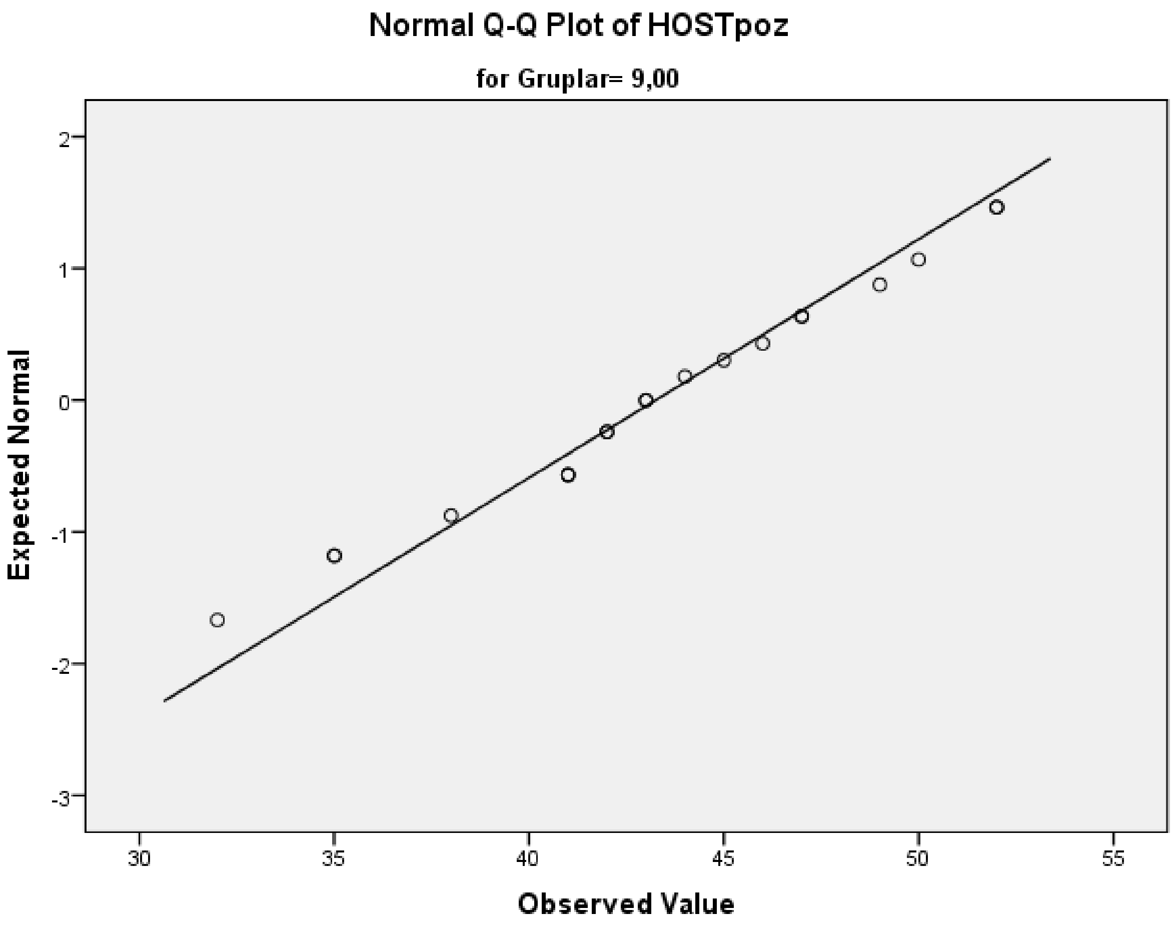

Normal Q-Q Plot of HOSTpoz
for Gruplar= 9,00
Expected Normal
Observed Value
2
1
0
-1
-2
-3
30
35
40
45
50
55

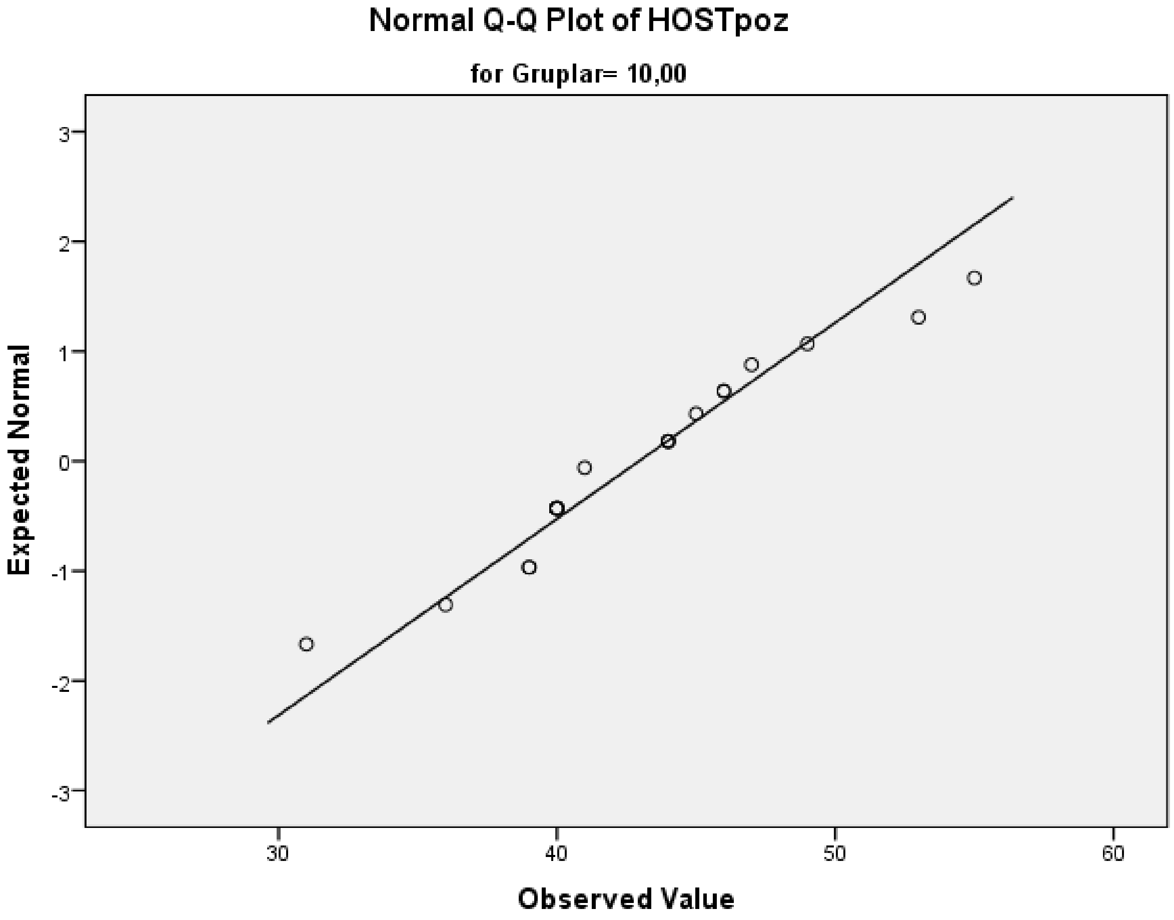

Detrended Normal Q-Q Plots

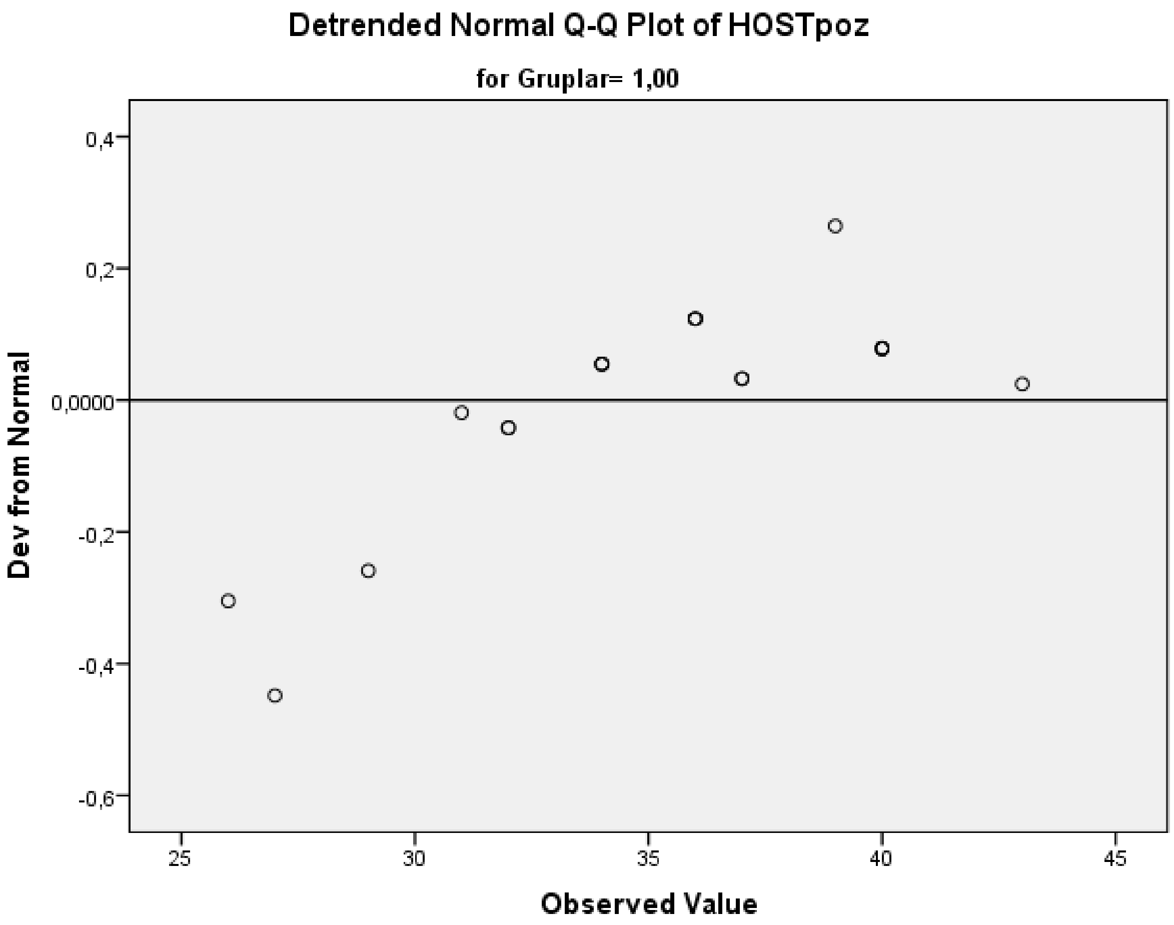
Detrended Normal Q-Q Plot of HOSTpoz
for Gruplar= 1,00
0,4
0,2
0,0000
-0,2
-0,4
-0,6
Dev from Normal
25
30
35
40
45
Observed Value

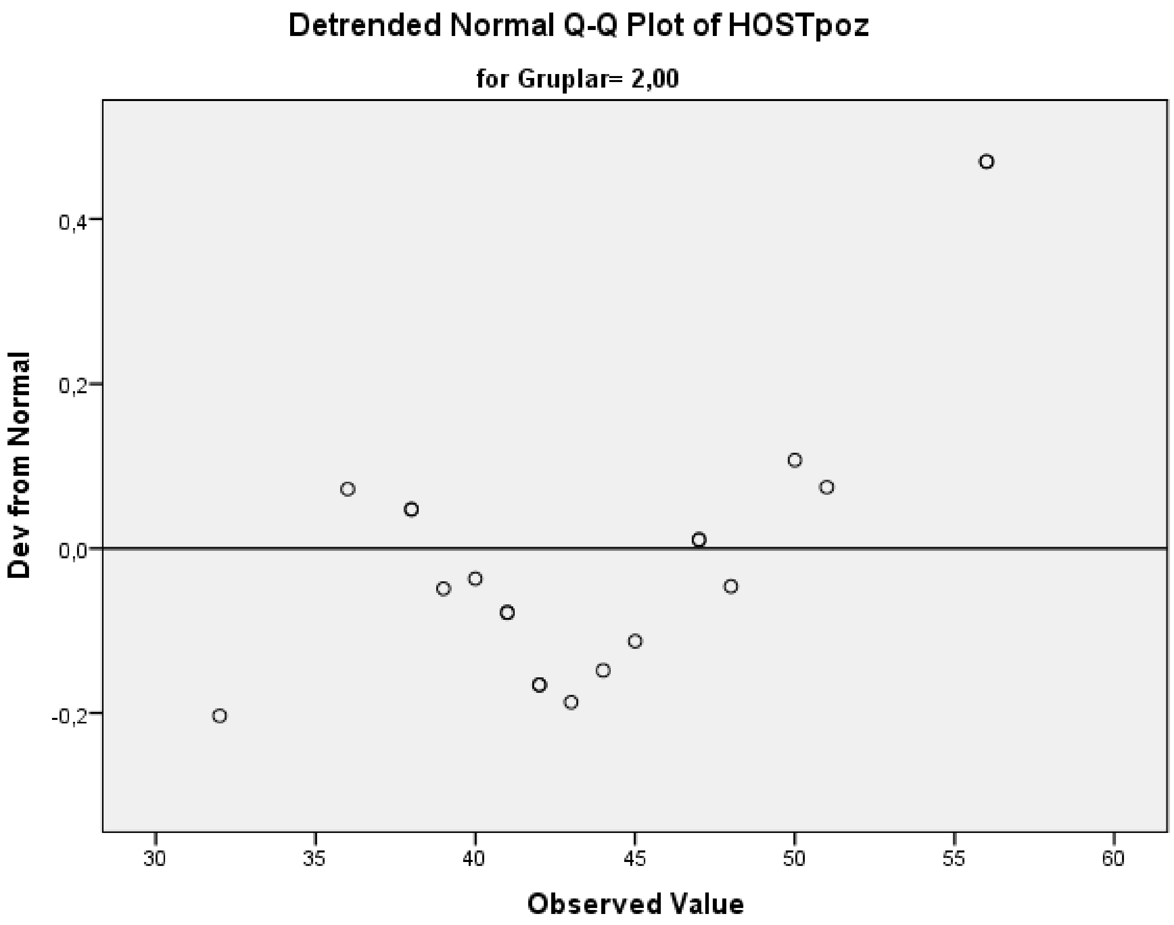

Detrended Normal Q-Q Plot of HOSTpoz
for Gruplar= 2,00
Dev from Normal
0,4
0,2
0,0
-0,2
30
35
40
45
50
55
60
Observed Value

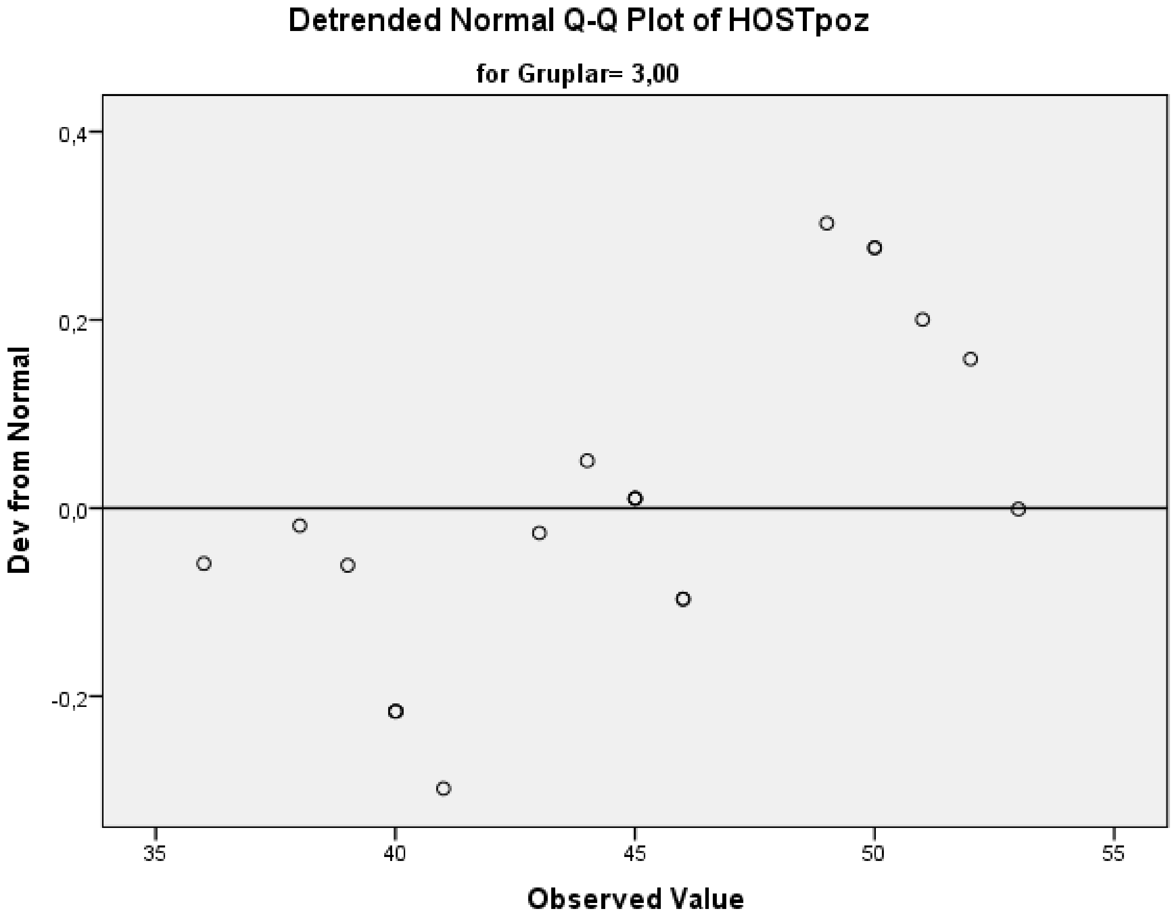

Detrended Normal Q-Q Plot of HOSTpoz
for Gruplar= 3,00
Dev from Normal
0,4
0,2
0,0
-0,2
35
40
45
50
55
Observed Value

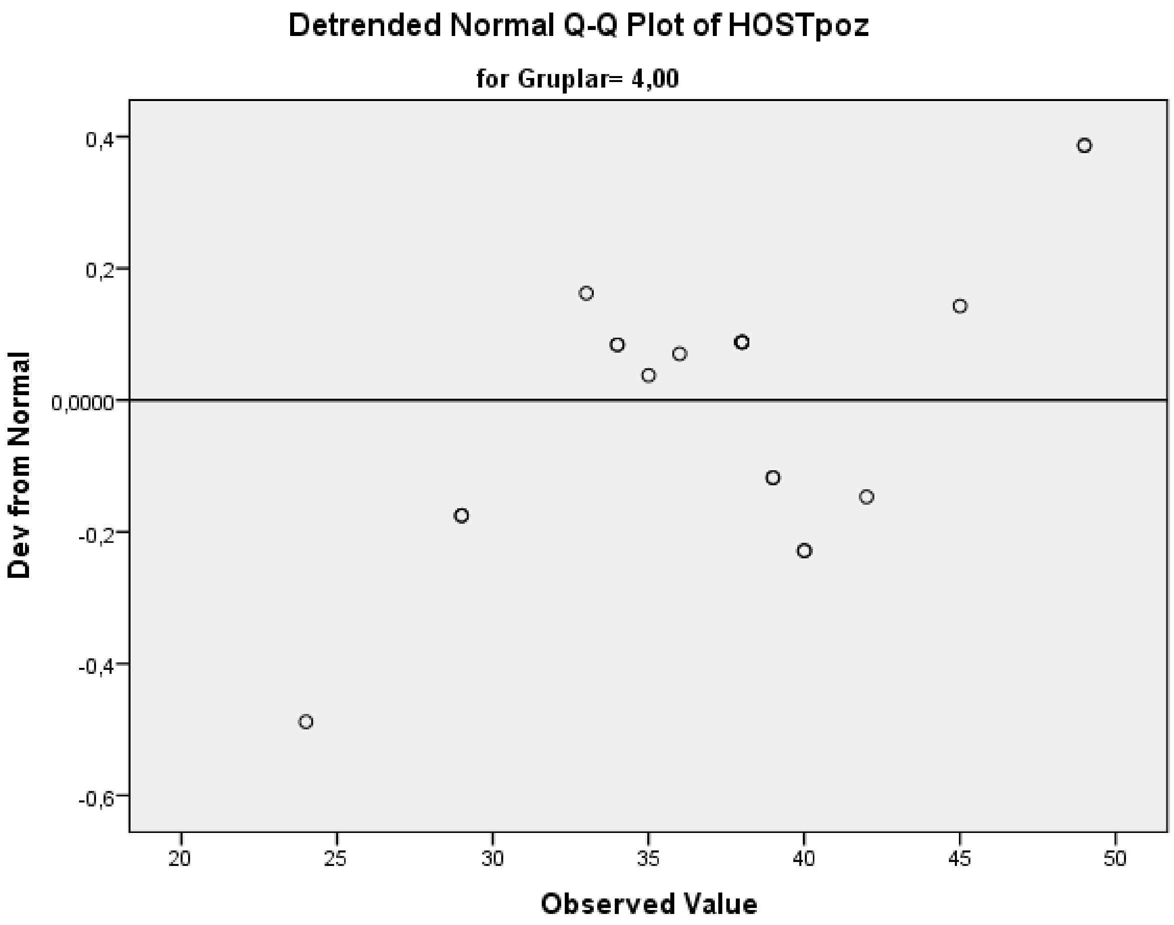
Detrended Normal Q-Q Plot of HOSTpoz
for Gruplar= 4,00
Dev from Normal
0,4
0,2
0,0000
-0,2
-0,4
-0,6
20
25
30
35
40
45
50
Observed Value

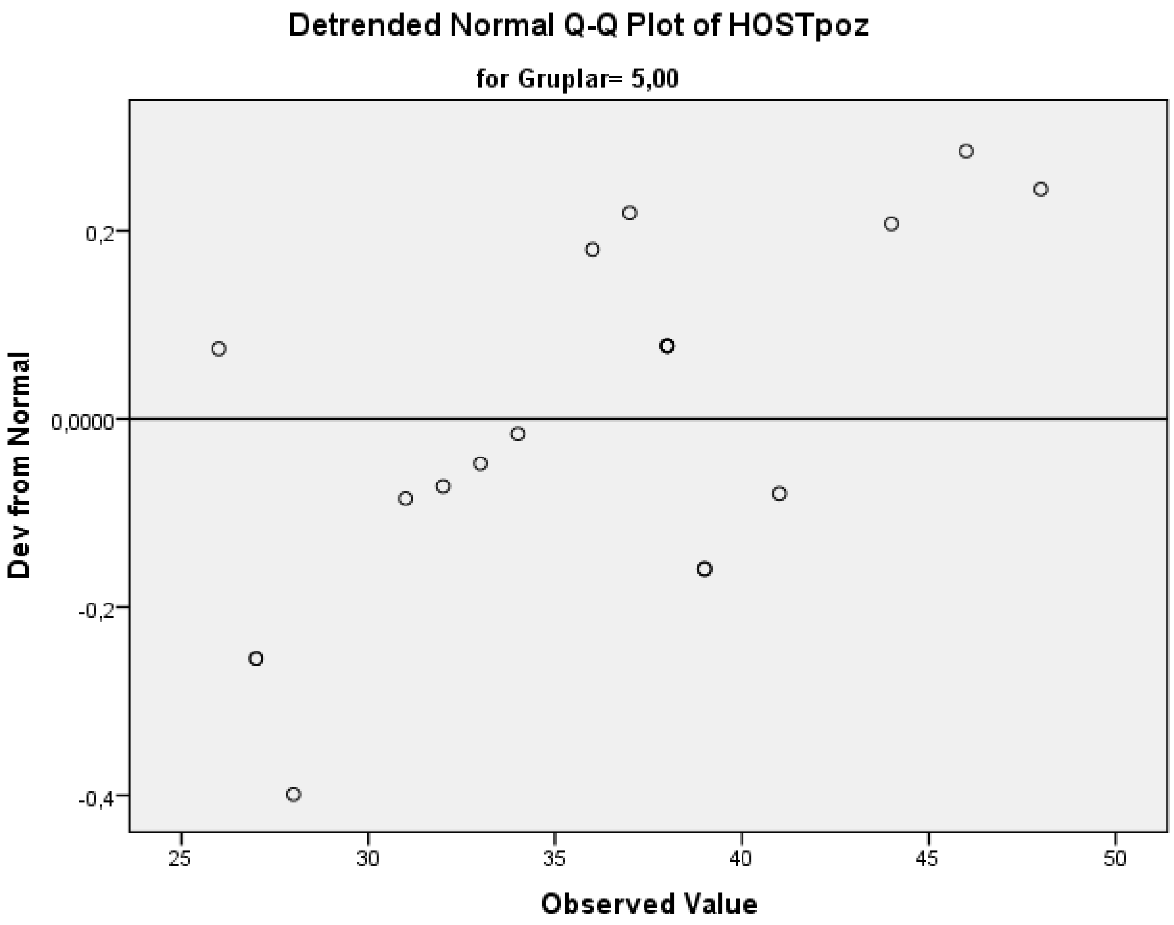

Detrended Normal Q-Q Plot of HOSTpoz
for Gruplar= 5,00
Dev from Normal
0,2
0,0000
-0,2
-0,4
25
30
35
40
45
50
Observed Value

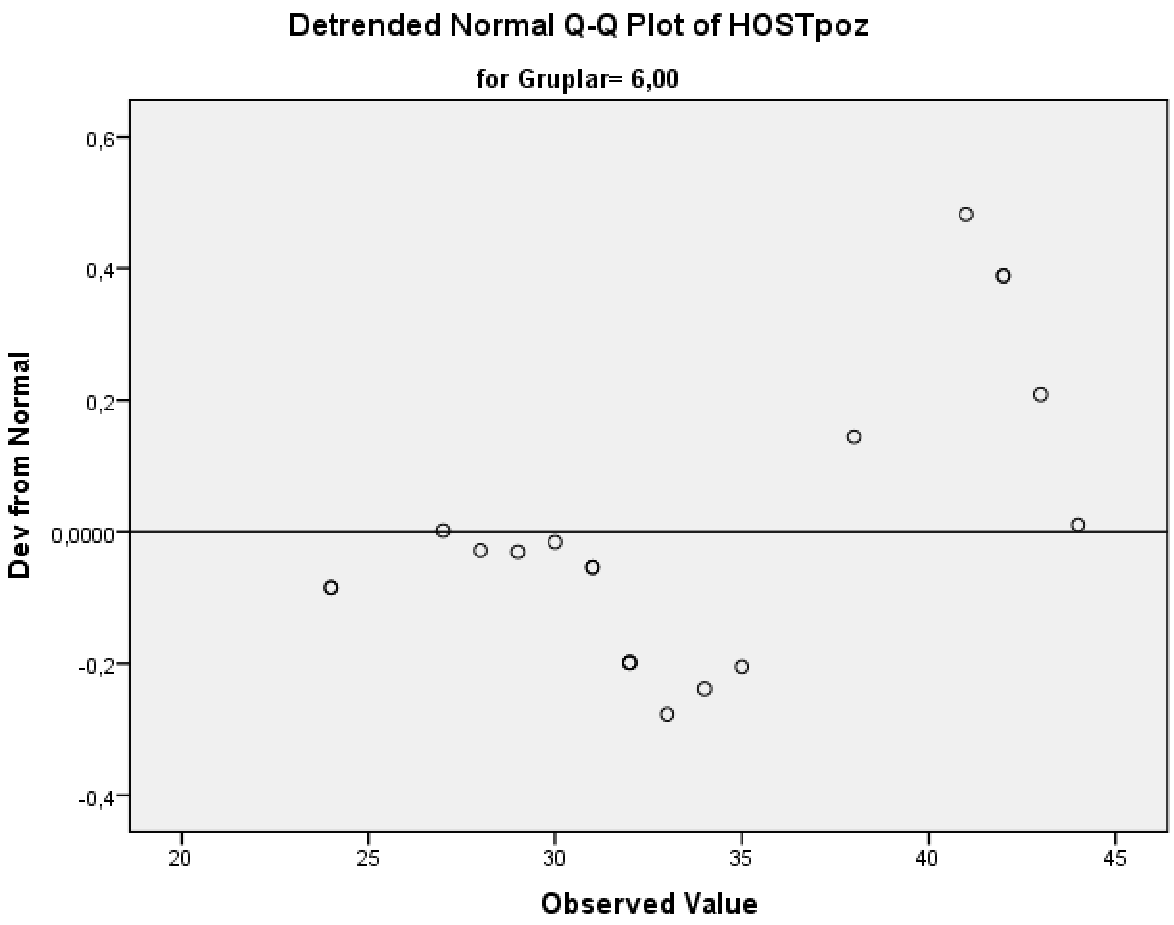
Detrended Normal Q-Q Plot of HOSTpoz
for Gruplar= 6,00
Dev from Normal
0,6
0,4
0,2
0,0000
-0,2
-0,4
20
25
30
35
40
45
Observed Value

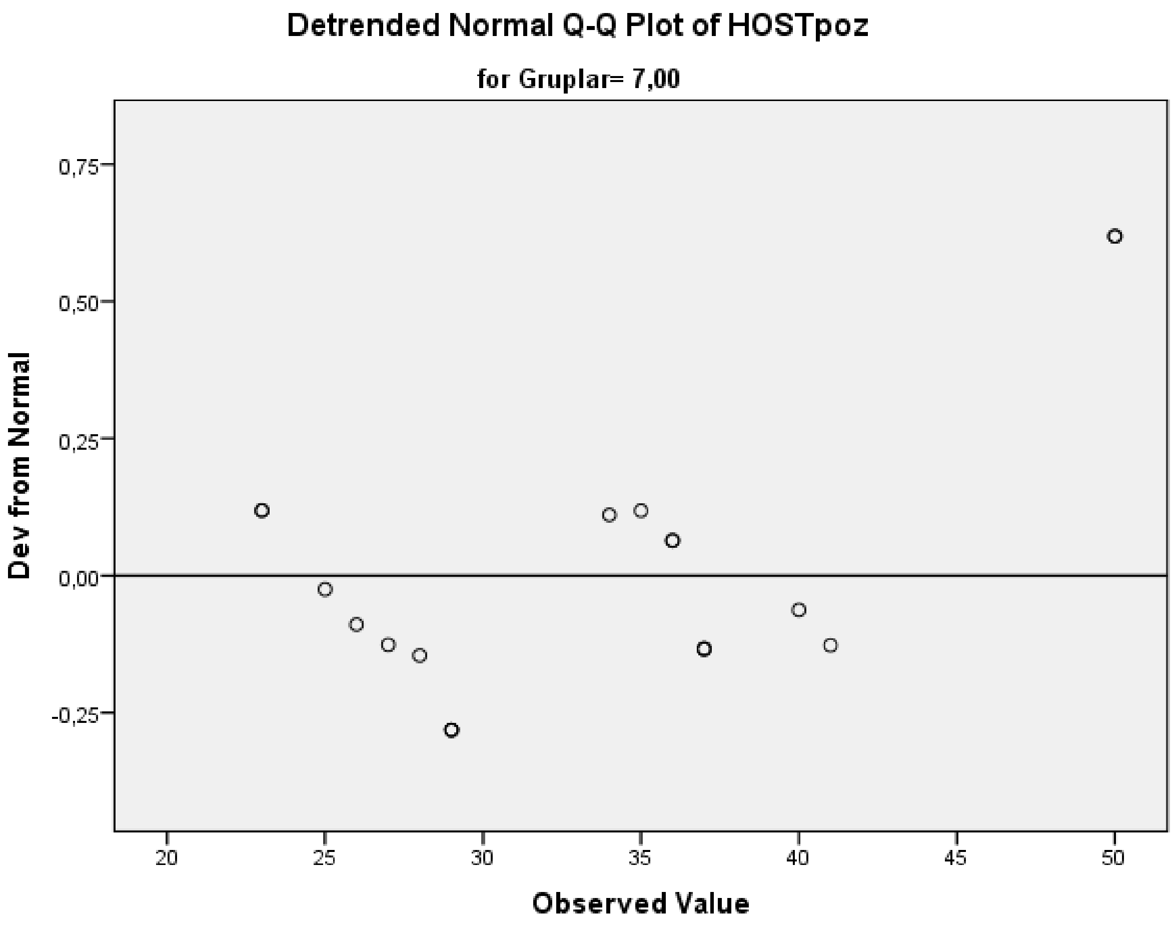
Detrended Normal Q-Q Plot of HOSTpoz
for Gruplar= 7,00
Dev from Normal
0,75
0,50
0,25
0,00
-0,25
20
25
30
35
40
45
50
Observed Value

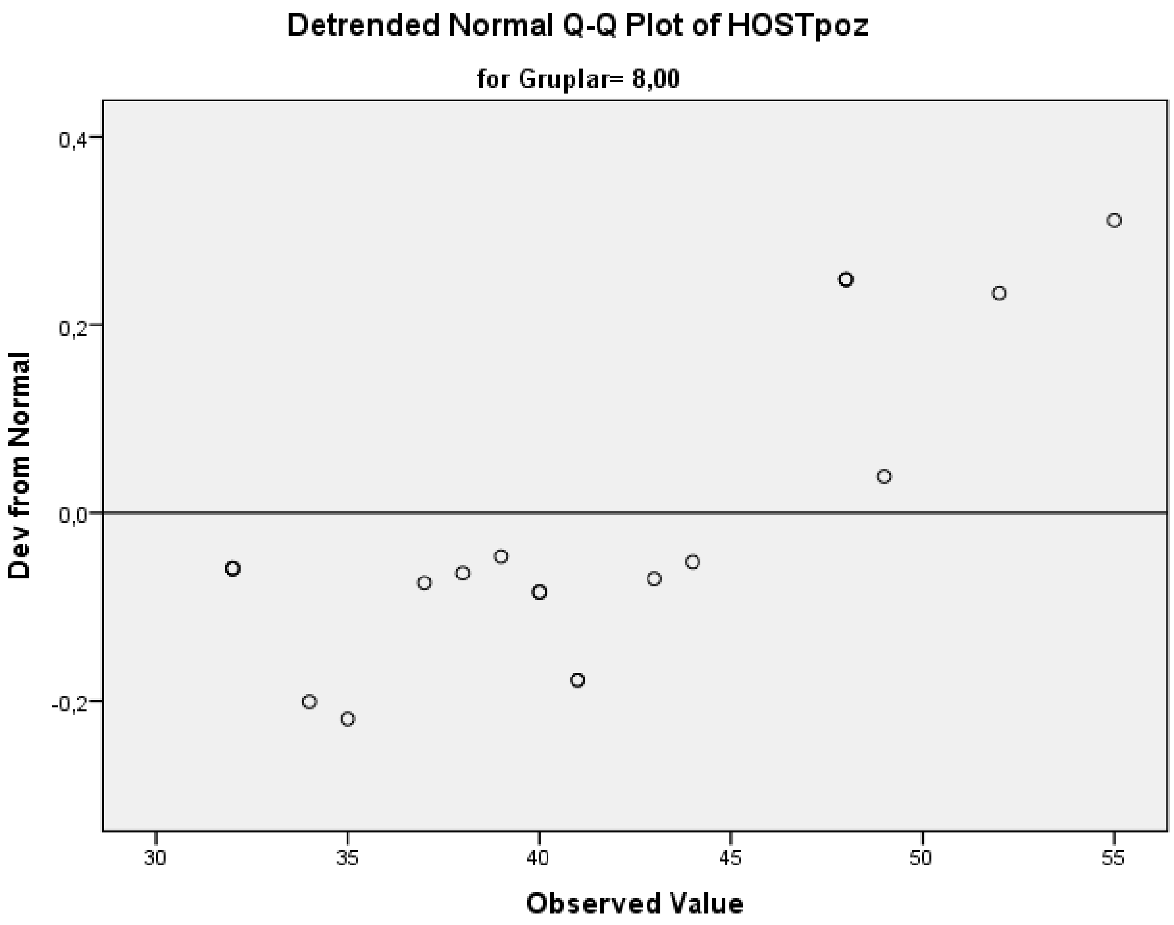
Detrended Normal Q-Q Plot of HOSTpoz
for Gruplar= 8,00
Dev from Normal
Observed Value
0,4
0,2
0,0
-0,2
30
35
40
45
50
55

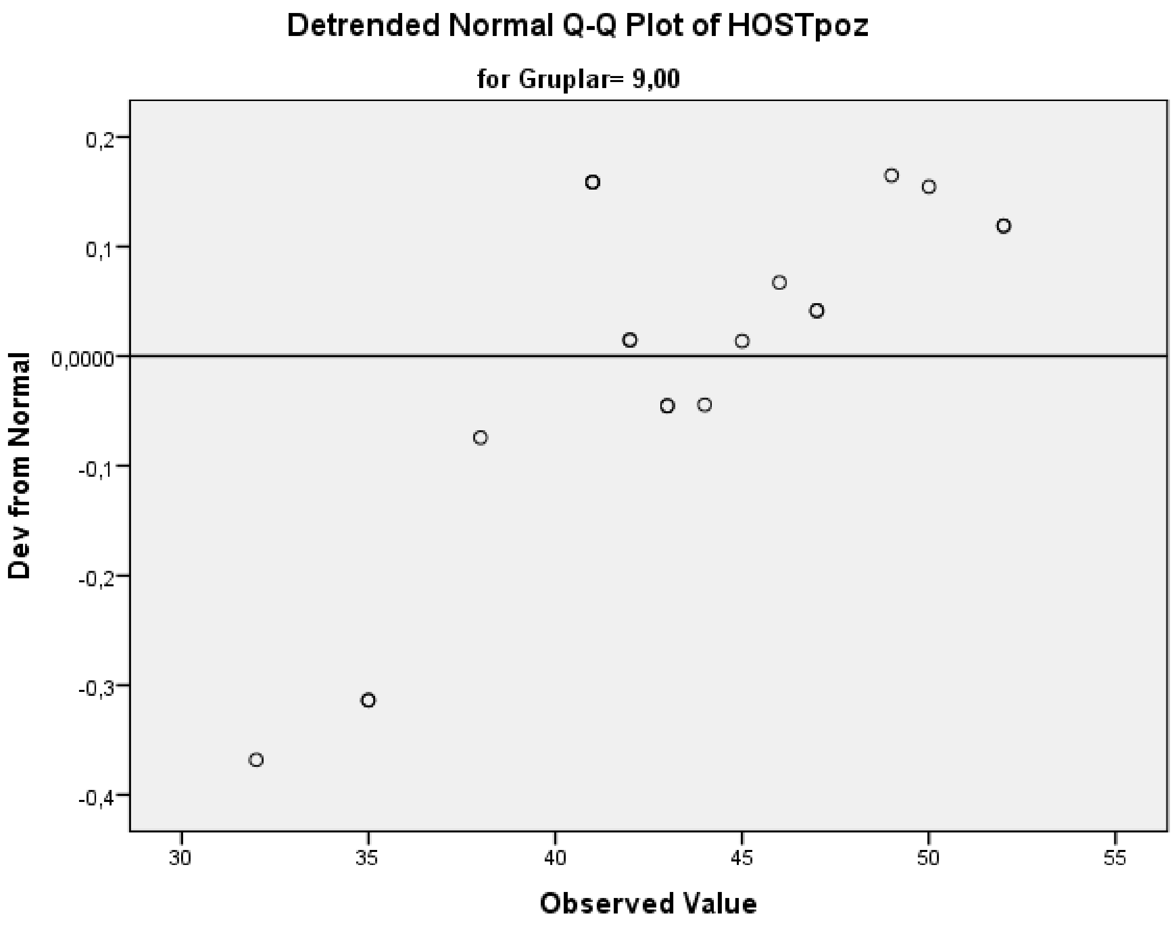
Detrended Normal Q-Q Plot of HOSTpoz
for Gruplar= 9,00
Dev from Normal
0,2
0,1
0,0000
-0,1
-0,2
-0,3
-0,4
30
35
40
45
50
55
Observed Value

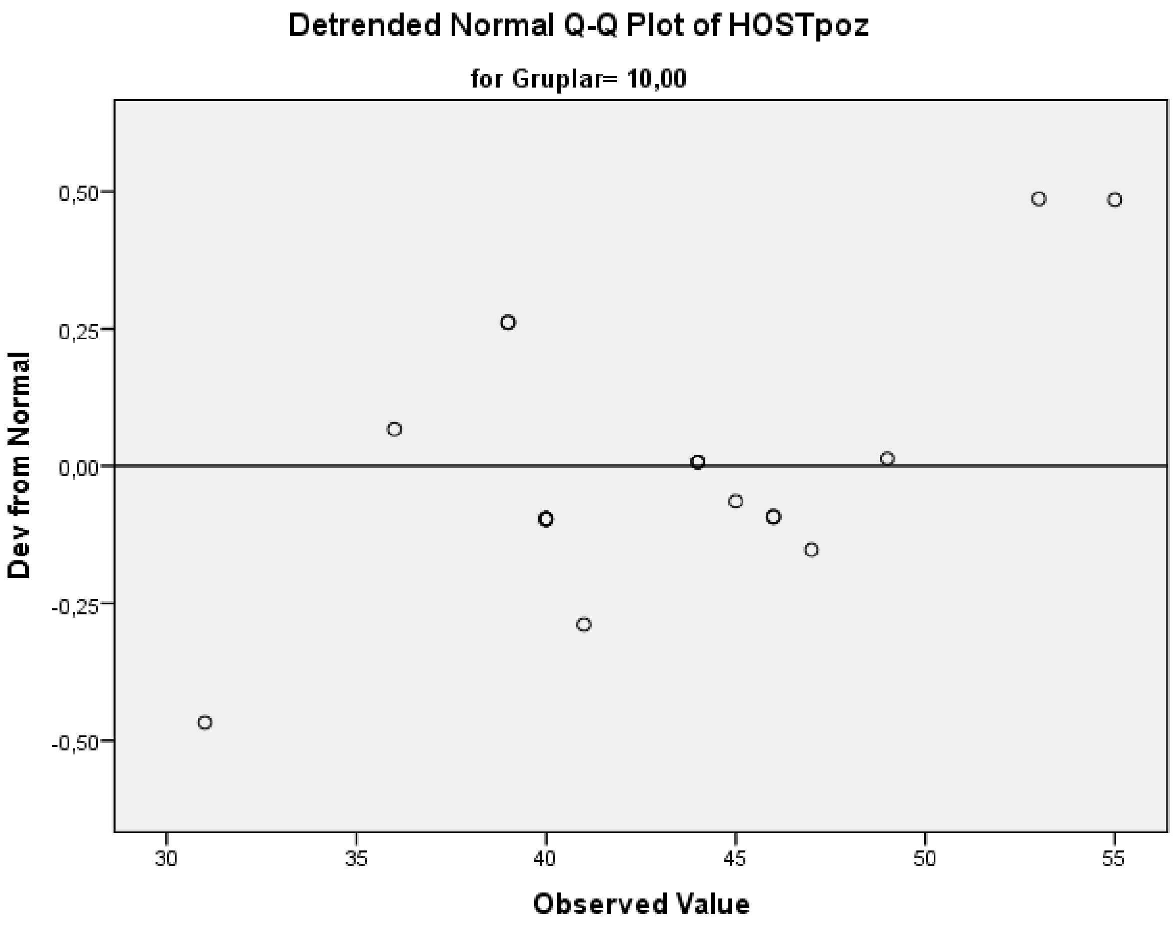
Detrended Normal Q-Q Plot of HOSTpoz
for Gruplar= 10,00
Dev from Normal
0,50
0,25
0,00
-0,25
-0,50
30
35
40
45
50
55
Observed Value

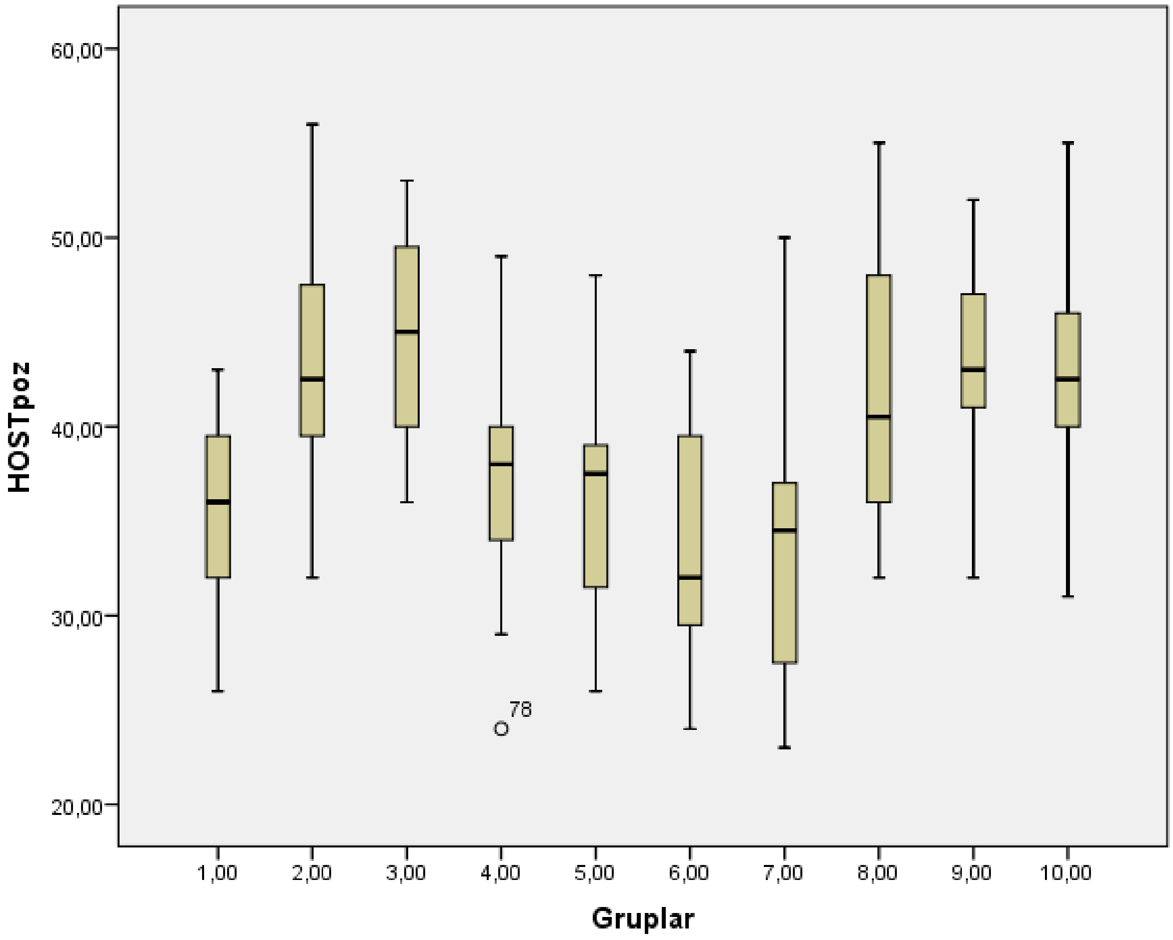


```
ONEWAY HOSTpoz BY Gruplar
  /STATISTICS DESCRIPTIVES HOMOGENEITY
  /PLOT MEANS
  /MISSING ANALYSIS
  /POSTHOC=DUNCAN ALPHA(0.05).
```

Oneway

Notes

Output Created		24-DEC-2020 14:16:53
Comments		
Input	Active Dataset	DataSet0
	Filter	<none>
	Weight	<none>

	Split File	<none>
	N of Rows in Working Data File	200
Missing Value Handling	Definition of Missing	User-defined missing values are treated as missing.
	Cases Used	Statistics for each analysis are based on cases with no missing data for any variable in the analysis.
Syntax		ONEWAY HOSTpoz BY Gruplar<br>/STATISTICS DESCRIPTIVES HOMOGENEITY<br>/PLOT MEANS<br>/MISSING ANALYSIS<br>/POSTHOC=DUNCAN ALPHA(0.05).
Resources	Processor Time	00:00:00,23
	Elapsed Time	00:00:00,32

35,1500
43,8000
44,6500
37,4500
36,0000
33,6000
33,6000
41,4000
43,2500
42,9500

Test of Homogeneity of Variances

HOSTpoz

Levene Statistic	df1	df2	Sig.
,927	9	190	,503

ANOVA

HOSTpoz

	Sum of Squares	df	Mean Square	F	Sig.
Between Groups	3571,805	9	396,867	10,614	,000

Within Groups	7104,350	190	37,391		
Total	10676,155	199			

Post Hoc Tests

Homogeneous Subsets

HOSTpoz

Duncan[a]

Gruplar	N	Subset for alpha = 0.05	
		1	2
6,00	20	33,6000	
7,00	20	33,6000	
1,00	20	35,1500	
5,00	20	36,0000	
4,00	20	37,4500	
8,00	20		41,4000
10,00	20		42,9500
9,00	20		43,2500
2,00	20		43,8000
3,00	20		44,6500
Sig.		,077	,138

Means for groups in homogeneous subsets are displayed.

a. Uses Harmonic Mean Sample Size = 20,000.

Means Plots

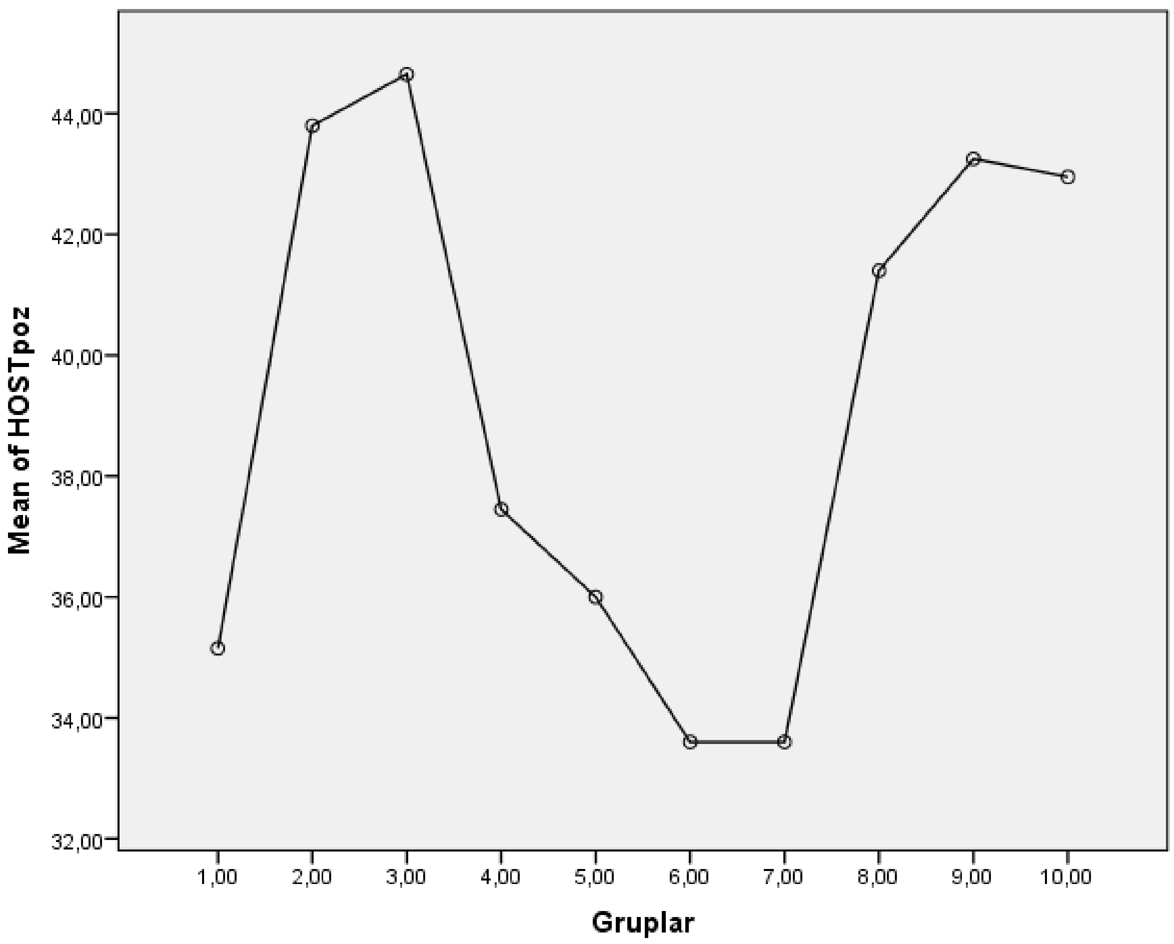

Mean of HOSTpoz
44,00
42,00
40,00
38,00
36,00
34,00
32,00
1,00
2,00
3,00
4,00
5,00
6,00
7,00
8,00
9,00
10,00
Gruplar

Plasma membrane integrity- ANOVA

MembranB: Plasma membrane integrity

Groups

1: Control

2: RES10

3: RES20

4: RES40

5: CD10

6: CD20

7: CD40

8: RLC10

9: RLC20

10: RLC40

Explore

Notes

Output Created		24-DEC-2020 12:14:19
Comments		
Input	Active Dataset	DataSet0
	Filter	<none>
	Weight	<none>
	Split File	<none>
	N of Rows in Working Data File	100
Missing Value Handling	Definition of Missing	User-defined missing values for dependent variables are treated as missing.
	Cases Used	Statistics are based on cases with no missing values for any dependent variable or factor used.

Syntax		EXAMINE VARIABLES=MembranB BY Gruplar<br>/PLOT BOXPLOT STEMLEAF HISTOGRAM NPPLOT<br>/COMPARE GROUPS<br>/STATISTICS DESCRIPTIVES<br>/CINTERVAL 95<br>/MISSING LISTWISE<br>/NOTOTAL.
Resources	Processor Time	00:00:08,31
	Elapsed Time	00:00:06,14

[DataSet0]

Gruplar

Case Processing Summary

	Gruplar	Cases					
		Valid		Missing		Total	
		N	Percent	N	Percent	N	Percent
MembranB	1,00	10	100,0%	0	0,0%	10	100,0%
	2,00	10	100,0%	0	0,0%	10	100,0%
	3,00	10	100,0%	0	0,0%	10	100,0%
	4,00	10	100,0%	0	0,0%	10	100,0%
	5,00	10	100,0%	0	0,0%	10	100,0%
	6,00	10	100,0%	0	0,0%	10	100,0%
	7,00	10	100,0%	0	0,0%	10	100,0%
	8,00	10	100,0%	0	0,0%	10	100,0%
	9,00	10	100,0%	0	0,0%	10	100,0%
	10,00	10	100,0%	0	0,0%	10	100,0%

Descriptives

Gruplar	Statistic	Std. Error

MembranB	1,00	Mean		30,0340	1,87231
		95% Confidence Interval for Mean	Lower Bound	25,7985	
			Upper Bound	34,2695	
		5% Trimmed Mean		29,8789	
		Median		27,5600	
		Variance		35,055	
		Std. Deviation		5,92075	
		Minimum		23,58	
		Maximum		39,28	
		Range		15,70	
		Interquartile Range		11,25	
		Skewness		,886	,687
		Kurtosis		-1,017	1,334
	2,00	Mean		34,8170	2,61345
		95% Confidence Interval for Mean	Lower Bound	28,9050	
			Upper Bound	40,7290	
		5% Trimmed Mean		34,4922	
		Median		30,6400	
		Variance		68,301	
		Std. Deviation		8,26447	
		Minimum		26,02	
		Maximum		49,46	
		Range		23,44	
		Interquartile Range		14,63	
		Skewness		,711	,687
		Kurtosis		-1,017	1,334
	3,00	Mean		30,8380	2,37217
		95% Confidence Interval for Mean	Lower Bound	25,4718	
			Upper Bound	36,2042	
		5% Trimmed Mean		30,8611	
		Median		29,8000	
		Variance		56,272	
		Std. Deviation		7,50146	
		Minimum		20,68	
		Maximum		40,58	
		Range		19,90	
		Interquartile Range		13,96	
		Skewness		,055	,687

	Kurtosis		-1,997	1,334
4,00	Mean		28,8220	2,05493
	95% Confidence Interval for Mean	Lower Bound	24,1734	
		Upper Bound	33,4706	
	5% Trimmed Mean		28,7056	
	Median		27,2200	
	Variance		42,227	
	Std. Deviation		6,49826	
	Minimum		20,12	
	Maximum		39,62	
	Range		19,50	
	Interquartile Range		11,43	
	Skewness		,539	,687
	Kurtosis		-,834	1,334
5,00	Mean		31,8820	2,00501
	95% Confidence Interval for Mean	Lower Bound	27,3463	
		Upper Bound	36,4177	
	5% Trimmed Mean		31,8967	
	Median		31,0300	
	Variance		40,201	
	Std. Deviation		6,34041	
	Minimum		22,12	
	Maximum		41,38	
	Range		19,26	
	Interquartile Range		10,67	
	Skewness		,122	,687
	Kurtosis		-1,163	1,334
6,00	Mean		25,6360	1,65042
	95% Confidence Interval for Mean	Lower Bound	21,9025	
		Upper Bound	29,3695	
	5% Trimmed Mean		25,6633	
	Median		25,9800	
	Variance		27,239	
	Std. Deviation		5,21907	
	Minimum		18,24	
	Maximum		32,54	
	Range		14,30	
	Interquartile Range		9,26	

	Skewness		-,061	,687
	Kurtosis		-1,317	1,334
7,00	Mean		23,4500	1,86978
	95% Confidence Interval for Mean	Lower Bound	19,2203	
		Upper Bound	27,6797	
	5% Trimmed Mean		23,2600	
	Median		22,3900	
	Variance		34,961	
	Std. Deviation		5,91276	
	Minimum		16,62	
	Maximum		33,70	
	Range		17,08	
	Interquartile Range		8,60	
	Skewness		,798	,687
	Kurtosis		-,513	1,334
8,00	Mean		36,4360	1,39672
	95% Confidence Interval for Mean	Lower Bound	33,2764	
		Upper Bound	39,5956	
	5% Trimmed Mean		36,3111	
	Median		34,8900	
	Variance		19,508	
	Std. Deviation		4,41682	
	Minimum		31,26	
	Maximum		43,86	
	Range		12,60	
	Interquartile Range		8,59	
	Skewness		,718	,687
	Kurtosis		-1,045	1,334
9,00	Mean		34,6740	1,79017
	95% Confidence Interval for Mean	Lower Bound	30,6243	
		Upper Bound	38,7237	
	5% Trimmed Mean		34,6556	
	Median		34,6400	
	Variance		32,047	
	Std. Deviation		5,66103	
	Minimum		24,82	
	Maximum		44,86	
	Range		20,04	

		Interquartile Range		6,49	
		Skewness		,233	,687
		Kurtosis		,536	1,334
	10,00	Mean		33,4840	2,67238
		95% Confidence Interval for Mean	Lower Bound	27,4387	
			Upper Bound	39,5293	
		5% Trimmed Mean		33,4889	
		Median		35,6900	
		Variance		71,416	
		Std. Deviation		8,45080	
		Minimum		18,14	
		Maximum		48,74	
		Range		30,60	
		Interquartile Range		10,78	
		Skewness		-,092	,687
		Kurtosis		,570	1,334

Tests of Normality

		Kolmogorov-Smirnov[a]			Shapiro-Wilk		
	Gruplar	Statistic	df	Sig.	Statistic	df	Sig.
MembranB	1,00	,320	10	,004	,810	10	,019
	2,00	,274	10	,033	,877	10	,120
	3,00	,257	10	,061	,873	10	,110
	4,00	,158	10	,200*	,941	10	,560
	5,00	,207	10	,200*	,949	10	,659
	6,00	,150	10	,200*	,931	10	,460
	7,00	,201	10	,200*	,888	10	,161
	8,00	,196	10	,200*	,887	10	,155
	9,00	,183	10	,200*	,969	10	,885
	10,00	,189	10	,200*	,950	10	,673

*. This is a lower bound of the true significance.

a. Lilliefors Significance Correction

MembranB

Histograms

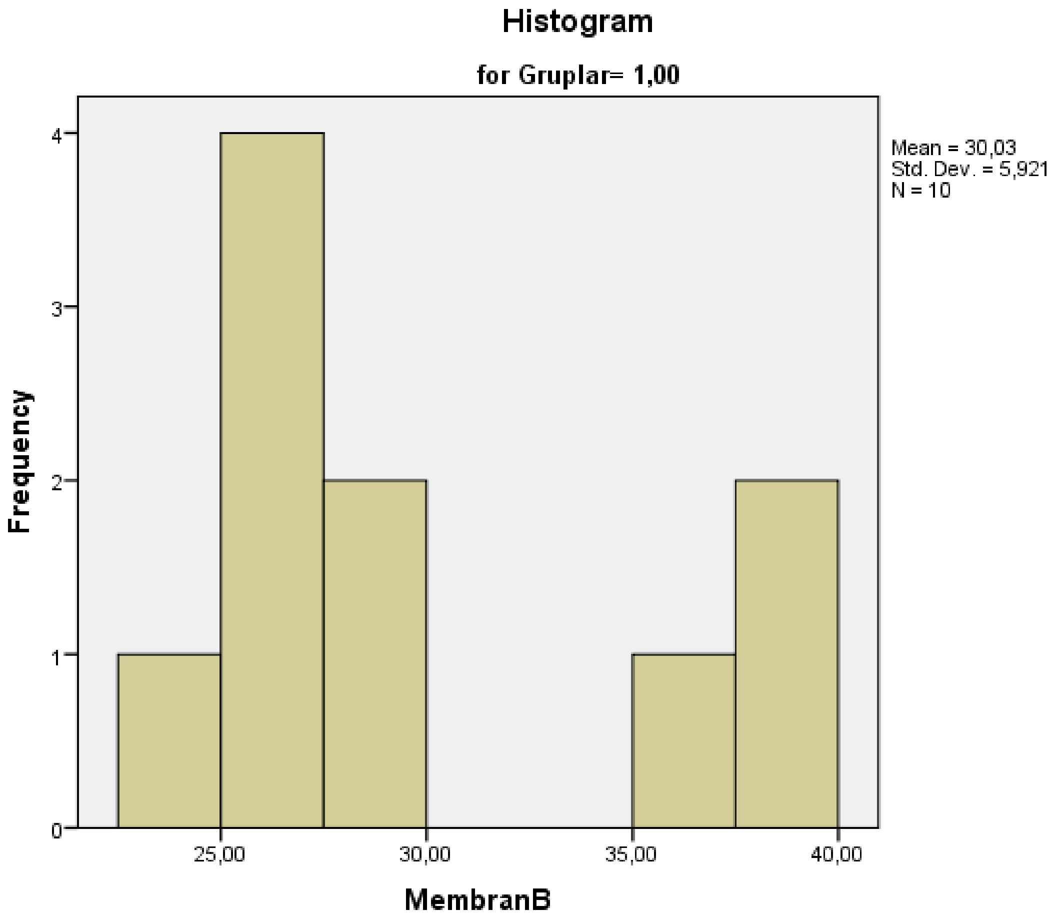

Histogram
for Gruplar= 1,00
Mean = 30,03
Std. Dev. = 5,921
N = 10
Frequency
0
1
2
3
4
25,00
30,00
35,00
40,00
MembranB

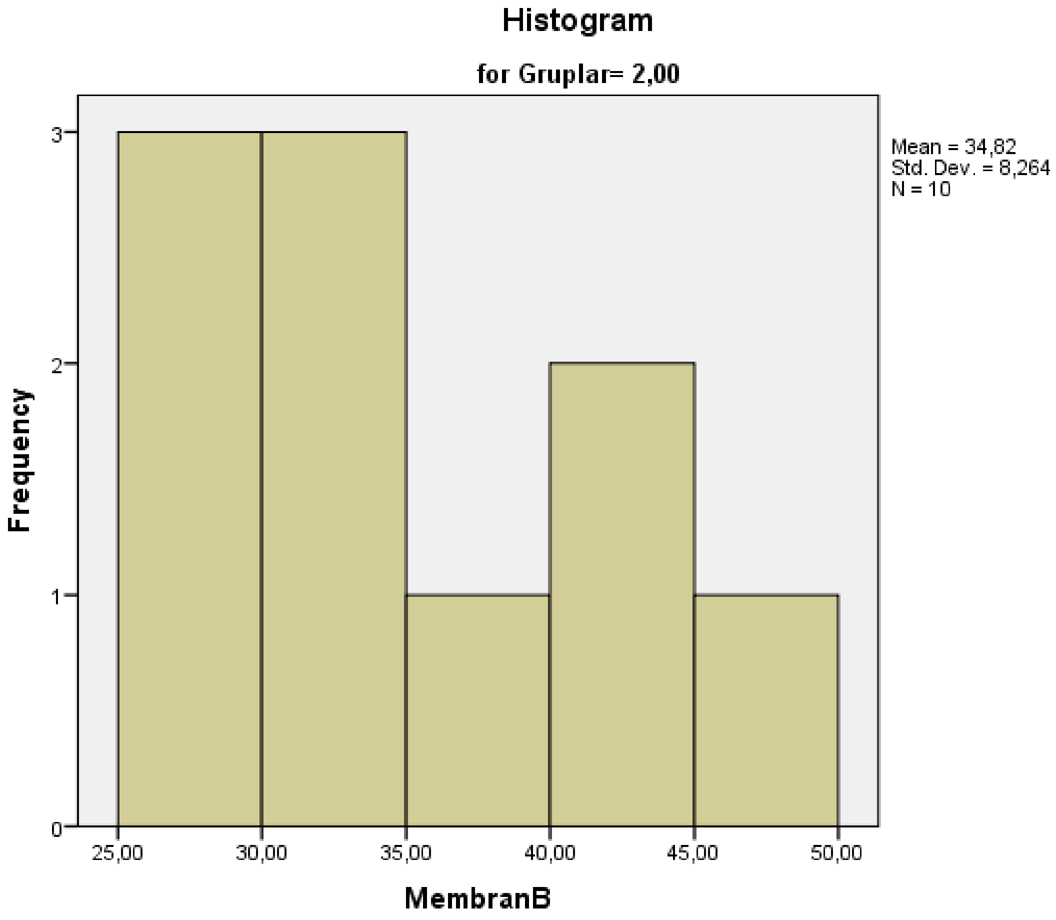
Histogram
for Gruplar= 2,00
Mean = 34,82
Std. Dev. = 8,264
N = 10
Frequency
0
1
2
3
25,00
30,00
35,00
40,00
45,00
50,00
MembranB

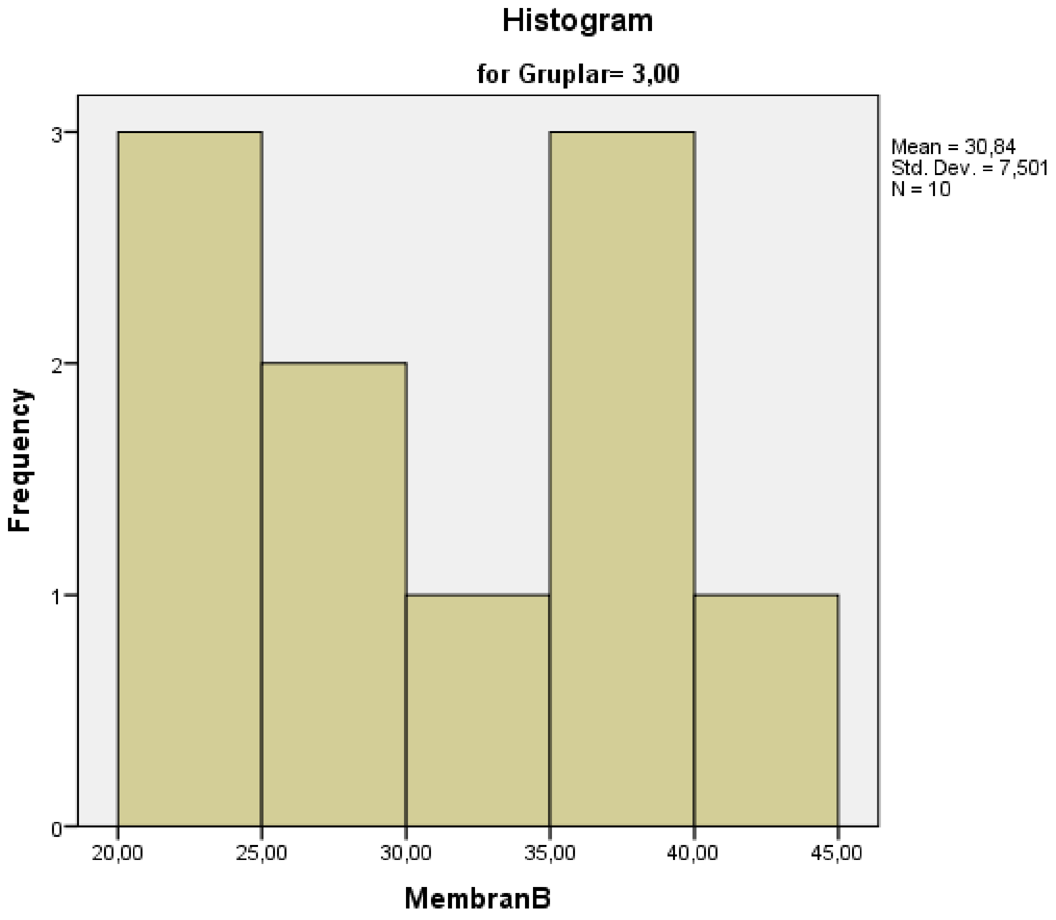
Histogram
for Gruplar= 3,00
Mean = 30,84
Std. Dev. = 7,501
N = 10
Frequency
0
1
2
3
20,00
25,00
30,00
35,00
40,00
45,00
MembranB

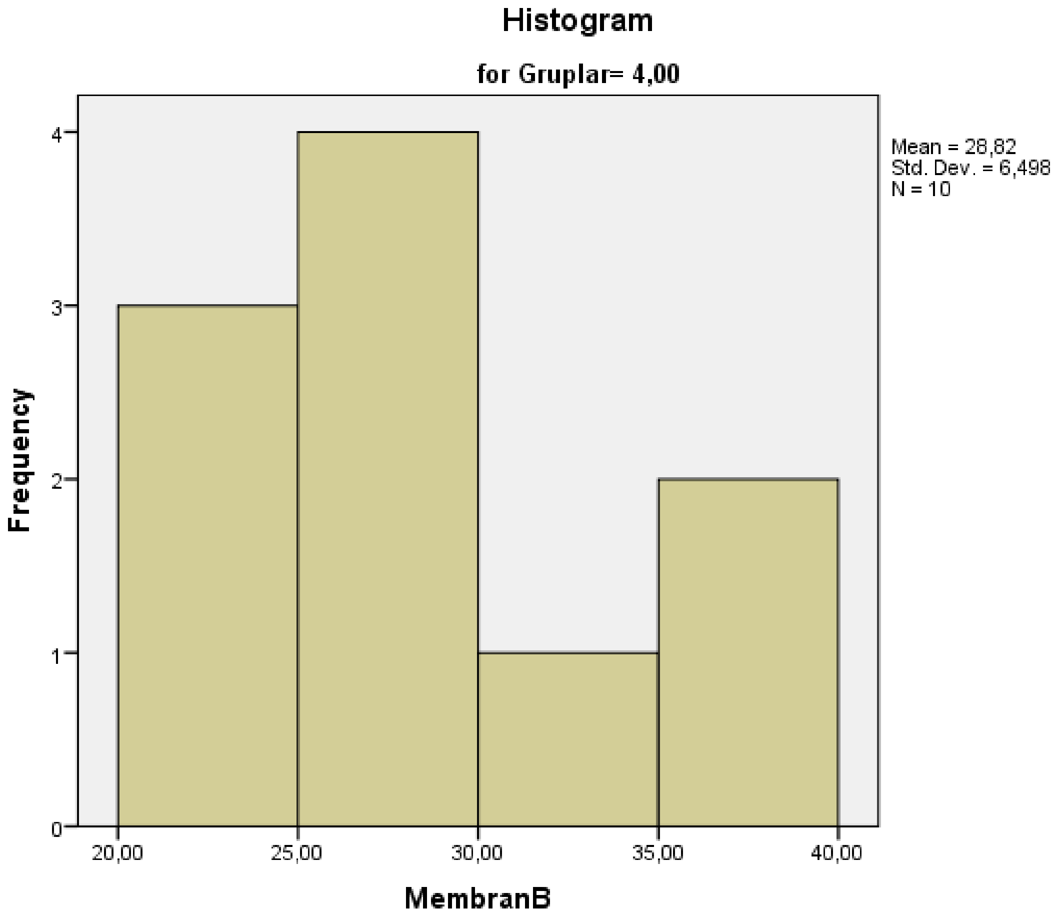

Histogram
for Gruplar= 4,00
Mean = 28,82
Std. Dev. = 6,498
N = 10
Frequency
0
1
2
3
4
20,00
25,00
30,00
35,00
40,00
MembranB

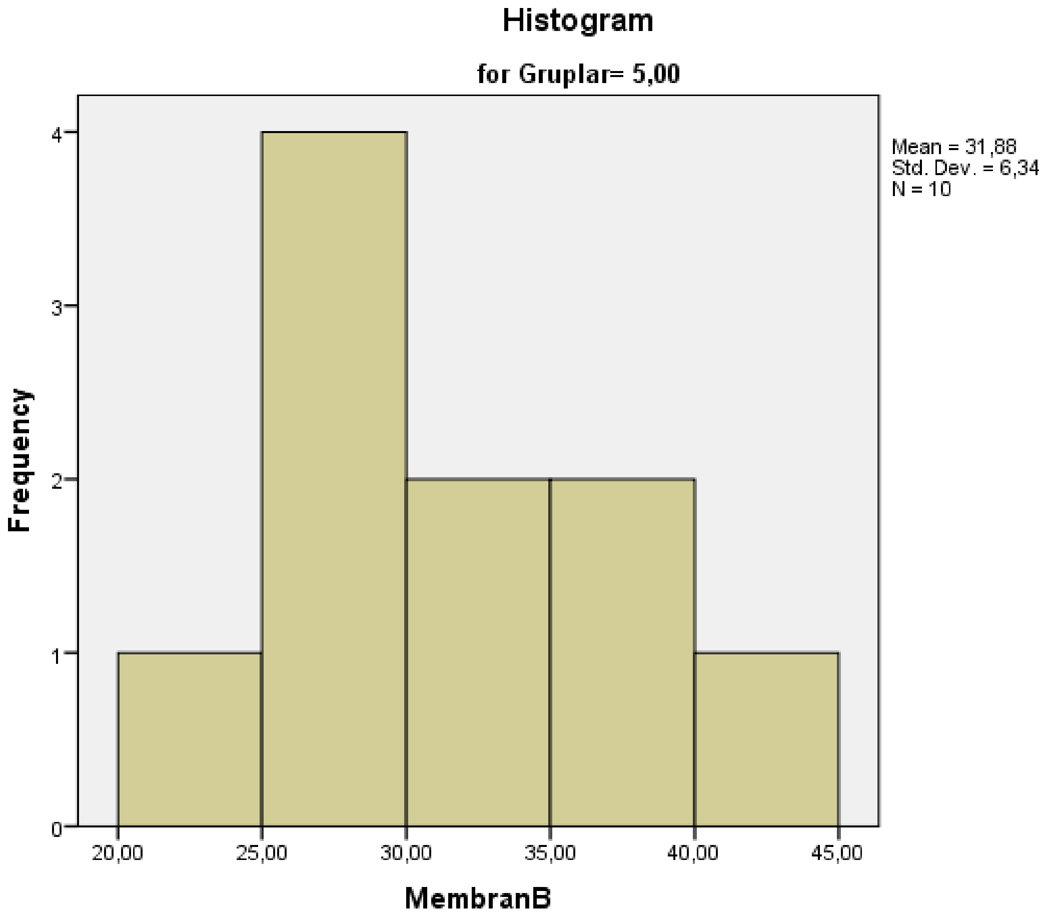
Histogram
for Gruplar= 5,00
Frequency
MembranB
Mean = 31,88
Std. Dev. = 6,34
N = 10
0
1
2
3
4
20,00
25,00
30,00
35,00
40,00
45,00

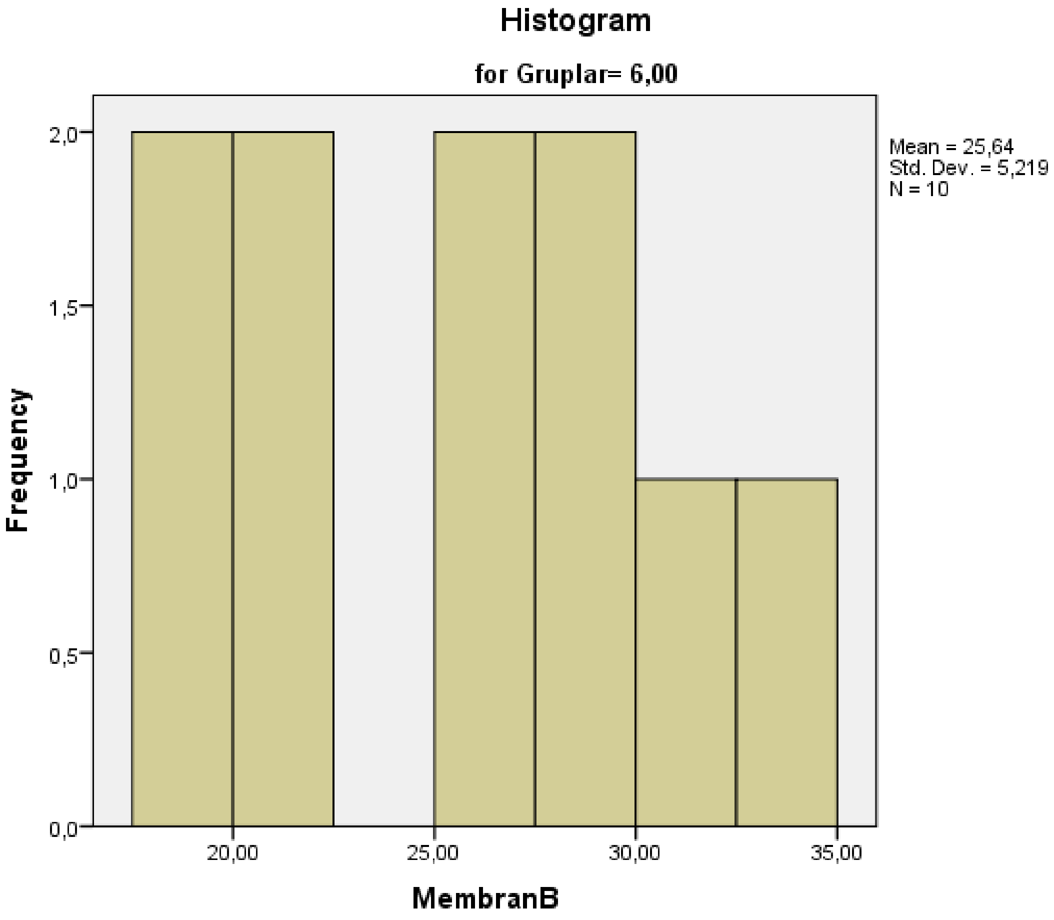

Histogram
for Gruplar= 6,00
Mean = 25,64
Std. Dev. = 5,219
N = 10
Frequency
2,0
1,5
1,0
0,5
0,0
20,00
25,00
30,00
35,00
MembranB

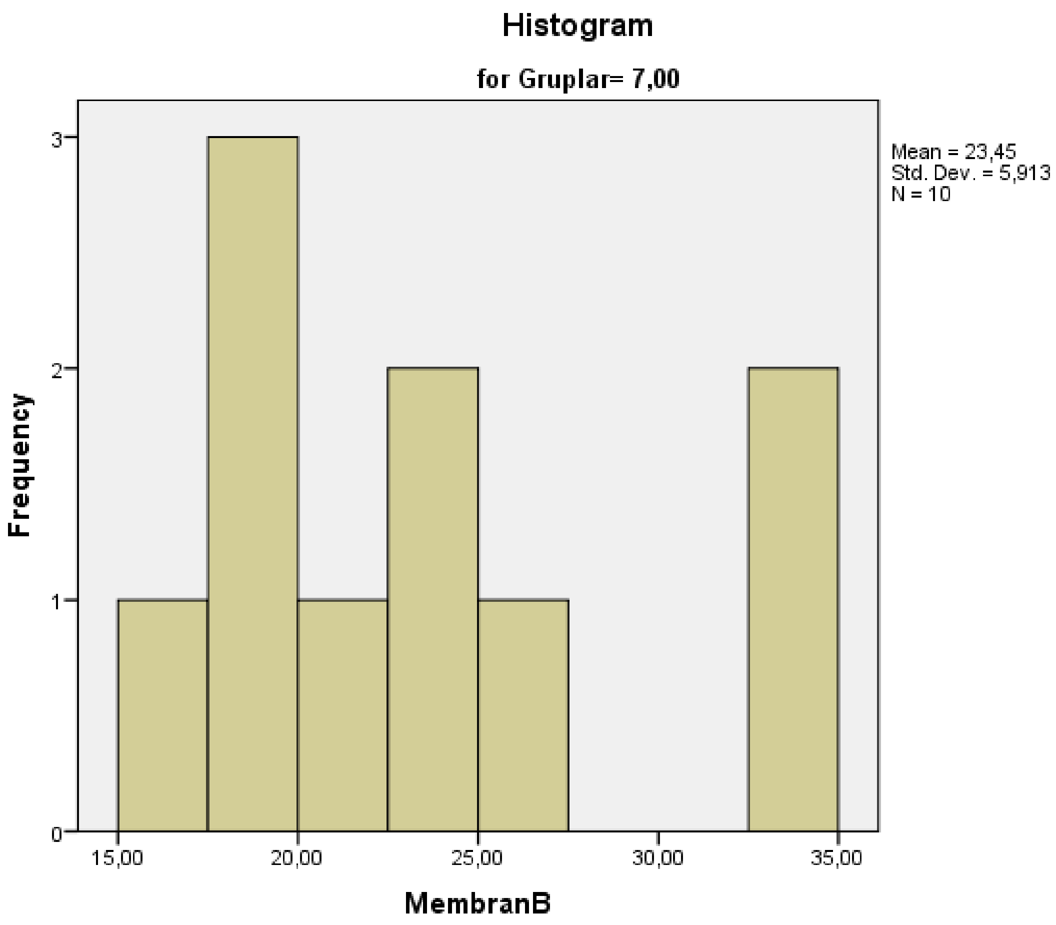
Histogram
for Gruplar= 7,00
Mean = 23,45
Std. Dev. = 5,913
N = 10
Frequency
0
1
2
3
15,00
20,00
25,00
30,00
35,00
MembranB

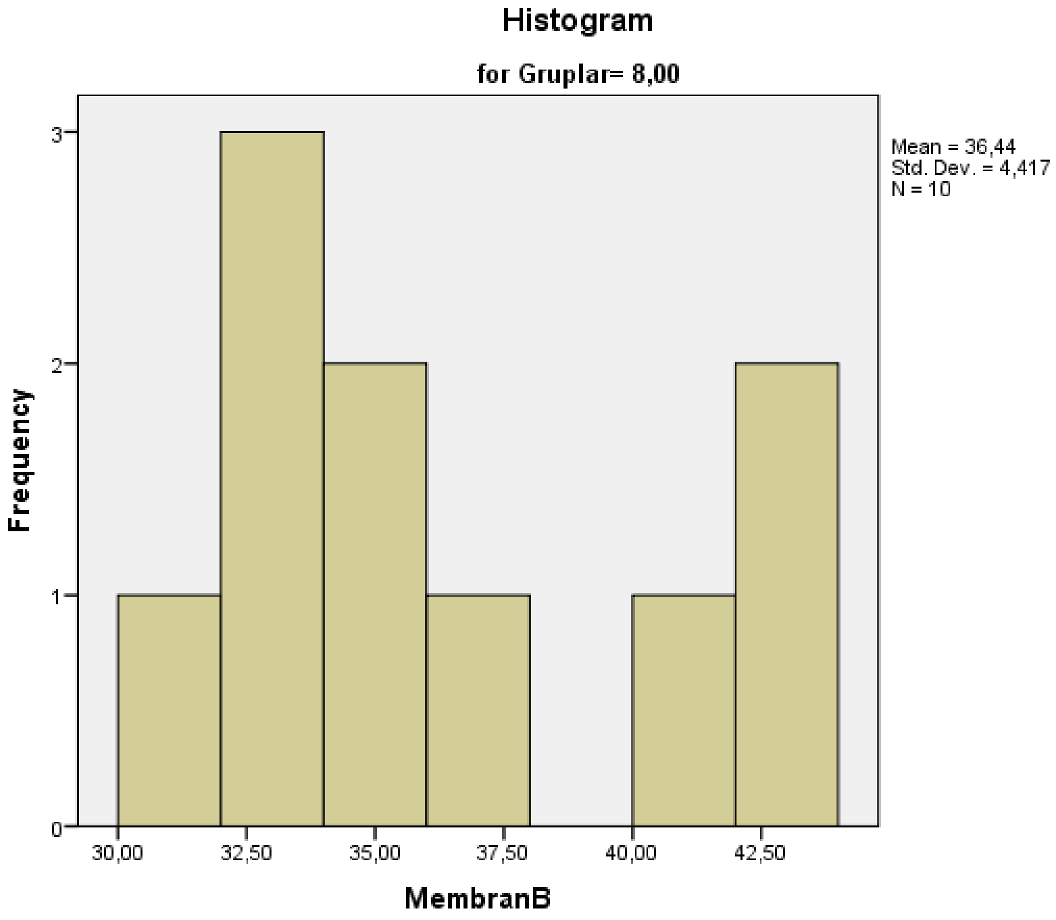

Histogram
for Gruplar= 8,00
Mean = 36,44
Std. Dev. = 4,417
N = 10
Frequency
0
1
2
3
30,00
32,50
35,00
37,50
40,00
42,50
MembranB

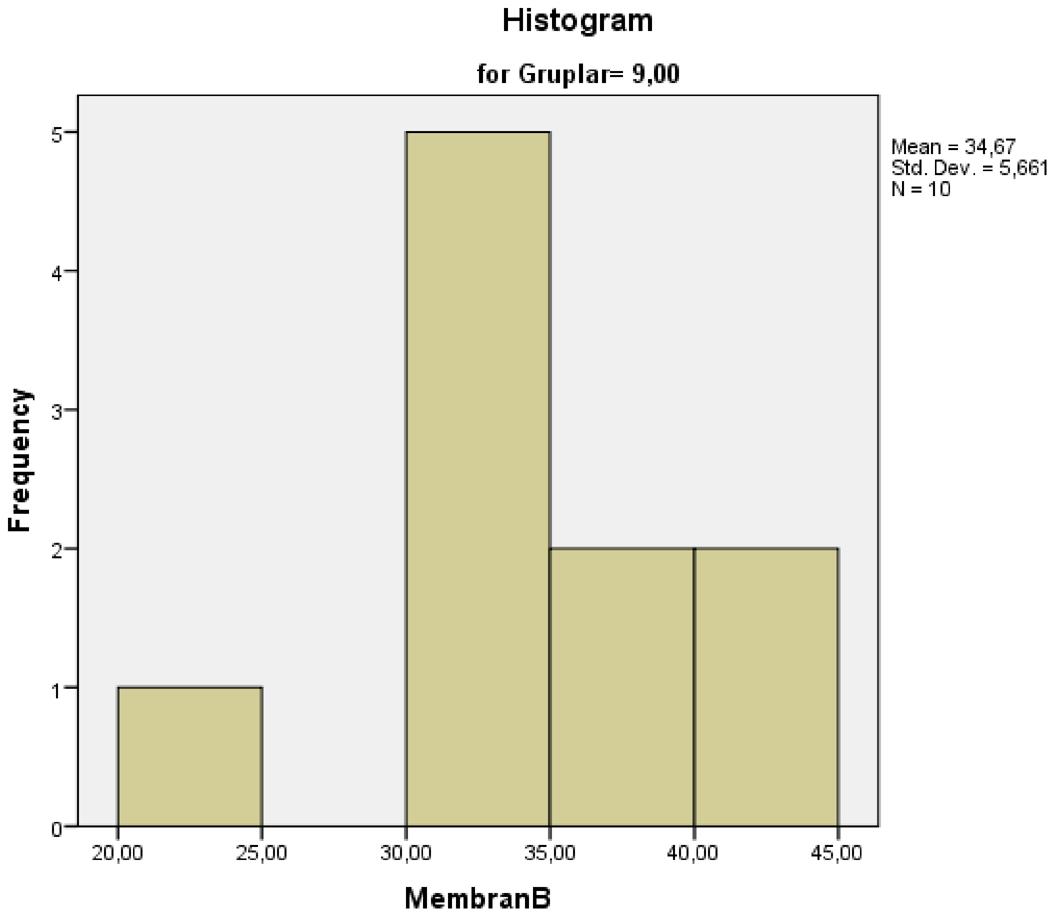

Histogram
for Gruplar= 9,00
Mean = 34,67
Std. Dev. = 5,661
N = 10
Frequency
0
1
2
3
4
5
20,00
25,00
30,00
35,00
40,00
45,00
MembranB

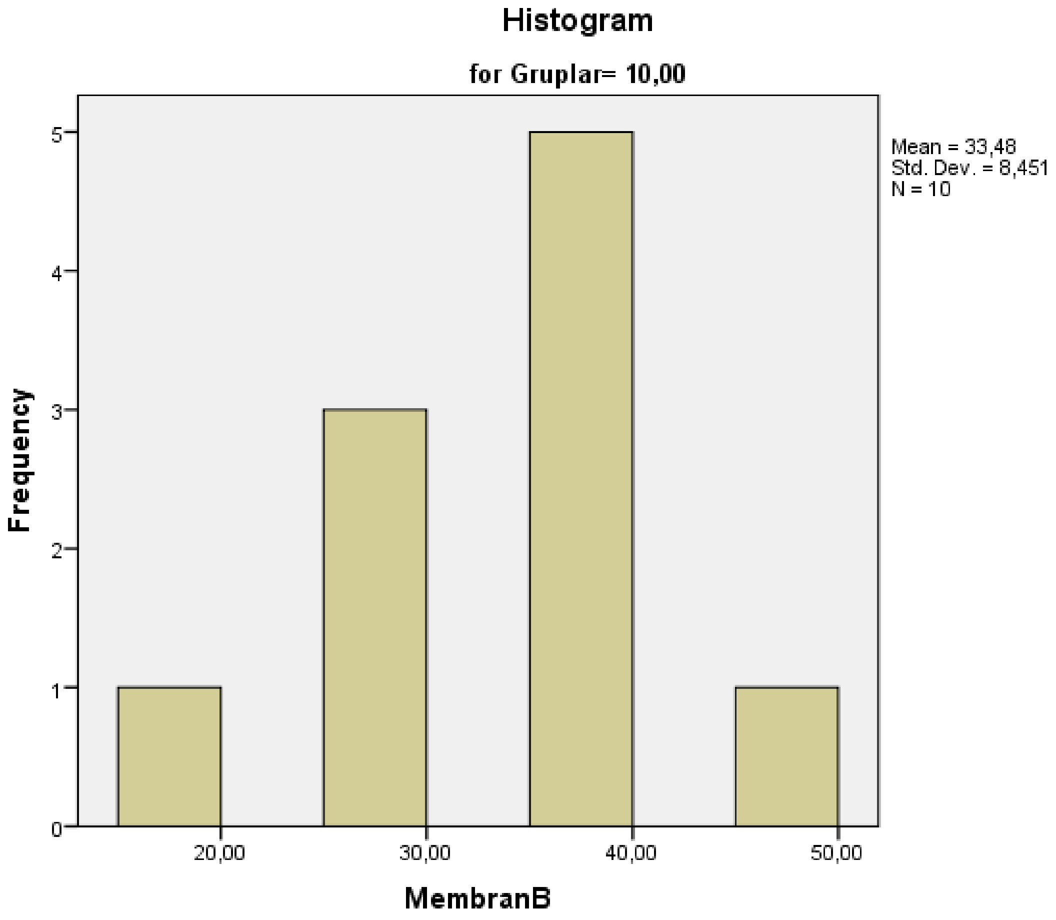

Stem-and-Leaf Plots

```
MembranB Stem-and-Leaf Plot for
Gruplar= 1,00

 Frequency    Stem &  Leaf

     1,00        2 .  3
     6,00        2 .  567778
      ,00        3 .
     3,00        3 .  699

 Stem width:  10,00
 Each leaf:       1 case(s)
```

```
MembranB Stem-and-Leaf Plot for
Gruplar= 2,00

 Frequency    Stem &  Leaf

     3,00        2 .  669
     3,00        3 .  001
     1,00        3 .  8
     2,00        4 .  34
     1,00        4 .  9

 Stem width:  10,00
 Each leaf:       1 case(s)
```

```
MembranB Stem-and-Leaf Plot for
Gruplar= 3,00

 Frequency    Stem &  Leaf

     3,00        2 .  034
     2,00        2 .  55
     1,00        3 .  3
     3,00        3 .  689
     1,00        4 .  0

 Stem width:  10,00
 Each leaf:       1 case(s)
```

```
MembranB Stem-and-Leaf Plot for
Gruplar= 4,00

 Frequency    Stem &  Leaf

     3,00        2 .  024
     4,00        2 .  5689
     1,00        3 .  4
     2,00        3 .  79

 Stem width:  10,00
 Each leaf:       1 case(s)
```

```
MembranB Stem-and-Leaf Plot for
Gruplar= 5,00

 Frequency    Stem &  Leaf

     1,00        2 .  2
```

```
     4,00        2 .  6788
     2,00        3 .  34
     2,00        3 .  69
     1,00        4 .  1

 Stem width:  10,00
 Each leaf:       1 case(s)
```

MembranB Stem-and-Leaf Plot for
Gruplar= 6,00

```
 Frequency    Stem &  Leaf

     2,00        1 .  88
     2,00        2 .  22
     4,00        2 .  5689
     2,00        3 .  22

 Stem width:  10,00
 Each leaf:       1 case(s)
```

MembranB Stem-and-Leaf Plot for
Gruplar= 7,00

```
 Frequency    Stem &  Leaf

     4,00        1 .  6889
     3,00        2 .  044
     1,00        2 .  5
     2,00        3 .  23

 Stem width:  10,00
 Each leaf:       1 case(s)
```

MembranB Stem-and-Leaf Plot for
Gruplar= 8,00

```
 Frequency    Stem &  Leaf

     5,00        3 .  12334
     2,00        3 .  56
     3,00        4 .  123

 Stem width:  10,00
 Each leaf:       1 case(s)
```

```
MembranB Stem-and-Leaf Plot for
Gruplar= 9,00

 Frequency    Stem &  Leaf

     1,00        2 .  4
      ,00        2 .
     5,00        3 .  01244
     2,00        3 .  56
     1,00        4 .  1
     1,00 Extremes    (>=45)

 Stem width:  10,00
 Each leaf:       1 case(s)
```

```
MembranB Stem-and-Leaf Plot for
Gruplar= 10,00

 Frequency    Stem &  Leaf

     1,00        1 .  8
     3,00        2 .  778
     5,00        3 .  56778
     1,00        4 .  8

 Stem width:  10,00
 Each leaf:       1 case(s)
```

Normal Q-Q Plots

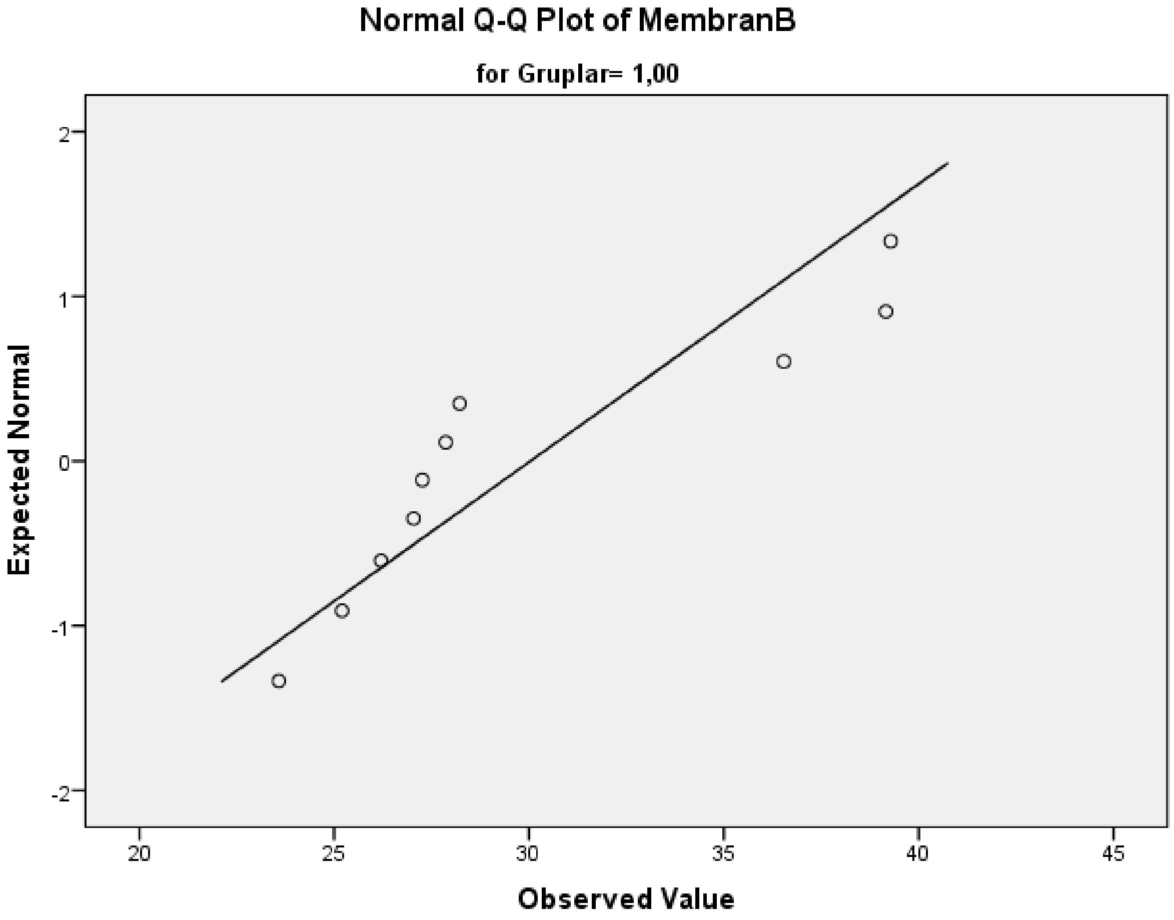
Normal Q-Q Plot of MembranB
for Gruplar= 1,00
Expected Normal
Observed Value
2
1
0
-1
-2
20
25
30
35
40
45

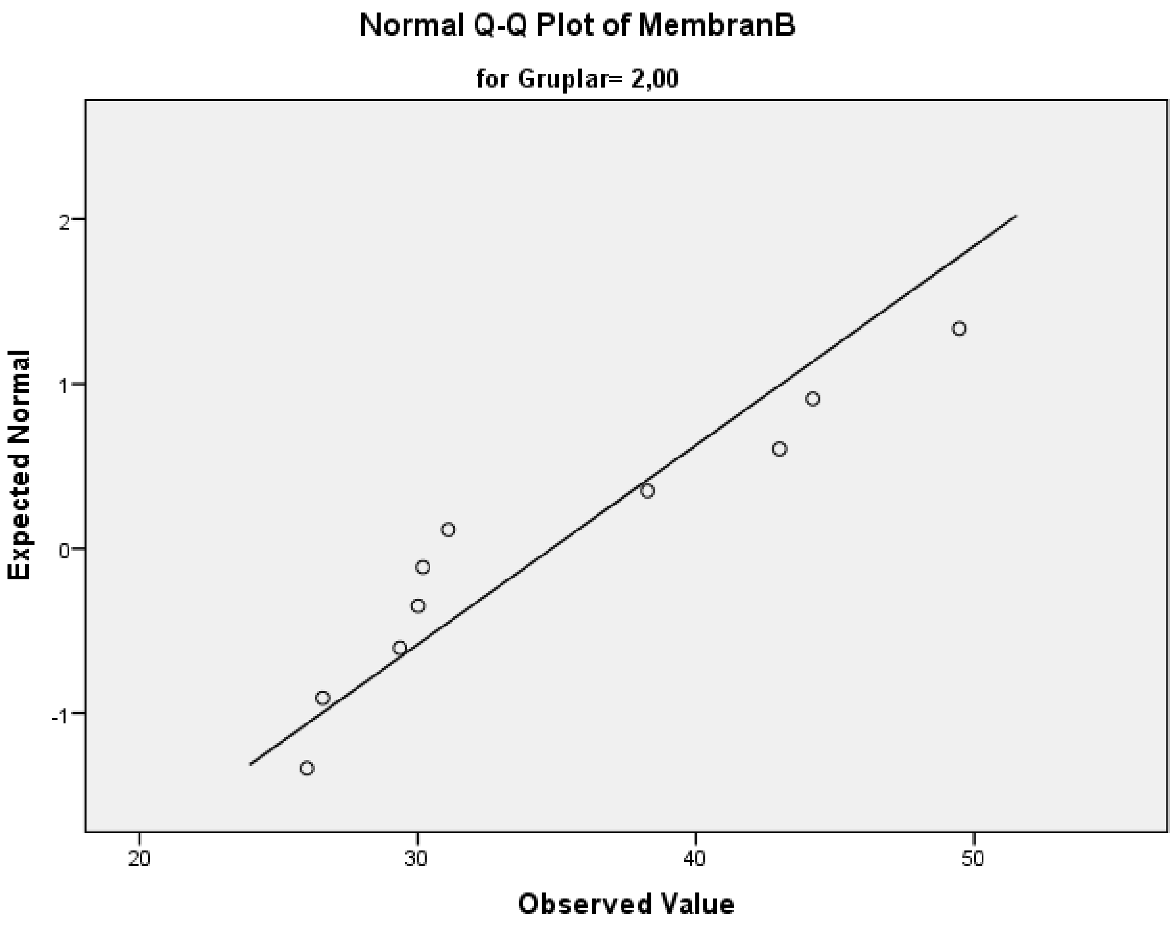
Normal Q-Q Plot of MembranB
for Gruplar= 2,00
Expected Normal
2
1
0
-1
20
30
40
50
Observed Value

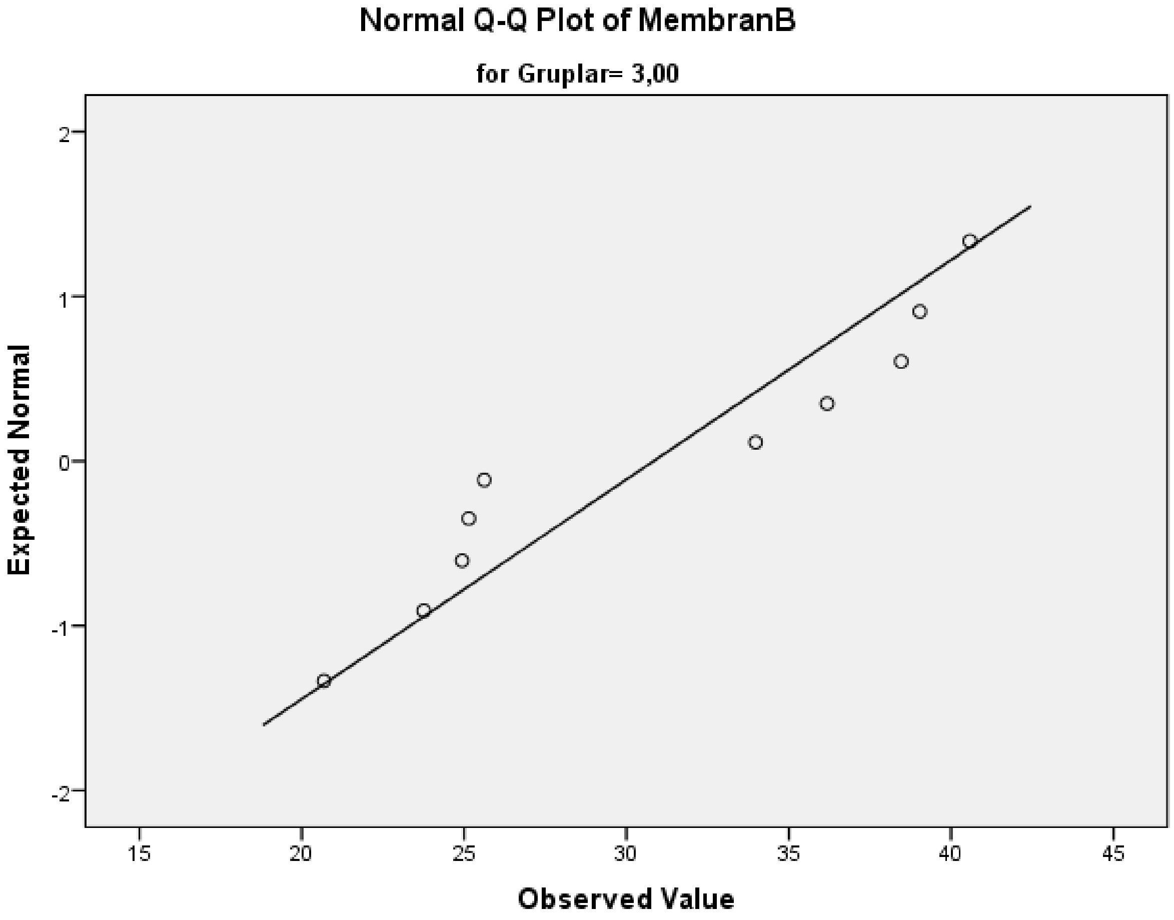
Normal Q-Q Plot of MembranB
for Gruplar= 3,00
Expected Normal
2
1
0
-1
-2
15
20
25
30
35
40
45
Observed Value

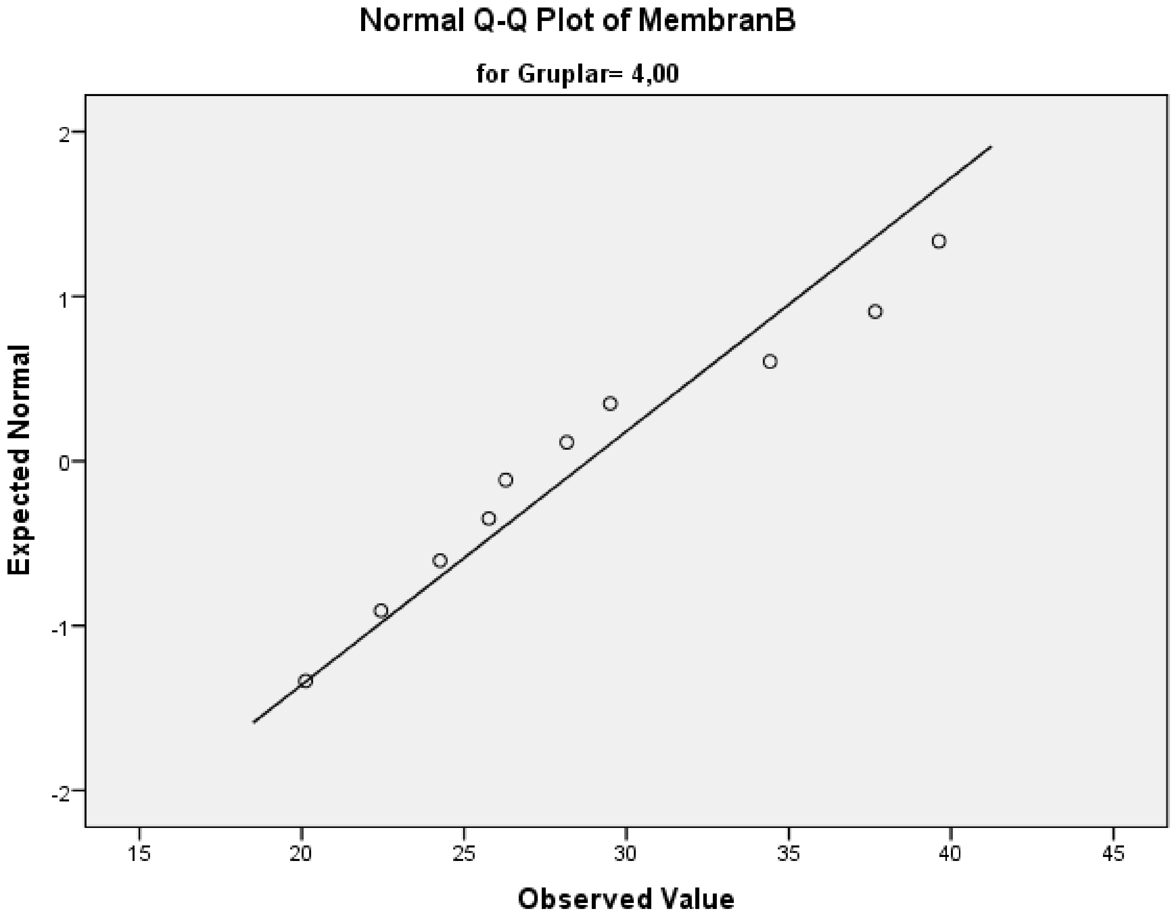
Normal Q-Q Plot of MembranB
for Gruplar= 4,00
Expected Normal
2
1
0
-1
-2
15
20
25
30
35
40
45
Observed Value

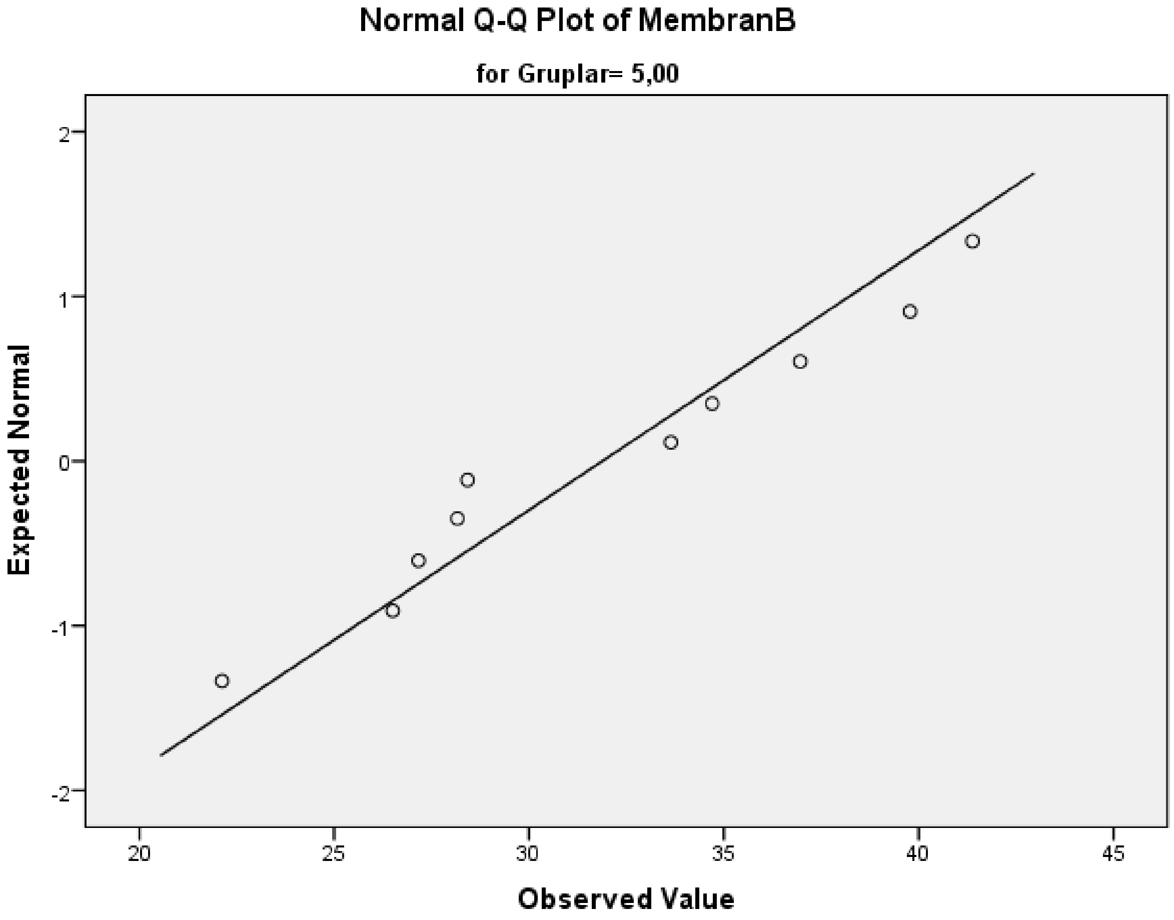
Normal Q-Q Plot of MembranB
for Gruplar= 5,00
Expected Normal
2
1
0
-1
-2
20
25
30
35
40
45
Observed Value

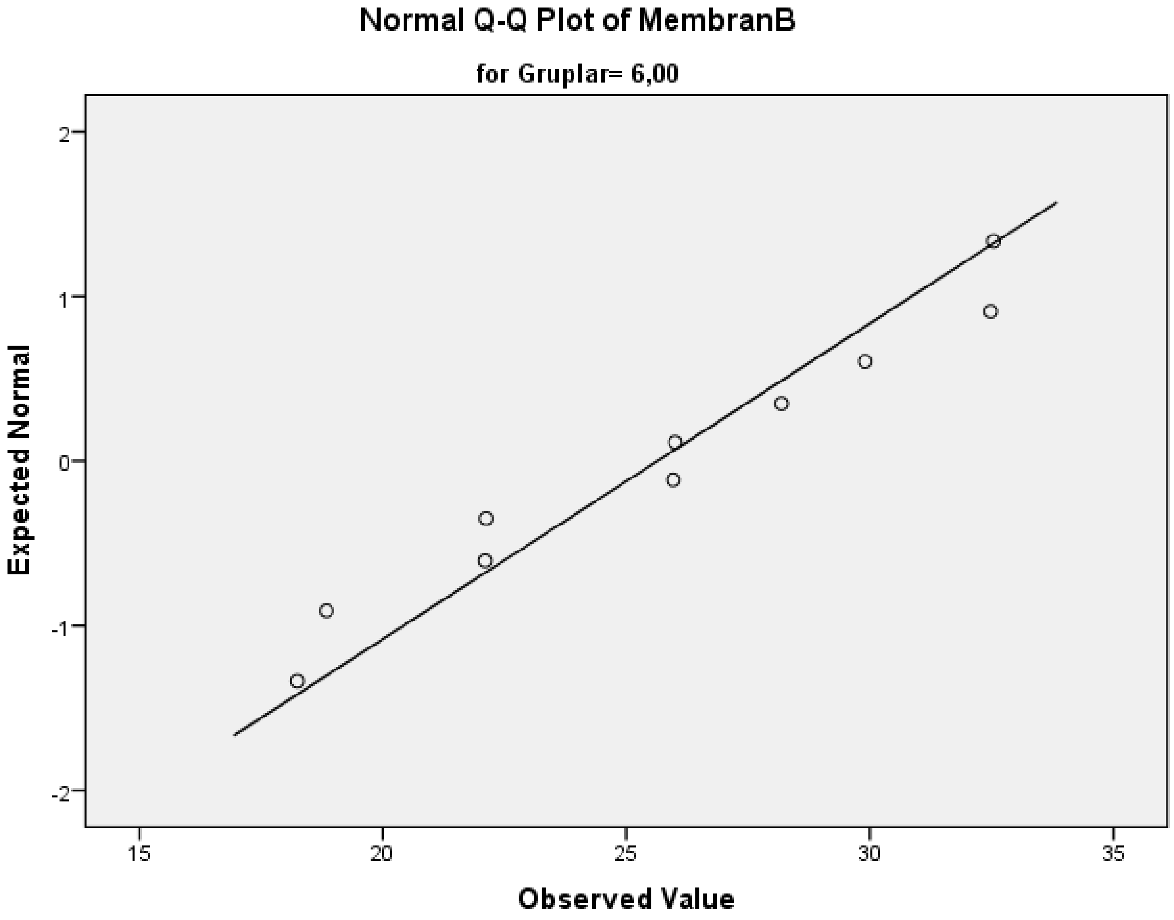
Normal Q-Q Plot of MembranB
for Gruplar= 6,00
Expected Normal
2
1
0
-1
-2
15
20
25
30
35
Observed Value

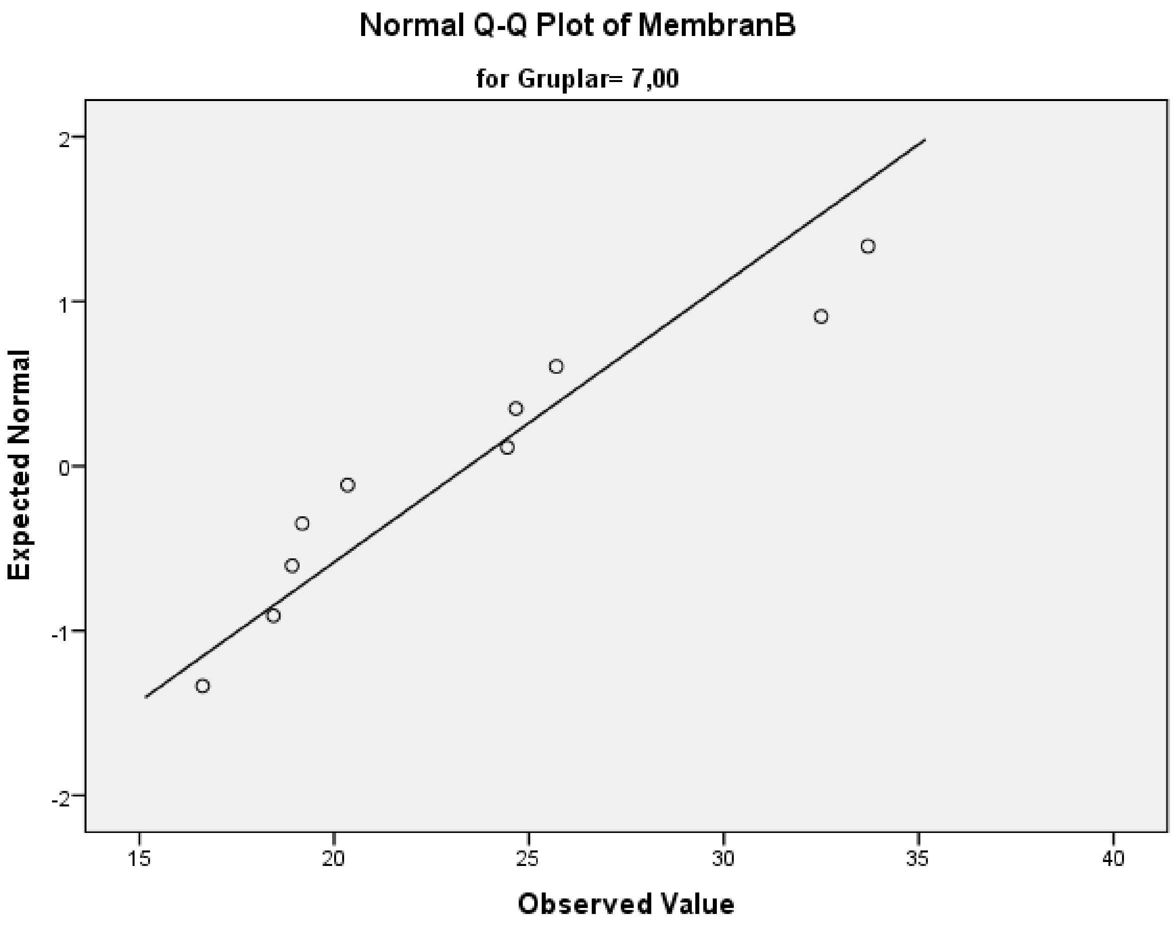
Normal Q-Q Plot of MembranB
for Gruplar= 7,00
Expected Normal
Observed Value
2
1
0
-1
-2
15
20
25
30
35
40

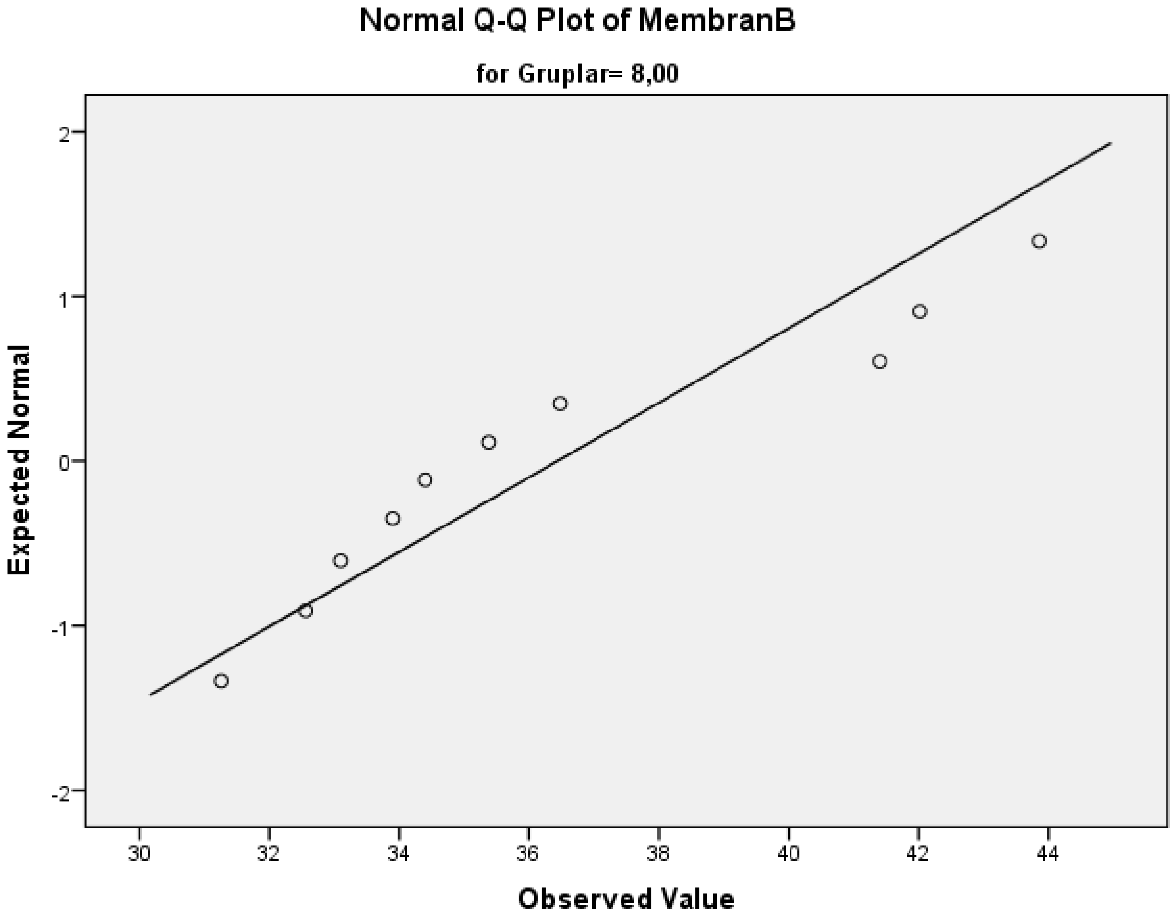

Normal Q-Q Plot of MembranB
for Gruplar= 8,00
Expected Normal
Observed Value
2
1
0
-1
-2
30
32
34
36
38
40
42
44

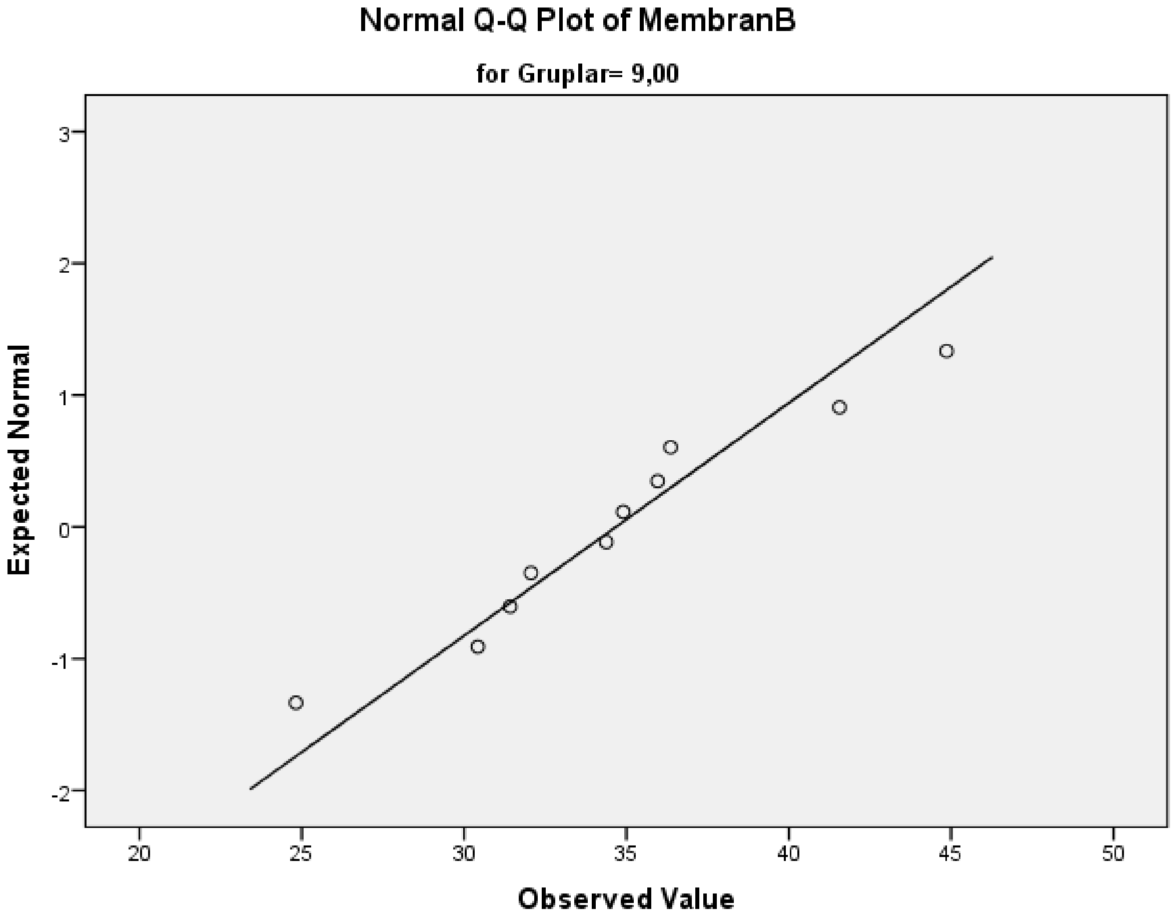
Normal Q-Q Plot of MembranB
for Gruplar= 9,00
Expected Normal
3
2
1
0
-1
-2
20
25
30
35
40
45
50
Observed Value

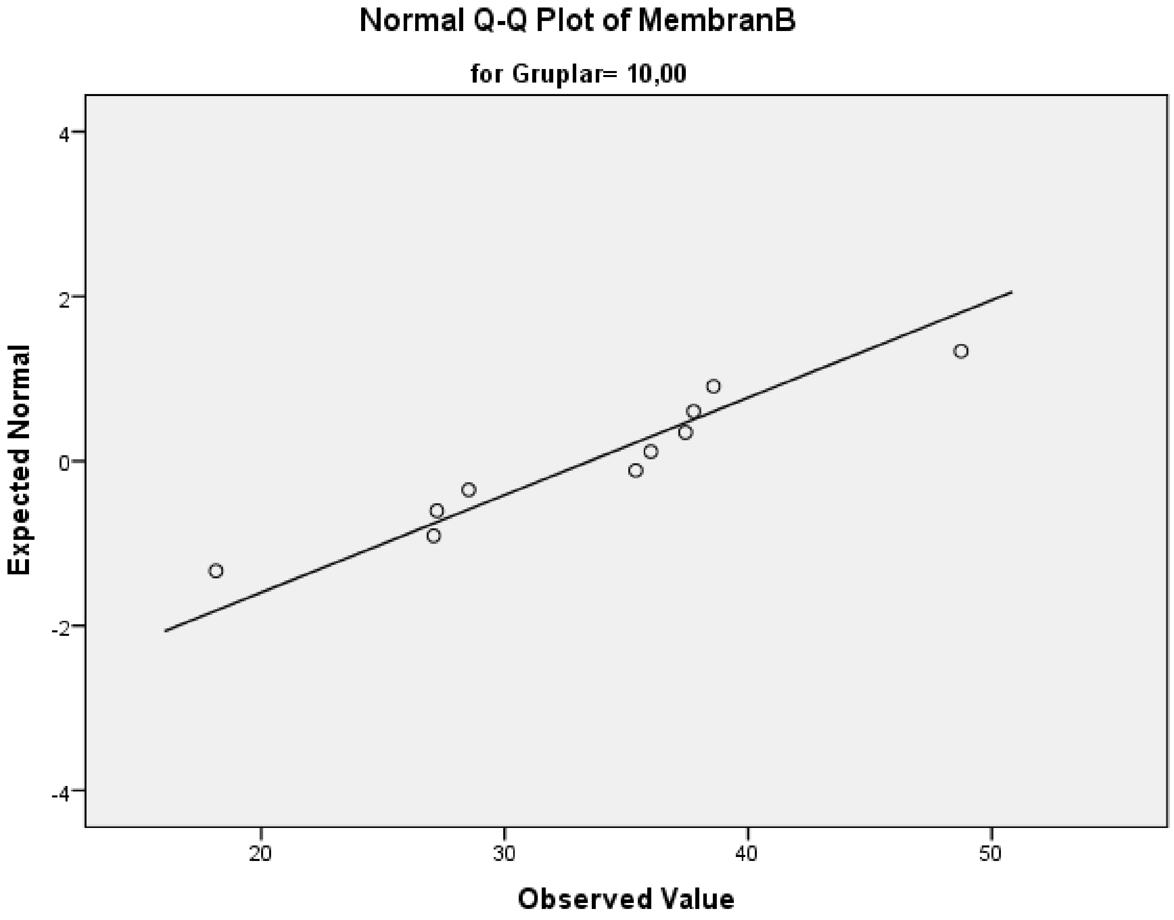

Detrended Normal Q-Q Plots

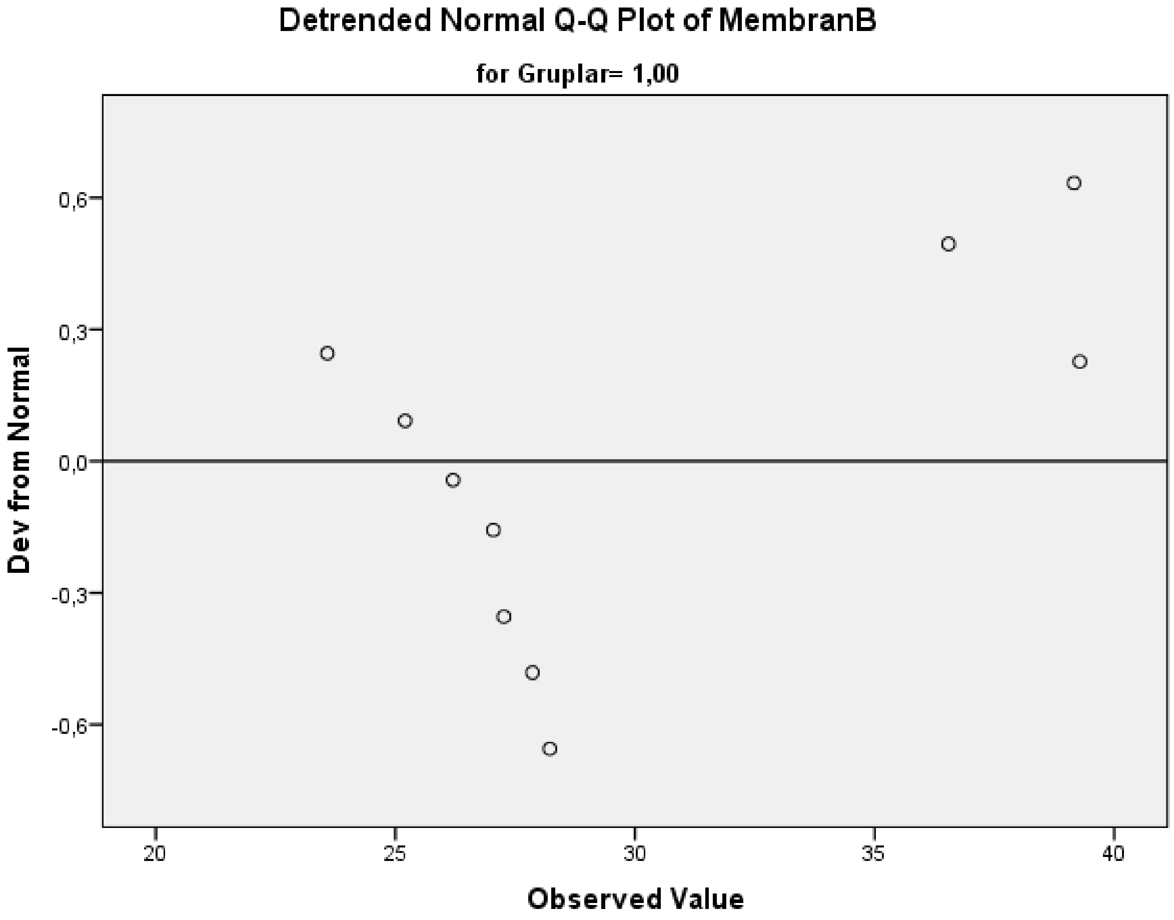

Detrended Normal Q-Q Plot of MembranB
for Gruplar= 1,00
Dev from Normal
0,6
0,3
0,0
-0,3
-0,6
20
25
30
35
40
Observed Value

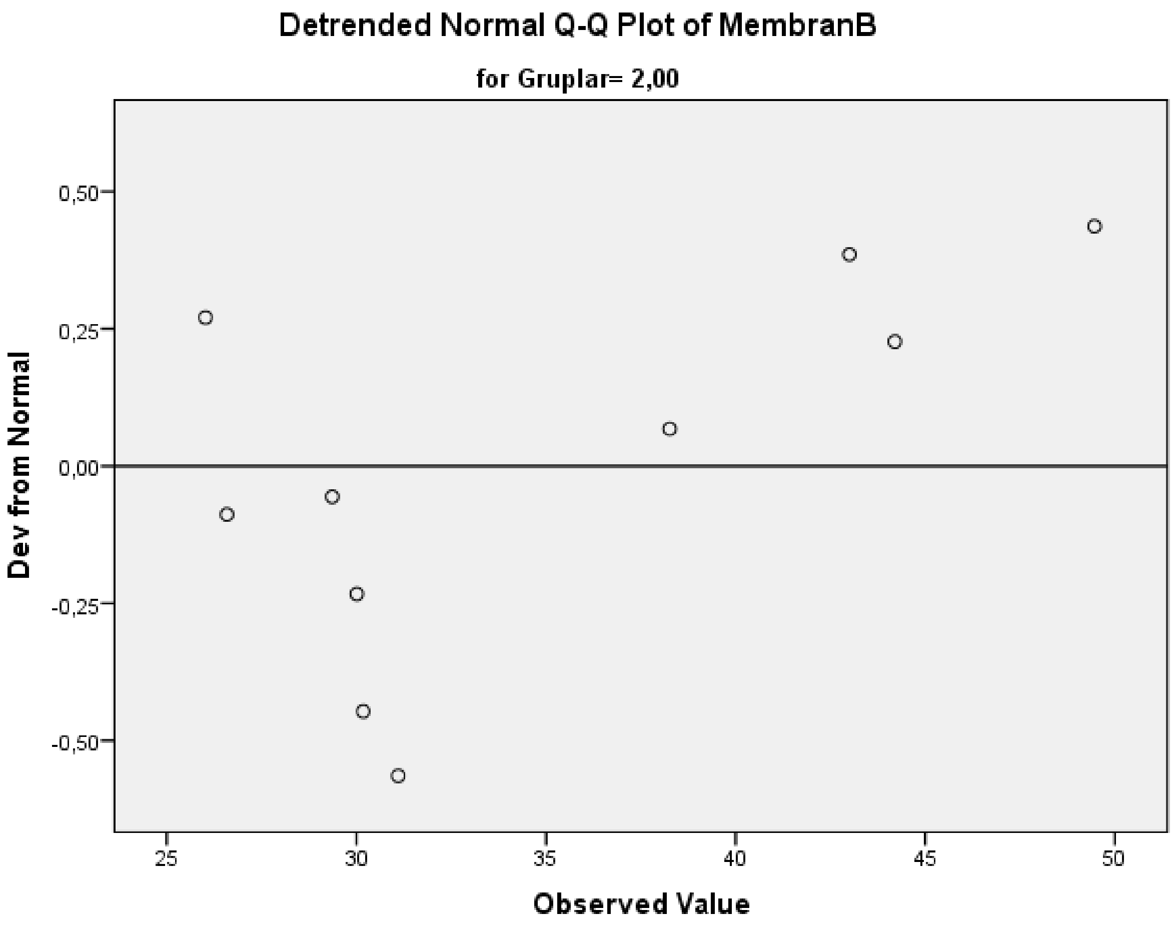
Detrended Normal Q-Q Plot of MembranB
for Gruplar= 2,00
Dev from Normal
0,50
0,25
0,00
-0,25
-0,50
25
30
35
40
45
50
Observed Value

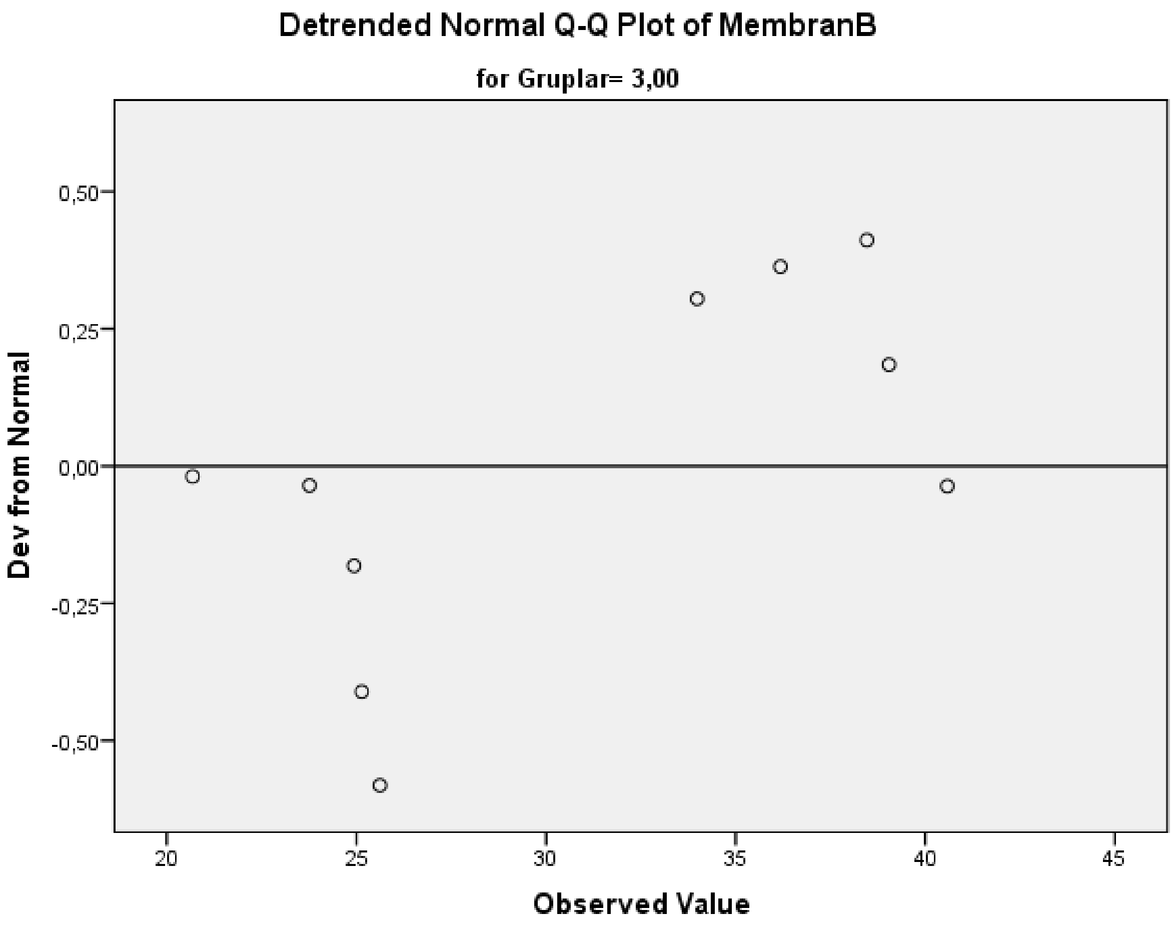
Detrended Normal Q-Q Plot of MembranB
for Gruplar= 3,00
Dev from Normal
0,50
0,25
0,00
-0,25
-0,50
20
25
30
35
40
45
Observed Value

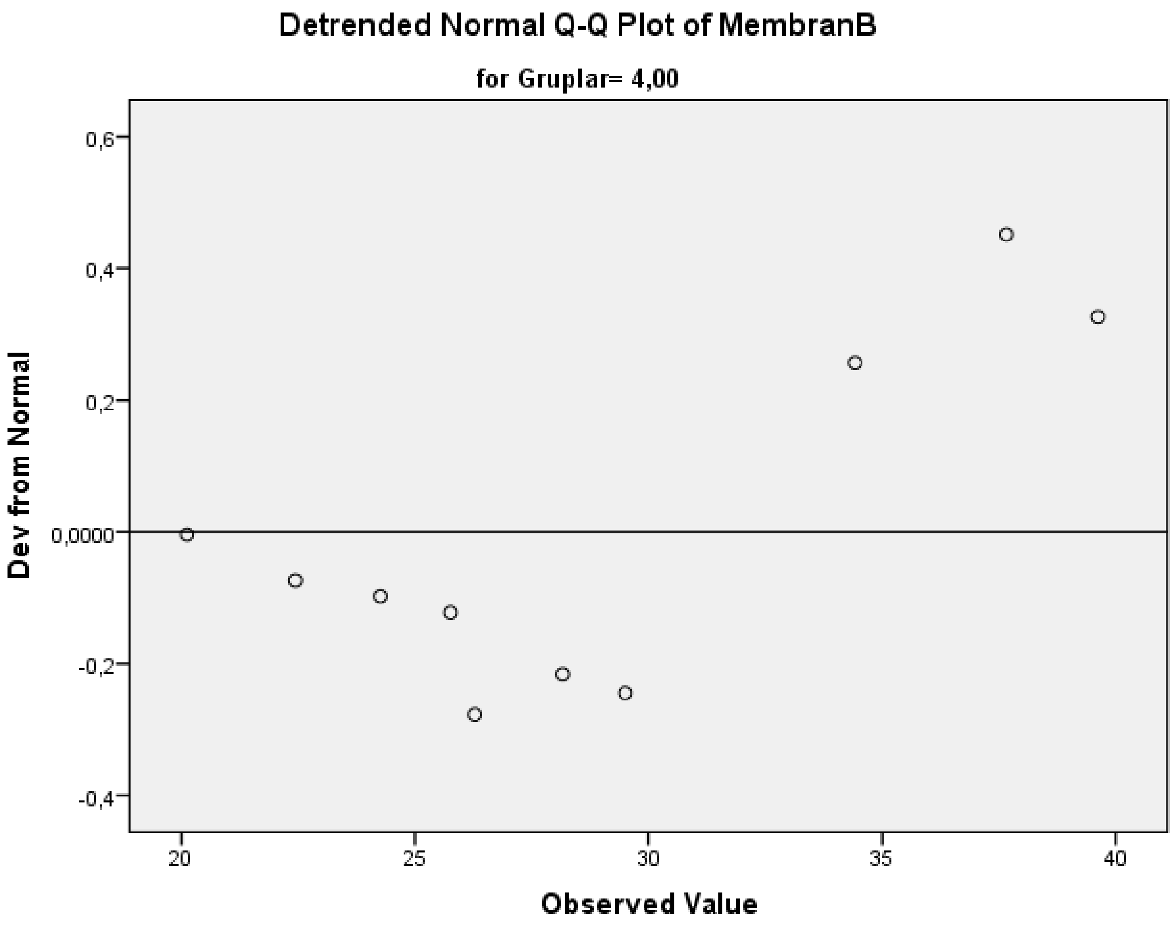

Detrended Normal Q-Q Plot of MembranB
for Gruplar= 4,00
Dev from Normal
0,6
0,4
0,2
0,0000
-0,2
-0,4
20
25
30
35
40
Observed Value

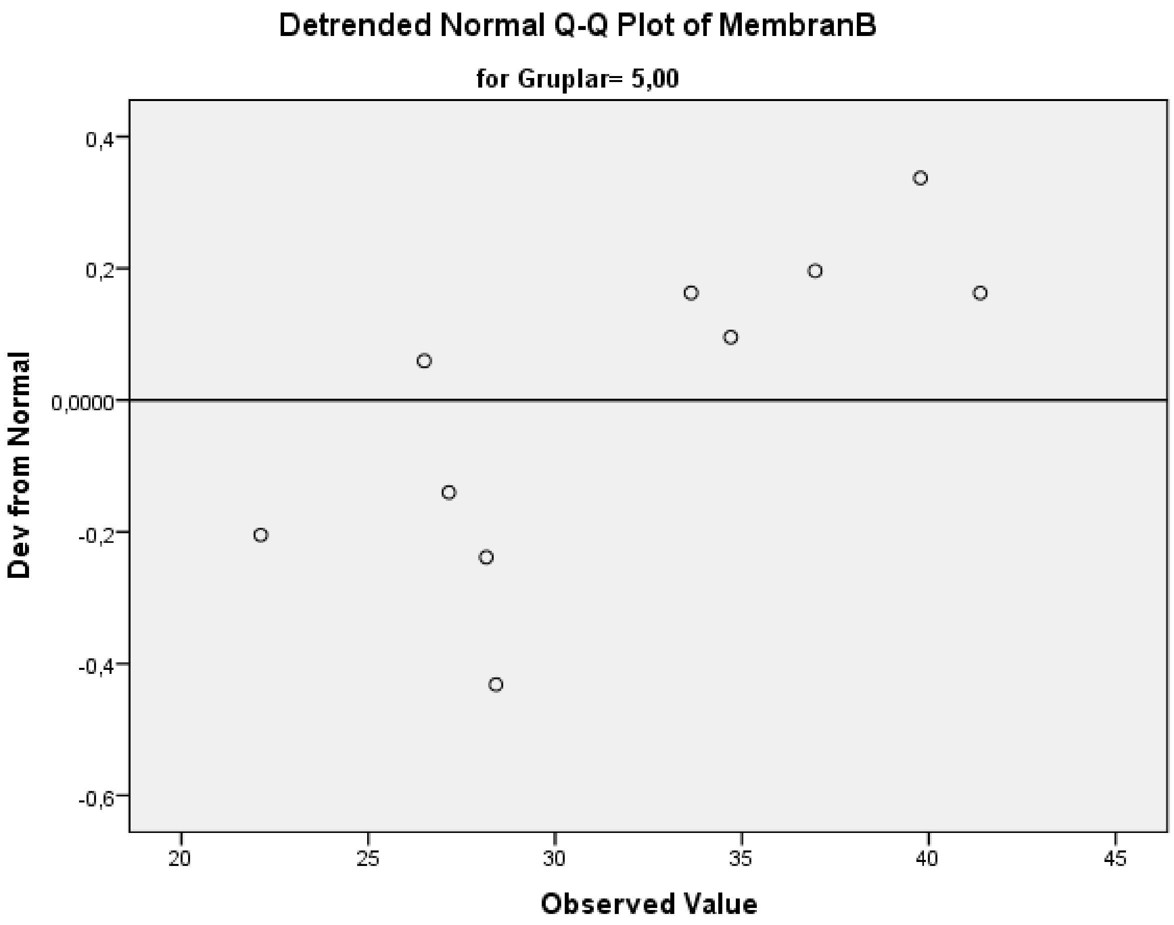

Detrended Normal Q-Q Plot of MembranB
for Gruplar= 5,00
Dev from Normal
0,4
0,2
0,0000
-0,2
-0,4
-0,6
20
25
30
35
40
45
Observed Value

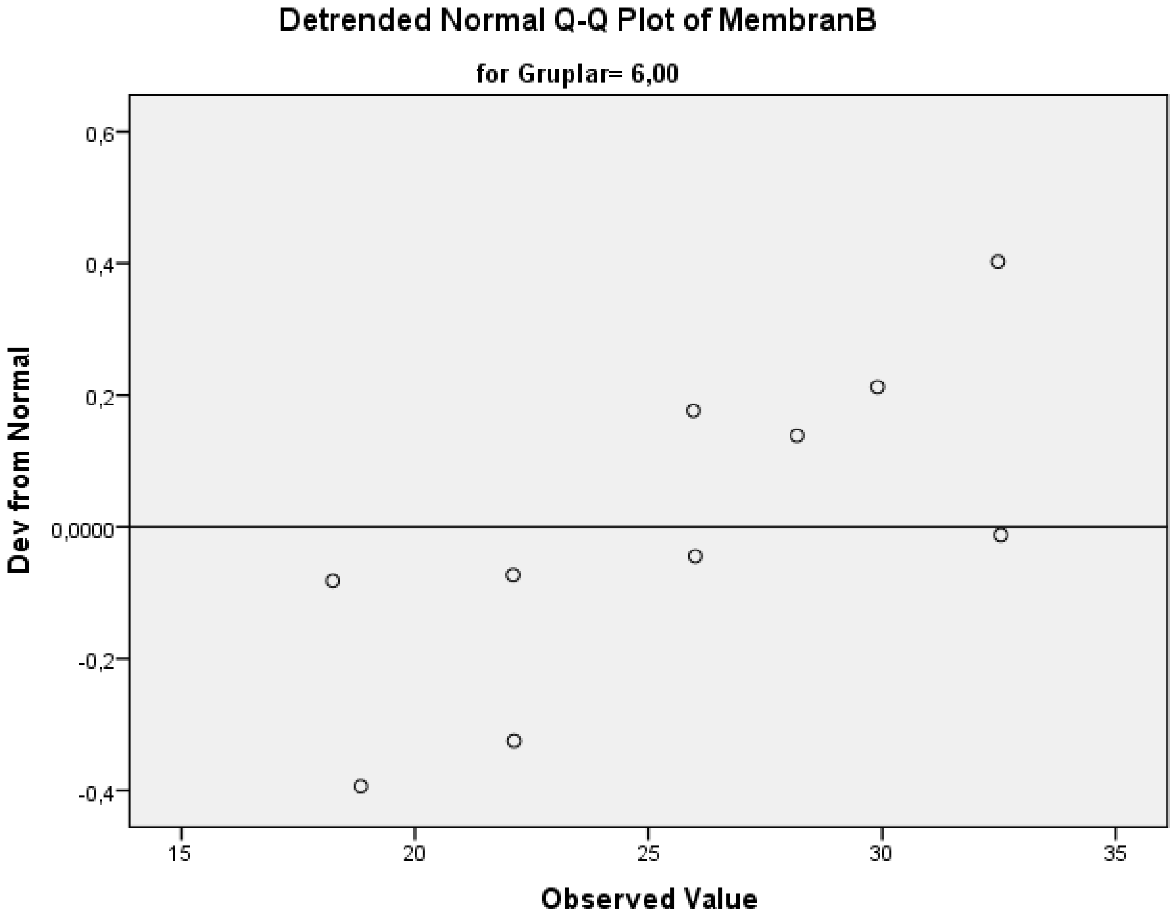
Detrended Normal Q-Q Plot of MembranB
for Gruplar= 6,00
Dev from Normal
0,6
0,4
0,2
0,0000
-0,2
-0,4
15
20
25
30
35
Observed Value

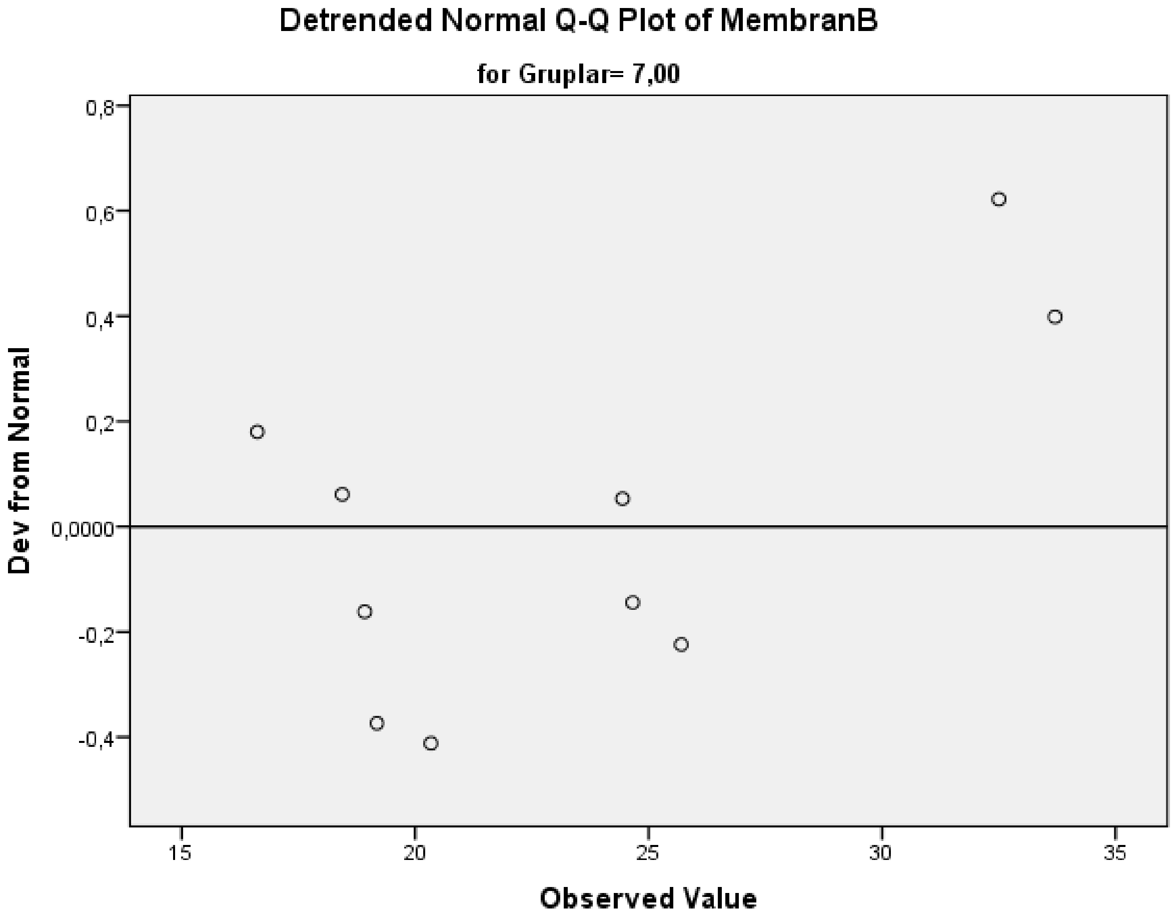
Detrended Normal Q-Q Plot of MembranB
for Gruplar= 7,00
Dev from Normal
0,8
0,6
0,4
0,2
0,0000
-0,2
-0,4
15
20
25
30
35
Observed Value

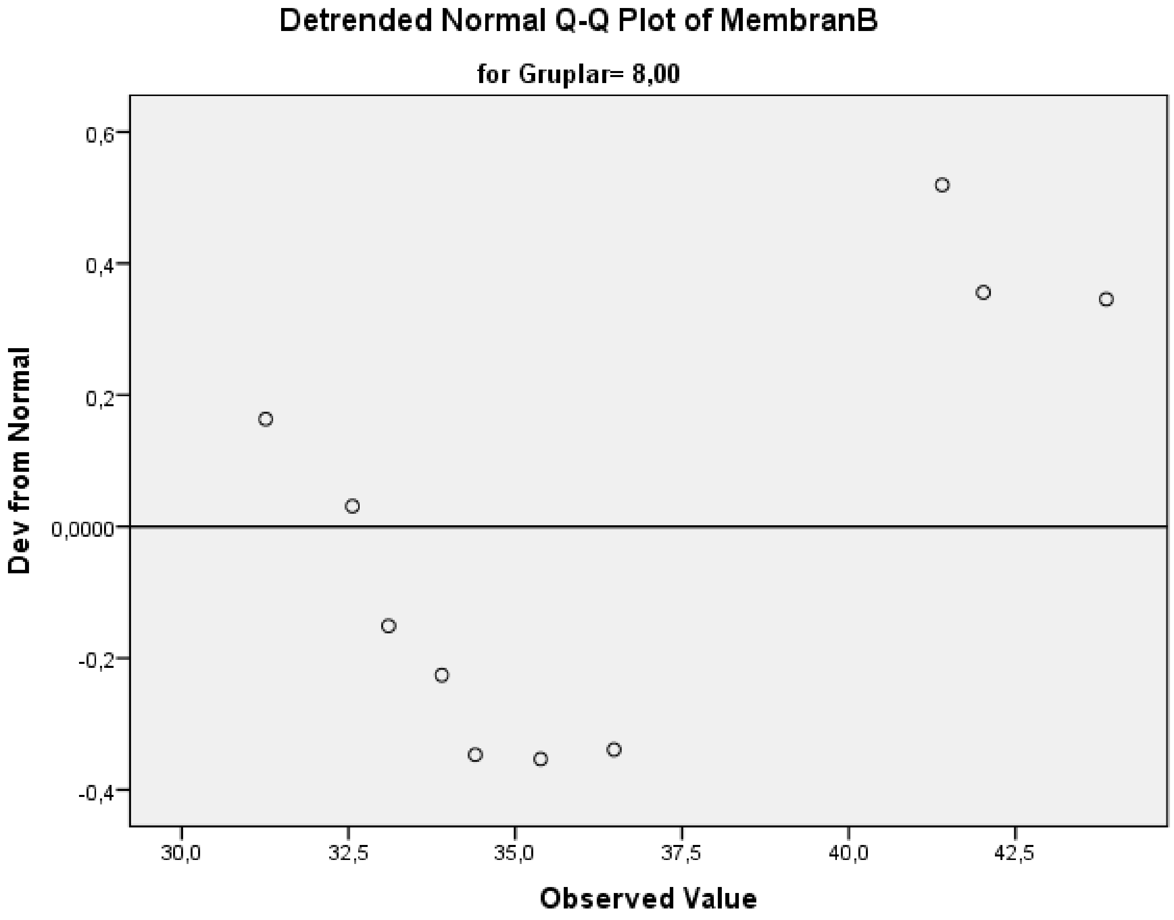
Detrended Normal Q-Q Plot of MembranB
for Gruplar= 8,00
Dev from Normal
0,6
0,4
0,2
0,0000
-0,2
-0,4
30,0
32,5
35,0
37,5
40,0
42,5
Observed Value

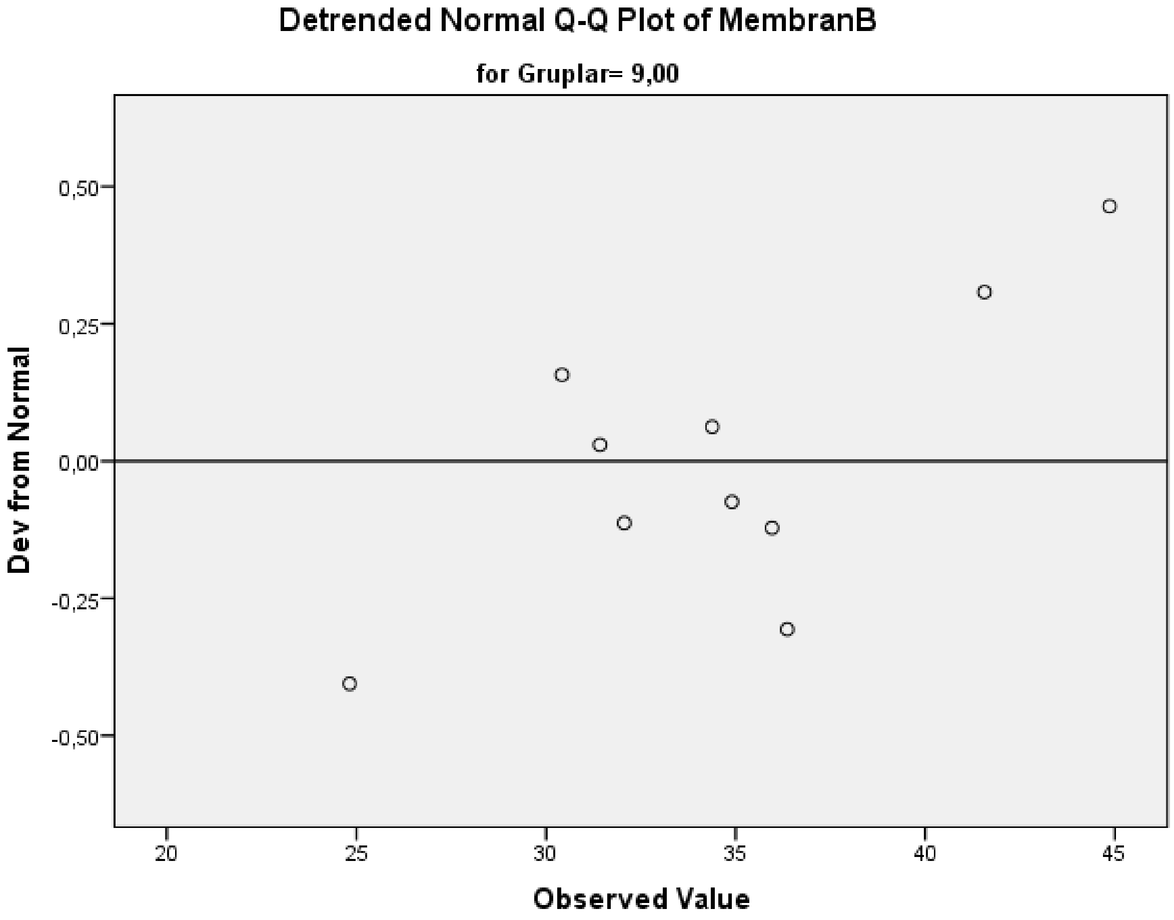

Detrended Normal Q-Q Plot of MembranB
for Gruplar= 9,00
Dev from Normal
0,50
0,25
0,00
-0,25
-0,50
20
25
30
35
40
45
Observed Value

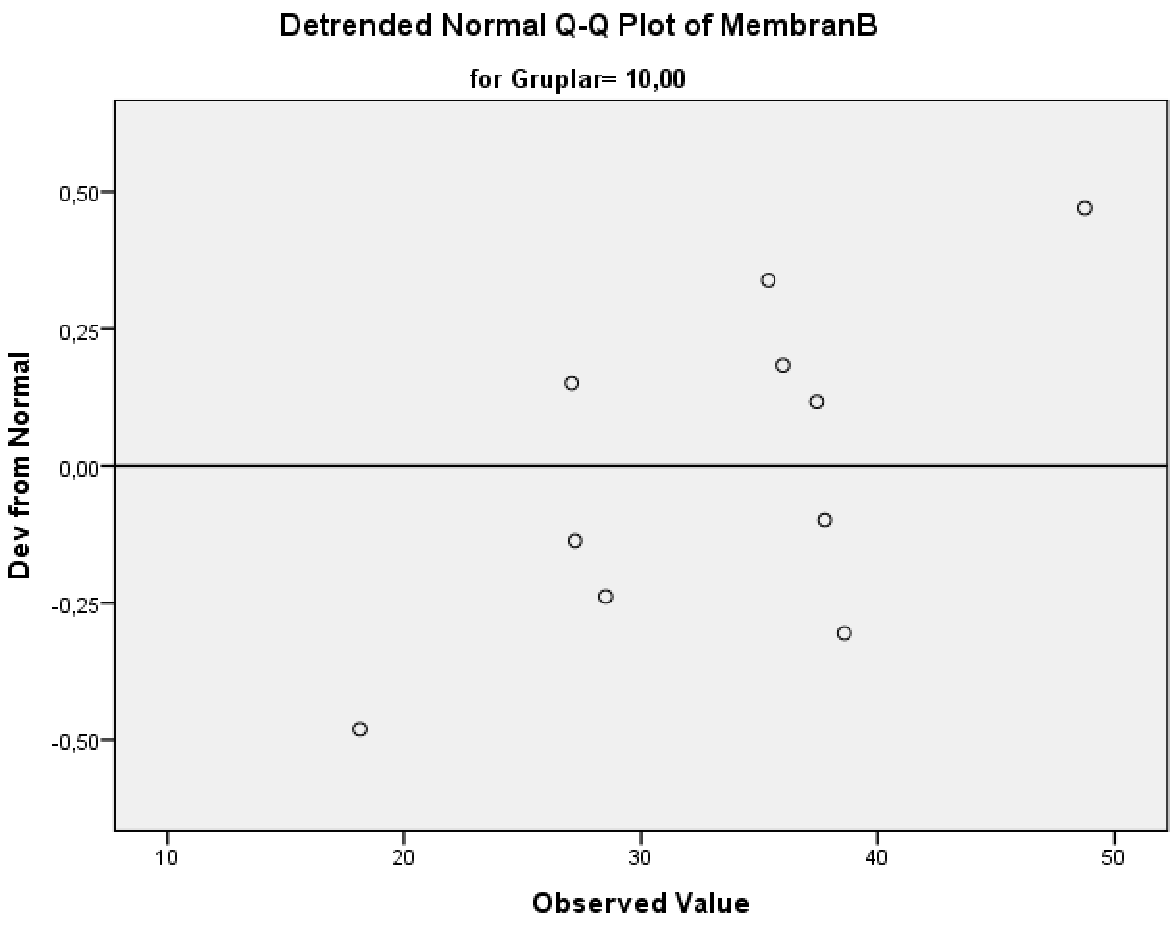
Detrended Normal Q-Q Plot of MembranB
for Gruplar= 10,00
Dev from Normal
0,50
0,25
0,00
-0,25
-0,50
10
20
30
40
50
Observed Value

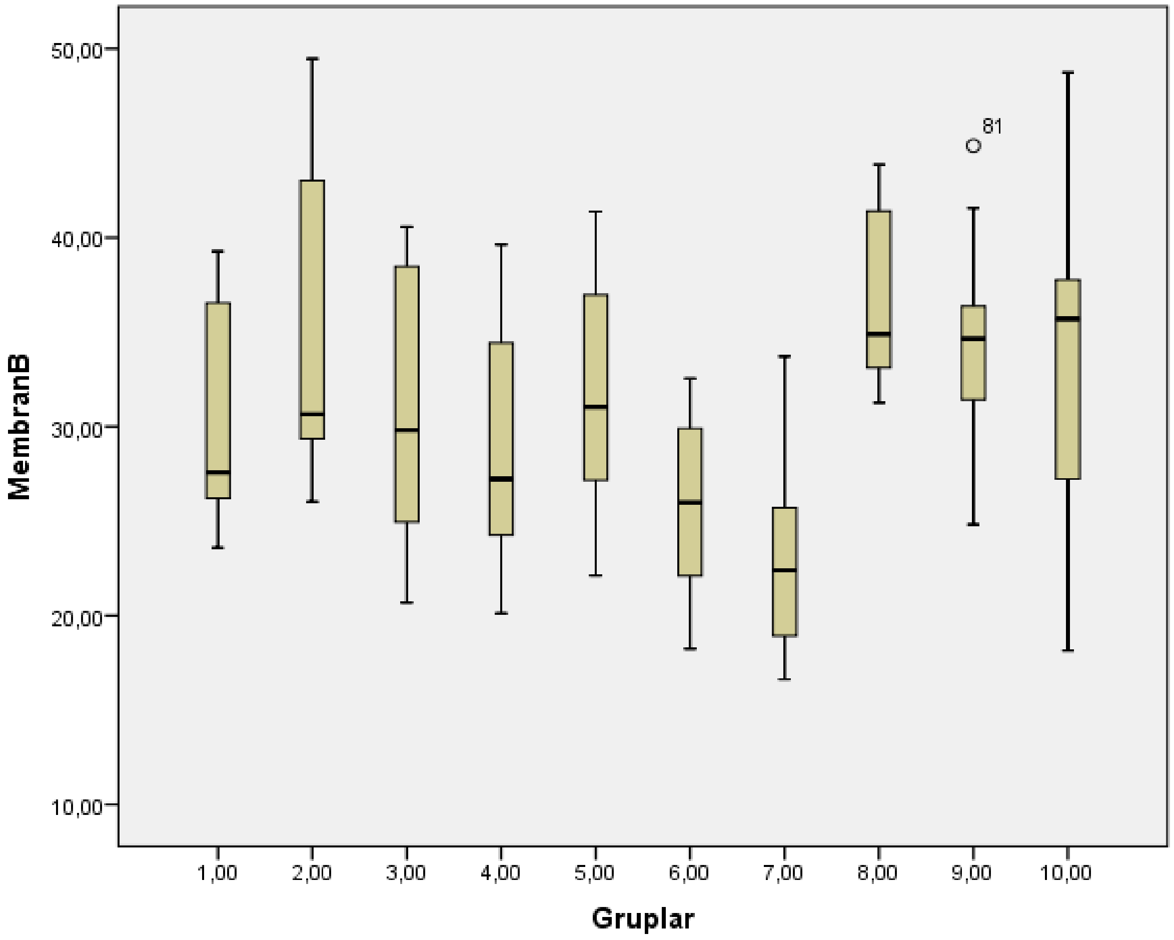


```
ONEWAY MembranB BY Gruplar
  /STATISTICS DESCRIPTIVES HOMOGENEITY
  /PLOT MEANS
  /MISSING ANALYSIS
  /POSTHOC=DUNCAN ALPHA(0.05).
```

Oneway

Notes

Output Created		24-DEC-2020 12:15:37
Comments		
Input	Active Dataset	DataSet0
	Filter	<none>
	Weight	<none>

	Split File	<none>
	N of Rows in Working Data File	100
Missing Value Handling	Definition of Missing	User-defined missing values are treated as missing.
	Cases Used	Statistics for each analysis are based on cases with no missing data for any variable in the analysis.
Syntax		ONEWAY MembranB BY Gruplar<br>/STATISTICS DESCRIPTIVES HOMOGENEITY<br>/PLOT MEANS<br>/MISSING ANALYSIS<br>/POSTHOC=DUNCAN ALPHA(0.05).
Resources	Processor Time	00:00:00,25
	Elapsed Time	00:00:00,38

30,0340
34,8170
30,8380
28,8220
31,8820
25,6360
23,4500
36,4360
34,6740
33,4840

Test of Homogeneity of Variances

MembranB

Levene Statistic	df1	df2	Sig.
1,429	9	90	,188

ANOVA

MembranB

	Sum of Squares	df	Mean Square	F	Sig.
Between Groups	1560,436	9	173,382	4,058	,000

Within Groups	3845,052	90	42,723		
Total	5405,488	99			

Post Hoc Tests

Homogeneous Subsets

MembranB

Duncan[a]

Gruplar	N	Subset for alpha = 0.05			
		1	2	3	4
7,00	10	23,4500			
6,00	10	25,6360	25,6360		
4,00	10	28,8220	28,8220	28,8220	
1,00	10		30,0340	30,0340	30,0340
3,00	10		30,8380	30,8380	30,8380
5,00	10		31,8820	31,8820	31,8820
10,00	10			33,4840	33,4840
9,00	10			34,6740	34,6740
2,00	10			34,8170	34,8170
8,00	10				36,4360
Sig.		,085	,058	,079	,060

Means for groups in homogeneous subsets are displayed.

a. Uses Harmonic Mean Sample Size = 10,000.

Means Plots

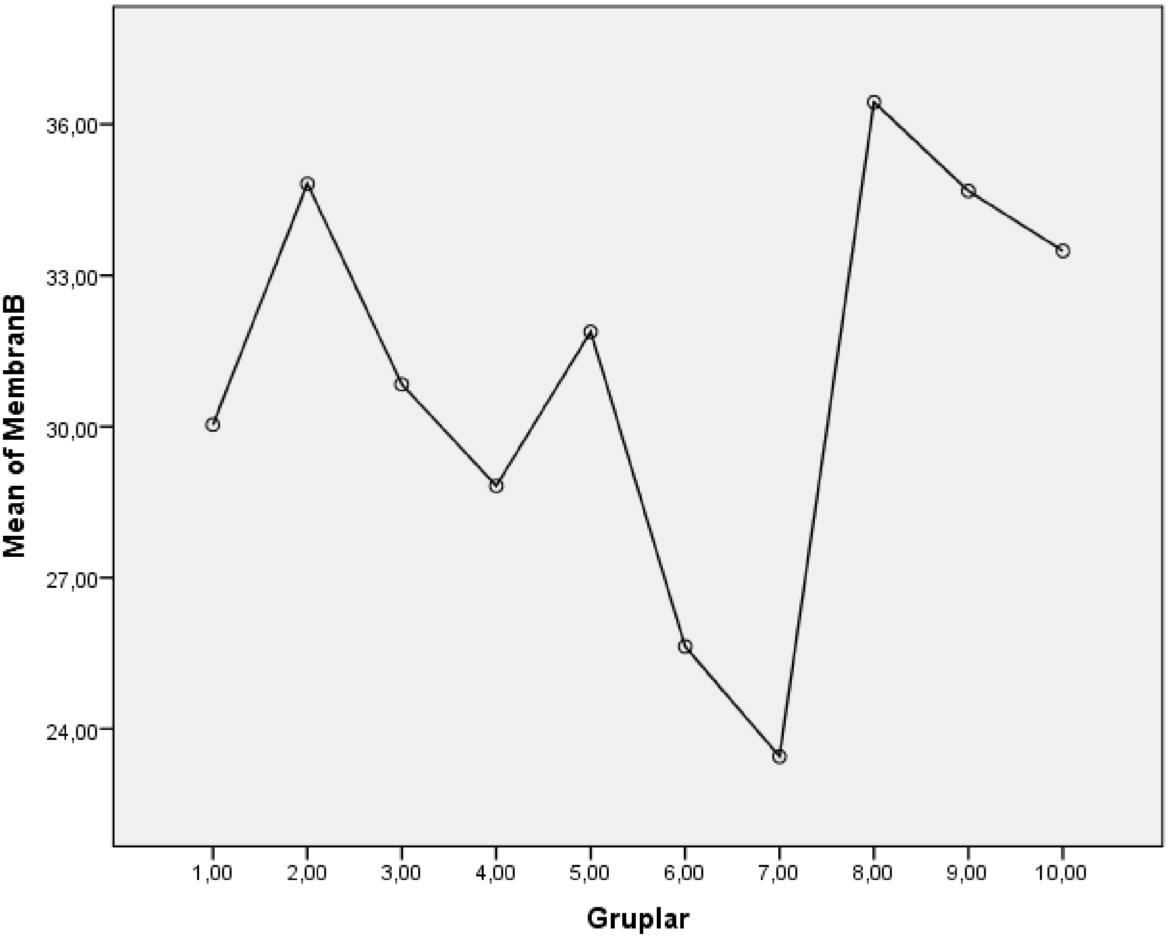

Mean of MembranB
36,00
33,00
30,00
27,00
24,00
1,00
2,00
3,00
4,00
5,00
6,00
7,00
8,00
9,00
10,00
Gruplar

Acrosome integrity-ANOVA

AkrozomB: Acrosome integrity
Groups
1: Control
2: RES10
3: RES20
4: RES40
5: CD10
6: CD20
7: CD40
8: RLC10
9: RLC20
10: RLC40

```
EXAMINE VARIABLES=AkrozomB BY Gruplar
  /PLOT BOXPLOT STEMLEAF HISTOGRAM NPPLOT
  /COMPARE GROUPS
  /STATISTICS DESCRIPTIVES
  /CINTERVAL 95
  /MISSING LISTWISE
  /NOTOTAL.
```

Explore

Notes

Output Created		24-DEC-2020 11:32:39
Comments		
Input	Active Dataset	DataSet0
	Filter	<none>
	Weight	<none>
	Split File	<none>
	N of Rows in Working Data File	103
Missing Value Handling	Definition of Missing	User-defined missing values for dependent variables are treated as missing.
	Cases Used	Statistics are based on cases with no missing values for any dependent variable or factor used.

Syntax		EXAMINE VARIABLES=AkrozomB BY Gruplar<br>/PLOT BOXPLOT STEMLEAF HISTOGRAM NPPLOT<br>/COMPARE GROUPS<br>/STATISTICS DESCRIPTIVES<br>/CINTERVAL 95<br>/MISSING LISTWISE<br>/NOTOTAL.
Resources	Processor Time	00:00:03,98
	Elapsed Time	00:00:03,99

Gruplar

Case Processing Summary

		Cases					
		Valid		Missing		Total	
	Gruplar	N	Percent	N	Percent	N	Percent
AkrozomB	1,00	7	70,0%	3	30,0%	10	100,0%
	2,00	10	100,0%	0	0,0%	10	100,0%
	3,00	10	100,0%	0	0,0%	10	100,0%
	4,00	10	100,0%	0	0,0%	10	100,0%
	5,00	10	100,0%	0	0,0%	10	100,0%
	6,00	10	100,0%	0	0,0%	10	100,0%
	7,00	10	100,0%	0	0,0%	10	100,0%
	8,00	10	100,0%	0	0,0%	10	100,0%
	9,00	10	100,0%	0	0,0%	10	100,0%
	10,00	10	100,0%	0	0,0%	10	100,0%

Descriptives

	Gruplar			Statistic	Std. Error
AkrozomB	1,00	Mean		30,9586	1,68781
		95% Confidence Interval for Mean	Lower Bound	26,8287	
			Upper Bound	35,0885	

	5% Trimmed Mean		30,8917	
	Median		29,5600	
	Variance		19,941	
	Std. Deviation		4,46552	
	Minimum		25,70	
	Maximum		37,42	
	Range		11,72	
	Interquartile Range		9,34	
	Skewness		,641	,794
	Kurtosis		-1,041	1,587
2,00	Mean		32,4320	2,01745
	95% Confidence Interval for Mean	Lower Bound	27,8682	
		Upper Bound	36,9958	
	5% Trimmed Mean		32,4172	
	Median		34,0050	
	Variance		40,701	
	Std. Deviation		6,37975	
	Minimum		21,65	
	Maximum		43,48	
	Range		21,83	
	Interquartile Range		9,13	
	Skewness		-,057	,687
	Kurtosis		-,209	1,334
3,00	Mean		33,7010	1,60926
	95% Confidence Interval for Mean	Lower Bound	30,0606	
		Upper Bound	37,3414	
	5% Trimmed Mean		33,7544	
	Median		34,0750	
	Variance		25,897	
	Std. Deviation		5,08893	
	Minimum		26,13	
	Maximum		40,31	
	Range		14,18	
	Interquartile Range		9,86	
	Skewness		-,109	,687
	Kurtosis		-1,322	1,334
4,00	Mean		33,5590	1,68955
	95% Confidence Interval for	Lower Bound	29,7370	

	Mean	Upper Bound	37,3810	
	5% Trimmed Mean		33,4939	
	Median		35,5150	
	Variance		28,546	
	Std. Deviation		5,34284	
	Minimum		25,87	
	Maximum		42,42	
	Range		16,55	
	Interquartile Range		8,75	
	Skewness		-,107	,687
	Kurtosis		-,853	1,334
5,00	Mean		29,5860	1,87628
	95% Confidence Interval for Mean	Lower Bound	25,3416	
		Upper Bound	33,8304	
	5% Trimmed Mean		29,6378	
	Median		28,9400	
	Variance		35,204	
	Std. Deviation		5,93331	
	Minimum		20,75	
	Maximum		37,49	
	Range		16,74	
	Interquartile Range		11,08	
	Skewness		-,113	,687
	Kurtosis		-1,341	1,334
6,00	Mean		28,7830	1,44949
	95% Confidence Interval for Mean	Lower Bound	25,5040	
		Upper Bound	32,0620	
	5% Trimmed Mean		28,4878	
	Median		27,3850	
	Variance		21,010	
	Std. Deviation		4,58370	
	Minimum		24,70	
	Maximum		38,18	
	Range		13,48	
	Interquartile Range		6,26	
	Skewness		1,328	,687
	Kurtosis		,889	1,334
7,00	Mean		25,9430	1,47521

	95% Confidence Interval for Mean	Lower Bound	22,6058	
		Upper Bound	29,2802	
	5% Trimmed Mean		25,5111	
	Median		24,4250	
	Variance		21,763	
	Std. Deviation		4,66503	
	Minimum		22,28	
	Maximum		37,38	
	Range		15,10	
	Interquartile Range		5,65	
	Skewness		1,883	,687
	Kurtosis		3,863	1,334
8,00	Mean		29,3000	2,80841
	95% Confidence Interval for Mean	Lower Bound	22,9469	
		Upper Bound	35,6531	
	5% Trimmed Mean		29,3256	
	Median		30,3250	
	Variance		78,872	
	Std. Deviation		8,88098	
	Minimum		16,09	
	Maximum		42,05	
	Range		25,96	
	Interquartile Range		16,38	
	Skewness		-,170	,687
	Kurtosis		-1,034	1,334
9,00	Mean		34,0160	1,79644
	95% Confidence Interval for Mean	Lower Bound	29,9522	
		Upper Bound	38,0798	
	5% Trimmed Mean		33,7494	
	Median		34,5050	
	Variance		32,272	
	Std. Deviation		5,68085	
	Minimum		26,82	
	Maximum		46,01	
	Range		19,19	
	Interquartile Range		7,09	
	Skewness		,805	,687
	Kurtosis		1,123	1,334

	10,00	Mean		32,8640	2,76172
		95% Confidence Interval for Mean	Lower Bound	26,6166	
			Upper Bound	39,1114	
		5% Trimmed Mean		32,7644	
		Median		31,2450	
		Variance		76,271	
		Std. Deviation		8,73333	
		Minimum		20,03	
		Maximum		47,49	
		Range		27,46	
		Interquartile Range		13,43	
		Skewness		,466	,687
		Kurtosis		-,547	1,334

Tests of Normality

		Kolmogorov-Smirnov[a]			Shapiro-Wilk		
	Gruplar	Statistic	df	Sig.	Statistic	df	Sig.
AkrozomB	1,00	,219	7	,200*	,898	7	,317
	2,00	,172	10	,200*	,966	10	,853
	3,00	,131	10	,200*	,943	10	,586
	4,00	,226	10	,161	,927	10	,420
	5,00	,153	10	,200*	,940	10	,553
	6,00	,227	10	,156	,823	10	,028
	7,00	,216	10	,200*	,786	10	,010
	8,00	,152	10	,200*	,945	10	,615
	9,00	,173	10	,200*	,935	10	,501
	10,00	,127	10	,200*	,958	10	,766

*. This is a lower bound of the true significance.

a. Lilliefors Significance Correction

AkrozomB

Histograms

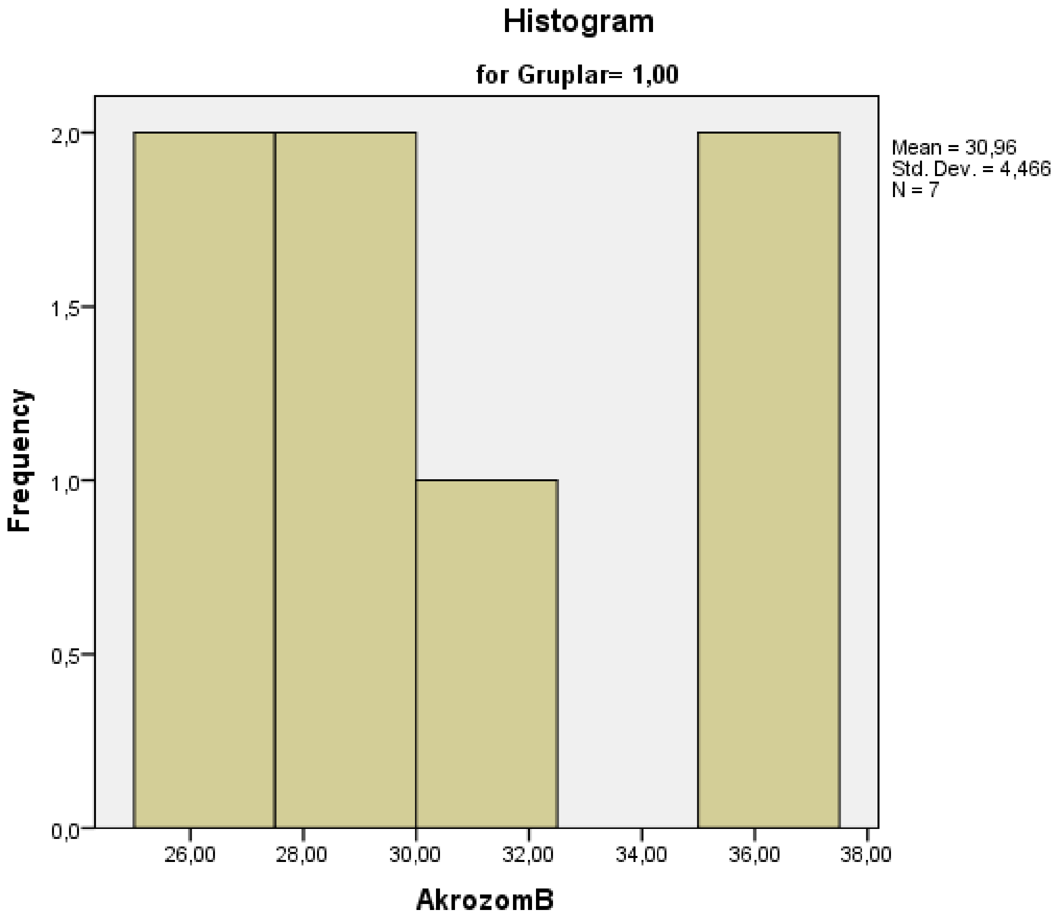

Histogram
for Gruplar= 1,00
Mean = 30,96
Std. Dev. = 4,466
N = 7
Frequency
2,0
1,5
1,0
0,5
0,0
26,00
28,00
30,00
32,00
34,00
36,00
38,00
AkrozomB

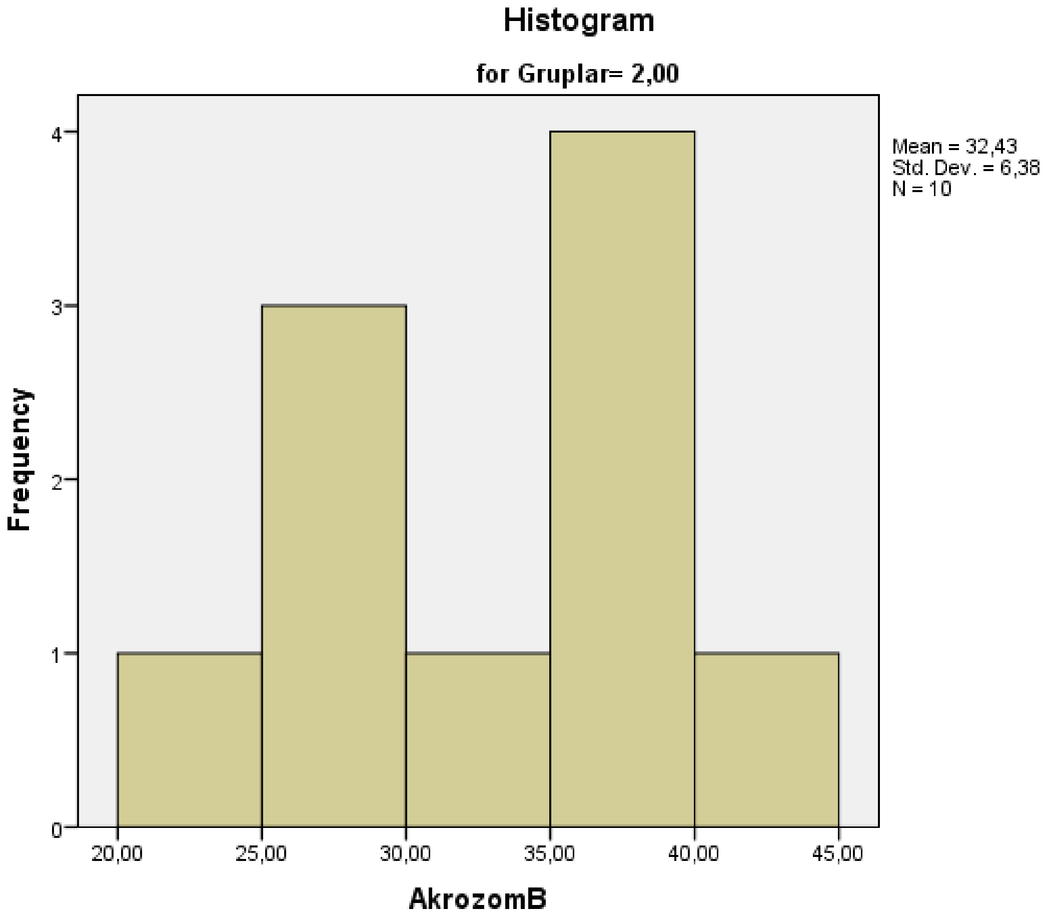
Histogram
for Gruplar= 2,00
Mean = 32,43
Std. Dev. = 6,38
N = 10
Frequency
0
1
2
3
4
20,00
25,00
30,00
35,00
40,00
45,00
AkrozomB

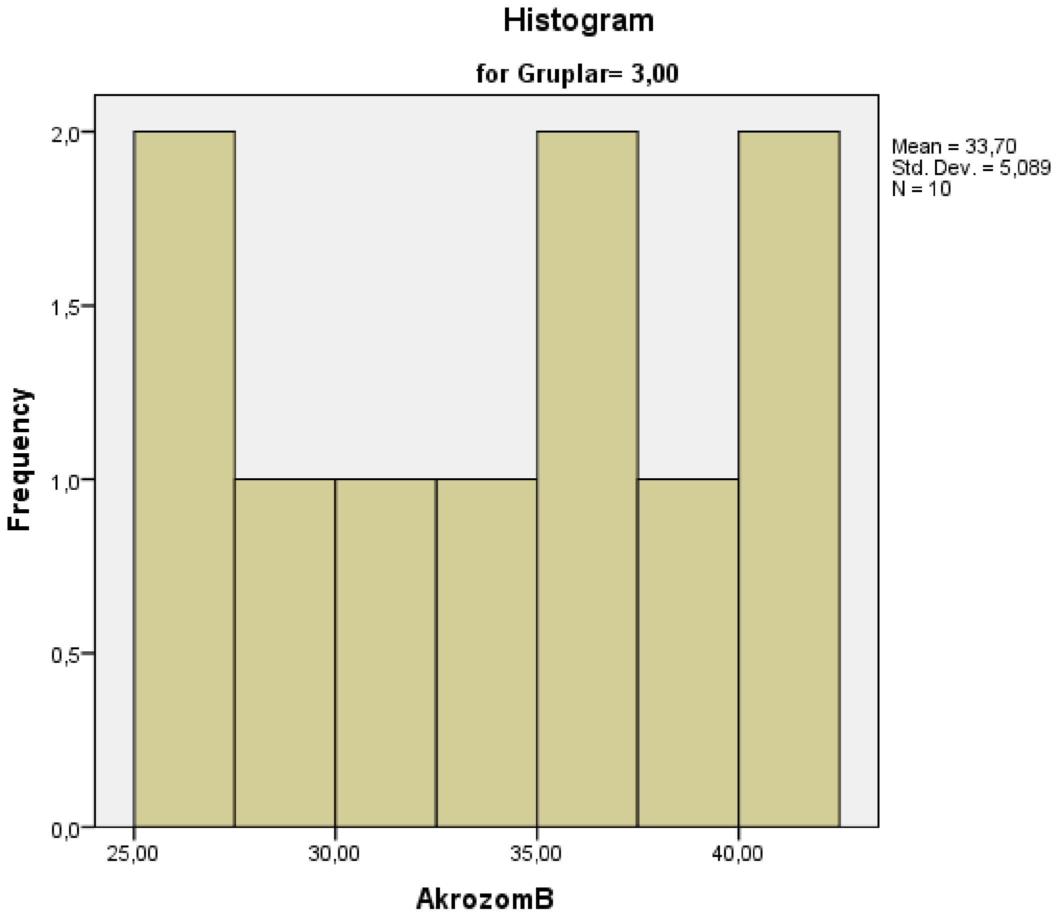

Histogram
for Gruplar= 3,00
Mean = 33,70
Std. Dev. = 5,089
N = 10
Frequency
2,0
1,5
1,0
0,5
0,0
25,00
30,00
35,00
40,00
AkrozomB

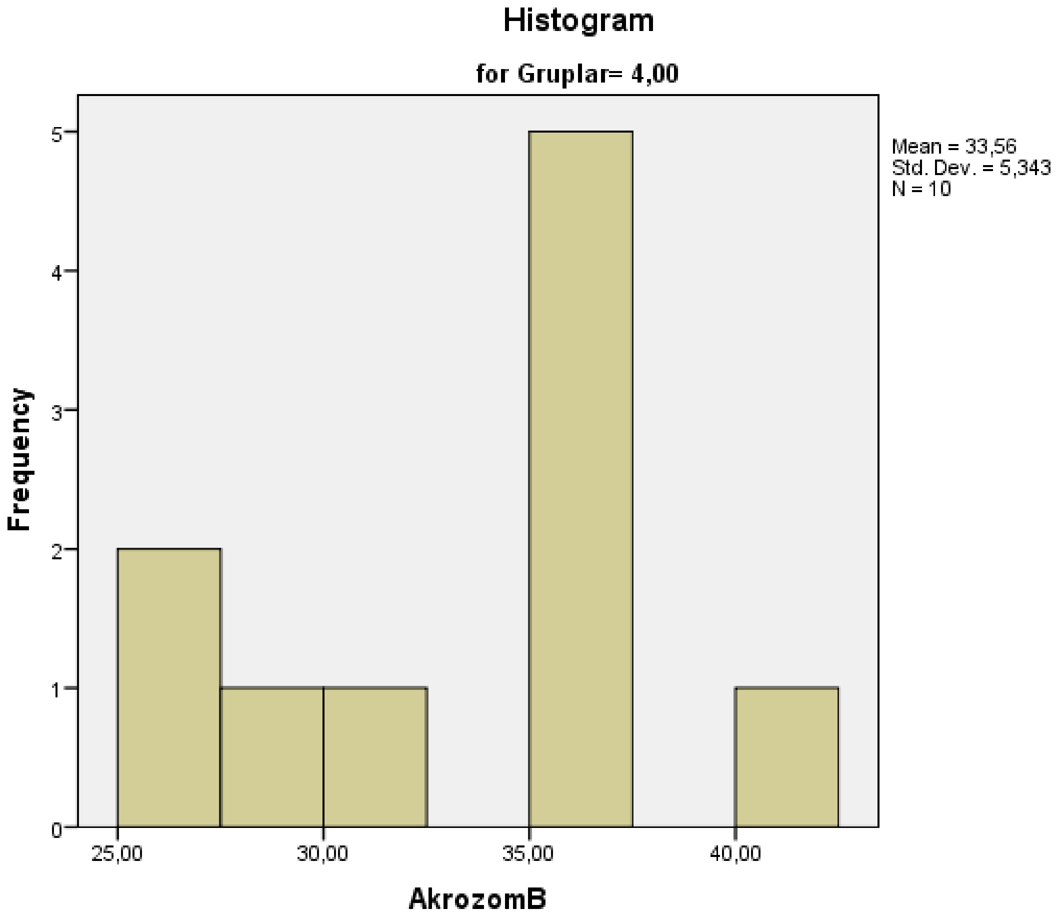

Histogram
for Gruplar= 4,00
Mean = 33,56
Std. Dev. = 5,343
N = 10
Frequency
0
1
2
3
4
5
25,00
30,00
35,00
40,00
AkrozomB

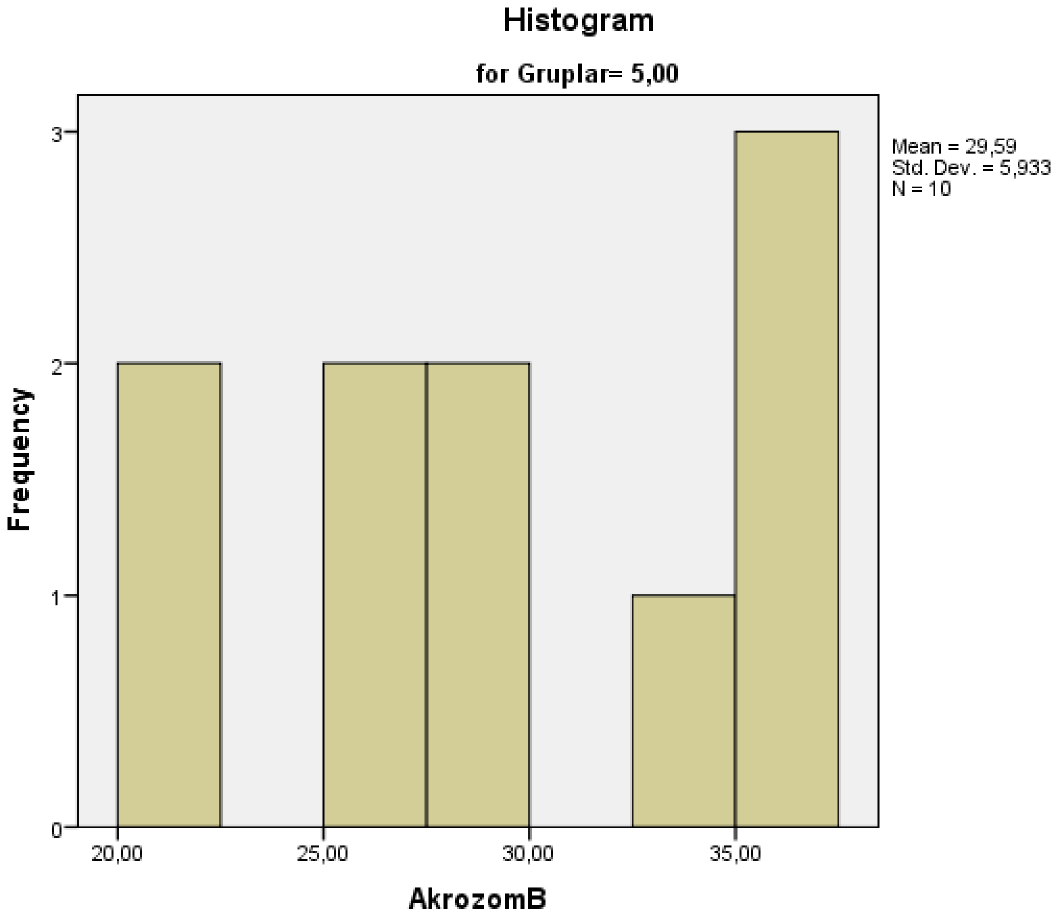
Histogram
for Gruplar= 5,00
Mean = 29,59
Std. Dev. = 5,933
N = 10
Frequency
0
1
2
3
20,00
25,00
30,00
35,00
AkrozomB

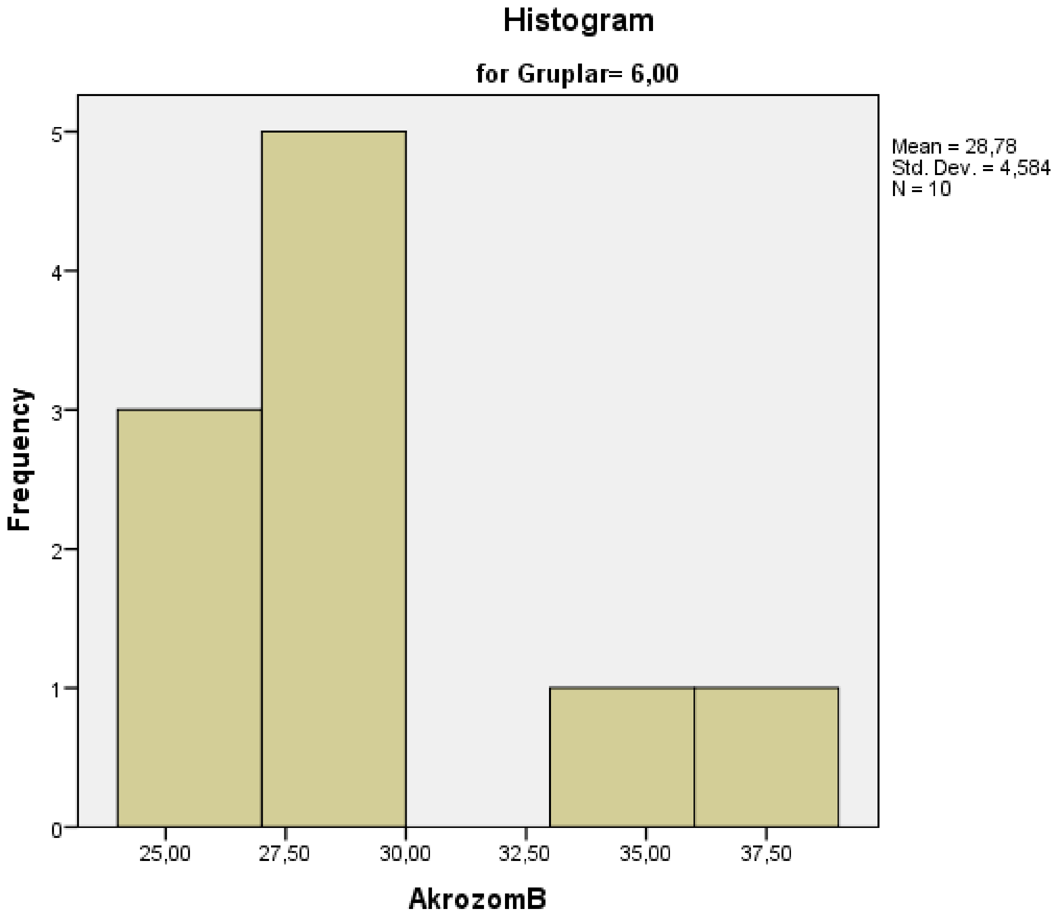
Histogram
for Gruplar= 6,00
Mean = 28,78
Std. Dev. = 4,584
N = 10
Frequency
0
1
2
3
4
5
25,00
27,50
30,00
32,50
35,00
37,50
AkrozomB

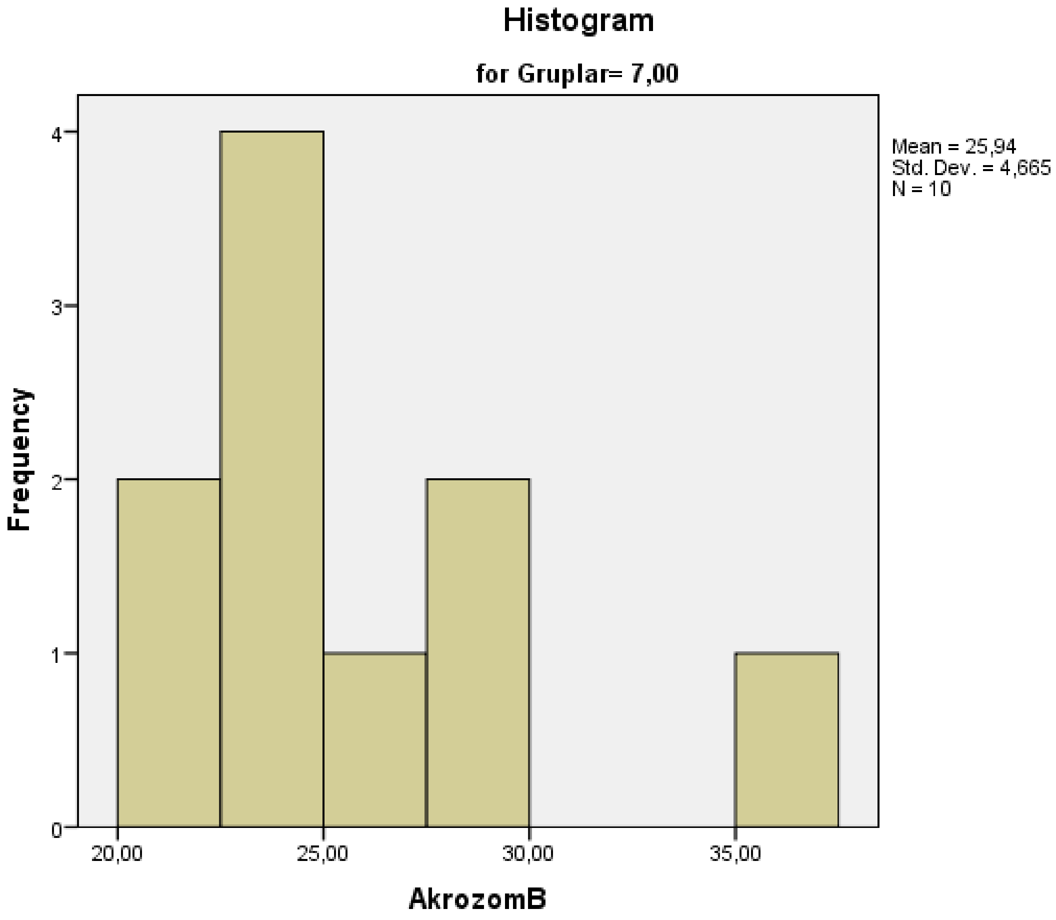

Histogram
for Gruplar= 7,00
Mean = 25,94
Std. Dev. = 4,665
N = 10
Frequency
0
1
2
3
4
20,00
25,00
30,00
35,00
AkrozomB

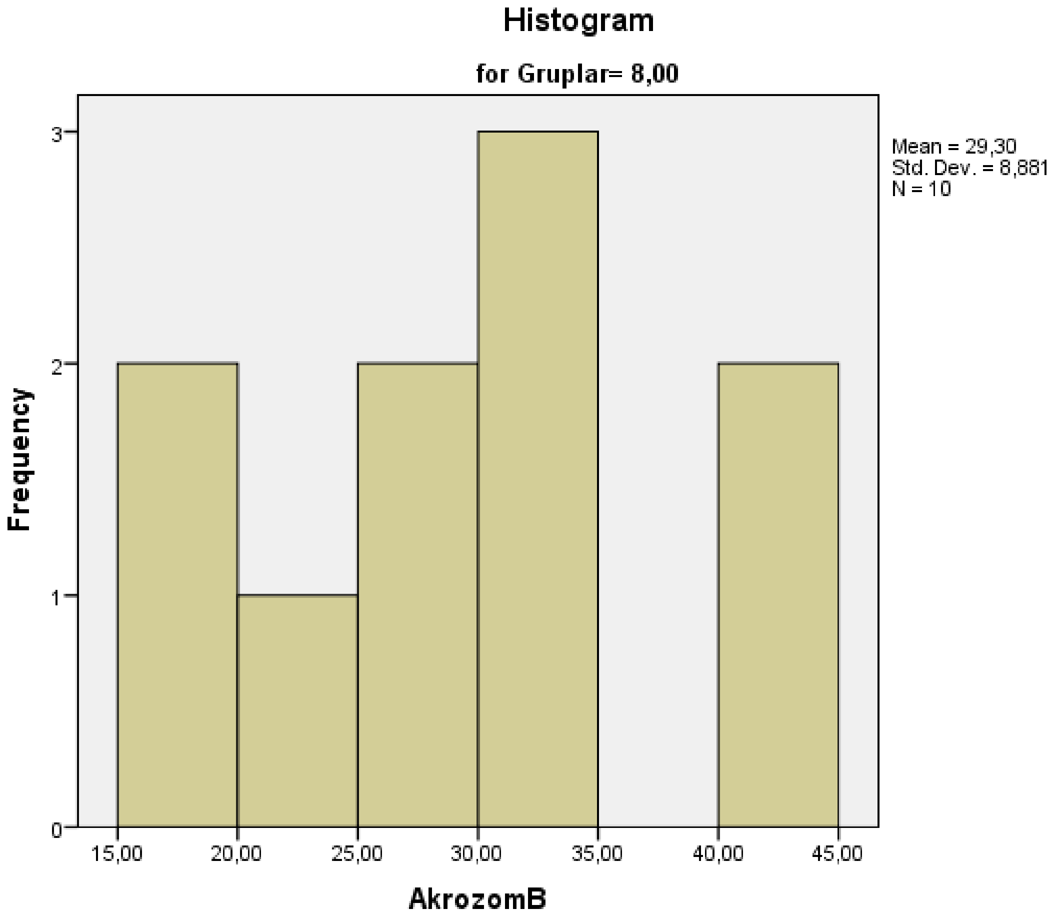
Histogram
for Gruplar= 8,00
Mean = 29,30
Std. Dev. = 8,881
N = 10
Frequency
3
2
1
0
15,00
20,00
25,00
30,00
35,00
40,00
45,00
AkrozomB

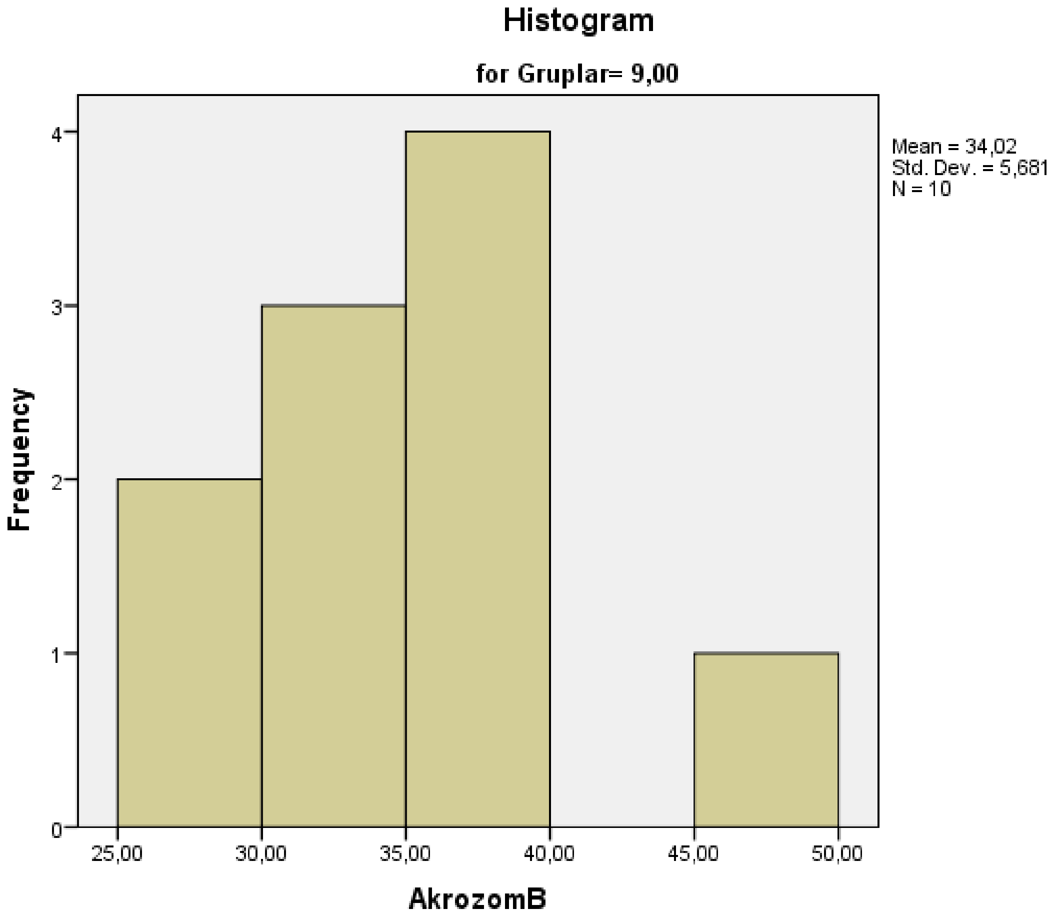

Histogram
for Gruplar= 9,00
Mean = 34,02
Std. Dev. = 5,681
N = 10
Frequency
0
1
2
3
4
25,00
30,00
35,00
40,00
45,00
50,00
AkrozomB

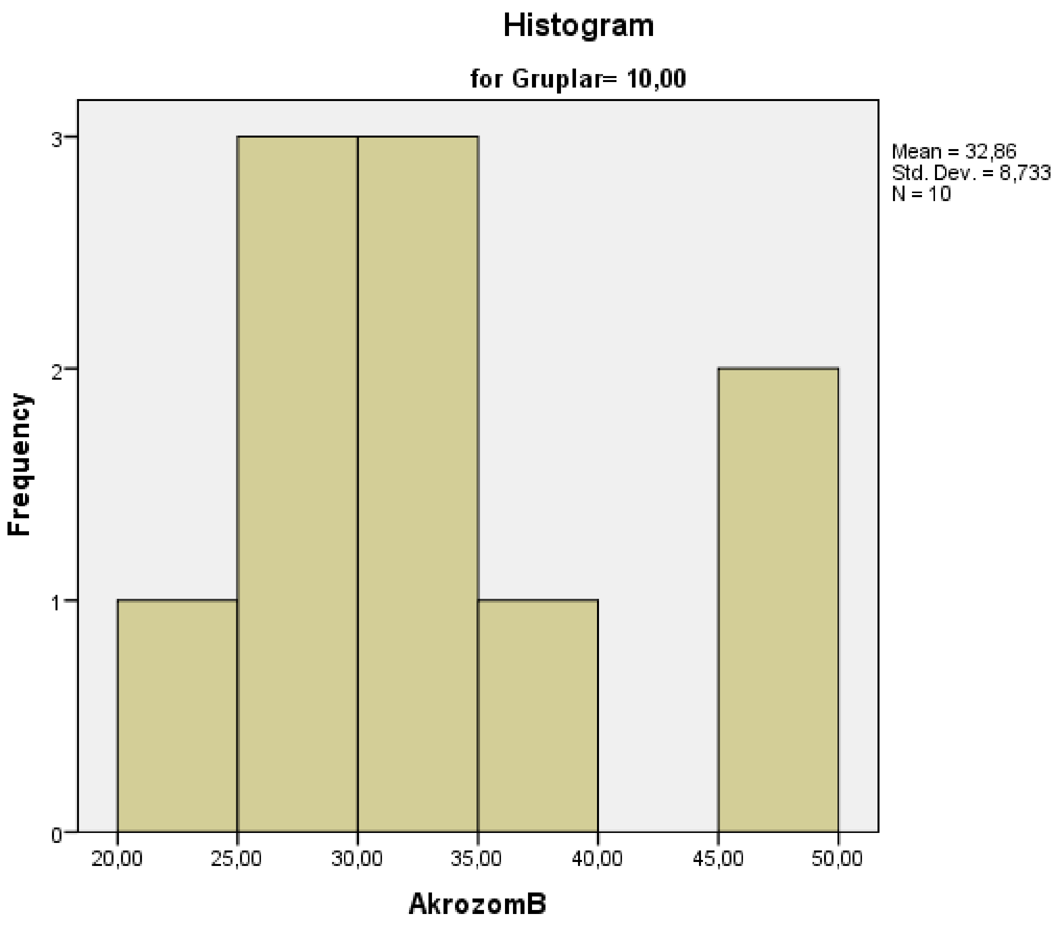

Stem-and-Leaf Plots

```
AkrozomB Stem-and-Leaf Plot for
Gruplar= 1,00

 Frequency    Stem &  Leaf

     4,00        2 .  5799
     1,00        3 .  0
     2,00        3 .  67

 Stem width:  10,00
 Each leaf:       1 case(s)
```

```
AkrozomB Stem-and-Leaf Plot for
```

```
Gruplar= 2,00

 Frequency    Stem &  Leaf

     1,00        2 .  1
     3,00        2 .  678
     1,00        3 .  2
     4,00        3 .  5566
     1,00        4 .  3

 Stem width:  10,00
 Each leaf:       1 case(s)




AkrozomB Stem-and-Leaf Plot for
Gruplar= 3,00

 Frequency    Stem &  Leaf

     3,00        2 .  679
     2,00        3 .  13
     3,00        3 .  558
     2,00        4 .  00

 Stem width:  10,00
 Each leaf:       1 case(s)




AkrozomB Stem-and-Leaf Plot for
Gruplar= 4,00

 Frequency    Stem &  Leaf

     3,00        2 .  568
     1,00        3 .  0
     5,00        3 .  55667
     1,00        4 .  2

 Stem width:  10,00
 Each leaf:       1 case(s)




AkrozomB Stem-and-Leaf Plot for
Gruplar= 5,00

 Frequency    Stem &  Leaf

     2,00        2 .  02
     4,00        2 .  5789
     1,00        3 .  3
```

```
     3,00        3 .  567

 Stem width:  10,00
 Each leaf:       1 case(s)
```

AkrozomB Stem-and-Leaf Plot for
Gruplar= 6,00

```
 Frequency    Stem &  Leaf

     3,00        2 .  444
     5,00        2 .  77789
      ,00        3 .
     1,00        3 .  5
     1,00 Extremes    (>=38)

 Stem width:  10,00
 Each leaf:       1 case(s)
```

AkrozomB Stem-and-Leaf Plot for
Gruplar= 7,00

```
 Frequency    Stem &  Leaf

     4,00        2 .  2223
     3,00        2 .  445
     1,00        2 .  7
     1,00        2 .  9
     1,00 Extremes    (>=37)

 Stem width:  10,00
 Each leaf:       1 case(s)
```

AkrozomB Stem-and-Leaf Plot for
Gruplar= 8,00

```
 Frequency    Stem &  Leaf

     2,00        1 .  68
     3,00        2 .  088
     3,00        3 .  124
     2,00        4 .  02

 Stem width:  10,00
 Each leaf:       1 case(s)
```

```
AkrozomB Stem-and-Leaf Plot for
Gruplar= 9,00

 Frequency    Stem &  Leaf

     2,00        2 .  67
     3,00        3 .  013
     4,00        3 .  5558
     1,00 Extremes    (>=46)

 Stem width:  10,00
 Each leaf:       1 case(s)
```

```
AkrozomB Stem-and-Leaf Plot for
Gruplar= 10,00

 Frequency    Stem &  Leaf

     1,00        2 .  0
     3,00        2 .  577
     3,00        3 .  024
     1,00        3 .  8
      ,00        4 .
     2,00        4 .  57

 Stem width:  10,00
 Each leaf:       1 case(s)
```

Normal Q-Q Plots

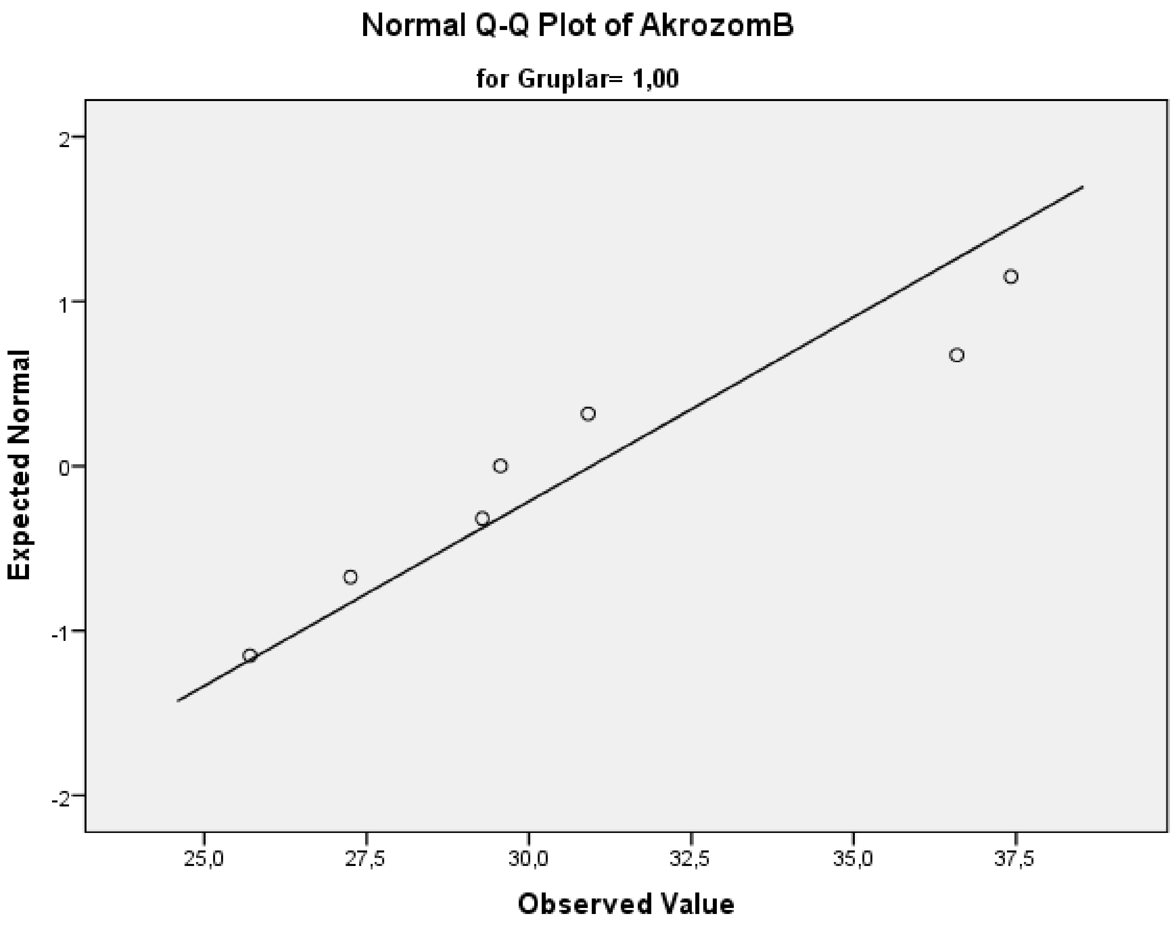

Normal Q-Q Plot of AkrozomB
for Gruplar= 1,00
Expected Normal
2
1
0
-1
-2
25,0
27,5
30,0
32,5
35,0
37,5
Observed Value

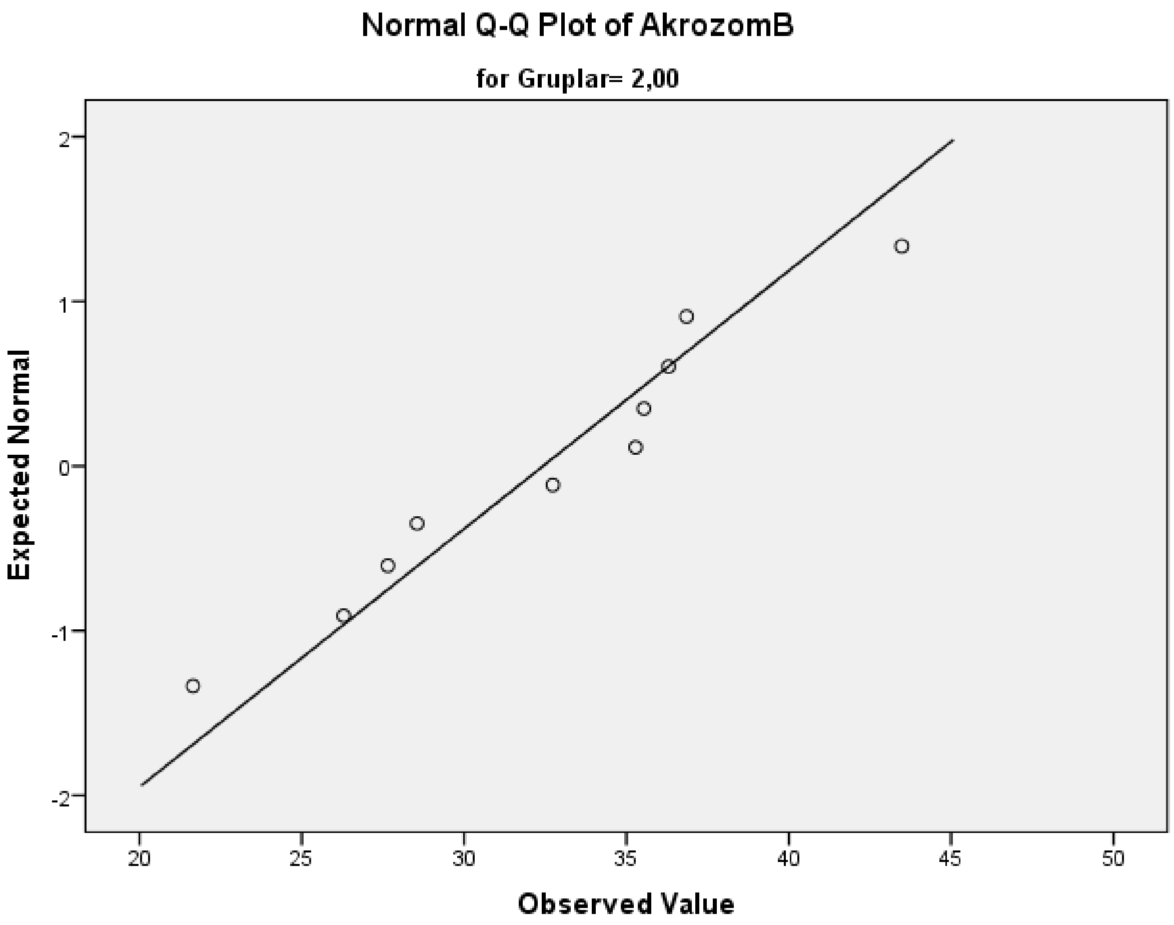
Normal Q-Q Plot of AkrozomB
for Gruplar= 2,00
Expected Normal
Observed Value
2
1
0
-1
-2
20
25
30
35
40
45
50

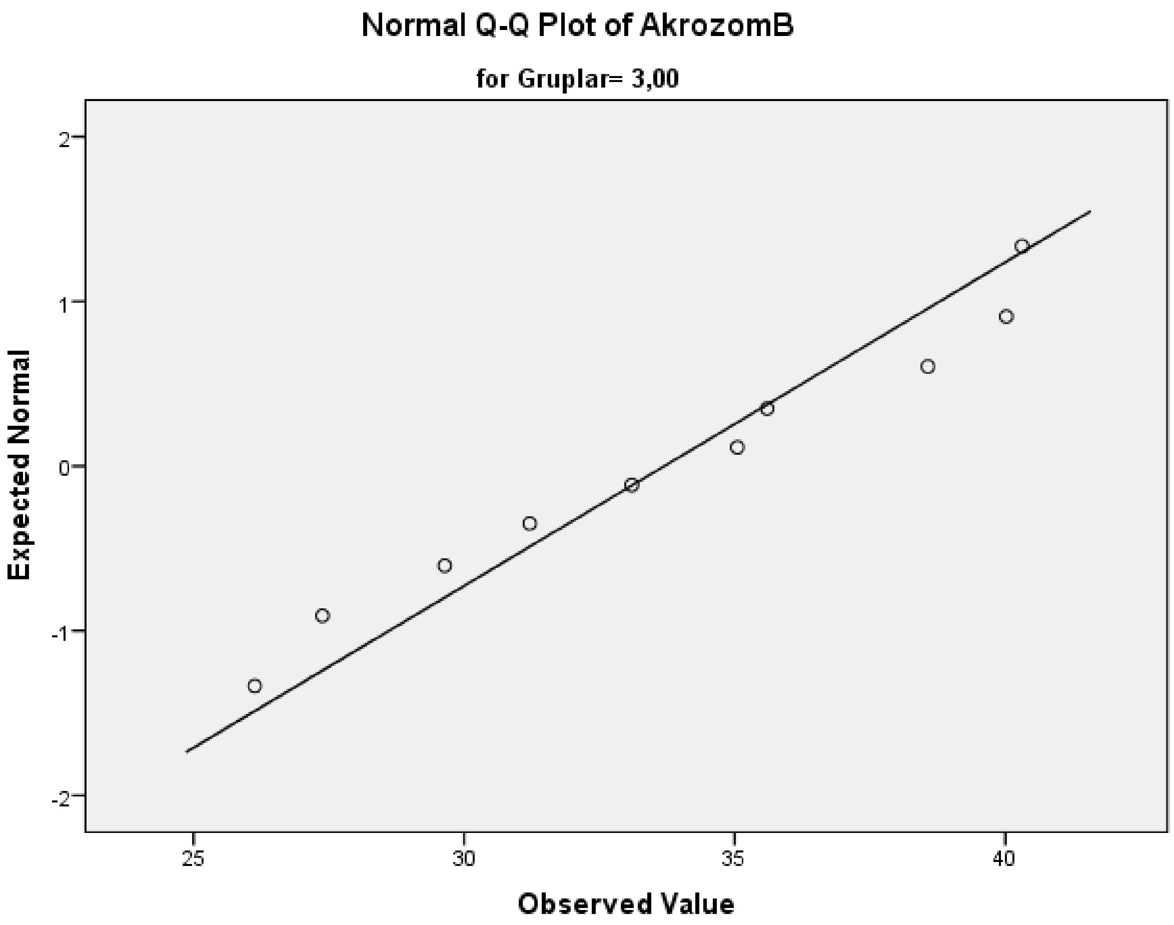

Normal Q-Q Plot of AkrozomB
for Gruplar= 3,00
Expected Normal
Observed Value
2
1
0
-1
-2
25
30
35
40

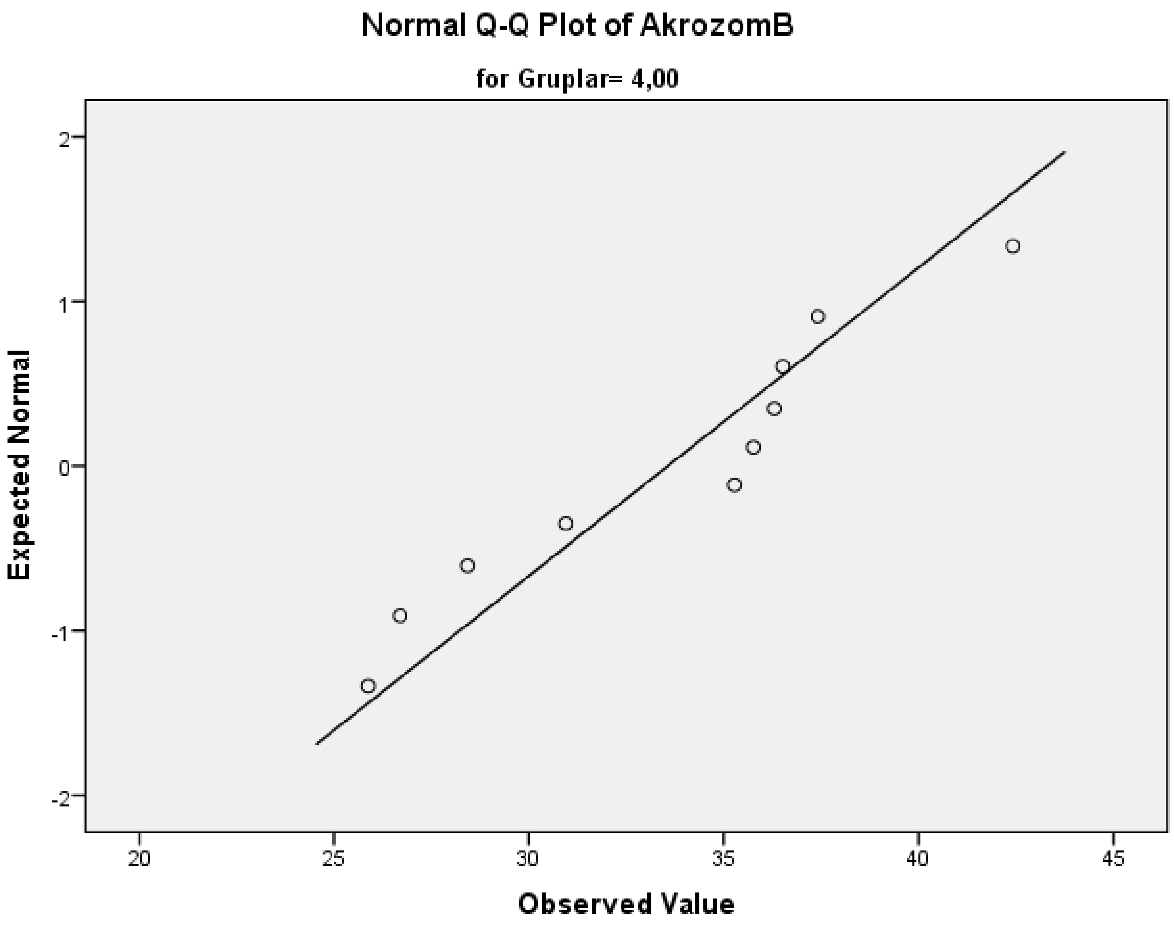
Normal Q-Q Plot of AkrozomB
for Gruplar= 4,00
Expected Normal
2
1
0
-1
-2
20
25
30
35
40
45
Observed Value

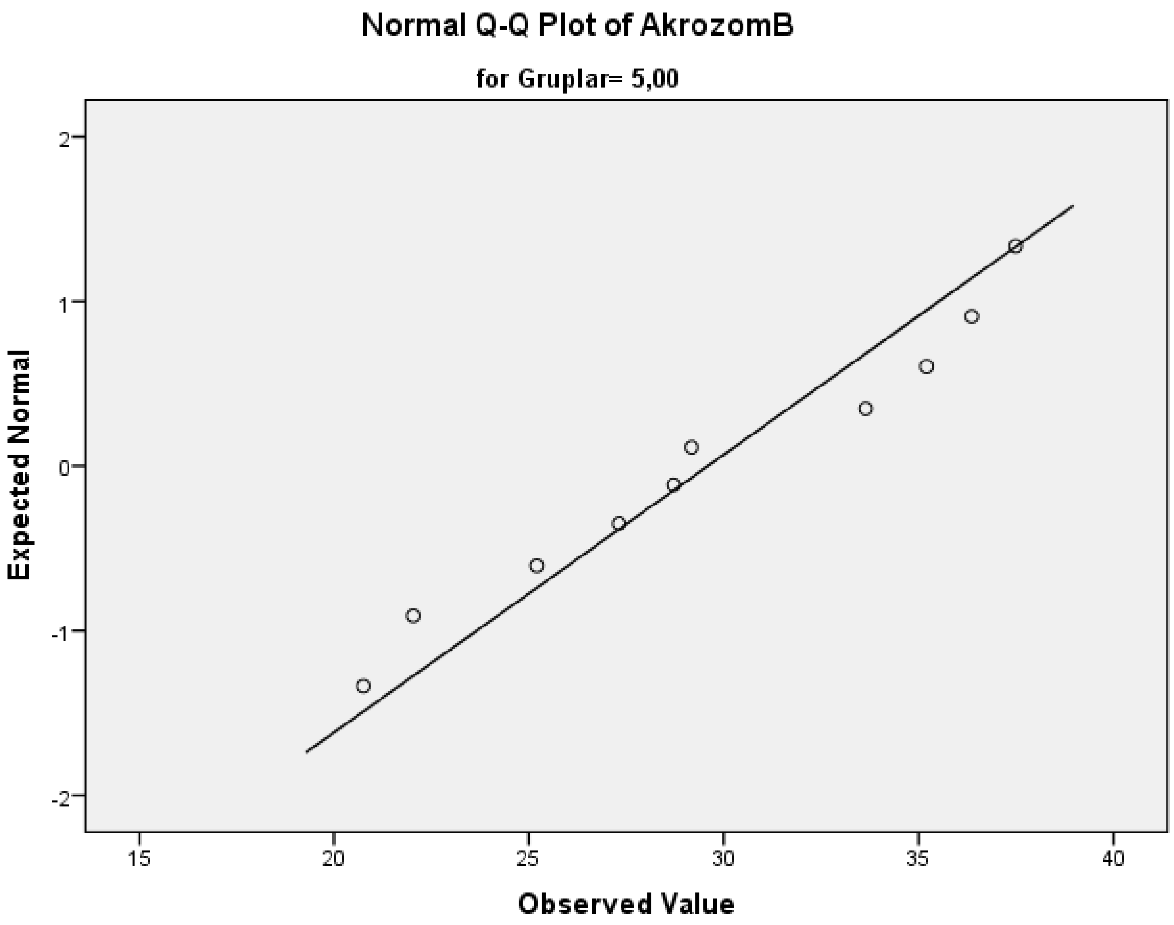

Normal Q-Q Plot of AkrozomB
for Gruplar= 5,00
Expected Normal
2
1
0
-1
-2
15
20
25
30
35
40
Observed Value

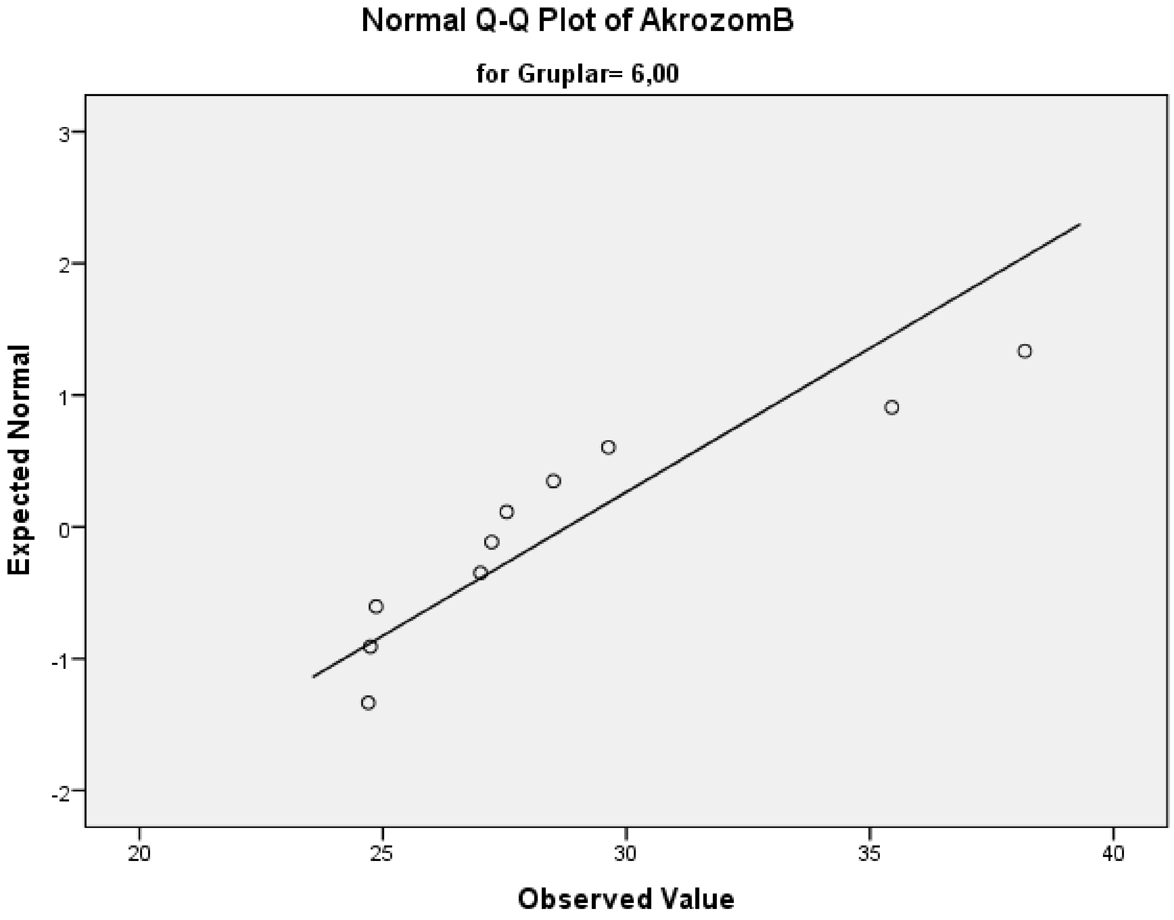

Normal Q-Q Plot of AkrozomB
for Gruplar= 6,00
Expected Normal
Observed Value
3
2
1
0
-1
-2
20
25
30
35
40

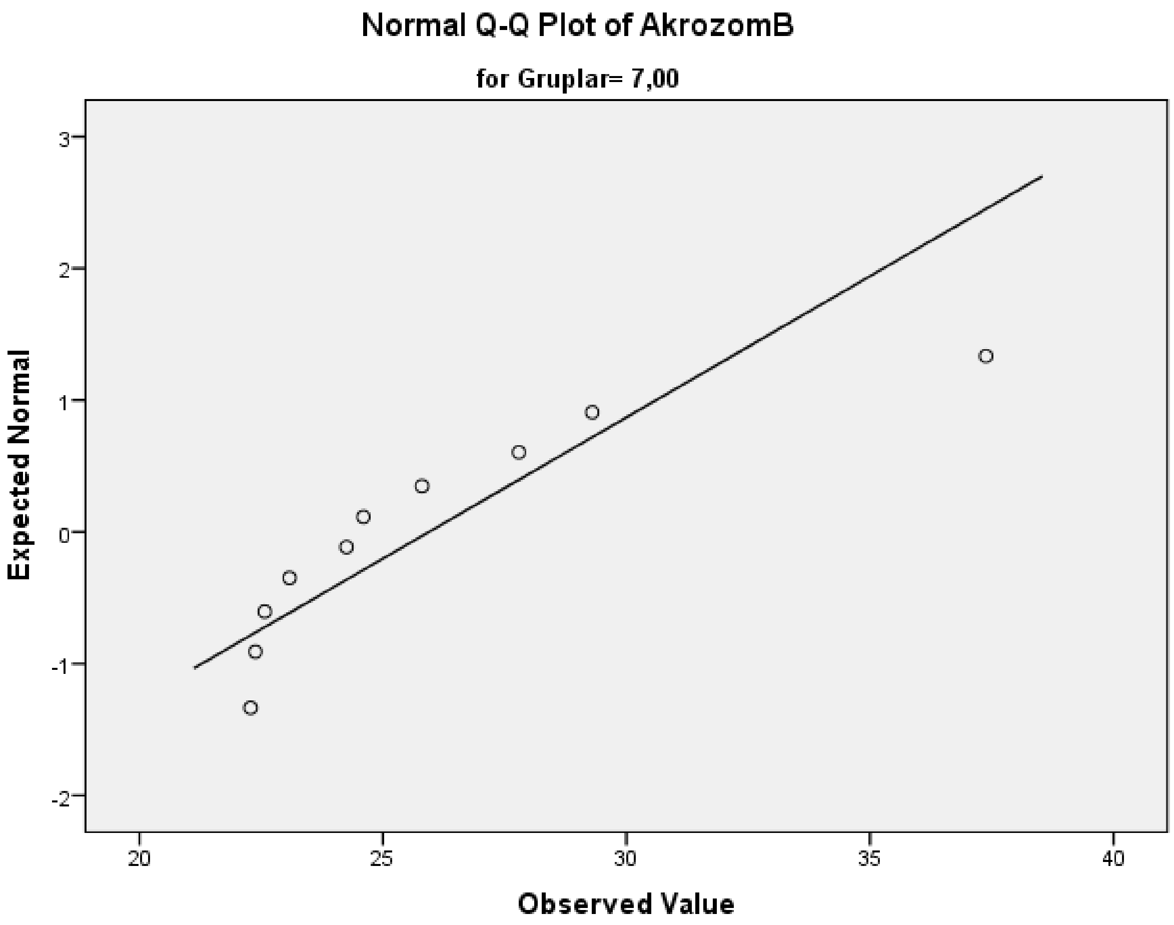
Normal Q-Q Plot of AkrozomB
for Gruplar= 7,00
Expected Normal
Observed Value
3
2
1
0
-1
-2
20
25
30
35
40

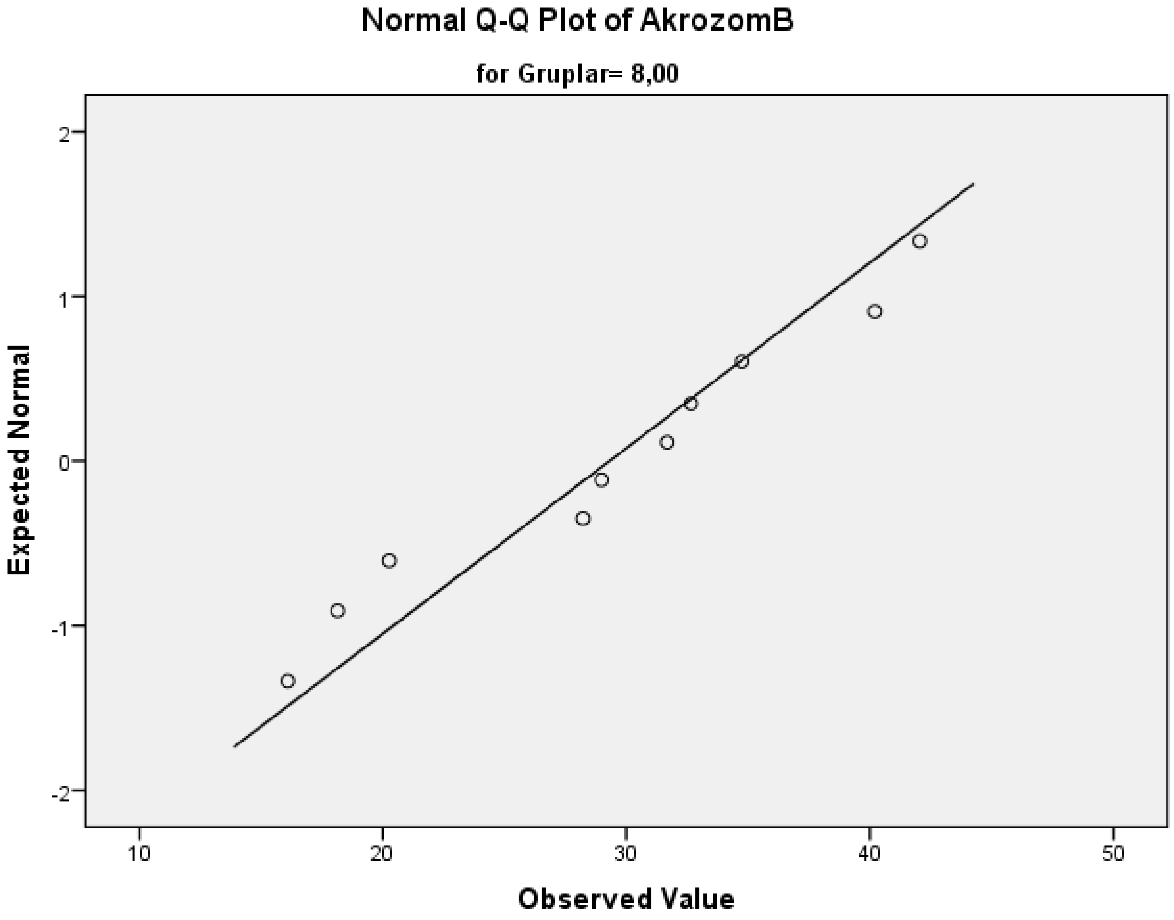
Normal Q-Q Plot of AkrozomB
for Gruplar= 8,00
Expected Normal
2
1
0
-1
-2
10
20
30
40
50
Observed Value

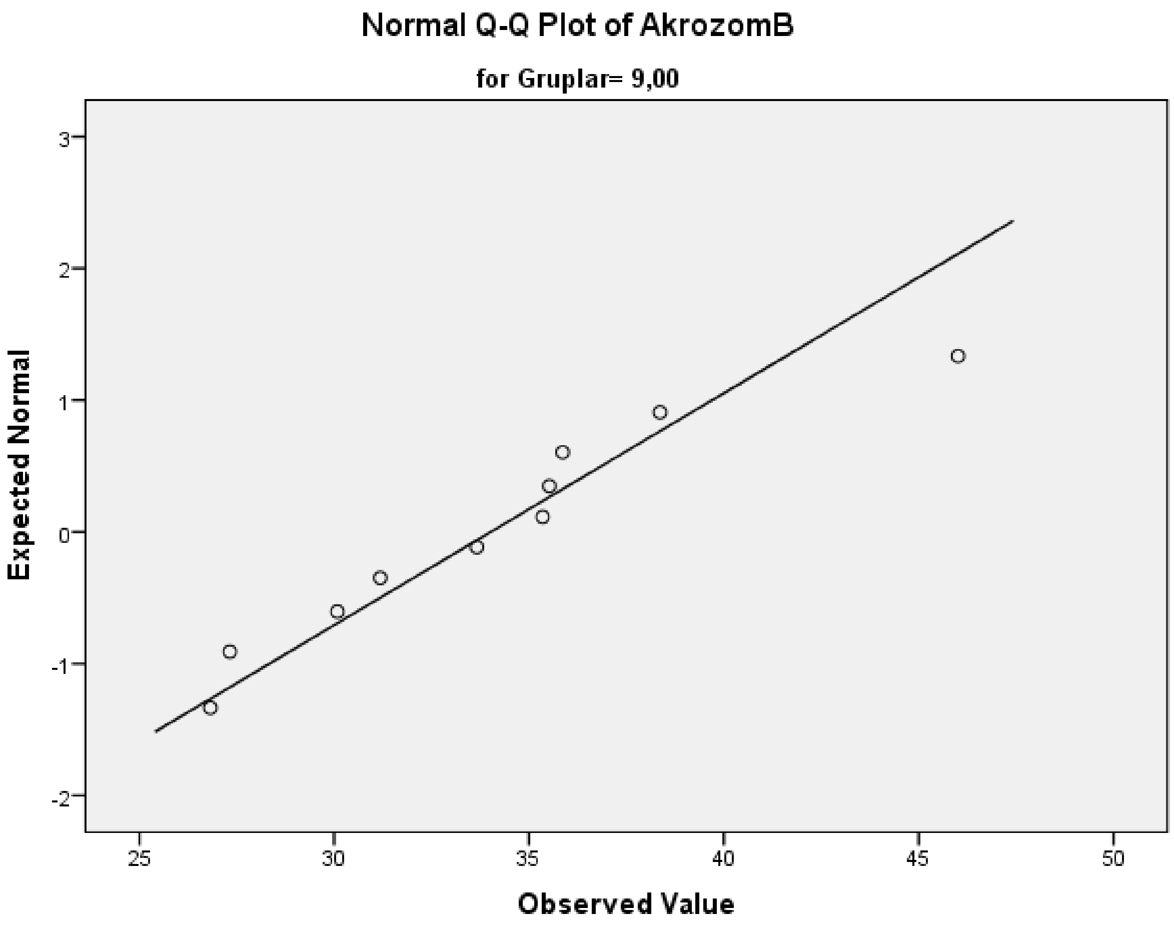
Normal Q-Q Plot of AkrozomB
for Gruplar= 9,00
Expected Normal
Observed Value
3
2
1
0
-1
-2
25
30
35
40
45
50

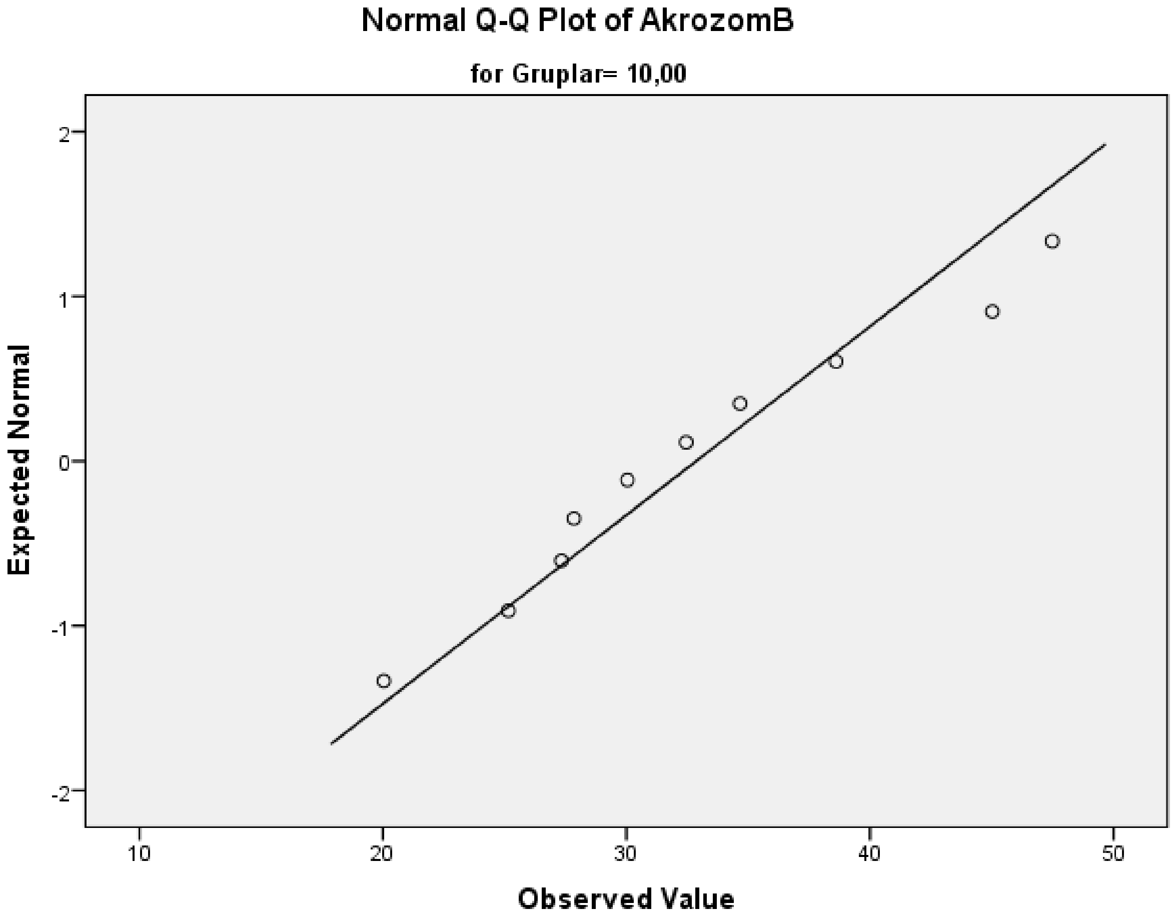

Detrended Normal Q-Q Plots

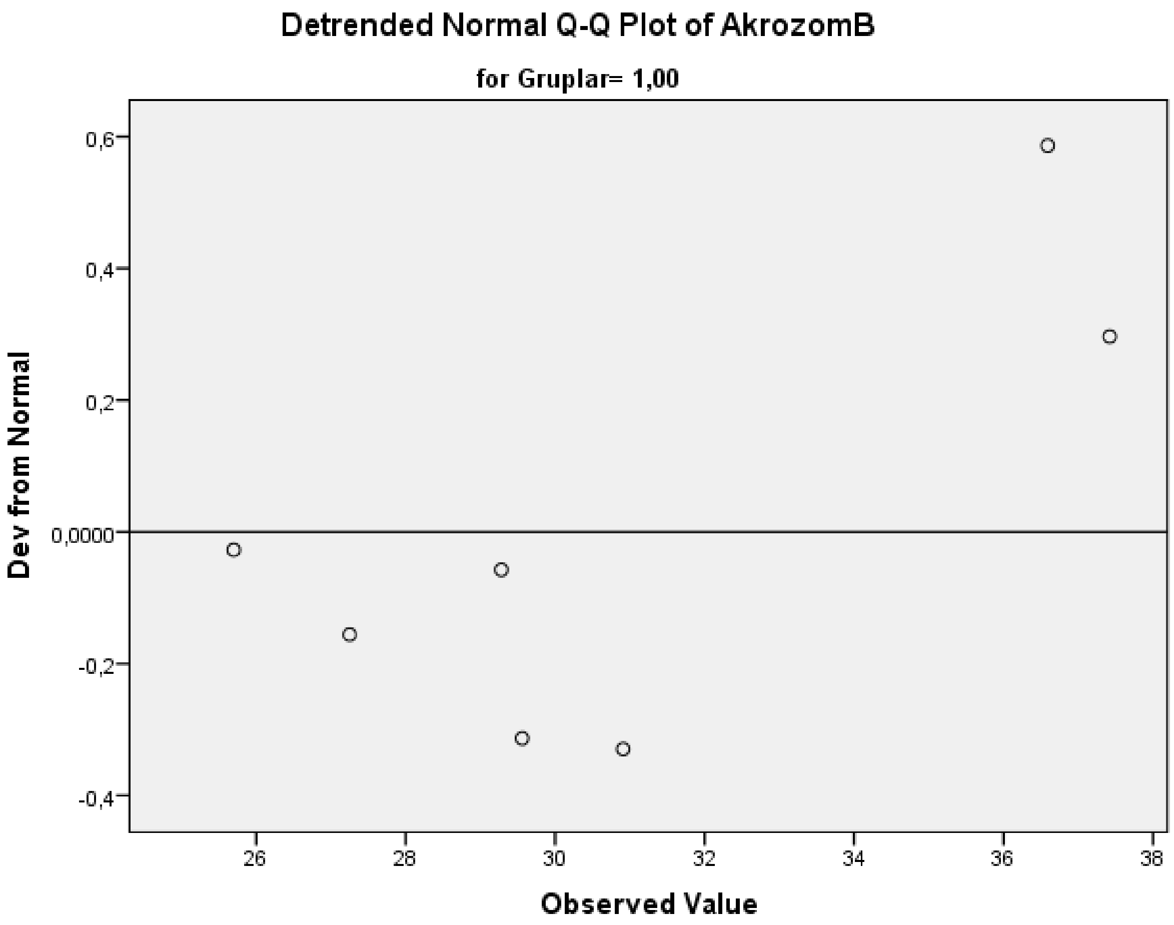

Detrended Normal Q-Q Plot of AkrozomB
for Gruplar= 1,00
Dev from Normal
0,6
0,4
0,2
0,0000
-0,2
-0,4
26
28
30
32
34
36
38
Observed Value

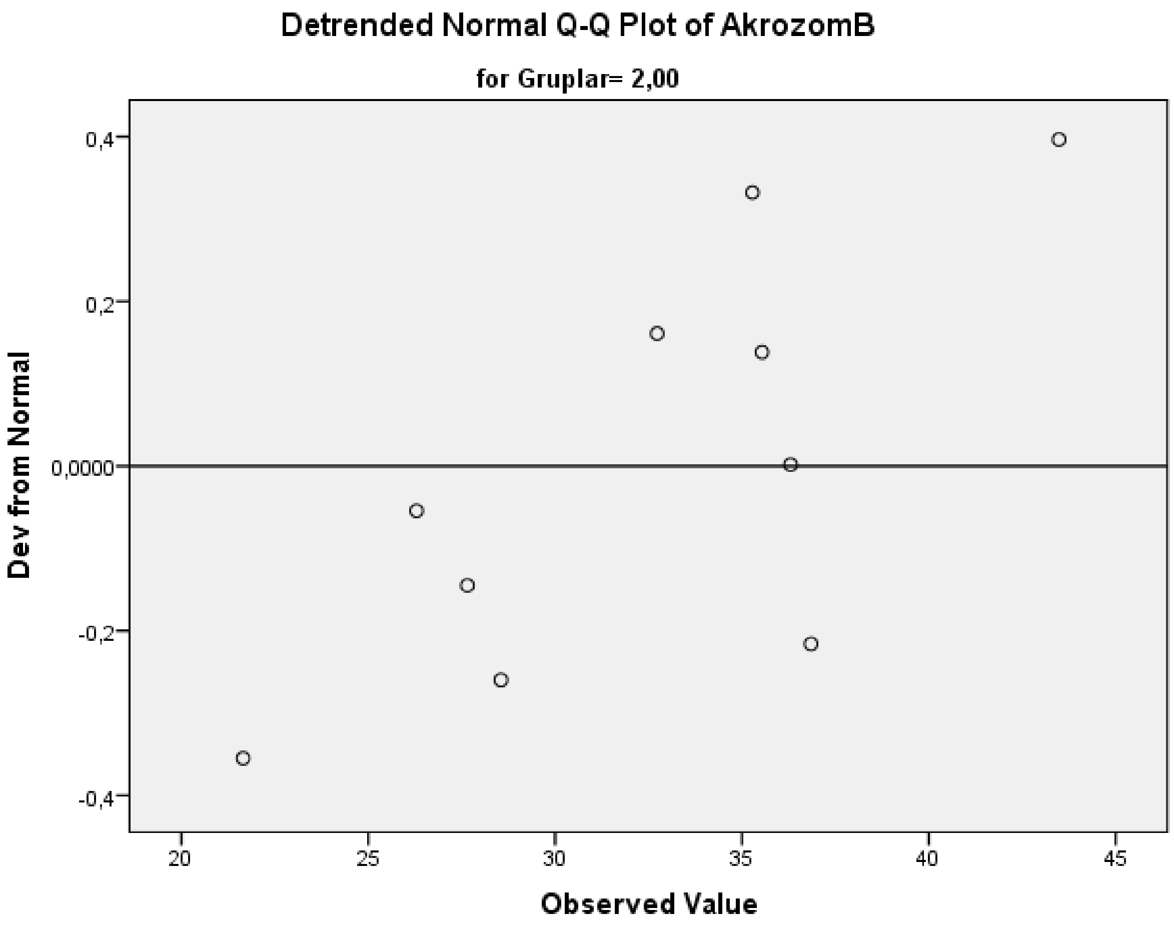
Detrended Normal Q-Q Plot of AkrozomB
for Gruplar= 2,00
Dev from Normal
0,4
0,2
0,0000
-0,2
-0,4
20
25
30
35
40
45
Observed Value

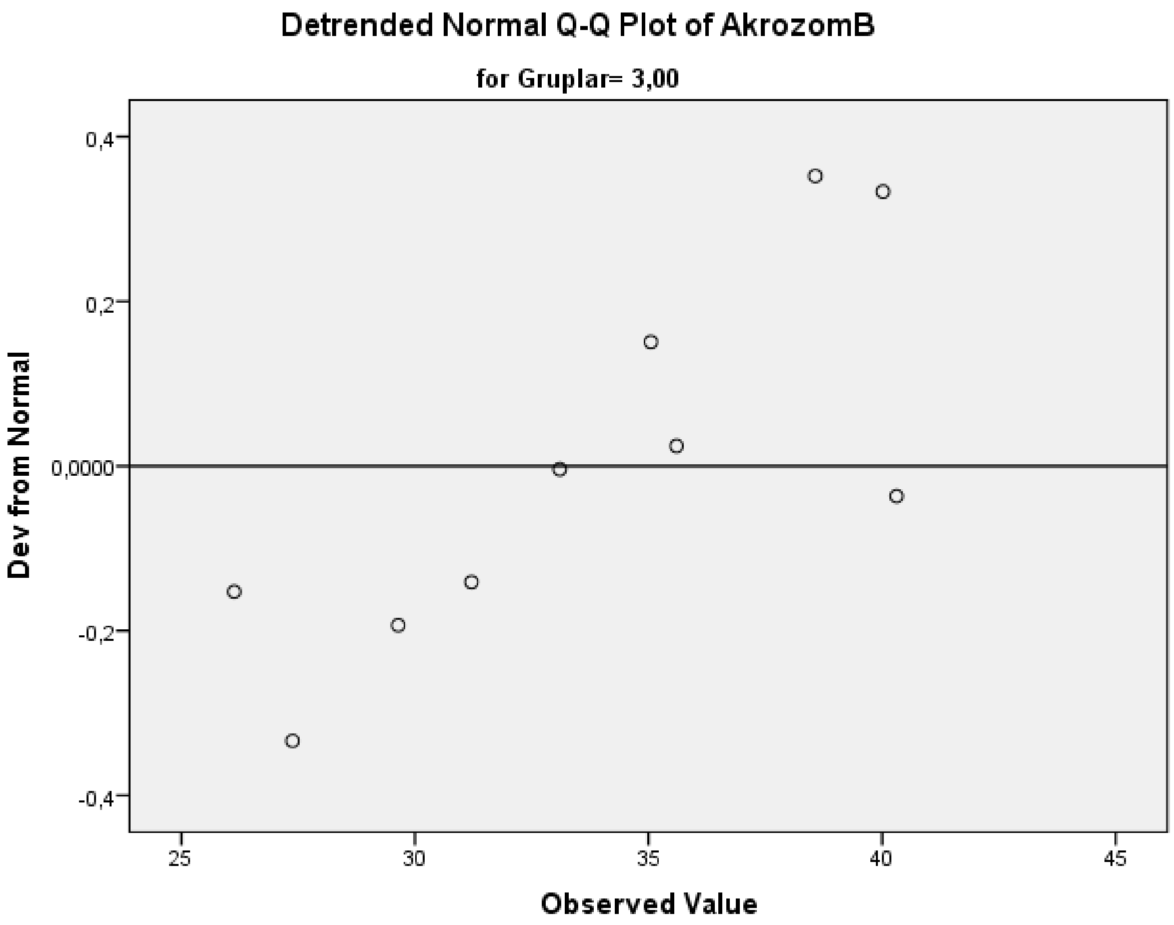

Detrended Normal Q-Q Plot of AkrozomB
for Gruplar= 3,00
Dev from Normal
0,4
0,2
0,0000
-0,2
-0,4
25
30
35
40
45
Observed Value

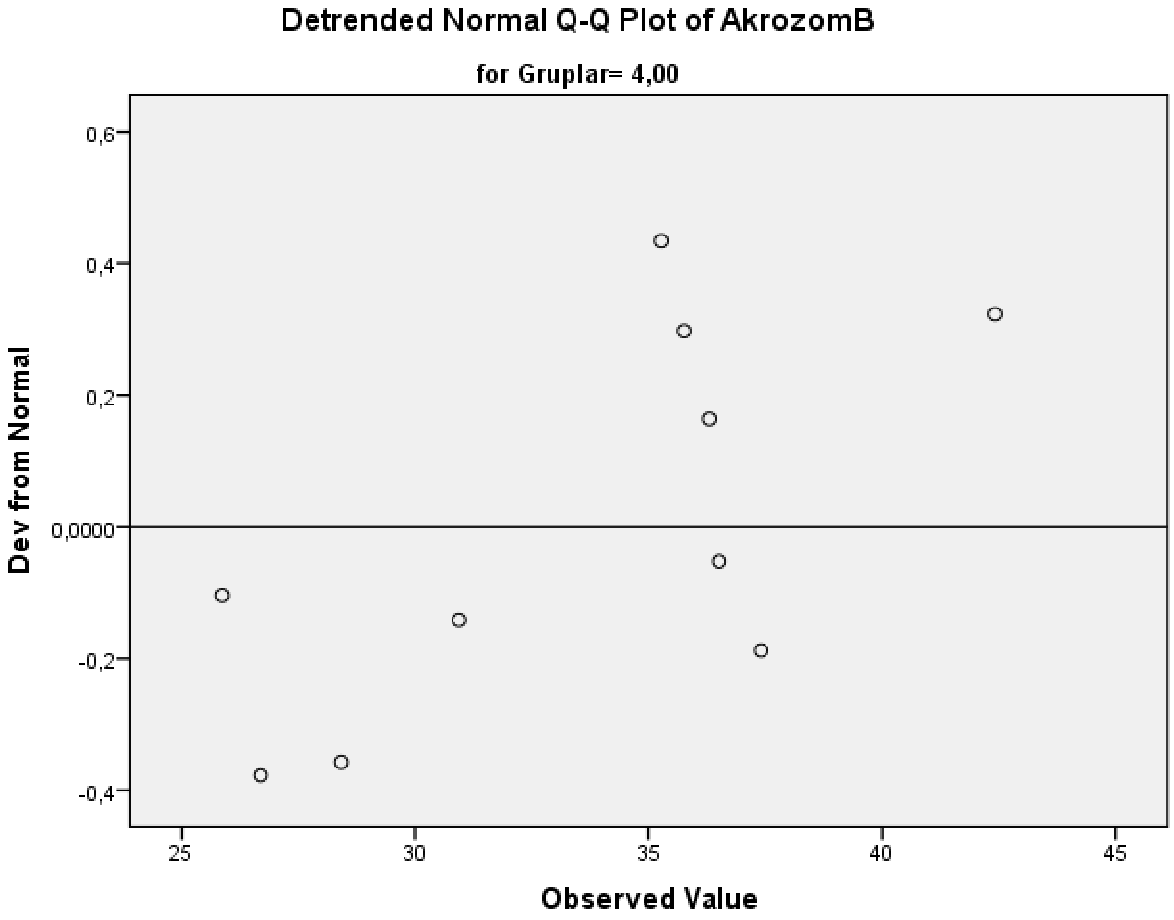

Detrended Normal Q-Q Plot of AkrozomB
for Gruplar= 4,00
Dev from Normal
0,6
0,4
0,2
0,0000
-0,2
-0,4
25
30
35
40
45
Observed Value

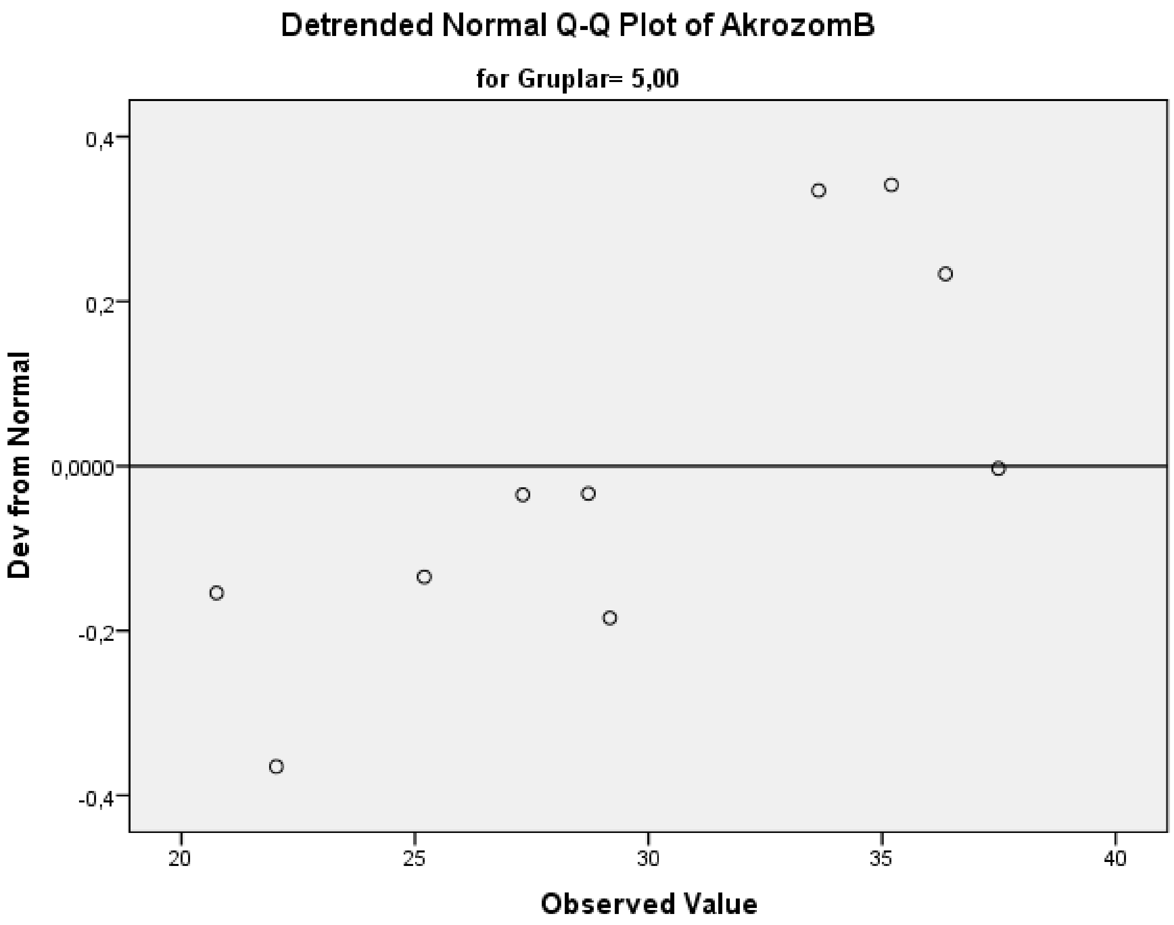
Detrended Normal Q-Q Plot of AkrozomB
for Gruplar= 5,00
Dev from Normal
0,4
0,2
0,0000
-0,2
-0,4
20
25
30
35
40
Observed Value

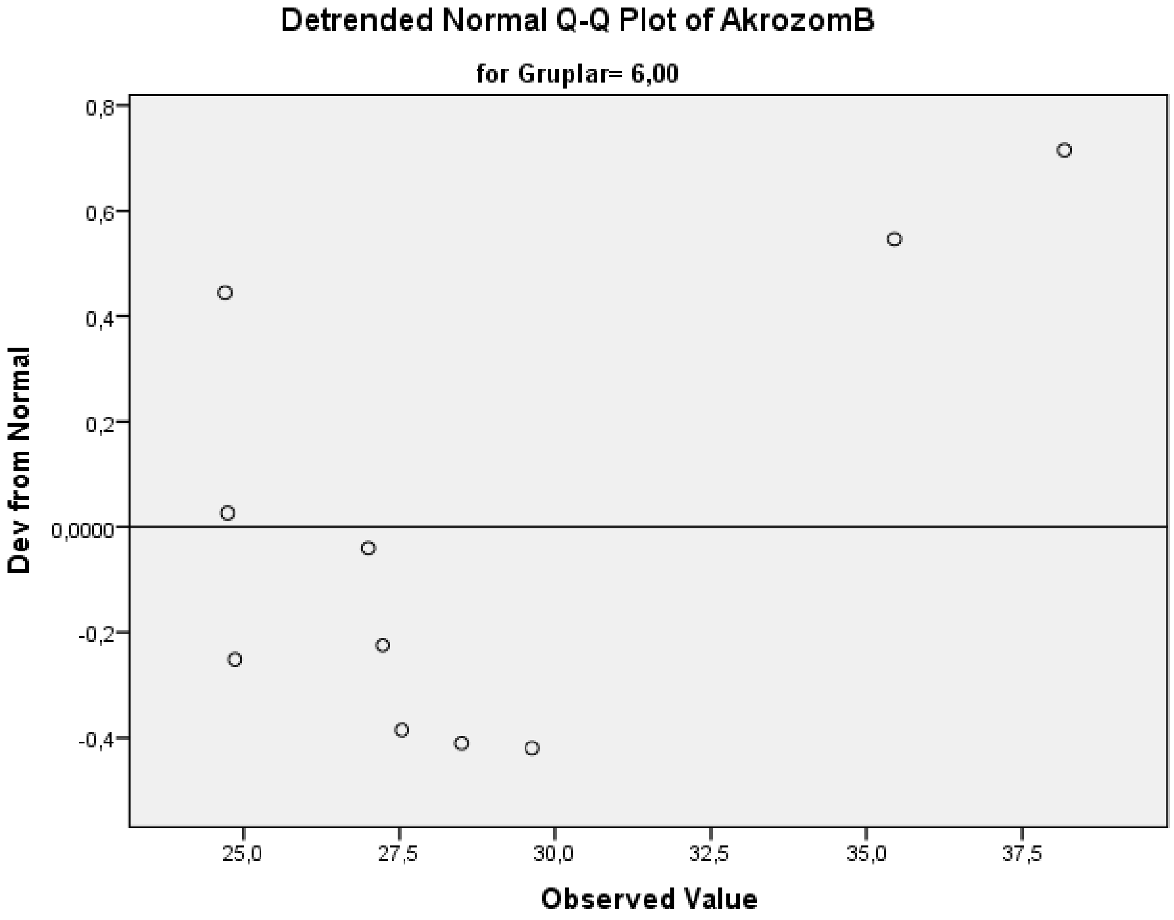
Detrended Normal Q-Q Plot of AkrozomB
for Gruplar= 6,00
Dev from Normal
0,8
0,6
0,4
0,2
0,0000
-0,2
-0,4
25,0
27,5
30,0
32,5
35,0
37,5
Observed Value

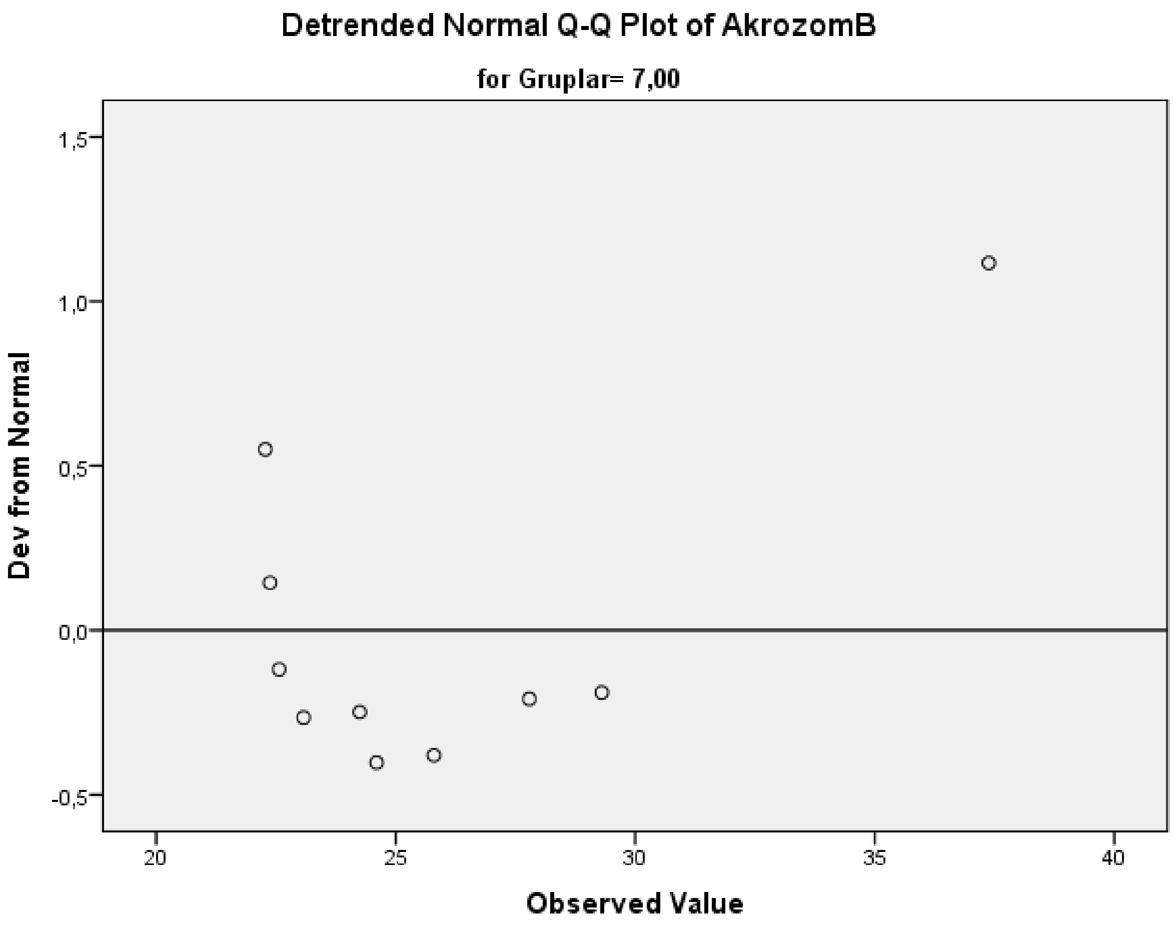

Detrended Normal Q-Q Plot of AkrozomB
for Gruplar= 7,00
Dev from Normal
1,5
1,0
0,5
0,0
-0,5
20
25
30
35
40
Observed Value

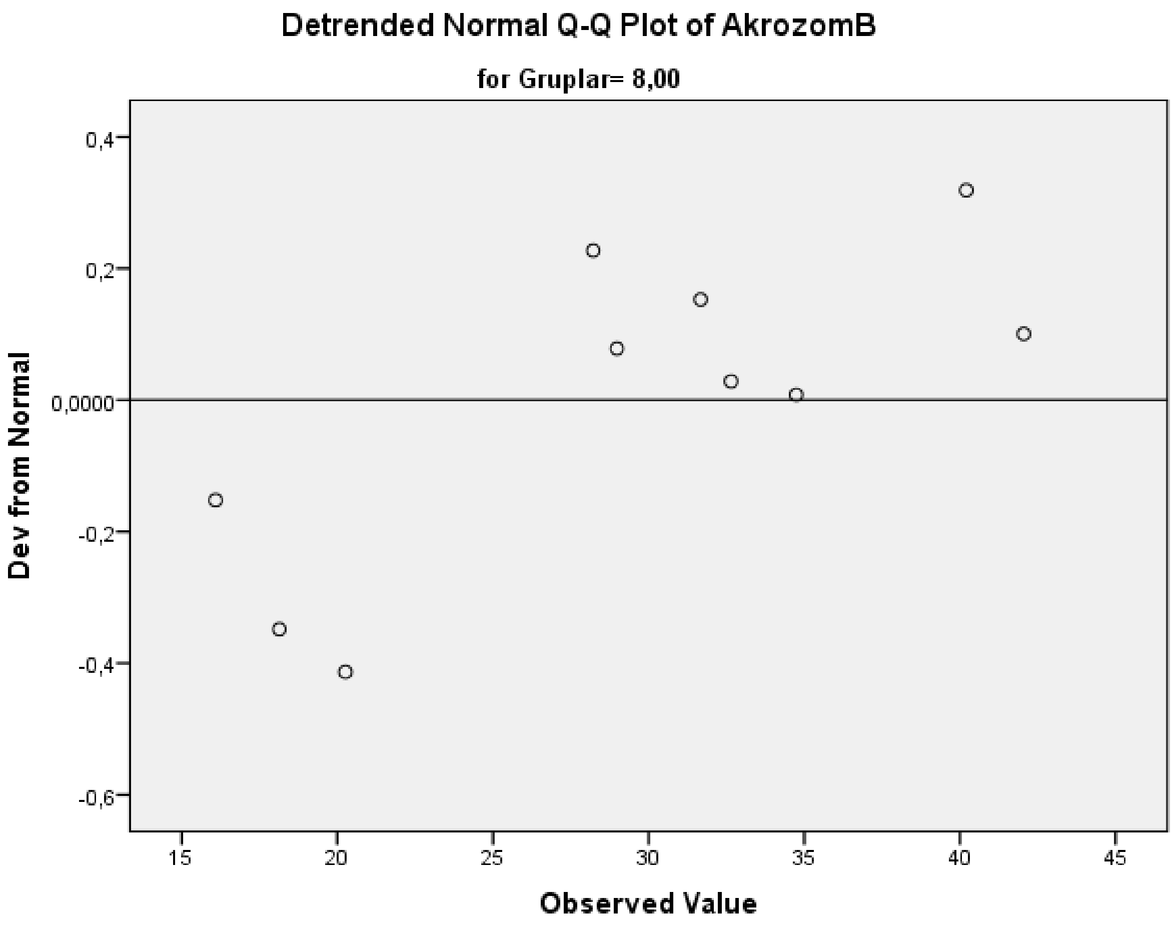

Detrended Normal Q-Q Plot of AkrozomB
for Gruplar= 8,00
Dev from Normal
0,4
0,2
0,0000
-0,2
-0,4
-0,6
15
20
25
30
35
40
45
Observed Value

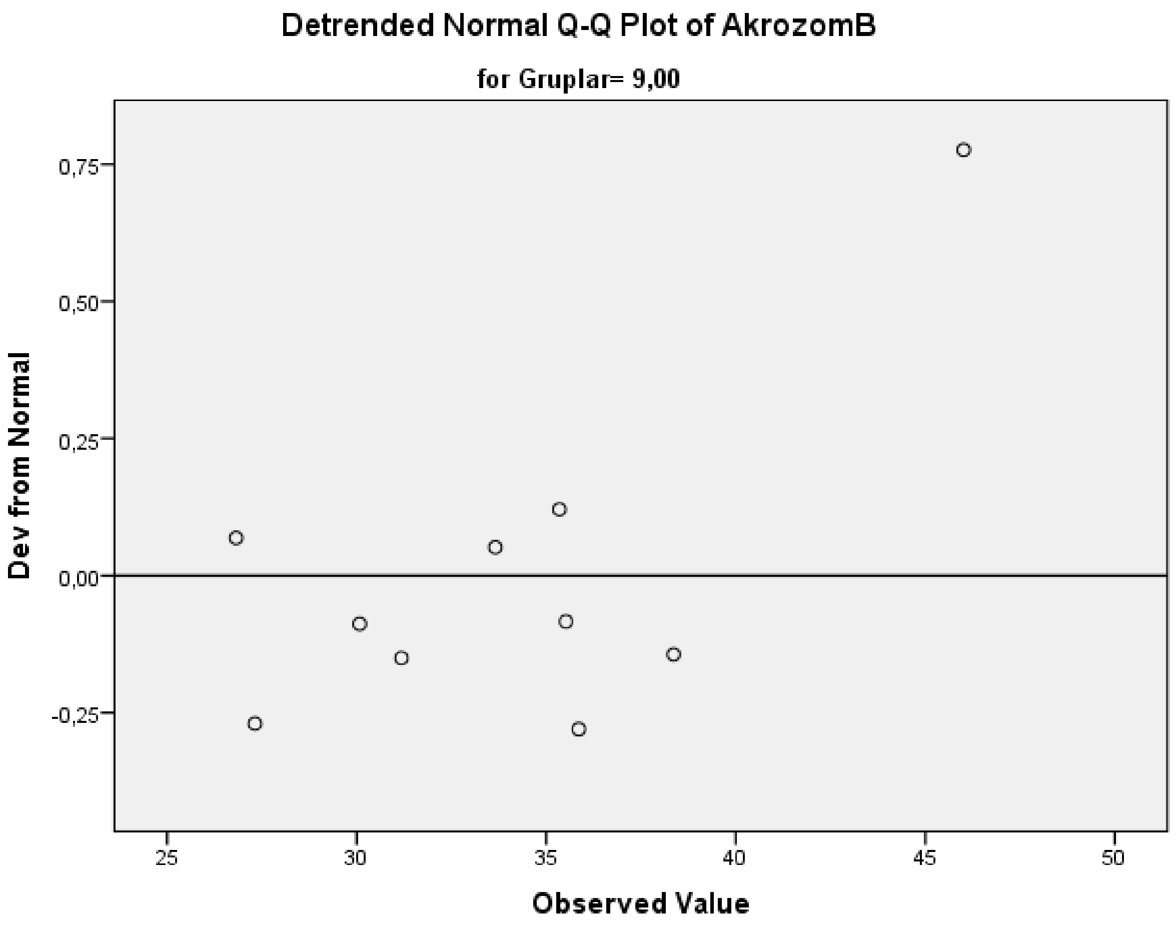
Detrended Normal Q-Q Plot of AkrozomB
for Gruplar= 9,00
Dev from Normal
0,75
0,50
0,25
0,00
-0,25
25
30
35
40
45
50
Observed Value

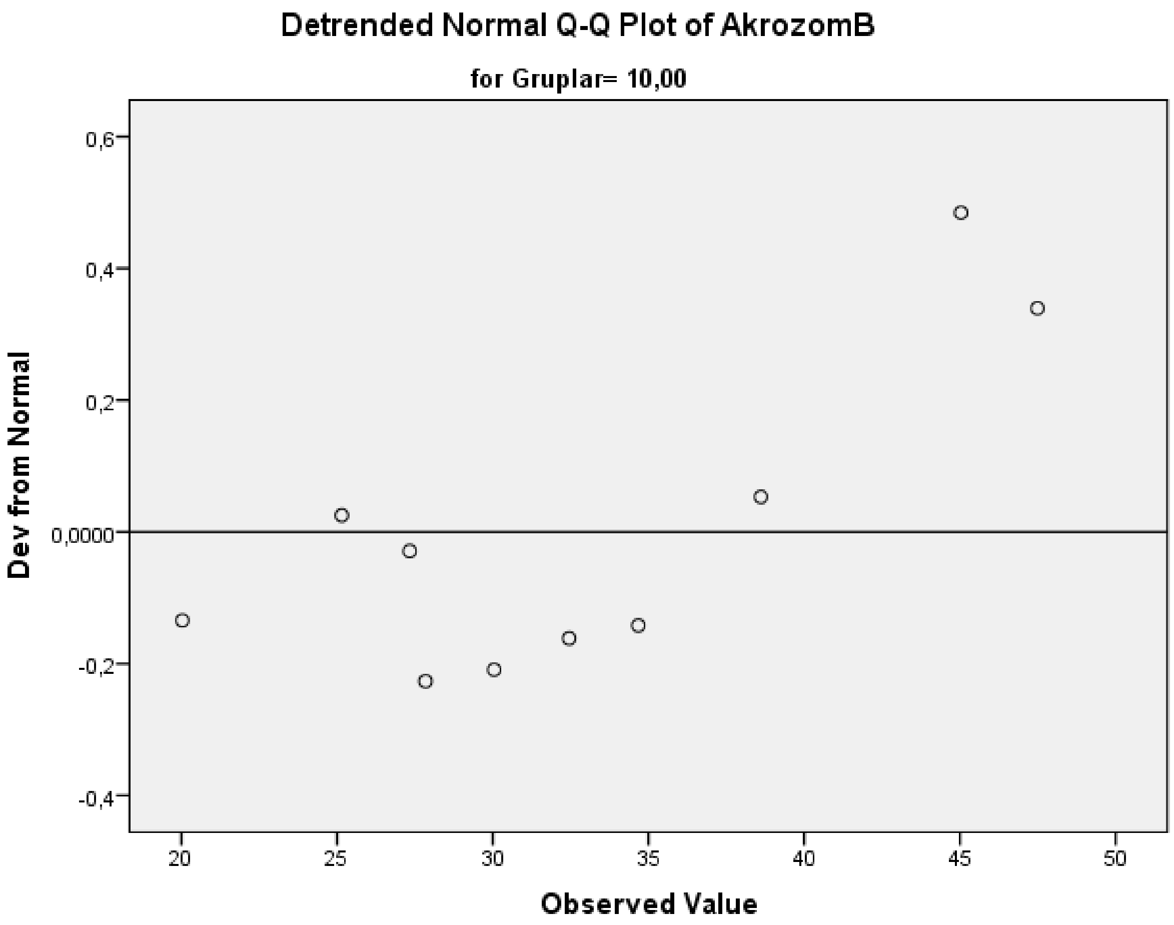
Detrended Normal Q-Q Plot of AkrozomB
for Gruplar= 10,00
Dev from Normal
0,6
0,4
0,2
0,0000
-0,2
-0,4
20
25
30
35
40
45
50
Observed Value

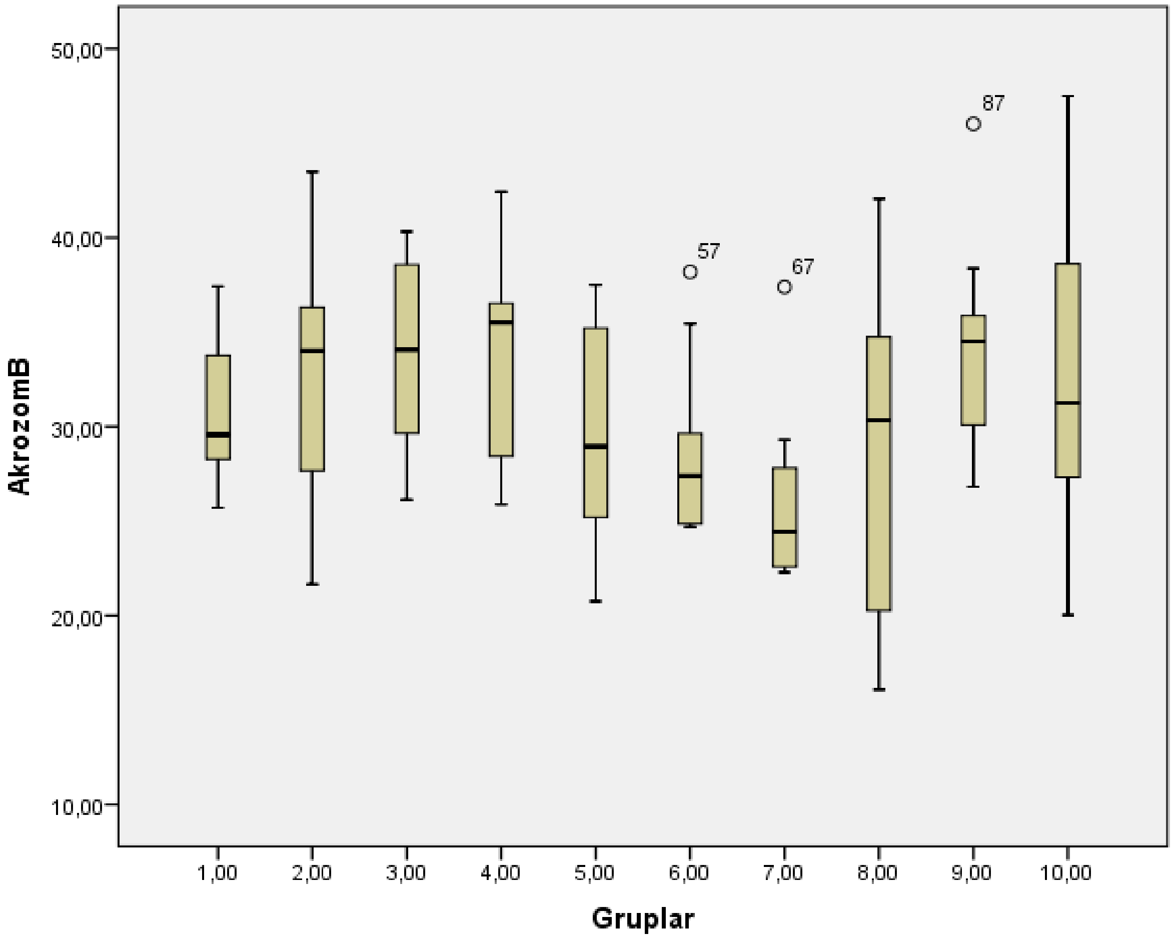


```
ONEWAY AkrozomB BY Gruplar
  /STATISTICS DESCRIPTIVES HOMOGENEITY
  /PLOT MEANS
  /MISSING ANALYSIS
  /POSTHOC=DUNCAN ALPHA(0.05).
```

Oneway

Notes

Output Created		24-DEC-2020 11:32:52
Comments		
Input	Active Dataset	DataSet0
	Filter	<none>
	Weight	<none>

	Split File	<none>
	N of Rows in Working Data File	103
Missing Value Handling	Definition of Missing	User-defined missing values are treated as missing.
	Cases Used	Statistics for each analysis are based on cases with no missing data for any variable in the analysis.
Syntax		ONEWAY AkrozomB BY Gruplar<br>/STATISTICS DESCRIPTIVES HOMOGENEITY<br>/PLOT MEANS<br>/MISSING ANALYSIS<br>/POSTHOC=DUNCAN ALPHA(0.05).
Resources	Processor Time	00:00:00,23
	Elapsed Time	00:00:00,38

30,9586
32,4320
33,7010
33,5590
29,5860
28,7830
25,9430
29,3000
34,0160
32,8640

Test of Homogeneity of Variances

AkrozomB

Levene Statistic	df1	df2	Sig.
1,409	9	87	,197

ANOVA

AkrozomB

	Sum of Squares	df	Mean Square	F	Sig.
Between Groups	637,067	9	70,785	1,830	,074

Within Groups	3364,471	87	38,672		
Total	4001,538	96			

Post Hoc Tests

Homogeneous Subsets

AkrozomB

Duncan[a,b]

Gruplar	N	Subset for alpha = 0.05	
		1	2
7,00	10	25,9430	
6,00	10	28,7830	28,7830
8,00	10	29,3000	29,3000
5,00	10	29,5860	29,5860
1,00	7	30,9586	30,9586
2,00	10		32,4320
10,00	10		32,8640
4,00	10		33,5590
3,00	10		33,7010
9,00	10		34,0160
Sig.		,120	,124

Means for groups in homogeneous subsets are displayed.

a. Uses Harmonic Mean Sample Size = 9,589.

b. The group sizes are unequal. The harmonic mean of the group sizes is used. Type I error levels are not guaranteed.

Means Plots

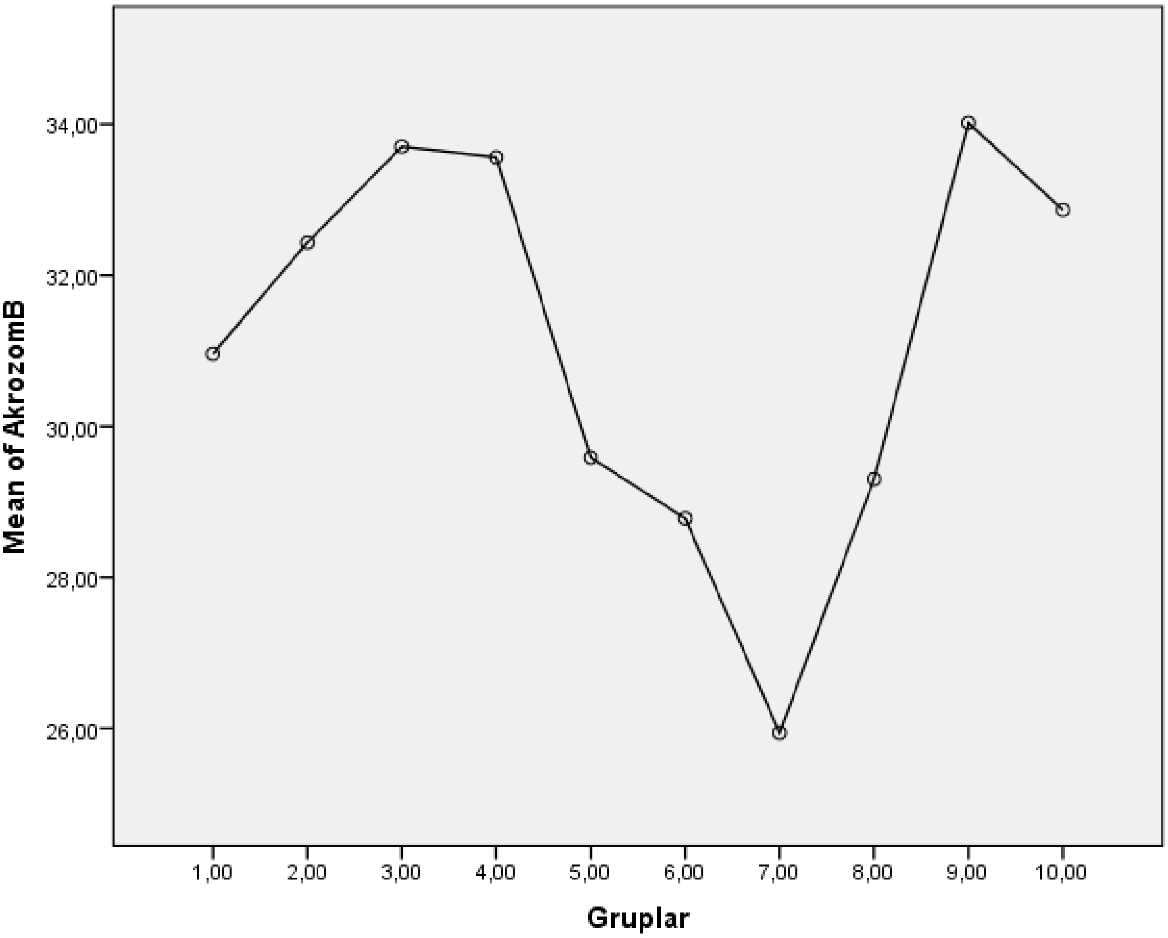

Mean of AkrozomB
34,00
32,00
30,00
28,00
26,00
1,00
2,00
3,00
4,00
5,00
6,00
7,00
8,00
9,00
10,00
Gruplar

High mitochondrial activity- ANOVA

```
Mitokondriyal: High mitochondrial activity
```

Groups

```
1: Control
2: RES10
3: RES20
4: RES40
5: CD10
6: CD20
7: CD40
8: RLC10
9: RLC20
10: RLC40

EXAMINE VARIABLES=mitokondriyal BY gruplar
  /PLOT BOXPLOT STEMLEAF HISTOGRAM NPPLOT
  /COMPARE GROUPS
  /STATISTICS DESCRIPTIVES
  /CINTERVAL 95
  /MISSING LISTWISE
  /NOTOTAL.
```

Explore

Notes

Output Created		23-FEB-2021 19:55:15
Comments		
Input	Active Dataset	DataSet0
	Filter	<none>
	Weight	<none>
	Split File	<none>
	N of Rows in Working Data File	110
Missing Value Handling	Definition of Missing	User-defined missing values for dependent variables are treated as missing.
	Cases Used	Statistics are based on cases with no missing values for any dependent variable or factor used.

Syntax		EXAMINE VARIABLES=mitokondriyal BY gruplar<br>/PLOT BOXPLOT STEMLEAF HISTOGRAM NPPLOT<br>/COMPARE GROUPS<br>/STATISTICS DESCRIPTIVES<br>/CINTERVAL 95<br>/MISSING LISTWISE<br>/NOTOTAL.
Resources	Processor Time	00:00:03,64
	Elapsed Time	00:00:03,70

gruplar

Case Processing Summary

		Cases					
		Valid		Missing		Total	
	gruplar	N	Percent	N	Percent	N	Percent
mitokondriyal	1,00	10	100,0%	0	0,0%	10	100,0%
	2,00	10	100,0%	0	0,0%	10	100,0%
	3,00	10	100,0%	0	0,0%	10	100,0%
	4,00	10	100,0%	0	0,0%	10	100,0%
	5,00	10	100,0%	0	0,0%	10	100,0%
	6,00	10	100,0%	0	0,0%	10	100,0%
	7,00	10	100,0%	0	0,0%	10	100,0%
	8,00	10	100,0%	0	0,0%	10	100,0%
	9,00	10	100,0%	0	0,0%	10	100,0%
	10,00	10	100,0%	0	0,0%	10	100,0%

Descriptives

	gruplar			Statistic	Std. Error
mitokondriyal	1,00	Mean		55,9200	2,73165
		95% Confidence Interval for Mean	Lower Bound	49,7406	
			Upper Bound	62,0994	

	5% Trimmed Mean		56,0678	
	Median		55,9450	
	Variance		74,619	
	Std. Deviation		8,63824	
	Minimum		40,13	
	Maximum		69,05	
	Range		28,92	
	Interquartile Range		12,84	
	Skewness		-,306	,687
	Kurtosis		-,332	1,334
2,00	Mean		55,2240	2,63336
	95% Confidence Interval for Mean	Lower Bound	49,2669	
		Upper Bound	61,1811	
	5% Trimmed Mean		55,1767	
	Median		54,6150	
	Variance		69,346	
	Std. Deviation		8,32743	
	Minimum		45,57	
	Maximum		65,73	
	Range		20,16	
	Interquartile Range		16,67	
	Skewness		,089	,687
	Kurtosis		-2,150	1,334
3,00	Mean		53,1010	2,89102
	95% Confidence Interval for Mean	Lower Bound	46,5611	
		Upper Bound	59,6409	
	5% Trimmed Mean		53,1350	
	Median		55,4900	
	Variance		83,580	
	Std. Deviation		9,14221	
	Minimum		41,73	
	Maximum		63,86	
	Range		22,13	
	Interquartile Range		19,25	
	Skewness		-,289	,687
	Kurtosis		-1,943	1,334
4,00	Mean		52,8980	2,23037
	95% Confidence Interval for	Lower Bound	47,8525	

	Mean	Upper Bound	57,9435	
	5% Trimmed Mean		53,0256	
	Median		53,6350	
	Variance		49,746	
	Std. Deviation		7,05306	
	Minimum		40,49	
	Maximum		63,01	
	Range		22,52	
	Interquartile Range		11,12	
	Skewness		-,472	,687
	Kurtosis		-,454	1,334
5,00	Mean		55,4440	3,10675
	95% Confidence Interval for Mean	Lower Bound	48,4160	
		Upper Bound	62,4720	
	5% Trimmed Mean		56,1900	
	Median		57,9800	
	Variance		96,519	
	Std. Deviation		9,82441	
	Minimum		32,96	
	Maximum		64,50	
	Range		31,54	
	Interquartile Range		8,38	
	Skewness		-1,692	,687
	Kurtosis		2,471	1,334
6,00	Mean		64,7610	1,51788
	95% Confidence Interval for Mean	Lower Bound	61,3273	
		Upper Bound	68,1947	
	5% Trimmed Mean		64,7389	
	Median		65,3950	
	Variance		23,040	
	Std. Deviation		4,79996	
	Minimum		57,35	
	Maximum		72,57	
	Range		15,22	
	Interquartile Range		7,87	
	Skewness		-,115	,687
	Kurtosis		-,765	1,334
7,00	Mean		65,7750	2,89650

	95% Confidence Interval for Mean	Lower Bound	59,2227	
		Upper Bound	72,3273	
	5% Trimmed Mean		66,2483	
	Median		64,7500	
	Variance		83,897	
	Std. Deviation		9,15955	
	Minimum		45,82	
	Maximum		77,21	
	Range		31,39	
	Interquartile Range		12,67	
	Skewness		-,826	,687
	Kurtosis		1,723	1,334
8,00	Mean		54,5280	2,97675
	95% Confidence Interval for Mean	Lower Bound	47,7941	
		Upper Bound	61,2619	
	5% Trimmed Mean		54,7711	
	Median		55,2850	
	Variance		88,611	
	Std. Deviation		9,41332	
	Minimum		38,94	
	Maximum		65,74	
	Range		26,80	
	Interquartile Range		15,80	
	Skewness		-,437	,687
	Kurtosis		-,986	1,334
9,00	Mean		49,5710	2,60016
	95% Confidence Interval for Mean	Lower Bound	43,6890	
		Upper Bound	55,4530	
	5% Trimmed Mean		49,7378	
	Median		50,9500	
	Variance		67,608	
	Std. Deviation		8,22243	
	Minimum		36,75	
	Maximum		59,39	
	Range		22,64	
	Interquartile Range		15,42	
	Skewness		-,478	,687
	Kurtosis		-1,180	1,334

				Statistic	Std. Error
	10,00	Mean		48,3370	1,99830
		95% Confidence Interval for Mean	Lower Bound	43,8165	
			Upper Bound	52,8575	
		5% Trimmed Mean		48,6661	
		Median		50,4600	
		Variance		39,932	
		Std. Deviation		6,31919	
		Minimum		35,56	
		Maximum		55,19	
		Range		19,63	
		Interquartile Range		10,25	
		Skewness		-,907	,687
		Kurtosis		,094	1,334

Tests of Normality

		Kolmogorov-Smirnov[a]			Shapiro-Wilk		
	gruplar	Statistic	df	Sig.	Statistic	df	Sig.
mitokondriyal	1,00	,176	10	,200*	,962	10	,813
	2,00	,249	10	,080	,845	10	,051
	3,00	,246	10	,087	,833	10	,037
	4,00	,135	10	,200*	,971	10	,903
	5,00	,342	10	,002	,783	10	,009
	6,00	,148	10	,200*	,966	10	,855
	7,00	,237	10	,119	,854	10	,065
	8,00	,153	10	,200*	,936	10	,510
	9,00	,156	10	,200*	,914	10	,313
	10,00	,230	10	,144	,893	10	,181

*. This is a lower bound of the true significance.

a. Lilliefors Significance Correction

mitokondriyal

Histograms

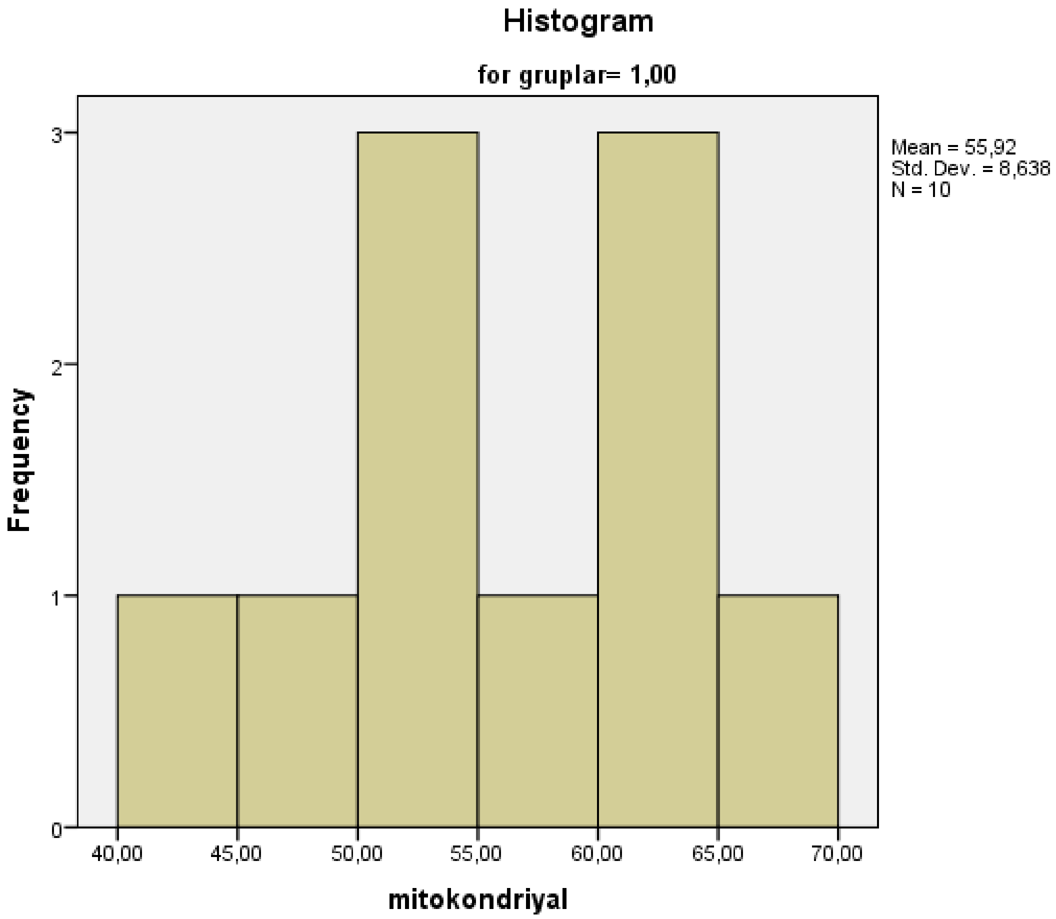
Histogram
for gruplar= 1,00
Mean = 55,92
Std. Dev. = 8,638
N = 10
Frequency
0
1
2
3
40,00
45,00
50,00
55,00
60,00
65,00
70,00
mitokondriyal

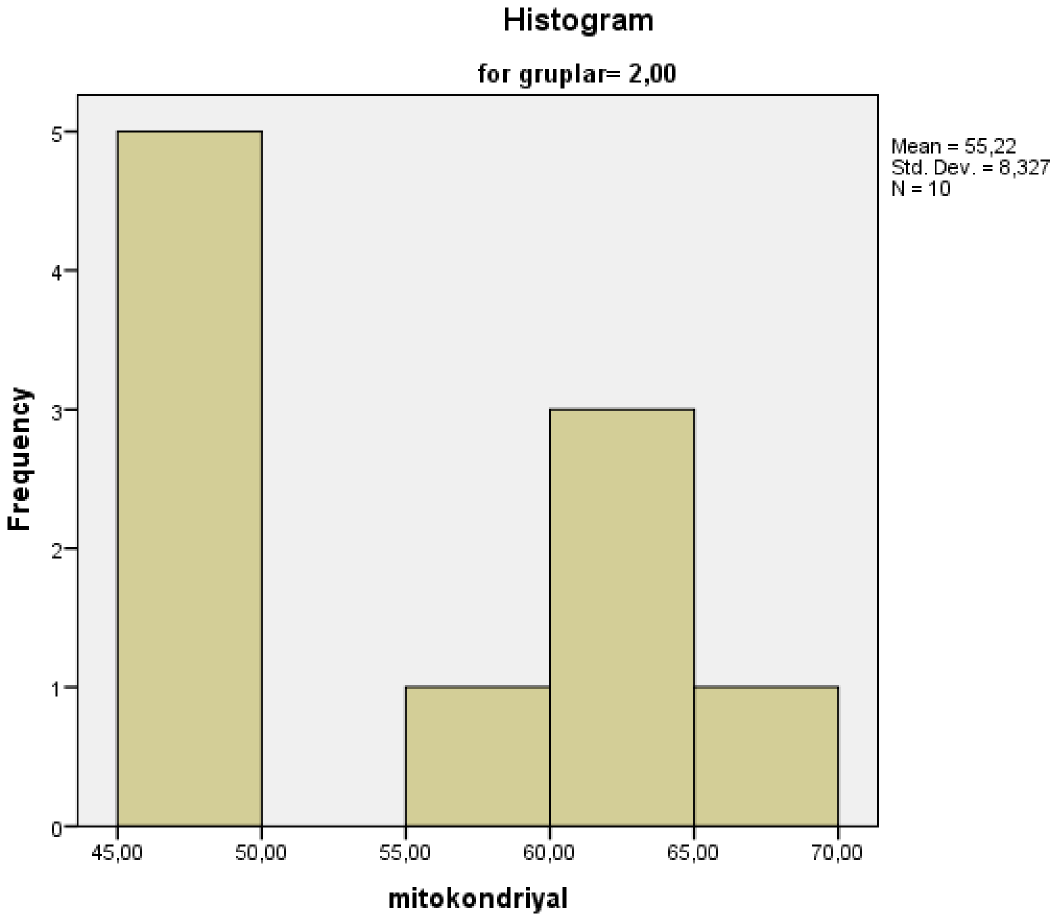
Histogram
for gruplar= 2,00
Mean = 55,22
Std. Dev. = 8,327
N = 10
Frequency
0
1
2
3
4
5
45,00
50,00
55,00
60,00
65,00
70,00
mitokondriyal

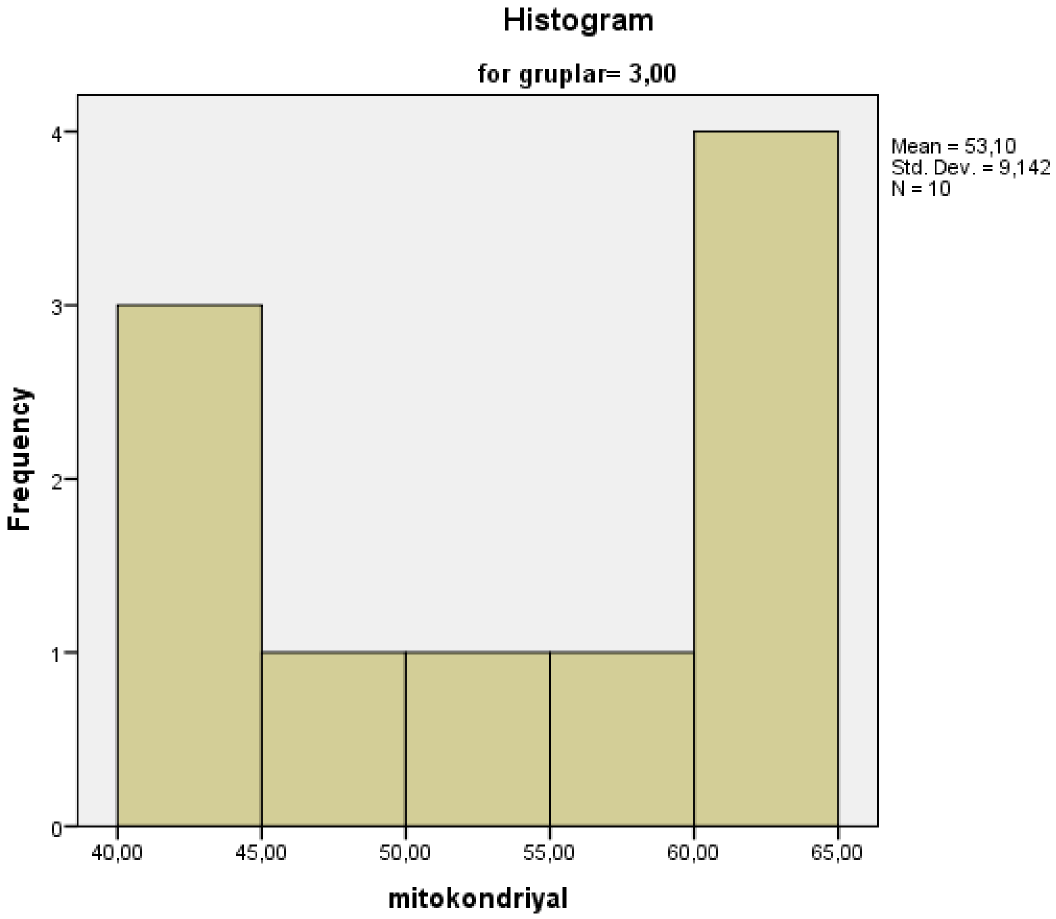

Histogram
for gruplar= 3,00
Mean = 53,10
Std. Dev. = 9,142
N = 10
Frequency
0
1
2
3
4
40,00
45,00
50,00
55,00
60,00
65,00
mitokondriyal

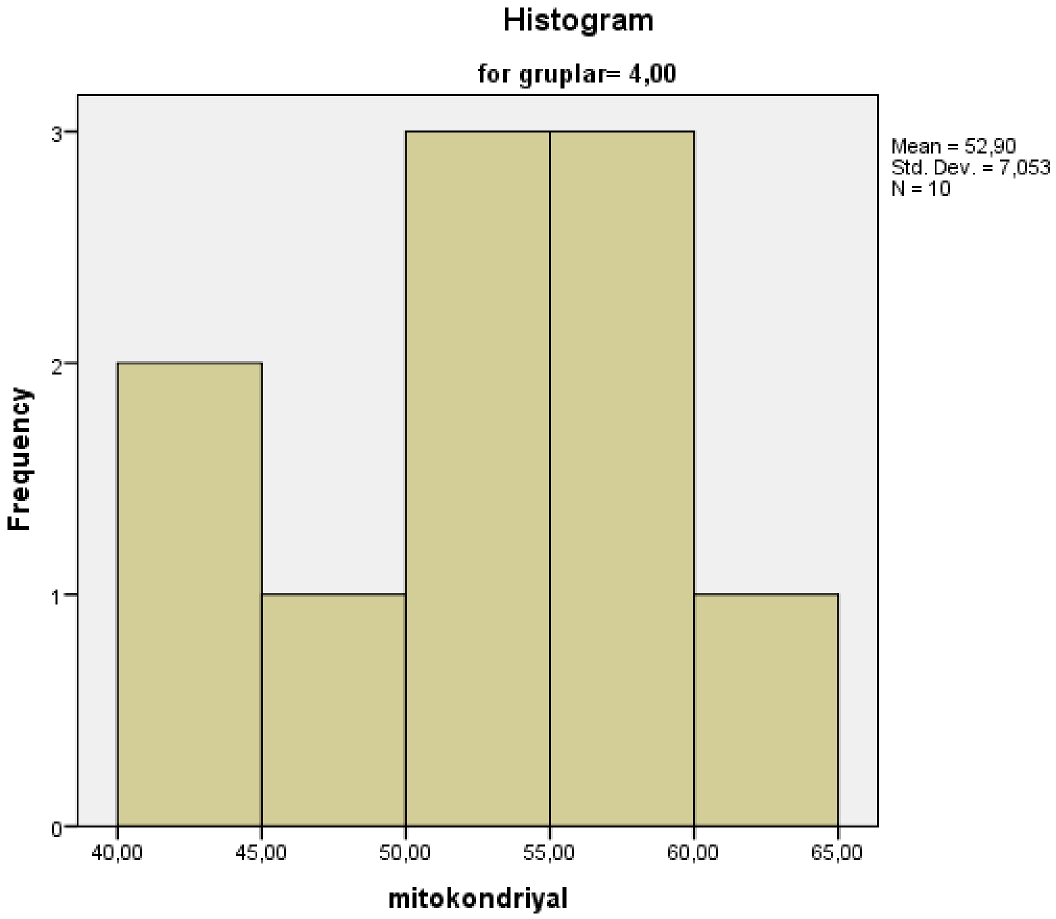
Histogram
for gruplar= 4,00
Mean = 52,90
Std. Dev. = 7,053
N = 10
Frequency
0
1
2
3
40,00
45,00
50,00
55,00
60,00
65,00
mitokondriyal

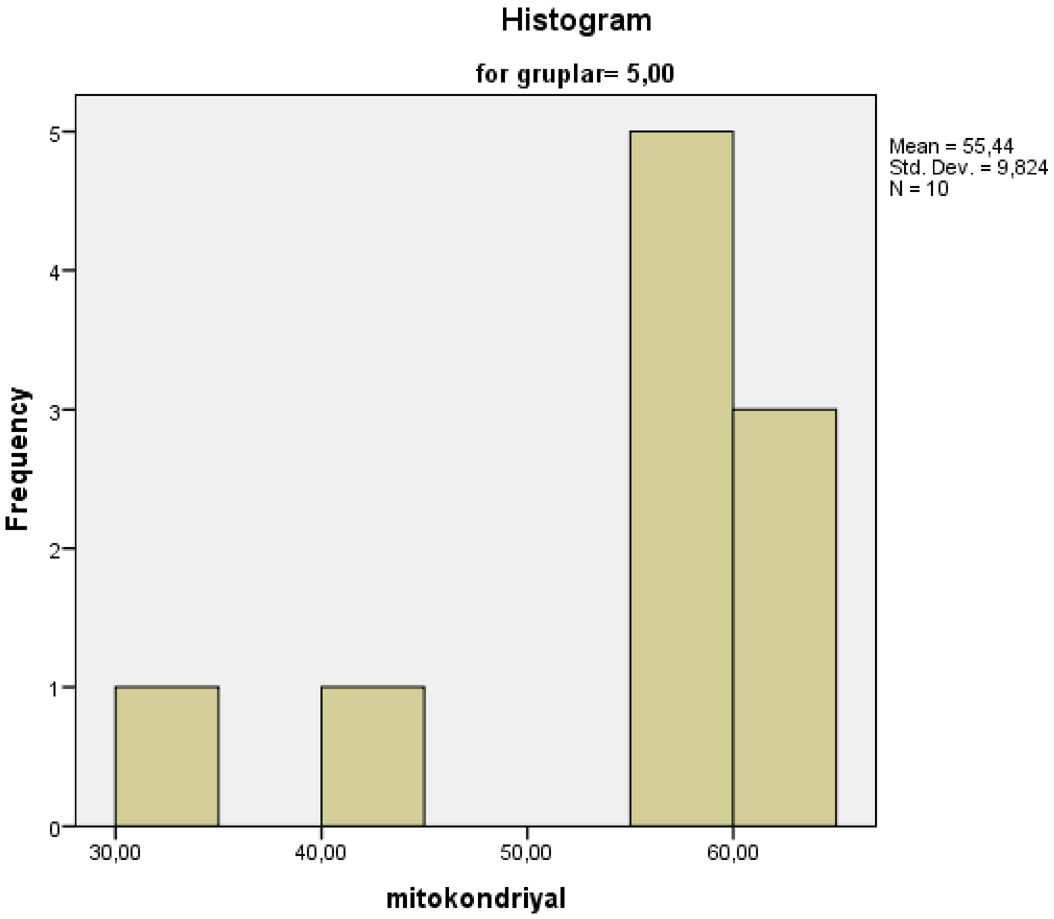

Histogram
for gruplar= 5,00
Mean = 55,44
Std. Dev. = 9,824
N = 10
Frequency
0
1
2
3
4
5
30,00
40,00
50,00
60,00
mitokondriyal

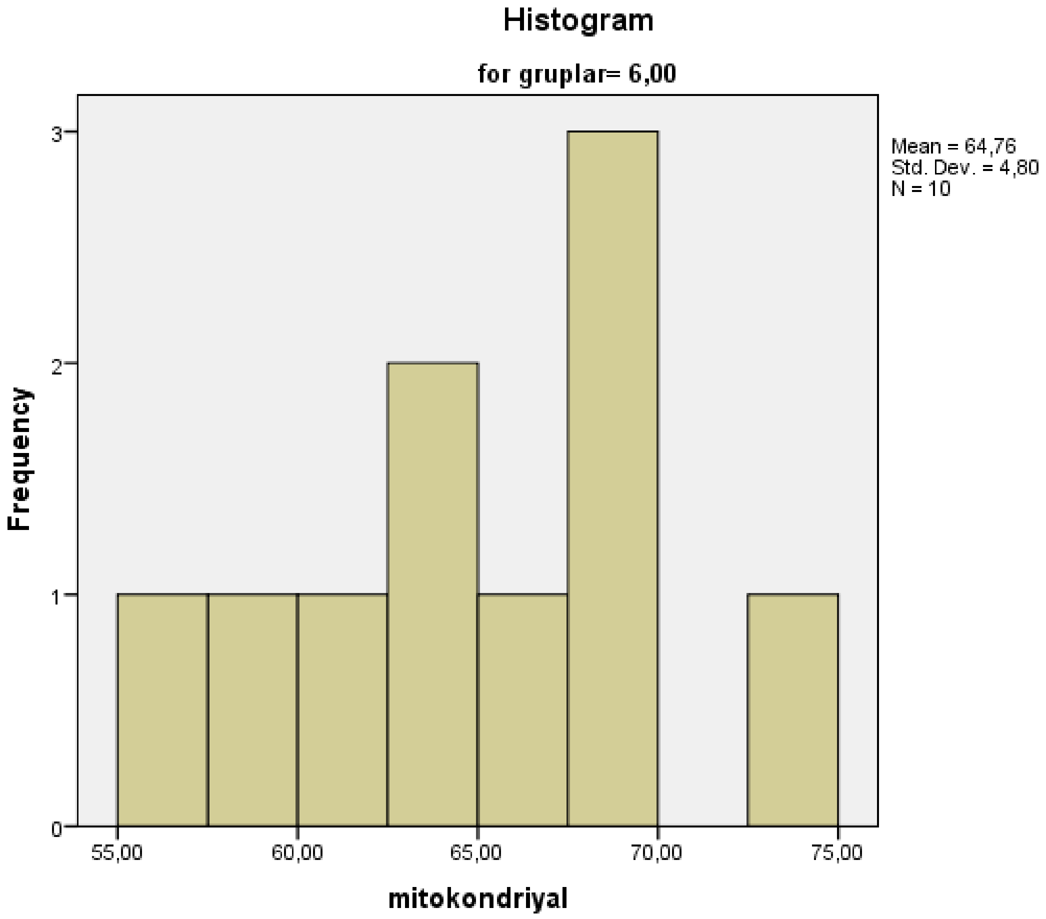

Histogram
for gruplar= 6,00
Mean = 64,76
Std. Dev. = 4,80
N = 10
Frequency
3
2
1
0
55,00
60,00
65,00
70,00
75,00
mitokondriyal

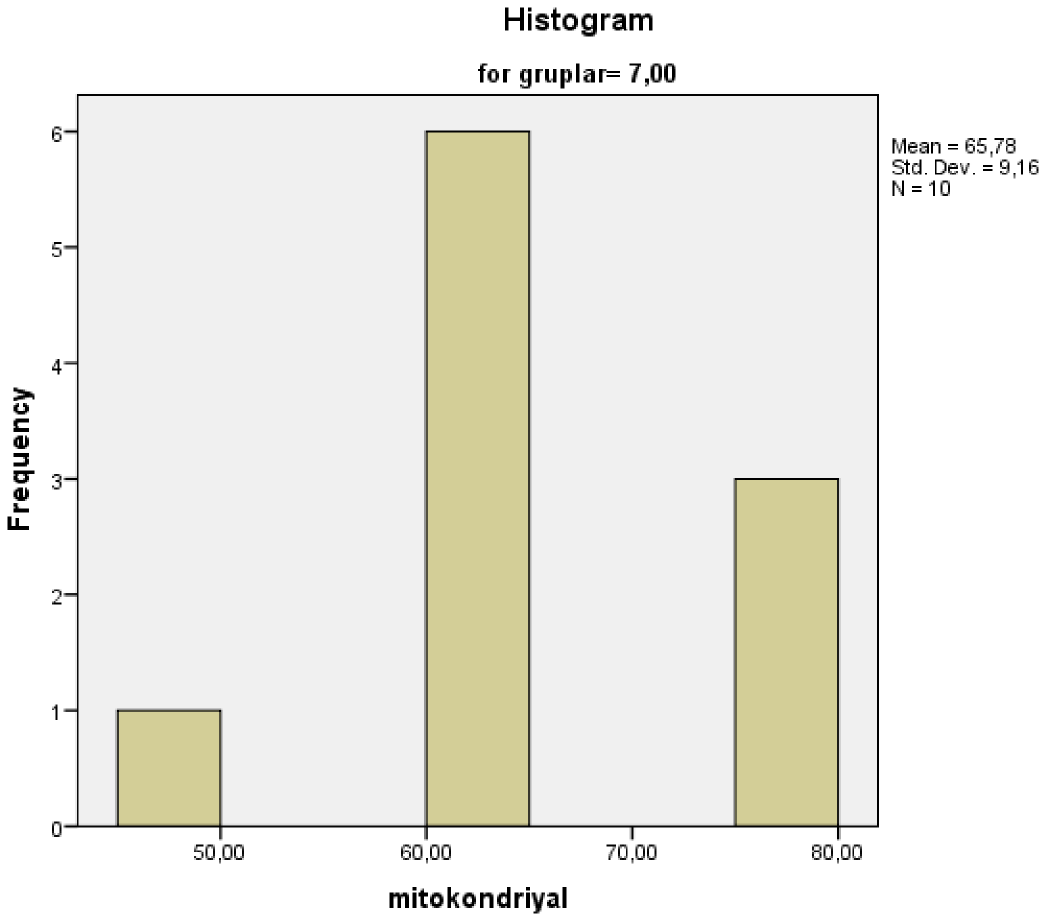
Histogram
for gruplar= 7,00
Mean = 65,78
Std. Dev. = 9,16
N = 10
Frequency
0
1
2
3
4
5
6
50,00
60,00
70,00
80,00
mitokondriyal

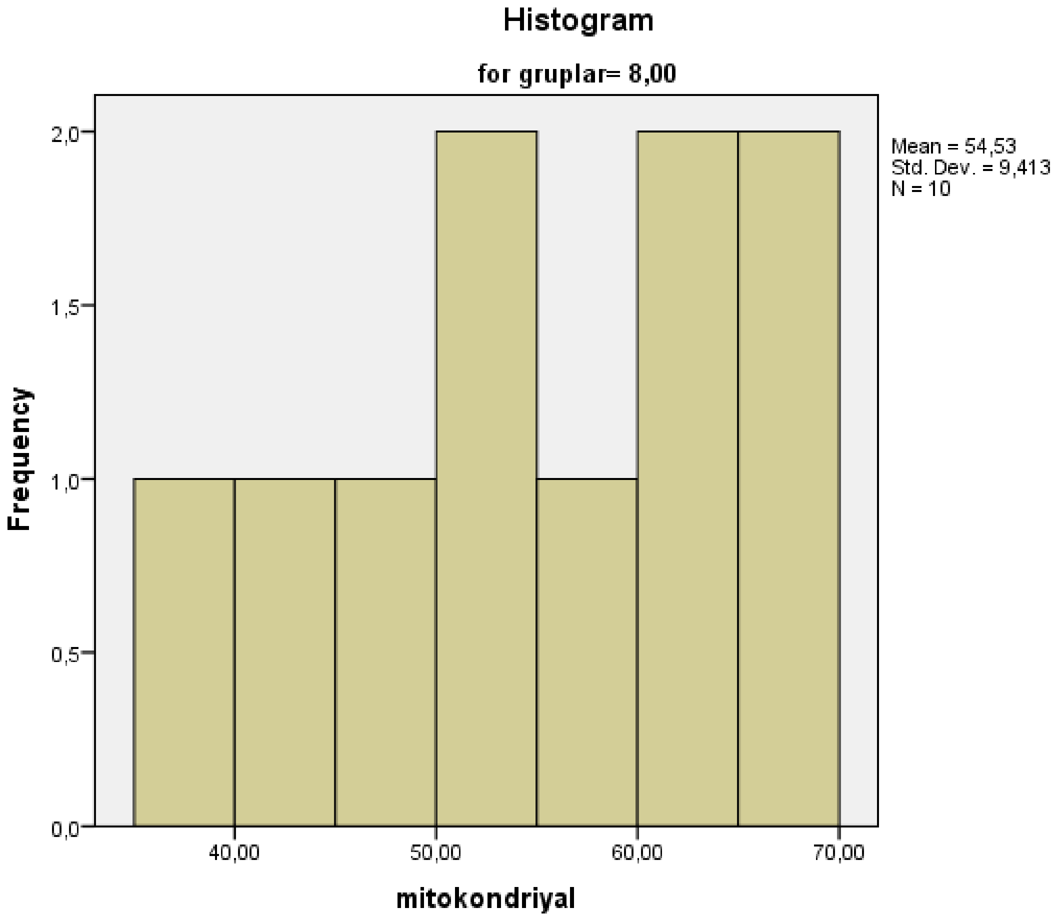
Histogram
for gruplar= 8,00
Mean = 54,53
Std. Dev. = 9,413
N = 10
2,0
1,5
1,0
0,5
0,0
Frequency
40,00
50,00
60,00
70,00
mitokondriyal

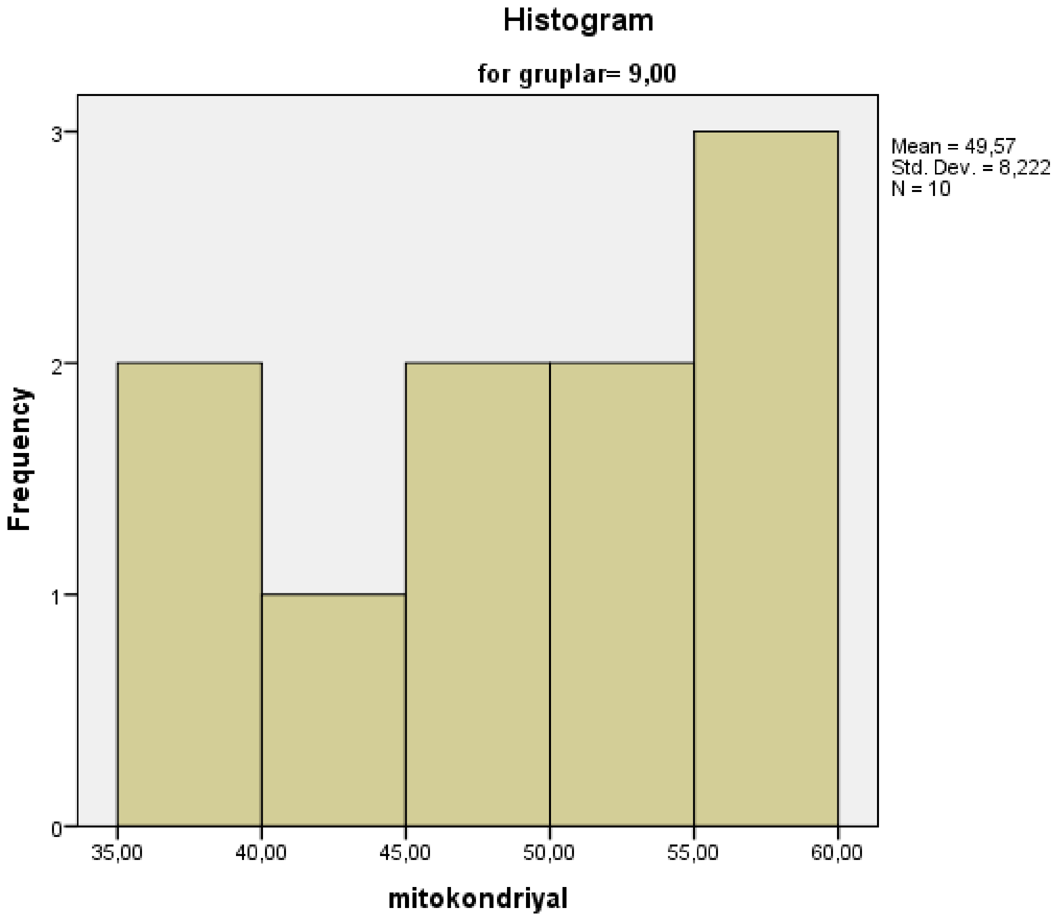

Histogram
for gruplar= 9,00
Mean = 49,57
Std. Dev. = 8,222
N = 10
Frequency
0
1
2
3
35,00
40,00
45,00
50,00
55,00
60,00
mitokondriyal

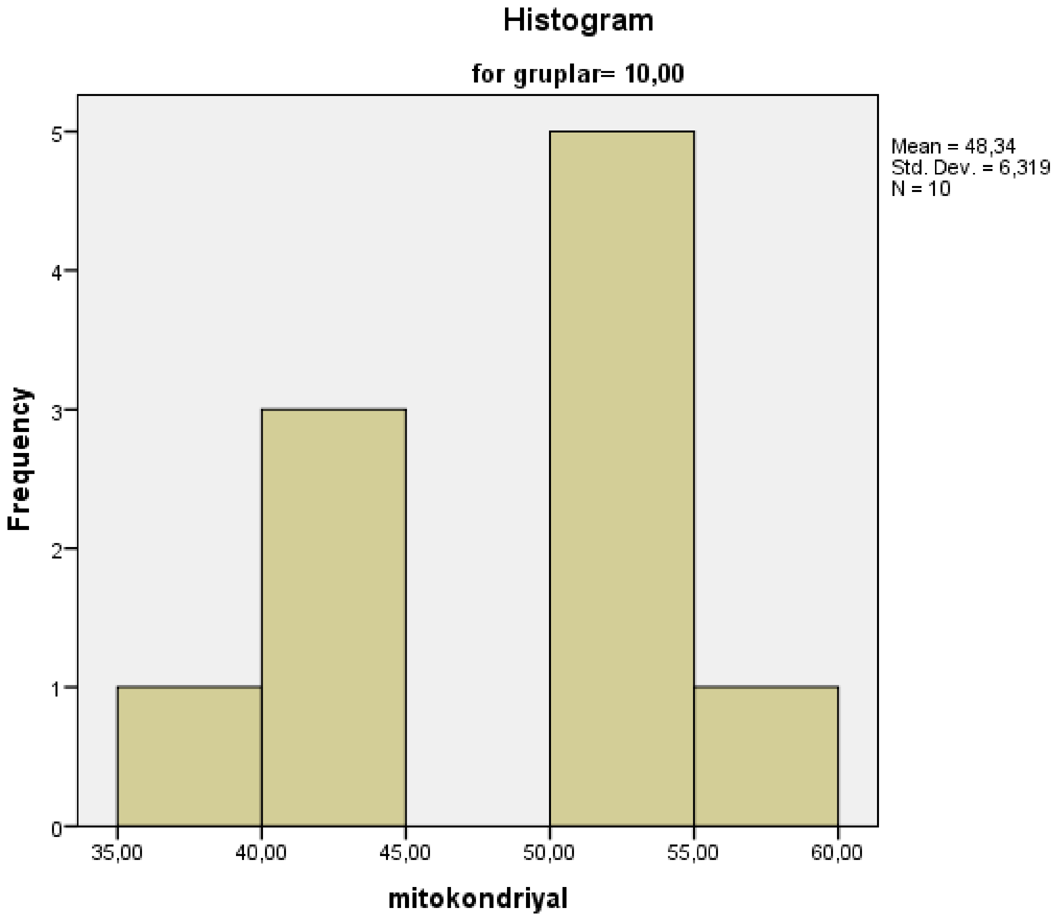

Stem-and-Leaf Plots

```
mitokondriyal Stem-and-Leaf Plot for
gruplar= 1,00

 Frequency    Stem &  Leaf

     1,00        4 .  0
     1,00        4 .  8
     3,00        5 .  013
     1,00        5 .  8
     3,00        6 .  222
     1,00        6 .  9

 Stem width:  10,00
 Each leaf:       1 case(s)
```

mitokondriyal Stem-and-Leaf Plot for
gruplar= 2,00

Frequency	Stem &	Leaf
5,00	4 .	56799
,00	5 .	
1,00	5 .	9
3,00	6 .	034
1,00	6 .	5

Stem width: 10,00
Each leaf: 1 case(s)

mitokondriyal Stem-and-Leaf Plot for
gruplar= 3,00

Frequency	Stem &	Leaf
3,00	4 .	111
1,00	4 .	8
1,00	5 .	1
1,00	5 .	9
4,00	6 .	0113

Stem width: 10,00
Each leaf: 1 case(s)

mitokondriyal Stem-and-Leaf Plot for
gruplar= 4,00

Frequency	Stem &	Leaf
2,00	4 .	03
1,00	4 .	8
3,00	5 .	124
3,00	5 .	679
1,00	6 .	3

Stem width: 10,00
Each leaf: 1 case(s)

mitokondriyal Stem-and-Leaf Plot for
gruplar= 5,00

```
 Frequency    Stem &  Leaf

     2,00 Extremes    (=<43)
     5,00        5 .  67789
     3,00        6 .  044

 Stem width:  10,00
 Each leaf:       1 case(s)
```

mitokondriyal Stem-and-Leaf Plot for
gruplar= 6,00

```
 Frequency    Stem &  Leaf

     2,00        5 .  78
     3,00        6 .  034
     4,00        6 .  6778
     1,00        7 .  2

 Stem width:  10,00
 Each leaf:       1 case(s)
```

mitokondriyal Stem-and-Leaf Plot for
gruplar= 7,00

```
 Frequency    Stem &  Leaf

     1,00        4 .  5
      ,00        5 .
     6,00        6 .  133444
     3,00        7 .  557

 Stem width:  10,00
 Each leaf:       1 case(s)
```

mitokondriyal Stem-and-Leaf Plot for
gruplar= 8,00

```
 Frequency    Stem &  Leaf

     1,00        3 .  8
     2,00        4 .  18
     3,00        5 .  036
     4,00        6 .  0255

 Stem width:  10,00
```

```
 Each leaf:       1 case(s)
```

```
mitokondriyal Stem-and-Leaf Plot for
gruplar= 9,00

 Frequency    Stem &  Leaf

     2,00        3 .  68
     1,00        4 .  1
     2,00        4 .  89
     2,00        5 .  24
     3,00        5 .  589

 Stem width:  10,00
 Each leaf:       1 case(s)
```

```
mitokondriyal Stem-and-Leaf Plot for
gruplar= 10,00

 Frequency    Stem &  Leaf

     1,00        3 .  5
     3,00        4 .  334
      ,00        4 .
     5,00        5 .  00233
     1,00        5 .  5

 Stem width:  10,00
 Each leaf:       1 case(s)
```

Normal Q-Q Plots

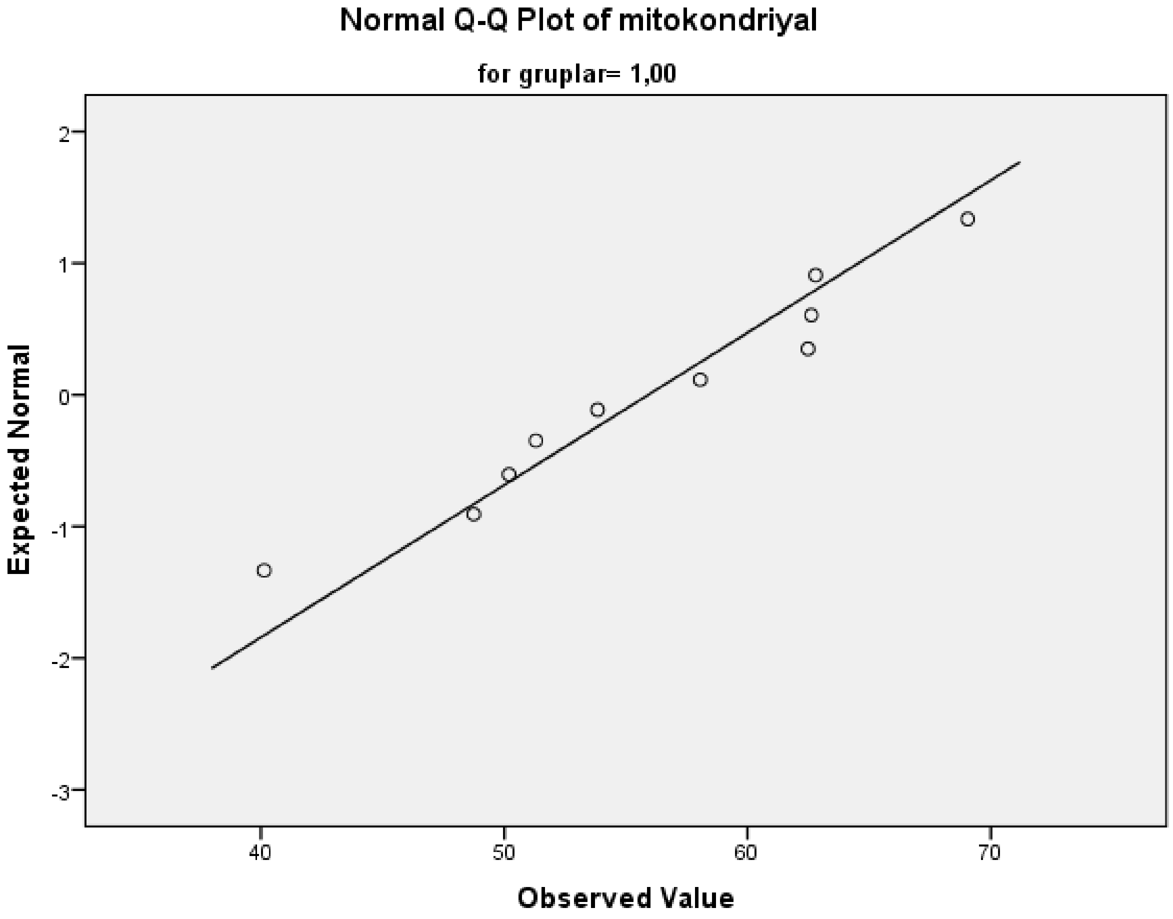
Normal Q-Q Plot of mitokondriyal
for gruplar= 1,00
Expected Normal
Observed Value
2
1
0
-1
-2
-3
40
50
60
70

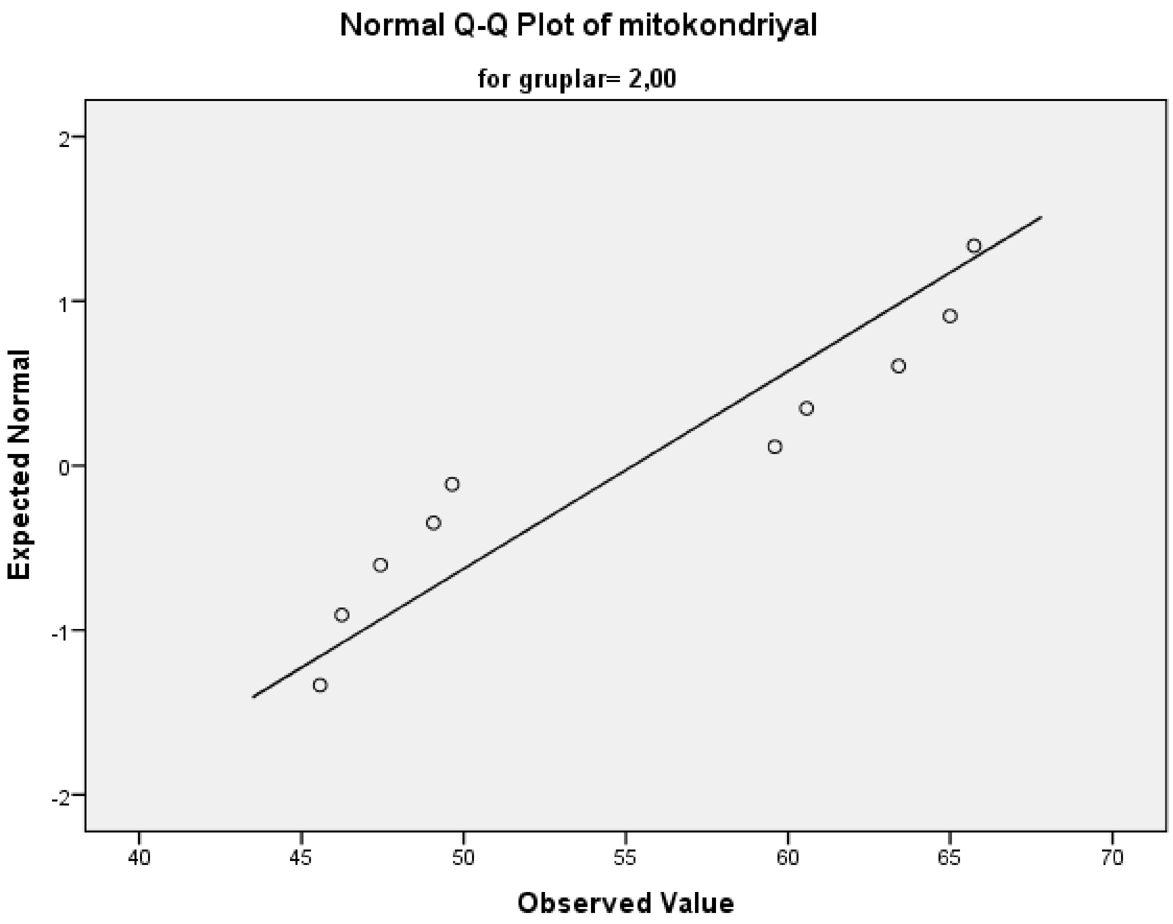
Normal Q-Q Plot of mitokondriyal
for gruplar= 2,00
Expected Normal
Observed Value
2
1
0
-1
-2
40
45
50
55
60
65
70

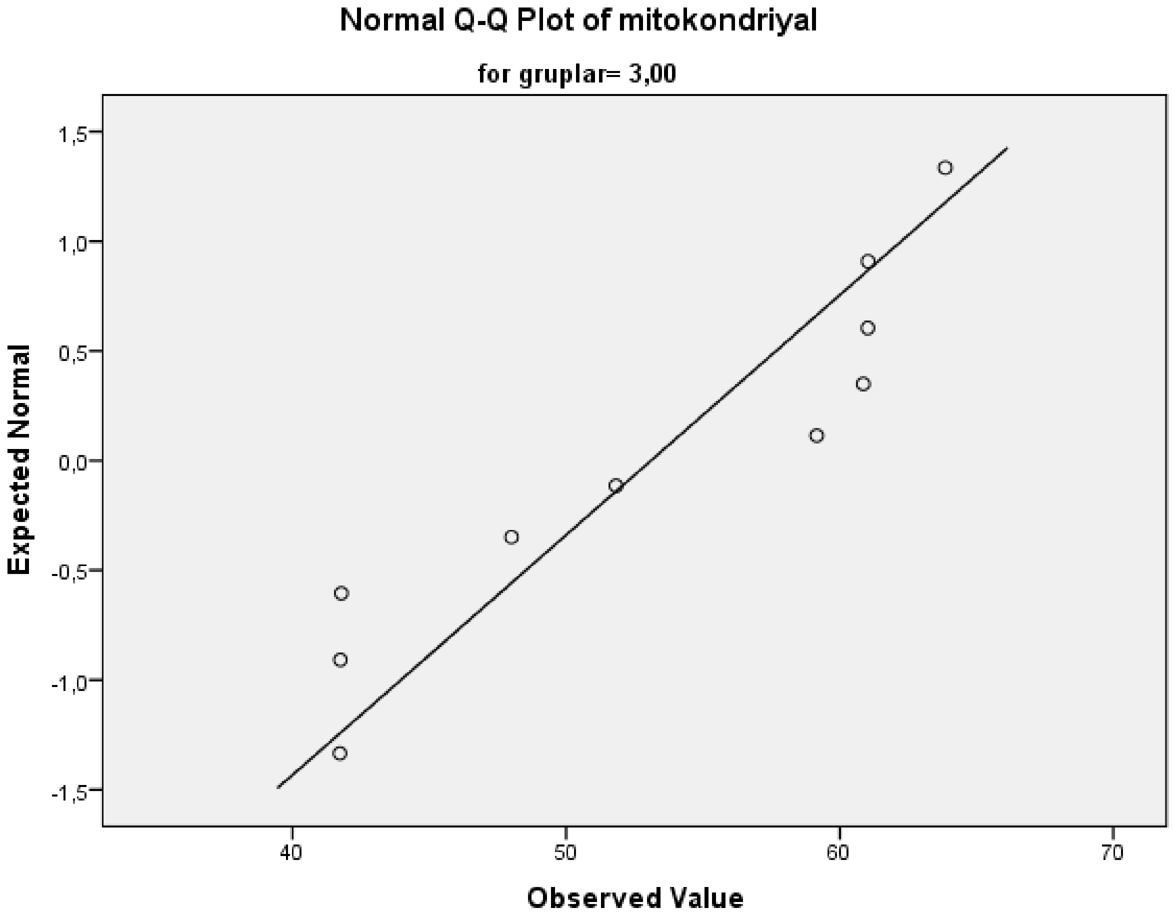
Normal Q-Q Plot of mitokondriyal
for gruplar= 3,00
Expected Normal
1,5
1,0
0,5
0,0
-0,5
-1,0
-1,5
40
50
60
70
Observed Value

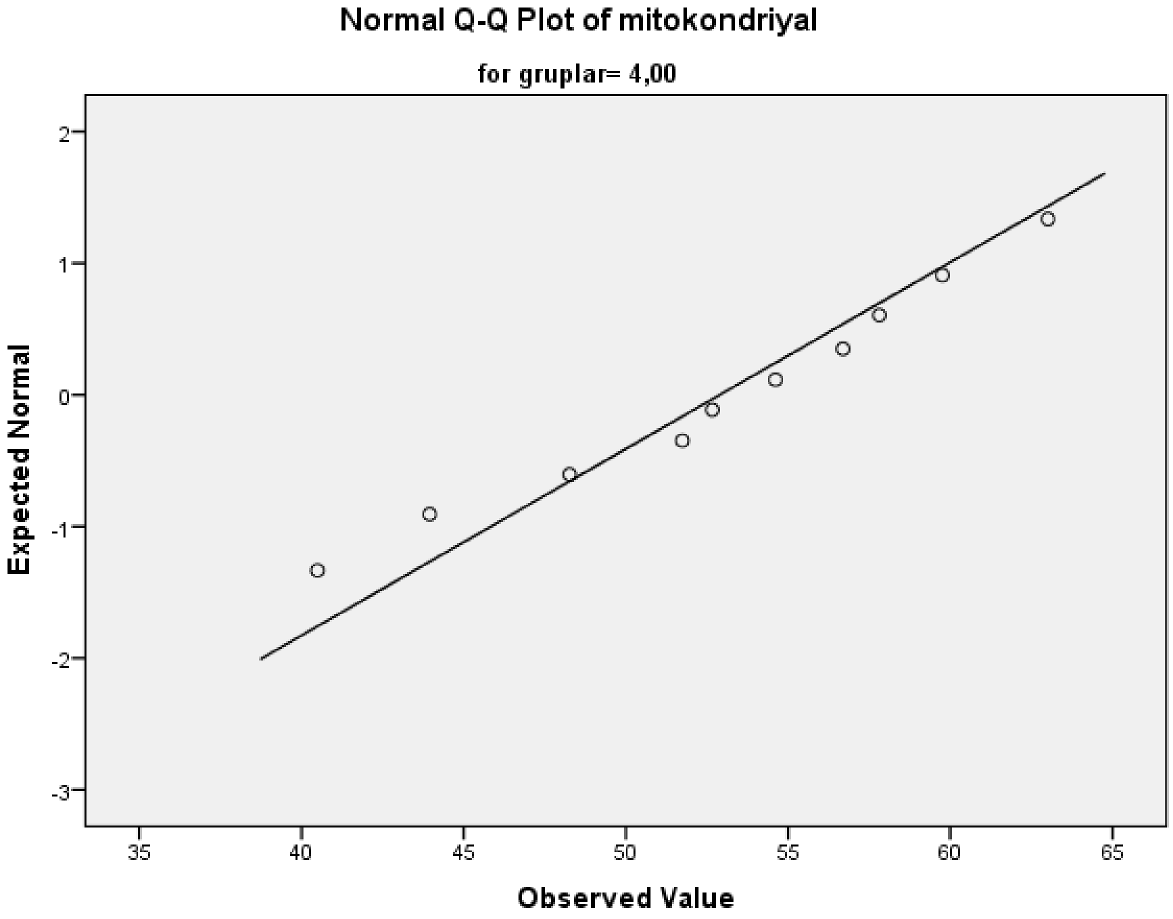
Normal Q-Q Plot of mitokondriyal
for gruplar= 4,00
Expected Normal
Observed Value
2
1
0
-1
-2
-3
35
40
45
50
55
60
65

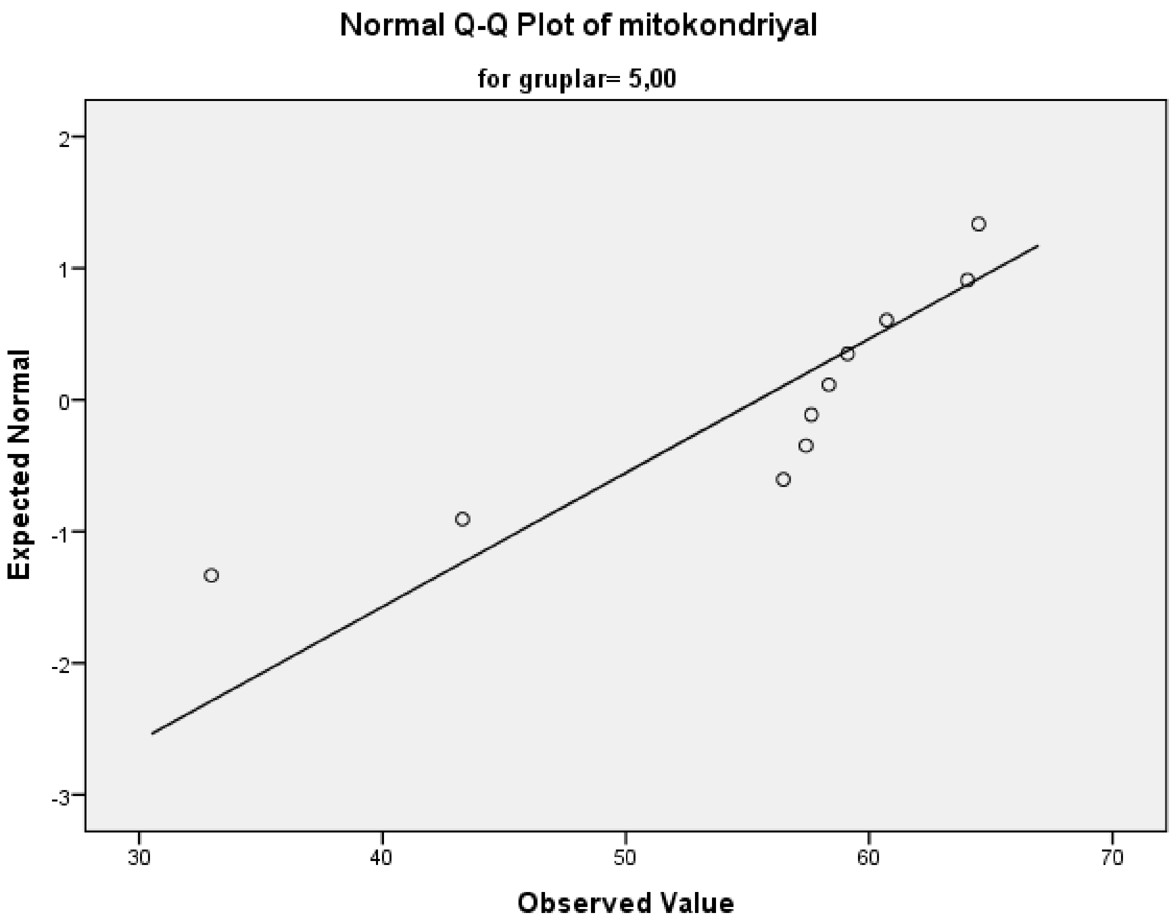
Normal Q-Q Plot of mitokondriyal
for gruplar= 5,00
Expected Normal
Observed Value
2
1
0
-1
-2
-3
30
40
50
60
70

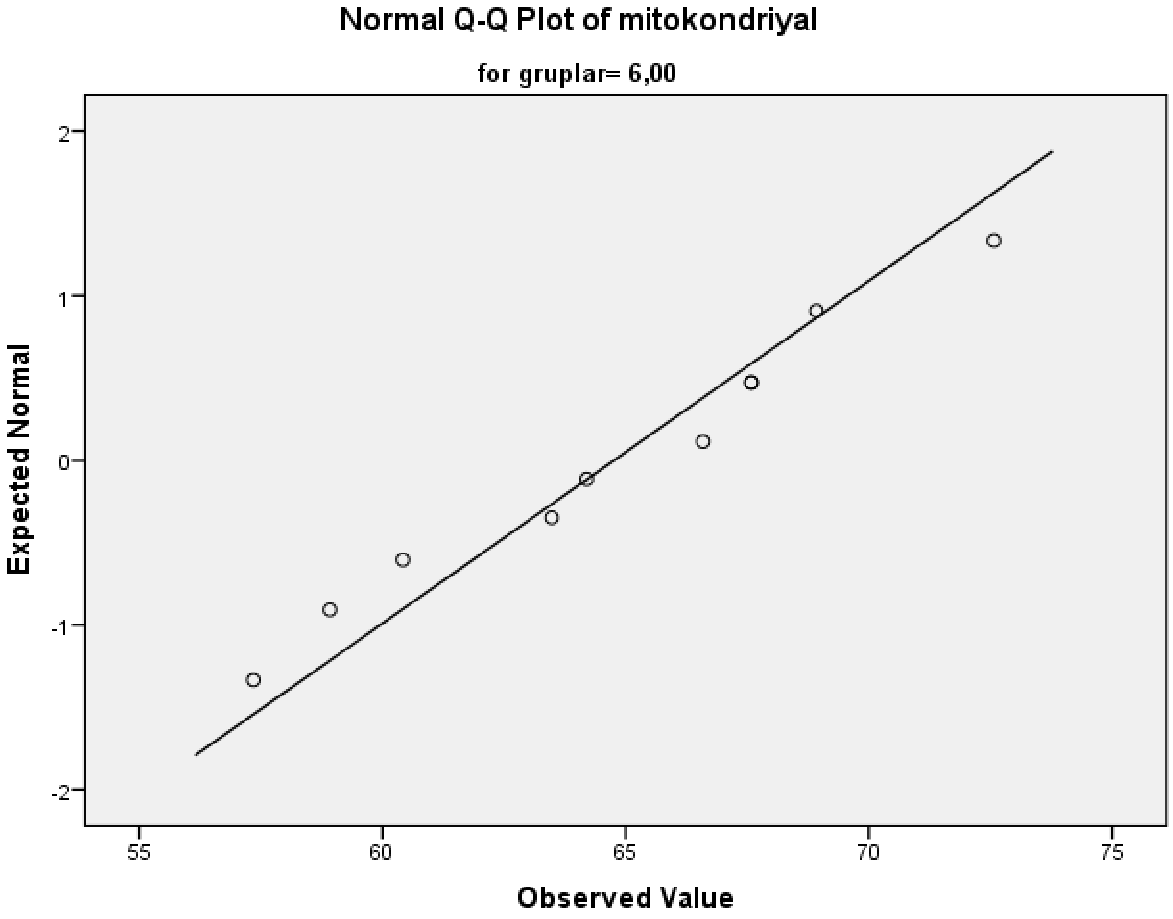

Normal Q-Q Plot of mitokondriyal
for gruplar= 6,00
Expected Normal
Observed Value
2
1
0
-1
-2
55
60
65
70
75

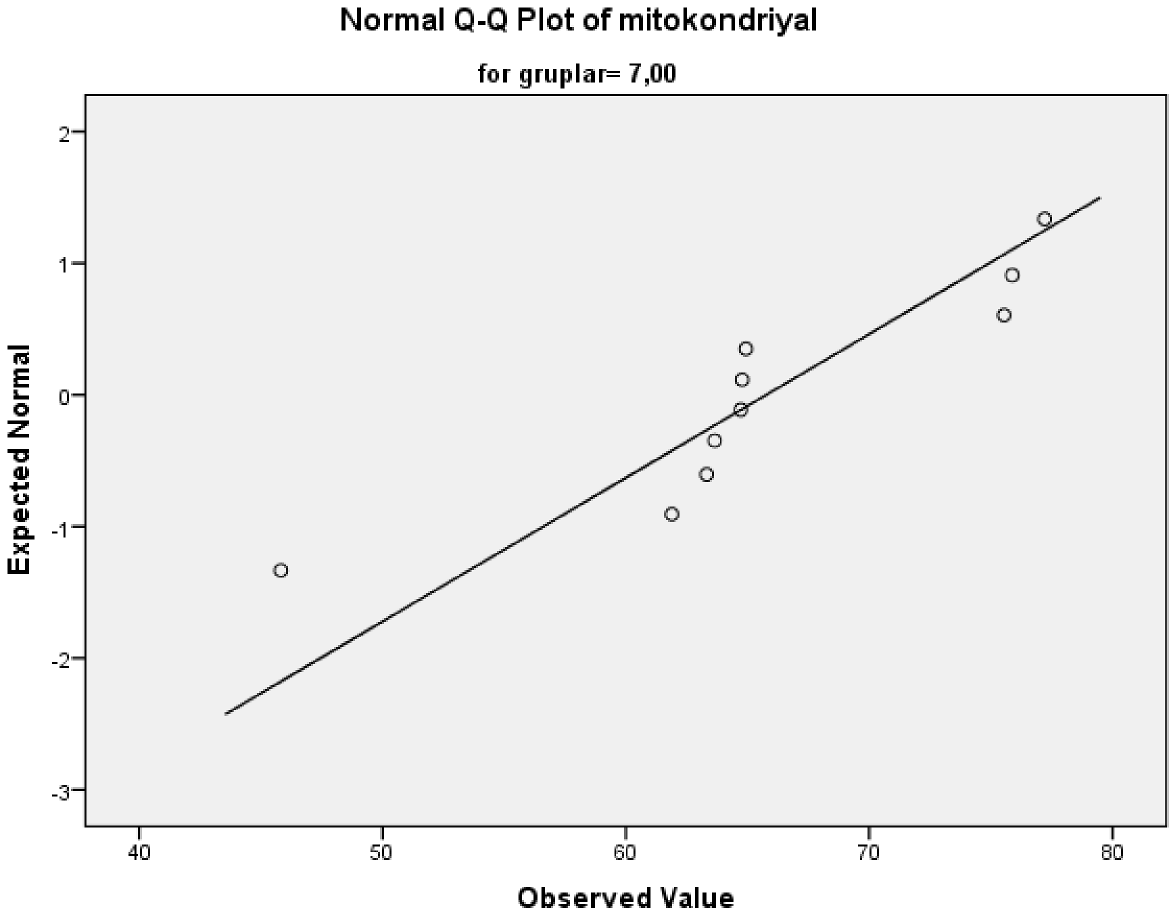

Normal Q-Q Plot of mitokondriyal
for gruplar= 7,00
Expected Normal
Observed Value
2
1
0
-1
-2
-3
40
50
60
70
80

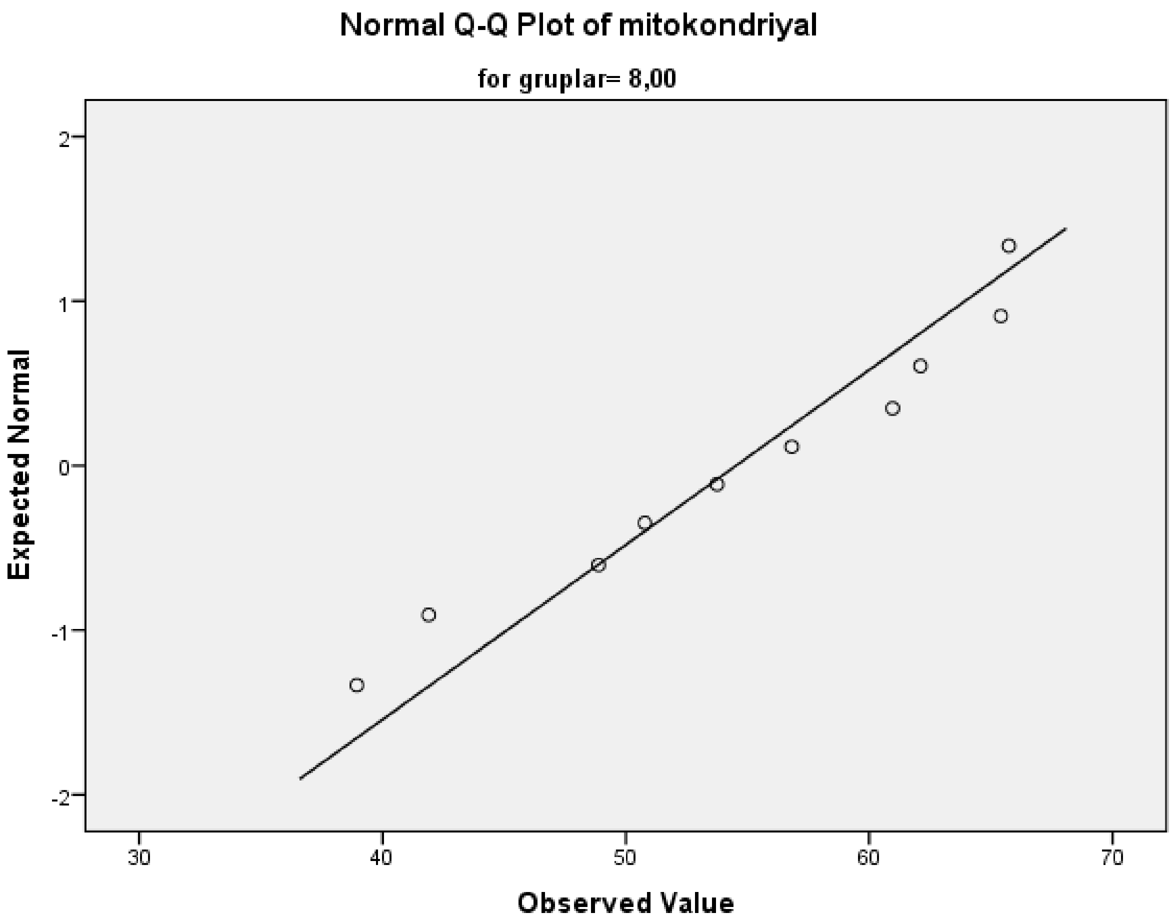
Normal Q-Q Plot of mitokondriyal
for gruplar= 8,00
Expected Normal
Observed Value
2
1
0
-1
-2
30
40
50
60
70

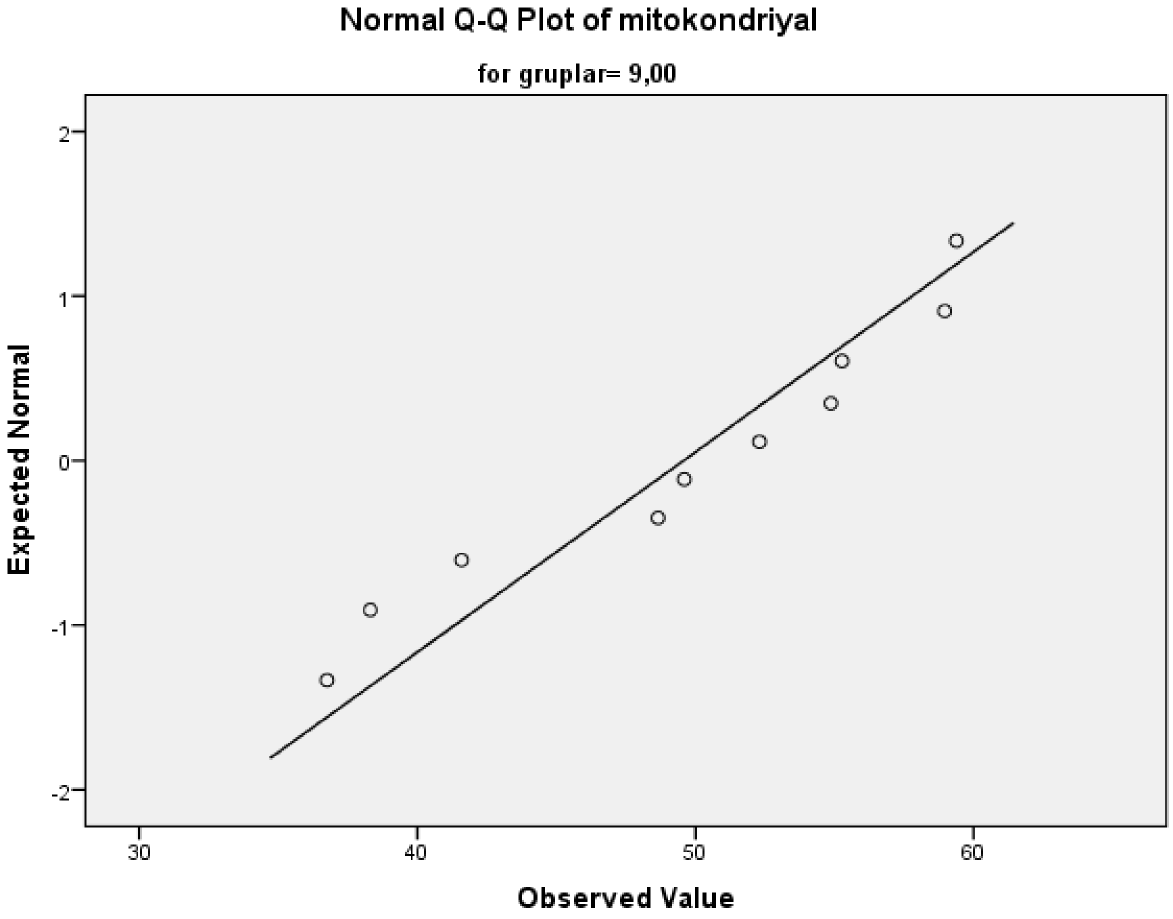

Normal Q-Q Plot of mitokondriyal
for gruplar= 9,00
Expected Normal
2
1
0
-1
-2
30
40
50
60
Observed Value

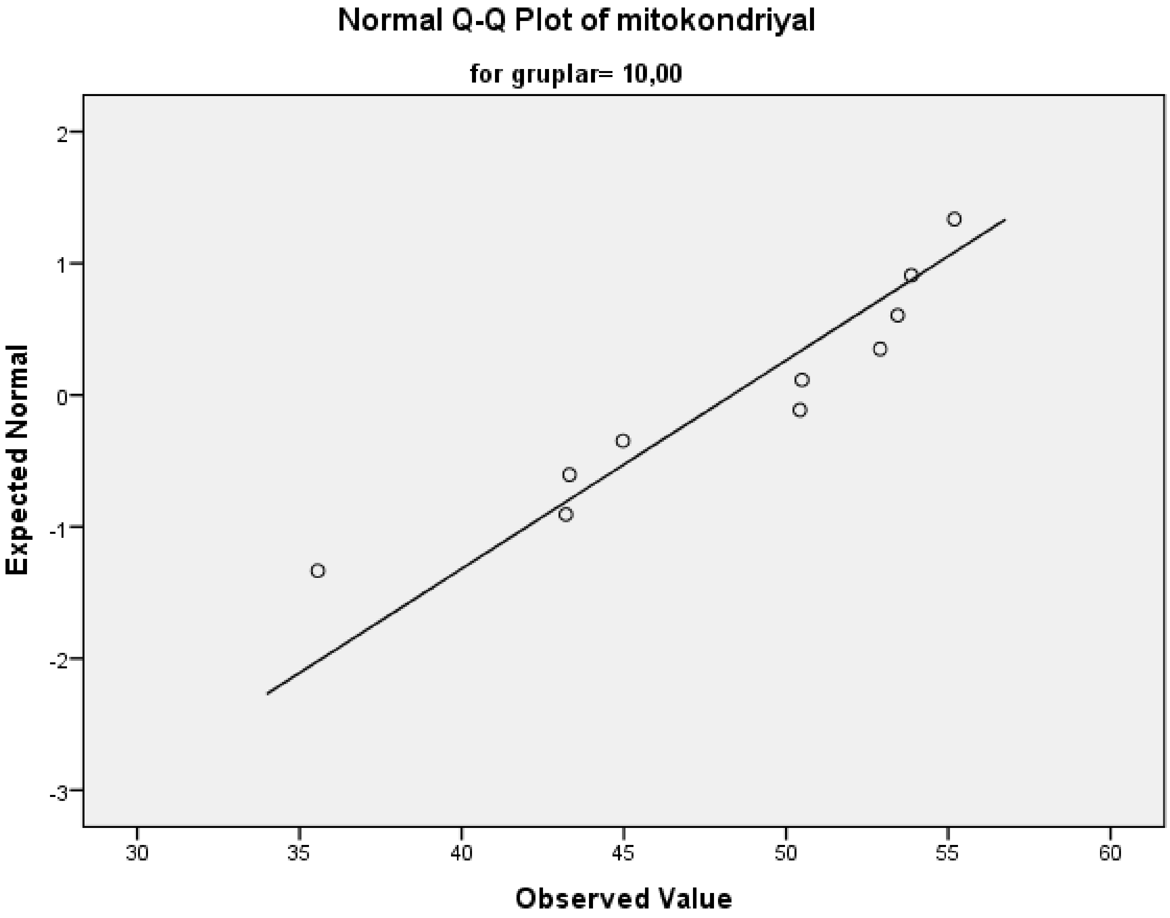

Detrended Normal Q-Q Plots

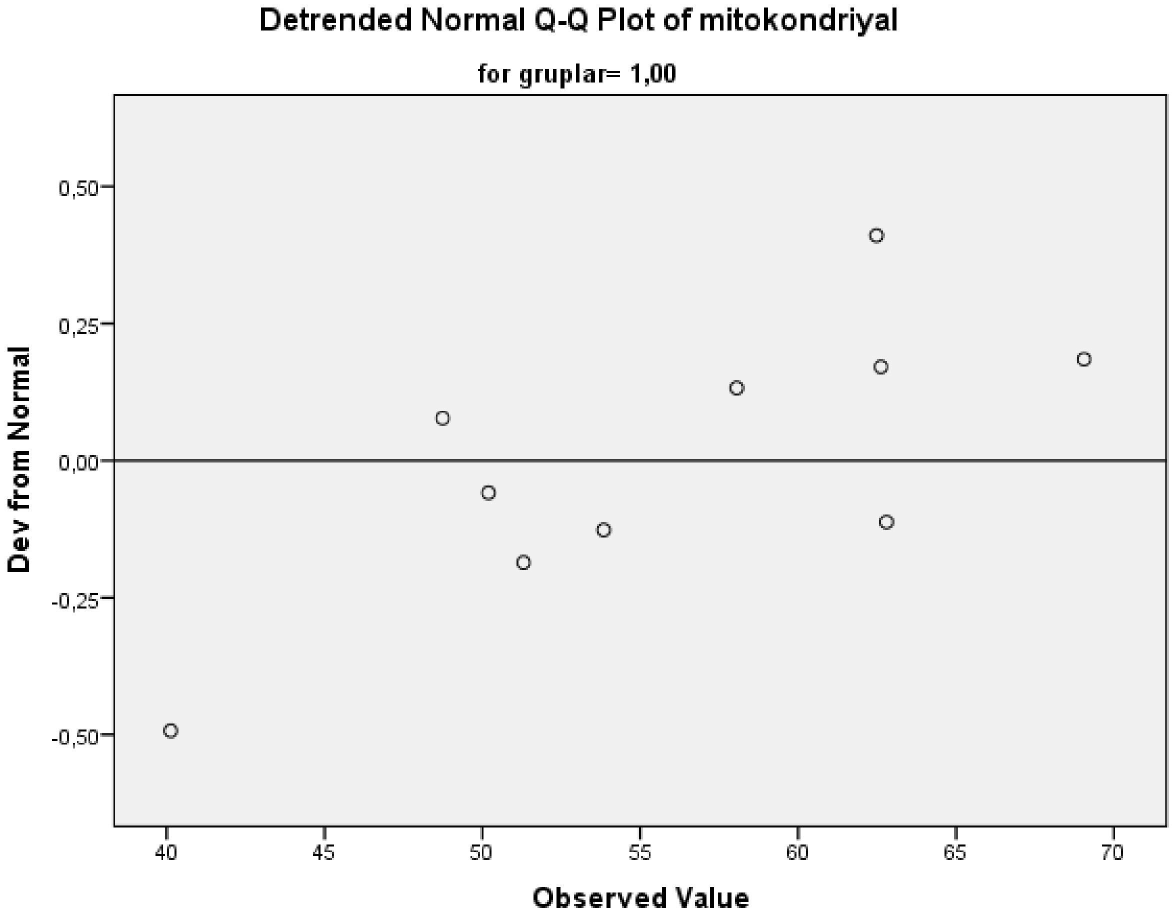

Detrended Normal Q-Q Plot of mitokondriyal
for gruplar= 1,00
Dev from Normal
0,50
0,25
0,00
-0,25
-0,50
40
45
50
55
60
65
70
Observed Value

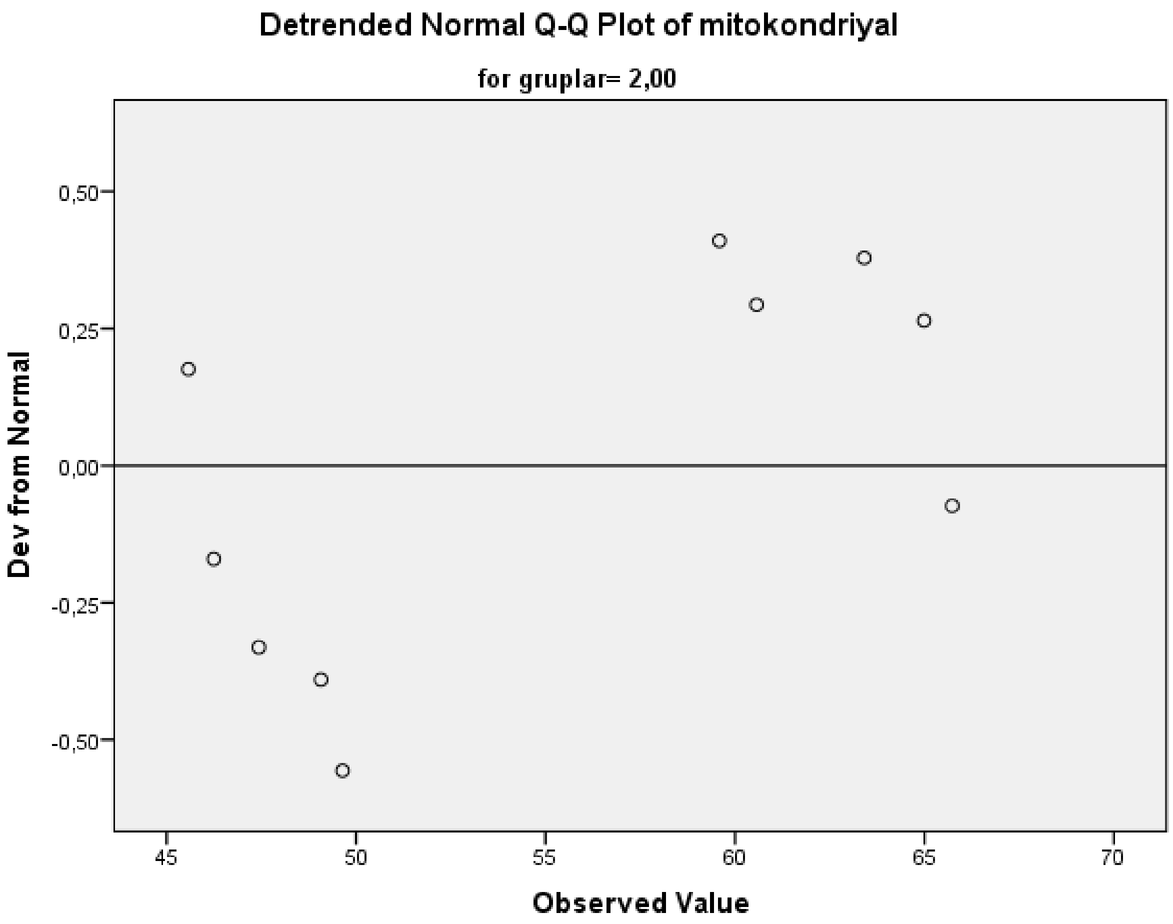

Detrended Normal Q-Q Plot of mitokondriyal
for gruplar= 2,00
Dev from Normal
0,50
0,25
0,00
-0,25
-0,50
45
50
55
60
65
70
Observed Value

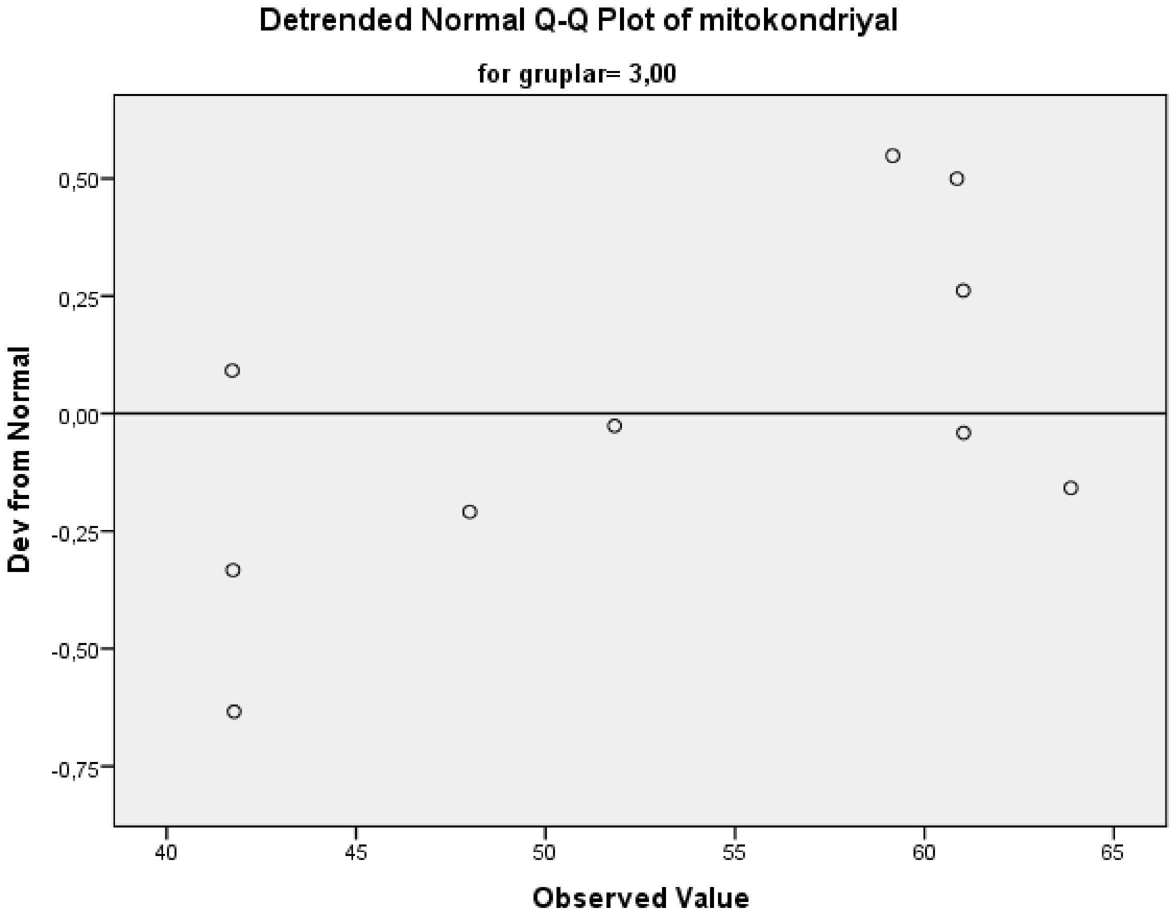
Detrended Normal Q-Q Plot of mitokondriyal
for gruplar= 3,00
Dev from Normal
Observed Value
0,50
0,25
0,00
-0,25
-0,50
-0,75
40
45
50
55
60
65

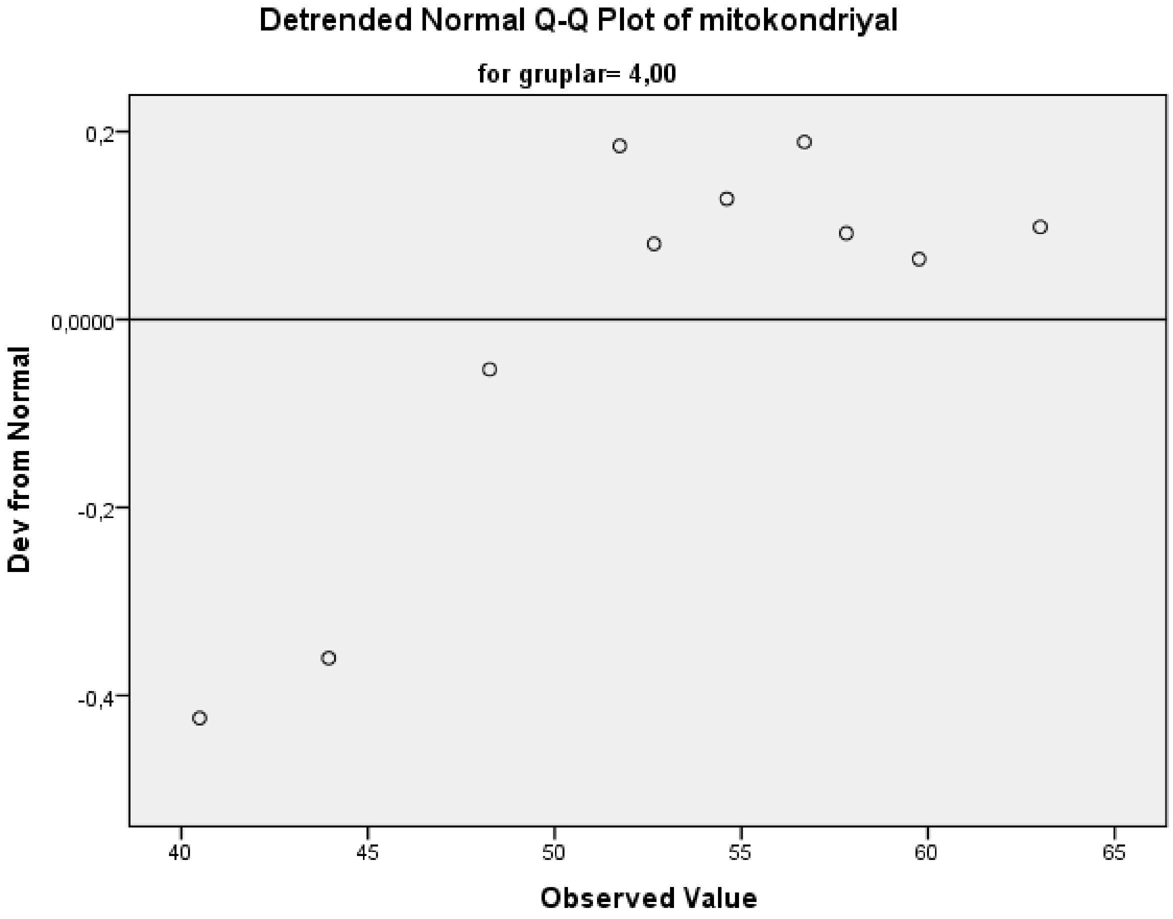

Detrended Normal Q-Q Plot of mitokondriyal
for gruplar= 4,00
Dev from Normal
0,2
0,0000
-0,2
-0,4
Observed Value
40
45
50
55
60
65

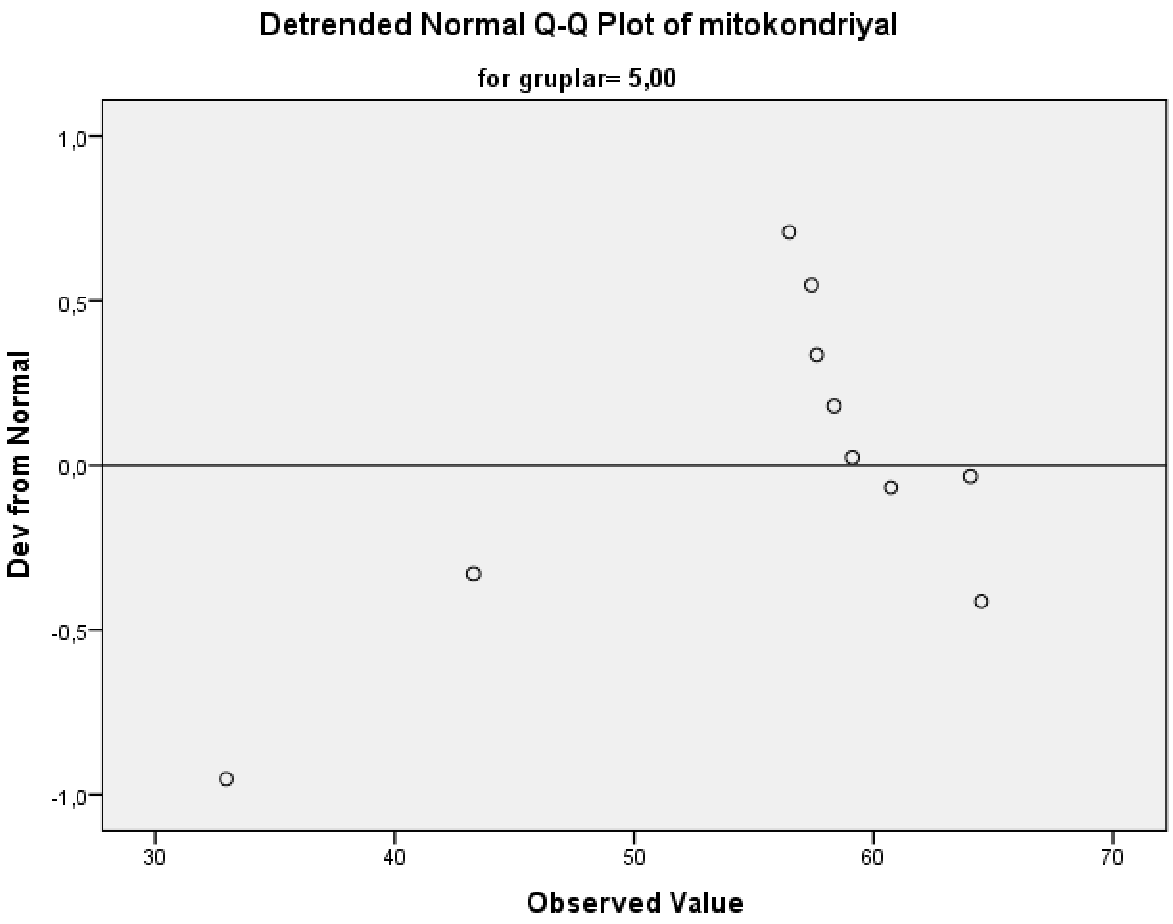
Detrended Normal Q-Q Plot of mitokondriyal
for gruplar= 5,00
Dev from Normal
1,0
0,5
0,0
-0,5
-1,0
30
40
50
60
70
Observed Value

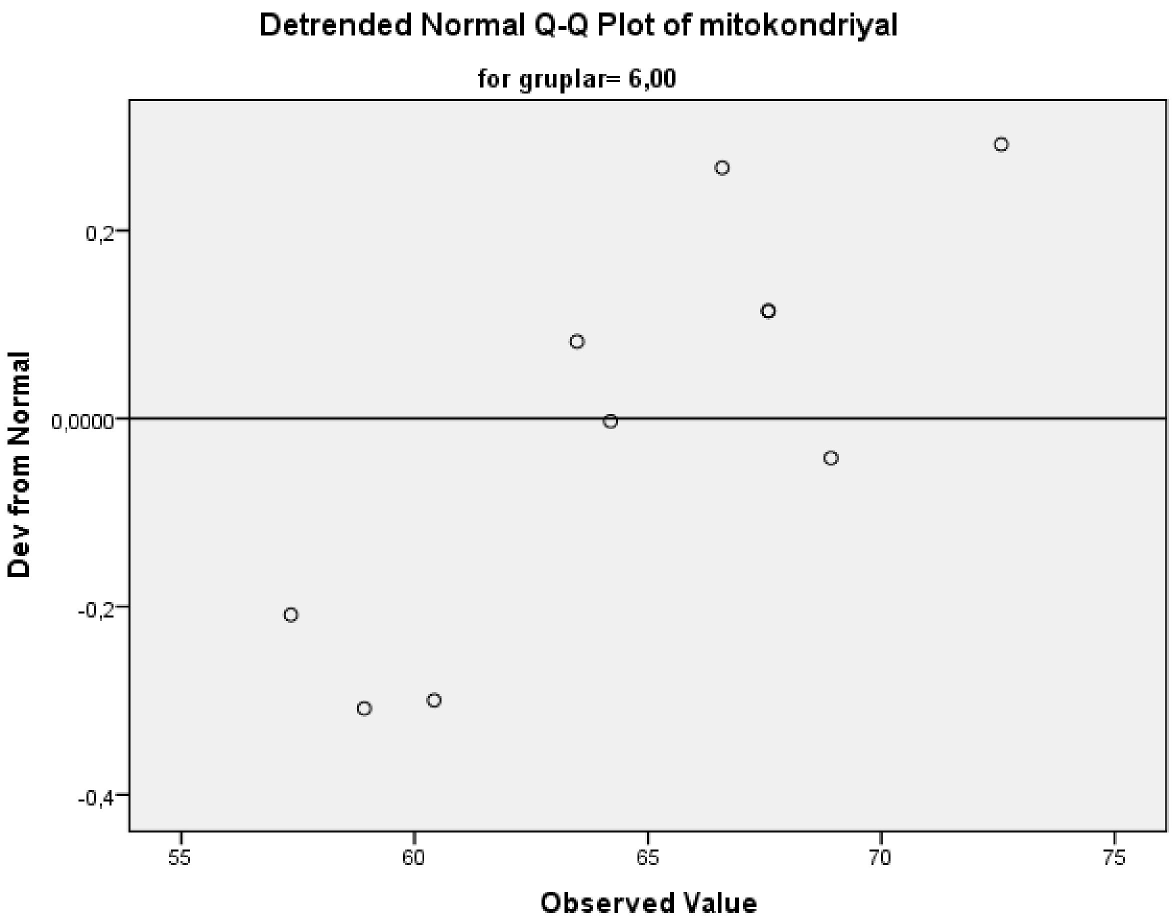

Detrended Normal Q-Q Plot of mitokondriyal
for gruplar= 6,00
Dev from Normal
0,2
0,0000
-0,2
-0,4
55
60
65
70
75
Observed Value

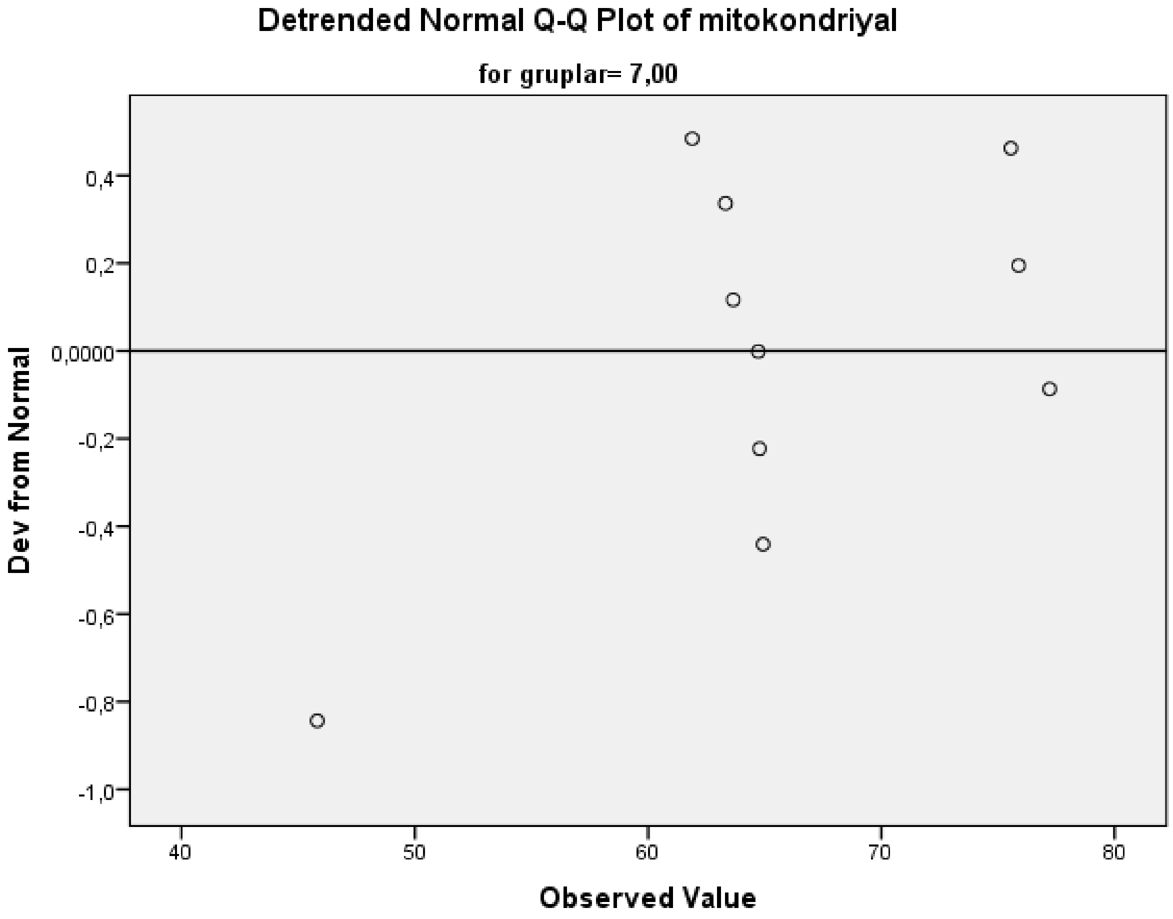

Detrended Normal Q-Q Plot of mitokondriyal
for gruplar= 7,00
Dev from Normal
0,4
0,2
0,0000
-0,2
-0,4
-0,6
-0,8
-1,0
40
50
60
70
80
Observed Value

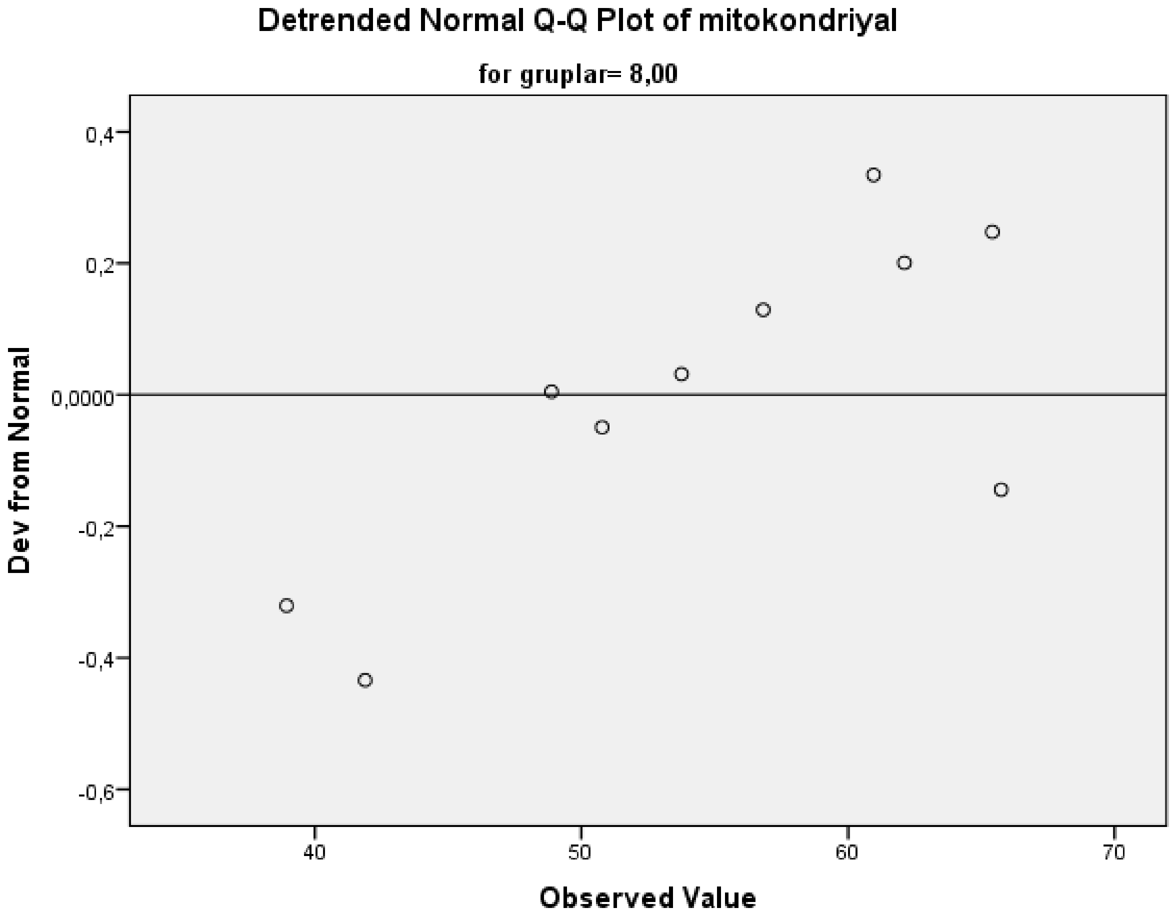
Detrended Normal Q-Q Plot of mitokondriyal
for gruplar= 8,00
Dev from Normal
0,4
0,2
0,0000
-0,2
-0,4
-0,6
40
50
60
70
Observed Value

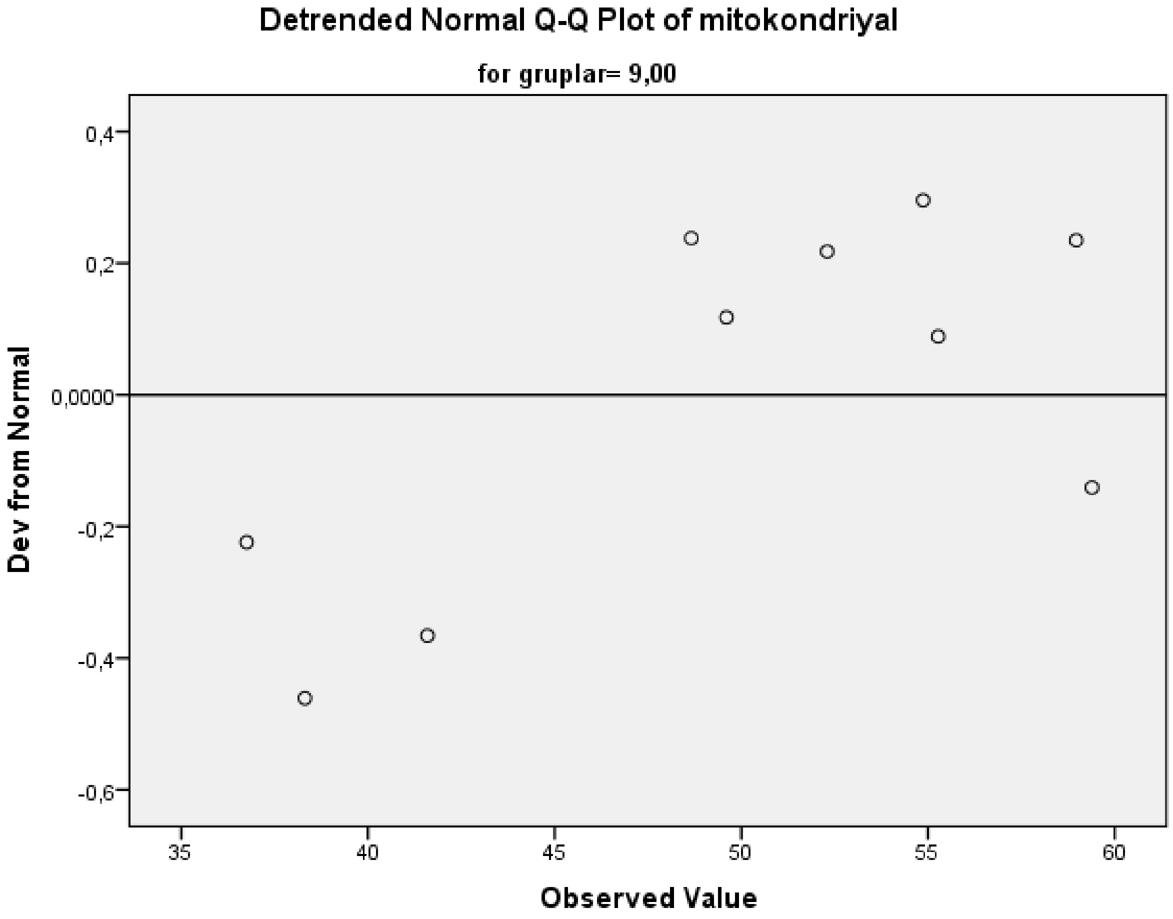
Detrended Normal Q-Q Plot of mitokondriyal
for gruplar= 9,00
Dev from Normal
0,4
0,2
0,0000
-0,2
-0,4
-0,6
35
40
45
50
55
60
Observed Value

Detrended Normal Q-Q Plot of mitokondriyal

for gruplar= 10,00

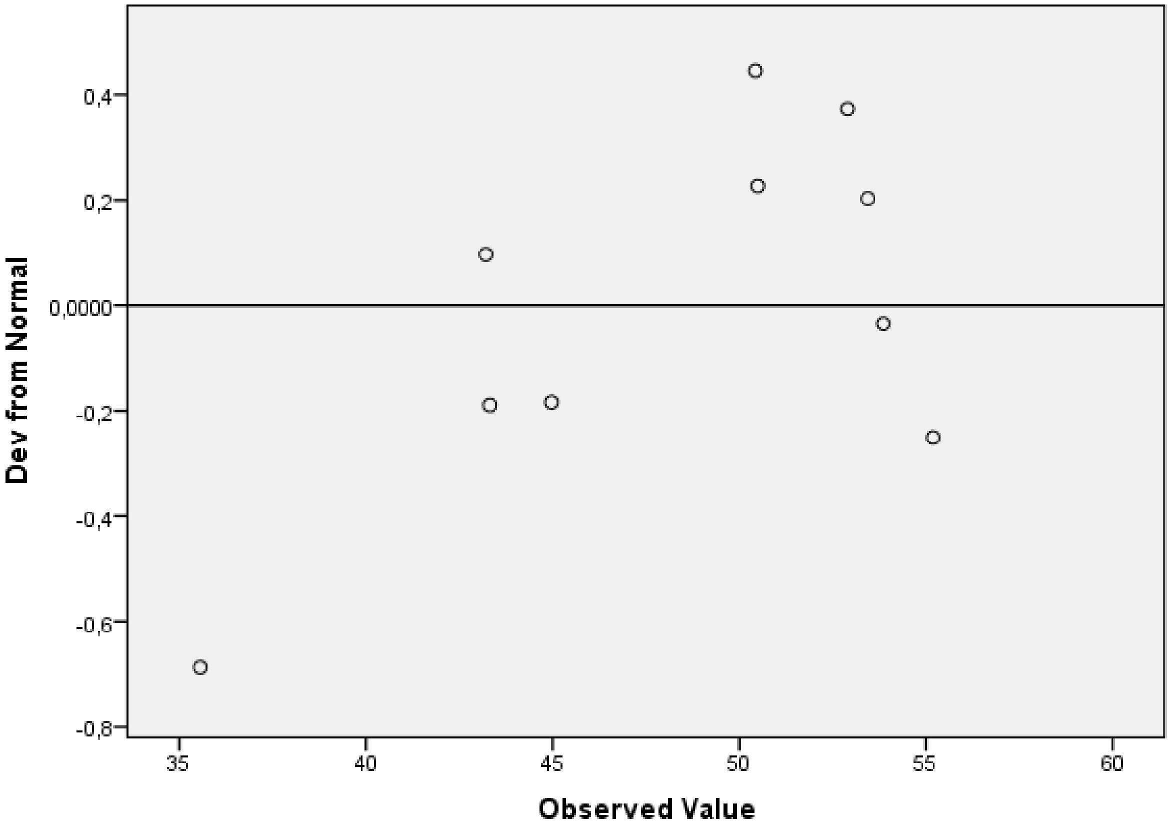

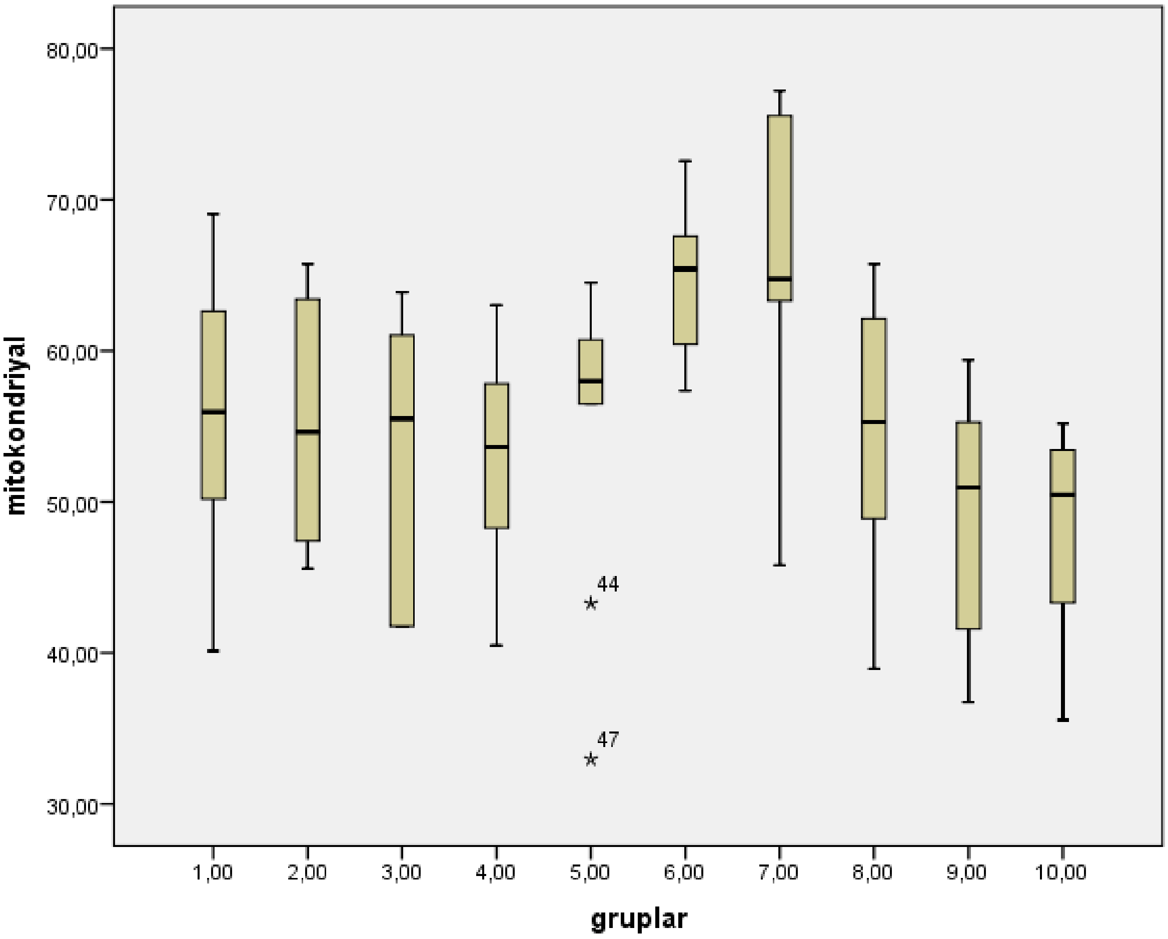


```
ONEWAY mitokondriyal BY gruplar
  /STATISTICS DESCRIPTIVES HOMOGENEITY
  /PLOT MEANS
  /MISSING ANALYSIS
  /POSTHOC=DUNCAN ALPHA(0.05).
```

Oneway

Notes

Output Created		23-FEB-2021 19:55:26
Comments		
Input	Active Dataset	DataSet0
	Filter	<none>
	Weight	<none>

	Split File	<none>
	N of Rows in Working Data File	110
Missing Value Handling	Definition of Missing	User-defined missing values are treated as missing.
	Cases Used	Statistics for each analysis are based on cases with no missing data for any variable in the analysis.
Syntax		ONEWAY mitokondriyal BY gruplar<br>/STATISTICS DESCRIPTIVES HOMOGENEITY<br>/PLOT MEANS<br>/MISSING ANALYSIS<br>/POSTHOC=DUNCAN ALPHA(0.05).
Resources	Processor Time	00:00:00,25
	Elapsed Time	00:00:00,30

Descriptives

mitokondriyal

	N	Mean	Std. Deviation	Std. Error	95% Confidence Interval for Mean	
					Lower Bound	Upper Bound
1,00	10	55,9200	8,63824	2,73165	49,7406	62,0994
2,00	10	55,2240	8,32743	2,63336	49,2669	61,1811
3,00	10	53,1010	9,14221	2,89102	46,5611	59,6409
4,00	10	52,8980	7,05306	2,23037	47,8525	57,9435
5,00	10	55,4440	9,82441	3,10675	48,4160	62,4720
6,00	10	64,7610	4,79996	1,51788	61,3273	68,1947
7,00	10	65,7750	9,15955	2,89650	59,2227	72,3273
8,00	10	54,5280	9,41332	2,97675	47,7941	61,2619
9,00	10	49,5710	8,22243	2,60016	43,6890	55,4530
10,00	10	48,3370	6,31919	1,99830	43,8165	52,8575
Total	100	55,5559	9,53837	,95384	53,6633	57,4485

Descriptives

mitokondriyal

	Minimum	Maximum
1,00	40,13	69,05
2,00	45,57	65,73
3,00	41,73	63,86

4,00	40,49	63,01
5,00	32,96	64,50
6,00	57,35	72,57
7,00	45,82	77,21
8,00	38,94	65,74
9,00	36,75	59,39
10,00	35,56	55,19
Total	32,96	77,21

Test of Homogeneity of Variances

mitokondriyal

Levene Statistic	df1	df2	Sig.
,878	9	90	,548

ANOVA

mitokondriyal

	Sum of Squares	df	Mean Square	F	Sig.
Between Groups	2914,982	9	323,887	4,785	,000
Within Groups	6092,081	90	67,690		
Total	9007,063	99			

Post Hoc Tests

Homogeneous Subsets

mitokondriyal

Duncan[a]

gruplar	N	Subset for alpha = 0.05	
		1	2
10,00	10	48,3370	

9,00	10	49,5710	
4,00	10	52,8980	
3,00	10	53,1010	
8,00	10	54,5280	
2,00	10	55,2240	
5,00	10	55,4440	
1,00	10	55,9200	
6,00	10		64,7610
7,00	10		65,7750
Sig.		,081	,783

Means for groups in homogeneous subsets are displayed.
a. Uses Harmonic Mean Sample Size = 10,000.

Means Plots

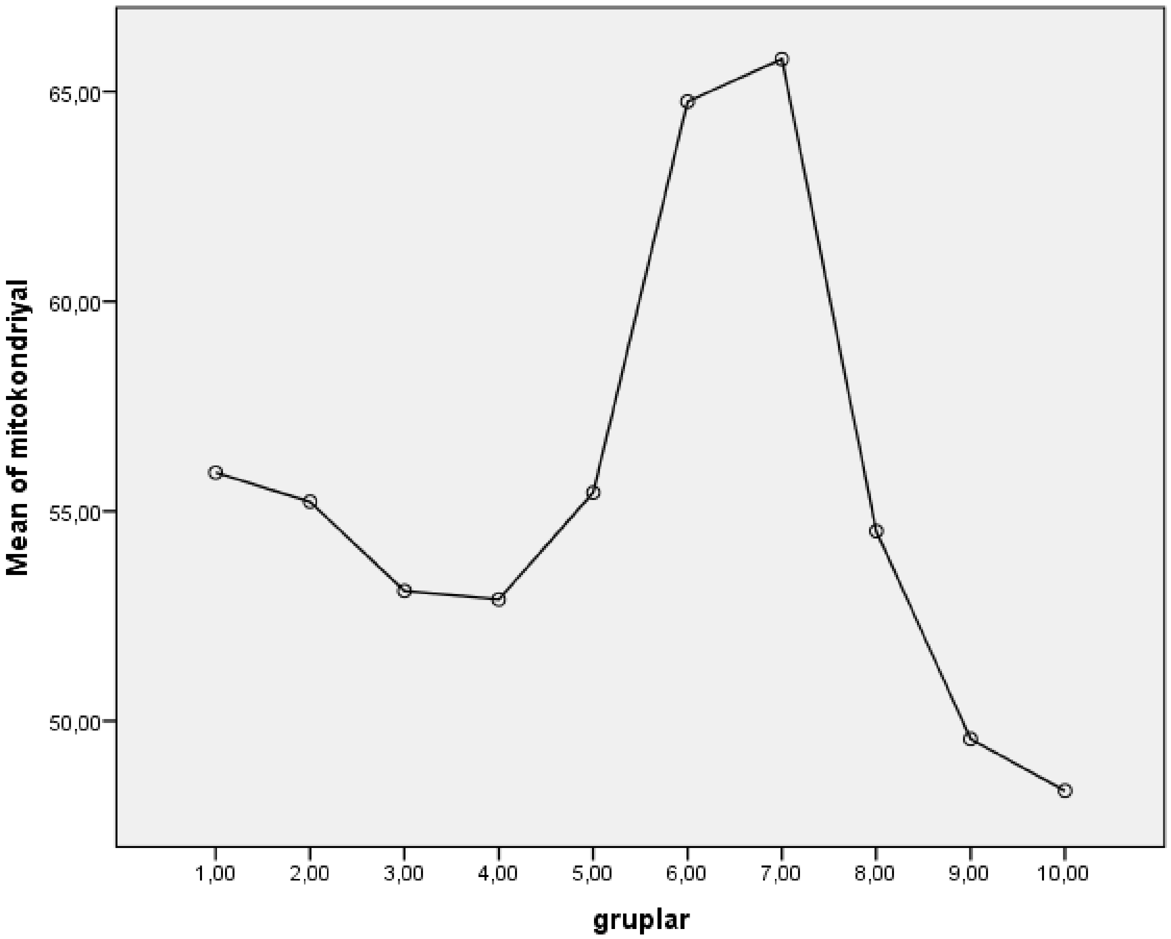

Oxidative stress- ANOVA

OksidatifS: Oxidative stress
Groups
1: Control
2: RES10
3: RES20
4: RES40
5: CD10
6: CD20
7: CD40
8: RLC10
9: RLC20
10: RLC40

```
EXAMINE VARIABLES=OksidatifS BY Gruplar
  /PLOT BOXPLOT STEMLEAF HISTOGRAM NPPLOT
  /COMPARE GROUPS
  /STATISTICS DESCRIPTIVES
  /CINTERVAL 95
  /MISSING LISTWISE
  /NOTOTAL.
```

Explore

Notes

Output Created		24-DEC-2020 12:42:42
Comments		
Input	Active Dataset	DataSet0
	Filter	<none>
	Weight	<none>
	Split File	<none>
	N of Rows in Working Data File	100
Missing Value Handling	Definition of Missing	User-defined missing values for dependent variables are treated as missing.
	Cases Used	Statistics are based on cases with no missing values for any dependent variable or factor used.

Syntax		EXAMINE VARIABLES=OksidatifS BY Gruplar<br>/PLOT BOXPLOT STEMLEAF HISTOGRAM NPPLOT<br>/COMPARE GROUPS<br>/STATISTICS DESCRIPTIVES<br>/CINTERVAL 95<br>/MISSING LISTWISE<br>/NOTOTAL.
Resources	Processor Time	00:00:04,42
	Elapsed Time	00:00:04,40

Gruplar

Case Processing Summary

		Cases					
		Valid		Missing		Total	
	Gruplar	N	Percent	N	Percent	N	Percent
OksidatifS	1,00	10	100,0%	0	0,0%	10	100,0%
	2,00	10	100,0%	0	0,0%	10	100,0%
	3,00	10	100,0%	0	0,0%	10	100,0%
	4,00	10	100,0%	0	0,0%	10	100,0%
	5,00	10	100,0%	0	0,0%	10	100,0%
	6,00	10	100,0%	0	0,0%	10	100,0%
	7,00	10	100,0%	0	0,0%	10	100,0%
	8,00	10	100,0%	0	0,0%	10	100,0%
	9,00	10	100,0%	0	0,0%	10	100,0%
	10,00	10	100,0%	0	0,0%	10	100,0%

Descriptives

	Gruplar			Statistic	Std. Error
OksidatifS	1,00	Mean		38,8820	1,63076
		95% Confidence Interval for Mean	Lower Bound	35,1930	
			Upper Bound	42,5710	

	5% Trimmed Mean		38,8622	
	Median		40,2550	
	Variance		26,594	
	Std. Deviation		5,15692	
	Minimum		31,66	
	Maximum		46,46	
	Range		14,80	
	Interquartile Range		9,36	
	Skewness		-,165	,687
	Kurtosis		-1,508	1,334
2,00	Mean		37,6330	1,58982
	95% Confidence Interval for Mean	Lower Bound	34,0366	
		Upper Bound	41,2294	
	5% Trimmed Mean		37,5600	
	Median		38,0500	
	Variance		25,275	
	Std. Deviation		5,02746	
	Minimum		31,13	
	Maximum		45,45	
	Range		14,32	
	Interquartile Range		8,78	
	Skewness		,031	,687
	Kurtosis		-1,499	1,334
3,00	Mean		37,6640	1,95446
	95% Confidence Interval for Mean	Lower Bound	33,2427	
		Upper Bound	42,0853	
	5% Trimmed Mean		37,7717	
	Median		39,9650	
	Variance		38,199	
	Std. Deviation		6,18055	
	Minimum		28,04	
	Maximum		45,35	
	Range		17,31	
	Interquartile Range		10,76	
	Skewness		-,344	,687
	Kurtosis		-1,682	1,334
4,00	Mean		39,4960	1,98381
	95% Confidence Interval for	Lower Bound	35,0083	

	Mean	Upper Bound	43,9837	
	5% Trimmed Mean		39,6906	
	Median		40,6800	
	Variance		39,355	
	Std. Deviation		6,27337	
	Minimum		29,06	
	Maximum		46,43	
	Range		17,37	
	Interquartile Range		11,34	
	Skewness		-,470	,687
	Kurtosis		-1,206	1,334
5,00	Mean		39,1750	1,47091
	95% Confidence Interval for Mean	Lower Bound	35,8476	
		Upper Bound	42,5024	
	5% Trimmed Mean		39,1850	
	Median		39,7350	
	Variance		21,636	
	Std. Deviation		4,65142	
	Minimum		32,73	
	Maximum		45,44	
	Range		12,71	
	Interquartile Range		8,59	
	Skewness		-,181	,687
	Kurtosis		-1,594	1,334
6,00	Mean		38,9160	1,35959
	95% Confidence Interval for Mean	Lower Bound	35,8404	
		Upper Bound	41,9916	
	5% Trimmed Mean		39,1556	
	Median		41,6500	
	Variance		18,485	
	Std. Deviation		4,29939	
	Minimum		30,84	
	Maximum		42,68	
	Range		11,84	
	Interquartile Range		6,37	
	Skewness		-,967	,687
	Kurtosis		-,544	1,334
7,00	Mean		38,7950	1,27951

	95% Confidence Interval for Mean	Lower Bound	35,9006	
		Upper Bound	41,6894	
	5% Trimmed Mean		38,8256	
	Median		40,3800	
	Variance		16,371	
	Std. Deviation		4,04615	
	Minimum		33,04	
	Maximum		44,00	
	Range		10,96	
	Interquartile Range		7,60	
	Skewness		-,349	,687
	Kurtosis		-1,589	1,334
8,00	Mean		36,9620	2,30147
	95% Confidence Interval for Mean	Lower Bound	31,7557	
		Upper Bound	42,1683	
	5% Trimmed Mean		37,1867	
	Median		37,9550	
	Variance		52,968	
	Std. Deviation		7,27789	
	Minimum		25,27	
	Maximum		44,61	
	Range		19,34	
	Interquartile Range		12,57	
	Skewness		-,396	,687
	Kurtosis		-1,561	1,334
9,00	Mean		37,1800	1,71314
	95% Confidence Interval for Mean	Lower Bound	33,3046	
		Upper Bound	41,0554	
	5% Trimmed Mean		37,3217	
	Median		38,1500	
	Variance		29,349	
	Std. Deviation		5,41744	
	Minimum		28,11	
	Maximum		43,70	
	Range		15,59	
	Interquartile Range		9,74	
	Skewness		-,344	,687
	Kurtosis		-1,224	1,334

				Statistic	Std. Error
	10,00	Mean		37,7050	1,67757
		95% Confidence Interval for Mean	Lower Bound	33,9101	
			Upper Bound	41,4999	
		5% Trimmed Mean		37,6400	
		Median		37,6000	
		Variance		28,142	
		Std. Deviation		5,30493	
		Minimum		29,27	
		Maximum		47,31	
		Range		18,04	
		Interquartile Range		7,00	
		Skewness		,248	,687
		Kurtosis		-,114	1,334

Tests of Normality

		Kolmogorov-Smirnov[a]			Shapiro-Wilk		
	Gruplar	Statistic	df	Sig.	Statistic	df	Sig.
OksidatifS	1,00	,237	10	,118	,912	10	,296
	2,00	,211	10	,200*	,915	10	,319
	3,00	,260	10	,054	,886	10	,152
	4,00	,167	10	,200*	,916	10	,321
	5,00	,160	10	,200*	,919	10	,351
	6,00	,332	10	,003	,803	10	,016
	7,00	,236	10	,122	,899	10	,212
	8,00	,253	10	,070	,867	10	,092
	9,00	,151	10	,200*	,933	10	,483
	10,00	,151	10	,200*	,980	10	,965

*. This is a lower bound of the true significance.

a. Lilliefors Significance Correction

OksidatifS

Histograms

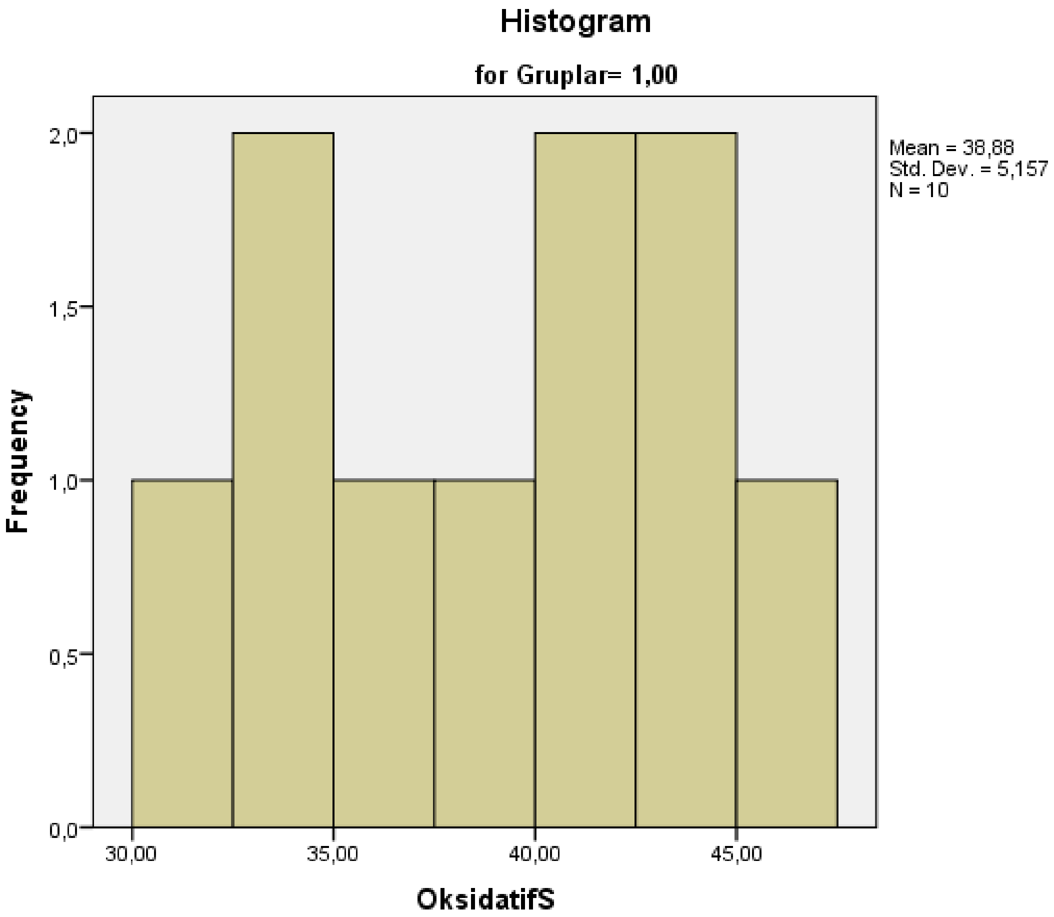

Histogram
for Gruplar= 1,00
Mean = 38,88
Std. Dev. = 5,157
N = 10
Frequency
2,0
1,5
1,0
0,5
0,0
30,00
35,00
40,00
45,00
OksidatifS

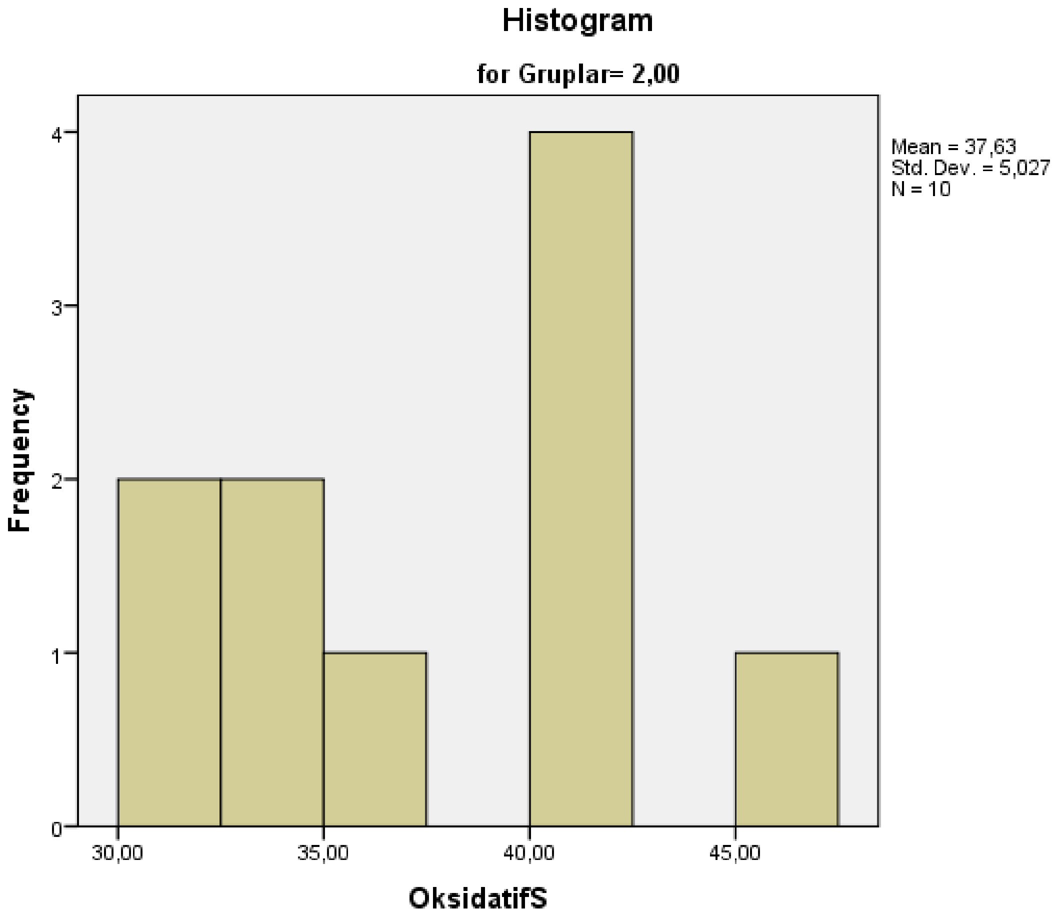
Histogram
for Gruplar= 2,00
Mean = 37,63
Std. Dev. = 5,027
N = 10
Frequency
0
1
2
3
4
30,00
35,00
40,00
45,00
OksidatifS

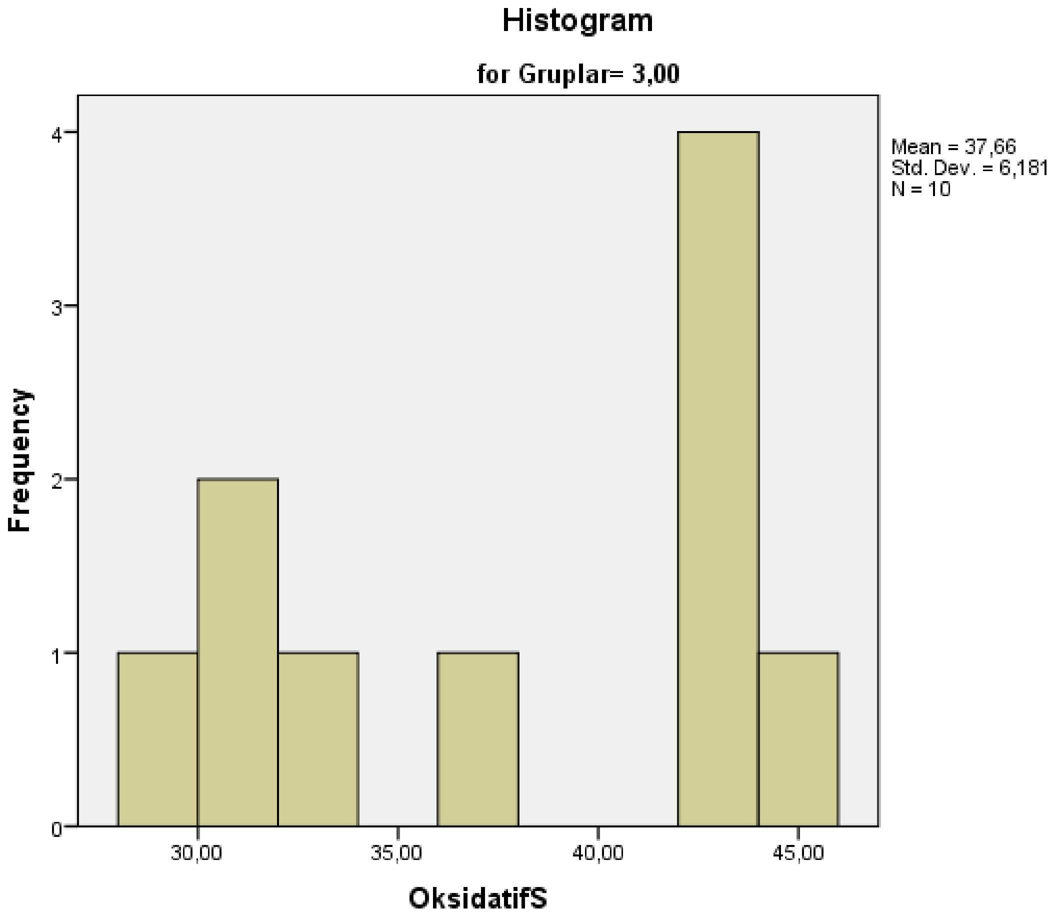

Histogram
for Gruplar= 3,00
Mean = 37,66
Std. Dev. = 6,181
N = 10
Frequency
0
1
2
3
4
30,00
35,00
40,00
45,00
OksidatifS

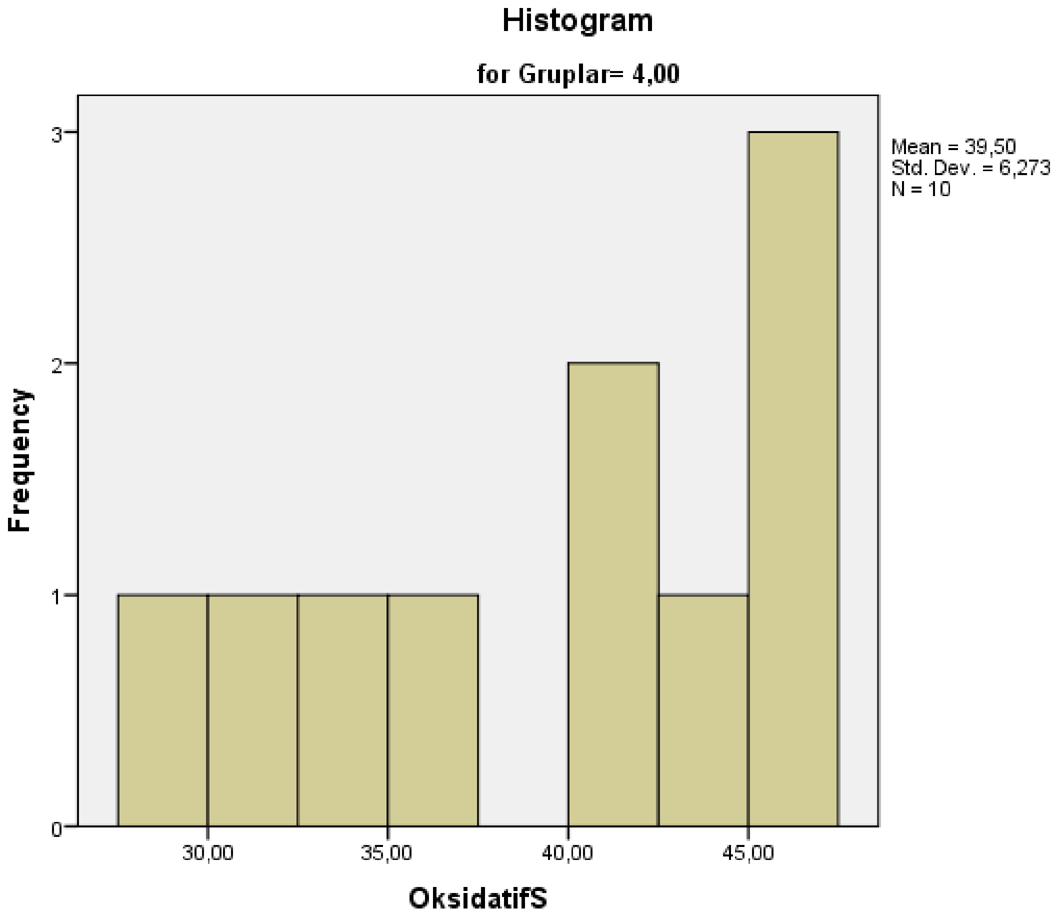
Histogram
for Gruplar= 4,00
Mean = 39,50
Std. Dev. = 6,273
N = 10
Frequency
0
1
2
3
30,00
35,00
40,00
45,00
OksidatifS

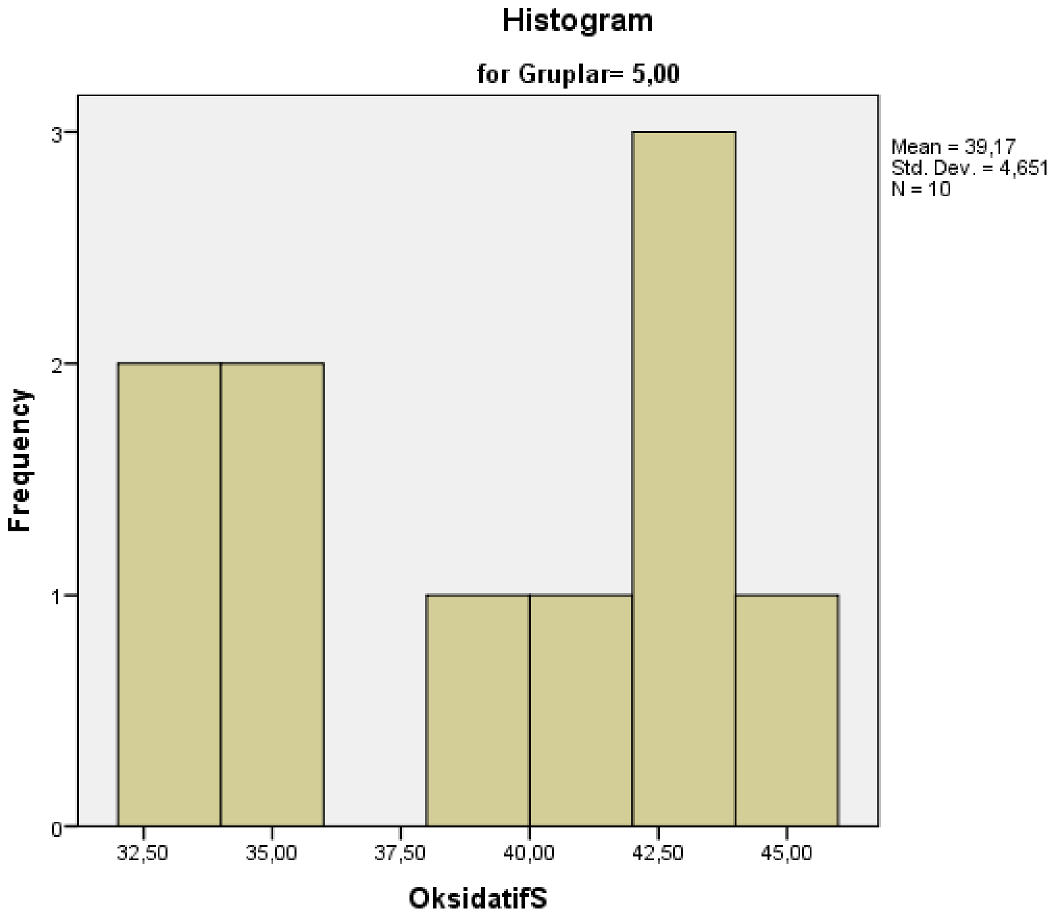
Histogram
for Gruplar= 5,00
Mean = 39,17
Std. Dev. = 4,651
N = 10
Frequency
0
1
2
3
32,50
35,00
37,50
40,00
42,50
45,00
OksidatifS

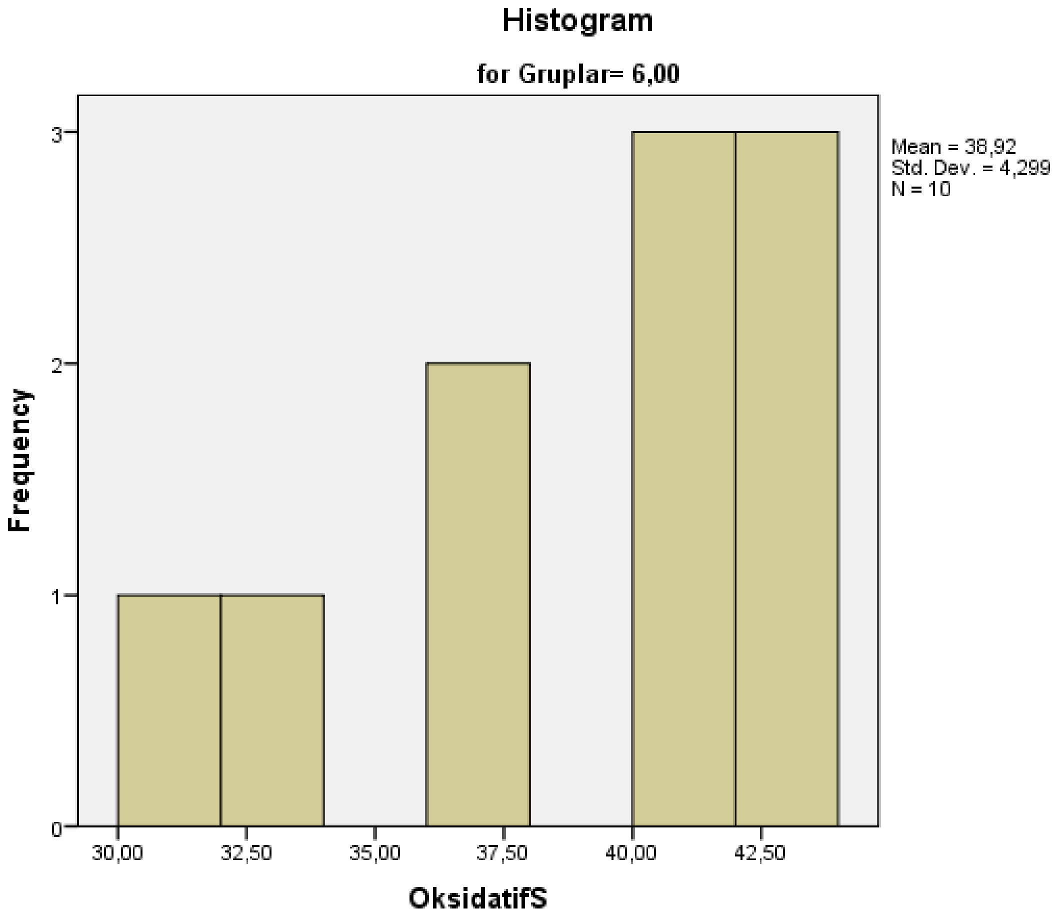

Histogram
for Gruplar= 6,00
Mean = 38,92
Std. Dev. = 4,299
N = 10
Frequency
0
1
2
3
30,00
32,50
35,00
37,50
40,00
42,50
OksidatifS

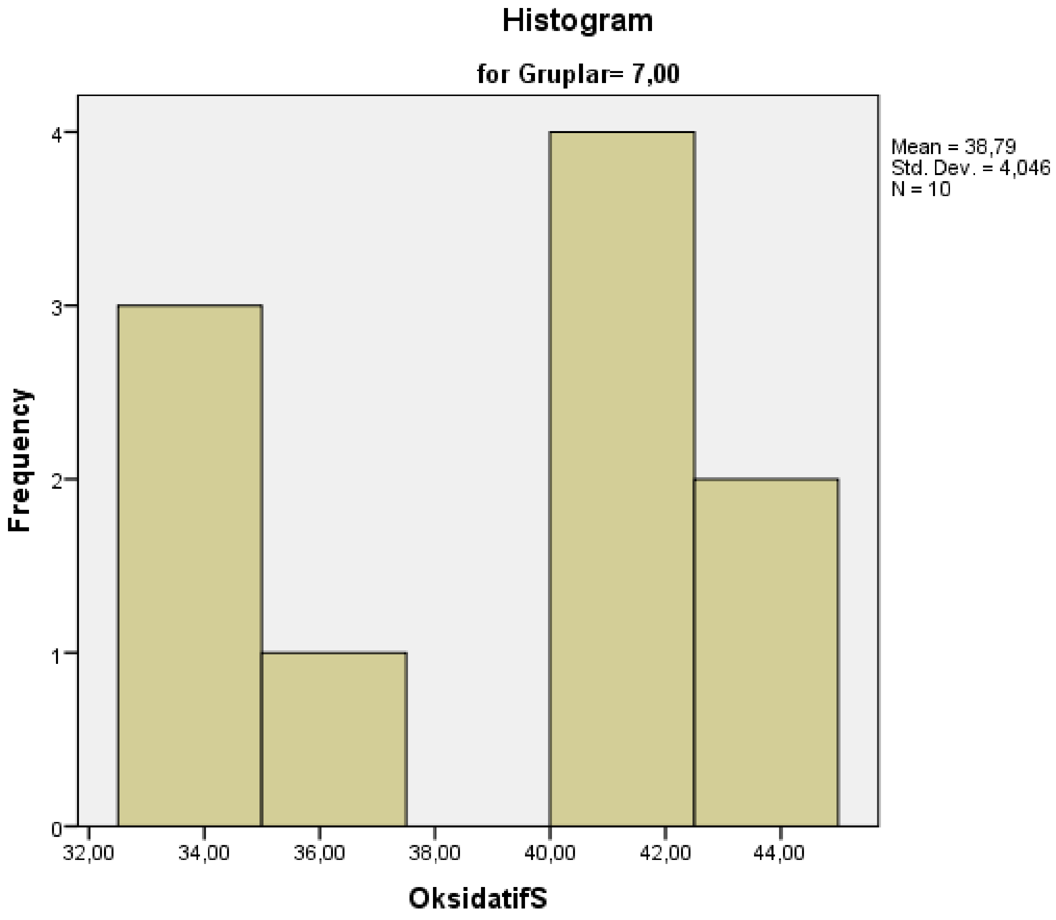
Histogram
for Gruplar= 7,00
Mean = 38,79
Std. Dev. = 4,046
N = 10
Frequency
0
1
2
3
4
32,00
34,00
36,00
38,00
40,00
42,00
44,00
OksidatifS

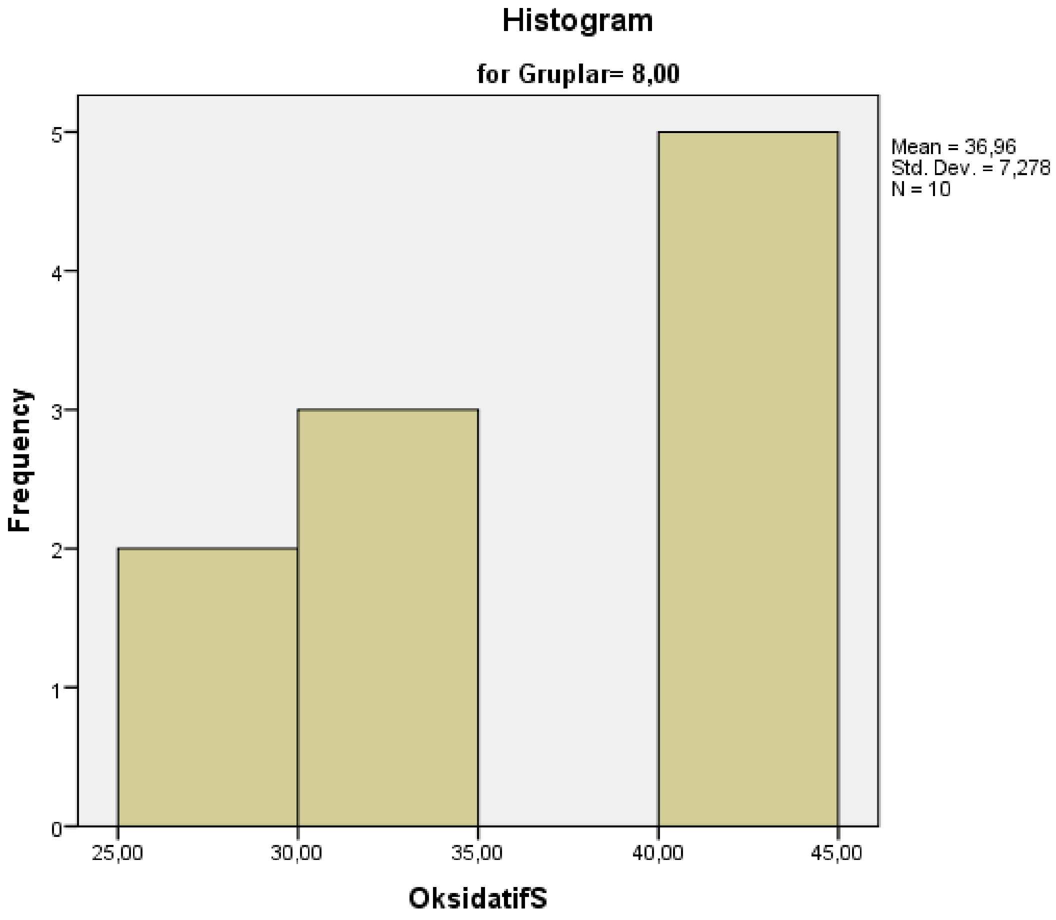
Histogram
for Gruplar= 8,00
Mean = 36,96
Std. Dev. = 7,278
N = 10
Frequency
0
1
2
3
4
5
25,00
30,00
35,00
40,00
45,00
OksidatifS

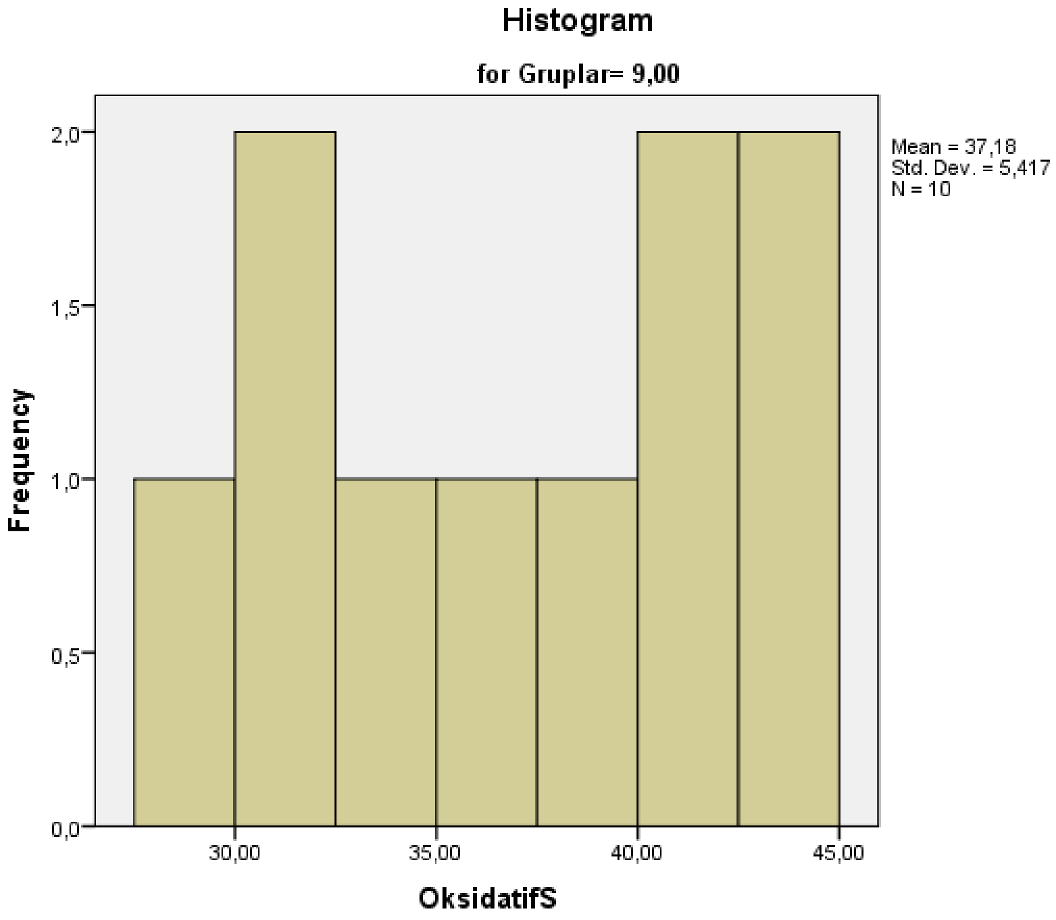
Histogram
for Gruplar= 9,00
Mean = 37,18
Std. Dev. = 5,417
N = 10
Frequency
2,0
1,5
1,0
0,5
0,0
30,00
35,00
40,00
45,00
OksidatifS

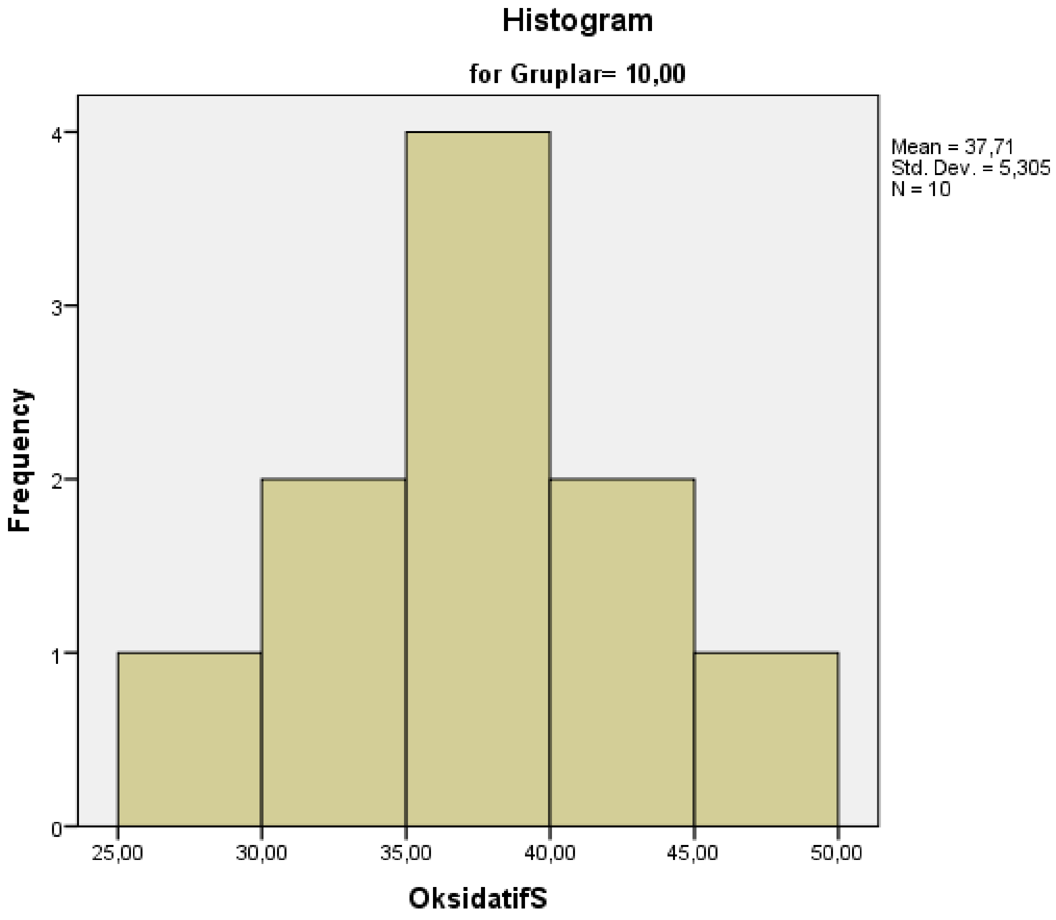

Stem-and-Leaf Plots

```
OksidatifS Stem-and-Leaf Plot for
Gruplar= 1,00

 Frequency    Stem &  Leaf

     3,00        3 .  123
     2,00        3 .  68
     4,00        4 .  2223
     1,00        4 .  6

 Stem width:  10,00
 Each leaf:       1 case(s)
```

OksidatifS Stem-and-Leaf Plot for
Gruplar= 2,00

```
 Frequency    Stem &  Leaf

     4,00        3 .  1134
     1,00        3 .  5
     4,00        4 .  0111
     1,00        4 .  5

 Stem width:  10,00
 Each leaf:       1 case(s)
```

OksidatifS Stem-and-Leaf Plot for
Gruplar= 3,00

```
 Frequency    Stem &  Leaf

     1,00        2 .  8
     3,00        3 .  112
     1,00        3 .  7
     4,00        4 .  2223
     1,00        4 .  5

 Stem width:  10,00
 Each leaf:       1 case(s)
```

OksidatifS Stem-and-Leaf Plot for
Gruplar= 4,00

```
 Frequency    Stem &  Leaf

     1,00        2 .  9
     2,00        3 .  14
     1,00        3 .  6
     3,00        4 .  014
     3,00        4 .  566

 Stem width:  10,00
 Each leaf:       1 case(s)
```

OksidatifS Stem-and-Leaf Plot for
Gruplar= 5,00

```
 Frequency    Stem &  Leaf

     2,00        3 .  22
```

```
     3,00        3 .  558
     4,00        4 .  0233
     1,00        4 .  5

 Stem width:  10,00
 Each leaf:       1 case(s)
```

```
OksidatifS Stem-and-Leaf Plot for
Gruplar= 6,00

 Frequency    Stem &  Leaf

     2,00        3 .  03
     2,00        3 .  66
     6,00        4 .  111222

 Stem width:  10,00
 Each leaf:       1 case(s)
```

```
OksidatifS Stem-and-Leaf Plot for
Gruplar= 7,00

 Frequency    Stem &  Leaf

     3,00        3 .  334
     1,00        3 .  6
     6,00        4 .  000134

 Stem width:  10,00
 Each leaf:       1 case(s)
```

```
OksidatifS Stem-and-Leaf Plot for
Gruplar= 8,00

 Frequency    Stem &  Leaf

     2,00        2 .  57
     3,00        3 .  133
      ,00        3 .
     5,00        4 .  13334

 Stem width:  10,00
 Each leaf:       1 case(s)
```

```
OksidatifS Stem-and-Leaf Plot for
Gruplar= 9,00

 Frequency    Stem &  Leaf

     1,00        2 .  8
     3,00        3 .  123
     2,00        3 .  79
     4,00        4 .  0133

 Stem width:  10,00
 Each leaf:       1 case(s)
```

```
OksidatifS Stem-and-Leaf Plot for
Gruplar= 10,00

 Frequency    Stem &  Leaf

     1,00        2 .  9
     2,00        3 .  24
     4,00        3 .  5599
     2,00        4 .  02
     1,00        4 .  7

 Stem width:  10,00
 Each leaf:       1 case(s)
```

Normal Q-Q Plots

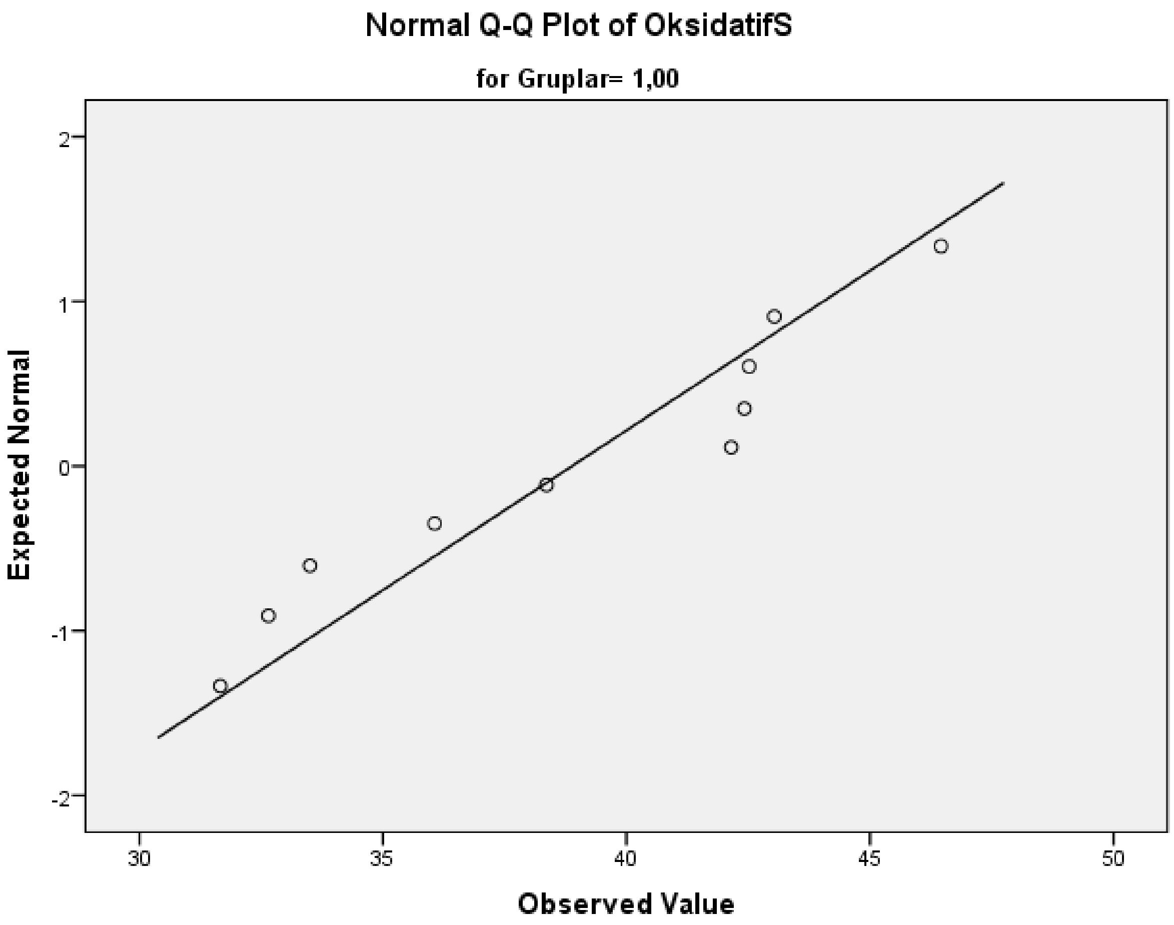
Normal Q-Q Plot of OksidatifS
for Gruplar= 1,00
Expected Normal
Observed Value
2
1
0
-1
-2
30
35
40
45
50

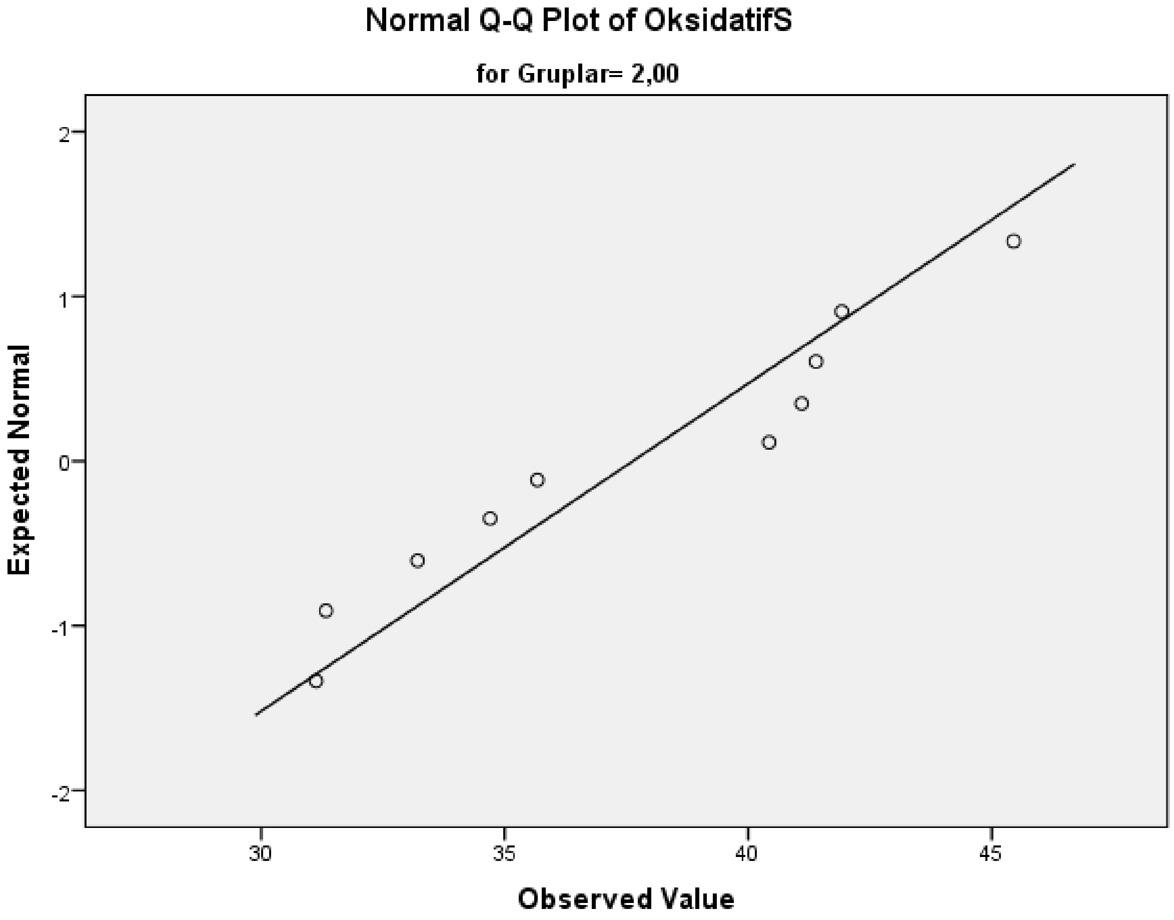

Normal Q-Q Plot of OksidatifS
for Gruplar= 2,00
Expected Normal
2
1
0
-1
-2
30
35
40
45
Observed Value

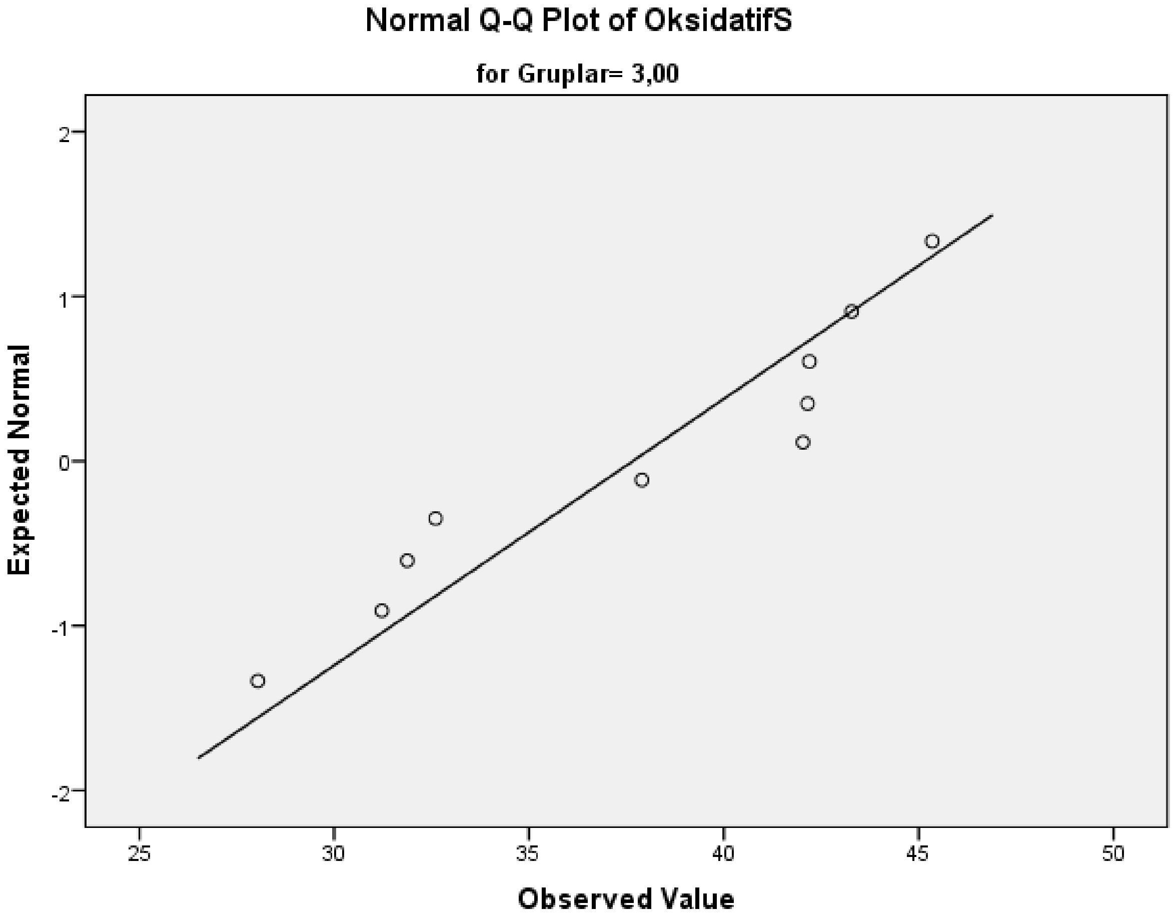
Normal Q-Q Plot of OksidatifS
for Gruplar= 3,00
Expected Normal
2
1
0
-1
-2
25
30
35
40
45
50
Observed Value

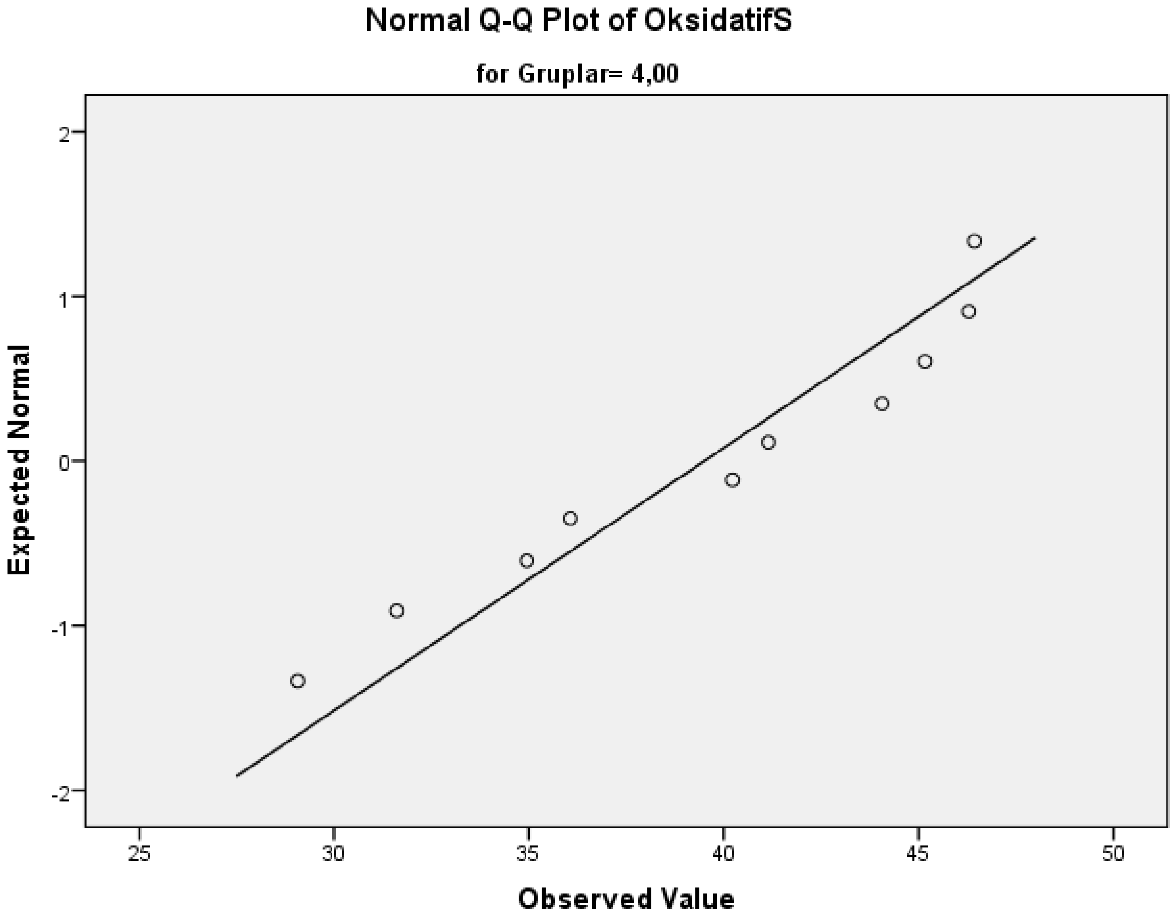
Normal Q-Q Plot of OksidatifS
for Gruplar= 4,00
Expected Normal
2
1
0
-1
-2
25
30
35
40
45
50
Observed Value

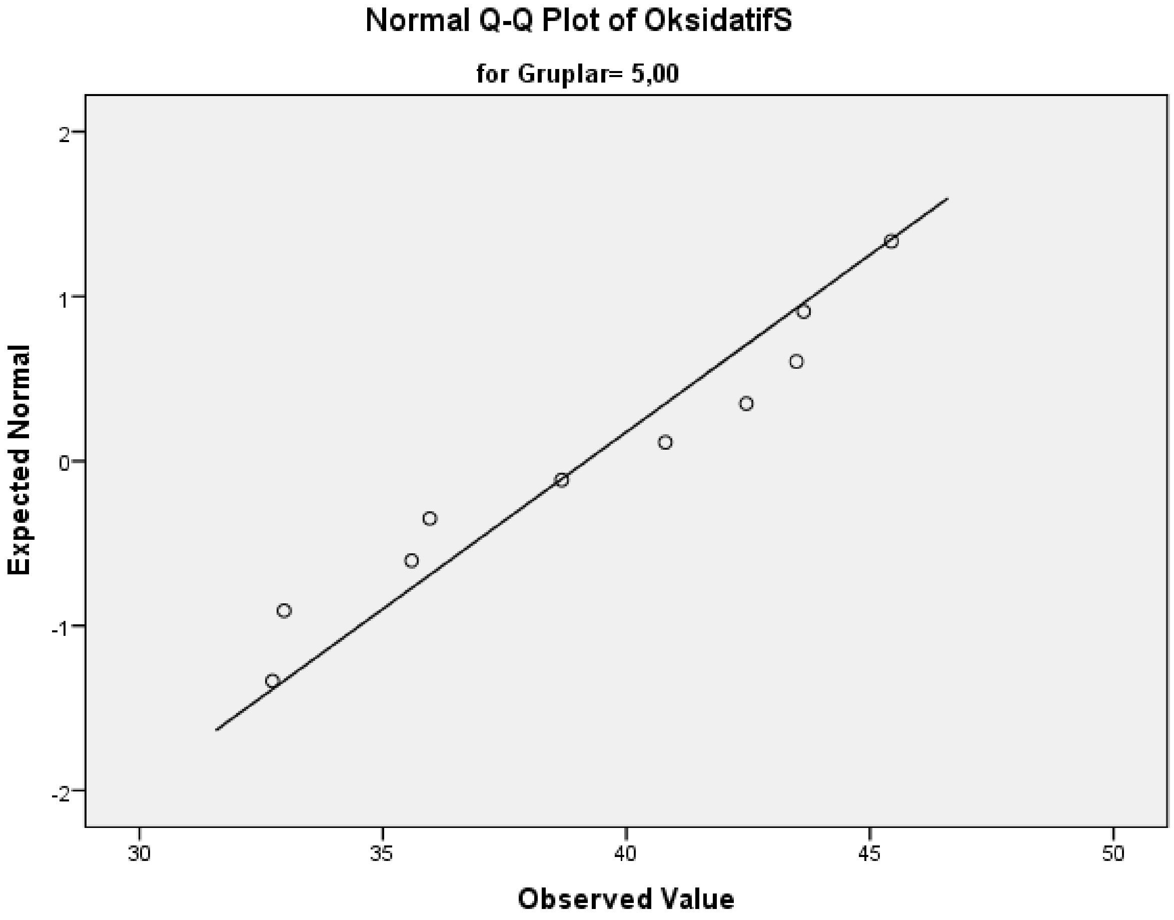
Normal Q-Q Plot of OksidatifS
for Gruplar= 5,00
Expected Normal
2
1
0
-1
-2
30
35
40
45
50
Observed Value

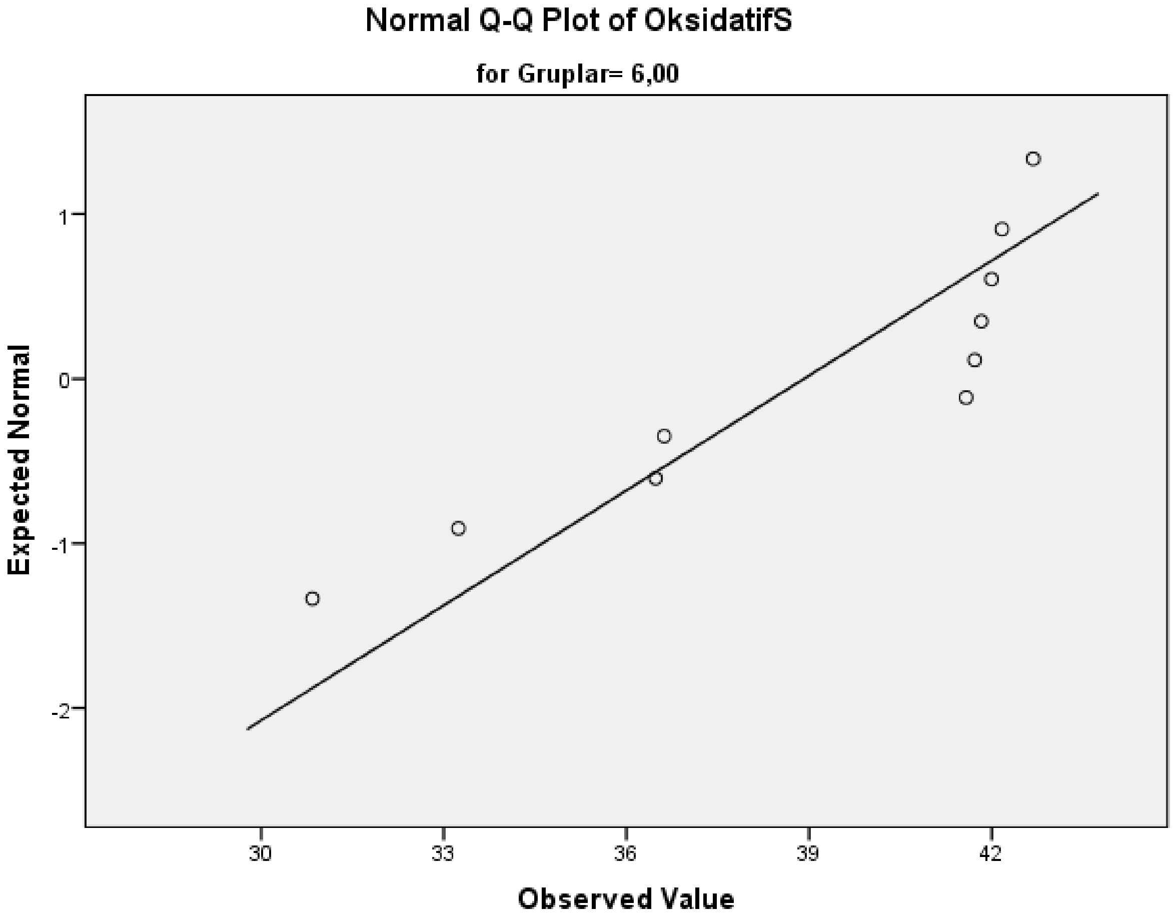
Normal Q-Q Plot of OksidatifS
for Gruplar= 6,00
Expected Normal
1
0
-1
-2
30
33
36
39
42
Observed Value

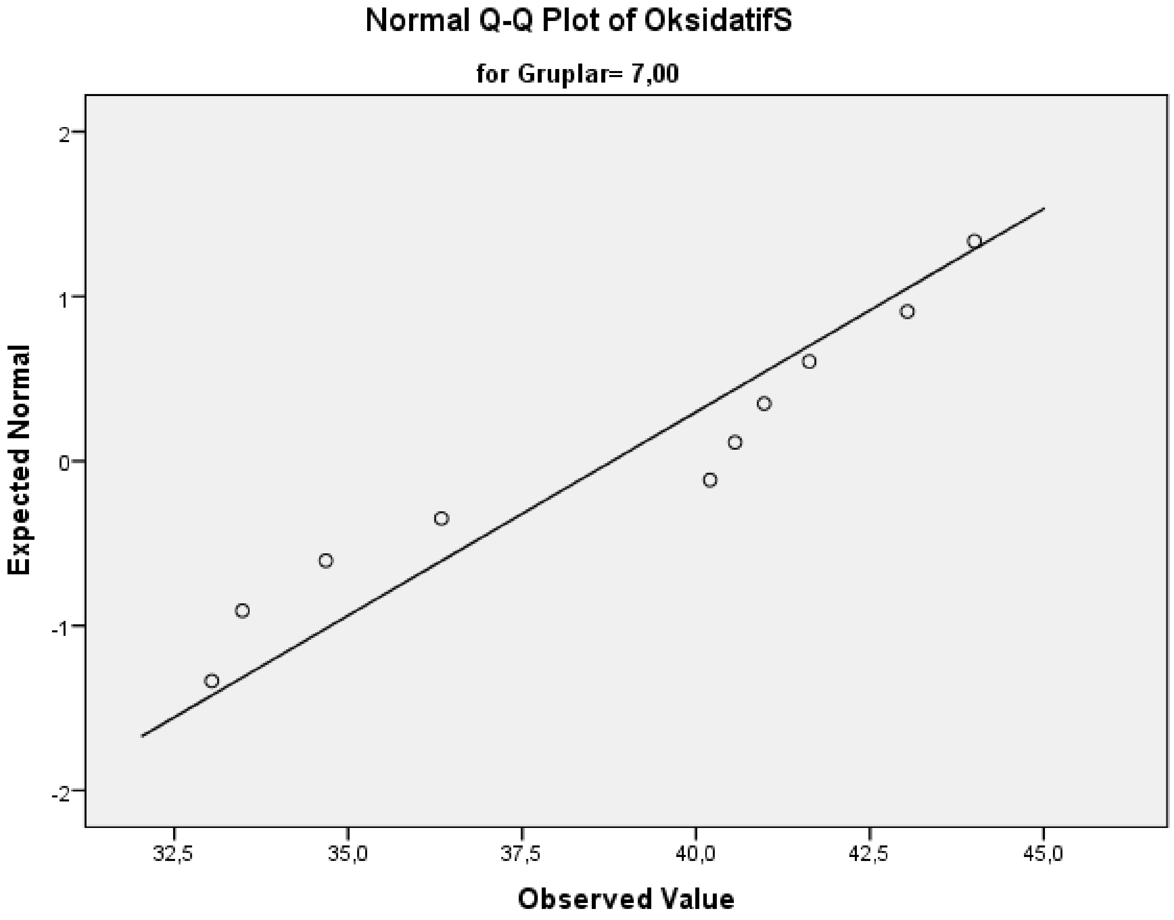
Normal Q-Q Plot of OksidatifS
for Gruplar= 7,00
Expected Normal
2
1
0
-1
-2
32,5
35,0
37,5
40,0
42,5
45,0
Observed Value

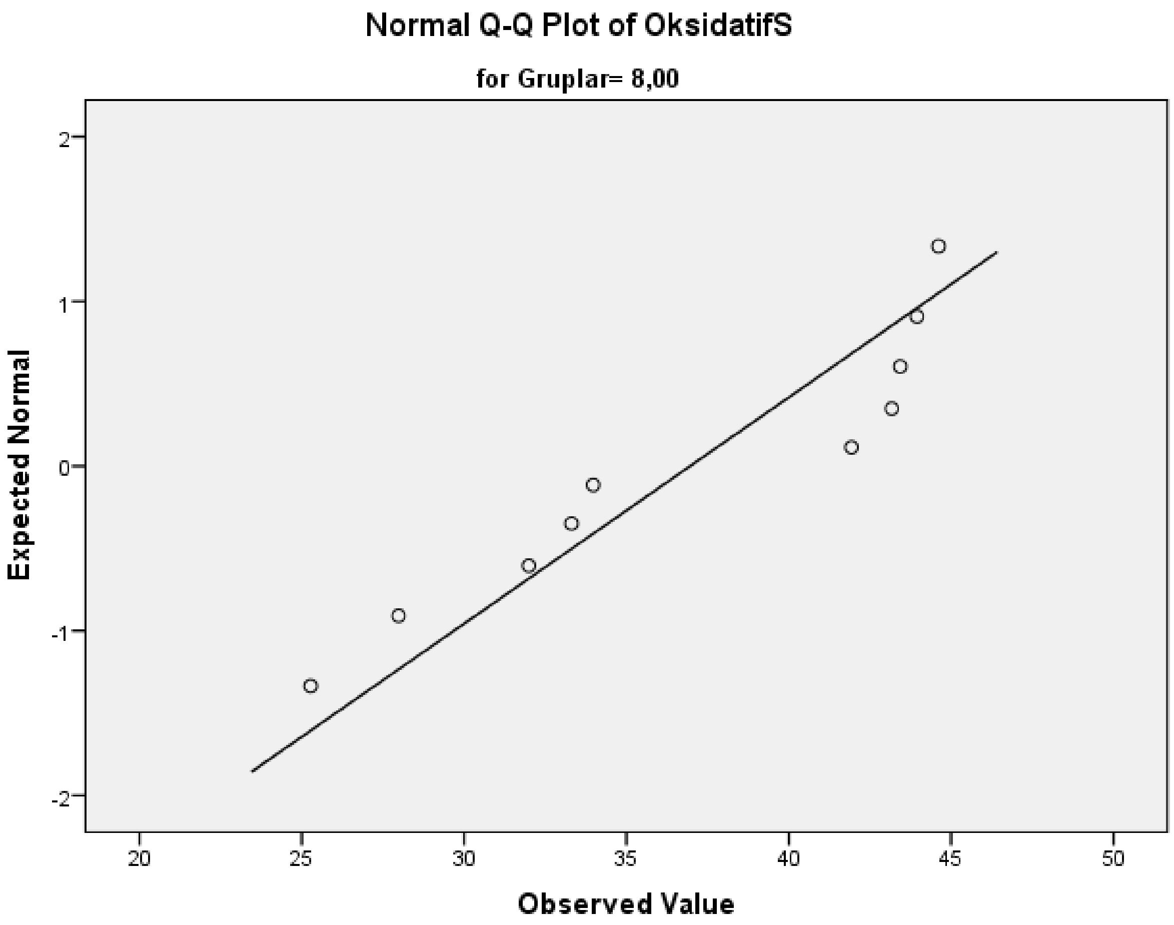

Normal Q-Q Plot of OksidatifS
for Gruplar= 8,00
Expected Normal
2
1
0
-1
-2
20
25
30
35
40
45
50
Observed Value

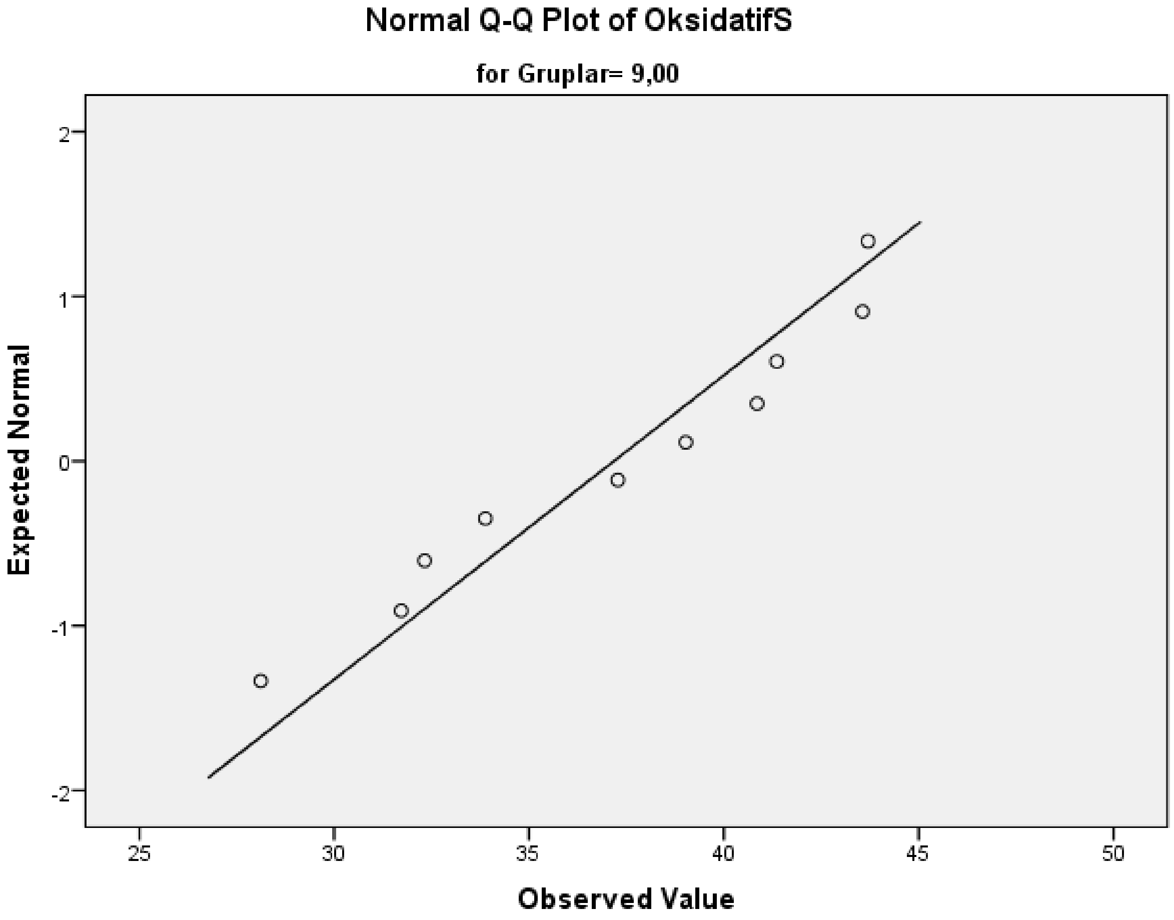
Normal Q-Q Plot of OksidatifS
for Gruplar= 9,00
Expected Normal
Observed Value
2
1
0
-1
-2
25
30
35
40
45
50

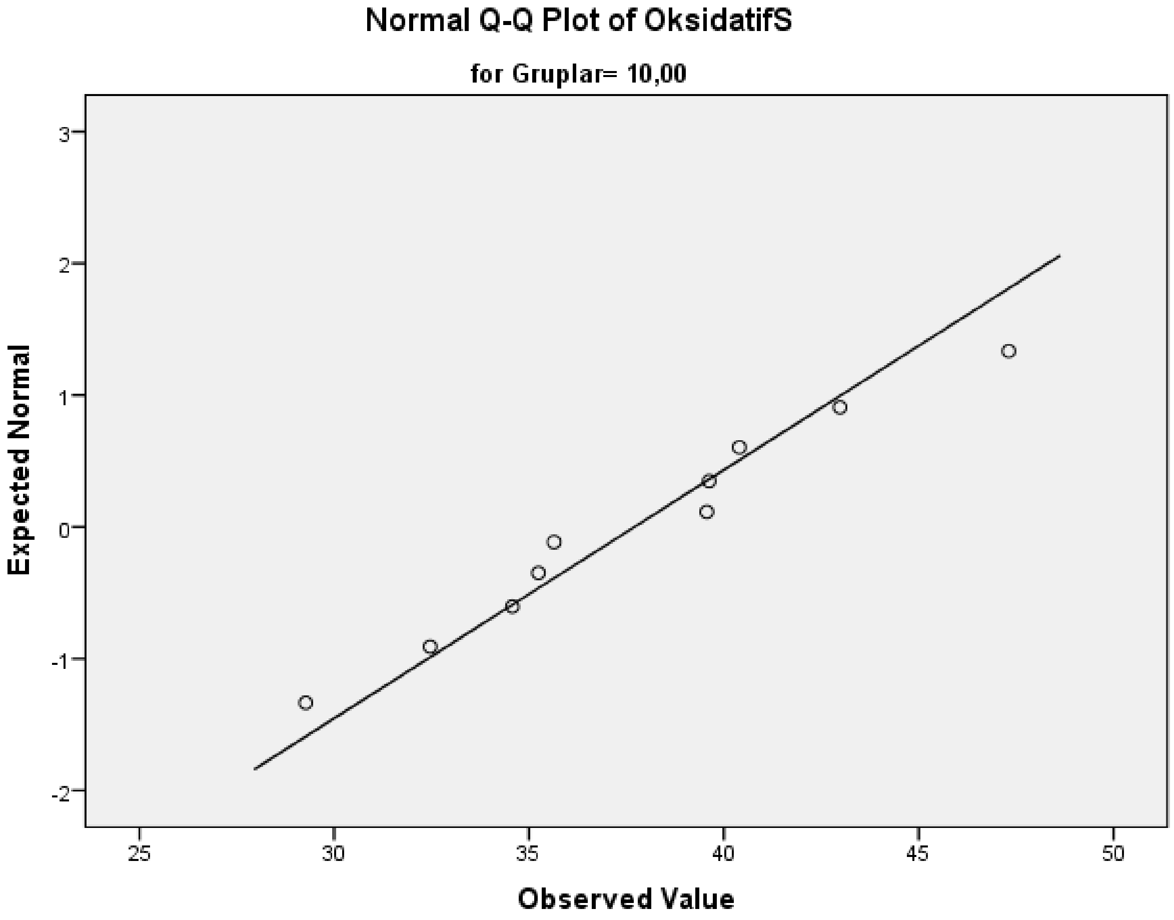

Detrended Normal Q-Q Plots

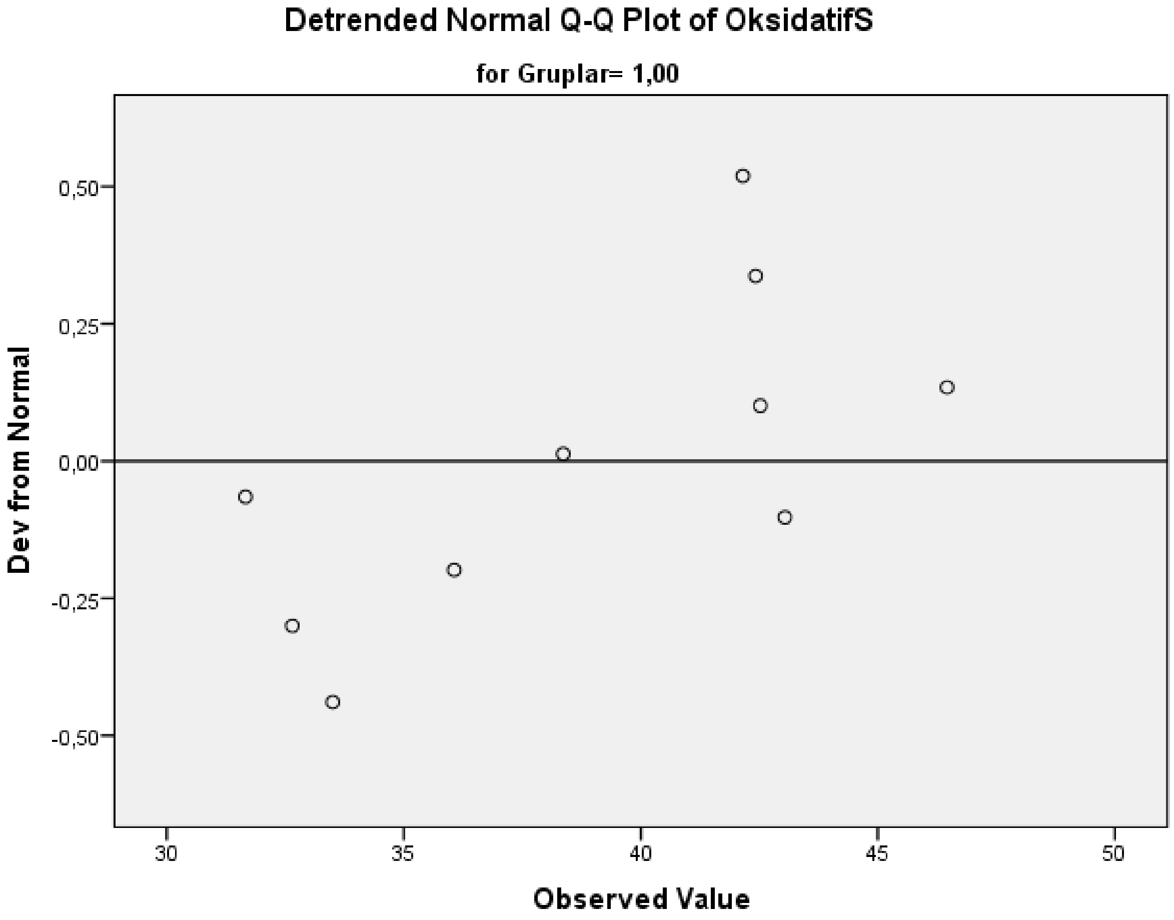
Detrended Normal Q-Q Plot of OksidatifS
for Gruplar= 1,00
Dev from Normal
0,50
0,25
0,00
-0,25
-0,50
30
35
40
45
50
Observed Value

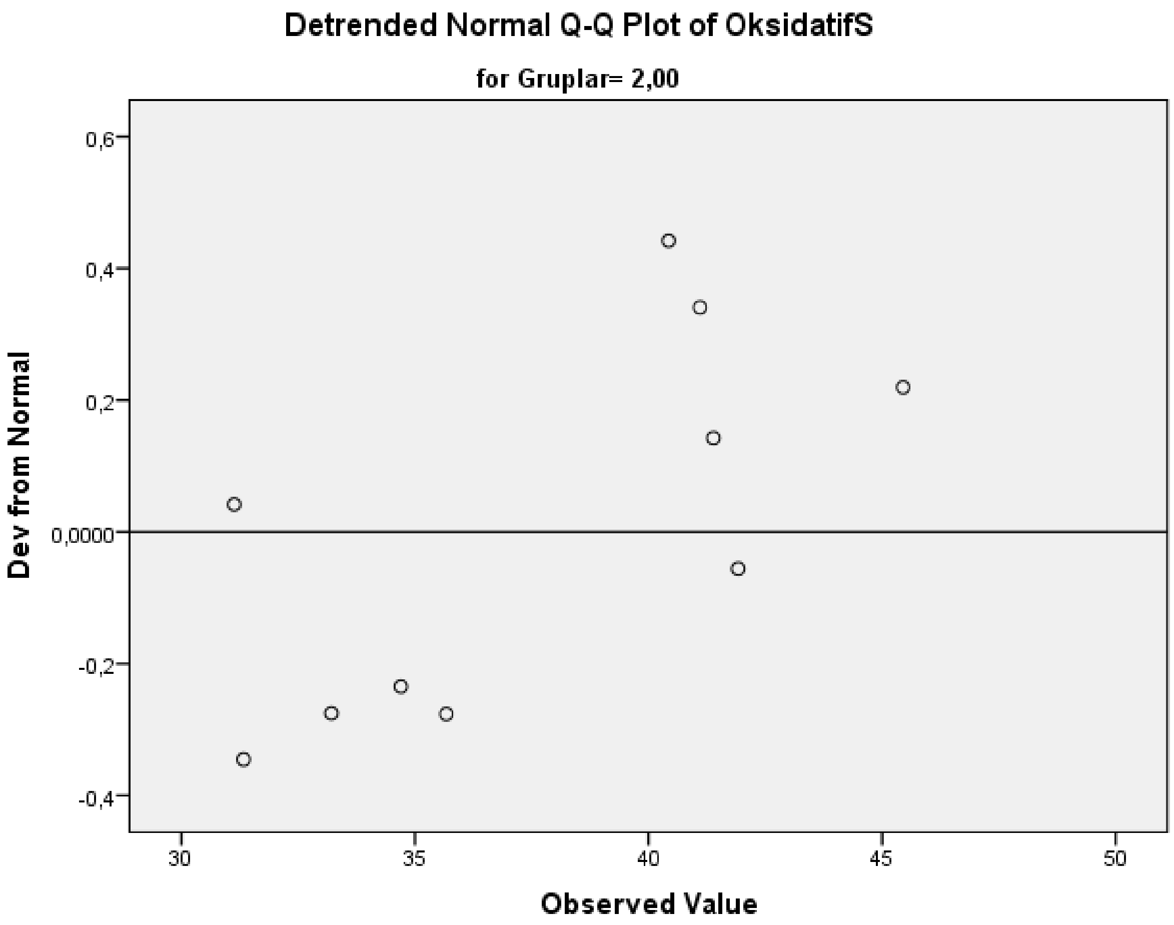

Detrended Normal Q-Q Plot of OksidatifS
for Gruplar= 2,00
Dev from Normal
0,6
0,4
0,2
0,0000
-0,2
-0,4
30
35
40
45
50
Observed Value

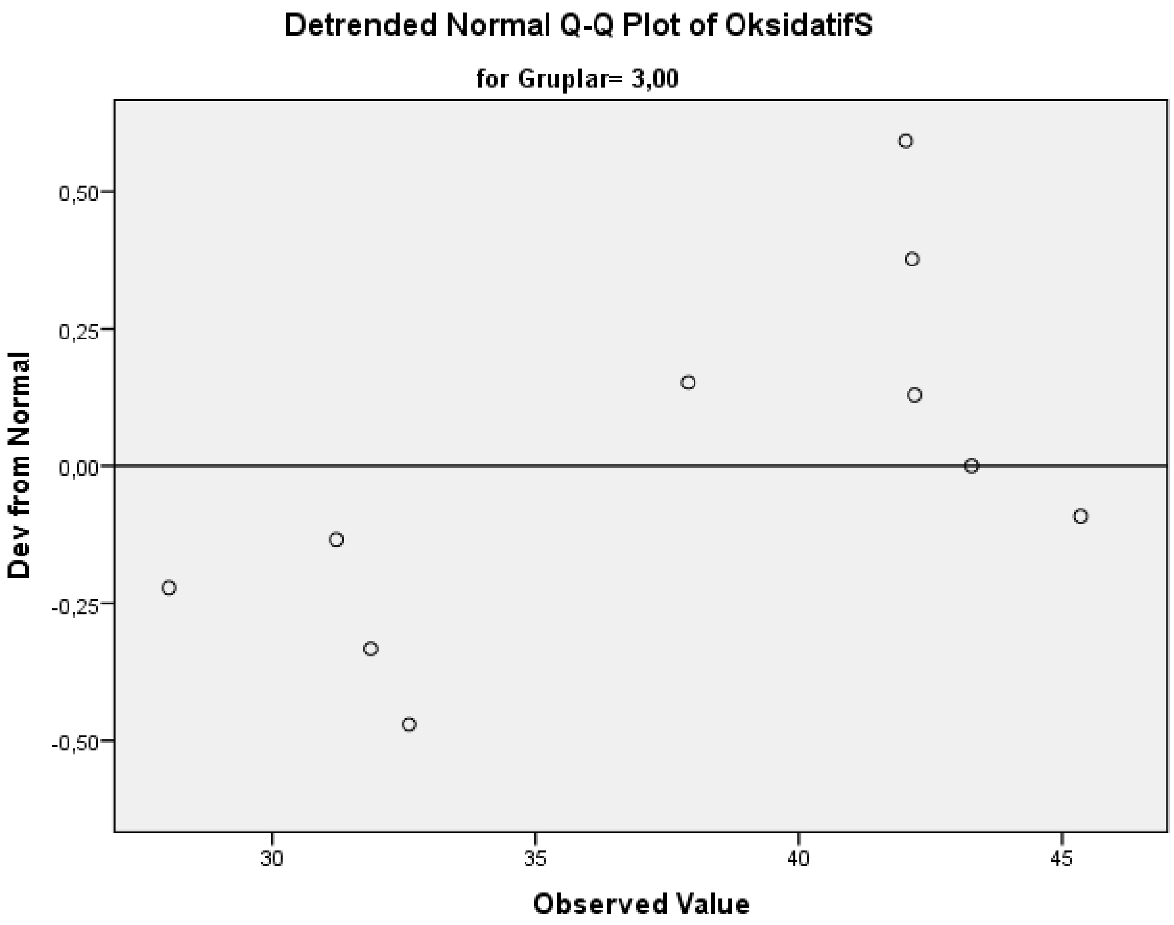

Detrended Normal Q-Q Plot of OksidatifS
for Gruplar= 3,00
Dev from Normal
0,50
0,25
0,00
-0,25
-0,50
30
35
40
45
Observed Value

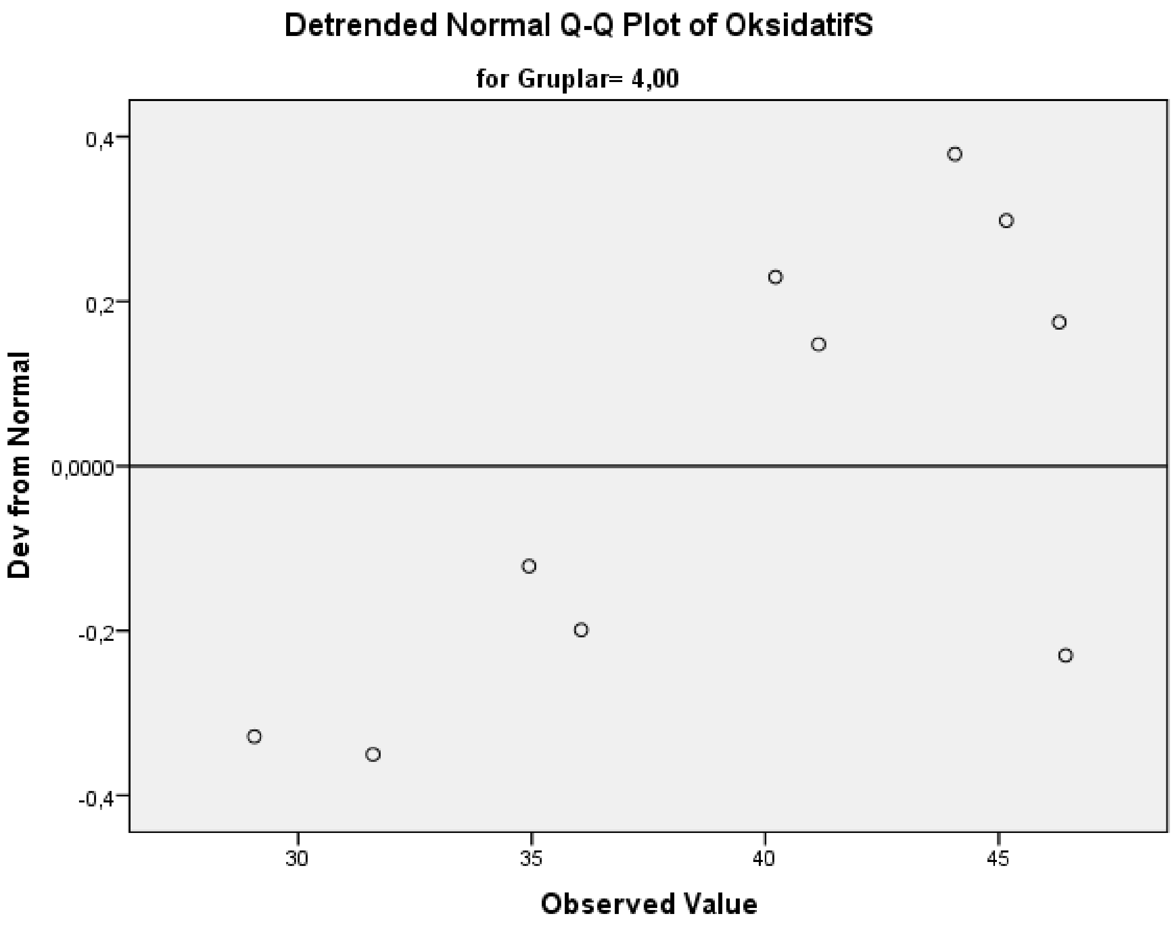
Detrended Normal Q-Q Plot of OksidatifS
for Gruplar= 4,00
0,4
0,2
0,0000
-0,2
-0,4
Dev from Normal
30
35
40
45
Observed Value

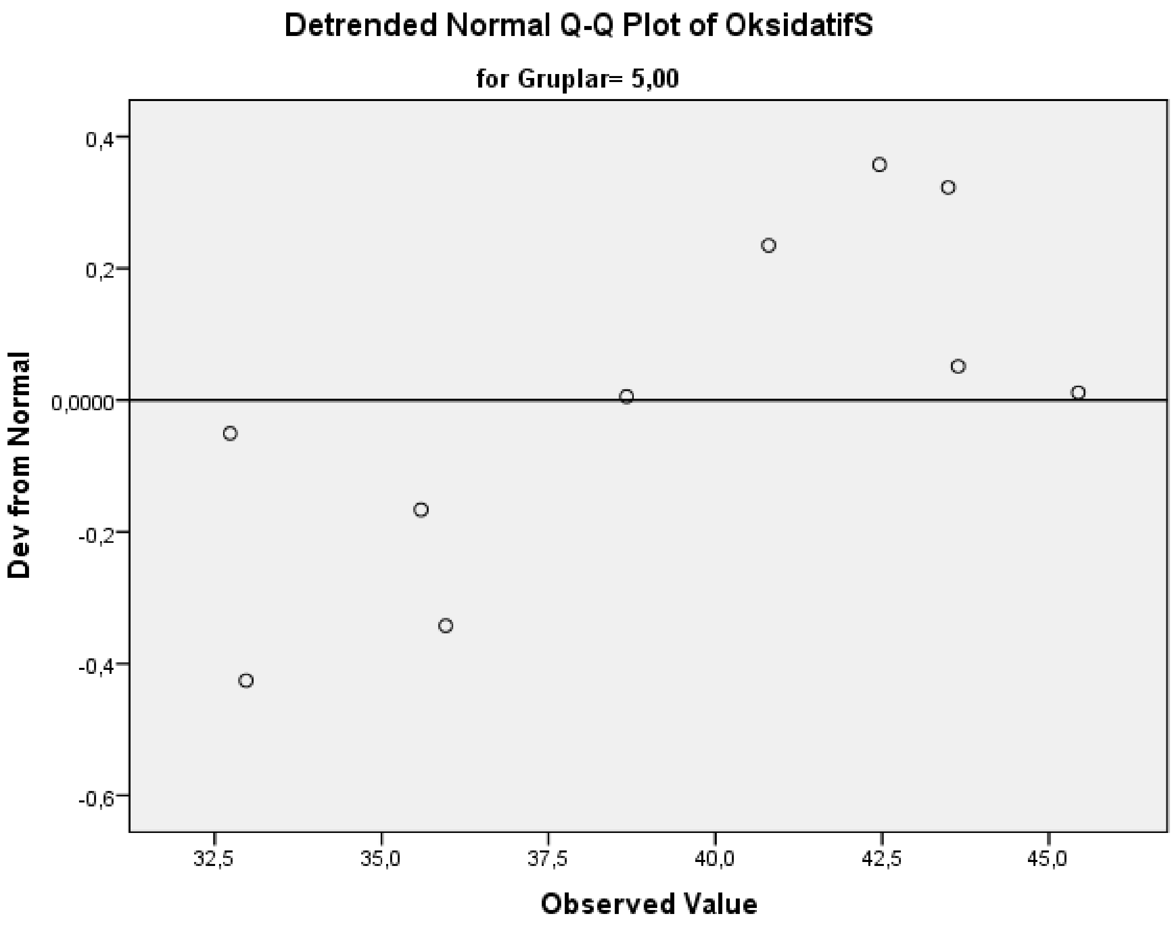

Detrended Normal Q-Q Plot of OksidatifS
for Gruplar= 5,00
Dev from Normal
0,4
0,2
0,0000
-0,2
-0,4
-0,6
32,5
35,0
37,5
40,0
42,5
45,0
Observed Value

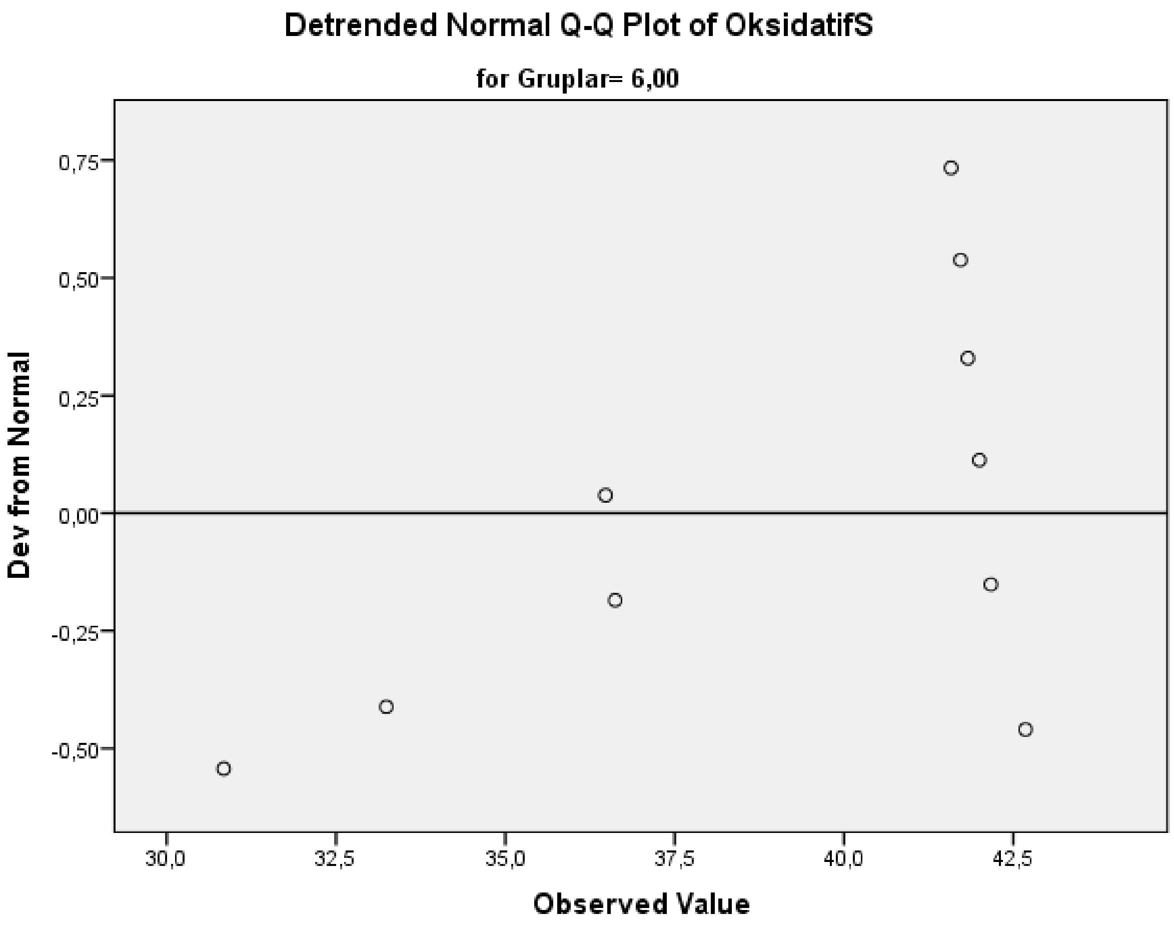
Detrended Normal Q-Q Plot of OksidatifS
for Gruplar= 6,00
Dev from Normal
0,75
0,50
0,25
0,00
-0,25
-0,50
30,0
32,5
35,0
37,5
40,0
42,5
Observed Value

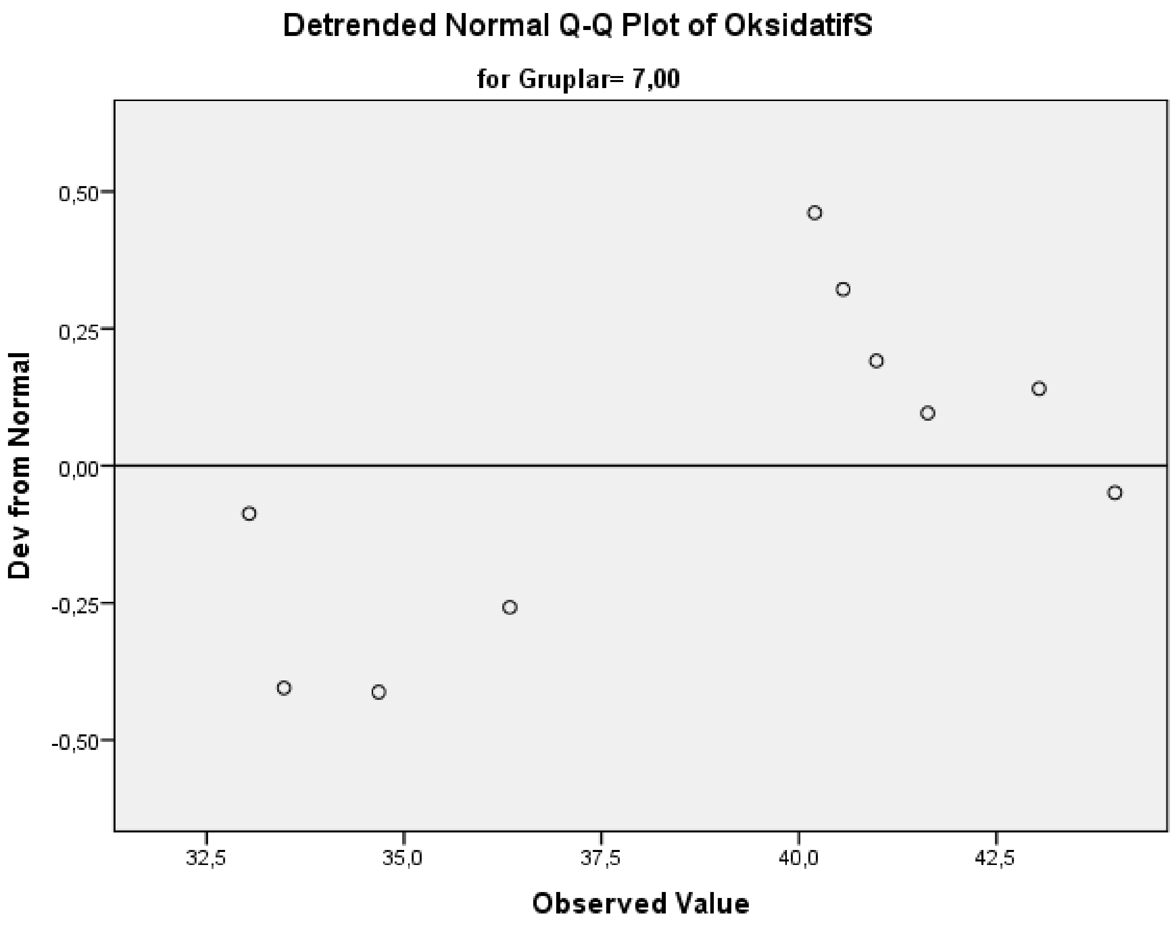

Detrended Normal Q-Q Plot of OksidatifS
for Gruplar= 7,00
0,50
0,25
0,00
-0,25
-0,50
Dev from Normal
32,5
35,0
37,5
40,0
42,5
Observed Value

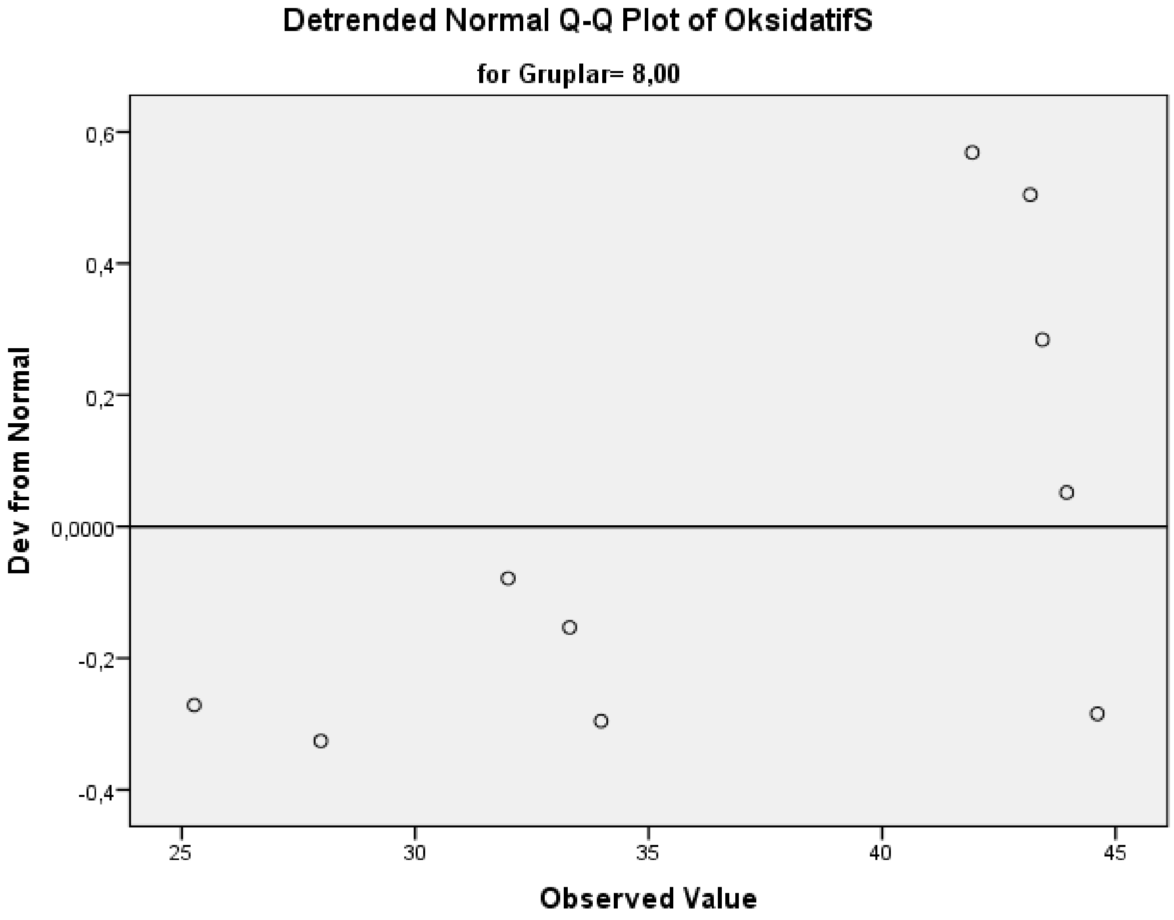

Detrended Normal Q-Q Plot of OksidatifS
for Gruplar= 8,00
Dev from Normal
0,6
0,4
0,2
0,0000
-0,2
-0,4
25
30
35
40
45
Observed Value

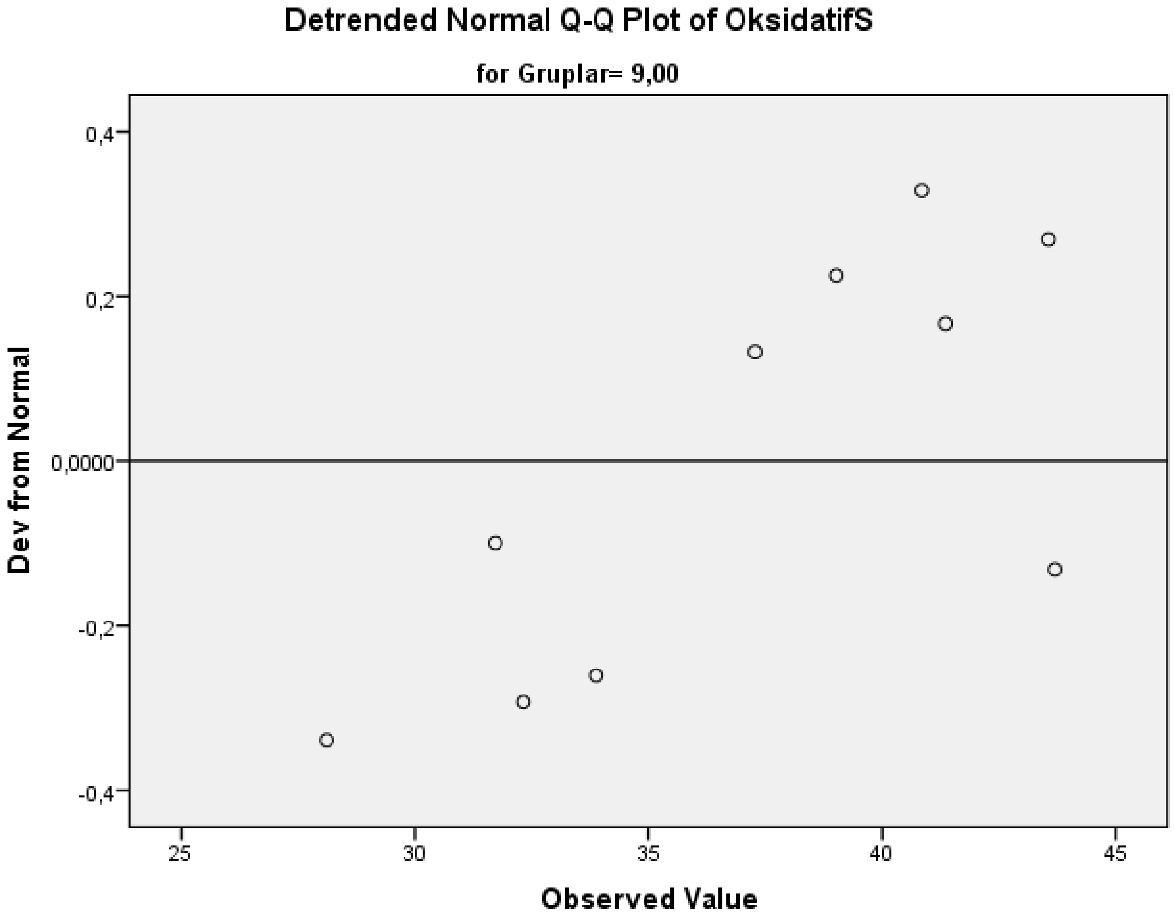
Detrended Normal Q-Q Plot of OksidatifS
for Gruplar= 9,00
Dev from Normal
0,4
0,2
0,0000
-0,2
-0,4
25
30
35
40
45
Observed Value

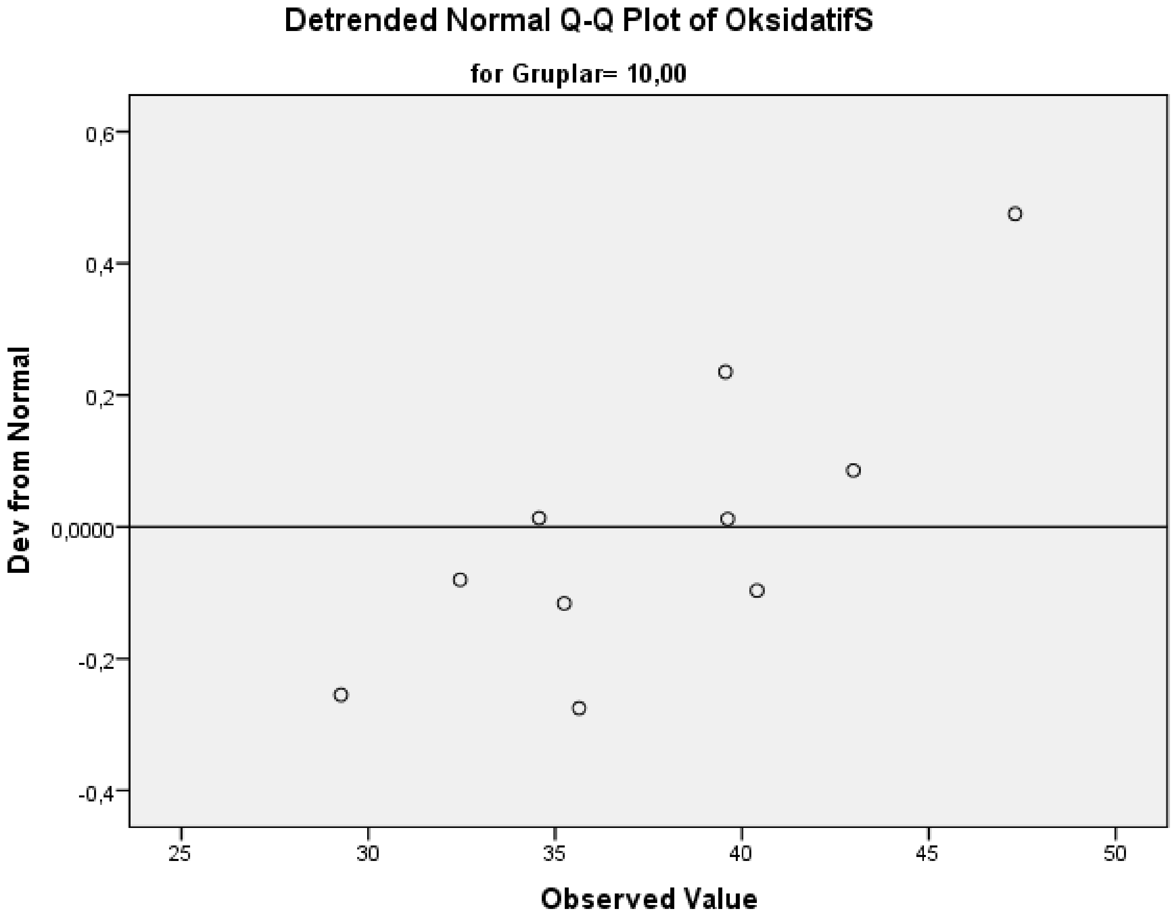

Detrended Normal Q-Q Plot of OksidatifS
for Gruplar= 10,00
Dev from Normal
0,6
0,4
0,2
0,0000
-0,2
-0,4
25
30
35
40
45
50
Observed Value

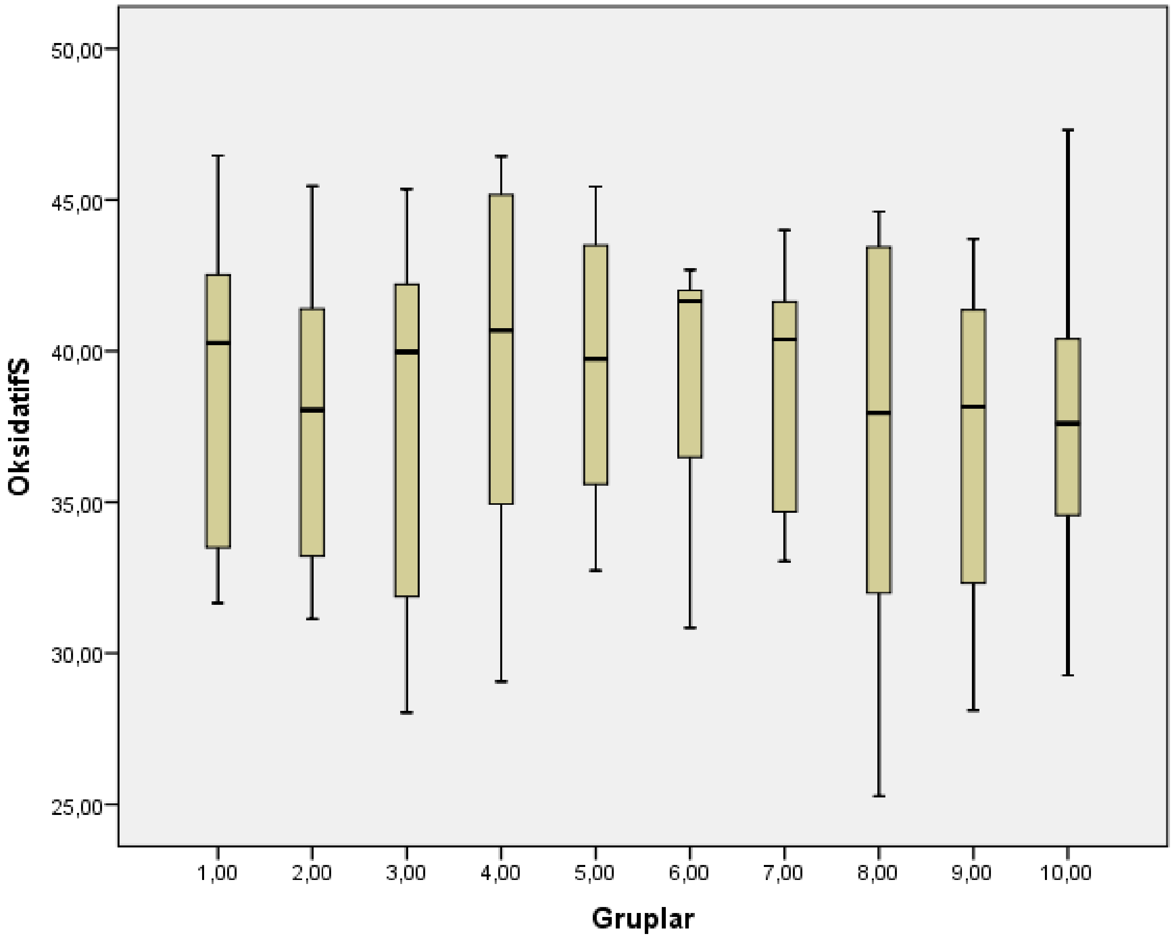


```
ONEWAY OksidatifS BY Gruplar
  /STATISTICS DESCRIPTIVES HOMOGENEITY
  /PLOT MEANS
  /MISSING ANALYSIS
  /POSTHOC=DUNCAN ALPHA(0.05).
```

Oneway

Notes

Output Created		24-DEC-2020 12:43:33
Comments		
Input	Active Dataset	DataSet0
	Filter	<none>
	Weight	<none>

	Split File	<none>
	N of Rows in Working Data File	100
Missing Value Handling	Definition of Missing	User-defined missing values are treated as missing.
	Cases Used	Statistics for each analysis are based on cases with no missing data for any variable in the analysis.
Syntax		ONEWAY OksidatifS BY Gruplar<br>/STATISTICS DESCRIPTIVES HOMOGENEITY<br>/PLOT MEANS<br>/MISSING ANALYSIS<br>/POSTHOC=DUNCAN ALPHA(0.05).
Resources	Processor Time	00:00:00,16
	Elapsed Time	00:00:00,30

38,8820
37,6330
37,6640
39,4960
39,1750
38,9160
38,7950
36,9620
37,1800
37,7050

Test of Homogeneity of Variances

OksidatifS

Levene Statistic	df1	df2	Sig.
1,454	9	90	,178

ANOVA

OksidatifS

	Sum of Squares	df	Mean Square	F	Sig.
Between Groups	73,723	9	8,191	,276	,980

Within Groups	2667,365	90	29,637		
Total	2741,087	99			

Post Hoc Tests

Homogeneous Subsets

OksidatifS

Duncan[a]

Gruplar	N	Subset for alpha = 0.05
		1
8,00	10	36,9620
9,00	10	37,1800
2,00	10	37,6330
3,00	10	37,6640
10,00	10	37,7050
7,00	10	38,7950
1,00	10	38,8820
6,00	10	38,9160
5,00	10	39,1750
4,00	10	39,4960
Sig.		,393

Means for groups in homogeneous subsets are displayed.

a. Uses Harmonic Mean Sample Size = 10,000.

Means Plots

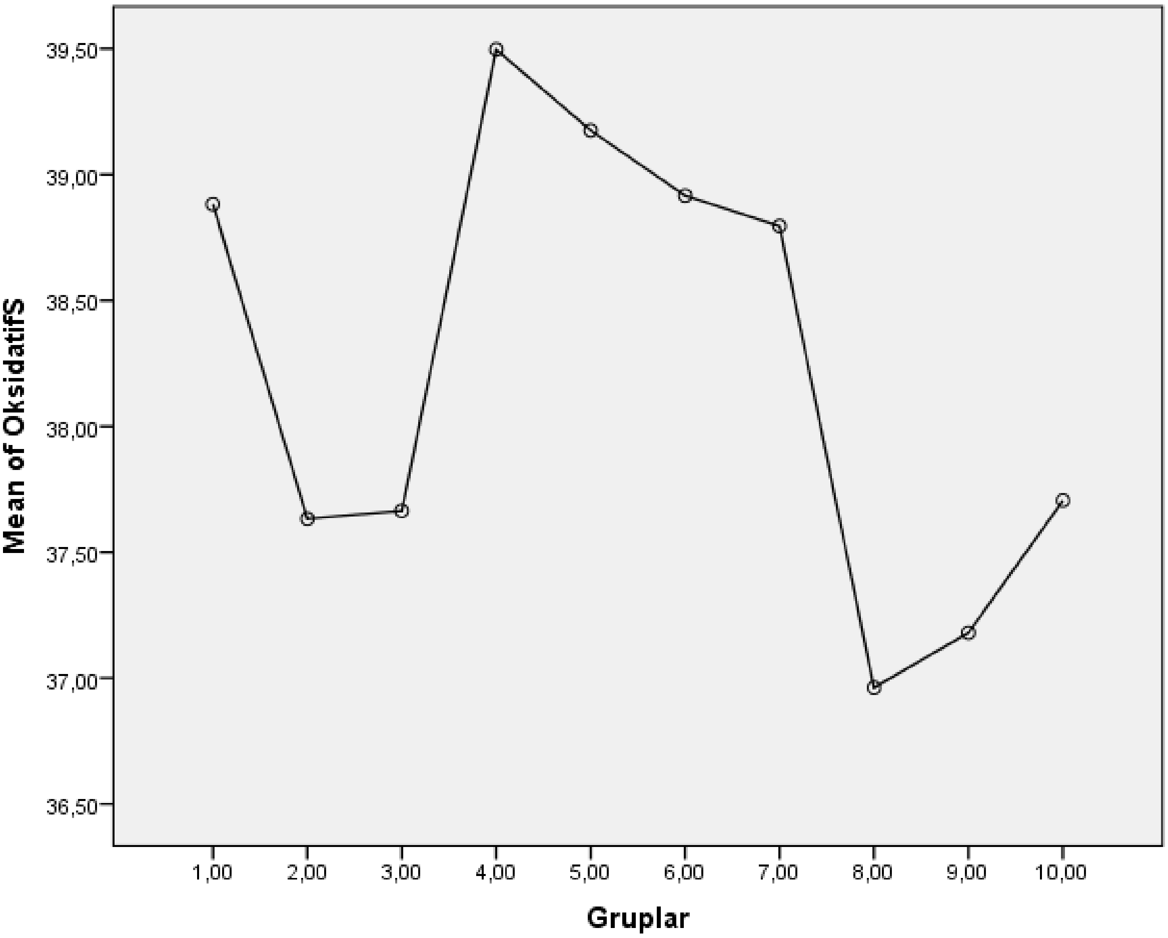

Mean of OksidatifS
39,50
39,00
38,50
38,00
37,50
37,00
36,50
1,00
2,00
3,00
4,00
5,00
6,00
7,00
8,00
9,00
10,00
Gruplar
